# Contrasting genetic structure between mitochondrial and nuclear markers in the dengue fever mosquito from Rio de Janeiro: implications for vector control

**DOI:** 10.1111/eva.12301

**Published:** 2015-09-07

**Authors:** Gordana Rašić, Renata Schama, Rosanna Powell, Rafael Maciel-de Freitas, Nancy M Endersby-Harshman, Igor Filipović, Gabriel Sylvestre, Renato C Máspero, Ary A Hoffmann

**Affiliations:** 1 Pest and Environmental Adaptation Research Group, School of Biosciences, Bio21 Institute, The University of MelbourneParkville, Vic., Australia; 2 Laboratório de Fisiologia e Controle de Artrópodes Vetores, Instituto Oswaldo Cruz, FiocruzRio de Janeiro, Brazil; 3 Laboratório de Biologia Computacional e Sistemas, Instituto Oswaldo Cruz, FiocruzRio de Janeiro, Brazil; 4 Laboratório de Transmissores de Hematozoários, Instituto Oswaldo Cruz, FiocruzRio de Janeiro, Brazil; 5 Gerencia de Risco Biológico da Coordenação de Vigilância Ambiental em Saude, Superintendência de Vigilânciaem Saude – SMSRio de Janeiro, Brazil

**Keywords:** *Aedes aegypti*, genetic structure, microsatellites, mito-nuclear discordance, RAD-seq, Rio de Janeiro, vector control

## Abstract

Dengue is the most prevalent global arboviral disease that affects over 300 million people every year. Brazil has the highest number of dengue cases in the world, with the most severe epidemics in the city of Rio de Janeiro (Rio). The effective control of dengue is critically dependent on the knowledge of population genetic structuring in the primary dengue vector, the mosquito *Aedes aegypti*. We analyzed mitochondrial and nuclear genomewide single nucleotide polymorphism markers generated *via* Restriction-site Associated DNA sequencing, as well as traditional microsatellite markers in *Ae. aegypti* from Rio. We found four divergent mitochondrial lineages and a strong spatial structuring of mitochondrial variation, in contrast to the overall nuclear homogeneity across Rio. Despite a low overall differentiation in the nuclear genome, we detected strong spatial structure for variation in over 20 genes that have a significantly altered expression in response to insecticides, xenobiotics, and pathogens, including the novel biocontrol agent *Wolbachia*. Our results indicate that high genetic diversity, spatially unconstrained admixing likely mediated by male dispersal, along with locally heterogeneous genetic variation that could affect insecticide resistance and mosquito vectorial capacity, set limits to the effectiveness of measures to control dengue fever in Rio.

## Introduction

Dengue fever, also known as the ‘breakbone fever’, is the most prevalent global arboviral disease that affects over 300 million people every year (Bhatt et al. 2013). Brazil has the highest number of dengue cases reported annually to the World Health Organization (WHO 2012). The spread of dengue in Brazil has been exacerbated by rapid urbanization and underdeveloped water supply infrastructure (Barata et al. 2001). Such conditions are favorable for breeding of the primary dengue vector, the mosquito *Aedes aegypti* (Linnaeus 1762), that prefers to bite humans in densely populated areas and lay eggs in man-made water containers (Natal 2002). Various insecticide-based control programs are thought to have eradicated *Ae. aegypti* from Brazil in 1958 (Soper 1965). After a brief period of re-infestation and re-eradication from 1967 to 1973, the mosquito is thought to have returned to Brazil in 1976 (Brathwaite Dick et al. 2012), presumably from the neighboring countries that were never declared free of *Ae. aegypti* (Monteiro et al. 2014). Dengue appeared soon after the vector's reintroduction and is now endemic in Brazil, representing a major public health threat (Teixeira et al. 2013).

Because an effective vaccine against dengue is still not available, disease control relies almost entirely on suppression of the vector *Ae. aegypti* (WHO 2012). Sustainable and lasting control of *Ae. aegypti* in Brazil has proven challenging, despite the extensive efforts of public health organizations and local communities (Claro et al. 2006; Santos et al. 2013). Recent activities that involved intensive mechanical and insecticide applications failed to significantly reduce *Ae. aegypti* numbers and also led to an increase in insecticide resistance that rapidly spread through the neighboring populations (Maciel-de-Freitas et al. 2014). Such undesirable consequences of traditional control measures have prompted the development and implementation of alternative biocontrol strategies (McGraw and O'Neill 2013), including the replacement of local *Ae. aegypti* populations with the *Wolbachia*-infected mosquitoes that have reduced potential for dengue transmission (Hoffmann et al. 2011; Walker et al. 2011).

Efficient implementation of control programs is highly dependent on understanding the dynamics of local mosquito populations, their genetic diversity and spatio-temporal structuring (Endersby et al. 2011; Campos et al. 2012; Rašić et al. 2014a). Genetic structure in *Ae. aegypti* populations from Brazil has been mostly examined at broad geographic scales using mitochondrial DNA variation and nuclear markers such as RAPDs, allozymes, and microsatellites. Mitochondrial markers (COXI, ND4) revealed co-occurrence of two divergent lineages (Bracco et al. 2007; Scarpassa et al. 2008) and substantial gene flow among *Ae. aegypti* populations from different regions of Brazil (Gonçalves da Silva et al. 2012). Conversely, nuclear markers have generally demonstrated a high level of genetic structure and isolation of mosquito populations from different regions of the country (Ayres et al. 2004; da Costa-Ribeiro et al. 2007; Monteiro et al. 2014). When genetic structure was analyzed at finer geographic scales (within one region or a city in Brazil), microsatellites and a few single nucleotide polymorphisms (SNPs) revealed genetic homogeneity and high gene flow in the dengue fever mosquito (Campos et al. 2012; Mendonça et al. 2014).

Our objective was to assess the level of genetic structuring in *Ae. aegypti* from the city of Rio de Janeiro (Rio). Rio is considered a primary entry point for dengue viruses in Brazil from which they spread rapidly throughout the country (Lourenço-de-Oliveira et al. 2004). Furthermore, the largest number of recent dengue epidemics has been concentrated in this city (Roriz-Cruz et al. 2010). High dispersal distances and densities of *Ae. aegypti* in Rio have been suggested as important contributors to the intense risk of dengue epidemic in the city (Maciel-de-Freitas et al. 2007, 2014). Large populations and weakly restricted dispersal should lead to a low level of genetic structuring (Wright 1943), yet two previous studies found high genetic structure in *Ae. aegypti* in Rio using a few nuclear markers (isozymes and microsatellites) (da Costa-Ribeiro et al. 2006a,b).

Because ecological data and previously collected genetic data have led to inconsistent views on dispersal, we re-examined population genetic structuring in *Ae. aegypti* from Rio. We used multiple marker systems: nuclear and mitochondrial genomewide SNPs generated *via* Restriction-site Associated DNA sequencing (RAD-seq), as well as traditional microsatellite markers. Analyses of multiple marker systems allowed us to compare genetic diversity and structure with different spatio-temporal resolution, because different markers have distinct rates of evolution, levels of variability, and modes of inheritance (Avise 2004). The high density RAD-seq markers also provide an opportunity to consider functionally important variation across populations. We discuss implications of our findings for the implementation of current and future strategies to suppress dengue fever mosquito in Rio.

## Methods

### Ethics statement

No specific field ethics approval is needed for the collection of wild mosquitoes in the study areas. The Health Municipality of Rio de Janeiro and Niteroi use ovitraps as a routine tool to estimate the frequency of *Ae. aegypti* and *Ae. albopictus* throughout the cities as well as to evaluate the efficiency of vector control strategies by measuring post-treatment mosquito infestation. Verbal consent was obtained from residents at each location where collections occurred on private property. The locations were not on protected land, and the collections did not involve endangered or protected species.

### Collections of *Aedes aegypti*

**Figure 1:**
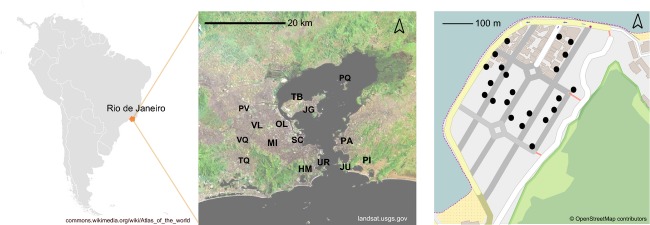
Sampling design. Sites of Rio de Janeiro and trap locations within one such site (500 × 500 m area) where *Aedes aegypti* were collected. Codes for sampling location are listed in Table1.

We collected mosquito larvae in 15 locations throughout the city of Rio de Janeiro and the surroundings (Table1, Fig.1). Site location was intended to represent the diversity of urban infrastructure, history of dengue transmission, water storage systems, mosquito, and human density. From each sampling area, larvae were collected using ovitraps. The greatest distance between sample collection points is 29 km, and each location comprised a 500 × 500 m^2^ area (Fig.1). Mosquitoes were collected within a single 3-week period in November–December 2011 at the beginning of the wet season (wet 2011), followed by the collection in the next wet season during December 2012 (wet 2012) and the dry season during August 2013 (dry 2013). The GPS coordinates of each sampling site and collection date were recorded. No more than five individuals were collected from each ovitrap to reduce the number of sibling individuals obtained, as related individuals tend to co-occur within the same trap (Apostol et al. 1993; Hoffmann et al. 2014; Rašić et al. 2014b). Larvae were then reared in insectary under standard conditions (25 ± 1°C, 80 ± 10% relative humidity and 12 h light/dark cycle) until the third instar stage for species identification. All samples identified as *Ae. aegypti *were stored in absolute ethanol at 4°C. DNA was extracted from one larva per ovitrap using the Qiagen DNA Blood and Tissue kit with RNAse treatment (Venlo, Limburg, the Netherlands) for SNP typing and the CTAB protocol (Weeks et al. 2002) for microsatellite typing.

**Table 1:** Sample sites and sizes

Location	Code	Lat	Long	Wet 2011	Wet 2012	Dry 2013
Humaitá	HM	−22.955	−43.198	13	–	5
Jardim Guanabara	JG	−22.808	−43.199	22	–	5
Jurujuba	JU	−22.932	−43.113	14	15	4
Méier	MI	−22.905	−43.278	31	–	9
Olaria	OL	−22.839	−43.263	31	–	7
Paquetá	PQ	−22.764	−43.107	19 (17)	13	10
Pavuna	PV	−22.811	−43.359	33	–	11
Piratininga	PI	−22.933	−43.074	36	9	6
Ponta D'Areia	PA	−22.884	−43.124	34	10	–
Sao Crístovão	SC	−22.891	−43.222	24	7	5
Taquara	TQ	−22.924	−43.381	22	8	8
Tubiacanga	TB	−22.785	−43.226	36 (30)	15	9
Urca	UR	−22.945	−43.162	35 (29)	–	9
Valqueire	VQ	−22.886	−43.366	31	15	7
Vaz Lobo	VL	−22.859	−43.324	28	11	6
Total				409	103	101

Geographic coordinates (lat/long) and sizes of *Aedes aegypti* samples collected in Rio de Janeiro, Brazil, during three seasons (wet 2011 and 2012, dry 2013). Sample sizes analyzed at restriction-site associated DNA sequencing (RAD-seq) loci in addition to the microsatellite loci are found in parentheses.

### RAD-seq data processing and SNP typing

Seventy six individuals collected in Tubiacanga, Urca, and Paquetá island during the wet season in 2011 were screened for nuclear and mitochondrial genomewide SNP variation (Table1). The three locations were chosen as areas with contrasting urbanization levels that are characteristic for different parts of Rio and as sites intended for *Wolbachia* releases (http://www.eliminatedengue.com/rio-de-janeiro/noticias/article/404/project/rio-de-janeiro). We used a customized double-digest RAD-seq method (Peterson et al. 2012) following the procedure described in Rašić et al. (2014b). Briefly, 100 ng of genomic DNA from each larva was digested with 100 units of restriction enzymes *Nla*III and *Mlu*CI (New England Biolabs, Beverly MA, USA). One hundred picomolar P1 and 300 pm P2 Illumina adapters with customized barcode sequences were ligated to the genomic fragments using 100 units of T4 ligase at 16°C overnight (New England Biolabs). Following the sample pooling and purification, size selection of fragments 300–450 bp in length was completed with the Pippin Prep protocol (Sage Sciences, Beverly, MA, USA). We used 12 PCR cycles with standard Illumina primers for the final library enrichment: 98°C for 30 s, 12 cycles of 98°C for 10 s, 60°C for 30 s, 72°C for 90 s, and the final elongation at 72°C for 5 min. The libraries were sequenced with HiSeq2000 using the 100-bp paired-end chemistry.

We used FASTX Toolkit (http://hannonlab.cshl.edu/fastx_toolkit/index.html by Hannon Lab) to trim all raw FASTQ reads to the same length of 80 bp and retained those with a phred score ≥20 over the entire sequence length. High-quality reads were aligned to the *Ae. aegypti* reference nuclear genome AaegL1 (Nene et al. 2007) and the mitochondrial genome (Behura et al. 2011) with the short read aligner program *Bowtie* (Langmead et al. 2009). The alignment parameters included a maximum of three mismatches permitted in the seed, a ‘try-hard’ option to find valid alignments and suppression of reads with more than one optimal alignment. Uniquely, aligned reads were then analyzed with the stacks pipeline v.1.09 (Catchen et al. 2013). Variant and likelihood-based genotype calling was performed using the default parameters on RAD stacks with a minimum depth of reads of five.

### Microsatellite genotyping

Eight microsatellite loci previously described (AG5, BbH08, BbA10, AC1, 12ACG1, M201, 69TGA1, BbB19) (Slotman et al. 2006; Chambers et al. 2007; Lovin et al. 2009) were amplified and directly labeled with fluorescent dye as described in Rašić et al. (2014a). The PCR conditions contained: initial incubation step with 94°C for 15 min, 35 amplification cycles of 94°C for 30 s, 60°C for 90 s, 72°C for 60 s, followed by eight labeling cycles with 94°C for 30 s, 53°C for 90 s, 72°C for 60 s, and a final elongation step at 60°C for 30 min. Fragment analysis was carried out with Applied Biosystems (Waltham, MA, USA) 3730 DNA Analyser and 500 LIZ as the size standard. We used GeneMarker v.2.2.0 (Softgenetics, State College, PA, USA) for allele scoring. In total, 613 individuals were genotyped and used for subsequent analyses (Tables1 and S1).

### Analyses of genetic diversity

#### Diversity parameters

The number of mitochondrial haplotypes (_mt_*N*_h_), nucleotide diversity (_mt_*π*), haplotype diversity (_mt_*H*_d_), and theta based on the number of segregating sites (_mt_*θ*) was calculated in r package *pegas* (Paradis 2010). A mitochondrial haplotype network was constructed using the statistical parsimony method implemented in r package *TempNet* (Prost and Anderson 2011). To assess the level of nuclear genomewide variation, we estimated the observed and expected heterozygosity (*H*_O_, *H*_E_), nucleotide diversity (_n_*π*), and theta (_n_*θ*) using the program stacks v.1.09 (Catchen et al. 2013). For microsatellite loci, we calculated the observed and expected heterozygosity (*H*_O_, *H*_E_) and allelic richness (*N*_A_) using the rarefaction method in r package *gstudio* (http://cran.rproject.org/web/packages/gstudio/index.html).

#### Phylogenetic analyses

To assess the evolutionary relationship among *Ae. aegypti* lineages found in Rio, we performed a maximum likelihood (ML) phylogenetic analysis using (i) the rapid bootstrap heuristic algorithm followed by a thorough ML search implemented in the program RAxML (Stamatakis et al. 2008) and (ii) the model-averaged phylogeny reconstruction in jModelTest 2.1.7 (Darriba et al. 2012). In addition to the samples from Rio, we included a previously published RAD-seq dataset that contains 17 *Ae. aegypti* individuals from Australia, 15 individuals from Vietnam, and 13 individuals from Indonesia (Rašić et al. 2014b) (NCBI SRA accession numbers: PRJNA241150, PRJNA273913). For each individual, we extracted informative mitochondrial polymorphisms (SNPs detected among individuals) from the RAD tags and concatenated them into the final mitochondrial sequence with 92 variable positions. Informative nuclear polymorphisms were concatenated into the final sequence that contained 5815 variable positions. Intra-individual site polymorphisms (i.e. heterozygous SNP loci) were denoted with ambiguous IUPAC codes (e.g. W for adenine or cytosine, Y for cytosine or thymine, etc.), and the RAxML program considers these ambiguous sites in the phylogenetic reconstruction (Potts et al. 2014). We tested 44 substitution models with the Gamma-distributed rate variation among sites (+G) and calculated the AIC weight as a measure of model support in jModelTest2. These results were used to compute the average phylogenetic reconstruction as the consensus of the ML trees for every model weighted with their AIC weight (Posada 2008). RAxML implements only GTR as the most complex and general model of nucleotide substitution, suitable for datasets with genomewide variation (Stamatakis 2014). We excluded models with the proportion of Invariant sites (+I) in both approaches because invariant sites were absent from our datasets. Separate phylogenetic trees were produced for mitochondrial and nuclear genome data (Files S1 and S2).

#### Neutrality tests and inferences of demographic change

For the mitochondrial dataset, we calculated the neutrality tests statistics Tajima's *D* (Tajima 1989) and *R*_2_ (Ramos-Onsins and Rozas 2002), determining the statistical significance with 1000 simulations of populations under the drift-mutation equilibrium in *pegas* (Paradis 2010). Negative Tajima's *D* value indicates departures from mutation-drift equilibrium due to processes such as population expansions or directional selection, while positive value indicates balancing selection, bottlenecks, and/or cryptic population structure (Tajima 1989). We used *R*_2_ test for detecting population growth because it has greater power than a classical mismatch distribution test, particularly for smaller sample sizes (Ramos-Onsins and Rozas 2002).

Due to uncertainty of diploid genotype calls, nuclear data are prone to an artificial inflation of rare polymorphisms (Nielsen et al. 2012). We therefore used the empirical Bayesian approach in calculating the neutrality statistics as implemented in the software angsd (Korneliussen et al. 2013). This approach accommodates the uncertainty of genotype data in statistic calculations and intrinsically deals with missing data and variable depth of loci that are common in next-generation sequencing experiments (Korneliussen et al. 2013). It is particularly beneficial for low- to medium-coverage data (<20×) such as ours. Tajima's *D* was estimated from the genotype likelihoods obtained using the GATK-based genotype calling method and presence in at least 70% of individuals. We also used this method to calculate the site frequency spectrum (SFS) as folded because information on ancestral polymorphisms is not available for *Ae. aegypti*. To generate the distribution of population parameters expected under drift-mutation equilibrium, we performed coalescent simulations using the programs msms (Ewing and Hermisson 2010) and angsd. We generated 100 replicates, assuming a recombination rate of 1 cM/Mb (Juneja et al. 2014) and *theta* that was fitted to the empirical estimate (Table1). We then simulated genotype likelihoods with an 8× read depth (as in the empirical dataset, see Results) and an error rate of 0.5%, from which we then calculated the folded SFS as in Nielsen et al. (2012).

### Analyses of genetic structuring

#### Individual-based inferences

We used two individual-based clustering approaches: (i) discriminant analysis of principal components (DAPC) implemented in the r package *Adegenet* (Jombart and Ahmed 2011) and (ii) Bayesian clustering implemented in *TESS* (Chen et al. 2007).

Discriminant analysis of principal components is a nonspatial multivariate method that we applied to the nuclear SNP data and microsatellites. We calculated the *a*-score as the criterion for determining the optimal number of PCs to be retained in a discriminant analysis. Strength of discrimination was measured using the reassignment of supplemental individuals into the clusters that were predefined with the subset of 10–15 individuals from each sampling location, where higher percentage of correct reassignment indicates stronger discrimination (i.e. higher structure). The optimal number of genetic clusters was calculated using the successive *k*-means procedure, where different clustering solutions were compared with the Bayesian information criterion (BIC). The lowest BIC value indicates the optimal number of genetic clusters (Jombart et al. 2010).

Spatially explicit Bayesian clustering was applied to microsatellite data only, because the underlying *TESS* model requires a larger number of spatial sampling points (François and Durand 2010). We used the admixture model with a weighted geographic distance between individuals, 100 000 iterations and 40 000 burn-ins. For consistent visualization of the overall replicate results, outputs from 10 independent replicates for each *k* were permuted in the program clumpp (Jakobsson and Rosenberg 2007).

Existence of isolation-by-distance between individuals was tested using the Mantel test with 999 permutations on matrices of linearized genetic distance between individuals (Smouse and Peakall 1999) and a corresponding log-geographic distance in GenAlEx 6.5 (Peakall and Smouse 2012).

#### Group-based inferences

We calculated Nei's *G*_ST_ parameter of genetic structure (Nei 1973) for the nuclear SNPs and microsatellite datasets, assuming sampling sites as genetic groups. Statistical significance of parameter deviation from zero in pairwise comparisons was tested using 1000 permutations in *gstudio* (http://cran.r-project.org/web/packages/gstudio/index.html). For the mitochondrial dataset, we calculated the genetic structuring parameter *Phi*_ST_ (Excoffier et al. 1992) and used 1000 permutations for significance testing in the r package *pegas*.

#### Power analysis for detecting genetic structure

Due to a modest number of microsatellite loci (eight) and low sample sizes in the second and third sampling season that were available for this study (Table1), we assessed the statistical power to detect genetic structure in our microsatellite dataset. We used the program powsim (Ryman and Palm 2006) to simulate the degree of structuring (quantified as Nei's *G*_ST_) for different effective population sizes and number of generations under isolation, while assuming the empirical number of samples, sample sizes, number of loci and alleles, and allele frequencies. Power to detect a given level of structuring and type I error were estimated using 1000 simulations.

## Results

### Genetic diversity

#### Mitochondrial genome diversity

Because *Ae. aegypti* has abundant mitochondrial pseudogenes in the nuclear genome (Black and Bernhardt 2009; Hlaing et al. 2009), we carefully checked for any mitochondrial RAD loci that were heterozygous or had premature stop codons, indicating paralogs or nonfunctional gene sequences. Validity of our RADseq mitochondrial data was additionally supported by the phylogenetic analyses that produced the same tree topology as the amplicon sequence data from two mitochondrial genes (COXI and ND5) analyzed in mosquitoes from the same locations (H. L. Yeap, unpublished data; File S4). We found 24 mitochondrial RAD tags that were present in ≥80% of individuals and polymorphic among individuals (File S5). RAD tags distributed across 11 mitochondrial genes were concatenated into 39 haplotypes detected in 76 individuals from the three localities in Rio (Table S2). The sequences contained 32 parsimony informative SNPs and 23 singletons. Haplotype diversity in the combined dataset was 0.812, nucleotide diversity was 0.004, and theta was 11.22 (Table2). Each locality, however, had a specific pattern of mtDNA diversity, with Paquetá island having much higher mitochondrial diversity indices than Tubiacanga (Table2).

**Table 2:** Genetic diversity parameters for the nuclear and mitochondrial single nucleotide polymorphisms (SNPs) in *Aedes aegypti* from Rio

			*θ*		Tajima's *D*			
Location	_nuc_*H*_E_	_nuc_*F*_IS_	nuc	mt	nuc	mt	_mt_*R*_2_	_mt_*H*_d_
Paquetá	0.249	0.034	6.11	12.72	1.06*	−1.82	0.07*	0.948
Tubiacanga	0.236	0.105*	4.07	3.31	0.96	−2.30*	0.15	0.377
Urca	0.223	0.136*	4.29	10.60	1.19*	−2.10*	0.05*	0.936
All	0.239	0.114*	5.52	11.22	1.30*	−2.26*	0.04*	0.812

Mean expected (*H*_E_) heterozygosity and fixation index (*F*_IS_) for nuclear loci; mutation-scaled effective population size (*θ*) and Tajima's *D* for nuclear (nuc) and mitochondrial (mt) genomewide SNPs; mitochondrial haplotype diversity (_mt_*H*_d_) and *R*_2_ parameter for demographic expansion. Parameters are presented for each location separately and for the combined dataset (all).

^*^Significant parameter estimates (*P *<* *0.05).

#### Nuclear genome diversity

The final catalog of nuclear SNPs from the Stacks v.1.09 pipeline contained on average 6620 polymorphic RAD tags (SD = 2792) and 9369 SNPs (SD = 3924) per individual (File S3); 5123 RAD tags were present in ≥80% of individuals. After removing biallelic variants with the minor allele frequency lower than 0.05, we retained a subset of 1496 SNPs. For this subset, mean depth of coverage per site per individual was 8.3×. Overall *F*_IS_ was 0.114 in the pooled dataset and theta (_n_*θ*) was 5.52, but these values varied among the three locations (Table2). The sample from Paquetá island had higher estimates of nuclear diversity when compared to Tubiacanga and Urca (Table2).

#### Microsatellite diversity

All eight analyzed microsatellite loci were polymorphic, with an allelic richness around 4.7 across seasons (Table3). Observed heterozygosity was on average lower than expected, but highly variable across loci (Table3). Three loci (12ACG1, 69TGA1, BbB19) showed significant heterozygote deficiency that was consistent across seasons, indicating the presence of null alleles, while one locus (M201) showed significant excess of heterozygotes (Table3). None of the pairs of loci exhibited significant linkage disequilibrium.

**Table 3:** Descriptive statistics for microsatellite data

	*F*_IS_										
Season	AG5	BbH08	BbA10	AC1	M201	12ACG1	69TGA1	BbB19	*N*_A_ (SE)	*H*_O_ (SE)	*H*_E_ (SE)
Wet 2011	0.001	−0.001	0.144	0.081	−0.411*	0.413*	0.243*	0.261*	4.71 (0.19)	0.513 (0.069)	0.551 (0.051)
Wet 2012	0.050	−0.111	0.071	0.021	−0.258*	0.443*	0.224*	0.426*	4.53 (0.13)	0.498 (0.073)	0.540 (0.047)
Dry 2013	0.166	0.051	−0.160	−0.227*	−0.263*	0.631*	0.336*	0.351*	4.72 (0.14)	0.549 (0.091)	0.585 (0.051)

Overall inbreeding level (*F*_IS_) at eight microsatellite loci across *Aedes aegypti* samples from three seasons. Samples were pooled across localities within a season (15 localities in wet 2011, nine in wet 2012, and 14 in dry 2013). Mean and standard error (SE) for: *N*_A_ – allelic richness (rarefaction for sample size of 50 individuals), *H*_O_ – observed heterozygosity, *H*_E_ – expected heterozygosity, calculated across all individuals and loci within each season.

^*^Significant departure from Hardy–Weinberg equilibrium.

#### Phylogenetic analyses

**Figure 2:**
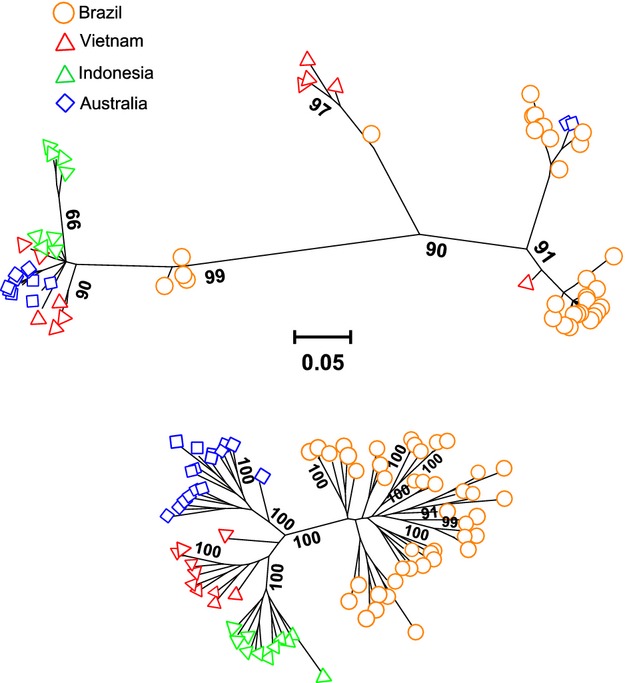
Maximum likelihood (ML) phylogeny. ML phylogenetic reconstructions from RAxML based on mitochondrial genomewide single nucleotide polymorphisms (SNPs) (upper) and nuclear genomewide SNPs (lower) for *Aedes aegypti* samples from Rio de Janeiro (Brazil), along with samples from Australia, Indonesia, and Vietnam. Only bootstrap support values ≥90% are shown. Scale represents mean number of nucleotide substitutions per site.

Mitochondrial and nuclear SNP datasets produced contrasting ML phylogenies: samples from Rio had a homogeneous nuclear background divergent from those found in Asia and Australia, but a highly heterogeneous mitochondrial background (Fig.2). These results were obtained with both phylogenetic approaches, the randomized axelerated ML reconstruction in RAxML, and the model-averaged reconstruction in jModelTest (File S6). Samples from Vietnam and Australia also had highly divergent co-occurring mitochondrial genomes, while those from Indonesia fell into one clade (Fig.2). Nuclear data, on the other hand, separated each of the geographic samples into its own highly supported (≥99%) monophyletic clade. Overall level of divergence between the clades was an order of magnitude greater for the mitochondrial than for the nuclear dataset (1.3% vs 0.2%), reflecting the elevated mutation rate for the mitochondrial sequences when compared with the nuclear sequences.

#### Neutrality tests

**Figure 3:**
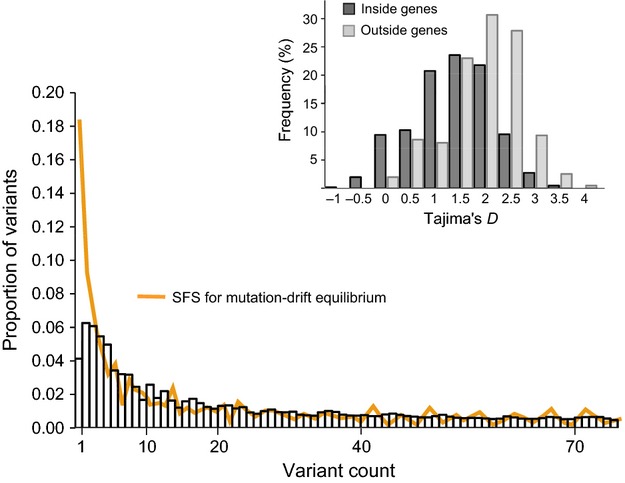
Tajima's *D* and site frequency spectrum (SFS) for nuclear single nucleotide polymorphisms (SNPs). Tajima's *D* values for sites in regions inside and outside genes and folded SFS for the genomewide SNPs and in the empirical dataset (*Aedes aegypti* from Rio de Janeiro) and the dataset simulated under mutation-drift equilibrium.

Neutrality test statistics showed contrasting patterns for the mitochondrial and nuclear datasets. For the mitochondrial genome, significant negative Tajima's *D* values were present in Paquetá island, Urca, and in the pooled dataset (Table2). The *R*_2_ test for population growth was also significant in these samples (Table2). Conversely, Tajima's *D* was positive for the nuclear genome, and this was recorded for regions within and outside genes (Fig.3). A deficit of rare nuclear alleles was also evident in the folded SFS analysis (Fig.3).

### Genetic structuring

**Figure 4:**
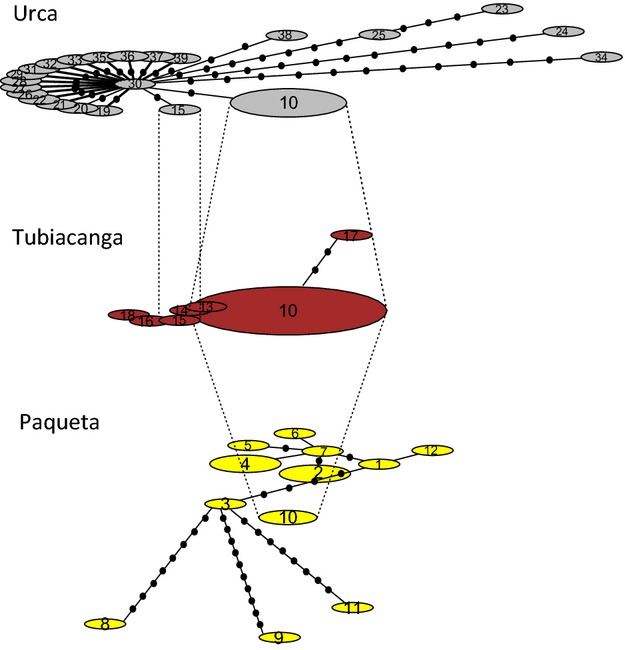
Mitochondrial haplotype network. Haplotype networks in the three localities in Rio. Haplotypes shared among localities are connected by dotted lines.

We detected high spatial structure for the mitochondrial variation, with an overall *Phi*_ST_ of 0.163 (*P *<* *0.001). The distribution of different mitochondrial haplotypes within the three localities in Rio is portrayed in the haplotype network analysis (Fig.4).

**Figure 5:**
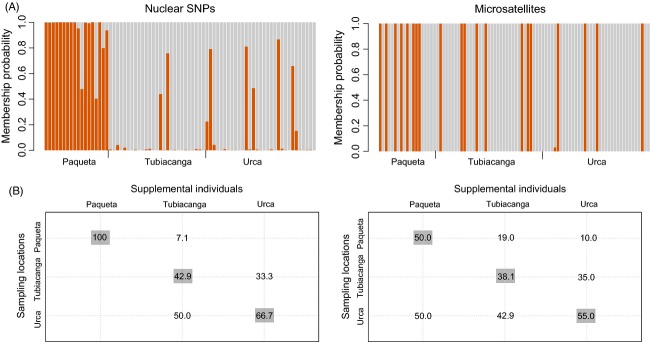
Discriminant analysis of principal components (DAPC). (A) Membership probabilities to two genetic groups for *Aedes aegypti* individuals, inferred using DAPC of genomewide single nucleotide polymorphism (SNP) and microsatellites; individuals were sampled in Paquetá island, Tubiacanga, and Urca; (B) blind assignment success for supplemental individuals to the three sampling locations; diagonal values in darker boxes represent assignment to the original sampling location (‘correct reassignment’).

In contrast, nuclear variation exhibited a low level of spatial structuring and this was observed in both individual- and group-based analyses. In DAPC, the optimal number of genetic clusters was one (*k *=* *1) for both nuclear SNPs and microsatellite datasets, as indicated by the lowest BIC value in the successive *k*-means procedure (Figure S1). Mosquitoes from Paquetá island did show greater genetic isolation and were more separated from mosquitoes in Tubiacanga and Urca based on their nuclear SNPs but not microsatellites (Fig.5). Overall assignment of individuals to their sampling location was 58% for the nuclear SNP dataset and 46% for the microsatellite dataset (Fig.5). *TESS* analysis of the microsatellite datasets indicated that mosquitoes sampled across Rio (Table1) had the highest membership probability to one genetic cluster, but several individuals showed different or mixed membership (Figure S2). This was observed in all three sampling seasons regardless of the predefined number of clusters (*k*) set in the analysis and (Figure S2). We also detected significant but weak isolation-by-distance between individuals across the city (Mantel *r *=* *0.032, *P *=* *0.001). However, after removing individuals from Paquetá island, no isolation-by-distance was evident (Mantel *r *=* *0.019, *P *=* *0.090).

Group-based analysis also revealed low overall spatial structuring for the nuclear variation in samples collected across all three seasons (Table S3). In the 2011 dataset, *G*_ST_ for genomewide SNPs was 0.014 (*P *>* *0.05) and *G*_ST_ for microsatellites was 0.008 (*P *=* *0.002). Sample from Paquetá island showed the highest pairwise *G*_ST_ values, but only one of 105 pairwise comparisons in the entire 2011 microsatellite dataset remained statistically significant after Bonferroni correction (Table S3). Power analysis indicated that our microsatellite marker set had an acceptable type I error rate (*а *= 0.051) and a 100% power to detect *G*_ST_ ≥ 0.008 based on sample sizes from our 2011 collection. Sample sizes of the 2012 and 2013 collections (Table1) decreased the power to detect weak structure to 65% and 30%, respectively.

Despite a very low level of spatial structuring for the entire nuclear marker set, 2.7% of nuclear SNPs (40/1496) had highly significant *G*_ST_ estimates (*P* ≪ 0.001) that ranged between 0.065 and 0.261 (Table4). Two-thirds of these SNPs (26/40) are located in 26 genes that have a function in nucleic acid, protein and ion binding, transmembrane and neuropeptide signaling, and in protein kinase and oxidoreductase activity (Table4). All but one of these genes (25/26) have a significantly altered expression under experimental conditions such as blood meal and infection with pathogens (dengue viruses, bacteria), including the novel biocontrol agent *Wolbachia* (*w*Mel and *w*MelPop), as well as after exposure to insecticides and xenobiotics (Table4).

**Table 4:** Within-gene single nucleotide polymorphisms that exhibited significant spatial structuring (*G*_ST_ with *P* ≪ 0.001) in *Aedes aegypti* from Rio de Janeiro

*G*_ST_	Gene ID	Molecular function (GOSlim terms)	Experimental factor
0.261	AAEL018040	Transporter activity, ATP binding	Xenobiotic,* insecticides,†‡ bacterial infection,§ blood meal¶
0.239	AAEL008354	Extracellular ligand-gated ion channel activity	Xenobiotic,* insecticides,† bacterial infection,§ blood meal,¶** wMelPop infection††
0.222	AAEL008961	Nucleic acid binding, metal ion binding	Bacterial infection§
0.218	AAEL001939	Nucleic acid binding, ATP binding	Xenobiotic,* bacterial infection,§ blood meal,** wMel/wMelPop infection‡‡
0.218	AAEL001996	Nucleic acid binding, zinc binding	Xenobiotic,* bacterial infection,§ blood meal,** wMel/wMelPop infection‡‡
0.215	AAEL001399	Protein binding	Bacterial infection,§ blood meal,¶** wMelPop infection,†† dengue infection§§
0.212	AAEL009666	DNA binding, ion binding, methyltransferase activity	Insecticides,†‡ blood meal¶**
0.207	AAEL002055	Neuropeptide signaling	Xenobiotic,* insecticides,† bacterial infection,§ blood meal¶**
0.201	AAEL009424	Protein serine/threonine kinase activity, ATP binding	Xenobiotic,* blood meal**
0.192	AAEL011309	Orotidine-5′-phosphate decarboxylase activity	Xenobiotic,* insecticides,† bacterial infection,§ blood meal,¶** wMel/wMelPop infection‡‡¶¶
0.192	AAEL009063	?	Blood meal,¶ wMelPop infection¶¶
0.190	AAEL002852	?	Xenobiotic,* insecticides,‡ bacterial infection,§ blood meal¶
0.170	AAEL003415	Structural molecule activity (lamin)	Xenobiotic,* insecticides,‡ bacterial infection,§ blood meal**
0.166	AAEL002924	Binding	–
0.164	AAEL002798	Ion binding, oxidoreductase activity	Insecticides,† bacterial infection§
0.160	AAEL018040	ATP binding, ATPase activity	Xenobiotic,* insecticides,†‡ bacterial infection,§ blood meal¶
0.160	AAEL018133	?	Xenobiotic,* insecticides,† bacterial infection,§ blood meal,¶** wMelPop infection††
0.154	AAEL005929	ATPase activity, transmembrane transporter activity	Xenobiotic,* insecticides,†‡ bacterial infection,§ blood meal,¶** wMel/wMelPop infection††‡‡
0.151	AAEL013811	Lysophospholipase activity	Blood meal,** circadian***
0.132	AAEL000682	?	Xenobiotic,* insecticides,† bacterial infection,§ blood meal¶**
0.131	AAEL007258	Protein binding	Blood meal¶**
0.114	AAEL006301	Protein binding, metal ion binding	Insecticides,‡ blood meal,¶ wMel/wMelPop infection‡‡
0.091	AAEL008318	Calcium ion binding	Xenobiotic,* wMelPop infection,¶¶ circadian***
0.088	AAEL012395	ATPase activity, transmembrane transporter activity	Xenobiotic,* insecticides,† bacterial infection,§ blood meal,¶ circadian***
0.087	AAEL009305	ATP binding, protein kinase activity	Xenobiotic,* insecticides‡
0.065	AAEL004480	Protein binding (cell division cycle 20)	Bacterial infection,§ blood meal¶

?: unknown gene function; –: no experimental data.

Gene ID and ontology terms follow the annotation from VectorBase. Significantly altered expression has been recorded under conditions that are listed as experimental factors.

^*^Poupardin et al. (2012), †Riaz et al. (2013), ‡Tetreau et al. (2012), §Choi et al. (2012), ¶Dissanayake et al. (2010), ^*^^*^Bonizzoni et al. (2011), ††Ye et al. (2013), ‡‡Rancès et al. (2012), §§Behura et al. (2014), ¶¶Kambris et al. (2009), ^*^^*^^*^Ptitsyn et al. (2011).

## Discussion

We report novel insights into the complex pattern of genetic diversity and spatial genetic structuring in *Ae. aegypti* from Rio de Janeiro as revealed by multiple marker systems: RAD-seq generated SNPs in the mitochondrial and nuclear genomes, and microsatellites. We found several divergent mitochondrial lineages and strong spatial structure for mitochondrial variation, in contrast to the overall homogeneity in nuclear markers across Rio. Despite a low overall differentiation for the nuclear markers, we detected strong spatial structuring for variation in over 20 genes that have a significantly altered expression under the exposure to insecticides, xenobiotics, and pathogens, including the novel biocontrol agent *Wolbachia*.

### Low nuclear but high mitochondrial spatial structure

The various analyses we carried out such as spatially explicit Bayesian clustering, DAPC, isolation-by-distance, and *G*_ST_ comparisons all indicated very weak spatial structuring of nuclear variation despite the highly heterogeneous landscape of the Rio de Janeiro city (including mountainous and coastal regions, slums, urban, and rural areas). One exception was the geographically isolated Paquetá Island (Fig.1) that showed significant differentiation from the rest of the city. These results suggest extensive mixing of *Ae. aegypti* across Rio unlike patterns detected in previous reports (da Costa-Ribeiro et al. 2006a, b), but they are consistent with patterns found in another city and a region in Brazil (Campos et al. 2012; Mendonça et al. 2014). Mitochondrial variation, on the other hand, had a very strong spatial structuring, with only one haplotype shared among locations considered here. Contrasting spatial pattern between mitochondrial and nuclear markers (i.e. mito-nuclear discordance) is not rare in animals, including insect pests (Sun et al. 2015), but is generally found across much broader geographic scales (Toews and Brelsford 2012).

### Potential drivers of mito-nuclear discordance in *Aedes aegypti* from Rio

Endosymbionts like *Wolbachia* can provide a strong mechanism underlying the discordant spatial patterns between mitochondrial and nuclear variation in many insects (Toews and Brelsford 2012). Because *Wolbachia* and mitochondria are both maternally inherited, reproductive incompatibilities caused by *Wolbachia* infections (e.g. cytoplasmic incompatibility) influence the population dynamics of mitochondria (Turelli et al. 1992). Even though *Wolbachia* are not natively found in *Ae. aegypti*, we did confirm the *Wolbachia*-free status of *Ae. aegypti* in Rio. We tested 683 individuals from our 2011 collection using the quantitative real-time PCR/high-resolution melt assay for *Wolbachia* detection (Lee et al. 2012) and found no signs of *Wolbachia* infection in Rio.

Because the mitochondrial genome is haploid and uniparentally inherited in most animals, it has a smaller effective population size than the nuclear genome (Hudson and Turelli 2003). Locally varying population dynamics and stochastic events are reflected in an increased genetic structuring for the mitochondrial markers when compared to nuclear markers, particularly in the early stages of the population divergence (Larsson et al. 2009). Local samples of from Rio had different estimates of effective population size (*θ*) and parameters of demographic change (Tajima's *D, R*_2_) for the mitochondrial genome but not for the nuclear genome (Table2).

*F*_ST_-like indices should be up to four times higher for mitochondrial than for nuclear markers when populations are diverging under a relatively high gene flow (Larsson et al. 2009). This ratio between the two marker sets (mtDNA *F*_ST_/nuclear *F*_ST_) is much greater than four if dispersal is sex-biased such that males show higher dispersal propensity, and much smaller than four if females are the dispersing sex (Karl et al. 2012). In our dataset, the *G*_ST_ ratio for mtDNA and nuclear SNPs was 11.81 (and 9.59 for mtDNA and microsatellites), suggesting that *Ae. aegypti* males in Rio may have higher dispersal than females.

Male-biased dispersal in *Ae. aegypti* has not been detected in mark–release–recapture (MRR) studies conducted in Rio or elsewhere (Harrington et al. 2005; Maciel-de-Freitas et al. 2007; Valerio et al. 2012). However, the marking methods and fluorescent dusts in MRR experiments can have a significant impact on differential survival of males and females in *Ae. aegypti* (Dickens and Brant 2014), biasing the estimates of dispersal in the two sexes. Moreover, genetic parameters such as *G*_ST_ measure effective dispersal that leads to gene flow, unlike the ecological (MRR) parameters. Perhaps *Ae. aegypti* females that disperse far have a lower reproductive output than females that remain local, while this is not the case in males. Some evidence in support of this hypothesis comes from the MRR experiments that recorded lower daily survival of females in parts of Rio where they showed higher dispersal propensity (Maciel-de-Freitas et al. 2007). These results warrant further investigation where patterns of genetic relatedness in males and females could be compared, which was not possible in our study because we used nonsexed larvae.

Human-assisted introductions are another mechanism contributing to mito-nuclear discordance in animals (Toews and Brelsford 2012). Multiple reintroductions of *Ae. aegypti* into Brazil from neighboring countries have been suggested in previous studies based on an analyses of mitochondrial genes (COI, ND4) and microsatellite markers. These studies pointed to two divergent mitochondrial clades (Bracco et al. 2007; Scarpassa et al. 2008; Paduan and Ribolla 2009) and two major nuclear genetic groups in larger regions of Brazil (Monteiro et al. 2014). In this study, we found four mitochondrial clades in Rio de Janeiro alone, suggesting that the use of more markers across the mitochondrial genome as in the current study is likely to reveal an additional level of complexity in *Ae. aegypti* introductions into Brazil. As a comparison, in the city of Yogyakarta, Indonesia, we found only one mitochondrial clade using the same ddRAD approach to screen samples of comparable sizes collected at the same spatial scale (G. Rašić, unpublished data). This suggests that the large ‘gaps’ between clades found in Rio are unlikely to represent missing haplotypes due to insufficient sampling.

We also found an overall deficit of rare SNPs across the nuclear genome of *Ae. aegypti* from Rio. Introductions from diverse sources coupled with extensive gene flow can lead to these patterns of genetic diversity in invasive populations (Cutter et al. 2012; Lockwood et al. 2013). Positive Tajima's *D* values can also be interpreted as evidence for population balancing selection, bottlenecks, and/or cryptic population structure (Tajima 1989). A strong influence of balancing selection is unlikely in our dataset, given that Tajima's *D* remained positive when only regions outside genes were analyzed. Also, the level of nuclear structuring we observed was too low to cause a substantial artifact due to sample pooling (and separate analyses for each of the local samples also gave positive Tajima's *D* values). In a long-term survey of *Ae. aegypti* abundance in Rio, there was no major decrease in mosquito numbers (Figure S3), suggesting that bottlenecks are not strong. Furthermore, a dramatic decline in effective population size was not detected in our microsatellite bottleneck tests (Table S4). The pattern of genomewide SNP diversity observed in *Ae. aegypti* from Rio therefore appears most consistent with complex re-introductions combined with high subsequent gene flow.

Despite high gene flow in *Ae. aegypti* across Rio, we detected local genetic structure for variation in 25 genes with a putative role in the mosquito immune response and insecticide resistance (Table4). *Aedes aegypti* in Rio could be responding to locally varying intensities of insecticide application and pathogen load that are strong enough to counteract the effect of high gene flow, but the analyses of selection signatures on a more comprehensive dataset are needed to adequately test this hypothesis.

### Implications for the control of *Aedes aegypti* in Rio

For the past 40 years, the Brazilian National Program for Dengue Control has been using insecticides of the organophosphate and pyrethroid classes as the main strategy to control *Ae. aegypti* (Lima et al. 2011). Due to intensive insecticide application, *Ae. aegypti* populations evolved resistance that is now widespread across all regions of Brazil (Montella et al. 2007). New agents, such as *Bacillus thuringiensis israelensis* toxins (*Bti*), have been replacing organophosphates in Rio de Janeiro states since 2001 (Lima et al. 2005). Nevertheless, the control of *Ae. aegypti* populations that intensifies after every new dengue epidemic has generally had a very limited success in suppressing the vector populations or dengue transmission. As an example, recent efforts in the city of Boa Vista not only failed to significantly reduce the mosquito numbers, but led to a rapid increase in pyrethroid resistance even in the adjacent areas that were not directly targeted (Maciel-de-Freitas et al. 2014).

Our study elucidates processes that set limits to the effectiveness of traditional control measures in Rio and possibly other parts of Brazil. *Aedes aegypti* in Rio harbors high genetic diversity and populations experience spatially unconstrained admixing that is likely mediated by effective male dispersal, but also exhibits locally heterogeneous genetic variation that could affect insecticide resistance. Resistance to organophosphates and pyrethroids in populations from Brazil has been associated with higher fitness cost (Belinato et al. 2012; Brito et al. 2013), limiting the spread of resistance variants into areas that are not under intense insecticide applications. However, the level of gene flow we found in Rio would allow for a rapid spread of resistance if the intensity of insecticide application increases locally. We detected significant spatial structure in the face of high gene flow for genes that have an altered expression in *Ae. aegypti* that are resistant to or exposed to insecticides such as imidacloprid, permethrin, and *Bti* (Table4). Even though resistance to *Bti* has not been reported in *Ae. aegypti* from Brazil (Araújo et al. 2013), strong spatial structure for several genes that respond to this insecticide could indicate locally selected variants. These findings suggest that it would be worthwhile monitoring and comparing insecticide resistance in *Ae. aegypti* collected in different parts of Rio.

We also found spatial heterogeneity for variation in genes showing differential expression under the infection with bacteria and dengue viruses, or after a blood meal (Table4), and these genes could affect the vectorial capacity of *Ae. aegypti*. A recent study found differential vectorial capacity for chikungunya viruses (CHIKV) in *Ae. aegypti* from different parts of Rio (Vega-Rúa et al. 2014). For example, mosquitoes from Paquetá island and east bay (Jurujuba) showed higher transmission efficacy for CHIKV than mosquitoes from the west bay (Vega-Rúa et al. 2014). Based on that study and our results, a fine scale assessment of *Ae. aegypti*'s vectorial capacity for dengue viruses in Rio may be worthwhile.

Our study also provides useful information for the implementation of novel biocontrol strategies like those that use *Wolbachia*-infected *Ae. aegypti* for population replacement or suppression (Hoffmann et al. 2015). If female dispersal is limited, the potential for the spread of *Wolbachia* infection outside the release zone may be restricted because emigrant individuals are more likely to be males that do not transmit this maternally inherited endosymbiont. However, with male-biased dispersal, the release of *Wolbachia*-infected males for population suppression may be worth considering (Hancock et al. 2011). In the absence of strong spatial variation in nuclear genes, it may be possible to release mosquitoes in any part of the city with a nuclear background derived from one Rio population rather than being concerned about local backgrounds (Hoffmann et al. 2015). However, with strong spatial structure for genes involved in responses to insecticides and *Wolbachia* infection (Table4), it would seem prudent to check for phenotypic effects in resistance and endosymbiont parameters that might vary locally as a consequence of population variation.

In conclusion, using multiple marker systems we uncovered a complex genetic structure of *Ae. aegypti* in Rio de Janeiro, reflecting underlying processes that limit the effectiveness of local measures to control the mosquito populations. These factors need to be considered in future *Wolbachia* releases, and they contrast with patterns found in other parts of the world. The data we have collected also point to some intriguing patterns of differentiation in genes that may influence the local success of release programs.

## Data archiving statement

Illumina sequences for the ddRADseq data are archived within the NCBI SRA under accession numbers: PRJNA241150, PRJNA273913. All other data are available as the Supporting Information files in the online version of the manuscript.

## Supporting Information

**Figure S1** DAPC analysis with nuclear genome-wide SNPs in *Aedes aegypti* from Rio de Janeiro, with the BIC values for the successive number of genetic groups (upper) and the alpha-score optimization process for retaining the optimal number of PCs in the analysis (lower). **Figure S2**. TESS results. **Figure S3.** Mosquito numbers. **Table S1.** Sampling coordinates and microsatellite genotype data. **Table S2.** List of *Aedes aegypti* mitochondrial genes with ddRAD loci. **Table S3.***F*_ST_. Pairwise *F*_ST_ (bellow diagonal) and the corresponding *P*-values (from 999 permutations) across eight microsatellite loci among samples of *Aedes aegypti* collected from 15 location in Rio de Janeiro during wet season 2011. **Table S4.**BOTTLENECK analysis.

**Files S1 and S2.** Relaxed phylip format files used in RAxML. Unique nuclear and mitochondrial SNPs concatenated into the sequences used for the ML phylogenetic analysis.

**File S3.**VCF for nuclear SNPs.

**File S4.** Amplicon sequences from mitochondrial genes COXI and ND5 in *Ae. aegypti*.

**File S5.**RADseq generated mtDNA sequences.

**File S6.**jModelTest-2.1.7 results.
