# Supplementary material for: Contrasting genetic structure between mitochondrial and nuclear markers in the dengue fever mosquito from Rio de Janeiro: implications for vector control
Source: Evol Appl. 2015 Sep 7;8(9):901–15. doi: 10.1111/eva.12301 (PMC4610386; doi:10.1111/eva.12301)
Supplement: Supplementary file 1 — Figure S1 DAPC analysis with nuclear genome-wide SNPs in Aedes aegypti from Rio de Janeiro, with the BIC values for the successive number of genetic groups (upper) and the alpha-score optimization process for retaining the optimal number of PCs in the analysis (lower). Figure S2. TESS results. Figure S3. Mosquito numbers. Table S1. Sampling coordinates and microsatellite genotype data. Table S2. List of Aedes aegypti mitochondrial genes with ddRAD loci. Table S3.FST. Pairwise FST (bellow diagonal) and the corresponding P-values (from 999 permutations) across eight microsatellite loci among samples of Aedes aegypti collected from 15 location in Rio de Janeiro during wet season 2011. Table S4.BOTTLENECK analysis. [file eva0008-0901-sd1.pdf]

S1 Fig. DAPC analysis with nuclear genome-wide SNPs in *Aedes aegypti* from Rio de Janeiro, with the BIC values for the successive number of genetic groups (upper) and the alpha-score optimization process for retaining the optimal number of PCs in the analysis (lower).

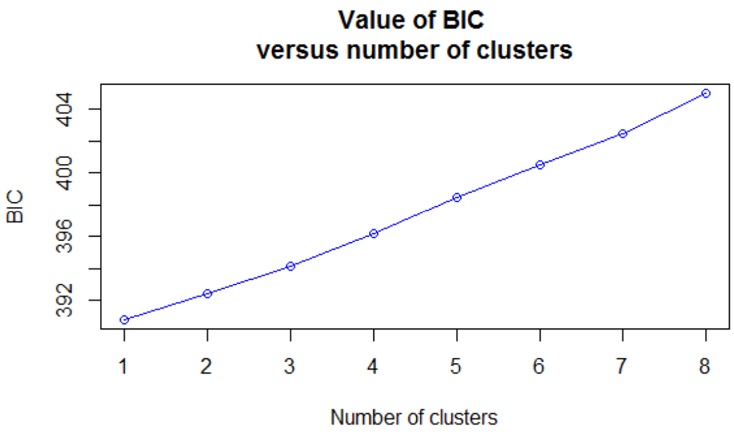

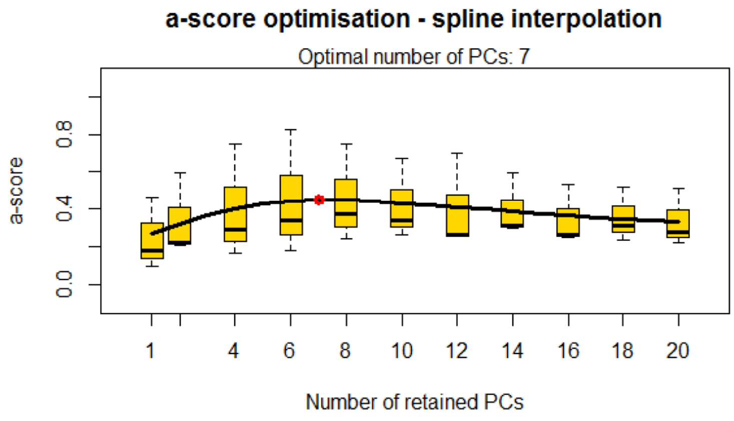

S2 Fig. TESS analysis for the microsatellite datasets from *Aedes aegypti* collected in Rio de Janeiro across three seasons. Probabilities for individual membership to a different number of genetic clusters (K=2,3, or 5) are depicted as vertical lines.

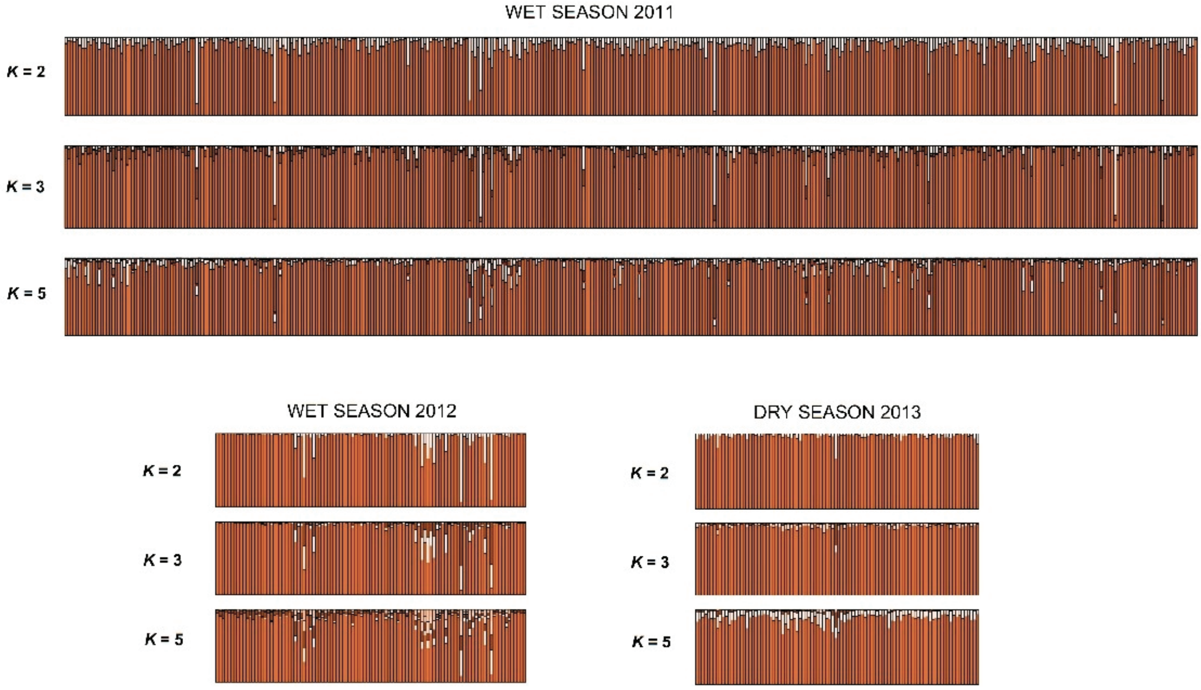

S3 Fig. Average number of *Aedes aegypti* females caught daily in BG traps at four locations in Rio de Janeiro over a period of 95 weeks (since October 2012).

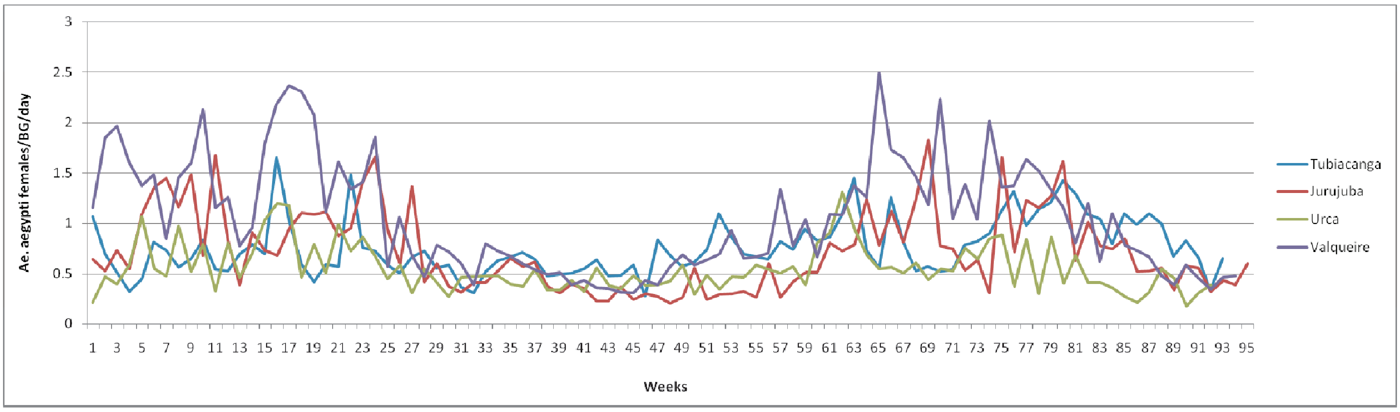

S2Table. List of *Aedes aegypti* mitochondrial genes with ddRAD loci, their orientation in the mitochondrial genome, start codon position within the recovered sequences and the mitochondrial genome (in base-pairs). Fragment lengths greater than 80 bp were reconstructed by concatenating the partly overlapping paired Illumina reads.

gene	gene orientation	codon start position in the sequence	sequence start position in the genome (bp)	fragment length (bp)
ND2	+	2	357	80
		2	606	82
COX1	+	3	1317	80
		2	2128	97
		1	2393	125
		3	2592	80
ATP6	+	2	4255	145
COX3	+	3	4758	163
		1	5255	99
ND5	-	2	6329	80
		3	6675	80
		2	7277	80
		2	7490	80
		3	7746	80
		2	7830	82
ND4	-	3	8903	80
ND4L	-	na	9422	80
		na	9522	80
ND6	+	2	9962	109
CYTB	+	2	10457	144
		3	10831	80
ND1	-	3	11808	80
		1	11908	80

S3 Table. Pairwise F_{ST} (bellow diagonal) and the corresponding *p*-values (from 999 permutations) across eight microsatellite loci among samples of *Aedes aegypti* collected from 15 location in Rio de Janeiro during wet season 2011. Location codes are listed in Table 1.

	**PA**	**JG**	**HM**	**JU**	**MI**	**OL**	**PQ**	**PV**	**PI**	**SC**	**TQ**	**TB**	**UR**	**VQ**	**VL**
**PA**		0.486	0.038	0.082	0.076	0.039	0.039	0.013	0.288	0.094	0.029	0.003	0.058	0.076	0.184
**JG**	0.011		0.853	0.161	0.881	0.874	0.334	0.344	0.717	0.582	0.113	0.744	0.884	0.793	0.578
**HM**	0.027	0.012		0.128	0.649	0.558	0.039	0.119	0.051	0.320	0.137	0.235	0.754	0.406	0.146
**JU**	0.024	0.023	0.031		0.021	0.141	0.097	0.029	0.006	0.498	0.246	0.064	0.280	0.162	0.144
**MI**	0.014	0.007	0.012	0.028		0.330	0.026	0.667	0.643	0.671	0.125	0.135	0.794	0.390	0.624
**OL**	0.015	0.007	0.014	0.021	0.010		0.002	0.272	0.297	0.389	0.015	0.120	0.555	0.210	0.522
**PQ**	0.022	0.015	0.031	0.027	0.021	0.026		0.002	0.003	0.035	0.005	0.021	0.005	0.009	0.004
**PV**	0.017	0.012	0.021	0.027	0.007	0.010	0.035		0.368	0.618	0.145	0.017	0.725	0.205	0.708
**PI**	0.010	0.008	0.023	0.029	0.007	0.009	0.024	0.009		0.405	0.004	0.008	0.185	0.051	0.318
**SC**	0.015	0.010	0.017	0.015	0.008	0.010	0.021	0.008	0.009		0.342	0.030	0.780	0.545	0.876
**TQ**	0.019	0.018	0.024	0.021	0.015	0.021	0.027	0.014	0.022	0.012		0.002	0.143	0.187	0.033
**TB**	0.018	0.008	0.017	0.021	0.011	0.011	0.020	0.016	0.016	0.016	0.023		0.169	0.093	0.101
**UR**	0.014	0.006	0.011	0.017	0.006	0.008	0.025	0.006	0.010	0.007	0.014	0.010		0.748	0.645
**VQ**	0.014	0.008	0.015	0.020	0.009	0.011	0.024	0.011	0.013	0.009	0.013	0.012	0.006		0.890
**VL**	0.012	0.010	0.020	0.021	0.008	0.009	0.027	0.007	0.009	0.006	0.018	0.012	0.007	0.006	

S4 Table. Potential reductions in effective population size of *Aedes aegypti* from Rio de Janeiro were tested with the program BOTTLENECK (Luikart *et al.* 1999) using microsatellite datasets from three collections seasons (Wet 2011, Wet 2012, Dry 2013), assuming the two phase model of microsatellite evolution.

	wet 2011							wet 2012							dry 2013						
locus	n	ko	He	Heq	S.D.	DH/sd	Prob	n	ko	He	Heq	S.D.	DH/sd	Prob	n	ko	He	Heq	S.D.	DH/sd	Prob
AG5	812	8	0.738	0.667	0.102	0.696	0.278	206	7	0.729	0.671	0.104	0.566	0.333	202	7	0.728	0.674	0.101	0.529	0.346
BbH08	802	3	0.646	0.308	0.186	1.818	0.009	204	3	0.638	0.353	0.177	1.614	0.021	202	3	0.66	0.357	0.174	1.74	0.007
BbA10	792	8	0.585	0.673	0.104	-0.838	0.165	206	6	0.683	0.623	0.118	0.504	0.368	202	8	0.746	0.71	0.087	0.414	0.406
AC1	810	10	0.602	0.729	0.083	-1.534	0.079	206	5	0.518	0.561	0.136	-0.312	0.305	202	5	0.625	0.561	0.129	0.492	0.361
M201	792	4	0.505	0.427	0.17	0.457	0.42	206	4	0.527	0.467	0.16	0.376	0.426	194	4	0.591	0.479	0.156	0.717	0.257
12ACG1	774	2	0.242	0.163	0.168	0.471	0.305	206	3	0.333	0.35	0.178	-0.097	0.435	202	3	0.296	0.357	0.176	-0.344	0.352
69TGA1	768	5	0.555	0.509	0.154	0.294	0.448	186	4	0.446	0.476	0.16	-0.19	0.355	202	5	0.539	0.557	0.137	-0.126	0.378
BbB19	802	6	0.54	0.581	0.128	-0.316	0.308	194	5	0.47	0.566	0.134	-0.716	0.199	192	5	0.517	0.554	0.138	-0.27	0.315

SIGN TEST
Assumptions: all loci fit T.P.M., mutation-drift equilibrium
Expected number of loci with heterozygosity excess: 4.44 (Wet 2011); 4.62 (Wet 2012); 4.63 (Dry 2013)
loci with heterozygosity deficiency: 3; 4; 3
loci with heterozygosity excess: 5; 4; 5
Probability: 0.49006; 0.45910; 0.54354

WILCOXON TEST
Probability (one tail for H deficiency): 0.67969 (Wet 2011); 0.76953 (Wet 2012); 0.98047 (Dry 2013)
Probability (one tail for H excess): 0.37109; 0.27344; 0.02734

MODE-SHIFT
0.0 0.1 0.2 0.3 0.4 0.5 0.6 0.7 0.8 0.9 1.0
| 0.565 | 0.152 | 0.065 | 0.022 | 0.065 | 0.043 | 0.065 | 0.000 | 0.022 | 0.000 | (Wet 2011)
| 0.486 | 0.189 | 0.054 | 0.054 | 0.054 | 0.054 | 0.027 | 0.054 | 0.027 | 0.000 | (Wet 2012:)
| 0.475 | 0.200 | 0.050 | 0.050 | 0.125 | 0.025 | 0.050 | 0.000 | 0.025 | 0.000 | (Dry 2013)
normal L-shaped distribution for all three seasons.
