# Supplementary material for: Contrasting genetic structure between mitochondrial and nuclear markers in the dengue fever mosquito from Rio de Janeiro: implications for vector control
Source: Evol Appl. 2015 Sep 7;8(9):901–15. doi: 10.1111/eva.12301 (PMC4610386; doi:10.1111/eva.12301)
Supplement: Supplementary file 3 — Files S1 and S2. Relaxed phylip format files used in RAxML. Unique nuclear and mitochondrial SNPs concatenated into the sequences used for the ML phylogenetic analysis. [file eva0008-0901-sd3.docx]

63 92

Br30-1 GATCAAATTATCGTACTCAAAAATTGAGTGTTAGCTCTTCGTTGGTGGTAGTATTACACAGCAAACTTCATTATAGGATCTGTCCCTGTTTT

Br22-1 NNNNNAATTATCGTACTCAAAAATTGAGTGTTAGCTCTTCGTTGGTGGTAGTATTACACAGCAAACTTCATTATAGGATCTGTCCCTGTTTT

Br22-2 AATCANNNNNTCNNNCTCAAAAATCGAGTATTAGCTCTTCGNTGGTGGTGATATTACGTGGCAAACNNNNNNATAGGATCTGTACCTATCTT

Br26-2 GATCAAATTATCGTACTCAAAAATTGAGTGTTAGCTCTTCGTTGGTGGTAGTATTANNNAGCAAACTTCATTATAGGATCTGTCCCTGTTTT

Br30-2 NNNNNAATTATCGTACTCAAAAATTGAGTGTTAGCTCTTCGTTGGTGGTAGTATTANNNAGCAAACTTCATTATAGGATCTGTCCCTGTTTT

Br32-1 GATCAAATTATCGTACTCAAAAATTGAGTGTTAGCTCTTCGTTGGTGGTAGTATTACATAGCAAACTTCATTATAGGATCTGTCCCTGTTTT

Br32-2 NNNNNAATTATCGTACTCAAAAATTGAGTGTTAGCTCTTTGTTGGTGGTAGTATTACACAGCAAACTTCATTATAGGATCTGTCCCTGTTTT

Br47-1 GATCANNNNNCCGCACTTAAAAACTGAGCACTAATTCTCTGTTGGTGATAANNNNNCACAGCGGACTTTATTATAGGTTCTGTATATATTCT

Br48-1 AATCAAATTATCACACTCAAAAATCGAGTATTAGCTCTTCGTTGGTGGTGATATTACGTGGCAAACTTCGTCATAGGATCTGTACCTATTTT

Br53-1 GATCAAATTATCGCACTTAAAAATTGAGTATTAGCTCCTCGTTGGTAATAATGTCACATAGCAGATTTCATTATAGGATTTGTACATACTTT

Br57-1 NNNNNAATTATCGTACTCAAAAATTGAGTGTTAGCTCTTCTTTGGTGGTAGTATTACACAGCAAACTTCATTATAGGATCTGTCCCTGTTTT

BrK02-1 AATCAAATTATCACACTCAAAAATCGAGTATTAGCTCTTCGNNNNNGGTGATATTACGTNNCAAACTTCGTCATAGGATCTGTACCTATTTT

BrK11-1 AATCAAATTATCACACTCAAAAATCGAGTATTAGCTCTTCGTTGGTGGTGATATTACGTGGCAAACTTCATCATAGGATCTGTACCTATTTT

BrK47-1 AACCAAATTATCACACTCAAAAATCGAGTATTAGCTCTTCGTTGGTGGTGATATTACGTGGCAAACTTCGTCATAGGATCTGTACCTATTTT

BrK55-1 GATCGAATTATCGTACTCAAAAATTGAGTGTTAGCTCTTCGTTGGTGGTAGTATTANNNAGCAAACTTCATTATAGGATCTGTCCCTGTTTT

BrM02-1 AATCAAATTATCACACTCAAAAATCGAGTATTAGCTCTTCGTTGGTGGTGATATTACGTGGCAAACTTCGTCATGGGATCTGTACCTATTTT

BrM03-1 AATCAAATTATCACACTCAAAAATCGAGTATTAGCTCTTCGTNNNNGGTGATATTACGTNNCAAACTTCGTCATAGGATCTGTACCTATTTT

BrM11-1 AATCAAATTATCACACTCAAAAATCGAGTATTAGCTCTTCGTTGGTGGTGATATTACGTGGCAAACCTCGTCATAGGATCTGTACCTATTTT

BrM17-1 AATCAAATTATCNNNCTCAAAAATCGAGTATTAGCTCTTCGTTGGTGGTGATATTACGTGGCAAACTTCATTATAGGATCTGTACCTATTTT

BrM21-1 GATCAAATTACCGCACTTAAAAACTGAGCACTAATTCTCTGTTGGTGATAATATTACACAGCGGACTTTATTATAGGTTCTGTATATATTCT

BrM22-1 GATCANNNNNCCGCACTTAAAAACTGAGCACTAATTCTCTGTTGGCGATAATTTTACACAGCGGACTTTATTATAGGTTCTGTATATATTCT

BrM23-1 AATCAAATTATCGTACTCAAAAATTGAGTGTTAGCTCTTCGTTGGTGGTAGTATTACGTAGCAAACTTCATTATAGGATCTGTCCCTGTTTT

BrM26-1 AATCAAATTATCACACTCAAAAATCGAGTATTAGCTCTTCGTTGGTGGTGATATTACGTGGCAAACTGCGTCATAGGATCTGTACCTATTTT

BrM31-1 AATCAAATTATCACACTCAAAAATCGAGTATTAGCTCTTCGTTGGTGGTGATATTACGTGGCAAACTTCGCCATAGGATCTGTACCTATTTT

BrM45-1 AATCAAATTACCNNNCTCAAAAATCGAGTATTAGCTCTTTGNTGGTGGTGATATTACGTGGCAAACTTCATTATAGGATCTNNATCTATTTT

BrM48-2 GATCGAATTATCGTACTCAAAAATTGAATGTTAGCTCTTCGTTGGTGGTAGTATTANNNNNCAAACTTCATTATAGGATCTGTCCCTGTTTT

BrM51-1 AATCAAATAATCACACTCAAAAATCGAGTATTAGCTCTTCGTTGGTGGTGATATTACGTGGCAAACTTCGTCATAGGATCTGTACCTATTTT

BrM54-1 GATCANNNNNCCGCACTTAAAAACTGAGCACTAATTCTCTGTTGGTGATAANNNNNCACNNCGGACTTTATTATAGGTTCTGTATATATNNN

BrM55-1 AATCAAATTATCACACTCAAAAATCGAGTATTAGCTCTTCGTNNNNGGTGATATTACGTGGCAAACTTCGTCATAGGATCTGTACCTATTTT

BrM56-1 AATCANNNNNTCACACTCAAAAATCGAGTATTAGCTCTTCGTTGGTGGTGATATTACGTNNCAAACTTCATCATAGGATCTANACCTATTTT

BrM59-1 GATCGAATTATCNTACTCAAAAATTGAGTGTTAGCTCTTCGTCGGTGGTAGTATTACACNNCGAACTTCATTATAGGATCTGTCCCTGTTTT

BrM60-3 AATCAAATTATCACACTCAAAAATCGAGTATTAGCTCTTCGTTGGTGGTGANNNNNCGTGGCAAACTTCGTCATAGGATCTGTACCTATTTT

Gv10-A-01 GATCAAACTACCGCATTTAAAAACTGAGTACCAACTCTCTGTTGGTGACAATTTTACACNNCGGACTTCATTATAGATTCTGTATATATCTT

Gv10-A-08 GATCGNNNNNTCNTACTCAAAAATTGAGTGTTAGCTCTTCGNTGGTGGTAATATTATGTAGCAAACTTCATTATAGGATCTGTCCCTGTTTT

Gv10-A-10 GATCAAACTACCNNNTTTAAANNNNNNGTACCAACTCTCTGNTGGTGACAATTTTACACAGCGGACTTCATTATAGATTCTGCATATATCTT

Gv10-B-02 GATCAAACTACCGCATTTAAAAATTGGGTACCAACTCTCTGNTGGTGACAATTTTACACAGCGGACTTCATTATAGATTCTGTATATATCTT

Gv10-B-03 GATCAAGCTACCGCATTTAAAAATTGAGTACCAACTCTCTGTTGGTGACAATTTTACACAGCGGACTTCATTATAGATTCTGTATATATCTT

Gv10-B-06 GATCAAACTACCGCATTTAAAAACTGAGTACCAACTCTCTGTTGGTGACAATTTTACACAGCGGACTTCATTATAGATTCTGTATATATCTT

Gv10-C-01 GATCAAACTACCGCATTTAAAAACTGAGTACCAACTCTCTGTTCGTGACAATTTTACACAGCGGACTTCATTATAGATTCTGTATATATCTT

Gv10-C-02 AATCAAACTACCGCATTTAAAAACTGAGTACTAACTNNNTGTTGGTGACAATTTTACACAGCGGACTTCATTATAGGTTCTGTATATATCTT

Gv10-E-05 GATCAAACTACCGCATTTAAANNNNNNGTACCAACTCTCTGTTGGTGACAATTTTACACAGCGGACTTCATTATAGATTCTGTATATATCTT

Gv10-F-08 GATCAAACTACCGCATTTAAAAATTGAGTACCAACTCTCTGTTGGTGACAATTTTACACAGCGGACTTCATTATAGATTCTGTATATATCTA

Gv10-F-10 GATCGAATTATCGTACTCAAAAATTGAGTGTTAGCTCTTCGTTGGTGGTAATATTANNNAGCAAACTTCATTATAGGATCTGTCCCTGTTTT

Gv10-H-03 AATCAAACTACCGCATTTAAAAGCTGAGTACTAACTCTCTGTTGGTGACAATTTTACACAGCGGACTTCATTATAGATTCTGTATATATCTT

In501a GATCAAACTACCGCATTTAAAAACTGAGTACTAACTCTCTGTTGGTGACAATTTTACACAGCGGACTTCATTATAGGTTCTGTATATATCTT

In501 GATCAGACTACCGCATTTAAAAATTGGGTACTAACTCTCTGTTGGTGACAATTTTACACAGTGGACTTTATTGTAGGTTCCGTATATATCTT

In521 GATCAAACTACCGCATTTAAAAACTGAGTACTGACTCTCTGTTGGTGACAATTTTACACAGCGGACTTCATTATAGGTTCTGTATATATCTT

In526 GGTCAGACTACCGCATTTAAAAACTGAGTACTAACTCTCTGTTGGTGACAATTTTACACAGTGGACTTTATTGTAGGTTCCGTATATATCTT

In534 GATCAAACTACCGCATTTTAAAACTGAGTACTAACTCTCTGTTGGTGACAATTTTACACAGCGGACTTCATTATAGGTTCTGTATATATCTT

In535 GGTCAGACTACCGCATTTAAAAACTGAGTACTAACTCTCTGTTGGTGACAACTTTGCACAGTGGACTTTATTGTAGGTTCCGTATATATCTT

In553 GATCAAACTACCGCATGTTAAAACTGAGTACTAACTCTCTGTTGGTGACAATTTTACACAGCGGACTTCATTATAGGTTCTGTATATATCTT

In556 NNNNNGACTACCGCATTTAAAAACTGAGTACTAACTCTCTGTTGGTGACAACTCTACACAGTGGACTTTATTGTAGGTTCCGTATATATCTT

Vi10-1 AATCAAATTATCGCACTCAAAAATCGAGTATTAGCTCTTCGTNNNNGGTAATATTACGTNNCAAACTTCATCATAAGATCTGTACCTATTTT

Vi10-2 GATCAAACTACCGCATTTAAAAACTGAGTACTAACCCTCTGCTGATGACAATTTTACACAGCGGACTTCATTATAGGTTCTGTATATACNNN

Vi10-3 GATCAAACTACTGCATTTAAAAACTGAGTACTAACTCTCTGTTGGTGACAATTTTACGTAGCGGACTTCATTATAGGTTCTGTATATATCTT

Vi10-5 GATCAAATTATCGCCCTTAGGAATTGAGTATTAGCTCCTCGTTGGTAATAATGTCACATATCAGACTTCATTATAGGTTTTGTACACACCTT

Vi12-1 GATCAAACTACCGCATTTAAAAACTGAGTACTAACTTTCTGTTGGTGATAACTTTACACAGCGGACTTCATTATAGGTTCTGTATATATCTT

Vi12-3 GATCAAACTACCGCATTTAAAAACTGAGTACTAACTCTCTGTTGGTGACAATTTTACATAGCGGACTTCATTATAGGTTCTGTATATATCTT

Vi12-5 GATCAAATTATCNNNCTTAGGAATTGAGTATTAGCTCCTCGTTGGTAATAATGTCACATNNCATATTTCATTATAGGTTTTGTACACACTTT

Vi23-1 GATCAAACTACCACATTTAAANNNNNNGTACTAACTTTCTGTTGGTGATAACTTTACACAGCGGACTTCATTATAGGTTCTGTATATATCTT

Vi23-2 GATCAAACTACCGCATTTAAAAATTGGGTACTAACTTTCTGTTGGTGATAACTTTACACAGCGGACTTCATTATAGGTTCTGTATATATCTT

Vi23-4 GATCAAATTATCGCACTTAGGAATTGAGTATTAGCTCCTCGTTGGTAATAATGTCACATAGCAGTCTTCATTATAGGTCTTGTACACACTTT

Vi23-5 NNNNNNNNNNTCGCACTTAGGAATTGAGTATTAGCTCCTCGTTGGTAATAATGTCACATAGCAGTCTTCATTATAGGTCTTGTACACACTTT
