# Supplementary material for: Contrasting genetic structure between mitochondrial and nuclear markers in the dengue fever mosquito from Rio de Janeiro: implications for vector control
Source: Evol Appl. 2015 Sep 7;8(9):901–15. doi: 10.1111/eva.12301 (PMC4610386; doi:10.1111/eva.12301)
Supplement: Supplementary file 4 [file eva0008-0901-sd4.docx]

80 5812

Br22-1 GTGATTTACTCGTYRYYNTANNGGCCGCCGGAGTCGTCGACCTAGNCGGGNCTAGGAGGTTTACCCACNGACYRTGATCTACTGGCCATNACGCCTCTTGTCTANGGAGYCCACTGCYCGGGAYCCATCTTGGGTNNGGGCYRTGYTGYGCYNACAATGGCCAGGCAGCRCNCAGRNGAGCCGAAAGTANNRRNAGGTTTGGACCTGGNYYNGGAACGGTAGAYYNYATAATACGGCAGTCTGTAAGTAGGTTGGGYACGNACAYCCTAGGCCTYRTRAGRAGGTTYTNRACRANYGYGGATCACYCRCAGGTCATTGACTATTYCCAGTNCCCGNGGATAGNCCGGTTGATGRYNTAGCTTGRCGARCRGTGGAGCCATTCTGGAGYTGCTANGGYGRRGCGGGCGYCGCAGGTTCGGAACCNNRGTRCRTTACAGTGCTGCCAGCTAGCCTTCAAGCRCYCTCYCTCTACTGCTGNRYGGNCTGCGTYTRGTTACCTTTGGCTGYCAAYGAGCCCTAGCGNANACAGYTAATYATCCTNGTCTARACTNGTNTNAGAGTCNRGARCCGACGCGTAYGRTTTCANCACCRACGGGGCRARGAAAGAACACCTCCGGCTACNAACRGTGGACRTTCCCCTNRAAACNARCGRYNGCTRAGGCTGAGCAGRCCNRTCTCYTCRATCYGNTCCYYTRTGYGCAAGCCCCYRCCGCCGCTTACRNCAGTANCTRGYGGACTGCGCCGTGCGGTGGGTACCAAGTGGCTCCTATTGTATGCGAATGGYNGGCACTACCAAGGACNGGCCGGCTGGTAGCNAGGAACTGATATTATGTGARTRCRNRCCCCGCTCGNAGTCCGGTNNCTGCTAGCCCTGTACACAACGGRAYYGTYCNNNGGCTTGCNAATACGGANAAGTTCGGAANAANRGGNNTYNYGGTYYRGYCNGCCACTNAAGTTGYRANTNGTNGYAACTAGCGGYANCCATGGCTCNTAAGTTCGGRNTANCCCNCGGTGTAAAGGCGRNCCGCRTCGGTAAGGACCGCTTTGCTGCRAGGGACAATYAGRCGNTTAGATTATGTYNGRAARANCGCACNCCTCTTACCAANGGYCACACNNNGTGGCTNCNCTTTYGGCGGCCGGGGGATTAGGCAYGNYCGGGGGCCGNTGANTACYNGGGNCTAGRAACAAGNATGCCCAGACGATGGGGCCAGCCACACCGRYAARTTCTCGCAGGGGGCRYCAGTTGGCTTGNTGCAGAGAATTTCTNTGGCCCGACGGTAACGCGNCRGNNYTTGAAAAGACTTANRCGTGAGNNCANACGCCNGAAATGTYRNTTAGGAATYNACTAGYACTGACGYRYANRYATCAGGCGCNTNGGTATCTCTCCGGGCGGCTRRCGYNNNTGTTGTGGGCTCGCCCCTGGACGAAGGAAGGGAAAAAYCYCRRGACYCTGAGGACTCNNCGACCCTANTGNACATAGAGCATCGNNYAGTTTAGCATATGGTCTCTGGRNGCRGCYRNCCTCTGACCTNNCCAGTGGCTTCCGCAGGAYNYTRYACAGCCAGAAAGCGRANNYCYCRCNCCTCCCNAGTGGTTTNNCANRTANCCTNCCYANRNCGCTCCGAYGGAATTGCTCTGCYNGTGCTGYTGTRANCCCTATATCTRTTAACCRGGTGGGCACTRNTYTCGGRNTYGTAGTRCTCGATNTAAGGTCGGRNNYCAYTATATACAGGTGGACYRTATTAGTCTGCTTYAYTGAGATGGCATRRTCTGTTTGGATNNCGTCNNNTTGGTTAACTGCTCGAAGCCCRTCCYCNCNNRTCCCGAAGAGTGACGGTGAGGCCGCGGATTCTCGCTGGACANTTTCNAAAGCGTCCAGAGRATACTTCGTTNTCTNTTCATTTACGCTACCCAATTTCGYNATRTNYCGGYRGCGTAGAGCTGGCRCCGCARTRRGTGTCTTGCAGAAYGGCTCAAGYRTCGGCGGCGCGTCACTATCRAGCCCYTGGCATGGCCGCGACAGCNNGCCGTCCCACYCRATGGANCGYGTYCCCACGAGGYANGCGGGATGAACACGGNRACGCTATGACGGGGTTTACACANGYTCCACACRGTTCAGTCGTTNYGNAAGGGCGGNNNGGGCCCCCANRCCGGTATGGGGCGTAATTGGGGTGYTCGCRAATACCTRNGGAATACYCRRYYGCTAATCGGCGCATCGTGGYNANGGCGCCTCGCTTCTRNAYTAANRTTGGCTACGTAGATGCATNYCGNAYNNTGCRGYGGGAAAAAGCAAATCCRTGGCAACGGGCNNYGCGACCACATTAGTTACTTAATGYNNNARAGAATGATTGTTCGTGTCCNYCCCCGCGNNGCGAAGAAAYTCAAARNNCCGCTRNNNRAAANCCTGTCAAGTAAGGCCCAATNYCCNGCNCNNGCAGCGNGGTCGCGNYAGCNGRTTGCRRGCNATTCGRGGAYTANNNCTACTNCTAGAAACGGCCGGTACAGGRARCCCGCTGGTANNYTACGACTTRATTAGCCCGTCCCGGAGCRATCGTCCTNGTCGTTAGNGNCTACTYYRGYGRCATACATCGCATYANACCYACGCANCAGCGGCAGCGTYTTRCAANACGCYRRACGCCTACANCRTCTCGGGCTAATACTTYTCCNNNCTCCTGATCACCGGTGAGCANTAGRCNNYNGNNCNTGGATACTCGAAAYGGGCYTYCNAAGCTCGCCRTCTTACNAGACNCCTGACAGAATGYGGCGTTTTATCCATGTNACTNCTGAGCGTNGGTGACNANNCRCGACCGCCGGTTTATGAACCGCACTACCGGRCTTNCAGGAGAAGAGACGCGGYATCNTAGCGCAGGTGTCRACCTGYANNAGCCGAGGAAATGGRACCGCGATCACGTNAYYCCTTACTCCGCATGGACGCTRGNCYYNAGCGRGGGTGNCCANRYCRATCCCGGGCCTGAAAGTCGRRCATCTAGTTCNAGCYGRRNTACCCCCCGATCCGTGCATAAAGTTANCACGCTNNCCGNACTGGGACYYACCGNNATTRCGARGATTANNGCAACTCNRNTGNTCCGTCACAGCGGTATTAANNYCTCNCGTCCTTTATGGGAGACGGRGTGAAACATTARRGYRCCGNNGGACCATCCGGCCCGCRTCACGTRTGTAANAGTCNYTAGGTATGTGAGAGGAGTTCGRGCACCAGYGTYTTNACCTCGAGTTTTCRRRYYRNCCAYCCRNCNCCRYAGACRNNGGNAGCTCNCAANCGATRGCYYATCCCACTAGNNGYNACTCCAGRNGACGCGAAAGCCTCAARGTNCAATCATTNTNGTGTTGGTNGGNNGAGAAGRGTAGNACRAYYCNNANNTCNCAGGAATGGTCCRTGCAGAAGGAGACATCACCANGGNNAGTNNNCGCGAGANNCGCTACTTGCCCNNTGTNAGTYTRCATTNCATAATANAATCRNCGCTGCCTCGTCNYGACCGTACGYANAAGNYYYYAGAACCYAAATCGCCGGCTACCCCGACGCCAGCCTCGCTCAGCCTGAGCTACTGTATCTAAATNGACTGTCCTCNTTGTAGTTGNCNGGCCCCTYTYANTNNYGTNGCNANRACNCACAGGCANTTTGAGAAANNATACAYGCGGYAATYYGTARGGCNAGGYTACACAGCCTGAGRGGCCAGCCTACTGCAGATRRRTYNNNCARTYTCGGCCYRGATCGGGTCCAYNGTGRGCGGANCGGTCCGCAGGCGGCCTGYTTGANCGGACTCGCGACGGGCCCCTGCGATCTANNCGTCTGAACCCGGGCTYYGTGGYCGTTCCGTRTNACTGATCYNRGNACTTRGATGCCCTTGTAAGCACNATGGAATAGGTTGTACCACGCCACACGGCGGCATAGGACGCCAGCGGYNYCTTRYYTNAGAGGTCAAGTTTCNNCGYRTTGTTCCCAAGCAATCATGCGTAANRGGATGGGRTCATGGYAGYGCGAATYAGGCGCRRTCTCTRGTCCCRAARAYTGCANTCATNNATGCGCACGTCGAATGCACCRYGGCRTTGCTNGCGGRTACCGGCGRCYRTCATNGYGTTCATGGTGTTNTGAGCTCTYCTTAGGCGCGGACCGAAANCGCCNCGATCNTGCGTARNARGCNNRAATRTGNNCANNGCGTCAGCACGGANCNTGAAAGCGCNGNNATTACGGCYCAYTCCNATAYAGGNCTAGGTACGNCYYCACNCTGGCTGTACCCYYGGGGGACNNNCGTYGCGCANNTTCACNGRRGGGGAGYCAACACTYTGTTRNNGYGGGCYCTCGNGGTNATTAAATNGTGNCNTCTCCYYAAYCRNARGAGAATTCTGCTRYCNCGGAGGNCGCCGGCATGRTGTCGCTTCACTCCCGATAYGTNTCGCANYRYAACGAAGCCAGRCGTYGTATAACAGAANNATNACAAANNAAGATACGCAGTNYGGGACGACGTNGCYAACNCGGANCCTGATCGTGTNYYGNANCRACACAACATCGGTCTCTGRCTTTANCCGGGTGCAGACNYCTYAGTCAGCTGGGATYACYGGNCGNRTARGNCGAGAGCCGRRNNAACCYGAGTCTAGCGRNTCGYGNACNNCCCGAARRGCRCCNCACATNTRYAYTTNNYTCAGGGCGGCAGCGCCNAAAYANNCAGTTCCCACCTGAGGTCAGCCCTRARATCTAAGGTAAGCCCCATNNNCGATTGGGAGGCYGRATCCGGAYGTYNGGTCNTATTCCTCGAACCGACGGTAAAGTACRTGRRGAATTTGTTARCGTGACCYGCTAGRCCNCRTGTYGGYNCACAGCTGCCTCAGTNNAATGGCRYTTCYAAGGTTTGYCTCYNAGCGGCAGGRRTTAGTTNGCNCACCCTCAATATGAYAYAATCTNYAGYAAGCCGNYAAARGGCACTCGCCAGCATTTGCATCCACATGCGTCNCTGAYTTTTCCATGNCTGGCTGRCGGAACGGARGTNYTNRRTCYGTGANGNNTAGCCGTCGCRGANGTNRCYGGCTGTNNTRTGCCACGGRYTGTGCCCAGATTGTNYNNNAAGTGNYAGATNGRYCYTNRCYTRGGGCGTYGRGACATAATCGCAANCGNCTCARGTCYTARGAACGTNNGATCCTGATCGCNNGACCYCAYCTGTCRNAGCTACCGCTACGTCTTCTCAGCGACTGGCTAGCTYGAACCNNNGAYAACGRGGGTGTTAAGGGACTCTAGAAGGNCAACCTTRNRNTGYNCGYANGATCCTTARACCGNYTYGYGRCRTGNNGGCAGAGCCCCTAGGCTGAGNCCTAGGCCGTCCGCAAACNGGCCRGTTNGRCCRNAGGCCGAAAGGGGCGCAACGTAACATRRCTYTACTAAGTNCRCCGAGCNNNYCTAGCAGNAGCCGGTGTGYATRCCCNTACCATTCGCYTGAGGCTTATCTGTCRGTACAACCATGGCGCCAACTGRNGAGTGCNTGGTACTTTGTATAAATGCCNTTNYCGGRAGCATYNGATCGYNCGGTCCCGAGATCAGCCATTRGTYAGGCATTGCCTTTGCACGGCNACGGCCCGRTGTCRCGACCNCGGGTTAGTATGTRGTGGATTTTTTRRNTYCGACTCGATTACCCAAGATGGAGNCNCCAGCATCCTTNCNCTGGRRTTTAYCGTNAAGACGATCGGGCANAGCRCTACCTGCTGGCGGAGTCYGTTRRTNNNNNAANCNAGTGAYCAGATTTNGNACCCGNNNNYYTACAGTGGCGAGTTATCGCNTTCAGTCCGACNCGGYCTCGCAGTRCRCANN

Br22-2 NCGATTTACTCATCNNNNNAATNNNCGCCGGAGTCGTCGACCTAGACGGGTCTAGGAGGNNTACCCACNGATCGCGATCTACTGGCCATNANNNNNCTTGTCTAAGGGNNNCACYGCCCNNGRTCNNNCTTGGGTATRGGCCNTNCTGCGACTCCAATGGCYNGGCAGCGYRCAGGAGAGCCGAAAGTANNRACAGGTTTGGAYYNGGCTTTGGRNCNGYRRAYYTYANNATACGGCAGTCTCCAAGTACGNTTGGNACGAANANNCTAGGCCTCANNNNGAAATTNTNRACNNNNNNNNATCACNCNCAGGTCATYRNCNNYNYCCANNNYCCGNGGATAGCCCGGTTGATRRCTTAGNTTGRNGAACGGTGGAGCCATTCTNGAGNTGCTANGGYGRNNNNGGCGCCGCAGGTTCGGAAYCTAAGTACATTACATTGCCGCCAGCTAGCCTYNAANCRCNNNCTCTYTACTGCTGNRYGGNCTGCATTTGGTTACCTTTGGCTNNCAATGAANCCTANCRGACACAGCTANTTATCCTAGNNNNNACTNRNGTRAGAGTCNRNNNNCGACGCGTATGGTTTCAACACNRACGGGGCRAGGAAAGAACACCTCCGGCTACCAACGGTGGNCATTCCCCTNRAAACAARYGNNCGCNNNNNNTGAGCARGCCNRTCTCYTCRATCYGNTCCYYTRTNYGCANNCCCCYRCCNCCGCTTAYGNNARNACCNAGCGGANTGCNNNNNNCGGTRRGTACCAGGTGGCTCCTATYNCATGNGAATRGNNGGCACTACCAAGGACTAGCCGGCTGGTAGCTANNAACTGATNNNNNGTGAGTACATGCCCCGTNNGCACTACAGATACAGCTAGCTCTGTACACAACGGNNTTGTNNNNNNGCTTGCGAATACGGATAAGTTCGGAAAAACRGGCNNCNTGNNNYGGTCANNCACNAANGTTGYRANTNGNAGTAACTCGNGGCAGCCNNGNCTCNTAANTTCGGRNTARCCCRCGGTGTANNNGCGGGCCGCRNCGRCGRRRGYCGCTTTGCTRCGAGGGNNNATTANRCGNTTARAYNNNNNCAGRARRAANGCACTCYTCTTAACRNAGGYNAYACGCTGTGGCTCCAYTTTYNRNGGCNNGGGNNTNNNGCANNNCCGGGGGNNGNNRANTACTNNNNCCTAGGAANANGAATGNNNNNNNGATGGGGCCANNNACACCGACAARTTCTCGCAGAGGGCNNNAGTTNGNTTGCTGCAGRRNATNNCNNNNGCCCGACGGTAACGCGNYRGNNYYYGAAAAGACATANNNGTGNNATCRGNNNNNAGAAATGTCGNNTAGGNNTYNACTAGNNNNGNNNTAYACGTRNCNNGCGCCTNNNTATCTCTCCGGGCNRCCRRCGCNNNTGNNRTNNNNNCGCCCNNGRNNNNNGGANNGGAAAANCCTCGGGANNCTGANGACTYNNNGACCCTARTGNNCATAGAGNRTNGCNYAGTTTGACATANNNNNNCTGGGAGCAACCGTNNNNNGACTTACCCAGTGGCTTCCGCAGGAYTTTGTACAGCCAGAAAGYGAACCYCCNGCNNCNCNCAAGYGGTTTTTCACATACNCTACCYAGGTCNNTCCGAYGGAATTGCTCTATTNGTGCTNNNNTGAGCCCTATATCTATTAACCGGGTGGNNNNNNNNCTCGGRNTNNTAGTNNTCGATANNNNNNNGGNNANNACTATGTGCAGGTNNNNNNTANTAGNCNGCTTCNNNNAGATGGCAYGGTNTGTTTGGATATCGTCCGCTTGGTTANNNGCNCGAANCCCNNNNNCACGNGTCCCGAAGAGTGNNRGTGAGGCCGCGGATNCTNGCTGGACACTTTCYANAGCGNNCAGAGGATACTTCGTTYTCTTTTCATTTACGCTACNNAATTTCGCAATGTGCCGGYRNCGYAGAGNNNGCRCCGCAATGGGTGTCTTGCAGAATGGCTCAAGCRGCAGCGGCGCNNCANNATCNNGCCCCTGGCAYNNNNGCGRCAGCNRGCCGACCCACNCNATGGNTCGYGTNCCCACGAGGNNGGCGGGATGAATACGGAGACGCTATGACGGNNNTTNNNCAAGTYYCANGNGGTTCNGCCGTNNCGCANGGACGGTTCGGGCCCCCAGANNNNTNNGGGNNGTAATTGGRGTNNTCGCNANNACCTRNGRAATNNYCRRYYGCNNNNCGGNNNNNCGTGGYNANGGCNNCTCGCTTCTANNNNAACATNGGCAACGTAGATGCATGYGGAACTTTGCAGTGGGAARRAGCRARNTCGCGGCAACGGGCTCNGCGACNACATTAGTNACTTNNTGNGTCAAAGAATGATYGTTCGTGTCCGCCYNNNCGNCRNNAAGNRACYCAANRNNCCGNNNNNNRAAANCCTGTCAAGTNNNNNNCAAYNTCCNGCGCACNNNNNRNGGTCGCGNYAGNNNGTNGCGAGYRNTYCNAGGNCTAGCACTACNNCTAGAAACGRYCNRNACANGAARCYYGCTGGTNNNTTACGACTTRATTAGCTCGNNCCGGNGCAATCNNNNNCGTCGTNAGNGNCTANNYCGGTGACATNCATCGCATTANACCCACGCANCAGCGGCAGCGTCYNACAANACNCTANACGCCCACAGCNTCTNNNNCTAANACTTCTCCNCANNYNYGATCACCGNNGGCCACTAGNCGCNNNNNNNTGGATAACCGAAAYRGGCYTYCNGAGCTCGCNNNNNNACAAGACNNNNGATANTGTGYGGCGYNTTNYCCRYGTCAACTYTGAGCGTCGGTGACGATACACGACCGNNGGTTNATGNACCGCNNNACCGGGNNTNCAGGRGARGAGACGNGNTATCNNNACGCAGGNGTCGACCTGTTNNARCCGANNNNNTGGRACTACAGACACGYGACCCCNTACYNCGCAYGGACGCYNGNNNNNAGNNRGGNNNTCTANNNCNATCCCNGRTCYNAAAGTCGAACNNYTNNNTCNAGCYGAAATACCCCCCGATCCGTGYNTAANNNTACCACGCTNNCAGTANTGGGACYYACCGNGTTTGCGARGATTANAGCAACNNNRTTNNTCCGNNNCAGAGGTATTNAANYCTCNCGTCCTCTATAACAGNCGGGGTGRAACACTCAGNCANCGYYGGACCNNCCGGCNCGCATCACGTGTGCAANAGTCNNTRGGTNTGTGAGAGGAGTTCGRGNNCNNNTGTNTTNACCTCNNNTTTTCNNNNYRNCCACCCAGCTCNRCAGACRNNGGNNNCTNTCAAGNNNTNGCYTNTNCCNCTAAAGGYNAYTCCAGRNGACGCNNNAGCATCAARGNRTAATCANNNTNGTNTTGGTCGGACGANNAGAGTARNAYRATCCCTATTTNNCAGGNNTGGTCCNTGCNGNAGGAGNYATCACCANNGAAAGTAAYCGCGAGNTGCGCTACATGTNCTNYGNNNNTYTRCAYTGCATAATAAACTCGTCGCTGCCTNNNNNNGACCGTACGCANNAGNYYYYAGAACCYAANTCGCCGGCTACCCCGACGCCNGCCTCGYNCRGCCTGAGCTACTGTATCTAAATNNNCTNTNYYCNTNGTAGTTGGCGGATCCCTTTTAAYCTCGTCGCNANRACNCACAGGCARNNNGAGAAATCATACACGCGGTAAACCGTANNNCTAGGCTANACAGCCTGAGRGGCCANCCTACNNNNNNTRRRTCGCNCANNNNNRGCYTRRATCGGGTTCATCGTGRRNGGANCGNTCCGCAGGCGGCCYRNNNGATCGNNNYYRCNNNNNNNNCCTGCNATCTACCNNTCCGCGCCCNGGCGCCGTGGYCRTTCCGYATTACTGATCCNGGGANNTAGATGCCCTNNNNNNNNCCNYNGNNNAGATTGTACCANNCCACACGRCGGCAGAGGACGCYAGCNNNNCCTTACYTYAGNNGTCAAGTTTNCTNNTGTTGCCCCCAAGCAATCATNCNNNNAAGGATGNGGTCNNGGCNNCGCGANTNAGGYGCGGTCTCTNNYCCCNAANAYNGCTGTCATNNATGCGCRCGTCGANTGCACCGNNNCGTTGCTTNCGGRTNCNGGCGGCNNTCATAGCGTNCATGGTRTTATGANCTCTNCTTAGGCGCGGACCGAAANCRCCACGNNCATGNNNARNARGCCTRAATNTGTCCRAAGCGTYAGCNNGGANNTTGAANGCGYGAATATTACGGCTCACTCCTATACAGGTCTAGGTACGRCNTCACANTGGCNNNNYNYYYGGGNGNNNNNCNTYGCGCAAANNNNNNNNNNGGGAGCCAACACTCTGTNRNNGYNNGCTCNNNNGGTTACNRNAYTGTGGCGTCNCCYYAATCNAARNAGNNTTCTGNNACCNCRGNRGNCGCCGNCNTGGTGTCGCTNCNNTCTTNATATGTATYGCAATNNANNNAAGCCAGGCGYYGYANNNNNNANANAYGACRANACAARRYRCGCAGTNYGGGACGACGTNGCNANCNCRGNCCCTGATCGTGTNNYGNANCRNCACRNCNTCAGTCTCTGACTTTAACCRNNNGCAGACGTCTCAGTCAGCAGGGATTTCTAGCCGNRTARGNCRTGTGNCGGGCCAACCCGAGTCTAGCGRGTCGTGAACNNNNCGNAGGGCACCACACANCTATATNTCACTCAGGGCGGCAGCGCCNNNACACTCAGTNCCCACCTNNGGTTAGCCCNRNNANCTAAGGTAAGCCCYATGAGCGATTGGGAGGCNGRATNNGGNCATCCARTCCYATTCNNCGANNCGACCATAAAGTACGTGAAGAATTTGTTANCTTRACCYGCTAGRCCRCRTGTNGGNACACAGCTGCCTCAGTNNARTGGNACTNNNNAGRTTTACCACCAAGCGRCAGGAGTTAGTTGGNCCRYCCTCAATNTGAYAYAANNTACAGCAAGCCNNTRRAAGGNNCTYGCCAGCATTTGCATCNNCATGCGTCNCNRTYNNTYCYRTGNCTGGNCGRCGGAACGNNGGTTCTCGATCTGTRNNGRNTAGCNGTCRCAGAGGTTACYNGCTGTNNTRTGNCACGGRYTGTGCNNNNNNNNNNCNNNAANNNNNNNATAGACYCTNACCTAGGGCGTYGAGAYATANNCGNANTCGNNNCAGGTCCTAGGAACGTRRNNNCCTNNTCGNYNGACCCCNCCTGTCANNNTTNCGGATNCGNCTTCNCNRCGACTGGCTAGCTTGAACCCTAGATAACGAGGNTGTTAAGNNNCTCTAGAAGGGCATCCTTGGRNTGCCCGTRTGATCCNNARACCGNNNTGTGGCATGGNGGCAGAGCCCCTNGGCAGGGNCCTNGNCCGTCCGCNNNNNNGYCAGTTTGGCNRNAGGNCGACNGGGGCGCAACNNNNNATRRCNYYACNRAGAACANTNNGCTATTTTAGCAGNAGCCGGTGNNCATACCNNNNNNNYNCGCNTGAGGNTTRTCTGTCRGTANANNCATGGCGCCAACTNNNGAGTGCNCGGTACTNTNTATAAATGCCCTYNYCGRGAGCATNNRNTCNNNCGGNCCCGAGNYANRYCATTARYYAGRCATTGCCTTTGCACGGCNACGGNCCGRTGTCGCGACNNCGNNNNNNNATGTGGYRGAYTTTTTAGATCCGACTCGATTACCCAAGATGGAGNNNCCAGCATCCTTACGCTGNNATTTATTGTGAAAGNGATNGGGCNAAGCACTANCNNNNNGCGRNGTCCGTTGRNGACCGNANCRANNGATCNNATTTTGTACCTAGCATCCTACANTGGAGGGNNNTYRCATTCAGTCCGACGCNNNCTCGCAGTGCRCNNN

Br26-1 GCGATTTACTCGTYRYCNTAATGGCNGCCRGRGTCGTCGACCTAGACGGGTCTAGGRGGTTTACCCACNNAYYGYGATCTACTGGCYATCNCGCCTTTTGTCTRGGGAGCCCACTNCTCGGGATCCRTCTTGGGTATAGGTTATGCTCCGCTTAGAATGGCCTGGCAGCGYRCAGNNGAGCCGAAAGTAACARGAGGTTTGGACCTGGCTTAGGGACAGCGAACTTNACAATACGGCAGTCTNYAAGTACGTTTGGTAYGNANAYCCTAGGYCTYRTNAGRAARYTTTTGACGATCGTGGATCACTCGTTGGTCATCACCYATNNCCAATGCCCGTGGATAGTTTGGTTRATGGCTTAGAYTNACNAACGRTGGAGCCNNTCTGNAGYTGCTANRNTGRRGCGGGCGNCGYNGGYYNGGAACCTNRGTNCATTACATTGCCGCCAGCTAGCCTYNAAACGCTCTCYCTCTACTGYTGARTGGGCTGCATTTGGTTACCTTTGGCTGYNAATGAATCCTANCRGANACAGCTAATCATCCTAGTCTAGACTCGTTTAAGAGTCTAGAGCCGACGCGTAYGRTNTYAACACCNACGGGGCGARGAAAGAACRYCTCCGGCTACNANCRGTGGRCATTCCCCTTAAAACRAGNGACNGCTGAGNNTGAGCANRCCAATCTCCTCRATCTGCTCCYYTRTNYGCAAGCCCCTGYCGCCNCTTACGCCAGTACCTGGTRNACTNCGCCGTGCGGTRRGTACCARGTGGCTCCTATYGYRTGCGAATNNTNNRCACTACCGTAATCTAGCCGGCTGGTAGCNAGGAACTGATATTATGYGAGTACATGCCCCGCTCGNAGTNCRGNTACAGCTAGCYCTGTACACAACGGAATTGTTCTNAGGCTTGCGAATNNGNNTNAGNTCGGNAAAACGGCCGCCNTGRNCCGGTCAGTCACTRAAGTTGYRANTNGNNGYAACTNGCGGTAGCCRYGRCTCATAAATTAAGAATAGCCCGCGGTGTARRGNCGANCCGCRTCGGCGAGRGCCGCYYTGYCGCRAGGGNNNATYANACGATTARAYTATGTYARRARRAACGYACTCCTCTTGACGCAGGTAACACGCTGTGGCTCCAYTTTNNRCGGCCGGGGNNTTNNNNACGTCCGGGGGNCGCTNANTACNAGGGCCTAGGAAYANGAATGCCCAAACGATNGGGCCAGCCACACCGACAARTTCTCGCAGAGGGCGCCAGTTGGCTTGCTGYAGGRTAYYTCTNTGGCCCGANRGTAACGCGACGGTATYYGAAAAGACATAGRYRNGNGATCAGACNCCAGAAATGTYRNTNAGGAATTGACTAGYACTGACGTANACGTATCAGGCGCNTNGGTANCTCTCCGGGCGGCCRRCGYNNNTGTTNTNGGCTCGCCCCTNGACRANGGAANGGAAAAGCCTCRRGACCCTGAGGACTCNNCGACCCTARTGAACATAGAGCRTCGCCCAGTTTRNCATATGGTCTCTGGGANCAACCGTNNNNNNNNCCACCCAGTGGCTTCCGCAGGACTTTGTACAGTCACTAAGNGAACCTCCTGCACCTCCCNAGTGGTTTNNCAAGTANCCYNCCYACRNCGCTCCGANGGAATTGCTCTNNNNGTGCNATCGTGAGCNCTATATCTGTTAACNRGGTGGGCACTRNTYTCGGRNTCGTAGTACTCGATNTAAGGTCGGGGATCAYTATGTGCAGGTGGACYRTATTAGCCTACTTTATNGAGATGGCATGGACTGTTTGGATNNCGTCCGCTTGGTTANYTGCTCGAAGCCCGTCCNCACGCGTCCCGATGAGTGACGGTGAGGCCGNGGNTTCNCGCTGGACACYTTCNAAAGCGTCCAGAGGATACTTCGTTCTCTTTYCATTTACGCNANCCAATTTCGTAATATGCCNNCACCGYARAGCTNGCRCCGCARTGGGTGTCTTGCAGAAYGGYNCAAGCRTCGGCGGCGCGTCACNATCRAGCCCYTGGCACGGCGGCGACAGCTAGCCGTCCCACYCRATGGAGCGCGTYCCCACGAGGYANGCGGGATGAATACGGAGACGCTATGACGGGGTTTATCCANRTNTCAYGYGGTTCNGYNGTTRCGCAAGGRCGGTTCGGGCCCCCNRACCGNTATGGGGYGTAATTGGRGTGYTCGCNAATACCTRNGGAATACYCRRYYGCTGATCGGNGCANCGTGGTNAGGGCGCCTCGCTTCTRNATTAACATYGGCAACGTAGATGCRTGTGGAACTNYGYAGYGGGAAAGAGCGAATYCRYGGYRRCGRGCYCCGCGACNACATTAGTTACYTAATGCNNNAGAGANTGANCGTTCGTGTCCNYCYCCGCGGNGCGAAGNRNCTNAAAGGCCCACTACGCAAAAGCCTGTCAAGTNRNGNNCAAYNTCCGGCGCACGCGGAGAGGTCGYGCCAGTCRGTTGCRRGCNATTCGRGGAYTAGCACTACTNCTAGARACNGCCGGTACAGGRARCCCGCTGNTAGGCTNCGRCTTRATTAGCTCGYYCCGGAGCAATCGTCCTCGTTGTCAGGGGCTACTTCGGTGRCRNACANCGCATTAGACGCACGCANCAGCGGCAGCGTYTTRCAANNNTCYRAACGCCYACATCGTCTCGGGCTAATACTTYTCCNNACTCCTGATCACCGGTGANCACTAGGYGYCTGTTCCTGGATANYCGAAATGGGCYTYCNAAGCTCGCCATCTTACNNNACTCCYGANAGAATGCGGCGTNTTAYNCRYGTNANYNNTGAGCGTCGGTGRCGATGCACGANCGCCGGTTTATGAACCGCACCACCGGRCYTGCAGGRGARGAGACGYGNYATCTTAACGCAGGTGTCRACCTGTTTNAGCCGAGRRAATRRGRCYRCRRNCACGYGACCCCTTACTNCGCATGGACRCYAGCYTCAAGCGGGGGTGNCTACAYCRATCCCRGRYCTGAAAGTCGAACATYTCCTTCTAGCTGRRNTACCCCCCGAYCCGTGCATAAAGTTACCACGCTTACNRTNCTGGGACYYACCGGGNTTGCGAGNATTANAGCAACCCCGTTGCYCNNTCACAGCNGTNNTAAAGCCTCACGTCCTTTATGGGAGACGGGGTGAAACATNAAGGYRCCGTCGGACCGTCCTGCCCGCGTCACGTRTGYAATAGTCNYTNGRTATGTGAGNGGAGTTCGGGCACCACTGTCAGGACCTCGAGTTTTCGGACTAGCCATACAGCTYNRCAGACATTGGCRRCTCACNAGCGGTAGCCCRTNCCNCTAAAGGNNACTCCAGGGGACGCGGAAGCATCAAAGTGYAATCATTNNCGTATTGGTCGGACGARAAGAGTAATATAACTCCTATGTCGCAGGATTAGTTCGTGCNGRAGGAGACATCNCNANGGANAGTRNCCGCGAGNTGCGCTACNTGYCCTTTGTCAGTYTRCAYTRCATAANNNRANCRNCGCTGCCTCGTCNYGACCGTACGCANNANNCYCCAGAACYTAAATYGCCGGCTACCCCGACGCCAGCCTCGCNCAGCCNGAGCTACTGTATCTAAATNGRCTRTYYYCNTTGTAGTTGGCGGATCCCTTTTANTNNYGTCNNNNNNACNCACAGGCAGTTTGAGAAATCATACACGCGGTAAACCGTAGGGCNAGGCTANACAGCCTNAGNRGCCANCCTACTGCAGATGAGTYGCNCAGNCTCGNYYTAAATCGGGTTCATCGTGRRCGGACNGNTCCGCAGGCGGCCTGYCTGAYCGNNNYCGCRNYGGGCCCCTGCGAYYNACNCGYCYGCGCCCCGGCNCCGTGGCCGTTCCGNRTTNCTGNTCCNGGGACTTRGATGCCCTTGTAAGCACCNTGGAATAGATTGTAYCACGCCACACGNCGGYNTAGGACGCYAGCGGNNYCTTGTCTTAGAGGTCAAGTTTCCTCGTGTTGCCCCCGGGYAATCATGCNNNNNNNGATGNGRTCATGGCARYGCGAATCAGRTGCGGTCTCTRNTCCCNAARAYTGCANTNATNNATGCGCACGTCGAATGCNCCRCGGCATTGCTTACGGRTRCTNGCGRCCRTCATNGYGTTYATGRTGTTATGAGCTCTCCTTARGCGCGGACYGAAANCGCCACGATCNTGCGTAGAARGCNYGAATRTGAGCAAARCGTTAGCANGGACYTTGAANGCGYGAATATTACGGCTCACTCCTATACAGGNTTAGGTACGRCYYCACNCTGGCTGTACCCYYGGGGGNCYGCCGYYGCGCARNTTCANGGGGGGGGAGCTGACACCTTGTTGNNGCGGGYTCNCGAGGTTACTAAATNGTGRCRTYGCYYYAAYCRAARGAGARTTNTGYTACCACGGAGGACGCCGNCNTGGTGTCGCTTCANTCYYNATAYGTATYGCATTGCAACGAAGCYAGGCGYCRTATRATANAAAGATGACAAANNAAGATACGCAGTNYGGGNCRRCGTAGCYANCACGNRCCCTGATCGTGTACCGAACCGACACRACNTCRGTYTCTGGCTTTACCCGGGTGCAGACGTCTCAGTCAGCTGGGATNACYRGCCGNRTAAGCCGAGNGYCGAGCCAACCCGAACCTAGCGGGTCGTGAACAACCCGGAGGGCACCACACATCTATATTTCACTCAGGGCGGNAGCGCCNAAAYANTYNGTTCCCACCTNNGGTYAGCCCTRAAATCTAAGGTAAGCCCYANNNGNNNNNGGGAGGCCGAATYYNNATGTCCGRTCCTATTCCTCGAACCGACCRTAAAGTACGTGAAGAATTTGTTAACTTGACNCGCTRGACCRCGTGTTGGNNCACAGCTGCCNCRGTNNAATGGNRYTTCCAAGATTTACNNNNAAGCGRCAGGAGTTAGTTGGNCCRCCCCCAATNTGAYAYAATCTACAGCAARCCGATRRAAGGCACTNNCCAGCATTTGCATCCACATRCGTCNCYRTCTTTTYCATGNCTGGYTGGCGGAAYRGARGNTCTCGATCTGTRANGAATAGCCGTCRCAGAGGTTACYGGCTATTGTGTAGCGCGGRYTGTRCCCAGATTACTCTCTAAGTTGCAAGTAGACCCTGGCTTAGGGCGTCGAGAYATAANCGYAATCGNCTCAGGTYCTAGGAACGTGGGATCCTGATCGCTCRATCCCACCTNTCRNNNCTACCGCNRCGTCTTCGCTACGACTGGCTAGCTNGAACCCTAGATAACGRGGRTGTTAAGGGACTCTGTCAGGGCATYCTTGGRNTGYNCGTATGATCCTTAGACCGNYTTGTGGCATGGAGGCAGAGCCCNTAGGCNGRGNCCTAGGCCGTTNNNAAACNGGCNARYTGGRCCAGAGGCCGANAGGGGCGCNACGTAACATAGCTCCACGGAGAACACYGAGCTATTTTAGCAGGAGCCGGNGTGYNNGCCCGTACCATTCGCCTGRRGCTTATCTGTCRGTANRRCCATGGCGCCAACTRGNGAGTGCNCGGTNNNYTNTATAAATGCCCTTTCCGGNNNCATYNGANYGYNCGGNCCCGAGATCAGCCATTRRYYAGGYATTGCCYNYGYACGGCAACGGCNNGRTGTCRCGACCTCGGGNCGGTATGTGGYRGAYTTTNTRRRTCCGACTCGATTACCCAAGATGGAGNCNCCAGCATCCTTACRCTGGGATTTATCGTRAARRCGATNGGGCRNAGCACTACCTGCTGGCGGAGTCCGTTGATGACCGNATCAAGNGATCAGATTTTGTACCYRGYATCYYANANTGGAGGGTYNTYRCATTCAGTCCGACNCGRCCTCGCAGTGCGCRNN

Br26-2 GCGATTTACTNGYCRTCTTAATGGCCGCCGGAGTCGTCGACCTAGACGGGTCTAGGNGGTTTACCCACTGACTGTGATCTACTGNNNNTCNCGCCTTTTGTCTAGGGAGCCCACTGCTCGGGAYCCRTCTTGGGTATGGGNNATGCTNCGCTTANAATGGCCTGGCAGCGCGCAGNNGAGCCGAAAGTAACAGGAGGTTTGGACCTGGCNNTGGGACRRNNRAYYTTRCAATACGGCAGTCTCCAAGTACGTTTGGTANGNACRTCNTAGGTCTTGTAAGAAARYTTTNRACNANNGNGGATCACTCGTTGGTCATCACCYATNYCCAATGYCCGTGGATAGNNYGGTTRATGRYNTAGACTTANGAACGRTGGAGCCATTCTGNAGNTGCTACGGTGAAGCGGGCGCCGTTGGYYNGGAACCTNNGTRCATTACATTGCCGCCAGCTAGCCTTCAARCRCNCTCTCTNNNCTGCTGAAYGGNCTGCATTTGGTTRCCTTYNGCTRNNAAYGAAYCCTANCRGANACAGCTAATYATCCTNGTCTAGACTCGTTTAAGAGTCTAGAGCCGACGCGTACGRTNNYAACACCNACGGGGCGAAGAAAGAACACCTCCGGCTACNANCGGTGGRCRTTCCCCTNAAAACRAGTNNNNGYTGAGGCNNNNCAGNCCAATCTCCTCRATCTGCTCCNNTRTNYGCAAGCCCCTGYCGNNNCTTACGCCAGTACCTGGTNNACTGCNNNNTGCGNNGGGTACCAAGTGGCTCCTATYNYATGCGAATRGTNNRCACTACCAAGGACTAGCCGGCTGGTAGCTAGGAACTGRTNTTATGNGAGTACATGCCCCGNYCGCANTNCNGNTNCNGCTANCCCTGTACACAACGGAATTGTTCTTAGGCTTGCGAATNYGRNTRAGNTCGGAAAAACGGNCGCCNTGRNCCGGTCAGTCACTRAAGTTGYRANTNGNNGTAACTNGCGNNAGCCRYGRCTCATAAATTAAGAATAGCCCGCGGTGTARRGNCGRNCCGCRTCRGCGARNGCCGCYYTGNYGCRAGGNAYAATCRNACGATTARAYTATGTNAGRARRAACGCACTCYTCTTRANNNNNNTAACACGCTGTGGCTCCATTTTYNGCGRCCGRGGNNTTAGGCNCGTCCGGGGGYCGCTRANTACYAGGGCCTAGGAANANGAATGCCCANACNNNNGGGCCAGCCACACCGACAARTTCTCGCAGRGGGCRYCAGNNGGCTTGCTGCRRRRNAYYTCTNTGGCCCGACGGTAACGCGNCRGYNYYYGAAAAGACATAGACGTGAGATCAGRCTCCAGAAATGTCRNTNAGGAATYNACTAGNACTGACGTAYANGTATCAGGCGCCTNRRTANCTCTCCGGGCNRCCRRCGCNNNTGNNRTGGGCTYGCCCCTNGNCRANGGARGGGAAAARCCTCRRGACCCTGAGNACTCNNCGACCCTARTGAACATAGAGCRTCGCCCAGTNTRGCATATGGTYNCTGGATGCGGCTATNNNNNGACNNACCCAGTGGCTTCCGCAGGACTTTGTACAGNNNNNRAGCGAACCTCCYGCNCCTCCCNAGTGGTTTNNCAAGTAGCNTTCCYANRNCNCTCCGACGGAANTGCNCTGCYNGTGCNATCGTGAGCYCTATATCTGTTAACNNGGTGGGCACTRNTTTCGGACTCGTAGTACTCGATNTAAGGTCGGGGNTCANTATGTGCAGGTGGACYRNATTAGYCTRCTTYATNGAGATGGCAYGGNCTGTTTGGATATCGTCCGCTTGGTTATTNGCYCGAAGCCCGTCCYCNCNCRTCCCGANGAGTNNCNNNGAGGCCGNGGATYCTCGCTGGACANYTTCCAAAGCGTCCAGAGGATACTTCGTTNTCTTTTCATTTACGCYACCCAATTTCGTAATATGCCGGTGGCGTARAGCTNGYRNCGCARTGGGTGTCTTGCAGAAYGGCTCAAGYGNCRNCGGCGCGTNNCTATCGAGCCCYTGGCACGGCGGCGACNNNNNNNNGTCCCACTCGATGGANCGYGTNCCCACGAGGNANGCGGGATGAATACGGAGACGCTATGACGGGGTTTNTNCANRTYTCAYGYGGTTCAGTNGTTRYGNAAGGGCGGTTCGGGCCCCCANRCCGNTATGGGGYGTAATTGGNGTGYTCGCRAATACCTRNGGAATACCCAGTTGCTGATNNNTGCAGCGTGGYTAGGGCGCCTCGCTTCTAGATTAACATTGGCAACGTAGATGCATGTGGAACTNYGYAGYGGGAAAGRGCRAATYCRYGGYRRCGRGCTCCGCGACNACATTAGTTACYTAATGTNNNARAGAATGAYCGTTCGTGTCCGCCNCCGCGGNGCGAAGCGACTNAAAGGCCCACTACGCAAAAGCCTGTCAAGTAAGGCCCAACATCCGGCGCACGCRGNRAGGTCGCGCCAGTCAGTTGCGAGCRATTCGAGGANTAGCACTACTNCTAGANACGGCCGGTACAGNRARCCYGCTGGTAGGYTNCGRCTTAATTAGCTCGYYCCGGAGCAATCGTCCTCGTYGTYAGNGNCTACTTCGGTGRCRNACANCGCATTAGACNCACRCAGCAGCGGCAGCGTYTTNNAAGNNNCTARACGCCYACATCGTCTCGGGCTAATACTTCTCCNCACYYCTGATCACCGGTGAGCACTAGGYGCCNNNTCCTGGATAACCGAAAYGGGCCTCCAAAGCTCGCCRTCTTACAAGACTCCCGACAGAATRCRGCGTATTACCCGCGTCAACTTTGAGCGTCGGTGACGAYGCRCGACCGCCGGTTTATGAACCGCACCACCGGRNNTNCAGGAGAAGAGACGYGNYATCTTAACGCAGGTGTCRAYCYGTNTYAGCCGAGRRAATGRGANCGNRRTCACGTGACCCCTTACTCCGCATGGNCRCYRGCCTCAANNRRNNGTGTCTANATCGATCCCRGRYCTGAAAGTCGAACATNTCCTTCTAGCTGRRNTACCCCCCGATCCGTGCATAAAGTTACCACGCTTACCRNNNTGGGANCCRCCGGGNTTGCGARGNTTAAAGCAACCCCGTTGNYCYRTCACAGCNGTNNTAAAGCCTCACGTCCTTTATGGGAGACGGGGNNRAACATTAAGGYRCCGTCGGACCRTCCNGCNCGCRTCACGTRTGTAATAGTCNCTNGGTATGTGAGAGGAGTTCGGGCACCACTGTCAGGACCTCGAGTTTTCGGACTAGCCATACAGCTYAACNGACATTGGCRRCTCANNAGCGGTRGCYYRTNCCNCTAAAGNYNACTCCAGGGGACGCGGAAGCNTCAAAGTGYAATCANYNTNGTRTTGRTCRGACGAGAAGAGTAATATAAYYCCTATNTCGCAGGANTRGTYCRTGCCRRAGGAGACATCACCATGGAAAGTRNYCGCGAGNTGCGCTACNTGTCCNTTGTYRRTTTGCACTGCATAANNNNANCGTCGCTGCCTCRTCNYGACCGTACGCANNANNCYCCAGAACTTAAATNGCCGGCTACCCCGACGCCANCCTCGCNCAGCCNGAGCTACTGTATCTAAATNGRCTRTYYYCNTTGTAGTTGGCGGATCCCTTTTNTYCTCRYCGCNANRACNCACAGGCAGTTTGAGAAATCATACACGCGGTAAACCGTAGGGCNAGGCTANACAGCCTRAGGRGCCATCCTACTGCAGATGNGTCGCNNAGNCTCGNYYTAAATCGGGTTCATCGTGARCGGACTGGTCCGCAGGCGGCCTGYCTGATCGNNACYRCRTYGGGCCCCTRCGAYYTNNCCGTYYGCGCCCNGGCGCCGTGGCCGTTCCGTRTNRCTGNTCCTGGGACTTRGATGCCNTTGTAAGCACCATGGAATAGRTTGTAYCACGNNACACGRCGGNNTAGNAYGCCNRCGGYACCTTRYCTTAGNGRTCAAGTTTCCTCGTGTTGCCCCCGGGYAATNATGCGTAACGGGATGNGNTYNNGGCAGCGCGANTYAGATGCGGTCTCNNGTCCCRAARAYTGCNNTNATNNATGCGCACGTCGAATGCCCCGYGGCRTTGCTTRCGGNTRCYGGCGRCYRTCATNGYRTTYATGRTGTTATGAGCTCTCCTTAGGCGCGGACCGAAAACGNNNCGATCATGCGTAGAAAGCCTGAATATGNNCAAAGCGTTAGCANGGANYTTGAAAGCGCGAATATTACGGCTCACTCCTATACAGGTTTAGGTACGRCYYCACNCTGGCTGTACCCYYGGGGGNCCGCCGTYGCGCARNTTCNNNGGGGGGGAGCYRACACYYTGTTGTCGCGGGCTCNCGAGGTTACNNAATNGTGGCGTNNCCTCAATCRAARGAGAGTTNYRYTACCNCGGAGGACGCCGNCATGGTGTCGCTTCANYCYYNATACGTATTGCANNGCAACGAAGCCAGGCGTNRTATRAYANAAAGAYGACAAANNAAGATACGCAGTTTGNNNCGACGTNGCYANCACGNACCCTGATCGTGTNYCGNANCRACACAACNTCRGTYTCTGANTTNANCCRGGTGCAGACGTCTCAGTCAGCNGGGATTACTAGCCGTGTAAGCCGNGAGCCGNGCCAACCCGARYCTAGCGRGTCGTGAACAACCCGGAGGGCACCACACATCNATNTTTCACTCAGGGCGGCAGCGCCAAAACANTYNGTTCCCACCTGAGGTTAGCCCTRNRAYCTAAGGTAAGCCCCACATGNNNNNNNGAGGCCGNATNNGGATGTCCGNTCCTATTCCTCGAACCGACCATAAAGTACGTGNNNNATTYGTTAACTTGACCYGCTAGRCCRCRTGTTGGYNCACAGCTGCCCCAGTACAATGGGACTTCCAAGNTTTACCACCAAGCGGCAGGRRYYNGTTGGNCCGYCCCCAATCTGATACAATCTACAGCAAACCGNTGGNNGGCACTNNCCAGCATTTGCATCCACATNCGTCNCYRTCTTTTYCATGNCTGGCYGGCGGAATAGAAGATCTCGATCTGTNANGRNTAGCYGTCACAGAGGTTACYGGCTRTCATRTNGCGCGGRYTGTNCCCAGATTACTCTCYAAGTNGYARRTAGACCCTNRCTTAGGGCGTCRAGAYAYAANCGNAATCGCCTCAGGTYCTARGAACGTGGGATCCTGATCGCTNRAYCCCACCTGTCRNAGYTACNGNTRCGTCTTCGCTACGACTGGCTAGCTTNAACCCTAGATAACGRGGRTGTTAAGGGACTCTAGAAGGGCATYCTTGGRNTGTNCGTATGATCCTTNNNNNGCTTYGTGGCATGGRGGCAGANCCCCTAGGCTGAGNCNNAGGCCNTYCACAAACNNGYNARYTGGRCCAGAGGCCGACANGGGCGCNACGTAACATRNCNYCACGGAGAACACTGAGCTATTTTAGCAGRAGCCGGNGTGCNNRCCCGTACCAYNCGCCTGRRGCTTATCTGTCRGTANRRCCATGGCGCCAACTRRAGAGTGNNCGGTANNYTNTATAAATRCCCTTTCCGGGAGCATNNRNNYGYNCGGNCCCGAGNTNNGCCATTAACYAGGNATTGCCYNYGYACGGCAACGGCCCGRTGTCRCGAYCTCGGGTYRGTATGTGGNNGAYTTTTTNRRTNCGACTCGATTRCCCNAGATGGAGGCCCCAGCATCCTTACGCTGGGATTTATCGTGAAAGYGATNGGGCRAAGCACTAYCTGCTGGCGRNGTCCGTTGRTGACCGGANCNAGNGAYCAGATTTTGTANNCGGTATYYYAYARTGGNGRGTYATNNCATTCAGTCCGACGCGRYCTCGCANTGCGCGGG

Br30-1 GCGATTTACTNGYCRNCNTAATGGNNGCCRGRGTCGTCGACCTAGACGGGTCTAGGANGTTTACCCACTNANNGNGATCTACTGGCCATNNCGCYYYTTGTCTARGGRGYCCNCTGCCCGGGRTCCATCTTGGGTATGGGCCRTRCTGCGCTTACAATGGCYTNNCAGCGYACAGGAGAGCCGAANGNNNNNNNAGGTTTGGANNTGGCCCTGGNACRGYRGATCTNAYAATACGGCAGTCNNYAAGTANGTTNGGYACGNANRYCCTAGGYCTCATGAGGARRTTTTNRANAANYGYGGATCRYYCRYNGGNCATNRNTYATTYCCARTNCCCGNGGATAGTYYGGTTRATGRCTTAGNYTNRCGAACGRTGGAGCCATTCYGGAGCTGCTAGRNYGGGGCGGGCGCCGCAGGYYNNGAACCNNGGTGCRTNNNATTGCCGCCAGCTAGCCTTCAARCGCYCTCNCTYTACTGNTGANYGGNCTGCATTTGGTTACCTTTGGCTGYGAATGAACCNTAGCGGANACAGCTAANYATCNNNNTNTARACTCRNNTAAGAGTCTAGAGCCGACNCRTACNRTTNCAACACCRACGGGGCGAGRANAGARCGYYTCCGGCTACCAACRGTGGACRTNCNCCTNRGAATRARCGACAGCNGAGGCTGAGYAGRCCNRTCTCCYNNATCCNNTCCYYTRNGTGNAAGCCCCYRCCNCCGCTTACGCCAGTACCTAGCNNANTGCGCCGTGCGRTGGGTACCARGTGGCTCCTATYGYRTGNGAATRGTAGGCACTACCAAGGACNRGCCGGCTGGTAGCTAGGRRCTGRTNTTATGTGAGTACATGCCCCGTCCGCANTNCRGNTNCNGCTAGCCCTGTACACAACGGAATTGTTCTNAGGCTYGCGAATNNGNNTNAGTTCGGARANNCRNGCNYCNTGNNCCGGTCAGYCACTRAAGTTGYRANTNGNAGYAACTCGCGGNAGCCATNNCNYNTAARTTCGRRNTAGCCCGCGGTGTANNGGCGGGCCRCRTCRGCGARGGCCGCTTTRYYRCRAGGGATAATCACACGATTAAACTATGTYNGRAARAACGCACNCCTCTTACCRNAGGYNACACGCTGTGGCTCCACYTTYNGNGGCCGGGGGATTAGGCACRTCCRGGNRTCGCTAAGTACTNGGGCCTAGRAACAAGNATGCCCARANGATGGGGCCAGCCACACCGACAARTTCYCGCRGRGGRCRYCAGTTGGCTTGCTGCAGRGNATTTCTNTGGCCCGACGGTAACGCGNCNGYNNYYNRAAAGACNTANRCGTRNGATCRGACNCCTGAAATGTYATTTAGGAAYCCANTAGCACTGACGTATACGTATCAGGCGYNTNGGTANCTCNNNGGGCGGCTRRCGCNNNTGNNRTGGGCTCGCCCCTGGACRAAGGARGGGAAAAACYTCRRGACCCTRAGGNNNCTGCGACCCTAGTGAANATAGAGNATNGCGTAGTTTAGCATATGGTCTCTGGRNGCRRCCGTYCYCTGACCCACCCAGTGGCTTCCGCAGGAYTTTGTACAGCCAGAAAGYGAACCTCCCGCACCTCNCNAGTGGTTTNNCANRTANCYTACCYACRNCGCTCCGACGGAATTGCTNNRYTAGTGCNGTYGTGAGCCCTNNATCNGTNAACYGGGTGGGCACTRNTCTCGGACTCGTAGTACTCGATNTAAGGTCGGRNAYNANTATRTRCAAGTGGACTGTATTAGCCCGCTGCANTNARAYGGCACGGTNTGTTTGNNNNNCGTCCNNTTGGTTANYNGCYCGAAGCCCGTCCTCACGCRTCCCRRNGAGTGACGNTGNGGCCGCRGATYCTCGCTGGACANYTTCCAAAGCGYCCAGAGGATACTTCGTTCTCTTTYCATTTACGCTANCCAATTTCGYAATRTGCCGGCACCGYAGAGCTGGCGCCGCANTRGGTGTCTTGCAGAATGGCNCAAGYGNCRGCGGCGCGTCACNATCRAGCCCCTGGCAYGGCGGCGACAGCNRGCCGACCCAYYYRATRRANCGYGTCCCCACGAGGYAGGCGGGAYGAATATAGAGACGCTATNRCGGGGTTTAYNCNCACTCCACACGRNYCNNCCGTTAYGNAAGGAYGGTNCGGGCCCCCARRNNNNTATGGGNNGNNATTGGRNNGYYCGCAAATACCTRNGAAGTACTCGACCGCTAATCGGCGCATCGTGGTTANGGCGCCTCGCNTCTGTATTAACATTGRCNACGTAGATGNNTGTNGAACTTTGCRGYGGGAAAGAGCRAGACCATGGCAACGGGCTCTGCGACCACATTAGTTACYTAANGYGTCAAAGANTGATTGTTCGTGTCCNNCCCCGCGGCGCGAAGNRANTCAAAATGCCGCTACGCGAAAGCCTGTCAAGTAAGGCCYAATCTCCNGCGCACGCRRNGNGGTCGCGATAGCGGGYTGCGAGCGANTCNNGGACTAGCACTACNNCNAGANACNGCCGGTACAGGAAGCCCGCTGGTRNNNNACGACTTAATTAGCCCGYYCTGAAGCAATCNTNCTCGTYGNNNGNGNCTACTCTAGYGGCRTACATCGCATTAGACCYAYGNNNNAGCAGCAGCGTCTNACAAGNNGCTAGACGCCTACANCRTCTCGGNCTAATACTTCTCCNCNCYYCTGATCACCGGTGAGCACTAGGTGCCTGTTCCTGGATACYCGNNNCGRGCCTCCTAAGNTCRYNATCTTACAAGACTCCTGAYAGNRTGCGGNGTTTTATCCATGTNACTTYTGAGCGTCGGTGRCGATNCACGACCGCYGNTTTRTRARCCRCNCCACCGGNNNTNCAGGRGARGAGACGCGGYRYCNTAGCGYRNGTGTCRAYCYGTNTNAGYCGRGRRAATGRGACYGCRRNCACRYGAYYCCTTACTCCGCATGGACGCYGGYNNCAAGCGRGGGTGTCYACACCNATCCCGGRCYYGARARTCGAACATCTNNTTCTNGYYGNGTTACCCCCCGANNCGTGCATAAAGTTACCACGCTTACNNTNNTRGGANCCNCCGNGTTNGCRNRGATTAAAGCAACYCCGATGCYCYRNCACAGNGGTATTNAANYCTCRCGTCCTTTGGAAGAGACGGGGTGAAACAYTNAGGYRCYGTCGGACCRTCCNGCCCGCRTCACGTGTGTAANAGTCNCTRGNTATGTGAGAGGAGNTCGGGCACCANYGTCNNGRCCTCGAGTTTTCGGACTAGCCACCCAGCTCAACNGACATTGGCAGCTCAGAANCGATGNCYYRYNCCTCTARANGTTACTCCAGGNGNNNCRRAARNNYCAAAGTGCAATCANYNTNGTRTTGGTCGGACGANAAGAGTANNANRAYYCCTATNTCGCAGGATTAGTTCGTGCCGRAGGAGTTNTCACCRANGANAGTNNYCGCGAGAYRCGCTACNTGYCCTTTGTTNNTYTRCATTGCAYAATATAAACRNCGCTGCCTCRTCNYNACCRTACGCANAAGNYYNNAGAACCNAAATCGCCGGCTACCCCGANGCCARCNNNGCACAGCCAGAGCTNNNGTATNTAAATNGACTRTTYYCATTGTAGTTGNCRGRYCCCTYTTANCNTCNNCGCNANRACNCACAGGCARNTTGAGAAATCATACACGCGGTAANNNNNNGGNYNNGGCYACACAGCCTNAGAGGCCANCCTATTGCAGATGAGTCGCGCAGNCTCGCTCTAAATCGGGTYCATCGTGAGCGGANCGGTCCGCAGGCGGCNTGTCTGATCGNNACYRCRTYGGGYNCCTGCGACCTACNCGTCCGCGNCCGGNCGCCNTGGCCGTTCNNNGTTRCTGATCCAGGGACTTGGATGCCCTTGTAAGCACCCCTGAATAGGTTGTACCRCGCCACACGACGGCATAGGACGCCAGCGGTACCTTACYTTAGCCGTCAAGTTYCCTCGTGTTGNNCCCNGGCAATCATGCGYNACRRRATGGGRTCATGGCAGCGCGANTNAGGYGCGGTCTCGNGTCCCRAARAYTGCNGTYATNNATGCGCACGTCGAATGCNCCGTGGCGTTGCTNRCGGRTRCYGGCGRCYATCATNGTGTTCATGGTNTTRYGAGCTCTCCTTAGGCGCGGACCNAAATCNCCNCGATCATGCGTAGAARGCNYRAATRTGNNCAANGCGTYARCACGGACYTTGAANGNGCGAATATTNCGGCNCANTCCTATACAGGTNTAGGTACGRCYYCACNCTGGCTGTNCCCCTGRGNNACNGCCGYCRNGCAAATTCACGGGGGGGGAGCYRACACYYTGTTGTCGCGGGCTCNCGAGGTNNNNRRAYAGTGGCGTCGCCYYAATCRAARGAGAATTNTGTTRYCTCRGRGGACGCCGGCATGGTGTCGCTNCANCCTTGAYAYGTNTYGCAACACANCGAAGCCAGRCGTTGTATAACAGAANNAYNACAAAACAAGATACGCAGTANGGGANNNCGTNGCYACCNCGGANCCTRATCGTGTNYCGNACYGACACAACATNAGTCTCTGACTYTANCCNGGTGCAGACNNCTYAGTCAGCNGGGATTNCTAGCCGNRTAAGCCGANNGCCGNGCCAACCYGANNCTNNNNANTCGYGNACNNCCCGAAGAGCGNNNNACATCNATNTTTCCCTCAGGGCGGNAGCGCYNAAAYANTCNGTTTCCACCTGNNNNYAGCCCTRAAATYTAAGGTAAGCCCYATGAGCGATTGGGAGGCYGGATCCNNATGTCCGRTCYTATTCCTCGAACCGACNRTAAAGTACRTGAAGAATTTGTTARCGTGACYYGCTRNNCCNCGTGTTGGNNCACAGCTGCCTCRGTCTAATGGNACTTCCAAGGTTTACCNCYNAGCRRCAGGRRTTAGTTGGCCCRYCCYCAATNTGAYAYAAYNTACAGYAAGCCNNTGGARGGYACTCGCCAGCATTTGCATCCACATGCGTCNCYRTYTTTTCCATGNYTRRNYGRCGGAACGGAGGTTCTCGATCTGTGANGNNNAGCCGTCGCAGANGTNRCYGGCTGTTGTGTRNCRCGGRYTGTGCCCARNTTACTCTCCAAGTNGYARATAGRCCYTNANNTRGGGCGTYGAGANATAATCGCAATCGGCTCAGGTCCTNRGAANNTGGGATCCTGANCGCTNRATCCCACCTGTCRNAGCTACCGCTACGACTTCNCNRCGACTGGCTAGCTTNRACYNNNNNTAACGAGGATGTTAAGGGACTCTAGAAGGNCANCCTTGGGATGTNCGCNTGANNCTTAGACCRNNTTGTGGCATGGGGGCAGAGCCCCTAGGCTGAGCCCNAGGCCGTYCGCAAACTNGYNRGTTGGGCCAGAGGCCGAAAGGGGCGCAACRYARCATRNCTYYACNRAGNACACYNNGCTNTNNTAGCAGNAGCCGGTGTGCATACCCGTACCANNCGCCTGAGGCTTRTCTGTCRGTANARYCATGGCGCCAACTRGNGATCRCACGGTACTTTGTRTAAATGCCYTTTCCGGGAGCATTGNNTCGNNCGGTCCCGAGNTNNGCCNNTARYYAGRCATTGYCTTTRCACGGCNACGGCCCGATGTCGCGACCTCGGGNCGGTATGTGGNNGAYTTNTTRRRTCCRACYYGATTGCCCRAGATGGAGNCCCCAGCATCCTTNCGCTGGGRTTTATTGTGAARRYGATCGGGCAAAGCRCTAYCTGCTGGCGRNGTCCGTTRGTGANCGAATCAAGTGAYCAGATTNNGNACCTAGCGTCYTACARTNNNNNNTYNTYRCATTCAGTCYNACGCGGTCTCGCANTNCACATT

Br30-2 GCGATTTACTNGYCRYCNTAATGGCCGCCNGNGTCGTCGACCTAGACGGGTNNNNGNGGTTTACCCACTNATCGCGATCTACTGGCNATCCCGCYYTTTGTCTARGGRGTCCACTNCYCGGGATCCATYTTGGGTATGGGCCATACTGCGCTTACAATGGCCNGGCAGCGTRYRGGAGAGCCGNANGTACAGACAGGTTTGGAYYNGGCCCTGGAACGRTAGATCTYACAATACGGCAGTCTNYAAGTANGTTNGGCACGNACRYCCTAGGYCTCATGAGGAGGTTYTNRANAANYGYGGATCRYYCRCAGGNCATTGAYYATTYCCAGTNCCCGGGGATAGTCCGGTTGATGACTTAGCTTGGCGAACGGTGGAGCCATTCYGGAGNTGCTAGATTGGGGCGGGCGCCGCAGGTTCGGAACCTCGGTGCATTACANTGCCGCCAGCTAGCCTYNAAGCRCCCTCTCTNTACTGNTGANTGGGCTGCATTTGGTTACCTTTGGCTRYNAATGARCCCTAGCGGACACAGCTAAGTATCCTNGTYTARACTCAAGTAAGAGTCTAGAGCCGACGNNTACGGTTTCAACACCRACGGGGCGAGRANAGAACGTCTCCGGCTACCANCGGTGGACRTNCNCCTTARAANAARCGRYNGCNGAGGCNNNNYAGRCCNRTCTCCTCRATCYGNTCCTCTGTNCGCAAGCCCCYRYCNCCGCTTACGCNAGTANCTAGCNNACTGCGCCGTGCGGTRRGTACCAAGTGGCTCCTATCNCRTGCGAATGNTAGGCACTACCAAGGACNRGCCGGCTGGTAGCTAGGNNCTGRTNTTATGYGAGTACATGCCCCGNYCGCAGTACAGATACAGCTAGCCCTGTACACAACGGAATTGTTCTGAGGCTTGCGAATNYGRNTRAGNTCGGAANNNCRRGCNYCNTGRNYYGGTCAGYCACTAAAGTTGCAAATTGTNGTAACTCGCGGTAGCCATGRCATCTAARTTNGRRNNNGCCCGCGGTGTAAAGGCGGGCCGCRTCRGCGARRGCNNCYYTACTACRAGGGATAATCANACGATTARAYTATGTCNGAARGAANGCACYCNTCTTANCAAAGGCCACACGCTGTGGCTCCACTTTYNGNGGCCGGGGGATTAGGCACRTCCGGGGGYCGCTRANTACTAGGGCCTAGRAACAAGCATGCCCARANGATGGGGCCAGCCACACCGACAARTTCCCGCGGGGGACATCNNTTGGCTTGCTGCNNAGAATTTCTTTGGCCCGACGGTAACGYGNCRGYNYTTGAAAAGACATAGRYRTRNGNNCGGACNCCTGRAATGTCRNTNAGGAATYNACTAGCACTGACGTANACGTATCAGGCGCATCGGTAACTCNNNGGGCGGCYGACGYGAANNNNATGGGCTCGCCCCTGGACRAAGGARNGGRRAAACYTCRRGACTCTRAGGACTCNNCGACCCTAGTGAACATAGAGCATCGCCCAGTTTAGCATATGGTCTCTGGRNGCRGCCGTYCYCTGACYYACCCAGTGGCTTCCGCAGGATTTTGTRCRRCCAGAAAGNGAACCTCCYGCACCTCCCAAGTGGTTTTTCANRTANCCYACCYANGTCNCTCCGACGGAATTGCTCTRYYNGTGCTGTTGTGAGCCCTNNATCTGTTAACCGGGTGGGCACTRNTNTCGGRNTCGTAGTRCTCGATNTAAGGTCGGRNGYYACTATRTRCAAGTRGRCYRNATTAGYCYGCTNYATNGARANGGCAYGGTCTGTTTGNNNATCGTCCNNTTGGTTANYNGCYCGAAGCCCGTCCTCNNNCRTCCCGANGAGTGACGGTGAGGCCGCGGATYCTCGCTNGACACTTTCCAAAGCGCCCAGAGGATACTTCGTTCTCTTTTCATTTACGCTACCCAATTTCGTAATATGCCGGTGGCGYARAGCTGGCRCCGCARTGGGTGTCTTGCAGAAYGGCTCAAGYRNCRGCGGCGCGTCACTATCGAGCCCNTGGCAYGGCGGCGACAGCNRGCCGACCCAYYYRATRRANCGYGTCCCCACGAGGYAGGCGGGACGAANATAGAGACGCTATNNCGGGGTTTAYNCNCACTCCAYRYGGGCCATCCGTTACGCAAGGNNGGTNCGGGCNCCNAGRCCGGTATGGGGCGTAATTGGRGTGYTCGCRAATACCTRNGRANTACYCRRYYGCTRATCGGYGCANCGTGGTNANGGCGCCTCGYTTCTGTATTAACATTGGCTACGTAGATGCATGTCGAACTTTGCAGYGGGARAGAGCRAGAYCRYGGNNRYGGGCYCYGCGACCACATTAGTNACTTAAYGTGTCAAAGANTGATTGTTCGTGTCCGCCCCCGCGGCGCGAAGNRACTNAAAATGCCGCTACGCGAAAGCCTGTCAAGTAAGGCCCAATCTCCNGCGCACGCAGCGAGGTCGCGATAGCGGGNTGCGAGCNANTCNNGGACTAGCACTACNNCNAGAAACGGCCGGTACAGGRARCCCGCTGGTRNGNTACRRCTTRATTAGCCCGCTCYGRAGCAATCGTCCTCGTCGTYAGGGGCTACTYCGGTGRCRTACANCGCAYTANACCCACGCAGCAGCNGCAGCGTYTNRCAAGNNNCTARACGCCYACANCRTCTCGGGCTAATACTTCTCCNCNCYYCYRRTCACCGGTNNNCACTNGGYGCNNGNNCNNGGATANCCGAANYGRGCCTCCNARGNTCNNCATCTTACAAGACYCCTGATANTGTGCGGCGYTTTNTCCATGTNANYTCTGAGCGTCGGTGACGATACACGACCGCNGNTTTRTNANCCNCNCYACCGGGCTTNCAGGGGAGGAGACGYGNTATCTTARCGYNNGTGTCGANCNGYATCAGTCGGGGAAATGGGACYGCRRNCACGCNAYYCCTTACTCCRCATGGACGCTNGTCCCAAGYRAGGGTGTCTACACCRATCCCGGGYCYNARARTCGAACATCTCCTTCTNGYYGRGTTACCCCCCGATCCGTGCATAAAGTTACCACGCTTACNGTAATRGGACNNACCGTGNTTGCRNNGATTAANGCAACNCCGNTGCYCYRTCACAGNGGTATTAAAGCCTCACGTCCTYTRNAANAGNCGGGGTGAAACACTCAGNCACCGTCGGACCATCCGGCCCGCATCANGTGTGYAANAGTCNYTRGNTATGTGAGNGGAGTTCGGGYRYCANYGTCNNGACCTCGAGTTTYCGGACTAGCCACCCAGCTCAACNGRYRTTGGCAGCTCANAAGCGGNRGYYTAYCCCNCTARANGYNACTCCAGGNGACGCRRAARNNYCAARGTRCAATCANYCTAGTRTTGGTCGGACGARAAGAGTAGGACGATCCCTATTTCGCAGGANTRGTYCRTGCCGRAGGAGTTATCACCAANGANAGTNNNCGCGAGATGCGCTACNTGYCCTTTGTTRRTYTRCATTGCATNNTATAANCNNCGCTGCCTCGTCNYGACCGTACGCANAANNCNCCAGAACYCAANTCGCCGGCTACCCCGANGCCARCCTCGCNCAGCCNGAGCTACTGTATCTAAATNGACTGTYCTCATTGTAGTTGACAGGCCCCTTTTATYCTCACNGCTATAACACACAGRCARTTTGAGAAATCATACACGCGGTAAACCGTAGGGCNAGGCTACACAGCCTRAGAGGCCAGCCTAYTGCAGATGAGTCGCGCAGNCTCGCTCTAAATCGGGTTCATCRTRARCGGACCGNTCCGCAGGCGGCNTGYCTGATCGNNACCGCATTGGGCCCCTGCGACCTACGCGTCCGCGCCCNGGYGCCGTGGCCGTTCCGTRTTRCTGATCCAGGGACTTGGATGCCCTTGTAAGCACCCYNGAATAGGTTGTAYCRCGCCACACGRCGGCANAGGACGCCAGCGGTACCTTACYTTAGCCGTCAAGTTTCCTCGTGTTGNNCCCGGGCAATCATGCGYNACRRRATGGGATCATGGCNNCGCGANTCAGGCGCGGYCTYGGGTCCCAAANATTGCANTCATTTATGCGCACGTCGAATGCNCCRYGGCRTTGYTTACGGGTRCTNGCGRCYRNCATAGYGTTNATGGTRTTRYGAGCTCTCCTTAGGCGCGGACCGAAATCRCCACGANCATGCGTAGAARGCNYGAATRTGTCCAAAGCGTTAACACGGACYTTGAAAGCGYGAATATTRCGGCYCAYTCCTATACAGGTYTNNGTACGGCTTCACACTGGCTGTACCCCTGGGNGACYGCCGYCNCGCAAATTCACGGGGGGGGAGCTGACACCTTGTTGTCGCGGGCTCNCGAGGYAACNGRANNGTGGCGTCGCCYYAATCGAAAGAGARTTNTGTTACCNCRGRGGACGCCGGCATGGTGTCGCTTCACYCTTNATATGTATTGCAANACACCGAAGCCAGGCGYYGTATAACAGAAAGAYGACAAAACTARRYRCGCAGTACGGGACGACGTNGCTANNACGGACCCTRATCGTGTTTCGTANNRACNNNACATYNGTCTCTGRCTTTANCCRGGTGCAGACNYCTYAGTCAGCTGGGATTACTAGCCGTRTAAGCCGAGTGYCGRGCCAACCCNNGTCTNNNNRGTCGYGNACNNCCCGAAGAGCGCCACACATNTATATTTCNYTCAGGGCGGNAGCGCYNAAAYACTCNGTNYCCACCTGTGGTTAGCCCTAAAATNTAAGGTNAGYCCTATGAGCGATTGGGAGGCYGRATCCGGATGTYNGNTCYTATTCCTCGAACCGACCRTAAAGTACRTGAAGAATTYGTTARCTTNACYCGCTRGACCRCGTGTYGRYNCACAGCTGCCTCRGTCTAATGGGRYYTCYAAGGTTTRYCTCYNAGCGGCAGGRRTTAGTTGGNCCRCCCCCRRTNTGATACAAYNTACAGYAARCCNNTGGARGGYACTCGCCAGCATTTGCATCCACATGCGTCCCTGTYTTTTCCATGNYTRRYCGACGGAACGGAGGTTCTCGATCTGTGANGRNNAGCCNNNNNNNNTGTAGCCGGNTGTNNTGTRNCRCGGRYTGTGCCCAGATTRYTCTCCAAGTGGTAGATAGACCCTTACCTAGGGCGTYGAGAYATAATCGCAATCGGCTCAGGTYCTNNGAAYRTGGGATCCTGATCGCTCAATCCCACCTNTCAARGCTACCGCNRCGNNTYCNCNRCGACTGGCTAGCNTNRACYYYANNTAACGAGGRTGTTAAGGGACTCTAGAAGGNCANCCTTGGATTGTNCGCATGATCCTTAGACCRCTTTGTGGCANGGGGGYAGAGNNCCTAGGCNGRGACCNRGNCCGTYCGCAAACNGGCCRRYTGGGCCAGAGGCCGANAGGGGCGCAACRYARCATGACTTCACGGAGTACACCGAGCTTTCCTAGCAGGAGYCGGTGTGCATACCCGTACCATTCGCCTGAGGCTNNTCTNNCRGTAAAGNNNTGGCGCCAACTRGNGANYRCCCGGTACTTTGTATAAATGCCCTTTCCGGGAGCATTNRNTCGYTCGGNNNNNNNCTATGCYRYTARYYAGRCATTGYCTTTRCACGGCNACGGCCCGATGTCRCGACCTCGGGNNNGTATGTGGCAGACTTTTTAGATCCGACYCGATTRCCCAAGATGGAGNCCCCAGCATCCTTNCGCTGGGATTTATTGTGAARRYGATCGGGCAAAGCRCTAYCTRCNGGCGGAGTCCGTTRRTGAYCGRATCAAGNGAYCAGATTTTGTACCTAGCGTCCTACARTGGCGGATTNTCGCATTCAGTCTTACGCGRYCTCGCANTGCACATT

Br32-1 GYGATTTACTCGTYGNNNNAATGGNNNNNGGAGTCGTCGACCTAGACGGGNCTAGNRGGTTTACCCACNGACTGTGATCTACTGGCYATCACGCCTYTTGTCTGGGGAGTCNNNNGCYCGAGANNCATCTTGGGTATNGGCCATRCTGCGCTTACAATGGCCTGGCAGNRNGCAGGAGAGCCGAAAGTAACANNAGGTTTGGACCTGGNYYNGGNANGNYAGATCTNANAATACGGCAGTCNNTNNGTAGGTTGGGTACGAACATYNTAGGCCTNRTNAGRAGGTTTTNRACRANCGYGGATCACNCNCAGGNCATTGAYNNTTYCCANTGYNNNNNNNNAGYYYGGTTGATRGNNTAGNTTGRAGAACGGNGGAGCCATTCTGGAGCTGCTAGGNYGGGGCGGGCNCNNCAGGTTCGGAACCTARGTAYATTACATNGCCGCCAGCTAGCCTNNAARCGCTCTCTYTNNNNNNCTGAATGGGCTGCNNNNNGTTACCTTTGGCTRCNNNNGAANCCTANCNGACACAGCTANNYATCCTNGTCTARACTNNAGTAAGNNTNNNGARCCGACGCGTACGRTTNCAACACCRNNNGGGCRANGANAGAACNNCNNCGGCTANNNACRGTGGACATTCCCCTAGAAACAAGTGNNNGCTRAGNNTGAGCANGCCAATCTCCTCRATCYNNTCCCTTRNGTGNNNNCCCCCACNNCCGNNNATNNCAGTACCTARCNNACTGCNNCGTGCGGTGGGTACCNNNNNGCTCCTATYNCNNGCGAATGGTNGGCACTACCAANNNCNNGCNNNNNGGTAGCTAGGNNCTGANNTTATGNGAGTACATGCCCCGCTCGGANNACAGANACNGCTNGCCCTGTACACAACGGAATTGTTCTNAGGCTTGCGAATACGGATAAGTNCGGNAAAACGGNCGCCNTGGTCCGGNNNGTCACNAAANTTNNNANTNGNAGTAACTAGCGGCANNNATGRCTCCTANANNCGGRATANCCCNCGGTRTAGGGNCGRNCCGCGTCRGCGARNGCCGCNTNGCYGCRAGNGNNNATCAGGCGNTTAGATTATGTCAGRARRAANGCACTCYTCTYAACGCAGGTAACACGCTGTNNNNNNNTNNNYGGCGGCCGGGGRRTTAGGCACGTCCGGGGGYCGCTGACTACNNGGGNCTAGNAACAAGAATGCCCANACGRTGGGGCCAGCCACACCGGTAARTNCYCRCARGGGGCATCAGTTNNNNNGCTGNAGRRNAYYTCTNTGGCCCGACGGTAACGCNNNAGCCCTTGAAAAGAYNTRNGYRYGAGATCGGNNNNNNNNAATGTCRNTTAGGAATYNACTAGNACTGACRYRNAAGTGGCCTGCGCCTNNRTNNCTCTCCGGGCNRCCRRCGCNNNTGNNRTGNNNNCGCNCCTNGACNAAGNAAGNGAAAAACYTCGGGACTCTGAGGACTCNNCGNNNNNARTGNNCATAGAGNANNGCCCAGTTTAACATTNGGTTACTGGRNGCRRCYRTCCTCTGACTTACCCAGTGGCTTNNGCAGGACTTTGTACAGCCAGAAAGCGRRCCNYCTGCACCTCCNNAGNGGTTTNTCAAGTANCCNNCCCANGTCGCTCCGAYGGAATTGCTCTGCCTGTGCTRYYGTGAGCYCTNNATNNNNNNACYGGGTGGNNACTGGTCYCGRRNTCGTAGTACTCGANATRAGGTCGGGGATCACTATRTRCANGTGGACYRTATTAGNCTNCTNNATTNARANGGCACGGTCTGTTTGGATATCGTCCGCNTGGTTAACTGCTCGANNCCCRTCCTCACGAGTCCCGAARAGTGANNNNGAGGNCGCGGATNCNCGCTGGACACTTTCCAAAGCGTCCAGAGRATNNNTCGTTNTCTTTYNNNTTACGNTANCCAATTTCGNAATNTGCCGGCACCGCANNNCTGGCACCGCARTGRGTGTCTTGCAGANNGGCTCAAGCNTCGGCGGNGCGTCAYNATCANGCCCCTGGCAYGGCGGCGACAGCTAGCCGACCCANNNNNNNNNNCNNNNCCCCACGAGGNANGCGGGACGAAYACGGAGNNNNNATGACGGGGTTTAYNCACRTTYCACGCNNNYNNNNNGTTNCGCNANNNCGNTNCRGRCCCNCAGRCCGNTATGGGGTGTAANTGGGGTGNTCGCAAATACCTRNGGANNACNCRRYYGCTRATCGGCGCATCGTGGTGANNNCGCCTYGCTTCTGTAYTAANATTGGCAACGTAGATGCATNNGGAACTNTGCRGNNNNNAAGAGCGANNYCRYGGYRRCGRGCTCTGNGACCACATTAGTNACNTAATGNGTCAAAGAATGATTGNNCGTGTCCNYCCCYGNNGCGNNAAGNRNCTNAAAGGCCCACTACGCANAAGCCTGTCAAGTAAGGCCCAATCTCCNGCGCACNNNNNGCGGTCGCGATAGCGGGTTGCGANNGATYCNAGGAYTARCACNACNNNNAGAAACGGCCGGYACAGGRARCCCGCTGNTGGGYTACGACTTRATTAGCTCGYCCCRGAGCAATCGTCCTCNNNNTCAGNGNCTACTNNNGTGRCRTNCATCGCATTNNNNNYACGNNGCAGNGGCAGCGTNTTRCAANACTCYNNNCGCCTACANCRTCTNNNNCTNNNNCTTNTCCNNNCTCNNGGNNACCGNTNNCCACTNGGTGCCTGTTCCTGGATNNNCGNNNNGRGNCTNCANAGCTCATCATCTTACAAGACTCCTGACAGAATGCGGCGTNTTAYCCRYGTNAACTYTGAGNNTCNNNGAYGACGYGCNNNNNCCGGTTTNNNNNNNNNNCCACCGGGNNTGCAGGGGAGGAGACGCGGTATCGTAATGYRNGTGTCNATCCGYNTYARNCGRGGAAATGGGAYCGYRRTCACGTGACCNNNTACNCCGCATGGTCGATNGCCTCAAGNRRGGGTGTCNACRCCGATCCCGGGCCTGAGRATCGAANANCTNNTTCNAGCTGGGTTNCCCCCCGANNNNNGYNTAAAGTTANCACGCNNNCAGTANTGGGACCCACNNNGTTTGCGAGGATTANAGCAACYCNRNTGCCCCGTCACAGNGGTATTAAAGCCTCACGTCCTYTATRRNAGNCGGGGTGAAACATTAAGGCACCGTCGGANCATCCNGCACGCRTCACGTGNGNAATAGTCNYTGGRTATGTGAGANNNGTTCGAGNNCCAGYGTCTTGACCTCAGATTTTCNNNNYRNCCANNCAGCTCNRCAGACRTTGGNNNCTCACAAGCGRTAGCCCATCCCACNNNAGNTTNNNCCAGATGACRCNNNAGCCTCAAGGTANAATCANYNNNGTNTTGGTNGGACGARNAGANNNATATAATCCCTATTTCNCAGGANTRGTYCGTGCCGAAGGAGNYATCACCAATGAAAGTAACCGCGAGATGCGCTACNTNNCCNTTGTNAGTYTRCAYTRCATNNTATAANCGTCGCTGCCTCATNNNGACCGTACNCANAANNNNNNAGAACNTNANTNGCCGGCTACCCCGACGNNAGCCTCRYNCRGCCTGAGCTACTGTNNCTANATNGRCTNTNYYCATNGTNGNNGACNGGCCCCTYTYAATCTCRYCGCNANNACNCACAGGCNNNNTGAGAAATCTTACRYGNNGTAAACCGTAGGGNNNGGCCACACAGCCTGNGRGGCCANCCTAYTGYRGATRRNNCGCGCAGTCTCGGCCTGGNTCRGGTCCATCGTGNGCGGNNNNNNCCGCAGGCGNNNYRYNNGATCGGAACYRCGTCGGGNNCCTNNGAYNNACNCGCCTGCGCCCCGGCTCCGTGGNCGTTCCGYNTAACTNNNCCNNNNNCTNRGATGCCCTTGTAAGCACNNTGGAATAGRTTGTACCACGCCACACGACGGCANAGGACGCCAGCGGYNYCTTNNNTTAGNGRTNAAGTTTYCTCGTGTTGCCCCCAAGCAATNNNGCGTAACGGGATGRGGTCNNNNNNGNNCGNNNCAGATGCGGTCTCTGGYCCCNAAAAYYGCNNTCANNNANGCGYACRTCGAATNNNNNGYGGCRTTGCTGGCGGRTACYNGCGNCCRNCATAGTGTTCATGGTGTNNNGAGCTCTCCTTARGYGCNNNNYGNNNNNGCCACGRTNATGCGTAGNNGGCGCNAATGTGTCCAAAGCGNTAGCACGGANYTTGAAAGCGCGAATATNRCGGCTCACTCCTATACAGGTYYAGGTANGRCTTCACANTGGCTGTNCCNYYGNNNNNNYGCCGYCNNGCAAATTCACNGNNGGGGAGCCAACACTCTGTTGTCGCGGGCTCTCRNGGTNACGGNAYNGTNGCGTCNCCCTANYCRNAGNAGAATTCTGYTNNCTCRGRGGACGACNGCATGGTGTCGCTTCACTCTTGATATGTNTCNNNATGCAACGAAGCNAGGCNNNGTATANNNNAAAGATGACAANACNAGATACGCNGTACGGGACGACGTAGCCACCACGGRCCCTNANNNNNNTTYGNANCRNCACAACNNCGGTCTCTGGCTTTACCCGGGTGCAGACGTCTCAGTCAGCAGGGATTNCYRGNCGAATARGNCGAGTGTCGAGCCAACCCGAGTCTAGCGAGTCGTGAACAACCCGAAGRGCRCCACACANCTATATATCACTCGGGGCGGANGCGCCNNNACACNCAGTTCCCACCTGAGGTYAGCCCTGNRAYCTAAGGTAAGCNNNATGAGCGATTNNGARNCNGAATCCNRATGTCCGNTCCTATTCNNCGAACCGACNNTAAAGTACRTGRRGAATTTGTTAACTTGACCNNNNNGRCCGCATGTYGGYNCACARCYNNNNNGGTCTNNNGGGACTYCCAAGRTTTNCCNCYAAGCNRCAGGNNTTAGTTGGACCACNYTNAATANGAYANNAYNTACAGYAAGCCGNYRRARRRCACTCGCCAGCANNNGCATCCANATGCGTCCCTRTCTTTTTCATGTCTGGYCGGCGGAATAGANGNTCTCGATCTGTGNNGRNTAGCYGTCRCAGATGTAGCYGGCTRTTGTGTRGCNCGNACTGTGCCCAGATNNNNNTCCAAGTTGCAANTNGACCCTNACTTAGGGNGTCRRGAYAYNANCGYAATCGNCTCAGGTCNTAGGAACGTRRGATCNTGATCGNTNGAYCCCACCTNTCAAAGYTACNGNNRCGTCTTCNCNNCGACTGGCTAGCTTGRACYNNNNNTAACGRGGGTGTTAAGGGACTCTRGAAGGGCANCCTTNNNNTNYCCGTATGANYCTTAGACCRCTTTGTGGCATRGRGGCAGRGCCYCTNNGCNGNGCCCTAGGCCGTYCGCAAACNGGCCAACTGGGNCAGAGGCCGANAGGNGCRCAACNNNNNATAGCTYYACNRAGAACNNNGAGCTATTTTAGCAGGAGCCGGTGTGNNNNNNCNTACCAYNCGCTTGAGGCTTATCTGTCRGTAAAGTCATGGCGCCAACTGNNGAGTGCCNGGTACTTTGTNNAAATGCCCTTTCCGGGAGCATTNGATCGYNCGGNCCCGAGANNANNCATTAACYANGCATTGCCTTTGCACGGCANCGGCCCGGTGTCACGACCTCGRGNYRGTATGNNGYRGAYYTTTTAGATNCGACNNNATTACCCNAGATGGAGGCCCCAGCATCCTTACGCTGGGATTNANNGTRAAGACGATCGGGCANAGCRCTACCNGCTGGCGGAGTCCGTTGATGACCGRATCAAGNGAYCAGNTTTNNNAYYTAGCATCCTACAGTNNNNNNNTTTCGCNTTCAGTCYNNNNNNNNCTCGCAGTGCACATT

Br32-2 GYGATTTACTCGTYGTCTTAATGGCCGCCGGAGTCGTCGACCTAGACGGGNNNNNGRNGTTTACCCACNGNCTGTGATCTACTGRCYRTCACGCCTYTTGTCTRGGGAGYCCACYGCTCGNGANCCRTCTTGGGTATRGGCCATRCTGCGNYTNCAATGGCNTGGCAGCGTRYRNGAGAGCCGAAAGTAACANNAGGTTTGGACCTGGNYYNGNRANGRYAGNNNNCAYAATACGGCANNNTNYAARTAGGTTGGRNACGNACATYGTAGGNNTYRNRAGRAGGTTTTNRACRATCGTGGATCACYCRCAGGNCATYRNCCAYNYCCARTNYCCGNGGATAGYCCGGTTGATRRCTTAGNTTGRNGNNNNNTGGAGCCATTCTGGAGCTGCTAGGTTGGGGCGGGCGCCGCAGGNNNNNANYCNNRGTNCATTACANNGCYGCCAGCTAGCCTCAAANCRCYNNCTCTYTACTGNTGANNGGNCTGCRTYTGGTTACCTTTGGCTRNNAATGAATCCTANCRGACACAGCTAAGTATCNTNGTCTAGACTNNNNNNAGAGTNNRGARCCGACNCRTACGNTTTCAACACCRACGGGGCRAGGANNGAACGTCTCCNNCTACCNACRGTGGACNTTCCCCTAGAAACAARYGRYNGCTGAGGNTGAGCAGRCCAATCTCCTCGATCTGCTCCCTTATGTGCANRCCCCCACCCCCGCTTAYGNNAGTANCTARCGGACTGCGCCGTGCGGTNRGTACCAGGNGGCTCCTNNCNCNTGCGAATGGNNGGCACTACCAAGGACTAGCNNNNNGGTAGCTAGGAGCTRANCTTATGTGAGTACATGCCCCGYYCGCAGTACAGANNNNNNNNGCCCTGTACACAACGGAATTGTTCTNAGGCTTGYGAATNYGRNTRAGTTCGGANNAACNGNCNYCTTGGTYNGGTCAGYCACTAAANTTNCAAATTNNNGTAANNNGNNGCNGCCATGGCNYCTAAANNCNGRNTARCCCRCGGTRTAGGGNCGGGCCGCNTCRGYRARGRCCGNNNTACTRCAAGGGATAATCAGRCGANNAGATTATGTCANAAGGAACGYACTCYTCTTANCRNAGGYNACACGCTGTGGCTCCAYTTTYGRCGGCCGGGGRRTTAGNNACRTCCGGGGGYCGCTRANTACYNGGGYCTAGGAACRNGNATGCCCARACGNTGGGGCCNGCCNNACCGGTAARTNCYCRCARGGGGCRYCAGTTGGCTTGCTGYAGRRNAYYTCTNTGGCNNGACGGTAACGCGTCRGYNNYYNRAAAGACATAGGTRTGNGATCGGACGCCAGAAATGTCATTTAGNAANCCACTAGYACTNACGTANAAGTATCAGGCGCATCGGTNNCTCTCCGGGCGGCCGACGTGAATGTTNTGGGCTCGCCCCTNRNCGANGGAAGGGRRAAACYTCRRGACYCTGAGGACTCNNCGACCCTARTGAACNNNNAGNANNGCCCAGTTTANCATNTGGTYNNNGNATNNNNCYRTNCNCTGACYYACCCAGTGGCTTNNRCAGGAYTTTGTACAGCCAGAAAGCGAACCTCCTGCACCTCCCAAGTGGTTTTTCANRTACCCNAYCCNNNNCNCTCCGACGGAATTGCTCTGCCTGTGCNGCTGNNNGCNCTNNATCTRTTAACNNGGTGGGCACTGGTCYCGGNNTCGTAGTACTCGATATAAGGTCGGNNRYYACTATGTGCAGNTGGACYRTATTAGCCYRCTGCATTNAAACGGCACGGTCTGTTTGNNNATCGTCCGCTTGGTTANYNGCYCGAANCCCRTCCTCACGNGTCCCNNNRAGTGAYNNNGAGGCCGCRGATTYTCGCTGGAYACTTTCCAAAGCNYCCAGAGRATACTTCGTTNTCTTTTCATTTACGCTACCCAATTTCGTAATATNYCGGCANCGYARAGCTGGCRCCGCAATGRGTGTCTTGCAGAATGGCNCAAGYGTCGGCNGCGCGTCACTATCGAGCCCNNNNCNTGGCGGCNNNAGCTAGCCGACCCAYYYRATRRCTCGCGTCCCCACGAGGYAGGCGGGAYGAAYACGGAGACGCTNNGACGGNNTTTATCCACATTYCACGCGGGCCNGCCGTTNCGCAAGGRCGRTNCRGRCCCCCARNCCGGTATGGGGYGTAANTGGRGTGYTCGCNAATNCNNGAGGANNACYCRRYYGCNNNTCGGCGCATCGTGGYNAGGGCGCCTTGCTTCTGTATTAACATTGGCNACGTAGATGNNTGTNGAACTNYGCNGNRRGANRGAGCNAATTCGCGGTGGCGAGCTCTGCGACCACATTAGTTACTTAATGYGTCARAGAATNNTTGNNCGTGTCCGCCCCYGCGGCGCGAAGNRNCTNAAAAGCCCRCTACGCAAAAGCCTRTNRAGTAAGGCCCAATNYCNNNNNCRYGCAGCGNGGTCGCGATAGCGGGTTGCRRGCAATTCGAGGACTAGCACTACTNCNAGAAACGGCCGGTANNNGAAGCCCGCTGNTRNGTTACRRYTTGATTAGCCCGTCNNNNAGCAATCNTNCTCGTYGTNAGGGGCTACTTCGGTGRCRTNNNNCGCATTAGACCYAYGNNGCAGYAGYARCGTCTTACAATACTCTAGACGCCTACAGCATCTCGGGCTAATACTTCTCNNNNCTCCTGRTNACCGGTGANCACTAGGTGCCTGTTCCTGGATACCNNNNTCNNNNNTTCAAAGNTCRYCATCTTACAAGACYCCTGANANNRTGCGGYGCTTTCTCCATGTCAACTTTGNNNNNNNNNGACGAYRCRCGACCGCCGGTTTRNNNNNNNNNCCACCGRGYYTGCAGGGGAGGAGACGCGGNATCGTARCGYNNGTGTCGANNNGYNTCARYCGRNNNNNTRGGRCCGCGRNCNNRTGACCATATACCCCGCATNNNCGNNGGYCNCAAGYRRGGGTGTCTANAYCGATCCCRGRYCTGAGRATCGAAYAACTCCTTCTAGCTGGGTTACCCCCCGAYCYGYGCATAAAGTTAGCACGCTNNCAGTANTGGNACCCACCGNGNTTGCGARGATTANAGCANNNNCGTTGNYCCGNNNCAGCGGTATTAANGCCTCACGTCCTYTATRRGAGNCGGGGTGAAACATTARRGCACYGTCGGACCATCCGGCNCGCATCACGTGNNNAANAGTCNYTGGGTATNTGAGAGGAGTTCGRGYRCCANTGTCNNNNNYNCRRRYNTTCRRRYYRNCCAYNCAGCTCNRCAGACGTTGGCAGCTCACAAGCGNTRGCYYATCNNNNTAAAGGNNNNNCCAGRNGACRCGRAAGCCTCAAGGTRCAATCATTCNNGTRTTGNNNGGNNGARNAGAGTANNAYNNNNNNNNNTTCGCAGGANTRGTYCGTGCCGAAGGAGNYATCACCAATGAAAGTAACCGCGAGATGCGCTACNTGYCCTTTGTYAGTNTRCANTRCATAANNNANTCGTCGCTGCCTCNTNNNGACCGTACRCANAANNCYCCAGAACCNNANTCGCCGGCTACCCCGACGYYAGCCTCRCNCAGCCNGAGCTANTGTATCTAAATGGRCTGTCCTCATTGTTGCNGGCGGATCCCTYTCAATCTCACNGCTATAACNCACAGGCARTTTGAGAAATCNTACNCGCGGTAAACCGTAGGGCGAGGCYACNNNGCCTRTGGGGCCATCCTAYTGYRGATGAGTCNNNCARTYTCGGCCYRGATCGGGTTCATCRTRNRCGGANCGNTCCGCAGGNNGCCTGYCYGATCGGAACTACGNCGNNNNCCTNCGAYCNACGCGYCTGNRCCCNGGCTYYGTGGCCGTNNNGYANAACTGATCCNGGGACTTNGATGCCCTTGTAAGCACNNTGNAATAGRTTGTACCACGCCACACGRCGGNNTAGGNCGCCNNNGGYNCCTTGTCTTAGNGNTCAAGTTTCCTCGTGTTGCCCCNAAGCAATCATGCGTAANNGGATGNGGTYNNGGCARCTCGAATCAGRYGCGGTCTNNNNTCCCNAAAATTGCANTCANNNACGCGTACGTCGAATGCACCGCGGCATTGCTNNCGGRTRCTNGCGRNCRTCATNGTGTTCATGGTGTTGYGAGCTCTCNNNNRGNGCGGACYGAAANCGCCACGGTTATGCGTAGAAGGCGCRAATGTGTCCAAARCGTTARCACGGANCTTGAANGCGCGAATATTRCGGCYCAYTCCTATACAGGNCNAGGTANGGCTTCACANNNNCTGTNCCCYYGRGNNNCYGCCGNCNCGCAAANYCATGGNGGGGGAGCTGACACCTTGTTGNNGCGGGYTCTCGAGGTNACNRRAYNGTGGCGTCTCCCTAATCGAAAGNNAATTCTGTTACCTCAGGGGACGCCGGCATGGTGTCGCTNCACTCTTGATAYRTNTYNNANTGCAACGAAGCCAGGCGCCGTATAACAGAAAGATGACAAAACTARRYRCGCAGTACGGGACGACGTNGCCACCACGGRCCCTRATCGTGTNNCGNANCRACNNNACNTCGGTCTCTGGCTTTACCCGGGTGCAGACGTCTCAGTCAGCAGGGATTNCYRGCCGAATARGNCRAGTGTCGAGCCNACCCGAGTCTAGCGRGTCGYGNACNNCCCGAAGAGCGCCACACATNNNNNNNNNAYTCAGGGCGGARGCGCCNAAAYACTCAGTNCCCACCTGNGGTTARCCCTANNNNCTAAGGTAAGCCCNATGAGCNATTGGGANNCYGAATCCGGANNTYNGGTCYTATTCCTCGAACCGACCATANNNTACGTGAAGAATTTGTTANCTTAACCCGCTAGACCACGTGTYGGYACACAGCTGCCTCGGTNNARTGGNACTYCCAAGNTTTACCNCYNAGCGRCAGGAGTTAGTTGGACCRCCCTCAATNTGATACAAYNTNYAGCAAGCCGATGGAARRCACTCGCCAGCATTTGCATCCACATGCGTCCCTGTYTTTTYCATGNCTGGYCGRCGGAAYRGARGNTCTCGATCTGTGATGNNTAGCYGTCGCAGATGTAGCYGGCTRTNNTGTRGCGCGGACTGTGCCCAGATTRYYCTCCAAGTNGYARRTAGACCCTNACNTAGGGNGTNGNGANATAANCGNNATCGNCTCAGGTCCTARGAACGTGGGATCCTGATCGCTNGAYCCCACCTCTCAAGGCTACCGCAGCGNCTTCTCAGCGACTGGCTAGCTYGRACYYYANNTAACGRGGGNGTTNRRNRNCTCTRNNAGGGCANCCTTGGATNNTCCGTATGANYCTTANACCGCTTYGTGGCATRGGGGNAGAGNNCCTANGCNGRGNCCTRGNCCGTYCGCAAACNGGCCARYTNGRCCAGAGGCCGANAGGNGCRCAACGTAACATAGCTYCACGGAGNACACYGAGCTNTYNTAGCAGGAGCNNGTGTGCATACCCGTACCATTCGNNTGAGGCTTATCTGTCNNTAAAGTCATGGCGCCAACTGGTGAGTGCCCGGTACTTTGTATAAATGCCCTTTCCGGGAGCATTNGATCGTTCGGNNCCGAGNTNNGCYATNARYYAGGCATTGCCTTTGCACGGCNACGGCNNGGTGTCACGANNTCGGGTTAGTNYGTGGCAGACTTTTTAGATCCGACYCGAYTGCYCNAGATGNNNGCCCCAGCATCCTTNNGCTGGGATTTATTGTRAAGACGATCGGGCANAGCNCTACCTGCTGGCGRNGTCCRTTGATGACCGAATCAAGGGAYCAGATTTTGTANNTAGCATCCTACAGTNNNNNNNTTTCGCATTCAGTCNNNNNNNNNCTCGCANTGCACATT

Br46-1 GYGATTTACTCGTCANNNTANNGGCCGCCGGAGTCNNNNACCTAGRCGGNNCTAGGAGGTTTACCCACTGACTGTGATCTACNGGCCATNACGCYYNTTGTCTRRGGRGYCCACYNCCCGGGATCCRTCTTGGGTATGGGNNATRCTNCGCTTANNNTGGCCTGGNAGCGYNCAGRNGAGCCGAAAGTANNNNNAGGTNTGGACCTGGCTTNGNRANGRYAGATCTTACAATACGGCAGTCTNYAAGTACGTTTGGYACGNACNYCNTAGGYCTTGTAAGAAAATTTTTGRCGANYGYGGATCACTCGYNGGNCATTGACYATTCYCARTGNCCGTGGATAGTTYNNYTGATGGCTTAGAYTNAAGAACGGTGGAGCYATTCYGGAGCTGYTANRNTGRRGCGRGCGCCGCAGGYYNNGAAYCTCGGTGCRTTACANTGCCGCCAGCTRGYYYYNAARCGCCYTCTCTYTACTGCTGNRYGGNCTGCGTYNGGTTACCTTTGGCTRYNAATGAACCCTANCRGACACRGCNAANYATCCTCGTNTARACTCRNNTAAGAGTCTAGAGCCGACGCGTACGATTNCAACACCRACGGGGCGAGGANAGAACGYCTCCRRCTACCAACGGTGGACNTNCNCCTTARAAYAAGCGRYCGCNRAGNNTGAGCARGCCNRTCTCCTCNATCNGCTCCCTTATGTGCATACCCCTGYCGCCGCTTACGCCAGTACCTARCRNACTGCGCCGTGCGGTRRGTACCARGTGRNTCCTNTYNCATGCGAATGGNNGGCACTACCAAGGACTAGCNNNNNGNYNGCTAGGAACTGATNTTATGTGAGTACATGCCCCGCYCGCAGTACAGANACNRCTNGCCCTGTACACAACGGAATTGTTCTTAGGCTYGCGAATNYGRNTRAGTTCGGAAANNCGRNCGCCNTGRNCCGGTCAGTCACNAAAGTTGYRANTNGNNGTNACTNGCGRTAGCCATGGCTCNTAARTTCGRRNTNGCCCGCGGTRTARRGGCGRNCCGCRTCGGCGARGGCCGCTTTRNTRCGAGCGATAATCAGACGNTTAGATTATGTCAGRARRAANGCNCTCCTCTTRNAAAAGGTCNNACGCTGTGGCTCCACTTTYGRCGGCCGGGGNNTTAGGCNCGTCCGRGGGYCGNTAAGTACYAGGGCCNAGRAAYRTGAATGCCCAAACGATGGGGCCAGCCACACCGACAARTTCTCGCAGRGGGCRYCAGTTNGYTTGCTGCAGAGAATTTCTTTGGCCCGANRGTAACGCGTCAGCCCTTGAAAAGACANNGRYNTGNRNNCGGACGCCNGAAATGTCATTTAGGAATYNACTAGNACTNACGTANAAACRNCNNGCGCNTNGGTANCTCTCCGGGCGGCCRRCGCNNNYGNNRTTGGCTCGCCCCTGRNCGAAGGAAGGGAAAAACTTCGGGACCCTGAGTACTCNNCGACCCTAGTGAACATAGAGCATCGNCCAGTTTRACATNTGGTYNCTGGRNGCRGCYRTCCTCTGACTTACCCNGNGGCTTCYGCAGGAYTTTGTNNNNCCAGAAAGCGRRCCCYCYGCACCTCCCAAGYGGTTTTTCACATACCCTAYCYAGGTCNYTCCGAYGGAATTGCTNNATTAGTGYTRTCRTGAGCCCTATATCTRTTAACYGGGTGGGCACTGGTCTCGRRNTCGTAGTACTCGATATRAGGTCGGGGRTCACTATGTGCAGGTGGACYRTATTAGCCTACYTCATTGAGAYGGCAYGGTCTGTTTGGATATCGTCCNNTTGGTTANYNGCYCGAANCCCRTCCYCACGCRTCCCGAAGAGTRAYGGTGAGGCCGCGGATTCTCGCTGGACANYTTCYAAAGCGNCCAGAGRATACTTCGTTCTCTTTYCATTTACGCTACCCAATTTCGCAATGTGCCGGYRNCGTAGAGCTGGNRCCGCAATRRGTRTCTTGCAGAATGGCTCAAGYRTCGGCGNCNCNTCRYNANCATGCCCCTGGCATGGCGGCGACAGCTAGCCGNCCCACYCRATGGNTCGYGTCCCCACNAGGNAGGCGGGAYGAATACGGAGACGYTATGACGRGGTTYAYNCAAGCTCCACACGGGCCAGYCGTTNCGCAAGGRCGRTNCRGRCCCCCAGACCGGTATGGGGTGTAATTGGRGTGYTCGCRAATNNCTRNGRANTACYCRRYYGCTGATCGGCGCATCGTGGNNANGGCGCCTCGCTTCTRNATTAACATTGGCAACGTAGATGCATGCGGAACTNYGCAGTGGGARARAGCRARNNCNYGGYRRYGGGCTCYGYGACCACATTAGNNNCYTAATNYGTCARAGANTGATTGTTCGTGTCCNNCTCYGCGGCGCGAAGNNNCTNAAAGNNCCGCTACGCRAAANCCTGTCAAGTNRNGYYCAAYNTCCNGCGCACNNAGCGCGGTCGCGATAGCGGGNTGCGARYGATYCNRGGAYTNGCACTACNNCTAGARACGGCCGGNACRGGGAACCYGCTGNTNGGNTNCGRCNTRATTAGCYCGYYCCRGAGCAATCNTNCTCGTYGNCAGGGGCTACTYCGGTGACANACANCGCAYTAGACCCACGCATCAGCRGCAGCGTCTNACAANACTCTARACGCNCACANCGTCTCGGGNNAATACTTCTCCTCNCTCCYGATCACCGGTGGCCACTAGRYGCCTGTTCCTGGATANYCGAAACGGGYYTTCARAGNTCGCCATCTTACAAGACYCCCGATAGAATRCRGCGTATTACCCGCGTCAACTTTGAGCGTCGGTGACGAYRCRCGACCGCYGNTTTRTNANCCNCACCACCGGGTCTNCAGGRGAGGAGACGYGNTATCNTARCGYRNGTGTCGACCTGCNTYAGCCGAGRRAATGGAAYYGYAGNCACGYGACCNYNTACYCCGCATGGACGCTAGYCTYAAGNRRGGGTGTCTACAYCRATCCCGGGTCCTARARTCGAACATCTNNTTNNANCCRNNNTACCCCCCGATCCNYGCATAAAGTTANCACGCTTACAGTANTGGGACCCACCGGGTTTGCRNRGATTAAAGCAACCCCGTTGNTCCGTCNCAGCGGTATTAAAGCCTCACGTCCTTTATGGGAGACNNGGTGAAACATTNRRGYRCYGTCGGACCATCTGTCCCGCATCACGTGYGTAANAGTCNYTRGGTATGTGAGAGGAGTTCGRGYRYCAGYGTCTTGRCCTCGAGTTTTCGGACTAGCCACCCAGCTCAACAGACATTGGCAGCTCACAAGCGNTRGCYYNTNCCNCTAAAGGTTNNNCCAGGTRACGCRRAARNCTCAARGTRNAATCANYNTNGTATTGGTNGGACGARAAGAGTAANNNAATCCCTATTTCGCAGGATTAGTTCGNNCAGAAGGAGNYATCACCAATGAAAGTGTCCGCGAGNTGCGYTACTTGTCCTTTGTYAGTTTGCATTGNAYAATATAANCNNCGCTGCCTCRTCATGACCGTACGCAGNANNCYCCAGAACCTAAATCGCCRGCTACCCCGACGYYAGCCTCGCACAGCCAGAGCTACTGTATTTAAATCGACTRTYYYCNTTGTAGTTGGCGGRYCCCTTTTANTNTCGTCGCTANRNNNCACAGGCAGTNTGAGAAATCANACACGCGGTAAACCGTAGGGCTAGGCYACACAGCCTRAGGGGCCATCCTATTGTGGATGARTCGCGCARTYTCGNYCYARATCGGGTTCATCGTGRRCGGANCGGTCCGCAGGCGGCGYRYCTGATCGGAACYRCRTYGGGCCCCTGCNATCTACNCGTCCGCGCCCGGNYGCCNTGGNCGTTCCGTNTARTTGATNCNGGGRCTTRGATGCCNTTGTAAGCACNAYNGAATAGATTGTACCRCGCCACACGRCGGCANAGGACRCCAGCGGCACCTTGTCTTAGCGATCAAGTTTCCTCGTGTTGCCCCCGGGCAATCATGCGYNACRRRATGNGRTCATGGCAGCNCGAATCAGGYGCGGCCTCTGGYCCCRAAAAYYGCNGTCANNNAYGCGYACGTCGAATGCACCGYGGCGTTGCTTNCGGRTACYNGCGGCYRTCATAGCGTTCATGGTGTTGTGAGCTCTCCTTAGGCGCGGACYNAAATCACCACGRTYNTGCGTAGAARGCNYNAATRTGTCCAANGCGNTARCANGGANYYTGAAAGCGNGRNNATTACGGCCCATTCCTATACAGGTYNAGGTANGRCTTCACANTGGCTGTACCCYYGRGNNACYGYCGYYGCGCAAATTCACCGRRGGGGAGYYRACACYTTGTTAAAGYGGGCYCNCGAGGTAACGGGACAGTGGCGTCTCCYYAATCRAARNAGARTTNTGYTRCCNCRNAGGTCGCCGGCNTGGTGTCGCTTCACTCTTCATATGTNTYGCANTRCANCGAAGCCAGRCGYYRTATRAYANAAAGATGACAAAACTANNNNCGCAGTACGGGACGACGTNGCYACCNCGGGCCCTGATCRTGTTTYGNACYGNCACARCNNCNGTCTCTGNCTYTACCCGGGNGCAGACGTCTCAGTCAGCNGGGATTACYRGNCGNATAAGCCGNGTGYCGAGCCAACCCGARYCTAGCGRGTCGTGAACAACCCGAAGAGCGCCACACATCNATNTTTCCCTCAGGGCNGCRGCGCCNNNAYNCTCNGTTYCCACCTGAGGTYAGCCCTNNRAYCTAAGGTAAGCCCCAYRNGCGATTGGGAGGCYGAATYYNRATGTCCGATCTTATTCCTCGAACCGACCATAAAGTACGTGAAGAATTTGTTANCTTRACYCGCTNNNCCGCRTGTTGGYACACAGCTGCCTCRGTCTAATGGGGTYYCCAAGGTTTRCCNCTTAGCGRCAGGRRTTAGTTGGNCCRCCCTCRRTNTGAYAYAATCTACAGCAARCCGNTRRARGGCACTCNCCAGCATTTGCATCCACATRCGTCNCYRTCNNTTCCATGNCTGGTYGGCGGAAYRGARGNTCTCGATCTGTGANGNNTAGCYGTCACRGAGGTTACYGGCTRYTGYGTANCNCGGRYTGTGCCCAGATTGTCCTCCAAGNGGNNGATAGACCCTGACTTAGGGCATCGAGACATAATYGCAATCGCTTCAGGNCCTAGGAAYGTGGGATNCTGATCGCTGGACCCCACCTGTCANAGNNACNNNTACGNCTTCTCAGCGACTGGCTAGCTCGAACCCTAGATAACGAGGGCGTTCGATATCTCTAGANGGNCATCCTTGGRNTGTCCNNATGANNCTTAGACCGCTTTNYGGCATRGNGGCAGNNCCCCTRNGCNGRGNCCTAGGCCGTCCRCAAACNNGYCARYTNGACCRNAGGCCGACAGGNGCRCAACRYARCATRRCTYNACNAAGAACACTGAGCTATTTTAGCAGGAGCNRGNNTGCTARCCCNTACCAYNCGNNTGAGGCTTRTCTGTCRGTAAAGYCATGGCGCCAACTRGNGANYGCAYGGTACTTTGTRTAAATGCCCTTTCCGGNAGCATTNGANYGTNCGGACCCGAGNYANRYYATTARYYAGRYATTGCCYNYGYACGGCAACGGCNNGRTGTCGCGACCTCGNGTCGGTATGTGGYRGANTTTYTAGATCYGACTCGATTACCCAAGANNGAGGYCCCAGCATYCYYACRCTGGGATTTATTGTGAARRCGATNGGGCRAAGCRCTACCTGCTGGCGGAGTCCGTTGATGACCGGATCANNNGATCAGATTTTGTACCYRGTAGTCTACAGTGGCGAGTTATNNCATTCAGTCYNACGCGGYNTCGCANTGCACANN

Br46-2 GCGATTTACTCGTCAYYNTANNGGCCGCCGGAGTCGTCGACCTAGRCGGGTCTAGGAGGTTTACCCACNGACTGTGATCTACNGGCCATNACGCYYYNTGTCTRRGGRGCCCRCTNCYCRGGANCCGTCTTGGGTATGGGYYATRCTNCGCTTANNNTGGCYTGGCAGCGTACAGRNGAGCCGAAAGTANNRRNAGGTTTGGAYYTGGCTTAGGRACRGYRGATCTYAYAATACGGCAGTCTNYAAGTANGTTNGGCACGNACRYCNTAGGTCTYRTRAGRARRTTYTTGRCGANYGYGGATCACTCGYNGGNCATTGACTATTYYCAATGYCCGTGGATAGTTTGGTTRATGGCTTAGAYTNANGAACGRTGGAGCYATTCYGGAGYTGYTANRNTGRRGCGGGCNCCGCAGGYYNNGAANCTCGGTGCRTTACANTGCCGCCAGCTRGYYYNNAAGCGCCCTCYCTTTACTGCTGAATGGGCTGCRTYTGGTTACCTTTGGCTRYCAATGAAYCCTATCAGACACAGCTAAGTATCCTNGTTTAAACTCNNNTAAGAGTNTAGAGCCGACGNGTACNATTNCAACACCRACGGGGCGAGGANNGAACGTCTCCRRCTANNNACGGTGRACRTNCNCCTTANAANAAGCGRYCGCNGAGGCTGAGCANNCCNNTCTCCTCRATCYGCTCCCTTATGTGCATACCCCTGYCGCCGCTTACGCCAGTANCTAGCNNACTGCGCCGTGCGGTAAGTACCARGTGRNTCCTNTCNCRTGCGAATGGYNGGCACTACCRNRRNCTAGCCGRCTGNTAGCTAGGAACTGATNTTATGTGAGTACATGCCCCGYYCGCAGTACAGATACAGCTNGCCYTGTACACAACGGAATTGTTCTTAGGCTYGCGAATNYGRNTRAGTTCGGAAAAACGGCCGCCNTGRNCCGGTCAGTCACTAAAGTTGCAAATTGTNGTAACTNGCGRTAGCCRYGGCTCATAARTTCGRRNTNGCCCGCGGTGTAAAGGCGRNCCGCRTCGGCGARNGCCGCTTTANTNCRAGGGATAATCAGACGNTTAGATTATGTCNGAARGAANGCNCYCCTCTTACNNAAGGTCAYACGCTGTGGCTCCAYTTTYNRCGGCCGGGGAGTTAGGCNCGTCCGRGGGCCGNTRANTACYAGGGCCTAGGAATATGNATGCCCARANGATNGGGCCAGCCACACCGACAARTTCTCGCAGRGGGCRYCAGTTGGCTTGCTGCRRAGAATTTCTTTGGCCCGANRGTAACGCGTCAGCCCTTGAAAAGACATAGACGTGNGNNCNGACGCCNGAAATGTYATTTAGGAATYNACTAGYACTGACGTATANRYATCAGGCGYATCGGTANCTCTCCRGGYGRCCGACGCGAAYGAAATNGGCTCGCCCCTGRNCGAAGGAAGGGRRAAACYYYRRGACCCTGAGTACTCNNCGACCCTAGTGAANATAGAGCATCGNCCAGTTTRACATNTGGTYNCTGGRNGCRGCYRTCCTCTGACYYACCCAGTGGCTTCNGCAGGAYTTTGTACAGCCAGAAAGCGAACNCCCCGCACCTCCCAAGTGGTTTTTCACATACCCYAYCCANGTCNCTCCGAYGGAATTGCTNTRYTAGTGYTRTCRTGAGCYCTATATCTRTTAACYRGGTGGGCACTGGTCTCGRACTCGTAGTACTCGATATAAGGTCGGGGGTCACTATGTGCAGGTGGACYRTATTAGCCCGCYTYATTGAGAYGGCATGGNCTGTTTGGATATCGTCCGCTTGGTTATTGGCCCGAATCCCGTCCYCACGNRTCCCGAARAGNRACGGTGAGGCCGCGGATTCTCGCTGGACANYTTCYAAAGCGYCCAGAGRATACTTCGTTYTCTTTTCATTTACGCTACCCAATTTCGYAATRTGCCGGYNNCGTAGAGCTGGYRCCGCAATRRGTRTCTTGCAGAATGGCTCAAGYRNCRGCGNCGCRTCNCTANCRNGCNNCTGGCAYGGCGGCGACAGCTAGCCGTCCCACYCRATGGATCGTGTCCCCACNAGGYAGGCGGGATGAATACGGAGACGYTATGACGRGGTTNATCCAAGCTCCACACGGGCCNGYCGTTNCGCAAGGGCGGTTCGGGCCCCCAGGCCGGTATGGGGYGTAATTGGRGTGYTCGCRAATACCTRNGRARTACYCRRYYGCTGATCGGCGCATCGTGGYNATGGCNYCTCGCTTCTRNATTAACATTGRCAACGTAGATGCATGCGGAACTNYGCAGTGGGARARAGCAAGAYCRYGGYRNNGGGCTCYGYGACCACATTAGTTACYTAATNCGTCARAGANTGATTGTTCGTGTCCNYCYCCGCGGCGCGAAGCGNCYCAAAGGCCCGCTRNNNAAAACCCTGTCAAGTCGCGTTCAAYNTCCNGCGCACGCAGCGNNGTCGCGATAGCGGGTTGCGARCGANTCNGGGAYTNGCACTACNNCNAGAGACRGCCGGTACNGGGAACCYGCTGGTGGGYTNCRGYTTGATTAGCYCGYYCCGGAGCAATCGTCCTCGTCGTYAGGGGCTACTYCGGTGACAAACANCGCAYTAGACNCACGCAGCGGCAGCAGCGTCTNACAANACTCTARACGCNCACATCGTCTCGGNCTAATACTTCTCCTCACTCCTGATCACCGGTNNNCACTAGRYGCCTGTTCCTGGATANYCGAAACGGGCYTYCAAAGNTCGCCATCTTACAAGACNCCCGATAGAATNCGGCGTTTTATCCATGTCAACTTTGAGCGTCGGTGACGAYRCRCGANCGCYGNTTTRTRARCCRCACCACCGGGNNTTCAGGGGAGGAGACGNGNTATCTTAGCGTGTGTGTCGACCTGNNTCAGCCGAGNNAATGGRACYGCRGACACGYGRCCCCTTACYCCGCATGGNCGNTAGNCTYNAGYRAGGGTGTCYACRYCRATCCCGGGYCYNAAANTCGAACATYTCCTTNTANCYRAAATACCCCCCGATCCNYGCATAAAGTTACCACGCTTACAGTAATGGGACCCACCGGGNTTGCNNRGATTANAGCNACCCCGTTGNTCCGCCACAGCNGTATTAAANNCTCRCGTCCTYTATRRNAGACCGGGTGAAACATTNRRGYRCNGTCGGACCRTCYNNCCCGCRTCACGTGTGNAANAGTCCYTRGGTATGTGAGAGGAGNTCGAGCACCAGYGTCTTGRCCTCGAGTTTTCRRNNTAGCCAYNCAGCTCNRYAGACATTGGCAGCTCACNAGCGNTRGCYNATCCCACTAAAGGTTNNNCCAGRTGACGCRRAARNNTCAARGTRCAATCAACCTAGTGTTGGTAGGACGARAAGAGTAATATAAYYCCTATNTCGCAGGATTAGTTCGTGCCGRAGGAGTTATCACCANNGAAAGTGTCCGCGAGNTGCGCTACNTGTCCNTYGNYAGTTTGCANTNCATAANNNRATCRNCGCTGCCTCRTCATGACCGTACGCAGAANNCNCCAGAACCTAAATCGCCRGCTACCCCGACGNNNGCCTCGCNCAGCCNGAGCTAYTGTATYTAAATCGACTGTYCTCNTTGTAGTTGGCGGRYCCCTTTTATTCTCRYCGCTANRACACACAGGCAGTTTGAGAAATCATACACGCGGTAAACCGTAGGGCNAGGCTACACAGCCTRAGGGGCCATYCTATTGTGGATGARTCGCNCARNYTCRGCCNNGATCGGGTTCATCRTRAACGGANCGGTCCGCAGGCGGCGTGCCTGATCGGAACYRCNTNGGGCCCCTGCNATCTACGCNTCCGCGCCCGGGTGCCGTGGCCGTTCCGTGTTRYTGATNCTGGGRCTTRGATGCCNTTGTAAGCACNAYNGAATAGATTGTAYCRCGCCACACGRCGGCAGAGGACGCCAGCGGYNCCTTRYYTTAGCGATCAAGTTTCCTCGTGTTGCCCCCRRGCAATCATGCGCCANANRATGAGRTCATGGCRGCGCGAATCAGRYGCGGYCTCTGGTCCCAAAAATTGCAGTCATNNATGCGCACGTCGAATGCACCRYGGCRTTGCTTGCGGRTRCTNGCGRCYRTCATAGYGTTCATGGTGTTGTGAGCTCTCCTTAGGCGCGGACCGAAATCRNNNCGATCNTGCGTAGAAAGCCTGAATATGTCCRAARCGTTARCANGGANCYTGAAAGCGCRGNNATTACGGCYCAYTCCTATACAGGTCYNRGTAGRGCTTCACACTGGCTRTACCCYYGRGNNACCGTCGTTGCGCARNTTCACNGGGGNGGAGYYRACACYNNNTTRNNGNGGGCYCNCGAGGTNACNRRAYNGTGGCGTCTCCYYAATCGAAAGAGARTTNTGYTRCCNCRNAGGACGCCGNCNTGRTGTCGCTTCACYCYYNATATGTNTNGCANYRCANCGAAGCCAGGCGTTRTATRANANAAAGATGACAAAACTARRYRCGCAGTNNGGGACGACGTNGCYACCNCGNRCCCTGATCRTGTTTCGTACTGACACAACATCNGTNTCTGRNTTNACCCGGGTGCAGACGTCTCAGTCAGCNGGGATYACYRGCCGNRTAAGCCGAGTGYCGAGCCAACCCGAGTCTAGCGRGTCGYGNACNNCCCGAAGAGCGCCACACATCNATNTNTCNCTCAGGGCGGCAGCGCCAAAATNCTCNGTTYCCACCTGNGGTTAGCCCTRARATCTAAGGTAAGCCCCANNNGCNATTGGGAGGCYGAATCCCAATGTCCGATCYYRTTCCTCGAACNGACCATAAAGTACGTGAAGAATTTGTTARCTTGACCCGCTAGACCGCATGTTGGYNCACAGCTGCCTCRGTCTARTGNNRYTCCCAAGRTTTACCACCNAGCRACAGGNNTTAGTTGGACCGCCCNCRRTCTGATACAAYNTACAGCAARCCGTTGGARGGCACTCNCCAGCATTTGCATCCACATRCGTCCCTGTCTTTTYCATGNCTGGYYGGCGGAAYRGAAGATCTCGATCTGTGANGNNTAGCYGTCACNGANGTNRCYGGCTRTYRNGTANCRCGGRYTGTGCYCNGATTGTCCTCCAAGTGGTAGATAGACCCTNACTTAGGGCRTCRAGANAYAATYGCAATCGCYTCAGGNCCTAGGAANGTGGGATCCTGATCGCTGGACCCCACCTGTCANNNCTACCNCTACGACTTCTCARCGACTGGCTAGCTYGAACCCTAGATAACGAGGNNGTTNRRNRNCTCTAGAANNNCATCCTTGGATTGYCNGTATGATCCTTAGACCGCTTYNYGGCATGGRGGCAGAGCCCCTAGGCTGAGNCCTAGGCCGTCCRCAAACNGGCCARYTNRACCAGAGGCCGACAGGGGCGCAACRYARCATRRCNTCACGGAGAACACTNAGCYANTYTAGCAGNAGCCGGNGTGCNNGCCCGTACCAYNCGNNTGRRGCTTRTCTGTCRGTAAAGYCATGGCGCCAACTRGNGANYGCATGGTACTTTGTRTAAATGCCCTTTCCGGGAGCATTNGANYGTNCGGNYCCGAGCTATGCCATTARYYAGGYATTGCCYNYGYACGGCAACGGCYYGRTGTCRCGACCTCGRGTYRGTATGTGGCAGACTTTYTAGATCYGACTCGATTACCCAAGATGGAGNYCCCAGCATYCYYACRCTGGGATTTATTGTRAARRCGATNGRGCRAAGCRYNNCCTGCTGGCGGAGTCCGTYGRTGNCCRGATCANGNGATCAGATTTTGTACCCGNNNNTCYAYAGTNNNNNNTTNTCGCATTCNNNCTTACGAGGCCTCGCAGTGCRCRNN

Br47-1 GCGATTTACTCRTYGNNNNANNGGCCGCCGGAGTCGTCGACCTAGACGGGNCTAGGAGGTTTACCCNCTGANNGNNNTCTACTGGCTATNACGCCTTTTGTCTAAGGAGTCCRCTNCYCGGGRYCCATCTTGGGTATGGGYYATGCTNCGCTTANAATGGCYNGGCAGCGNRCAGNNGAGCCGAAAGTACAGACAGGTTTGGACCTGGNYYTGGAACGGTAGANNTYANNATACGGCAGTCTNTRNGTAGGTTGGGYACGNANACCCTAGGCCTCATGAGGAGGTTCTTGAGAANCGTGGATCACTCGTTGGTCATNNNCTATTCCCAATGYCCGTGGATAGNNNNNTTRATNGCTTAGAYTNANGNNNNNTGGANCCATTCTGGAGCTGCTANGGYGRNGCGGGCNNNGCAGGYYNNGAANCTNNGTRCRTTACANTGCYGCCAGCTRGCCTNNAAGCGCNCTCTCTTTACTGTTGAGCGGTCTGCRTYTGNTTACNTTYNGCTGYNAATNNAYCCTANCRGACACAGCTAATYNNNCTNGTYTAAACTAGTGTGAGAGTCAGGAACCGACGCGTATGNTTTCAACACNRACGGGGCRAGGANAGAACRCCTCCGGCTACCAACGGTGGNCRTTCCCCTNRAAACAAGYGACAGCTGAGGCNNNNCAGRCCNRTCTCCYCGATCYGATCCTCTGNGYGNAAGCCCCNNCCNCCGCTTAYGNNAGTANCTAGCNNACTNCGCCGTGCGGTAAGTRCCAGRTNGCTCCTATNNCATGNGAATRGTNGGCACTACCRNRRNCNRGCCGGCTGGTAGCTAGGAACTGATATTATGTGAGTRNATGCCCCGTCCGCAGTACAGATACAGCTAGCNCTGTACACAACGGAATTGTTCTTAGGCTYGCGAATNYGRNTRAGNTCGNAAAAACGGNCGCCNTGNNCCGGTCAGTCACTAANGTTGCAAATTGTAGTNACTNGCGATAGCCATGACTCNTAAGNNCGGGCTAACCCACGGTGTAAAGGCGGGCCGCRTCGGCGAGAGCCGCTTNRYCGCGAGGGNNNATTAGRCGATTAGATTATGTCAGAARGAANGCNCYCCTCTYAACAAAGGNNAYACGCTGTGGCTCCATTTTYGGAGGCCGGGGGATTAGGCACGTCCGGGGGCCGNNGACTACYNGGGYCTAGGAACAAGAATGCCCAAACGATGGGGCCAGCCACACCGACAARTTCTCGCAGGGGGCRYCAGNNGGCTTGCTGCRRGATATCGCAACGGCCCGANRGTAACGNGAYGGTATYYGAAAAGAYNTRCGCGTGNGATCRGACGCCNGAAATGTCGATTAGGAAYYNACTAGYACTGACRYRYAAANRNCNNGCGCNTCRRTATCTCNNNGGGCNRCCGACGCGAATGNARYGGGCTYGCCCCTGNNCRANGGAAGGGRRAAACCYYRRGACYCTGAGGACTCNNCGACCCTARTGAACATAGAGNATNGCNYNGTTTRRCATATGGTCTCTGGRNTCAACCGNCCTCTGACTTACNCAGTGGCTTNNNNNNNNCTTTGTACAGCCAGAAAGCGAACCYCCTGCACCTCCCAAGTGGTTTTTCACATACCCYACCCACGTCNCTCCGACGGAANTGCYCTNNYNGTGCTGTTGTGAGCYCTATATCTGNNNACCGGGTGGGCACTRNTTTCGGACTCGTAGTACNCGAYATAAGGTCGGGGNTCACTATGTGCARGTGGACCATANTAGTCTGCTTCATTNNNATGGCAYGGNCTGTTTGGATATCGTCCGCTTGGTTANYNGCTCGAAGCCCRTCCTCACGAGTCCCGAAGAGTGACGGTGAGGNCGNGGATTCTCGCTGGACAATTTCYAAANCGTCCAGAGRATACTTCGTTCTCTTTNCATTTACGNYANCCAATTTCGYAATRTNYCGGCANCGYARAGCTGGCRCCGCAATRGGTGTCTTGCAGAATGGCTCAAGCRTCGGCRGYGCRTCRCTANCANGCCCCTGGCACGGCGGCGACAGCTAGCCGTCYCAYYYRATNNNTCGYGTYCCCACGAGGCNGGCGGGACGAATACGGAGACGCTATGACGGGGTTTACANNNGTYYCAYRYGGTTCAGTCGTTACGCAAGGGCGGNGNRGRCCCCCAGRCCGGTATGGGGTGTAATTGGGGTGTTCGCRAATACCTRNGRANNNNTCNNNNGCNNNNCGGCGCATCGTGGYNANGGCGCCTCGCNTCTRNATTAACATTGRCAACGTAGANGCANGYGGAACTNTGCRGYAAGARAGAGCGAATYCRYGGCAACGGGCTCYGCGACNACATTAGTNACCTNNNNTGTCAAAGAATGATYGTTCGTRYCCGCCCCCGCGGCGCGAAGCGACTNAAAGGCCCRCTACGCAAAAGCNNGTCAAGTAAGGCCYAAYNTCCNGCGCACGCNGNGNGGTCGCGCCAGYCRGYTGCGAACGATTCGRGGAYTARCACNACTNCAAGANACNGCCGGTACRGGRARCCYGCTGGTNGGCTANGACTTNNTTAGCYCGCYCCGGAGCAATCGTCCTCGNCNTTAGNGNCTANNCTAGTGACATACATCGCATTANACNYACGCANCAGCGGCAGCGTYTTNNAANACTCYRRAYGCCYACATCGTCTCGGNCTAATACTTYTCCAAACTCCTGRTNACCGGTGACCACTAGNNGCCNNNTCCTGGATACTCGAAATGGGCCTCCAAAGCTCRYCATCTTACAAGACTCCYGACAGAATGCGGCGTNTTAYCCRYGTCAACNCTGNNNNNNGGTGRCGATGCACGACCGCCGGTTTATGAACCGCNCCACCGGGCTTNCAGGGGAGGAGACGYGNYATCTTAAYGTGTGTGTCGANCNGTNTCAGNNNNNNNNNTGGRACYRCRRNCACGYGACCCCTNACNCCGCATGGACGCYAGCNTNNNGTNRGGGTGTCCACGCCGATCCCRGRNCTGARRRTCGAAYATCTAGTTCNAGCYGRRNTNCCCCCCGAYCNGNGYNTAAAGTTACCACGCTTACNGTAATGGGACCCACCGGGTTTGCGARGATNAAAGCAACCCCGTTGNYCYRYCACANNNGTATTAAAGCCTCACGTCCTTTATGGGAGACNGGGTGNAACATTCAGNCACCGTCGGACCRTCCNGCNCGCRTCACGTGTGTAANAGTCNCTRGGTATGTGAGAGGAGTTCGRGCACCAGTGTYTTNRCCTCGAGTTTTCNNGTCGTCCATACAGCTNNRYAGACRNNGGCRRCTCACAAGCGNTRGCYNATCCCACTARAGGTTACTCCAGRNGNNNNGNAAGCNTCAARGTRYAATCAACCNNNNGTTGRTCRGNNGAGAAGAGTAATATAAYYCCTATNTCGCAGGAATGGTCCATGCCGRAGGAGACNTYACCAANGANAGTGTCCGCGAGATGCGCTACNTGNCCTTTGTCAGTCTACNNNRCATAATATAAACRNCGCTGCCTCRTNNNNACCRTACGCACAAGNYYYYAGAACNYAANTCGCCGGCTACCCCGACGCCAGCCTCGYNCRGCCNGAGCTANTGTATCTAAATNGACTGTCNNNNTTGTAGTTGNCNGRYCCCTTTTNNYCTCGTCGCTATAACACACAGRCAGNTNGAGAAATCNTACAYGCGGTAANCCGTAGGGCNAGGYTACACAGCCTGAGRGGCCAGYCTAYTGYRGATRGRTYNNNCAGTCTCGNCTTAAATCGGGTYCANNNTRARCGGACCGGTCCGCAGGCGGCCTGTCTGATCGGANYNNCGNCGGGCCCCTGCGATCTACGCGYCYGCGCCCNGGCNNNGTGNCCGTTCCGTGTAACTGNTCCNGGGACTTRGATNYCCTNGTANGYACCACTGAATAGRTTGTAYCACGCCACACGRCGGCATAGGACGCNAGCGGTTYCTTRYYTTAGNNGTCARGTTTCCTCNTGTTGCCCCNNNGCAATCATGCNNNNAAGGATGGGRTCATGGCRGYGCGAATCAGRTGCGGTCTCTGGTCCCNAAAATTGCAGTCATNNATGCGYACGTCGAATGCACCGCGGCATTGCTTACGGGTACTTGCGGCYATCATAGTGTTCATGGTGTTGYGAGCTCTCNNNNRGCGCGGACCRAARTCRCCACGATCNTGCGTAGAANGCNYRAATRTNTCCAANGCGTTANCACGGANNTTGANNGCGCRRATATTACGGCTCACTCCTATACAGGGYTAGGTACGGCTTCACACTGGCTGTACCCCTGGGNGACCGCCGTYGCGCANNTTCACGGGGGGGGAGYNNACACYTTGTTRNNGYGGGCYCNCRNGGTTACTAAATTGTGACATYGCCNNAATCGAARGAGAANNNTGNNRYCNCGGAGGNCNNCRGCATGRTGTCGCTTCACNCTTNATAYGTNTYGCANTGCAACGAAGCCAGGCGYYGTATAACAGAAAGATGACAAAACNAGATACGCRGNNNNGGACGACGTNGCCANCACGGACCCTGATCRTGTTTCGTANYRACACAACNTCGGTCTCTGANTTNANCCGGGTGCAGACGTCTCAGTCAGCNGGGATTNCTRGCCGNRTAGGACGNGTGYCGAGCNAACCCGAACCTAGCGNGTCRYNNACAACCCGAAGGGCACCACACAYCTATANNTNNCTCRGGGCGGNAGCGCCAAAAYNCNCNGTTYCCACCTGTGGTCAGCCCNGAAATCTAAGGTAAGCCCCATGAGCGATTGGGAGGNNNANTCCNRNYRTCCRGTCCTATTNCTCGAACCGACCATAAAGNACGYGRRGAATTNGTTARCTTRACCYGCTAGRCCGCATGTYGGYACACAGCTGCCTCRGTNYAATGGNACTTCYAAGGTTTRYCNCCAAGCGGCAGGRRTTAGTTGGNCCGYCCTCAATNTGATACAANNTACAGYAAGCCGNCAAANGGCNCTCGCNAGCATTTGCRTCYACATGCGTCNCTGNYACTNNNANGTNNNNNYGGCGGAAYRGARGNTCTNRRTCYGTGNNNRNTAGCNGTCRCNGANGTNRCNGGCTGTTGYGTGGYRCGRRYTGTGCCCAGATTGTYCTCCAAGTTGCAAATTGGCCTTGATTTGGGGCGTCGAGAYATAANCGYAATCGNCTCAGGNCYTAAGAACGTAAGATCCTGATCGCTCNATCCCACCTNTCANAGNNACCNCNNCGNNTYCNCNRCGACTGGCTAGCTYGAACCCTNGAYAACGNGGNYGTTNRRGGACTCTNGAAGGGCANYCTTGGATTGYCCRYRTGATCCTTAGACNGCTTNGTGGCATGGNGGCAGRGCCYCTAGGCTGAGNCNNAGGCCGTCCGCAAACNGGCCANNTNNNCCRNAGGCCGACAGRGGCGCAACRYARCNNNRCTTTACTAAGAACNNNNAGCYANTYTANCNGGAGCCGGTGTGYATRCCCGTACCATTCGCCTGAGGCTTRTCTGTCRGTRAAGTCATGGCGCCAACTGNNGAGTGCNYGGTACTTTGTRTAAATGCCCTTTCCGGGAGCATYNGANCGCTCGRTCCCGAGNTNNGCCATTANNYAGGCATTGCCTTTGCACGGCAACNNCYYGATGTCRCGANCNCGRGNYRGTATGTGGCAGACNTTYTAGATYCGRCYYGATTGCCCGRNANNGANNCNCCAGCATCCTTACRCTGGGAYTNACCGTAAAGACGATCGGGCANAGCACTNCCTGCTGGCGGAGTCCGTTGANGAYCGAANCNNGGGATCAGATTTNRNACCNNGCATNNTACAGTGGCGAGTNNNCGCATTCAGTCTTACNCGGYYTCGCAGTRCACATT

Br47-2 GCGATNNNCTCNTNNTCTTANNGGCCGCCGGAGTCNNNNACCTNNNNGGGNCTAGGRNGTTTACCCACNGAYYGYGATCTNNTGGCYATNACGCCTTTNNTCNNAGGAGTCCRCTNCYCNNGNYNCATCTTGGNNATGGGTTATGCTNCGCTTANAATGGCYNGGCAGCRCNNNGNNGAGCCGAAAGTANNRRNAGGTTTGGACCTGGCTTTGGAACGGTANANNNYACANNACGGCAGTCTNTRNGTAGGTTGGGNACNNNNACCCNNNNCCNCATGAGNNRRTTYTNRANNAACACGGATCNNTCGTTNNTCATTGANTATTNCCANTGNNNNNNNNNAGNNNGGTTNATRGCTTAGAYTNANGAACGRTGGAGCCNNNCTNGAGCTGNTANGNNNNNNCGGGCGYNGCAGGTTCGGNNNCNNRGTRCATTACANTGCYGCCAGCTRGCCTNNNNGNGCNCTCTCTNNNNNNNNNNNCGGTCTGCNNNTGNTTACCTNNNNNTNYNAATGAAYNNNANCNGACACAGCTAATCNNNCTNGNNNAAACTNRNGTRAGNNTNTAGANCCGACGCGTATGRTTTCAACACNRACGGGGCRAGGANAGANCNCNTCCGGCTACNAACNNNGGNNNNNCCCCTNRAAACAAGYGACAGCNNAGGCNNNNCARGCCTGTNNNNNCAATCNGNNNCYYNNNNNGCAAGCCCCCACCCCCGCTTAYGNNAGTANCTAGCRNNCTNCGCCGNNCGGTAAGTRCCAGNTNGCTCCTATYNCATGNGAATRGTNGGCACTACCRNRRNCNRGCCGGCTGGTAGCTAGGAACTGATATNNNGTGAGTRNANNNCCCNNCCGCANNACAGATACAGCTANCYCTGTACACAACGGNNNNNNYCNTNGGCTTGNNNATTTGACTGAGNTNGTANNAACGGNCNNCNTGNNNCGGTCAGTCACTAAAGTTGNNNNTTGTAGTAACTNGCGRTAGCCNNGACTCNTAAGNNNNGRNTARCCCRCGGNNNAAAGGCGGGCCGCNNCGGCGAGRRCCGNNTNGNCGCGAGGGNNNATTAGRNNNNNNNNNTATGTCNGAAAGNNCGCACCCCTCTNNACNNAGGTNNNNNNNNNTGGCTCCATNNNNGGAGGCCGNNNGATTAGGCANNNCCGGGGGCCGNNGANTACNNGGGYCTAGRAACAAGAATGNNNNAACGATGGGGCCAGCCACACCGACAARTTCNNNNNNGGGGCNNCAGNNGGCTTNNTGCRRNNNATYNCNNNNGCCCNNNNGTAACGCGAYGGTATYYGANNNNAYNTRNRCNTGNGATNNNNNNNNNGAAATGTCGATTAGGAANYNACTAGYACTGACRTACAAACRNCNNGCGCNNNNNNATCTCNNNGGGCNNCYGACGCGAATGNANNGGGCTNGCCCCTNRNCGANGGAAGGGAAAAACCYYNNGACYCTGAGNACTCNNCGACCCTARTGAACATAGAGCANCGNCCNGTTTRNNATATGGTCTCTGNRNTCAACCGNCCTCTGACNTACYCAGTGGCTTCCGCANNNCNNNNNACAGNNNNNAAGCGAACCYCCYRCACCTCCCAAGTGGTTTNTCACATNNNCYNCCCACRTNNCTNCNACGGAANTGCNCTRYYNGTGCTGTTGTGAGCNCTNNNNNNNNNNACCGGGTGGGCACTRNTNTCGGACTNNTAGTNNTCGNNNTNNGGTCGGGGATCACTATNNNCARNTGGACCATATTAGTCTGCTTCATTNAGATGGCANGGNCTGTTTGGATATCGTCCGCTTGGTTANNNGCCCGAAGCCCRTCCTCACGAGTCNNNNNGAGTGACGGTNAGGCCGNGGATNCTCGCTGGACAATTTCCAAAGCGTCCAGAGRNNACTNNGTTCTCTTTYCATTTACGCYANCCAATNNNNYAATRTGCCGGYRNNNNNRAGCTGGCRNNNNNNNNGGNNTCTTGCAGAATGGCTCAAGCRTCGGYGGCGCGTCACTANNNAGCCCYTGGCANGGCNNNNNNNNNNNNNNGTCYCAYYYRATRRNTCGYGTCCCCACGAGGCAGNNNNNNNNAAYACGGAGNNGNNATGACGGGGTTTACACANGNNNNNNNNGGTTCAGTCGTTACGCAAGGNNGGNGNNGNCNNNNNNNNNNNNNNNNNGTGTANNNNNGGTGNNNGCGAATACCTRNGGANNACYCRRYYGCTAATCGGCGCATCGTGGYNANNNCGCNNCGCTTCTRNATTAACANTGRCAACGTAGANGNNTGYGGAACTNTGNNNNAAGANNNNGCGAATNCNNGGCAACGGGCTCNGCGACNACATTAGTNACTTNNTGTGTCAAAGAATGATTGTTCGTRYCCNYNNCCGCGGCGNNAAGNNACTCNNNNGCCCRCTACGCAAAAGCNNNTNANNNAAGGCCNNNYNTCCNGCGCACNNRGNGNGGTCGNNCCAGNCRGTTNNRRRCRATTCGAGGNCTAACACAACTNNNNNNNACNGCCGGTNCANGRANCCNGCTGGTGGGCTNNGRCTNAATTAGCNCGCYCCGGNGNAATCNNNNNYGNCRTTAGNGNCNNCTTCGGTGRCRTNCATCGCNTTANACNYACGCANCAGCGGCAGCGTYTTRCAANNNTCNRRANNNNNACANNNNCTCGGNCTAATACTTYTCCAAACNNCNNNTNACCGGTGACCACTAGRYNNNNNNTCCTGGATACTCGAAATGGGCCTCCAAAGCTCGCCATCTTACAAGACNNNNGACANNNTGCGGNGTNTTAYNCRYGTNANNTYTGAGCGTCGGTGRCGATGCACNACCGCCGGTTTATGAACCGCNNNACCGGGNNTNCAGGRGARGAGACGYGNTATCNNNAYGYRNGTNNCNAYCNGTANNAGNCGNNNNAANNNNNCYRCRNNNNNGYGACCCCTNACTCCGCATGGACRCYAGCYTNNAGYGNGGGTGNCCACGCNNATNCCNGRYCTGANNNTCGRRNANCTNNNTCNAGCYGNNNTNCCCCCCGNCCTGCGTTTAANNNNACCACGCNNNCCGTANNGGGACNNACCGGGTTTNNNNNNATTAAAGCAACNNCGNTNNYCYRNNNCAGCGGTATTANNGCCTCACGTCCTTTATGGNAGACNGGGTGNAACATTCAGNCANCGTCGGACCRTCCNGCNCGCRTCACGTGTGTANNNGTCNCTRGGTATGTGNNAGGAGTTCGAGYRCCAGTGTCTTGNCCTCRRRTTTTCGGRYNNNCNNNNCAGCTNNNYAGANANNGGNNNCTCACAAGNNNNNNNNYATCCCACTANNNNNNACTCCAGRNGNNNNGRAAGCNNNAANGTNYAATCANNNNNNNNTTGRTCNNACGANAAGAGTAANNNNNNNCCTATNTCGCAGGNNTGGNNNNNNCCGNAGGAGNYNNNACCAANGANAGTGTCCGCGAGATGCGCTACNTNNCCTTNGNNAGTCTACACTNCATAATATAAACNNCGCTGCCTCRTCATNACCRTANGCACAANNCYCCAGAACNTNNNTCGCCGGCTACCCCGANNCCNGCCTCGYNCRNNNNGAGCTATTNNNNCTAARTNGACTGTYCTCNTTGTAGTTGNCNGRYCCCNNNYNNNCNCGTCGCNANNAYNCACAGRNNNNTTGAGAAATCNTACANGNNNNAANNNNNNNNNCNAGGYTACACAGCCNNAGRNNNNNNNCTAYTGYRGANNGGTYNNNCAGTCTCGNCTTAAATCGGGTCCAYNGTGNGCGGANCGGTCCGCNGGCGGCCNNTCTGATCGGAACTACGTCGGGCCCCTGCGATCNANGCGYCYGCGCCCNGGCNYYGTGGYCGTTCNNTRTAACTGNTCCNGGGACTNAGATGCCCTNNNNAGCACCNCTGAATAGRNNNNANCACGCCNNACGRCGGNNTAGNNNGCCAGCGGTTNCTTRYYTTAGNNGTCAAGTTTCCTCNTGTNNCCCNNNNNNNNTCATGNNNNNAAGGATGGGNTCATGGCAGNGNNAATCAGRTNNNNTCTNNNNTCCCNNNAAYTGCANTCATNNATGCGYACGTCGAATGCACCGNNNNNTTGCTNNCGGRTACTTGCGGCYATCATNGTGTTCNNGGTGTTATGNGCTCTCCTTANGCGCNNNNNRAANTCRCCACGNNCATGCGTAGAARGCNYRAATRTGTCCAANGCGTNAGCNNNNNNCTTGAANGCNCNNNNATNACGGCNNNNNNNTNNACAGGGYTAGGTACGNNNTCACNNNNNNTGTANNNCTGNNNNNNNNNCGNNGCGCARNNNNACNGNNGGGGANNNNACACYTTGTNAAAGYNNGCYCNCNCNGTTACTAAATTGTGACATYGCCNNAATCNAAGNNNNNTNCTGCTACCNCNNNNGNNGNNNGCATGRTGTCGCTTCACYNNNNATAYGTNTYGCANNRCAACGAAGCCAGGCNNNGTATAACAGAAAGATGACAAAACNAGATACGCRGNNNNGGACGANNNNGCNNNCACGGACCCTNNTCNTGTTTCGNNNCAACACANCNTNGGTCTCTGANTTNANCCGNNNNNAGACGTCTNAGTCAGCNGGGATTNCTRGNCGNNTAGGNCGNNNNNCGARNNAACCCGAACCNAGCGAGTCNNNNACNNNNCNNNGGGCACCACNNANCNATNNNNNNCTCGGGGCGGNAGCNNNNNNACACNCNGTTCCCACCTGTNNNNAGCCCNNAAATYTAAGGTAAGCCCNATGAGNNNNNGGNAGGNNNNNNCCNNNNNNNNNGTCCNATTNNNYRAACCGACNRTAAAGNACGTGNNNNATTTGTTARCNTGNNNTGCTAGGCCRCRTGTYGGYACACANNNGCCTCNGTCTAATGGGACTTCYAAGGTTTRYCNCCAAGCRNNAGGNNTTANTTGGNCCNNCCTCAATNTGATACAANNTACANNNNNNNGNYAAARGGCNNNNNCYAGCATTTNNNNCYACATGCGTCNCTGNCACTTCCATGTCTRRTNGGCGGAANNGANGNTCNNNNNNNNNGATGRNTAGNNGTCNCNGATGTAGCCGGCTGTTGTNTNNCACGNNNTGTGCCCAGATTGTYCTCCAAGTTGCAAATNGGCCTNGATTTGGGGCGTNGAGAYATAANCGYANTCGNCTCANGTCYTANGAACGTNNGATNCTNNTCGCTCRATCCCACCTNTCAAAGCTACCGCNACGNNNNNNCNRNNNNTGGCTANNTTGAACCCTAGATAACGAGGNYGTTNRRNNNCTCTGTCAGGNCANYNNNNNNNTNNNCRYNNNNTCCTTAGACCGNNNCGTNGCATGGRNNNAGRGCCYCTANNNTGANNNNNAGGCCGTCCGCNAACNGGCCARYTNNNNCNNAGGNNNACAGAGGCGCAACNNNNNNNRRCNNNNNNNNGAACACTNAGCYANTNTARCRGGAGCCGGTNNNNATNCCCNTACCATTCGCYTGAGGCTTRTNNGTCRGTNAAGTCATGGCGCCAACTNRNNAGNNCNYGGTANNYTNTRTAAATGCCNNNNNNNNNNNCNNYGGANCGCTCNNNCCCGAGNTNNGCNNNNNNNNAGNNNNNNNCTTTGCACGGCAACGGCYYGATGTCGCGANNNCGRGACGGTNNNTGNNNGNNNNTTTAGATYCGACNNNATTGCCCGRNANNGARNCNCCAGCATCCTNACNNNNGGAYTNACCGTAAAGACGATCGGGCANAGNACTNCCTNNNNGCNNNNTCCGTTRRTGATCGNANCRAGNGANCNNATTTNRNACCNNNNNNNYTACAGTGGCGAGTNNNCGNATTCNNNCNNACNCGGNNTCGCANTGCACATT

Br48-1 GYGATTTACTCGTCACCATAATGGNCGCCGGAGTCGTCGACCTNNNNGGGTCTANGRGGTTTACCCACNNNYYGYRATCTACTGGCNATCACGCCTYTTGTCTRRGGRGYCCACTGCYCGGGANCCATCTTGGGTAYGNNCCATACTGCGCTTACAATNGCCNNNCAGCRTGYRGGAGAGCCGAAAGTAACARNAGGTTTGGACCTGGCTTNGGGACNGYRGANNNTRCAATACRGYAGTCTNYAAGTANGTTNGGNACGNAGANCCTAGGYCTYRTRAGRAGGTTYTTGACGANYGYGGATCRYYCRCAGGNCATTGACYATTCCCAATGYCCGTGGATAGYYNGGTTGATNNYNTAGNTTGRAGAACGGTGGAGCCATTCTGGAGYTGCTAGRTTGGGGCGGGCGCCGCAGGYYNNGAACNTARGTRCRTTACANTGCYGCCAGCTAGCCTYNAARCRCYCTCTCTYTACTGNTGNGNGGNCTGCATTTGGTTRCCTTYNGCTRCNAAYGARCCCTANCNGACACAGCTAAGTATCCTNGTCTAGACTNGTNTRAGAGTCNNGAGCCGACNCRTAYGGTTTCAACACCRACGGGGCGNNNAAAGARCRCYTCCGGCTACCAACGGTGGNCRTTCCCCTNRAAACAAGTGACNGCTGAGGCTGAGYANGCCAATCTCYTCGATCCNCTCCCTTATGTGCANRCCCCTGCCGCCGCTTACRAGAGTACCTRRYATACTGNGYCGTGCGNNGGGTACCAGGTGAATCCTNTCNCRTGNGAATRGTNGGCACTACCRNRRNCTGNNCGGYTGGTAGCTAGGAACTGANNTTATGTGAGTACATGCCCCGYYCGCAGTACAGATACAGCTANCNCTGTACACAACGGRAYYGTYCNTNGGCTTGCGAATNYGRNTRAGTTNGGAAAAACGRGCGTCNTGAGCCGGTCAGTCACTAAAGTTGCAAATTGTNGTAACTCGCGGYNGCCATGGCTCATANATTNNGAATAGCCCGCGGTGTARRGNCGGGCCGCRTCGGCGAGRGCCGCNYTACCGCRAGGGATAATYAGACRATTNGATTATGTCNGAARGAACGCACNCYTCTTRACRNAGGTNAYACGCTGTGGCTCCAYYTTNNGCGANCGAGGNNNTAGGCACGTCCGGGGGTCGCTAAGTACTAGGGCCTAGGAACAAGAATGCCCAGAAGATGGGGCCAGCCACACCGRYAARTTCNNNCAGRGGGCNNNAGTTGGCTTGCTGTNNRGNATTTCTNTGGCCCGACGGTAACGCGNCRGYCCYYGAAAAGACATAGRYGTGAGATCNNRCNCCAGAAATGTCRNTTAGNAATNNACTAGCACTGACGTATAARYNNNNNGCGCATCGGTATCTCNNNRGGYGRCYGACGCGAATGAAATNGGCTCGCCCCTNGACGAAGGAAGGGRRAAACCTCGGGACYCTGAGNACTCNNCGACCCTARYGAACATAGAGCATCGCCCAGTTTRANATNTGGTYNCTGGATGCGGCNNTCCTCTGACCNACCCAGTGGCTTCCGCNGGACTTTGTRCRRCCAGAAAGCGAACCYCCCGCNCCTCCCAAGTGGTTTTTCAAGTACCCTNCCTANGTCNCTCCGACGGAANTGCYCTGCCTGTGCTGTTGTGAGCCCTATATCTGTTAACYRGGTGGGCACTGGTCNCGGRNTCGTAGTGCTCGATATAAGGTCGGGGRTCACTATGTGCAGGTGGACYRNATTAGYCTRCTTCATTGNNANGNNATGGTCTGTTTGGATATCGTCCNNTTGGTTAACNGCYCGAAGCCNNTCCTCACGAGTCCCGAAGAGTGACRGTGRGNCCGNGGNTTCTCGCTGGACACTTTCCAAAGCGTCCAGAGGATACTTCGTTYTCTTTYCATTTACTCTAGCCAATTTCGCAATGTGCCGGYRNCGCARAGCTGGCRCCGCANTRRGTGTCTTGCAGAATGGCYCAAGCRNCRGCGGCGCGTCACTATCNAGCCCCTGGCACGGCGGCGACAGCGGGCCGNCYCAYYYRATNNANCGYGTCCCCANGAGGYAGGCGGGANGAAYACGGAGACGCTATGACGGGGTTTRYACACRCTCCACACGRTTCNGCCGTTRCGCAAGGGCGGTTCGGGCYCCYAGRCCGGTATGGGGYGTAATTGGRGTGYTCGCRAATACCTRNGGANNACCCAGTTGCNNNTCGGCGCATCGTGGYNANGGCGCCTYGCTTCTGTATTAACRTTGGCAACGTAGANGCATGYNGAACTNTGCAGYGGGARAGAGCRAATTCGCGGYRRYGGGCYCYGCGACCACATTAGTTACTTNNTGTGTCAAAGATTGAYCGTTCGTGTCCNYCCCNGCGGCGCGAAGNRNCYCAAANGCCCRCTRNNNAAAANCCTRTCRNNNAAGGCCCAATCTCCTGCGCACGCGAAGCNGTCGCGNYAGCNGGTTGCGAGCGAGTCCRGGACTAGCACTACTGCTAGAAACGGCCGGTNYARGGAACCYGCTGGTANGNTNCGACTTGATTAGCYCGYYCCGGAGNAATCGTCCTCGTYGTTAGGGGNYANNYYRGYGRCATACATCGCACTATACCYACGCANCAGCGGCAGCGTYTTRCAANACTCNRAACGCCYACAGCGTCTCGGNCTAATACTTYTCCNNNCTCCYRRTCACCGGTNNNCACTAGGYGYCTGTTCCTGGATACYYGAANNGGGCYTYCNAAGCTCRYCRTCTTACAAGACNCCCGAYAGNRTGCGGCGYTTTNTCCATGTCAACTYTGAGCGTCGGTGACGNYGCACNACCGCCGGTTTATGAACCGCACCACCGGGCTTNCNNGGGAGGAGACGCGGTATCGTAACGCAGGTGTCGACCTGCNNNAGYCGRGGAAATGGGAYYRYAGNCACGTGACCNYNTACYGCRCATRGACGCYGGCCTYAAGYRAGGGTGNCTANANCAATCCCGGGYCTGAGGATCGAATATCTAGTTCTAGCYGNRNTACCCCCCGAYCYGYGCATAAAGTTACCACGCTTACNGTAATGGGACYYACCGGGNTTGCGARGATTAAAGCNACYCCGNTGNNCYRTCACAGCGGTATTAANGCCTCACGTCCATTRNRRNAGACGGGGTGAAACATTNAGGCACCGTCGGACCATCYGNCNCGCATCACGTGTGTAATAGTCNTTAGGTATGTNNNAGGAGTTCGGGCACCANTGTCNNGRCCTCGAGTTTTCGGACTAGCCAYNCAGYTCCRYAGACATTGGCNGCTCAGAAGCGGNRGYYTANCCCNCTARANGYNANTCCAGRNGACGCGGAAGCNTCAAAGTGCAATCAACCTAGTATTGGTNGGACGANAAGRGTAATRTAATCCCTATTTCGCAGGNNTGGTCCGTGCCGRAGGAGNNATCACCAAGGNTAGTAANCGCGAGNTGCGCTACNTGYCCTNTGTYAGTCTACAYTGCATAATATAATCAACGCTGCCTCRTCCCGACCGTACRCANAANNCYCCAGAACNTAAATCGCCGGCTACCCCGANGCCAGCCTCGYNCRGCCNGAGCTAYTGTATCTAARTNNACTGTYCTCATTGTAGTTGRCRGGCCCCTTTTANTGNYRYNGCTATAACACACAGACAGTTTGAGAAATCATANNNNCGGTAAACCGTAGGGCGAGGCYACACAGCCTANGRGGCCANCCTAYTGYRGANGRGTTNNGNAGNCTCGNYYTAAATCGGGTTCATCRTRRGCGGACCGNTCCGCAGGCGGCCTGCCTGATYGGANYCGCGNCGGGNNCCTRCGAYYTACGCGYCTGNRCCCGGGCNCCGTGGCCGTTCCGTRTNACTGATCCNGGGACTTAGATGCCCTTGTAAGCACNANNGAATAGRTTGTAYCACGCCACACGGCGGCATAGGACGCCAGCGGYNNCTTGTCTNAGAGGTCAAGTTTTCTCGTGTTGCCCCCAAGCAATCATGCGTAANNGGATGNGGTYATGGCANYNCGAATCAGRTGCGGTCTCTGNTCCCAAAAATTGCACTYATNNATGCGCACGTCGAATGCNCCGCGGCATTGCTNGCGGGTRCTNGCGRCYRNCATAGTGTTCATGGTGTTATGAGCTYTCCTTANGCGCGGACCGAAANCGCCACGANCATGCGTAGAARGCNYGAATNTGTCCAAARCGNTAGCACGGAACTTGAANGCGYGAATATTRCGGCTCACTYCTATACAGGNYYAGGTANGGCTTCACACTGGCTGTACCCTCGGGGGTCCNCCGTYGCGCAAATTCACNGRRGGNGAGCNNNCACYNNNTTGNNGCGGGCTCNCGAGGTTACGGAATTGTRNCNTCTCCCTAAYCGAAAGAGAATTNTGNTGTCTCGGAGGACGCCGGCATGGTGTCGCTNCACNCTTGATAYGTNTCNNNNYAYANCGAAGCCAGNCGYYGTATANNNGNNAGATGACNAAACNANNNNCNCAGTACGNNNYGACGTNGCCANCNNNGACCCTGATCGTGTTTCGNRACAACACAACNTCAGTTTCTGRNTTNACCCGGGNGCAGACNNCTYAGTCAGCAGGGATTACTAGCCGTRTAAGCCRAGTGYCGAGCCAACCCGAGTCTAGCGGGTCGYGNACNNCCCGRAGGGCACCACACAYCTATATNTCACTCAGGGCGGNRGCGCYNAAAYANTCAGTTCCCACCTGAGGTYAGCCCTGNRAYCTAAGNTAAGCCCCATGAGCGATTGGGAGGCYGRATCCGGNTGTCCGRTCCTATTCNNCGAACCGACCATAAAGTACGTGAAGAATTTGTTANCTTRACYCGCTRGACCRCGTGTYGGYNCACAGCTGCCTCRGTCTAATGGCACTTCCAAGGTTTRCCTCTAAGCGRCAGGAGTTAGTTGGNCCACCCTCAATATNATACAACATACAGTAAGCCGNYRRARAACACTYGCCAGCATTTGCATCCACATGCGTCCCTGTCTTTTTCATGNCTGGCCGGCGGAAYRGAAGATCTCGATCTGTGATGRNTAGCYGTCACAGATGTAGCNGGCTGTCAYRTRGCGCGGRYTGTGCCCANNTTACCCTCCAAGTGGTAGATAGACCCTGACTTAGGGNGTCRRRAYACAATCGCAATCGCCTCAANTCYTNNGAAYGTNNGNNCCTGATCGCTCRATCCCACCTNTCAAAGCTACCGCTRCGTCTTCGCTACGACTGGCTAGCTTGAACCCTAGATAACGAGGRTGTTAAGGGACTCTAGARGGGCANCCTTGGATTGYNCGTATGATCCTTAGACCGNYTTGTGGCAGGGGGGCAGAGCCCCNRGGCTGAGNCCNAGGCCGTYCGCAAACNGGCNARYTGGGCCAGAGGCCGACAGGGGCGCAACRYAGCATRRCTYTACTAAGAACACTNAGCYANTYTAGCAGRAGCCGGTNTGYATNCCCTTACCAYNYRCCTGAGGCTTATCTGTCRGTANRRCCATGGCGCCAACTRRAGANNGCNTGGTANNYTNTAYAAATGCCCTTTCCGGGAGCATTNGATCGCTCGGTYCCGAGNTNNGCCATTAACYAGGTATTGCCCGCGTACGGCANCGGNCCGGTGTCGCGACCTCGGGNYRGTATGTGGCANACYTTTTAGATCYGACTCGATTRCCCAAGATGGAGNCNNNAGYATCCTTACRCTGGGATTTATYGTAAAGACGATCGGGYANAGCACTACCTACAGGCGRNGTCYGTTAGTGATCGAANCRAGNGAYCAGNTTTTGTANNYRNNNNYCTACAGTGGCGAGTTATNNCATTCNNNCTTNCNCRGCCTCGCAGTGCACATT

Br53-1 GYGATTTANTNRYCGYCNTAATGGCCGCCGGAGTCGTCGACCTAGACGGNNCCTGGRGGNNTACCCACTGATCGCGATCTACTGGNTATCNCGCCTTTTGTCNNARGRNYYCACTGCCCGGGATCCRTCTTGGGTNNAGNCTATGTTGTGCCGACAATGGCYNGGCAGCGCGCAGGAGAGCCGAAAGTRNNGRCAGGTTTGGACCNGGCCCAGGRACGRTAGAYYNYRYAATACGGCAGTNTGTAAGTANGTTNGGTACGCACATCCTAGGYCTYRTRAGRAGGTTCTTGRNNANNGNGGATCACTCGCAGGNCATTGACTATNCYCAGTGCCCGTGGATAGCCCGGTTGATGGTGTAGCTTGANNAGCAGTGGAGCCANTCTGGAGYTGCTAGRNYGGGGCGRGCGCCGNNNNTNNGGAACCTARGTAYATTACANTGYCGCCAGCTAGCCTYNAARCRCCNTCTCTCTACTGTTGAGTGGGCTGCATTTGNTTACCTTTGGCTRYCAACGAGCCYTAGCGCACACRGCNAATCATYCTNGTCTARACTNGTNTRAGAGTCNNGARCCGACNCATACGATTNYAACACCRACGGGGCRAGGANNGARCRCYTCCGGCTANNNACRGTGGACNTTCCCCTARAAACAAGCGACAGYTRAGGCTGAGCANGCCAATCTCNTCRATCNGNTCCTCTGNNYGCAAGCCCCCACCCCCGCTTATRNCAGTACCTAGCNNATTGCNCCGTGCGGTAAGTACCAAGTGGCTCCTATTGYATGCGAATGGTNGGCACTACCAAGGACTAGCCGGCTGGCCGCTAGGANCTRRTCTTATGTGAATGCGGACCCCGCYCGNAGTACAGATACARCTARCCCTGTACACAACGGNANNGTYCNTNGGCTYGCGAATNYGRNTRAGTTCGGAAAAAAGGGAGTTGCGGTCCAGCCTGCCACNAAAGTTGYRANTNGNNGTANCTNGCGGTATCCATGGCTCNTAAGTTCGGGCTARCCCRCGGTGTARRGNCGANCCGCRTCGGCGARRGCCGCTTTGCTGCAAGGGATAATCAGACGNTTAGATTATGTCNGRAARAACGCACYCCTCTTRACAATGGTCACACATGGTGGCTACCCTTTCNGNGGCCGGGGAGTTAGGCATGATCGGGGGYCGCTAAGTACNAGGGTCNAGRAANNTGAATGCCCARANGATGGGGCCAGCCACACCGACRANTTCYCGCRGGGGRCRYCAGTTNGNTTGCTGYAGGGTATTTCTNTGGCCCGACGGTAACGCGACGGTATCCNRAAAGACATACGCGCGTRATCAGACGCCNGAAATGTCATTTAGGAATYNACTAGYACTNACGNGNACGTRNCNNGCGCNTNGGTATCTCTCCGGGCGGCTRRCGYNNNTGTTGTGGGCTYGCCCCTGRNCRAAGGAANGGRRAAATCCCGGGACYCTGAGGACTCNNCGACCCTAATGNNCATAGAGCATCGNCCAGTTTRRCATATGGTCTCTGGGAGCAACCGTYCYCTGACYTACCCAGTGGCTTCCGCAGGACACTACACAGTCACTAAGYGAACCTCCCGCACCTCCCAAGYGGTTTTTCAAGTANCCTACCYAGGTCGYTCCGAYGGAATTGCTCYGCTAGTNYNRTCATAAGCCCTATATCNGTNAACCNGGTGGRNACTRNTYTCGANNNTGTAGTACTCGATATRAGGTCGGGGRTCACTATGTGCAGGTGGACYGTATTAGTCTGCTNCATTGARAYGGCATAATNTGNNTGGATATNNNNCGCTTGGTTANNGGCYCGAANCCCGTCCYCNCNCRTCCCRRTGAGTGNCGGTGAGGCCGCRGATYCTNGCTGGACANYTTNCARAGCGTCCAGAGNATNNNTCGNTCTCTTTTCATTNNNNNNNNNNAATTTCGCAATGTNYCGGCANCGYARAGCTGGCRCNNCAATGRGTGTCYTGNARAAYGGCTCAAGYGTCGGCGGCGCGTCAYNATCRNNCCCCTGGCATGGCNGCGACAGCTAGCCGNCCCACCCAATGGAGCNNNNCCCCACGAGGYATGCGGGAYGAANATAGAGACGTTATGACGGGGTTTATCCACAYTCCACGCAGNNCAGCCGTTACGCAAGGRCGNTNCGGGCCCYCARRCCGGTATAGGNNGTAATTGGGGTGTTCGCNRNNACCTNNGRAGTACYCRRYYGCTAATCGGNGCANCGTGGTNANGGCGCCTTGCTTCTGTACTAAGGTTGGCTACGTAGATGCATRCCGTATANTGCGGCAAGANAGAGCRAATCCATGGCAACGGGCTCTGCGACCACATTAGTTACYTAATGTGTCAAAGAATGATYGTTCGTRYCCNYCCCYGCGNNGCGAAGAANCTNAAARGCCCACTACGCAAAAGCCTGTCAAGTCGCGTTCAATNYCCNGCNCRYNYAGCGCNRTCGCGCCAGCCGRTTGCRRGYRATYCNAGGAYTAGCACTACTNCAAGNNACGGCCGGYACAGNRARCCYGCTGGTAGGYTANGNCTTRATTAGCCCGYYCCGGAGNGATCGTCCTCGTYGTYAGGGGCTACTYYRGTGRCRTACATCGCNNCNTACCYAYRCAGCAGCRGCAGCGTCTTAYAANACTCTAGACGCCYACATCGTCTCGGGCTAAYACTTCTCYANACTYYYGRNCACCGNTNGCCANNNNGCNNTGGAACANNGATACYCGAANCGRGCCTCCAAAGCTCGCCRTCTTACNAGACNCCCGACAGAATRCRGCGYNTTNYCCRYGTCAACNYTGNGCGTNGGTGACRATACACGACCGCCGGTTTATGAACCGCNCCACCGGGNNTGCAGGAGAAGAGACGNNNCATCNTARCGCAGGTGTCGACCTGCNNNAGCCGAGRRAATGGRAYCGYAGNCACGCGACCATATACYCCGCATGGACGCTNGCCTCAAGCGRGGGTGNCTACAYNNATCCCGGGYCTGAGRATCGAAYANCTNNTTCTAGNTGGGTTACCCCCCGATCCGTGCATAAAGTTAGCACGCTGCYNRTNCTGGGACCCACCGGCATTACGAGRATTANAGCNACNCCGTTGCYCNNTCACAGCGGTATTAAAGCCTCACGTCCNYTATRRNAGACGGRRTRAAACAYTNRRGCACCGYYGGACCATCCGGCCCGCGTCACGTGTGNAATAGNNNCTNGGTATGTGAGAGGAGTTCGGGCACCACTGTYNNNACCTCGAGTTTTCRRRYYRNCCATCCGACACNRCAGACATTGGCAGCTCNNAAGCGGTRGYYYRTNCCNCTARAGGTTACTCCAGGGGACGCGGAAGCCNCAARGTRCAATCNTTNTNGTGTTGGTNGGACGARRAGAGTAGTACAACTCTCACGTCTCAGGNNTGGTCCRTGCCRAAGGAGACATCCCAAAGGATAGTAACCGCGARANNCGCTACATGTCCTTYGNYAGTCTACATTGNAYNNTAANNNCAACGCTGCCTCRTCCCGACCGTACGCAGTAGATCTTAGAACCYAAATCGCCGGCTACCCCGANGCCARCCTCGCACAGCCAGAGCTACTGTATTTAAATNGACTRTYYYCATTGTAGTTGGCGGGCCCCTCTNNTTCTCGTNGCTATAACACACAGGCAGTTTGAGAAAGTATACATGCGGCAATTTGTAAGGNNNGGYYACANNGCCTGAGRGGCCANCCTAYTGCAGATRRRTTGCGCANNNNNGGCYTRRATCGGGTTCATCRTNRGCGGACCGGTCCGCAGGCGNCNYRTTTGATCGNNNYCGCRNYGGGCCCCTGCGACCTACNCGTCYGNRNCCGGGYNYYGTGGTCGTTCCGTATAACTNNNCTTAGTACTNRGATGCCCTTGTAAGCACNANNGAATAGRTYGNACCACGCCACACGRCGGCANAGNAYGCCNRCGGTNCCTTGTCTTAGNNGTCAAGTTTCCTCGCATTGTTCCNNNGCAATYATGCGTAACGGGATGGGRTYATGGCAGCTCGAATCNGGCGCAATCTCNGNYCCCRAAAAYYGCTGTCANNNNCGCGCACATCGAATGCACCGCGGCRTNNNTGGCGGGTACYNGCGGCCGTCATAGTRTTCATGGTGTTATGAGCTCTTCTTARGCGCGGACYGAAATCGCCACGGTTATGCGTAGATGGCGCAAATGTGTCCANNRCGNTAGCACGGACNTTGAAAGCGYGAATATTACGGCTCACTCCANNATAGGTYYAGGTANGRCTTCACANTGGCTRTNCCCTCGGGGGNCTGCCGYCGNGCAAATTCACNGRRGGNGAGCYRACACYYTGTTRNNGCNNGCTCNCGNGGTAATNNAATTGTGGCGTCNCCCTAACCRAAGNAGAATTNTGTTRYCTCRGRGGACGCCGGCATGRTNNNNCTTCACYCYYGATAYGTNTCGCAACACAACGAAGCCAGGCGTCGTATAACAGAAAGATGACAAAACTARRYRCGCAGTACGGGACGACGTAGCCANCACGGACCCTGATCGTGTTTTGNANCRNCACARCATCRGTYTCTGRNTTNACCCGGRNGCAGACNNCTCAGTCAGCAGGGATTAYTAGNCGAATARGNCAAGAGCCGGGCCANNNCGANNCTAGCGRGTCGTGAACAACCNGAAAGGCACCGCACATCTGCACTTGCCTCAGGGCNGNAGCGCCNNNAYACNCNGTTYCCACCTGNGGTYAGCCCTNNNANCTAAGGTAARCNNNATGAGCNATTGGGAGGCYGANTYYNRACGTCCGGTCCTATTCCTCGAACCGACCATAAAGTACGTGRRGAATTTNTYAACTTAACCCGCTAGACCRCRTGTYGGYACACAGCTRNNTCRGTCTAATGGGACTTCCAAGGTTTRCCNCYAAGCRRCAGGAGTTAGTTNGCNCACCCYCAATATGAYAYAAYNTACAGCAAGCCGATGGAAGGYNCTCGCYAGCATTTGCNTCYACATGCGTCNCYRNYTTTTCCATGTCTGGCTGGCGGAANNGAAGTAYTNGRTCYGTRRTGNNTAGCCGTCRCRGAGGTTACYGGCTGTCATRTAGCNCGRRYTGTGCCCAGATTRYYTNNNNNGTGTCAGATTGGTCTTGGCTTGGGGCGTCGGGACATAATCGCAAACGCCTCAGGTCCTARGAACGTRRGATCCTGANCGCTCRATCYCAYCTGTCAAAGNTACNGNTACGNCTTCTCAGCGACTGGCTAGCTTNRACYNNNNNTAACGRGGGTGTTAAGGGACTCTAGAAGGNCANCCNNRNRNTGTCNRYATGATCCTTARACCNCTTYGCGACGTGAAGGCAGAGCCCCTRGGCTGAGACCTRGNCCGTCCRCAAACNNGYCARYTNGRCCAGAGGCCGANANRGGCGCAACGTAACATAGCTYYACNRAGTGCGCCCAACNNNTCTAGCAGGAGCCGGTGTGCATACCCNTACCATTCGNNTGAGGCTTATCTGTCRGTACAACCATGGCGCCAACTGNNGAGTGCCYNGTACTTTGTATAAATGCCCTTATCGGAAGCATCNNNNCGYTCGATCCCGAGAYNARYCATTRRYYAGRYATTGCCYNYGYACGGCAACGGYCCGATGTCNCGACCTCGNGTCGGTATGTRGTGGATTTTTTAGATCCGACYCGAYTRCYCAAGATGNNGGCCCCAGCATCCTTACNCTGGAATTTAYYGTRRARRCRATCGGGCAAAGCRCTNCCTNNNNGCGGAGTCCGTTGATGACCGNATCAANTGAYCAGATTTGGGACCTAGCATCCNACARTNNNNNNTYNTYRNTTTCAGTCCGACGCGGTYTCGCAGTRCGCANN

Br57-1 GCGATTTACTCGTYRYCNTAATGGNCGCCRGRGTCGTCGACCTAGACGGGTCTAGGRGGNNTACCCACNGAYYGYRATCTACTGGCTATNNCGCCTTTTGTCTRRGGAGNCCACTCCTCGGGANCCRTCTTGGGTATRGGYYRTGCTNCGCTTANAATGGCCTGGCAGCGCGCAGGAGAGCCGAAAGTANNRACAGGTTTGGACCTGGCYYNGGNACNGYRGAYYNYACAATACGGCAGTCTCCAAGTACGNTTGGYANGCACATCNTAGGYCTNRTNAGRARGYTYTTGANRANCGYGGATCACTCGNNGGTCATYRNCYATTYCCARTNCCCGNGGATAGTYYGGTTRATGRCTTAGNYTNRCGAACGRTGGAGCCANTCTGGAGCTGCTAGGGCGGGGCGNGCGCCGCAGGTTCGGAACYTAAGTACATTACATTGCCGCNAGCTRGYYYYNAARCRCCYTCTCTYNNCTGCTGAATGGGCTGCATTTGGTTACCTTTGGCTGYCAATGARYNNNANCNGACACRGCNAATYATCCTAGTCTAGACTCRNNTAAGAGTNTARAGCCGACGCGTACGRTTTTAACACCANNNGGGCGARGAAAGAACACCTCCNNCTACCANCGGTGGACRTTCCCCTNRAAACAAGYGRYCGCTGAGNNTGAGCAGNCCAATCTCCTCGATCYNCTCCYYTRTNYGCANRCCCCYRCCNCCNCTTACGNNAGTACCTGGTNNAYTGCGCCGTGCGGTRRGTRCCARRTNGCTCCTATYNCATGCGAATGGTNGGCACTACCAAGGACTAGCCGRCTGGYNGCNAGGAACTGATATTATGYGAGTACATGCCCCGTCYGCACTNCRGNTNCTGCTAGCCCTGTACACAACGGRAYYGTYCNNNGGCTTGCGAATNYGRNTRAGTTCGGAANAACRGNCNYCNTGRNYYGGTCAGYCACNAAAGTTGYRANTNGNAGTAACTAGCGGYAGCCRYGGCTCATAARTTNNGRATAGCCYGCGGTGTARRGNCGRNCCRCGTCGGCGAGRRCCGCTTTGYYGCAAGGGATAATCAGACGATTAGATTATGTCAGRARRAANGCACTCYTCTTRACRNAGGNNACACGCTGTGGCTCCACTTTCNGNGGCCGGGGNNTTAGGCACGTCYGGGGGYCGCTGACTACTNGGGCCTAGGAACRNGNATGCCCAAACGATGGGGCCAGCCACACCGRYAANTTCTCGCAGGGGGCGCCAGTTNGNTTGCTGYAGGGTATTTCTNTGGCCCGACGGTAACGCGNCRGYNYYYNRAAAGACATACGCGTGNNATCANGCTCCAGAAATGTCRNTTAGGAATNNACTAGYACTNACGTACANRYRNCNNGCGCCTNRRTANCTCTCCGGGCNRCCRRCGCNNNTGNNRTGGGCTYGCCCCTCGACGANGGARGGGAAAARCCTCRRGACNCTGAGNACTCTGCGACCCTAGTGAACATAGAGCNTCGNCCAGTTTNRCATATGGTCTCTGGRNGCRRCNNTNNNNNGACTTACCCAGTGGCTTCCGCAGGAYTTTGTACAGYCANNNAGCGAACCTCCTGCACCTCCCAAGYGGTTTTTCANRTANCCNNCCTAGGTCNTTCCGAYGGAATTGCTCTGCYNGTGCANTCGTGAGCNCTATATCTRTTAACYAGGTGGGCACTGGTTTCGRRNTCGNRGTACTCGATATRAGGTCGGGGRYYACTATGTGCAGGTGGACTGTATTAGNCTNCTNYATNGARAYGGCATGGNCTGTTTGGATATCGTCCGCTTGGTTANYNGCYCGAAGCCCRTCCYCACGNGTCCCRRNRAGTGACGNTGAGGCCGNGGATTCTCGCTGGACACYTTNNAAAGCGTCCAGAGGATACTTCGTTYTCTYTTCATTTACGCNACCCAATTTCGTAATATGCCGGTGGCGTAGAGCTGGYRCCGCARTRRGTRTCTTGCAGAAYGGCTCAAGNRTCGGCGGCGCGTCAYNATCRNGCCCYTGGCATGGCNGCGACAGNTAGCCGTCCCACYCRATGGAGCGCGTYCCCACGAGGYAGGCGGGACGAATANNGAGACGCTATGACGGGGTTTAYNCANRTTTCACGCGGTTCAGTNGTTRYGNAAGGGCGGNNNNNNCCCCCAAACCGGTANGGGGYGYRATTGGRGTGTTCGCGAATACCTRNGRAGTACYCRRYYGCTRATCGGYGCANCGTGGYNAGGGCGCCTYGCTTCTRTATTAACATTGRCNACGTAGATGCRTGTNGAACTNTGCAGTGGGAAARRGCRAATCCATGGCAACGGGCTCTGCGACCACATTAGTNANNNAATGCGTCARAGANTGAYYGTTCGTGTCCGCCYCYGCGGCGCGAAGCGACYCAAANGCCCACTACGCAAAAGCCTGTCAAGTAAGGCCCAATCTCCTGCGCACNYRGNGANGTCGYGNYAGYNGGTTGCGAGYRATYCNAGGAYTAGCACTACTNCTAGANACGGCCGGYACAGGNARCCCGCTGNTANGYTNCGRCTTAATTAGCTCRCYCCGGAGCAATCGTCCTCGTYGTYAGNGNCTACTTCGGTGRCRNACANCGCATTAGACNCACGNNGCAGYGGCAGCGTYTTGCAANACTCTARACGCCCACANCGTCTCGGNCTAATACTTCTCCTCNCTCCTGATCACCGGTGRNCACTAGRCGYCTGTTCCTGGATANCCGNNNYGRGCYTYCNAAGCTCGCCATCTTACAAGACCCCYGACAGAATGCGGCGTATTACCCGCNNCAACGCNNAGCGTCGGTGACGAYGCRCGACCGCCGGTTTATGAACCGCACCACCGGACTTGCNNGAGAAGAGACGNGGTATCTTAACGCAGGTGTCGACCTGTNTYAGYCGRGRRAATGGRACCGCGATCACRTGACCNYNTACCNCGCATGGACRCYRGYCYCAAGYRRGGGYGTCNACATCRATCCCAGATCTGARRRTCGAAYANCTCCTTCTAGCTGGGTTACCCCCCGATCCGTGCATAAAGTTANCACGCTNNCNGTANTGGGACCCACCGNGNTTGCGAGGATTANAGCAACCCCGTTGNTCNNTCACAGCNGTNNTANNGCCTCACGTCCNTTATGGGAGACGGGGTGAAACATTAAGNCANCGTCGGACCNTCCNGCCCGCRTCACGTRTGTAANAGTCNTTAGNTATGTGAGAGGAGTYYGGRCRCCRNTGTCAGGACCTCGANNNNTCGGRYYAGCCAYNCAGCTYAACAGACATTGGCRRCTCACNAGCGATRRCCCRTNCCNCTAAAGGYNANTCCAGRNGACGCGGAAGCATCNARGTNYRAYCANYNTNGTRTTGGTNGGACGARAAGAGTANNAYRAYYCCTATNTCGCAGGANTRGTYCGTGCCGRAGGARNYATCNCNAAGGNTAGTNNCCGCGAGNTGCGCTANNNGTCCTTTGTYAGTTTGCATTGCATAANNNRATCGTCGCTGCCTCGTCNYGACCGTACGCANNAGNYYYYAGAACCYAAATCGCCGGCTACCCCGACGCCAGCCTCGCNCAGCCNGAGCTACTGTATYTAAATNGACTRTYYYCATTGTAGTTGGCGGATCCCTTTTANNCTCRYCGCNANRACNCACAGGCARTTTGAGAAATCATACACGCGGTAANCCGTAGGGCTAGGCTACACAGCCTANGGGGCCANCCTACTGCAGATGAGTYGCTCAGTCTCGNCTTAAATCGGGTTCATCGTGARCGGACCGCTCCGCAGGCGGCNYRCCTGATCGNNNYCGCATTGGGYNCCTGCGAYCTACCCGTYYGCGNCCGGGCNCCGTGGCCGTTCCGTATARCTGNTCCNGGGACTTRGATGCCCTTGTAAGCACCNYNGAATAGRTTGTANCACGCCACACGRCGGYNNAGGACGCCAGCGGYNCCTTGTCTTAGCCGTCAAGTTTCCTCGTGTTGCCCCCGGGCAATCATGCGTAACGGGATGRGNTCATGGCRGCNCGAATCAGRYGCGGTCTCTGNYCCCRAAAAYYGCTGTCATTTAYGCGCGCGTCGAATGCNCCGYGGCGTTGCTGGCGGRTRCYGGCRRCCRTCATAGTGTTYATGGTGTTATGAGCTCTCCTTAGGCGCGGACCRAARNCRNCNCGGTNATGCGTARNAGGCGCAAATGTGAGCAAARCGTTAGCACGGANNTTGAANNCGTGAATATTACGGCTCACTCCTATACAGGNYTAGGTACGNCTTCACANTGGCYGTNCCCCTGGGGGACYGCCGTYGCGCAAATTCACGGGGRGGGAGCCAACACTCTGTTRNNGYGGGCTCNCGAGGTTACTAAATNNNGRCRTYTCCCTAATCRAARNAGARTTNTGTTACCNCRGRGGACGCCGNCNTGGTGTCGCTTCANTCTTNATAYGTTTCGCATYAYANCGAAGCCANGCGTCRTATRAYANAAAGATGACAAANNNAGATACGCAGTACGGGACGACGTAGCCANCTCGNAYCCTGATCGTGTTTYGNANCNNCACANCATCARTYTCTRANTTGANCCGGGTGCAGACGTCTCAGTCAGCNGGGATTNCTANCCGAATAAGCCGAGAGCCGAGCCAACCCGARYCTAGCRRGTCGYGNACNNCCCGNAGRGCRCCACACATNTATATTTCAYTCAGGGCNGNAGCGCCNNNACACTCNGTTCCCACCTGNGGTYAGNNCTGNRAYCTAAGGTAAGCCCCAYRNGCGATTGGGAGGCNGAATCCNRATGTYNGRTCCNATTCCTCGAACCGACCRTAAAGTACGTGAAGAATTYGTTAACTTRACCYGCTAGRCCRCRTGTYGGYNCACAGCTGCCTCAGTACAATGGNACTTCCAAGRTTTRCCNCYAAGCGNCAGGAGTTAGTTGGNCCGYCCYCAATNTGAYAYAATCTNYAGCAARCCGATRRAAGGCACTNNCCAGCATTTGCATCCACATRCGTCNCYRTTTTTNNCATNNCTGGYYGRCGGAAYRGARGNTCTCGATCTGTRATGNNTAGCCGTCACRGAGGTTACYGGCTRTTGTRTRGCRCGRRYTGTGCCCAGATTACTCTCNAAGTNGYARRTAGACCCTNRCYTAGGGCGTYGAGAYATAANCGYAATCGNCTCAGGTYCTAGGAACGTGGGATCCTGATCGCNNNAYCCCACCTGTCAAAGTTACGGATRCGTCTYCNCNRCGACTGGCTAGCTNGAACCCTAGATAACGAGGATGTTAAGGGACTCTAGAAGGGCANCCTTGGRNNGTNCGTGTGATCCCANNNNNRCTTCGTGGCATGGRGGCAGAGCCCCTRGGCTGAGACCTAGGCCGTNCACAAACNNGYNAGTTNGGCCAGAGGCCGANAGGGGCGCAACRYARCATRRCTNCACGGAGNACACCGAGCTNTYYTAGCAGGAGCNNNNGTGCTARCCCGTACCACGYRCCTGRRGCTTATCTGTCAGTACAACCATGGCGCCAACTAGAGAGTGCCTGGTACTTTGTATAAATGCCYTTTCCGGGAGCATTNGANYGTTCGGACCCGAGNNANRYYATTARYYAGRYATTRCCYNYGYAYGGCNACGGCCCGATGTCGCGAYCTCGGGTYRGTATGTGGYRGAYTTTTTAGATCCNACTCGATTGCCCGAGATGGAGGCCCCAGCATCCTTACRCTGGGATTTATCGTGAARRCGATNGGGCRAAGCRCTACCTGCTGGCGRNGTCCGTTRGTGACCGGAACGANNGATCAGATTTTGTACCNNNNNNYYYANAATGGAGGGTCATTACATTCAGTCCGACGCGRYCTCGCANTGCNCATT

Br57-2 GCGATTTACTCGTYRYCNTANNGGCCGCCGGAGTCGTCGACCTAGACGGGTCTARGRGGTTTACCCACNGAYYGYRATCTACNGGCTATAACGCCTTTTGTCTGGGGAGCCCACTNCYCGGGRTCCRTCTTGGGTATRGGYYATGCTNCGNYTNNAATGGCCTGGCAGCGYRCAGNNGAGCCGAAAGTANNRRNAGGTTTGGACCTGGCYYNGGRACRGYRGACTNNACAATACGGCAGTCTCCAAGTACGNTTGGYAYGCACATCCTAGGYCTYRTRAGRANGNTYTTGANRAACGCGGATCACTCGNNGGTCATYRNCCATTYCCARTGCCCGTGGATAGNNYNNYTRATGGCTTAGACTTANGAACGRTGGAGCCANTCTGGAGCTGCTAGGGCGGGGCGGGCGYCGCAGGTTCGGAACCTAAGTACATTACATTGCCGCCAGCTNGNNNYNAARCGCCYTCTCTYTACTGCTGAATGGGCTGCATTTGGTTAYNTTTGGCTRNCAAYRRRYCCTATCAGACACRGCNAANYATCNNNNTYTARACTCRNNTAAGAGTNTARAGCCGACGCGTACGRTNTYAACACCRACGGGGCGAGGAAAGAACGYCTCCGGCTACCANCGGTGGACRTTCCCCTNRAAACAAGYGRYCGCTGAGNNTGAGCARNCCAATCTCCTCGATCCNCTCCYYTNTNNGCANRCCCCYRCCNCCNCTTACGNNAGTACCTGGTATAYTGCGCCGTGCGGTANNNRCCAGRTNRNTCCTNTYNCATGCGAATGNTNGGCACTACCAAGGACTRGCCGRCTGGTAGCTAGGAACTGATATTATGYGAGTACATGCCCCGYNYGNANTACAGANACNGCTAGCCCTGTACACAACGGAATTGTTCTTAGGCTYGCGAATNYGRNTRAGTTNGGAANAACRGNCNNCNTGRNCCGGTCAGTYACNAAAGTTGYRANTNGNAGTAACTAGCGGTAGCCRYGGCTCATAARNNNRGRATAGCCYGCGGTGTARRGNCGGGCCACGTCGGCGAGRRCCGCTTTRYCGCAAGGGATAATCAGACGATTAGATTATGTCAGRANNAANGCNCTCCTCTTRNNRNAGGTNACACGCTGTGGCTCCACTTTYGGNGGCCGGGGRRTTAGGCACRTCYGRGGGTCGCTGANTACTNGGGCCTAGGAACRNGAATGCCCARACGATGGGGCCAGCCACACCGRYAARTTCTCGCAGGGGGCGCCAGTTNGYTTRCTGCAGGATAYCNCNAYGGCCCGANRGTAACGYGNCRGYNYYYGAAAAGACATACGCGTGTRATCAGRCNCCAGAAATGTCRNTTAGGAATNNACTAGCACTNACGTANANRYNNCNNGCGCNTNGGTANCTCTCCGGGCGGCCRRCGCNNNTGNNRTTGGCTCGCCCCTCGACGANGGANGGGAAAARCCNNRRGACYCTGAGNACTCTGCGACCCTAGTGAACATAGAGCRTCGNCCAGTTTRRCATATGGTCTCTGGATGCGGCTATNCNCTGACTTACCCAGTGGCTTCCGCAGGACTTTGTACAGYCANNAAGNGAACCTCCTGCACCTCCCAAGTGGTTTTTCAAGTANCCYNCCTAGGTCNYTCCGAYGRAANTGNTCTNNTAGTGCNGYYGTGAGCYCTATATCTRTTAACCGGGTGGGCACTRNTTTCGANNTCGNRGTACNCGATATNAGGTCGGGGNTCACTATGTGCAGGTGGACYRTATTAGYCTRCTNYATNGANAYGGCAYGGNCTGTTTNGATATCGTCCGCTTGGTTANYTGCTCGAAGCCCRTCCYCACGNGTCCCGANRAGTGACGNTGAGGCCGNGGATTCTCGCTGGACACYTTCNAAAGCGTCCAGAGGATACTTCGTTCTCTTTYCATTTACGCYANCCAATTTCGTAATATGCCGGTGGCGTAGAGCTGGYGCCGCARTGGGTGTCTTGCAGAAYGGCTCAAGYRTCGGCGGCGCRTCRYNANCATGCCCCTGGCACGGCNGCGACAGNTAGCCGTCCCACYCRATGGAGCGCGTYCCCACGAGGCAGGCGGGAYGAATACGGAGACGCTATGACGGGGTTTAYNCANRTTTCACGCGGTTCAGTNGTTRCGCAAGGGCGGTTCGGGCCCCCAAACCGGTANGGGGYGYRATTGGAGTGTTCGCGAATACCTRNGNARTACYCRRYYGCTRATCGGYGCANCGTGGTNAGGGCGCCTYGCTTCTGTATTAACATTGRCAACGTAGATGCRTGCGGAACTTTGCAGTGGGAAARRGCAAATCCATGGCAACGGGCTCTGCGACCACATTRRTTACTTAATGYNNNARAGATTGAYYGTTCGTGTCCNYCCCYGCGGCGCGAAGCGACTCNNNGGCCCACTACGCAAAAGCCTGTCAAGTAAGGCCCAATCTCCTGCGCACNNRGNRANGTCGYGCCAGTCRGTTGCGARYGATYCNRGGANTAGCACTACTNCTAGANNCRGCCGGNACRGGRARCCCGCTGNTRNGYTNCGRCTTAATTAGCYCRCYCCGGAGCAATCGTCCTCGTYGTYAGGGGCTACTTCGGTGRCRNACANCGCATTAGACNCACGNNGCAGYNNCNGCGTYTTRCAANACTCTARACGCCCACATCGTCTCGGNCTAATACTTCTCCTCNCTCCTGATCACCGGTGRNCACTAGGCGYCGNNTCCTGGATANCCGAANYGRGCYTYCNAAGNTCGCCATCTTACAAGACNCCYGATAGAATGCGGCGTNTTAYCCNNNNCAACNYTGAGCGTCGGTGACGAYGCACGACCGCCGGTTTATGAACCGCACCACCGGACTTGCNNGRGAGGAGACGCGGTATCTTARCGNNNGTGTCGACYTGTNTYAGNCGGGRRAATGGAACCGCGATCACRYGACCNYNTACCNCGCATGGACRCYRGCCTCAAGYRRGGGTGTCYACATCRATCCCRGRTCYNARARTCGAACANCTCCTTNNNGCTGGGTTACCCCCCGATCCGTGCATAAAGTTANCACGCTNNCNGTANTGGGACCCACCGGGTTTGCRNGGATTANAGCAACCCCGTTGCCCYRNNNCAGCNGTNNTNAAGCCTCACGTCCTTTATGGGAGNCNGGGTGAAACATTAAGNCACCGTCGGACCRTCYNTCCCGCRTCACGTRTGTAANAGTCNTTAGRTATGTGAGAGGAGTYYGGRCRCCRNTGTCAGGACCTCGAGTTTTCGGRYYAGCCAYNCAGCTNAACAGACATTGGCRRCTCACNAGCGATGACCCRTNCCNCTAAAGGNNACTCCAGRNGACGCGGAAGCAYCAAAGTGCRAYCANYNTNGTRTTGGTNGGACGAGAAGAGTAATATAAYYCCTATNTCGCAGGANTRGTYCGTGCCGRAGGANNNNTCNCAAAGGATAGTAACCGCGAGNTGCGCTACNTGTCCTTTGTYAGTTTGCAYTGCATAANNNRATCGTCGCTGCCTCGTCNYGACCGTACGCANNAGNYYYYAGAACCNAAATCGCCGGCTACCCCGACGCCAGCCTCGCNCAGCCNGAGCTACTGTATNNNAATNGACTRTYYYCATTNTAGTTGGCGGATCCCTTTTANTCTCRYCGCTATAACACACAGGCARTTTGAGAAATCATACACGCGGTAAACCGTAGGGCNAGGCTACACAGCCTANGGGGCCANCCTACTGCAGATGAGTTGCTCAGNCTCGNYYTAAATCGGGTTCATCGTGGRCGGACCGCTCCGCAGGCGGCNYRCCTGATCGNNNYCGCATTGGGYNCCTGCGAYCTACNCGYCYGCGCCCNGGCTYYGTGGCCGTTCNNNRNARCTGNTCCTGGGACTTRGANNYCCTNGNAAGCACCNCTGAATAGRTTGTANCACGCCACACGRCGGYNNAGGACGCCAGCGGYNCCTTGTCTTAGCCGTCAANTTTYCTCRTGTNNCCCCCGGGCAATCATGCGTAACGGGATGRGRTCATGGCRGCNCGAATCAGRTGCGGTCTCTGGYCCCRAAAAYYGCNGTCATNNAYGCGCGCGTCGAATGCNCCGCGGCRTTGCTNRCGGGTGCTGGCRRCCATCATAGTGTTNATGGTGTTATGAGCTCTCCTTAGGCGCGGACCRAAANCRCCACGRTYNTGCGTAGAAGGCGCRAATGTGAGCANNRCGNTAGCACGGACTTTGAAAGCGYGAATATTACGGCTCACTYCTATACAGGNTTAGGTACGRCTTCACANTGGCYGTNCCCCTGGGGGACYGCCGTYGCGCAAATTCACGGGGRGGGAGCYRACACYYTGTTRNNGCGGGCTCNCGAGGTTACTAAATNGTGRCRTYTCCCTAATCRAARNAGARTTNTGTTACCNCRGRGGACGCCGNCNTGGTGTCGCTTCANNCTTNATATGTTTCGCANTRYAACGAAGCCANGCGTCRTATRAYANAAAGATGACAAANNNAGATACGCAGTANGGGACGACGTAGCNNNNTCGNRCCCTGATCGTGTTTYGNACYGNCACARCNTCARTYTCTGGCTTTACCCGGGTGCAGACGTCTCAGTCAGCNGGGATTNCTANCCGAATAAGCCGAGAGCNGAGCCAACCCGAACCTAGCNRGTCGYGNACNNCCCGRAGRGCRCCACACATCTATATNTCACTCAGGGCGGAAGCGCCNNNAYNCTCNGTTYCCANNTGTGGTCAGCCCTGAAATCTAAGGTAAGCCCYAYRNGCGATTGGGAGGCYGAATCCCAATGNCCNATCCTATTCCTCGAACCGACCATAAAGTACGTGAAGAATTYGTTAACTTRACCCGCTAGACCRCRTGTYGGYNCACAGCTGCCTCRGTNNAATGGCGTYYCCAAGGTTTRCCNCYAAGCGNCAGGAGTTAGTTGGNCCNCCCYCAATNTGAYAYAATCTACAGCAARCCGATRRAARRCACTYNCCAGCATTTGCATCCACATRCGTCNCYRTTTTTTCNANGNCTGGCTGGCGGAATAGAAGATCTCGATCTGTRNTGAATAGCCGTCACAGAGGTTACYGRCTRTTGYGTAGCGCGGRYTGTRCCCAGATTACTCTCYAAGTNGYARRTAGACCCTNRCYTAGGGCGTCRARAYANAANCGYAATCGCCTCAGGTYCTAGGAACGTGGGATCCTGATCGYTCGATCCCACCTGTCANAGNNACNNNTNCGTCTYCGCTACGACTGGCTAGCTYGAACCCTAGATAACGAGGRTGTTAAGGGACTCTAGANGGGCANCCTTGGRNTNTNCGTRTGANNCYNAGNCCRCTTYGTGGCATGGRGGCAGAGCCCCTRGGCTGAGACCTAGGCCGTYCACAAACNNGYNARYTGGGCCAGAGGCCGANAGGGGCGCAACGTARCATAGCTYCACGGAGAACACNGAGCTATTTTAGCAGGAGCNRGNGTGCTARCCCGTACCAYNYRCCTGRRGCTTATCTGTCAGTACAACCATGGCGCCAACTAGAGAGTGCCTGGTACTTTGTATAAATRCCCTTTCCGGNNNCATYNGANYGTTCGGACCCGAGNYANRYYATTAACYAGRTATTGCCYNYGYAYGGCNACGGCYYGATGTCRCGAYCTCGGGNCGGTATGTGGYRGAYTTTYTAGATCCGACYCGATTGCCCGRNAYRNNGGCCCCAGCATCCTTACNCTGGGATTTATCGTGAARRCGATNGGGCRAAGCRCTACCTGCTGGCGGAGTCCGTTRRTGACCGGANCNNNNGAYCARATTTNRNAYYCGGTATYYYAYAATGGAGGGTCATTACATTCAGTCYNACGCGNNCTCGCANTGCRCATT

BrK01-1 GTGATTTACTCGTCAYCNTANNGGCCGCCGGAGTCGTCGACCTAGACGGGTCTAGGAGGTTNACCCACNGANNGNGATCTACNGGCTATCAYGCCTTTTGTCTARGGAGYCCACTGCYYGGGANCCATCTNNNGTATRGGCCATRCTGCGCTTACAATGGCCAGGCAGCGCGCAGGAGAGCCGAAAGTACAGACAGGTTTGGATTTGGCTTNGGRACNGNNGATCTNACAATACGGCAGTCTGTAAGTANGTTNGGYACGNAGATCCTAGGYCTCATGAGGAGGTTYTNRANAATCGTGGATCACYCRNNGGGCATYNACTATTYCCAATGCCCGTGGATAGTTNGGTTGANGRTGTAGCTTGGAGAACGGTGGNGCCATTCTGGAGCTGCNNGNNYGGGGCGGGCGCCGCAGGTTCGGAAYCTNRGTRCATTACATTGCCGCCAGCTAGCCTTCAARCRCYCTCTCTCTACTGCTGNRCGGTCTGCRTYTGGTTACCTTTGGCTGNCAACGAACCCTATCAGANACRGCNAAGTATCCTNGTCTNRACTCRNNTAAGAGNNTAGAGCCGACGCRNACGGTTTCAACACCRACGGGGCGAGGAANGAACACCTCCGGCTACCAACGGTGGRCRNTCCCCTAGAAACANGCGRYCGCTRAGNNTGAGCANRCCAANCTCCTCGATCYGNTCCYYTRTGYGNNNGCCCCYRCNNCCGCTTAYGNNAGTACCTARCNTACTGCGCCGTGCGGTGGGTACCAGGNGAATCCTNNYNCATGCGAATGGTNGGCACTACCGTAATCTAGCCGGNTGGTAGCTAGGAACTGATATTATGTGAGTACATGCCYNGCYCGCNGNACAGANACNGCNAGCCCTGTACRCAACGGAATTGTTCTNAGGCTTGCGAATTTGACTGAGTTCGGAAANNNNGGCGCCGTGGTCCGGTCAGTCACTAAAGTTGCAAATTGTNGTARCTAGCGGYAGCCATGGCTCATAARTTNRGAATAGCCCGCGGTRTAGGGGCGANCCGCRTCGGYRAGAGCCGCTTNGCCGCRAGNGATAATCAGACGATTAGATTATGTCNGAAAGAANGCNCNNCTCTTANCRNAGGTNACACGCTGTGGCTCCACNNTCNGCRGCCGRGGGATTAGGCACGTCCGGGGGCCGNTGANTACNAGGGCCTAGGAACAAGAATGCCCAGAAGATGGGGCCAGCCACACCGRYAARTTCTCGCAGGGGGCATCAGTTNGYTTGCTGYAGRRNATTTCTTTGGCCCGACGGTAACGCGTCAGCCCTTGAAAAGAYTTAGGTRYNNGNNCRNACGCCAGAAATGTCRNTTAGGAATYNACTAGYACTGACGYRCANRYATCAGGCGCATCGGTANCTCTCCGGGCGGCCGACGCGAANGAAATTGGCTCGCCCCTGGACAAAGGAAGGGAAAAACCTCNNGACYCTGAGNACTCTGCGACCCTAATGAACATAGAGCATCGCCCAGTTTARCATNTGGTYNCTGGGAGCAGCCGTCCNCNGACTTACCCAGTGGCTTCCGCAGGACTTTGTACAGCCAGAANGCGAACCYCCYRCACCTCCCAAGTGGTTTTTNACATANCCTNCCYANGTYGCTNCRANGGAATTGCTCTATTAGTGCARTCGTGAGCCCTATATCTRTTAACCGGGTGGNNACTACTYYCGRRNTCGTAGTNNTCGATATAAGGTCGGGGRTCACTANGTGCNNGTGGACTGTATTAGTCTGNNTCATTNRRAYGRYAYGGTCTGTTTGGATATCGTCCGCTTGNTTANYNGCTCGAAGCCCNTCCTCACGAGTCCCGAAGAGTGACGGTGGGTCCGGGGNTTCTCGCTGGACANTTTCCAAAGNGTCCAGAGRNTACTTCGTTCTCTTTNCATTTACNCTANCCNATTTCGYAATGTGCCGGYRGCGTAGAGCTGGYRCCGCARTRRGTGTCTTNCAGAATGGCYCAAGCGNCNGYGGCGCATCGCTANCRNGCCCYTGGCAYGGCCGCGACAGCNRGCCGNCCCACCCAATGGAGCNNNNCCCCACGAGGYNGGCGGGACGAATACGNAGNCGCTATGACGGGGTTTGTACANRNTCCACRCGGTTCANYCGTTGNGCAAGGGCGGNNNGGGCCCCCAAACCGGTATGGGGYGTAATTGGGGTGYTCGCAAATACCTRNGGAGTACYCRRYYGYNAATCGGCGCATCGTGGTNAGGGCGCCTYGCTTCNGTATTAACATYGGCAACGTAGATGCATGYGGAACTNNGCAGTGGGARRGRGCGARNYCRYGGYRRYGGGCTCTGCGACCACATTAGTNANNNAATGCGTCAAAGANTGATCGTTCGTGTCCNYCCCNGCGGCGCGAAGNRACTNAAARGCCCACTACGCAAAAGCCTGAAGAGTNRNGYYCAATNYCCGGCNCRYGCNGNRAGGTCGCGCCAGYCRGTTGCGAACRATTCGRGGAYTAGCACTACTNCNNGAAACGGCCGGTACGGGAAGCCYGCTGGTRGGYTTCGRCTTAATTAGCTCGYCCCGGNGCAATCGTCCTYGTYGNNNGGGGCTACNCTAGTGGCGNACATCGCAYTATACCYAYGCAGCAGCGGCAGCGTTTTGCAAGACNCTAAACGCCYACAGCGTCTCGGNCTNATACTTCTCCTCNCTCCTGATCACCGGTGACCACTAGGCGCNGTATCCTGGATACYYNAAATGGGYCTYCAAAGCTCGCCATCTTACAAGACYCCCGACAGAATRCRGCGYNTTNYCCNNGTCAACTYTGAGCGTCGGTGNNNTTGCACGACCGCCGGTTTATGAACCGCACYANCGGGYCTGCAGGRGARGAGACGYGNTATCNTAAYGCAGGTGTCGAYCYGCATCAGTCGGGGAAATGGRACCGCGATCACGTGACCNYNTACYCCRCATAGACGCYRGCCYCAAGYRAGGGTGTCYACATCGANYCCRGRNCTGARRRTCGRRYANCTNNTTNTAGCYGNNNTACCCCCCGATCCGTGCATANNGTTACCACGCTNNCCGTAATRGGACCCACCGGGNTTGCGAGRATTAAAGCNACTCAANTGCCCTAYCACAGAGGTATTNNANYCTCRCGTCCNTTRNRRNAGACGGAGTGAAACATTAGAGCACCGTCGGAYCRTCTGTCCYGYATCACGTGTGTNATAGTCNTTAGGTATGTGAGAGGAGTTCGAGYRCCAGYGTCTTGACNNNNNNNNNTCGGRYYRGCCACCCAGTTCNAYAGACATTGGCAGCTCNNNAGCGRTAGCCCATCCCACTAGNNGTTACTCCAGRTRACGCGGAAGCNTCAARGTRYAATCAACCTAGTATTGGTCGGACGAGAAGRRTNATRTAATCCCTATTTCGCAGGAATGGYCCGTGCCGRAGGAGTTATCACCAANGNNAGTRNCCGCGAGNTGCGCTACNTGTCCTTTGTTAGTNTNCATTGCAYAATATAATCRNCGCTGCCTYANCCCGACCGTACRCANAANNCYCCAGAACNYAAATCGCCGGCTACCCCGACGCCNGCCTCGCNNANNNAGAGNTNCYRYNYYYRAATCGACTGTYCTCATTGTNGYTGRCRGNYCCCTTTTANTNTCACAGCTATAACACACAGRCAGTTNGAGAAATCATACRCGCGGTANNCCGTAGGGCNAGGCYACACAGCCTRAGRGGCCANYCTAYTGYRGATGRGTTGCTCAGTCTCGNYYTAAATCGGGTYCATCGTGRGCGGANCGNTCCGCAGGCGGYGTGCCTGATCGNNACYRCNNNGGGCCCCTGCGACYTACGCGTCCGCGCCCGGGYNYYGTGGNCGTTCYGYRTNACTGANCCAGGGACTTAGATGCCNNTGTAAGCACNATGGAATAGATTGTACCACGCCACNCGRCGGCANAGGACGCCAGCGGTNCCTTNNCTTAGNNGTCAAGTTTYCTCGYRTYGCCCCCRRGCAATCANGCGTAAAAGGATGGGRTYATGGCARCNCGANTCAGATGCGGYCTCTGNTCCCAAAAATTGCAGTCATNNATGCGYAYGYCGAATGCACCGTGGCGTTGCTNGCGGGTRCTNGCGRCCNTCATAGTGTYCATGGTGTTNTGAGCTCTCCTTARGYGCGGACCRAAATNGCCNCGANCATGCGTNGAAGGCGCRAATGTGTCCNNNGCGNTAGCACGGANYTTGAANGCGYGAATATTRCGGCTCACTCCTATACAGGNYTAGGTACGGCTTCACACTGGCTGTNCCCCTGGGCGACCGCCGTTGCGCARNTTCACCGRRGGGGAGYNNACACNTTGTTAAAGTGGGCCCNCGNGGTTACNRANTTGTRACATCNCCCTNACCGNAAGAGAATTNTGYTGTCTCGGAGGACGCCGGCATGGTGTCGCTNCACYCYYGATNTGTATCGCAACACACCGAAGCCAGGCGTCRTATRAYANAAAGATGACRAAACTAGATANGCAGTACGGGACGACGTAGCCANCACGGRCCCTGATCGTGTTTCGNNNYNACNNNACNTCAGTCTCTGACTYTANCCGGGTGCAGACNNCTYAGTCAGCAGGGATTTCTAGCCGAATAAGCCGAGAGCCGAGCCAACCCGAGTCTAGCGGGTCGYGNACNNCCCGAAGRGYRCCACACAYCTATATTTCNCTCRGGGCGNNRGCGCYAAAATCCNCNGTTCCCACCTGAGGTCAGNNCTGAAATCTAAGGTAAGCCCCAYRNGCGATTGGGAGGCYGRANCCNRNYGTCCGRTCCYRTTCYYYRAACNGACCATAAAGTACGYGRRGAATTTGTTARCTTGACCCGCTAGACCRCRTGTYGGYNCACAGCTRNNTCRGTCTAATGGNACTTCCAAGRTTTACCACCNAGCGGCAGGRRTTAGTTGGCCCACCYTCAATNTGNNANAAYNTACAGYAAGCCGNTRRAARRCACTYNCCAGCATTTGCATCCACATRCGTCACTGNCTTTTYCATGTCNNNYYGGCGGAACGGAAGATCTCGATCTGTRATGNNTAGCNGTCACAGAGGTTACCNGCTGTTGYRNAGCGCGGGTTGTGCCCANNTNNNCCTCCAAGTGGTAGATAGACCCTNACTTAGGGAGTCGGRAYACAATCGCAATCGCCTCAGGTCYTAGGAACGTGGGATCCTGATCGCYCGRYCCCACCTGTCANAGCTACCNCTGCGTCTTCNCNRCGACTGGCTAGCTNGAACCCTAGATAACGAGGGTGTTAAGGGACTCTAGAAGGGCATYCTTGGATNGTNCGTATGATCCTTAGACCNNYTYGYGGNANGGGGGCAGAGYNCCTAGGCTGAGNCCNAGGCCGTCCGCAAACNGGCNARYTGGGCCAGNNNCCGANAGGGGCGCAACRYARCATGACTTYACNRAGNACACCNNGCTATTTTAGCAGGAGCCGGNGTGCNNRCCCNTACCACGCGNNTGNNGCTTATCTGTCRGTANARYCATGGCGCCAACTRRAGAGTGCCCGGTANNYTNTATAAATGCCCTTNCYGGGARCATTNGANNGYTCGGTTCCGAGNTNNGCCAYTNACYAGGCATTGYCTTTGCACGGCAACGGCNNRRTGTYACGANCTCGGGNCGGTATGTGGCANNCTTTYTAGATCCGACTCGATTNNCCRRNATGGAGGCCCCAGCATCCTTNCGCTGGGATTTATTGTRAARRCGATCGRGYAAAGCRYNNCCTRCNGGCGGAGTCCGTTGANGACCGAANCRAGNGATCAGATTTNRNNNNYRGCGTYCTACAGTGGCGAGTTTTYRCATTCNGTCYNNCGCRGYCTCGCANTGCACATT

BrK02-1 GCGATNNNCTNNNCANCNNANNNNNNNNNNNNNNCGTCGACNNNNNNGGGNCTAGGNGGNNTNNCNACTGANNNNNNNNNNNTGGCNANNNNNNNNNTNNTCNNNNNNGNCNNNNNNTCGNNNNCNNNNNTGNGTATNNNCCNNANNNNNNNNNNNNTGNNNNNNCAGCNYNCAGNNGAGCCGNNNNNNNNNNNNNNNTTGGATTTGGCTTNGGAACGGNNGATCTNANNATACGGCNGTCNNTNNGNNGGTTGGGNNCGANCATCNTAGGYCTTGNNNNNNNNNNNNTNACNNNNNNNNATCACYCNNNNNNNATNNNCTATTYCCNATGNCCGTGGANAGCCNNNNNRATNNYNTAGACTNANGNACGRNGGANCCATTCTGNNNYTGNTANNNNNNNNNNGGCGCNNNNNNNNNNNNNYCTNRGTRCRTTACNNNNNCGNNNNNNNNNNNNANANNNCTCTCTCTNNNCTGCTGAANNNNCTNNRTYTGGTTACNNTTNNNNNNCAAYGARYNNNNNNNNNNACRRCNAAGTATCCTNGTCTARACTCNNNTANNAGNNNRNNNNNNACGCGNNNNRTTNNAACACCNNNNNNNNRAGGNACGANNNNNNNCNNNNNCCANNGGTNNNCATNCCCCTAGNNNNAAGTGACNGCNNNNNNTGAGCAGRCCNNNNNNNNNNATCNGCTCNNNTATGTGCANRCCNNNNCNNCCNNNNAYGNNAGTACNNNNNNNNNTGCGCCGTGNNGTNNNNRCNNNNNNNNNNNNATNNNRTGCGAATNGTNNNNACTACCNNGNNCTAGCCGGCNNNNNGCTAGGNNNNNNNNNNNNGTGAGTACATGCNNNGYYCGNANNNCNGNTACAGCTNGCCTNNNACACAANNGNANNGTNNNNNNNNNNNCGANNNNNNNNNNNNNCNNNAANNNNGNNNNNNNNRNCCNNNNNNNCACANNNGTTGNNANTNNNAGTNNCTNGCNGYAGCCNNNNCNNNNANANNNNGNNTARCCCRCGGNNNNGNGNNNNNNNGCGTNNNNNNNGRCYRCYNNNNCGCAAGGGATAATCAGACGATTAGATNNNNNCANNNNNAANGYNNNCYTCTTAANNNNNNTANCNNNNNNTNNNNNNNNTTTNGNNNNNCGGGGAGNTAGNNACGTCNNNNGGTCGNNNNNNACNNNNNNNNAGGNNNNNGANNNNNNNAACGATNNGGCCAGNCACNNNNACRARTTCNNNNNNGGGACATCAGTTNNNNNGNNNCNNNNNNNNNNTNTGNCCCGANNRYANNNNGANGGTATYYNRNNNNACNTANNNNNNNNNNNNNACTCCAGAAATGTCGATNNGNNNNNNNCTNNYACTGACGCNNACGTGGCCTNNNNATCGGTNNNNNNNNNNNNNNNNNNNGYGAATGNNNTNGGCTNNNNNNNNNNCGAANNNNNGNNNNAACCNNNNGACYNNNAAGACTYNNNGACCCTNATGAACATAGNNNNTCGCCCNNNTTNACATATGGTCTNNGNNNGCGGCCGTCCTCTGACNNACCCAGTGGCTTCCGCAGGANNNNNNNNNNCCAGAAAGCGAACNNNNNNNNNCTCCNNNGNGGTTTNNNNNNTNNCCYNCCNANGTNNNNCCGNNNNNNNNNNNNNNNNNGTGCTNTNGNNNNNNNTNNNNNNNNNNNNCNNNNNNNNACTACTNNNGGNNTCGTAGNNNTNNAYATNNNNNNNNGGATCACTATRTRNNNNNGGACNNTATNNNNNNNCTTCNNNNAGATGGCATGGTNTGTNNNNATNNCGTCNNNTTGNTTATTNNCTCGNAGCCCGNNNNCNNNNNNNCCGANGAGTNNNGGTNAGNCNNGRGANNCNCNCTGGACACNNNCNAAAGCGTCNNNAGRATACTNNNTNCNNNNNNCNNTNNNNCNANNNANNNNNNNNNNNNGCCGGNNNCGYANNNCTGNNANCGNNNNNNNNNTCTTGCAGNATNNNNCAAGCGNNNGCGGCGNNNCANNNTCRNNNNNCTGGCNYGGCNNNNNNAGCTAGCCGACCCACTCGATGGCTYGCGTNNNNNCGAGNNANNNNNGANGAATACGGAGNCGNTNNNNNNNNNNNNNNNNNNNTTYCACGCNNNNCAGCNNNTNNNNNNGGACGNTTCGGGCYCCYAGACCGGNNNNNNNNGNNATTGGGGTNNNNNNNAATNNNNNNNNNNTACYCRRYYGCTAATNNNCGCATNGTGGTNNGGGNGCNNNNNTNCTNTNNNNACATTNNNNANNNNGATGNNTGTNNNACTNNGNNNNNNNNANNANNNNNNCCATGNNNACGGGCTCTNNNNNNNCATTNNTTANNNAANNYGTCNNAGANTNNNNNNNNNNGTNNNNNNNNNCGNNNNNNNNNRNCTNAANGNNNCANNNNNNNANNNNNNGAANAGTNNGGCCNAANNTCCGGCNNNNNNNNNNNGGNNNNNNNNNNNGNTTGCGAGCGATCCCNGGNNNNNCACNANTNNNNNNNNCNGCCGGNNCAGGRANNNNNNTGNNGNNTNNNNNNNNGATTAGCCCGCYCCGGNGCNATCGTCCTNGTNGTNANNNNCTACTNNNNTGACATACATCGCNNTAGACCNNNNNNNNNGCNNCAGCGTCTTNNAANACGNNAAACGCCCANNNCNTNNCGGGNNNNNNNNNNNNCTNNCNCNNNNTNACCGNNNNNCACTAGGTNNCTGTNNNTGNNNAACCGAANNNNGCCTCNNRAGNNNGCNNNNNNACAAGACTCCCNACAGAANNNNGNNNNTNNNYCNNGTNANYGCTGAGCGTCGGTNNNNATGCANGACNNCCGGTTNATGAACCGCNNNNNNNNNCYTGCAGNGGAGGAGACGCGGCATCNTAATGCNNGTNNNNNCTTGTTNNAGCCGAGRRAATGGGANNNNNNNCNNANGANNNNTTACCNNNNNTNNACGCYNGCNNYAAGYNNGGNNNTCTNNNNCGNNNCCRGRTCNNNNNNNNNAANAACTCCNNATAGNNNGGTNNNCCCCNNNNNNNNGYNTAANNNNNNCACGCTTACCGTANTGGNACYYACCGGGATNNNGANNNNNNNNNNNACNCCGTNGCNNNNYCANNGCGNNNNNAAANNCNCNCGNCCTTTATGGNAGACGGGNNNNAACANNNAGNNNNNNNNNNNNCATCCGGCACGCNNNNNGTGNNNNNNNGTCNNTNNNNNNNNNNNANNNNTTNNNNNNNNNNNNNNNNGACNNNNNNNNNNNNNRYTAGCNNNNCANNNNAACNNNNNNNGGCAGCTCNCNAGCGGTAGCCCATCNNNNNANNNNYNACTNCAGNNNNNNNGGANNCCNNAANNTNNAATCNNNNNNGTNNNNNNNNNACGANNNNNNNNATATNNNNCCNNNTTCGNNGGAATGGNNNNTGCCGNNNGNNNNTTNNNCAAGGATNGNNNCCGCGAGNTGCGCTACNTNNNNNNNNNTAGTCTACNNNNCAYAANNNNNNCGTNNNNNNNNNNNNNNNNNNNNACNNANAAGNYYYYAGAACNYAANTCNCCGGCTNCCCCGNCGCCAGCCTCGNNNNGCCTGNNNNNNNGTATCTAAATNNNCTNNNNNNNNTGTAGNNNRCRGNNNCCNNNNNNNNNNNNNGCNANRNCNNNNNNNNNNNNNGATCAATCATACACGNNNNANNCCGTAGGNCNANNCTACNNNNNNNNNNNNNNNNGNCTANTGNNGANGRNNNGCNNNNNNNNGCNCTAANTCRNNNNCANNNNNNGCGGNNNNNNCCGCNNNCGGYGTGNCTGNNNNNNNNNNNNNNNNNNNNCTNCGACCTACNCNYCYGCGCCCNGGNGCCNNNNNNNNNNNNNRNAACNGNTNNNAGGACTTRGANGCCCNTGTANNNNNNCNNNNNNAGNNNNNACCANNNNNNNNGNCGGCANNNGACNNNAGCNNCACNNTACNNTAGNNNTNNNNNNNNCTNNNNNNNNNNNNNNGCNNTNNNNNGTAAAAGNNNNRGNTCNNNGNNNCGNNNNNNNNNNNNNNNNNNNNNTCCCAAANNNNNNNNTNNTTTATGNNTACGTNNNANGYANCNNNNNNTNNNNNNNNNNNACYNNNNNCNNNCATNNNGNTCNNGNNGNNNTNAGNNCTNCTTANNNNNGGACCAAARTCNCCANNNNCNTGNGTAGAANNCGCGNNNNNNTCCANNNNNNTARCACGGANYNNNANNGCNNGAATNNTACGGCNNNNNNNTNNNNNNNTNNNRGTNCGRCNTCACNNNNNNTGTANNNCTGNNNNNNYNCCNNNNNNNNNNNNNANGNGGGGNGAGYNNNNNNNNNNNNNNNGNNNNNNNNNNNNNNNANNNNATNNNGGCGTNNCNCTNATCNNANNNNAANNNNNCTACCACGGAGGACNNNNGCATGNNGTCGNNNCACTCTTNANAYGTNNNNNNNTGTNNCGNAGCCAGGCGCCNNATANNNNAAAGATGACANNNNAAGATACGCAGNNNNGGNNNNNNNNNNNNNNACGNNNNNNRANNNTGTTTNGNNNCNNNNCANCATCGGTCNNNGACNNNNNNNNGGTGCNNNNNNNTTANTCAGCAGGGNNNNNTAGCCGNNNANGNNNNNNNNCGRNNNAANCCGAACCNNNNNNNNNNNNCACNNNNCGAAGRGCRNNNNNNNNNTATATNTCNCNNNGGGCGNNNNNGCCNNNAYACTCNGTNNNNNCCTNNNNNNNNCCNNGAANTCNAAGGTAAGCNNNNTGAGCGNNNGGGNNNNNNAATNNGGNTGTCCGNNNCNATTNCTCGNNNCGACNGTAAAGTACGTGRRGANTTTGTTAACNNNNCYCGCTNGANNNNNTGTYGGYNNNNNNNNNNNNNNNNNNAANNNNNNTTCNAAGNTTTRCCNCNAAGCNACNNNAGTTAGTTGRNCNNNNCTCNNTNTGNNNNAAYNTNNANNNNGCCNNTAAAAGGCACTNGNNNNNNTTTGNNNCCANNNNCGTCNNNNNNTTNNNNNNGNNNNNNTNNNNGAANNGANNNNCNNNNNNNNTGNNGNNAAGCNGTCAYAGANNNNNCYGGNTGTNNTNTAGNNNNNGTTGNNNCNNNNNNNNCCNNNAAGNNGCNNNNNGNCCNTTACTNNNNNNNNYNNGANATANNNNNANNNNCNNNNNNNNNNANGAACGTGGGATNCTGATCGCTNRAYCCCACCTNTCANNNNNNNNNNNGCGNCTTCNCNRCGACTGGCNAGCNNNNACNNNNNNTANNNNNNNYGTTNRRGGACTNNNNNAGGGCNNCCTTGGATNGCNNNNNTGANYCTTAGACCNNNNNGTGNNNNNNNGGCAGRGNNNCTRNNNNNNNNNNNRGNCCNNCCGCNNNNNGGCCANNTNGGCNNNNNNNCGNNNNNNNNNNAACNNNNNNNRGCNNCACGNNGNNNNNNNNNNNNNTNTAGCAGNNNNCGNNGNNCATACCCNTNNNNNNNNNNNNNNGCTNNNCTGTNNNNANNNNNNTGGCGCCANCNNNNGNGNGNNNNNTANNNNNNNNAAATGCCNNNNNNNNGAGNNNNNNNTCGTNNNNNNCCGAGNNNNNNCATTAACNNNNCATTGCCTTTGCACGGCNNCNNNNNGNTGTCRCGACNNCGGGACGGTATGTGNNNGNNNTTNTAGATNCGACCCGATTNNNNGGCACATCGNCNNNNNNNNNNNTACNCTGNNNNNTATTGNNNNNNNNNTCGAGNNNNNCGNNTCCNNNNNGCNNNNTCCGNTRRTGANCGAATCANGGGACCNNATTNNNNNNNNNNNNNNNTNNARTGGCGRRNNNNNNNNNNNAGTCNNNNNNNNNCTCGCAGTGNNNNNN

BrK04-1 GCGATTTACTCATCRTCTTANNNNNCGCCGGAGTCGTCGACCTAGACNNGNCTARGRGGNNTACCCACTGACCRTGANNNACTGGCNATNACGCCTTTTGTCTRGGGAGCCCRCTNNYCRRGATCCNTCTTGGGTNNNGGCCATRCTGCGCTTNCNNNNGCCNGGCAGCRNGCAGGAGAGCCGNANGTANNRRNAGGTTTGGACCTGGCTTTGGRACRGTAGATCTYATAATACGGCAGTCNNYAAGTANGTTNGGNACGCANAYCCTAGGYCYYRNNAGRANNNNNNGAACAATCNNNNATCACTCGNNNNTCATCACCYATTYCCANTGTCCGTGGATAGCCNGGTNGATGRYNTAGACTTANGNACGANGNNNNNNNTCTGNNNNTGTTAGRTTGGGGCGGGCGCCNCAGGYYNNGAAYCTNRGTRCATTACAGTGCNNCCAGCTGRTCCYNAARNNCYCTCTCTNTACTGCTGAATGGGNNGCATTTGGTTRCCTTYGGCTRYCAAYGARYCNTNNCRNNNACRGCNAAGTATCCTNGTCNARACTNNNGTNNNAGTCNRGANCCGNCGNNTANGATTNNNACACCRACGGGGCRAGGTACGNACRYCTCCGGCTANNNACRGTGGACNNTNNNNYANAAACAANNNNNAGCTGAGNNTGAGCAGRCCTGTNNNNNCGNTCCGNTCCCTTATGTGCNNNCCCCYRYCNCCGCTTACGNNAGTACNNRGYGGANTGCGTCGTGCGGTGGGTRCCNGNTNGCTCCNATTGCATGNGAATGGTNGGCACNACCAAGGACTAGCCGGYNGGTAGCTAGGAACTGATNTNNNGTGAGTACATGCCCCGNYNGCANTNCRGNNACANCTAGCCYTGTACACAACGGAATTGTTCTTAGGCTYGCGAATNNGNNNNARNTCGGAAAAANGGNNNNCGTGNNCCGGTCAGTNNNTAANGTTGCAAATTGTNGTARCTNGCGNTAGCCRYGGCTCANAANTTNNGGNTARCCCRCGGTGTARRGGCGNNCCGCGTCGGCGAGGRCYRCYNNNYCGCRAGGGNTAATCANNCGATTAGATTATGNCTGAAAGNNCGCACCNCTCNTANCGCAGGTANNACGCTGTGGCTCCAYTTTCNNCGGCCGGGGRRTTNNGCACGTCCGGGGGNNNCTNAGTACNAGGGCNNAGNAACRNGAATGCCCAAACGATNGGGCCAGCCACACCGACRANTTCTCGCAGNNNNNRYCAGTTCGTTTGCTGCRRGRTATYNNNANGGCCCNNCGRYAACGCGNCNGNNYYYNRNNNNACATAGGTRYGNRATCRGACTCCTGNNNNNNCRNTNNGGAANNNNCTNGYACTGACRTANNCGTATCAGGCGCATCGGNANNNNNNNGGGCGGCYGACGCGAATGAAATGNNNNCNNCCCTGGACAAANNNRGGGRRANACCTCGGGACYCTGARNACTYNNNGNNNCTAATGAACATAGAGCATCGCNNNGTTTNRCATATGGTCTCTGGRNGCRGCCGTNCNCNGACTTACCCAGTGGCTTCCGCAGGACTTTGTACAGCCAGAAAGNGAACCYCCYGCACCTCNCAAGTGGTTTTTNNAGNANCCTNYCCACGTTGCTCCGACGGAANNNNNNNGCYNGTGCTRTYRTGAGCCCTNNATCTNTTAACCNGGTGGGCANTGGNNNCGGNNTCGTAGTNNNCGATANAANNNNGGNNRNNACNNNGTGCAGGTGGACCATNTTAGTCTGNNTCATTGAGAYGGCATGGTCTGTTNGNATNNCGTCCNNTTGNTTATTGGCTCGAANCCCRTCCNCACGCRTCCCRRNGGGYNACRGTGAGGCCGNNNNNNCTNRCTGGACANYTTCCAAAGNNTCCAGNNNNTACTTCGTNYNNNNNTCATTTACNCNANCCAATNNCGTAATATGCCGGCACCGYAGAGCTGGYNCCGCARTRAGTGTCTTGCNNNATGGCTCAAGCRTCGGCGGCGCGTCNCTNNCANGCCCCTGGCACGGCGGCGACAGCTAGCCNNCCCACTCGATGGNNYACTTNCCCACGAGNNAGGCGGGAYGAAYANNGAGNNGCTNNNNNGNGGNTYAYNCANNYYYCAYRYNGNNCAGCNRTTRCGCAAGGACRGTTCGGGCYCCYARACCGGTATGGGNNGTAATTGGRGTGTTCGCAAATNNCTGANANGTACTCGACCGCTARTCGGYGCANCGTGGYNAGGGCGCCTYGCTTCTGTNNNNNNNNTGGCAANNNNGATGCATGTGGAACTNTGCGGCGGGARRGANNNNATTCGCGGCAACGGGCTCTGCGNCCACATTAGTTACCTRNTNYGTCAANGANTGATNGTTCGNGTCCNYCNCCGCGGCGCGAAGAANCTNAAAGNNCCNCTACGCRNAAGCCNGAANAGTCGCGTTNAACATCNNNNNNNNNNRRNRNNGTCGCGNYAGCNGGNNGCNNACGANTCNRGGAYTAGCRNNACTNCNNGAAACGGCCGGTNYRRNNANCCCGCTGNTNGGNTNNNNNNTAANNNGCYCGCTCCGGAGNANNNGTCCTNGTTGNNNGNGNCTACNTCGGTGNCNNACANCGCATTAGACCYAYGCAGCGGCAGCAGCGTCTTNCAANACTCTAAACGCACACAGCATCNCRRGCNAATACTTCTCCAAACTCCTGATCNCCGGTGANCACTAGGTGCCNNNTCCTGGATAACCGAAACGGGCCTCCNRAGCTCGCNNTCTTACAAGACYCNNGAYAGAATGCGGCGTTTTATYCATGTNANYTCCCAGCGTCGGNGACGACGCACGACNGCNGGTTYRTGARCNGCACCANCGGGCCTGCAGNGNRGGAGACGYGNYATCNTANCGTGTGTGTCGACYTGTNTCAGYCGRGGAAANNNNNCCGCGRNCACNNGACCNYNTACYCCGCATGGACRCNGGNCYNAAGCGAGGGTGNCTACAYCRATNCCGGGNNNNAGAATCGAACANCTCCTTATANCYNGGTNNNCCCCCGAYCYGYGNATAANGTTANCACGCTTACNNNNATGGGACCCACNNNNATTRCGAGGATNNNNNNNNCTCNRNTGNTCCGTYACAGCGGTATNAAANYCTCRCGNCCTYTATNNGAGNCNNGGTGAAACATTAAGGTGCCGYYGGACCATCCGGCACGCATCACGTGTRYNNTAGTCNCTANNNNNGNNNNANGRGTTCGGGYRYNNNNGTYTTNNNCTCGAGTTTNNNNGTYRNCCATACAGCTNAAYNGACRNNGGCAGCTCTCNANCGNTAGCCCANCCCNCTAGATGYNACTCCAGGNGACGCRAAAGCNTCAAGNTACRAYCNNNNTNGTRTTGGTNGGACGANANNRGTAATATAATCCCTATTTCGCAGGANTRGTNCGTGCCGNNNGAGNYNTNNNCAANGANNGNRNCCGCGAGATGCGCTACNTGTCCTNTGTNNNTNTNCATTGCATAANNNRATCNNNGCTGCNTCATCNYGACCGTACGCAGNAGAYCTTAGARCCNNANTCGCCGGCTNCCNNNACRNNAGNCTCGYNCRGCCTGNGCTAYTGTATCTAARTNGACTGTTCTCATTGTAGTTGRCGGGCTCCTNTYANTGTCGTAGCTATAACNNNNAGGNARTTTGANNAATCNNNCAYGCGGTNAACCGTAGGNYGRGGCTACACAGCCTAANGGRCYATYCTAYTGNNGATGAGTYGCGNAGTCTCNNYCTRRATCGGGTTCATCNTRRGCNNANCGGNCCGCAGGNGGCGTGCCTGATYGNNACCGNNNNNGGNNCCTNCGATCTACGNNNNNNNGCCCGGTCGCCNTGGYCGTTCCGTGTNACTGNTCCTRGGANNTGGANGCCCTNNNNNNNNCNCCTGAATAGGTTGTNNNNCGCCACACGRCGGCATAGNACGCCNNNGGTTCCTTNNCTNAGNNGNNNNGTTTNCTCGTNNTGCCCCYRRGYAATCATGCGTAACNNNNNNGGRTCATGGCNNCNCGAATCNGNTGCGGNNNCTGNTCCCANNAATNNNTGTNANNNNYGCGTACGTCGAATGCACCGYGGCGTTGCTNNNNNNNRCTNGCGACCGTCATNGTGTTCNNGGTGTTGTGAGCTYTNCTTARGCGCGGACNRAAANCRCCNCNNTCATGCGTNRNAGGCGCRAATGTGNNNNAARCGTTAACACGGANYTTGAAAGYGCGAATNNTACGGCNCANTYCTATACAGGTCTNRGTANNGCYNNNNACTGGCTRTACCCYYGRGNNACYGCCGTTGCGCAAATTCACCGGGGGGGAGCCAACACTCTGTTRNNGYGGGCTCNCNNGNNNNYTAAATTGTGGCGTYNCCCTNATCGANRGAGANTTNTGYTGYCTCRNAGGNNGCCGGCATGGTGTCGCTTCACTCTTNATANGTNTYGCAATGYNACGANNCCAGGCGYCRTATRANATANARATNACAAAACTAGATANGCNNTACGNNNYNNNNNAGCYANYNCGNRCCCTGANNNNNNTTCGNRACAACACARNNTCRGTYNNNGACNNNANCCGGGTGCAGACNYCTCNGTCAGCTGGGATTNCTAGCCGAATARGNCNAGNGYCGAGCCAACCCGARYCTAGCGNNNNNNNNACAANNCGAAGRGCRCCACACAYCTATATNTCNCTCAGGGCGGCAGCGCCAAAAYNNTCAGTTCCCACCTNNGGTCANCCCNGNRAYCNANGGTAAGCCCNACATGCNATTGGGNGGCYGRATYYNRATGTCCGATCYTATTCCTCGAACCGACCGTAAAGNACGNGAAGAATTYGTTANCTTNNNNCGCTRGANNNCNTGTYGGYANNNNGCTGCCTCNNNCTAATGGCACTYCCAAGGTTTRCCTCTNAGCNGYNNNAGTTAGTTGGCCCACCCYCAATATGANANAACATACAGYANRCCGNNNNARGGCACTNNCCAGCATTTGCATCCACATRCGTCNCTGTCTTTNNNNNGNCTGGYTGGCGGAATAGAAGATCTCGATCTGTAATGAATAGCTGTCACAGANRYTRCCGGCTGTNNTATRGCGCGGRYTGTGCCCAGANTGTCCTCCAAGNNGCNRATNGACCCTNACTTAGGGNGTYGGGANATAANNGYANTCGCCNCANGNCCNAGGNNNNNGGGATCCTGATCGCYNGNNCCCACCTGNYAAAGNNACCGCNRCGTCTTCNNNNCGACTGGCTAGCNTGAACCCTAGATAACNNNNNTNTTAAGNNAYNCTRGNRGGGCAACCTTNNNNTGTNNACATGATCCTTAGANNGNYTYGTGGCATGNNGGCANAGCCCCNRGGCNNNGACCTNNNNCGTCCGCNAACNGGCCRGTTNGNNCAGAGGNCGACANNNNNNNAACNNAACNNRGCTNCANGRNGNNNACYGAGCTATTTTAGCAGGAGCCGNNGTGCNNRCCCGTACCANNNNNNTGAGGCTTATCTGTCRGTAARGYCATGGCGCCAACTAGAGANYGCNNGGTACTTTGTANAAATGNNCTNNNCGNGAGCATTNGANYGTNCGGNNNNNNNATCAGCCATTNNNYAGNNATTGCCYNYGYACGGCANCNNNNNGNTGTCRCGACCTCGGGNCGGTATGTGGCANACYTTTTANATCCGACNCGATTACCCAAGATGGAGGCCCCAGCATCCTTACNCTGNNATTTAYCGTANNGACGATCGRGCAAAGCRYNTCCNGCTRNNNNNNTCCGTYGRTGNCCRGTTCANGTGATCAGATTTTGTNNNYRGTANNNTNNANTGGCGGANTTTCGNANTCNGTCYNNNNNNNNNTCGCANTGNNCATT

BrK06-1 RNGRYTNRCTCGTCACYATNNNGGCCGCCGGAGTCGTCGACCTAGRCGGNNCYNGGNGNTTTACCCACTGACCATRATCTACNGGCCANNNCGCCTNTTGTCTARGGRGCCNNNNGCCCGGGATCCNTCTTGGGTATGNNCYATGNTGNGNCNNNNNTGNNNNGGCAGCGTNNNGGAGAGCCGNNNGTANNRACAGGTTTGGACCNGGCTTTGGAANNNTANNNNTYACAATACGGCANNNTCCAARNNNGTTNGRCACGAACANCNTAGGYCNTGTAAGAAGGTTTTGAACAAAYGCGGATCACNCGTTGGGCATTGACNNYTYCCAGTNCCCGNGGATAGYCCGGTTGATGRYNTAGCTTGRNNARCRGTGGAGCCATTCTGGNGCTGCTANNNNNNNNCGRGCGCCGCAGGYYNGGNNCCNNRGTRCNTTACANTGCYGNNNNNNGRTYCCAAANNNCCCTCTCTTNNCTGCTGAAYGGNCTGCRTTNGGTTACCTTTGGCTAYNAAYGANCCCTAGCGNANACAGCTANNTATCCTNGTNTNNNNTNNNGTNAGAGTNNRGANCCGACGCGTAYGRTNNCANCACYRACGGGGCNAGGNNNNAACGYCTCCGGNNNCCANNGGTGGACNTNCCCCTARNNNNAANCGACNGCTNNNNNTGAGCAGRNCNRTCTCCTNNATCTGCNNNNNTRTNYGCNNNCCCCTGCCGCCGCTTACGCCNNNNNCTGGTNNANTGCGCCGTGCGGTGGGTANCAAGTGGCTCCNNNYNCATNCGAATGGYNGGCNNNACCNNGGACTRGCCGGTTGGTAGCTAGGNNNNNNNNTTATGTGARTRCRNRCCCCGCNNGCACTACAGATACAGCTNNCNCNNNACACAANNGAATTGTTCTTAGGCTYGNGAATACGGATAAGTTCGGAAANNNGNGNGCYGNGRNCCRGYCNGYCACNNNNNTTNYRANTNGNNNNAACTNGCNRYANCCATGGCTCNTAANNNCGGAATAGCCCGCGGTGTAAAGNNNGGCCGCNNCGGCGAGNNNCGNNTNNCTNCGAGNGATAATNAGACGATTAGATTATGTCANNNNNAANGCTCTCCTCTTANNNNNNNNNACNNNNNNNGGCTNCNNTTTYGGCGGCCGGGGGATTAGGCANNNYCGGGGGNNNCTRAGTNNTAGGGNCTAGRAACRNGAATGCCCARANGATGGGGCCAGCCACACCGACAANTTNTCGCAGRGGNNRYCAGNNNGNTTGNNNCRRNNNATTTNTTTGGCCCNNCGNNAACGCGAYGGTATTTGAAAAGACATANNNGTGNRATNNNNCTCCAGANNNNNCRNTTAGGAATNNACTAGCACTNNNNNNTNNNNRNCNNGCGCNTNGGTATCTCTCCGGGCGGCCRRNGCNNNYGNNRYGGGCTNGCCCNNGNNCANNGGARGNGAAAAAYYNCGGGANNCTNNNNACTCNNNGACCCTARTGNNCATAGNNNNTCGNNNAGTTTNRCATANNNNNNCTGGGAGCAGNNNTCCTCTGACTTACCCAGTGGCTTNCGCAGGAYNYTRYNNNRNNNNNNNNNNRRCCCNCTGCACCTNCCAAGYGGTTTTTCACANACCCTAYCYAGGTNNNTCCGAYGGAATTGCTNNRYTAGTGCTGYTGTNANCCCTATATCTRTTAACCNGGTGGGCACTGGTCTCNGACTYGTAGTNNTCGATANRANNNNGGRNANNNCNNNGTGCAAGNGGNNCATNTTAGNCTNCTNCATTGNNANGGCAYRRTNTGTTTGGNTNNCGTCCGCTTGGTTANNNNCYCGANNCCCGTCCCCNNNNNTCCCGANGAGTGACNGTGAGGCCGCGGNTTCNNGCTGGACANTTTCNAAAGNGNNCAGAGGATNNNTCGNNCNNNNNTCATNNNNNNNNNNNAATTTNNCAATGTNNCGGNNGNNNNGAGCTNGTRCCGCANTGRGNNTCTTGCAGANNGGCTCAAGCNTCGGCGGCNCGTCAYNATCNNGCNNYTGGCANGGCCGCNNNNNNNNNNNGTNNNNYCYAATNNNTCGYGTNNNNNCGAGGYAGNNNNGACGAATACGGAGACGCTATGACGGGGTTTAYNCACNYTCCACNCNGGCCAGTCGTTACGCAAGGRCGGTTCGGGCNNNNNNRCCGGTANNNNGCGTANNNNNAGTGNNNGCRNATACCTGAGAAGTACTCGACCGCTANTCGGNNNNNCGTGGYNNNGGCGCCTNNNTTCTANAYTAANRTNGGCNNCGTAGATGCANGCNGNANNTNGNNNNRRGANAGNGCGAATYNGYGRCARCRGGCTCCGCGACNANNNNNNTTANNNAANNCGTCAAAGAATNNTTGNNNNNGTCCGCCCNCNCGNNGCGAAGCGNCTNAANRNNCCRCTACGCNNAAGCCTNNCRAGTNRNNNNCAATCTCCNGCNNNNNYAGCNCGNTCGCGNYAGCNGRTTGCGAGNNATYCNAGGANTARCACNACNNCTNNNAACGGCCGGNANNNGAAGCCYGCTGNNNGGTTACNNNTTNATTAGCTCGCNCCGGANNRNNNGTCCTCNNNNNNNGGGGCTACTYYRGNNNNNTANNNNNNATNANNNNCACGCAGCANCNNNNNNNNNTTNNAAGACTNNNNNCGCCNACANNNNCTNNNNCTNNNNCTNCTNCNCANTCNYGATCACCGNTNNNNNNNAGGYNNYGGANCNTGGATACYCGNNNYGRGCTTTCTGNNNTCGCCNNCTNACNAGACYCCCGAYAGNNTGCGGCGCTTTCTCNNNGYCAACNCTGNGCGTNGGTGACRNTACNCNACCRCCRGYTNRTGARCCGCACNACCGGGNNTNCAGNGGANNNGACGNNNNATCTTANCGTNNNNGTCGACYTGNATNAGTCGGGGAAATGGAACNNCNRNCACGCGACCATNTACNNNNNNTGGACGCYRNYCTCAAGYNNGGGTGTCTNNANNGATCCCGNNTCTGAAAGTNNAANANCTNNNTCTAGCTGGGTTACCCCCCGAYCYGYGCANNNAGTTAGCACGCTTANNGTAATGGGACCCACNNNNNTTRNGARGATNNNNNNNACNNNNTTGCNCYRTCACAGNGGTATTAANGCCTCACGTCCATTATGGGAGACGGNGTGNAACANTNRRGYNCCGCTGGACNATCCGNNNNGCNTCACGTGTGTAATAGTCNCTGGGTATGTGAGNGGAGTTCGGGCACNNNTGTTTTTNNCTCGAGTTTNNNNGTNNNCNAYCCRNNNNAACAGACRNNGNCAGNNNNCAANNNGTRGCYYNTNCCNCTAANNGYNACTCNNNNNNACGCGRAAGCCTCAANGTRTNAYNNNNNNCGTRTTGGTNGGACGAGRNNRGTANNACNANNCNNANNTNNCANNNNTGGTCCRTGCNGGNNGNNNNNTCACCANGGANAGTRNCCNNNNNATGCGCTACNTGTCCNTTGTTAGTCTACATTGNNNNNTANNNNCGTCGCTGNCTCRTCCCNNNNNNACGCAGAANAYCYYAGAACYTTAANCGCCGGCTNNNNCGACGCCAGCCTCGNNNNGCCTGAGCTACTGTATCTAAATNGANTNNNYYCNTTGNNGNNNACAGNNNCCTTTTATNCTCGTCGCTATAACNCACAGGCAATTTGAGAAANYATACAYGCGGNAANNNNNNRGGCTAGGCTACACANNNTGAGRGGCCNGCCTNNTGNNGATNNNTNGCGCNRTYTCGGNNNNNNTCGGGTYNNNNGTGNRCNNACCGNTCCGCAGGCGGCCNNTYYGATCGGAACCGCNNNNGGNNCCTGCGAYCNACNCGNNNNNGCCCNGGTGCCGTGGNCGTNNNGYNNARCTNNNCYNRGNANNNGGATGCCCTNNNNAGCACCNCTGAATAGRNTGTNNNNCGCCACACGACGGCATAGGACGCYAGCGGCACCTTNNCTYAGNNNNCAAGTTTNCTNNNNNTGYYNCCGGGNNNTCATNCNNNNAAGGATGRGRTNATGGCAGCTCGNNNCANNNGCRRNNNCTGNNNNNRAAAANYGCNNTCAATTNCGNNYACGTCGAATNNNNNNCGGCRTNNNTTACGGRTNNNNGCGGCYNTCATAGTGTTCATGGTNTTRNNAGCTNNNCTTAGGCGCGGACCNAANNCGCCACGGTTATGNGTARNARGCNYRAATNTGAGCAAANCGTTAGCACGGANYYTGNANGCNCRGNNATTACGGCNCATTCCNNNANAGGTNTAGGTACGNCYTCACNNNNNCTGTACCCYYRGGGGACCGCCGTTGCGCAAATTCACCGNNGGGGANNCAACACTNNNTTRNNNNGGGCTCNCGNGNNNNNNNRAYAGTGGCGTCNCCYYAAYCRAANNAGAATTCTGYTNNNNNRGRGGNNGCCGGCATGGTNNNNCTTCACNCTTNATACGTNTYGCANTGCAACGANNNNNNRCGYYGTATAACAGAAAGATGACRAANNNANNNNNNNNGTNYGGGACGACGTNNNNNNNNCRNACCCTNNYNGTGTTTCGTANNNACNNNNCATCRGTYTCTGANTTNANCCGGGTGCAGACNCCNCAGNNNNNAGGGATTAYTANNNNNNTNNGCCNNGTGNCGRNNNNACCCGAGTCTAGCGNGTCRYNNACAACCNGAARRGCRCCNCNNATCTGCANTTNCCTCRGGGCGGCAGCGCCNNNNNNNTCNGTNCCCACCTNNGGTNNRCCCTGNNNNCTANGNTAARCCCNATGAGCGATTGGGAGGNNNNNNCCGGAYGTCCGRTNNTATTCCTCGAACCGACCRTAAAGNACGNGAAGAATTNGTTAACNNNACCNNNNNGANCRCRTGTYGGYNCACAGCTNNNNNAGTCTNNNGGNRYYYCCANNNNNNRCCTCYNRNNGRCAGGNNYYNGTTGGNCCNNCCNCAATNNNNNANAAYNTACAGNAAGCCGNNNNARGGCNNNCGCCAGCATTTGCATCCACATGCGTCNCNRTCTTTTNCATGNCTGGCNNNNNGANCGGARGTNNTCGATCTGTNATGRNTAGCNGTCACRGATGTAGCCGGCTGTNNTGTNNNNNNGACTGNNNCCAGATTGTCNNNNNNGTGNNAGATNGRYCYTNNCYNNNGGCGTYGNGACATANNCGNAANCGNNNCAGGTNNNAGGAACGTGGGATCCTGATCGYYNGNNCCCACNNNTCANAGTTACNNNTNCGTCTTCNCNRCGACNGGNNNGCTCGAACCCTAGATAACGAGGGTGTNNAGGGACTCTRNNGGGGCATNCTTGGRNNNNNCGTRTGATCCNNARACCGCTTYGNGNCNTGRRGGCAGAGCCCCTNGGCTGAGNCCTAGGCCGTCCRCAAACTNGYGAGTTGGRCNNNAGGCCGANAGGGGCGCAACGTAACATAGCNTNNCNNAGNNNNCNNNNNNNNNYTAGCAGGAGCNRNNNTNCNNACCCNTACCATTCGCCTGAGGCTTATCTGTCRGTANARYNNTGGCGCCAACTGRNGANYRCCTGGTNNNYTNTNTAAATGCCNNNNNNNNRAGCATYGNNTCGYNNGGTNCCGAGCTATGCCATTRRYYAGRYATTGCCYNYGYACGGCNACGGNCCGATGTCRNNNNNNCGAGNYRGTATGTGGCANACNTTNTAGNNNNNNNTCGATTGCCCGNNANNGANNCCCCAGCATCCTNACGCTGNNANNTATCNTRRANNCNATCGGGCAAAGCGYNNCCNNNNNGCGGAGTCCGTTGATGACCGAANNNNNNGACCAGATTTNGNACCCGNNNNYYTACANTGGAGGGNCATNNNNTTCAGTCYNACGCGGTNNNGCANNNCGCANN

BrK47-1 GTGATTTACTCGTCACCATNNNGGCCGCCGGAGTCRTCGACCTAGACGGGNCTAGGAGGTTTACCCACTNAYYGYGANNTNYNGGCYATAAYGCCTYTTGTCTARGGAGTCNNNNGCCCGGGATCCRTCTTGGGTATRGNCYATGNNNNNCNNANAATGGCCAGGCAGCRCGCAGGAGNNCCGNANGTANNRRNAGGNTTGGACCTGGCTTNGGRACGRTAGACTACACAATACGGCAGTCTNYAARTANGTTNGNCACGAANATCNTAGGNCTCATGAGGAGGTTTTNRACRATCGTGGATCACTCGYNGGTCATTGACYATTYCCARTGCCCGTGGATAGYYCGGTTGANGGTGTAGCTTGNNGARCRGTGGAGCCATTCTGGAGNTGYNNGNTTGGGGCGGRYGNCGCAGGYYNGGAAYCTNRGTAYATTACANTGYCGCCAGCTAGCCTYNAAACGCCTTCTCTCNNCTGYTGARTGGGCTGCATTTGGTTACCTTTGGCTGYCAAYGARCCCTANCRNANACAGCTAANYATCCTCGTCTAGACTNRNGTRAGAGTCNRGARCCGACGCGTACGATTNCAACACCNACGGGGCRAGGANAGAACNNCTCCGGCTACCAACGGTGGANATNCCCCTAGAAACAAGCGGTCGCTRAGNNTGAGCANGCCAATCTCNTCNATCTGNTCCYYTRTGYGCAAGCCCCYRYCNCCGCTTACGACAGTACCTRGYATACTNCGCCGTGYRNNGGGTACCARGNGNNTCCTANYNCATGNGAATGGYAGGCACTACCGTAATCTAGCCGGTTGGYNGCTAGGAACTGATATTATGTGARTRCRNNCCCCGYYYGCAGTACAGANACNGCTAGCCCTGTACACAACGGAATTGTTCTTAGGCTTGNGAATTTGACTGAGTTCGGAAAAANGGGNGNYGNGRNCCRGYCNGYCACNAAAGTTGNNANTNGNAGTANNTCGNGNTANCCATGGCNYCTAANTTNGGRNTARCCCRCGGNGYNGGGNCGACCCGCGTCGGCGAGRRCCGCTTTGCYGCRAGNGATAATCAGACRATTNGATTATGTCARAAGGAACGYACNCYTCTTRNCAANGGTCACACRYNGTGGCTNCNCTTTYGRCGGCCGGGGNNNTAGGCACGTCCGGGGGCCGCTGACTACTNGGGTCTAGGAATATGAATGCCCANACGATGGGGCCAGCCACACCGNNNNRTTCTCGCAGGGGGCNNNAGTTNGYTTGCTGCNNRGNATTTNTNTGGCCCGACGGTAACGCGNYRGYNYYYGAAAAGACATACGCGYGNNATCAGACGCCNGRAATGTCGATTAGGAATNNACTAGYACTGACGTANANNNRNCNNGCGCATCGGTANCTCTCCGGGCGGCCGACGYGAATGNNATNGGCTCGCCCCTGGACAANNNNRGGGAAAAAYCYCRRGACYCTGARNACTYNNCGACCCTAATGAACATAGAGCNTCGNCNAGTTTAGCATATGGTCTCTGGGAGCANCCGNNNNNNGACNNACCCAGTGGCTTCCGCAGGACNYTRYACAGYCANNAAGYGAACCYCCYGCACCTCCCAAGNGGTTTTTNANRTAGCCTNCCTAGGTCNYTCNNACGGAATTGCTCYRYTAGTGCNRTYGNNNGCCCTATATCTGTTAACCGGGTGGGCACTGGTCYCGRACTYGTAGTGCTCGATATRANNTCGGGGATCACTATNTNCAGGTGGACTGTATTAGTCTGCTTCATTNAGATGGCAYRRTCTGTTTGGNTATCRTCCGCTTGNTTATTGGCYCGAAGCCCGTNCYCACGAGTCCCGAAGAGTGACGGTGRGNCCGNGGTTTCTCGCTGGACACTTTCCAAAGCGTCCAGAGGATACTTCGNNCTCTTTTCATTTNNGNNNNCCAATTTCGYNATGTNNCGGCANCGYAGAGCTGGCACCGCARTGGGTGTCTTGCNNAACGGCTCAAGYAGCAGCGGNGCGTCAYNATCRNGCCCYTGGCATGGCNGCGACAGCNNGCCGNCCCACYCRATGGNNCGCGTNNNNNCGAGGNANGCGGGATGAANACGGAGACGCTATGACGGGGTNNNNNNNNGYTCCACRCNGTTCAGYNRTTRCGCAAGGRCGGTNCGGGCCCCCAGRCCGGTATNGGNNGTAATTGGRGTGNNNGCRAATACCTGAGGAATACCCAGTTGCTAATCGGCGCATNGTGNNNANGGCGCCTCGCTTNNRTAYTAANRTNGGCNACGTAGATGCATRCNGNNNNNYGCGGCAAGANRGAGCGARNNCGNGGYRRYGGGCTCTGCGACCACATTAGTNACTTAATGCGTCARAGAATGATTGTTCGTNNCCNYCCCYGCGGAGCGAAGNRNCTNAAARGCCCACTACGCAAAAGCCTGTCAAGTAAGGCCCAAYNTCCGGCGCACNYRGNGNNGTCGCGCCAGCCGRTTGCGAGNNNTYCNAGGACTARCACNANTNCNAGAAACGGCCGGYACAGGAAGCCTGCTGGTRGGTTACGACTTAANNNGCCCGCNCCGGANNRATCGTCCTYGTCGTTAGGGGCTACTCTAGTGGCGNACATCGCATNATACCYACGCAGCAGCRGCAGCGTYTTRCAANACNCTARACGCCCACANCNTCTCGGGCTAATACTTCTCCTCACTCTCGRNCACCGNTNNNNNCTAGRCNNYNGNNCNTGGATAACCGAAAYNGGCCTCCAAAGCNNGCCATCTTACNAGACTCNNGACAGAATGCGGCGTATTAYCCNNGTNNNNTTTGNGCGTNGGTGACRNTRCACGACCGCCGGTTTATGAACCGCACCACCGGGCTTGCAGGAGANNNNNNNNGNTATCGTARCGYRNGTGTCGACCTGCNTCAGYCGRGGAAATGRGACCGCGATCACGYGANNNNTTACCNCGCATRGACGCYNGCCTCAAGYGGGGGTGTCYACACCNATCCCRGRYCTGAAAGTCGAACATYTNNTTCNAGCYGAAANNNCCCCCNNTCCGTGCATAAAGTTACCACGCTTACCGTACTGGGACCCACCGGNATTRCGAGGATTAAAGCAACCCCGTTNNTCYRTCANNGNGGTATTNAAGCCTCACGTCCTNTATAACAGACGGRRTGAAACATTNAGGTGCCGCTGGNNCNTCYGNCCYGYRTCACGTGTRCAATAGTCNYTGGGTATGTGAGNGGAGTTCGGGYRNNNNYGTNTTNACCTCGAGTTTNCNNGTYRNCCACCCRRYNCNANAGACATTGGCAGCTCACAATCGATAGCCCANCCCNCTANAGGYNACTNCAGRNGACGCGGAAGCCTCAAGGTAYRAYCANNCNCGTRTTGNTCNGACGAGAAGRGTARTATAAYYCYYAYGTCNCAGGTTTGGTCCGTGCCGAAGGAGNYATCNCNAAGGNTAGTRNYCGCGAGCTGCGCTACATGTCCTTTGTTAGTTTGCATTGNNNAATANNNNCGTCGCTGCCTCATCCCGRCCGTACRYNNNANNCYCCAGAACTTAAATYGCCGGCTACCCCGNCGYYAGCCTCGCTCAGCCTGAGCTAYTGTATCTAANTCGACTGTNCTCATTGTNGYTGGCGGRYCNNTTTNANTCTCRYNGCTATAACACACAGGCAGTTTGAGAAANYNNNNNYGCGGCAANYYGTARGGCGAGGCTACACAGNCTGTGGGGCCATNCTACTGCAGATAGATYGCGCNNTNTCGGCCYRGATCGGGTTCATCGTGRGCGGACCGNTCCGCAGGCGNYGNNYYTGATCGNNNNNNNRNYGGGCCCCTGCGACCNACNCGYCYGCGCCCCGGCNCCGTNNYCGTTCCGNATAACTGATCYNNGNACTNAGATGCCCTTGTANNNNCCATGGANTAGRTTGTACCACGCCACACGRCGGCATAGGACGCCAGCGGNACCTTACTTYAGNNGTCAAGTTTCCTCGCATNGYYCCCRRGCAATCATNCGTAANRGGATGNGNNNATGGCANCGCGAATCNGRYGCAANCTYNNGYCCCRAAAAYNGCNGTYATNNATGCGNNNRTCGAATGCNCCGNGGCGTTGCTTRCGGGTACYNGCRGCCGNCATNGYGTTCATGGTGTNGTGAGCTCTNCTTAGGCGCGGACCAAAGTCNNCACGANCATGCGTARNNNGCGCAAATGTGTCCAACGCGNTAGCACGGACTTTGGAAGCGNGRNTATTACGGCYCAYTCCNATAYAGGTCYAGGTANGRCYTCACANNGGCTGTACCCNNNGGGGACCGCCGNNNCGCAAATTCAYNGRRGGNGANNCAAYNCTYTGTYGNNGYGGGCTCTCGNGGTNAYTAAATNGTGGCGTCNCCCTAACCRAAGNAGAATTCTGYNNNCNCRGARGNNGCCGGCATGGTNNNNCTTCACCCYYNATANGTATCGCAACACACCGAAGCCAGGCTTCGYAYNACANANARATGACRAAACAAGATACGCAGTACGGGAYGACGTAGCYANNTCGGACCCTNNTCGTGTTTTGAACCGCCACAGCNTYRGTYTCTGANTTNANCCGGGTGCAGACTCCTTAGTCAGCNGGGATTNCTAGCNGAATARGACNNGNGYCGAGCCAACCCGANNCTAGCGRGTCGYGNACNNNNCGAARGGYACCGCACAYCTRYAYTTNNCTCAGGGCGGAAGCGCCNNNACACTCARTYYCCACCTGNGGTTARCCCTRANATCTAAGGTAAGCCCYATGAGCGATTGGGANNCYGAATCCGGNCRTCCRGTCYNNNNCCTCGAACYGACCNTAAAGTACGTGAAGAATTTGTTANCTTGACCNGCTAGRCCRCRTGTTGGYNCACAGCTGCCTCAGTNYARTGGCACTTCCAAGGTTTACCNCCNAGCGGCAGGRRTTAGNNNNNNNACCCYCNNNNTGACATAAYNTACAGYAAGCCGTYRRARRRYNCTCGCCAGCATTTGCATCCACATGCGTCCCTGTNTTTCCTRNGNCTGGYYGGCGGAACGGAAGNNYTNNATCTGTRATRNNNAGCNGTCNCRGAGGTTACCGGCTGTTGTGTAGCRCGRGTTGTGCCCAGATTGTYYNNNAAGTGNYAGATNGRYCYTNRCTTRGGGNGTCGGGACAYAATCGCAANCGCCTCAGGTCYTAGGAACGTNNGATCCTGATCGCTCRATCCCNCNNNTCAARGCTACCGCNRNNNCTTCTCAGCGACNGGNTNGCNTGNACYNNANNTAACGNGGRYGTTNNNGGACTNNRNNAGGGCANCCTTGGATTGCCCNNRTGATCCTTAGACCRCTTYGCGRNRTGRRGGCRGAGCCCNTGGGCNGRGNCCNNNNNCGTCCGCAAACNGGCCAACTGGGCCNNNNNCCGACAGRGGCGCAACACAGCNNGACTNNACNRAGTRCRCCCNRCNNNTYTAGCAGGAGCCGGTGTGNNNNCCCNTACCATTCGCYTGRRGYTTRTCTGTCRGTANARCCANRRCGCCAACTGAAGANTGNNCGGTACTTTGTATAAATGCCCTTAYYGGNANCATNNNNTCGYNCGRTCCCGAGACAAATCATTRRYNAGGYATTGCCYNYGYACGGCAACNNNNNGATGTCNCGACCTCGGGNNNGTATGTGGTGGATTTTTTAGNNYCGACTCGATTACCCAAGATGGAGGCCCCAGCATCCTTACRCTGGRAYTTATTGTAAAGACGATCGAGCAAAGCGTGTCCTGCTGGCNNNNTCCGTTRRTGAYCGRANCRAGNGAYCAGATTTGRGAYYYRGCATYCTACAGTGGCGAGTTATCGCNTTCAGTCCGACGCGACCTCGCANTGCRCATT

BrK55-1 RCGATTTACTCRTCNYCNTANNGGCCGCCGGAGTCGTCGACCTAGACGGGTCTAGGAGGNNTACCCACTGACYGTGATCTACTGGCCATCNNNNNNTTTGTCTRRGGAGYCCNCTGCCCNNGATCCGTCTTNNGTATAGGCCATACTGCGCTTACAATGNNNNGGCAGCNTACAGGAGAGCCGAAAGTAACARGAGGTTTGGACCNGGNYYAGGAACGGTAGAYYNCACAATACGGCAGTCTCCAAANNGGTTGGAYANNNANATCCTAGGCCYYRTRAGNARRTTYTTGANRATCGTGGATCACTCGYNGGNCATTGACTATTYCCAATGCCCGTGGATAGTTTGGTTGATGRTGTAGCTTGRAGNNNNNTGGAGCCATTCTGGRGYTGCTANNTTGGGGCGGGCGCCGCAGGTTCGGAACCTNRGTRCATTACATTGCCGCCAGCTRRYCYYNAAGNNCCCTCTCTTTACTGCTGAACGGTCTGCGTYTNGTTACCTNNGGCYRNCAACGARCCCTANCRGANACAGCTAANTATCCTNGTTTANACTCAAGTAAGAGTNTAGAGCCRACNNNTACNRTTTCANCACCGACGGGGCGAGGNANGAACGCCTCCGGCTACCAAYGGTGGACATNNGCNYNRRAAYAAGNGACAGCTGAGNNTGAGYAGGCCAATCTCCTCRATCYNCTCCYYTRTNYGNNNGCCCCTGTCGCCGCTTAYGAGAGTACCTAGCRNACTGCGCCGTGCGGTNGGTACCAGRTNNNTCCTNTNNCATGCGAATGGTNGGCACTACCAAGGACTAGCCGGTTGGTAGCTAGGAACTGATATTATGTGAGTACATGCCCCGTNYGCANTACAGATACAGCTNGCCCTRTACACAACGGAATTGTNNNNNNGCTYGYGAATNYGRNTRAGTTCGGAAANNNNNGCGCCGTGNNCCGGTCAGTCACNAAAGTTGYRANNNGNNGTNACTAGCGGYAGCCATNNCTCNTAAATTAGGRNTARCCCRCGGNNNANNNGCGRNCCGCGTCGGCGAGNNCCGCTTTNCCGCAAGGGATAATCAGACGANYAGATTATGTCAGRAAAAANGCNCTCCTCTTANCRCANGTNAYACGCTGTGGCTCCACTTTYGRCGGCCGGGGRRTTAGGCNCGTCCGNGGGTCGCTRAGTNNYNGGGCCTAGGAACAAGAATGCACGGACGATGGGGCCAGCCACACCGRYAARTTCTCGCAGGGGGCATCAGTTNGNTTGCNNCAGRGNATTTCTTTGGCCCGACGGTAACGCGTCAGCCCTTGAAAAGACATANGYGTGNGATCGGACNCCAGAAATGTCGATTAGGAATYNACTAGCACTGACGYRTANNYRNCNNGCGYATCGGTANCTCTCCGGGCGGCCNNCGCNNNYGNNRTGGGCTCGCCCCTNNNCGAAGGAAGGGAAAAACCTCGGGACTCTGARGACTCNNCGACCCTARTGAACATAGAGCATCGNNYAGTTTGACATATGGTCTCTGGGATCAACCGNNNNNNGACTTACCCAGTGGCTTNNGCNNNNCTTTGTACAGCCAGAAAGYGRANNTCYCRCACCTCCCAAGYGGTTTTTCANRTANCCTNCCCAGGTCNNTCCGACGGAATTGCTCTGCCNNNGCNGTYGTGAGCCCTTAATCTATTAACNGGGTNNAAACTGGTCTNGRGGTCGTAGTACTCGATATRARRYYRRGGATCACTATGTGCANGTGGACTGTATTAGCCCGCTTCATTGAGATGGCACGGTCTGTTTGGATATCGTCCGCTTGGTTATTGGCYCGAAGCCCRTCCYCACGCGTCCCGAARAGTGACGNTGRGNCCGNRGNTTCTCGCTGGACANTTTCNAAAGCGTCCAGAGNATACTTCGTTCTCTTTCCATTTACNCTAGCCAATTTCGCAATGTGCCGGNNNCGTANAGCNGGNACCGCAATRNNTNTCTTGCAGAATGGYYCAAGNAGCAGCGGCGCNTCRYNANCNTGCCCYTGGCAYGGCNGCGRCAGCTAGCCGTCCCAYYYRATNNNNCGCGTCCCCACGAGGNNNGCGGGATGAAYACGGAGACGCTATGACGGGGTTTAYNCACRYTYCACRCGGTTCNGCNGTTACGCAAGGRCGGTTCGGGCYCCYARACCGATATGGGGTGTAATTGGAGTGYTCGCRAATACCTGAGNARTACYCRRYYGCTAANNNNYGCANCGTNGTNAGGGCNYCTYGCTTCTGTATTAACGTTGGCNACGYAGATGCATGYNGAACTTYGCNGNGGGARRGAGCGANNTCGCGGYRNNGGGCTCTGCGACCACATTAGTTACTTAATGCGTCAAAGATTGATYGTTCGTRYCCNYCCCCGCGGCGCGAAGCGNCNCAAARNNNCNCTACGCRAAAGCNTGTCAAGTNNNGYYCAATCTCCNGCGCACNNRGNRAGGTCGCGCCAGCCGGTTGCGARYGATYCNRGGANTAGCACTACNNCTNNNNNCGGCCGGYACRGGAAGCCTGCTGGTRGGYTANGNCTTNNTTAGCYCGCTCCGGAGCAATCNTNCTCGTTGACAGGGGNYACTNNNGYGRCATACATCGCATTAGACCYANGCAGNGRCAGCRGYGYCTTACARNACNCTRRACNCCYACAGCATCTCGGGNNAATACTTCTCCNNNCTCCTGATCACCGGTGAGCACTAGGYGCCNNNTCCTGGATACTYGAAATGGGCCTCCAAAGNTCGCCRTCTTACAAGACTCCTGATAGAATRCRGCGCTNTCTCCRYGTCAACTTTGAGCGTCGNTGACGNYGCRCGACCGCCGGTTTATGAACCGCACCACCGGRCTTGCRGGGGAGGAGACGNGNYATCTTARCGTGTGTGTCGATCCGCNTCAGTCGGGGAAATGGGANNNNNGACACRCGGCCNYNTACYGNGCATNGACGCYAGCCYCAAGYRRGGGTGTCYANAYCGATCCCGGGTCYNAAAGNCGNNNATYTNNTTCTAGCTGRRNTACCCCCCGAYCYGYNCATAAAGTTACCACGNTTACNGTANTGGNACCCACCGGGNTTGCGAGGATTANAGCNACCCCGTTGCCCYRNCACAGCGGTATTAAAGCCTCACGTCCTYTATRRNAGACGGRGTGAAACAYNCAGGTGCCGNNGGACCATCYGNCNCGCRTCACGTGTGYAATAGTCCTTAGGTATGTNNNNGGAGTTCGRGTGYCAGYGTCTTGRCCTCGAGTTTTCRRRYTAGCCACCCNGYTCAATAGRYRNAGGCAGCTNTCAAGCGGTAGYCNATCCCACTAAAGGYNACTCCAGGGGACGCGGAAGCNTCAARGTRCAATNANYNTCGTRTTGGTCGGACGANAAGAGTAATRTAATCCCTATTTNNCAGGNNTGGTCCRTGCCNNAGGAGTTTTYACCRAGGATNGTGTNCGCGAGATGCGCTACNNGTCCTTYGNCAGTCTACATTGCANAATATAATCGTCGCTGNCTYATCCCGRCCGTACGCAGAATACCCCAGAACYTAAATCGCCGGCTACCCCGACGCCAGCCTCGCTTATCAAGAGATCCCACTCCCGNNNCGACTRTYYYCATTGTAGNNNRCRGGCCCCTYTTANTCTCACCGCTATAACNCACAGRCAGTTTGAGAAATCNTACRCGCGGTAAACCGTAGGGCGAGGCTANACAGNCTGNGGGGCCATCCTATTGTGGATGRGTYNNNNNGTCTYGGCCTGGATCGGGTTCANNGTGARCGGACCGNTCCGCAGGCGGCCNNCCTGATYGGANYNNNGNCGNNCCCCTGCGATCTACNCCTCCACNNNCGGGTGCCGTGGCCGTTCCGTRTTRCTGATCCAGGGACTTRGATGCCCTTGTAAGCACNCCTGAATAGGTTGTACCACGCCACACGGCGGCANAGGACRCCAGCGGTAYCTTNNYTTRGCGATCARGTTTTCTCGNATCGCCCCCNNGCAATCATGCGTAACGGGATGGGRTYATGGCNGCNCGAATCAGGTGCGGYCTCTGGYCCCRAAAANYGCANTCATNNATGCGYRCGTCGAATGCACCRCGGCATTGCTTGCGGRTNCTNGCGACCATCATNGNGTYCATGGTGTTGTGAGCTCTCNNNNRGYGCGGACCNAAANCNCCACGANCATGCGTAGAAGGCGCRAATGTGTCCAAARCGTTAGCACGGANYTTGAAAGCGYRRATATTACGGCTCACTCYTATACAGGTNTAGGTACGRCTTCACANTGGCTGTNCCCNNGGGNGACCGCCGTTGCGCAAATTCACNGGRRGNGAGCCAACACYNNNTTNNNGCGGGCTCTCGAGGTTACTAAATNGTGGCGTCTCCCTAACCRAARNNNAATTCTGYTGTCTCGGARGACGCCGGCATGATGTCGCTTCACYCTTNATAYGTATTGCAATGCAACGAAGCCAGGCGYCGTATAACAGAAAGATGACAAANNNANNYRCGCAGTAYGGGACGACGTAGCNNCCNCGGGCCCTGATCGTGTNYCGNACYGACACNACATYGGTCTCTGGCTTTACCCNGGTGCAGACNYCTCAGTCAGCTNGGATYAYYGNCCGAATANGACNAGNGYCGARNNAACCCGANNCTAGYGNGTCGYGNACNNCCCGAAGRGCRCCACACAYCTATATNTCNCTCAGGGCGGNAGCGCCANNAYNCTCAGTTYCCACCTGNGGTCAGCCCTGAAATCTAAGGTAAGCCCCATGAGCNATTGGGAGGCYGAATTTGGAYRTCCRGTCCTATTCCTCGAACCGACCGTAAAGTACRTGAAGANNNTGTTARCTTRACCCGCTAGANNNCRTGTYGGTNCACAGCTGCCTCGGTNNAATGGNNNCTCCAAGRTTTACCACCAAGCGRCAGGRRTTAGTTGGNCCRCCNTCAANNTGANANANNNTACAGTRAGCCGNYRRARGGNACTCGCCAGCATTTGYATCCACATRNGTCNCYRTTTTTCCTAAGNCTGGCYGGCGGAACGGAAGATCTNRRTCYGTGRTGNNTAGCCGTCRCAGANGTNNCYGGCTRTTGYGTRGCGCGGNNTGTGCCYAGATTGTCCTCCAAGTGGTAGATAGACCCTNAYTTNGGGNGTCRRNAYAYAAGCGCAATCGCCTCAAGTCCTAGGAACGTGGGATCCTGATCGNTCRRYCCCACCTGTCANAGNNACNNNTACGACTTCNCNRCGACTGGCTAGCTYGAACCCTAGATAANGAGGRYGTNNRRGGACTCTAGAGGGGCATCCTTGGRNTGCCNACNTGANNCYNAGACNGCTTTGTGGCATRGRGGCAGAGCCCCTRGGCNGRGNCCNGGCCCGTCCRCAAACTNGYCANNTNGNCCAGAGGCCGACAGRGGCGCAACNNARCATRRCTYCANNNAGNACACCNNGCTATTTNNACGGGAGCCGGTGTGCATRCYCGNACCNCNNNCCTGAGGCTTATCTGTCNGTANARYCATGGNNNNAACTRGNGAGTGCCNNNTACTTTGTRTAAATGCCNNTTCCGGGAGCATTNGANCGCNCGNTNCCGAGATCAGCYATTRRYYAGGCATTGYCTTTGYRCGGCAACGGCNNGRTGTCACGAYCTCGNGTCGGTNYGTGGCAGACTTTYTAGATCCGACTCGATTACCCAAGATGGAGTCCCCAGCATCCTTNNGCTGGGATTTATTGTAAAGACGATCGGGCAAAGCACTTCCTGCTGGCGGAGTCCGTTRRTGACCGAANCRNGNGATCNNNTTTTGTACCTAGCATCCTACANTNNNNNNNNNTYRCATTCAGTCYNACNCGGNCTCGCANTNCACATT

BrK60-1 RYGATTTACTCGTCAYCNTANNGGCCGCCGGAGTCGTCGACCTAGACGGGTCTAGGRGGTTTACCCACTGAYYGYGATCTACNGGCYATCNCGCCTYTTGTCTAANGNGYCCACYGCNCGGGATCNNNCTTGGGTNYNGGYYRTGCTNCGCTTANAATGGCCNGGCAGCGCGCAGGANNNNNNNANGTANNRANAGGTTTGGACCTGGCTTTGGRACNGNNNAYYTCACAATACRGYAGTYTGTAAGTAGGTTGGGYACGNACRTCNTAGGCCTNNTRAGNNGGTTCTNNNNAANCGYGGATCACYCRYNGGNCATTGANCATTYCCAATGYCCGTGGATAGYYYGGTTGATRGYNTAGNTTGANNAACGGTGGAGCCATTNTGGRGYTGCTAGATTGGGGNNGGCGCCGCAGGYYNGGAACYTARGTAYATTACATTGCCGCCAGCTNGNNNNANARCRCCYTCTCTYNNCTGCTGAAYGGNCTGCNTTNGGTTACCTTTGGCTGCNAAYGARCCCNANCNGACACAGCTAATCATTCTCGTCNARACTCGTTTANGAGTATAGARCCGACGCRYACGGTTTNAACACCNACGGGGCGAGRAANGAACGTCTCCGGCTACCAACGGTGGACRTTCCCCTAGAAACANNNGACCGCTGAGGCTGAGYAGGCCAANCTCCTCRATCYGNTCCCTTATGTGCATACCCCCACCCCCGCTTNNGAGAGTACCTRGYATACTGCRCCGTGCGGTGGGTACCARGNGGCTCCTATTGCATGCGAATGNYNGGCACTACCNNRRNCTAGCCGGCTGGNNGCTAGGAACTGATATTATGTGAGTACATGCCCCGYYCGCAGTNCNGNNACNGCTNRCCCTGTACACAACGGRAYYGTYCNTNGGCTYGCGAATNNGNNNNARNTCGGAAAAACGRGCNNCNTGAGCCGGTCAGTCACNAAAGTTGYRANTNGNAGTARCTANCGGYAGCCRYNNCNYNTANATTNNGAANNGCCCGCGGNGYNRRGNCGAGCCGCANCAGCGAAGGCCGCCTAGCTNCRNNGNANAATCRGACRNTTNGATTATGTCNGRARRAACGCACNCCTCTTAACRNAGGYNNCACGCTGTGGCTCCACTTTTGGCNGCCGGGGGANTAGGCNCGTCCGGGGGCCGCTRAGTACYAGGGCCTAGGAACAANNNTGCNCRRACGRTNRGGCCNGCCACACCGACAAGTTCTCGCAGAGGGCGCCAGTTCGTTTGCTGCAGRGNATTTCTTTGNNCCNNCGGTAACGCGACGGTATYYNRAAAGACATAGGTRTGTRNNCRGACTCCNGAAATGTCNNTTAGNAATCCACTAGNNNNGACATACAANNRNCNNGCGCNTNGGNNTCTCTCCGGGCGGCTAGNGCNNNNGNNRYGGGCTYGCCCCTGGACAAAGGAAGGGAAAAACYTCGGGACCCTGRGGACTCGCCGACCCTAGTGAACATAGAGNATNGNCCAGTTTGACATATGGTCTCTGGGAGYAGCCGTNNNNNGACTTACCCAGTGGCTTCCGCANNNCTTTGTRYRRCCAGAAAGYGRRCCTNCTGCACCTCCCAAGNGGTTTTTCACATANCCTNCCYANGTNGYTCCGACGGAATTGCTNNRYYNGTGCNRTYGNGAGCCCTATATCTGTTAACCGGGTGGRNACTGGTYTCGANNNCGTAGTRCTCGATATRANNNNGGGGATCACTATGTGCARGTGGACYRTATTAGTCTGCTTCNTTGRRAYGRYATGGNCTGTTTGGATATCGTCCGCTTGGTTAACTGCTCGAAGCCGATCCYCACGAGNCCCGANGAGTNACGGTGAGGCCGCNGATNCTCGCTGGACANTTTCCANAGNGTCNAGNGGATACTTCGTTNTCTTTTCATTTACGCTACCCAATTTCGTAATRTNNCGGCAGCGTANAGCTGGCGCCGCARTRGGTGTCTTGCAGAATGGCTCAAGCRNCRGCGGCGNRTCRYNANCATGCCCCTGGCATGGCGGCGACAGCTAGCCGTCCYAYYYRATRRNNYACTTNCCCACGAGGYNNGCGGGACGAAYANNGAGNCGCTNTGACGGGGTYTNNACACRTYYCAYGYGGNYCAGYCGTTRYGCAAGGRCGGTNCRGRCCCCCAAACCGGTATGGGGNGTAATTGGGGTGTTCGCGAATACCTNNGNNRTACYCRRYYGCTAATNNNCGCANCGTNGTNAGGGYGCCTYGCTTCTGTATTAACRTTGGCTACGYAGATGCATGYCGAACTTTGCGGCARNAAAGAGCGARNYCRYGGYRRYGGGCTCTGCGACCACATTAGTNACCTAATGCNNNARAGAATGATYGTTCGNACCCCTCCCCGCGGCGCGAAGNRNCTNNAARNNCCGCTACGCRAAANCCTGNNRNGTNRNGYYCAAYNTCCNGCGCACNYAGCNNNGTCGCGNYAGCNGGYTGCGANNGATYCNNGGACTAGCNCNACNNCNAGAAACGGCCGGYACRGGRARCCYGCTGGTAGGYNANGNCTTAANNNGCCCGYCCCGGAGNAATCNTNCTCGTYGNNANNNNCTACTYYRGTGGCGTACATCGCAYTANACNYACGNNGCAGYNNCAGCGTYTTRCAANACGCTARACNCCCACAGCATCTCGGGCTAATACNTCTCCNNACTCCTGATCACCGGTGACCACTAGGYGCCTGTTCCTGGATACTCGRRAYGGGCCTCCAAAGCNNGCCATCTTACAAGACYCCNGAYAGNNTGCGGNGTNTTAYCCNNGTCAACTCTGAGCGTCGGTGACGNTRCACGACCGCCGGTTTATGAACCGCACYNYCGGGCYTGCAGGRNRNNNNNNNCGGYATCTTANNGCAGGTGTCGACCTGTTTYAGCCGAGRRAATGGGANCGNRGNCACGTRACCNYNTACTCNNNNTGGACGCTAGNCNCNNGCGGGGGTGTCNACAYCGATYCCRGRYCTGNAAGTCGAACANYTCCTTCTAGCTGGGTTACCCCCCGATCYGYGCATAAAGTTACCACGCNNNCAGTANTGGGACCCACCGNGTTTGCRNGAATTAAANNAACCCCGTTGCNCCGTYACAGCGNNNNNAAAGCCTCACGNCCTYTATRRNAGACGGGGTGAARCATTNAGNYGCNGYNGGACCATCCGGCNCGCRTCACGTGTGYAANAGTCNYTAGRTATGTGAGAGGAGTTCGGGCACCANYGTCNNGACCTCGAGTTTTCRRGTYRNYCACCCAGCTCCGCANNNRNNGGCAGCTCACAANCGGTAGCCCRTNCCNCTARANGYNAYTCCAGATGACGCGGAAGCCTCRAGGTAYRAYCANYNTCNNGTTGGTAGGNCGARRNNRRTNNNAYRATCCCTATTTCNCAGGAATGGTCCRTGCCGAAGGAGNYNTCACCAANGANNGTGTCCGCGAGAYRCGCTACNTGCCCTTTGTCAGTCTACATTGNNNAANNNRATCGTCGCTGCCTCATCNYGACCGTACGCACAAGNYYYYAGAACCTAAATCGYCGGCTNCCCCGACGCCAGCCTCGCTCAGCCTGAGCTACTGTATCTAAATGNACTRTTYYCATTGTAGTTGGYGGGCCCCTYTTANTNTCGTNGCNANRACNCACAGRCAGTTTGAGAAATCATACACGCGGTAAACCGTAGGGCNAGGCYACACAGNCTRAGNRGCCANCCCACTGYRGATRRATCGCGCNGTCTCGGCCTGGNTCRGGTCCATCGTGRGNGGAGCGGTCCGCAGGCGTCGYRCCTGATCGNNACCGCGNCGGGTACCTGCGACCTACNCGTCCGCGCCCGGGCTCCGTNNYCGTTCCGTRTNACTGATCCNGGGACTTRGATGCCCTTGTAAGCACCNTGGAATAGANNGNNCCACGCCACACNNNGGCAGAGGACGCCAGCGGYACCTTNNYTTAGNNGTCNNNNNNCCTCGTRNTGCCCCCRRGCRNTCATNCGTAACGGGATGRGGTYATGGCAGCNCGAATCNGGTGYGGNNNCTGNYCCCRAAAAYYGCNGTCATNNATGCGTACGTCGAATGCACCRCGGCGTTGCTNRCGGNTACYNGCGRCCNTCATAGYGTTCATGGTGTTGNGAGCTCTNCTTARGYGCGGACYGAAANCGCCNNGAYCNTGCGTAGAAGGCGCRAATGTGNNCNAAGCGNTAGCACGGANYTTGAAAGCGYGAATATTACGGCTCACTCCTATACAGGNTTAGGTACGRCYTCACANTGGCTGTACCCYYGRGNNACNGCCGTTGCGCANNTTCACNGRRGGGGAGCCAACACTCTGTTRAAGYGGGCTCNNNNGNNNACNRRAYNGTGGCGTCNCCCTAACNNNAGNAGNNTTNTGYTGTNTCGGAGGNNGCCGGCATGGTGTCGCTTCNCNCYYNATANGTNTYGCAATGYANCGANNCCAGGCGYCGYNYRACANANAGATGACGAANNNAGATACGCAGTACGGGACGACGTNNNNNCCNCGNNCCCTGATCGTGTTTNGNANCNNCACAGCATCRGTYTCTGACTCTACCCGGGTGCAGACNYCTCAGTCAGCNGGGATNNCYRGNCGAATAAGCCRAGNGYCGANNNAACCCGAGTCTAGCGGGTCRCNCACNNCCCGAAGAGCGCCACACATCTATATTTCCCTCGGGGCGGAAGCGCCNNNNNNNTCNGTTCCCACCTGAGGTCAGCCCTGAAATCTAAGGTAAGCCCYATGAGCNATTGGGAGGCNGAANYYGGNNNTCCNGTCCYATTCTCTAAACCGACCRTAAAGNACGYGAAGAATTNGTTAACTTGNYCCRNNRRRCCGCRTGTYGGYNNNNNGCTACGTCRNNCTAATGGGACTYCCAAGGTTTACCACCAAGCGGCAGGAGTTAGTTGGCCCACCCTCAATATGACATAAYNTACAGYAAGCCGNYRRARAACNCTCGCCAGCATTTGCATCCACATGCGTCACYRTCTTNTYCATGNCNRRCYGGCGGAATANNRGNTCTCGATCTGTGRNGRNTAGCNGTCGCAGAGGTTACNGGCTRTYRTRTAGCGCGGNNNNTGCCCAGANTACCCTCCAAGTNGYARRTAGACCCTNACTTAGGGNGTCNNNAYAYAANCGNAATCGNCTCARGTCNTAGGAAYGTGGGATNCTGATCGCTCGATCCCNCCTGNYAAAGYTACNGNNACGTCTTCTCAGCGACNGGNTNGCTYGAACCCTAGATAACGRGGRYGTTNRRGGACTCTRGNNGGGCANYCTTGGRNTNTNNGTANNNTCCTTAGACCGGCTTGTGGCANGGRGGCAGAGCCCCTRGGCNGNGNCCTAGGCCGTCCRCAAACTNGYCAACTNGRCCRNAGGCCGANAGGGGCGCAACRYARCNNRRCTYYRCNRAGNACACCGAGCTNTYYTAGCAGRAGCCGGTGTGCATACCCNTACCANNCGCCTGRRGCTTATCTGTCNGTAAAGTCATGGCGCCAACTRRAGAGTGCCYGGTACTTTGTATAAATGCCCTTTCCGGNNNCATTGGANCGCTCGRTCCCGAGAYNARYYATTRRYNAGGYATTGYCNNNGNACGGCANCGGNNNGNTGTCRCGAYCTCGGGTTAGTATGTGGYRGAYTTTTTANATNNNNNTCGATTGCCCGRNATGGANGCCCCAGCATCCTTACRCYGGGANTTATNTTGAGNNNGGTCGGGCAAAGCACTACCNRCNGGCGGAGNNCGTYGGTGNCCRGANCGAGTGATCAGATTTTGTNCCNNGCATYCYAYAGTNNNNNNNTTTCGCANNCAGTCCGNNNNNNNCTCGCAGTGCRCATT

BrM02-1 RCGATTTACTCGTCANCNTANNGGCCGCCRGRGTCGTCGACCTNNNNGGGTNNNNNRGGNNNACCCACTGANNNNGATCTNNNGGCYATNNNNNNNTNNNTCTGGGGANNNCACTGCCCGGGATCCNNCTTGGGTATNGGCCGTGCTGCGNNTNCAATGGCCANNCAGCGCGCAGGAGAGCCGANNGNNNNNNNANGTTTGGANNTGNNNNNNNNANNNTAGAYYNYACANNACGGCAGTCTNTRNGNNGGTTGGGNNNNNACATCCTAGGCCTTGTAAGAAGGTNNNTGANAATCGTGGATNNNTCGCAGGTCATYRNNTANNCNNNATGYCCGTGGATAGTTYNNYTNNNGNYNTAGATTGANNAACGGTGNAGCCATTCTNGAGYTGYTAGNNNGGGGCGGGCGCCGNNNNNNNNGAATCTNGGTGCANNNNATTGCCGCCAGNNNGCCTNNAAGCANNNNCTCTTTACTGYTGNGYGGNCTGCATTTGGTTACCNTTNNNTRNNAAYGAAYCCTANCNGACACGACGAANNATTCTNGTCTAAACTAGTGTGAGNNTCAGGAACCGACGNNNNNNRTTTCAACACCRACGGGGCNNNNNANGAACACCNNCRRCTACCANNGGTGGACATNCNCCTTARAAYAAGCGACNGCTGAGGCNNNNCAGGCCNNNCTCYTNNATCCNNTCCYYTRTGYNNAAGCCCCYRYCNCCGCTTACNNCARNNNCTAGCATNCYGCNNNNTGNNGTGGGTACCAAGTGGCTCCTATTGCATGCGAATGGTNNNCACTACCGTANNCTRNNCGGTTGGTAGCTANNAACNGATATNNNGYGAGTACANNNCCCGYTTGCANNNCGGNTNCNRCTAGCCCTGTAYANNNCGNNNNNNNNNNNNNNNNNNCGAATACGGANAARNTCGGAAAAACGNNNGCCGTGGTCCNGTCAGYNACNNNNNTTNYRANNNGNAGTANCTNGCGGYAGCCATGGCTCCTATATTNNGAATAGCCCGCGGNNNNGGGGCGACCCNNGTNGGCGARNGCCGCYTNGNTNCNAGGGATAATCANNCGNTTAGATTATGTCNNNNNNNNCGCACTCCTCTTAACNNAGGYNATACGCTGTGGCTCCANYNTYNRNGGCNNNNNNNTTAGGCANNNNCGNGGGNNNCTGANTACTAGGGNCTAGGAACAAGAATGCCCAGAAGATGNNNNNAGCCNNACCGACAANNNCTCGCAGGGGGCNCCNNTTCGTTTNCTGCAGNNNAYYTCTNTGGCCCGACGGTAACGCGNCNGNNNNNNAAAAGNNNTANNNNYATGATCRNACTCCAGAAATGTYRNTTNNNAATCCACTAGYACTGACRNNCANGTNNCNNGCGYATCGGTATCTCTCCGGGCGGCCRRCGCNNNTGNNRNNGGCTNNNCNCTNNNCNNNNNNRGGGNNNAACCYYRRNNCCNNNAGTACTNNNNNACCCTANTGAANATAGNNNNTCGNCCAGTTTRNCATATGGTCTCTNGGAGCAACCGTCCTCTGACTTACCCAGTGGCTTCCGCAGGATTTTGTACANNNNNNAAGCGAACCTCCNNNNNCTNCCAAGYGGTTTTTNACATACCCTACCTANGTYGYTNNNACGNNNNNNNNCYGCNNGYGCTNNNNTGANCYCTATATCTGTTAACCGGGTGGGCACTNNTYYNGGGGTCGTAGTACNCGATANRAGGTCGGRNAYYNCTATGTGCARGNRGNNYRNATTAGTCTGCTTYATTGAGATGGCAYGGTCNNTTTGGATATNGTCCGCTTGGTTATTGGCCCGAAGCCCGTCCNCNCNCNTCNNNNNGAGNNACGGTNRGNCNNNRGATTCTNNCTGGACANTTTCCAAAGCGNNNNNAGGATNNNTCGNNNTCTTTNNNNTTACGCTANCCNATTTCGYAATRTGCCNNNGGCGTNNNNCTGGNACCGCANTANGTRTCTTGCAGAACGGYTCAAGYNNNNNCRGCGCNNCNYNANCATGCCCCTGGCATGGCGGCGRCAGCTAGCCNNCNCANCYAANRRNNCNNNNCCCCACGAGGNANGCGGGACGNNTACGGAGACGCTATGACGGGGNNTATCCACAYYYCAYRYGRNNCNNCCGTTRTGNNAGNGCGGTTCGGGCCCCCNRANNNNTATGGGGCGTAATTGGGGTGTTCNNNAATACCTNNGNARTACTCGACCRCNAATNNNNGCANNGTGGNNAGGGCNYCTCRCTNCTGTATTAACNTYGGCAACGTAGATGCATNNNNNNNTNYNCRGYRRGAANNNNNNNGATCGCGGCAACGGGCTCNGCGACCACATTAGTNACYTNNTGNGTCNNAGAATGATCGTTCGTGTNNCTNNCCGCGGCGCGAAGAANCNNAAARGCNCRCTACGCAAAANCCTGTCAAGTNRNGYYCAANNTCCGGCGCACNNRGNNAGGTCGCGNNNNNNNNYTGCGARNNNTYCNRGGACTARCRCAACNNCTAGAAACRGCCGGYACRGGAAGCCYGCTGNNNGGNTNNNNNCNGATTAGCTCGNCCCGGAGNAATCNTNCTYGNNNNNNGNGNCTACTCTAGTGGCGNNCATCGCATTANACCCACGCAGNRRCRGCRGYGYYTTRCARGNNTNTNNNCGCCYACANCNTNNCGGGCTAATACTTCTCNNCANTNCTGATCACCGGNGANCACTAGGCGCCNNNTCCTGNATNNNCGAAAYGGGCYTYCTANNNTCGCCATCTTACAAGNNNNCYGATAGNNTGCGNNGTNTTAYCCRYNNCAACNNTGNNNNNNRGTGACGNNNCRCGACCGCCGGTTTATGAACCGCACCACCGGGCNTGCNNGRNRRGAGACGNGNNATCNTAGCGCAGGTNNCGACYTGNNTYAGCCGANNNNNNNNNNCCGCGATCACRCGNCCNNTNACYCNNNNNGGNCNNNRGTCYCAAGCGNGGNNNTCTACANCRATCCCGGGNCYNAAARNNNRRCATYTCCTNNNNGNTGNNNTNCCCCCCGATCYGYGCATAANNNNNCNACGCTGCCCGGANTGGGACNNACCGGGNTTGCGAGGATTANANNAACTCNRNTGCTCTANCACAGCGGTATTAAANYCTCANGTNNNTTATGGNAGACGGRRTGAAACATTAAGNYRNCGTCGGANCGTNTGTCCTNNATCNCGTGTGTAANAGTCNNTAGGTATGTGAGAGGAGTTCGGGCANNNNTGTTTTTACCTCGAGTTTTCAANNCGTCCACCCANNNNCGCAGACANNGGCAGCTCANANGCGNTAGNCYRTNCCNCTARANGYNACTCCAGGGGACGCGGAAGCATCAANRTRYRAYCNACCTCNNATTGGTCGGACGNNAAGAGTAATATAATCCCTATNNCGNAGGAATGGNNNNTGCCGNAGGAGNYATCACCAANNNNNGTGTNCGCGAGATGCGCTACNTGCCNTTTGTNNNTCTACANNGCATNNTANANNCGTCGCTGCCNCGTCNNGACCGTACGCACAAGCCTCCAGAACCYNAANCGYCGGCTACCCCGACGCCNGCCTCGCTCNGCCTGAGCTAYTGTATCTANNNCGANTNTTYYCATTNTNGYNGGCGGATCCCNNNTATYNNNNNCGCNANRNNNCACAGGCANNNNGAGAAATCATACACGCGGTAAANNGTANNGNNANNCYACACAGNCTGAGGGGCCANCCTATNNNNGATNNNNNGCNCAGACTCGCTCTAARNNNNNNNCRTCGTGARCNNANCGGTCYGCNNNCGNNNNNNCNNATCGNNNNTACGNCGGGCCCCTGCNAYYTACNCNTCNNNNNNCGGNNNNNNNNNTNGNNYCGTNNAANTGATNCNGGGNNNNNNNNNNNNNTGTAAGCACCACTNAATAGNTTGTATCACGCCACNCGNNGGCANAGGACGCCAGCGGTACCTTACTTTAGNNNNCAAGTTTNCTNNNNNNNNNCCCGGNCAANNNNGCGTAACGGGATGGGGTNNNGGYRGCGCGAATCAGGYGCGGCCTNNGGYCCCNAAAANYGCAGTCATNNANNNNTACGTNNNATNNNNNGCGRCGNTGCTTGCGGRTACYNNNNNCCNNCATNGYGTTYATGGTGTTNYGAGCTNNCNNNNRNNNNGGACNNNNNNNRCCNCGATCATGCGTAGAAGGYNCNANTGTNTCCAANGCGTTAGCACNNNNNTTGANAGYGNNNNNATTACGGCTCACNNCTATACAGGTCNAGGTANGRCCTCACANTGGNTGTACCCNNNNNNNACNNNCGTTGCGCAAATTCANGGGGNGNGARYNNNNNNNNTGTCRAAGTNNNNNNNCACGGTTACNNAATNGTGGCGTCNCCNNAATCRNAGNNNTGNNNTGNTACCNCGGAGGANNCCGGCATGNTGTCGCTTCACNCNNNATANGTNTCNNNNTGTNNCGAAGCCAGNNGCCGTNNNACANAAAGNNNNNAAANNNNGATACGCNNTACGGGACGACGTAGCYANCNNNGAYCNTNNTCGNNNTTNGTANNAACACARCNTCRGTYNNNGRCTTTANCCNGRNNNAGACGNCTCAGTCAGCAGGGNNNNNNNGCNGAATANGCCGAGNGYNGNGCCAACCCGAGTNNAGCNRGTCGYGCACNNNNCGAAGAGCGCCACACNNNNATNTNTCNCNNGGGGCGGNNNNGCNTNNAYNCTCNGTTNNNNCCTGTGGTCAGCCCTRARATCTAAGGTAAGCCCCNNNNNNNNNNGGGAGGCYGAATNNGGANNACCAATCCTATTCNNNNAACCGANNATAAAGTACGTGRRGAATTYGTTANCTTRANCCRNNRRRCNNCNTGTYGGYACACAGCTGCCTCNGTNYARTNNNACTTCCAANGTTTNNCTCCNNNCRRCAGGRRTTAGTTGGCCCRYNNCNAATNTGNYAYAAYNTACAGYRARCCGANNNAANNCNNNCNCCAGCATTTGCATCCNCNNNNNNCNCYRNCTTTNNNNNGNNNNNNNNNNNGAAYRNNNNNNNTCGATCTGTGATGAATAGCTGTCGNNNNTGTAGCCGGYNNNCATNTANCGCGGNNNNTGCCCAGATNNNNCTCCAAGTGGTAGNNNGACCCTNACTTAGGGNRTCNNGANATAANNGYAATCGCYTCANGTYCTAGGAACGTGGGATACTNNNNNCYNGACCCCACCTGNYAAAGYTACNGNNNCGTCTTCTCAGCGACTGGCTAGCTCGAACNCTNNATAACGAGGAYGTTNNNNRNCTCTRGNNNNGCNNCNNNGGRNNNCNCGTATGANYCTTAGACYGCTTTGTGGCATGNNGGCNNAGNNNNNNGGCTGAGACNNGGCCCGTCCGCNAACTNGYNANNNNRGCCNNAGGCCGACAGGGGCGCAACNNAACATGACNTCACGNNGNACACYGAGCTATTTTAGCAGGAGCCGGTGTGCATRCCCGTACCATTCGCCTGAGGCTTATCTGTCRGTANARYNNNNNCGCCAACTRNANANNGCNNGGTANNNTNTRTAAATGCCYTTTCCGGGAGNATYNGATCGTTCGGNYCCGAGNNNNNNNNNTNNNTAGANATTGNNNNNNNNNNNNNACGGCNNGATGTCACGACCTCGGNNNNNNNNNTGNNNGANTTTTNAGATNNNNNTCGATTRCCCNRNANNGARNCCCCAGCATCCTTACRCTGGGATTNANNNTRARGACGNTCGGGCAAAGNANNNCNTGCTGNNNNNNNNNNNTGATGACCGGANNNAGTGATCAGNNNTNNNANNNNGCATCCTACAATGGAGGGTCATTACATTNAGTCNNACGNNNNCTCGCANTGCANNTT

BrM03-1 GNNNNNNNCTCGTCANNNNAATGGNCGCCGGAGTCGTCGNNCTAGACGGGTCTAGGAGGTTTACCCACTNACTGTGANNNACNGGCYANNACGCCTNTTGTCTANNNNGTCNNNNGCNCGGGATCCGTCTTGGGTATGGNCCATACTGCGCTTANNNNNGCNNGGCAGCGNNCAGGAGRRNCNANNGNNNNNNNNNNNTTGGACCTGGCYYNGGRACGGTAGATCTNNCAATACNGNAGTYTNNNNNNNNGTTNGGNNNNNNNNTCCTAGGNNTCATGAGNNNNNNNNTGANRANYGYGGATCACNCNYNGGNCATYRNCNANTYNNNATGNCCGTGGATAGCCCGGTTGATRRNNTAGAYTNAAGAACGGTGGAGCNRTTCTRGAGYTGNNRGNTTGGNNCGGGCGCNNCAGGTTCGGAANNNNRGTAYATNNNATNGCCGCCAGCTAGCCTTCNARCACCCTCTCTTNNNNNNNNNNNNNNCTGCNNNTGGNNNNCTNNNGCNNYNNNYGAAYNNNANCRGANACRGCNAANNATYCTCGTCTANACTCGTTTANNAGTNTAGAGCCGACGNNTACGGTNTYAACACCRACGGGGCGAGGANAGAACNNCNNCGGCTACNANNGGTGGACRNTCCCCTNNNNNNANNTGRYCGCTGAGNNNNNNCAGNCCNNNCTCNTNNATCNGNNNCYYTRNGTGGNNNCCCCNNNNNNNGCTTAYGNNNNNNNNNGGTRNANTGCNNNNTGNNGTGNNNACNNNNTGGCTCCTNNNNCATNCGAATGGTAGGCACNACCAANGACTNGCCGGCTGGTAGCNRGGAACNGATATNNNGNGAGTACANNNCCCGTCYGCANTNCRGNTNCNGCTAGCCCTGTNNACAANNGAATTGTTCTTAGNNNNNCGAATACGNNNNAGNTCGGANNNNNNNNNGNCGTGNNNNGNTCANNCACNAAAGTTGYRANNNGNAGTANNTNGCGGYNGCCATGRCTCCTANANNANGANTANNNNNCGGTGTANNNGCGNNNNGCGTCGGCGAGRGYCGNNTNGCCGCAAGGGATANNNNGACGNTTAGATTATGTCNGRARRAACNNNNNCCNNNTRACGCAGGTAACACGCTGTGGCTCCAYTTTYGACGGCCGGGGNNTNAGGCACGTCNNNNGGYCGNTNNNNNNTNGGGNNNAGGAACAANNATGNNNNNNNGATGNGGCCAGCCACACCGACAARTTNTCGCAGGGGGCATCNNNTNNNNNGCTGCNNNNNNNNNCTATGGCCCGACGGTNNCGCGTCAGCCCTTGAAAAGNNNTANGYGTGNGNNCRGNNNNNNGAAATGTCNNTTAGGAANTGACTNNNACTGACATAYACGTRNCNNNNNNNTNGGTANNNNTCCNNNCGGCTAGCGCNNNNGNNRTNGGNNCGCNNNNNGACRAANNNNNGGAAAAGCCTCRRGACYCTGARNACTNNNNGACCCTANTGAACATAGAGCRNCGNCCAGTTTNRCATATGGTCTCTNGGATCAACCGTCCTCTGACTTACCCAGTGGCTTCCGCAGGACNNNNNACAGCCAGAAAGCGNNNCTCCNGNNNCTCCCAAGTGGTTTTTCNNNTAGCCTNCCNANGTYGNTCNNACGGAATTGCTCTGCCTGTGCTRTYGTGANCCCTNNNNCTRTTAACCGGGTGGGCNNNNNNCTCGNACTCGTAGTNCTCGNNNNRAGGTCGGNNAYYANTATNTNCAGGNNGNCYRTNTNNNNNNNCNNNNNNGAGAYGGCATGGTCTGTTTGGATATCGTCCNNNNNNNNNTTNGCYCGNNNCCCGTCCTNACGNGTCCCGAANAGTNACGGTGGGTCNNGGGNTTCTNGCTNGACANYTTCCAAAGCNTCCAGAGNATACTTCGTTCTCTTTNCATTTACNCNNNCCAATNNCGCNATGTGCCRRNRNCGYRGAGCTGNNACCGCAATNRGNNTCTTGCNNAAYNNNTNNNGYRTCGRCGGCGNRTNNNNANCNNGCCCCTGGCANNNNNGCGACAGCTAGCCGNCCCANNNNATRRCTYNNNNCCCCACGAGGNAGGCGGGATGAACNNNGAGACGNNNNNNNGNGGNTYANNCANNNNNCANNNNNTTNNNNCGTTGNGCANGGGCGGTTCGGGCNCCNANANNNNTATGGGNTGNNATTGGRGTNNTCGCRAATACNNNNGGNATACTCGACCGCNAANCGGNGCANNGTGGTNANGGCGCNNNGCTTCNGTATTAACATTNNNAACGTAGATGCATGTGGAACNNNGNNNNGGGANRRAGCNAGACCATGGNNRYGGGCTCNNNNACCACATTAGTTACTTNNTNCGTCNNAGAATGATYGTTCGTGTNNNNCNCCGCGGCGCGAAGAACCNCAAANNNCCGNNNNNNRANNNNCTRTCRNNNNNNGNNCAAYAYCNNNNNCRYGCNNNNNNNTCGYGCCAGTCNGNNGCNNNYGATYCNNNNAYTAGCRCNACTNNNNNNAACGGCCGGNACNGGRNNNNTGCTGGTANGCTNCRGCTNRANNNGCYCGYCNNNNNGNAATCNNNNNYGTCGTTAGGGGCTANNTCGGTGACANACANCGCACNATACCNNNGCAGCAGCGGNNNNNNYYTNNAAGACTCTNNNCNCNCACANCRTNNCGGGCTAATANNNNNNCTCNCNNCNNATCACCGNNGRNCANNAGNCGCCGTATCCTGGATNNNCGNNACGGGYYTTCNNNGGNNGCCATCTTACARGACYCCCGACANNNTGCGGCGTNTTANCCRYGTNNNNNYTGNNNNNNGGTNACGNNGCNCGAYCNCCGGTTTATGNACCGCNNNACCGGGTCTGCGGGNNNGGANNNNCGGTATCGTARCNNNNGTGTCGACCTGCTTNAGNNNNGNNAANNNNNCCGCGATCACRYGACCNNNTACYNNNNNNNNNCGCTAGCNNNAANNGGGGGTNTCTACAYCNATCCCRNNNNNNARARTCGAACANCTCCTTNTANCCRNNNNNNCCCCCGANCTGCGNATAAAGTTACNACGCTNNCCGTAATGGGACCCACCGGGNTTGCGANNNNTANAGCANNNNNRNTGCNCYRTCACANNNGTATTNAANYCTCRCGTCCTCNNNAACAGACGGGNNNAAACANTNAGNNNNTGTCGGACCATCCGNNNNGCATCACGTGTGCAANAGTCNYTNGGTATGTGAGANNNNNNCGGNNNCCANYGTCNNNNNCTCGAGTTTTCRRNNNNNYCANNCAGCTCAACANNNATTGGCAGCTCNCNAGCRATAGCCCGTACCNCTANANGNNACTNCNNNNGACGCGGAAGCNNNAANGTNCRAYCANYNTCGTNTTGNNNGGACNNNNAGNGTANTATAATCNNNNNTTNNCNGGNNNNNNNNNTGCCGGNNGAGNYNNYACNNNNNNNAGNRNCCGCGAGNTGCGCTANNNGYCCTTNNNNAGTYTRCNNNNNNNNNTANAANCRNCGNTGCCTCATCNNNNNNNNACNNNNNANNNNNNAGAACYTAAATNGCCGGCTACCCNNNNNCCAGCCTCGCTTANNNNNNNNNNCNNNNYYYRNNNNGRNTRTTCTCNNTGTNGYTGGYGGNNNCCTTTTANNCTCACAGCAAGGACNCACAGACNNNNNGAGAAATCATACACGCGGTAANCCGTAGGGCGAGGCYACACAGCCTGNNNGGCCANNCTAYNNNNGATRRNTCGCGNANNNNNNNCCYRGNTCAGGTCNNNNGTNRNCGGANCGGTCYGCNNNNNGCCYRNCTGATCGNNACYRCNNNGGGCCCCTGCGAYCTNNNNNTCCRCNNNNNNGCTTTGTGGNCGTTCCGTGTAACTGATNNNGGGACTTGGATGCCNTNNNNNNNNCNNNNGAATAGNTTGTNNNNCGCCNCACGACGGNNGAGGACGCCAGCGGTACCTTGTCTTAGNNRTNAAGTTTNNNCGTNTTGCCCCNNNGCAATCATGCGTAACGGGATGRGNNNATGNNNNCNNNAATCAGRTNNNNCCTYNGGYCCCRNNNNNNNNNGTNNTTTATNCGTACGTCGAATNNACCRYGGCGNTGCTTGCGGRTACYNNNNNNNNNCATNGTGTYYNNGNNGTTGNGAGCTCTCCTTAGGCGCGGACNGAARTCNCCNCGANNATGNGNNNNNGGCGCRNNNNNNAGCAAAGCGNTAGCACGGACYTTGAAAGNGNGAATNNTNCGGCNCANTCCTATANAGGTNYAGGTANGNCYTCACANTGGNNNNNCCCNNNNNNNACNGCCNYYGCGCARNTTCACGGGGGGGGAGYCAACACTYTGTTGNNGYNNNNNNNCRNGGTTACNNAATTGTGGCGTCNCNCTANNNNAAGNNNNNNNNNNNTNNCTCNNAGGANGCCGNCNTGGTGTCGCTNCNNCNNNNATAYGTATTGCAATNNNNNNAAGCCAGNNGCCNNAYNANATANAGATGACAAANNAAGATANGCNGNNNNGGANNNCGTAGCNANCNCGGACCCTNANNNTGNTTNNTAACNNNACARCATNNGTCTCTGGCTTTACCCGGGNGCNNNNNNNTYAGTCAGCNGGGATTNCYRGCCGAATAAGCCGAGTGTCGGRNNAACCYGAGTCTAGCRNNNNGYGNNNAACCCGGAGGGCACCACNNNNNNNNNNNNNACTCNNNNNNGCAGCGCCANNAYNCTCAGTTCCCACCTGAGGTYAGCCCTNAAATNTAAGGNNNNNCCCNNNNNCNATTGGGNNNCYGAATNNNNNNNTCCGNTCYTATTCNNYRANNCGACCATAAAGTACGTGAAGAATTTGTTAACNTNNNNNNNNNGRNNNNNTGTYGGYNCACANNNGCCTCAGTNNAATGGGRYTYCYAAGGTTTGYNNNNNAGCNNNNNNNNTTAGTTGGCCCACCNTCAATATGANNNAAYNTACANNNAGCCNNTAAAAGGNACTNNCCAGCATTTGCATCNNCNNNNNNCACNRTCTTNNNNANGNCTGGNTGGCGNNNNNNNAGATCNNGATNNGTNATGNNNAGCNNNNNCNGANNNNNCYGGCTGTNNTRTNGCGCGNNNNNTGCCNNNNNTACNNTCCAAGTGGTAGATAGACNNNNNCTTAGGGAGTCGGRAYACAATCGCAATCGNNNCARGTNNNARGAACGTGGGATNCTGATCGNTCGATCCCACCTGNYAAAGYTACNNNNACGNNNNNTCARNNNNTGGCTNNNNNNAACCCTAGNNNACGAGGGYGTTNNNNNNCTCTNGNNGGGCANCNNNNNNNTGYNNGYANNNNYNNNAGACCNCTTNGTGGCAGGGGGGCANAGCCCYTNNNNNNNNNNNNRGGCNNNNNNNAAACNNNNCANNTNGGNCNNAGGNCGACAGGGGCGCAACACARCATRNCTTNANNANGNACACYNNNNTATTTNNNNNNGAGCNNGNGTGCNNRCCCNTNNNNNGCGNNTGNNGCTTATCTGTCRGTANNNNCATGGCGCCAACTRRAGAGTGCCYNNTACTTTGTATAAATGCCCTTTCCGGGAGCATYGGANNGTNCNNNNNNNNNNNNNNNTATTAACNAGGCATTGTCTTTGCACGNNANCGGCYYNNNNNNNCGACCTCGGNNNNNNTCGNNNNNNANTTTTTANANCCRACTCGNNNACCCAAGANNGAGTNNCCAGCATCCTTACNCNGGGATTTATCGTGAANNNNNTNGGGCRAAGCRCTACCTACAGGCGGAGTCCGTYGRTGNCCRAANNNNNTNNNNAGATTTTGTACCTAGCATNNTACAATNNNNNNTCATNNCANNCAGTNYNNNNNGGNNTCGCAGTGNNCATT

BrM11-1 GCGATTTACTCGTCRTCTTAATNGCNGCCGGAGTCGTCGACCTANACNNGNCTAGGNGGNNTACCCACTGACNGTNATCTNNNGGCYATCNNNNNTNNTGTCTAGGGAGNCCNCTNCTCGGGATCCNTCTTGGGTNNGGGCCATRCTGCGNYTNCAATGGCCAGGCAGCRYGCAGGAGRRNCRANNGTACAGACAGGTTTGGACCAGGCYYNGNRANGNNAGAYYNCACAATACGGCAGTYTNTGTGTAGGTTGGGTNCGNANATCCTAGGCCTTGTAAGAARRTTYTNRANAANYGYGGATCACTCGYNGGTCATYRNCYATNYCCAATGYCCGTGGATAGYYNGGTTGATNNCTTAGNTTGNNGAACGGTGGAGCCANTCTGGAGYTGCTANNTTGGGGCGGGCGCCGCAGGTTCGGAACNTAAGTACATTACANTGYCGCCAGCTGGNNNYARAACGCTCTCTCTCTACTGYTGARYGGNCTGCATTTGGTNACCTTTGGCTGCNNNTGAAYCCTANCRGANACAGCTAANYNNCCNCGNNNAGACTAGTGTGAGAGTCAGGAACCAACGCATACNRTNTYAACACCGACGGGGCRAGGAANGANNNNNTCCNNCTACCANYGNNGGACATTCCCCTTAAAACNAGNGACCGCTGAGGCTGAGYANGCCAATCTCCTCNATCCGCTCCCTTATGTGCAAGCCCCTGCCGCCGCTTACRANAGTACCTAGCNNNCTGCGCCGTGCGGTRRGTANCAGGNGGCTCCTANTGCATNCGAATGGTNGGCACTACCNNNNNCTRGCCGGTTGGNNGCTAGGAACTGATNTTATGNGAGTACATGCNNNGYCYGCANTACAGANNNNNNNAGCCCTGTACACAACGGRNYYGTYCNTNGGCTTGCGAATACGGATAAGTTNGGAAANNCGRGCGYCNTGRNCCGGNNNGTCACNAAAGTTGYRANTNGNNGTAACTAGCGGTAGCCATGRCTCCTANATTCGGGCTAACCCANGGNGYNAAGGCGRGCCNCNNNNNNNNNRRCCGCYTNGCTRCANNGGATAATCAGACGATTAGATTATGTCNGNAARAACGCACNNCTCTYAACGCAGGTAACACGCTGNGGCTCCAYYTTYGRCGGCCGGGGAGTTAGGCACRTNCGRGGGTCGCTNNNNACTAGNNCCTAGGAACAAGAATGCNCRNACGATGGGGCCAGCCACNNNNACAARTTCTCGCAGGGGGCGCCAGTTNGTTTGCTGYAGNNAATTTCTTTGGCCCGACGGTAACGCGNNNNNNYYYGAAAAGAYNTNGGTRTGNRNNCAGACTCCTGAAATGTCRNTTAGGAATNNACTAGCAYTGACRYRYNNGTRNCNNGCGYNTNGGTANCTCTCCGGGCGGCCRRCGCNNNTRNNRYGGGCTYGCCCCTNGACRANGGAAGGGAAAAACYTCRRGACTCTGARNACTYGCCGACCCTARTGAANATAGAGCATCGCCCAGTTTGACANATGGTCTCTGGGANCAACCGNNNNNNGACTTACCCAGTGGCTTCCGCAGGANNNNNNACAGCCAGAANNNNAACCTCCYGCNCCTCNCAAGYGGTTTTTCACANACCCYACCYANGTNGYTCCGANGGAATTGCTCTRYCNNNGCNATCNNNNGCCCTNNATCTATTAACYNGGNNNGCACTACTCTCGAACTCGTAGTACTCGATATNAGGTCGGRNNYYACTATNNNCANGTGGACTGTATNNNNNNNCTTYATNGAGATGGCAYGGTCTGTTNGNNNNNCNTCCGCTTGNTTATTGGCYCGAANCCCRTCCTCACGNGTCCCGAANAGNNAYGGTGAGNCCGCGGATNCTCGCTGGACACTTTCCAAAGCGNCCAGAGRATACTTCGTTYTCTTTTCATTTACGCTACCCAATTTCGNAATGTGCCGGYRNCGYANAGCTGNNGCCGCAATNRGTGTCTTGCARAATGGCTCAAGNRTCGGCNGYGCRTCRYNANCATGCCCCTGGCNTGGCCGCGACRGCNNGNCGACCCACTCGATNNAGCACTTCCCCACGAGGCANGCGGGATGAACANNGAGACNYTATGACGGGGTNTATCCACATNTCAYGYNNNYCNGCCGTNNCGCAAGGNCGNTNCNGNCYCCYARACCGGTAGGGGGNGTAATTGGGGTGNTCGCRNATACCTRNGGAATACCCAGTTGYGAATCGGNNNNNCGTGNTNAGGGNNYCTCGYTTCTGTAYTAANANNGGCAACGTAGATGCATGTGGAACTTTGCAGTAAGAARGAGCGAATCCATGGCAACGGGYTCYGCGACCACATTAGTNACTTAATGTGTCAAAGANTGATNGTNCGTGTCCNYCCCCGCGGCGCGAAGAACCNCAANGNNCCRCTACGCRAAAGCNTGNCAAGTCGCGTTCNNCATCCGGCGCACNYNNNNNGGTCGCGNYAGYNRGYTGCGARYGATYCNRGGAYTAGCACTACNNCTNNNNNCGGCCGGNACRGGAAGCCCGCTGNTGGGTTACRRYTNGATTRRCCYGYCCCNGAGCNATCGTCCTCGNNNTYAGGGGCTACTTCGGTGRCRTACATCGCATTAGACCCACGNRGCANNGGCAGCGTYYTNCAAGNNTCTANACGCCCACANCGTCTCGGNCTAATACTTNTCCTCNCTCCYRRTCACCGGTGACCACTAGGTGCCTGTNNNTGNATANYCGAAANGGGCCTCCNANGNTCGCNNTCTTACAAGACYCCCGNNNGNNTGCGGCGTATTACCCGCGTCAACNYTGAGCGTCGGTGACGANNCNNNACCGCCGGTTTATGNACCGCACNNNNNNGCCTGCRGGGGAGGAGACGNNNYNNCNTAATNNRNGTNNCGACTTGTNTNAGCCGAGAGAATGGGANNNNNRNNNNGCGRYYCCTTACYNCGCAYGGACGCYGGCCYNAAGCGNGGGTGTCTACACCRATCCCGGGYCNNARRRTCGAANATYTCCTTCTAGCNGNNNTACCCCCCGATCYGYGCATAAAGTTACCACGCTGCCNGTANTGGGACCCACNNNNTTTGCNNGRATNAAAGCAACCCCGTTGCYCYRYCACANNNGTATTAAATTCTCRCGTNCTTTATGGGNNANGGRGTGAAACATTNAGNCRCYGNNGGAYCATCCNGCACGCRTCACGTGYGTAANANNNNYTNGGTATGTGAGAGGAGTTCGGGCACNNNTGTTTTTACCTCGAGTTTTCAANNCGTNCAYNCAGCTCCGCAGACRNNGGNAGCTCNCAAGCGNTRRCCCNNNCCNCTAAAGGYNAYTCNNNRNGACGCGGAAGCAYCAARGTRCAATCANNNTNGTGTTGGTCGGNNGAGAAGNNNNATATAATCCCTATTTCGCAGGANTRGTYCGTGCCGNAGGAGACTTTACNNNNNNNAGTRNYCGCGAGATGCGCTACNTGCCCTTTGTCAGTCTACANTNNANNNTATAANCGTCGCTGCCTCATCNNGACCNNACGCAGNATACCCCAGAACCNAAATCGCCGGCTACCCCGANNCCAGCCTCGCTCAGCCTGAGCTAYTGTATCTAARTCGACTRTTYYCATTGTAGTTGRCRGNNNCCTCTTATTNNYNNNGCNANNACNCACAGGCANTTTGAGAAATCATACACGCGGTAAANNNNNGGGNNNNNCCACACAGCCTGAGGGGCCAGCCTNYTGCAGATGARTCGCGCAGTCTCRGCCTGGRTCRGGTCCATCGTGNGCGGACCGGTCCGCNNNCGNCNYRNCTGATCGGAACNNCGNCGGGNNNCTGCNACCTACNCNTCCGCGCCCGGNCNYYNTGGYCGTTCCGTRNAANTGATCCNGGGACTTRGANGCCCTTGTAAGCACNNTGGAATAGRTTGTAYCACGCCACACGACGGCANAGGACGCCAGCGGNNCCTTACTTTNGCGATCAAGTTTCCTNNNNNTGCCCCYGGGCAATCATGCGTAACGGGATGRGRTYATGGCNNCGCGAATCAGGYGCGGTCTCTGNNNNNRAAAANNGCNGTNNTNNATGCGTACGTCGAATGCACCGYGGCGTTGCTNGCGGRTACCGGCGRCCRTCATAGTGTTYATGGTGTTRYGAGCTCTCCTTAAGCGCGGACNGAAANCRCNNCGAYCNTGCGTAGAAGGCGCGAATGTGTCCAAAGCGTTARCACNNNNNTTNANAGCGCGAATATNACGGCTCACTCCTATACAGGTYTAGGTACGRCTTCACANTGGCTRTACCCNNGRGNNACCGCCGTTGCGCAAATTCNNNGGGGGGGAGCCAACACTCTGTTRNNGYGGGCTCNCGAGGTNACGGRAYNNNGGCGTCNCCCTAATCRAANNAGNGTTATGTTACCACGGAGGACGCCNGCATGGTGTCGCTTCACTCYYNATATNNATTGCANTGNANNNAAGCCAGGCGTCGTATANNNGAAAGATGACRAANNNAGATACGCANTACGGGANNNCGTAGCCANCACGGRCCCTRATCNTGTTTYGNACCGNCACAACNTCGGTCTCTGANTCTACCCGGNNGCAGACTCCTCAGTCAGCNGGGATTNCTAGCCGAATAGGACGAGAGCCGAGCCAACCCGAGTCTAGCGAGTCGCGCNNTCCCCGGAGGGCACCACACATCTATATTTCACTCGGGGCGGNAGCGCCNNNAYNCNCAGTTYCCACCTNNGGTYAGCCCNGNRAYCTNNNGTAAGCCCCATGAGCGATTGGGAGGCTGAATCCGGNTGTCCGGTCCTATTCCTCGAACCGANNRTAAAGTACNTNAAGAATTYGTTAACTTGAYCCRNNRRRCCRCGTGTYGGYNCACAGCTGCCTCRGTCTAATGGCRYYTCCAAGGTTTGCCTCCAAGCNRCAGGNNTTAGTTGGNCCACCNCCNNTATGAYAYAATCTACANNNARCCGATGGAAGGCACTCGCCNGCATTTGCATCCACATNCGTCNCYNNYTTTTCCATGNCTGGCYGRCGGAAYRGARGNTCTNRRTCYGNGNTGNNTAGCYGTCGCRGANGTNRCYGGCTGTNNTGTRNCRYGGRYTGTGCCCAGATNNNCCTCCAAGTGGTAGATAGACCCTTACYTAGGGCGTYNAGANAYAATCGCNATCGNCTCARGTNCTNNGAACGTRRNNNNNTGATCGCTCRRTCCCACCTGTCAAAGYTACNGNTRCGTNTYCTCAGNNNNTGGCTNGCTNGAACCCTAGATAACGAGGAYGTTNRRGGACTCTAGAANNGCATCCTTGGRNTNYCCRYRTGANNNNNAGACCNGCTTGTGGCATGGRGGCAGAGCCCCTNNNNNGRGNCCTAGGCCGTCCGCAAACTNGYCAACTGRGCCAGAGGCCGACANNNGCNCAACRYARCNNRGCTCCACGNAGAACACTNNGCTATTTTAGCAGGAGCCGGTGTGCATACCCGTACCAYNCGCCTGAGGCTTATCTGTCRGTAAAGTCATGGCGCCAACTNNNGAGTGCNNGGTACTTTGTATAAATGCCCTNNNNNNGAGCATYNGATCGYNCGGTYCCGAGAYNARYNNCTRNNTAGGNNNTGYCTTTGCACGGCNACGGNYYGRTGTCACGACCTCGGGNCGGTNNNTGGYRGAYYTTTTAGATCCGACTCGATTRCCCRRNATGGARNCNCCAGCATCCTTACNCTGGGATTNATTNTRARGACGRTCGGGCAAAGCRCTACCTGCTRGCGGAGTCCGTYGRTGNCCRGNTCAAGTGATCAGATTTTGTANNTAGCATCCTACAATGGNGGNTTATCGCATTCAGTCNNACGNGNCNTCGCANTGCACATT

BrM17-1 NCGATTTACTCGTCNTCTTAATNNNAGCCNNNNNNGTCGNNNNNNNNNNGTCTANGNGGTTTACCCACYGANCGNGANNNACNGGCCATCNCGCCTTTNNTCTANNNNNYYCRCTNCNNNNGANNCRTNNTGGNNAYNNNCCNNANNNNNCTTACAATGGCCARRCAGCGCNNNGGANNNNNNAAAGTAACARNAGGNTTGGACCTGGCNNNNNNACGGTAGACTAYNNANNNNNNNNGTCTNNAAGTANGTTNGGNNCGNNNANNCNNNNYCTTGTAAGNNAATTTTTGACGATCGTGGATCACTCGTTGGTCATYRNNTANNCNNAATGYCCGTGGATNNNNNNNNNNNNAGCTTAGATTNNAGAACGGTGGAGCCANTCTGGAGYTGCTAGGNNNNNNCGGGCGYCGNNGGTYNGGARCCTCGGTGCRTTACATTGCCGCCAGCTRGYYYYARANCRCNCTCTCTNTACTGCTGNNNNNNCTNNGTCTGGTTACCTTTGGCNNNNNNNNNACCCTATCAGACACRGCNAANTATCNNNNNNNARACNNNNNNNAGNNTATAGAGCNNACGCACACGNNNNCAACACNRACGGGGCNAGGNANGANNRYCNNCGGNNNCNAACRNNNNNCNTTCCCCTNNNNNNANNYNNNCGCTGAGNNTGAGCAGRCCNNNNNNNNCNATCTGNTCCNNTRNGTGCATACCCCTGYCGCCGCTTACGNNAGTNNCTRGYATNCTGCNNNNNNYGGTGGGTACCAGGTGNNNNNNNNNGCRTGNGAATGGTNGGCACTACCNNRRNCTRNNCGGCTGGTAGCTANNAACTGATATNNNGTGNNNNNNTGCCCCGYYYGCANTNCRGNTACANCTANCCCTGTNNACAACGGANTTGTNCTTNGGCTTGYGANNNNNNNNNAGNTNGGAGANNNNGGCGNCNNNNNNCGNTCANNCACTAAAGTTGCAAATTNNAGTAANNNNCGGTANNNNNGGCNNNTAANTTNNGANTAGCCCGCGGTGTAAAGNCGRNCCNNNTNRGCGAAGGCCGCTYNRNCGCNNNNNATATTCAGNCGATTAGATNNNNNNNNNAGGAANNNNNNCCTCTTACNNNNNNTNANACGCTGTGGCTCCAYTTTNNRCGGCCGGGGRRTTNNGCACGTCCGGGGGCCGCYNNNNRCTNNNNYCTNNRAACRNGAATGCNCNGACGATGNGGCCANNNACACCGRYAANTTCTCGCAGGGGGCNNNAGTTNGYTTGCYNNAGNGNNNNNCTNTRNCCCATCGACNNCGCGNYRGYNYYYGAAAAGACATACGCGTNNNNNCNGNNNNNNGRAATGTNNNTTNNNAANYNACTAGNNNNGACGNNTACGTNNNAGNNNNATCGGTANNNNNNNGGGCNNCTAGCNNNNNTGAAANNNNNNYNNNNCTNNNNNNNNNNNNGGAAAAACCYYRRGANNCTGNNGNNNYNNNGACCCTAATGAANATAGAGCATCGCCCAGTTNRRCANATNNNNNNNNGGAGCARCCGTCCTCTGACNNACCCAGTGGCTTNNGCAGGACTTTGTRYRRCCAGAAAGYGAACCTCCYGNNNCTCNCAAGTGGTTTTTCNNNTAGCCTTYCYACGTYGCTNCGAYGGAATTGCTNTGCNNGYGCTRYYGNGAGCNCTATATCTATTAACNRGGTGGGCNNNNNNNTNRNACTCGTAGTRCNCGATANAAGGTCGGNNNTCANTATGTGCAANTGGACYRTNTNNNNNNNCTTNAYTNAGATGGCATGGTCTGTTNNGATATNNNNCGCTTGGTTATTGGCCCGAAGCCCGNNNNCACGCGTCCCGANNAGTNACGNTNNGNCNNNGGNTNCTNGCTGGACANCTTCYAAAGCGTCCAGAGRATACTTCGTTYNNNNNNCATTTACNCTAGCCAATNNNNNNNNNNGCCGGCNNNNNNGAGCTGNYACCGCARTARGNNTCTTGCAGNATGGYTCAAGCGTCGNNNNNNNATCGNNAACATGCCCCTGGCATGGCNGCGACAGCTAGCCGTCCCANCNAATRRAGCNCNTCCCCACGAGNNNNGCGGGAYGAAYACGNNNACGNNATGANGGGGTTTACACANGCTCCACACGGTTCAGNNGTTRNNNANGGGCGNNNNGGGCCCCCNNACCGGTATGGGNNGTAATTGGNGTGYTCGCRAATACCTRNGRARTNNYCRRYYGCTAATNNNYGCANCGTGGNNAGGGCGCNNCGCTNCTATNNNAACATNGGCNANNNNGATGCATGTNNNACTNNNCNGNNNNNAAGNGCGANNCCATGGCAACGGGTTCCGCGNNCACATTAGTNACYTNNTGCNTCANAGANTNNTYGYTCGTGTCCNYCCNNNCGGNGNNAAGNNNNNCAAANNNNCACTRCGCNAAAGCCTNTCNNNNAANGCCNAAYNTCNNNNGCACGCNGNNANGTCGCGNYAGYNNGTTGCGAACGATTCGNGGAYTAGCGNNANTNCTAGAAACGGCCGGTNCGGGAAGYCCRCTRGNAGGNNANNNNTNAATTAGCYCGCTCCGGAGNAATCNNNNNCGTNGNNNNNNNCTANNYYRGTGRCRNACATCGCACTATACCCACGCANCAGCNGCAGCGTCYTNNAANACGCTANACGCCYACAGCATNNCGGNNNAATACTTCTCCNNNNNNCNNANNACCGNNGRCNNNNAGNNGCNNNNTCCTGNANANYCGAANNNNGCTNNCTAAGCTCGCCNTCTTACAAGACNNCNGACAGAATGCGGCGNNTTATCCRTGTCNNNTYTGAGCGTCNNNGNNNNNGCACGNNNGCCGGTTTATGAACCGCACCNNNNNNCCTGCAGGGTGNNNGACGTGTTATCTTARCGCAGGTGTCGACCTGCNTCAGCTGGGAGTGNNNNNNNNNNATCACGNGACYCCTTACNCCGCATNGACGCYAGNNNCAAGCGRGGNNNTCTACRCCRNNCCCGGGCCTGNNNNNCGAACANCTNNTTNNAGNYGRRNTACCCCCCGAYCTGCGNNTAANNNNNCNAYGYTNNCCGTAATGGNACCCACCGGGNNNGCGAGGNTTANNGCAAYCCCGTTNNTNNNTCRNNGNGGTATTAAAGCCTCACGTCCTYTATNNNAGAYGGGNTNAAACAYTNAGGTGNCNNNNNACCATNYGNCNCGCNNNNCGTGTGYAATAGTCNTTRGGTATGTGGTANGRGATCGGGCACCANNGTCNNGACYNCGAGYNTTCRRGTYAGCCAYNCAGYTCNNYANNNANNGGNAGCTNNNNAGCRNTAGCCCATCNNNNTARANNYNANTNNAGNNGACGNGGAAGCANNAAAGTGCGACCNACCTCGTNNNNGTCGGACGAGAAGRGTAATRTAATCCCTATTTNGCANNNNTGGTNCGNNCNGNAGGNNNNNTCACCAANGANAGTNNYCGCGAGATGCGCTANNNGTNCTTTGTYRRNTTGCACTGCANAATATNNNNGTCGCTGNCTCGTCCCGNCCGTACGCACAAGNYYYYAGAACNYAANTCGCCGGCTAYCCCGACGCCANNCTCGYNCNGCCNGNNNNATTGTATCTAARTNNNCTGTTNNNATTGTAGTTGGCGGNNCCCTTTTATNNNYGTAGCNANRACTNNNAGNCAGNNTNNNNNNNNATACACGCGGTAAACCGTAGGGCTAGGYTACNNNGCCTGNGNGGCCANNNNATNNNNNNTGAGTTGCNCANNNNNGNYCTRRATCGGGTTCATCNNNNGCGGNNNNGTCCGTAAAAGGCGTGCCTGATCGNNNNCGCATTGNNNNCNNNCGACCTACNCGTCCGCGCCCGGGTGCCNNGGYNGNNYCGNRTNRCTGATCCNGGGACTTRGANNNNNNNNNNAGCACNANNGNNNAGNTTGTNNNNNGCCACACGGCGGNNGAGGACGCCAGCGGCACCTTRYNNTNNCGATNAAGTTTNCTCRTGTNNCCNNCANNNNNNYATGCGTAAAAGGNNNNGRTYATGGCRGCGYGNNNCAGNTGCGGYCNCTNGTCCCANNANNNNNNGTCATNNATGCGTACGTCGAATGCACCGCGGCATNNNNTGNNNNNRCYGGCGACCNNCATANNGTNNNNGGTGTTNYNAGCTYTCCTTAAGCGCNNNNNGAAATCGCCNCGAYCATGCGTARNANNCGCAAATGTNNNCAANNNNNTAGCACGGANNNNNAGNGCNCGAATNNTNNNNNNCAYTYCTATNNAGGTCTAGNNNNNGCCTCACNNNNNCTGTACCCTCGGGGGACYNCCGNCNNGCAAATTCACGGGGGGNGAGYCAACACTNTGTNRNNGNGGNNNNNNNCGGTTACTAAATNNNGGCGTCGCCCTAANNGAAGGAGAATTCTGCTRYCNCNNNNGNAGCCNGCATGRTNTCGCTTCACTCCCGATATGTATTGCANNGTNNNNAAGNNNNNCGTCNNNNRACANANAGATGACNAAACNAGATACGCRGNNNNGGACGANNNAGCYANCTCGGAYCCTGANNNYGTTTCGTANNAACACANYATCNNNNTCTGGCTTTACCCGGGTGCAGACNNCTYAGTCAGCNGGGATTACTAGCCGAATAANNCGNGNGNNGANNNANNNCGAGTCTAGCNAGNCGYGNACNNNNCGAAGRGCRCCACNNAYCTATATATCACTCAGGGCNGAAGCGCCNAAATCCTCAGTTCCCACCTNNGGTNAGNNCTRAGATCTAAGNNNNNNCCCATGAGNNATTGGGARNCNGAATNNNNANNNNNNNNNNTATTCCYYRNNNCGANNNTAAAGTACGTGAAGAATTTGTTARCTTGNNCCRNNRRRCCGNNTGTYGGYNCACANNNRNNNNANNACAATNNCACTTCCAANGTTTGCNNNNNAGCRNNNNNAGTTAGTTGGNCCRYCNNCAATATGANNNAANNTACAGYAAGCCGATAAAAGGCNCTCNCCAGCATTTNCATNCNCNNNNNNCNNNGTCNNNNNNNNGNNNNNNCGGCGNNNNNGAAGATNTCGATCTGTRATGAANAGCNGTCRCNGAGGTTACYNGCTGTTGTNNNNCGCGGRYTGTGCCNNNNNTACCCTCCAAGTNGYARATNNNNNNTNANNTAGGGNGTCNNGAYACAANCGNAATCGNCTCARGTNNNNNGAACRTNNNNNCCTGATCGCCCGNNCYCNCNNNNNNANNNNACCNCANCGNCTTCTCAGCGACTGGCTAGCTTGNACCNNNNATAACGGNNNYGTNNNNNNNCTCTNNNNGGGCANCCTTGGATTGCCNNNATGATCCTTAGACCGCTTNGTGGCAGGNNGGCNGAGCCCCNNNGCTGAGNCCTNGGCNNNCCGCAAACTNNNCAACTGGGCNRNAGGNCGNNNGGGGCGCAACNNNNNNNRRCTNNACNRNGNACACNGAGCTATTTTAGCAGGAGCNGGTGTNCATACCCGTACCACGCGCTTGAGNCYTATCTGTCNGTNNNNNNNTGGCGCCAACTRGNGAGTGCNTGGTACTTTGTATAAATGCCCTTTCCGGGAGYATYNNNNYNNTCGRTTCCGRGNNNNNNNNNNNNNYANNCATTGCCYNYGYACGGCANCNNNNNGNTGTCACGACCTNNGNNNNNNNNNNNGCAGACTTTTTANATCCGACTCGNNNRCCCNNNATGGARNCCNYAGNATCCTTACRNNNGGACTTATYGNNAARRCGAYCGGGNNNNNCGCTACCTACAGNNGGAGTCCGTNNNTGACCGGATCANGTGAYCAGATTNNNNANNCGNNNNNNTACAGTNNNNNNNTTTNNNATTNAGTNNNACGCGRYCTCGCANTGNNCATT

BrM21-1 GCGATTTACTCNTCRTCTTANNNNNNGCCRGRGTCGTCGNNCTAGACGGGTNNNNGAGGNNTACCCACTGAYCGYGANNNNNNGGCYATCACGCCNYTTGTCTANNNNGTCCRCTNCCCNNGANCCGTCTTGGGTATRGGCCNNRCTGCGNNTNCAATGGCCNGGCAGCNCGCAGGANNNNCGNNNNNNNNNNGAGGTTTGGACCTGGCTTNGGRACRGNNGANNNCACAATACGGCAGTCTGTNNNNNGGTTGGGYANNNNNATCCTAGGTCTTGTAAGNNGGTTNTNNACNANCGYGGATCACTCGCAGGNCATTGACYANTYNNAATGYCCGTGGATAGYYNNNNNGATRRCTTAGNTTGRCGAACGGTGGAGCCANTCTGGNGNNNYTAGGNNNNNNCGGGCGNNNNAGGTNNGGAAYCTCGGTGCATTACATTGCCGCCAGCTAGCCTTCAANCRCTCTCTCTYTACTGCTGAATGGGCTGCRTTTNGTTACCTTTGGCTRYNAANGAACCCTANCRGACACNGCNAATYATCCTNGTCTARACNNNNNNNAGAGTNNNGARCNNACGCGNNNNRTNNNAACACNRACNGGGCRAGGNNNNANNRYCTCCGGCTACNAACRGTGGACNTTCCCCTAGAAANRAGCGGTCGCTRAGNNTGAGCAGRCCAATCTCYTCGATCTGNTCCTCTGTCCGCANNCCCCYRCNNNNGCTTAYNNNAGTACCTGGTRNANTNNNNNNTGNNGTNNNNNCCAGGNGNNNNNTNTTGNNNNCGAATGGYNGGCACTACCNNNNNCTRGCCGGYTGGTAGCTANNAACTGANCTNNNGTGANTRCRNNNCCCGCTCGNAGTNCRGNTACANCTAGCCYTGTNNANNACGGNNNNNNYCNTNGGCTTGCGAATACGGANANNNTNGGAAANNNNRGCGCCGNNRNCCGGTCAGTNNNTRAAGTTGYRANTNNNAGYAANNNNNGGTAGCCNNGGCTCATANATTCGGGCTAACCCACGGTGTAAAGNCGRNCCRCGTNGGCGARGNCCGNNNNGCNNCAAGGGAYAATCAGNCGNTTNNNNTATGTNNGNARRAACGCNNNCCTCTTACCRNNGGTNACACRYNGTGGCTNCNCYTTYNGNGGCCGGGGAGNNAGGCACNTCCGGGGGNNNCTGACTACTNGGGNCTAGGAAYANNNATGCNCRNACNNNNGGGCCAGCCACACCGACAARTTCTCGCAGGGGGCGCCAGTTNNNNNGCTGNAGAGAATTTCNNNNNCCCNNCGGTAANNCNNNRGYNNYYGANNNNACNTANNNRTGNGATNNNACGCCNNNARYGTCNNNTNNNAATTGACNNNYACTNACGNRTACGTATCAGGCGCNNNNGTATCTCNNNGGGCGGCNAGCGCNNNTGNNNTGNNNNCGCCCCTNGANNAANNNRGGGAAAAAYYYCGGGANNCTGAGGACTCNNCGACCCTARTGNACATAGAGCGNCGNCCAGTTTGACATATGGTCTCTGGGANCAACCGTCCTCTGACNNACCCAGTGGCTTNNGCAGGANTTTGTNNNNCCAGAAAGNGNNNNTNNYGNNNCTNCCNAGTGGTTTTNCANRNACNCTANNNNNNNYGCTCCGACGGAATTGCTCTGCNNGYGCTATCRTGAGCCCTATATCTATTAACCGGGNGGRNACTRNTNTCNNACTCNTAGTRCTCGATNTAAGGTCGGRNAYYAYTATGTGCAAGNNNACNNTATNANNNTGNTNNAYTGAGNNNNNNYGGNCTGTTTGGATATCNTCCGCNNNNNNNNYNGCYCGAANCCCGNNNNCNCNNNTCCCGANNAGTGACGGTGAGGCCGCRGATTCTNNCTGGACANTTTCNAAAGCGYNCAGAGRATACTTCGTTCTCTTTNCATTTACGCTANCCAATNNCGYNATRTNNCGGTGGCGTAGAGNTGGNNCNNNNNNNGGTGTCTTGCAGAAYGGCTCAAGCGTCGGCGNNNNNTNNCTANCANGCCCCTGGCATGGCNNNGACAGCTAGCCGNCCCACYCRATGGNNCNCNTYCCCACGAGGYNNGCGGNNNGAAYACGGAGACGCTATGACGGNNTTTGTAYANGCTCCACACGGNYCAGTNNNTRCGNAAGGGCGGTTCGGGCYCCNNGACCGGTANGGGGYGTAATTGGGGTGYTCGCANATNNCTRNGGAATACCCAGTTGCTANTNNNCGCATNNNGGTNAGGGCGCNNCGCTNCTGTACTAANATTGGCNANNNNGATGCATRYNGNAYNNNGCAGTRRGAANNNNNNNGAYCRYGGCAACGGGCTCTGCGACCANNTTAGNNNCTTNNTGNNNNAAAGANTGATCGTTCGTGTCCNYNNNNNCGGCGCGAAGNNNCNNAANRNNNCRCTACGCAAAAGCNNGTCNAGTNNNGYYNAANNTCCGGCGCACGCRRNGCGGTCGNNNNNNNNNGNTNNNNRCGATTCGGGGNCTAGCNCNACTNNNNNNAACGGCCGGTACRGGNANCCTGCTGGTNNRCTTCGGCTTAATTAGCYCGCCCCGGAGNAATCGTCCTYNTCGTNANNNNCTACTTCGGTGACAAACANCGCATTAGACNYAYGCAGCAGCNGCAGCNNCCTACAANACNNNAAACGCNCACANCNTCTCGGNCTNNNNCTTCTCCTCNCTCCNNRTCACCGGTNNNNNCTAGGYGCCNNNTCCTGGNNAACCGNNACRGGCYTNCAARGNNNGCCRTCTTACAAGAYYCCNGAYAGAATGNGGCGTNTTAYCCGNGTNANNNNNNNNNNNNGGTGNNNATRCACNACCNCCGGTTTATGNACCGCNNNNNNNNNYCTGCAGGRNRNGAGACGYGNNNNNNTAGCGCAGGTGTCNNNNNNTNTYAGCCGANNNAATGGGACCGCGRNCNNRYGACCCCTNACTCCGCATNNNNNNTRGYNYCAAGCGGGGGTGNCTACACCRATCCCGGGTCYNARRRTNNNNYATYTCCTTCTAGNTGNNNNNNCCCCCGANCTGCNNNTAAAGTTACCACGCTTAYNNNNNNGGGACCCACNNNNTTTGCNNNNNNTANANNANNTCNRNTGCYCYRNCACAGCGGTATTAAANYCTYRCRTCCTTTATGGGAGNCNGGGTGANRCATTNRRNCRNCNNNNNANCRTNCTGCACGCRTCACGTGNNNAANAGTCNYTAGGTATGTGAGAGGANTTCGGGCACNNNTGNNNNNACCTCGAGTTTNNNNGTYRNCCAYNCNGCTCNAYANNNNNNGGCAGCTCNNNAGCGRTRRYCNNTNCCNCTARANNTTACTNCAGATGACGCGRAAGCNTCAAGGTANAATCANYNNNGTGTTGRTNNGNNGAGAAGRGTAATATAATCCCTATTTNNCAGGNNNNNTCCRTGCCGGNNGAGACNTYACCAANGANAGTRNCCGCGAGATGCGCTACNTGYCCTNYGNTAGTNTRCATTGCATAATATAANCNNCGNTGCCTCATCNYGACCGTANGNANAANNCYCCAGAACYYAANTCGCCGGCTACCCCGANNCCANNCTCGYACRGCCNGAGCTAYTGTATCTAAATNGRCTRTTNNCATTGTAGNNNNCRGRYCCCTYTTANTCTCRYNGCNANRACTCACAGGCARTTTGAGAAATCATACACGCGGTAAACCGTAGGGNNNGGCYACNNNRCCTGNNNRGCCATCNNAYTGCAGATGARTCGCGNANNNTCGNYCYARATCGNNNNCATCGTGRRCGGANCGGTCCGCAGGCGGCNTGNNNNATCGNNACCGCGTCGNNTACCTNCGAYNTANNCNTCCRCNNNCGGGCNYYGTGGYYGNNNNNNGNNNCTGATCCAGGGACTTRGANGCCNTNNNNNNNNCNNNNGAATAGATTGTACCACGCCACACGNCNNCANAGGACGCCAGCGGYACNNTRYNTTNNNNGTNAAGTTTNCTCGYRTTGNNCCCAAGCAATCATGCGTAACNGGATGGGGTYATGGCRGCGNNAATYANNNGCGGTCTNNNGTCCCRAARNNTGCANNCATTTATGCGTACGTCGAATGCACCRNNNCATTGCTTRCGGATRCYGGCGRCCGTNNNANNGTNCNNGGTGTNGCGAGCTCTCCTTAGGCGCGGACNGAAANCNCCNCGATCNTGCNNAGAANNNNNNAATGTNNNCANNGCGTTAGCACGGACCNNNNANGCGCGAATNNNACGGCNCANTCCTATACAGGTYTAGGTACGACCNCACNNNNNCTNTANNNCTGGGNGACYGCCGYYGCGCAAATTCACNGGGGGGGAGCNNACACNYTGTTGTCNNGGGCTCNCRNGGTNACGGRAYNGTGGCGTNGCCCTAATCRAAGGAGANTTCYGNNNNCNCNGANGANGCNGGCATGRTGTCGCTTCACCCTTNATAYGTATCNNNATGCAACGANNCYAGGCGYCNNATGATATAANNNNNNNNNNACAAGATACGCRGTACGNNNCNNCGTAGCCACCACGGNCCCTNATCGTGTNNCGNANNNACACANCATCGGTCTCTGAGTTGACCCGGGTGCNNNNNNNTYANTCAGCNGGGATNACYRGCCGAATARGACGAGAGCCRNRNNNACCCGARYCTNNNNANNCGYGNACNNNNCGRARGGCACCRCNNATCTNNANTTNNCTCAGGGCGGAAGCNNNNAAAYNNTCANTNCCCANNTGAGGTYAGCCCTNAAATCTAAGGTNAGNCCCACATGCNATTGGGAGGCCGNNNYYGGNNNNCCRRNCYTATTCCYYRANNCGANNGNNNNNTANNNGAAGAATTYGTTAACTTGAYCYRNNRRGCCGCNTGTYGGYNCACANNNGCCTCAGTCTARTNNNRYTNCYAAGGTTTNNCTCCAAGCGNNAGGRRTTAGTTGGCCCRCCNCCAATATGACATANNNTACAGCAAGCCGNNNNAAGGCACTNTCCNNNNNNNGCATCNACNNNNNNCACYRNYACTNNNNNGTNNNRYYGNCGNNNNNNNGGTTCTNNNNNNNTNATGRNNNGCCRTCRCRGANGTNNCNGGCTRTTGTNTGNNNNNGNNNNTGCCNNNNNTGTCCTCYAAGTNGNANRTANNNCYTNAYYTRGGGCGTYGAGAYATAATCGCAATCGGCNCAGGTCCTNNGAACGTGGNNNCCTGATCGCTNRAYYCCACCTGNYRNNNCTNNCGCNACGTCTTCTCAGCGACTGGCTAGCTTGAACCCTAGATAACGRGGGTGTTANNGGACTCTNNNAGGGCATCCTTNNNNTGCCCGCATGATCCTTAGACNGCTTNGTGGCATGGRGGCAGAGCCCCNNNNNNGAGNCCTRGGCNRTCCGCAAACNNGYCARYNNNNCCAGNNNCCGACANNNNNNNAACACAGCNNGNNTCNNCGGAGNRCNCNCNRCTATTYTAGCAGGAGCCGNAGNNNNNNNNCGTACCATTCGCTNNNNGCTTATCTGTCRGTAAAGTCATGGCGCCAACTNNNGANYGCNYGGTANNNNNNATAAATNCCCTTTCCGGGAGCATYNGAATGTNCNNNCCCGAGNNNNNNNNNTARYYAGACATTGTCTTTGCACGGCNACGGCYYGNTNNNNCGACCTCGGGACGGTATGTGGYRGAYNTTNTRRNTYCGACTCGATTACCCAAGATGGAGNCNNNNNCATCCTTACNCTGGGATTTATYGTRAAGACGATCGRGCAAAGCGNNNCCTGCTGGCGRNGTCCGTTGATGACCGNANCRNNNNAYCAGATTTNGNACCCGGTATNNTACANNGGCGRRTTATCGCATTCAGTCCGACGCGACCTCGCANTGCACATT

BrM22-1 RCGATTTACTCRTCRTCTTANNGGCCGCCGGAGTCGTCGACCTAGACGGGNCTAGGANGTTTACCCAYTGAYCGYAANNNACTGGCCATNACGCCTTTTGTCTAAGGAGNCCRCTNCCCGGGATCCRTCTTGGGTATRGGYYATNCTNCGCTTANAANNGCCAGGCAGCGYRCANGAGGANCANNTGTANNGANAGGTTTGGACCTGGCTTTGGAACGGTAGATCTNACAATACGGCAGTCTCYRNGTANGTTNGGCANGCAGATCCNNNNCCTCATGAGGANNTTTTNRACRATCGTGGATCACTCGTTGGGCATTGANTATTYNNAATGTCCGTGGATAGCCCGGTTGATAGCTTAGATTGAAGAACGGTGGAGCCATTCTGGAGYTGCTAGGNTRGGGCGGGCNCCGCAGGYYNNGAANNTNNNNRYAYTACANTGCNGCCAGCTAGCCTTNRAGNGCCCTCTYTTNNCTGYTGARYGGNCTGCATTTGGTTAYNTTTGGCTGNNAAYGAACCCTANCRGANACAGYTAANNNNNCTNGTNTAAACTNGNNNNAGAGTCNRNNNNCGACCCNTACTGTTTCAACACCNACGNNNNRARGAANGAACRYCTCCGGCTANNNAYGGTGGACGTNCNCCTNRRAAYRAAYGACNGCTNARGCTGAGCARRNCAATCTCCTCAATCTGNTCNTCNNNNNGCAAGCCCCTGYCGCCGCTTACGNNAGTACCTAGCGGACTGCNNNNTGCGGTGGGTANCAGGNGNNNCCTATNGYRTGCGAATGGTNGGCACNACCNNNNNCTRNNCGGNTGGTAGCTAGGNNCTGATNTTATGTGARTRCRNNNCCCNNCCGCAGTNCRGNNACNGCTAGCCCTGTACACAACGGANTTGTNNNNNNGCTTGCGAATACNGANAANNTCGGNAANNCGRGCGCNNTGNNNCGGTCAGTCACTNAAGTTGYRANNNGNAGTAACTNGCGNNAGCCATGACTCNNANATTNRGRNTARCCCRCGGTGTAAAGNCGGGCCGCGTCGGCGAGRRCCGCNYNGCCGCAAGGGATAATCACACGNTTARAYTATGTYAGRARRAACGCACTCYTCTYANCRNNGGTNACACNNNGTGGCTNCNCTTTNGRNGGCCGGGGRRTTAGNNACGTCCRGGNRNNGNNGANTACNTGGGYCTAGGAACAAGAATGCNCRNACGATGGGGCCAGCCNNACCGACAAGTTCTCGCAGGGGGCNNNAGTTGGCTTGCTGCRRNNNATTTCTNTGGCCCGACGGTAAYRCGNCAGCNYNNNNAAAGAYNTRNGYNTGTGATCGGACNCCTGRAATGTCGATTAGNAATYNACTAGNACTNACRNAYACGTATCAGGCGYNTNGGTNNCTCTCCRGGYGRCCAGNGCNNNTGNNRNGGGCTYGCCCCTGRNCRAAGGAAGGGAAAAAYYYCGGGACYCTGARNACTNGCCGACCCTARTGNNCATAGAGCNTCGNNNANTTTRRCATATNNNNNCTGGGATCAACCGTCCTCTNNNNNACCCAGTGGCTTNNNNNNNNCTTTGTACAGCCAGAAAGYGAACCTCCTGCNNCTCCCNAGTGGTTTNNCANRTACCCTACCCNNNNCGCTCCGACGRAANTGNTCTATTANNGCTATCNTGAGCCCTNNATCTRTTAACCGGGNGGGCACTRNTYTCGRACTCGTAGTACTNNATNTAAGGTCGGGGRTCAYTATGTGCARGTGGACYRTATTAGNNNNCTNNATTGAGATGGCATGGACTGTTNGGATATCGTCCGCTTGGTTATTGGCCCGAANCCCGTCCTNNCNNNNCCCGANRAGTGACGGTGAGGCCGNGGNTYCTCGCTGGACANTTTCCAAAGCGTCCAGAGGNNACTTCGTTCNNNNNCCATTTACNCTAGNNAATTTCGTAATATGCCGGYRNCGTAGAGCTGGYACCGCANTAGGTATCTTGCAGAATGGYTCAAGCRTCGGCGGCGCNNCACNATCRANNCCCTGGCATGGCNGCGACAGCTAGCCGTCCCATCNAATRRCTCGCGTCCNCACGAGGYAGGCGGGATGAACANNGAGACGCTATGACGGGGTYTAYNNNNRYTCCACACGRNYCAGNNGTTRCGCAANGRYGGTNCGGGCNNNNNNACCGGTATGGGGTGTAATTGGNGTGYTCGCRAATACNNNNGGAATACYCRRYYGCTARNCGGNGCANCGTGGTNAGGGCGCCTTGCTYCTRGAYTAACATTGGCNACGTAGATGCATRYNGNAYNTNGYAGYRGNARARRGCGARNCCATGGCAACGGGCTYYGCGACCACATTAGTNACTTNNTGYGTCARAGAATGATCGTTCGTRYNNNYNNCCGCGGCGCGAAGNRNCTAAAAGTGCCGCTACGCGAAAGCCTGTCAAGTAAGGCCCAANNTCCNGCGCACNNRGNNNNGTCGNGCCAGNCGGTTGCGAGCGANTCNGNNAYTARCACNACTNCNAGAAANNNNNNNNNNNNGAAGCCYGCTGNNGGGNTANAGNTTRATTAGCCCGCYCYGRAGCAATCGTCCTCGTYGNNNGGGGCTACTCCGGTGRCRTACATCGCATTAGACNCACGCAGNRRCNGCRGYGYCYTACARGACTCTAAACGCCCACAGCATCTCGGNCTAATACTTCTCCNNNCTCNCGATCACCGGTGGCCACTAGGTGCCNGTTCCTGGATACCTGAANNGGGCCNCCAAAGNTCGCNATCTTACAAGACTCCCGACAGAATGCGGCGTTTTATCCATGNCNNNTTTGAGCGTCGGTGRCGATGCACGACCGCCGGTTTATGNACCGCACCACCGGGYYTGCAGGRGARGAGACGCGGTATCTTAANGTGTGTGTCRACCTGTNTCAGYCGRGGAAATGGRACCGCGRNCACNTGACCATNTACTCCRCATRGACGCYGGCYNCAAGNNRGGGTGNCYACNCCNATCCCGGGTCTGARARTCGRRCANYTCCTTNTAGCTGNNNTACCCCCCGATCCNNGCATAAAGTTAGCACGCTGCCNGTAATGGNACCCACCGGGTTTGCGAGGNTTANNGCAACTCNRATGCNCYRNCACAGCGGTATTAAAGCCTCACGTCCTTTRNNNGAGNCNNGGTGAAACANTCRRGYRCCGCTGGACCRTCCNGCACGCATCACGTGNNNAANAGTCNCTRGGTATGNGRNANNNGTTCGGGCACNNNTGTTTTTACCTCGAGTTTTCAARYYRNCNNNNCAGCTCAANAGACANNGGCAGCTCANAANCGGNAGYCNATCCCNCTAAAGGYNAYTCCAGATGACGCGRAAGCNYCAANGTRYRACCANYNTNGTGTTGGTNGGACGAGAAGAGTAATANAATCCCTATTTCGCAGGNNTGGTCCGTGCCGNAGGAGNYATCACCAATGAAAGNRNCCGCGAGATGCGCTACNTGYCCTNTGTCRRTYTACATTGCATAATATAAACGTCGCTGCNNCRTCCCGRCCGTACGCANAAGNYYYYAGAACCTTAATCGCCGRCTACCCCGANNCCAGNNNNGCTYANCNNGAGNTNCNRYNYCYRNNNCGACTRTYYYCNTTGTAGTTGNCNGGCCCCNNNTANCGNNRYCGCTANRNCNCACAGGNARTTTGANNAATCATANNNNCGGTAAACCGTAGGGCGAGGCTACACAGNCTRNGGGGCCATCCTACTGNNGATRRNNCGCGCAGACTCGCTCTAAATCGGGTNCAYNGTGAGCGGACCGNTYCGCAGGCGGCGTGTCTGATCGNNACNNYRTYRRRYNCCTGCGANYTACNNNYCYRCNNNCGGNNGCCTTGGCYGYNYCGTRTNRCTGATCCNNGGACTNRGATGCCCTTGTAAGCACNNYNGAATAGGTTGTANCACGCCACACGACGGCAGAGTAYGCCNRCGGTNCCTTGTCTTAGCCGTCARGTTTCCTCGNNTTGYYCCCRRGCAATCATGCGTAAAAGGATGNGNTNATGGCAGCTCGAATNAGNTGCGGYCTYNRNTCCCRAANANNNNAGTCANTTNCGCGTACGTCGANTGCACCGCGGCATTGCTNNCGGRTRCTNGCGRCNGNCATNGYGTYCATNGTGTTNTGAGNNYTCCTTAGGCGCGGACCAAAATCGCCACNGTNATGCGTAGAAGGCGCAAATGTGTCCAAARCGNTAGCACGGANYTTGNANGCGCAGATATTACGGCNNNYTYCTATACAGGTCCAGGTAGGRCYYCACNCTGGCTRTACCCTCGGGGGACNGCCGTTGCGCAAATTCACGGGGGNGGANNYRACACYNTGTTGTCGCGGGCTCNCGNGGTTACGGRAYAGTGGCGTCGCCCTAATCRAANGAGAATTCTGNTGTCTCGGANGANGCCGGCATGNTGTCGCTTCACNCYYGATAYGTNTYGCATTGNANCGAAGCCAGGCGCCGTATAACAGNNAGATGACAAANNNAGATACGCGGTACGGGACGACGTAGCCANCTCAGACCCTGATCGTGTTTCGTANYRACACAACATCNNNNTCTGACTYTANCCGGGNGCAGACGTCTYAGTCAGCNGGGATTACTAGCCGAATAAGCCGNGTGTCGRRNNAACCCGARYCTAGCNAGTCGNGNACNNCCNGNARGGCACCNCACATCNRYNNNNNNCTCRGGGCGNAAGCGCYTAAAYANNYAGTTTCCACCTGAGGTYAGNNCTGNRAYCTAAGGTAAGCCCCATGAGCGATTGGGAGGCTGATTYYNRATGTCCGATCYYATTCCTCGAACCGACCGTAAAGTACGTGAAGAATTTGTTAACTTNACCCGCTAGACCNCNNNNYGGYNCANAGCTGCCTCRGTCTAATGGNACTTCCAACGTTTGCCTCCAAGCGGCAGGRRNNNGTTGGCCCACCCNCAATNTGATACAATCTNYAGYRAGCCGNTGGAAGGCCCNCGCTAGCATTTGCRTCYACATGCGTCCCTRTCACTTYCATGNCTGGYYGGCGNNNYRGARGNTCTCGATCTGTGANGRNNAGCTGTCACRGANGTNRCCGGYTRTTGNGTRNCGCGGGTTGTGCCCAGATTGTCCTCYAAGNNGCNRNNNGRCCYTNANTTRGGGCGTYGRGANATAATNGCAATCGNCNCARGTCCTNNGAACRTRRGATCCTGATCNCTCGATCCCACCTGTCRNAGCTNNCGCTRCGTCTTCTCAGCGACTGGCTAGCTTGAACCCTAGATAACGAGGGTGTTAAGGGACTCTNNNAGGGCAACCTTGGRNNNTCNRYNNGATCNNNANACCGNYTTGTNGCATGGAGGCAGAGCCCNTAGGCTGAGACCNGGCCNGTCCGCAAACNNGYCAGTTTGACCNNAGGCCGACAGGGGCGCNACNNANCNNNGYTTCANGGAGNRCRCCNANCTATTYTAGCAGGAGCNRGTRTGCNNACCCGTACCAYNCGCYTGAGGCTTRTCTGTCGGTAAAGYCANNRNNNNRACNGGTGAGTGCCCGGTNCTTTGTATAAATRCCCTTTCCGGGAGCATYNGANCGYNCGRTYCCGAGCTATGCCATTAACYAGGNATTGCCNNYGYACGGCAACGGNYYGRTGTCRCGANCTCGGGNCGGTATGTGGCAGACTTTTTRRRTCCGACNCGATTACCCAAGANNGAGGCCCCAGCATCCTTACGCTGGGATTTANNGNNAAGACGATCGGGCAAAGCNCTNCCTGCTRGCGGAGNNNNNYGRTGNCCRNAACGAGNGAYCAGATTTNGNACCYRGCATYYTACARTNNNNGGTYANYRCANTCAGTCNNTCGCAGCCTCGCANTGCACATT

BrM23-1 NYGRYTNRCTNNNCRCYATAATGGCNGCCNNNNYCGTCGNYCTNNNNNNNNCNNNGAGGTTYACCCNCTGAYNGYGANNNACTGGCYANNNCGCCTTTTGTCNNRGGAGNCCRCTGCCCGGGRTCNNNCTTGGGTAYRGGCNRTGCTGCGCYNACNNTGNNNNGGCAGCGNGYNGRNGAGCNNAAAGTANNRRGAGGTTTGGACCTGGCTTNGGAACGGTAGATCNYAYAATACGGCAGTCNNTGTGNNNGTTNGGYAYGNANNTCNTAGGNCTCATGAGGANNNNNNTGRNRANCGYRGATCACNCGNNGGNCATTGACNATNNYCNRTGYNNGTNNATARNCCGGTTGATGRNNTAGNNTNRAGARCRGTNNAGCCATTCTGGAGYTGYTANGNTGNNGCGGGCNCCGCAGGNNNNNAANNTANNNACATTACANTGCYGCCAGCTRGYYYCAAANCNNNNNCTCTYTACTGNNNNNNGGNCTGCNNNTGNNTACNNTTGGCTRNNNNNNNACCCTANCRGACACRGCNAATNATCCTNGTCTAGACTCNNNTAAGAGTCTAGAGCCGACGCGTACGGTTTCANCACCRACGNNNNGAGGNANGAACNNCTCCGGCTACCAACNNNGGANNNTCCCCTAGAAACAAGYGNNNGCTGNNGCTGAGCAGGCCAATNNNNNCAATCTGCTCCNNNNNNNGNNNNCCCCNNNCCCCGCTTATGNGNGTACCTAGCRNACTGCNNNNTNNNGTGGGTACCAGGAGGCTCCNNNYNCATGCGAATGGYNGGCACTACCRNRRNCTNGCCGGCTGGTAGCTAGGAACTGATNNTATGTGAGTACANNNNNNGYYCNNNNTNCRGNTACAGCTAGCCCTGTACACAACGGRAYYGTNCNTNGGCTTGCGAATNNGNNTNAGTTCGNNAAAACGGGCNNCGNNNNCCNGTCAGYCACNAAANTTNNNANTNGNNGTARNTCGCGGYNGCCATGGCTCCTATANNNGGAANNGCCCGCGGNGNNRRGNCGNNNNGCNTCGGCGAGNGCCGCYYNNCYGCAAGGGATANTCANACGATTARAYNNNNNCAGAAGGAANGCACTCTTCTTRACRCANGTNAYACGCTGTGGCTCCANNNNYNRCGGCCGGGGGANYAGRCNCGTCCGGGGGNCGCTNANTACYNGGGTCTNNGNNNNNGAATGCCCANACGATGGGGCCANNNACACCGNNNNNTNCTCGCAGGGGGCNNNAGNNNGNTTNCTGCNNRGNATTTCTTTGGCCCGACGGTAACGCGNCRGNNNYYNNAAAGACATANNNNNGAGATNNNACGCCNGAAATGTCGNTTAGGAANNNNCTAGCACTGACGYRYANRYRNCNNGCGYATCGGTNNCTCTCCAGGTGACCNNNGCGAANGAANTGGGCTCGCCCCTCGANNAAGGAAGNGAAAAACYNNRRGACTCTGNNNACTYNNNGNNNNNAGTGAANATAGNNNNTCGCCCAGTTTNRNATATGGTCTNNGGGANNANCCGNCCTCTGACTTACCCAGTGGCTTCCGCNGGANNNNNNRCRRCCAGAAAGNGRRYCTCCTGCNCNNNCCAAGNGGTTTTTCANNTANCCTNCCYAGGTNNNTCCGANGGAATTGCTCTGCCTGTGYTGNNNNGAGCCCTATATCTRNNNACCGGGNGGGCACNGGTYTCGNACTYGTAGTRCNCGATATNNNNNNGGRNRNNANTATNNNCAGRTGGACTGTATTAGNCTNCTTTNNNNAGATGGCATGGNNNRTTTGGATATCGTCCNNTTGGTTATTNNNNNNNAGNNNNTNCCNACGNGNCCCGANNAGYGACNNTNRGNCNNCNNNNCCTCGCTNGACAAYTTCYAAAGCGTNCNGAGGATNCTNNNTTNNNNNNTCNTTTNNNCTAGNNAATTTCGTAATATGCCGGCANCGNANNNCTNGCACCGCARTAGGTGTCTTGCAGAAYGGNTCAAGYANCNACGGCGCGTNNYNATCRNGCCCCTGGCATGGCGGCNNNAGCTAGCCGTCCCANNYRATGGANCGCGTCCCCACNAGGNAGGCGGGANGAANNCGGAGACGCTATGANNNGGNTTGTACACNTTCCACGCGRTTCTGCNNNNNNNNNNGGGCGGTNCRGRNCNCCANACCGGNNTGGGGYGYRATTGGGNNGCTCGCAAATACCTRNNAAGTACTCGNCNGCTAATCGGCGCATCNNGGNTANGGNTTCTCGCTTCTNNAYTAANRTTGGCNANNNNRATGCATGTNGNNNTNYGCRGYNGNAANGNGCRARNYCRYGNYRRYGGGCTCYGCGACNNCATTAGTTANNNNNTGNGTCAAAGANTNNTCGTTCGTNNCCNNCCCCGCGGNGCGAAGNNNCYCNNNNTGCCNCTNCGCGAAAGCNTGNNAAGTCGNNNNCNNTCTCCTGCGCACGCGRANNGGNNNCGNNNNCNGNTTNNGAGNNNTYCNAGGACTANNNCTANTNNNANAAACGGCCGGYANNNGNANNCNGCTGGNAGGNTACAGNTTAATTAGCYCGCCCCGGAGNRATCGTCCTCNTTGTTANNNNCTACTTCGGTGACATACATCGCACTATACCNANNNNGCAGCNNCAGCGTNNTNCNNGACNCTAGANNNNNNNNGCATCTCGGGCTAATACTTNNCCNNNCTCCTGRTCACCGGTGGCCACTAGGYGCCTGTTCCTGGATACTCGAAACGGGCCTCCNNNNNTCNNCNTCTTNNAAGACNCCCGNNNGAATGCGGCGTNTTAYYNNNGTNANYTTTGAGCGTCNNNGACGAYGCANGACCGCCGGTTTATGAACCGCACCACCRGANNTGNRGGRNNRGANACGNNNTATCNTAACGYRNGTNNCNACCTGNNTCAGCYNRGAGAATGGRACCGCGATCACGNGGCCCCTTACNCCGCANGNACGCYGGCCNCAAGTAAGGGTGTCTACNYCGATCCCGGGNNTGAAANTCGNNCATYTCCTTCTAGCTGAAANNNCCCCCGAYCNNNNNNNNNNNNTACCACGCTGCCNGTAATGGGACYYACNNNNNTTGCGANNNNTNAAGCAACCCCGTTGNTCTATCANNGNGGTATTNNNTTCTCGCGTCCTCNNNAANAGACGGGNTGAAACANTNAGGTGCTGTCGGNNCNTYCNGCNCNNGTYACGTGNNNAANAGTCNNTAGGTATGNNAGAGGANNTNNNGCACCAGCGTYTTGANCTCGAGTTTTNRRGTTAGNCNNNNARCNCNNNAGACGAAGNCAGCTCNCAANCGNNAGNCYATCCCNCTAAANGYNACTCNNNGGGACGCGGAAGCNTCAANGTRCRANCAACCTNGTGTTGGTCGGNNGARNAGGGTARNNNAATCNNNNNNTCNCAGGNTTNGTYCGTGCCGRAGGAGNNATCACCAAGGNTAGTNNCCGCGANATGCGCTACNTGYCCTTNNNYAGTCTACATTGNNNAATANANTCGTCGCTGCCYCATCNNGGCCGTACGYANANNNCYCCAGAACYTAAATNGNCGGCTANNNCGACGCCAGNCTCGCTCAGCCTGAGCTAYTGTATCTAANTGGANTGTCNNNNTTGNNNYTGGCGGRYCCCTTTTATNCTCGTNGCTATAACNCACAGNCNNNNTRAGAAANCATACAYGCGGTNAANNNNNGGGNNNNNCTACACAGNCTRAGGGGCCATCCTATTRYGGATRAGTYGCGCAGNCTCNNNCTRRATCGGGTYCATCGTGNRCGGANCGNTCCGCAGGCGGCNNNNCTGATCGNNNNYRNNTNNRRYNNNNNNGACYTNNNCNTCCRCGCCCGGNNNNNGTGGCCGTTCCGTNNAACTGNTCCTNNNNNTNRGATNYCCTNGNAAGCACNNTGGAATAGRTTGTACCANNCCNCACGRCNNCATAGGNCGCCAGCGGTNNNNTACTTTNNCNNTCANGTTTNCTNNNNNNNCCCCCRRNCRNNCATGCNNNNAAGGATGNGRTCATNNYRGCGCGAATCNGGYRCRRYCTYNGNYCCCNAAAAYYGCNNTCATTTATGCGYACGTCGANTGCACCGCGGCATTGCTNNCGGNTACTTGCGGCCATCATAGYGTTCATGGTGTTNYNANNTNTNCTTAGGCGCGGACCGAAANCGCCACGATNATGNGTAGNNNNCGCNNNNNNGTCCAAAGCGTTAGCNNGGANYNTGAAAGCGCGNNTATTACGGCTCACNNCNNNACAGGTTTAGGTACGNCNNNNNANNNNCTRTACCCCTGNGNNACNGCCNTTGCGCANNTTCNNNNNNNGNGAGYNNNNNNNNTGTTRNNNNGGGCTCNCGAGGTTACNNAATTGTNGCGTCTCCYYAAYCNAARNRRARTNNTGYTACCACGGAGNANNCCGGCATGRTNNNNCTTNACYCYYGATAYNNATTNNNNNGCAACGAAGNNNNGCGNCGYAYRACANANAGATGACAANTAAAGATACGCAGTACGGGACGACGTAGCCANCNNNGACCCTGATCGTGTTTCGTAACAACACAACNTCRRTCTCTNNNNNNAACCGNNTGCNNNNNYCTNAGTCAGCNGGGNNNNNNNNNNNAATARNNCRAGNGYCGNGCCNACCCGANNCTAGCGNGTCGTGAACAACCCGANGGGCACCACACANCNATNTTTCACTCNGGGCNGCAGCNNNANNNCACTYAGTNNCCACCTGAGGTNAGNNCNGNRAYCTAARRTAAGCCCCATGAGNNNNNGGNAGGNNNAATCCNRNNNNCCARTCCNATTNCTCGAACCGACCGTAAAGTACGTGAAGAATTNGTTAACTTRNNNCGCTNGACCGCATGTTGGNNCACANNNACGTCGGTCTANNGGNACTCCCAAGGTTTNNNNNNAAGNRRCAGGNNTTAGTTGGACCNNCCCCRRTCNGNTACNAYNTACANCAAGCCGATAAAAGGCACTCNCNAGCANNNGCATCCANATGCGTCCCTGTNTTTNNNNNGNCTGGCTNNNNNNAYRGAAGNNCTNNATCTGTRNNGNNNAGCCGTCRCAGAGGTTACYGGCTGTTGTRTRNCRYGNNNNNTGCCNNNNNTACNCTCYANGTGGTAGATNNNNNNTNACTTRRGGNGTCGNGACANAANCGYANNCGCNNCAGGTCCTARGAACGTGGGATCCTGATCGCTCGRTCCCACCTGTCAAAGCTACCGCTGCGNCTTCTCAGCGACTGGCTAGCNTGAACCCTAGATAACGGGGRNGTTNAGGGACTCTAGANNRGCATCCTTGGATTGYNCRCATGATCNNNAGACYGNNNYGNGGCANGGGGGCRGAGCCCCTNGGCTGAGACNNAGGCNNNYNNNAAACNNNNNNNNNNGRCCNNNNNCCGACNGGGGCGCAACGTAACATAGCTNCACGGAGNACACYGAGCTATTTTAGCAGGAGCNGGNNTGCATACCCNTACCACGYRCTTGNNNNNTATCTGTNAGTAANGTCANNNCGCCAACTRGNGAGNNNNTGGTACTTTGTATAAATGCCCTTNYCRGGAGCATTNGATCNNNCGRNNCCGAGNNNNNNYATNNNNTAGGCATTGYCTTTGCACGGCANNGGCCCGRTGTCACNNCCTNNNNNCGGTNNNNNGCAGACTTTTTAGATYCGACTCGATTACCCAAGATGGAGGNNCCAGCATCCTTACRCTNGGATTTATTNTRAAGACGATCGGGCAAAGCRCTNCCTGCTNNNGGAGTCCNTYGRTGNCCRRANCRAGGGATCAGANYTNRNACCTAGCANCCTACAGTNNNNNNNTNTCGNNTTNAGTCYNACGNGRCCTCGCAGTGCANATT

BrM27-1 RYGATTTANTNRYCGCCATAATGGCNNNNAGGGNNGTCGACCTAGACGGGTNNNNNANGTTYACCCACNGACYGTNNYYTCTTGNNNNNNNCGCCTTTTGTCTAGGGAGTCNNNNGCNNGGGANCCNTNNTGGNNATAGGCCATGCTGCGNNTNCAATGNNNNGGNAGCGCNCANNNGAGCCGAAAGTACAGACAGGTTTGGANNNNGCTTTGGNACRRYRGATCNNANNNNACGGCAGTCTNNNNNTANNNNNGGNANNNACRTCNNNNNTCTNNTNAGRAGGTTTTNNNCAANCGNGGNNCACTCGNNGGNNATTGANYANTNNNAGTNCCCGGGGATNNNNCGGTTGATNNNNTAGCTTGGCGNNNNNTGGAGCCATTCTNNNNNNNCTANNTTGGGGNNGGCGCCGCAGGNNNNGNNCCTNNGTNCNTTACNNNGCCGCCAGCTANNNNNNNNNCRCNCTCTCTNTACTGCTGAANGGNCTGCNTTTNGTTACCTTTGGCNNNCAACAGGCNNNATCAGACACAGCTAATYNNNCTNGTCTANACTCGTTTAAGNNTATAGAGCCGACNCGTACGNNGTTNNNNNNNACNGGGCRNNNAAAGAACGTCTCCNNCTACCAACNGTGGACRTTCCCCTNNAAACRNNCNNNNGNTAAGGCTGAGCARGCCAATCTCCTCGATCCGNTCCYYTRTNYGCANRCCCCYRCCNNNGCTTNNGANAGTACCTNGNATANTGCGCCGTGCGRTGGGTNCCAGGAGGCTCCTATNNNATGNNNNNGNYNGGNACTACCGTAATCTAGCNNNCTNNNNGCTANNAACTGAACTTATNTGAGTACATGCCCCGCNCGCAGTNNNNNNNNANCTANNNNTGTNNACAACGGNNNNNNNCTTNNGCTTGCGANNNNNNNNNAGTTCGGRNNNNNNGGCTTCTTGGTCCNNNNNNNNNNTRAAGTTGYRANNNGNAGYANCTNGNGGNAGCCATGGNTCATAANTTCGGRNTANCCCNCGGTGTARRGNCGAGCCNNNNCGGCGAGGGCCGCTNNGCTACRAGGNACAATCNGGCGATTAGATTATGTYNGNANNAACGCACNCCTCTNNNCAAAGGCCACACGCTGTGGCTCCACTTTNNGNGGCCGGGGAGTTNNNNACATNNNNNGGTCGCTAAGTACTAGGGNNNAGGAAYANNNNNNCCCAAACNNNNGGGCCANNNACACCGACAARTTCTCGCAGAGGGCGCCAGTTNGYTTGNNNNNNAGAATTTCTTTGGCCCGACGGTNNCGCNNNNNNCCTTGANNNNACNTACGCGTGTGATNNNNNNNNTGAAATGTCANNTAGNAANCCANTAGCACTGACGYRTNNGTATCAGNNNNNTNGNNATCTCTCCGGGCNNNNAGCNNNNNYGNNRTGNNNNCGCCCNNGGANNAANNNAGGNNNNAACTTCGGGACTCTGANGACTYNNNGACCNNAGTGNNCATAGAGCATCGNNNAGTTTAGCATATGGTCTNNGNGANCARCCGTCCTCTNNNTTACCCAGTGGCTTCNGCAGGATTTTGTACAGNNNNNRAGCGRANNNNNTGCACCTCCCNAGTGGTTTNNCANNTACCCTANNNACNNNGCTCCGACGGAATTGCTCTATTAGTGCTNNNNNNNGCCCTATATCTATTANNTNNNNNNRNACTGGTYTCGAACTCGTAGTACNCGNNNNNNGGTCGGGGNTCAYTATATANNNGTNNNCCATATTAGTCTGNTNNAYTGAGNNGGNNCGGNCTGTTTGGATNNCGTCCGCNNNNNNNACTGCTCGAAGCCCGNNNTNCCTCATCCCGAAGAGTGACNNNGNNGCNNNGGNTNCTNRCTGGANANTTTCCAAAGCNCCNNNAGRATACTTCGTTCTCTTNTCATTTACGCCACCCAATTTCGTAATATGCCGGCACCGNANNNNTGGCANNNNNNNRNNTGTCTTGCAGNAYGGCTCAAGYNNNNGCGGCGCNTCRNNNNNANNNCCCTGGCATGGCCGCNNNAGCTAGCCGNCYCAYYYRATRRAGCACTTCCCCACGAGGYNGGCGGGANGAACACGGAGACGNNATGACGGNNNTTGTAYANGCTCCACACNNTTCAGTCGTTRCGCAAGGGCGGTNCGGGCNNNNNNACCGGTATGGGNNGTANNNNNGGTGCTCGCANATNNCTRNGGNATACNNNNNNGCTAATCGGCGCATCGTGGYTAGNNCGCCTCGCTTNNGTATTNNNNNTGGCTACGTAGATGCATNNCGANNNNTNCGGCNNNNNAGANNNNRNCCATGGNNACGGNNNNCGCGACCACATTAGNNNCNTAATGCNNNNNNNNNNGATCGTTCGTNNCCNNCYNNNCGGCGCGNNNNNNCYNAAANNNNCRCTACGCRAAAGCCTGTNAAGTNNNGYYCAATCTCCGGCGCACGCAGCGCNNTCGNNATAGCGGNYTNNGARCGANNNNGGGNNNNNNNCNACTNNNAGAAACGGCCGGTANNNNGAAYCYNCTNGNNNRYTNNGRCNTNNTTANNNNNNNCCGGAGNAATCNTNCTNGTYGNNNGNGGCNNCTTCGGTGACANACANCGCATTAGACCYAYNCAGCAGCAGCAGCGTCYTAYAANNNNNNARACGCNYACANCRTCTNNNNNNNNNNNNNNNCNNCNCNNCTGATCACCGGNGAGCACTAGGCGCCGTATCCTGGATAACCGAANNGGGCCTCCNANNNTCGCCATCTTACAAGACNNCYGANAGAATGCGGYGTNNTANCCGCGTNNNNTTTGNNNNNNGGTGACGATACACNACNGCCGGTTTATGAACCGCACCACCGGGCCTGCAGGRNRRGAGACGNNNYATCNTAGNNNNNGTGTCNACTTGTATYAGNNNNGRRAATGGRAYCGYRNNCNNGNGANNNNNNACTCCGCANGGACGCTGGTNNCAAGCGGGGGTGNCNNNNCCRNNNCCGGGCCTGAAAGTCGAACANYTNNTNNNNGCTGRRNTNCNNNNCGAYCNNNGCATANAGTTANCACGCTTAYNGTANTGGGACCCACCGTGTTTGCRNGAATTAAANNANNYCCGNTGNTCYRYCANNGNGGTATTNAANNNNNNNNNCCTNTATRRNNRNCNGNGTNAARCATTAGAGCACTGTCGGNNCATCCGGCCCNNATCANGTGTGYAANAGTCNCTRGGTATGTGNNAGGAGTTCGGGCACCACTGTCAGGACCTCGAGTTTTCAAGTTAGCCACCCAGCTCAACNGACANNGGCNGCTCANAAGCGGTAGCCCRTNNNNNTAAAGGTTACTCCAGNNGACGCGNAAGCCNNAANGTGCAATCANYNTCGTGTTGNNNNGACGAGAAGAGTAGGACGATCCCTATNNNNCAGGNNNNNNNNNTGCCRRAGGAGNYNNNNNNNNNNNNAGTGTYCGCGAGATGNGCTACTTGNNCNTNGNNNNTYTRCATTNCATAANNAACTCGTCGCTGYCTCATCNNGACCGTANNCANAAGCCTCCAGAACTNAATNCGCCGGCTACCCCGACGCCARCCTCGYNCRGCCTGAGCTANTGTATCTANNNCGACTRTTNNCANTGTNGNTGGCGGRYCCCTYTTAATCTCNNNGCNANRACTCACAGGNAATTTGAGAAATCATACACGNNNNAAACCGTANNGCTANNCTACNCAGCCNNNNNNGCCATCCTATTGCAGATGAGTCGCGCARNYTCGNYCYARATCGGGTNCATCGTGNNCNNACCRGTNNNTARRCGGCCTGTCCGATCGNNACYRCNTNGGGYNCCTGCNACNTANNCGNNNNNGCCNNNGTGCCGTGGCCGTTCNNNNNAACTGATCCAGGGACTTAGANNCCCTTGTAAGCACNNTGNAATAGNTTGTACCANNCCNCACGRCGGCANNGNANGCCNRCGGTANCTNNNCTTAGCCGTNAAGTTTCCTCGTGTTGNCCCCRRGCAATCATGCNNNNCGGGATGGGRTNATGGCNNNNCGANTNNGGCGCGGTCTCNNGTCCCNAARANTGCNGTCANTTANGNNCGCGTCGAATGCACCNCGGCATTGCTGGCGGATNCNGGCGACCGACATTGYRTTCNNGGTGTTNYNAGNNCTCCTTAGGCGCGGACCGAAATCGCCNCGATCATGCGTNNNNGGCGCAAATGTNAGCAAAGCGTTAGCACGGANCTTGAAAGCNTGAATATTNNNNNNCANTCNTNNACAGGNTNNNGTACGNCCYCACACTGGNTGTACCCCTGGGGGACNNNCNNCGCNNNNNNNNACGNNNNGGGAGCYRACACYYTGTTGTCGCGGNNNNNCRNGGTTACTAANTNNNNGCGTCGCTCTAATCRAAGNNNAGTTCCRNNRCCNCGGAGNANNNNGGCATGGTGTCGCTTCACNCCCGATNNNNATYGCATTNNACCGANNNNNNGCGTCRTATRAYANAAAGAYGACAAAACTAGATACGCANTACGGGACNNCGTAGCNNNNNNNNACCCTNNTCGNGTNNCGNANCRACACARCNTCRGTCTCTGRNTTNACCCNGGTGCAGACGTCTCAGTCAGCNGGGATNACNNGCCNAATARGNNGAGAGCCRRNNNNACCYGAGTCTAGCGANNCGNGNNNNNCCCGAAGAGCGCCACNNATCTATATTTCCCTCAGGGCGGAAGCGCNNNNANNNTCAGTTNNNNCCTGAGGTNNNCCCTANNNNCTAAGGTARGCNNNAYRNGCGATTGGGAGGCCGRATTTGGANNNNNNANCYTATTCCYNNANNCGANNNNNNNNTACRTGAAGAATTTGTTANCTTGNNNNNNNNRGCCGCRTGTCGGNACACAGCTNNNNNAGTNNAGTGNNNNTTCTANNNNNNGTCTCCNNNCNRNAGGAGNNNGTTGGNCCRCCCNCAANNTGACATNATCTACAGCAARCCGNTNNNNNNCACTCNCNAGCATTTNCATCCACATNCGTCNCNRTYTTTNNNNNGNCTGGYCGACGGAACGNNNNNNCTCGATCTGTGNNNNNNAGCCATCANNNAGGTTACYGGCTGTCANNTGCCACGGRYTGTGCCCAGATTRYYCTCCAAGTGGTAGATAGACCCTTANNTAGGGCGTYGAGAYATNATCGCAATNNNCTCAGGTCCTARGAACGTGGGATCCTNNTCGCTGGANYCCACNNNNNNNNNNNACCGCNACGNNTYCTCAGCGACNNNNTAGCTTNAACCNNNNATANNGNNNNNNNNNAGGGACTCTGGCNNNGNATTNNNGGRNTNYNNGCNTGATCNNNAGACNGCTTYGTGGCATGGGGGCAGNNCCCCTAGGCTGANNNCTRGGCCGTCCGCAAACTNGYGANNTGGGCCAGNNNCCGACAGGGGCGCAACACAGCTGGACTNCANNGNGTACACCNNNNTTTNNNNGCAGGAGCCGGNGTGCNNNCCNNNACCANNCGCYNNAGGCTTATCTGTCRGTNAAGTCANNNCGCCAACTNNNGANNGCNTGGTACTTTGTNTAAATRCCCTTTCCGGGAGNATTNGNNYNNNCNNNCCNNNNATCAGCCATTRRYTANNNNNNNNCTTTGCACGGCNACGGCNNNNNGTCGCGACCTCGGGACGGTATGNNGNNGAYTTNTTRRNNNCGACTCGATTACCCNAGANNNNNGCCCCAGNNNNNNNNNGCTNGGATTTATTNTGAARRYGATCGGGCAAAGCNCTAYNNGCTGNNNNNNTCYGTTRRTNNNNNNATCAAGTGATCNNNNNTTGTANNNNGNATCTTACNGTGGCGAGTTATNNCNTTCAGTCCGACGCGRYCTCGCANTGCACATT

BrM45-1 GCGATNNNNTCNTCNYCNTANNGGCNGCCRGRGTCGTCGACCTAGACNNNNCTAGGAGGNNNACCCACTGACYGTGANNNACTGNNNNTCACGCCTYNTGTCTANNNNGTCCGCTNCYCNNGATCCRTCTTGGGTATNGGCCNNRCTGCGNYTNCAATGGCCAGGCNGCRCGCAGGAGAGCCGAAAGNNNNNNGAGGTTTGGANNNNGCTTNGGNACRGNNGNCTANANNATACGGCNGTCTGTNNNTANNNNNGGNACGAANNNNNNNNNCCTNNTRAGNNGGTTCTNNNCNATCGTGGATCRYNCRCAGGGCATTGACTATTYCCAATGCNNNNNNNNAGTTTGGTTNNNGANNTAGCTTGGNGAACGGTGGAGCCANTCTGGAGNNGNTAGGNNNNNNNNGGCGNNGCAGGCCATGNNNCTNNGTNCATTACANTGYCGNNNNNNNGNNNTNRANCACCNNCTCTTTACTGCTGAANGGNNNNNRTTTRGTTACNTTTGGCTANNAAYGAACCCTANCRGACACAGCTAATYATNNNNNTCTAAACTAGTGTGAGAGTCNNGAACCGACGCNTACNGTNNYAACACCGACGGGGCNAGGAAAGANNNNNTCCRRCTACNANCANNGGACATTCCCCTNAAAACAARCGACNGNTNAGGCNNNNCANNCCNNNNNNNNCGATCYGNTCCYYTRTGYGCANRCCCCYRYNNCCGCTTANNANAGTANCTRGYNNNCTGCGCCGTGCGGTRRGTACNAGGNGNNNNNTNNTGNATGNGAATGNTNGGCACNACCRNRRNCTAGCCGGYTNNTAGCNANNAACTGRTNNTATGNGAGTACATGCCCCGCTTGCACTCCGGTTACAACTANNNNTGTACACAACGGAATTGTTCTNAGGCTTGYGAATNYGRNNRARNTNGGAAANNCGRGCGCCGTGRNCNNGTCANNCACTAAAGTTGCAAATTGTAGTAACTNNNNGYAGCCATNNCTCNTANANNCGGGNTAACCCACGGTGTAAAGGCGGGCCNCGTCGGCGAGRGCNNNNYNGNNNNRAGGNAYAATCRGRCGNTTAGATTATGTCTGAAGGAACGCACCCCNNNTANNRNAGGTNNNACGCTGTGGCTCCACYTTCGRCGGCCGGGGAGNNNNNNNCNTCCGGGGGCCGCTGACTACTNGGGCCTAGNAACAANNATGCNCRRACGATGGGGCCAGCCACACCGNNNNRTTNTCGCAGGGGGCRYCAGTTCGTTTGCTGCRRRGNATTTCNNNNGYCCGACGGTNNCGCNNNGGTATCCGAAAAGNNNTAGGTRTNNNNNCAGNNNNNTGANNNNNCRNTTNNNAATTGACTAGNNNNGACRYRYANRYATCAGNNNNATCGGTATNNNNNNGGGCNNCTAGCGCGAAYGAAATGGGCTCGCCYCTGGANNANNNNNNGGAAANNNTTCGGGACCCTGAGTACTCNNNGACCNTAGTGAANATAGAGCNNCGNNNAGTTTANCATNNGGTYNCTGGGAGCANCCGTCCTCTGACTTACCCAGTGGCTTCCGCAGGANTTTGTRYRRCCAGAAAGNGNNNNYCYCGCANCTCCCAAGTGGTTTTNCNNNNAGNCTTCCNACGTTGCTCCGACGGAANTGCYCTRYNNGTGCNNTNGTGAGNCCTNNATCTRTTAACCGNNNNNRNACTRNTCTCNNRNTCGTAGTACTCGATATAAGGTCGGNNAYYACTATGTGCARGNRGACTGTATNNNNNNNNNTYNNNGAGATGGCAYGGTCTGTTNGGATATNGTCCGCTTGGTTANNNNCYCGANNCCCGNNNNCNNNNNTCCCGANGAGTNACGGTGNNNNCGNNGATTCTCNCTGGACANTTTCCAAAGCGYNCAGAGGANACTNNGTTNNNNNNYCNNTTACNCTANCCANNNNCGNNNNNNGCCGGNGGCGTAGAGCTGNTGCCGCAGTANNTGTCTTGCAGANNGGCTCAAGCRGCAGCRGYGCATCGCTAACNNGCCCCTGGCANGGCGNNGACAGCTAGCCGNCCCAYYYRATRRAGNRCNNCCCCACGAGGNNNGCGGGAYGAACAYRGAGACGCTATGANNNNNTNNATCCACACTCCACACGGNNCAGCNGTTANNNAAGGGCGNTTCGGGCNCCNANACCGGTANGGGGCGTAATTGGGGTNNTCGCAANNNNCTGAGRAGTACTCGACCGCTAATCGGYGCANCNNGGTNAGGGCGCNNCGYNNCTGTAYTAANATTNNNAANNNNGATGCATGCNGAACTTYGCAGTNNNNAGGAGCRAGANCNYGGYRNNGGGCTCNGCGACCANNNNNNTGACYTNNTGYGTCNNAGANTNNTCGTTCGTNNCCNYCCCCGNGGCGCGAAGNNNCYCAAAANNCCRCTACGCRAAAGCCTGTCANNNCGCGTTCNNTCTCCGGCGCACGCNNNGCGGTCGCGATAGCGGGYTGCGAACGATTCGNNNACTAGCGCAAYNNCTAGAAACGGCCGGTACGGNAAGNNTGCTGGTAGGTTACRRYNTGATTAGCTCGCYNNNNAGCANTCNTNCTCNTYGNNNGGGGCTANNYYRGTGNCNTACANCGCATTNGACCNNNNCAGCANNGGCAGCGTYTTRYAAGACTCTARACGCCCACAGCATCTCGGGCTAATACTTCTCCNNANTCCNNNTCACCGGTGAGNNCTNNRYNNNTGTTCCTGGNNANNCGAAAYGGNNNNNCTAAGCNNGCCATCTTNYAAGACYCNNGACANNNTGCGGYGTTTTNTCCGTGTCACTTTTGAGCGTCGGTGACGNTRCANGACCGCCGGTTTATGAACCGCACCANCGGNCNTGCAGNGTGGGAGACGNNNTATCGTAGCNNNNNNGTCGAYYYGYNTCAGCCGAGNNAATGGRAYCGYNNNCACGYGAYYCCTTACTCNNNNTNNACGCTRGYCNNARGCGGGGNNNTCTACAYCGATCCCGGGCCTGAAAGTCGAACATCTNNTTCTAGCTGGGTNNNCCCCCGANCTGCNNNTAAAGTNNCCACGCTTACNGTAATGGGACCCACCGGGTTTGCGANNNNTANANNANNYCCGNTNCTCCGTCACAGCGGTATTAAANYCTCNCGTCCTTTRNGGGNNACGGNGNNAAACANTCNNGNNNNGTCGGAYCATCYNNCNCGCRTCACGTGTGTAATAGTCCNTAGGTATGNGAGAGGAGTTCGGGCACNNNTGNNNNNNNCTCGAGTTTTCAAGTCGTCNNNNCAGNTCNNCNNNNATTGGCAGCTCANAAGCGATGACCCATCCCACTARANGYNACTNCNRGGGACGCGGAAGCNTCAAGGTATAATCAACCTCGTRTTGGTCGGNNGAGGAGGGTAATRTAATCCCTATTTCGCNGGNNTGGTCCGTGCCRRNNGNNNNTTNNNCAAGGATNNTGTNCGCGAGATGCGCTACNTGNCNNNNNNCNNTCTACATTGCATAATATAATNGTCGCTGCCTCNTCNYNNNNNNACACAGAANAYCNNAGAACYCAAATCGYCGGCNANNNCGACGCCNGCCTCGYACRGCCAGAGCTACTNNNNCTAAATNNNCTGTTNNNATTGTAGTTGGCGGRYYCCTTTTNNNNNNRYAGCNANRACTNNNAGGNANTTTNNNNNNNNATACACGCGGTAAACCGTAGGGCNAGGNNNNACANNNTGNNNGGCCATCCTACTGCAGATRRANCGCGCAGTCTYGNCYTRRRTCRNNNNCATCGTGANCGGANCGNTCCGCAGGNNNNNTGCCTGATCGNNNNNNNNNNNNNTANCNGCNAYCTANNCGTCCGCGNCCGGGCNNNNNNNCCGTTCCGTGNNRNTGATCCAGGGACTTNNNTGCCCTNNNNNNNNCNACTGNNNRNNTTGTNNNNNNNNACACGGCGGNNNNGGACGCYAGCGGTNCCTTACNNTRGNGGTNAAGTTTNCTNNTGTTGCCCNCRRGCAATCATNCNNNNCGGGNNNNGGTYATGGCNGCGCGANTCANNNGCGGYCTNNGNTCCCANNANNTGCTGTCATTTATNCGTACGTCGAATGCACCRNGGCNTNNNNTGNNNNNRCYGGCGACCGTCATANNRTTYATNGTGTTGCGAGCTCTCCTTAGGCGCGGACCGAAATCGCNNNNAYCATGCGTNNNNGGNNNAAATGTGNNCAAAGCGTNNNNACNNNNCTTGAAAGNNCRRATATTNNNNNTCACTCCNATNNAGGTCTAGGTACGGCCTCACACTGGNTGTACCCYYGGGGGACNNNCGTTGCNNNNNTTCACGGGGGGNGAGCCAACACTCTGNNGTCGCGGGCTCNNNNGNNNNNGGNANTGTNGCGTCGCCCTAANCNAAGGAGARTTATGTTNNCNCGGAGGAAGCCGGCATGRTGTCGCTTCACNCCCGATNNGTATNGCANNRCNNCGANNCYNNGCGTCGNAYRACANNNAGANGACAAAACNAGATACGCAGNNNNGGACGACGTNNNCACCACGGNNNCTRATCGTGTTTNNNNNNAACACANCNTCGGTCTCNGACTNTANCCNGGTGCAGACGTCTCAGTCAGCNGGGATTACTAGCCGAATAGNNCGNGAGCCNAGCCAACCCGAGTCTAGCGNGNCRYNNACAACCNGGAGGGCACCACACATCNATNNNNNACTCNNGGCGGNRGCGCYNAAATCCTCAGTTCCCACCNGNNNNNNNNNCTRAAATYTAAGGTAAGCCCCATGAGCGATTGGGAGGCYGRATCCGGNNNNNNNGNCCTNTTCNNTAANYCGANNNNNNNNNANNNGAAGAATTYGTTAACTTGNNNCAGAGAGCCGCGTGTTGGYNNACANNNNNNNNRGTCTAATGNNACTTCCANNNNTTACNNNNAAGCGRCAGGAGNNNGNNNNNNNACCCTCNNTATGACATAATCTACAGCAARCCGATGGAARRCNCTCGCCAGCATTTGCATCCACATGCGTCNCTGTYTTTTCCATGTNNNNNCNNNNGAACGNNNNNNCTNGRTCYGTGANGNNTAGCNGTCGCNGATGTAGCCGGCTGTTGTANNGNNNNGNCTGTGCNNNNNNTGTCCTCCAAGTNGYARATNNNNNNTNANNTAGGGNGTYGRNAYAYANNCGCAATCGNCTCARGTNCTARNNNNNNRRGNNNNTGATCGNNNNATCCCACCTGATANNNNNACCGCAACGTCTTCTCAGCGACTGGCNAGCTYGAACCCTAGATANNNNGGNCGTTCGAGGANNCTGGCAGGGCAACCYYGGATNGTCCGTNNNNNYNNNAGACCGGCTTGTGGCATGNNGGCNNNNCCCCNNNGCNNNGACCTAGGCCGTCCGCAAACNGGCNNRYTGGGCCNNAGGCCGAANNNNNNNNAACNNNNNATNRCNTNACNRAGAACACTNNGCNNNTTTAGCAGGAGCCGGTGTNCATACCCGTACCACGCGCYTGRRGYTTRTCTGTCRGTNNNNNCANNRCGCCAACTNNAGAGTGCCNGGTNCTTTGTNTAAATGCCCTTTCCGGNNNCATTNRNTCGCNCGRTCCCGAGNNNNNNYATTARYYAGRCATTGYCTTTGCACGGCNACGGCCCGGTGTCACGACCTCGGGNYRGTATGTGGCAGACTTTTTNGATCCGACNCGNNNRCNNNNNNTGNNNGCCCCAGNNNNNNTACGCTGNNATTTATCNTRARGACGNTCGGGNNNNNCACTNCCTGCTRGCGGAGTCCGTYGRNGACCGGANCRNGTGATCAGNTTTTGTACCNNNNNNNNTNNAATGGAGGGNCATNNCATTCAGTCCGNNNNNNNYTCGCAGTGCNCATT

BrM46-1 RYGATTTACTCGTCATCTTANNGGCNNCCGGAGTCNNNNACCTAGACGGGNCNAGGANGTTNACCCACTGANCGNGATCTNNNGGCNATCACGCYYTNTGTCTANGGAGTCCNCTGCYCGGGANNCATCTTGGGTATGGNCCATNCNGCGNYTNCAATGNNNNGGCAGCRCGNANNNGNNNCNANNGTACAGANNNNTTTGGACCTGGCTTNGGNNCNGNNGATCTCANNATACGGCAGTCTNYAARTAGGTTGGNTANNNACNTCCNNNNYCNNNNNNNGARRTTTTNNACNANCGYGGATCNNTCGCAGGNCATTGACTATTYNNANTGNCCGTGGATNNYYNNNNNGATRRCTTAGNTTNNNGAACGGTGGAGCCNNNNNGNNNYTGCTAGNTTGGGGNNGGCGCCGNNNNNNNNNAACCTNRGTRCATTACAGTGCNGCCAGCTNGNCNNANAGCRCNCTCTCTNNNCTGNNNNNTGGGNNGCRTTTNGTTACCTTCNGCTNCNNNTGANNCCTANCRGANACAGYTAATNNNNCTNGTCTNNACTAGTGTGAGAGTCAGGAACCNACGNNTACNRTNNYAACACCNACGNNNNRNNNAAAGANNNNNTCCGGCTACCAAYGGTGGANNNNCNCCTNRRANYANNYGACNGCTGAGNNNNNNCAGGCCAATCTCCTCGATCTGCNNCNNTRTNYGCNNNCCCCYRYCNCCGCTTACGCNAGTRCNNNNNATANTGCGCCGTGCGGTGGGTANCAGGAGGCTCCTATNNCATGNGAATGGNNGGCACTACCGTANNCTAGCCGGYTGGTAGCTANNAACTGATATNNNGTGAGTACANNNCCCGCTCNNNGTNCGGNTACAACTNNCCCTGTACACAACGGNNNNNNYCNNNNGCTTGNGAATACGGATAAGTNCGGAAANNCGRNCGCCGTGAGCCGGTCAGTNACNAAANTTNNNNNTTGTAGTAANNNNNGGNAGCCATNNCNNNNANNTTCGGGCTAACCCACGGTGTAAAGGCGRGCCNCRNNNNNNNNGRCCGCTNNGCNNNAAGGGATAATNNGACGATTAGATTATGTCNNNANGNNCNNACCCCTCTNANCGCAGGTAACACGCTGTGGCTCCACYTTYGRNGGCNNGGGNNTTAGGCNCATCCGGGGGTCGCTGACTACNAGGGCCTAGNAACAAGAATGCCCANNNGATGGGGCCANNNNNACCGACAANTNCNNNNNNGGGRCRYCAGTTNGNTTNCTGNNNAGAATTTCTTTGGCCCGACGGTAACGCGNCNGYNYYYGNAAAGAYNNNNGYRNNNNNNNNNACGCCNGAAATGTCNNTNAGGAATYNACTNNCACTNACNYRYANGTRNCNNGCGCNTCRRTATCTCNNNGGGCNNCYNNCGCGAANGNARTGGGCTNGCCCCTNNNCRAAGGANNGGAAANNNNTCGGGACCCTGAGTACTCTGCGACCCTAGTGAACATAGAGCATCGNNNAGTTTNACATNTGGTYNCTNGGANCAACCGTCCTCTNNNTTACCCAGTGGCTTCNGCAGGANTTTGTACAGCCAGANAGYGNANNTCYYGCNCCTCCNNAGTGGTTTTTNNNNNANCCTNCCNACGTYGCTCCGAYGGAATTGCTCTGCNNGYGCTATCRNGAGNCCTATATCTGTTAACCGNNNNNAAACTGGTNNCGAACTCGNNNNACTCGAYANAAGGTCGGRNANNACTATGTGCANGTNGNNYRTATYANYYTGNNTCATTNAGATGGCANGGTCNNTTNNGATATCGTCCGCNNNGTTANYNGCYCGAANCCCGNNNNCNCNNNNNCCGANGAGTNACGGTNRGNCCGNRGNTTCTCGCTGGANACTTTCCAAAGCGTCCAGAGNATNNNTCGTTYTCTTTNCATTTACGCTACCCAATNNCGNNATNTNNNNNCAGCGTAGAGCTGGNGCCGCAGTARGTRTCTTNNAGANNNNNTCAAGCRNNNGCGGCGCNNCRCTANCNNGCCCCTGGCAYGGCCGCNNNAGCTAGCCGNCCCACYCRATNNNNCRCNTCCCCACGAGGYANGCGGNNNNAACANNGAGNNNYTATGACGGGGTTTGTACANNYTCCACNCNGNYCAGTCGTNNCGCANGGNCGGNNNNNNCTCCTAGACCGGTANGGGGYGTAATTGGRGTGYTCGCGNNYACCTRNNNAATACNNNNNNGCTARNNNNCGCATCGTGGCNAGNNCGCNNCGCTTCTGTAYTAANATTGGCAACGTAGATGCATGTGGANNTTTGCRGNNNGARNNNNNNNGATCGCGGCAACGGGCTCNNNNACCACATTAGNNNCNTAATGNGYNNNAGANTGATYGTTCGTACCCCTNNCCGCGNNNCGAAGCGNCTNAAARNNCCGCTACGCGAAAGCCTGTCAAGTNNNGYYCAACATCCGGCGCACGCGRAGNNNTCGNNNNNNNNGGNNGCNNNCGATTCGRGGNYTANNNNNACNNCTAGAAACGGCCGGTACRGGNARCCTGCTGGTAGGNTANGRCTTRATTAGCYCGCCCCGGNGCNATCGTCCTCGTCGTYAGGGGCTACTCYRGTGACATACATCGCATTNNACCCANGCANNNRCNNCNGNGNNTTNCNNGNNGCTARACGCCYACANNNNCTYGGNCTAAYACTNNNCYANANTYCNNGTCACCGNNNNNCACTNGGNGCCTGTTCCTGGATNNNCGAAAYGGNNNNNNNANNNTCGCCRTCTTACAAGANNNCNGAYAGNNTGCGGYGYTTTNTCCGYNNCANYNCTGAGCGTCGGTGNNNAYRCRCGACCGNNGGTTNNNGAACCGCACCAYCGGGCNTGCAGGGNRGGANNNNNNNYATCNTANNGYRNGTNNNNANNNGYNTCAGCCGAGAGAATGGRACCGCGATCACGNGACCCCTNACTNCGCAYGGACGCYAGYCYCAAGNGGNNNNNNNYANANCRATCCCGGGTCYNARRRTCGAANANYTCCTTNTAGNTGNNNTACCCCCCNNNNNNNGYNTANAGTTACCACGCTTACCGTAATGGGACCCACCGGGTTTNNGAGNATTAAANNAACNNCGTNGCTCCGNCACAGNGGTATTNAANYCTYRCRTCCTYTATNNNAGNCGGGGTRAAACATTCRRGYNCCGYYGGAYCATCNNGCACGCRTCACNNNNNNAANAGTCNNTRGRTATGTGAGAGGAGTTCGGGCACNNNTGTTTTTNNNNNNNNNNNTCAANNCGTNCAYCCAGCTCNNCAGACRNNGGNNNCTNNCNNNNNRNNNNNNNNNCCACTARNNNNNNNNNNNNGGGACGCNNNAGCATCAARNNNCAATCAACCNCGTGTTGNNNNGNNGAGAARARTNATATAATCNNTATTTNNCAGGNNTGGTCCNTGCCGRNNGAGACNTNACCAANGANAGTGTYCGCGAGATGCGCTACNTGYCCNTTGTNAGTCTACAYTNNAYAATANANTCRNNNNNNCCTCATCCCGACCGTACGYNNNAGCCTCCAGAACNNNNNTCGCCGGCTNCCCNGNCGCCAGCCTCGCTCAGCCTGAGCTACTGTATNNNAATNGNCTGTYCTCATTGTNGYTGNNNNRYCCCTTTTANTCTCACCNNNNNNNCTCACAGGNARTNTGRGAAANYNTACACGNNGTAAACCGTAGGNNNNGGTTACACAGCCTGAGNGGCCNGCCYNNNNNNNNTGANTYGCGCAGNCTCGNYCTNNATCGGGTTCATCGTGNNNGGNNNNNNCCGCAGGCGGCNTGNCTGANCGGAACTACGTCGGGCCCNNNCNACCTACGNNNNNNNNNNNNNGNNNNNNNNCYGYTCNNNGNNACTGATCCAGGGACTTRGANGCCNTTGNNNGCACNNNNGNNNAGATTGTAYCANNCCACACGNCGGNNNAGGACGCCAGCNNTNCCTTACNTTAGCNNTCAAGTTTCCTNNNNNTGCCNCNNNGCAANCATGCGTAANRGGATGNNRTCATGGCRGNGNNANTCAGGYGCGGYCTCTGNTCCCNAAAATTGCNNTNNNNNNTGCGNNCGTCGAATGCACCRCGGCATTGCNTRNNNNNGCTGGCGNCCNTNNNTGYGTYCATGGTGTTRTGAGCTNNCCTTAAGCGCNNNNNGAAANCGCCNCGATNATGCGTAGAAGGCGCRAATGTGAGCANNGCGTTARCNNNNNNCTTGNANGCGNNNATATNACGGCNNNNTCCNATACNNNNYTAGGTACGGCYTCACANNNNCTRTACCCCTGGGGGACYGCCNTYGNNNNNNNNNACGGGGGGNGANNNNACACYYTGTTGTCGCGGGCTCNCACGGTTACNRNNYTGTGRCRTCNCNCTAATCNAANGAGANNNNNNCTRYCTCNNNNGANGNCRGCATGGTGTCGNNTNACCCTTNATAYNNATCGCAATNNAACGAAGCCAGGCGYCNNAYNACAGAANNNNNNNNAAACTANNNNCGCNGTACGGGACGACGTNNNCACCACGGRCCCTGATCGTGTNNCGTAACNACACARCATCNNNNTCTGGCTTTACCCNGGTRCAGACGTCTCAGTCAGCNGGGATYNCYRNNNNAATARNNCGAGNGYCGNRNNAACCCGANNCTNNNNAGNCACCCNNAACCNGRAGGGCACCACACAYCTATATNTCACTCAGGGCGGAAGCGCCAAAAYNCNCARTYCCCANNTGAGGTCAGNNCTGNNNNCTAAGGTAAGCNNNNNNNNNNNNNGGGAGGCCGGATNNNNNNNNCCNRTCCNNNNNCYYRANNCGANNGTAAAGNNCGYGNNGAATTNGTTAACTTGAYCCRNNRNNNNNCRTGTYGGYNCACAGCTGCCTCAGTACAATGGNACTTCYAAGGTTTGYCTCCAAGNNGCNNNAGTTAGNNNNNNNRCCCTCRRTATGAYAYANNCTACAGCANNNNGNNNNNNNNNACTNNCCNNNATTTGCAYNNACNNNNNNCNCTNNTTTTNNNNNGNCTGGCYNNNNGAACGGAGGTTCTGNNNNNNTAANGRNTAGCNNTCACRGAGGTTACCGGCTGTTGTATGNCRYGGACTGTGCNCAGATTGTCCTCCAAGTGGTAGATAGACCCTTNCYNNNNNNNNYRAGAYATNATCGYAATCGNCTCANGTCCTNNNNNNNNRRGATNNTGATCGCTNRANCCCNCCTNNNAAAGCTACCGCAACGTNTTCNCNRCGACTGGCTNNNNNNAACCNNNNATAACGRGGANGTTNRRGGACTCTGGNRGGGCNTYCTTNNNNNGYNNGTATGATCCTTARACNGCTTTGTGNNNNNGGGGCAGRGNNNCTANNNNNNNNNCTGGNCANTNNNNAAACNGGCNARYTGGGCNNNNNNCNNACAGGGGCGCAACACAGCTGGRCTTCANNGANAACACYNNGCTATTTTAGCAGGAGCCGGTGTGCATACCNNNACCANNNNNNTGAGGCTTATCTGTCRGTAARGYNNTGGCGCCAACTRGNGNNTGCCNNNTACTTTGTATAAATGNNCTTTCCGGGAGCATTNGAACGCNCGATCCCGAGATCAGCCRYTRRYYANNNATTGCCYNYGYACGGCNANGGYCCGGTGTCANNNNCTCGGGTTAGTNNNTGNNNGACYTNTNNGATCCGACTCGNNNRCNNNNNNTGNNGGYCNYAGYNNNNNNACNCTGGNAYTNATCNNNNNNNNRRNNNNNCAAAGCRCTACCTNNNGGCGGAGNNNNNTRRNNNNNNNNNNNANNNNNNNNATTTTGTACCCGNNNNYYTACANTGGCGRRTTATCGCATTCAGTCNNACGCGRYCTCGCANTGCACATT

BrM48-1 RCGATNNNCTCGTYRYCNTNNNGGCNNCCNNNNNNNNNNACNNAGACGGGTCTAGGNNGNNTACCCACTGANNNNRANNNACTGNNNNTCANNNNNNTTGTCTNNNNNGCCNNNNNCNNGGGAYCNNNCTTGGGTATGGGNNNNNCTGCGCTTACAATGGCCNGGCAGNNTNCAGNNNNNNCNANNGTAACARNNNNTTTGGACCNGGCNNTGGAACGGTAGATCTNNCAATACGGCAGTCTGTNNNTAGGTTGGGTAYGNAGATCCTAGGCCNCANNNNRAGGTTTTNRACRATCGTGGATCACCCACAGGNCATTGACYATNYNCNNTGYCCGTGGATAGNNNGGTTGATGATGTAGNNTNNAGAACGGNGGAGCNNNTNNGGRGYTGNTAGNTTGGGGCGGGCNCCGCAGGTTCGGNNNCNNNNNACATTACANTGCYGCCAGCTRNNCNYNAARNGCYCTNNNNNTACTGYTGNGNNNNCTGCNNNNNGTNNNCTNNNNNTNYNAAYGANNCCNANCNGANACRRCNAANNANCCTNGTCTARACTCGTTTAAGNNTANNNNNNCGACGNGTACNRTNNYANCACCRACGGGGCGAGRAANGAACACCTCCGGCTACCANCGNNGGACNTTCCCCNTAAAACAAGYGACNGCTNAGTGTGAGCARNCCAATCTCYTCGATCTGCTCCCTNNNNNNNAAGCCCCNNNNNCCNNNNACNANAGTNNCTRGYNNNYTGCGNCGTGCGGTRRGTNCCARGTGRNTCCNNNNNCATNNGAATGNNNGGNACTACCNNRNNCTRNNCGGCTGGTAGCTANNAACTGATATTATNNGAGTACATGCCCCGNYYNNNNNNCNGNNACNGCTAGCCCTGTACANNNCGNAATTGTTCTTAGGCTYGCGANNNNNNNNNAGNTCGNANNAACGGGCGYNNTGRNCCNNTCANNNNNNAAANTTNNNANTNNNNGTNRNNNNCGNTAGCCNNNNCTCNTANNNNCGGAATAGCCCGCGGTGTAAAGNCGNGCCNNNTCGGCGAGGGYCGCYTNNCNNNRAGNGATANTCAGACGATTNNNNTATGTCAGRNNNNNNNNNNNCCTCTTNNCGCAGGNNANACGCTGTGGCTCCANTTTNNNCGGCCGGGGAGNTAGGCACGTCNNNNGGYCGCTRANTNNNAGGGNCTAGNAACAAGAATGCCCANANNNNNGNNNNANNNACACCGACAANTTCNNNNNNNNNNCATCNNTTNGYTTNCTGCAGRGNNNNNCTNTRGCCCRNCGNNAACGCGNYNNNNNYYGNAAAGNNNTAGRYNTGNGATCRGACGCCNGAAATGTCNNTTAGGAATTGACTNNCACTGACNNNYAAGTRNCNNNNNNNNNNNNANCTCNNNGGGCGGCTNNCGCNNNTGNNRTNNNNNCGCCCNNNGACRAANNNRNGGRRANNNCTCAANNCCCTGNNNACTCNNNGACCCTARTGNNCATAGAGCNNCGACCAGNTTNNNATATGGTCTNNNGGAGCARCCGNCCTCTGACYTACCCAGTGGCTTCCGCNNNNCTTTGTACANNNNNNRAGNGRRNCTCCYGNNNCTCNCAAGNGGTTTTTCAAGTAGCCNNCCCAGGTCNNTCCGACGGAATTGCTNNGCNNGTGCTGNNNTGAGCNCNNNATCTRTTAACCGGGTGGGCACTRNTNTCGGNNNCGTAGTACNNNNNNTNNGGTCGGNNRTCACTATGTGCAGGTGGNNTGTANTAGTCTGCYTCATTNNNNNNNNNTGGTCTGTTNNRATATCGTCCGCTTGGTTANNNGNNCGNNNCCCGTCCYCACGCGTCCCGAARAGTGACNNNNRGNCNNNGGATYCTNGCTGGACANTTTCYAAAGCGTNNNNAGGATACTTCGTTNTCTTTNNNNTNNNNCTAGCCNATTTCGNNNNNNGCCGGCACCGCAGAGCTNGNRCCGCAGTNNNTRTCTTGCAGANNGGCTCAAGYNTCGNCGGCGCGTCAYNATCANGCCCCTGGCATNNNNGCGACRGCTAGNCGTCNCACCCAATGGNNCGYGTCCCCACGAGGYAGGCGGNNNNNNCACGGAGNNGNNATGANGGGGTTTNNACANNCTCCACNCGRTTCAGTNGTTGNGCAAGGRCNGNNNGGGCNCCNNGACCGRTNTGGGGYGNNATTGGRGTGYTCGCAAATACCTRNGGAATACNCNNNNGCTANTNNNYGCANNGTGGTNAGGGCNNNNNGCTNNNGTATTAACATTNNNNACGTANNNNNNTNNGGAACTNYGCAGTGGGARGGAGCGAATCCATGGCAACGGGCTCTGCGACCACATTAGTGACYTAANNNGYNRRAGANTGATTGTTCGTGTCCNYCCCCGCGGCGCGNNNAANCNNAAANTGNCGCTACGCGAAAGCCTNTCAAGTAAGGCCCNNTCTCNNNNGCACNYRRNRNNGTCGCGCCAGYCNGTTNNNNNYGANYCCANNANTAGCACTNCTNCTNNNNNCGGCCGGNNNANGAAGCCNGCTGGTRGGCTNCGNCTNGATTAGCCCGTCCCNGAGCANNNGTCCTYNTCGTYAGGGGCNNCTNNNNTGNCNTACANCGCAYTNNACCCACGNNNCAGCGGNNNNNNCTTRCNNNACNCCRAANNNNNACANNNNCTCGGGCTAATACTTNTNCANACYYCTGNTCNCCGGTNNNNNCTNNNNGCCNNNTCCTGGATAACNNNNNNGGGCNNNNNARGNTCNNNNNNNNACAAGACTCCCGACAGNNNNNNGCGTNTTATCCATGTCAACGCNNAGCGTCNNNGNNNNTGCACGACNGCCGGTTTATGAACCGCACYACCGRNNCTGCRGGGGAGGAGACGCGGCNNNNNNAYGNRNGTGTCGACYTGNATCAGCCGAGGAAATGGGACCGCGNNCACGYGACCCCTTACTCCGCANRGACGCNGGCCYCAAGCNNGGGTGTCTACNYNNATCCCGGGTCNNAAAGTCGAANATTTNNNTCNAGNNNAAANNNNNNNCGNNNNNNGNNNNNAGTTACCNNNNTNNNNNNNATGGGACCCACCGGGTNNGCGANNNNNANAGCNACCCCGTTGNNNNNNNNCAGCGGTATTAAANNNNNNNNNCCTNTANGGNAGACGGGNNNRARCATTARRNCANCGTCGGACCATCNNNCNCGCATCACNNGNNNNATAGTCNCTAGRTATGNGNNNGGAGATCGAGCACCAGCGTCTTGACCTCGAGTTTNNNNGTTAGCNNNNCNGYTCCGCANNNNNNNNCRRCTCANNAGCGNTNNCCCNTNCCNCTAAAGNYNAYTNNNNNNGACGCNNNNNNNTCAAGGTANAATCANNNNNGTRTYGGYCRGNNGNGAAGAGTANNNNAATCNNNNNTNCGCAGGAATGGTCCANNCNGNAGGAGNYNNNNNCANNGAANGTGTCCGCGAGNTGCGCTACTTNNCCNNTGTCAGTCTACNNNGNNNAANNNRANCGTCGCTGCCTNNNNNNGRCCGTACGCANNANNCYCCAGAACYNAAANCGCCGGCTACCCCGACGCCAGCCTCGCACANNNNNNNNNACTGTATCTAAATCGACTRTYYYCNNTGTAGNNNNNNNNNCCCTCTNANYCTCNNCGCNANNANNCACAGNNARTTTGANNAATCATACACGNNNNANNCCGTAGGNCGANNCNACNNNRCCTNANGGRCYANNCTANNNNNNNTGAGTNNNNCAGNCTCGCYCTRRNYYGNNNNNNTCNTNAGNGGANCGGTNNNNAGGNNGCCCAYNNNATYGGAACNNNNNCGGGNNYCTGCGATYTNNCCGNNNNNNNCCGGGCTYYGTGGCCGTTCNNNGNNNCTGATCCNRGGANNTAGANNNNNNTGTANNNNCNNTGNNNNAGRTTGTNNNNCGNNNCACGNCGGCANANNNNGCCAGCGGYNNCTTGTCTTAGCCGTCAAGTTTTNNCGTNTTGNNNCNNNGCAATCATGCGTAANNGGATGRGGTNATGGCRGNNCGAATNAGRTGCGGTCTNNGNNNNNNAAANNNNNNNNCATNNATNCGYACGTNNNNNGCACCGCGGCATTGCTNNCGGRTACYNNNNNCCNNCATNGYGTTYATGNNGTTNTGAGCTNNCNNNNNGCGCGGACYNAAATCRCCNCGATCATGCGTNNNNNGCGCRAATNTGNNNNAANNNNYARCNNGGANCNTGNANGCGNGAANATTNNNNNNCANTNCTATNCAGGNCNAGGTANGRCTNNNNANTGGCNNNNCCCCTGRGNNACTGCCNNNNNGCAAATTCACGGGGGGGGAGCCAANNNNCTGTTNNNNNNNGCTCNCGAGGTTACGGANTNGTNGCGTCNNCCTAAYCRAANNAGARTTCYRNNNNNNNGGAGGNNGCCGGCATGGTNNNNCTNCACYNNNNATAYGTATTGYANNNNAACGAAGCCAGGCGCCNNNNNNNNNAAAGATGACAAAACNAGATACGCAGTNYGGGACGACGTNGCCANCNCGNNCCNNGANNNTGTNNCGNNNNRACNNNACNTNAGTTTCTNNNNNNACCCNGGNGCNNNNNTCTCANTCAGCAGGGATNANTAGCCRNRTAAGCCGAGNGYCGNGCCAACCCGAGTCTAGCRNNNNGNGNACNNCCCGRAGNGCNNNNNACATCTATATTTCNCNNGRGGCGGCAGCGCCNNNACANTCAGTTCCCACCTGANNNTAGNNCTRNRAYNTAAGGTAAGCCCCAYRNGCNATTGGNNNNNNNNNNTTGGNNNTCCRGTCCTATTNNNNNNNNCGANNNTAAAGTACGTGAAGAATTTGTTARCTTGNNNNNNTAGACCGCANNNYGGYACNNNNNNGCCTCNGTACNNNGGNACTTCCAAGGTTTNCNNNNNNNCGGCAGGRRTTAGTTGGNCCNNNCNCAATATGAYAYAANNTNYANNNARCCGNTNNNNGGCACTCNCNNNNATTTGCATCNACATRCGTNNCYNTCNNTYYYANGNNNNGCCGGCGNNNYRNNAGATCTCGATCTGTRATRAANNNNYNNNNCAGANGTNRCCGGCTGTNNNNNAGNNNNRACTGNNNNCAGATTGTNCTCCAAGNGGYNGATAGACCCTNNCTTAGGGNGTYGGGAYANAATNNNAATCGCNNCAGGTNCTAGNNNNNNGGNNNNCTGATCGCTNNNNCCCACCTGNNNNNNYTACNGNTACGTNNNNTCAGNNNNTGGCTNNNNNNAACCNNNNATANNGAGGGTGTTAAGGGACTNNNNNNNNNCATCCTTNNNNNGNNNGTANNNTCCTTANACYGNNNCGTGGCANGGNGGCAGAGCCCNNGGGCTGAGACNNAGGCCGTCCNCAAACNNGYCAACTGNRNNRNAGGCCGACNGGGGCGCAACRYARCNNNGCTNNNCNRAGAACNNNNNNNTATTTNNNNNNGAGCCGGNGNNYNNGCCCGTACCANNNNCCTGAGNNNTATNNGTCRGTAAGGCCATGGCGCCAACTRGNNAGNNCNCGGNNCTTTGTATAAATGNNNNTTCCGGNNNCNNNNGANNNNNCGGNCCCGAGNNNNNNNATTNGTTAGGTATTGCNNNNNNNNNNNANCGGCNNNNNGTCACRNCCTCGNNNCGGTNNNTGGCANACYTNTTAGATNNNNNTCGNNNACCCANNNTGNNNNCNCCAGCATCCTTACRCTNGGATTTNTYGTNAARRYGANNNNNCANAGCACTAYNTRCNRGCGGAGTCCGTTNNTGAYCGAAACGNGNGAYCAGATTTNNNANNCGNNNNYYYAYAANGGCGGATNNNYRCANTCAGTNNNACCCGGCCTCGCANTGCRCGNN

BrM50-1 GTGATTTACTCNTCGTCTTAATGGCCGNNGGAGTCGTCGACCTAGACGGGTCTAGNRGGNNTACCCACYGACYGTGATCTACTGGCCATCACGCCTNTTGTCNNNGGNGYCNNNNGCNNGGGAYNCATCTTGGGTNTGGGNNANNCTNCGNNNNNAANNGCYNGGCAGCGYRCAGGANNNNNNNNNGTANNRRNAGGTTTGGATTTGGCTTNGGGACGATANNNNNYACAATACGGCAGTCTNYAAGYNNGNNNGGTACGCNCATCNNNGGCCTTGTAAGNNAATTTTNRANNANYGYGGNNCACTCGTTGGTCATYRNCTATTCCCNATGNCCGTGGANAGTTTGGTTRATGRNNTNGNYTNNCGANCNNTGRNGCCNTTCTGNAGCTGCTAGRTTGGNNCGGGCGCCGCAGGYYNNGAACCTNRGNRCANTACAGTGCTGCCAGCTAGCCTTCAAGNNCCCTCTCTTNNNNNNNNNNNGGNCTGCATTTGGTTANNTTYCGCTACNAAYNNGCCCTANCRGACACAGCTAATCATNCTNGTCTAAACTCGTTTAAGAGTCNNNNNNCGNCGCGTACGRTTTYNANNNCNACNGGGCGNGGAAAGAACRCCTCCNNCTACCAACAGTNNNCGTTCCCCTAGAAACANGCGGTCGCTAAGGCTGAGCAGRCCAATCTYCTNNATCNGCTCCYYTRTNYGCAAGCCCCTGTCGNNGCTTACGNNAGTACCTNNCNNATTGCGTCGTGCGGTGGGTACCNGGTGNNNCCTNTCNCATNCGAATGGTNGGCACNACCAAGGACTAGCCGGCTGGTAGCTAGGAACTGATATTAAGTGAGTRNATGCCCCGCTYGNANTNCGGNNNNNNNNAGCCCTGTNCACAACGNAATTGTNNNNNNGCTYGCGAATNYGRNTRNGTTCGGANNAACRGGCNTCTTGRNCCGNTCAGTYACTAANGTTGCAAATTGTAGTAANNNNCGNNAGCCRYGGCTCATAAATTNNGAATAGCCCGCGGTGTAGGGNNNNGCCRCRNCGGCNNNRRCCGCCNNRYNGCAAGGGACAATCAGRCGATTAGATTATGTCARAAGGAACGNACTCYTCNTANCACACGTCNNACGCTGTGGCTCCANTTTYGRCGRCCGRGGNNNTNGGCACGTCCGGGGGCCGNTNACTACNAGGGYNNAGRAACAAGAATGCCCARACGATNGGGCCAGCCACNNNNACAAGTTNNNNNNNGGGGCATCAGNNGGCTTGCTGCNNRGNATTTCTTTGGCCCGACGGTAACGCGNCRGYNNYYGAAAAGACATANRCNYNNNNNCAGACNCCNGRAATGTCGATTAGGAATNNANNNGCACAGNNNTACACGTATCAGGCGCATCGGNATCTCTCCGGGCGGCCRRCGYGAANGNNNTGGGCTNGCCCCTCGACGNNGGAANGGAANNACNTCNNGACYCTGANNACTCNNCGACCCTNNNNAAANNNNAGNRTNGNNNNNTTTRNNNTATGGTCTCTGNRNGCAGCCGTNNNNNGACNNACCCAGTGGCTTCNGCAGGACTTTGTACAGTCACTAAGYGAACCTCCCRCACCTCCCAAGTGGTTTTTCAAGTAGCCTACCNANGTNNCTCCGACGRAANTGNTNNNNNNGTGCTRTYRTGAGCCCNNNATCAGTAAACCGGGTGGGCACTGGTTTNGRACTCGTAGTACNCGNTANAAGGTCGGGGATCACTANGTGCAAGTNNACCRTATTAGYCYGCTTCATTNAGATGGCAYGGNCTGTTTGGATATCGTCCGCTTGGTTANNNGCNCGNAGCCCATCCNCNNNNNTCCYGAAGGGTGNNGNTGNGNNNNNGGATTCTNGCTGGACANNTTCCAAAGCGTNNNNNGRNTACTTCGTNCTCTTTTCATTTACGCYACCCNNNTTNNTAATATGCCGGYRNNNNNGAGNNGGNNCNNNNNNGGGTGTCTTGCAGAACGGCTCAAGYATCGGCGGCGNNNCACTNTYNAGCCCCTGGCAYNNNNNNGACAGCNRGCCGTCCCACTCGATGGNNNGTGNCCCCACGAGGCANGCGGGAYGAATNNNGNRACGYTNNNNCGGGGTTTATCCACGYYYCANNNGGTTCAGCCGTTGYGCAAGGGCGGTTCGGGCYCCYARNCCGGTATGGGGCGTAATTGGNGTGCNCGCAAATNCCTRNGANGTACNNNNNNGCTAATCGGCGCATCGTGGNNAGGGNGCCTCGCTTCTGTATTAACATTGNCAACGTAGATGCRNGYGGAACTTYGCRGYRRGARRGAGCRARNTCGCGGNNACGNGCTCTGCGNNNNCATTAGTNANNNNNNNCGNNRRNGANTGATCGTTCGTGTCNNYCCCCGCGNNGCGAAGNNNCTNAAAGGCCCRCTACGCAAAACCCTGNNRNGTAAGGCCCAANNNNCGGCGNRYNNAGCNNNGTCGCGATAGCGGGTTNNAGGNNNNTCNANNANNNRNNCAANTNCTNGARACNGCCGGNNNARNGAACCYGCTGNNAGGTNNNAGTTTNNNNNGCNCGTCCCGGNGCAATCGTCCTTGTCGTTANNNNCNNCTYCGGTGNCNTACATCGCATTANNNNTATGCAGCGGCAGCAGCGTCTTACAATACTCTAGACGCCTNCAGCATCNCGGGCNNATACTTCTCCTCTCTCCYRRTCNCCGGTGAGCACTAGNCGCCNNNTCCTGGATACTCGGGACGGGCCTCCAAAGCTCGCCATCTTNNAAGACYCCCGACAGAATGCGGCGNNTTNTYNNNGTNNNNNYTGAGCGTCGGTGACGNTGCANGACCGCCGGTTTATGAACCGCACCACCGGRCTTGCAGNGTGGGAGACGNNNYNNCNNNGCGCAGGTNNCGNYCNGYTTCAGCCGAGGAAATGGGACCGCGATCACGTGACCCCTTNCTCCGCATNNACRCNGGYCYCNAGCRRGGGTGTCNACACCNATCCCAGATCTGAAAGTCGAACAACTCCTNCTAGCCGNNNNNNCCCCCGATCCGTGCATAAAGTTACCACGCTTACAGTAANGGNACNNACCGGGNTNGCNNGRATTANANNAACTCCGATGNTCCGTCACAGCGGTATNANANNNNCGCGNCCTTTATGGGAGACNNGNTGAAACATTAGAGCACCGTCGGACCGTCYNNCCYGYNNNNCGTGTGTANNNGTCNYTRGGTATGTNAGAGGAGTTCGGGCACCACTGTCAGGACCTCGAGTTNTCGGACTAGYCAYNCNGCTYCGCAGACGAAGGCAGCTCTCANGCGRTNNCCCATCYCNCNARNNGYNACTCCAGATGACGCGAAAGCCTCAAGGTANAATCANNCNCNTGTTGATCAGNCGARANNNGTAANNTAAYYCCTATNNCGCNGGAATGGTCCRTGCCGGAGGAGACATCNCNANGGANAGTNNCCGCGAGATGCGCTACNTNNCCTTNNNCAGTCTACACTNCATAANNNNATCGTCGCTGCCTCRTCATGACCGTACGCACANNNNNNNAGAACCYNANTYGCCGGCTNCCCCGACGCCAGCCTCGCTCAGCCTGAGCTACTGTATCTAAATNGACTGTCCTCATTGNNNTTGNNRGRYCCCTTTTAAYNTCGTAGCTATAACACACNGGCAGTTTGAGAAATCNTACGCGCGGTNAACCGTAGGGNNANNCCACACAGCCTANGGGGCCANNCTATTGTGGANGANTCGCGNAGACTCGCTCTAAATCGGGTTCANNGTGRGCGGNCNGGTNNNNAGGCGGCCYRCCTGATCGGAACYRCRTYGGGYNCCTGNGNNYTAACCNTCCNCNNNCNGGCGCCNNNNCCGTTCCGTGTANCTGNTCCNGGGACTTNNNTGCCCNTGTAAGCACNATGGAATAGGTTGTNNNNCGCCACACGNCGGYNNNNNNNGCYTGCGGTTCCTTACTNYAGNNGTCNNNNNNCCTCGTGTTGCCCCCAGGCAATCATGCGTAACGGGATGGNGTYATGGCRNCGNNAATCAGRTGYGGTCTCTGNTCCCAAAAACTGCAGTCATNNNTGCGNNNNNCGAATGCACCGYGGCRTTGCTTGCGGGTRCYGGCGRCCATCATNGTGNNNATGGTGTTRTGAGCTCTCCTTAANNNNGGACCNAANNCRNNNCGATCNTGCGTAGANNGCNYRNATRTNTCCAAAGCGTNAGCACGGANYTTGAAAGNGCNNATATTACGGCTCACNNNTATAYNNNTYTAGGTACGNNCNNNNACNGNNTRTATATCTGGGCGACCNNCGTTGCGCAGCTTCACNGRRGGNNNGCCAACACTCTNTTNNNGCGGGCTCNCRCGGTTACTAANTAGTGGCGTYNCCCTAANNGAAGGAGAATTCTGCTACCTCGGAGGAAGCNGGCATGGNTTCGCTNCNCTCTTCATNCGNATTGCAATRTANCGAAGCCAGGCGTCNNATNACAGAANNATGACAAAACNAGATANGCANTNYGGGNCGACGTNNNNANCNCGNACCCTGATCNTGTTTCGTAACAACACANCNNCAGNNCTNGGCTTTACCCGGGTGCAGACGTCTCAGNNNNNAGGGATTANCGGCCGNRTAAGCCGAGNGYCGRRNNAACCCGARYCTAGCGRGTCGTGAACNNCCYGAARGGCACCACACAYCTATATNTCNCTCRGRGCGGNAGCGCTTAAACANTCNGTTCCCACCTGANNNCAGCCCTNARATCTAAGGTAAGCCCCNCATGCGANNNNNAGGCTGAANCCGGNNNNCCNNTCCTATTCYYYRAACCGACCNTAAAGTACGTGAAGANTTTGTTANCNNNACCYGCTAGRCCACNTGTYGGYACACAGCTRCNTCRGNNYNNNGGNRYYNCYAAGGTTTRYCNCCAAGNRRCAGGNNNNNGTTGRNCNACCCTCAANATGAYAYNAYNTACAGNAAGCCGATAAAAGGTACTCGCCAGCATTTGCATCCACATGCGTCCNTGNCTTTTCCATGNCTGGYTNNNNGAAYRGARGNTCTCGATCTGTGATGGCTAGCCGTCACRGANGTNRCYGGCTNTYRYRTAGCGCGGACTGTGCTCTGATTACCCNNNAAGTTGCAARTAGACCCTGRCTTAGGGCRTCGAGAYATNANCGYAATCGCCNCAGGTCCTAAGAACGTGGGATCCTGATCGNTNNAYCCCACCTNTCAARGCTACCGCAGCGNCTTCTCANNNNNTRRCTAGCNNNAACCCTAGATAACGRGGRTGTTAAGGGACTCTGNCAGGGCNTCCTTNNNNNGTNCACGNNNNNCCAAGACCGNNNNNNGGCATRGAGGCNGAGCCCNNNGGCTGAGCCCTRGNCNNNCCGCAAACNGGCCARYTNGGCCAGAGGCCGNNAGGGGCGCAACNNNNNNNRRCTTTACTAANTACNNNGAGCTATTNTAGCAGRAGCCGGTGTNTATGCCNNNNCCAYNCGCTTGGANNNTNTCTGTCRGTAAAGYCATGNNNNNAACTGAAGNNTGCCNGGNACTTTGNRTAAATGNNCTTTCCGGGAGCATTNGATCGTNCGGTTCCGAGNNNNGCNATTANNYAGATATTGCCCGCGTACNNNAACGGCNNGGNGTCRCGACCTCGGGTTAGTATGNNGCANACTTTTTAGATYCGACTCGATTGCYCNNNNYGGARTCACCAGCATCCTTACGCYGGGGTTTANNGTAAAGACGATCGGGCAAAGNNCTACCNGCTGGCGGAGNCCGNYGRTGNCCRNNTCAANGGACCAGATTNNNNNNNTAGTAGTCYANARTGGNGRGTNATYRCNTTCNNNCCGACGCGGTCTCGCANTGCGCNNN

BrM51-1 GCGNNNNNCTNNNCANCNNNNNNNNNNNNNGNGNNNNNNNNNNNNNNNNNNNNNNGRNGNNTACCCACTGAYYGYNNTCTACNGGCNANCNNNNNNNTNNNNNNRRGANNNNNNNNNNNGGGANCNNNNNTGGGTACNNGNNNNNCTGCGNNNNCAANNNNNNGGCAGCGNGNNGGANNNNNNANNNNNNNNNNNNNTTTGGACCNGGCYYNNNNNNNNTANNNNNCNCANNNNNNNNGTNTNNNNGTANGTTNGGCACGNNNATCNNNNNTCNNNNNNNRANNNNNNNNNNNNNNNNNNNNCACNNNNNNNNCNNNNNCNNNTCCCAATGNNNNNNNNNNNNNNNNNNNNNNNNNTAGATTGANNNNNNNNNNNNNNNTTNNNNNNNTGNTANNNNNNNNNNGGCGCNNCAGGNNNNNNNCCTCNNNNNNNTACNNNNNNNCCAGCTNGNNNNNNANNNNNNNNNNNNNNNNNNNNNNNNNNNNGCNNNTNNNTAYNNNNNNNNNNNNNNNNNNCCTNGCGNNCNNNNNNANNNATCCTCGNNNNNACNNNNNNNNNAGNNNNGAGCNNNNNCGTACNNNNNNNACACNNACNGGGCNNRNAAAGNACGNCNNCGGCTANNNNNNNNNNNCANNNNNNNNNNNNNANNNNNNNNNTNNGNNNNNNCARNCCNNNNNNNNCNATCYNNNNNNNNNNNNNNNTGNNCCTGCNNNNNNNNAYNNNNNNANNNAGCNNNNTGCNNNNNNNNNNGGGTNNCNNNNNNNNNNNNNTGCATNCGAATGGNNNNNNNNACCNNNATNNNNNNNNNNNNNNNCTANNNNNNNATNNTATNNGAGTACANNNNNNNNTNGCANNNCRGNNNNNNNNNGCCCTGTNNNNNNCGNANTTGTTCTNAGNNNNNNNNNNNNNNNNNNNNNCGGANNAACGNNNGTNNNNNNNCNGTCAGTNNNNNNNNTTNNNNNTNGNNNNAACTCGNNNNNGCCATNNCNNNTANNNNCGNNANNNNNNNCGGNNNNAAGNCGACCCGCNNNNNNNNNAGCCGNNYNNCNNNNNNNNNNNNTCAGNCGATTAGATNNNNNNNNNNNNAANNNACYCCTCTTNNNNNNNNNNACNNNNNNNGGCTNNNNNNNNGNNGRCNNNNNNNNNNGGCNNNNCCGGGGNTCGNNAAGTACNAGGGCNNNNNNNNNNNNNNNNNNNNACGATGNGGCCNNNNACACCGNNNNNNNNNNNNNNNNNNNNNNNNNNCGTTTNNNNNNNNNNATTTNTNTGNNCCNNNNNNAACGCNNNNNNNNNNNANNNNNNNNNNGNNNNNNNNNNNNNNNNNGANNNNNNNNNTNNNNNTNNNCNNNYACTGACGYRNNNNNRNCNNGCGCNNNNNNNNCTCTCCGGGCNNNNNNNNNNNNNNNNNNNGGCTNGCCCNNGGANNNNGGANNNNNNNNNNNTCNNGANNNNNNNNNNNCGCCGACCNNNNNNAACATNGNNNNTNNNCCNNTNTNNNNNNNGGTNNCTNNGANNAACCGNCCTCTNNNNNACNNAGTGGCTTNNNNNGGANNNNNNNNNNNNNNNANNNNRANNNNNNNNNNNNNNNANGTGGTTTTTNNNNTNNNCTANNNNNNNYGNTNNNACGNNNNNNNNNNNNTANNGCANNNNNNNNNNNTNNNNCTNNNNACNNGGNGGNNNNNNNNNNNNNNNNNNNNNNNNNNNNTANRANNNNGGNNANNNNTATGTGCAAGTGGNNCANANTAGTCTGNNNNNNNNAGNNNNNNNNNNCTGNNTGNNNNNNNNNNNNNNNNNNNTTGGNNNNNNNCCCGTCCNCNNNNNTCNNNNNNRGTNNNGGTNNNGNYRNNNNNNCNNNCTGGACANNNTCCANAGCNNNNNNAGGNNNNNNNGNNNNNNNNNNNNTNNNNNNNNCCANNTTCGTAATATNNNNNCAGNNNNNNNCNNGNNCCNCANTNNNNNNNNNNNNNNNNNNNNNNNNYNNCGGCGGCGCRTNNNNNNCNNNNNNCTGGNATNNNNNNNNNAGCNNGCCNNNNNNNNNNNNNNNNNNCNTCCCCANNNNGYNNGCGNGACGNNTANNGAGNNNNNNNNNCGGGGNNTNNNNNNNNNNNNNNNNNNNCAGYNNNTANNNNNNNNNGNTTCGGGNNCCNANNCCGNNNTGGGGNGNNATTGGNGTGNNNGCRNNNNCCTNNGNNNNNNNNNNNNGCNNNNNNNCGCATCNNNNNTANGGNNNCTNNNNNNNNNNNNNACATNGGCAANNNNNNNNCRNGTNNNACNNNNCRGYGGGANGGNGCGANNNNNNNGNNACGNGCTCNGCGNNNNCATTAGNNNCYTNNNNNNNNAAAGAATNNTYGTNCGTNNNNNNCCNNNCGNNNNNNNNNNNCTNNAANTGCNNNNNNNNNANNNNCTNNCANGTAANGCCCAANNNNCGGCNCRYNNGAANNNNNNNNNNYAGNNNGNNNNGANNGANNNNNGGANNNNNNNNNNNNCTNNNNNCGGCCGGNNNNNGNNNNNNGCTGNNNGGNNACGANTNAANNNGCYCNNNNNNNNNNNNNNNNNNNNNTCGNNNGNGNCTACNYNNGTGGCGTNCANCGCNNNNNNNNYATNCANNNNNNNNNNNNNNNNRCNNNNNTNNAAANNNNNNNNNCGTNNNNNNCTNNNNNNTCTNCNCANTCCNNANNNNNNNNGAGNNNNAGNNNNNGTANNNTGNNNNNNNNNNNNNNGCCTCNNNNNNNNGCNNNNNNACNNNACNNCCGNNNGNNYGCGNYGNNTNNNNNNNNNCNNYNNNNNNNNNNNNNGNNNANNNNCNACNNCCGGTNTNNNNNNNNNNNNNNNNNNNNNNNAGNRNRNNNNNNNNNNNNNCNTANNNNAGGNNNNGNNNNNNNTNNNCYRRNNNNNTGGAACCGCGNNCACGNGNNNNNNNNCTCCGCATNNNCNNNNGNNYYAAGCNNNNNNNNNNACNNNNATNCCGGGTCNNAAAGTCGAANANNNNNTNNNNNNNGNNNNNNNNNNNGANNNNNNNNTAAAGTTANCNNNNNTANNGTANNGGGACCCACNNNNTTTNNGANNNNTANANNAACNNNNNNNNNCNNNNNCANNNNNNNNNAANNNNNNNNNNNNNTATNNGNNNNNNNGTNNNACANNNAGGTNNCNNNGGANNRNNNGNNACGCNNNNNNNNTGYNANANNNNCNNGRTATNNNNNNGGANNNNNNNNNNCAGNNNNNNNNNCTCGAGTTTNNNNNNNAGNCATACAGCTCCANNNNNRTTNNCAGCTCNCNNNNNNNNNNNCNNNCCNCTAAANNNNNNNCNNNNNNACGCNNNAGCNTCAAGNNNNAANNNNNNTCGTNTTGNNNNNNNGAGNNNNNNNATATNNNNNNNNNNNCGNNNNNNTGGTCCGNNNNNNAGGAGACNNNNNNNNNNNNNNNAAYCGCGAGATGCGCTANNNNNNCTNNNNNAGTNTNCNNNGNNNNNNNNNNNNNNNNNNNNCTCNTCATNNCCRTNCNNNNNNTACCCCNNNNNNNNNNNNNCCGGCTANNNCGNNNCCANNCTCGCNYANCNAGNGNNNNNNNNNNNNAATNNNNNNNNYYCATNGNNNNNNNNNNNNCNNNNNNNNNNNNNNCNNNNNNNCNCACNNNNNNNTNGAGAAATCNNNNNNNNNNNANNNNNNNGGGNNNNNNNNNNNNNNNTGNNNGGCCNGNCTAYTGNNNNTGNNNCGCNNARNYTCNNNNNNNNNNNGGTTCANNNNNNNNGGACCGNTNNNNNNNNNNNNNNNCNGATNGNNACNNNRTYGGGNNNNNNNNNYCTNNNCGNNNNNNNNNNNGCTTTGTGGNCGTTCNNNNTNNNNGATNCNNGGRNNTNNNNNNNNNNNNNNNNNCNANNNAATNNNTTGTANCANNNNNNNNNNNGGNNNNNNNNGCYNNNGGNNCCTTACNNNNNNNNNCAAGTTTNNNNNTGTNNNNNNNNNNNNNTNANGCGTAANNNNNNNNGGTYANNNNNNNNNNNNNNANNNNNGGNNNNNNNNNNNNAAAAYYGCNGNCANNNNNGCGNNNNNNNNNNNNNNNNCGGCRTNNNNNNNNNNNACCGNNNNNNNNNNNNNNGTNNNNNNNNTNNTGNNNNNNNNNNNRNNNNGGACCGNNNNNNCCACGNNCNTGNNNNNNNNNNNNNNNNNNNNNNNNNNNNNNNNNNNGGANNNNNNNAGCNNNNNNNNNNNNNNYCANNNNNNNNNNNNTNNNNGTANNNCNTCACNNNNNNNNNNCCCNNNNNNNNNNNNNNYYRCGCAAANNNNNNNNNNNNNNNNNNACACNNNNTTGNNNNGGNNNNNNNNNGTTNNNNNNNNNNGNNNNNGCNNNNNNNRANNNNNNNTTNNNNNNNNNCNNNNNNNNCNNGCATGNNNTCGCTNNACNNNNNANACGTNNNNNNNNNNNANNANNNNNNGCNNNGTNNNNNNGNNNNNNNNNNNNNNNNGATACGCNNTACGNNNNNNCGTNNNNNNNNNNNNNNNNNNNNNNNTTTYGNNNCNNNNCAGCNNCNNNNTCTNNNNNNNNNNNGRNNNAGACTCCNNNNNNNNNNGGGATTNNNNGCCGNATANNNNNNNNNNNGNRNNNNNNCGANNCNNNNNNNNNNNNNNNNNCCNNNNNNNNNNNNNNNATCNNNNNNNNACNNNNGGCNGNNNNNNNNNNNYNCNNAGTNNNNNNNNNNNNNNAGCCCTNNNNNNTNNNNTANGCNNNNNNNNNNNNNGGGNNNNNNAATNNNNNNNTCCNNTCNNATTCNNNNNNNCGANNANNNNNNNNNNNNNNANNNNGTTAACTTRNNNNNNNRRRNNNNNTGTYGGYNCACAGCTNNNNNANNNNNNNGGNNNNNNNNNNNNNNGCNNNNNAGCNNNNNNNNNNNGTTGGNCCNNCNNCAANNNGANNNNNNNTACANNNNNNNNNNNNNNNNNNCNYGCNNNNNNNNGNNNNYNNNNNNNNNNNNNNNTTNTYCATGNCTGGCNNNNNNNNCGGANNNNCNNNNNNNNNGANGNNNNNCNNNNNCAGANNNNNCCGGNNNNNNNNNNNNNNNGNNNNNNNNCAGATTGTNCNNNNNGTTGCAAATNNNNNNNNNYTTRGGGCRTNNNGAYATANNNNNANTCGNNNCANGTCCTNNNNNNNNNNNNNCCTNNTCGNTNNNNCCCNCCTGNYAAAGNNNNNNNNNNNNNNNNTCAGNNNNNNNNNNGCTYGNNNNNNNNNNNNNNNNNNTGTTAAGNNNCTNNNGNAGGNCNNCNNNNNNNNGNNNNNNNNNTCCTTNNNNNNNNNNNNNGCANGNNGGCANAGCCCNTRNNNNNNNNNNNNNNNNNNNNNNANNNNNGNNNNNNNNNNCRNNNNNNNNNANNNNNNNAACGTAACATAGCNNNNCNRAGNNNNNNNNNNNNNNNTAGCAGGAGCNNNTNTGNNNNNNNNNNCCANNCGNNTGNNNNNTANNNGTNNNNNNANNNNTGNCGCCAACTNNNNNNNNNNNNNNNNNTTGTNTAANTGCCNNNNCCGGNNNCNNNNGANNGNTCNNNNNNNNNNNNNNNNNNTNNNYANNCATTGNCTTTGCACGGCAANGGCCCNNNGTCANNNNCTNNRNNNNNNNNNNNNNNGNNNNNNTAGNNNCGACNNNAYTNNNNANNNTGNNNNCCNYAGYNNNNNTNNNCTGNNNNNTANNNNNNNNNNGANNNNNNNNNNNNNNTCNNRCNGNNNNNNNNNNNNGNTNNNNNNNNNNNGTGANCNNATTTTGTAYYNNNNNNNNTACANNNNNNNNTNNNCGNNNNNNGTCCGNNNNNNNNNNGCAGTGNNNNNN

BrM52-1 GCGATTTANTAGCCGTCTTANNGGCCGNNRGRGTCNNNNACCTAGACGGGTCTARGRGGNNTACCCACNGAYYGYRATCTACTGGNTATNACGCYYTTTGNNNNAGGGGYCCACTGCCCGGGRNNNANCTTGGGTACAGNCYATRYTGYGCYNANNNTGGCCNNNCAGCGCGCAGNNGRRNCRANNGNNNNNNGAGGTNTGGACCTGGCYYAGNGANGAYAGAYYNYACAATACGGCAGTNTNNAARTAGGTTGGRNNYGNNNRTCCTAGGNNTCATGAGGAGGTTCTTGACGANCGNGGATCANTCGNNGGNCNNNNNNNATTYCCNRTGNCCGTGGATAGCCCGGTTGATRGYNTAGNTTGANNAACGGNGGAGCCATTCTRGAGCTGCTAGGGCGGNNCGNGCGCCGCAGGTYNGGAACCTNNGNNCATTACANTGCCGCCAGCTANNNNNNAARCRCCYTCTNTYTACTGYTGARNNNNNNGCATTTGGTTACCTTYNGCTGYCAACGAGCCCTAGCGCACACGGCGAANNATYCTNGTCTANACTCGTTTANNAGNNTAGAGCCGACGCGTACGRTTTCAACACCRACGGGGCNAGGATAGAACRYCTCCGGCTACCAANGGTGGACRTNCNCCTNAAAACANGYGACAGYTRNGGNNNNNNANGCCAATCTCCTCNATCCTATYCYYTNTGNGCAAGCCCCYRCCNCCGCTTAYRNGAGTRCCTANCATACTGCNNNNTGCGGTGGGTNCCAGRNNGCTCCNATTGCATGCGAATRGTANNCACTACCAAGGACTRNNCGGCNGGNNGCTAGGAACTGRTNTTATGTGAATGCGGACCCCGTCCGCAGTACAGANACAGCTARNNNTGTACACAACGGAATTGTNNNNNNNNNNNCGANNNNNNNTNAGTNCNNAAAAAAGGGAGTTGCGGTCCAGCCTGCCACNAAAGTTGCAAATNGNAGTAACTAGCGGTANCCATGGCTCNNAAATTANGAATAGCCCGCRGTRTAGGGNCGRNCCRCGTCGGCGAGGGCNNCNYNGCNGCRAGGNAYAATCRGRCGATTAGATTATGTCNGNAGGAACGCACNCCTCNTANCAATGGTNNNACNNNGTGGCTNCNCTTTYGGCGGCCGGGGNNTTNGGCNYGNYCGGGGGYCGNTRANTNNYAGGGYCTNNNAACRNGAATGCCCAAACGANGGGGCCAGCCACACCGRYRARTTCTCGCAGRGGGCGCCNNTTCGTTTGCTGYNNNNNATYNCTTTGNYCCGACGGTAACGCGNCRGYNNYYGAAAAGNCATANNNGYGTRATCAGACGCCNGAAATGTCRNTTAGGAATYNACTAGCACTGACNNNYAAGTRNCNNGCGCNTNGGNATCTCTCCGGGCGGCTAGNGCNNNTGNNRTNGGCTCGCCCCTNNNCAAAGGANNGGAAANATCCCNNNNCTCTGANGACTYNNCGACCCTAATGRACATAGAGNATNGNCCNGTTNAGCNTATGGTCTCTGGGANCANCCGTTCCCTGACTTACCCAGTGGCTTCCNNNGGACNYTRYACAGYCANNNNGCGAACCTCCCGNNNNNCCCAAGYGGTTTTTCNNNTAGCCTNCCCANGTNNYTCCGACGGAANTGCYCTGCTAGTGCTATCGTNAGCCCTNNNNCTATTAACNGGGNGGGCACTRNTCTCGAACTNNTAGTACTCGATANNANNTCNNNNANNANTATGTGCARGTNNACYRNATTAGNCTNCTTCATTGNNATGGCAYRRTNTGTTTNGATATNNNNCGCTTGGTTATTNGCYCGAAGCCCRTCCTCNCNNNTCCCGAAGAGTNAYGGTGAGGCCGCGGNTNCTCGCTGGACANTTTCYAAAGCGTNCAGAGGANACTTCGNNNTCTTTTCATTTNNNCTAGNNNNTTTCGYAATRTGCCGGCACCGCARAGCTGGCNCCGCAGNRNNTGTCTTGCNNAAYGGCTCAAGCGGCAGCGGCGCNTCNYNANCATGCCCCTGGCNNGGCGNNGACAGCTAGCCGTCYCATCTAATAACTCGNGTYCCCANNNNGYANGCGGGANGAAYNCGNNNNCGYTNNNNCGGGGTYTNNNCANNCTCCACACRGTTCAGNNRTTGCGCAAGGNNGNTGCRGRCCCCCAAACCGGTATAGGGTGTAATTGGNGTGTTCGCGAATNCCTRNGGNGTACYCRRYYGCTRATCGGCGCATCGTGGNNAGGGCGCCTCGCTTCTGTAYTAANRTTGGCNACGTAGATGCANNNNGNANNTTGCAGTGGGAAAAAGCGAATNNNYGGCAACGGGCTCTGCGACCACATTAGNNNCTTAATGNGTCAAAGANTGATCGTTCGTGTCCGCCCCCGCGGAGCGAAGNRCCYCAAAGNNCCGCTACGCGAAAGCCTGTCAAGTNRNGYYCAATNYCCGGCNCRYNNAGCGCGRTCGYGNNNNNNGRYTGCGARNNNTYCNNGGACTAGCNCNACTNCTNGAAACGGCCGGYACRGNAAGCYYGCTGNTNGGTTACGACTTAATTAGCCCRYCCCRGAGGNATCGTCCTCGNNNTTAGNGNCTACTYYRGTGGCGTACATCGCNTNANACCCACNCAGNRNNNGCGGTGNCTTAYNNGNNGCTAGACGCCTACAGCATCTCGGGYNAATANNTCTCCACACCTCTGNTCACCGGTGRCCACTAGGYGCYNGNNCNTGGATAACCGAANNNNGYCTYCNAAGNTCGCCATCTTACNAGACTCCCGACAGAATGCGGCGTANTACCCGCGTCNACNCTGNGCGTNGGTGACNAYRCANNACCGCCGGTNTATGAACCGCNNNACCGGGTCTGCAGGRNRRGAGACGYGNNATCNTAGNGYRNGTGTCGAYCYGCATYAGYCGRGNNAATGGRAYCGYRRTCACGTGNCCCCTTNCNCCGCATGGNCGCNNGNCTCNANNGGGGGTGTCTACAYCNATCCCRGRTCNNANNNTCGAAYATTTCCTTCTAGCTNAAATNCCCCCCGAYCTGCGCANNNAGTTACCACGCNTANCGNANTNNNNCCCACCGGNATTNCNNGGNNYANAGCANCYCNRTNGCTCCGTCACAGAGGTATNCAANNCTCNCGNCCNYTRNAANAGACNGGGTGAAACATTNAGGYRNCGTCGGNNCANCCGNNNNGCNTCACGTGYGYAANAGNNNYTAGGTATGTNNNANGAGTTCGNGCACCAGCGTCTTGACNNNNNNNNNTCNNNNTAGNCATNCRRCNCAACCGACRTTGGCAGNNCACANNCGRTGACCYNNNCCNCTARANGYNANTCCAGRNGACGCGGAAGCCTNAARGTRCRANCANNNTCGTGTTGRTCGGNCGAGAAGAGTARTAYANNNCYYAYNTCNCNNNANTRGTYCRTGCCRRAGGAGTTNTCNCNRAGGATAGNAACCGCGAGANNCGCTACATNNCCNTTNTTGATTTGCNNNGNANNATANNATCGTYGCTGCCTCATCNYGACCGTACGYAGAANANCYYAGAACCTAANTCGCCGGCTACCCCGACGYYAACCTCGNNNNGCCNGAGCTANTNNATNNNAATNGRCTNTTYYCATTGNNGYTGRCRGGCCCCTTNTNNYGNYACNGCNANRACTCACAGGCAGTTNGAGAAANYNTACRYGCGGYAANYYGTARGNCGAGGNNNNACAGCCTAAGRGGCCAGYCTNNTGYRGATGARTTGCGCANNNNNNNCCTNNNTCRGGTYCATCGTGNGCGGNNNNNNCCGCNGGCGNNNYRNYTGAYCGGAACCGCNTNGGGTACCTNCGATCTACNCGNNNNNNNNCNGGCNCCGTGGCCGTTCCGTRTAACNGATCYTRGNACTNRRATGCCATNRTAAGCACNNTGNAATAGATTGTAYCACGCCACACGNCGGCANAGTATGCCNNNGGTACCTTACNNTAGCNRTCAAGTTTYCTCGCATTGYYCCCGGGCAATCATGCNNNNNNGGNNNGGRTYATGGYNNNNCGACTNNGGYGCRRTCTCGGNNCCCRAAAANNGCTGTCATAAANGCGCRCRTCGAATGCNCCGCGGCRTNNNTNNCGGATRCYGGCGACCRTNNNAGYRTTCATGGTGTTRTGAGCTNTYCTTAGNNNNGGACTRAARTCRCCNCGATCATGCGTAGANGGCGCAAATGTGNNCAAAGCGNTAGCACGGACCNTGAAAGCGCRRATATTACGGCYCAYTNCNATAYAGGTCNNRGTAGGRCYNNACANTGGCTGTACCCYYGGGGGNCNGCCGTTGCGCARNTTCACNGGGGGGGAGCYRACACYNNNTTRNNGYGGGCTCNCRNGGTNAYNNAATNNNGGCGTCNCCCTAAYCNNAGNAGARTTCYRYTNNCTCGGANNANGCCGGCATGGTNNNNCTTCNCNCTTNATNYGTNTYGCANTATNNCGAAGNNNNGCGYCGTATAACAGAAAGACGACAAANNNNGATACGCAGTAYGGGACGACGTAGCNANCACGGRCCCTGATCGTGTTTYGNANCNNCACANCNTCARTCTCTGACTYTANCCAGGTGCAGACGTCTCAGTCAGCNGGGATTNCTAGCCGAATAAGCCNAGNGYCGARNNNACCCGANNCNAGCNNNNNGTGAACAACCNGAAAGGCACCGCACATCNNNNCTTGCCTCRGGGCNGARNCGCCNNNAYNCTCAGTTNCCACCTGAGGTTANNNCTNNGAYCNAAGGTAAGCCCCNTGAGCNATTGGGAGGCCGGANNNNNACGTCCGGNCNTATTCYYNNAACCGACCATAAAGTACGTNAAGANTTNNTNANCTTRACCCNNNNRRCCGCRTGTYGGYACACAGCTAAGNNAGTNNAATGGCRYTTCCAAGNTTNACCNCCNAGCRRCAGGRRTTAGTTGGCCCNNNCYCRRTCTGAYAYAACATACANNNARCCGNNNNRRGGNACTNGNNAGCATTNGCATCCACATGCGTCACYNTCTTTTNCATGNCTGGCTGGCGGAATAGAAGNNYTCGATCTGTGNNNGCTAGCYNNNNCNGATGTAGCYGGCTRTTGTRNANCGCGGACTGTGCCCAGATTACTNNNNNNGTNNCARNTNGRYCYTGNCTTNGGGNGNCNNGACATAANCGYANNCGCCTCANGTCCTNNGNNNNNGGNNNCCTGATCGCTNGANCCNACNNNNYANNNYTACNGNNACGTNNNNTCAGCGACTGGCTAGCTTCAACCCTAGATAACGGGGRTGTTAAGGGACTCTRGNAGGGCANCCTTNNNNTGTNCNNATGANYCTTNGACCGNNNNGYGNCNTNRAGGCAGAGNNNNTRNGCNGRGNCCNAGGCCGTNCGCAAACTNGYNARYTNGRCCAGAGGNCGACAGGNGCNCAACRYARCATGRCTYCANGGAGNRCRCCNARCNNNNNTAGCAGGAGCCGGTGTGCATACCCNTACCANNCGCCTGAGGCTTATCTGTCNGTACAACCATGGNNNNNNNNGRNGAGTGCCTGGNNCTTTGTATAAATGCCCNTNYCGGRAGCATCNATTCNNNCGGTCCNNNNAYNARYTATTRRYYAGACATTGCCTTTGCACGGCAACGGCCCGRNGTCRCGACNTCGGGNCGGTATGTGGYRGANTTTTTNGATCCNACYCGATNNNNNRRNAYRNNGGYCCCAGCATYCYNACNCTGGNATTTATTNTGAAAGTGATCGGGCAAAGCANTNTNNNNNNNNGRNGNNNNNYGRTGNCCRGNNCNAGTGAYCAGATTTGGGACCTAGCATCYTACAATGGNGRGTCATTACNTTCAGTCCGACGCGGTCTCGCANTGCNNNTT

BrM54-1 GYGATTTACTCGTCNNNNNAATGGCNNCCGGAGTCNNNNACCTAGACNNGNCTAGNAGGNNTACCCACTGANNNNGATCTNNNGNNNNTCACGCNNNTTGTCTAARGRGCCCACTNNYCRGGANNNNNNNTNNGTATNNGCCNNRNNNNNCTTACAATGGCCAGGCAGCRNGCANNNNNNNNNAAAGTANNNNNNNNTTTGGACCTGNNNNTNNAACGGNNGAYYNNNCAATACGGCAGTCTNNAAGTANGTTNGGYANNNAGRNCCNNNNCCNYRTRAGRAGGTTNTTGRCGATCGTGGATCACTCGNNGGTCATYRNCNNTTCNNANNNCCCGTGGATNNNNTGGTTGATGRNNNNNNNNGRNNAACGGTNNNGNYATTNNGNNNNTGNTAGGNTGGGRCGGGCGCCGCAGGNNNNGAANNTNRGTRCATTACAGTGNCGNNNNCTAGCCTNANANNGCCYTCTCTYTACTGCTGAAYGGNCTGCGTTNNNNTACCTTTGGCTGNNNNCGANCCCTNNNNNNCACAGCTAANNNNNNNNGTCTARACTNGTNTRAGAGTCNRGARCCGACGCRYACGRTNNYAACACNRNNNNNNNRNNNNNNNAACACCNNCGGCTACCNNNGGTGGACATNNNNNNNAAAACAAGNGACAGCNGAGGCTGAGCANGCCNRTCTCYTCGNNNYNCTCCCTTATGTGCNNNCCCCYRYCNCCGCTTAYNNCAGTNCNNAGCATANTGCGCCGTGNNNNGGGTANCARGNGGCTCCTATYNCATGNNNNNNNNAGGCACTACCNNRRNCTAGCNNNNNGGNNGCTAGGANCTGATCTTATNNGAGNACANNNCCCGTCYNCANTNCAGANNNNNNNAGCCCTGTACACAANNGAATTGTTCTNAGGCTTNNGAATACGGANAARNNNGGAAAAACGAGCNNCGNNNNNCGGTCAGTCACNNNANNNNNNNNTNGNAGTAACTAGNGRYAGCCATNNCNYCTNNANNNNGGCTAACCCACGGNGYNNNNNCGNNNNGCGTCGGCGAGRGCCGCCNNNCCGCRAGGGATAATCAGACGATTAGATTATRTCAGRARRAACNNACYCNTCTYAANNNNNNTANNACGCTGTGGCTCCACNNNCGGCGGCNNNNNRRTTAGGCNCGTCCGGGGGNCGCTRANTNNNAGGGNCTAGNAACNNGANNNCNCRNNNNNNNGGGCCAGCCNNNNNNNNNNRTTCNNNNNNGGGGCATCNNTTNGYTTNNNNCRRNNAATTTCTTTGGCCCGACGNNAANNNGTCAGCCCTTGANNNNACATAGNNRNGNGNNCGGACNCCNGAAATGTNNNTTAGNAATCCANTAGCNCTNACNTAYNNNNNNCNNGCGCNTNGGTANCTCTCCGGGCGGNCRRNGCNNNTGNNNYGGGCTCGCCNCTGGACAAAGGARGNGAAANNNNNNGGGACTCTGAGNACTCTGCNACCCTAGNGAACATAGAGNATNGNCCNNTTTNRCATANNNNNNCTGNNNGCNNCCGNNNNNNGACNNACCCAGTGGCTTCCGCAGGACTTTGTRCRRNNNNNNAGNGNNCCNYCNNCACCTNCNNAGYGGTTTTTCANRTNNCCTNNCNAGGTCGNTCCGAYGGAATTGCTCTGCYNNNGYTNNNNTGANCNNTATATCTRTTAACCGGGTGGRNACTRNTNTCGNNNNCGTAGTGCTNNATATNAGGTCGGNNAYYNNTATRTRNNNGTGGACTGNANTAGTCTGNNTCATTGNNAYGGCANNNNNNNTTTGGATATCNTNNNNTTGGTTANNTGCTCGAANCCCRTCCTNACGNNTCCCGANGAGTRNNGGTNGGTCCGGNNNNTCNCGCTGGACANTTNCCAAAGCNNNCAGAGGATACTTCGTTYTCTTTTCATTTACTCTAGCCAATTTCGCNATGTGCCGGTGGCGTANNNNNGGCNNNNNNNNRGGTRTCTTGCAGNATNNNTCAAGYGNNNGNGGCGNNNCANNATCRNNNCCCTGGNANGGCGGCGNCNNNNNNNNGNNNNNYYYRATNNNNNGTGTCCCCACGAGGNANGCGNGANGAANNNNGAGACGCTATGACGGGGNTNRTNCANNYYYNNNNNNNNNCNGYCGTTGYGCNAGGGCGGTTCGGGCCCCCAAACCGNNNTGGGGYGNNATTGGRGTGNTCNNNAATACCTRNGANGTNNYCNNNNGNNAANCGGYGCANCGTGGTNAGGGCGCCTYGCTTNNRNNNNNNNNNNGGCAACGTAGATGCGTGYNGANNTNTGCGGCAAGAAAGAGCGANNNNNNNGCAACGGNNTCNGCGNNNNCATTAGTGACTTAATNNGTCAAAGAATGATYGTNCGTGTCCNNCYCYGCGGCGCGAAGNRACYCAAANTGCCGNNNNNNGANNNNCTGTCAAGTNNNGYYCAANNTCCGGCGCACNYRRNRNGGTCGCGATAGCGGNTTNNGAGYRATYCNANNAYTANCANNNNTNNNAGAAACGGCCGGYACAGNRARCCCGCTGNNGNNYTNCGNCTTRATTAGCCCNNNCCGGNGCAATCGTCCTCGTCRNNNNNNNCNNCTYYRGYGGCRNACANCGCAYTANACCYAYNCANNNGCNNNNNNGTNNNAYAANACTCTNNNCGCNCACAGCATNNNNNNCTAATACTTCTNNNNNNNCCTGANNACCGGTGANNNCTAGGYGCCTGTTCCTGGATANCNGAANNGGGCYTYCNANNNTCGCCATCTTACAAGACNNCYGNNNGNRTGCGGYGNNTTAYCCRYGTNNACGCTGAGCGTCNNTGACGNNNNNNGACCGCCGGTTTATGNACCGCACNNNNNNNCNTGCRGGAGANNNNNNNCGGYATCNNNGCGNRNGTGTCGANCNGNNTNAGNCGNGNNAANGGGACYGCRNNCACNYGACCNYNNACTCCNCATNGNCGCYGGCNYNNANNNNGGNNNTCTNNRNCRATCCCGGGNNYNNNNNTCGAACNTCTNNTNNTANCYRNNNNNNNNNNCGATCCNNGCATAAAGTTANCNCGCNNNCNGTANTGGGACCCACCGGGNTTNNGAGGNTTANAGCAACCCNNNTNNTCNNYCACANNNGTATTNAATTCTCGCGTNCTNNNNNNNAGNCGGGGTGNAACATNNNNNNNCCGCTGGNNCRTCTGNNNCGCNNNNCNNGTGTNATAGTCNTTRGGTATNTGAGANNNGNTCGRNNNCCAGYGTTTTNACCTCGAGTTTTCAANNYRNCNNNNCAGNTCNACAGACATTNNCAGCTNNNNNNCGNNRGYYNNNNCCTCTAAAGGYNNNNNCAGRNGACGCGGANNNNYCAAGGTATGACCANNNNCGTNTTGGTNGGNNGANAAGAGTAATRTNNNNNCTATNNCGCAGGNNNNNTNCGTGNNNRNNGAGACNTNACCAAGGATNGTNNYCGCGARNTGCGCTACNTGYCCTTTNTYAGNNNNNATTNCATNNNNTAANCGTNNNNNNCTNATCCCGACCGTACGYNNNAGNYYYYAGAACNYAAATCGCCGGCTNCCCCGACGNNNGCNNNNCTNANCNNGAGNTNNNRYNYCYRNNNNGRCTRTYNNNNTTGTANTTGGCGGGCCCCTCTTATTNNNNNNGCTANNNNNCACAGGCAGTTNNNNNNNNNATACRNNCGGTNAACCGTAGGNCGAGGNNNNACAGNCTGNNNGGCCNNCCTNNTGCAGATGAGTCNNNCAGNCTYGNYCTRRATCGGGTYCANNGTGRGCGGACCGGTCCGCNNNNNNCGNNNCTGAYNGGAACYRCGTCGGGCCCCTGCGNNCNACCCGTCCGCGCCNNNGYNYYGTGGCCGTTCNNNATNANNGATCCANNNNCTTNNNNNCCCTTGTAAGCACNNNNGAATNNATTGTAYCACGCCNNNNGNNGGCATAGGACGCCAGCGGNNCCTTGTNNNAGNNNNCAAGTTTTCTNNNNNTGCCCCCARGCAATCATGCGNNANARNNNNRNNTCNNNNNNGNNCGAATCANNNGCGGTCTCTGNYCCCRAAAAYYGCANTCANNNAYGCGYACGTCGAATGCACCRCGGCNTTGCTTRCGGNTRCTNNNNNCCNNCATAGYGTNCNTGGTGTTNNGAGNNCTCNNNNGNNNNGGACNNAAATCNCCACGRTNATGNGTAGNNRGCNYNAATRTGTCCANNRCGNTAGCACGGAANTTGANAGCNCRGNNATTACGGCTCACTCCTATACNNNTNNAGGTACGNCYTCACANNGGCNRTNCCCTCGGGGGACYGCCGTYGNGCAAATTCACGGGGNGNNNGCCAACACYNNNTTGAAGCGGGCTCNCGNNGTTNNNNAATTGTGNNNNCNCCCTAACCRAAGNNNNNNNNTGTTNNCTCNNANNNNGCCGGCATGRTNTCGCTACNNTNNNNNTATGTATNGYAATRYAACGNNNNNAGGCNNNRTATRNNNNNNNNATGACNAANNAAGATANGCANNNNNGGACGACGTNNYCACCNNNNGCCNNNNNNNTGTNNYGNANNRNCACANCNTCRRTCTCTNNNNNNANCCGGGNNNAGACGNCTNAGTCAGCNGGGATCACNNNNNNNNTARGACGAGTGTCGNGCCANNNCGARYCTAGCNNNNNGNGNACNNNNCGAANNNNNNNNNACNNNTATATNTCNNTCAGGGCGGANNNGCCNNNNNNNTYNGTTNNNNNNNGNGGTNNRNNCTGNNNNNTAAGGNNNNNCCCANNNGCGATTGGGAGNNNNANTNNNNAYRTCCRGTCCNNNNCCNNNANNCGACCNTAAAGTACGTGAAGAANNYGTTAACTTNACCCGCTAGACCRCRTGTYGGYNCACAGCTGCCTCRGTCTAATGGCACNNCCAAGGTTTGCNNNNNNNCGGCAGGAGTTAGNNNNNCNNCNCNCAATNTGANNNNNNNTACAGCAANCCGNTNNNNRRCACNCNNNAGCANNNGNNNNCANATNCGTCNNNATNACTYCYANGTNNNNNNGGCGGAANNGAAGATCTNNRTCYGTGATGAAAAGCNNNNNCAGAGGTTACYGGCTGTNNNGNNNCRYGGGNTGTGCCCARNTTRYCCNNNAANNNNNNNNTAGACCCTNACTTAGGGAGTCGGNANAYAANCGCAANCGCCTCAGGNNNNARGAANGTNNNNNCCTGNNNNCNCNAYCCCACNNNTCANAGYTACNGNTACGTCTYCTCAGNNNNTGGCTAGCTYGAACNCTAGANAACGRGGGTGTNAAGGGACTNNRNNAGGNCATCNNNGGRNNNTNNGYATGANYCNTAGACCRNNNYGTGGCAGGGGGGCNGAGCCCCTRGGCNNNGACCTAGGCCGTNNNNAAACTCGTCANNTGGNNCNGNNNCCGACAGGGGCGCNANNNAACATARCTNYANNAAGAACACCNNNNTATTNTAGCAGGAGCNNGNGTGNNNNNNCGTNNNNCGYRCCTGAGGCTTATCTGTCRGTANARCCANNNCGCCAACTNGNGANNGCCCNNTACTNNNNATAAATGCCNNTTCCGGGAGCATNNNNNYGNNCGGNCCCGAGNYANRYNNNTAACTAGGCATNNNCTTTGCATGGCANCGGYYYGNTGTCRNNNNCTCGGGNYRGTNNNNNGYRGANNTNNTAGATCCGACTCGATTRCCCRRNANNGARGTCNNNNCATTCCNACNNNNGGNNNTNTTGNNNNGACGATCGGGNNNNNCNCTACNNRCNGGCNNNNTCCGTYGRTNNNNNGANCNAGTNAYCARATTTTGTACCYRGCATYCTACANTGGAGGGTYNTYRCATTCAGTCNNNNNNRGCNTCGCAGTRCACATT

BrM55-1 GNNATTTACTCGTCRNCNTANNGGCNGNNGGAGTCGTCGNNCTAGACGGGTCTAGGNGGNNTACCCACTGACTGTGATCTACTGGCTATCACGCCTYTTGTCTAAGGGNYYCACYGCNCGGGAYCCNTNNTGGGTATNGGNNATNCTGCGNNNNNAATGGCCTNNCAGCGNRCAGGAGAGCCGAAAGTANNNNNAGGTTTGGAYYNGGCYYNGGAACGGTAGNTCTYAYAATACGGCAGTYTNTNNGTAGGTTGGGNNCGNACATCCTAGGCCNNNTNAGRAGGTTCTTGANNANCGNGGATNNNTCGCAGGNNATYRNCNNTTYCCAGTNCCCGNGGATNNTCCNNYTGATGRNNTAGNYTNRNGAACGGTGGAGCCATTCTGGAGYTGCTAGGNYGGGGCGGGCGNCGCAGGTTCGGAAYCTAAGTACANTACANTGCCGCCAGCTAGCCTCAAARCGCYCTCTNTYTACTGCTGAACGGTCTGCRTTTRGTTACCTTTGGCTRYNAANGAGCCCTAGCGNACACAGCTANNTATCCTAGNNNARACNNNNNNNAGAGTCTAGAGCNNACGNNTACGRTNTNAANACCNACGGGGCGAGGNANGAACACCTCCGGCTACCANCGGTGGACRTTCCCCTNNNNNNAAGCGACAGCTGAGNNTGAGCARRCCAATCTCCTCAATCYGCTCCCTTATGTGCATACCNNTGTCGCCGCTTACGAGAGTANNNAGCGGACTGCGCCGTGCGGTRRGTACCAAGTGGCTCCTATYNCATGCGNATGGTAGGCACTACCRNAATCTAGCCGGCTGGNNGCTANNAACTGATATNNNGYGNNNNNNTGCCCCGCTYGCANTACAGATACAGCTAGCCYTGTACACAACGGAATTGTTCNNNNGCTYGCGAATNNGNNNNARNTCGGAAAAANGGGNGTYNNGNNNCRGYCNGTCACNAAAGTTGYRANTNGNAGTNACTCNCGGTAGCCNNGNCTCNTANATTAGGANTAGCCCGCGGTGTARRGNCGNNCCRCGTCGGYRAGRRCCGCTTTRNCGCAAGGGATAATCAGGCGAGCAGATTATGTCAGRARRAANGCACTCYTCTTACNNAAGGYCACNNNNNNTGGCTCCANTTTNNRCRGCCGGGGNNNTAGGCACGTCNNNNGGYCGATAAGTACTNNNNCNNAGAAACAAGAATGCCCAGAAGATNGGGCCAGCCACACCGACAANTTCTCGCAGGGGGCNNNAGTTNGYTTGCTGCAGRGNNTYTCTNTRGCCCGACGGTAACGCGTCAGCCCTTGAAAAGNNNNNGNNNTGNRATCRGACTCCNGAARYGTNNNTTNNNAATTGACTAGCACTNACGYRNANRNNNCNNGCGCNNNNGTATCTNTCCGGGCNGCCRRCNNNNNNGNNRYGGGCTYNNCYCTCGACGANGGAAGGGAAANNNYTCRRGACTCTGAAGACTTTGCGACCCTAGTGAANATAGAGAATCGCCCAGTTTRNCATATGGTCTCTGGGAGCAGCCGTNNNNNGACTTACCCAGTGGCTTCCRCAGGAYTTTGTACAGCCANNAAGCGRANNTCYCGCNNCYCNCAAGTGGTTTNTCAAGTAGCCNACCYAGGTNNYTCCGAYGGAATTGCTCTNNTAGTGCNRTYGTGAGCCCNNNATCTNNNNACYGGGTGGGCACTGGTTTCGGRNTCGTAGTACTCGATANRAGGTCGGNNAYYACTATRTRCAGGTGGACTGNNTCACCTTGCTTTNNNGAGATGGCATGGNCNNTTNNGATRYCNTCCGCTTGGTTANNNGCYCGANNCCCRNNNNCACGNGTCCCGAARAGNGNNGNTGAGGCCGGRGATTCTCNCTGGACACTTTCCAAANCGYCCAGAGRATACTTCNTTCNNNNNNCATTTACGNNNNCCAATTTCGTAATGTGCCGGTGGCGTAGAGCTGNCGCCGCAATRRGTRTCTTGCAGAAYGGCTCAAGNRTCGGCGGCGNRTCNYNANCNNNCNNYTGGCAYGGCGGCGACAGCNNGCCGNCCCACYCRATGGANCRYNTCCCCACGAGGYNNNNNNGATGAAYAYRGAGACGYTATGACGGGGTTTRTNCACATTCCACACNNTTCAGTNRTTANNNAAGGGCGGTTCGGGCNNNNNNRCCGNTATGGGNNGTAATTGGGGTGTTCGCAAATACCTRNGGAATACCCAGTTGCTAATNNNNNNNNCGTGGCNANGGCGCCTNGNTTCTGTATTAACATTGGCNACGTAGATGCATGYNGAACTTYNCAGTGNGAAAGAGCRARNCCATGGCAACGGGCTCTGCGACCACATTAGTNACYTNNTGYNNNAAAGANNGATCGTNCGTNNNCNNCCCCGCGGAGCGAAGAAACTCAAARTGCCGNNNNNNNAAAGCNTGNCANNNNRNGNNCAAYNTCCGGCGCACNYAGCGCGGTCGCGATAGCGGGYTGCGARNGATYCNRGGANNNGCACTACTNCTAGAAACGGCCGGYACRGGGAACCTGCTGGTNNGYTANNNNTNRANNNGCCCGCCCCGGAGCAATCGTCCTCGTCGTCANNNNCTACTCTAGTGGCGNACANCGCATTAGACNNNNGCAGCAGCNGCAGCGTYYTNCAANACTCTARACNCCCACANCNTCTCGGGCTANYACTNNNCYACACTYCNNATCACCGNNGRCCANNNGGCNNNGNANCNNNGATANYCGAAANGGGCCNNCAAAGNNNGCCRTCTTACNAGACYCCNGAYAGNRNNNNGCGNNTTNNCCNNGTCNACNNNNAGCGTCGGTGNNNATGCNCNACCGCCGGTTTATGAACCGCNNNNNNNNNTCTNCAGGRGANNNGACGCGGYRYCTTAGCGYNNGTGTCGACNTGTNTNAGCCGAGNNAATGGGACCGCGNNCACRCGAYYNYNTACCNCGCATNNACGCTAGYNNCAAGYNNGGGTGTCTACACCRATCCCRGRYCNNAAAGTCGAACATYTCCTTCTAGCTGRRNTACCCCCCGATCCNYGCATAANNNTACNACGCTTACCNTNNTGGGACCCACCGNGATTRCGAGGATTANAGCNACCCCGTNNNTCNNTCRCAGCGGTATTAAAGCCTCACGTCCNYTATRRNAGNCGGGNNNAAACAYTNRRGYRNCGYYGGACCATCYGNCCCGCATCANNNGTGNAATAGTCNCTNGGTATGTGAGAGGAGTTCGGGTGTCAGCGTCTTGACCTCGAGTTTTCAAACTAGCCACCCAGCTCNANANNNGAAGGCAGCTCTCAAGCGNTNNCCCRTNCCNCTAAAGGYNACTCCAGRNGACGCGGAAGCNNNAANGTRNAATCNNYNTNGTATTGGTCGGACNNNANNNNNNNNAYRATCNNNNNTTCGCAGGAATGGTCCATGCCGNAGGNNNNATNNNCANNGAANNTGTCCGCGAGATGCGCTANNNGTCCNTNNNTAGTCTACNNNGNNNAANNNNNNCAACGCTGCCTCRTCCCGACCGTACGCAGTAGATCTTAGAACCCAAATCGCCRGCTACCCCGACGCCNGCCTCGTACGGCCAGNNNNACTGTATCTAAATCGACTNTNYYCATTGTAGTTGGCGGGCCCCTTTTAATNNNNNCGNTANNNNNNACAGGNANTTTNNNNNNNNATACACGCGGTAANCCGTAGGGCNAGGCYANACAGCCTRTGGGGCCNNCCTACTGCAGANAGATYGCNCAGTCTYGNYCTRRANNNGGTCCACAGTGNGCGGAGCGGTCCGCAGGCGGCCYRNCTGATCGNNNNCGCNNNNGGCCCCTGCGAYNTACNCGNNTGCGCCCNGGCTCCGTGGYCGTNNCGTNTNACTGNTCCTRGGACTTAGATGCCCTTGTANNNNCNATGRAATAGGTTGTATCACGNNACACGGCGGCANAGGACGCCAGCGGCACCTTNNNTTAGCCGTCAAGTTTCCTCGTRTTGCCNCCARGCAATCATGCGTAACGGGATGNGGTCATGGCRGCNCGAATCAGGNGCGGTCTCTGGNCCCRAAAANYGCANTCATTTATGCGYACGTCGAATGCACCRCGGCRNNNNTNGNNNNNACNNNNNNCCRTNNNAGYGTNNATGNNGTTRTGAGCTCTCCTTAGGCGCGGACNGAARTCGCCACGACCNTGCGTAGNNRGCNYGAANNNNTCCRAAGCGNTAGCNNGGANYTTGAAAGCNNNGNTATTACGGCYCAYTYCTATNNAGGTYTNNGTACGRCTTCACANTGGCTGTACCCNNGGGNGACCGCCGTTGCGCAAANNNACNGGGGGGGANNNNACACYNNNTTRAANNGGGCTCNCGAGGTTAYTANATNGTGGCGTCNCCCTANNNRAANNAGARTTNTGNTACCACGGAGGACGNCGGCATGRTGTCGCTNCACCCTTGATACGTATTGTANTGTANCGAAGCCAGGCGTNRTATRAYANAAAGATGACRAAACTAGANNCGCAGTNNGGGACGACGTAGCNANCNCRNACCCTGATCGTGTTTYGNNCYGNCACANCNNCARTCTCTGANTTNANCCGGGTGCAGACTCCTCAGTCAGCNGGGATTACYRGCCGTRTAAGCNGNGNGYCGAGCCAACCCGANNCTAGCGGGTCGTGAACAANNCGGAGGGCACCACACATCTATATTTCNCNNNGGGCGGNNNNGCCNNNAYNCNCAGTTCCCANNNGNGGTCAGNNCTAAGATCTAAGGTNARNCCCAYRNGCGATTGGGAGGNNNAATCCGGNNNTCCNGTCYYNTTCCTCGAACCGACNATAAAGTACGTGAAGAATTTGTTANCNTGACCYGCTAGGNCRCNTGTYGGYACANANNNGCCTCGGTCTAATGNCACTCCCAAGGTTTGCCTCCNAGCGGCAGGNNTTAGTTGGNCCACCNYCRRTNNGATNCAAYNTACANCAARCCGATRNAAGGTACTCGCCAGCATTTNNNNCYANATRCGTCNCYRTCTTTTNCATGTNNNNNYGGCGNNNTAGAAGATCTNNATCTGTGNTGNNTAGCCGTCNCRGAGGTTACYGGCTNTYRNGTRGCNCGGACTGTGCCNNNNNTGTCCTCNAAGNNGCNRNTAGACCCTNRCTTAGGGCGTYGRGAYATAANNGYAATNNCCYNNNGTYCTAGGNACGTGGGATCCTGATCGCTNGRYCCCACCTGTCANNNNNACNNNTNNNNCTTCNCNRCGACTGGCTANNNNNAACCCTAGNNNACGRGGGTGTTAAGGGACTCTAGARGGGCAACCTTGGRNNGYCCRYATGATCCTTAGACNGCTTYGTGGCATGNNGGCNGAGCCCCTRNNNNGNGCCCTGGGCNATNNNNAAACNNGYCAGTTTGNCCNNAGGCCGACAGGGGCGCAACRYARCATRRCTNCACGGAGNACACNGAGCTNTYYTAGCAGGAGCCGNNGNNCNNNCCCGTNNNNTTCGCTTGAGGCTTATCTGTCRGTANNNNCATGGCGCCAACTAGAGAGTGCCTGGTNCTTTGTATAAATRCCCTTTCCGGNNNCNNYGGANCNNNCGNTYCCGAGNNANRYCNYNAGTNAGGCATTGCCTTTGCACGGCAACGGNNNGRTGTCACGANNNCGAGACGGTATGTGGYRGAYTTTYTAGATCCGACYCGAYTACCCAAGATGNNNGCNCCAGCATCCTTACRCTGGGATTTATTGTGNARRCRATCGGGNNNNNCGYNNCCTGCTRGCGRNGTCCGTTGRTGACCGRANCNANNGACCAGATTTTGTACCCGGTATNNYAYARNGGCGRRTNNTNNNNNNCAGTCNNACGNGRCCTCGCANTGCRNNNN

BrM56-1 GNNATNNNCTCRTNNCYATNNNGGNNNNNGGAGNCNNNNACNYAGRCGGGNCTAGGNGGNNNACCCACTGACNNTGANNNACNGNNNNTNANNNNNYNTGTCTNGGGANNNCACTGCYCAGGRTCCATCTTGGNNNNNGGNNATNCTGCGNNNNCAATGNNNNGGCAGYNTRNANRNGRRNCRANNGTANNRRNAGGTTTGGACCTGGCTTTNNNACRGNNNNNNNNNCAATACGGCAGTCTNYAAGNNNGTTNGGNNNGAANNCCCTAGGYCNNNTNNNNNNNNNNNNNNCANNNNNNNNNCNNNNNCANNNNATTGACYANTYNNARNNCNNNNGGATAGTTNNNNTNNNNNYNTAGNYTNNAGAACGGTGGNGCCNTNCYNGNGCTGCTANGNNNNNNNNGGCGCNNCAGGNNNNNNNCCTNNGTRCATTNCANTGCCGCCAGCTAGCCTNANANCGCNCTCTCTNTACTGNNNNNTGGGNNNNNNNTGNNTACCTNNGGCTRYNAAYNNRYNNNATCAGACACAGCTAANNNNNCTNGTYTNNNNNNNNNNNAGAGNNNRGANCCGACGCRTACGGTTTNAACACNRNNNGGGCRAGGNNNNAACRYCNNCRRCTACCAACGGTNNNNNNNNNNNNNNNNNNNNNNNNNNGCGGAGGCTGAGCAGGCCNRTNNNNNCRATCTNNNNCYYTRTNYGCNNNNNCCNNNNNCCNNNNANGNNAGTRNCTNNNATACTNCGCCGTNNNNNNNNNACCARGTGNNTCCTATYGNNNGNGAATNNNNGGCACTACCNNNNNCTAGCCGGTTGGNNGCTANNAACTGATNNNNNNNGAGTACATGCCCCGCTCGCANNNNNNNNNNNNNNANCNNNNNNNNNNNNNNAATYRTYCTNNNGCTTRCGANNNNNNNNNAGTTCGGAANAACGGGCNNNNNNAGCCGNTCANNNNNNAAANTTNNNANTNNNNGTANCTNGCGNNAGCCNNGNCNNNTAANTTCGGRNTARCCCNCGGNNNAAAGGCGNGCCGCRTCRGCGARNGCCGNNTTRCNNNNNNNNNNNATCANNCGATTAGATTATGTCAGRNNNNNNGCACTCNTCTTANNNNNNNTAACACGCTGTGGCTCCANYTTYNGCNNNCGGGGNNNTNNNNACGTCNNNNNNTCGNNNNNNACNTGGGNNNAGNAACRNGAATGNNNNNNNGATGNNNNNAGCCACACCGNNNNRTNCTCGCAGGGGGCNNNAGTTNGNTTNCTGNAGNGNNNNNNTNTNNCCCGACGNNAACGCGACGGTNNNNNNAAAGACATANNNNTGTRATCGGACNCCNGAAATGTNNATTAGGAATNNNNTAGNNNNGNNNYRNACGTRNCNNGCGCNNNNNNANCTCNNNNGGNNNCCNNNGCNNNTGNNNTGNNNNNGCCCNNNGACRANNNNNNNGAAAARCCTCNNGACYCTGAGNACTCTGCGACCCTARYGNNCATAGNNNNNCGNNNAGTNTNNCATATGGTCTNNGGNNNCANCCGNNNNNNGACYYACCCAGTGGCTTCCGCNGGACTTTGTNNNNCCAGAAAGCGNNNNYCCNNCACNNCNCAAGTGGTTTTTCNNNNAGNNNNYCCNNNNNNNTNCGACGNNNNNNNNCTNNNNNNGCTNNNNNNNNNYCTNNNNCTATTANNNGGGTGGGCACTNNTYTCGNNNNCGTAGTACNNNATATRAGGTCGGNNNYYANTATNNNCAGGNGGNNNNNANNNNNNNNCTNNNNNNNNATGNNNNNNTNNNTTNGGATATCGTCNNNTTGNTTANNNNNNCGNAGNNNNTCCTNACGCGTCCCNNNGAGTNNNGNTGAGGNNNNGGATNCTNNCTGGACAAYTTCNAAAGCNTNNNNAGRATNNNTCGTTNTCTTTNCATNTACGCTAGCCAATTTCGYAATRTGNCGGNNNCGNAGAGCNGNYRCNNCAGTGGGTGTCTTGCAGNATNNNTCAAGYNNCNGCGGCGCGTNNNNANNNNGCNNCTGGNATGGCCGCGACAGCNRGCCNNNNNNYYYRATNNNNCGCGTYCCCACGAGGYNGGCGGGANGNNNNNNGAGNNGNNATGACGGNNTNNNNNCANGCTCCACACGGNYNNNNGATNNCGCANGGNNGNTTCGGGCCCCCNRANNNNTANGGGGNNNNATTGGRGTGYTCGCNNATNNCTRNGNARTACYCRRYYNCTNATCGGNNNNNNGTGGCNANNNCGCNNCGCTNNNNNATTAACNTNGGCAACGTAGATGCATGNNGAACTNYNCRGYNNNNRRGAGCGAATNNNYGGNNNNNNGCNNCGCGACCACATTAGTNANNNRNTGTNNNAAAGANTNNTCGTTCGTACCCNNNNCCGCGGCNCGAAGNNCCYNAANGNNNNNCTACGCNANNNNNTGNNANNNNNNNNNCAAYAYCNNNNNCRYGCRRNNNGGTCGCGATAGCGGGYTGCNNNNNNTNCNNGGNNTAGCACTACNNNNNNNNNNNNNNNNNNNNNGNAACCNGCTGGTGGGNNACGACTNAATTANNNNNNNNNNNAGCAATCTTGCTNNTTGACAGNGNCNNNTNNNNNGGCNTACATCGCATTAGACCTATGNNNNAGCNNCAGCGTNNNNCAANACNCYNNACGCCYNNNGCATNNNNNNCTNNNNCTNNNCNNNACNNCTGANNACCGNNGRCCANNAGRYNNCNNNNNNTGGATNNNNNAAAYNNGCCNNCAGAGCNNGCCAYCTYACAAGACTCNNGACAGAATGNGNNNTATTACCCGCGTNNNNTTNNNGCGTCNNNGRCGANNNNCGACCGNNNNNNNNNGAACCGCNNNNNNNNNNNTGCAGGGGANNNNNNNYGNTATCTTANNGYRNNNNNNNAYYYGNNTYNNNNNNNNNNNTGGAACNGCNNNCACNYGNNNNNNTACYNCGCATNNACGCYNGCYTNNNGNNNGGGTGNNNNNACCGATNCCGGGTCNNAAANNNNNNCATYNNNNTCTAGNNNNNNTACNNNNCGANNNNNGCANNNNNNTACNACGCTNNCNNNNNTNNNNCNNACCGGGNNNNNNNGNATNNNNNNNACNCAATTGGTCYRNNNNNGCGGTATTAAANYCTNNNNNCCACTATAACAGACNNNNTRAAACANTNNNNNNNNNNNNNANCNTNTGNNNCGCRTCACNNGTGTAANAGTCNCTAGGTATNTNAGAGGAGTTCGGNNNCNNNTGTTTTTNNCTCGAGTTTTCAARYYRNCCANNCNGCTCNNNANNNANNNGCNNCTCNNAANCGGNNNNNNRTNNNNNTAAAGGYNACTNCAGGNGACGCRRAARNCNNAANNTNCAANNNNNNTCGTNTNGNNNGGNNGANAAGNGTAATRTAATCNCTATTTCGNAGGNNNNNTCCGTGCCGNAGGAGNYATCACCAANGNNAGNNNCCGCGAGNTGCGCTACNTGTCCNTYGNCAGTCTACATTGCATAATATAAACGTCGCTGCNNNNNCCCNNNNNNACGCANAANNCNCCAGNNNNTAANTNGNCGNNNACCCCGNAGCCANNCTCGYNCRGCCNGAGCTNNNNNNNCTAAATGGANNNNNYYCNTTNTNGNNNNCGGNNNCCNNNTANTCTCGTCGCNANRACNCACAGGNANTTTGANNAATCNNNCACGCGGTNAACCGTAGGGCNNNNCTAAACAGCCNNNNNNNNNNNYNNACTGCAGATGAGTNGCNNARTYTCNNNCNANNTCNNNNNCATCGTGNNCGGANCGGTCCGCNNNCGNNNNNTCTGATCGNNACNNNNNNNNNCCCCTGCGATCTNNNCGTCCGCGCCCGGNNNNNGTNNYNNNTCCGYNNNACTGATNNNRGGACTTGGATGCCCTNNNNNNNNCNAYNGAATAGRTTGTNNNNCGCCACACGNNGGNNTAGGACGCCNNNGGTNNNNTNNYTNNGNNNNNAAGTTTCCTCGTRTTGCCCCCRRNCAANNNNNCGYNANARNNNNNGGTCNNNNNRGNGCGAATCAGGTNNNNNCTCTGGNNNNNNNANNYGCTGTNNTAAATGNNTACGTCGAATGCACCGCGGCNNTGCTNNNNNNNNCNGNNNNNCATCATNGTGTNNNNNGTGNTNTGAGCTCTNCTTAGNNNNGGACTGNNNNNNCCACGNNNNTGCGTAGAAGGCGCNAATGTGTCCANNNCGTTAGCACGGANCNNNAAAGCNCNNNNNNNNNNNNCCATTYCNATNNAGGTCNNNGTAGGNNTNNNNAATGGCNNNNCCCNNGNGCNACNNNCNNNNNNNNNNTTCACNGGGRNNGAGCCAACACTNNNNTRNNGNNNGCTCNNNAGGTTACTANATTGTNGCGTCNCCNNNNNCRAARNNNAATTCTGNNNNCNCNNANGACGNCGGCATGNTNTCGCTTCACNNNNNATATGTNTNNNNANNNNNCGAAGCCAGGCGYCGYNNNACANANANATGACRAANNNANNNNCGCRNNACGGGACGACGTAGCYANNNNNGNNNCTNNNNNNNNTTNNNNNNGNCNNNACATYRRTCNNNGANNNNNNNNNNNNNCAGACNNNTCAGTCAGCNGGGATTNCNNNNNNAATARNNCRAGTGTNGANNNANNNCGAACCNAGCGNNNNGTGAACAANNCGRAGNGCNNNNNNNNNNNATNTTTCNNTCRGNNNNGCAGCNNNANNANACTCTGTTCCCANNNNNGGTYAGCCCNNARATCTAAGRTAAGCCCCATGAGCNATTNNGAGGCYGAATNNNNANNNNNNATNTTATTCNNCGANNCGACNATAAAGNNCGTNAAGAATTTGTTARCNNNANCNNNNNRRCCRNNNNNYGGYACACAGCTGCCTCRGTACAATGGNACNTCYANNNNNNNNNNNNTAGNGGCNNNRRTTAGTTGGNCCACNNNNNNNNTGANNNNNNNTNCAGYAAGCCNNNNNANNNCNCTNGNNAGCATTTGCATNCNCNNNNNNNNCYRTYTTTNNNNTGNNNNNNNGGCGNNNNNGANNNNCTCGATCTGTGATGNNNNNCCGTCNCAGANNTTACNGGCTGTNNNNTANCRCGGRYTGNNNNCAGATTGTNCTCCAAGNGGYNGATAGRCCYTNANNTRGGGCGTYGRGANATANNNNNNATCGNCNCANGTNNNANNNNNNNRRNNNCCNGANNNNTCGNNCCCACCTGTCAAAGNTACNGNTRNNNCTTCNNNNNNNNNNNNTAGCTNGAACCCTAGATAACGGGGRNNNNNNNGGACTCTNNNNNNNCATNNNNNNNNTNCCCNNANNNTCCTTNNNNNNNNNNGTGGCATGNNGGCNGAGCCCCTNGGCNNNGNCCTNNNNCNTCCGCNAACTNGNNNNNNNNNNCAGAGGNCGNNAGGGGCGCAACNNNNNNNNNNTYYACNNNNAACACYGAGCTATTYNNNNNNNAGCCGNNGNNYATRCCCGTACCANNNNNNNNNNGNTTRTCTGTCNGTRAAGTCATGGCGCCAACTNNNGAGNNNNNGGTNCTTTGTNTAAATGCCNNTTCCGGGAGNATNNNNTCGCTCGATCCCGAGNYANRYTATTAACYANNCATTGNNNNTGCACGGCNACGGCCCGRTNNNNNNNNCTCGNGTNNGTNYGNNNNNNANNNNTTNNATCYGACCCGNNNNNNNRNNNYRTCGGNNNNNNNNNNNNNNNNCTGNNATTNANNNNNNNNNNNNTCGRGCAAAGCRNNTCCNNNNNNNNNNNTCCRTTGANNNNNNNNTCAANNNNNNAGATTTTGTANNYRGCATYCTACANTGGNNNNTTATNNNANNCAGTNYNACNCGGCCTCGCANTGCANNTT

BrM57-1 RYGATTTACTAGCCGNNNNAATGGCCGCCGGAGTCGTCGNYCTNGNNGGGTCNNGGRGGTTTACCCACTGANNNNNNTCTACNNNNNNTNACGCCTYTTGTCNNAGGGNNNCACTNNCCGGGANCNNNCTTGGGTATNGGTTATGCTCCGCTTAGAANNGCCTNNCNGCGYNCAGNNGAGCCGAAAGTAACAGGAGGTTTGGATTTGGCYYNGGGANNNNNGATCNYANNATNNNNNAGTCTNTNNGNNGGTTGGGCANNNNCATCCNNNNNNTCATGAGGAGGTTCTNRANRATCGTGGNNCACYCRCAGGNCNTTGATNNTTYCCAGNNCNNGTNNANARTYCTTCTGATGGCTTAGNYTNNNGARCRGNGGNGCCATTCTGGAGCTGCTAGGNYGGGGCGGGCGCNNCAGGTCAGGAACCNNAGTACATTACAGTGCTGCCAGCTRGYYYCAAANCNNNNNCTCTNTACTGNNNNNTGGGCTGCATTTGNNTACCTTTNNNTGNNNNNNNNNCYTATCANNCACAGCTAATCATCNNNNTYTANACTCAAGTAAGNNTATAGAGCCGNCGNNCACGNTTTCNACACNGACNGGGCGAGGNNNNAACACCNNCGGCTACCAACGNNNNNNATTCCCCNAAAAACANGNGACAGCTGAGGCTGAGCANRCCAANCTCCTCGNTCNGCTCCCTTANNNGCAAGNNCCTGCCGCCGCTTNNGCCAGTACNNAGCATACTGCGCCGNNCGGTGGGTNNCAGRTNRNTCCNNTTGNATGCNNNNGGTNGGCNNNACCAAGGANNNNNNNNNNATNNGCTAGGAACTGGTCTTATGTGARTRCRNNCNNNGNCNGCAGTNCNGNTACAGCNAGCCCTGTACACAACGNANTTGTNCTTAGGCTTGNNNATNYGRNTRAGNTNGNAAAAANGGNNNNYGYGRNCCRGNNNNNCACNAAANNNNTGANTNGNAGTAACTCGCGGTNNNNGCGGCTCATNTATTAGGAANNGCCCGCNGTGTAAAGGCGGGCCGCGTNNNNNNNRGCCGCCTNNCYGCAAGGGNNNNNNNGACGATTAGATTATGTCANNNNNNACGCACNCCNNNYANNANANGTCNYACGCTGTGGCTCCACTTTYGNNNNNCGGGGAGNTNNNNNNNNCCGGGGGCCGCTGACTNNTAGGGCCTNNGAANANNNATGCCCAGACGATCNGGCCAGCCACACCGACAANTTNTCGCAGGGGGCNNNNNNNCGTTTGCTGCAGAGAATTTCTTTGGCCCGACGGTAACGCGTCAGCNNTTNANNNNNNNTANNNGNGNRATCAGGCTCCNNNAATGTNNTTNNGGAATTGACNAGYACTGACATAYACGTNNNNNGCGCCTNRRNATCTCTCCGGGCNNNNRRCGCTCTTGTNGTGGGTTTGCCCCTNNNCAAAGGARGGGAAAAAYNYCGGNNNNCTGAAGACTTTGCGACCCTNNNNAACATAGAGCAANNCCCAGNTNNNCNTATGGTCTCTGNNNGCAGCCGTNCTCNGACTTACNCAGTGGCTTCCGCAGGACNYTRYACAGNNNNNGNGCGAACCTCCNNCNCCTYCNNAGTGGTTTTTYNNNTACCCTACCCNNNNYGYTCNNANGGAATTGCTCTGCTAGTGCNATCGTGAGCCCTATATCTGTTAACYGGGNGGGCACTACTTTCGGACTNGNNNNACTCGATANRAGGTCGGGGATCACNNNGTGCANGTGGACCANNTTAGCCTACTTCATTGNNNNNNNNNNNNCTGTTTGGATATCGTYNNNTTGGTTANNNNCYCGAANCCCGTCCTNCCTCANNNNNNANNGTGACGGTNRGNCNNNGGATNCTCGCTGGACACYTNCYAAAGCGTNCNGNGRNNNCTTCNNNNTCTTTNCNTTNNNNNNNNCCANTTTCGTAATGTGCCGGCACNNNNNNNCTNGCANCGCAANNAGTGTCTTGCAGAATGGCNNAAGYGGCANCGGCGCNTCNYNANCATGCCCCTGGNATGGCNNNGACNNNNNNNNGTNNNNCTCGATGGATCGTGTCCCCACGAGGYATGCGNGAYGAANNNNGAGACGTTNTGACGGGGTTTGTACAAGCTCNNNNNNGGCCAGCCGTNNNNNNNGGACGGTTCGGGCYCCYAGGCCGGTATGGGGYGNNATTGGRGTGTTCGCGAATNCCNRNGNNGTANNCNNNNGCTAATCGGYGCANNGTGGTTANNNCGCCTCGCTTCTGTNNTAANRTTGGCNACGTARATGNNTNNNGNNNNNTGCRGYGGGARNNNGCAAGACCATGGCANNNNGCTCNGCGACCACATTAGTNACYTAATGCTTCNGAGATTGATNGTTCGNGTCCGCNNNNNCGNNGCGAAGNRNCYNAANNNNCCGCTACGCNANNNNCNGTCANGTNNCGTTCAANNTCCGGCGCACNNRGNNNNNTCGCGNYAGCNGGNNGCGARYGATYCNRNNACTAGCNCNACTNCNNNNNNNNNNNNNYNCNGNAAGCCTGCTGNNGNNNNACGACTTGATTAGCNCGTCCCGGNGNRATCTTGCTCNTCGTTAGNGNCTACTCCGGTGACATNCATCGCNNTANACNCACNCAGCAGCGGNNNNNNCTTATAAGNNNCTAGACGCCTNCAGCATCTCGGNCTNNNNCTNNNNCACACCTCYRRNNACCGGTNNNCACTNNGNNCNNGNNCNTGGATAACNNAAACGGGCCTCCAARGNTCGCCATCTTACNAGACTCCNGACAGAATGCGGCGNNTTATNCATGTNNACTTTGAGCGTCGGTGACGATGCANNACCGCCGGTTTATGAACCGCACCNCCGGGNNTTCAGGGNRGGAGACGYGNTNNNTTAATGTGTGTGTCGACCTGYNTCAGCYGRGAGNRNNNNNNNNNGGACACNTGNCCCCTTACTCNNNNTGNACGCYGGNCTCAAGNGGGGGTGACNAANTCRATNCCGGGNCYNAAAGTCGAANATTNCCTTCTAGCTGAAATNCCCCCCNNNCNGNNNNTAANGTTACCNNNNTNNCCGTAANGGGACCCACNNNNATNNCGAGGNNTAAAGCAANCCCGTTGCCCTATCACAGCGGTATTCAAGCCTCACGTCCTTTGGAAGNNNNGGRGTGAAACATTAAGGTGCNGYYGGNNCATNYGNCCCNNRTYANGTGNNNANNANNNNCTGGGTNTGTGNNNGGANTTNNNNNNCCAGTGTCTTGACCTCAGATTTTCGGACNAGCCRCCNARCNCAACCNNNNNNNNNAGCTNTNANNCGGTAGCCCRTNCCNCTARAGGTTACTCCAGNNNACGCNNNAGCATCAAGGYRNAATNANYNTCGTATTGGTNGGACGAGAAGAGTANTATAATCCYYAYNTCNCNGGNNNNNNNNNTGCCAANNGNNNNATCACCATGGAANNNRNYCGCGAGANNCGCTACNTGTCCTTNNNTGATTTGCATTGNAYNNATNGATCNNCGCTGNNNCATCATGACCGTNCGNAGTAGATCNNAGAACCTAAATCGCCGGCTACCCCGACGCCANNCTNNCTCAGCCTGAGNNATTGTATCNAAATNNNCTRTYNNNATTGTTNCGGGCGGNNNCCNNNNATTCTCACCNNNNNNANNCACNGGCNNTTNNAGAANNCNNNCANGCGGTANNCCGTAGGGNGAGGNNNNACAGCCNNTGGGGCCNTYCTANTGNNGANNNNTYGCGCAGACTCGCTCTAAAYYGGGTCCNNNGTGNNCGGNGCGNNCCGCNNNNNGCCYRNCTGATCGGAACCGCNNNNGGTACCTGCGNCCTNNNCCNNNNNNNNNNNGCGCCGTNNNCGTTCCGTGNTACNGNTCCTNNNNNTTAGANNCCCNTGTAAGCACNNTGGAANAGGNNNNACCANNCCACACGRCGGCANNGTATGCYNNNGGTACCTTACCTTAGCNRTCAAGTTTCCTCGTGTTGCCCNCGGGCAATNANGCGTAACGGGATGGGRTYATGGCNNCGNNACTCAGGYRCRRTCTCGGNNCCCRAAAANNGCAGTCATNNANGNNCACGTCGAATGCACCGNNNNNTTGCTTNCGGRTACNNGCGGNYATNNNAGTANTCANGGTGTNGYGNGNTCTYCTTAGGCGCNNNNCGAAATCNCCNCGATCATGCGTNGNNAGNNNGAATATGNNNNAAGCGNTAGCACGGACYNTGNANGCGCGAATATNACGGCTCACTTCNNNACAGGTNTAGGTACGRCYTCACANTGGCTGTACCCNNNGGNGACTGCCNTCNNGCAAATTCACGGGGGGGGAGCNNNNNNNYTGTTGNNGCGGGCTCNCRNGGTNNNNNRAYNNNNGCGNCNNCNNAANCNAAGNAGAGTNCNNCTACCTCNNAGGANGCCGGCATGGTGTNRCTTNACCCNNNNTATNNTTCGCANNRYAACGAAGNNNNNNGTTGTATAACAGAAAGACGACAAAACAAGATACGCAGTTTGNNACGACGTAGCCANNACGCACCCTGANNNTGTTTNNTANNNACACANCNTYAGTCTCTGACTCTACCCAGGTGCAGACGTCTCNGTCAGCAGGGATTTNTANNNNTGTAANNCGNGAGCCGNRNNAACCCGAACCNAGCGNNNNGTGAACAACCCGAANGGCACCRCNCATCTNNANTTNCCTCNNGGCGGAAGCGCCANNAYNCNNAGNTYCCACCNGAGGTNAGCCCTNAAATNTAAGGTAAGCCCYATGAGCGATTNNGNGNCCGGATCCGGNCNTCCNGTNCCATTNCTNNANNCGACNRTAAAGTACGTGAAGANTTNGTTARCTTRACCCGCTAGACCNCRTGTYGGYNCACAGCTANGNNAGNCTNNNGGGACNNCCAAGNTTNACCACCAAGCNGCAGGRRTTAGTTGGNCCNNCNTNNNTATGNCATAATCTACANCANNNNGTTGGGGAACACTCGCCNNNATTTGCATCCANATGCGTCACYRNCTTNNNNNNGNCTGGTYGGCGGAANNGANNNNCTCGATCTGTNNNNRNTAGCNGTCNNNNNGGTTACCGGCTGTNNTRNNCCRCGGACTGNNNCCAGANTGTTCTCYANGNGGNNGATNNNNNNTNACTNNNNNNNNNGRGAYATNATYGCNANCGNNNCARGTCCTAGGAACGNNNGATCCTGATCGCTNRAYCCYACCTGNYANAGNNACNGNNNNNNNNNNTCAGCGACTGGCTAGCTTCAACCCTAGCTAACNNGGGTGTTAAGGGACTCTRGNAGGGCNNCCTTGGRNTGTNNNNATGANNCTTAGACCGNNNTGYGGCATGGRGGCANNNCCCNTRGGCNGANNNCNAGGCCGTCCACNAACTNGYCANNTGGGCCAGAGGCCGACAGGGGCGCAACNNNNNNNGGCTTCACGGAGNNNACYGAGCTATTTTAGCAGGAGCCGGTGTGCATACCCGTACCANNCGNNTGAGNNNTATCTGTCRGTANARYCATGGCGCCAACTGAANNGTGCCYGGTNCTTTGTAYAANTGCCCTTTCCGGNNNCATYNATNNGCNCNNNNNNNNNNTNNGCYATTRRYYAGGCATTGCCTTTGCACGGCANCGGCNNGNNGTCACGACCTCGGGNNNGTATGTGGCAGNCTTTTTAGATCCGACTCGATTACCCAAGANNGAGGCCATAGTNNNNNTNCNCTNGGATNNNTNGTGAAAGTGATCGGGCAAAGCACTANNNGCTGNNGATGTCCRNTGATGACCGAANCRAGNGATCANANYTNGNACCNNNNNNNNCNYAATGGCGAGTNNNYRYNNTCNNNCYNACGCGGTCTCGCANTGCGNNGG

BrM59-1 GCGATNNNCTNNNNNNNNNNNNNNNCGCCNNNNTCGTCGACCTNNNNGGGNNNNNNNGNNNNACCCACNGAYNGYNNNNNNNNNNNNNNNNCGCCNTNTGNNNNGGGANNNNNNNNCNCRGNNNNNRTNNTGGGTNNGGGNNATNCTNCGCTTANAANNNNNNGGCAGCGNNCANGANNNNNNNNNNNNNNNNNNNNNNTGGANNNNNNNNNGGNANNNNNNNNNNYNCANNNNNNNANNNNGTNNNNNNNNNNGGNNCGCAGNTCNTAGGNNNNNNRAGNARRTTYTNNANANNNNNNNNNNNNNNNNNNNNNNTTGACNANTYCCNANNNNNNNGGATNNNNNNNNNNNNNNNNNNNNNNNNNNNNNNNNNNNGNNATTNNNNNNYTGCTANNNNGGGGNNNNNNCNNCAGGNNNNNNNYCTNNNNACNNNNNNNNNNYGNNNNNNNNNNNNNNNNNNCYCTCTCTNTANNNNNNNNNNNNCTNNATTTNGTNNNCTNNGNNTNNNAAYGANNNNNNNCRGACNNNNNNNNNNNNNNNNNNNNNNNNTAGTGTGNNAGNNNNNNNNCGNCGNNNNNNNNNNNNNNNNNNACGNNNNNNNNANAGNNNNNNTCNNNNNNCCAANGNNGGANNNNNNNNNTANNNNNNGNGRYNGCNNNNNNTGAGCAGRCCAANNNNNNCRNNNNGCTCNNNNNNNNNNNNNCCCCNNNNNCCNNNNNNGANNNNNCCTNNNNNACNNNGCCGTNNNNNNNNNNNCNNNNNGCTCCNNNNNCATNCNNNNNNTNNNNNNNNNNAANNNNNNNNNNNNNGGTANCTAGGNACTGATATNNNGYNAGTRNANNNNNNNNNNNNNNNNNNNNNNNNNNNNGCCCTGTACANNNNNGNNNNNNTCTTAGNNNNNNNNATNYGRNTRNNNNNGGNNNNNNNNNNGCNNNNNNNCNNTCAGTNNNNAAANNNNNNNNNNNNNGTAANNNNCGNNANNNNNNNNTCNTNANNNNNNNNTANNNNNCGGTGTANNNNNNGGCCGCGTNNNNNNNNGCCGNNNNGYCGCNNNNNNNNNNNNGNNNNNNNNNNTATGTCNNNNNNNANNNNNNNNNNNNNANNNNNNTANNNNNNNNNGGCTCCANNNNNNNNNNNCGGGGNNTTNNNNACGTNNNNNNNTYGNNNAGTNNNNNNNNNNNNNAACRNGANNNNNNNNNNNNNNNGGCCNNNNNNNNNNNNNNRTNNNNNNNNNNNNNNNNAGNNCGTTTNNNNYNNNNNATTTNNNNNGCCCNNNNNNNANNNNNNRGYNNNNNANNNNACANNNNNNTGTAATCNGNNNNNNNNNNNNNNNNNTAGGNANTGANNNNNACTGACRNNNNNNNRNCNNGCGNNNNNNNNNNNNTCCGGGCNGNNNNNNNNNNNNNNNNNGGCNNNNCCNNNGACRNNNNNANNNNNNNNNNNNNNGACTCTGNNNACTNNNCNACCNNNNNNAANATAGAGCANNNNNNNNNNTNACNTANNNNNNCTGNNNNNNNCCGTNNNNNNNNNNACCCAGTGGCTTNNGCANNNNTTTGTACAGNNNNNANGNGAACNNNNYGNNNNNNNNNNGNGGTTTTTNNNNNNNNNNNNNNNNNNNNNTNNNACGGAATTGCTCTNNNNNNGCTGTTGNNNNNNNTNNNNCTRTTAACNNNNNNNNNACTNNTCTNGGNNNNNNNNNRCTNNNNNTNAGGTCGGNNAYYNCNNNNNNNNNGTGGACTGTNNTAGNCNGCTNNNTNNAGATGGCANGGTCNNNNNNGATNNNNNNNNNNNNNNNNNNNNCTCGNANCCNNTCCTCNNNNNNNNNNNNNNGTGNNGGTNNNGNNNNNNNNTCNNNCTGGACAANNNCCANAGCNNNCAGAGGATNNNTCNNNNNNNNNNNNNNNNNNNNNNCCNATNNNNNNNNNNNNCGGCNNCGTNNNNCTNNYACNNNNNNNNNTNTCTTNNAGANNNNNTCAAGNNTNNGCNGNGCNNNNNNNNNNNNNNNNNNNNANNNNNGCNNNAGCNNGCCNNNNNNNNNNNNGGCTCGCGNYCCCACGAGGNNNNNNNNNNNNNYNNNGAGNNNNNNNNNNNNNNNNNNNNNNNNTTCCANNNGNNNCAGCNNNNNNGCNNGGNNNGNNNNNNNNNNNNNANNNNNNNNNNGNGTAATTGGGGTNNNNNNNNNNNNNNNNGNANNACNNNNNNNNTAATNNNCGCATNNNNGTNANNNNNNNNCGCNNNNNTNNNNNNNNNNNNNNNNNNNNNNNATGTNNNACNNYGCNGYRGNANNNNNNNNNNYCRYGNYRNNNNNNNNTGCGACCANNNNNNNNNNNNNNNNNNNNNNAGANTNNTYGTNCGTNNNCNNCNCYGCGNNNNNNNNNNNCTCNNNNNNCCNCTRCGCGNAAGCNNGTNNNGTNNNNNNCAANNNNCNGCNNNNNYGRANNNNTCGYGNYAGNNNNNTNNNNNNNNTYCNNNNNCTAGCRCAANTGCTNNNNNCGGCCGGNANNNNGANCCNGCTGNNNGGNNNNGACTTNATTAGCCCGTCNNNNNNNANNNTTGCTNNNNNNNNNNNNNNNCNNNNNNNNNNTACATCGCATNNGACCCACNNNNNNNCNNNNNNNNNNNNNNNNACTNNNNNNNNNNACANCRTNNNNNNNNNNNNYTNNNNNNAACTCNNNNNNACCGNNNNNNNCTNNNNNNCNNNNNNTGNANANYCNNNNNNNNNNNNNNNNNNNNGCNNNNNNACAAGACTCCYGNNNNNNTNCNNYGNNTTNNCNNNNNNNNNNNNGAGCGTCNNNNACGANNNNNNACCNNNNNNNTATNNNNNNNNNNNNNNNNNNNNNRGNGNRNNNNNNNNNNNNNNNTANNNNNNNNNNNGNNNNNTNTYAGCCNNNNNNNNNNNNNNNNNNNCACNNNNNNNNNTNNNNCGCANNGNNNNNNNCNNNNNGCNNNNGTGNCTNNANCGNNNCCGGGNNNNANNNTCGNNNNTNTNNNTNTANNNNRRNNNNNNNNNNNNNNNNNNNNNNNGTTANNNNNNNNNNNNNNNNNNNNCNNACNNNNNNNNNGANNNNTANANNANCNNNNTTGNNNNNNNNNNNNNGTATTNNNNNNNNNNNNCCNNNNNNNNAGACNNGGNNNNNNNNTNAGGYRNCGTCNNNNNNNCNNNNNNNNATCACNNNNNNANNNGTCNNNNGGTATNTGAGAGGANNNNNNNNNNNNNNNNNNNNNNNNNNNNNNNTCAANNNNNNNNNNCNNNNNNATNNNNNNNNNNAGNNNNNNNNNNNNNNNNNRTNNNNNNNNNNGYNNNNNNNNNNNACGCNNNNNCNTCAAGGTANNANCANNNNNGTNNNNNNNNNNNGARNNNNNNNNNNNAATCCCTATNNNNNNNNNNNNNNNNNNNCCGRNNGNNNNNNTACNNNNNNNNNNGTCCGCGAGNTGCGCTACNTNNCCTTNNNNNNNNNNCNNNNNNNNNTANNNNNNNCGNTGNCTNNNNNNGRCCGTACNNAGAAGNNNNNAGAACCYAANTCNNNNNNNNNNNNNNNNCCAGCNNNNNNNNNNNNNAGCTANTNNNNNNNNNNNGNNNNNNNNCNNNGTAGTTGNNNNNNNNNNNNNNTNNNNNNNNNNNNNNNNCACNNNNNNNTNNNNNNNNNNNNNNNNCGGTNNNNNNNNNNGNNNGGNNNNNNNNNNTRNNNNNNNNNYNNACTGNNGANNNGTNGCGCAGACTCNNNNNNNGNNNGGTNCANNNNNNNCNNNNNNNNNNNNNNNNNNNNNNNCTNATNGGAACNNNGACGGGNNCCTGNNATYTNNNNNNNNNNNNNCGGGNNNNNNNNNCGTTCCGTRNNNCNGNNCCTNNNNCTNNGATGCCNTNNNNNNNNCNNNNGNNNNNGNNNNNNNNCGNNNNNNNNNNNNNNNNNNNGCCNNNNNNNCNNNNNNNNNNAGGTNNNNNNNCCTCGTGTTGCCNCCAANNNNTNNNNNGYNANNNNATGRGNTNNNGGCNNNNNNNNNCNGNTGCNNTCTNNNNNCCCNAANNNTGCTGTCANNNNNGCGTACGTNNNNNNNNNNGNNNNNTTGCNNNCGGRTNNNNGCGRCNNNNNNNNNGTNNATGNNGTNNCNNGNNNNNCTTAGNNNNNNNNNGNNNNNGCCNCGAYCNTGNGTAGAAGGNNNANNNNNGNNNNAANNNNNNNNACGGANNNNNNNNGCNNNNATNNNNNNNNNNNNNNNNNNNCNNNTNTNNGTANNRCNTCACANTGGCNNNNCCCNNNGGNGNNNGCCGTTGCNNNNNNNNNNNNNNNNGGANNCAACACTNNNNTAAAGTNNNNNNNCNCGNNNNCNNNNNNNNNNNNNYTCCNNAATCAAANNNNNNNNNNNNNACCACNNNNNANGNNNGCATGNNNNNNNNTNNNNNNNNNNACNNNNNNNNATGCNNCGNNNCCAGNNNNNGTNNANNNGAAAGNNNNNNANNNNNGATACGCNNTACGNNNNNNNNNNNNNNNNNNNNNCCNNNNTCGTGTTTNNNANNNNCNNNNCNTNNNNNNNNNNNNNNNNNNNGRNGCNNNNNNNNYNNNNNNNNGGGNNNNNNNNNNNNNNNNGNNNNNNGNNNNNNNNACCCGANNNNAGCNNNNNNNNNNNNNNNNNNNNNNNNCCACNNNNNNNNNTTTCNCTCNNGGCGNNAGCGCCNNNANNCNNTGNNCCCANNNNNNNNTAGCCCNGNNANCNNNNNTAAGCCCCNNNNGNGATTNNGNNNNNNAATNNNNATGTCCGNNNNNATTNNNNNANNCGANNNNNAAGNNNNNGNNNNNTTTGTTARCNNNNNNNNNNNNNNCGCRNNNYGGYNCACAGCTNNNNNNGTNNNNNGNNNNNNNNNNNNNNNACNNNNAAGNNNNAGGRRTTAGNNNNNNNNNNNYNAANNNNANNNNANNTACANNNARCCGATNNAANNCNNTNNNNAGCATTNNCNTNNNNNNNNNNCNNNNNYNNNYCYRTGNCTANCNGGCGGAANANNNNNNCNNNNNNNNNNATGNNNNGCNNNNNCAGANNNNNCNGGCNNNNNNRNNNNNNNNNNNNNNNNCAGATTRYNCTCYNNNNNNNNNNNNNNNNNTNNCTNNNNNNNNNNNGACNNNNNNNNNNNNNNCYNNNGTNNNNNNNNNNNNNGATCCTNNNNNNNNNNNNNNNNNNNNNNNNNNNNNNGNNACGTNNNNNNNNCGACTGGCTNGCTYGNNNCCTAGATANNGGNNNNNNNNRRGGANNCTNGNNNRNCANNNNNGGNNNGNNNNNNNNNNNNNNNGACNNNNNNNNNGCATGNRGGCANNNCCCNTNGGCNNNNNNNNAGGCNNNNNNNNAACNNNNNNNNNNNNNNNNNNNCCGNCNGRGGCGCAACNNNNNATAGCNYCANNNNNANNNNNNNNNNNNNTNNNNNNNAGCNNNNGNNNNNACCNNNNNNNNNNNNNTGRRGCTTANCTGTCNGTNNNNNCATGGNNNNNNNNNNNNAGTGNNNGGTANNNNNNNTAANTNNNNNTTNNNNGAGCATNNNNTCNNNCGGNYCCGAGNNNNNNNATNNNNYANNNNNNNNNNNNNNNNGGCNNNGGNNNNNNNNNNCGACNTNNGNNNNNNNNNNNNNNNNNNNTNTANNNNNNNNTCGATNNNNNNNNNNNGAGNNNNNNNNNNNNNNNNNCTNGGANNNATCNNNNNNNNNNTCGGGNNNNNNNCTNCCNNNNNNNNNNNNNNNNTGANGACCGNANNNNNNNNNNNNATTNNNNNCCTAGYANNNTACANNNNNNNNNNNNNNNNNNCNGTNNNNNNNNNNNNNGCANTGNNNNTT

BrM60-3 ACGNNTNNNTCGTCANCNTNNNNNNNNNNGGAGNNGTCGACNYANAYGGGTCTAGGAGGTTTACCCACNGACTGTGATCTNNNGNNNNTCNCGCCTNTTGTCTRRGGANNNCRCTGCNNGNGANCNNNCTTGGGTATNGGYYATNCTNCGCTTANNNNNGCCNGGCAGCRYGCAGGAGAGCCGNNNNTANNRRNAGGTNTGGANNNNGCTTNGGANNNNNNGANNANAYAATACGGCAGTNTNTNNNNNNGTTGRGYACGNAGNTCNTAGGYCTTGTAAGAAGGTTTTNNNCGANCGNGGATCACYCRCANNGCATTGACNANTYCCAANNTCCGTGGATAGYYNNNNNGATGNYNTAGATTGANNAACGGTGGAGNNATTCTGGAGCTGCTANGNTGRNGCGGGCGCCNCAGGTTCGGAANYYARRTACANTACATTGCNNNNNNNNNNNNNNNNNRCRCYCTCTCTYTACTGNTGANNGGNCTGCATTTNGTTACCTNNGGCNNYNAANGAAYNNNNNNNNNNNNNNNNNANYNNNNNNNTCTAGACNNNNNNNAGAGTNNNGARCCGACGCGNNNNRTNNYAACACCNACGNNNNRAGRNANGAACRYCNNCRRCTANNNNNGNNNNNCNTNCNCCTTARAAYNAGNGRYCGYNNAGNNTGAGNAGGCCNNNCTCCNCGATCNNCNNCNNTRNNNGCAAGCCCCTGCCGCCGCTTACNNNAGTACCTNGNATANTNNGCCGTNCGNNGGGTANCARGNGGCTCCTATNGCNTGCGNATGGTNGGCNNTACCRNNNNCTRNNCGGYTGGNNGCNANNAACTGATNTTATGTGAGTACATGCNNNGCNCGCNNNNNNNNTACARCTARCCCNNNANNNNRNNTRAYCRTCCNTTNGCTNNCGAATTTGACTGAGTTCGNAAAAACGNNNGCCNTGGTCCGGTCAGTNNNNAAAGTTGYRANTNGNAGTNACTAGCGRTAGCCRYGNCTCNTNNRTTNNNNNTARCCCNCGGNGYNNNGNCGGGCCGCGTNNNNNNNRGCCGNNNNNCCGCRAGGNAYANNNNGACGAGCAGATTATGTCANNARRAACGYACTNNTCTNNNNNNNNNYNNNACGCTGNGGCTCCAYTTTYNGCGRCCGRGGNNNTNNNNACGTCNNNNGGTCGNTRAGTACTNGGGNCTAGAAACGTGAATGCCCAGACNNNNNGGCCNGCCACACCGACAANTTCTCGCAGGGGGCGCCAGNNNNNNNGNNNYNNNNNAYYTCNNNNGCCCNNCGRYAACGCNNNNNNATCCGNNNNNACANNNGNGNGTAATNNNACNCCNNNAATGTNNATNAGGAANYNANTAGCACTGACRCNNANNNNNNNNNNNNNTNGGTANCTCTCCGGGCGGNNNNNGYNNNYGNNNNGGGCTYGCCCCTCGACGANGGAAGNNNNNAACYTCGGGACYCTGANNACTYNNNNACCCTNNNNNNCATNGAGCATNNNNNAGTNTRACATNNGGTNNCTNNGAGCAACCGNCCTCTGACYYACCCAGTGRCTTCCRCAGGANNNNNNACAGCCAGAAAGCGAACCNNNYGCNCNNNNCAAGYGGTTTTTNANRTACCCTANNNANGTCGNTCNNACGGAATNNNNCYGCYNGTGCTGNNNTGAGNNCTATATNNNNNNACCNGGTGGNNACTACTTTCRRACTCGTAGTRCTNNNNNTRAGGTCGGNNRYYACTATNNNCANGTGGACCANATNNNNNNNNNNNATNNAGATGGCATGGNCTGNNTGGATNNCNTCCGCNNNNNNNNNTGCTNNAANCCCGTCCCCNNNNNTCCNNNAGAGTGACGNTGAGGCNNCGGATTCTCNCTGGACANTTTCCARAGCNTCCAGAGNATNNNTCGTTCTCTTTCNNNNNNNNCTAGCCAATTTCGNNNNNNGCCGGYNNCGNANNNCTGGYACNNNNNNRNGTRTCTTNNANNATNNNTCAAGCNNCNGCGGYGCGTCAYNATCNNGCCCCTNNCATGGCCGCGACAGCNRGCCNNCCCACTCGATGGAGCNNNNCCCCACGAGGYAGGCGGGANNAAYACGGAGACGNNATGACGGGGTTTRYANNAGNNNCACACGGTTNNNNCGTTRNNNAAGGGCGNNNNGGGCCCCCAAACCGGNNNGGGGYGNNNNNNNNGTGTTCGCRAATACCTNNGGANNACNCNNNNNNNNNTCGGTGCNNCNNGNYNAGGGNNNNNNGCTTCTNNATTAACATCGGCAACGTAGATGNNNGNGGAACTNYNCRGYGGGARNNNGCGAATNCNNGGYRRCGNNNNNNGCGNNNNCANNNNTTANNNAATGCGYNNNAGAATGATNGNNCGTACCCNNCCCCGCGGCGCGAAGNNCCTNNNTGNNCCNCTACGCRAAAGCNNGTCAAGTAANGCCCAAYNTCCGGCNNNNNNGGANNGGTCGCGNNAGNNGGCTGCGAGNNNNYCCNGGNNNNGCACTACNNCNAGAAACGGCCGGYNCAGGRANNNTGCTGNNNGGYTANGRCTNAATTANNNNGCNCCGGAGCNATCGTCCYNGTCGNNNGGGGCNNCTNNNNTGGCGNNCATCGCATTAGACNCACGCANNNGCRGNNNNNNCTNACAANACNCTNNNNNNNNNNNGCRTCTCGGGCTAATACTTCTCCNNACTCCTGATCACCGGTGGCCACTAGGCGCCGTATCCNNNATANYCGAAATGGGCTNNCNNAGCTCGCCRNCTNACAAGACTNCTGAYAGTGTGNGGCGYNTTNNCCGCGTCNNNNYTGAGCGTCGGTGNCGATGCACGACNNCCGGTTTATGAACCGCNNNACCGGRNNNNNGGNNNNRGAGACGCGGNNNNTTANNGCNNGTGTNGACCTGTNTNAGCCGAGGAAATGGRANCGNRNTCACGYGANNNNNTACTNCGCATGGACGCNNGCYYNNANNGGGGGTGNNTACACCNATCCCRGRTCYNAAAATCGNNNANNTNNTTCAAGCCGAAATACCCCCNRAYCNNNNNNTAAAGTTACCACGCTTANCRTNNTNNNNCYYACCGGGNTTGCGAGGATNANAGCNACNNNRNTNNTCCGYCACANNNGTATTANNNNNNCGCGTCCTTTATGGGAGACNNGGTGAAACATTNNNNNNCNGCTGGNNCGTCTGNNNYGYNTCACNNNTGTAATAGTCNNTRGGTATGTGAGAGGANNNCGNNNNCNNNNGTTTTNAYNNNNNNNNNTNNNGTNNNCNNNNCAGCTCCGCAGACRNNNNCAGCTCNCAANCGGNAGTCNNTNCCNCTAAAGNNNANTCNNNGNNACGCGGAAGCCTCAAGGTACAATCNTTNNNGTRTTGGTCNNACGARAAGRGTANNNNAATCNCTATNNCGCAGGAATGGTCCATGNNNRAGGAGNYATCACCAANGNNAGTAANCGCGAGATGCGCTACNTGTCYTTTGTNAGTCTACNNNGCATNNNNTAAACGTCGCNGNNNTATCNYGACCGTACGCACAAGATCTTAGAACCNAAATCGNCGNNNACCCNNACGNNNNNNNNNTACGNNNNNAGCTACTGTATCTAAATCGACTRTYYYCNTNGNNNNTGGCGGRYCCCNNNTNTNNNNRYCGCAAGGNNNCACAGGCAGTTNNNNNNNNNATANNCGCGGTAANCCGTAGGNNNNGGYTACACAGNCNGNNNNGCCANCNNACTGCAGANRRNTCGCNCAGTCTCRNCNTNNNNNNGNTCNRNNNNNGACGGANCGNTCCGCAGGCGGCGNNNNNGATCGNNACCGCATTGGGNNCCTGNNAYCTNNNCGTCCGCGCCCGGGYGCCGTGGNCGTTCCGTATARCTGATCCNGGGACTTGGANNNNNNTGTAAGCACNATGGAATAGNTTGTACCACGCCNCACGGCGGCANNGGACGCCNNNGGYACNNNNNNTNNNNNNNCAAGTTTCCTCATGTNNCCCNCNNGCAATCATNNATAAAAGGATGNGRTCATNNNNGCNCGAATCAGGTGCGGYCTCTGNNNNNRAANAYYGCNNTNNTNNANGCGTACGTNNNATGCNCCGNNNNNNTGCTNNCGGAYACNNGCRGNCATNNNNGYGTTCATGGTNNTGNGAGCTCTCCTTARNNNNGGACNANNNNNACCACGANCATGCGTARNAGGCGCRAATGTGNNCANNGCGTTAGNACGGANNTTGNAAGCGNGNATRTNACGGCTCACTYCNATACNNNTTTAGGTACGNCTTCACANTGGNTNTACCCTCGGGNGACYGCCGTTNNNNNNNTTCACCGGGGGGGANNCAACACTCTGNTNNNGTGGGCTCTNNAGGTTNNTAAATAGTGGCGTNNCCCTAANCRAANNRRNNTTCNNNNRYCNCGGARGNNNNCGGCATGGTGTCGCTTNACCCTTCATANGTATYGYAATNNAACGNAGCCAGGCGTCGYAYNNNNNANARNNNNNNAAACNAGATACGCAGTACGGGACGACGTAGCCACCNNNNACCCTGATCGTGTNNYGNACYGNCNNNACNTNNNNNTCTNNNNNNNNNNGGNNNNAGACNYCTYANTCAGCNGGGATTNCNNNNNNAATARGACGNNNNYNGARNNAACCCGAACCTNNNNNNNNGTGAACAANNCGNAGGGCACCACACANCTATATNTCACTCGGGGCGGCNNNNNNTNNANNCNNTGTNNCCACCTGAGGTNNGNNCNRARATCTNAGNTAAGCCCCATGNGCGATTGGNAGGCNGAATTTGGNTGTCCGNNCCTATTNCTCNRACCNNCGATAAAGNACGYGAAGAATTTGTTARCNNNAYCCRNNRNNNCGCNTGTYGGTNCACAGCTNNNNNRGTNYAATGGNRYNTCCAAGGTTTNCCNCCAAGCGGCAGGAGTTAGTTGGNCCRYNCTCRRTATGAYAYNAYNTACAGNANNNNNNTRRAANNNNNNCGCCAGCANNNNCATNNNNATGCGTCNCYRTNTTNNCNNTGTCTGGCNNNNNGAANNGARGNTCNNNNNNNNTGNNGAANNNCNNNNNCNGANGTNRCCGGNTGTYRTGNNNCRCGGNNTGNNNCCAGATTNNNCTCCAAGTNGNANATNGRCCYTNAYTTRGGGCGTCRAGACAYAATCGCNNTCGNCTCANGTNNNNNNNNNNNRRGATNNTGANNNNTCGNNCCCACCTNTCAANGYTACNGNNRCGTCTTCNNNGNNNNTGGCTAGCNNNAACNCTAGATAACGNGGNNGTNNRRGGANNCTNNNAGGGNATCCTTGGRNTGYNCNNGNGANYNNNAGNNNGGCTTGTGGCATGGRGGCNGAGNNNNTRGGCTGANNNCTRGNCNGTCCNCAAACTNGYCANNNNNACNRNAGGCCGACAGGGGCGCAANNNAGCNNNGCTNNACTAANAACACNGAGCTATNTTAGCAGNAGCNNGNGTGNNNNNNCGTACCATTCGCTTGAGGNTTNTCTGTCRGTNNANNCANRRCGCCAACTGRNGANYGNNYNNTNCTTTGTATAAATRCCCTNNNNNNNNNCATTGNNNNGCTCGNTCCCGAGNYANRYNNCNARYNAGACATTGYNNNNNNNNGGCAACGGNCCGATGTCANNNNNNCGRGNCGGTNYGNNNNNGANNNNNNNGATCCGACTCGATTGCNNGGCANNNNNNCCCCAGCATCCTTNNGNTGGGATTTATYNTAAANNNNNTCGNGCAAAGCNNNTCCTGCTGNNNNNNNNNNNTGANGACCGGATCAAGNGATCNGNNNTTGTACCNNNNNNYYYAYANNNNNNGGNNNNYRCANNCAGTNNNACGCGRYCTCGCANTGNNCATT

Gv10-A-01 RCGATTTACTNNNCGCCATAATGGCCGCCGGAGYCGTCGACCTNGACGGNTCTARGRGGNNTACCCACTGANNNNGATCTACNGGCCAYCACGCYYYTTGTCTARGGRGYCCACTGCTCGGGATCCGTCYTGGATATNGGCTRTGCAGCGCYNACAATGNNNNGGCARCGTNCAGGANRRCYRNANGYANNGRCANGTTTGGACCTGGNCCTGGAACGGTAGAYYNCRCAATACGGCAGTCTNTAAGTAGGTTGGGTACGCANRYCNTAGRTCTNNTGAGGARGYTYTGAACRNNNNNNNATCACYCRNNGGGNATTGATNATTCCCNANGCCCGTGGATRGYYTGGTTGATGATGTAGAYTNANNAACGGTGGNNCCATTCYGTAGYTGCNNGGNYGGGGCGGGCGCCGCAGGCCAGGAACCTAGGTACANTACANTGCCGCCAGCTRRYCYYARAGNNCCCTCYCTTNNCTGCTGAATGGGCTGCRTTNGGTTACCTTYNGCYGYCAATGAAYCCTNNNNNNCAYAGCTAANYATCCTNGTCTNRACYCGTNTRAGAGTNTAGAGYCGACGCGNACGGTNTCANCACCRACGGGGCGAGGAAAGAACGCCTCCRRCNRCNAACGGTGGACATNCNCCTNRRAAYAARCGRYNRCGGAGGNTGAGCAGGCCNNTNTCNTCGATCTGCTCCCTTATGTGGAAGCCCCYRCCNCCGCTTACGCCAGTRCTTAGCRNANTGCGCCGTGCGGTGGGTACCAGGAGRNTCCNANYNCAYGCGAATGNCNGGCACTACCGTAATCTAGCCGGCTGGTAACTANGNNNNNNNNTTATGTGAGCGCGTGCCCCGCYCGCANTACAGATACAGCNAGCCCTGTACACAACGGAATTGTTCTTAGGCTYGCGAATACGGATANGNTCGNAAAAACGGNCGCCNTGGTCCGGNNNGCCACTRAAGTTGCAAATTGTAGTAACYCGCGATAGCCATGGCTCATANATTAGGAATAGCCCGCGGTGTARRGGCGRNCCGCGTCGGCGAGAGCCGNCTTRCCGCRAGNNNTANNCAGGCGNTTAGATTATGTCTGAAGGAACGCACYCCTCNTAACNNAGGTNAYNNNNNNTGGCYCCACYTTCNACGGCCGGGGGATTAGGCACGTCCGGGGGCCGNTGAGTACTAGGGCYTNRGAACGTGAATGCNCRGACGATGGGGCCAGTCACCCCGNNNNRTTCCYACARGNRGCATCAGTTNGNTNGCTGCRRGGTATTTCTATGGCCCGANRGTAACGCGACGGTNNTTGNAAAGACAYAGGTRCGTGATCGGACTCCNGAARYGTYRNTTAGGAAYCCACTAGCACTGACRNNTAANYATCAGGCGCATCGGTANCTCTCCGGGCGGCCAGCGCGAATGAAATTNNCTNGCCCCTGRNCRAAGGNAGGGAAAAACCTCGGGACYCTGAGNNCTCGCCGACCCTARTGAACATAGAGCATCGCCTAGTGNGACATATGGTCTCTACGAGCARCCGTNNNNNGACCTACCCAGTGGCTTCCGCNGGACTTTGTACAGCCAGAAAGCGRRCCCYCCGNNNCTCCCAAGTGGTTTTTCNAGTANCCTAYCYACGTYGCTYCRACGNAAATGNTCYGCCTATGCAATCGTGAGNCCTATATCTATTAACCAGGTGGGCNCTGGTCTNGRGGNCGTAGTACTCGAYANAANNYYGGRNNYCACTANNTNCAGGTGNACCGNATTAGYCYGCTTYAYTGAGATGGCATGGTCTGNNTGGATATCGTCCGCTTGGTTAACTGCTCTAANCCCGTCYTCACGCRTCCCGRTGAGTNACGNTGGGTCCGGGGANTCTCRCTGGACANYTTCCAANGCGTCCAGNGGNTANNTCGTTYTCTTTTCATTTACGCTACNCANTTTCGTAATGTGCCGGCAGCGTAGAGCTGGCACCGCAATGGNTRTCTTGCAGAATGGCYCAARYANCRGCGGCGCGTCACTATCGAGTCCCNNNCACGGCGNYGACAGCNRGCNNNCYCRCCCAATGGCTCGCGTCCCCACGAGGCAGNNNGGAYGAATACGGAGANGTTATNRCGGGGNTTACACNCATTCCACGCGGNYNNNNNRTTGYGNANGGNCGGTNNGGGCCCCCAGACCGGTANNNNGCGTAATTGGGGTGNNNGCGRNNACNTRNGGARTNNTCGACCGCNARTCRGCGCATCGTRGCNNGGGCGCCTYGCTTTTRTAYTAANATTGGCTACGTNGATGCATGTNGAACTTNGNNGCAGNRRAGAGTGARTCCGTGGCAACGGGCTCTGCGAYNACATTAGTNAYYYAANGYGTCAANGATTGATTGTNCGTGTCCNYCCYCNCGGNGCGAAGNRNCTAAAARGCCCGCTRCGCANAAGCCTGAAGNGTAACGCCCRNTCTCNGNCGCACNYRGNGNGGTYGCGATAGCGGGYNGYRRRCGATTYGAGGAYTAGCRNNACTNCTAGAAANNGCCGGTACAGGRAACCCGCTGGTAGGTTACGRCTTNATTAGCCCNCNYCGGAGCAATCNTNCTCGTCGTTAGGNGCTACTTCGGTGGCGTACANCGCNCTATACCCACGCAGCNGCAGCAGCGTCTNACAANACTCTARACGCCYNCANCRYCTNNNNCTNNTNCTTCTCCANACTCCNNRTNACCRGTGACCACTAGGCGCCTGTTCCNNGATANNCGAAAYGGGCTTTYAAAGNTCGCCATCTTACAAGACYCCTGATATTGTGCGRTGTTTTATNCATGYCANYGCTGAGYNTNGGNGAYGNYGYRCGACCGYYGNTTTRTRARCCRCACCATCGGGNNTGCNNNGNRGGAGACGCGGCRCYNTARCGCAGGTGTCGNNNNNYATNAGCYRRRAGNRTGGGACYGCRRNCACGNGACCNNNGACTCCGCACGGACGCYGGCCYCNAGCNARGGTGTCCACAYCGATCCCGGGCCTGARARTCGAACANCNCCTNNNNGYCGARNTACYCCCCGATCCGCNNNTANAGTYRCCACGCTTATNRTNNNGGGACCCACCAGCTTTGCGAGGATTANAGCAACTCNRTTGCTCCGTCGCNNCGGTATNAAAGCCTYACRTCCNNTGGGGGAGACGGAGTAAAAYATTAGAGCACYGNNGGNCCATCTGNNNYRYNTCACGAGTGCAATAGTNACTAGNTATNTGAGAGGAGTTCGRGYRCCACTGTCNNGRCCTCNNNTTNTCGGACTAGCCATNCNGCTCAACAGACNNAGGYGRCTGTNNAGCGGTAGYCYATCCCNCNNNATGCGAYTCCAGNTGNCGNRAAARNNCCAAGGTAYAATCATTCTNGTGTTGNNNGANCNAGAAGRGTAATATARTCCCTNTTTCGCAGGNNTNGTTCGTGCCGAAGGAGTTATCACCAATAAAAGTGTYCGCGAGATGCGCTACNTGYCCTTTGTCAGTCTACACTGCATAATANAATCGTCGCTGCCTCNNCATGRCCGTACGCANAANANCNNAGARNYYAATTCGCCGRCTACCCCGACGCCAGNCTCGCNCAGCCAGAGCTAYTGTATNNNAATGGACTATTTCCATTGTNGYNGRCGGGCYCCTCTTAAYNTCGTCNNNNGAACTCACNGGCAGTTTGAGAAATCATACACNCGGTANACCGTAGGGCTAGGCTNCNCAGCCTGAGAGGCCATCCTAYTGNNGATGAGTCNNNCAGNCTCGNCCTGGATCGGGTYCACAGNGRNCAGACCGGTTCGCNGGCGGCNCACCTGNTCGGAACYRNGTCGGGYNCNTGYGACYTACGCGTCCGCGCCCGTGTGCCGTGGCCATNNCGTRGNACTGATCCTGGGANNTGGATGCCCTTGTAAGCACNCTGGAATGTATTGTACYRCGCCACACGACRRCNTAGTACGCCAGCGGTACCTTRYNNTAGNGNTNAAGTTTCCTCGTGTTGCYCYCAGGCAATCANGCGCCANANRRNGGGGTYATNGYAGCNCGAANCNGGTGCGGNCTYNGNTCCCAAAAATTGCNGTCATTTATGCGYACGTYGAANGYANCGCGGCANTGCTTGCGGATACCGGCGGCCGTNATAGYGTTCATGGTGTNATNAGCTCTCCTTAGGNGNGGACYGAAATCGCCACGAYCATGNGCNGAAGGCGCRAATGTGTCCAANGCNTTAGCACNGACYTNGGANGCGCGAATATTACGGCYCACTCCTNNACAGGTCTAGGTAGGNNTTNACACTGGCTGTNYNYNNGGGGGACCGCCNTCGCGCAAATTCACCGGAGGGGAGNCAACACTTTGTTGAAGCGGACTCNCRNGGTTACTAAATTGTGGCGTCTYYCTAAYCGAARGAGARTTCTGTYRYCTCRGRGGNNGCCGGCATGGTGNNRCTTCACTCNNGATANGTNYYGCAAYRYAACGANNCCAGGCGCCGYACRACANANARATGACGARACANGATACGCNGTACGGGACGACGTAGCYACCNNGGACCYTGAYNGTGTTTCGTANYRACACAACATCAGTCTCTGANTTNNNNNGGRNGCAGACNYCTNAGTCAGCTGGGATTNYYGGCCGAATAAGCCGNGNGTNGGACAAACCCGARYCTAGCGGGTCGCGCACTCCYCGNAGGGCACCACACATCTATATTTCNNNNAGGGCGGNRGYGCCAAANCACTYNRTCCCYACCTGNGGTCRGCNYNRTGACCNAAGGTAAGCNNNNTGNRCGNNNNNGNNNCCGRATCCCAACATCCAGTNCYATTCTCTAAACCGACCGTAAAGNACRTGAAGANTNYGTTARCNTGACCYGCTAGRCCRCNTGTYGGTNNACANCNACGTCRNNNNAATGGCACTYYYRNCGNTTGCCNCCAAGCGGCAGGAGTTAGTTNGCNCACCCTNRRNNTGATACAATCTACAGYAAGYYNNTGGARRRCACTCGCCAGNATTTGCACCCAYATGCGTCACTGATACTYCYRTGNCTAACTGGCRGAACGGAGGTTCTNGATCTRTGATGAATAGCCGTCRCAGANRYTRCYGGCTRTYRYGTRGCGCGGGTTGTGCCCNGATTGTYCTCCAAGTGGTAGATARACCCTGAYTTRGGGCGTCRAGANAYAANCGCANNCGCYYCAGGTCCTNRGAACGTGGGNTCCTGATCGCTNRAYCCCACCTCTCAAAGCTACCGCNRCGTCTTCTTAANGACTGGCNAGCTNGAACCCTAGATAANGGGGGTGTTAAGTATCTCTRGNAGGGCCTCCTTGGATTGTNCACRTGATCCTTAGACCRCTTNGTGGCATRGRGGCAGARCCCCNNGGCTGAGCCCNNGGNCGYCCGCAAACTGGCGAACTGGGCCAGAGGCNGNNAGGGGCGCAACRYARCATGRCTTYACNRAGNAYACCGAGCTNTNYTARCRGGAGCCGGTGTGNNNNNNCNTNCCATTCGCTTGNNGCTTRTCTGTCGGTAAAGYCAAAACGCCAACTRGNGANYRNNNGGTACTTTGTATAAATGCCYNNNNCGNGAGCATNNNNTCGCNNGGNCCCGAGNTNNGCCATTARYYAGGCATTGYCTTTGCACGGCAACGGCCCGGTRTCACNNCCTCGGGNCGNTATGTGGCANACTTTTTAGATCCGACCCGATTGYCCGRNANNGAGGYCCCAGCATYCYYNCRCTGGNATTTATCNTRAARRCGATCGGGCAAAGYRYNNCCTGCTGGCGGAGTCCGTTAGTGACCGRNTCAAGTNACCAGATTTNRNNNNTAGCATCCTACAGTGGCGAGTTATCGCTTNCAGTCCGACGCGRYCTCGCAGTGCACATT

Gv10-A-08 ACGATNNNCTCGNCRCCATNNNGGNCGCCGGAGYCGTCGACCTAGACGGGTCTANGRGGAGTACCCACTGACCGTGATCTACTGGCCANCACGCCTYTTGTCTGAAGAGCCCACTGCTCGAGATCCGTCYTGGGTATGGGCCRTNCTGCGCTTACAATGGCNTGGCAGCGTGCARGANGACNANNTNYANNGRNAGGNTTGGANNTGGCTTTGGRNCGGTAGANNNCAYAATACGGCNGTYNGTAAGTAGGTTGGGYANNNAGATNNTAGGTCTCATGAGGARGYTNTTGANRAANNCGGATCACTCGYNGGTCNNNNNTTATTCCNAATGCCCGTGGATNNNNYNNYTGATGGTGTAGCTTNAAGAACGGNGGAGCCANNCTGGAGYTGTNNGGGCGGGGCGGGCGCCGCAGGCCAGGARCCTAGGTACATTACANTGCCGCCAGATRRYCYNNNNNAGCCCTCYCNTNNNNNCTGAATGGGCTGCNTNTGNNTACCTTYNGCTGNCAAYGAAYCCTATCAGACACAGCTAATCANCCTCGTCTAGCACCGTGTGAGAGTATAGAGCYGACGCRTACGRTNTCAACACCRACGAGATNAGGAANGARCGCYTCCNNCTACNAACGGTNNNCATTCCCCTNRAAACAAGCGGTNNNTAAGGNTGAGCAGGCCAATNTCCTCRANCTNACCCTTTRNGTGNANRCCCCTGCNGCCGCTTATGNCAGTANNNGGTGGACTNCGCCRAGCGGTGGGTANCAGGNNRNTCCTANTGCNNGCGAATGNNNGGCNNTNCCNNGRATTGNNCGGCTRNNNGCTAGNAACNGATATTATGTGAGTACATGCCCCGCTCGCAGTACAGATACAGCTAGCCCTGTACACAACGGAATTGTTCTTAGGCTNGCGAATNNGNNTNAGNTCGNANNAACGGGCGYCNTRAGCCGGTCAGCCACTATTNNNNCATACTGTNNNAACCCGCGATAGCCATGGCATNTAAATTNGGAATAGCCCGCGGTGTAAAGGNGGGCCNNGTCGGCGAGAGCCGCCTTGCCGCAAGGGATAATCANNTGATTAGATTNTGTCTNAAAGNNCGCACCCCTCTTAACGNAGGYNATNNNNNNTGGCYCCACCTTCGGNRGCCRRGGGATTAGGCNCGTCCGGGGGNCGATGAGTACTAGGGCTTTANAACGTGAATGCNCRGACGATGGGGCCAGCCACCNCRAYAAGTTCTCGCAGGGGGCGCCAGTNGGCTCGCTGCNNGGTNNNNNTATGGCCCATCGNNAACGCGACGGTATTTGAAAAGACATANGNRYGTGATCGGACTCCTGAAATGTYRNTTAGGAATNNNCTAGCACTGNNNCGCAAACATCAGGCGCATCGGTANCTCTCCGGGCGGCCAGCGCGAATRAAATNGGCTYGCCCCTGNACRANGGAAGGNNNNAACYNNRRGRCCCTGAGNNCTCGCCGNNNCTAGTGAACATAGNNNNTCGCNTAGTTTGACATTTGGTCTACGNRNGCRGCCRTCCTCTGACNTGCCCAGTGGCTTCCGCAGGANNNNNNACAGCCAGAAAGCGAACCNCCCGTTAYTCCCAAGTGGTTTTTCAAGTACCCTNCCTACATCGCTCCAACGNNNNTGNTCTGCTARTGCAATCGNNNGCNNNATATCTATTANNNAGGTGGGCACTGGTCTCGGRNNCGTAGTNNTCGNNNTAARRYYNNGGAYCNCTATGTGCAGGTNNACCGNATTAGYCYGCTTCATTGAGATGGCATGGTCTGTTTGGATATCGTCCGCYTGGTTANNNGNNCNAANCCCRTCCTCACGAGNCCCGRTGRGTNACGGTGRGNCNNNGGATTCTCGYTGNACAATTTCCAAAGCGTCCAGARRATANNTCGTTCTCTTTYCATTTACGCYANCCAATTTCGTAATNTGCNGGCAGCGYAGAGCTGGCACNGCAATGGATGTCTTGCAGAATGGCCCAAACRNCRNCGGCGCNNCACTATCGAGYCCCNNNCACGGCGNCGACAGCTAGCNNNCNCATCTAATRRCTCGCGTNNNNNCGAGGCAGGCGGGATGAATACGGAGACGTTNNGACGGGGTTTAYNNNCRTTCCACRCGGNYCAGTGATTGTGAAAGGRCGGTGCRGRCCCCCARACCGGYATGGGGNGTANNNNNGGTACTCGCRGTCACCTNNGGANNNNTCGRCYGCTAANCGGCGCATCGTNGCNNGGGCGCNNYGCTYTTGTATTAACATTGGCTACGTNGATGCATGCGGAACTTTGCGGCNGGNRAGAGCAAGTCCGTGGCAACGGGCTCTGCGACCACATTAGTNACTTAATGCNNNAAAGATTGNYYGTNCGTGTCCCTCCYCNCGGCGCGAAGCGCNNAAAAGGCNCGCTACGCATAANCCTGAAGAGTAANGCCYAATCTCCGGCGNRYNYAGCGCGGTCGCRNNNNNNGGCTGTAGGCGATTTGAGGGTTAGTGCANNNTNNAGARACRGCCGGTTTAAGGAANNCGCTGGTAGGTTACGNCTTAANTAGCCCACCCCGGAGCAATCNTNCTNGTCGTTAGGGGCTACTCCGNTGGCGTACANCGCATTAGACCYATGCAGCRGCAGCAGCGTCNTACAANACTNNAGACGYCYACATCGTCTCGGGCTAATACTTCTCCTCACNCCTGATCACCRGTGRNCACTAGGYGCCGTATCCTGGATAACCGAAACGGGCYNNCANARGTCGCCATCTTACAAGACYCNNGATANNNTGCGNNNTTNTATCCATGTCAACNCTGAGCGTCGGTGNNNAYGYRCGACCGCCGGTTTATGAACCGCACCACCGGGYYTGCNNGGGAGGANACGCGGCNCNGTAACGCAGGTGTCGACCTGCATCAGCCGAGAGAANNNNNCCGCGATCACGNGACCNYNTACCCCGCAYGGACNCYGRCCYNAAGCGNAGGTGTCYACAYNNATCCCRGRYCTGARARTCGGGCAANGCCTTCTAGCCGAAATACCCCCCGATCCGTGCATAAAGTTACCANGCTNNCNRTNATGGGACYYACNNNNNTTGCNNGRATTANAGCAACNNAATTGCTCCGTCGNNGCGGNATTNNNGCCTYACRTCCNNTRNGGGAGNCCGGGTGAAATANTNAGNYGCCGTCGGACCRTNYGNNNYRCRTCACGTGYGTAATANTCAYTANNNNNGTGAGNNNNGTTCGGGCACCAGYGTCTTNRCCTYGARTTTTCRRRYTAGCCATNCAGYTCNAYANNNATNGGNAGCTNNNAAGCGNTAGYCYNNNCCTCTTGATGYNAYTCCAGNNGACGCGAAAGCCNNAAGGCGNAATCNTTCNNGTGTTGGTNGNACGANRNNAGTAATATANTCCCTNTTTCGCNGGANTRGTTCGTGCCGRAGGAGTTATCACCAATGAAAGTRNCCGCGAGAYRCGCTAYNNGCCCTTTGTYAGTCTACAYTGCAYAATAAACTCGTYGCTGCCTCRTCCCGACCGTACGCAGNNGATCTTAGAACNNAANTCNCCGNCTACCCCGACGCCAGCCTCGCTCAGCCAAAGCTACTGTATCTAANNNNNCTGTCCTCNNTGCTACGARCGGRYCYNTCTTAACNTCRYCGCTATAACACCCAGGCARTTTGAGAAANNATACACGCGGTAAACCGTAGGGCTAGGNNNNAYRGCCTNAGAGGCCANTCTATTGNNGATGAGTCGCNCNNNNNNGNTCTAANTCGGGTCCATCGTGAACGGACCGNNTCGCAGGCGGCNTGCCTGNNNNGAACCGCGNCGGGYNCCTGCGACCTACGCGTCCGCGCCCGNGNGCCGTGGCCRTNNCGYNNNNCTGATCCNGGGACTNGGATGCCCTTGTANNNNCCNTGGAATRNATTGTAYCACGCCACACGACAACATAGNACGCCNGCRGNNCCTTNNYTTAGAGGTCAAGTTTNCTCGTGTTGCCCCCRGGNNNTNNNGCGCCANRGGATGGGGTNATGGCAGNNCGAANCAGNTGCGGCCTTGGNTCCCNAAAACTGTNNNCATNNATGCGTACGTCGAATGCNCCGTGGCGTTGCTTGCGGGTACNGGCGANCGTCNTAGTGTTCATNGTGTTATGAGCTCTCCTTAGGYGYGGACNRAAATCGCCANNATCATGTGCAGAAGGNNNGAATGTGNNNNAAGCGATAGAACGGANNTNGRAAGCGCGAATATTACGGYTCACTYCTATACARNTYTAGGNACGACCTCGTACTAGCTGTAYNYYYGRGNNACYNCCGTYGCGCAAATTCACGAGGGGGGAGCCAACACTCTGTTGTCGCGGGTTCNCGCNGTTACTAAATTGTGGCGTYTTCCTAATCNAANGAGAGTTNYRCTACCACGGAGGTNGCTGGCATGGTGTCGCTNCACTCTTCANAYGTNTYGCRTNRTANCGATACCAGGCGYCNNAYRACANAAAGATGANGANACAANNNNNNNANTACNNNNCGANNNNGCCACCACGGACCCTGATCGTGTTTTGTAACAACACAACNTCAGTCTCTNNNTTNACCCGGGTGCNGACTCCTCAGTCAGCTGGGATTACTAGCCGAATAAGCCNAGTGTCNGGCCAACCCGARYCTNNNNGGTCGCGCACAANNCGGAGGGCACCACACATCNNNNYTTNACTCNNGGCGGARGCGCCNAAACACTTTATCCCCNCCTRNGGTCAGCCNNATGACCTAAGGTAAGCCCTATGNRCNATTGGGAGGCTGAATTTGGACATCCAGTCCTATTCNNTAAACCGACCATAAAGTACRTGAAGAATTTGTTAACNNNACCCGCTAGACCACGTGTCGGTNNNCNGCTRCNYCGGTNNAATGGCRYTTCCAAGGTTTNCNTCCNAGCGNCAGGAGTTATTTGGCCCACCCNCRRTATGACATAACATNCAGYRAGCCGATRRAAGGYAYTCGCCAGNATTTGCACCCANATGCGTCACTGATTTTCCTAAGTCTAACNGGCGGAACGGAGGTTCTGGATCTATGATGNNTAGCCGTCGCAGATGTAGCYGGYTGTCACATGNCRYGGACTGTGCCCANNTTGTNCTCCAAGTTGCAAATAGACCCTGACTTRGGGCGTCRANNNANAANCGNAANCGCYYCAGGTCCTNRGAACGTNNNNTNCTGNTCGCTNGACCCCACCTGNNNAATCTTCCGCTANNNNTNCTTAAAGACTGGCTNNCNTGAACCCTAGATAAYGRNNNTGTTNAGNRNCTCTGTCAGGGCCTCCTTACATTGTNCGCNTGATCCNNARACCNCTTTGTGGCANGNNGGCAGGGCCCCAGGGCTGAGCCCNNGGNCGYCCGCAAACNGGCNARYTGGGNCAGAGGCTGACAGGGGCGCAACNNARCATGNCTTTACTAAGAACACYNNGCTATTNTAGCAGGAGCCGGTGTGCATRCCCTTACCACGCGCCTGAGGCTTRTCTGTNNNNAARGNCAAAACGYCAACTAGAGANYGNNNGGTNCTTTGTRTAAATGCCCTTNCNGGGNGCATNGGATCGYTCGGNCCCGAGATCAGCCATTAGTNNGGCATTGYCTTTGNNCGGCAACNNNNNRRTGTCACGACCTCGGGTCGGTNYGTGGCATACTTTTAAGATNNNNNYCGRTTACCCRNNATGGARNNNCCRRCATCCTTACRCTGGGATTTANNGTNAAGACGATCGGGCAAAGCRNNNCCTGCTRGYGGAGTCCGTTAGTGACCGRATCAAGGTACCAGANNTNNNACCTAGCATCNTACARNGGCGRRTTNTNNCATTCAGTCCGNNNNNNNCTCGYANTGNNCNTT

Gv10-A-10 RCGRYTNNCYCGTCRCCATNNNGNCCGCCGNAGYYGTCGACCTAGACRGNTCTAGRAGGNNTACCCACTGACYGTGATCTACTGNNNNTCACGCYYTTTGTCTGARGAGCCCACTNNYCGRGRTCCGTCTTGGATAYGGGCYRTNCTGCRNYTNCAATGGCCTGGCAGCGNRCARNNGAGCCGAANGTACAGACAGGTNNGNACCTGGCTTTGGAACGGTAGAYYNCNCAATACGGCAGTCTGTAAGTACGTTTGRCACGCAGNNCNNNNNTCTCATGAGGANNNNNNNRACRAAYRCGGATCACCCANNGGNCATTGACNATTCCCAANNNCCGTGGATAGTTYGGTTGATGATGTAGATTGANNAACGGTGGAGCCANTCYGGAGNTGTTAGGNYGGGGCGGGCGCCGCANGYCAGGAACCTAGGTACAYTACANTGCCNCNAGNTAGCCTYARAGNNCCCTCTCTTGCCTGCTGAATGGGNNGCRTTNGNNTACCTTTNNNTNTCAACGAATCCTATCAGANACAGCTANGTATCCTCGTCTAAACTCGTTTAAGAGTCNNGRGCCGACNCRTACGNTNTCANCACNGACGRGRYGAGGNNNNAACNNNTCCGGCTACNAACGGTGGACATTCCCCNTAAAACAAGNGGTNRCNGAGGNTGAGCAGNCCNNNYTCCTCGATCYNNYCCYTTRNGTGNAAGCCCCTGCCCCGGCTTAYNNCAGTNAYTAGCRNNCTGCGCCGNGCGGTNNNNAYCAGGTGAATNNNATYNNNNGCGAATGGTNNNCACTNNNNNNNNCTAGCCGGCTRNTAGCTANGAACTGATNTTATGTGAGTRNATGCCCCGCNYGCANTANRGATACAGCTAGCCCTGTACACAACGGAATTGTTCTTAGGCTYGCGANNNNNNNNNAGTTCGNARAAACGGNCGYCTTNRNCCGGTCANNNNNTANNGTTGCANANTGTAGTAACCCGCNGTAGCCATGGCNYNTANATTAGGAATAGCCCGCGGTGTAGGGGCGRNCCGCGTCGGCGAGNGCCGNYTTNCYGCRAGGGATAATCAGGYGATTAGATTATGTCNGAAGGAACGCACYCCTCTTAANRNAGGYNANAYGCTNTGGCNCCACTTTYNNCNGCCGGRRRRTTAGGCACGTCCGGGGGTCGATNNNNACNAGGGNNTAGGAACGTGAATGYCYAGANGATGGGNCCAGCCACACCGACANGNTCCNACANGGGGCATCAGTNGGCTTGCTGNRRGGTATNTCTATGNCCYGANRGTAACGCGACGGTNYTTGAAAAGACATANGYANGTGNNCGGACTCCTGAAATGTCGATTAGGAATCGGCTAGCACTGACGYRNNNGTATCAGGCGCATCGGTATCTCTCCGGGCGNCCAGCGCGAATRAAATGGGCTTGCCCCTGGACAAANNNAGGGAAAAACCTCRRGACCCTGANGNCTCNNCGACCCTAGTGAACAYAGAGCRNNNCCCAGTTTRACATATGGTCTNYGNGAGCAACCGTCCTCTGACYTACCCAGTGGCTTCCGCNGGACTTTGTACAGCCAGAAAGCGRRCCTYCNGYNNCTCCCAAGTGGTTTTTNAAGTACCCTATCCACGTTGCTTCAACGNAAATGNTNYGCCTNTGCNATCGNNNGCCCTATATCTATTAACCAGGTGGGCACTRNTYTCGGRNNCGTARTACTCGAYATAAGGTCGGGGRYCACTATGTGCAGGTGNACCGNATTAGYCTRCTTCATTGAGATGGCATGGTCTGNNTGGATATCGTCCGCTTGGTTATTNGCTCGARGCCNRTCCTCANGAGTCCCGANGAGTRACGGTGRGNCCGNGGATTCTCNCTGGRNANYTTCCAAAGCGTCCAGAGGATANNTCGTTCTNNTNTCATTTACGCTACCCAATTTCGTAATGTGCYGGCAGCGTAGAGCTGGCACCGCAATGGNTGTCTTGCAGNATGGCNCAANCRTCGGCGGCGCGTCACTATCGAGYCCCTGGCACGGCGNCGACAGCTAGCNNNCCCACCCAATRRCTCGCGTCCCCACGAGGCAGGCGGRRYRAATACGGAGACGTYATGACGGGGTTTAYNCANNNNNCACGCGGTTCTGCGATTRNNNRAGGGCGGTGCAGACYCCNAGACCGGTANNNNGYGTAATTGGGGTNNNNGCGGTCNCCTNNNNAATANTCGACCNCTAATCGGCGCATCGTRGNGNGGGCGCCTCGCTTNNRTATTAACATTGGCTACGTAGATGCATGYGGAACTNTNCGGCGGGAAAGAGYGRGNCCGTNGNNNCGNGCTCNGCGANNACATTAGNNNCTTAATGYGTCAAAGRTTGATCGNNNNNGTCCNYCCNCNCGGCGNRAAGAAACTAAAARGCNCGCTRCGCANAAGCCTGNNNAGTAANNNNNRNYNTCCGGCGNRYNNGGAGANNTCGCNNYAGCNGGTTGTAGGCGATTTGNGGRYTARYRCAACNNCTAGAARCGRYCRRTTYARGGRRCYTGCTGGTAGGTTACGRCTTAAYTAGCCCACCCCGGAGNAATCGTCCTCGTCGTTAGGGGCTACTCYRNTGGCGTACANCGCATTANNNNCAYGCAGCAGCRGCAGCGTCYTACAANACNNNAGACGCCCACATCGTNNNNNNCTAATACTTCTCCTCACTCCTGATCANCNGNGGCCACTAGGYGCCNNNTCCTNGATACTCGAAAYGGGCYTYCANGGGTCNNCRTCTTACAAGACTCCTGATANNNTGCGNNGYTTTNTCCRYGNCANYNCTGAGCGTCGGTGACGAYGCACGACCGNCGGTTTATGAACCGCACCACCGGGTCNGCAGGGNRGRRGGCACGGCRCYNTANCGCNNGTGTCGACCTGTATYAGCCGAGNNAATGGAACYGCRRNCACNTRACCNYNTACCCNNNNNGGACGCYNGCCYNAAGCGRAGGTGTCYACATNGATCCCRGRYCTGAGAATCGNNCNACGCCTTCTAGCNGAAATACCYYCNNNYCYGYGYNTAAAGNTANNACGCTTACAGTAATGGGACYYACCRGNTTTGCNNGNNTTAAAGCAACTCNRTTGCTCCGTYRCANCNNNNNNAAAGCCTYACRTCCNNNNNGGGAGNCGGNGTNAAATATNAAGNYGCCGTCGGACCGTCCNNCCYRCGTCACGTGTGTAATAGTNAYTAGGTNTGTGAGAGGAGTTCGGGCACCANYGTCNNGACCTCGAGTTTNNNNNNNAGCCATNCAGCTCNACAGACRNNGGCNRCTCACAAGCGATAGCCNATCCCNCTARRNGYNNNNCCAGGNGNCGYRAAARNCNNAAGGYRNAATCATTCTCGTGTTGGTANNACGAGGAGAGTAATATAATCCCTNTNTCGCAGGAATGGTTCGTGCCGNAGGAGTTATCACCAANAAAAGNGTCCGCGAGAYRCGCTANNNGYCCNTTGTCAGTCTACRYTGCNTAATANANTNGTCGCTGCCTCRTCCCGGCCGTACGCAGTANNCYCCNNNNNYTAAATCGCCGGYTACCCCGACGCCAGCCTCNCTCAGCCTGAGCTATTNNNNCTANNNGNANTNTTYYCNNTGNNNNTGNCGGGCCCCTCTNNNYGTCGTCGCNANRAYNCACAGGNNNTTTGAGAAATCTTNCANNCGGYAANCCGTANNGCTAGGCTANACAGCCTNAGRGGCCANNCCATTGTGGATGAGTCGCTCAGTCTCRNYCTRRATCGGGTCCAYNNNNGACGGACCGGTTCGCAGGNGGCNNNNCNGATCGGAACCGNGNCGGGYNNCTGCNACYTACGCGTCCGCGCCCGGNCGCCGTGGCCRTTCCGYNTAACTGATCCNGGGACTNRGATNYCCTNNNNAGCACACTGGAATAGATTGTNNNNCGCCACANGRCAACNTAGNACGCCAGCGGTACCTTRYNNTAGAGGTCAAGTTTNCYCGTGTTGCCCCCGGGCAATCATGCGYNACNNNNNGGGGTCATGGNAGCGCGAATCAGGTGCGGCCTTGGNTCCCAAAAACTGYAGTCANNNAYGNNTACGTYGAANGYANCGCGGYRTTGCTTGCGGRTNNNNGCGRCCGTNATNGYGTYCNNNNNGTTATGARNNCTNNNTANNNNNGGACCGAAANCGCCACGNNYATGTGCAGAAGGCGCGARTGTGAGCAAAGCGNTAGCACGGANYTYGRANGCGCNNNNNNTACGGCTCACTCCTNNANARNTCTAGGTACGRCYTCNNACTRGCTGTACCCTCGGGGGNCNNNCGTTGCNNNNNTTCACGAGGGGNGAGYCAACACTYTGTTGTCGCGGGTTCNNNCGGTTACNNAATTGTGGCGTCTTCNNAATCGAAGGAGAGTTATGCTACCACGGAGGTCGCNRGCATGRTGTCGCTTCACTNNNNATANGTAYTGCAATRTAACGATACCAGGCGTCGTATAACAGAAAGATGANGAAACANGATACGCAGTACGGGACGACGTNGCNACYNCGGRCCYTGATCGTGTTTYGNNACAACACRRCATCAGTCTCTGACTTTACCCGGRNNNAGACTCCTCANTCAGCNGGGATTANTAGCCGAATAAGCCNAGTGTCNGRCNNACCCGARYCTNNNNAGTCGCGCACTCCYCGRAGRGCRYCACACATCNRYNNTTNACNNGGGGCGGNRGYNCCAAAACACTCTATCCCCNCCTRTGGTCGGCCCNNNGAYCTAAGGTAAGCNNNAYRNGCGATTGGGNNNCTGRATCCCAAYRTCCNGTCCTATTCTCTAAACCGACCRTAAAGTACNTGGGGANNNYGTTAACGTGACCTGCTAGGCCACGTGTNGGTNCACANNNACGTCAGTCTAATGNNGTTNNNRAGGTTTACCTCCNAGCGNNRGGAGTTAGTTGGCCCACCCYCRRTCTGAYANAAYNNNNNGYRAGCCGATRRAARRYAYTCGCCAGCATTTGYAYCCACATGCGTNACTGANNNTYCYRTGNNNNACNGGCGGAAYRGAGGTTCTNGATCTRTGATGAANAGCCGTCGCNGANGTNRCYGGNTRTYRTRTANNNNNGACNGTGCCCAGATTRYYCTCNAAGTTGCAARTAGACCCNGNNNTAGGGCGTCAANAYAYAANCGYAATCGNCTCAGGTCCTNRNAACGTGGGATCNNGATCGCTCRAYCCCACCTCTCAAAGCTACCGCTANNNCTTCTTAANGACTGGCTNNCNTGAACNCTAGATAANGGRRGYGNTNAGNRNCTCTGNNNGGGCNTCCTTGGATTGTNCNCATGANCCCAAGACYNCTTTGTGGCANGNNGGCAGGGCCYCAGGRCTGAGCCCNNGGCNNNCCGCAAAYNGGCNANNTNGGCNAGAGGCNGANNGGGGCGCAACACAGCATGRCTTTACTAAGNAYACYNAGCTATTTTAGCAGGAGCCGGTNTNCATGCCCNTACCACGCGCYTGAGGCTNNNCTGTCGGTAAANNCANRRNGYCARCNNNNGANYGCNTGGTACTTTGTRTAAATGCCNTTTCCGGNNNCATCNGATCGNTCGGNNNNNNNNTNNGCCATTRRYYAGRCATTGTCTTTGNNCGGYAACNNNNNGRTGTCANNNNYTCGGGNCGNTTCCTGGCANACNNTTNAGATCCGACTCGNNNRYCCAAGATGGAGGYCCCAGCATYCYNACGYTGGGRTTNATCGTRAAGACGATCGGGCAAAGCNYNNCCTGCTGNNGGAGTCCGTTRRTGACCGGATCANNNGACCAGATTTNRNNNNNNGYATNNTACANYGGCGRRTYNTNNNANNCAGNCYNACGCGRYCTYGCANYGNNNNTT

Gv10-B-02 RNGATTTACTCGTCNCCATAATGGCCGCCNNNNYCGTCGACNNAGACGGGTCTARRNGGAGTACCCACTGACYGTGATCTACTGGCCATCACGCTCCTTGTCTANNNNGCCCACTGCNNGGGANNCGTNNTGGRTATGGGCCATGCNGCGCTTANNNTGGCCANNCAGCGTNCAGGANNNCNNNATGTACAGACAGGTTNGNACCTGGCCCTNNNACGGTAGATCTCACAATACGGCAGTYNGTAAGTANNNNNGGTACGCAGNCCNTAGNTCTCATGAGGANNNNNNTGACAAATGCGNNNCACYCRNNNNNCATTGACNATTCCCAATGCCCGTGGATAGTTTGGTTGATGRNNTAGAYTNAAGAACGGTGGAGCCATTCCGNAGTTGCTAGGNNNNGGCGGGCGCCGCNGGCCAGGAACCTNGRTRCNYTACAGTGCCGCCAGNTRRYCYTAGARNNCYCTCTCTNGCCTGYTGARTRGGNNRYGTTAGGTTACCTTCCNNNNNCAATGANYCCTAGCGGANACRRCNAANNATCCTCGTCTANACYCRNGTRNNARTNTAGAGYCGACGCGTACGATTTCANCACCGACGNGNCGAGGNNNNARCGCYTCCRRCTACGAACGGTGGACATNCNCCTNRRAAYAARNGNNNGCGGAGGCTGAGCAGGCCTGTYTCCTCRATCYNCTCCCTTATGTGNANRCCNNCACCCNNGCTTNNGNCAGTRCNTNGNGGACTGCGCCNTGCGGTGGGTACCAGGTGAATNNTANYGCAYGCGAATGGYAGGCACTACCGTAATCTAGCCGGCTNNTARNNNNGNNCTGRTNTTATNNGAGTGAATGCNNNGNTCGCANNANNNNTACANCTAGCCCNNNACACAACGGAATTGTTCTTAGNNNNNCNAATNNGNNTNAGTNNGGAANAACNNNNNNNNTGRNYYGNTCAGTNNNTNNNGTTGTATAYTNNNGTAANNNGCRNTAGCCATGNCTCNTAAATTAGGAATAGCCCGCGGTGTANNNNCGNNNNGCGTNNNNNNNRGCCGNCTTGNCGCNNNNNNNNATCAGATGATTAGATTATGTCTGAAGGAANGCACYCCTCTTAACAAANGNCANACGCTGTGGCTCCANYTTNGGCAGCNNNNNGATTAGGCACGTCCGGGGGYCGNTGAGTACTAGGGCNNNNGAACGTGANNNCNCNGANGATGGGGCCAGCCACNNCRANAANTTCTCGCAGGGGGCGCCAGTTGGCTTGCTGYRRNGNATNTCNNNNGCCCGANNGTAAYRCGACGNNATTTGAAAAGAYNTANGYANGNGNNNNNACTCCTGAAATGTCATNTAGGAANYNACTAGNNNNGACRNAYAANNATCAGGCGCATCGGTANCTCTCCGGGCNNCCAGCGCGAATGAAANGGGCTNGCCCCYGNANNAAGGANNGGAAAAACYYYAAGACYCTGNGNACNCGCCGACCCTARTGAACATAGAGCATNGCNTAGNTTGACATNTGNTCTNNNNRNNNNNNNNNCCTCTGACYTACCCAGTGGCTTCCGCNNNNCTTTGTGYGAYCANNNNNNNGGCCNTCCNYNNYTCCCAAGTGGTTTNTCAAGTANCCTNTCCACATYGCTCCGNCNAAAATGATCYGCTAATGCNRTYGTGNGNCCTATATCTRTTAACCAGGTGGGCACTGGTCTCGGGGNNNTAGTACTCGATATAAGGTCGGRNANCACTATGTGCAGGTGGACCGNATTAGCCCGCTTNAYTGAGATGGCATGGTCTGNNTGGATNNCNNCCGCYTGGTTAACNNCTNNANGCCNATCCTNNNNCATCCCGANGAGTAACGNTGAGGNCGGGGATTCTCGYTGGACAATTTCYAAAGCGTCCAGAGGATNNNTCGTTCTCTTTCCATNTACGCTAGCCAATTTNNTAATNTGCCGGCAGCGTANNNCTGGYNNCGCAATNGRTGTCTTGCAGAATGGCNNAARCAGCANCGGCGCGTCACTATCGAGNNNCATTCNCGGCGGCGACAGCTAGCNGACCCANNNNNNNNCTCGCGTCCCCACGNGGCAGGCAGNNCNRRTNCGGAGACGTNATGACGGGGTTTANACNCATTCCACGCGGNYCNGYNNNTGTGAAAGGGCGNTGCAGACYCCYARACCGRYATGGGGNGNNNNNNNGGTNYTCGCGRNYACNNNNNGNGTACTCGACCGNNAANCGGNNNNNCGTNGCNANNNCGCCTNGCTYTTGTAYTAANATTNNNTACGTNGATGCATGNGGAACTTCGNNNNGGGAAAGGNNNNNNNNNTGGYRNCGNGCTCNNNNANNANNTNAGTTNCTTRNTGCGTCAAAGATTNNTCGTNCGTGTCYNYTCYCNNNGCGCGAAGNRANTAAAAAGCYCGNNNNNNANAANCCTGTNGAGTAANGCCTNNTCTCCGGCGCACGCAGCGCGGNNNCRCCAGTCAGNTGCNNGCNATTCGAGGANTAGCRCNACTNCTAGARACRGCCGGTAYARGNAACNYGCTGNTAGGTTACRRNTTNNNTAGCCCGCTCCGGAGCAATCGTCCTCGTCATTAGGGGCTACTCCGNTGGCGTAYNTCGCATTAGACCNNNGCANNNGCAGCAGCGTNNNACAANACTCTNNNNGCCNACAGCATNNCGGGNNAATACTTCTNCTCACNCCNNATCACCGGTGRNCACTNNNNGCCNNNNNNNNNATANYCGAAATGGGCTTTTAANNNTCGCCATCTTACAAGACTCNTRNTNNNRTGCGNTGTTTTATTCATNYCANYNNNNAGCGTNNNTNACGACGTGCGACCGNNNNNNTGTAAGCCACACCANCGGGCTTGCAGNGNRGGAGACGCGGCRCYGTANNGCAGGTGTCGACCTGYATYAGNNNNNAGNRNGGRACTGCAGANNNGYGNCCCCNTACCNCGCACGGNCGCYGGCCYCAAGCRANGGTGTCYACANCGATCCCAGATCTGAGAATCGRRCNACNCCTTCTAGCCNAAATACCYYCCNNTCCGYGCANNANNNTACCACGCNTACCRTNNNGGGACNNACCGGGNTTGCGAGGATTNAANNAACNNCGTNNNNCCGTYRCANCGNNNNNCANGCCTCACGTCCNTTATGGGAGNCGGRGNNAAAYATTCAGGTGCNGYYGGACCRTCYGNNNYRCNTCACGTGTGTAATANTCAYTAGNNNTGTGAGAGGANTTCGNGNNCCAGYGNNNNGACCTCGAGTTTTCNGNNTAGCCATANNGCTCCRYAGRYATANNNAGCTNNCAANCGNTNNCCCATNCCNCTARANGCGACTCCAGGTGNNNCAAAAAGCYCAARGYGTAATCANNCTNGTGTTGGTNGGACGANRAGAGTANTATARTCCCTNTTTCGCAGGAATGGTTCGTGCCGRAGGAGTTATCAYCAANGANAGTGTCCGCGAGATGCGCTANNNNNCCTTTGTTAGTTTACNNNGCANAATAANNNCNNCGCTGCCTCGTCNNGACCGYNNGCAGNNGATCNNNNNNNNNAANNCGCCGGCTACCCNNACGCCAGCCTCGCACAGCCTGNNNNATTGTATCTAAATNNNCTRTTYYCATYGTTGCGGGCGGNNNCCTYTTANTGTCGTCGCTATAACNCACNNNCAGTTTNNNNNNNNNNACRCGCGGTAANNNNNNGGGCTAGGCTACACANNNTGNNNGGCCANCCTATNNNNNNNRAGYCGCGCNGNCTCNNYCTRRATCGGGTYCANNGNGRNNNNACCGGTCCNCAGGNNGCGTGCCTGATCGNNACCGCGTCGGGCCCNNNYGAYYTANGCGYCYGCGCCCGNGCGCCGTGGCCRTTCCGYANAACTGATCCAGGGACTTGGATGCCCTTGTAAGCACNNTGGAATAGNTTGTACCGCGNNNNNNGRCAACNTAGNACGCNAGCGGTANNNTRYYTTAGAGGTCAAGTTTNCTCGTRTNNCCCNCAGGCAATCATGCGCCACAAANNNGGGTCATGGTAGCNCGNNNCAGGTNNNNNNNTGGNTCCCAAAAACTGCNGNCATTTATGCGTACGTTGAAANNAGCGYGGCRTTGCTNGCGGNTNNNNGCGNCNNNCATAGYGTTCATGGTGTTATGNNCTCTCCTTAGGCGCGGACYGAAATCGCCACGAYCATGTGCAGAAGGCGCGARTGTNNNCAANNNNNTAGNACGGANNTTGAAAGYGNNNNTATTACGGNNNNNTCCTATACARNTCTAGGTANGRCNTCANNCTGNCTGTANNNCTGRGNNACCGCCNNNGCGCAGCTTCACNGGNGGGGAGCCAACACTTTGTNGTCGCGGGYTCTNNNGNNNNNNNAATNGTGNNNNCTTYCTANYCGAARGNNNNTTCNNNNACCACGGAGGTNNCCGGCATGNNGTCGCTTCNNTCYYGNNATGTACTGCAAYATANCGNNNNCAGGCNNCNNATAACANAAAGATGANGAAACAAGANNCGCNGTACGGGTCGRNNNNNNNANNTCNGRCCCTGATCGTGTTTCGTACTGACACAACNTCAATCTCTGACTTTACCCGGGTGCAGACTCCTNANNNNGCTGGGATTTCCGGCCGNNNAAGCNGAGTGTCRRNNNAACCCGANNCTAGCGGGTCNNNNACNNCCCGAAGRGCRYCACACATCNATNTTTCACTCAGGGCGGNRGCNNNNAANCACTYTATCCCCACCTRTGGTCAGCCNNANRAYCTAAGGTAAGCNNNNNGAGCNATTGGNAGGCYGAATCCCANYRTCCRGTCCTATTCTCNNNACNNNNCGTAAAGNNCGTGAAGAANTYGTTAANNTRACCTGCTAGGCCNCRTGTYGGYCCACAGCTGCCCCAGTCTAGTGRCACTTTTNNNNNNNGCNTYCNAGCGACRGGAGTTAGNTGGNNCRCCCTCRRTATGNYAYAATCTACANNNAGCCRNTGGANRRCACTCGYCAGCATTTGNNNCCACATGAGTCNNNGANACTNNNNNGNCNAACCGGCGGAACGGAGGTTCNNNATCTANGATGAANANNCGTCACAGATGTAGCYGGCTRTYRYGTRCCGCGGNNTGTGCCNNNNNTGTCCTCCAAGTGGTAGATAGACCCTGACTTAGGGCGTCRAGACACAATCGCAATCGCNNCAGGTCCTNRGAACGTGGGATCCTGATCGNTCAACCCCNCCTCTCAAAGCTNNCGCTRCGTNNYCTTAACGACTGGCTNGYTTGANNNYYAGATNNNNNGGGTGTTAAGNNNCTCTNGNAGGGCNNCCTTGGATTGYNNGCNTGATCCTTAGACCNCTTTGTGGCATAGAGGCNGARNNNCTAGGCTGAGCCCNAGGCCGTCNNNAAACTNNNNANNTGNGNCAGAGGCTGAAAGGGGCGCAACGTAACATGACTNNNCTAAGNACACCGAGCTNTYNNNRCRGGAGYCGGTGTGCATGCCCNTANNANNCGCTNNNNGCTNGTCTGTCGGTAAAGYCANRRCGYCNNNNGGTNANYRNNTGGTNCTTTGTATAAATGCCCTNNNCGNNNNCNNCGGATCNNTCGGTCCCGAGNTNNGCNNNTRGTTAGRCATTGCCTTTGCACGGCAAYNNNNNRRTGTCACGAYNTCGGGACNNTNYGTGGCAGANYTTTTAGATNCGACYCGNTTRCCCGAGATGGAGGTCCCAGNNNNNNCNCGCTGGGANTNATCGTAAAGACGATCGGGCAAAGNNTGTCCTGCTNGCGNNGTCCGTTRGTGNCCRGNNCNNGTTGCCAGNNNNNGNANNTAGCATCNTNNANTGGCGRGTNATYRCATTCAGTCCGACGCGRYCTCGCANTGCANATT

Gv10-B-06 RNNNNTTACYCGTCGCCATAATGGCNNNNGGAGTCGTCGACACAGACNNGTCTARGAGGNNTACCCACTGACYGTGATCTACTGGCYATCACGCNYNTTGTCTRAAGAGCCCACTGCTCGGNNTCCRTCYTGGNNATNGGCCATGCAGCGCTTACAATGGCYNGGCAGCGTNCANGAGAGCCGAANGTACAGACANGTTTGGACCTGGCTTTGGRACRGTAGAYYNCAYAATNCGGCNGTCTGTAAATANNNNNGGTACGCAGGYYNTAGGTCTCATGAGGAAGCTTTTGANAAATGCGGATCACTCGCAGGTCATTGAYYATTCCYNATGCCCGTGGATAGTTYNNYTGATGGCTTAGANTNAAGAACGGNGGAGCCANTCTGNAGTTGCTAGGNNNNGGCGGGCGCCGCANGCCAGGAACCNNGGTAYANTACAGTGCCGCCAGCTGATCCTCAAGNNNNNNCTCTYNNCTGCTGAANNNNCTNNATTTGNNTACCTTCCNNTRYCAACGAATCCTATCAGANAYAGCTAANTATCNNNNTCTAAACTCGTNTNAGAGTNTAGRGYNNACGCNTACGRTGTCAACACCGACGAGAYGAGGAAAGAAYGCYTCCRRCTACGAAYGRYNNNCRTTNNCCNNRAAACAANYGRYNRCNRAGGNTGAGCAGGCCNRTCTCYTCGANCNGCTCNNNTATGTGNAAGCCCCYRCCNCNGCTTACGNCAGTRNTTAGCGGANTGCGCCRTGCGGTGGGTACCAGGAGGCTCCTATNNNATGCGAATGGTNGGNNNTACCGTRANCTAGCCGGCTNNTAGCTANNAACTGATATTATGTGAGTRNATGYCCCGYNCGCANTNCRGNTACAACTNGNNNTGTACACAACGGAATTGTTCTTAGNNNNNCGAATACGGATAAGTTCGNAGAAACGGNCNNCNTGGTCCGGTCNGYCACTANTNNNNYATANNGTNNNAACNCGCRRYAGCCATGNCNNNTAAATTNGGAATAGCCCGCGGTGTARRGNCGACCCGCGTCGGCGAGAGCCGNNTNNCNGCRAGGGATAATCAGACGATTAGATTNTGTCTGAAGGAACGCACNCYTCTTAANAAAGGYCNNACGCTNTGGCTCCACTTTCGGCAGCCGGGGGATTAGGCNCGTCCGGGNNYCGNNGAGTACNAGGGCCTAGGAACGTNNATRCNCNRACGATGGGGCCAGYCNNCCCGACAANNTNNNNNNNGGGGCATCAGTYGGCTYGCTGYRRAGAATTTCTATGNCCCGACGGTAACGCGACGGTNYYYGRAAAGACATAGGTANGTGNNCRGACTCCTGAARYGTCGATTAGGRNTCNNNTAGCACTNACNNNNAARYATCAGGCGCNNNNGTATCTCTCCGGGCGGCCAGCGCGAATGAAAYGGGCTTGNCCCTGATCGAAGGNAGGGAAAAACNYYAAGACCCTGAGNNCTCNNCGNCCCTAGTNAACNTAGAGCATCGCCNAGTTTRACATTYGGTTANYGNRNGCARNNNTCCTCTGACCTRCCYAGTGGCTTCCGCNGGACTNTGTNNNNCCAGAAAGCGAACCCCCCGCACCTCCCAAGTGGTTTTTNAAGNNNCCTNCCTACRTYGCTTCANNNGAAATGCTCYRYNNNTGCAATCGNGAGCCCTATATCTATTAACCNGGTGGGCACTNNTNNYGGGGNCGTANTACTCGATATAARRYYNNRNRYCACTATGTGNNNGTNNACCGTATTAGNCNGCTTCATTGNNNNNNNNTGGTCTGTTTGGATATNGTCCGCTNNGTNNNYNGCTNNAAGCCCRTCNTCANGNGTYCCGANGRGTRACGNTGRGNCCGGGGATTCTCGCTGGACANYTTCCAANGCGTCCAGAGGATANNTCGTTCNNNNTCCAANTACGCTAGCCAATTTCGTAATRTGCNGGCAGCGTAGAGCTGGCACCACAATGNNTGTCTTNCRGAATGGCTCAAGCATCGGCGGCGNGTNACTNNNGAGCCCCTGGCANGGCGNYGACAGCTAGCNGACCCACCCAATRNCTCGCGTCCCCANNNNNNAGNNNGGAYGNNTACGGAGACGTYATGACGRRRNNNANNCACRTTYCACGCGGNYCTGCGATNNTGAAANNNNGGTNNGGGCNCCNANACCGGYATGGGGNGTAATTGGGGTRYTCGCAAATACNTGAGRANTACTCGACCGCTARTCGGCGCATNGTNRYNAGGGCGCCTCGCTTTTNTATTNNNNNTNNNNACGTAGANGCATGTGGARCTTYGYRGCANGNAAGAGCGRRNCCRTGGYRNCGNGCTCTGCGNNCANNNNAGTNNYCYNNTGTGTCAAAGATTGATYGTTCGTGTCCNYCCCCGNNGCGNGAAGAAACTAAAAANNCCRCNACGCAAAANNNTGNNRARTAANGCCTNNTCTCYGRCGNNNNYAGCGCGGTCGCRCCAGCCGGTTNNAGNCGATTYGAGGRYYAGYGCANCTNCTAGANACNGCCGGTANAGGGAACYTGCTGGTAGGTTACNNNTTAATTAGCCCRCYCCGGAGCAATCGTCCTCGTCGTTANNNNCTACTCYRNTGGCGTACANCGCANTANACCCACGCAGCAGCRGCAGCGTCTTACAANACTCTAGACGTCCACANCRTNNCGGNCTANTNCTTCTCCNCACTCCTGATCANCRGTGAGCANNAGGNGCCNNNTCCTCGATNNNCGAAANGGGCYTCYAAAGNNNGCCATCTTACNNNACTCCTGATNNNNNNNNRTGTTTTATYCATNNCACTNCTGAGCGTCGGTGACGAYGYRCGACCNTCGGTTTATGAACCGCACYACYGGGYYTGCAGGGNRGNNGGCACGGCGCCNTAACGCAGGTGTCGAYCYGCNTYAGCNNGGAGAATGGGACYGCRGANNNGTNACCCCTTACCCCGCAYGGACGCYRGCCYCNAGCGRRGGTGTCNACACCGATCCCRGRYCTGAGAATCGRRCAACGCCTTCTAGCCGAAATNCCCCCCNNYCNGNRYNTAAAGTNNCCACGCTNNCAGTAATGGGACCCACNNNNNTTGCNNGGATTANAGCAACTCCGTTGCTCCGTYRCANCGGTATTAAAGCCTYACRTNNNTTGGGGGAGNCGGRGTRANRCATTCRRGYRCTGTCGGACCGTNCNNCNYGCNTCACGAGTGNAATAGTNACNAGGTNTGTGAGNNRAGTTCGGGCACCAGCGTCTTNACCTNGANTTTTCGGRYTAGCCATNCAGCTCCNTAGACNAAGGYRNCTNTNAAGCGRTAGYCYATCCCNCTARANNCGACTCCAGGGGNCGYGGAAGCCNNAAGGYRYAATCATTCTAGTGTTRGTCGGNNNAGAAGGGTAATATAATCCCTATTTCNCAGGNNTGGTYCGTGCCGRAGGAGTTATCANCAANGANAGTNNCCGCGAGAYRCGCTACNTGCCCTTNNNTAGTYTACNNNGCATAATANANTCGTYGCTGNCTCATCNYGRCCGTNCGCAGTAGACCTTAGARNYTAANNCNCCGRCTACCCCGACGCCAGCCTCGCACAGCCTGAGCTATNGTATCTAAATGGACTATTTCCATTGTAGNNNGYGRRYCCCNNNTANCNNYNNCGCNANRNNNCACAGGCNNTTNNNNNNNNNNNNCACGNNNNAANCCGTAGGGCTAGGCTNCACANNNTGAGANNNNANNCTATTGCAGATRAGYCGCGNAGTCTCRGCCTGGATCGGGTCCATCNNGNACGGACCGGTCCNCAGGAGGCGTGCCTGATCNNNACCGCGTCGGGNNCCTGNGACCTACNCGYCYGCGCCCGGNCGCCGTGGCCRTTCCGNNTNANTGATCCNGGGACTNGGATGCCCTTGTAAGCAYCCTGGAATNNATTGTACYACGCCACACGRCGGCATAGGACGCCAGCGGTANCTTNNTTTAGANNNCAAGTTTTNNCGTGTTGCCCCCAGGNNNTCATGCNNNNCRRRATGGGGTCATGGYNNCNCGNNNCAGGTGCGGYCNYNGNTCCCAAAAAYTGCAGTCATTTATGCGTACGTCGAATNNNNNGCGGYRTTGCTTGCGGRTACTTGCGGCNNNNATNGYGTTCATGGTGTTATRAGCTCTCCTTAGGCGCGGNCCNAAATCGCCANNATCATGTGCAGAAGGCGCGAATGTGNNNNAAGCGTTAGCNNGGANNTTGAAAGCGCGAATATTACGGCYCACTCCTATACAGGTTTAGGTACGACNTCRTACTNNCTGTAYNYNNGNGNNNCYGCCGTTGCNNNNNNNNANNNGNGNGGAGCNNNNNNNYTGTTGTCGCGGGTTCTCRCGGTTACTAAATTGTGGCGTCTNCCTAAYCGAAGGAGAGTTNYRYTNNCNCGGAGGTNGCCGGCATGATGAGACTTCACNCNNGATNNNNNTNGCAAYRYAACGATACCAGGCGCCGTANRACANANARATGANGAAACACNNNNCGCAGTACGGGACGACGTAGCNNCTNCGGACCCTGATCGTGTTTTGTANNNACACAACATCARTCTCTGACTTTNNNNGGRNNNAGRCTCCTCANNNNNNTGGGATTANNNGCCGAATAAGCCGAGTGTNNGGCCAACCCGAGTCTAGCGGGTCGCGCACNNCYYNNNRGGCACCACAYATCTATATTTCNCNNGGGGCGGNNNNGCCAAAACACTCNRTCCCCACCTGANNNCAGCCNTGNGAYCTAAGGTAARCNNNATGNNCGATTGGGAGGCYGAATNNCANTGTCCGGNNTTATTCTCTAAACNNNCNRTAAAGTACRTGGGGAATTYGTTARCTTGACCCGCTAGACCRCNTGTYGGTNNACARCYACGTCRNNCTARTGGCRYTTNNAANGTTTGCCTCCNAGCGNNRGGAGTTAGTTGGCCCACCNTCRRTATGACATAAYNTCCANNNAGYYGATGGAAAACAYTCGCYAGNATTTGCAYCCAYATGCGTCACTGAYACTCCTGTGNCNNNCTGGCGGAACGGAGGTTCTNGATCTNTGATGRNNARNCGTCNCAGATACTGCYGGCTGTCAYATNNCNYGGGTTGTGCYYAGATTGTCCTCYAAGTTGCAANNNGACCCTGACYTAGGGCGTCNNGAYANAANCGYNANCGCYTCAGGTCCTNRRAACGTGGNNNNCTGANCGCTCGACCCCACCTCNYAAAGCTAYCGCNNCGTNNNNTTAACGACTGGCTAGCTYGAACCCTAGANAACGGGGGTGTTANNGGACTCTNGNAGGGCCTCCYYGGATTGTNNRCNNNNTCNNNAGACCANYTNGTGGCATRGRGGCAGARCCCNNRNGCTGAGCYCTAGGCCGTCCGCAAACNGGCNNACTGGGCCAGAGGCTGANAGGGGCGCANCRYARCATGRCTTNNCTAAGTAYACCNNNNTATTTTAGCAGGAGCCGGTGTGCATGCCCTTACCACGCGCYTGAGGCTTGNCTGTNNNNAAAGNCAAAACGCNAACTRRAGAGTGCCTGGNACTTTGTANNAATGCCNNNNNCGNGAGCATYNGATCGCTCGGNYCCGAGNTNNGCNNNTARYYAGGCATTGYCTTTGCACGGCAACGGCNNGGTGTCACRNCCTNGGNNNNNNATGTGGCANACTTTTTAGATYCGACNCGNTTACCCRAGATGGAGGNNCCAGCATYCYNNCGCTGGGNNNTATCNNRAAAGCGATCGGGCAAAGNNYNNCCTNNNNGCRRNRTCCGTTRGTGACCGRATCAAGTGACCAGANYNGRGACCTAGCATCYTACAGTGGCGAGTTATCGCATTCAGTCNNACGCGGTCTCGCAGTGCACATT

Gv10-B-10 RCGATTTACNCGTCGCCATAATGNNCGCCGGAGTCGTCGACCTAGACNNGTCTARGAGGTTTACCCACTGACTGTGATCTACTGGCCATCACRCYNCTTGTCTGARGAGCCCACTGCTCGRGATCCGTCTTGGNNATGGGCYRNGCTGCGCTTACAATGGCYNGGCAGCGTRCAGGACGACTATATGTACAGACANGTTTGGACCTGGCTTTGGRACGRTAGAYYTTRYAATAYGGCAGTYTGTAAGTAGGTTGGGTACGCNGNNCNTAGRTCTCATGAGGARGYTYTTGANRAACRCGGATCACTCGTTGGGCATTGATYATTCCCAATGYCCGTGGATAGTTYNNYTGATGRYNTAGAYTNANNAACGGTGGAGCCRTTCYGGAGCTGYTAGRGYGGGGCGGGCGCCGCTGGCCAGGANCCTAGGNAYAYTACAGTGCCGCCAGNTGNNNNTNRARCRCYCTCYCTYNNCTGTTGAGTAGGCTGCRTTNGGTTACCTTTGGCTGYCAACGAATCCTANCRGANACAGCTAATCATCNNNNTCNARACTCGTTTAAGAGNNTAGGGCYGACGCRTACGRTNTCAACACCRACGNGNCGAGGAANGAACGCNTCCNNCNRCGAACGGTGGACRTTNNCCYTAARACANGYGRYNNCNRAGGNTGAGCAGNCCNRTCTCCTCRATCCGCTCCCTTATGTGNAAGCCCCNNCCNCCGCTTNYNNCAGTGATTAGCRNACTNCGCCRTGYGGTGGGTACCNGGTGAATCCTAGCNCATGCGAATGNCNGGCNNTACCGTNATNNNGCYGGCTGGTAGCTAGNAACTGATATTATGTGAGYRCRTGCCCCGCTYGCACTNCRGNTACANCNNGCCCTGTNCACAACGGAATTGTTCTNAGGCTYGCGAATACGGATAAGTTCGGARAAACGGCCGNCTTGGTCCGGTNNGTCACTAANGTTGYANAYTGTNGTAACYCGCGGCAGCCATGGCTCNNAAATTNGGAATAGCCCGCGGTGTAGGNNYGRNCCGCGTCGGCGAGRRCYRCTTNGCCGCRAGGGNTANTCANRYRATTNGATTATGTCNGAAGGAACGCACTCCTCTTAACRNAGGTAAYAYGCTGTGGCTCCACTTTCGRCRGCCGGRRGANTAGGCNCGTCCGNGGGCCNNTRAGTACTNGGGCCTAGGAACGTGAATGCCCAGAAGATGGGGCCNGCCACACCGAYAARNTCYYRCAGGGGGCGCCAGTTGGCTYGCTGYRRGGTATTTCNNNNGCCNNNNRNNAACGCGACGGTATTTGAAAAGACTTACGCRTGTGATCRGACTCCTGAAATGTTATTTAGGAATYGRCTAGCACTGACAYRCAANNATCAGGCGCATCGGTANCTCTCCGGGCGGCCAGCGCGAATGAAATNGGCTYGCCCCTGRNCRANGGAAGGGAAAAACYTCGGGAYYCTGRGGACTCGCCGACCCTARTGAACRTAGAGCATNGCNYAGTTTAACATTYGGTYNNYGNATGCGGCCNTCCTCTGACCTRCCCAGTGGCTTCCGCNGGACTTTGTACAGCCAGAANNNNRRCCNYCCGCNNCTCCCAAGTGGTTTTTCAAGTACCCNNCCTACATYGCTYCNNCRRAAATGNTCTGCTARTNCNATCGTGRGCCCTATATCTATTAACCAGGTGGGCACTGGTCTYGGACTCGTAGTACTCGATATAARRYYRRNNNYCACTATGCGCAGGTGCAYCGNATTAGTCTGCTTYNNNGAGATGGCACGGTCTGTNTGGATATCGTCCGCNTGGTTAACTGCTCGARNNNNNTCCTCACGNRTCCCGGTGAGTRACGNTGRGNCCGGGGATTCTCGCTGNACACYTTCNAAAGCGTNCAGNGNNTANNTCGTNCTCTTTYCATNTACGCTANCCANTTTCGTAATRTGCYGGYRGCGTAGAGCTGGCACCGCAATGGRTRTCTTGCAGAAYGGCTCAAGYAGCANCGGCGCGTAGCTNTCRAGCCCCTGGCACRGYGNCGRCAGCTAGCNGACYCACCNAATNNCTCGYGTCCCCACGAGRCAGGCRGGANGAATACGGAGNCGTTNTGACGGGGNTTANNCANGTTYCACGCGGTTCAGTNRTTRNGAAAGGGCGGTNCRGRYCCCCAGACCGGNATGGGGYGTAATTGGGGTGTTCGCGAATNCCTGAGGARTNCTCGACCGCTAACCGGYGCNNCGTGGYGAGGGCGCCTYGCTTNTNTATTNACATTGGCAACGTAGATGCATGTGGAACTTCGCAGTGGGARAGARYRAGTCCGTGGCAACGGGCTCTGCGACCANNGAAGTTAYYYAATGCGTCAAAGATTGRYCGTTCGTGTNCNYCCCCGNNGCGCGAAGAAACYNAANRGCCCRCTNCGCANAAGCCTGTCAAGTAACGCCCAATCTCCGGCGNNNGCAGCGCGGTCGCRCCAGCCGGTTGCGAGCAATTCGGGGACTAGCRCNACTGCTAGAAANNGCCGGTATAANGAACCTGCTGNTAGGYTACGACTTAAYTAGCCCACCYCGGAGCAATCGTCCTCGTCGTTAGNGNCTACTCYRNTGGCGTACANCGCNYTANACCCAYGCAGCNGCAGCAGCGTCNNACAAGACTCTAGACGCCYNCAGCATCTCGGGCTAATACTTCTCCTCACTCCTGATCACCGGTGGCCACTAGGCGCCTGTTCCTGGATAACCGAAACGGGCTNNYAAAGNTCGCCATCTTACAAGACYCCTAATATTGTGCGRTGTTNTATYCATGTCANYTCTGAGCGTNGGTGACGACGTGCGACCGCYGNTTTRTRARCCRCACCNYCGGGCTTGCAGRGNRGGAGACGNGNCGCCGTAACGCAGGTGTCGACCTNNATYAGCTAGGAGAANNNNNCYGCRRNNNNGTNACYNYNNACCCYGCACGGACGCYGGCCYCAAGCGRRGGYGNNTACATCGATCCCAGATCTGNGAATCGRRCANCNNNTTCTAGCYGRRNTACCCCCCGAYCYGYRCANNANGNTACCACGCTTAYAGTAATGGGACYYACYGNGNTTGCRNGGATTANAGCAACTCCGTTGCTCCGTCGCAGCGGNATTNAAGCNTCACGTCCTYTGGGGGAGACGGAGTAACGCATTARRGYRCCGTCGGACCRTCCTNCCYGCGTCACGAGTGTNNTANTCATNAGGNATGTGAGNNNAGTTCGRGYRCCANYGTCNNGRCCTCRRRTTTTCGGACTAGCCATNCAGYTCNAYAGRYRNANRYAGCTCNCANGCGGTAGYCNATCCCACTAAATGCGNNNCCAGGNGNNNNRAAARNCNCAAGGTATAATCATTCTNGTGTTGGTCGAACGARRNGAGTAATATAATCCCTATNTCGCAGGTTTGGYCCGTGCCNRAGGAGTTATCAYCAANRANAGTGTCCGCGAGANGCGCTACTTGYCCTTTGTTAGTTTACAYTGCANAATAAACTCGTCGCTGCCTCRTCCCGACYGYNCGCAGTAGNYYYYAGAACYTANNTCGCCGGYTACCCCGACGCCAGCCTCGCNCAGCCNGAGCTAYTGTATCTAAATGGACTATTTCCATTNYNRYNRRCGGGCYYNTCTTANCGTCRYCGCNANRAYNCACAGGCAGTTTGAGAAATCATACACNCGGTAAACCGTAGGGCTAGGCTNCACAGCCTGAGAGGCCAGCCTAYTGCAGATAAGCCNNTCNGTCTCGNCCTGGATCGGGTCCATCGTGANCGGACCGGTYCNCAGGCGGCCCACCTGATCGNNACYRCGNCGGGYNCCTGCGACYTACGCGNCNGCGNCCGGNCGCCGTRRCCGTTCCGYRGAACTGATCCNGGGACTTNNNTGCCCTTGTAAGCACNCTGGAANAGATTGTACCNCGCCACACGACAACATAGNACGCCNGCRGTACCTTRYNNTAGAGGTCAAGTTTYCTCGNNNTGCCCCCRGGCAATCNTGCGYNACRRRATNGGGTCATGGYAGCNCGAATCAGGTGCGGCCYTGGGTCCCAAAAAYTGCAGTCATNNATGCGYAYGYYGAANGYANCGYGGCATTGCTTGCGGRTACTTGCGGCCATCATTGYGTTCATGGTGTNATNAGCTCTCCTTARGYGYGGACCRAAAACGCCACGATCAYGTGCAGAAGGCGCGAATGTGTCCAAAGYGNTAGCACGGACYTYGRANGNGCGRNTATTACGGYYCACTCCTATACAGGTTTAGGTACGRCYTCAYACTGNCTGTNYNYYYGRGCNACCNCCGTCGCGCAAATTCACNGGNGGGGAGCCAACACTYTGTTGNNGCNRGNTCNCRNGGTTACTAANTTGTGGCGTCTCYCTAAYCGAARGAGARTTNYRYTRYCNCGGAGGNNNCCGGCATGRTGTCGYYTCNCYCNNGATATGTNYYGCAAYRYAACGANRNNAGGCNNNGYAYRACANAAAGATGACAAAACANNNNNCGCANTACGGGACGACGTAGCCACCTCGGACCCTGAYNGTGTTTCGTACTGACACAACATCAGTCTCTGACTTTACCCGGGNGCAGACNYCTYAGTCAGCNGGGATTTCYRGCCGAATAAGCCGNGTGTYGGGCCAACCCGAGTCTAGCGRGTCGCGCACTCCTCGRAGGGCACCACACATNTATATTTCACNCRGGGCGGARGCGTCAAAACACTCNRTCCCCACCTGTGGTCAGCCCTRNGAYCTAAGGTAAGCCCCATGNRCGANNGGGAGGCCGAATCCCACTGTCCGGTCCTATTCTCTAAACCGACCGTAAAGTACRTGAAGAANTYGTTAACTNNNCCCGCTRNNCCGCRTGTYRGTANNNNRCYRCNYCGGNNYARTGGCGTTTTTGRNGYTTGYNTCCAAGCGRCGGGAGTTAGTTGGCCCACCCTCAATCTGAYAYAATCNNNNGYAAGCCGATAAAAAACACTCGCCAGCATTTGYATCCACATGNGTCACTGATACTYCYANGNCNAACCGGCRGANCGGAGGTTCTNGATCTRNGATGAANARYCGTCGCRGATACTGCYGGCTRTYRTGTGGCGCGGNNTGTGCCCARNTNGNYCTCYNNGTGGTAGATANACCCTGAYTTNGGGCGTCRAGACAYAANCGCAANCGCYYCAGGTCCTCAGAACATGGGATCCTGATCGCTCAATCCCNCCTCTCAAAGCTACCGCTRCGNCTTCTTAACGACTGGCTANCNTGAACCCTAGATAAYGGGGGTGTTAAGNRNCTCTRGNAGGNCNTCCYYGGATTGYNCGCRTGANNCCAAGACCACTNTGTGGCANRGAGGCAGARCCCCAGGGCTGAGCCCNAGGCCGTCCGCAAACNGGCCAACTGGGCCAGAGGCTGANAGGGGCGCAACGTAACATGACTYTACTAARTACANYGAGCTATTYTAGCAGGAGCCGGTGTGNNNACCCNTNCCACGCGCYTGRRGCTNRNCTGTCNGTAAAGCCANRRCGCCAACTRRAGAGTGCCNGGTACTTTGTATAAATGCCYTTTCCGGGAGCATYGGATCGCTCGGACCCGAGATCAGCCATTRGTTAGRCATTGYCTTTGCACGGCAACGGCCCAATGTCACGAYCTCGGGTCGGYNYNTGGCANACTTTTTAGATCCGACYCGATTGYCCGAGATGGAGGYCCCAGCATYCYNTCRCTGGRATNTATCNTRAAAGCGATCGGGCAAAGYATGTCCTGCTGGCGGAGTCCGTTGRTGACCGRATCAANTNRCCAGATTTTGTNNYTAGCATCYTACAATGGCGAGTCATTACNTTCAGTCCGACGCGGTCTCGCANTGCACATT

Gv10-C-01 RCGRYTNRCTCGTCGYCNTAATGGCNNCCGGAGYCGTCGACCTNGACGGGTCTARGAGGAGTACCCACTGACYGTGATCTACTGGCCATCACGCCTNTTGTCTRAGGRGYCCACTGCYCGRGATCCRTCNTGGNNATGGGCCATGCNGCGNNNACAATGGCCAGGCAGCGNRCAGGAGAGCCGNNNGTACAGACAGGTNTNGNCCTGGCTTTNNNACGGTAGACTACANNATACGGCAGTYTGTAAGTAGGTTGNNYACGNNCATCCTAGGTCTCATGAGGARRTTYTTGACAAACACGGATNANYCRCAGGGCATTGATTATTCCYAATGYCCGTGGATNNNNTGGTTGATGATGTAGACTTANNAACGGTGGAGCCANTCTGGAGYTGCNNGGTTGGGGCGGGCGCCGCNGGYCAGGARCCTARGTACRNTACATTGCCCCCAGATAGCCTTNRANCRCYCTNNNNYGCCTGYTGARTGGGCTRYRTYNGGTTACCTTYNGCTRTCNACGAATCCTANCRGANACAGCTAAGTATCCTCGTCTARACTCRNNTAAGAGNNTAGRGCYGACGCATACGGTTTCNACACCRACGGGGCGAGGAAAGAGCGCTTCYGGCTACCAACGGTGGACATTCCNNTNAAAACAAGYNACARCTRAGGNTGANYAGGCCAATCTCCTCGATCCGCTCCCTTATGTGGANRCCYCTGCCNNNGCTTACGCCAGTANCTAGCGGAYTGCGCCGTGCGGTGGGTACCAGGTGAATCCNANYNCAYGCGAATGNYNGGCACNACCRNGRACTAGCYGGCNGGTARCNAGNAACTGATNTTNTRTGAGYRCRTGCCCCGCYCGCAGTACAGATNCNGCTAGNCCTGTNCACAANNGAATTGTTCTNAGGCTTGCGARTNYGACTRAGNTCGTARAAACGGNCGNCTTGGTCCGGTCNGTCACNAAAGTTGCAAATTGTNGTAACTCACGRTAGCCATGGCATNTNAATTNGGAATAGCCCGCGGTGTAAAGGCGRNCCGCGTCGGCGAGAGCCGNNTNGCCGCAAGGGATAATCAGATGATTAGATTATGTCNGAAGGAACGCACYCCTCNNNACRNAGGTAANAYGCTGTGGCTCCACTTTCNGNRGCCRRGGGATTAGGCNCGTCCGGGGGYCGNTNAGTACTAGGGCNTAGGAACGTNNATGCNCRGANGATGGGGCCNGCCACNCCGATAANTTCCYACARGGGGCNNCNNTTGGCTTGCTGCRRRGNATNTNTNTGGCCYGACGGTAACGYGACNNNNNTTGAAAAGACATANNNNYGNGTACGGACTCCNGAAATGTCRNNTAGGAATNNACTAGCACTGACRTATNNNNATCAGGCGCATCGGTATCTCTCCGGGCGGCCRRCGCGAATGAAAYGGGCTYGCCCCTGGACAAAGGAAGGGAAANACYTCRRGAYYCTGGGGACTCGCCGACCCTAATGAANATAGAGCATNGCNTAGTTTRACATTTGGTCTNYRNRNGCNGCCRTCCTCTGACYTACCCAGTGGCNTNCGCNGGACTTTGTRCRRCCAGAAAGCGAACCTCCCGCACCTCCCAAGTGGTTTTTYAAGTACCCTACCTACNTNGCTYCRACGAAAATGATYTRYTAGTGCAATCGNGRGCCCTNNNNCTATTAACCRGGTNNGCACTGGTCTCGGRNTCGTARTACTCGATATAAGGTCGGGGRYCNNTANGTACAGGTGGACCGNATTAGYCYGNNTYAYTGAGATGGCATGGTCNNTTTGGATATCGTCNNNNNNGTNNACTGCTCGAANCCNNTCCTCACGAGNCYCGGNGAGTGACGGTGRGNCCGGGGANTCNCGNTGGACANYTTNNNNNNCGTCCAGNNNNTANNTCGTTNTCTTTNCATNTACGCTANNCAATTTCGTAATGTGCYGGYRGCGTAGAGCTNGCANCGCAATGNNTGTCTTNNNGNATGGCNCAANCRTCGGCGGCGCGTCANNATCGAGCCCCTGGCACGGCGNNGACNNNNNNNNGACYCACCNNATRRCTCGCGNNNNNNCGAGNCAGNNNGGAYGAATACGGAGNNGTTNTGACGGGGNTTAYACACATTNNNNNNGGTTCAGTGATTATGANAGGGCGGTNCNGNCCCCCAGACCTGYATGGGGYGTAATTGGNGTGTTCGCGGTCACNNNNNGARTACTCGRCYGCNAAYCGGCGCATCGNNGTGAGNNCGCCTYGCTYTTRTATTAACATTGGCNACGTAGATGCATGNGGAACTTYGCRGYGGGARAGRGYGRRTCCGTGGCAACGGGCTCNGCGATAACATTAGTTAYCYAATGCGTCAAAGATTGATYGTGCGTGTCCNYCCCCGCNGCGCGARGNRACTANAAGGCNCGCTRCGCANNNNNCTGNNGAGTAACGCCYAATCTCCGGCGNRYNYAGCGCGGTCGCRCCAGYCRGNNGYRRRCGATTYGAGGAYTARCACNACNNCTAGAAACGGCCGGTNCAGGAAACYTGCTGNTAGGTTACGACTTAAYTAGCCCACCCCGGAGCAATCGTCCTCGTCGTTAGGGGCTACTYYRGTGGCGTACATCGCATTAGACCYACGCAGCAGCGGCAGCGTCTTACAATACNCTAGACGCCYACAGCATCNCGGGCTAATACTTCNCCNNACTCCTGRTNACCNGTGRCCACTAGGCGCCTNNTCCTGGATANYCGAAAYGGGCYTNTAAAGNTCGCCATCTTACAAGACTCCCGATANNNTGCGGTGTTTTATCCATGNNAACTCTGAGYNTCGGTGAYGNYGYRCGACCGCCGGTTTATGAACCGCACCAYCGGGYYNGCAGNGTGGGAGRCRCGGCGCCNTAACGCAGGTGTCGNNNNNYATYAGCYRRGAGAATGGRACCGCGRNCACNCRACCATAGACYNYGCACGGACGCTGGNCYYAAGCGRGGGTGTCTACATCGATCYCRGRCCTGANANTCGAACAACNCCTTCTAGCCNAAATACCCCCCNNTCCGYGCATAAAGNTANCACGCTNNCCATTATGGGACCCACCGNGNTTGCRNGGATTNNNGCNACTYCGTTGCTCCGTCGCAGCGGNATNNAAGCNTCACGTCCNCNNNNNGAGNCGGNGNGAAATATTAAGTCNCCGYYGGNNCGTCCNNCCNGCRTCACGTGYGYAATANTCATTAGNTATGTGAGNNNAGTTCGNGYRCCAGNGTCTTNRCCTYRRATTTTCRRNNTAGCCATACNGYTCNRCAGNYRTNNNNAGCTNNCANGCGATAGCCCATCCCTCTAGRNGCGACTCCAGRNGACGCGAAAGCCYCAARGNGTAATCANYCTNGTGTTGGTNGAACGANAAGAGTAATATAATCCCTATTTCGCAGGNNTGGTYCGTGCCGRAGGAGTTATCACCAATRAANNNGTCCGCGAGANNCGCTANNNGYCCTTTGTNAGTCTACAYTGCATAATAAACTCRNCGCTGCCTCATCCCGRCCGTACGCANNNGATCTTAGAACNTAAATCGCCGGCTNCCCCGACGCCAGCNNNGCTCAGCCTGNGCTATTGTATCTAAATGNACTRTTYYCATTNTNGYNGNNGGGCCYNTCNTAATCTCGTCGCTANAACNCACAGGNAGTTTGAGAAATCATACANGCGGTAAACCGTAGGGCTAGGCTANAYRGCCTAAGAGGCCAGYCTATTGCAGATAANNCGCNCAGTCTCAGCCTGGATCGGGTNCATCGTGGRCGGACCGGTNNNCAGGNGGCCYRCCTGATCGGAACCGCGTCGGGCCCCTGCGAYCTANGCGYCYGCGNCCGGGCGCCGTGGCCRTTCCGNNGAACNGATCCNGGGACTTGGATGCCCTTGTAAGCACNNTGGAATAGATTGTNNNACGCCACACGACAACATAGTACGCCTGCGGTNCCTTGTCTTAGAGGTNAAGTTTYCTCGTGNTGCCCCCAGGCAATCATGCGTAANNGGATGRGGTYATGGNAGCNCGAATCNGGTGCGGCCNTGGNTCCCAAAANNTGCAGTCANNNAYGCGTACGTCGAATGCACCGYGGYGTTGCTNGCGGRTNNNNGCGACCGTCATNGYNNTCATGGTGTNATGAGCTCTCCTTAGGCGCGGACCRAAAACGCCACGRTNATGTGCAGAAGGCGCRAATGTGNNCAANGCNTTAGCNNNGANNTTGRANGCGYGAATATTACGGCCCACTCCTATACAGGTYTAGGTACGGCTNNACACTGGNTGTACCCYYRGGNGACCNCCGTCGCGCAAATTCACNRGRGGGGAGTCAACACTTTGTTGNNRCGGGYYCNCRNGGTTNCTAAATTGTGGCGTCTCYCTAATCGAANGAGARTTNTGNYACCACGGAGGTCGCCRGCANGNNGTCGCTTCACYCCCGATAYGTATTRYAAYAYAACGANRCCAGRCGCCGYAYRNNNNANARATGANGAAACACGATACGCAGTACNGGACGACGTAGCCACCTCGGACCCTGAYNGTGTTTCGTANNRACNNNNCATCAGTCTCTNACTTTACCCGGRTGCAGACNYCTCAGTCAGCTGGGATTATTNGCCGAATANNNCGAGTGTCNGGCCAACCCGARYCTNGCGRGTCGCGCNNTCCYCGRAGGGCACCACACATNNATNTTTCACGCNGGGCGGNRGCGCCAAAACACTCNRTCCCCACCTNNGRTYRGCCCTRNGAYCNAAGGNAAGCCCCAYRNGCNATTGGRARGCTGRATCCCANTGTCCGGTNTTATTCTCTAAACCGACCRTAAAGTACATGRRGANTTYGTTANCNTGACCYGCTAGNCCACGTGTYGGTNNACARCYRCNTCANNCTAATGANGTTTYYRRNGYTTRCNTCCTAGCGNNGGGNNTTAGTTGGCCCACCCTCRRTNTGAYAYANNNTACNGYAAGCCGATNNAAGGTAYTCGCCAGNATTTGNATCCACATGCGTCACTGATACTNCNNTGNCTAACNGGCGGANYRGAGGTTCTNGATCTRTGANGAANAGCCGTCNCRGAGGTTACTGGCTNTYRTGNNGCGCGGRCTRTGCCCAGATTGTNCTCYAAGTGGTAGATAGACCCTGACTTAGGGCGTCGAGAYAYAANCGNAATCGCNTCNNGTCCTNRGAACNTGGGATCCTNNTCGCTCAATCCCACCTCTCAAAGCTACCGCTACGNCTTCTTAANGACTGGCTANCNTGAACCCTAGATAANGGNNGTGNTAAGNATCTCTRGAAGGGCNNCCTTGGATTGTNCACRTGATCCYNAGACCACTTTGTGGCANRGAGGCAGRRCCYCNRGGCTGAGCCCNAGGCCGTCCGCAAANNGGCCAGTTGGNCCAGAGGCYGANAGGGGCGCARCRYARCATGNCNYTACTAAGTACANYGAGCTATTYTAGCAGGAGCCGGTGTNCATRCCCGTNCCAYNCGCTTGNNGCTNANCTGTCGGTAAAGCCANRRCGCCAACTAGAGATCGNATGGNACTTTGNATAAATGCCCTTTCCGGGAGCATYNGATCGYTCGGTCCCGAGCTATGCCATTRGTTAGACATTGCCTTTGCACGGCAACGGCYYGGTNTCANGACYTCGGGTCGGTNYNTGGCANNCCTTTTANATYCGACCCNGTTRCCYGNNNNNNNNGCCCCAGCATCCTTNCGCTGGAATNTATCNTGAARRCGATCGGGCAAAGCRYNNCCTRYNGGYGGAGTCCGTYRGTGACCGGATCANGNNRCCAGNNNTNRNACCTAGCATCYTACANTGGCGAGTYATYRCATTCAGTCYNACGCGNNCNNGCANTGCACATT

Gv10-C-05 RCGATTTACTCNTCRYCNTANNGNCCGCCGGRRYCGTCGACCTNGACGGGTCTARNANGTTTACCCACTGACCGTGATCTNNTGGNYATNACGCCTYNTGTCTRNNNNGCCCACTGCYCNNNNTCCRTCTTGGGTATGGGCCANGCTGCGCTTACAATGGCCAGGCAGCGTNCAGGAGAGCCGAAANTACAGACAGGTTTGGACCTGGCTTTGGGACGGTAGNYYNYAYAATACGGCAGTYTCTAAGTAGGTTGGGTACGCNGATYNTAGGTCTCATGAGGAAGCTTTTGACRAACNNNNATCACYCRTTGGNCNTTGACYATTCCCANTGCCCGTGGATAGTTTGGTTGATGRYNTAGACTTAAGAACGGTGGNGCCANTCTGGAGNTGCTAGGNYGGGGCGGGCGCCGCNNGCCAGGAACYTAGRNAYATTACANTGCCGCCAGATGATCCTAGAGCACCCTCTCTTNNCTGCTGAATRGGCTGCRTYTGGTTACCTTCCNNTRNCAACGARYCCTAGCGGANACAGCTAANYATCCTCGNNNNAACNCNNGTRNGAGTATAGRGCTGNCGCATACGRTNNCAACACCRACGRGRYGNGGAAANAACNNNNNCGGCTACNAACGGTGGACANTCCCCTAGNNNNNNGCGRYNRCTAAGGNTGARCAGRCCAATCTCCTNGANCYNCTCCCTTATGTGGAAGCCCCNNCCCCNGCTTACGNCAGTANCTNGCATANTGCGCCRAGCGGTGGGTACCAGGTGRNTCCNANNGCAYGNGAATGGYNGGNACNNCCGTNNNCTAGCCGGCNGGTAGCTATGAACTGATATTATGTGAGTACATGCCCCGYTYGNACTCCGGTTACARCTAGNNNTGTACACAACGGAATTGTTCTTAGGCTCGCGAATNNGNNTNNGATCGNNAAAACGGGCGYCGNNGTCCGGTCAGYCACTRNNNNNNYRNNYNGNAGTAACNCGCRGYAGCCATGGCATNTAANTTAGGAATAGCCCGCGGTGTARRGGCGRNCCGCNTCGGCGAGGRCYRNNTAGCTGCAAGGGNTAATCAGACGATTARAYTATGTCTGAAGGRACGCACTCYTCTTAACAAAGGTCNCACGCTGTGGNNCCANTTTCGRCNGCCGGRRGATTAGGCACGTCCGGGNGCCGCTNNGTACTAGGGCYTNRGAACGTGAATRCACGGACGATGGGGCCNGCCACCCCGACAAGNTCYYRCAGGGGGCGCCAGTTGNNNNGNNNCRRAGAATTTCTATGGCCCGACGGTAACGCGACGGYATYYGRAAAGAYNNNCGCATGNGNNCGGACTCCTGAAATGTCGATTAGGAAYCCANTAGCACTGACAYRNANRYATCAGGCGCATCGGTATCTCTCCGGGCGNCYGACGCGAATRAAATGGGCTYGCCCCTGNNCRAAGGARGGGAAAAACYNNRRGACYCTGAGGNCTCNNCGACCCTARTGAACATAGAGCRNCGCCCAGTNTRACNTNTNNNNNNNRCGAGCARNNNTCCTCTGACNTACCCNGNGGCTTCCGCAGGACTTTGTRCRRYCANNAAGCGAACNYCCYGNNCYTNCCNAGTGGTTTNNNAAGTNNCNTNTCCACRTYGCNCCGNNNNNNNTGNTCTRYCTRTNCNATCGNNNGCCCTNNNNCTATTAACCAGNTNNGCNNNNNNCTCGGGGNCGTAGTACTCGAYNTAAGGTCGGRNAYCANNNNGTGCAGGTGCACCGTNTTAGTCTGCTTCATTGAGATGGCAYGGTCTGNNTGGATAYCGTCCGCCTGGTTATTGGCTCTNANCCCGTCCTNACGNGTCNNNNNGAGTGACGGTGGGNCCGNGGATTCTCRCTGGACAATTTCCAAAGCGTCCAGAGNNNANNTCGNNNTCTTTYCANTTACGCTANNCNATTTCGTAATGTGCCGGCAGCGYAGAGCTGGNACNNNNNNGGRNNTCTTNNRGAATGGCTCAAGNANCRGCGGCGCGTCACTATCRAGYCCCNNNCACRGYGNYGGCNNNNNNNNGTCCCACCCAATAACTCGCGNCCCCACGNGGCNGGCGNGACGAATNCGGAGNNGTYATGACGGRRNTTATCCACRTTCCACGCGGTTCNGYGATTANNNAAGGACGGTGCAGACCCCCAAACCGGYATGGGNNGTAATTGGGGTAYTCGCNRNNACCTRNNANGTNNTCGACCGCNAAYCGGYGCANCGTGGYNNGGGCGCCTCGCTNNTRTNNTAANATTGRCTANNNNNNNNCANNNGGAACNTYGCRGNAAGAAAGAGCAANNCCRTGGCAACGGGCTCTNNNNNNNCATTAGYTRCTTAATGCGTCAAAGATTGATYGTTCGTGTCCNYCCCYGCGGCGYGAAGNRNCTAAANANCCNNCTGCGCAAAAGCCTGTCRAGTAANGCCYAATCTCCGGCGAGTGNGGANNGGTCGCGATAGCGGGTTGYRRGCGATTTGNNRNYTAGYRCNACTGCTAGAAACNGCCGGTNTAAGAAACYNGCTGGTAGGTTACGACTNNAYTAGCCCGCTCCGGNGCAATCGTCCTCGTCNTTAGGGGCTACTCTAGTGGCGTACANCGCAYTAGACCTACGCAGCAGCRGCAGCGTCTNACAATACTCTARACGCCNNNNNCNTCTCGGNCNNNTNCTTCTCCNCNCTNCTGATCACCRGTGANCACTAGGTGCNNNNNNNTNNATANNCGAAACGGGCCTCYARRGNTCNNCRTCTTACAAGACYCCTGATANNRTGCGRTGNNNTATTCATGTCACTGCTGAGCGTNGGTNNNNAYGCANGACCNCCGGTNTATGAACCGCACCNNNNNNCYTGCAGNGNNGGAGACGCGGCRCYNNNACGCAGGTGTCRACCTGTNTYAGCCGAGAGTGTGGRACCGCGATNNNGYGACCCCTTACNCNNNNNGGACGCYGRNCNCAAGCGRNNGTGTCNACACCGATCCCGGGCCTGNGAATCGAANAACGCCTTCTAGCCGAAATACCCCCCGANCYGYGCATAAAGTTACCACGCNNNNNRTNATGGGACYYACCGGGTTNGCRNGGATTNNAGCANCTCCGTNGCTCCGTCGCAGCGGAATTCNNGCCTCACGTCCNYTGGRRNAGACGGNGNNAAANATNCAGNYGNCNYYGGNCCRNCNGTCCTRYRTCACGNRTGTAATANTCACNRNNNNNGTGAGAGGAGTTCGNGCACCAGYGTCTTNACCTYGARTTTTCNNRYNAGCCANCCAGCTCCRTAGRYANNGGCAGCTNNNAAGCGGTAGNCNATCCCACTARATGNNACTCNAGATGACGCGAANGCCCCAAGGTATAATCANNCTCGTGTTGGTCNNACGARAAGRGTAATATARTCCCTNTTTCGCAGGNNTGGTNCGTGCCGRAGGAGTTATCACNNNNNNNAGNGTNCGCGAGNTGCGCTANNNNNCCTTTGTNAGTNTNCAYTGCANAATAAACTCGTCGCTGCCTNNNCATGACCGTACGCAGTNGCCTCCAGAGATNAAANNGCCGRYTNCCCCGACGCCAGACTCGNNNAGCCTGAGCTATTGTATCTAAATNGACTGTCCTCATTGTTGCGGNNNNRYCCCNNNTANCGTCGTCNNNNTAACACNCAGGCAGTTTGAGAAATCNNNCAYGCNGTANNNNNNNGGNCTAGGCNACNCAGCCNGAGAGGCCATCCTATTGTGGATRAGNCNNTCNGTCTCGCNCTNNNTCGGGTTCANNGTNRACGGACCGGTCCTCAGGAGGCNNNNNNGATCGNNACCGCGNCGGGCCCCTGYNNYYTNNGCGCCTGCNCCCGGNYGCCGTRRCCRTTCCGTNNTAYTGATCCNNGGACTNGGATGCCCTTGTAAGCACNCTGGAANNNATTGTAYCACGCCACANGRCRRCATAGTACGCCNGCNNTNCCTTACNNTAGNGNTNNAGTTTCNTCGTATTGCCCCCNGGCAATCNNGCGNNAAAGGATGGGGTYATGGYAGCGCGAANYAGGTGCGGYCYYNGNNCNCRAAANNTGCAGNNNNNNATGNNTACGTCGAATGCACCGYGGCRTTGCTTGCGGGTNNNNGCGRYCGNCAYAGYGTTCATGNNGTTATGAGCTCTNCTTARGCGCGGCCYGNNNNNGCCANNNNCATGNGCAGAAGGCGCGARTGNGNNNNAAGNGNTAGCACGGACCTTGAANGCGNGRNTATTACGGCTCACTCCTATAYAGGTNTAGGTACGACCYCRYNCTNGCTGTNCCNNNNGGGGTCCGCCGYCRCGCAAATTCNNNNNNNGGGAGTNNNNNNNTTGTTGAARCGGNNNNNCRCNGTTACTAAATNGTGGCGTCTCYCTANCCRAAGGAGAGTTATGCTACCACGGAGGTCGCNGGCATGATGTCGCTTCACYYTTCATAYGTANTGCRATRTANCGATACCAGGCGCCGYACGACATATAAATGANGAAACANRRYRCGCNGTACGGGNCGRCGTAGCCACCACGGACCCTGATCGTGTTTYNNNNNRACACAACATNAGTCTCTGACTTTACCCGGGTGCAGACNNCTCNRTCAGCTGGNATTAYYRGCCGAANAAGCCGTGNGTCNGRCNAACCCGANNCTAGCGRGTCGCGCACNNCNCGNAGGGCACCACACATCNRYNYTTNACTCRGGGCGGCNGCGCCAAAACANTCARTCCCCACCTRNGGTCRGCCYTNTGACCTAAGGTNANNNNNATGTACGATTGGGAGGCCGAANCCCAAYRTCCRGTCCTATTCCTCGAACCGACCNTAAAGTACATGNNGANNNYGTTARCTYGACCNNNTNGGCCGNNTGTNGGTNCACAGCTNNNNNRNNCTARTNNCGTTTNNNANGTTTRCCTCCAAGCRRCNGGAGTTAGTNGGNCNACCCYCNNNATGAYAYNANNTACAGCAAGCCGATRRAANNNACTCGCCAGNATTTGCAYNCACNNNNNNCNCTGACACTTCCATRNYNAACTRGCGGAANNGARGNTCTNGATCTNTGANGNNNARYCGTCRCRGANGTNRCNGGCNNNTGYRTNNCGCGGRYTGYGCCCNGATTGTYCTCCAAGTGGTAGNTAGACCCTNRCTTAGGGCGTCAAGAYAYAATCGCNATNGNCTCAGGTCCTAGRAACGTGGNNTCCTGANNNCTCAATCCCACCTCTCAAAGCTAYCGCAACGTCTTCTTAACGACTGGCTAGCTYGAACCCTAGAYAACGGGGGYGTTNRRGGAYNNNGTCAGGNCNNCCYYGGATNGTNCNNGTRATCCTTNGNNNACTTTGTGGCATRGRGGCNGRGCCCCNNGGCTGAGCYCNNGGTCGTCCGCNAACTNNNNANNTGGGNCAGAGGCYGANAGGGGCGCAACGTAACNNNACTTTACTAANTAYACNGAGCNNNTTTAGCAGGAGCCGGTGTGCNNACCCGTAYNATTCGCTNNAGGCTNRNCTGTCGGTAAAGYCANRRNNNNNNNNAGANAGTGCCNGGTACTTTGTATAAATGNNCTTTCCGGGAGCATCGGATCGYTCGGTCCCGAGNTNNGCCATTARYYAGACATTGYCTTTGYRCGGCAACNNNNNRRTGTCANGAYCTCGNGNCGGTATGTGGCANACTTTTNRRNTNNNNNYCGRTTACCCNAGATGGAGGCCCCAGCATCCTTNNNCTGNNATTTATCNTGAARRCGATCGGGCAAAGNNYNNCCNGCTGGCRATRTCCGTTGRNGNCCRRANCNAGNNACCAGANYTNRNACNTAGCATCTTNCARTGGCNNNTTATCGCNNNCAGTCCGNNNNNNNCTYGCAGTGCANANN

Gv10-C-10 ACGATNNNCTCGTCGCCATANNGTCCGCCGGRRCCGTCGACNNNNNNGGGTCTARNAGGNNTACCCACTGACTGTGATCTACTGGCTAYCACGCCTYTTGTCTNAAGAGCCCACTGCCCGRGATCCGTCTNNNNNATGGGCCATGNNNNNCTTACAATGGCYNGGCAGCGNGNNGGANRRCYRTATNTACAGACAGGTTNGNAYYTGGCYYTGGAACGGTANNNNTYATAATANNNNAGTYTNTAAGTAGGTTGNNTACGCAGAYYNTAGRTCTCATGAGGANNNTCTTGANRAAYRCGGATCACTCGNNGGGCNNNNNNNNTTNCYAATGCNNNNNNNNAGTTYNNYTGATGRNNTAGANTNAAGAACGGTGGAGCCRNTNNGNNNCTGNTAGRNTGGGGCGGGCGNCGCANGTCAGGAGCCNNAGTACGNTACANTGCCGCCAGATNNNCNYARANCNCNNNCYCTTNNNNNYTGARTRGGNNGCATTNNGTTACCTTTGGCYGYCAATGARCNNNAGCGGANACNNCGAAGTATCNNNNTCTARACYCRNGYRAGAGNNTAGAGCTGACGCANNNNRTNNCANCACCNACGNNNNGAGGAAAGNNNGCNTCCRRCNNCNAACGGTGGACATTCCCCTAGAAACAAGCGGTCRCTRAGGGTGAGYAGNCCAATTTCCTCNATCTGCTCCCTTATGTGGAAGNNCCTGCCGCCGCTTNNGCCAGTNCYTNGNNNATTGCGCCGNGCGGTGGGTNYCAGGTGAATCCTNNCTCATGCGAATGGNNGGCACTACCNNAATCTAGCCGGCTGGNNGCTANGAACTGATATTNTNTRTGTACATGCCCCGNNCGNACTNNNNNTACAGCTANNCCTGTACNCAACGGAATTGTTCTNAGGCTYGCGAATNYGRNTRAGTTCGGAANAACRGGCNNCGTGRNYNNGTYNGTCACTAAAGTTGCAAATTNNNNTAACTCGCGGCAGCCATNNCATCTAAATTNGGAATAGCCCGCGGTGTAAAGNCGACCCGCGTCGRCGRRRRCCGCTTNNCYGCAAGGGATANNNNNNCGATTAGATTATGTCTGAAGGAACGCACCCYTCTTAACRNAGGTNAYATGCTGTGGCCCCACTTTYGGCRGCCGGGGGATNAGGCACGTCNNNNGGYCGNTNNNNACYAGGGCCTNRGRACGTGAATGCCCANANGATGGGGCCAGCCACCNCRANAAGTTCYYRCAGGGGGCGCCAGTYNGYTTGCTGYRRGGTATTTNTATGNCCCNNNRNNAACGCGNCNGYNNCCTGAAAGANNTANNNRNGTGATCGGACTCCTGAAATGTTANTNAGGAANCNRCTAGCACTNNNNNNYNNNNATCAGGCGCATCGGTANCTCTCCGGGCGGCCAGCGCGAATRAAATGGGCTYGCCCCTGGACAANGGAAGGGAAAAACCTCRRGANNCTGNNNNCTCGCCGACCCTARTGAACATAGAGCATCGCGTNNTTTRACATNTGGTCTNYRNGAGCAGCCGNCCTCTGACCTACCCAGTGGCTTCCGCNGGACTTTGTRCRRNCANNAAGCGAACCYCCCGNNNYTCCCAAGTGGTTTNTCAAGTACCCTANNNACNTNNCTTCAACGGAAATGCTCTGCYNNNNCNATCGTGGGCCCNATATCTATTAACCAGGTGGGCACTGGTCTCGGNNNNNTAGTACNCGATATAARRYYRRGGGTCACTATNTNNNNGTGCNNNNNATTAGYCYGCTTCATTGAGATGGCATGGTCTGTTTGGATANCGTCCGCYTGGTTAACNGCTCNAANCCCRTCCTCANGAGTCCCGRTGAGTGACNNNGGGTNCGNNNNNTCNNRCTGGACACYTTCCAAAGCGNNCAGAGNATANNTCGNNCTCTTTNCNNNNACGCTANCCAATNNNGTAATGTGCTGGCAGNNNNGAGCTGGYACCGNNNNGGGTGTCTTNCRGAATGGYTCAAGNANNNGCGGCGCGTCACTATCNNGYCCCTNNNNNGGCGNNGRCAGCTAGCNGNNNNNCCCRATRRNNNGYGTCCCNACGNGGCNGNNNRRRCRRRTNCGGAGACNNNATNNCGGGGTTTNNNCACGTTTCACGCGGGCCNGNCGTTRTGAAAGGRCNGTGCAGACCCCCANACCNGTATGGGGYGTAATNGGNGTRTTCGCGRNNACCTNNNGANTNCTCGACCGCTAGTCGGCRNANCGTRNNNNGGGCGCCTNGCTTYTATATTAANATTGRCTACGTNGANGCATGTNNNACTTNGNNNCGGGAAAGAGYGANTNNNNNGCAACGGGCTCTGCGACCANNNNAGTTACCTAATGNGTCAAAGATTGATTGNNNNNGTCCNYCCCCGCGGNGYGAAGNRACTAAAAGGCCCGCTRCGCANAAGCCTGNNGAGTAACGCCCRNTCTCCGGCGNRYNNNGNGCGGTCGCNNYAGYNRGTTGNAGGCRATTYGAGGNYTARCACNNCTGCTAGANNNNNNNNNTNYARGRAACCTGCTGCTAGGTTACNNNTTAATTAGCCCGCCCCGGANCAATCNYNYTCANCNTTAGGGGCTACTCTAGTGGCGTACATCGCATNAGACCTATGCAGCAGCRGCAGCGTCTTACNNTNNTNNANANNNNNNNNNCRTNNCGGNCTACTNCTTCTCCNCACTCNTGATCACCNGTGRNCACTNNGYGCCNNNTNCTGGATNNNCGAAACNNGCCTCYAAAGCTCGCCANNNNACAAGACYCCTNNNNNNRTGCGGTGTTTTATCNNNGTNAACTCTGNNNNNNGGTGACGACGCACNACCGCCGGTTTATNNNNNNNACCANCGGGCTTGCAGGGNRRGAGGCACGGCACTNNNACGCAGGTGTNGAYCYGNATYAGCTAGGAGAATGGAACCGCGNNCNNNYGACCNYNNACYNCGYRTGGNCGCYNGCCYCCAGCNNGGGTGTCNACANCGNNCCCRGRYCTGAGAATCGRRCAACGCCTTCTNGNCGAAATACCCCCCGAYCYGYACATAANNNTACCACGCTTACAGTAATGGGACYYACCGGGNTTGCGAGGATTANAGCAACTCNNNTGCTCCGTCGCANCGGTATTAAAGCNTYACRTCCNNNNNRRGAGACGGNGNNAAATATTCRRNCRCCGYYGGNNCRTCTGNNNNGYRTCACGNGTGTAATANTCACNNGNNATGTGAGAGGAGTTCGGGCACCANTGTCNNNACCTYGARTTTTCGGRYTAGCNNNNCANNNNNNTAGACNNNGGNAGCTNTNNAGCGGTAGCCCANCCCNCTNNATGCGACTCCAGNNGNCGYGRAAGCNTCAAGGTATAANCATTCTNGTGTTGGTCGGACGNRNAGAGTAATATAATCCCTATNTCGNAGGTTTGGNCCGTGNNNAAGGAGNNATCACCAATAAAAGTGTCCGCNAGATGCGCTACNTGCCCTTTGTTAGTNTACACTGCAYAATAAACTNGTCGCTGNCTCATCNYGACTGTACGCAGTNGNNNNNAGARNYTAANNCGNNNNNTACCCCGNCGCCAGCCTNNCNCAGCCAGAGCTNNNGTATNNNNATNGACTRTYYYCNNTGYNRYNRNNGGRYYCCTCTTAAYNTCGTCGCNANRNNNCACAGGCAGTTTNNNNNNNNNTACAYGCGGTANNCCGTAGGNCTAGGCNACACAGCCTGAGANNNNNNCCTACTGCAGATNAGTCGCNCAGACTCNNCCTGGATCGGGTNCANCGTGRNCGGACCGGTYCGCAGGNNGCCTGCCCNNTCGGAACCGCNNNNGGNNNCTGYGATCTACNCGCCTGCGNCCGNGYGCCGTGGCCGTTCCGYRNAACTGNTCCNRGGACTNGGATGCCCTNNNNAGCACNCTGGAATNNANNNNNNNNCGNNACANRRCRRCNTAGNACNNNNGCGGTNCYNTNNYTTAGNGNTCAAGTTTNTTCGTRTTGCCCCCGGGCAANCATGCGYNAAAGGATGGGGTYATGGCAGCNCGAANCAGGNNNGGTCTCTGTTCCCAAANATTGCAGTNATNNANGCGTACGTNNNATGCNCCNCGGCNNTGCTNNCGGRTANTTGCGRYCRNNATNGNGTTCATGGTGTTATGAGNNCTCNNNNGGCGCGGACCGNNNNNGCCACGATNATGTGCAGAAGGNNNNARTGTGNNCAANGCGATAGCRCNNNNNTTGAANGCGCGGCTATTACGGYCCACTNCTATAYAGGTNNAGGTACGNNTTCACACTGGCTGTNCCCTCGGGNGNCNNNCGTCGCGCAAATTCACNGGRGGGGANNCAACACTNTGTTGNNGCGGRYTCTCNNGGTTACTAANTTGTGGCGTNTYTCTANCNGAARGNNAGTTNNNCTNCCACGGAGGTCGCNRGCATGATGTCGCTTCNNTCNNGATATGTNTYGCAANANANCGAAGCCAGRCNYCGCNNGACATAAAGATGACGNAACAAGATACGCAGTACGNGNYRRCGTAGCNNCNNCGGACCCTGANNGTGTTTCNNRNYNACNNNRCATCAGTCYYNGANTTNACCCGGRNGCAGACTCCTCAATCAGCNGGGATTTCYRGCCGAATANGCCNNGTGTCGGRCNANNNCGAGTCTAGCGNNNNGCGCACNNCYNGAAGGGTACCACACATANATNTTTCACNNNNNNNNGNNNNGYCAAAACACTCNRTCNNNNCCTNNRGTYAGCCYTANNANCTAAGGTAANCCCCATGTACGANNGGGAGGCCGAATNNNNCYRTCCRRTCYTATTCTCTAAACCGACNGTAAAGTACRTGRRGAATTNGTTANCTTNACCCGCTAGACCGCATGTCGGTCYACAGCTACGTCNGTNNNATGGNACTNYYRGCGCTTGCNTCCNNNCGRCRGGAGTTAGNNNNNNNACCNNCRRTNTGNTACAATCTACAGCAAGCCGANNNNNNNNAYTCNCNAGNANNNGCAYCCANATGCGTCACTGNYACNYCYRTGNCNAACYGGCGNNNTAGAGGTTCTCGATCTGTGATGAANANYNGTCACAGATACTGCYGNCTNTYRNGNNGCRCGGGYTGTGCCCNGATNRCTNTCCAAGTGGTAGNTAGACCCTTANTTAGGGCNTCAANNYANNATCGCNATCGNNTCAGGTCCTAGGAACGTGGNNTCNNGAACGCTNNANCCCACCTCTCAAAGCTNNCGCTRCGNNNNNTTAACGACTGGCTANNNNNAACCCTAGACANNNGNNGTGTTAAGGRNCTCTNNNAGGTCNTCCYYGGATTGTNCRCGTRANNCTTAGACCNCTTNGTGGCANGGGGGCAGRRNNNCNRGGCTGAGCCCNAGGCCGTCCGCNAACTGGCCANNTGGGCCAGNNNCTGANAGGGGCGCAACGTAACNNGACTTTACTAAGNANACYGAGCTATTNTAGCAGGAGCNNGTGTGCATACCCNTACCANNCGCCNNGAGCNNNNCTGTNNNNAAAGYCAAAACGCCAACTAGAGANYGNNTGGTACTTTGTATAANNGCCCTTTCCGGGAGCNNNGGATCGCTCGGTCCCGAGNTNNGCCATTNRYNAGRCATTRCCTTTGCACGNNANCNNNNNRRTGTCACGACCTCGGGNCGNTTCCTGNNNNACNTNTTAGATNNNNNNCGATTRYCCRAGATGGAGGCCCCAGCATCCTNNNRCTGNNATTTATCNTGAAAGCGATCGGGCAAAGNNTGTCCNGCTGGCNNNNTCCGTTRGNNNNNNAATCANNNNRCCAGNNNNNGNNNNNNGYATNCTACARTGGCGAGTTATCGCATTCNNNNYNNNNNNNNCTCGCANNNNNNATT

Gv10-E-05 NCGATTTACTCGTCRCCATAATGNCCGCCNNNNYYGTCGACCTAGACGGGTCTANRRRGAGTACCCACTGACCGTGATCTNNTGGNTATCACGCTCTTTGTCTNARGAGCCCACTGCNCGGGATCCGTCTTGGGTATGGGCYRTNCTGCRCTTACAATGGCCTGGCAGCGNGYRGGAGGACCAATTNCAACGGCANGTTTGGAYYTGGNCCTGGAACGGTAGATCTNACAATACGGCNGTYTCTAAGTAGGTTGGGTACGCANGCCCTAGGTCTCATGAGGAAGCYTTNNNNRAATGCGGATCACTCGTTGGNCNNNNNYTATTNCCAATGYCCGTGGATAGTTTGGTTGATGGTGTAGCTTGRAGAACGGTGGAGCCATTCCGGAGYTGYTAGGTTGGGGCGGGCGCCGCAGGCCAGGAACCTNGNTRCNYTACAGTGCNNCCAGNTAGCCTTAGARCRCYCTCTYTYGCCTGYTGARTGGGCTGCATTTGGTTACCTTTGGCTANCAANGAATCCTAGCGGANACAGCTAATYATCCTCGTCTARACTCAAGYAAGAGTNTAGAGCCGACGCGNNNNRTGTCAACACCRANGGGGCGAGGAAANAACGCCNNCRRCTACGAACGGTGGACATTCCCCTAGAAACAAGCGRYNGCGGAGGCTGAGCAGGCCTGTYTCCTCGATCTGCTCCYTTNNGTGNANRCCCCTGCNNNNGCTTAYGNCAGTRATTAGCNNACTNNGCCGTGCGGTGGGTACCAGGNGRNTCCTANYNCAYGCGAATGNTNGGCNNTNCCRNRRNCTAGCCGGCTGGTAGCTATGAACTGATATTATRTNNGNNCNTGCCCCGYYYGCANTACRGATACARCTAGCCCTGTACACAACGGAATTGTTCTTAGGCTYGCGAATACGGATAAGTTCGGARAAACGGNCGYCTTGGTCCGGTCNNNCACTAAAGTTGCAAATTGTNGTAACTNGCGGTAGCCATGGCATNTAAATTNNGAATAGCCCGCGGTGTAAAGNCGGGCCGCGTCGGCGAGAGCNNNYTTRCCGCGAGNNATAATCANNCGNTTAGATTATGTCTGAAGGAACGCACCCCTCTTAANAAAGGTCACACGCTGTGGCTCCACNTTYGGCRGCCGGGGRRTTAGGCNCGTCCGGGGGCCGATGAGTACTNGGGCCTNRGRACGTGAATGCNCNGACGATGGGGCCAGCCACCCCGATAAGTTCNNNNNNGGGGCRYCAGTYGNNNNGCTGCGARGNATTTCTATGGCCCRNCGGTAANNNGACGGTATTTGAAAAGACATAGGTRTGTGATCGGACTCCTGAAATGTCANNTAGGAATCGGNTAGCACTGACRYRTAAACATCAGGCGCATCGGTANCTCTCCGGGCGGCCAGCGCGAATRAAANNGGCTNGCCCCTGRACRAAGGARGGGAAAAACCTCAAGACCCTGRGGNCTCNNCGACCCTARTGAACATAGAGCRTCGCNNAGTTTGACATTTGGTTAACGGRNGCRRCCNTCCTCTGACYTACCNAGTGGCTTCCGCANNNCTTTGTACAGCCAGAAAGCGAACNCCCNNYNNCTCCCAAGTGGTTTTTCAAGTACCCTNYCYACRTNGCTYCRANGGAAATGCTCYGCYNATGCAATCGTGANCCCTATATCTGTTAACCAGGTGGRCACTGGTCTCGGNNNCGTAGTACTCGATATAARRYYRRGGRYCACTATGTNCAGGTGGACCGNATTAGYCTRCTTTANNGAGATGGCANGGTCTGTTTGGATATCGTCCGCTTGGTTAACTGCTCGAAGCCCATCCTCACGNRTCCCGATGAGTAACGGTGAGNCCGNGGATTCTCRCTGGACAACTNCCAANGCGTCCAGNNNATACTTCGTTCTCTTTCCATTTACGCTAGCCAATTTCGTAATGTGCCGGCAGCGTAGAGCTGGCACCGCAATGGATRTCTTGCAGAATGGCCCAAAYANCNNCGGCGCGTCACTATCGAGTNNCATTCACGGCGNCGACAGCTAGCCGACCCACCCAATAACTCGYGTCCNCNCGAGGCAGGCGGNNNNRRNACGGAGACGTTATGACGGGGTTTATACACATTCCACGCNNTTCNGNNRTNNTGAAAGGANGGTGNNNNCCCCCAAACCGGYATGGGGYGNNATTGGGGTACTCGCRGTCACCTNNNGAATACTCGRCYGCTARTCGGTGCANCGTGGCGAGGGCGCCTTGCTYTTATATTAACATTGGCAACGTAGATGCATGCNNAACTTCGNNNNAGGARAGAGYRAGNCCNTRGCAACGGGCTCTGCGAYNACATTAGYNACTTNNTGCGTCAAAGATTGATNGTNCGTGTCCNYYCYCNCGGCGNNAAGAAACTAAAAGGCYCRCTACGCANAACCNTGAAGAGTAANGCCTAATCTYCGGCGCRYNYRGNGCGGTCGCRNYAGYNRGTTGTAGGCGATTTGAGGACTANNNCNACTGCTAGARACRGCCGGTNYARGGAACCTGCTGGTAGGTTACRGYTTNATTAGCCCGCCCCGGAGCAATCNTNCTCGTCNTTAGNGNCTACTYCGGTGGCGTANNNNNNAYTANNNNCAYGCAGCRGCAGCAGCGTNTTACAATACTCTANACGCCTACATCGYCTCGGGCTAATACTTCTCCTCACTCCTGATCACCGGTGANCACTAGGCGCCNNNTCCNNGATAACCGAAATGGGCCTCCAAARGTCGCCATCTTACAAGACTCCTGATATTGTGCGGNNYTTTNTCCRYGTCAACNCTGAGCGTCGGTGNCGAYGYRCGACCGCYGNTTTRTRARCCRCACCACCGGGCTTGCAGGGNRGGAGGCACGGCRCYGTAACGCAGGTGTCGANCYGNATNAGCTAGGAGAATGGGACTGCAGACACGTGACCATATACCCNNNNYGGACGCNNGCCYYARGCAARGGTGTCCACACNGATCCCAGATCTGARARTCGGGCAACGCCTTCTAGCCGAAATACCCCCCGATCCGTGYNTAAAGNTACCACGCTNNCNRTNNNGGGACYYACCRGNNTTGCRNGGATTANAGCAACTTCGTTGCTCCGTCGCAGCGGTATTAAAGCCTYACRTCCNTTNNGGGAGNCNGRGTGAAAYATTCAGGTGCTGTCGGACCNTCYGNNNNRCRTCACGNGTGTAATANTCACNRNGNATGTGAGNNRAGNTCGRGCACCANTGTYNNNACCTCNNRTTTTCGGGTTAGCCATACAGYTYCNNAGACNNNGGCNGCTGTCAANCGGTAGYCYAYCCYNCTNRATGNNNNNCCAGGTGNNNNAAAAAGCNNAAGGYGNAATCATTCTNGTGTTGGTNRGACGARRAGAGTAATATNNNNCCTNTTTCGCAGRAATGGTCTGTGCCGGAGGAGTTATCACCAATAAAAGTGTCCGCGAGATGCGCTAYNTGCCCTTTNTTAGTTTACACTGCAYAATAAACTNGTCGCTGCCTCATCCCGGCCGTACGCAGTNGNYYYYAGAGATTAATTCNCCGRCTACCCCGACGCCAGNCTCGCACAGCCTGAGCTACTGTATCTAAATGGACTATTTCCNYTGTNGYNGNNNNRYYCCTCTNAACGTCRYCGCTATAACACACAGGCAGTTTGAGAAATCTTACRYGCGGTAANCCGTAGGGCNAGGCNACACAGCCTNAGAGGCCANCCTATTGCAGATAAGCCGCNCNGTCTCGNYCTRRNTCGNNNNCATCGTGRACGGACCGGTYCNCAGGNGGCGTGCCTGRTCGNNACCGCGACGGGNNCCTGCGACYTACNNNTCCGCGCCCGNGYGCCGTGGNCRTTCCGTRNAACTGATCCAGGGACTTGGATGCCCTTGTAAGCACNNTGGAATAGATTGTACCGCGCCACACGRCAACATAGNACGCNAGCGGTACCTTGTCTTAGAGGTCAAGTTTYCTCGTNTTGCCCNCRGGCAATCATGCGCCACAAANNNGGGTTATGGCAGCTCGAATCAGGTGCGGYCTTGGNTCCCAAAAACTGCTGTCATAAATGCGTACGTCGAATGCNCCGCGGCATTGCTTGCGGGTACNNGCGNYCGNCATTGYGTTCATGGTGTTATNAGCTCNCCTTAGGYGYGGACCAAAATCGCCACGATCATGNGNAGAAGRCGCGARTGTGTCCAACGCGTTAGCNNGGANYTTGNAAGCGCGGCTATTACGGYTCACTCCTATACAGGTCTARGTACGGCTTCACACTGGCTGTATATCTGAGCTACCGCCGTYGCGCAAATTCAYGGGGGGGGAGCCAACACTTTGTTGNNGCGGNCTCTCRNGNNNACTAAATNGTGGCGTCTYNCTAATCGAARGAGAATTNTGTCACCACGGAGGTCGCCRGCATGRTGTCGCTTCACTCTTNATAYNNACTGCAATNTANCGANNCCAGGCGYCGTAYRACANANARATGANGAAACACGATACGCAGTACGGGACGACGTAGCCACCACGGACCCTGATCGTGTTTCGTANYRACACRRCNTCAGTCTCTGACTTTACCCGGNNGCAGACTCCTNAGTCAGCNGGNATTACCGGCCGAATAAGCCGNGNGTCGGACAAACCCGAGTCTAGCGGGTCGCGCACNNCYCGRAGGGNACCACACATNNATNTTTCACGCRGGGCGGCRGCGNCAAAACACTCNRTCCCCACCTGNGGTCAGCCCTANRAYCTAAGGTAAGCNNNATGNRCGATTGGGAGGCNGAATCCCANYRTCCRGTCCTATTCYYYRAACCGACCRTAAAGNACGTGRRGAATTYGTTAACTTGACCYGCTAGRCCRCRTGTTGGTCCACARCYRCNTCGGTCTAATGRCRYTTNNNRNGYTTGCCTCCAAGCGACGGGAGTTAGTTGGCCCRCCCTCRRTNTGNYAYAATCTACAGTAAGCCGATGGAANNCACTNGYCAGCATTTGCAYNCAYATGNGTCACTGATACTNNNNNGNCNAACTGGCGGANYRGARGNTCTNGATCTRTGATGRNNANYCGTYRCAGATRYNGCYGGNTGTYRTGTGNCRYGGGTTGTGCCCANNTTGTNNTCCAAGTNGYARRTAGACCCTNACTTAGGGCGTCGAGAYAYAATCGNAATCGCYTCARGTCCTAGRAACGTGGGNTNCTGATCGCTNRAYCCCACCTCTCAAAGCTAYCGCNGCGNNTYCTTAAAGACTGGCTAGNTNGAACCCTAGATAACGGGGGYGTTNRRGGACTNNRGAAGGTCNTCCYYGGATTGTNCGCATGANNCCAARACCACTTTGTGGCANGGGNNNAGRRCCYCNRGGCTGAGCCCNAGGCCGTCCGCAAACNGGCNANNTGGGCCAGAGGCTGANAGGGGCGCAACRYARCATGNCTYTACTAAGTACACCGAGCTATTYTAGCAGGAGCCGGTGTGCATRCCCGTACCATTCGCYTGAGGCTTGTCTGTCGGTAAAGCCATGGCGCCAACTRGNNAGYGNNTGGTACTTTGTATAANNNCCNNTTCCGGGAGCATCNGATCGCTCGNNCCCGAGNTNNGCCATTRGTTAGRCATTGNNNNTGNNCGGCAACGGCNNGGTGTCACGACCTCGGGNCGNYNNNTGGCANACTTTTTAGATCCGACCCGATTGNCCGRNAYRNNGGYCCCAGCATYCYYNCRCTGNNATTTATCGNNNNGACGATCGGGCAAAGCATGTCCTGCTGGYGGAGTCCGTTRRTGNCCRGATCAAGTNACCAGATTAGGGANNTAGCATCCTACARYGGCGGATTTTCGCATTCAGTCYNACGCGGTCTCGYAGTGCACATT

Gv10-F-02 AYGNNTTACTCGTCGCCATANNGTCNNCCNNNNNCGTCGACNNAGACGGNTCTAGRRGGAGTACCCACTGACCGTGATCTACTGGCYATCACGCCTNTTGTCTAAGGGNNNCNNTGCTCGRGATCNNNCNTGGRTATGGGCNNNGNNNNNNNNACAATGGCCAGGCAGCGTGCAGGANNNCNNNANGTACAGACAGGNTNGNACCTGGCTTNGGAACGGTANNNNNNACAATNNNNNAGTCTGTAARTANNNNNGNTACGNNNATCNNNNNTCNCANNNNGARRTTYTNNNCNAACRCRGATNNNNCGTTNNGCNNNNNYYATTYCCNATGCCCGTGGATAGTTTGGTTGATGRYNNNGANNNNNNAACGGNGGAGCCATTCTNGAGYTGYNNGGGCGGGGCRGGCGCCGCAGGCCANGAACCTNGRTRCNYTACATTGCCNCCAGANRNNNNNNNNGCACCCTCTCTTNNCTGYTGARTGGGNNGCRTYTGGTTACCTTTGGCTGYCAAYGANNCCTNTCAGANACAGCTANTCANCCTCGTCTNNNNTCAAGTAAGAGTCTAGAGCNNACNCAYACGRTGTCNACACCRACGGGGCGNGGAAAGNACGCCTCCGGCTACCANNGRYRGACATTCNNNTAAAAACNAGNNNNANCTANGNNTGARCAGNCCAATTNCCTCRATCTGCTCCCTTATGTGGAAGCCCNNNCCNCNGCTTNCGNCAGTNAYTAGCGGNNTGCGCCGNGCGGTGGGTACCAGGTGAATCCTAGTGNNNGCGAATGGNNGGCACNACCNNGAACTAGCCGGCTNNNNGCTANGAACTGATNNTNTNNGAGTACATGCCCCGYTNGCACTNNNNNNNNNNNNANNNNTGTACNCAACGGAATTGTTCNNNNGCTCGCGAATTTGACTGNNNTCGGARAAACGGCCNNCNTGAGCCGGTCNGTCACTAANGTTGCAAATTNTAGTAANNNNNNRYAGCCATNNCATATAAATTNGGAATANNNNNCGGTGTAAAGNNNNNCCGCGTNNNNNNNGGCNNCCNTGCTGCANNGGATANNNNNNTGATTAGATNNNNNCTGAAGGAACGCACNCYTCTTAACNNAGGTNNNANGCTGTGGCTCCACYTTNGRCRGCCGGGGGANTAGNNNCGTCCGGGGGCCGNTRAGTACTNGGGCYTNNGAACGTGAATGCCCARACRATGGGGCCNNNNACCCCGATAAGTNCCYANANGGGGCGCCAGTTNNNNNGCTGNRRRGNATTTCTATGGCCNRNCGGTAACGCGACNNNNYNNNNAAAGAYTTAGGNANGTGATCGGACTCCNGAAATGTNNTATAGGNANCCACNNGCACTGACANNYANNNATCAGGYRCATCGGTANNNNTCCNNNNGGCCNNCGCGAATGNNATGGGCTNNNCCCTGGAYRAANNNANGGAAAAACYTCGGGATTCTGAGGNCTCTGCGACCCNNRYGAACATAGAGCNNCGCCYAGNTTRACNTTTGGTCTCTRNATGCGGNNNTCCTCTGACCTNCCCAGTGGCTTCCGCNNNNCTTTGTRCRRCCAGAAAGCGAACCYCCCGNNNCTCCCAAGTGGTTTNTCAAGTANCCTAYCYACATCNCTCCRACGRAAATGNTCTRYCTRTGCAATCGNGAGCNNNATATCTATTAACCAGCTCCNNACTGGTCTCGGRNTCGTAGNACTNNNNNTAANNYYGGNNRYCNCTATGNGCAGGNGNAYCGNNTTAGNCNGCTTTACTGAGNNNNNNYGGTCTGTTTGGATAYCGTCCGCYTGGTTAACTGCTNNNANCCCGTCCTCACGNGTCCCGNTGRGTGACGGTNGGTCCGGGGANTCNCGYTGGACAAYTTCCAAAGCGTCCAGNRRNTANNTCGTTCTCTTTYCANNTACGCTANCCAATTTNNTAATGTGCYGGYRGCGTANNNCTGGCACCRCAANGGRTRTCTTGCAGNATGGCCCAAACANCNGCGGCGCGTCACTATCGAGYCCCATTCNCGGCGNCNNNNNNNNNNNGACYCACCNNATRRCTCNNNNCCCCACGAGNCANGCGGNNNNAATANNGAGNNGTTATGACGGGGNTTNNNCACATTYCNCGCGGNYNNNNNNNTGCGCAAGGGCGNTGCNGNCYCCYARACCNGTATGNGGCGTAATTGGRGTGNNNNNNGTCNCCTNNNGNATACTCGACCGNNNNTCGGCNNANCGTNGCNNGNNCGCCTTGCTYNNGTATTNNNNNTGACTNCGTNNNNNCATGTCNANNTTNGCNGNRRGAAAGRGTGANNCCGTGGCAACGGGCTCTNCGACCACATTAGCTGCYTNNTGYGTCAAAGATTGANNGTNCGTGTCCNNCCYCNCGGCGCGARGANACTNNAAGGCCCACTGCGCAANNNNCTGTCAAGTAAGGCCCAATCTCNNNNNNNNGCRGNGCGGNNNCGNYAGYNRGTTGYNNNCGATTNGAGGAYNANCRCANCTGCTAGARANNNNNNNTNYARNGNNCYYNNNNNTAGGTTACGNCTTAAYTAGCCCGCTCCGGAGCAATCGTCCTNGTNGTYNGGGGCTACNCYRGTGGCGTANNNNNNNNTAGACCYACGCAGCAGCGGCAGCGTCTTACAATACTCTAGANNNNNACANNNNCTNNNNCTNCTCNNTCNCCANACTCCTGANNAYCGGTGRNCACTAGNYGCCNNNTNCTGNATAACCGAAATGGGCYTYNAARGNTCGCCATCTTACAAGACNCCTGATANTGNGCGRTGTTGNNNYCATGNCANYTCTGNGCGTCGGTGACGAYGCRCNACCGCCGGTTTATGAACCGCACCAYCGGGCYTGCAGNGNRGGAGACGCGGNGCCNTAANGCAGGTGTCGNNCNGCNTNAGCNNNGRRAANNNNNCCGCGGACACNTGACCATAGACCCCGCACGGACGCTNGCCYCNANNGRNGNNNTCNACNNCGNNCCCGGGCCTGANANTCGGGCAACNCCTTCTAGCCGNNNTACCCCCCGATCCGTGYNTAAAGNTANCACGCNTANAGTAATGGNACYYACCGGGTTTGCNNGRATTNNAGCAACTTCNTNGCTNNNTCGCANNNGTATTNNNNNNNNNNNNNNNCTGGRRGAGACGGNGTRAAATANTCRRNCRCNGTCGGACCGTCNNGCACGCRTCACNNGTGCAATANTCAYNRGGNRTNTGAGANNNGTTNNNGCACCAGNGTCTTNACCTYGANNNNTCNNGTTAGCCATNCNGCTCCRCAGACRTTGGCNRCTNNCAAGCGNTAGTCNNNNCCNCTANANGCGNNNCCAGATGACGNGAAAGCCTCAAGGTANAATCATTCTCGTGTTGNNNGNACGARNAGAGTAANNTNATCCCTATTTCNCNGGAATGGTYCRNNCCGAAGGAGTTATCACCAATGAANNTGTCCGCGAGATGCGCTACNNGYCCTTTGTTAGTYTACACTNCATAATAAACTNGTNGCTGCCTCGTCNYGNCNGTACGCANNANNNNCCAGARNYNAANTCGCCGGCTNCCCCGNCGCCAGCNNNGCNCAGCCNGAGCTAYTNNNNCTAAATGGACTATTTCCATTNNNNYNGRYGRGCCCCTYTTANTGTCGTCGCTATAACACACNGGCAGTNNGAGAAATCNTANNNGCGGTAANCCGTAGGNCNAGGCTACNYRNNNNNAGAGGCCAGCNNACTGCAGATGAGTNNNNCAGTCTCGGCTTAAATCGGGNNCATCGNGGACGGNNNNNNYCGCAGGCGGCCTGCNNGATCGGAACYRCGTCGGGNNCNTGCGATNTANGCGTCCGCGCCCGGGTGCCGTGGYCRTTCNNNRNANCNGATCCTGGGACTTGGATGCCCTTGTAAGCACNNNNRAANAGATTGTACYACGCCACACGGCGGCNTAGNACGCYNGCGGTNCCTTNNCTTAGNGRTCNNNNNNNNTCGTANTGCCCYCAGGCAANCNTGCGTAAAAGNATGGGGTYNNGGTNGCTCGAATCAGGTGCGGNNNTGGTNNNNAAAANCTGYTGNNNNNNNTNCGTACGTCGAATGCACCGCGGCANTGCTTGCGGGTNNNNGCGGCNNNCATTGYGNTCATGGTGTTANGAGCNCTCNNNNNGCGCGGACYNAAATCGCCACGATCATGTGCAGAAGNCGCRNATGTGAGCANNGCGTTAGCACGGACTTTGNNNGCNCRRATATTACGGYNCATTCCNATNNAGGTCNAGGNANGNNTTCACACTGGNTGTANNNNNNGGNGACCNCCGTYGCGCAAATTCACGGGGGGGNAGCCAACACTYTGTTGTCGCGGGTNCNCACNGTTACTAAATTGTGGCGNCNNNNNNAYCGAANGAGNNTTNTGNNACCACGGAGGTCGNCAGCATGATGNNNCTTCACNCTTCNTNCGTNTNNNANTNTNACGNNNCCAGRCGNCGCACGNNNTANARATGACGAAACAANNNNNNNNGTACNGGACGACGTNNNCACCACGGRCCNNGAYNGTGTTTCNNRNYRACACAACATCARTCNNNNNNTTTACCCGGRNNNAGACNYCTCAGTCAGCTGGGATTNYTGGCCGAATAAGCCGAGTGTCNGRCNANNNCGAGTCTAGCGNNNNNNNNACTCCYCGNAGGGCACCACACATCNATNTTTCACTCNNGGCGGCNNNGCCANNNNNNTTNRNCCCCACCTRNGGTCAGNNCTNTGACNTAAGGTNAGNNNNATGNRCGATTNNRANGCYGAATCCCAANNTCCNGTTTNATTCTCTAAACCGACCATAAAGNNCRTGRRGAATTYGTTAAYTNGACCNNNNNGGCCGNNTGTCGGTNNNNNNNNRCCNNAGNCTNNNGGCNNTTNNNAGGTTNGCNNNNAAGCGACRGGAGNNNGTTGGCCCACCCTCRRTNTGATACAATCNNNNGTAAGCCGATGNAAAACNYNCGCCNNNATTTGNNNCCANATGCGTNACTGATACTTCNNNGTCTAACTNNNNGAANNGAGGTTCTGGATCTATGANGAATAGCNRTCANNNNNRYTRCCGNNNNNNNTGTGNNNNNGNNNNYGCCCAGATNGYCNTCCNNGTTGCAAATANNNNNTNNNNTAGGGCGTCRARAYANAAGCGTAANCGCCTCNGGTNNNNRGAATGTGGGATCCTGATCGCTNNACCCCACNNNTCAAANCTNCCGCTACGTCTTCTTAANGACNNNNTAGYTTGNNNCCTAGATAAYGGNNGTGNTAAGNATNNCTGGAAGGGCNNYNNNNNATTGTNNACNTGRTCNNNAGACCACTTTNNNGCATGGGGGCAGGGNNNCAGGGCNNNGCCCTAGGCCGTCYGCNAACNGGCNANNNNGNNCNNAGGCTGNNAGGGGCGCAACACARCATGNNATTACTNAGNACACYGAGCTNTYYTAGCAGGRGCCGGTGTGCNNACCCGTAYNACNCGCTTGRRGCTTRTCTGTNNNNAAANNNNAAANGTCAGCGAGAGNNYGNNTGGTNCTTTGTATAANNGCCCNTTCCGGGAGCATYGGATCGCTYGGNCCCGAGNTNNGCCATTAGTTANNCATTGYCTTTGCACGGCAAYNNNNNGGTGTCACGACCTCGGGACGCTNYNNNGCANACTTTTTAGATYCGACNCGATNNNCCAAGATGGAGNCCCCAGCATCCTTNCRCTGNNATTTANNTTGAAAGCGANNNNNCAAAGNATGTCCTNNNGNNRATRNNNNNTGRTGNCCRGANNNNGTTRCCAGATTTTGTACCTAGCATCCTACAGTGGCGRGTNNNYRCATTCNRTCNNACGCGGTCTCGYAGTGYRNNNN

Gv10-F-08 ACGNNTTACTCGTCGCCATNNNGNCNNCCGGAGYCGTCRACNNNGACGGGTCTAGNAGGAGTACCCACTGACYGTGATCTACTGGCTATCACGCCTTTTGTCTAARGRGYCCACTGCTCGRGANCCGTCTTGGNNATRGGCNATRCTGCGCTTACAATGGCYNGGCAGCGTGCAGGACGACTATATGTACAGACAGGTTTGGACCTGGNCCTGGRACGGTANNNNTYACAATACGGCAGTCTNTAAGTAGGTTNGGTACGNANAYCCTAGGTCTCATGAGGAGGTTCTNNNCRAANNNNNATCAYNCNYNGGNCNNNNNYYATTCCCNATGNCCGTGGATAGTTTGGTNGATGRNNTAGATNGAAGAACGGTGGAGCCATTCTGGAGNNNYTAGRGYGGGGCGGGCGCNNCAGGNCAGGAACCTAGGNACNTTACATTGCCNCCAGNNRRYCYTAGAACGCTCTCTCTCTACTGNTGANTGGGCTGCATTTGGTTACCTTNNGCNNYNAATGARCCCTAGCGGANNCAGCTAANNATCCTNGTCTAGACCCGTGTGAGAGNNTAGRGCNNACNCGYACNGTNTCNNCACCNACGGGGCGAGGAAAGARCGCYTCCGGCTACNAACGGTGGACATNCNCCTNAAAACAAGNNNNCNCTAAGGNTGARCAGGCCAATYTCCTCRATCCGCTCCCTTATGTGGAAGCCCCYRCCCCNGCTTAYNNCNGTANCTAGCGGACTGCGCCGTGCGGTGGGTACCAGGAGGCTCCNATCTCATGCGAATGNYNGGCACNACCGTNRNCTAGCCGGCTNNNNGCTANNAACTGATANTATGTNNGTACATGCCCCGYTCGCANTACAGATACANCTAGNNNTGNACANNACGGAATTGTTCTTAGGCTCGCGAATACGGATAAGNTCGNAAAAACRGNCNTCNTGGTTTGGTCANNCACTAAAGTTGCAAATTGTAGTAACCCGCRRYAGCCATGGCTCNTAAATTNGGAATANNNNNCGGTGTANNGGCGRGCCGCRTCGGCGAGNGCCGCCTNGCYGCAAGGGNTNATCAGACGATTAGATTATGNCTGAAGGAANGCACYCYTCTTAACAAAGRTCNNACGCTGTGGCTCCAYYTTNGGCAGCCGGGGGATNNNGCACGTCCGGGGGCCGATNNGTACTAGGGCYTNNGAACGTGAATGCACGNNNGATGGGGCCAGCCNCNNCRACAARTTCCYACARGGGGCRYCAGTTGGCTTGCTGCRRGGTATCTYTATGGCCCGACGGTAACGCGACNNNATTTGNAAAGACATANGYAYGTGTANNNNNNNNTGAAATGTCATNTAGNAAYYNACTAGYACTGACNCGTACGTRNCAGGCGCATCGGTATCTCTCCGGGCGGCCRRCGCGAATGAAATNGGCNNGYCCCYGRNCRAAGGNAGGNNNNAACYTCRRGACYCTGAGGNCTNGCCGACCYTAGTAAACNTAGAGCATCGCCTAGNNNGACATATGGTCTCTRNGAGCAANNNTCCTCTGACNTACCCAGTGGCTTCCNCAGNACTTTGTNNNNCCAGAANGCGAACCCCCYGCACCTCCCAAGTGGTTTTTCAAGYACCCTNCCYACGTTGCTNCAACGNAAATGNTCTATYNRTGCAATCGTGRGCNNNATATCTATTAACCAGNTNNGCACTGGTCTYGGNNNCGTARTACTCGNNNTAAGGTCGGGGRYCACTATGTGCAGGTGGNNCGTATTAGYCYGCTTNNYTGAGATGGCATGGTCTGTTTGGATATCGTCCGCNTGGTTAACTGCTCNANNCCCRTCCTCACGNGTCNNNNTGRGYGACGGTGGGTCCGNGGATTCTNRCTGGACANTTTCYAAAGCGTCCAGNGGNTANNTCGTTYTCTTTYCATNTACGNNNNNCAATTTCGTAATRTNYCGGCANCGTAGAGCTGGCACNNCAATGGATATCTTGCAGAATGGCCCAAACAGCAGCGGCGCGTCACTATCRAGYCCCNNNCACGGCGGCGACAGCTAGCNNNCNCNCYCRATNNCTCGCGTCCCNACGAGGCAGGCNGGACGAATNCGGAGACGTTATGACGGGGNTTANACACNTTCCACGCGGGCNNNNGATTRTGAAAGGRCGGNNNNNNCCCCCARNCCNGTATGGGGCGTAATTGGGGTACTCGCRGTCACCNNNGGNRTACTCGACCGCNAATCGGCNNNNCGTNGYNAGGGCGCCTYGCTTYTRTNNNAACATNGGCAACGTAGATGCATGTGGAGCTNYGCGGCRRGAAAGNGCRRATCCGTGGCAACGGGCTCNGNNAYNACANNAGTTAYCYNNTGYGTCAAAGATTGNYYNTNCGTGTNYNNYCYCNCGGCGYGRAGAAACTNNATGNCYCGCTACGCATANGCCTGNNRAGTAANGCCTAATCTCNNNNGCACNYNGNGNGGTCGCRATAGCGGGTTGYAGGCRATTYGANNAYTANCACTACTGCTNGANNCRGCCGGTACAGGAAACCCGCTGGTAGGTNACGACTTAAYTAGCCCGCTCCGGAGCARNNGTCCTCGTCGTTAGNGNCTACTCYRNTGGCGTNCATCGCATTAGACCCAYGCAGCAGCGGCAGCGTCTTACAANACNCTARACGCCYNCATCGTCNNNNNCTAATACNTCTCCTCACTCCNNATCACCGGTGRNCACTAGGCGCCTGTTACTGGATAACCGAAATGGGCYTYCAAAGNTCGCCATCTTACAAGACYCCYGATATTGTGCGGTGTTNTATCCATNNAAACNYTGNGYNTCGGTGAYGNYGCRCGNNCGCCGGTTTATGAACCGNACCAYCGGGCTTGCNNNGNRGGAGACGCGGCACTGTAACGCAGNNGTCGNNCTGYNTYAGCCGAGRRAATGGGACCGCGNNCNNNTGACCNNNNNCYNCGCACGGACGCYGGCCYTCAGCGRRGGNGTCTACATCGATCCCRGRYCTGAAAGTCGNNCAACNCCTTCTAGNCGAAATACCTTCNGANCNNNGCATANAGTTACCACGCTNNNCATTNNGGGACNNACCAGCTTTGCGAGRATTANNGCAACTNNRTNGCTCCGTCGCAGCGGNATTNNAGCNTCACGTCCNTTRNGGGAGACGARRTAAAANATTNAGGYRCYGYYGGAYCRTCYNNNNYRYNTCACGNGTGNAATANTCAYTANNNNNGTGAGAGGAGTTCGGGCACCANTGTCNNNACCTYGARTTTTCGGACTAGCCATACAGCTCNAYNGACNTTGGCAGCTNNNAAGCGNTAGNCYATCNNNNTAGNNGNNACTCCAGATGACGCGAAAGCCYCAARGTNTAATCANYCTAGTGTTGGTARRACGANAAGAGTAANNNARTCNNNNNTTCGCAGGNTTRGTYCGTGCCGRAGGAGTTATCACCAANNANAGTGTCCGCGAGATGNNYTANNTGCCCTTTGTYAGTYTACAYTGCATAATAAACTAGTNNNNNCNNCGTCCCGRCCGTACGCNNNAGATCTTAGARNCTAATTCGCCGGYTACCCCGACGCCAGCCTCGCNCAGCCTGAGCTATTGTATCTAAATGNACTATTTCCATTGYTRCGRGYGRNNCYNTCTNANCCTCGTCRYNNTAACACACAGGCNRTTNGAGAAANYNTACACGCGGTAAACCGTAGGGCTAGGCTACAYRGCCTRANRGGCCATYCTATTGYRGATGAGTCGCNCAGTCTCGGCYTRRANNNGGTTCAYNGTGAACGGACCGGTCCNCAGGNGGCNYRYCNGATCGNNACCGCGTCGGGCCCCTGYNACYTANGCGCCTGCGCCCGGNCGCCGTGGCCRTTCCGYANAACTGATCCTGGGACTTGGATGCCCNTGTAAGCACNCTGGAATAGATTGTAYCACGCCACANGGCAACANAGGACGCYNNNGGTACCTTGTCNTAGAGGTNAAGTTTYCTNNTATTGCCCCCAGGNNNTCATGCGTAACGGGATGGGGTYATGGTAGCNCGAATNAGGTGCGGTCTCTGTTCCCAAAANYTGCNGTCATNNATGCGYAYGYNNNNNGCACCGCGGCATTGCTTGCGGGTNNNNGCGNNNNNCAYTGYGTTCATGGTGTTATGAGCTCTCCTTAGGCGCGGACCGAAANCGCCACGAYCATGNGCNGAAGGCGCRNATGTGTCCAAAGYRNTAGCNNGGANNTTGNANGCGCGRNTATTACGGYNCAYTCCTATNCAGGTCTAGGTAGGGCTTCACACTGGCNNNNYNYYYGGGGGNCCGCCGYCRCGCAAATTCACNNGNGGGGAGCCAACACTTTGTTGNNGNGGGCYCNCGAGGTTACTAANTTGTGGCGTCTTYCTAATCGAAAGAGARTNCTGNNRYNNNGGAGGTCGCCGGCATGATGTCGCTTCACYCTTNNTANGTACTGCANNNNAACGANNCCAGGCGCCGCACGACANAAAGATGANGNAACAAGATACGCAGTACGGGACGACGTAGCNACYTCGGNCCCTGATCGTGTTTCGTACTGACACANCNTCAGTCTCTGACTTTACCCGGNNGCAGACTCCTCARTCAGCTGGGATTTCNNGCCGNATAAGCCGNGTGTCAGNCNAACCCGAACCTAGCGAGTCGCGCACTCCYNGAAGRGYRCCACNCATNNATNTTTCNNNCAGGGCGGNAGCGCCAAAACACNCANNCCCYACCTRTNNNCAGCCCNRNGAYNNAAGGTAAGCCCCATGNRCGATTGGGAGGCNGAATNNCAAYRTCCRRTYYTATTCTCTAAACCGACNATAAAGTACGTGAAGAATTTGTTAAYTTGACCTGCTAGGCCGCATNTCRGTACACARCYGCCNCGGTCTANNGGCACTTNNAAGGTTTNNCTCCAAGCGRCGGGAGYYNGTTGGCCCACCCYCRRNNTGATACAATCTACAGNAAGCCGATRRAAAACACTCGCCAGCATTNGCACCCACNNNNNNCACTGATACNYCYANGTYAAACYGGCRGAAYRGAGGTTCTGGATCTATGATGAANANYCGTCANNNNNNNNGCNGGCTATYRTGTNCCGCGGRYTGTGCCCAGATGGCTCTCCNAGTNGNANATARACCCTNAYTTRGGGCGTCRAGAYATAAGCGYANNCGCCYCAGGTCCTAGRAACGTGGGATCNNGATCGCTCAACCCCACCTNTCAAAGCTNNCGCTACGTCTTYNNNACGACTGGCTACCATGAACCCTAGATAACRGGGGTGTTAAGGATNNCTAGAAGGGCNNCYTTGGATNGYNCRCATGATCCYNANACCACTNTGTGGCANGGGGGCAGAACCCYTANGCTGAGCCCNRGGYCGNCCGCANNNNGGCGAACTGGGCCAGAGGCTGNNAGGGGCGCAACRYARCATGNCNCTACTAAGTACACYGAGCTNTNYTAGCAGGAGCCGGTGTGCNNRCCCGTACCAYNCGCNTGNNGCTTRTCTGTCGGTAAAGCCANRRCGCCAACTAGANNGTGNNTGGTACTTTGTANNNNNNCCCTNNNNNNGAGCNNYNGATCGYTCGGTNCCGNNATCAGCCATTRGTTAGGCATTGCCTTTGCACGGYAAYNNNNNRRTGTCANGACCTCGGGNCGGTATGTGGCAGACTTTTTAGATYCGACCCGRTTRYCCGAGATGNNNNCCCCAGNNNNNNTNCRCTGGNNNNTATCNTRAARRCGATCGGGCAAAGNNTGTCCNGCTRGCGGAGTCCGTTRGTGNCCNRANCRAGTGACCAGATTNNGNANYNNGYATCNTACARYGGCGGATTNTCGCNTTCAGTCNNNCGCNGNCTCGCAGTGCACATT

Gv10-F-10 AYGATTTACTCGTCGCCATANNGNCCGCCGGAGTCGTCGACCTAGACGGGTCTARGAGGAGTACCCACTGACYGTGATCTACNGGNTATCACGCCTCTTGTCNNRGGANNNCACTGCTCGRGRTCCGTCYTGGRTATGNGCTATNCNGCRCYNACAATGGCYNGGCAGCGTGCAGGAGRRCCRANNNTACAGACANGTTTNGNYYTGGCCCTGGAACGGTAGACTACRCAATACGGCNGTYTNTAAGTAGGTTGGRYACGCANATYCTAGATCTCATGAGGARGYTYTTGACGAACRCRGATCACYCRYNGGGYATTGATNATTCCCAATGNCCGTGGATAGTTTGGTTGATGRTGTAGNYTNANNAACGGTGGAGCCANTCTRGAGCTGCTAGRNYGGGGCGGGCGCCGCNGGCCAGGAACCTNGRTRCRNTACANTGCCNCCAGATNNNNNTAGAGCACCCTCTNTTGCCTGYTGARTRGGCTGCRTYTGNTTACCTTTGGCTRTNAAYGAAYCCTANCRGANACAGCTAATYATCCTCGTCTAAACTCRNNTAAGAGTATAGRGCYGACNCAYACGRTNTCAACACCNACGGGGCGARGAAAGAGCGCTTCCGGCTACGAAYGGTGGACATTCCCCTAGAAACRARTGNNNGCTAAGGCTGAGCAGGCCAATCTCCTCGATCTGCTCCYTTRTGYGGANRCCCCYRCNGCCGCTTAYGACNGTACTTRGYGGACTGCGCCGTGCGGTGGGTACCAGGNGRNTCCTANCNCATGCGANTGGCNGGCACTACCGTAATCTAGCCGGCTNNTAGCTAGNAACTGATATTNTRTGAGTRNATGCCCCGYYCGCAGTACAGATACARCTAGCCCTGTACACAACGGAATTGTTCTTAGGCTTGCGAATTTGACTGAGTTCGGNRAAACGGNCGYCNTGRNCCGGTCAGYCACTAAAGTTGYRANTNGNAGTAACTCGCRNNAGCCATGGCNYATAAATTAGGAATAGCCCGCGGTGTARRGNNNACCCGCGTCGGYRAGGRCCGNNTTRCYGCAAGGGATANTCAGACNNTTNGATTATGTCTGAAGGRACGCACYCYTCTTANCRNAGGTNACACGCTGTGGCTCCANCTTYGRCRGCCGGGGGATTAGGCNCGTCCGRGGGCCNATRAGTACTAGGGCYTTAGRACGTGAATGCCCAGANGATGGGGCCAGCCACCCCGAYAAGTNCCYACARGNRGCGCCAGNNGGCTYGCTGCRRGGTATTTCTATGGYCCGACGGTAACGCGACGGTNYTTGAAAAGAYNYANGYRTGTGATCGGACTCCNGAAATGTCRNNTAGGAATCNRCTAGCACTGACNCGTANGTATCAGGCGCATCGGTATCTCTCCGGGCGRCYNNCNNNNATRAAAYNGGCTYGCCCCTGANCGAAGGAAGGGAAAAACTTCGGGACCCTGAGGTCTCNNCGACCCNAGTGANCATAGRGCRTCGCCCAGTTNGACATTTGGTCTNYGNATGCRGCCRTCCTCTGACTTACCCAGTGGCTTCCGCNGGACTTTGTACAGCCAGAAAGCGAACCYCCCGCACCTCCCNAGTGGTTTNNCAAGTACCYTNCCYACGTTGCYNCRAYGNNNNTNNTCTGCTAATGCAATCGNGAGCCCTATATCTATTAACCAGCTCCGCACTGGTCTNGGRNTNNTAGTACTCGATNTAANNTCGGNNAYCAYTATGTRCAGGTGGACCGNATTAGYCYGCTTTAYNGAGATRGCAYGGNCTGTTTGGATAYCGTCNNNTNRGTNNACTGCTCGAAGCCNRTCCTCAGGAGNCCCGRNGAGTGACGGYGGGTCCGNGGATTCTCGCTGGACAATTTCCAANGCGTCCAGAGRATANNTCGTTCTCTTTYCATTTACGCTANCCAATTTCGTAATGTGCYGGCAGCGYAGAGCTGGYACCGCAATGGATRTCTTNCAGAAYGGCCCAAACANCNGCGGCGNGTCACTATCRAGYCCCNNNCACGGCGCCGACAGCTAGCCGACCCACYCRATGGCTCGCGNCCCCACGAGGCAGGCRGGATGAATACGGAGACGTYATGACGRRRTTTATCCACGTTCCACRCGGTTCAGTNNNTATGAAAGGNCGGTGNRGRCCCCCARACCGGNATGGGGYGTAATTGGGGTAYTCGCANNYACCTRNGRARTNCTYGACCGCNAAYCGGCRNANCGTGGYGAGGGCGCCTTGCTTTTRTATTAACATTGGCNACGTAGATGCATGYNGAACTTCGCAGTARGARAGAGCRAATCCRTGGCAACGGGCTCTGCGATAACATTAGTTACCTRNTGCGTCAAAGATTGANNNTTCGTGTCCCTCCCCGCNGCGNGAAGAAACTAAAAGGCCCGCTGCGCAAAAGCCTGNAGAGTAACGCCTAATCTCCGGCGCACNYRGNGCGGTCGCRATAGCGGGTTGYRRGCGANTNNRNRAYTARCACNACNNCTAGAAACNGCCGGTNYARGNAACYTGCTGGTAGGTTACGACTTAATTAGCCCGCTYCGGAGCAATCNYNYTCRTCGTTAGNGNCTACTCNNNTGGCGTACATCGCAYTATACCCATGCAGCAGCAGCAGCGTCCTACAAGACTCTARACGCCTACAGCATCTCGGGCTANTNCTTCTCCANACTCCTGATCACCRGTGRNCACTAGGCGCCTGTTNCTGGATANYCGAAANGGGCYNNYAARGGTCGCCATCTTACNNNACNCCCGATATTGTGCGGTGTTTTATCCATGTNAACTNTGNGCGTCGGTGACGACGYGCGRCCGTCGGTTTATGAACCGCACCATCGGGCYTGCAGGGNRGGAGNCNCGGCRCYNTAACGCAGGTGTCRACCTNTNTYAGCYRRRAGNRTGGGACCGCGATCACGYGACCATATACYCCGCACGGACGCYGGCCYTAAGCRNAGGTGTCNACAYCGATCCCRGRYCTGAGAATCGRRCAACGCCTTCTAGCCGAAATACCYYCCGATCCGTGCATAAAGTTACCACGCTTACCGTAATGGGNCCCACCGGGNTTGCNNGRATTAAAGCAACTTCGTTGCTCCGTCGNANCGGTATTAANNYCTYACRTCCNTTRNGGGAGACNGNGTGAAAYATTNAGNYGCCGYYGGACCGTCYGTCCTGYATCACGNGTGYAATANNNNYTAGGNATGTGAGANNAGTTCGGGCACCAGNNTCTTNACCTCNNNTTTTCGGRYNAGCCATACNGCTCNACNGACRTTGGYAGNNNNNAANCGGTAGCCCAYCCYNCTNRATGCGACTCCAGRNGNCGYGRAAGCCYCAAGGYRTAATCANYCTCGTGTTGGTNGAACGARAAGNNNNATATAATCCCTATTTCGCAGGAATGGTYCGTGCCGGAGGAGTTAYCACCAATNAAAGTGTCCGCGAGATGCGCTACNTGYCCTTTGTCAGTCTACATTGCATAATAAACTAGTCGCTGCCTCATCCCGRCYGTACGCANNAGATCTTAGAACTTAANTNGCCGRYTACCCCGACGCCAGNNNNGCACAGCCNGAGCTACTGTATCTAAATGNACTRTYYYCNNTGNTNCGRNCNGRYCYNTCTYANNGTCRYCGCTATAACACACAGGCAGTTTGAGAAATCATACAYGCGGTNAACCGTAGGGCTAGGCNANACAGCCTRAGAGGCCANYCTACYGCRGATRAGYYNNTCAGTCTCGGNNTNNATCGGGTTCAYNGTGGACGGACCGGTCCGCAGGCGGCNTGCCYGNNNNGAACCGCGACGGGCCCCTGCGACCTACGCGYCYGCGCCCGGGYGCCGNGGTCRTTCCGTGNNACTGATCCNGGGACTNGGATGCCCTTGTAAGCACCCTGGAATAGATTGTACYACGCCACACGRCAACATAGNACGCCAGNGGTNCCTTGTCTTAGAGGTCAAGTTTYCTCGNNNTGCCCCCAGGCAATCATGCGTAAAAGGATGRGGTCATGGCAGCTCGAATYAGGTGCGGYCTNNGNYCYCRAAAAYTGCNGTCATTTATGCGTACGTCGAATGCNCCGYGGCRNTGCTTGCGGGTACTTGCGRYCGACATTGYGTCCATGGTGTTATGAGCTCTCCTTAGNNNNGGNCCNNAANCGCCACGATCATGTGCAGAAGGCGCAAATGTGTCCAANGCGTTAGCNNGGACTTTGAANGCGCGRNTATTACGGCTCACTCCTATACAGGTYTAGGTANNNCYNCACNCTGGCTGTACCCNNNGGGGNCCGCCGNNGCGCRRATTCAYGGGGGGNGAGCCAACACTYTGTTGNNGCGGRYTCNCACGGTTACTAAATTGTGGCGTCTYCCTNATCRAAGGAGAATTCCATYRYCNCGGAGGTCGCCRGCATGRTGTCGCTTCNNCCTTGATATGTNTYGCAATATANCGANRNCAGGCGCCGNACGACANANARATGACGAAACANGATACGCAGTACGGGACGACGTAGCCACCTCGGACCCTGATCGTGTTTCGTANYRACACRRCATCAGTCTCTGACTTTACCCGGRTGCAGACNYCTCAGTCAGCTGGGATTNCTAGCCGNATAAGCCRAGTGTCRGRCNAACCCGANNCTAGCGGGTCGCGCACTCCYCRRAGGRCRCCANANATNNATNTTTCACNCAGGGCGGNRGCGCCAAAACANTCNRTCCCCACCTGNGGTCAGNNCTANNANCTAAGGNAAGCNNNATGNNCGATTGGGAGGCCGAATCCCANTGTCCGGTTTTATTCYYYRAACCGACCATAAAGTACGTGNNGAANTNGTTANCTTRACCCGCTAGACCRCRTGTCGGTNCACARCYACGTCRGTCTAATGRCGTTTYYRGCGCTTGCNNCCAAGCGRCGGGAGTTAGTNGGNCNACCCTCRRTNTGAYAYAAYNTNCAGCAAGCCGATRRAARRCACTCGNNAGCATTTGCATCCACATGAGTCNCTGACTTTTCCATGNCAAACYGGCGGAATAGAGGTTCTGGATCTATGATGRNTAGCCGTCRCRGATRYNGCYGGYTGTTGYGTGGCGCGGRYTGTGCCCAGATNGYNCTCCAAGTNGYARATAGACCCTNACTNNNNNNNNCRAGAYAYAANCGYAATCGNCTCAGGTCCTAGGAACGTGGGNTCNTGATCGCTCRAYCCCACCTCTCAAAGCTAYCGCARCGNCTTCTTAANGACTGGCTAGCTTGAACCCTAGAYAACGRRRGTGTTAAGNATCTCTGGARGGNCCTCCTTGGATTGTNCACATGATCCYNANACYACTTNGTGGCAGGNNGGCAGRGCCYCNRGGCTGAGCCCTAGGCCGTCCGCAAACNGGCCARYTGGNCCAGAGGCTGANAGGGGCGCAACRYAACATGACTTTACTAAGTACANNGAGCTATTNTAGCAGGAGCCGGTGTGCATGCCCGTACCAYNCGCTTGAGNNNAAANNGTCGGTRNARCCAAAACGCCAACTRRAGAGTGCCTGGTACTTTGTATAAATGCCCTYAYYGRGNGCATTNGATCGCTCGGTCCCGAGNTNNGCCATTRGTTAGRCATTGCCTTTGCACGGCAAYNNNNNRRTGTCANGACCTNGGGNCGGYATGTGGCATACTTTTTRRRTCCGACYCGRTTRCCCGRNAYRNNGGCCCCAGCATCCTTTCGCTGGGATTTATCNTRAARRCGATCGGGCAAAGNNYNNCCTRYNGGCGRNGTCCGTTAGTGACCGGATCAAGTGACCAGATTTTGTACNYRGYATCYTACAATGGCGAGTCATTACATTCAGTCYNACGCGRYCTCGCAGTGCACATT

Gv10-H-03 NCGNNTTACTCGTCGCCATAATGNNCGCCNNNNTCGTCGNNCTAGACRGNTCTAGNNGGAGTACCCACTGACCGTGATCTACTGNNNNNNNCGCYYNTTGTCTNARGAGYCCACTGCTCNRGATCCGTNNNGGNTAYGNNCCGTGNNNNNNNNNNNNTGGCCANNCAGCGTNCAGGAGNNCCNANNNTACAGANNNNNNTGGANNNNGCYYTNNNNCGGTAGACTACNNAATNNNNNNGTYNCTAAGTAGGTTGGGNACGCAGNYYCNNNNTCTCATGAGGARRTTYTTGACNAANGCGGANCACNCNNNNNGCATTGANYATTCCYAATGNNNNNNNNNAGTTCTTCTGATGGNNTAGATTNAAGNNNNNNNNANNCNTTNNGNNNYTGNTANNGNGGGGCGGGCGCCGNAGGNNNNNNNCCTANGTACAYTACANTGCCGCCAGNTNNNCNTCAAGNNCCCTCTYTYNNCTGNTGANTNGGNNGCRTYTGNNTACCTTYNGCTGTCAANGAATCCTANCRGAAACAGCTAANNATCCTNGTCTAAACNNNNNNNNNNNTATAGAGCNGACGNNTACGNNNTCAACACCRACGAGACGAGGNNNNNNCNNNTCCRRCTANNNACGGTGGACATNCNCCTNRNNNNAAGNGGTCRCNNNNNNTGAGYAGGCCNRTCTCCTCGATCNNCTCCCTNNNNNGGAAGCCCNNNCCGNNGCTTACGACAGTRANNAGCRNAYTGCNNNNNNCGGTGGRTNCCAGGTGNNNCCTNNCGCATGCGAATGGNNGGCACTNCCRNNNNNNNNNNNNNNGGTAACTAGNNNCTGRTNTTNTNTGAGYGNRTGCCCCGTTNGCACTACRGATACANCTAGCCCTGTACACAACGGAATTGTTCTTAGGCTYGCGAATNYGRNTRNNNTNNNARAAACGNNNGCCNTGGTCCGGTCAGCCACTAATGTTGTATNNNNNNNNNACYCGNNGNAGCCATGGCATNTNANTTAGNNATAGCCCGCGGTGTANNNGCGRNCCGCGNCGGCGAGNNNYRNNTNGCTGCRAGGGATANTCAGACGATTNNNNTNTGTCTGAAGGAACGCACCCNTCTNNNNNNNNNNCNNACGCTNNGGCYCCACTTTCNNNNNNCGGRRGATTAGGCACGTCCGGGGGCCGNNRAGTACNNNNNNCTAGGAACGTGAATGCACGGACNNNNGGGCCAGCCACCCYGNNNNNNTCCNACANGGGGCGCCAGNNGGCTTGCTGCRRGGTATNTYTATGGNNNNNCGRYAACGCNNNGGTATTTGNAAAGAYNTANGNRNNNNNNNNNACTCCNGAAATGTCRNNNAGGAAYNNNNTAGCACTGNNNNNYANGTATCAGGNNCATCGGTANNNNTCCNNNCGGCCAGCGCGAATRAAATGGGCTTGYCCNNGRNCNAAGGNAGGGAAAAACYTCRRGACCCTGAGNNCTCGCCGACCCNNNNNNNCATAGAGCATNGCNTAGTTNRACATNYGNTYNCTGNNNNNANCCRTCCTCTNNNNTRCCCAGTGGCTTCCGCNNNNCTTTGTNCNNCCAGAAAGCGAACCYCCCGNNNNNCCCAAGTGGTTTTTCAAGTACCCTANNNACNTNNCTYCRNCRNAAATGNTCYGCCTNTNNNATCGNGRGCCCNNNNNCTNNTANNNANNNNNGCNNNNNNCTCNNGGNCGTAGTNNNCGNNNTAANNNNGGGGAYCANNNNGTNCANGTGGACCGNNTNNNNNNNCTNNAYTGAGATGGCANNNTCTGNNTGRATAYCGTCCGCTTGGTTAACTGCTCNNAGCCCGTCCTCNNNNNNNCCGATGAGTRACGNTNGGTCCGNGGATTCTCGYTGGACANNNNCCAAAGCGTCCAGAGNATANNTCGTTCTNNTTNCANNTACGCTANCCAATTTCGNNNNNNGCCGGNNNCGTANNNCNGGYACCRCAATNGATGTCTTNNNGAATGGCYCAARCGTCGGCGNNNNGTNNCTNNNNNNNCCCNNNNNCGGCGCNGACAGCTAGCNGACNNNNNNNNNAACTNGCGTCCCCACGAGRCAGGCGGGANNNNNANNGAGACGTTNNCGNNNAATTTAYNCNCNTTCCACNCGGTTNNNNNNTTGTNAAAGGRCGNTGCAGACYCCYARACCNGYNTGNGGCGNNATTGGGGTRYTCRCANNYACCTGAGGAATNNTCGACCNCNRAYCGGCGCATCGNGGYNAGNNCNNCTTGCTNTTGTATTARCATNGACNRCGTNGATGCATGTNNNACTTNGNNNNGGGAANNRGTGANTCCGTGNCANNNNNNNNYGCGATAACATTAGTTACCTAATGNGTCAAAGATTGNYCGTTCGTNNCCNYNCCNGCGGNGCGAAGNNNCTAAAAGGCCCNCNACGCAAAAGYNNGNNGAGTAANNNNTNNTCTCNNNNGNRYNNGGAGCGGNNNCNATAGCGRGYNGYAGGCNATTYGAGGANTAGCGCANCTNCTAGANACNGCCGGTNTAAGRNNCYYNNNNGTAGGTTNNNNNTNAATTAGCCCNCNNNNNANNAATCNNNNNCGNNNTTAGGGGCTACTNCGNTGGCGTACATNNNNNTAGACCYACNCAGCAGCRGNNNNNNNNNACAANACNNNNNNCGCCTACATCGCNNNNNNCTNNNNCTTCTNCTCANTCCNNNTCANCGGTGRCYACTAGGTGCCNNNTCCTNGATAACCGAAANGGGCYTYCANAGGNNGCNNNNNNACAAGACYCCTNNNGNNNTGCGATGYTTTNTCCNNNTNAACNNCCAGCGTCGGTGNNNACGCACRNNNGCNGNTTTRTNANCCNCNNNACYGGGCTNNNAGNGNNGGAGACGCGGTNNNGTAACGCAGGTNNCGNNNNNTANNNNCNNNGNNAATGGGACNGCNGANNNGTGACCATANACCNCGCATGGACGCYRNNCNNAANNNNAGGTGTCYACAYCGATCCCNGNNNTGAGAATCGNNCANCNCCTNNNNGYCNARNTACCCCCCGATCCGCNNNTAANNNTANCACGCTTAYAGTAATGGGACCCACCRGNNNNGCNNGNATTNNANNAACTCCGTTGNNNNNNNNCAGCGGTATTAAAGCCTCACGTCCNCNNNAANAGACNNAGTAAAATATNNNNNNNCNGNNGGANNGTCNNNCNYGCNTCACNNGTGNAATAGTCATTNGNTRTGNNAGAGGAGTTCGGGCACCAGYGTCTTNACNNNNNNNNNTCGGGTTAGCNAYNCAGCTCNNNAGGTANNCATAGNNNNNAANCGRNNNNNCATCNNNNTANANGNNANTCNNNRTGNCGYRAAARNNCCAAGGCGTAANCATTCNNGTGTTGGTAGAACGARANNRGTANTATAATCNNNNNTNCNCAGGAATGGNNNNTGCCGNAGGAGTTATCAYCAANGANAGNGTCCGNGAGNTGCGCTACNTGNCCTTTGTTAGTTTACANTGCAYAANNAACTCNNCGCTGCCTCATCCCNRCCRTCCGCNNNNGAYCTTNNANNNTAANTNGCCGRCTACCCCGACGCCANNCTCRCACNNNNNGAGCTANTGTATCTANNNGNACTRTTYYCNNYGYNRYNRGCGGNNNCCTCTTATCNNNGTCNNNNNNNCTCACNNNCAGNNTGAGAAANNNNNCNNGCGGTNATNNNNNGGGCTAGGCTANACANNNNNATGGACTNTCCTACTGCAGANGNNNCGCTCNGTCTCGGCYTRRATCGGGTYCAYNGNNNANGGACCGGTCCGCNNNCGGCGTGYCTNRTCNGAACCGCGNCGGGTACCTGYNACCTACGNNYCYGCGNCCGNNCGCCGTGGYCGTTCCGTNTAACTGATCCNNNNNCTNGGANNNNNNTGTAAGCACNNTGGAATNNNTTGTNNNNCGCCNNNNGGCNNCATAGNACGCYNGCGGTNCNNNGTCTTAGNNNNNAAGTTTCNNNNNNNTGNNCCCGGGCAATCATGCGNNACRRNATGNGGTYATNNNRGNNNNNNNCAGGTGCGGNCYYNGTTCCCAAANATTGCNNTCATTTANGNNTACGTCGAATGCCCCGCGGCATTGCTNNCGGATACCGGCGNCNNNNATAGCGTNNATGGTGNTRNNARCTCTNNNNNRNCNCGGACCRAAATCGCCNNNNNCATGNGCAGAAGGNNNNAATGTNNNNNAAGCGTNNNNACNNNNCTTGANNGCNCGRNNATTAYGGTNNNNTCCTNNATNNNTNTAGGTACGNNNNNATACTGTCTNTACCCTCGNNNNNCCGCCGTCGCGCAAANNNNNNNNNNNGGAGCNNNNNNNCTGNTGTCGCGGGTTCTCANGGTTANTAAATTGTGGCGTCNNNNNAAYCGAAGGAGNNTTCTGNNACCNCRGRGGNANCCGGCATGATGTCGCTTCACNCTTNATNNGTANTGCANTNNAACGANNNNNNGCGCCGTATNACANAAAGATGAGGAAACAAGATACRCNGNNNNGGACGACGTAGCNNCYACGGACCCTNNYNGTGTNNNGNNNYRACNNNACATCAGTCTCTGACTTTACCCNGNNGCNNNNNNNTNARTCAGCTGGGATTACNNGCCGAATAAGCCNNNNNNNRNGCCAACCCGAGTCTAGCGGGTCNNNNNNNNCYCGGAGGGCACCACACATCTATATTTCNNNNNNGGCGGAAGCGNCAAAACACNNAATCCCCACCTNNNNNCAGCCCNNNNANCTAAGGTAARCCCNATGAGCGANNGGGAGGNNNAATNNCAACATCCAGTNTTATTCNNYRANNCGACCATARARNNCRTGNNNAATTYGTTAACTTGACCNNNNNNNCCGCANNNCRGTACACAGCTACGTCAGTNNARTNNCNNTTYYRRNGYTTRCNTCNAAGCGGCGGGAGNNNGTTGGCCCACNNTNRRNNTGANNNAACATACAGTNAGCCRNTGGARAANANTCGCCAGAATTTGNNNCNAYATGCGTCACTGAYACTYCNNTGNCTAACTGGCGGAATAGAGGTTCTGGATCTATGANGNNNNNNCGTCNCRGANNNNNCNGGCTRTYRNNTANCNNGGGTTGTGCCCNGATTGTNCTCYAAGTNGYARATAGACCCTNNNNTAGGGCGTCRANAYAYAANCGNAATCGNCTCAGNNCCTNRGAAYGTGGGTTCCTNNTCGCNNNNNCCCACCTCNYANNNCTACCGCTNCGTCTTCTTAANGACTGGCTANCNTGAACCCTAGATAANGGNNNNNNNNAGNNNCTNNGGCNNNNCNNCNTTGGATTGTNCNNATGNNNNNNAGNCCANYTNGTNGCANGGGGGCNGAGCCCCNRGGCNNNGCCNNAGGCNGTNNNNAAACNNNNCAACNNGRCCNNAGGCNGAAAGGGGCGCAACACARCATGNNTTYACGRANTATACCGAGCTATTYNNRCRGGAGCNNNTGTGCATACCCNTACCACGCGNNTGNNNNNTATCTGTNNNNANNNNNNTGGNNNNNNNNGNNGANNRNNTGGNANNNNNNATAAATGCCCTYNYCGRGAGCATNGGATCGYNCGGNCCCGAGNTNNGCCATTNNNYAGNCATTGYCTTTGCACGGCAACNNNNNNNNNNNNNGANNTCGNGTCGGTNNNTGGCAGACNNTTNNGATCCGACCCGNNNGYCCGNNAYRNNGGYCNNAGCATYCYNNCRCTGGGANNTANNGNNNNNNNNNTCGGGCAAAGNANNTCCTGCTGGCAATATCCGTTGGTNNNNNGNTCAAGTNNNNAGATTNGNGAYYCGGTATTCTACANTGGCGGGTYATYRNATTCNNNCCGACGCGGTCNNGYAGTGCACATT

Gv10-H-05 NYGATNNNCTCGTCGCCATANNGTCCGCCGGRRYCGTCGACNNAGACGGGTCTARRRGGNNTACCCACTGACTGTGATCTACTGNNNNYCACGCCTNTTGTCTGAGGAGTCCACTCCTCGGGANCCGTCCTGGATATGGGCCNNGCNGCGCTTACAATGGCTTGGCAGCGTNNAGGANRRCYRNANGTACAGNGAGGTCTGGACCTGGCTTTGGAACRRTAGATCTYNTAATACGGCAGTYTGTAAGTAGGTTGGGTACGCACATTNTAGRTCTCATGAGGANNNTCTTGACANNNNNNNATCNNYCRTTGGTCATTGAYYAYTYCCNATGCCCGTGGATAGTTTGGTNGATGATGTAGATTGACCAACGGNGGANCCAATCTGGAGCTGNTAGGGCGGGGCAGGCGCCGCAGGCCAGGAGCCTAGGTACACTACAGTGCCGCCAGANAGCCTTCAAGCACCCTNTCTTGCCTGYTGARTNGGCTGCATTTGGTTACCTTCCATTGNCAACGAGCCCTAGCGGACNCAGCTAANNATCNNNNTCNNAACTCGTTTANGAGTCTAGAGCTGACGCATACGGTTTCNACACCRACGNGNCGAGGNNNNNGCGCTTCCGGCTACCAACGGTGGACRTTCCCCTAGAAACAAGTGNNNACTAAGGGTGAGCNGGCCAATYTCCTCGATCTGCTCCCTTATGTGGAAGCCCCCACCCCCGCTTATGCCAGTNCTTAGCGGACTNCGCCNTGCGGNGGGNACCAGGNNGCTCCTATYGCANGCGAATGGNAGGNACTACCGTAATCTAGCCGGCTGGTAACTANNAACTGATATTATGTGAGCGCGTGCCCCGCYCGNANTNCNGNTACAGCNAGCCCTGTACACAACGGAATTGTTCTGAGGCTCGCGAATACGGATAAGNTCGNAAAAACGGCCGCCGTGAGCCGGTCAGCCACNAANGTTGYANANTGTAGTAAATCGCRGCAGCCATGNCNNATNAATTAGGAATAGCCCGCGGTGTANNGNCGACCCGCNTCGGCGAGAGCCGCCTNGCCGCAAGGGATAATCANNNNNNNNNNNTNTGTCTGAAGGAACGCACTCCTCTTNACGCAGGTAATNTGCTGTGGCTCCACTTTCGGCRGCCGGGGGATTAGGCNCGTCCGAGGGCCTATAAGTACTAGGGCYTNNGAACGTGAATGYCYAGANNNNNGGNCCAGCCACCCCGATAAGTNCCYACARGGGGCRYCAGTTGGCTYGCTGCRRGGTATTTNTATGGCCCNNCGGTAACGCGACGGTATTTGAAAAGACNTAGGTAYGTGNNCGGACTCCTGAARYGTYRNTTNGGRNTCCACNAGCACTGACATATANNNATCAGGCGCATCGGTATCTCTCCGGGCGGCCAGCGCGAATGAAATGGGCTNGCCCCTGANCNAAGGAAGGGAAAAACNTCGGGATTCTGAGGACTCGCCGACCCTNGCGAACAYAGAGCATCGCNNAGTNTRACNTNTGGTCTNNRNRNGCRNCCNTCCTCNGACCTACNNNNNNGCTTCCGCAGGACTTTGTRYRRCCAGAAAGCGAACCCCCCGTTACTCCCAAGTGGTTTNTCAAGNACCCTACCTACATYGCTCCRNCRAAAATGATCYGCCTATGCAATCGTGAGCCCTATATCTATTAACCAGGTGGGCNCTGGTTTNGGRNTCGTAGNACTCGAYANAAGGTCGGGGATCNCTANNNNCAGGNGGACCGTNTTAGTCTGCTTNAYTGAGATGGCATGGTCTGTNTGGATATNGTCNNNCTGGTTAACTGCTCGAAGCCCNTCCTCACGAGTCCCGRTAAGTGACGNTGGGTCCGCGGANTCTCGYTGGACACTTTNNNNNNCGTCCAGAGGNTACTTCGTTCTCTTTCCATNTACGCTAGCCANTTTCGTAATGTGCTGGCAGNNNNNNNCTGGCACCGCAANGGRTGTCTTNCAGAATGGCTCAAGCANCRGCGGCGCGTCACTNTCGAGCCCCNNNCACGGCGCCGGCAGCTAGCCGACNCANNNNNNAACTCGCGNNNNNNCGAGGCAGGCGNGACGAATNCGGAGACGTTNTNNCGGGGTTTNNNCNCATTCYACGYGGTTNNNNNNTNNTGAAAGGGCGGTNCRGRCYCCYARANNNNTATGGGGYGTAATTGGGGTGTTCGCGGTCACNTGAGGNATACTCGACCGCTAATCGGCRNANCATGGNGNGNNCGCCTYGCTTTTGTATTAACATTGACTGCGTTGNTGCATGTCCANNTNCGCAGTAAGAAAGAGCGNGNCCGTRGCAACGGGCTCTGCGAYNACATTAGTNACTTAAYGCGTCAAAGATTGNYCGTTCGNGTCCCTYCCCGNNGCGYGAAGCGACTANNNRGCCCRCTNNNNAANNNNCTGNAGAGTAAGGCCYRNTCTCCGGCGNRYGCGGAGAGGTCGCRATAGCGGGTTGTRRGCRATTYGAGGACYANCNCAACNNCTAGARACGGCCGGTTTAAGRAACTTGCTGGTAGGTTACNNYTTRANNNGCCCACCCCGGAGCAATCGTCCTCGTCGTTAGGGGCTACTCTAGTGGCGTACATCGCNTNAGACCTATGCAGCGGCAGCAGCGTCTTACAATACTCTAGACGCCTACANCNTCTCGGGCTAATACNTCTCCTCACNCCTGATCACCGGTGRNCACNAGGCGCCTGTTACTGGANNACCGAAAYGGGCTTTCAAAGGTCGCCATCTTACAAGACNCCTGATANNRTGCGNTGTTNNATYCATGTNNNNTCTGAGCGTCGGTGACGACGTGCGACCGCCGGTNTATGAACCGCACCAYCGGGCTTGCAGNGNRGGAGACGCGGCGCCGTAACGCAGGTGTCGACCTGNATYAGCCGAGGAAATGGGACCGCGATCACNNGACCNNNTACCCCGCACGGACGCYRGCCYCAAGCGAGGGYGTCTACATCGATCCCAGATCTGAGAATCGGGCANCNCCTNCTAGNCGARNTACCCCCCGATCCGCNNNTAAAGTTACCACGCTTATAGTAANGGGACYYACCGGGTTNGCGAGGATTNAAGCAACTCCGNTGCTCCGTCRCAGCGGAATNCAAGCCTCACGTCCTTTATGGGAGACGGRGNGAAACATTCAGGTGCCGTCRRACCGTCCTGCACGCATCACGTGYGTANNNGTNACNRGRNATGTGAGAGGAGTTCGGGCACCACTGTCAGGACCTCGAGTTTTCGGGTTAGCCATACAGCTCCAYNGACGAAGGCAGCTNTNAANCGNTAGCCCATCCCNCTARRNGNNAYTCCAGNNNACGCGAAAGCCTCAAGGYRTAATCATTCTCGTGTTGGTANNACNANGAGAGTAATATAATCCCTATNTCNNNGGNNNNNTTCGTGCCGAAGGAGTTATCACNNNNNNNNNTGTCCGCGAGATGCGCTACNTGCCCTTTGTYAGTYTACATTGCATAATANANTAGTCGCTGCCTCATCATNNNNGTNCGCAGTAGATCTTAGAACTTAANTCNCCGACTACCCCGANNCCAGNCTCGCACAGCCTGNNNNACTNNNNCTAAATGNACTNNNTCCATTNTTGCNNGCGGATCCCTCTTAAYNTCGTCNNNNGAACTCACNGGCAGTTNGAGAAATCATACACGCGGTAANCCGTAGGGCTAGGCCACNCAGCCTGAGAGGCCAGCCTATTGCAGATAAGCCNNTCAGTCTCGGCNNANATCGGGTCCANNNNGGACGGACCGGTTCGCAGGCGGCCTGCCTGATCGGAACCGCGTCGGGCCCCTGCNACCTACNCGCCTGCGNCCGNGYGCCGTGGYCNTTCCGYNNAACTGATCCAGGGACTAGGATGCCCTTGTAAGCACANTGGAATNNATTATACCACGCCACACGRCNNCNTAGNACGCNAGCGGNNCCTTRYYNNAGAGGTCNNNNNNTCTCGTGTTGCCCCCRGGCAATCANGCGTAANNNGATGNGGTCATGGCAGCNCGAATCANNNGCGGYCYYNGNNNNNAAAAANTGCNGNCATNNNTNCGTACGTCGAATGCACCGCGGCANTGCTTGCGGATACCGGCGGYCNNCATNGCGNTCATGGTGTNATGNNCTCTCCTTARGCGCGGACCGAAATCGCCANNAYCATGTGCAGAAGGCGCNAATGTGAGCAACGCGNTAGCACGGACYTYGNAAGCGCRGNTATTACGGCCCACTCCTATACAGGTTTAGGTACGRCYTCAYNNNNNCTGTATATNNNRGNNACCGCCGTCGCGCAAATTCACGRGGGGGGAGYCAACACTYTGTTGTCGCNRGCTCTCACGGTTNCTAAATTGAGGCGTCNCYCTAATCGAAGGAGARTTCTGTYRYCTCNGNGGNNNCCRGCATGANGTCGCTTCACTCTTGNTNNNNACTGCAATGTAACGANNCCAGGNGCCGTAYRACANANAAATGANGAAACAARRYRCGCAGTACGGGACGACGTAGCCACCNCRGACCCTGATCGTGTTTCGTANNRACACAACATCAGTCTCTGACTTTACCCGGGTGCAGACNNCTNNRTCAGCNGGGATTACTAGCCRAATAAGCCGAGTGTCGGGCCAACCCGAACCNAGNGGGTCGCGCACTCCCCGGAGGGCACCACACATCTATATTTCACTCGGGGCGGNRGCNYCAAANNNNTNTATCCCCCCCTGNRGTTAGNNCNGNGAYYTAAGGTAAGCCCCATGNRCNATTGGGAGNCCGAATCCCAACATCCAGTCCTATTCTCNNAACCGACCGTAAAGTACATGRRGAANTYGTTAACTTGACCCGCTAGACCNCRTGTYRGTCNNNNGCTGCCCCRGNCTARTGGCGTTTTTGGCGCTTGCCTCCAAGYGACAGGAGNNNGTTGGCNNACCCTCRRTCTGAYAYANNNNNNNGCAAGYYGATRRAAAACACTCGYCAGNATTTGCAYCCACATGNGTCACTGATACTNCNNTNNYNAACNGGCGGAAYRGAGGTTCTCNATCTGTGATGRNNANYCGTYACAGATACTGCYGGYTRTCACGNNNCATGGRYTGNNNCCARNTGGCNCTCCAAGTTGCAAATNGACCCTNACTNNNGGCGTCAARAYACAATCGCAATCGNYTNNGGTCCTAGGAACGTGGGATCCTGATCGCTCGACCCCNCCTCTCAAAGCTAYCGCNACGTCTTCTTAACGACTGGCTANCNYGAACCCTAGATAACGGNNNTGTTAAGGATNNCTGGNAGGNCNNCCTTGGATTGTNCRCATGANNCCAAGANNACTTTGTNGCATRGRGGCAGRGCCYCAGGGCTGAGCCCTAGGCCGTCCGCNAACNGGCNNGTTGGGCCAGAGGCTGNNAGGGGCGCAACACAGCATGGCTTYACNNAGAACACCGAGCTTTCCTAGCAGGAGCCGGTGTGCATRCCCGTAYNANTCGCCTGAGGCTTATCTGTCGGTAAAGCCATGGCGCTAACTGAAGAGTGCCTGGTACTTTGNATAAATGCCCNYAYYGRGNGCATTNGATCGCTCGGTCCCGAGNTNNGCCATTRNNNAGNCATNGCCTTTGCACGGCAANNNNNNGRNGTCACGACNTCGGGACGGTNNNTGGCAGACTTTTNAGATCCGACYCGATTRYCCNAGATGNNNGCCCCAGCATCCTTNCRCTGNNAYNTANNNTRAARRCGATCGGGCAAAGNATGTCCNGCTAGCGRNGTCCGTTNNTGACCGAATCANNTNACCAGATTTTGTACCTAGCATCYTACAANGGCGGATTTTCGCATTCNGTCNNACGCGGTCTCGCAGTGCACATT

Gv10-H-07 GCGATTTACTCRTCACCANANNGNCCGCCGNAGNNGTCGACNYAGACGGGTCTARRNNGAGTACCCACNGACTGTGATCTACNGGCYATCACGCYYTTTGNNTRANGAGCCCACTGCTCGNGATCCGTCTTGGNTATGNGNNRNRNNNNNCTTACAATGGCCNGGCAGCGNRCANGAGRRCCRATTGNANNGRCAGGTTTGGACCTGGCCCNGGAACGGTAGAYYNCACAATACGGCNGTCTNTAAGTAGGTTGGGTANNNANNNCNTAGNTCTNNTGAGGARGYTYTTGACAAATGCGGATCACTCGTTGGNCATTGAYNNTTCCCAATGNCCGTGGATAGTTTGGTTGATGATGTAGAYNNAAGAACGGNGGNGCCATTCNGGAGNTGYTAGGGCGGGGCGGGCGCCGCNGGCCAGGNNCCTANGTACAYTACANTGCCGCCAGATNNNNNTAGAGCACCCTCYNTTGCCTGCTGAATGGGCTGCATTTGGTTACCTTCCATTNNCAACNNATCCTANCNGANACAGCTAANNATCCTCGNNNAAACYCRNGTRAGAGTATAGRGCCGACGCGNNNNGTTTCAACACCNACGNGNNGAGGAAAGARCGCYTCCGGCNRCCANNGGTGGACATTCCCCTNRAAACNNNNGGTCNCNRAGGNTGAGCNGNCCNRNCTCCTCGANCTNNYCCNTTRNGTGNAAGCCCCYRCCCNNGCTTACGNCNNNRATTRGYATACTNCGCCRTGCGGTGGGTACCNGGNGRNTCCTANCGCATGCGAATNNTNGGCNYTNCCAAGGACTAGCCGGCTGGTAGCTAGNAACTGATANTATGTGAGTACATGCCCCGCCCGGANTACRGANNNANCNAGCCCTGTACNNNNCGGAATTGTTCTGAGGCTTGCGAATNYGRNTRNGTTCGGNGAAACGGCCGCCTTGGTCCGNTCAGYCACTRNNNNNNYRNNNNGNAGTAACCCGCGRYANNNATGGCNYNTAAATTAGGAATAGCCCGCGGTGTANNNGCGRNCCGCNTCGGNNAGRGCCGCYTNGCYGCNAGGNATAATCAGAYGNTTAGATNNNNNCTGAAGGAACGCACYCYTCTTAANAAAGRYCAYACGCTNNGGCTCCACTTTCGGCAGCCGGGGNNTTAGGCNCGTCCGNGGGNNNATGAGTACNNNGGCCTNNGRNCGNGAATGCNCRGACGATGGGGCCAGCCACNNCNACAARTTCNNNCARGNRNNATCAGTYNNNNNGNNNTRRGGTATTTNTATGGCCCGACGGTAACGCGACGGYNYNNNRNAAGACTTAGGTAYNNNNNCGGACTCCTGAAATGTYRNTTNNNRNNYNACTAGCACTGACATATNAACATCAGGCGCATCGGTANCTCTCCGGGCGRCYRRCGCGAATGAAATNGGCTCGCCCCTGRNCRAAGGAANGGAAAAACTTCNNNNCCCTGAGGTCTCNNCGACCYTAGTNAACRTAGAGCRTCGCCCAGTTTNACATNTGGTCTNYRGRNGCRRNNNTCCTCTGACYTACCCAGTGGCTTCCGCNGGACTTTGTACAGCCAGAAAGCGAACCCCCTGYNNYTCNCNAGTGGTTTNNNAAGTACCCTTNNNACGTYGCYCCGACGGAAATGCTCTGCNNATGCAATCGTGAGCCCTATATCTATTANNCRGGTGGGCACTGGTCTNGGNNTCGNNNNACNCGAYNTAANNTCGGRNAYCNNTATGTGCAGGTGGACCGTNTTAGYCYGCTTYAYTGAGATGGCAYGGTCTGNNTGGATATCGTCCGCCTGGTTANYNGNNCNNANCCCNTCCTCANGAGGCNCGRTGAGTNACGGTGNGNNCGGGGATYCNCGCTGNACANTTTCYAANGCGTCNNNARRATACTTCGTNCTCTTTYCATTTACGCTANCCAATNNCGTAATRTGCCGGCAGCGYARAGCTGGCACCGCAANGGATNTCTTGCAGAATGGYYCAARCANCRGCGGCGCGTCANNNTCRAGNCCCTGGCACGGCGNCGACAGCNRGCNGACCCAYCYAATAACTCGCGTCCCCACGAGGCNGGCGGGACGAATNNNGAGACGTNNTGACGGGGNTTAYNCACRTTCCACGCGGNNNNNNGATTGTGAAAGGAYGGTGCRGRCNCCNANANNNNTATGGGGYGTAATTGGGGTRYTCGCGGTCACCTRNNNNGTACTCGACCGCTAANCGGCGCATCGTGGNNAGGGCGCCTYGCTYCTGTATTAACATTGGCNACGTAGATGCATGYGGARCTTCGNNNNRRGAAAGRGYRAGTCCGTGGYRRCGRGCTCTGCGATAACATTAGTTACYTNNTGCGTCAAAGANTGNNNNTNCGNGTCCNYCCYCNCGGNGNGRAGAAACTNAAANGCTCGCTACGCATAACCNNGNNNAGTAAGGCCYAATCTCYGRCGCACGCNGNGCGGTCGCRNYAGYNRGTTGYRRGCGANTYNRNNNCTAGYRCNNCNNNNAGARACGGCCGGTAYANGGNNCCYGCTGGTAGGTNACGRCTTAATTAGCCCRCYCCGGAGCNATCNTNCTCGTCGTTAGNGNCTACTCTAGTGGCGTACATCGCAYTNNACCYATGCAGCNGCAGCAGCGTCNTACAANACTCTAGANGCCNACANCRTCTCGGGCTAATACNTCTCCTCACNCCTGNTCACCGGTGGCYACTAGGYGCCGTANNNTGGATAACCGAAACNNNNNNNCAAARGTCGCCATCTTACAAGACNCCTNATATTGYGCGGNGNTNNNTYCRYNNCANYNCTGNGCGTCGGNNACGACGCGCGACCGYCGGTNTATGAACCGCACCACNGGGCTTGCNNRNNNGGAGACGCGGCRCYGTAACNNAGGTGTCGACCTNYNTYNNCCGAGNNAATGGGACYGCRNNNNNGTGACCNNNNACYNNNNNCGGACGCYGGCCYCAAGCRNRGGTGTCNACATNNATCYCRGRYCTGNGAATCGRRCAACGCCTTCTAGCCGAAATNCCNNCCGANCNGNGCATAANGTTACCACGCTTACNRTNATGGGACYYACCRGNTTTGCNNGGATTANAGCAACTTCGTTGNNCCGTCGCAGCGGNATTNAAGCCTCACGTCCTNTRNGGGAGACNRGNTGAAATANTCAGGTGCYGYYGGACCRTCNGNNNNNCRTCACGTGNNNAATAGTNAYNRGRNNTGNNAGNNNAGTTCGGGCACCAGYGTCTTNRCNNNNNNNNNTCRRACNAGCCATACAGCTCCAYAGACANNGGNAGCTGTCANNCGRTAGTCTATCCCACTAAATGNNANTCCAGRTGNCGYRAAARNCCCAARNNNTAATCANNNTCGTGTTGGTNGAACGNNNAGAGTAATATNATCCCTNTTTCGCAGGNNTGGTNCGNNCCGRAGGAGTTATCACNNNNNNNNNTGTCCGCGAGATGCGYTACNTGCCCTTTRTYAGTNTACATTGCATAATATAATCGTCGCTGCCTCATCCCGACCGTACGCNGTNGATCTTAGAACYTNNATCGCCGGCTACCCCGNCGCCAGCCTCGCACAGCCTGAGCTAYTGTATCTAAANNGACTRTYNNCNYTGCTACGAGTGNRYCCCTCTNANNGTCGTCGCTATAACACACNGGCRNTTNGAGAAANNNTACACGCGGNAANCCGTAGGGCNANNCYACANNGCCTAAGAGGCCANCCCATTGYRGATGAGTYGCNCAGTCTCGCNCTRRATCGGGTCCANNGTGRRNRGNNNNNNTCGCAGGCGGCGNNCCYGATCNGAACCGCNNNNGGCCCCTGCGACYTACGCGYCYGCGCCCGGGNGCCGTGGYCRTTCCGNRNANCTGATCCNGGGACTTRGATGCCCTTGTAAGCACCCTGGAANAGATTGTACCRCGCCACACGACRRCATAGNACGCCNNNRGTACCTTRYNTTAGAGGNCNNNNNNYCTCGTRNTGCCCCCGGGCAANCATGCGCCACRRRRNGGGNNNATGGYNGCNCGAATNNGGTGCGGYCNYNGTNCNCNAAAATTGCNNNNNTNNANGCGTACGTCGAATGCNCCGNGGCRTTGCTTGCGGGTACCGGCGGCCNTCATAGNGNYCATGGTGTNATGAGCTCTCYYTAGGCGCGGACCRAAATCGCCACGAYCAYGTGCAGAAGGCGCNARTGTGNNNAANGCGNTAGCACGGANNTTGANNGCGNGRNTATTACNGNTCACTCCTATACARNTYNNNGTACGACCNCNNNNNNNCTRTATATCTGRGNNNCCNCCGTCGCGCAAATTCACGRGGGGGGARTCAAYNCTTTGTYGNNGCGGNNNNNCRCGGTTACTAAATTGTGGCGNCTTCCTNATCRAAGGAGAGTTNTGYTNNCNCGGAGGTNNCYGGCATGNNGTCGCTTCNCYCYYGATNCGNNTYGCRATRTANCGANRCCAGGCGYCGNACGACATANARATGACRAAACANGATACRCAGTACGGGACGACGTNNNCACCNNGGACCCTGAYNGTGTTTCNNNCTGACNCAACNTNARTCTCTNNNNNNNNNNGGRNGCAGACTCCTCAGTCAGCAGGGATTACYRGCCGAATANGCCGAGTGTCRGNNNARCCCGANNCTAGNGGGTCGCGCACNNCCCGAAGGGCACCACACATNNATNTTTCACNCRGGGCGGCNNNRCCAAAACANTCNRNCCCCACCTRTRGTYAGCCCNRARATCTAAGGTAAGCCCNNTGTACNATTGGGAGGCCGAANCCCANNNTCCNGTCCTATTCTCTAANNCGACCNNNNNNNACRTGNNNNNNNYGTTAACTTGACCYGCTAGGCCGCATGTCGGTNCACARCYNCNYCGGTNYANNGGNNNTTNNNRNGYTTNCCTCCAAGCGGCRGGAGTTAGTNGGNCNACCCTNNNTCTGATACAACATCCAGTGAGCCGATGGAARRCAYTCGCCAGCATTTGCACCCACNNNNNNCNCTGAYACTCCTRNGNCTAACTGGCGGAAYRGAGGTTCNNNNNNNNTGANGNNNARYCGTCRCNGATGTAGCYGGCTRTCACGTGNCRYGGRYTGTGCCCAGATNGYYCTCCAAGTNGYARRTAGACCCTGACTTAGGGCGNCGAGANAYAANCGNNANCGNYTCNGGTCCTAGGAACGTGGGNTCNNNNTCGNTCRAYCCCNCCTCTCAAAGCTNNCGCNRCGTNTNCTTAANGACTGGCTAGCTTGAACCCTAGATANNGGRRGTGTTAAGNRNCTCTNNNAGGNNNNCCTTGGATNGTNNGCATGATCCCAANACCACTTTGTGGCATGGGGGCAGRGCCCCTAGGCTGANNNCNAGGCCGTCNNNNAACNGGCGAACTGGACCAGAGGCTGANAGGNGCGYAACRYARCATGNCTTTACTNAGTAYACYGAGCTATTCTAGCAGGAGNCGGNGTGCATGCCCNTACCACGCGCCTGAGNNNTRTCTGTCGGTAAAGNCAAAACGCYAACTAGAGNNYGNNTGGTACTTTGNATAAATRCCCTYAYYGRGNGCATTNGATCGCTCGGACCCGAGNTNNGCCANTRGTTAGGCATTGYNNNTGYRCGGCAAYNNNNNNNNGTCACGACCTCGGGTCGGYTCGNNGCANNCTTTTNRNNTCCGACYCGATTGCCCGRNANGGAGGNCCCAGCATNCNYACRCTGGGNYTTANNGTAAAGACGATCGGGCAAAGCATGTCCTGCTGGCRATRNNNNNTRGTGACCGGNTCAAGTNACCAGATTTNRNACCTAGCRTCYTACANYGGCGGATTTTCGCATTCAGTCNNACGCGGTCTCGCAGTGCACATT

In501 ACGATTTACTCGTCGNNNNACAGGCNNCCGGAGTCGTCGACCTAGACGGGTCTARGAGGAGTACCCACTGACTGTGATCTACTGGCTATCANNNNNNTTGTCTGNGGAGCCCACTGCTCGGGGTCNATCTTGGGTATGNNCCATACTGCGCTTANNNTGATCTGGCAGCGTGCAGGAGGACCAAAAGTAACGNNNNNTTTGGACCTGGCTTTGGANCGGTAGACTTYATAATACGGCANNNTGTAARTANNNNNGRYACGCAGATCCTAGGTNTCATGAGNNAATTTTTGACAAACACGGATCACTCGTTGGGCATTAAYNNTTNCCAATGCNNNNNNNNAGTTTGGTTGATGGNNNNGCTTGGAGAACGGTGGAGCCRNTCTGTAGCTGNTAGGNNNNGGCGGGCGCCGCAGGTCCGAAANNTAGGTACAYTACATTGCCGCCNGCTAGCCTTCAAGCACCCTCTCTTNNCTGTTGAGNGGGCTRYATTTGGTTACCNTCNGCTRNCAATGAACNNNAGCGGGAACAGCTAATNATCCTNGTCTAAACTCAAGTAAGAGTCTAGAGTCGACGCGTACGATTTTAACACNGACGRGRYGAGGAAAGAACRCCTCCGGNNNCCAACGGTGGACATTNNNNNAGAANCAAACNNNNNCTGAGNNTGNGNAGGCCAATCTCCTCAANCTGNTTCTCTGTGCNNAAGCCNNCACCGCCGCTTATGACAGTACCTAGCGGATTGCNNNNTGNNGTGGGTACCAGGTGAATCCTATYGCATGCTNNNGGCNGGAACTACCGTAATCTRNNCTGCTGGTAGCNAGGAACTGATNTTATGTGAGCGCGTGCCCCGTYCGCANNNCNGNTACNNCTAGCCCTGTNNNCGGCGNAATTGTTCTTAGGCTTGCNANNNNNNNNNAGATCNNNNNAACGGCCGCCGTGAGCCGNNNNGCCACTANNNNNNCANAYTGTAGTAACTAGCGGCAGCCATNNNTCNTAAATTAGGAATAGCCCGCGGTGTAAAGNCGACCCGCGTNNNNNNNAGCCGCCTNGCTGCAAGGGATAANCAGACGCTTAGATTATGTCNGAAGGRACGCACCCCNCTTACCAAAGGTCACACGCTGTGGCTCCACNNNNGGCAGCTGGAAGATTAGRYTCGTCCGGGGGCCGATAAGTACTAGGGCCTNNNAACGTGAATGCNCRNACGATGGGGCCAGCCACCCCGACAAGTTCCCACAAGCANNGCCAGTTNGYTTGNNNTNNAGAATTTCNNNNNCCCGACGGTAACGCNNNAGCCCTTGAAARRACNTAGGCGTGAGATCGGACTCATGANATGTNNNTTAGGRNTCCACTAGCACTGACAYRCAAACATCAGGCGCNTCRRTANCTCTCCGNNYGRCCGACGCGAATGNARTNRGCTNGCCCYTGAANNNAGGAAGGGAAANNNCTCAAGACTCTGAGGACTCGCCGACCCTAGTGAACATAGAGCATCGCCCAGTTTAACANNNGGTNNCTGGNNGCAACNATNTTCTGRCNNACNNAGTGGCTYCCGCNGGNCTTTGTACAGCCAGAANNNNAACCTCCTGCANCTCCCAAGTGGTTTTTCAAGTAGCCTNCYTACATCGCTCCGNCGAAGANNNNNNATCTGTCCTRTCGAGANCCCNNTATCTATTAACCNGGTNNGCACTACTCTCGAACTCGTAGTNNTCGNNNTAAGGTCGGGGATCACTATATACAGGAGGNNCANATTAGNNNNCTTYNNNGAGATGGCATGGTNTGTTNNGATANCGTCCGCTTGGTTAACTGCTCGARNCCCATCCYCACGCGTCCCGAAGAGTNAYGGTGGGTCCGCGGATYCNGACTGGACACTTTCCAAAGCGTCNAGAGGATACTTCGTTNTCTTTCCATTTACGCTAGCANNNNNCGTAATGTGCCGGTGGCGTAGAGCTGGNACCGCARTGGGTGTNNNGCAGANNGGCTCAAGCGNCRGCGGCGCGTCACTATCAAGCCCCTGGCACNNNNNCGRCAGCTAGCCGNCTCACCCGATNNNNCNNNNCCCCACGAGNNAGGCGNGAYGAATACGGAGACNTCATGACGGGGTTTRTNYACATTTCGCGTNNTTCAGTGATTANNNAAGGRYGGTNCRGRCCCCCAGNCCGATATGGGGNGTAATTGGGNNGCTCGCAAATACCTGANAAGTACTCGACCGCGAATCGGYGCANCGTGGCNAGGGCGCCTTGCNTTTATATTAACATTGGCTACGTAGAGGCATGCGGAGCTNNGCGGCAAGAAAGAGCGAGACCATGGCAACGGGCTCTGCGACCACATTAGTTACCTAATGCGTCAAAGATTGATTGTTCGTGTCCNYCCCCGCGNNGNNAAGAAACTAAAAGTGCCGCTGCGCAATAGCNNGNNGAGTAAGTCCCANTCTCCGGCGCACGCGGAGCGGTCGCGCCAGCCGGTTGYNNGNNNNTCNNNRNYTAGYGCAACTNCTAGAAACGGCCGGTACAGGGNNCTCGCTGGTANGTTACNNNTNRAYTANNNNNCCCCGGAGGAATCTTGCTCGNNNTTANNGTCTACTCCGGTGGCGTACATCGCACTATACCCACGCAGCAGCRGCAGCGTNNNACAATACTCTAGACGCCYACAGCATCTCGGGCTANTNCTTCTCNNCACTCCTGNTCACYGGTGGCCACTAGACGCCTGTTCCTGGATAACCGNNACGGGCCTCCAGGGGNNGCCATCTTACAAGACTCCTGATNNNRTGCGNTGTTTTATYCATNNCAACTCTGAGCGTCGGTGACGACGCACGACCGNNGGTTTATGAACCGNACCACCGGGCTTGCAGGGGAGGAGACGNNNCGCCTTANCGYRNGTGTCNACCTGTTNNAGCTAGGGAAATGGRACCGCGATCACGTNACCNNATACNCCGCATGGACGCYGGCCYCAAGTGRNNGTNTCNACANCGATNCCAGATCTGAGAATCGAACNNCNNNTTNNNGCCGNAATNCCCCCCGAYCYGYGCATAAAGTTAGCACGCTGCCAGTAATGGGACCCACCGGGAATGCGAGGATTANAGCANNTCCGTTGCTNNNTCGCAGCGGTATTAAANNNNNNNNNCTTYTNNGGNAGACGGGGTGACGCATTARRNCNYYGTCGGATCGTCTGTCCCACGTCACGTANNTAATAGTCATNAGGNATGTGAGAGGAGTTNNNGTGCCAGCGTCTTTACNNNNNATTTNNNNGTNAGCCATAGAGTTCNRTAGACRTTGGCAGCTCNCAANCGGTAGCCCATCCCACTAAAGGCGACTCCAGRNGNNNNGAAAGCNNNAAAGTGTAATNAACCTCGTGTTGGTAGATGGAGAAGAGTAANNNAATCNNNNNTTCGNNGGAAAGGTCCATGCNGNAGGAGACNNCACCAATGAANNTGTCCGCGAGATGCGCTACNTGNCCTTTGTYAGTNTACACTGCAYAATATAATCAACGCTGCCTNNNCATGANCNNACGTNNNAGCCTCCAGAGACTAATTCGCCGRCNACCCCGACGCCANNNNNGCTCAGCCNGAGCTACTGTATCTAAATGGACTGNTCTCATTNTAGTTGGCGGGCTCCTCTTANCNNNACAGCNAGGRCTCACAGGCNNTTTGAGAAATCATACATGCGGTAAANNNNNGGGNNNGRCYACACAGCCTRNNNGGCCATCCTATTGNGGATAGGTCGCGCRGTCTCGGCCTGGATCGGGTYCATCGTGRRCGGNNNNNNCCGCNNNCGNNNNNCNNGATCGCTACCGCGTCGGGTACCTGCGACCTANCYNTCCGCGCCCGGGYGCCGTGGYCGTTCCGNRTAACTATTCCTGGGACATGGATGCCCTTGTAAGCATCANNNAATAGATTGTACCNNNCCNNNNGACAACATNGNACGNCAGCNGNNCCTTGTCTTAGCGGTCAAGTTTCCTCGTGTTGCCNCNNGGCAATCATGCGCCAAAGGRNGGRGTCATGGCARCTCGAATNAGGTGCGGCYCCTGNTCCTAAAAAYTGCAGNCANNNAYGCGCACGTCGAATGCACCGCNACGNTGCTTGCGGRTACCGACGGNCATCATAGNGTTYATGGTGTTATNNNCTCTCCTTANGCGCGGACCGAAAACGCCACNGTTATGCGTAGAAGGCGCAAATGTGTCCATAGCGNTAGCACGNACYTTGAANGCGCGAATATTACGGTNCNYTTCTATACARNTCTTANNNNNNCNTCNNACTNGCTGTATATCTRGGGGACCGCCGCCACGCAAATTCACNGGNGGGGAGTCAACACTTTGTTGAAGCGGGCTCACNCNGTTNCGGAATNGTGGCGTTNCCCTATCCGAAGGAGARTTATGYTRYCNCGGAGGNNGCCGGCATGGTGTCGCTNCACNCTTGATATGTNNNNNNTTGTACCGATANYAGGCGCCGTAYGACANNNNNATGACANNACAARRYRNGCAGTACGGGACGAYGNNNNCACCACGGACCCTGATCGTGTTTCGTAACAACACAACNTCARTCNNNGANTTNACCCNNGTGCAGACNYCTCNRTCAGCAGGGATTACCGGCCGAATAAGCCGAGTGTCNGGCCAACCCGAGTCTAGCGAGTCGCGCANTCCCCNNNGGACGCTGCACATANNNNTTGCACTCAGNNNNNNARCGCTAAAANACTYTATCCCCACCTGTGGNNNRCCCNRARATYTAAGGTARGCCCCANNNGCGANNGGGAGGCCGAATYYNRNTGTCCGGGCCTNNNCCTCGAACCGACNGYRRNGTACGTGNAGANTTNGTTARCTTGACCYGCTAGRCCGCANNNYGGTNCNCRGCTRCNTCGGTCTRNNGGCACTTCCAGCGCTTGCCACCAAGCGACGGGAGTTANYTGGNNCACCCNCRRTNTNATACAATCNNNNGCANNNNNNTNNANGGCNCTCGCCAGCATTTGNNNCCACATGNGTCCCTGNTACTCCTRNGTCTAACTGGCGGAATAGAGATTCNNNNNNNNTGATGNNNARNCGTCACRGATACTGCYGGCTATTGNNNACCGCGGGYTGYGCCCAGATTACCCNNNAAGTTGCAAGTAGACCCTTACTTGGGGCGTCAAGANATAAGCGCAATCGCCTCAGGTCCTAGGAACGTGGGTTNNNNNTCGCNNNACCCCACCTNTCAAAGCYACCGCARCGTCTTCNNNACGACTAACTAGCTYGAACCCTAGATAATGNGGGTGTTAAGTATNNCTGNCGGGGCNTYCTTGGATTGTNCGYATAANNCTTAGACCACTTTNNNGCATGGGGGCAGRGCCCCAGGGCTGAGCTCNAGGCCGTCCGCAAANGGGCCAGTTGNGCCRNAGGCCGANNNNNNNNNANCNNNNNATGACNTCACTARGTACACCGAGCTATTCTAGCARGAGCCGGTGNNCATNCCCGTACCAYNCGCYTGGAGCTTGTCTGTCGGTAAAGCCATGGCGCCAACTGAAGAGTGCCTGGTACTTTGTNTARANGCCCTTTCCGGGAGCATNNGANNGCTCGGNTCCGAGNTNNGCCATTARYNNGACNTTGCNNNNNNNNGGCAACGGCCCRNTNNNNCGANCTCGGGACGGTNNNTGGCAGACNNTTTAGATNNNNNYCGGTTNNNNNNNANNNNGGCCCCAGCATCCTTACNCTGGAATTTACCGTAAAGACGATCGGGCAAAGCATGTCCTNNNNGCGRNGTCCGTTGGTGNCCRGATCAAGTGAYCARNNNAGGGACCTAGCATYYTACAGTGGCGGGTCATTACATTCAGTNNNACGCGNNCTCGCANTGCACATT

In501a RYGATTTACTCGTCGCCATRNNGGCCNCCGGNNCCGTCGACCTAGACGGGTCTAGGAGGNNTACCCACTGAYCGYGATCTACTGGCYATCACGCCTTTTGTCTAAGGGNNNCRCTGCTCRRGRTCCGTCTTGGGTATRGGCCATRCTGCGCTTACAATGGCCTGGCAGCGTGCAGGANGACYANNNGTAACRRCAGGYTNGNACCTGGCTTTGGAACGGTAGACTTNAYAATACGGCAGTCTNTRNGTAGGTTGGRYACGCANATCCTAGRTCTCATGAGGAAATTTTTGANAAACGCAGATCACNCNTTGGGCATTGAYCATNNCNAATGTCCGTGGATAGTTTGGTTGATGGYNTAGNTTGRAGAACGGTGGAGCCANNYTGGAGCTRCTAGRGYGGGGCGGGCGCCGNNNGYCNGRAGCCTAGGTAYATTACATTGCCGCCAGCTRGCCNTCAANCRCYCTCTCTTGCCNGTTGAGCGGGCTGCATTTGGTTACNTTCCATTRYNAATGAATNCTANCRGAAACAGCTAAGTNNNCTNGTCTARACTCAAGTAAAAGTCTAGAGTNGACGCATACNATNTCAACACCGACGGGGCGAGGAAAGARCRCYTCCGGCTACNAACGGTRGACRTTCCCCTAGAAACRARYGGTCGCTGAGNGYGAGCAGGCCAATCTCCTCAATCCGAYYCTYTGAGTGNAAGCCCCYRCCGNNGCTTACGNCAGTACYTAGCGGAYTGCGCCGNNCGGTGGRTACCAGGTGAATCCTANYGCRTGCNAATGGTNNNCACTACCGTAATYTRGCYGGCTGGTAGCTAGGAACTGATATTATGTGAGYRCRTGCCCCGTYCGCAGTNCRGNTNCNGCTAGNNNTGTACNCRACGGAATTGTTCTTAGGCTYGCGRRTNYGACTRARATCGNAAAAACGGCCGNCTTGGTNYGGTCAGCCACTRAAGTTGYRANTNGNAGTAACCCGCGGCAGCCATGGCTCNTAAATTAGGANTAGCCCGCGGTGTARRGGCGGGCCRCRNCGGYRAGRRCCGCCTNGCNNNAAGGGATANTCAGACGNTTAGATTATGTCNGAAGGRACGCACYCYCCTTANCAAAGGYCACACGCTGTGGCTCCACTTTYGGCGGCCGGGGGATTARGCANNNCCGGGGGTCGNTRRGNANTNGGGYCTNRRAACGNGAATGCCCAGRCGATGGGGCCAGCCACNCCGAYAAGTTCYCRCARGNRGGRYCAGNNGGCTCNCTGYRRAGAATTTYTATGGCCCGACGGTAACGCRNCGGYATYYNRAAAGACATAGGYATGAGNNCRGACTYCAGAAATGTCATATAGTAATCCACTAGCACTGACRNNNAARYATCAGGCGYATCGGTANCTCNNNGGGCGRCYGACGCGAATGAAAYGGRCTYGYCCCYGGACAAARGAAGGGAAAAACCTCRRGACYCTGAGNAYTCGCCGRCCCTARTGAACATAGAGCATCGCCCARTTTAACATTYGGTYNCTGNNNGCARCYATCCTCTGACNTACCCAGTNGCTTCCGCAGGACTTTGTRYRRCCAGAAAGCGAACCYCCCGCACCTCYCNAGTGGTTTNNCAAGTAGCCTNYCTACRTTGCYCCGNCANNNNYGCTCTRYCTGTGCTRTTGNNNGCCCTATATCTATTAACCAGGTCCGCATTGGGTTCGNNNTCNTAGTNCTCGATNTAAGGTCGGGGRTCAYTGTGYGCAGGAGGAYCATATTAGYCYGCTTCATTGAGATGGCATGGTCTGTTTGGATATCGTCCGCYTGGTTAACTGCTCGARGCCCATCCTCACGAGNCCCGRTRAGTGACGGTGGGTCCGGGGATTCNCGCTGGACACTTTCCAAAGCGTCCAGAGGATANNTCGTTCTCTTTYYATTTACGCTANCCAATTTCATNANRTGCYGGCAGCGYAGAGCTGGCACCGCAATGAGYRTCNTGCAGAAYGGCTCAAGCRNCRNCGGCGCGTCACTATCAAGCCCCTGGCANGGCGGCGACAGCTAGCCGTCNCACCCRATRRNNCGTGTCCCCACGAGGCAGGCGGGATGAATACGGAGACGTCATGACGGGGTTTAYNCACATTTCGCGTGGTTCAGYNRTTAYGNAAGGRCGGTGCAGACTCCTAGACCGATATGGGGYGTAANTGRGNNGTTCGCNAATACCTRNGRARTACTCGACCGCGAAYCGGTGCAGCGTNGCGAGGGCNYCTCGCTTYTRTATTAACATTGRCNACGTAGANGCATGYGGARCTTTGCRGYAGGAARGAGCGARNCCRTGGCANNNNGCTCTGCGAYNACATTAGTTACYTAATGCGTCAAAGATTGATYGTTCGTGTCCNYCCCCGCGGCGNNAAGAAACTAAAAGNNYNGCTNCGCANNAGCCTGNNGARTAAGNCCCGTTCTCCGGCGCACGCRGNGNGGTCGCGNYAGYNRGYTGCGAGCGAGTCCGNRAYTAGCACTACTNCTAGAAACGGCCGGTAYARGGAACCYGCTGGTRGGTTACRRCTTRATNAGCCCACCCCGGAGCAATCGTCCTCGTCATCTGGGGCTACTCCGGTNGYRTACATCGCACTATACCCACGCAGNRGCATCAGCGTCTTACAATACTCTARACGCCTACAGCATCTCGGGCTANTACTNCTCCNCACTCCTGATCACNGGTGRNCACTAGRYGCCNNNNNNTGGATANYCGAAAYGGGCCTCCARAGNTCGCCRTCTTACAAGACYCCCGATRGNRTGYGGTGYTTTNTCCRYGTCAACTYTGNGCGTCGGTGRCGAYGCACGACCGCCGNTTTATGAACCGCACCAYCGGGCTTGCANGNNNGGAGACGCGGCRCYGTNRCGCAGGTGTCRACCTGYTNNAGCNNGGAGAATGGGACCGCGATCACGTGACCCCTTACTCCGYRTGGACGCYNGCCTCAAGCGGRGGTGTCYACATCGATCCCRGRYCTGAAARTCGGGCANCTNNTTCTAGCCGAAATACCCNCYGACCTGCGCATAAAGTTACCACGCTNNYAGTAATGGGACYYACCAGCNNNGCGAGGATTAAAGCAACCCCGNTRNNCCGTCGCAGCGGNATTAAAGCCTCACGTCCTYYGGRRNAGACGGRRTGANRCAYTNRRNCACYGTCRRANCATCTGTCCTGCATCACGTRYGTAATAGTCACTAGGTRTGTGAGANGAGTTCGGGCACCAGYGTTTTNACCTNAGATTTTNGGACTAGCNAYNCAGCTCNRCAGACATTNRYNGCTCACAAGCGGTAGYCYATCCCACTARANGYNACTCCAGGGGACGCGAAAGCCCCAARGTRTANTGAACCTNGTRTTGGTNGRNNGANAAGAGTAATATAATCCCTATTTCGCAGGAATGGTNCGTGCCGRAGGAGNNATCACCAATGAAARTGTCCGCGAGATGCGCTACNNGYCCTTTGTNAGTCTACATTNCAYAATAAACTCGTCNNTGCCTCATCATGRYCGTACGCAGNAGCCTCCAGAACCTNATTCGCCGACTACCCCGACGCCAGCNNNGCNCAGYCNGAGCNACTNNNNCTAAATGGACTGTCCTCATCGTNGNNGGCGGGCTCCTCTTAACGTCRYAGCTATAACACACAGGCARTNTGANNAATCATACANNCGGTAANCCGTAGGGCGAGGCTTCNCAGCCTNAGAGGCCATCCTATTGNGGATAGATCGCNNAGNCTCGNYCTRRATCGGGTCCATCGTGRRCGGACCGGTCCGCAGGNRGCGYRCCYNATCGGAACCGCGTCGGGYNCCTGCGACCTANNCGTCCGCGCCCGNGYNCCGTGGTCGTTCCGYRTAACTGATCCAGGGACTNGGATGCCCTTGTAAGCACNNCTGAATAGATTGTAYCATCCCACACGACGGCCTAGGACGCCNGCRGTACCTTACTTNAGCGRTCAANNTTCCTCGTGTTGCCCCCGGGCAATCATNCGTAANRGGATGGGGTYATGGTARCGCGAATYAGRTGCGGYCTCTGNCCTCGAAAATTGCNGTYATNNATGCGYACGTCGAATGCACCGCGRCRYTGCTTGCGGGTACTTRCGRCCRTCATAGTGTTCATGGTGTTATGAGCTCTCCTTAAGCGCGGACCGAAATCGCCACGATCNTGCNNARNAGGCGCAAATGTGTCCAANGCGTTAGCACGGACNTTGAAAGCGCGRNTATTACGGCTYACTCCTATACARNTCTARGTANNACYYCACNCTGGCTGTATATCTGGGGGACCNCCGYCRCGCAAATTCAYGRGGGGGGARYCAAYNCTYTGTYGTCGCGGGCTCNCRNGNTTACTAAATTGTGGCGTYNNCCTANYCRAARGAGARTTNTGYTRYCTCGGAGGTCGCCGGCATGRTGTCGCTTCACNCYYGATATGTATTGCATTRYANCGATACCAGGCGYCGTAYGNCNTNNNGATNACAAAACANRRYRNNNAGTACNGGACGATGAAGCYACCTCGNNNNCTGATCGTGTTTCGTAACAACACAACNTNNGTCTCTGACTTTRCYYGGGTGCAGACNYCTTAGTCAGNAGGGATTACCGGCCGAATAAGCCGAGTGTCNGRNNAACCCGANNCTAGCGGGTCGCGCACNNCCCGRAGGGCACCACACANCNATNTTTCACTCRGGGCGGCRGCGCCAAAACANTYARTYCCCACCTGTGGTCARCCCTAARATCTAAGGTAAGCCCCATGAGCGATTGGGAGGCCGAATTTGGNTGTCCGGTCCTATTCCTCGAACCGACCRCGRNGTNTGTGRRGAATTTGTTAANTTGACCCGCTAGACCGCANGNNGGTNCACAGCTRCCCCAGTCTANTGGNACTTCCAANGTTTGCCTCCNAGCNRCAGGAGTTANTGGGACAACCCTCRRTATGAYAYAAYNNNCAGYAAGCCGNTRRAAGGCACTCGCTAGCATTTGCAYCCACATGCGTCCCTGNTNNTTCCATGNCNAACTGGCGGAATAGAGGTTCTCGATCTGTGATGRNTAGCCGTCACANANGTNRCNGGCTATNNCRNNGCACAGNNNNYGCCCAATTTRYTCTCCAAGTTGCAARTAGACYYTGAYTTRGGGCRTNRAGAYATAANNGCANTCGCTTCAGGTCCTAGGAACGTGGGNTCCTGATCGCTCGACCCCACCNCNYAAAGCTACCGCAACGACTTCNNNACGACTAACTAGCTTGAACCCTAGATAACGRGGGTGTTAAGGGATACTAGARGGGCAACCTTGGATTGCNCGCGTRATCCTTAGACCRCTTYGYGGCANGGGGGCAGANCCCCTAGGCTRAGCYCNAGGCCGTCCGCAAACNGGCNARYTGGNCCAGAGGYCGACGGGGGCGCARCGTAACATGACTTYACTAAGNACACYGAGCTNTYYYRGCAGGAGCCGGTGTGCATRCCYTTACCACGCGCCTGRRGCTTATCTGTCGGTAAAGYCANRRCGCCAACTGAAGAGTGCCTGGTACTTTGTRTAAATGCCYTTTCCGGGAGCATTNGATCGYTCRGNCCCGAGNTNNGCCATTAACYAGGCATTGYCTTTGCACGRCAACGGCCCAATGTCACGACCTCGGGACGGTATGTGGCANACTTTTTRRRTCNGACCCGGTTGTCCGAGATGGAGGCCCCAGCATCCTNACNCTGGRACTTATCGTAAAGACGATCGGGCAAAGCRYNNCCTNNNNGCRATRTCCGTTGGTGTCCANATCRAGTGACCAGATTTTGTACCTAGCATCCTACAGTGGCGAGTCATTACATTCAGTNYNAYGCGGYCTCGCANTGYRCATT

In521 ACGATTTACTCGTCRCCATANNGGCNNCCGGAGYCGTCGACCTAGACGGGTCTAGGAGGAGTACCCACTGACCGTNATCTACTGGCYATCACGCCTYTTGTCTAAGGRGCCCACTGCYCRGGRNNCATCTTGRNNANNNGCCRTRCTGCGACTNCAATGGCCTGGCAGCGTRCAGGAGGACCAANTGTAACGANNNNTTTGGACCTGRCYYTGGAACGGTAGACTNYAYAATACGGCAGTCNNYAARTANGTTNGRCACGCAGATCCTAGGYCTCANNNNGANNNNNNTGANRAACACGGATCACYCRTTGGGCATTGACTATTYCCAATGYCCGTGGRTAGTTTGGTTNNNGACTTAGNTTGRAGAACGGTGGAGCCANTCTGGAGCTGYTAGGGCGGGGCGGGCGCCGCAGGTCNGRAACCTNGATRCRCTACANTGCCGCCAGCTRRYCYTCAARCRCCCTCTCTYGCCTGCTGAACGGTNNATRNTTGGTTACATNCNGCTGYCAAYNNATCCTANCNGACACAGCTANNYATCCTCNTCTARACTCAAGTAAGAGTCTAGAGCCGACGCGTACNRTNNYAACACCGACGGGGCGAGGAAAGAACGCCTCCGGNTACNAACGGTGGACRTTCCCCTAGAAACAAGTGGTCGCTGAGGNTGAGCANGCCAATCTCCTCAATCYGCTCCCTTATGTGGATACCCCTGCCCCNGCTTACGNCAGTACYTAGCGGAYTGCGCCRNGCGGTGGNTACCAGGTGAATNNTNNCGCATGCNAATGNCAGGCACTACCGTAATCTAGCTGGCTGGTAGCTAGGAACTGATATTATGTGAGTACATGCCCCGYTYGNANTNCRGNTACAGCTAGCCCTGTACACAACGNAATTGTTCTTAGGCTTGCGAGTACGACTAAAATCGGAAAAACGGNCGTCTTGGTCCGGTCAGTCACTNNNGTTGCAAATTGTNGTAACYCGCNRYAGCCATGGCTCCTAAATTAGGAANNGCCCGCGGTGTAGGGGCGANCCGCGNCGRCGRRGACCGCCTAGCYGCAAGGGATAATCAGACGATTAGATTATGTCTGAAGGAACGCACCCCYCTYAACRNAGGTNAYAYGCTGTGGCTCCAYTTTCNGCAGCNNNNNGATTAGRCNCGTCCGGGGGCCGNTRAGTACTAGGGCCTNRGAACATNAATGCNCRRACRATGGGGCCAGCCANCCCGACAANTTCCNACANGNRNNRYCAGTTNGCTTGCTGCRRAGAATTTNNNNNGCCCGACGGTAACGCGACGGTATTTGAAAAGACATANGYRTGNGATCGGAYTCCNGRAATGTCATNTAGGGCCNNNCTAGCACTNACAYRYAAACRNCAGGYRCCTCAATANCTCTCCGRNYGNCTGACRCGANTRAAATGAGCTCGCCCCTGAAYGNAGGARGGGAAAAACYTCGGGACTCTGAGTACTNNNCGGCCCTARTGAACATAGAGCATCGCCCAGNNNGACATNTGGTCTNYGGGANNNNCYRTCCTCTGACCTNNCCAGTGGCTTCCGCNGGACTTTGTRYRRCCAGAAAGCGAACCTCCYRCNCCTCNCAAGTGGTTTTTCAAGTACCCTTCCTACRTCNCTCCGNNNRARATGCYCTATYNGTGCNRTTGNGAGCCCTATATCTATTANNYAGGTNNNNACTACTCTCGRACTCGTAGTACTCGATANAAGGTCGGGGRTCNCTATRTACAGGTGGACCATATTAGCCCGCTTNATTGAGATGNCATGGTCTGTTTGGANAYCGTCCGCTTGGTTAACTGCTCGAANCCCRTCCYNACGAGNCCCGRNGAGTGACGNTGAGNCCGNGGATYCTNACTGGACACYTTCYAAAGCGTCCAGAGGATACTTCGTTCNCTTTYCATTTACGCTANCCNATNNCGTAATRTGCCGGYRGCGYAGAGCTGGCACCGCAATGGATGTCTTGCAGAATGGCTCAAGCANCRGCGGCGCGTCACTATCGANNCCCNNNCANGRCNNCGGCAGCTAGCCRACYCACCCAATGGCTCGTGTCCCCACGANNNAGGCGGGATGRRNACGGAGACGTYATGANGRNNTCTACAYANRTTCCACRCGGTTCAGYNRTTGTNNRAGGRCGGTNCGGGYCCNNNNACCGNTATGGGGNGTAATTGGGNNRYTYGCGANNACCTRNGRAGTACTCGACCGCGAATCGGTGCAGNGTGGCGAGGGCGCCTTGCTTCTATATTAACATTGACNACGTAGATGCATGCCGAACTTTGCGGCRRGAAAGRGCGARNCCGTGGCAACGNGCTCTGCGACCNCATTAGTTANNNAATGTGTCARAGAAYGATYGTTCGTGTCCNYCCYCNNNNNGTGAAGCGCCTAAAAAGCYNRCTRCGCANAAGCCTGTNRAGTAAGGCCCRNTCTCCGGCGCACGCGGAGCGGTCGCGNNNNNNGGYTGTAGGCGANTYNRNRANTAGCRCNACTNCTAGAAACGGCCGGTACAGGNAACTYGCTGGTRNNYTACGACTTATYTAGCCCRCCCCGGAGCAANYNTNCTCRNCRTTAGGGGCTACTTCGGTGGCGTACATCGCAYNNNACCYAYGCAGCAGCAGCAGCATNNNACAATACTCTAGACGCCCACAGCATCTCGGNCTAATACTTCTCCNCACTCCTGATCACYGGTGGCCACTAGACGCCNNNTCCTGGATANYCGAAAYGGGCCTCCARAGNTCRYCATCTTACAAGACNNCYGATAGNRTGCGGTGTTNTATCCATGYNANYTCTGAGCNNCRGTGAYGNYGCRCGACCGCCGGTTTATGAACCGCACCAYYGGGYYTGCAGGGGAGGAGACGCGGCGCCGTAACRTNNGTGTCGACCTGTANNAGCYRRGRRAATGGGACCGCGNNCACGYGACCATANACYCCGCACNNACGCYGGCCYCAAGYGAGGGTGNCCACANCGNNCCCGGGCCTGAAAGTCGGGCATCTAGTTCTAGCCGAAATACCYYCCGACCTGCGCAYRAAGTTANCNCGCTNNCNGTANNNGGACCCACCGGNNNTGCGAGGATTANAGCANNYCCGNNGCTCCGTCGCAGCGGTATTAAAGCCTTACATCCTYTATGGGAGACGGRGTGANRCATTAGAGCACCNNNRRACCNNCCTGGACGCNNNNCGTGNNNAATANTCACTAGGNATGTGAGAGGARTTCGRGCACNAGYGTYNNTACCTCAGATTTTCGGACTAGCCATACANNNNNRCNGACRTTGGCAGCTCACAANCGGTAGCCCATCCCACTAGANGTTACTCCAGGGGACGCGRAAGCCYCNNGGCGTAATCAACCTNGTGTTGGTAGGACGANAAGAGTAATATAATCCCTATTTCGCAGGAATGGTCCNTGCCGAAGGAGTTATCACCAATGAAAGTGTYCGCGAGATGCRCTACNTGCCCNTTGTYAGTYTACAYNGCATAATANANTNGTCGCTGCCTCRTCATNNNNNNACGCAGTAGNCNCCAGARNCTAATTCRCCGACTACCCCGACGCCAGCCTCRYNCRNNNNNAGCTACTGTATCTAAATGGACTGTTCTCATTGTTGCGGGCGGGCTCCTCTTAACNNNRYAGCTATAACTCACAGGCAGTTTGAGAAATCTTACAYGCGGTAAACCGYAGRGCNAGGCTACACAGCCTGAGAGGCCANCCCATTGYRGATRRNTYGCNNAGTCTCGGCCTGGNTCGGGTNCATCGTGRRCGGACCGGTYCGCAGGCGGCGCACCTGATCGNNACCGCGTCGGGTACCTGCGACCTANGCGTTTGCGCCCGNGTGCCGTGGTCGTTCCGYGNAANTGATCCAGGGACTTGGATGCCCTTGTANGCRCCAYNGAATAGATTGTACCACGNCACACGACGGCNTAGGACGYCNGCRGTACCTTGTCTNAGCNGTCAAGTTTYCYCGTGTTGCCCCCRGGCAATCATNYGTAACGGGNNGGRGTYATGGCAGCTCGAATCAGGTGCGGYCTTGGNTCCCAARAAYTGCAGTCNNNNAYGCGCATGYCGAATGCACCGTGGCGNTGCTNNCGGATACTTGCGRCCGTCATANTGTTCATGGTGTTATGAGCTCTCCTTARGCGCGGACCRAANTCGCCACNRTYNTGNNNARNAGGCGCGARTGTRTCCAAAGCGATAGCACGGACNTTGAANGCGTRRATATTGCGGCNCNNTYCTATACAGGTCTAGGTACGGCTTCACACTGGCNNNNTATYYGGGNGNNYGCCGTNGCGCAAATTCRCNGGAGGGGNGYCAACACTYTGTNGNNGCGGGCTCNYRNGGTTACTAAATTGTGGCGTCTCYCTAACCGAANGNNAATTCTGCTACNTCGGAGGTNGCCGGCATGATGTCGCTTCACYCTTNANAYGTNTYGCANTGTAACGATANCAGGCNTCGCACNACANANAGATGACRAAACANGATACGCAGTACTGGTCGGCGTNNNCACCACGGRCCCTGAYNGTGTTTCGTAACAACACAACATYGGTCTCTGACTCTACCCGGGTGCAGACNYCTYNRTCAGCTGGGATTACYRGCCGAATAAGCCGNGTGTCNGGCCRACCCGAACCTAGCGAGTCGCGCACAANNCGGAGGGCACCACACATCNNNNNNNNACTCAGGGCGGAARCGCCAAAACACTCNRTNCCCACCTGTGGTCAGNNCNGNRAYCTAAGGTANGCCCCAYGAGCNATTGGGNNGCNGRATTTGGATGTCCGGTCCYATTCCTCGAACCGACGNTAAAGTNYGTGRAGANNNYGTTARCTTGACCCGCTGGACCGCATGTNGGTNCACAGCTACGTCGGTCTAATRGNACTTCCANNGNTTGCCTCCNAGCAGCNGGAGTTANTTGGCCCRCYCTCRRTNTGACATAAYNTACAGTNAGCCGATRRAARRCACTCGCYAGCATTTGCANCCACATGCGTCNNNGAYACTTCCATGTCTAACYGGCGGAATAGAGGTTCTCGATCTGTGATGRNNNGCCGTCACAGANNYTRCYGGCTRTYRNNTGGCACRGRCTGTGCCCNRNTTGCYCNNNAAGTTGCAAATNGRCCNTNACTTRGGGCRTCGRGANATAATCGCAATCGCCTCARGTCCTAGGAACGTGGGTTACTGATCGCTNRACCCCACCTGTCAAAGCNANCGCARCGNCTTCTTAACAAGTAACTAGCTTGAACNCTAGATANNGGGGGTGTTAAGNRNTACTRGNAGGGCAACCTTGGNTTGNNCGCGTGATCCYNAGACCRCTTYGTGGCANGGGNNNRGAGCCCCNRGGCTGAGCCCNNNGCCGTCCGCAAACNGGCNAGTNNGACCNNAGGCCGACGGGGGCGCANCGTAGCATRRCTTCACTNARAACACCNNNNTATTNTAGCAGGAGCCGGTGTGYATGCCCGTACCAYNCGCCNGAGGCTTATCTGTCRGYAAAGTCAAAACGCCAACTGAAGAGTGCCTGGTANNNTNTANAAATGCCCTTTCCGGGAGCATTNGATCGYTCGNTCCCGAGCTATGCCATTAGTTAGACATTGCYTTTGCACGGCANCGGCCCAATGTCACGACCTCGGGACGGTATGTGGCAGACYTTTTAGATCCGANCCGATTRCCCGGCACRNNGGCCCCAGCATCCTTACRTTGGGANNTRYCGTRAARRCGATCGGGCAAAGCRYNNCNTGCTAGCGGAGTCCGTTGGTGACCGNATCAAGTGAYCAGANNTTGTACCTAGCATCNTACARTGGCGGGTCATTACATTCAGTCCGACGCGGTNTNGYANTGYRNNTT

In525 NCGATTTACTCGTCGYCNTANNGNCNNNNNNNNNNGYNGNNCTAGNNGGGTCTARGAGGAGTACCCACTGACYGTGATCTACTGGCNATCACGCCTTTTGTCTRAGGGGTCNNNNGCTCGRGRTCNNNCTYRGNNNNRGGNNATNCNGCGCTTACAATGRYCTGGCAGCGTACAGGAGRRCCRNANNTANNGACANGTTTGGAYYTGGCTTTGGAACGGTAGNNNTYATAATACGGCAGTNTNYAAGTANGTTNGNYACGCAGATCCTAGGTNTCATGAGGARGYTNTTGANAAAYRCGGANCACNCNTTGGGCATTGATNNTTYCTAATGYCCGTGGATAGTTTGGTTGATGGYNTAGCTTGGAGAACGGTGGAGCCRTTCTGNAGCTGTTAGANYGGGGCGGGCGCCACNGGCCAGGAACCTAGGTAYATTACAGTGCNGCCAGCTAGCCTTNRAGAGNNNNCTCTTGCNTGNTGANYGGNNNATNNNTGGNTACNTTCCGCTGYNAAYGAATCCTANCNGACACAGCTAAGTATYNNNNTCTAAACTCAAGTAAGAGTCTAGAGCCGACGCGNNNNGTGTYAACACCGACGGGGCGAGGAAAGARCGCYTCCGGYTACCAACGGTGGANNNTCCCCTAGAANCRAGCGRYCRCTGAGNGTGNGCAGGCCAATCTCYTNNATCTGATTCTNTGAGCGNANNCCNNNNCCNCCGCTTACGACAGTACYTAGCNNANTNCGCCGTNCGGTGGRTACCAGGTGAATCCTNTTGCRTGCGAATGGYANRNACTACNGTRANCTAGCTGGCTGGTAGCTAGGAACTGATNTTATGTGAGTACATGCCCCGCYCGCAGTNCGGTTACAGCTAGANNNGTACACAACGGAATTGTTCTTAGGCTCGCGRNNNNNNNNNAGATCNNAAAAACGGCCGNCNTGNNCCNGNNNGTCACTRAAGTTGNNANTAGNAGTRACTNGCGGCAGCCANGGCTCCTAAATTAGGAATAGCCCGCGGTGTAGGGNCGANCCGCGTCGGCGAGRRCCGCYYNACCGTAAGGGATANTCANACGNTTNNNNTATGTCNGAAGGAACNNACTCCYCTTANCAAAGGTNACACGCTGTGGCTCCACCTTYNNCGGCCGGGGGATTNNGCACGTCCGGGGGCCGNNRAGTACTARGGCYTNRRAACGTGAATGNNNNGACGATGGGGCCAGCCNNCCCGACAAGTTNCCACANGGGGCRYCAGTTGGCTNGCTGYRRGGTATYTYTATGGCCCGACGGTAACGCGNCNGNNNYYNNAAAGACATAGGTRTGAGATCGGACNCCNNNAATGTCRNTTAGGNATCCACTAGCACTGNYRTACAARYNNCAGGNNCATCGGTATCTCTCCRGGYGNCCRRCRYGANTGTNGTGAGCTCGCCCCTGAATGRANNNAGGGGGRAACTTCGGGACTCTGNGNACTCGCCGACCCTAGTGAACATAGAGCATCGCNTAGTTTNAYANNTGGTYNNYGNGANNNNCCATCCTCTGACCTACCCAGTGGCTTCCGCNGGACNTTGTRYRRCCAGAAAGCGAACCTCCCGCACCTCCCNAGTGGTTTNNCAAGTAGCCTACCTACNTNNCYYCRAYGGAAATGCCCTATNNATGCTRTTGNGAGCNCTATATCTATTAACCAGGTCCGCANTGGNTTCGRNCTCTTAGTACTCRATNTAAGGTCGGGGATCAYTATRYRCAGGNGGAYYGTATTAGCCYRCTTCATTGAGATGGCATGGTCTGTTTGGATAYCGTCCGCTTGGTTAACTGCTCGAAGCCCRTCCTCACGCGNCCCGRTGAGYGACGGTGRGNCCGNRGATTCNNACTGGACACYTTCCRAAGCGTCCAGAGGATACTTCGNNYTCTTTTYATTTACGCTACCCAATTTCGTAATRTGCCGGTGGCGNAGAGCTGGCACCGCAATNGRNNNCTTGCAGAATGGYTCAAGCNTCGGCGGCGCGTCANNATCRANNCYCTNNCNCRGYGGCGRCAGCTAGCCNNCCCACCCRATGGCTCNNNNCCCCACGARGCAGGCGGGANGRRTACGGAGACGTTATGACGGNNTNNACACANRTTYCNCGTGGNYCAGNNNNTATGAAAGGNCGNTNCRGRNTCCTAGACCGATATGGGGYGTAATTGGAGTGTTCGCAAATACNNGAGRNGTNCTCGACCGCGAANCGGTGCAGCGTGGCNAGGGCGCCTNNNTNCTRTATTAACATTGACTACGTNGATGCRTGCNGAACTTYRCAGTNNNNAAGAGCRAGTCCGTRGCAACGGNNTCTGCGACCACATTAGTTACYTAATGCGTCARAGATTGATTGNNNNNGTNCNNCCNNNCGGCGYGAAGNNNCTAAAANGCTNGCTRCGCAAAAGCCTGTNRAGTAAGGCCCRNTCTCCGGCGCACGCAGCGCGGTCGCGNYAGCNGGTTGTRRGCGANTCNRCAACTAGCRCNNCNGCTAGAAACGGCCGGTACAGGGANCYYGCTGGTNGGTTACNNNTTRNTTAGCCCGCCCCGGAGCAATCGTCCTCGTYGTTAGGGGCTANNCCGGTNGYRTACATCGCATTATACCCAYGCAGCAGCAGCAGCGTCTTACAATACTCTAAACGCCCACAGCATCTNNNNCTANTNCTNCTCCNCACTCCTGNTCACCGGTGGCCACTAGRCGYCTGTTCCTGGATANNCRAAAYGGGCCTCCANAGNTCGCCRTCTTACAAGACYCCTGATRGNRTGCGAYGNNNTATYCATGYNACTTYTGAGCGTCGGTGNCGANGCACGNNCGCCGGTTTRTGAACCGYACCACCGGGCYTGCATRGNRGGAGACGCGGYNNCGYARCGCAGGTNNCRAYCYGYNTTAGCTAGGRRAATGGGACCGCGATCNNGYGANNNNNTACCCCGCAYGGACGCYNGNCTCAARYRAAGGTGNCYACATCGNNCCCGGGCCTGAGAATCGGGCAACGNNTTCNAGCCGAAATACCCCCCGANNTGCGCATAAAGTTACCACGCTGCCCNNNATGRGACYYACCGGGNTTGCGAGGATTAAAGCANNYCNNTTGNNCCGTCGCAGCGGTATTAAANNCTCANGTCCTTTGGGGNAGACGGAGTGANNCATTNRRGYRCCGTCRRAYCATCTGNCCTGCATCACGTRYGTAATAGTCACTAGGTNTNNNAGAGGAGNTCGGGCACCAGTGNCTTGACNNNNNNNNNTCGGRYTAGCCACCCNGCTCCRYAGACATTNRCAGCTCNCAAGCGGTAGYCYATCCCACTAGAGGYNACTNCAGGGGNNNNGRAAGCCNCAARGTRTAATCANYCTCGTGNTGGTCRRNNGARRAGAGTANTATAATCCCNATTTCGCAGGAANGGTCCATGCNGRAGGAGACATCACCAATGAAANTGTCCGCGAGNTGCGCTACNTGTCCTTTGTNAGTCTACAYTGCAYAATAAANTCRNCGCTGCCTCATCATGACCGTACGCAGTAGCCTCCAGAGCCTTANTCGCCGACTACCCCGACGCCAGCCTCGCTCAGCCNGAGCTANTNNATCTAAATGGACTGTCCTCNNNGTNGYNGGCGGGCTCCTCTTANCGTCRYCGCTATAACACACAGGCNNTGTGAGAAATCNTACACNCGGTNANCCGTAGGGCGAGGCTNCACAGCCTRAGRGGNCATCCYATTGTGGANARATCGCNCAGTCTCGNYCTRRRTCGGGTNCANNGTGAACGGACCGGTYCGCAGGCGGCCCACCTGATCGGAACCGCGNCGGGYNCCTGCGACCTACNCGNNNNNGCCCGGGTGCCGTGGYCRTTCCGYANAACTGATNCNGGGRCTNGGATGCCCTTGTAAGCAYNNNNGAATAGATTGTACCANNCCACACGACGGCCTAGGRCGCCNGCRGTANCTTRYYTYAGCGGTCAAGTTTCCTCGTNTTGCCTNCRGGCAATCATGNGTAACGGGRNGGRGTYATGGYAGCNCGAATYAGRTGCGGCCYYNGTTCCCAAAAANTGCNGTCATNNATGCGYACGTCGAATGCACCGCGGCANTGCTNGCGGGTACYNGCGNYCGTCATTGTGTTNATGGTGTTATGAGCTYTCCTTAGGCGCRRANCGANATCGCCACGATCTTGCGTAGAAGGCGCRAGTGTGNCCANAGCGNYARCACGNACNTTGAANGCGCGAATATTRCGGCTCACTCCTATACAATTYTAGGTACGANCYCACNCTGGCTRTATATCTGNGCNNNYGCCGNNRCGCAAATTCACCGGRGGGGNGYYRACACYTTGNTGAAGCGGGYTCNCACGGTTACTAAATTGTGNNNNYTCCCTANCCRAAGGNNARTTNTGCTACCNCGGARGTCGCCGGCATGNTGTCGCTNCACNCCCGATATGTATTGCAATGTACCGATACCAGGCGCCGYAYNACANANARATNACAAAACANRRYRCGCAGTACNGGNCGRYGNNNNCNCCTCGGGCCCTGATCGTRTTTCGTANYNACACAACATCRGTCYYNGACTYTRCYCGGGTGCAGACGTCTCAATGAGCTGGGATTNYCGGCCRAATNANNCGAGTGNCGGGCCAACCCGAGTCTAGCGAGTCGCGCACNNCYCGNAGGGCACCACACATCNNNNNNNNACTCAGGGCGGNAGCGCCNAAACANNCAGTYCCCACCTGTGGTNNACCCTAAAATCTAAGGTAAGCCCCANNNNCGATTGGGAGGCCGRATTTGGNTGTCCGGTYYYATTYCTCGAACCGACNGTAAAGTNCGTGAAGAANTTGTTAACTTGACCNGCTANNCCGCATGTCGGTACACAGCTACGNCRGTCTAATGGCACTTCCAANGTTTRCCTCCAAGCRNCRGGAGTTAGTTGGCCCRCNCTCAATCTGACATAAYCTACAGYRANCCGATGGAANNCAYTCGCCAGCATTTGCATCCACATGAGTCACTGATACTYCYANGTCTAACYGGCGGGATAGAGGTTCTCGATCTGTGANGRNNNNCCGTCACAGAGGTTACYGGCTNTNNYRTGNCACGGRCTGYGCCCAGATTACYCTCYAANNNNNNNATAGRCCYTNACTTAGNGCGTCRARANACAANCGCAATCGCTTCARGTCCTAGGAACGTGGGNTCNNRRTCGCNNNACCCCNCCTNNYAAAGCTACCGCANCGNCTTCTTAACRANTRRCTAGCATGNACCCTAGATAATGRGGGTGTTAAGNNNTACTNNNAGGGCAACCTTRNATTGYACGCATGATCCYNAGACCNCTTNGTGGCATAGANNCAGNNCCYCTAGGCNGRGCNCNAGGCCGTCCGCAAACNGGCCAGTTGGACCAGAGGCCGANRGGGGCGCANCGTAACATAGCATYACTNAGTACACCGAGCTATTCTAGCAGGAGCCGGTGTGCATACCCNTACCAYNCGCCTGGANNNTRTCTGTCGGTAAAGYCANRRCGCCAACTGAAGAGTGCCTNNTACTTTGTATAAATNCCCTTTCCGGGAGCNNCNGATCGYTCGGTCNNNAGCTATGCCATTAGTNNGGCANTGCNTTTGCACGGCAACGGCCCNNNGTCACGACCTCGGGACGGTATGTGGCAGACNTTTTRRNTCCGACYCGNNNGCCCGGCACAGAGGCCCCAGCATCCTNTNRCTGGAATTTANNNTGAAAGCGATCGGGCAAAGCRNNNCCTGCTGGCGGAGTCCGTTGNTGTCCNANTCAAGGGAYCARATTNNNNACCYRGYATCTTACAATGGCGRGTNNNTACATTCNNNNCGNCGCRGYNTCGYANTGYRCATT

In526 ACGATNTACTCGTCGYCNTRNNNNNCGCCGGAGYCGTCGACCTAGACGGGTCTARGAGRAGTACCCACTGACCGTGATCTACTGGCTATCACGCCTTTTGTCTAAGGRGTCCRCTGCTCRGGGTCCATCTTGRGTAYRNGCCATACTGCGCTTACAATGGCCTGGCAGCGTACAGGAGGACCAATTGYANNGRCNGNTTNGNACCTGGNYYTGGAACGGTAGAYYNYATAATACGGCAGTYTGTAAGTANGTNNGAYACGCAGATCNTAGGYNTCATGAGGARRTTYTNRANRAACACGGATCACYNRTTGRGCATTGAYTATTCCCAATGCCCGTGGATRGTTTGGTTGATGRNNTAGAYTNAAGAACGGTGGAGCCRTTCTGGAGCTGTTAGRNYGGGGCGGGCGCCRCNGGCCAGGAACCTNGGTACAYTACATTGCCGCCAGCTAGCCTTCAAGNRCCCTCTCTTGCCTGTTGAGTGGGNTRYRNTTGGNTACNTTCCGCTGYCAAYGAATCCTANCRGAAACAGCTAATCATCCTCNTCTAAACTCAAGTAARRGTCTAGAGNCGACGCATACNRTNTYAACACCGACGGGGCGAGAAANGARCGCNTCCGGCTACNAAYGGTGGACRTTCNCCTAGAAACAAGCGRYCGCTGAGNNTGAGCAGNCYAATCTCYTNNATYYGNTCCYTTRNGYGGAAGCCCCYRCCNCNGCTTACGACNGTACYTRGYGGATTGCGCCGTGCGNNGGRTACCAGGTGAATCCTATTGCATGCGAATGGCNGGNACTACCGTRANCTAGCYGGCTGGTAGCTAGGAACTGATNTTATGTGAGYRCRTGCCCCGTYCRCAGTNCRGNTACNGCTAGNCCTGTACNCRRCGGAATTGTTCTTAGGCTTGCGRGTACGACTAAGNTCGGAAAAACGGCCGCCNTGAGCCGGTCNGTCACTAAAGTTGCAAATTGTAGTAACYNGCRGCAGCCATGGCNYCTAAATTAGGAANNGCCCGCRGTGTARRGNCGRGCCRCGTCGGYRAGGRCCGCCTNRCTGCAAGGGATAATCAGACGCTTAGATNAYGTCTGAAGGRACGCACCCCTCTTANCAAAGGTNAYAYGCTNTGGCTCCACYTTYGRCRGCCGGGGGATTAGRCTCGTCCGGGGGCCGNTAAGTACTAGGGYCTNRGAACGTGAATGCNCRGACGATGGGGCCAGCCACCCCGACAAGTTCCYRNAGGGGGNATCAGTTGGCTYGCTGCRRAGAATTTNTATGGCCCGACGGTAACGCGNCNNYNYTTGAAARGACNTAGGTATGNGATCGGAYTCCNNNNATGTCRNTTAGGRNTCCACTANCACTNACACGYAAACATCAGGNNCATCGGTANCTCNNNGGGCGGCYRRYGYGAATGNNATNRGCTYGCCCCTGGANNAAGGAAGGGAAAAACYTCGGGACCCTGAGNAYTCTGCGRCCCTAGTGAACATAGAGCATCGCCCAGTNNAACATTTGNTCTCTANGAGCAGCCRTCYTCTGACCTACCCAGTGGCTTCCGCAGGACTTTGTACAGCCAGAAANCGAACCTCCCGCACCTCCCAAGTGGTTTTTCAAGTAGCCTNCCTACGTCGCYYCAACGAAGATGCTCTATYNGTNCTRTYGNNNGCCCTATATCTATTAACCAGNTCCGCACTGGTYTCGNACTCGTAGTNCTCGATATAAGGTCGGGGATCACTGTGYGCAGGNGGATCATATTAGYCYGCTTCATTGAGATGGCATGGTCTGTTTNGATAYCGTCCGCYTGGTTAACTGCTCGAAGCCCATCCTCACGNGNCCCGRNAANTGACGGTGGNTCCGNGRATCCTCGCTGGACAATTNCCRAAGCGTCCAGAGGATACTTCGTTCTCTTACCATTTACGCTAGCCAATTTCGTAATGTGCYGGCAGCGCAGAGCTNGCACCGCARTGGGTGTCTTGCAGAATGRYTNNNGYANCRGCGGCGCGTCACTATCGAGCCCCTGGCACGGCGNCGRCAGCTAGCCRNCYCACNCRATAANNCGCGTCCCCACGAGGCAGGCGGGATGAATATAGAGACRTYATGACGGGGTYTAYNCANRTTYCACRYGGTTCAGCGATTACGCAAGGGCGGTNCGGGYYCCYARACCGRTATGGGGYGTAATTGGRNNRTTCGCNAATACCTGAGRAGTACTCGACCGCGAAYCGGTGCAGCGTGGCNAGGGCGCCTTGCTYCTRTAYTAANATTGACTANGTNGATNCATGNGGANNTNNGCGGCRGNAAAGRGCGARNCCRTGGCAACGGGCTCTGCGNNNNCGTTAGTTACYTRNTGNGTCAAAGATTGATTGTTCGTGTCCNYCCCCGCGGCGNGAAGAAACTAAAANTGYNRCTRCGCATAAGCCTGTCGAGCAAGGCCCRNYNTCCGGCGCACGCGGAGCNNTCGCGCCAGYCRGNTGTAGGCGANTNNACAACYAGCGCANCNNCTAGAAACGGCCGGTACAGGGAACCNGCTGGTGGGYTACGACTTATTTAGCCCNCCCCGGAGNAANYNTNCTCRGCATTAGGGGCTACTCTAGTNGYRTACATCGCACTATNCCCACGCAGCAGCAGCAGCGTYTTRCAANACTCTARACGCCCACAGCATYTCGGNCTAATACTTCTCCNCACTCCTGRTCAYCGGTGACCACNAGRCGCCTGTTCCTGGATANYCGAAAYGGGCYTYCAGAGCTCGCCGTCTTACAAGACYCCTGATRGNRTGYGACGTTTTATCYATGTANNNTTTGARCGTCGGTRACGACGCACGACCGCCGGTTTATGAACCGCACCAYCGGGCTTGCAGRGNRGGAGACGCGGCGCCGYAACRYNNGTNNCGACCTGTNTYAGCYRRGAGAATGGGACCGCGATCACGTGACCNYNGACYCCGCATRGACGCYGGCCYCAAGCGNGGGTGNCCACAYCGATCCCRGRYCTGAGAATCNAACANCTNNTTCTAGCCGAAATACCYYCCGNYCYGYGTTTAAAGTTACCACGCTNNCCNNNATGRGACYYACCGGGATTGCGAGGATTANAGCAACYCNRTTGNYCCGTCGCAGCGGTATTNAANYCTCANGTCCTTTRNGGGAGACNGAGTRAAACACTCGAGCACYGNNRRACCRTCCTGNACGCGNCACGTGTGTAATANTCAYTRGGTATGNNAGAGGAGTTCGGGCRCCNGTGTYTTGACCTCRRRTTTTCGGRYTAGCCATACAGCTCCGTAGACATTNRYAGCTCACAANCGATAGCCCATCCCNCTAGAGGCGACTCCAGATGACGCGAAAGCCCCAAGGCGTAATNANYCTCGTGTTGGTNGGACGARNAGAGTAATATAATCCCTATTTCGCAGGAATGGTYCRTGCCGAARGAGTTATCACCAATGAAAGTGTYCGCGAGATGCGCTACNTGCCCTTTGTYAGTYTACAYTGCATAATANANTCGTCNNTGCCTCATCATGATCGTACGCAGTAGCCTCCAGAACCTAAATCGCCGGCTACCCCGACGCCAGCNNNRCNCAGCCNGAGCTACTGTATCTAAATGGACTGTTCTCATTGTAGTTGRCGGGCYCCNCTTANYGTCRYNGCTATAACACACAGGCAGTTTGAGAAATCNTACAYGCGGTAATCCGTAGGGCGAGGCYACACAGCCTRAGAGGCCATCCTATTGTGGATRRRTYGCNCRGTCTCGGCCTGGATCGGGTCCATCGTGNRCGGACCGGTYCGCAGGAAGCNCACCTGATCNGAACCGCGACGGGTACCTGCGACCTACCCGTYYGCGCCCGGGTGCCGTGGTCGTTCCGYGNAACTRNTCCNGGGACNTGGATGCCCTTGTAAGCAYNATGGAATAGATTGTAYCACGCCACACGACGGCCTARTAYGCCNGCRGTACCTTRYYTTAGCGGTCAAGTTTCCTYGTGTTGCCCCCRGGCAATCATNCRTAANRGGRNGGGGTTATGGCAGCTCGAATCAGRTGCGGTCTCTGNTCCCAARAAYTGCNGTYATNNATGNNTACGTCGAATGCACCGCGRCRTNNCTNGCGGGTACTTACGGYCNTNNNNGYGTTYATGGTGTTATGAGCTCTCCTTARGCGCRRACCGATATCGCCACGATCNTGCNNAGAAGGCGCRARTGTGNCCAAAGCGNTAGCACGGACYTTGAAAGCGYGAATATTACGGCTCACTTCTATACAGGTYTNRGTACGRCYTCACACTGGCTRTATATCTGGGNGACCGCCGTYGCGYAAATTCACNGGGGGGGAGYCANCACTYTGTYGNNGCNRRCTCTCACGGTTACTAAATTGTGGNGTCTCYCTAAYCGAAGGRRARYTNTGCTACGTGRGARGANGCCGGCATGRTGTCGCTNCACTCCCGATATGTAYTGCAATRYANCGATACYAGGCGCCGYACGACANANARATGACGAAACAARRYRCGCAGTACGGRACGACGTAGCCNCCTCGGACCCTNATCGTGTTTCGTAACAACAYAACNTYRGTCTCTNACNNNRCYYGGGTGCAGACGTCTCAGTCAGCAGNGATTACCGGCCGAATRAGCCGNGNGTNGGGCCAACCCGARYCTNNNNRGTCGCGCACNNCNCGAAGRGCRCCACACATCNATNTTTCACNCGGGGCGGARGCGCYAAAACACTCNRTCCCCACCTGTGGTCAGNNCNRAGATCTAAGGTAARCCCCATGNRCGATTGGGAGGYCGRATYYNRATGTCCGGTYYTATTCCTCGAACCGACGGTAAAGTATGTGRAGAATTTGTTANCTTRACCCGCTAGACCGCATGTYGGTNYACAGCTRCNTCRGTCTARTGGNACYTCCAANGTTTGCCTCCAAGCRACRGGAGTTANTTGGCCCRCCCYCRRTNTGACATAACATACAGNNARCCGATGGAARRCACTCGCYAGCANTTGCAYCCACATGNGTCCCTGAYACTYCYRTGTCTRRCTGGCGGAATAGAGRTTCTNGATCTNTRATGNNTAGCCGTCACAGATRYNGCNGGNTGTCAYANRGCACRGRCTGTGCCCNRNTTGYYCTCYAAGTNGYARRNAGRCTCTGRCTTRGGGCGTCGRGACRTAAGCRYAATCGCYTCARGTCCTAGRAACGTGGGATCCTRRTCGCTNRACCCCACCTNTCAAAGCTACCGCAACGANTYCNNNACGACTAACTAGCTTGAACCCTAGATAACGRGGGTGTTAAGGGATACTGGCGGGGCNNCCTTGGATTGTNCGTATRATCCCAAGACCACTTTGYGGCATGGRRRCAGRGCCYCNRGGCTGAGCCCTRGNCCGTCCGCAACCTNRYCARYTGGRCCRNAGGCCGACAGGGGYGCARCGTAACATRGCTTNACTAAGNACACTGAGCTATTYTAGCAGGAGCCGNTGTGCATGCCCNTACCACGCGCCTGRRGCTTATCTGTCRRTACAACCATGGCGCCAACTRRAGAGTGCNTNNTANNYTNTRNNNATGYCCTTTCCGGGAGCATCGGATCGYTCGGNCCCGAGCTATGCCATTAACNNGGCATTGCCTTTGCACGGCAACGGCYYRATGTCACGACCTCGNRACGGTATGTGGCANACYTTTTAGATCCGACTCGRTTGCCCGGCACGGAGGCCCCAGCATCCTTACRYTGGGATTTATCGTRAARRCGATCGGGCAAAGCGCTACCTNNNNGCGGAGTCCGTTGRTRTCYGRATCAAGTGACCAGATTTTGTACCTAGYATCYTACARTGGCGAGTYATYRCATTCAGTCCGNCGCRGYCTCGYAGTGCRCATT

In527 ACGATTTACTCGTCRCCATRNNGGCCGCCGGAGCCGYNGNNCTAGRCGGGTCTARGAGGNNTACCCACTGACYGTGATCTACTGGNTATNNCGCCTTTTGTCTAAGGGGCCCACTGCYCGRGRTCYATCYTGGNNAYNGGCCATRCNGCGCTTACAATGGCCTRRCAGCGYACAGGANGACYAAATNYAACGRNNGGTTTGGACCTGGCCTTGGGANGRTAGACTNYATAATACGGCAGTNTGTAARTACGTNTGANACGCAGATCNTAGGTCTCATGAGGAAGCTTTTGACRAACRCGGATCRYCCATTGGGCATTGAYTATTCCCAATGCCCGTGGATAGTTTGGTTGATGGTGTAGNTTGNNNAACGGTGGAGCCANTCTGGAGCTGTTAGANTGGGGCGGGCGCCGCAGGYCAGGAACCTNGGTAYANTACANTGCCGCCAGCTAGCCTNNNNGNRCCCTCTCTTGCCNGYTGARNNNNCTRYATTTGGTTACATTCNGCTRTCAATGAATCCTAGCGGAAACAGCTAAGTATYCTCGTCTAAACTCAAGTAAGAGTCTAGAGYCGACNCACACGRTNTTANCACNNACGGGGCGAGGAAAGARCGCYTCCGGCTACGAACGGTGGACATTCCCCTAGAAACAARYGNNCRCTGAGGCTGAGCAGGCCAATCTCYTCGATYYGATYCTYTGAGYGGAAGCCCCCACCNNNGCTTACGNNAGTANTTAGCNNATTNCGCCGTGCGNNGGGTACCAGGTGRNTCCTATTGCGTGCGAATGNTCGGNACTACCGTAATCTAGCCGGCTGGTAGCTAGGAACTGATATTATGTGAGTACATGCCCCGCTCGCAGTACAGATNCNGCTNGCCCTGTACACRRCGNAATTGTTCTTAGGCTYGCNNGAACGACTAARATCGGAAAAACGGCCGYCNTGGTCCGGTCAGTCACTAAAGTTGCAAATTGTAGTAACTCACGRYAGCCATGGCNYCTAAATTAGGACNNGCCCGCGGTGTARRGGCRGGCCGCGTCGGYRAGRGCCGCCTNRNCGCANNGGATATTCACACGATTAGATTATGTCTGANGGAANGCACCNCCCTTACCRNAGGTNAYACGCTGTGGCTCCACCTTCNGCAGCCGGGGGATTAGRNNCGTCCGGGGGCCGNTGAGTACTAGRGCNTNRGAACGTGNATGCNCRRACGATGGGGCCAGCCNNCCCGNNNNGTTCCNACANGNRGGGCCAGTTGGCTNGCTGCRRRGNATTTYNATGNNNNGACGGTAACGCGACGGTNNYYNRAAAGNNNTAGGYGTGNGATCGGATTCCNGAAATGTYATTTARGNNTYNACTANCACTGACNYRNAAACRNCAGGCGCNTCNNTNNCTCTCCGRNYGRCTGACGCGAATGAAATGRGCTCGCCCCTGRANNAAGGAAGGGRRRAACYTCNNNNCTCTGNNNACTCGCCGACCCTAGTGAANATAGAGCATNGCGTAGTTTNACATNTGGTCTCTGNGANNNNCCATCTTCTGACCTACCCAGTGGCTTCCGCNGGACTTTGTRYRRCCAGAAANCGAACCTCCCRCACCTCCCAAGTGGTTTTTCAAGTANCCTACCTACRTYGCYCCGACGAAGATGCTNNRYNNRTGCTGTTGTGARCNNNATATCTATTAACYAGGTCCGCATTGGGCTCGANNNCGTAGTACTCGATATRAGGTCGGGGNTCRCTATNNNYAGGTGGAYCRTATTAGYCYGCTTYAYTGAGATGGCATGGTCTGTTTGGATATCGTCCGCNTGGTTAACTGCYCGAANCCCGTCCYCACGNGNCCCGRTRAGTGATGGTGGGTCCGNGGATYCTCRCTGGACANTTTCCAAAGCGTCCAGAGGANACTTCGTTYTCTTTNCATTTACGCTANCCAATTTCGTAATRTGCCGGCAGCGTAGAGCTGGCACCGCARTGGRTGTCTTGCAGNACGGCTCAAGCRNCRGCGGCGCGTCACTATCGAGCCYCTGGCANGGCGGCGGCAGCTAGCCGTYCCACCCRATAANNNGCGTCCCCANNNNGCAGRCGGGATGAATAYRGAGACGTCATGACGGNNTYTANNCACAYTYCACRCGGNYCNGYNRTTRCGCAAGGGCGGTGCGGGCNCCYARACCGNTATGNGGCGTAATTGGRGTGNTCGCARATACNTGANRAGTACTCGACCGCGAATCGGTGCAGCATGGCNNGGGCGCCTYGCTTCTRTATTAACRTTNNNTACGTTGATGCATGCGGAACTTCGYAGCAAGAARGAGCGARNCCRTRGCAACGGGCTCTGCGATAACATTAGTTACNTAATGCGTCARAGATTGATTGTTNNTGTACGCCCCCGCGGAGYGAAGNRNCTNAAARNNTAGCTGCGCAANAGCCTNNAGAGTNNGGCCCRNTCTCCGGCNNNNGCGGANNGGTCGCGCCAGCCGGYTGTRRGCGANTCNRNRAYTAGCGCANCTNNNAGANACGGCCGGTACAGGGARCCYGCTGNTAGGTTACGACTTAAYTAGCCCACCCCGGAGNAATCNTNCTCGTCGTTAGNGNCTNCTCCGGTGGCGTACATCGCACTANACCYACGCAGCAGCRGCAGCGTCTTACAATACTCTARACGCCYACAGCATNTCGGGCTAATACTTCTCCAAACTCCTGATCANCGGTGGNCACTAGRCGCCGTATCCTGGATAACCGAAAYGGGCCTCCAAAGGTCGCCRTCYTACAAGACCCCTGATGNNRTGCGRTGTTTTATYYATGTCNCTTCTGAGCGTCGGTGAYGNTGCACGAYCGNNGGTTTATGAACCGCACCAYCGGGCTTGCAGRGNRGGAGACGCRGCGCCGTAGCGCAGGTGTNGACTTGTANNNGCCGAAAGTGYGGGRCCGCGATCACGTGACCNYNTACTCCGCAYGGACGCYRGCCYCAANNGRGGGTRTCCACATCGATCCCGGGCCTGAGAATCNAACANCNCCTTCTAGCNGRRNTACCCCCCGAYYYGYGCATAAAGTTANCACGCTNNCCGTAATGGGACYYACCGGGNNTGCGAGGATTANAGCAACYCCGTTGNTCCGTCGNNGCGGNATTAAAGCCTYACRTCCNYTRNRRNAGACNGRGTGACGCATTNRRNCACNNNNRRAYCGTCCNGCACGCGTCACGTGTGCAATANTCACTAGGTNTGTGAGAGGAGTTCGGGCACCAGCGTYTTNACCTCRRRTTTTCGGACTAGCCACCCNGCTCCGYAGACRNNGGCAGCTNNNAAGCGRTAGYCNATCCCACTAGANGNNACTCCAGRNGNNNNGAAAGCACCAAGGCGTRAYCANYCTCGTGTTGGTAGRACGAGNAGAGTAATRTAAYYCCTATGTCGCAGGNNTGGTCCANNCCGGAGGAGNYATCACCAATRAAARTGTCCGCGAGATGCGCTACNTGYCCTTTGTNAGTTYANACTGCAYAATANANTCGTCGCYNCCTTATCNYGACCGTACGCAGNNGCCTCCAGAACCTNAATCGCCGRCTACCCCGACGCCAGNCTCRCTCAGCCTGAGCNACTGTATCTAAATNGACYGTYCTYATTGTAGTTGRCGGGCCCCNNNTANYGTCRYAGCNANRACNCACAGGCAGTTTGAGAAATCNTACAYGCGGTAANCCGYANNGCGAGGCTNCNCAGCCTRAGRGGCCATCCTATTGYRGATARRYYGCGCAGTCTCGNYCTRRATCGRRYCCRYNGTGNRCGGACCGGTTCGCAGGCGGCNCACCTGATYGNNACYRCGTCGGGYNCCTGCGACCTAACYNTNTGCGCCCGGGTGCCGTGGCCGTTCCGYATAACTRNTCCNGGGACNTGGATGCCCTTGTAAGCAYCATGGAATAGATTGTACCRCGCCACACGACRRCCTAGNACGYCTGCRGTNCCTTRYYTYAGNGGTCARGTTTCCTCGTGTTGCCNYCGGGCAATCATGCGTAACGGGNNGGGGTTATRGYAGCNCGAATCAGGTGCGGTCTNNNNTCCCAAAAACTGCNGTCATTTATGCGYACGTCGAATGCACCGCGRCRTTGCTTGCGGNTRCTNGCGGCCRTCATAGYGTTYATGGTGTTATGAGCTCTCCTTAAGCGCGGACCRAAATCGCCACNRTYATGCNNAGAAGGCGCGARTGTGTCCAAAGCGNYARCACGGACYTTGAANGCGCRRATATNACRGYTCACTYCTATACAGGTTTAGGTACGRCYTCACACTGGCNNNNTATCTGRGNNACNGCYGTCGCGCAAATTCAYGGGGGGGGAGYCAACACTCTGNTGNNGCGGGCTCACACGGTTACTAAATNGTGGCGTCNCCCTNTCCGAAGGNNAATTNTGTTGTCNCGGAGGNCGCCGGCATGRTGTCGCTTCACYNNNNATATGTNTYGCAATRTAACGATACCAGGCNTCGYAYGNCNTNNNGATGACRAAACANGATACGCAGNNNNGGTCGGTGAAGCCACCTCGGACCCTGATCGTGTTTCGNNNNRACACAACNTYAGTCNNNGACTYTRCYCGGGTGCAGACGTCTYAGTCAGCTGGGATTNCYRGCCGAATRAGCCGAGTGTCGGRNNAACCCGANNCTAGCGAGTCGCGCACNNCNCGGAGGGCACCACACATCNATNTTTCACTNRGGGCGNNRGCGCCAAAACACTCNATCCCCACCTGTGGTYAGCCCARAGATCTAAGGTAANCCCCACATGCGATTGGGAGGCCGAATYYNRATGTCCGGTNYTATTCCTCGAACCGACNRTAAAGTAYGTGAAGAATTNGTTANCTTGACCYGCTAGACCGCATGTYGGTNCACAGCTGCCYCGGTCTARTGGCACTTCCAACGTTTGCCTCCNAGCGNCAGNAGTTNGTTGGCCCACCCCCNNTNTGACATAACANNCAGYAARCCGATAAARGGCACTCGCCAGCATTTGCACCCAYATGCGTCCCTGATACTTCCATGNCNAACYGGCGGAATAGAGRTTCTCGATCTGTGATGRNTAGCCGTCACAGATGTAGCYGGCTRTTGCATGGCRCGGACTGNGCCNNNNNTGTCCTCTAAGTNGYARATAGACCCTGACTTAGGGNGTYGAGACAYNAGCGCAATCGCNTCANGTCCTNRRAACGTGGGTTCCTGATCGCTNRACCCCACNNNATAAAGCTACCGCANCGNNTNCNNNACGNCTRRCTAGCTYGNNYYYYAGATAACGRGGNTGTTAAGNRNTACTRGNRGGGCATCCTTGGATTGCNCRCGTNATCCTTAGACCNCTTYGTGGCATRGRGGCAGGGCCTCNNGGCTGAGCCCTRRGCCGTCCGCAAACGGGCCAGTTGGGCCRNAGGCCGANAGGGGYGCAACGTAACATRGCTTNNCTARGAACACCGAGCTATTYTAGCAGGAGCCGGTGNGCATRCCCGTACCACGCGCCTGRRGCTTRTCTGTCGGTANARCCATGGCGCCAACTRRAGAGNNCCTGGTACTTTGTNTARATGCCCTTTCCGGGAGCATTNGATCNYTCGGNNCCGAGCTATGCCATTARYNRGRCANTGCCTTTGCACGGCAACGGCYYGATGTCACGACYTCGGGACGGTATGTGGCAGACTTTTTAGATCCGACTCGATTNCCCNGCAYRGAGGCCCCAGCATCCTTNCGCTGGAATTTAYCGTAAAGACGATCGGGCAAAGCANNNCCTGCTGGCGNNGTCCGTTGGTRTCYRAATCAAGTGACCAGATTNNGNACCTAGYATCYTACAGTGGCGAGTCATTACATTCAGTCCGACGCGRYCTCGCANTGYRCATT

In534 GCGATTTACTCGTCGCCATNNNGGCCGCCNNNNYCGTCGACCTAGRCGRGTCTANGAGGNNTACCCACNNNCCGTGATCTACNGGNNATCACGCCTTTTGTCTAAGGNGNCCNCTGCTCGRGRTCYGTCTTGGGTAYRNGCCRTGCTGCGCTTNCAATGGCCTGGCAGCGTACAGGAGRRCCRAATGYAACGRNNNNTTNNNNCCTGGCTTTGGAACGGTAGANNTNAYAATACGGCAGTCTNNAAGTANGTTNGRYACGCANATCCTAGGCCTCATGAGGANNNNNNNNNCRAACACGGATCRYCCATTNNNCATTGAYTATTCCYAANNYCCGTGGRTAGTTTGGTTGNTGNCTTAGNTTGRAGAACGGTGGNGCCANTCTGGAGCTGTTAGAGNGGGGCGGGCGCCACNNNCCAGGAANNTNGGTAYACTACANTNCCGCCAGCTRGYYYTCAAGAGCCCTCTCTNGCCTGTTGAGNNNNCTGCATTTGGTTACCTNCNGCTGNCAATGAATCCTAGCGGANACRRCNAANYATYCTCNTCTAAACTCAAGTAARAGTNTANNNNCGACGNNCACGRTNTNAACACCGACGGGGCGNNNAAAGAGCRCTTCCGGCTAYCAAYGGTGGACATTCCCCTNNAAACNAGTGGTARCTGAGGNTGAGCANGCCAATCTCCTNNATCNGNTNCTYTGAGCGCANRCCNNYRCCNCCGCTTACGACAGTANCTAGCGGANTGCGCCGNNCGGTGGGTACCAGGTGGCTCTTANTGCRTGCGAATGGCNGGAACTACCGNAATCTAGCYGGCTGGTAGCTAGGAACTGATATTATGTGAGNNCNTGCCCCATCCRCAGTNCRGNTACAGCTAGCCCTGTACANNNCGGAATTGTTCTTAGGNTNGCGRATNYGRNTRAGATCGNAAANNNNGNCGTCTTGNNNCGGNNNGTCACTRAAGTTGYRANTNGNAGTAACTCRCGGCAGCCATGGCTCNTAAATTNGNNNTAGNCYGCGGTGTAGGGGCGGGCCGCNNCGGYRAGGRCNNCCTNNNNNNATCGGNNNNTCANACGNTTNNNNTATGTCNGAAGGRACGCACNCCTCTNNNCAAAGGYCACACGCTGTGGCTCCACTTTCGNCNGCCGGGGGATTAGRCNCGTCCGGGGGCCGNTNAGTACTNGGGCCTNNGGACGTGAATGCNCRRACNNNNGGGCCAGCCACCCCGNNNNGTTCCYACARGCANNNNCNNTTGGCTYGCTGCRRNGNATTTYTATGGCCCGACGGTAACGNGACGNNNNCCNNAANGACNTAGGTRTGNGATCGGATTCCTRANATGTYATNTAGTRNNCCACTAGCACTGACRNNCAAACATCAGGCGCATCGGTANCTCNCCGGGCGGCYRRCGYGAATGNNATNRGCTCGCCCYTGAANNAAGGARGGGRRRAACNTCRRGACTCTGRGNACTCGCCGNNNCTAATGAACATAGAGCATCGCNYARTTTRACATNTGGTCTCTGGNNGCARCCNTCNTCTGACCTACCCAGTGGCTTCCGCNRGACTTTGTNNNNCCAGAAAGCNAACCTCCCGCACCTCCCNAGTGGTTTNNCAAGTAGCYTACCTACNTYNCYNCRNNNAAGATNCTCTATYNRTGCTRTTGNGAGCCCTATATCTATTAACCNGGTNNNNACTRNTYTCGRRNTCGTAGTACTCRATNTAAGGTCGGGGATCAYTATATACAGGAGGACCATATTAGCCNNCTTYAYTGAGATGGCATGGTCTGTTNNGATATCGTNCGCYTGGYTAACTGCTCGAANCCCATCCYCACGNGTCCCGATRAGYNAYGGTGGGTCCGGRGATNCTCACTGGACACYTTCCAAAGCGTCCAGAGGANACTTCGTTCNCTTTCCATTNNNNCTAGCCANNTTCGTAATATGCYGGYRGCGCARAGCTGGCACCGCAATGGATGTCTTGCAGAACGGYTNTTGCRNCNNCGGCGCGTCACNATCNAGCCCCNNNCACGGCNNCGGCAGCTAGCCGACTCACCCRATRRNNCGYGTCCCCACGAGGCANGCGGGATGAATACGGAGACGTYATGACGGGGTNNAYNCACANTNCNNNNGGTTYNGYGATNNTGCAAGGRCGGTNCRGRNNNCCAAACCGNTATGNGNNGTAATTGGRGTRTTCGCRANNACCTRNGAANTACTCGACCGCGAANCGGTGCAGCGTGGCNAGGGNNYCTYGCNTYTRTATTAACATTGACAACGTAGANGCATGYNGAACNTTGCGGCNNNNAAGAGCGARTCCGTGGNNACGGGCTCTGCNAYNACATTAGTTACCTAATGCGTCAAAGRTTGATTGTTCGTGTNNNNCCCCGCGNNGNNAAGNNNCTAAANAGCTNGCTRCGCAANAGCCTGNNGAGTAAGNCCYAATCTCCGGCGCACGCGGAGNGGTCGCGNNNNNNGGYTGTRRGCGANTCNRCAACNAGCRCNNCTNCTAGAAACGGCCGGTACNGGGAACCTGCTGGTNGGTTACRRCTTAANTAGCCCNCCCCGGAGCAATCGTCCTCGTNNTYNGGGGCTNCTNNNNTGGCGTACATCGCANTANACCCACGCAGCAGCGGCAGCGTCTTNNAANACTCTARACGCCTACAGCATCTCGGNCTAATACTTCTCCTCACTNCNNATCACNGGTGGCCACTAGACNNCTGTTCCTGGATANYCGAAACGGGCCTCCARAGNTCGCCRTCYTACAAGACYCCTGATNGTGTGYGATGTTTTNTCCATGTNAACTYTGNGCGTCGGTGNNNNYGCACGAYCGNNGGTTTATGAACCGCACCAYCGGGYCTGCAGAGTGGGAGACGCGGNRYCNNNRCGCAGNNGTCRACCTGYATYAGCYRGRRRNRTGGGNNNNNNATCACGTGACCNYNTACTCCGCAYGGACGCYNGCCYCAAGNRRGGGTGTCCACATCGNNCCCGGGCCTGARARTCGRRCAACNCCTTCTAGCYGNNNTNCCNNCCGAYCCGTGNNTAAAGTTANCACGCTNNCCGTAATGGGACNNACCRGNNNTNNGAGGATTAAAGCAACCCCGTTGCTCCGTCGCAGCGNNNNNAAANYCTCANGTCCTYTGGRRNAGACGGRGTGAAACATTNRRNCRYYGTCGGACCNNCYNNNNYGCRNCACGTNTGTAATNGTCACTAGGTATGTTAGAGGAGNTCGRGYRCCAGYGNYTTGACCTCRRRTTTTCGGACTAGCNATACAGCTCAANNGACRTTGGCAGCTCACAANCGGTAGYCNATCCCACTAGNNGNNACTNCAGATGACGCGRAAGCATCAARGTNTAATNANYCNNGTGTTGGTARRNNGAGAAGAGTAATATAATCCCTATTTCNCAGGAATGGTCCRTGCCGRAGGAGNNATCACCAATGAAARTGTCCNCGRGATGCGCNACNNGTCCTTTGTTAGTTTNCAYTGCAYAATANACTCGTNNNNNNCTNNNCATGRCCGTACGCAGNAGNCYCCAGARCCNTANNCGCCGRCTACCCCGACGCCAGCNNNRCNCAGCCAGAGCTACTGTATCTAANNGGACTGTYCTCATTGTNGYNGACNGGCYCCTCTTAANGNYGTCGCTATAACACACAGGCAGTTTGAGAAANCNTACANGCGGTAAACCGYAGRNCTAGRCYACACAGCCTNAGRGGNCATCCNRYTGYRGATAGATYGCTCRGTCTCGGCCTGGRTCGGGTCCAYNGTGRGCGGACCGGTYCGCAGGCGGCGNNCCTGATCGGAACCGCGTCGGGCCCCTGCNACCTACNCGTYYGCGCCCGNGTGCCNTGGNCGTTCCGYRTAANTGATCCAGGGACTNGGNTGCCCTTGTAAGCAYNCNNGAATAGATNNNAYCRCGCCACACGACGGCNTNGGACGYCAGCAGTACCTTRYYTNAGCGGTCAAGTTTCCTCGTGTTGCCCCCRGGCAATCATGCGTAACGGGRNGNGGTYATGGYRGCNCGAATYAGGTGCGGCYYCTGNTCCCAAANANTGCNGTCATTTATGCGYAYGYCGAATNNACCGCGGCATTGCTTGCGGGTACYNGCGGCCGTCATAGYGTTYATGGTGTTATGAGCTCTCNNNNRGCGCRRACCRAAATCGCCACNRTYTTGNNNAGAAGGCGCRARYGTGTCCANAGCGNTAGCACGGACYTTGAAAGNGCAGATATTNCGGNTCACTYCTATACAATTNNNNGTACGANCYCACNCTGGCTATATANCTRGGNGACYGCCGYCRCNNNNNTTCAYGGGGGGGGAGYCAACACTYTGTYGNNGCGGNCTCNCACGRTTACTAAATNGTGGCGTCTCCCTAAYCRAAGNNNAATTNTGTTRYCTCGGAGGACGCCGGCATGGTGTCGCTTCACTCCCGATANGTATTGCANTNTAACGATACTCGGCGCCGTAYGACATATAGATGACAAAACANRRYRNNNAGTACNGGNCGNNNNAGCCACCTCGGACCCTNNYNGTRTTTCGTANNRACNNNACNTCNNTCTCTGANTTNRCYCGNGTGCAGACNYCTCARNNNNNTGGGATTAYCGGCCGAATRAGCCGNGNGTNNGRNNAACCCGAACCTAGCGNGTCGCGCACNNCCCGRAGRGCRCCACACATNTATATTTCACTCRGGGCGGARGCGCYAAAACANTCNATNCCCACCTGTGGTNAGCCNNAAGATTTAAGGNNNNNCCCAYRNGCGATTGGGAGGCCGAATCCCAATGTCCGGNNTTATTYCTCGAACCGACNANNAAGTAYGTGAAGAANTTGTTAACTTGACCYGCTAGANCGCATGTYGGTNCACAGCTGCCTCGGTCTARTGGCRYNTCCAANGTTTGCCTCCAAGCGGCAGGAGTTANTTGGCCCACCCTCRRTNTGACATAAYNTACAGCAARYYGATNNARAACACTCGCNNGCATTTGCANCCACATGCGTCNCTGATACTCCTRNGNCTAACTGGCGGAATAGAGGTTCTCGATCTGTGATGRNNARTCNNNNCAGATGTAGCYGGCTRTTGYRNRGCACGGGTTGYGCCCAGATTNNCCTCYAAGTTGCAAATAGACYYTGAYTTGGGGCGTYGAGAYATAATYGCAATCGCYTCAGGTCCTAGGAACGTGGNTTCCTGATCGCTNGANCCCACCTGNNAAAGCTACCGCARNGNCTTCNNNACAAGTAACTNGCTYGAACNCTAGATANNGRGGGCGTTCNNGGAYNCTGGCAGGGCNTCCTTRNATTGCNCGCNTRATCNNNAGACCRCTTTGTGGCANGGGNNCAGRGCCYCTAGGCNGRGCCCNNGNCCGTCCGCAANCNNGYCANNTGGGCCAGAGGCNNACNNNNNNNNARCGTAACATNRCTTCACTARNNACACYGAGCTNTYNTAGCAGGAGCCGGTGTGCATACCCTTACCACGCGCCTGAGGCTTRTCTGTNRNTAAAGTNNNNNCGCCAACTRRAGAGTGCCTGGTACTTTGTRTARATGCCNTTTCCGGGAGCATTGGATCGNTCGGTNCCGAGCTATGCCATTAGTNAGGCNTTGCNNNTGYRCGGCAACGGCNNRATGTCACGACCTCNNRACGGTATGTGGCANACYTTTTNRRTCCGACCCGNTTGYCCGNNANGGAGGCCCCAGCATCCTTACGYTGGGAYTTAYCGTAAAGACGATCGGGCAAARCATGTCCTNNNNGCGGAGTCCGTTGGTRTCYRAATCRANNNACCAGNNNTTGTACCTAGCATYYTACARTGGCGRGTCATTACATTCAGTCCGACGCGGTCTCGCANTGYRNNNN

In535 GYGATTTACTCGTCRCCATRNNGGCCGCCGGRRCCGNNGACCTAGACGGGTCTARGNGGAGTACCCACTGACCGTGATCTACTGGCCATCACGCCTTTTGTCTNAGGGGYCCACTGCTCRGGGYCCATCYTGGGTAYRGGCCATRCTGCGCTTACAATGNNNTGGCAGCGTACAGGAGGACCAATTGTANNGACTGGTTNGNACCTGGNCYTGGAACGGTAGACTNYATAATACGGCAGTYTGTAAANNNGTNNGRYACGNAGATCNTAGGTNTCATGAGGAAATTTTNRACAAACACGGATCACTCGTTGGGCATTGACYATTNCCAATGYCCGTGGATAGTTYGGTTGATGRTGTAGNTTGRNNAACGGTGGAGCCATTCTGNAGNNNYTAGRNYGGGGCGNNNGCCGCANGCCAGGARYCTNGGTATAYTACANTGCCGCCAGCTGRTYCTCAARNGCCCTCTCTYGCCTGYTGARYGGNNNATATTTGGTTACNTTCCGCTGYCAATGAATCCTANCRGANACAGCTAAGTATYCTNGTCTARACTCAAGTAAGRGTCTAGAGCCNACCCATACNGTNTTAACACCGACGGGGCGAGGAAAGAACGCCTCCGGCTACNAACGGTGGACRTTCCCCTAGAAACRAATGRYNRCTGAGTGTGAGCAGGCCAATCTCNTCAATCYGNTCCYTTRTGYGNANRCCCCTGCCNCCGCNYACGCCAGTACCTAGCGGANTNCGCCGTGCGGTGGRTACCAGGTGRNTCCTNNTGCATGCGAATGNYNNNAACTACCGTRANCTAGCCGGCTGGTAGCTAGGAACTGATATYATGTGAGTACATGCCCCNNYCGCAGTACAGATACAGCTAGNCCTGTACACRRCGNAATTGTTCTTAGGCTYGCGAATACGGATAAGATYGGAAAAACGGCCGTCTTGRNCCGGTCAGTCACTRAAGTTGYRANTNGNAGTAACYCGCGGNAGCCATGGCTCCTAAATTAGGAATAGCCCGCGGTGTAGGGGCGRNCCGCGTCGGCGAGRRCCGCCTNRCYGCAAGGGATANTCAGACGNTTAGATTATGTCTGAAGGAACGCACTCCCCTTRNCRNAGGTNAYAYGCTGTGGCYCCACCTTNGACGGCCGGGGGATTAGNYACRTCCGGGGGYCGNTRAGTACTAGGGCYTNRGNNNNTGAATGCCCAGACGATGGGGCYAGCCACCCCGACAAGTTCCCACAAGNRGCGCCAGTTGGCTTGCTGYAGNGNATTTYTATGGCCCGACGGTAACGCGACGGTATCCTGAAAGACATAGGCGTGAGATCGGACTCCAGANATGTCATTTAGGRNTNNACTAGCACTGACRYRYAAACATCAGGYRCATCGGTANCTCTCCRGGYNACCGACRCGANTGNARTGAGCTCGCCCCTGGAYRAAGGAAGNGRRRAACYTCAAGACTCTGAGNACTCGCCRACCCTAGTGAACATAGAGCATCGCNYAGTNNGACATATGGTCTCTGCGAGCAGCNATCCTCTGACNNNNCCAGTGGCTTCCGCNGGACTTTGTACANCCAGAAAGCRAACCYCCYGYNNCTCCCNAGTGGATTNNCAAGTANCYTNCCTACATCNCTTCAAYGAAGATGCTNNRYCTGTCCTRTCGNGARCCCTATATCTATTAACCAGNTNNGCACTGGTCTCGANNNCGTAGTACTCGATNTAAGGTCGGGGRTCAYTRTGTGCAGGAGGACCATATTAGCCCGCTTCATTGNNAYGRYATGGTCTGTTTGGATATCGTCCGCTTGGTTAACTGCTCGAANCCCATCCTCACGAGNCCCGRTRAGYGACRGTGGGTCCGNRGATNCTNRCTGGACACTTTCCRAAGCGTCCAGAGGATANNTCGTTCTCTTTCCATTTACGCTAGCNAATTTCGTAATRTGCCGGYRGCGYAGAGCTGGYACCGCARTGGGTGTCTTGCAGAATGGYTCAAGCATCGGCGGCGCGTCACTATCRAGCCYCNNNCACGGCGGCGGCAGCTAGCCGACTCACCCAATGGNNNNNNNCCCCACGAGGCAGGCGGGATGAATACGGAGNCRTTATGANNGGGTTTACANACAYTCCACRYRGTTCAGCGATNGTGNAAGGGCGGTGCGGGYYCCYAGACCGRTATGGGGNGTARTTGGANNGYTYGCRAATACCTRNGAAGTACTCGACCGCNRATCGGTGCAGCGTGGCNNGGGNNYNNYGCTTTTATATTAANANTGGCTACGTTGATGCATGCNGAACTTCGCRGCARGAAAGAGCGARNCCRTRGCAACGGGCTCTGCGACCACRTTAGTTACCTAATGYGTCAAAGATTGATTGTTCGTGTCCNYCCCCGCGGCGYGAAGAAACTAAAAGTGYNRCTRCGCATAAGCCTGNCRAGYAAGGCCYANTCTCNNNNGCACGCRGNGCGGYCGCGNNAGCNGGTTGTRRGCGATTYGRNNAYTAGCACTACTNCTAGAARCGGCCGGTACAGGGAACYCGCTGGTNNGTTACGACTTATYTAGCCCRCCCCGGAGGAATCNTNCTCGTYGTTAGGGGCTACTTCGGTGGCGTACATCGCAYTANACCTAYGCAGNRGCRNCAGCGTCTTACAATACNCTAANYGCCCACAGCATYTCGGGCTAATACTTCTCCNNACTCCTGATCACCGGTGGCCACTAGRYGCCNNNTCCTGGATACTCGAAAYGGNCYTYCAGRGNTCGCCATCTTACAAGNCYCCTGATGGAATGCGATGTTNTATCYATGTNNCTTCTGAGCGTCGGTGAYGNYGCACGNNCGCCGGTTTATGAACCGCANTACCGGGCYTGCAGRGNRGGAGACGCGGCGCCGTARCNNRNGTGTCRACTTGTNTCNGCCGARRRNRTGGGACCGCGATCACGTGACCNYNNACTCCGCATGGACGCYAGCCTCNAGNGARGGTGNCCACATCGATCCCRGRYCTGAGAATCGRRCANCGAGTTCTAGCCGAAAYANCCCCCGAYYYGYGYNTAAAGTTACCACGCTNNYAGTAATGGGACYYACCGGGNTTGCGAGGATTANAGCANNYCNRATGCCCCGTCGCAGCGGNATTNAANYCTYACRTCYTTYGGAACAGNCGGRRTGANRCATTAGAGCACYGTCGGATCATCCGGCACACGTCACGTGTGTAATANNCACTAGGTNTGNNAGAGGAGTTCGGGCRCCAGTGTTTTNNNCTCAGATTTTCGGACTAGCCAYNNAGYTCCGCNGACRTTGGCAGCTCACAAGCGGTAGYCCATCCCACTARANGYNACTCCAGGGGACGCGNAAGCCYCAARGTRTAATCAACCTNGTGTTRGTNRGACGAGAAGAGTAATATAATCCCTATGTCGCAGGAANGNTCCRTGCCGRAGGAGTTATCACCAATRAAAGTGTCCGCGAGATGCGCTACNTRCCCNTTGTYAGTYTACAYTGCATANTANANTNRNCGCTGCCTCRTCNYGRCCGTACGCAGTAGCCTCCAGAGCCTAATTCGCNGRCTACCCCGACGCCAGCCTCRCACAGCCAGAGCTACTGTATCTAAATGNACTGTCCTCATNGTNGYNGGCGGGCTCCNNNTAACGTCRYAGCTATAACACANAGGCAGTTTGAGAAATCATANNCNCGGTAAACCGNAGRGCGAGGCYACACAGCNTRAGAGGNCANCCYATTGTGGANARATCNNGCAGTCTCGGCCTGGATCGNNNCCRNNGTGARCGGACCGGTYCGCAGGCGGCCYRCCTGATYGNNACCGCGNCGGGTACCTGCGACCTANNNNTTTGCGCCCGGGTGCCGTGGCCGTTCCGYRTAACTRNTCCNGGGACNNGGATGCCCTTGTAAGCACCATGAAATAGATTGTAYCACGCCACACGACGGCNTTGTACGCCNGCNGYACCTTRYNTYAGCNGTCAAGTTTCCTCGTNTTGCCCCCRGNCAATCATGCGYNANNNGRNGGGGTYATGGCAGCNCGAATCAGGTGCGGYCTNNGTTCCCAAAAAYTGCAGTYATTTATGCGTACGTCGAATGCACCGCGACGTTGCTTGCGGGTACTTGYGGCCGTCATNGYGTYCATGGTGTTATGAGCTCTCCTTARGCGCRRACCRAAANCGCCACGATCATRYGNAGAAGGCGCGAATGTGTCCATAGCGNYARCACGNACYTTGAAAGCGCGAATATTACGGCTCACTTCTATACARNTCTAGGTACGRNNYCACNCTGGCTGYATATCTGRGCNACCTCCGYCRCGCAAATTCAYGGGGGGGGAGYCAACACTYTGTYGAAGCNRGCTCNCNNGGTTACTAAATTGTGGCGTYTCCCTATCCRAARGNNTGTTNTGYTRYCNCGGAGGTCGCCGGCATGATNTCGCTNCACYCNNGATATGTNTYGCANTRYANCGATANCAGGCNTCGYAYGNCNTNNNNATGACRAAACANRRYRNNNAGTAYGGGACGAYGNAGCCACCNCGGACCCTGATCGTGTTTCGTANYRACACAACNTYRGTCTCTGACTTTACCTGGGTGCAGACNYCTYAATNAGCTGGGATTAYCGGCCGAATAAGCCGANTGTCNGGCNAACNCGARYCTAGCGRGTCGCGCACTCCCCGRAGGGCACCANANATANATNTTNCACTCAGGGCGGARGCGCNNAAACANTCNATYCCCNCCTGTGGTCAGNNCTRAGATCTAAGGTAAGCCCCAYGTRCNATTGGGAGGCCGAATYYNRNTGTCCGGTNYTAYTCCTCGAACCGACGRTAAAGTACGTGAAGAANTNGTTAACTTGACCYGCTAGACCGCATGTYGGTNCACAGNTNCCTCGGTCTARTGGNACTTCCARCGYTTRCCNCCNAGCGGCAGGAGTTAGTNGGNCNRCNCTCAATNTGACATAATCTACAGTAAGCCGATAAAARRCNCTCGCCAGCATTTGCAYCCACRNGNGTCNCTGATACTYCYANGNCTRRCYGGCGGAATAGAGRTTCTCGATCTGTGATGNNNNGCCGTCACAGAGGTTACYGGCTRTTGTRTGNCRCGGACYGYGCCCAATTTGTTCTCYAAGTNGYARRTARACCCTNACTTAGGGNGTCRAGAYATNANCGCAATCGCCTCAGGTCCTAGGAACGNGGNNNNCTGATCGCTNRACCCCACCTNTCAAAGCTACCGCNACRTCTTCTTAACGACTAACTAGCTYGAACCCTAGATAATGGGGGYGTTNAGNRNTACTAGARGGGCAACCTTGGATTGYNCNNRTRATCCTTAGACCACTTTGTGGCATAGARRCAGAGCCCCNRGGCNGRGCCCTAGGCCGTCCRCAAACNNNNCAGTTGGRCCRNAGGCCGACNGGGGCGCARCRYAACATGRCTTCACTAAGNACACCCCGCTATTTTAGCAGGAGCCGGTGTGCATRCCCNTACCACGCGCCNGRRGCTTGTCTGTCGGTAAAGCCRNRRCGCCAACTAGAGANNGNNTGGTACTTTGTRTAAATGCCCTTTCCGGGAGCATCNGATCGYTCGGNCCCGAGNTNTGCCATTRRYYAGGCANTGCCTTTGCACGGCAACGGCCCAATGTCACGACCTCGGRACGGTATGTGGCANACCTTTTRRRTNNNNNYCGATTGCCCGGCACAGAGGCCNYAGYATCCTTACGCTGRARTTTACCGTRAARRCGATCGGGCAAAGCGCTACATGCTGGCGGAGTCCGTTRRTGNCCRAATCAANNGAYCARATTAGGGAYCTAGCATCTTACAGTGGCGRGTCATTACATTCAGTCCGACGCRGTCTCGCAGTGCACATT

In547 RYGRYTNRCTCGTCGYCNTANNGNCATCCNGNGCTGYNGACCTAGACGGGTCTARGAGGNNTACCCACTGACCGTGATCTACNGGNYATCACGCCTTTTGTCTRAGGAGYCCACTGCTCGRGGTCYATCTTGGGTATGNGCCNTRCTGCGCYNACAATGGCCTGGCAGCGTACAGGAGRRCCRNATNYAACGRCAGGTTNNNNCCTGRCYYTGGNACGRTAGACTACATAATACGGCAGTYTNYNNNTANGTTNGRTACGCAGATCNTAGGTCTCATGAGGAARYTTTNRACGAACRCRGATCACYCRTTGGGCATTGATYATTCCCAATGNCCRTGGATAGYYTGGTTGNTGRYNNAGCTTGRAGAACGGTGGAGCCANTCTGGAGCTGTTAGANYGGGGYGGGCGCCGYNGGYCAGGARCNTCGNTAYACTACAGTGCCGCCAGCTRGYCYTCAAGAGCCCTCYCTTNNCTGCTGAACGGNCTNNATTTGGTTACNTTCNGCTGYCANTGAATCCNANCRGAAACAGCTAANYATYCTCGTCTAAACTCAAGTAAGAGTATAGAGYCGACGCACACGGTTNYAACACCGACGGGGCGAGGAAAGARCGCTTCCGGCTACCAAYGGTGGACRTTCCCCTAGAAACRAGNGRYARCTGAGGNTGAGCAGGCNAATCTCCTCAATCYGCTCCCTTATGTGGATRCCNNYRCCCCCGCTTACGNCNNNACCTAGCGGATTGCGCCGTGCGGTGGRTACCAGGTGAATCCTCTTGCNTGCGAATGNYNGGCACTACCGTRANCTAGCCGGCTGGTAGCTAGGAACTGATATTATGTGAGTACATGCCCCGTCCGCAGTCCGGTTACNGCTAGCCCTGTACAYRACGNAATTGTTCTTAGGCTYGCGNATACGGATAAGATYGGNAAAACGGCCGYCNTGAGCCGGTCAGTCACTRAAGTTGYRANTNGNAGTRACTCRCNGCAGCCNNGGCNYNTAAATTNGGAANNGCCCGCGGTGTAGGAGCGNGCCGCGTCGGTAAGGGCCGCCTNRCTGCANNGGATANTCANNCGCTTAGATTATGTCNGAAGGAACGCACTCCTCTTANCRNAGRTAATATGCTGTGGCTCCAYYTTYNRCRGCCGGGGGATTAGNCTCGTCCGGNGGCCGNTAAGTACTAGGGCCTTARAACRNNNATGCACGGACGATGGGGCCAGCCACCCCGACAAGTTCCCACAAGGGGCGCCAGTTGGCTYGCTGCRRAGAATTTCTATGGCCCGACGGTAACGCGACGGTATCCTGAAAGACNTANGNAYGNGATCNGAYNCCAGAAATGTYATNTAGGAATCCACTAGYACTGACRYNCAAACATCAGGYRCATCGGTANCTCTCCGGGCNRCCRRCGYGAATGNNATTGGCTYNNNCNTGGACAANGGAAGGGRRRAACYTCRRGACYCTGAGGACTCGCCGACCCTARTGAACATAGAGCATCGCGTAGTNTGACATNTGGTCTNYRNGANNNNCCATCYTCTGACCTNNCCAGTGGCTTCCGCAGGACTTTGTRNRRCCAGAAAGCGAACCTCCCGNNNCTCCCNAGTGGTTTNNCAAGTANCNTNCCTACRTYNCYYCRACGAAGATGCTCYGCCTRTGCTRTTGNGAGCCCTATATCTATTAACYAGGTCCGCATTGGGYTCGGACTCGTAGTNNTCNATNTAAGGTCGGGGATCAYTRTGYGCAGGNGGANCRTATTAGCCYRCTTYAYTGAGATGGCATGGTCTGTTTGGATATCGTCCGCYTGGTTAACTGCTCGAATCCCATCCYCACGCGTCCCGATRAGNGATRGTGGGTCCGNGRATNCTCRCYGGACACYTTCCAAAGCRTCCAGAGGATACTNNGTTCTCTTTCCATTTACGYTAGCNAATTTCGTAATRTGCCGGCANCGTAGAGCTGGCACCGCARTGGRTRTCTTGCAGNAYGGCYCAARCRNCRGCGGCGCGTCACNATCRAGCCCCTGGCACGGCGGCGGCAGCTAGCCGACTCACCCRATRRNNCGTGTCCCCACGAGGCAGGCGGGATGAATACGGAGACGTCATGACGGGGTTTAYNCACANTCCRCRYGGTTYNGCNRTTAYGNAAGGRYGGTNCGGGCYCCYAGACCGGTATGGRGYGTAATTGGRGTGYTYGCRAATANCTGAGNAGTACTCGACCGCGAAYCGGTGCAGCRTNGCNAGGGCGCCTYGCTTCTRTATTAANATTGGCTACGTNGATGYATGYNGAACTTCRCRGYARGRARGRGCRAGNCCGTRGCAACGGGCTCTGCGACCACATTAGTTAYCYNNTGCGTCARAGATTGATYGTTCGTGTCCGCCCCCGCGGCGYGAAGNRNCTAANNANNNNNCTRCGCANAAGCCTGTNRAGTAAGGCCCRNTCTCCGGCGNRYGCRGNGANNTCGCGCCAGCCGGYTGTRRGCGNNTCNNCAANTAGCRCNNCTNCTAGARACRGCCGGTACAGGGAACYCGCTGGTGGGTTACAGCTTANNTAGCCCACCCCGGAGGAATCNTNCTCGTCRTYNRNGTCTNCTTCGGTGGCGTACATCGCACTNTACCCACGCAGCAGCGGCAGCGTCTTACAATACTCTARACGCCTACAGCATCTCGGGCTANTACTTCTCCNCACTCCTGNTCACCGGTGGCCACTAGACGCCTGTTCCTGGATANNCGAAACGGGCCTCCAGAGCTCGCCGTCTTACAAGACYCCNGATRGAATGCGRCGTTNTATCYATGTNNNNTCTGNGCGTCGGTGACGACGCACGACYGCCGGTTTATGAACCGCACCACYGGGYCTGCAGAGTGGGAGACGCGGYNNCGTARCGCAGGTGTCRACCTGTNTYAGCYRRGRRAANGGNNCCGCGATCACNTGACCNYNTACTCCGCATGGACGCTNGCCYCAAGTRAGGGTRNCCACAYCGATCCCRGRYCTGAGAATCGRRCAACNCCTTCTAGCYGAAATACCTTCCGAYCCGTGYNTAAAGTTANCACGCTTACNGTAATGGGACNNACCGGGNTTGCGAGGATTANAGCAACYCNRNTGNTCCGTCGCAGCGGTATTAAANNCTCANGTCCTCTGGGGNAGACGGGGNGACGCRTTNRRGYRCYGYYGGAYCGTCYGGCNCRCRTCACGTRTGTAATAGTCAYTRGGTNTGNNAGAGGAGTTCGRGCACCAGYGTCTTNACCTCNNNTTTTCGGACTAGCCAYNNAGTTCNRYAGACATTGGCAGCTCACAAGCGRTAGYCNATCCCACTARAGRCGACTCCAGRNGNCGCGAAAGCNCCAAGGTATAATNANYCTCGTGTTGGTARGNNGARAAGAGTAATATAATCCCTATGTCGCAGGAATGGTYCGTGCCGRAGGAGNYATCACCAATGAAAGTGTCCGCGAGATGCGCTACNTGTCCNTTGTNAGTYTACATNGCANAATAAACTAGTCGCTGCCTCRTCATGAYCGTACGCAGTAGCCTCCNGAACCTNATTCGCCGACTACCCCGACGCCAGCNNNGCTCAGCCTGAGCTACTGTATCTAAATGGACTGTYCTCATTGTNGYNGGYGRGCYCCTYTTAAYNTCRYNGCTATAACACACAGGCAGTTTGAGAAATCNTACATGCGGTAATCCGTAGGGCGAGRCYACACAGCCTRAGRGGCCAGYYYATTGTGGANRRATYGCNCAGTCTCGGCCTGGRTCGGGTCCANNGTGGACGGACCGGTYCGYAGGAGGCGCACCNGATCGNNACYRCGNCGGGYNCCTGCGACCTAANCGTTTGCGCCCGGNYGCCGTGGCCGTTCCGYRNAACTRNTCCNGGGACNTGGATGCCCTTGTAANCAYNNTGRAATAGATTGTAYCRNNCCACACGACGGCCTAGGACGCCAGCRGTACCTTRYYTYAGNGGTCAAGTTTCCTCGTGTTGCYCCCAGGCAATNATGCGTAANRGGNNGGGGTYNNGGCAGCNCGAATNAGGTGCGGCYYCNGNTCCCAAAAAYTGCAGTCATNNATANNYACGTCGAATGCACCGYGGCRTTGCTNGCGGGTACYNGCGACCGTCATAGTGTTCATGGTGTTATGAGCTCTCCTTARGCGCGGACCGANRTCGCCACNRTYNTRNNNAGAAGGCGCRARTGTGTCCANAGCGNTAGCACGGACYTTGAAAGCGCGAATATTACGGYTCACTYCTATACARNTTTAGGTACGANCYCACNCTGGCTGTAYNYCTGGGGGACCNCCGTYGCGCAAATTCNNNNNNNRNGAGCYRACACYYTGNTGNNGCGGGCTNNCACGGTTACTAAATTGTGGCGTYNCCCTANYCRAAGGRRAATTNTGTTRYCTCGGAGGACGCCGGCATGGTGTCGCTTCACYCCCGATATGTATTGCAATRTANCGATANCAGGCGYCGTATRACATATARATGACAAAACANGATACGCAGTACGGGNCGRCGTAGCCACCNCGGACCCTGATCGTGTTTCGTANYRACAYAACATYARTCYYNGANTYNACCNGNGTGCAGACGTCTCAATGAGCTGNGATTACCGGCCGAATRAGCCGAGTGTCGGGCCAACCCGAGTCTAGCGRGTCGCGCACNNCCCGGAGGGCACCACAYATCNATNTTTCACTNRGGGCGGNAGCGCYAAAACANTCNRTYCCCACCTGTGGTTARCCCTRANATCTAAGGTAAGCCCYAYRNGCGATTGGGAGGCYRRATCCCAATGTCCGGTCYTATTYCTCGAACCGACNANNNNGTNYGTGAAGAANTTGTTAAYTTGACCCGCTAGACCGCATGTYGGYNYACAGCTGCCTCGGTCTRGTGGNACTTCCAACGTTTGCCTCCNNNCRGCAGGAGTTAGTTGGCCCACCCYCRRTNTGACATAAYNNNCAGCAARYYGATRRARRRCACTCGCNAGCATTTGCATNCACATGCGTCNCTGACACTNCNANGTCTRRCTGGCGGAAYRGAGRTTCTCGATCTGTGATGRNAARYCGTCACAGNTACTGCYRGCTNTYRYRNRGCACGGGTTGYGCCCNGATTACCCTCYAAGTGGTAGATAGGCCTTGRCYTAGGGCGTNGRGACRTNANCGCAATCGNCTCARGTCCTAGGAAYGTGGNNTCCTRRTCGCTCAACNNNNNCTNNNAAAGCTACCGCARCGACTTCTTAACGNCNGGCTAGCNTGAACCCTNGATAATGGGGGYGTTNRRGGAYNCTNNNAGGGYNTYCTTRNATTGCNNGYATGATCCYNAGACCACTTYGCGGCATGGGGGCAGRGCCYCTAGGCTGAGCYCNAGGCCGTCCGCAAACNGGCCAGTTGGACCRNAGGCCGAANGGNGCGYARCGTAACATRGCNTCACTARGNACACTNNGYTATTNTAGCAGGAGCCGGTGTGCATRCCYNTACCANNCGCNTGNNGCTTATCTGTCGGTANANCCATGGCGYCAACTGRNGAGTGCCTGGTACTTTGTRTARANGCCYTTTCCGGGAGCRNYGGATCGCTCGGNYCCGAGCTATGCCATTAACCAGRCNNTGCCTTTGYRCRRCAACGGCYYRRTGTCACGACCTCGGGNCGGTATGTGGCANACYTTTTRRRTCCGACCCGATTACCCAGCACGNNNGCCCCAGCATCCTTACRCTGGAATTTAYCGTRAAGACGATCGGRCAANGCNYNNCCTRCNGGCGGAGTCCGTTGGTGNCCRANTCAAGNGACYAGNNNTGAGACCTAGCATNNTACAATGGCGAGTCATTACATTCAGTNCGACGCGGTCTCGCAGTGCACATT
[truncated: 69,885 more chars]
