# Supplementary material for: Contrasting genetic structure between mitochondrial and nuclear markers in the dengue fever mosquito from Rio de Janeiro: implications for vector control
Source: Evol Appl. 2015 Sep 7;8(9):901–15. doi: 10.1111/eva.12301 (PMC4610386; doi:10.1111/eva.12301)
Supplement: Supplementary file 6 — File S4. Amplicon sequences from mitochondrial genes COXI and ND5 in Ae. aegypti. [file eva0008-0901-sd6.docx]

>Brazil-COXI-A09

TTTTGATTCTTTGGACACCCAGAAGTTTATATTTTAATTTTACCCGGATTTGGAATAATTTCTCATATTATTACTCAAGAAAGCGGAAAAAAGGAAACATTTGGAACTTTAGGAATAATTTATGCTATATTAACAATTGGATTATTAGGATTTATTGTTTGAGCTCATCATATATTTACAGTAGGTATAGACGTAGATACTCGAGCTTATTTTACTTCAGCAACTATAATTATTGCTGTTCCTACAGGAATTAAAATTTTTAGTTGATTAGCAACTTTACACGGAACTCAATTAACATATAGTCCAGCCCTTCTATGATCATTAGGATTTGTATTTTTATTTACAGTTGGAGGTTTAACAGGAGTAGTATTAGCTAATTCTTCAATTGATATTGTTCTTCATGATACTTATTACGTAGTTGCCCATTTTCATTACGTTTTATCTATAGGAGCTGTATTTGCTATTATAGCAGGATTTATTCATTGATACCCTTTATTAACAGGAATAGTTATAAACCCTTCATGATTAAAGGCTCAATTTAGTATAATATTTATTGGAGTAAATCTAACTTTCTTTCCTCAACATTTTTTAGGGTTAGCTGGAATACCTCGACGATACTCAGATTTTCCTGATAGCTACTTAACTTGAAATATTATTTCTTCTTTAGGAAGAACAATTTCACTATTTGCCGTTATTTTCTTTTTATTTATTATTTGAGAAAGTATAATTACTCAACGAACACCTTCTTTC

>Brazil-COXI-A10

TTTTGATTCTTTGGACACCCAGAAGTTTATATTTTAATTTTACCCGGATTTGGAATAATTTCTCATATTATTACTCAAGAAAGCGGAAAAAAGGAAACATTTGGAACTTTAGGAATAATTTATGCTATATTAACAATTGGATTATTAGGATTTATTGTTTGAGCTCATCATATATTTACAGTAGGTATAGACGTAGATACTCGAGCTTATTTTACTTCAGCAACTATAATTATTGCTGTTCCTACAGGAATTAAAATTTTTAGTTGATTAGCAACTTTACACGGAACTCAATTAACATATAGTCCAGCCCTTCTATGATCATTAGGATTTGTATTTTTATTTACAGTTGGAGGTTTAACAGGAGTAGTATTAGCTAATTCTTCAATTGATATTGTTCTTCATGATACTTATTACGTAGTTGCCCATTTTCATTACGTTTTATCTATAGGAGCTGTATTTGCTATTATAGCAGGATTTATTCATTGATACCCTTTATTAACAGGAATAGTTATAAACCCTTCATGATTAAAGGCTCAATTTAGTATAATATTTATTGGAGTAAATCTAACTTTCTTTCCTCAACATTTTTTAGGGTTAGCTGGAATACCTCGACGATACTCAGATTTTCCTGATAGCTACTTAACTTGAAATATTATTTCTTCTTTAGGAAGAACAATTTCACTATTTGCCGTTATTTTCTTTTTATTTATTATTTGAGAAAGTATAATTACTCAACGAACACCTTCTTTC

>Brazil-COXI-A11

TTTTGATTCTTTGGACACCCAGAAGTTTATATTTTAATTTTACCCGGATTTGGAATAATTTCTCATATTATTACTCAAGAAAGCGGAAAAAAGGAAACATTTGGAACTTTAGGAATAATTTATGCTATATTAACAATTGGATTATTAGGATTTATTGTTTGAGCTCATCATATATTTACAGTAGGTATAGACGTAGATACTCGAGCTTATTTTACTTCAGCAACTATAATTATTGCTGTTCCTACAGGAATTAAAATTTTTAGTTGATTAGCAACTTTACACGGAACTCAATTAACATATAGTCCAGCCCTTCTATGATCATTAGGATTTGTATTTTTATTTACAGTTGGAGGTTTAACAGGAGTAGTATTAGCTAATTCTTCAATTGATATTGTTCTTCATGATACTTATTACGTAGTTGCCCATTTTCATTACGTTTTATCTATAGGAGCTGTATTTGCTATTATAGCAGGATTTATTCATTGATACCCTTTATTAACAGGAATAGTTATAAACCCTTCATGATTAAAGGCTCAATTTAGTATAATATTTATTGGAGTAAATCTAACTTTCTTTCCTCAACATTTTTTAGGGTTAGCTGGAATACCTCGACGATACTCAGATTTTCCTGATAGCTACTTAACTTGAAATATTATTTCTTCTTTAGGAAGAACAATTTCACTATTTGCCGTTATTTTCTTTTTATTTATTATTTGAGAAAGTATAATTACTCAACGAACACCTTCTTTC

>Brazil-COXI-A12

TTTTGATTCTTTGGACACCCAGAAGTTTATATTTTAATTTTACCCGGATTTGGAATAATTTCTCATATTATTACTCAAGAAAGTGGAAAAAAGGAAACATTTGGAACTTTAGGAATAATTTATGCTATATTAACAATTGGATTATTGGGATTTATTGTTTGAGCTCATCATATATTTACAGTAGGTATAGATGTAGATACTCGAGCTTATTTTACTTCAGCAACTATAATTATTGCTGTTCCTACAGGAATTAAAATTTTTAGTTGATTAGCAACTTTACACGGAACTCAATTAACATATAGTCCAGCCCTTCTATGATCATTAGGATTTGTATTTTTATTTACAGTTGGAGGTTTAACAGGAGTAGTATTAGCTAATTCTTCAATTGATATTGTTCTTCATGATACTTATTACGTAGTTGCCCATTTTCATTACGTTTTATCTATAGGAGCTGTATTTGCTATTATAGCAGGATTTATTCATTGATACCCTTTATTAACAGGAATAGTTATAAACCCTTCATGATTAAAGGCTCAATTTAGTATAATATTTATTGGAGTAAATCTAACTTTCTTTCCTCAACATTTTTTAGGGTTAGCTGGAATACCTCGACGATACTCAGATTTTCCTGATAGTTACTTAACTTGAAATATTATTTCTTCTTTAGGAAGAACAATTTCACTATTTGCCGTTATTTTCTTTTTATTTATTATTTGAGAAAGTATAATTACTCAACGAACACCTTCTTTC

>Brazil-COXI-B09

TTTTGATTCTTTGGACACCCAGAAGTTTATATTTTAATTTTACCCGGATTTGGAATAATTTCTCATATTATTACTCAAGAAAGCGGAAAAAAGGAAACATTTGGAACTTTAGGAATAATTTATGCTATATTAACAATTGGATTATTAGGATTTATTGTTTGAGCTCATCATATATTTACAGTAGGTATAGACGTAGATACTCGAGCTTATTTTACTTCAGCAACTATAATTATTGCTGTTCCTACAGGAATTAAAATTTTTAGTTGATTAGCAACTTTACACGGAACTCAATTAACATATAGTCCAGCCCTTCTATGATCATTAGGATTTGTATTTTTATTTACAGTTGGAGGTTTAACAGGAGTAGTATTAGCTAATTCTTCAATTGATATTGTTCTTCATGATACTTATTACGTAGTTGCCCATTTTCATTACGTTTTATCTATAGGAGCTGTATTTGCTATTATAGCAGGATTTATTCATTGATACCCTTTATTAACAGGAATAGTTATAAACCCTTCATGATTAAAGGCTCAATTTAGTATAATATTTATTGGAGTAAATCTAACTTTCTTTCCTCAACATTTTTTAGGGTTAGCTGGAATACCTCGACGATACTCAGATTTTCCTGATAGCTACTTAACTTGAAATATTATTTCTTCTTTAGGAAGAACAATTTCACTATTTGCCGTTATTTTCTTTTTATTTATTATTTGAGAAAGTATAATTACTCAACGAACACCTTCTTTC

>Brazil-COXI-B10

TTTTGATTCTTTGGACACCCAGAAGTTTATATTTTAATTTTACCCGGATTTGGAATAATTTCTCATATTATTACTCAAGAAAGCGGAAAAAAGGAAACATTTGGAACTTTAGGAATAATTTATGCTATATTAACAATTGGATTATTAGGATTTATTGTTTGAGCTCATCATATATTTACAGTAGGTATAGACGTAGATACTCGAGCTTATTTTACTTCAGCAACTATAATTATTGCTGTTCCTACAGGAATTAAAATTTTTAGTTGATTAGCAACTTTACACGGAACTCAATTAACATATAGTCCAGCCCTTCTATGATCATTAGGATTTGTATTTTTATTTACAGTTGGAGGTTTAACAGGAGTAGTATTAGCTAATTCTTCAATTGATATTGTTCTTCATGATACTTATTACGTAGTTGCCCATTTTCATTACGTTTTATCTATAGGAGCTGTATTTGCTATTATAGCAGGATTTATTCATTGATACCCTTTATTAACAGGAATAGTTATAAACCCTTCATGATTAAAGGCTCAATTTAGTATAATATTTATTGGAGTAAATCTAACTTTCTTTCCTCAACATTTTTTAGGGTTAGCTGGAATACCTCGACGATACTCAGATTTTCCTGATAGCTACTTAACTTGAAATATTATTTCTTCTTTAGGAAGAACAATTTCACTATTTGCCGTTATTTTCTTTTTATTTATTATTTGAGAAAGTATAATTACTCAACGAACACCTTCTTTC

>Brazil-COXI-B11

TTTTGATTCTTTGGACACCCAGAAGTTTATATTTTAATTTTACCCGGATTTGGAATAATTTCTCATATTATTACTCAAGAAAGCGGAAAAAAGGAAACATTTGGAACTTTAGGAATAATTTATGCTATATTAACAATTGGATTATTAGGATTTATTGTTTGAGCTCATCATATATTTACAGTAGGTATAGACGTAGATACTCGAGCTTATTTTACTTCAGCAACTATAATTATTGCTGTTCCTACAGGAATTAAAATTTTTAGTTGATTAGCAACTTTACACGGAACTCAATTAACATATAGTCCAGCCCTTCTATGATCATTAGGATTTGTATTTTTATTTACAGTTGGAGGTTTAACAGGAGTAGTATTAGCTAATTCTTCAATTGATATTGTTCTTCATGATACTTATTACGTAGTTGCCCATTTTCATTACGTTTTATCTATAGGAGCTGTATTTGCTATTATAGCAGGATTTATTCATTGATACCCTTTATTAACAGGAATAGTTATAAACCCTTCATGATTAAAGGCTCAATTTAGTATAATATTTATTGGAGTAAATCTAACTTTCTTTCCTCAACATTTTTTAGGGTTAGCTGGAATACCTCGACGATACTCAGATTTTCCTGATAGCTACTTAACTTGAAATATTATTTCTTCTTTAGGAAGAACAATTTCACTATTTGCCGTTATTTTCTTTTTATTTATTATTTGAGAAAGTATAATTACTCAACGAACACCTTCTTTC

>Brazil-COXI-B12

TTTTGATTCTTTGGACACCCAGAAGTTTATATTTTAATTTTACCCGGATTTGGAATAATTTCTCATATTATTACTCAAGAAAGCGGAAAAAAGGAAACATTTGGAACTTTAGGAATAATTTATGCTATATTAACAATTGGATTATTAGGATTTATTGTTTGAGCTCATCATATATTTACAGTAGGTATAGACGTAGATACTCGAGCTTATTTTACTTCAGCAACTATAATTATTGCTGTTCCTACAGGAATTAAAATTTTTAGTTGATTAGCAACTTTACACGGAACTCAATTAACATATAGTCCAGCCCTTCTATGATCATTAGGATTTGTATTTTTATTTACAGTTGGAGGTTTAACAGGAGTAGTATTAGCTAATTCTTCAATTGATATTGTTCTTCATGATACTTATTACGTAGTTGCCCATTTTCATTACGTTTTATCTATAGGAGCTGTATTTGCTATTATAGCAGGATTTATTCATTGATACCCTTTATTAACAGGAATAGTTATAAACCCTTCATGATTAAAGGCTCAATTTAGTATAATATTTATTGGAGTAAATCTAACTTTCTTTCCTCAACATTTTTTAGGGTTAGCTGGAATACCTCGACGATACTCAGATTTTCCTGATAGCTACTTAACTTGAAATATTATTTCTTCTTTAGGAAGAACAATTTCACTATTTGCCGTTATTTTCTTTTTATTTATTATTTGAGAAAGTATAATTACTCAACGAACACCTTCTTTC

>Brazil-COXI-C09

TTTTGATTCTTTGGACACCCAGAAGTTTATATTTTAATTTTACCCGGATTTGGAATAATTTCTCATATTATTACTCAAGAAAGCGGAAAAAAGGAAACATTTGGAACTTTAGGAATAATTTATGCTATATTAACAATTGGATTATTAGGATTTATTGTTTGAGCTCATCATATATTTACAGTAGGTATAGACGTAGATACTCGAGCTTATTTTACTTCAGCAACTATAATTATTGCTGTTCCTACAGGAATTAAAATTTTTAGTTGATTAGCAACTTTACACGGAACTCAATTAACATATAGTCCAGCCCTTCTATGATCATTAGGATTTGTATTTTTATTTACAGTTGGAGGTTTAACAGGAGTAGTATTAGCTAATTCTTCAATTGATATTGTTCTTCATGATACTTATTACGTAGTTGCCCATTTTCATTACGTTTTATCTATAGGAGCTGTATTTGCTATTATAGCAGGATTTATTCATTGATACCCTTTATTAACAGGAATAGTTATAAACCCTTCATGATTAAAGGCTCAATTTAGTATAATATTTATTGGAGTAAATCTAACTTTCTTTCCTCAACATTTTTTAGGGTTAGCTGGAATACCTCGACGATACTCAGATTTTCCTGATAGCTACTTAACTTGAAATATTATTTCTTCTTTAGGAAGAACAATTTCACTATTTGCCGTTATTTTCTTTTTATTTATTATTTGAGAAAGTATAATTACTCAACGAACACCTTCTTTC

>Brazil-COXI-C10

TTCTGATTCTTTGGACACCCAGAAGTTTATATTTTAATTTTACCCGGATTTGGAATAATTTCTCATATTATTACTCAAGAAAGTGGAAAAAAGGAAACATTTGGAACTTTAGGAATAATTTATGCTATATTAACAATTGGATTATTGGGATTTATTGTTTGAGCTCATCATATATTTACAGTAGGTATAGACGTAGATACTCGAGCTTATTTTACTTCAGCAACTATAATTATTGCTGTTCCTACAGGAATTAAAATTTTTAGTTGATTAGCAACTTTACACGGAACTCAATTAACATATAGTCCAGCCCTTCTATGATCATTAGGATTTGTATTTTTATTTACAGTTGGAGGTTTAACAGGAGTAGTATTAGCTAATTCTTCAATTGATATTGTTCTTCATGATACTTATTACGTAGTTGCCCATTTTCATTATGTTTTATCTATAGGAGCTGTATTTGCTATTATAGCAGGATTTATTCATTGATACCCTTTATTAACAGGAATAGTTATAAACCCTTCATGATTAAAGGCTCAATTTAGTATAATATTTATTGGAGTAAATCTAACTTTCTTTCCCCAACATTTTTTAGGATTAGCTGGAATACCTCGACGATACTCAGATTTTCCCGATAGTTACTTAACTTGAAATATTATTTCTTCTTTAGGAAGAACAATTTCATTATTTGCCGTTATTTTCTTTTTATTTATTATTTGAGAAAGTATAATTACTCAACGAACACCTTCTTTC

>Brazil-COXI-C11

TTTTGATTCTTTGGACACCCAGAAGTTTATATTTTAATTTTACCCGGATTTGGAATAATTTCTCATATTATTACTCAAGAAAGCGGAAAAAAGGAAACATTTGGAACTTTAGGAATAATTTATGCTATATTAACAATTGGATTATTAGGATTTATTGTTTGAGCTCATCATATATTTACAGTAGGTATAGACGTAGATACTCGAGCTTATTTTACTTCAGCAACTATAATTATTGCTGTTCCTACAGGAATTAAAATTTTTAGTTGATTAGCAACTTTACACGGAACTCAATTAACATATAGTCCAGCCCTTCTATGATCATTAGGATTTGTATTTTTATTTACAGTTGGAGGTTTAACAGGAGTAGTATTAGCTAATTCTTCAATTGATATTGTTCTTCATGATACTTATTACGTAGTTGCCCATTTTCATTACGTTTTATCTATAGGAGCTGTATTTGCTATTATAGCAGGATTTATTCATTGATACCCTTTATTAACAGGAATAGTTATAAACCCTTCATGATTAAAGGCTCAATTTAGTATAATATTTATTGGAGTAAATCTAACTTTCTTTCCTCAACATTTTTTAGGGTTAGCTGGAATACCTCGACGATACTCAGATTTTCCTGATAGCTACTTAACTTGAAATATTATTTCTTCTTTAGGAAGAACAATTTCACTATTTGCCGTTATTTTCTTTTTATTTATTATTTGAGAAAGTATAATTACTCAACGAACACCTTCTTTC

>Brazil-COXI-C12

TTTTGATTCTTTGGACACCCAGAAGTTTATATTTTAATTTTACCCGGATTTGGAATAATTTCTCATATTATTACTCAAGAAAGTGGAAAAAAGGAAACATTTGGAACTTTAGGAATAATTTATGCTATATTAACAATTGGATTATTGGGATTTATTGTTTGAGCTCATCATATATTTACAGTAGGTATAGATGTAGATACTCGAGCTTATTTTACTTCAGCAACTATAATTATTGCTGTTCCTACAGGAATTAAAATTTTTAGTTGATTAGCAACTTTACACGGAACTCAATTAACATATAGTCCAGCCCTTCTATGATCATTAGGATTTGTATTTTTATTTACAGTTGGAGGTTTAACAGGAGTAGTATTAGCTAATTCTTCAATTGATATTGTTCTTCATGATACTTATTACGTAGTTGCCCATTTTCATTACGTTTTATCTATAGGAGCTGTATTTGCTATTATAGCAGGATTTATTCATTGATACCCTTTATTAACAGGAATAGTTATAAACCCTTCATGATTAAAGGCTCAATTTAGTATAATATTTATTGGAGTAAATCTAACTTTCTTTCCTCAACATTTTTTAGGGTTAGCTGGAATACCTCGACGATACTCAGATTTTCCTGATAGTTACTTAACTTGAAATATTATTTCTTCTTTAGGAAGAACAATTTCACTATTTGCCGTTATTTTCTTTTTATTTATTATTTGAGAAAGTATAATTACTCAACGAACACCTTCTTTC

>Brazil-COXI-D09

TTTTGATTCTTTGGACACCCAGAAGTTTATATTTTAATTTTACCCGGATTTGGAATAATTTCTCATATTATTACTCAAGAAAGCGGAAAAAAGGAAACATTTGGAACTTTAGGAATAATTTATGCTATATTAACAATTGGATTATTAGGATTTATTGTTTGAGCTCATCATATATTTACAGTAGGTATAGACGTAGATACTCGAGCTTATTTTACTTCAGCAACTATAATTATTGCTGTTCCTACAGGAATTAAAATTTTTAGTTGATTAGCAACTTTACACGGAACTCAATTAACATATAGTCCAGCCCTTCTATGATCATTAGGATTTGTATTTTTATTTACAGTTGGAGGTTTAACAGGAGTAGTATTAGCTAATTCTTCAATTGATATTGTTCTTCATGATACTTATTACGTAGTTGCCCATTTTCATTACGTTTTATCTATAGGAGCTGTATTTGCTATTATAGCAGGATTTATTCATTGATACCCTTTATTAACAGGAATAGTTATAAACCCTTCATGATTAAAGGCTCAATTTAGTATAATATTTATTGGAGTAAATCTAACTTTCTTTCCTCAACATTTTTTAGGGTTAGCTGGAATACCTCGACGATACTCAGATTTTCCTGATAGCTACTTAACTTGAAATATTATTTCTTCTTTAGGAAGAACAATTTCACTATTTGCCGTTATTTTCTTTTTATTTATTATTTGAGAAAGTATAATTACTCAACGAACACCTTCTTTC

>Brazil-COXI-D10

TTTTGATTCTTTGGACACCCAGAAGTTTATATTTTAATTTTACCCGGATTTGGAATAATTTCTCATATTATTACTCAAGAAAGCGGAAAAAAGGAAACATTTGGAACTTTAGGAATAATTTATGCTATATTAACAATTGGATTATTAGGATTTATTGTTTGAGCTCATCATATATTTACAGTAGGTATAGACGTAGATACTCGAGCTTATTTTACTTCAGCAACTATAATTATTGCTGTTCCTACAGGAATTAAAATTTTTAGTTGATTAGCAACTTTACACGGAACTCAATTAACATATAGTCCAGCCCTTCTATGATCATTAGGATTTGTATTTTTATTTACAGTTGGAGGTTTAACAGGAGTAGTATTAGCTAATTCTTCAATTGATATTGTTCTTCATGATACTTATTACGTAGTTGCCCATTTTCATTACGTTTTATCTATAGGAGCTGTATTTGCTATTATAGCAGGATTTATTCATTGATACCCTTTATTAACAGGAATAGTTATAAACCCTTCATGATTAAAGGCTCAATTTAGTATAATATTTATTGGAGTAAATCTAACTTTCTTTCCTCAACATTTTTTAGGGTTAGCTGGAATACCTCGACGATACTCAGATTTTCCTGATAGCTACTTAACTTGAAATATTATTTCTTCTTTAGGAAGAACAATTTCACTATTTGCCGTTATTTTCTTTTTATTTATTATTTGAGAAAGTATAATTACTCAACGAACACCTTCTTTC

>Brazil-COXI-D11

TTCTGATTCTTTGGACACCCAGAAGTTTATATTTTAATTTTACCCGGATTTGGAATAATTTCTCATATTATTACTCAAGAAAGTGGAAAAAAGGAAACATTTGGAACTTTAGGAATAATTTATGCTATATTAACAATTGGATTATTGGGATTTATTGTTTGAGCTCATCATATATTTACAGTAGGTATAGACGTAGATACTCGAGCTTATTTTACTTCAGCAACTATAATTATTGCTGTTCCTACAGGAATTAAAATTTTTAGTTGATTAGCAACTTTACACGGAACTCAATTAACATATAGTCCAGCCCTTCTATGATCATTAGGATTTGTATTTTTATTTACAGTTGGAGGTTTAACAGGAGTAGTATTAGCTAATTCTTCAATTGATATTGTTCTTCATGATACTTATTACGTAGTTGCCCATTTTCATTATGTTTTATCTATAGGAGCTGTATTTGCTATTATAGCAGGATTTATTCATTGATACCCTTTATTAACAGGAATAGTTATAAACCCTTCATGATTAAAGGCTCAATTTAGTATAATATTTATTGGAGTAAATCTAACTTTCTTTCCCCAACATTTTTTAGGATTAGCTGGAATACCTCGACGATACTCAGATTTTCCCGATAGTTACTTAACTTGAAATATTATTTCTTCTTTAGGAAGAACAATTTCATTATTTGCCGTTATTTTCTTTTTATTTATTATTTGAGAAAGTATAATTACTCAACGAACACCTTCTTTC

>Brazil-COXI-D12

TTTTGATTCTTTGGACACCCAGAAGTTTATATTTTAATTTTACCCGGATTTGGAATAATTTCTCATATTATTACTCAAGAAAGCGGAAAAAAGGAAACATTTGGAACTTTAGGAATAATTTATGCTATATTAACAATTGGATTATTAGGATTTATTGTTTGAGCTCATCATATATTTACAGTAGGTATAGACGTAGATACTCGAGCTTATTTTACTTCAGCAACTATAATTATTGCTGTTCCTACAGGAATTAAAATTTTTAGTTGATTAGCAACTTTACACGGAACTCAATTAACATATAGTCCAGCCCTTCTATGATCATTAGGATTTGTATTTTTATTTACAGTTGGAGGTTTAACAGGAGTAGTATTAGCTAATTCTTCAATTGATATTGTTCTTCATGATACTTATTACGTAGTTGCCCATTTTCATTACGTTTTATCTATAGGAGCTGTATTTGCTATTATAGCAGGATTTATTCATTGATACCCTTTATTAACAGGAATAGTTATAAACCCTTCATGATTAAAGGCTCAATTTAGTATAATATTTATTGGAGTAAATCTAACTTTCTTTCCTCAACATTTTTTAGGGTTAGCTGGAATACCTCGACGATACTCAGATTTTCCTGATAGCTACTTAACTTGAAATATTATTTCTTCTTTAGGAAGAACAATTTCACTATTTGCCGTTATTTTCTTTTTATTTATTATTTGAGAAAGTATAATTACTCAACGAACACCTTCTTTC

>Brazil-COXI-E09

TTTTGATTCTTTGGACACCCAGAAGTTTATATTTTAATTTTACCCGGATTTGGAATAATTTCTCATATTATTACTCAAGAAAGCGGAAAAAAGGAAACATTTGGAACTTTAGGAATAATTTATGCTATATTAACAATTGGATTATTAGGATTTATTGTTTGAGCTCATCATATATTTACAGTAGGTATAGACGTAGATACTCGAGCTTATTTTACTTCAGCAACTATAATTATTGCTGTTCCTACAGGAATTAAAATTTTTAGTTGATTAGCAACTTTACACGGAACTCAATTAACATATAGTCCAGCCCTTCTATGATCATTAGGATTTGTATTTTTATTTACAGTTGGAGGTTTAACAGGAGTAGTATTAGCTAATTCTTCAATTGATATTGTTCTTCATGATACTTATTACGTAGTTGCCCATTTTCATTACGTTTTATCTATAGGAGCTGTATTTGCTATTATAGCAGGATTTATTCATTGATACCCTTTATTAACAGGAATAGTTATAAACCCTTCATGATTAAAGGCTCAATTTAGTATAATATTTATTGGAGTAAATCTAACTTTCTTTCCTCAACATTTTTTAGGGTTAGCTGGAATACCTCGACGATACTCAGATTTTCCTGATAGCTACTTAACTTGAAATATTATTTCTTCTTTAGGAAGAACAATTTCACTATTTGCCGTTATTTTCTTTTTATTTATTATTTGAGAAAGTATAATTACTCAACGAACACCTTCTTTC

>Brazil-COXI-E10

TTTTGATTCTTTGGACACCCAGAAGTTTATATTTTAATTTTACCCGGATTTGGAATAATTTCTCATATTATTACTCAAGAAAGTGGAAAAAAGGAAACATTTGGAACTTTAGGAATAATTTATGCTATATTAACAATTGGATTATTGGGATTTATTGTTTGAGCTCATCATATATTTACAGTAGGTATAGATGTAGATACTCGAGCTTATTTTACTTCAGCAACTATAATTATTGCTGTTCCTACAGGAATTAAAATTTTTAGTTGATTAGCAACTTTACACGGAACTCAATTAACATATAGTCCAGCCCTTCTATGATCATTAGGATTTGTATTTTTATTTACAGTTGGAGGTTTAACAGGAGTAGTATTAGCTAATTCTTCAATTGATATTGTTCTTCATGATACTTATTACGTAGTTGCCCATTTTCATTACGTTTTATCTATAGGAGCTGTATTTGCTATTATAGCAGGATTTATTCATTGATACCCTTTATTAACAGGAATAGTTATAAACCCTTCATGATTAAAGGCTCAATTTAGTATAATATTTATTGGAGTAAATCTAACTTTCTTTCCTCAACATTTTTTAGGGTTAGCTGGAATACCTCGACGATACTCAGATTTTCCTGATAGTTACTTAACTTGAAATATTATTTCTTCTTTAGGAAGAACAATTTCACTATTTGCCGTTATTTTCTTTTTATTTATTATTTGAGAAAGTATAATTACTCAACGAACACCTTCTTTC

>Brazil-COXI-E11

TTTTGATTCTTTGGACACCCAGAAGTTTATATTTTAATTTTACCCGGATTTGGAATAATTTCTCATATTATTACTCAAGAAAGCGGAAAAAAGGAAACATTTGGAACTTTAGGAATAATTTATGCTATATTAACAATTGGATTATTAGGATTTATTGTTTGAGCTCATCATATATTTACAGTAGGTATAGACGTAGATACTCGAGCTTATTTTACTTCAGCAACTATAATTATTGCTGTTCCTACAGGAATTAAAATTTTTAGTTGATTAGCAACTTTACACGGAACTCAATTAACATATAGTCCAGCCCTTCTATGATCATTAGGATTTGTATTTTTATTTACAGTTGGAGGTTTAACAGGAGTAGTATTAGCTAATTCTTCAATTGATATTGTTCTTCATGATACTTATTACGTAGTTGCCCATTTTCATTACGTTTTATCTATAGGAGCTGTATTTGCTATTATAGCAGGATTTATTCATTGATACCCTTTATTAACAGGAATAGTTATAAACCCTTCATGATTAAAGGCTCAATTTAGTATAATATTTATTGGAGTAAATCTAACTTTCTTTCCTCAACATTTTTTAGGGTTAGCTGGAATACCTCGACGATACTCAGATTTTCCTGATAGCTACTTAACTTGAAATATTATTTCTTCTTTAGGAAGAACAATTTCACTATTTGCCGTTATTTTCTTTTTATTTATTATTTGAGAAAGTATAATTACTCAACGAACACCTTCTTTC

>Brazil-COXI-E12

TTTTGATTCTTTGGACACCCAGAAGTTTATATTTTAATTTTACCCGGATTTGGAATAATTTCTCATATTATTACTCAAGAAAGCGGAAAAAAGGAAACATTTGGAACTTTAGGAATAATTTATGCTATATTAACAATTGGATTATTAGGATTTATTGTTTGAGCTCATCATATATTTACAGTAGGTATAGACGTAGATACTCGAGCTTATTTTACTTCAGCAACTATAATTATTGCTGTTCCTACAGGAATTAAAATTTTTAGTTGATTAGCAACTTTACACGGAACTCAATTAACATATAGTCCAGCCCTTCTATGATCATTAGGATTTGTATTTTTATTTACAGTTGGAGGTTTAACAGGAGTAGTATTAGCTAATTCTTCAATTGATATTGTTCTTCATGATACTTATTACGTAGTTGCCCATTTTCATTACGTTTTATCTATAGGAGCTGTATTTGCTATTATAGCAGGATTTATTCATTGATACCCTTTATTAACAGGAATAGTTATAAACCCTTCATGATTAAAGGCTCAATTTAGTATAATATTTATTGGAGTAAATCTAACTTTCTTTCCTCAACATTTTTTAGGGTTAGCTGGAATACCTCGACGATACTCAGATTTTCCTGATAGCTACTTAACTTGAAATATTATTTCTTCTTTAGGAAGAACAATTTCACTATTTGCCGTTATTTTCTTTTTATTTATTATTTGAGAAAGTATAATTACTCAACGAACACCTTCTTTC

>Brazil-COXI-F09

TTTTGATTCTTTGGACACCCAGAAGTTTATATTTTAATTTTACCCGGATTTGGAATAATTTCTCATATTATTACTCAAGAAAGCGGAAAAAAGGAAACATTTGGAACTTTAGGAATAATTTATGCTATATTAACAATTGGATTATTAGGATTTATTGTTTGAGCTCATCATATATTTACAGTAGGTATAGACGTAGATACTCGAGCTTATTTTACTTCAGCAACTATAATTATTGCTGTTCCTACAGGAATTAAAATTTTTAGTTGATTAGCAACTTTACACGGAACTCAATTAACATATAGTCCAGCCCTTCTATGATCATTAGGATTTGTATTTTTATTTACAGTTGGAGGTTTAACAGGAGTAGTATTAGCTAATTCTTCAATTGATATTGTTCTTCATGATACTTATTACGTAGTTGCCCATTTTCATTACGTTTTATCTATAGGAGCTGTATTTGCTATTATAGCAGGATTTATTCATTGATACCCTTTATTAACAGGAATAGTTATAAACCCTTCATGATTAAAGGCTCAATTTAGTATAATATTTATTGGAGTAAATCTAACTTTCTTTCCTCAACATTTTTTAGGGTTAGCTGGAATACCTCGACGATACTCAGATTTTCCTGATAGCTACTTAACTTGAAATATTATTTCTTCTTTAGGAAGAACAATTTCACTATTTGCCGTTATTTTCTTTTTATTTATTATTTGAGAAAGTATAATTACTCAACGAACACCTTCTTTC

>Brazil-COXI-F10

TTTTGATTCTTTGGACACCCAGAAGTTTATATTTTAATTTTACCCGGATTTGGAATAATTTCTCATATTATTACTCAAGAAAGCGGAAAAAAGGAAACATTTGGAACTTTAGGAATAATTTATGCTATATTAACAATTGGATTATTAGGATTTATTGTTTGAGCTCATCATATATTTACAGTAGGTATAGACGTAGATACTCGAGCTTATTTTACTTCAGCAACTATAATTATTGCTGTTCCTACAGGAATTAAAATTTTTAGTTGATTAGCAACTTTACACGGAACTCAATTAACATATAGTCCAGCCCTTCTATGATCATTAGGATTTGTATTTTTATTTACAGTTGGAGGTTTAACAGGAGTAGTATTAGCTAATTCTTCAATTGATATTGTTCTTCATGATACTTATTACGTAGTTGCCCATTTTCATTACGTTTTATCTATAGGAGCTGTATTTGCTATTATAGCAGGATTTATTCATTGATACCCTTTATTAACAGGAATAGTTATAAACCCTTCATGATTAAAGGCTCAATTTAGTATAATATTTATTGGAGTAAATCTAACTTTCTTTCCTCAACATTTTTTAGGGTTAGCTGGAATACCTCGACGATACTCAGATTTTCCTGATAGCTACTTAACTTGAAATATTATTTCTTCTTTAGGAAGAACAATTTCACTATTTGCCGTTATTTTCTTTTTATTTATTATTTGAGAAAGTATAATTACTCAACGAACACCTTCTTTC

>Brazil-COXI-G07

TTTTGATTCTTTGGACACCCAGAAGTTTATATTTTAATTTTACCCGGATTTGGAATAATTTCTCATATTATTACTCAAGAAAGCGGAAAAAAGGAAACATTTGGAACTTTAGGAATAATTTATGCTATATTAACAATTGGATTATTAGGATTTATTGTTTGAGCTCATCATATATTTACAGTAGGTATAGACGTAGATACTCGAGCTTATTTTACTTCAGCAACTATAATTATTGCTGTTCCTACAGGAATTAAAATTTTTAGTTGATTAGCAACTTTACACGGAACTCAATTAACATATAGTCCAGCCCTTCTATGATCATTAGGATTTGTATTTTTATTTACAGTTGGAGGTTTAACAGGAGTAGTATTAGCTAATTCTTCAATTGATATTGTTCTTCATGATACTTATTACGTAGTTGCCCATTTTCATTACGTTTTATCTATAGGAGCTGTATTTGCTATTATAGCAGGATTTATTCATTGATACCCTTTATTAACAGGAATAGTTATAAACCCTTCATGATTAAAGGCTCAATTTAGTATAATATTTATTGGAGTAAATCTAACTTTCTTTCCTCAACATTTTTTAGGGTTAGCTGGAATACCTCGACGATACTCAGATTTTCCTGATAGCTACTTAACTTGAAATATTATTTCTTCTTTAGGAAGAACAATTTCACTATTTGCCGTTATTTTCTTTTTATTTATTATTTGAGAAAGTATAATTACTCAACGAACACCTTCTTTC

>Brazil-COXI-G08

TTTTGATTCTTTGGACACCCAGAAGTTTATATTTTAATTTTACCCGGATTTGGAATAATTTCTCATATTATTACTCAAGAAAGCGGAAAAAAGGAAACATTTGGAACTTTAGGAATAATTTATGCTATATTAACAATTGGATTATTAGGATTTATTGTTTGAGCTCATCATATATTTACAGTAGGTATAGACGTAGATACTCGAGCTTATTTTACTTCAGCAACTATAATTATTGCTGTTCCTACAGGAATTAAAATTTTTAGTTGATTAGCAACTTTACACGGAACTCAATTAACATATAGTCCAGCCCTTCTATGATCATTAGGATTTGTATTTTTATTTACAGTTGGAGGTTTAACAGGAGTAGTATTAGCTAATTCTTCAATTGATATTGTTCTTCATGATACTTATTACGTAGTTGCCCATTTTCATTACGTTTTATCTATAGGAGCTGTATTTGCTATTATAGCAGGATTTATTCATTGATACCCTTTATTAACAGGAATAGTTATAAACCCTTCATGATTAAAGGCTCAATTTAGTATAATATTTATTGGAGTAAATCTAACTTTCTTTCCTCAACATTTTTTAGGGTTAGCTGGAATACCTCGACGATACTCAGATTTTCCTGATAGCTACTTAACTTGAAATATTATTTCTTCTTTAGGAAGAACAATTTCACTATTTGCCGTTATTTTCTTTTTATTTATTATTTGAGAAAGTATAATTACTCAACGAACACCTTCTTTC

>Brazil-COXI-G09

TTTTGATTCTTTGGACACCCAGAAGTTTATATTTTAATTTTACCCGGATTTGGAATAATTTCTCATATTATTACTCAAGAAAGCGGAAAAAAGGAAACATTTGGAACTTTAGGAATAATTTATGCTATATTAACAATTGGATTATTAGGATTTATTGTTTGAGCTCATCATATATTTACAGTAGGTATAGACGTAGATACTCGAGCTTATTTTACTTCAGCAACTATAATTATTGCTGTTCCTACAGGAATTAAAATTTTTAGTTGATTAGCAACTTTACACGGAACTCAATTAACATATAGTCCAGCCCTTCTATGATCATTAGGATTTGTATTTTTATTTACAGTTGGAGGTTTAACAGGAGTAGTATTAGCTAATTCTTCAATTGATATTGTTCTTCATGATACTTATTACGTAGTTGCCCATTTTCATTACGTTTTATCTATAGGAGCTGTATTTGCTATTATAGCAGGATTTATTCATTGATACCCTTTATTAACAGGAATAGTTATAAACCCTTCATGATTAAAGGCTCAATTTAGTATAATATTTATTGGAGTAAATCTAACTTTCTTTCCTCAACATTTTTTAGGGTTAGCTGGAATACCTCGACGATACTCAGATTTTCCTGATAGCTACTTAACTTGAAATATTATTTCTTCTTTAGGAAGAACAATTTCACTATTTGCCGTTATTTTCTTTTTATTTATTATTTGAGAAAGTATAATTACTCAACGAACACCTTCTTTC

>Brazil-COXI-G10

TTCTGATTCTTTGGACACCCAGAAGTTTATATTTTAATTTTACCCGGATTTGGAATAATTTCTCATATTATTACTCAAGAAAGTGGAAAAAAGGAAACATTTGGAACTTTAGGAATAATTTATGCTATATTAACAATTGGATTATTGGGATTTATTGTTTGAGCTCATCATATATTTACAGTAGGTATAGACGTAGATACTCGAGCTTATTTTACTTCAGCAACTATAATTATTGCTGTTCCTACAGGAATTAAAATTTTTAGTTGATTAGCAACTTTACACGGAACTCAATTAACATATAGTCCAGCCCTTCTATGATCATTAGGATTTGTATTTTTATTTACAGTTGGAGGTTTAACAGGAGTAGTATTAGCTAATTCTTCAATTGATATTGTTCTTCATGATACTTATTACGTAGTTGCCCATTTTCATTATGTTTTATCTATAGGAGCTGTATTTGCTATTATAGCAGGATTTATTCATTGATACCCTTTATTAACAGGAATAGTTATAAACCCTTCATGATTAAAGGCTCAATTTAGTATAATATTTATTGGAGTAAATCTAACTTTCTTTCCCCAACATTTTTTAGGATTAGCTGGAATACCTCGACGATACTCAGATTTTCCCGATAGTTACTTAACTTGAAATATTATTTCTTCTTTAGGAAGAACAATTTCATTATTTGCCGTTATTTTCTTTTTATTTATTATTTGAGAAAGTATAATTACTCAACGAACACCTTCTTTC

>Brazil-COXI-H07

TTCTGATTCTTTGGACACCCAGAAGTTTATATTTTAATTTTACCCGGATTTGGAATAATTTCTCATATTATTACTCAAGAAAGTGGAAAAAAGGAAACATTTGGAACTTTAGGAATAATTTATGCTATATTAACAATTGGATTATTGGGATTTATTGTTTGAGCTCATCATATATTTACAGTAGGTATAGACGTAGATACTCGAGCTTATTTTACTTCAGCAACTATAATTATTGCTGTTCCTACAGGAATTAAAATTTTTAGTTGATTAGCAACTTTACACGGAACTCAATTAACATATAGTCCAGCCCTTCTATGATCATTAGGATTTGTATTTTTATTTACAGTTGGAGGTTTAACAGGAGTAGTATTAGCTAATTCTTCAATTGATATTGTTCTTCATGATACTTATTACGTAGTTGCCCATTTTCATTATGTTTTATCTATAGGAGCTGTATTTGCTATTATAGCAGGATTTATTCATTGATACCCTTTATTAACAGGAATAGTTATAAACCCTTCATGATTAAAGGCTCAATTTAGTATAATATTTATTGGAGTAAATCTAACTTTCTTTCCCCAACATTTTTTAGGATTAGCTGGAATACCTCGACGATACTCAGATTTTCCCGATAGTTACTTAACTTGAAATATTATTTCTTCTTTAGGAAGAACAATTTCATTATTTGCCGTTATTTTCTTTTTATTTATTATTTGAGAAAGTATAATTACTCAACGAACACCTTCTTTC

>Brazil-COXI-H08

TTTTGATTCTTTGGACACCCAGAAGTTTATATTTTAATTTTACCCGGATTTGGAATAATTTCTCATATTATTACTCAAGAAAGTGGAAAAAAGGAAACATTTGGAACTTTAGGAATAATTTATGCTATATTAACAATTGGATTATTGGGATTTATTGTTTGAGCTCATCATATATTTACAGTAGGTATAGATGTAGATACTCGAGCTTATTTTACTTCAGCAACTATAATTATTGCTGTTCCTACAGGAATTAAAATTTTTAGTTGATTAGCAACTTTACACGGAACTCAATTAACATATAGTCCAGCCCTTCTATGATCATTAGGATTTGTATTTTTATTTACAGTTGGAGGTTTAACAGGAGTAGTATTAGCTAATTCTTCAATTGATATTGTTCTTCATGATACTTATTACGTAGTTGCCCATTTTCATTACGTTTTATCTATAGGAGCTGTATTTGCTATTATAGCAGGATTTATTCATTGATACCCTTTATTAACAGGAATAGTTATAAACCCTTCATGATTAAAGGCTCAATTTAGTATAATATTTATTGGAGTAAATCTAACTTTCTTTCCTCAACATTTTTTAGGGTTAGCTGGAATACCTCGACGATACTCAGATTTTCCTGATAGTTACTTAACTTGAAATATTATTTCTTCTTTAGGAAGAACAATTTCACTATTTGCCGTTATTTTCTTTTTATTTATTATTTGAGAAAGTATAATTACTCAACGAACACCTTCTTTC

>Brazil-COXI-H09

TTTTGATTCTTTGGACACCCAGAAGTTTATATTTTAATTTTACCCGGATTTGGAATAATTTCTCATATTATTACTCAAGAAAGTGGAAAAAAGGAAACATTTGGAACTTTAGGAATAATTTATGCTATATTAACAATTGGATTATTGGGATTTATTGTTTGAGCTCATCATATATTTACAGTAGGTATAGATGTAGATACTCGAGCTTATTTTACTTCAGCAACTATAATTATTGCTGTTCCTACAGGAATTAAAATTTTTAGTTGATTAGCAACTTTACACGGAACTCAATTAACATATAGTCCAGCCCTTCTATGATCATTAGGATTTGTATTTTTATTTACAGTTGGAGGTTTAACAGGAGTAGTATTAGCTAATTCTTCAATTGATATTGTTCTTCATGATACTTATTACGTAGTTGCCCATTTTCATTACGTTTTATCTATAGGAGCTGTATTTGCTATTATAGCAGGATTTATTCATTGATACCCTTTATTAACAGGAATAGTTATAAACCCTTCATGATTAAAGGCTCAATTTAGTATAATATTTATTGGAGTAAATCTAACTTTCTTTCCTCAACATTTTTTAGGGTTAGCTGGAATACCTCGACGATACTCAGATTTTCCTGATAGTTACTTAACTTGAAATATTATTTCTTCTTTAGGAAGAACAATTTCACTATTTGCCATTATTTTCTTTTTATTTATTATTTGAGAAAGTATAATTACTCAACGAACACCTTCTTTC

>Brazil-COXI-H10

TTTTGATTCTTTGGACACCCAGAAGTTTATATTTTAATTTTACCCGGATTTGGAATAATTTCTCATATTATTACTCAAGAAAGCGGAAAAAAGGAAACATTTGGAACTTTAGGAATAATTTATGCTATATTAACAATTGGATTATTAGGATTTATTGTTTGAGCTCATCATATATTTACAGTAGGTATAGACGTAGATACTCGAGCTTATTTTACTTCAGCAACTATAATTATTGCTGTTCCTACAGGAATTAAAATTTTTAGTTGATTAGCAACTTTACACGGAACTCAATTAACATATAGTCCAGCCCTTCTATGATCATTAGGATTTGTATTTTTATTTACAGTTGGAGGTTTAACAGGAGTAGTATTAGCTAATTCTTCAATTGATATTGTTCTTCATGATACTTATTACGTAGTTGCCCATTTTCATTACGTTTTATCTATAGGAGCTGTATTTGCTATTATAGCAGGATTTATTCATTGATACCCTTTATTAACAGGAATAGTTATAAACCCTTCATGATTAAAGGCTCAATTTAGTATAATATTTATTGGAGTAAATCTAACTTTCTTTCCTCAACATTTTTTAGGGTTAGCTGGAATACCTCGACGATACTCAGATTTTCCTGATAGCTACTTAACTTGAAATATTATTTCTTCTTTAGGAAGAACAATTTCACTATTTGCCGTTATTTTCTTTTTATTTATTATTTGAGAAAGTATAATTACTCAACGAACACCTTCTTTC

>Australia-COXI-2F11

TTCTGATTCTTTGGACACCCAGAAGTTTATATTTTAATTTTACCCGGATTTGGAATAATTTCTCATATTATTACTCAAGAAAGTGGAAAAAAGGAAACATTTGGAACTTTAGGAATAATTTATGCTATATTAACAATTGGATTATTGGGATTTATTGTTTGAGCTCATCATATATTTACAGTAGGTATAGACGTAGATACTCGAGCTTATTTTACTTCAGCAACTATAATTATTGCTGTTCCTACAGGAATTAAAATTTTTAGTTGATTAGCAACTTTACACGGAACTCAATTAACATATAGTCCAGCCCTTCTATGATCATTAGGATTTGTATTTTTATTTACAGTTGGAGGTTTAACAGGAGTAGTATTAGCTAATTCTTCAATTGATATTGTTCTTCATGATACTTATTATGTAGTTGCCCATTTTCATTATGTTTTATCTATAGGAGCTGTATTTGCTATTATAGCAGGATTTATTCATTGATACCCTTTATTAACAGGAATAGTTATAAACCCTTCATGATTAAAAGCTCAATTTAGTATAATATTTATTGGAGTAAATCTAACTTTCTTTCCCCAACATTTTTTAGGATTAGCTGGAATACCTCGACGATACTCAGATTTTCCCGATAGTTACTTAACTTGAAATATTATTTCTTCTTTAGGAAGAACAATTTCATTATTTGCCGTTATTTTCTTTTTATTTATTATTTGAGAAAGTATAATTACTCAACGAACACCGTCTTTC

>Australia-COXI-2F12

TTCTGATTCTTTGGACACCCAGAAGTTTATATTTTAATTTTACCCGGATTTGGAATAATTTCTCATATTATTACTCAAGAAAGTGGAAAAAAGGAAACATTTGGAACTTTAGGAATAATTTATGCTATATTAACAATTGGATTATTGGGATTTATTGTTTGAGCTCATCATATATTTACAGTAGGTATAGACGTAGATACTCGAGCTTATTTTACTTCAGCAACTATAATTATTGCTGTTCCTACAGGAATTAAAATTTTTAGTTGATTAGCAACTTTACACGGAACTCAATTAACATATAGTCCAGCCCTTCTATGATCATTAGGATTTGTATTTTTATTTACAGTTGGAGGTTTAACAGGAGTAGTATTAGCTAATTCTTCAATTGATATTGTTCTTCATGATACTTATTATGTAGTTGCCCATTTTCATTATGTTTTATCTATAGGAGCTGTATTTGCTATTATAGCAGGATTTATTCATTGATACCCTTTATTAACAGGAATAGTTATAAACCCTTCATGATTAAAGGCTCAATTTAGTATAATATTTATTGGAGTAAATTTAACTTTCTTCCCCCAACATTTTTTAGGATTAGCTGGAATACCTCGACGATACTCGGATTTTCCCGATAGTTACTTAACTTGAAATATTATTTCTTCTTTAGGAAGAACAATTTCATTATTTGCCGTTATTTTCTTTTTATTTATTATTTGAGAAAGTATAATTACTCAACGAACACCTTCTTTC

>Australia-COXI-2G11

TTCTGATTCTTTGGACACCCAGAAGTTTATATTTTAATTTTACCCGGATTTGGAATAATTTCTCATATTATTACTCAAGAAAGTGGAAAAAAGGAAACATTTGGAACTTTAGGAATAATTTATGCTATATTAACAATTGGATTATTGGGATTTATTGTTTGAGCTCATCATATATTTACAGTAGGTATAGACGTAGATACTCGAGCTTATTTTACTTCAGCAACTATAATTATTGCTGTTCCTACAGGAATTAAAATTTTTAGTTGATTAGCAACTTTACACGGAACTCAATTAACATATAGTCCAGCCCTTCTATGATCATTAGGATTTGTATTTTTATTTACAGTTGGAGGTTTAACAGGAGTAGTATTAGCTAATTCTTCAATTGATATTGTTCTTCATGATACTTATTATGTAGTTGCCCATTTTCATTATGTTTTATCTATAGGAGCTGTATTTGCTATTATAGCAGGATTTATTCATTGATACCCTTTATTAACAGGAATAGTTATAAACCCTTCATGATTAAAGGCTCAATTTAGTATAATATTTATTGGAGTAAATTTAACTTTCTTCCCCCAACATTTTTTAGGATTAGCTGGAATACCTCGACGATACTCGGATTTTCCCGATAGTTACTTAACTTGAAATATTATTTCTTCTTTAGGAAGAACAATTTCATTATTTGCCGTTATTTTCTTTTTATTTATTATTTGAGAAAGTATAATTACTCAACGAACACCTTCTTTC

>Australia-COXI-2G12

TTCTGATTCTTTGGACACCCAGAAGTTTATATTTTAATTTTACCCGGATTTGGAATAATTTCTCATATTATTACTCAAGAAAGTGGAAAAAAGGAAACATTTGGAACTTTAGGAATAATTTATGCTATATTAACAATTGGATTATTGGGATTTATTGTTTGAGCTCATCATATATTTACAGTAGGTATAGACGTAGATACTCGAGCTTATTTTACTTCAGCAACTATAATTATTGCTGTTCCTACAGGAATTAAAATTTTTAGTTGATTAGCAACTTTACACGGAACTCAATTAACATATAGTCCAGCCCTTCTATGATCATTAGGATTTGTATTTTTATTTACAGTTGGAGGTTTAACAGGAGTAGTATTAGCTAATTCTTCAATTGATATTGTTCTTCATGATACTTATTATGTAGTTGCCCATTTTCATTATGTTTTATCTATAGGAGCTGTATTTGCTATTATAGCAGGATTTATTCATTGATACCCTTTATTAACAGGAATAGTTATAAACCCTTCATGATTAAAGGCTCAATTTAGTATAATATTTATTGGAGTAAATTTAACTTTCTTCCCCCAACATTTTTTAGGATTAGCTGGAATACCTCGACGATACTCGGATTTTCCCGATAGTTACTTAACTTGAAATATTATTTCTTCTTTAGGAAGAACAATTTCATTATTTGCCGTTATTTTCTTTTTATTTATTATTTGAGAAAGTATAATTACTCAACGAACACCTTCTTTC

>Australia-COXI-2H11

TTCTGATTCTTTGGACACCCAGAAGTTTATATTTTAATTTTACCCGGATTTGGAATAATTTCTCATATTATTACTCAAGAAAGTGGAAAAAAGGAAACATTTGGAACTTTAGGAATAATTTATGCTATATTAACAATTGGATTATTGGGATTTATTGTTTGAGCTCATCATATATTTACAGTAGGTATAGACGTAGATACTCGAGCTTATTTTACTTCAGCAACTATAATTATTGCTGTTCCTACAGGAATTAAAATTTTTAGTTGATTAGCAACTTTACACGGAACTCAATTAACATATAGTCCAGCCCTTCTATGATCATTAGGATTTGTATTTTTATTTACAGTTGGAGGTTTAACAGGAGTAGTATTAGCTAATTCTTCAATTGATATTGTTCTTCATGATACTTATTATGTAGTTGCCCATTTTCATTATGTTTTATCTATAGGAGCTGTATTTGCTATTATAGCAGGATTTATTCATTGATACCCTTTATTAACAGGAATAGTTATAAACCCTTCATGATTAAAGGCTCAATTTAGTATAATATTTATTGGAGTAAATCTAACTTTCTTTCCCCAACATTTTTTAGGATTAGCTGGAATACCTCGACGATACTCAGATTTTCCCGATAGTTACTTAACTTGAAATATTATTTCTTCTTTAGGAAGAACAATTTCATTATTTGCCGTTATTTTCTTTTTATTTATTATTTGAGAAAGTATAATTACTCAACGAACACCGTCTTTC

>Australia-COXI-2H12

TTCTGATTCTTTGGACACCCAGAAGTTTATATTTTAATTTTACCCGGATTTGGAATAATTTCTCATATTATTACTCAAGAAAGTGGAAAAAAGGAAACATTTGGAACTTTAGGAATAATTTATGCTATATTAACAATTGGATTATTGGGATTTATTGTTTGAGCTCATCATATATTTACAGTAGGTATAGACGTAGATACTCGAGCTTATTTTACTTCAGCAACTATAATTATTGCTGTTCCTACAGGAATTAAAATTTTTAGTTGATTAGCAACTTTACACGGAACTCAATTAACATATAGTCCAGCCCTTCTATGATCATTAGGATTTGTATTTTTATTTACAGTTGGAGGTTTAACAGGAGTAGTATTAGCTAATTCTTCAATTGATATTGTTCTTCATGATACTTATTATGTAGTTGCCCATTTTCATTATGTTTTATCTATAGGAGCTGTATTTGCTATTATAGCAGGATTTATTCATTGATACCCTTTATTAACAGGAATAGTTATAAACCCTTCATGATTAAAGGCTCAATTTAGTATAATATTTATTGGAGTAAATCTAACTTTCTTCCCCCAACATTTTTTAGGATTAGCTGGAATACCTCGACGATACTCGGATTTTCCCGATAGTTACTTAACTTGAAATATTATTTCTTCTTTAGGAAGAACAATTTCATTATTTGCCGTTATTTTCTTTTTATTTATTATTTGAGAAAGTATAATTACTCAACGAACACCTTCTTTC

>Australia-COXI-03

TTCTGATTCTTTGGACACCCAGAAGTTTATATTTTAATTTTACCCGGATTTGGAATAATTTCTCATATTATTACTCAAGAAAGTGGAAAAAAGGAAACATTTGGAACTTTAGGAATAATTTATGCTATATTAACAATTGGATTATTGGGATTTATTGTTTGAGCTCATCATATATTTACAGTAGGTATAGACGTAGATACTCGAGCTTATTTTACTTCAGCAACTATAATTATTGCTGTTCCTACAGGAATTAAAATTTTTAGTTGATTAGCAACTTTACACGGAACTCAATTAACATATAGTCCAGCCCTTCTATGATCATTAGGATTTGTATTTTTATTTACAGTTGGAGGTTTAACAGGAGTAGTATTAGCTAATTCTTCAATTGATATTGTTCTTCATGATACTTATTATGTAGTTGCCCATTTTCATTATGTTTTATCTATAGGAGCTGTATTTGCTATTATAGCAGGATTTATTCATTGATACCCTTTATTAACAGGAATAGTTATAAACCCTTCATGATTAAAGGCTCAATTTAGTATAATATTTATTGGAGTAAATCTAACTTTCTTCCCCCAACATTTTTTAGGATTAGCTGGAATACCTCGACGATACTCGGATTTTCCCGATAGTTACTTAACTTGAAATATTATTTCTTCTTTAGGAAGAACAATTTCATTATTTGCCGTTATTTTCTTTTTATTTATTATTTGAGAAAGTATAATTACTCAACGAACACCTTCTTTC

>Australia-COXI-04

TTCTGATTCTTTGGACACCCAGAAGTTTATATTTTAATTTTACCCGGATTTGGAATAATTTCTCATATTATTACTCAAGAAAGTGGAAAAAAGGAAACATTTGGAACTTTAGGAATAATTTATGCTATATTAACAATTGGATTATTGGGATTTATTGTTTGAGCTCATCATATATTTACAGTAGGTATAGACGTAGATACTCGAGCTTATTTTACTTCAGCAACTATAATTATTGCTGTTCCTACAGGAATTAAAATTTTTAGTTGATTAGCAACTTTACACGGAACTCAATTAACATATAGTCCAGCCCTTCTATGATCATTAGGATTTGTATTTTTATTTACAGTTGGAGGTTTAACAGGAGTAGTATTAGCTAATTCTTCAATTGATATTGTTCTTCATGATACTTATTATGTAGTTGCCCATTTTCATTATGTTTTATCTATAGGAGCTGTATTTGCTATTATAGCAGGATTTATTCATTGATACCCTTTATTAACAGGAATAGTTATAAACCCTTCATGATTAAAGGCTCAATTTAGTATAATATTTATTGGAGTAAATCTAACTTTCTTCCCCCAACATTTTTTAGGATTAGCTGGAATACCTCGACGATACTCGGATTTTCCCGATAGTTACTTAACTTGAAATATTATTTCTTCTTTAGGAAGAACAATTTCATTATTTGCCGTTATTTTCTTTTTATTTATTATTTGAGAAAGTATAATTACTCAACGAACACCTTCTTTC

>Australia-COXI-05

TTCTGATTCTTTGGACACCCAGAAGTTTATATTTTAATTTTACCCGGATTTGGAATAATTTCTCATATTATTACTCAAGAAAGTGGAAAAAAGGAAACATTTGGAACTTTAGGAATAATTTATGCTATATTAACAATTGGATTATTGGGATTTATTGTTTGAGCTCATCATATATTTACAGTAGGTATAGACGTAGATACTCGAGCTTATTTTACTTCAGCAACTATAATTATTGCTGTTCCTACAGGAATTAAAATTTTTAGTTGATTAGCAACTTTACACGGAACTCAATTAACATATAGTCCAGCCCTTCTATGATCATTAGGATTTGTATTTTTATTTACAGTTGGAGGTTTAACAGGAGTAGTATTAGCTAATTCTTCAATTGATATTGTTCTTCATGATACTTATTATGTAGTTGCCCATTTTCATTATGTTTTATCTATAGGAGCTGTATTTGCTATTATAGCAGGATTTATTCATTGATACCCTTTATTAACAGGAATAGTTATAAACCCTTCATGATTAAAGGCTCAATTTAGTATAATATTTATTGGAGTAAATCTAACTTTCTTCCCCCAACATTTTTTAGGATTAGCTGGAATACCTCGACGATACTCGGATTTTCCCGATAGTTACTTAACTTGAAATATTATTTCTTCTTTAGGAAGAACAATTTCATTATTTGCCGTTATTTTCTTTTTATTTATTATTTGAGAAAGTATAATTACTCAACGAACACCTTCTTTC

>Australia-COXI-06

TTCTGATTCTTTGGACACCCAGAAGTTTATATTTTAATTTTACCCGGATTTGGAATAATTTCTCATATTATTACTCAAGAAAGTGGAAAAAAGGAAACATTTGGAACTTTAGGAATAATTTATGCTATATTAACAATTGGATTATTGGGATTTATTGTTTGAGCTCATCATATATTTACAGTAGGTATAGACGTAGATACTCGAGCTTATTTTACTTCAGCAACTATAATTATTGCTGTTCCTACAGGAATTAAAATTTTTAGTTGATTAGCAACTTTACACGGAACTCAATTAACATATAGTCCAGCCCTTCTATGATCATTAGGATTTGTATTTTTATTTACAGTTGGAGGTTTAACAGGAGTAGTATTAGCTAATTCTTCAATTGATATTGTTCTTCATGATACTTATTATGTAGTTGCCCATTTTCATTATGTTTTATCTATAGGAGCTGTATTTGCTATTATAGCAGGATTTATTCATTGATACCCTTTATTAACAGGAATAGTTATAAACCCTTCATGATTAAAGGCTCAATTTAGTATAATATTTATTGGAGTAAATCTAACTTTCTTTCCCCAACATTTTTTAGGATTAGCTGGAATACCTCGACGATACTCAGATTTTCCCGATAGTTACTTAACTTGAAATATTATTTCTTCTTTAGGAAGAACAATTTCATTATTTGCCGTTATTTTCTTTTTATTTATTATTTGAGAAAGTATAATTACTCAACGAACACCTTCTTTC

>Australia-COXI-07

TTCTGATTCTTTGGACACCCAGAAGTTTATATTTTAATTTTACCCGGATTTGGAATAATTTCTCATATTATTACTCAAGAAAGTGGAAAAAAGGAAACATTTGGAACTTTAGGAATAATTTATGCTATATTAACAATTGGATTATTGGGATTTATTGTTTGAGCTCATCATATATTTACAGTAGGTATAGACGTAGATACTCGAGCTTATTTTACTTCAGCAACTATAATTATTGCTGTTCCTACAGGAATTAAAATTTTTAGTTGATTAGCAACTTTACACGGAACTCAATTAACATATAGTCCAGCCCTTCTATGATCATTAGGATTTGTATTTTTATTTACAGTTGGAGGTTTAACAGGAGTAGTATTAGCTAATTCTTCAATTGATATTGTTCTTCATGATACTTATTATGTAGTTGCCCATTTTCATTATGTTTTATCTATAGGAGCTGTATTTGCTATTATAGCAGGATTTATTCATTGATACCCTTTATTAACAGGAATAGTTATAAACCCTTCATGATTAAAGGCTCAATTTAGTATAATATTTATTGGAGTAAATCTAACTTTCTTCCCCCAACATTTTTTAGGATTAGCTGGAATACCTCGACGATACTCGGATTTTCCCGATAGTTACTTAACTTGAAATATTATTTCTTCTTTAGGAAGAACAATTTCATTATTTGCCGTTATTTTCTTTTTATTTATTATTTGAGAAAGTATAATTACTCAACGAACACCTTCTTTC

>Australia-COXI-08

TTCTGATTCTTTGGACACCCAGAAGTTTATATTTTAATTTTACCCGGATTTGGAATAATTTCTCATATTATTACTCAAGAAAGTGGAAAAAAGGAAACATTTGGAACTTTAGGAATAATTTATGCTATATTAACAATTGGATTATTGGGATTTATTGTTTGAGCTCATCATATATTTACAGTAGGTATAGACGTAGATACTCGAGCTTATTTTACTTCAGCAACTATAATTATTGCTGTTCCTACAGGAATTAAAATTTTTAGTTGATTAGCAACTTTACACGGAACTCAATTAACATATAGTCCAGCCCTTCTATGATCATTAGGATTTGTATTTTTATTTACAGTTGGAGGTTTAACAGGAGTAGTATTAGCTAATTCTTCAATTGATATTGTTCTTCATGATACTTATTATGTAGTTGCCCATTTTCATTATGTTTTATCTATAGGAGCTGTATTTGCTATTATAGCAGGATTTATTCATTGATACCCTTTATTAACAGGAATAGTTATAAACCCTTCATGATTAAAGGCTCAATTTAGTATAATATTTATTGGAGTAAATCTAACTTTCTTCCCCCAACATTTTTTAGGATTAGCTGGAATACCTCGACGATACTCGGATTTTCCCGATAGTTACTTAACTTGAAATATTATTTCTTCTTTAGGAAGAACAATTTCATTATTTGCCGTTATTTTCTTTTTATTTATTATTTGAGAAAGTATAATTACTCAACGAACACCTTCTTTC

>Australia-COXI-09

TTCTGATTCTTTGGACACCCAGAAGTTTATATTTTAATTTTACCCGGATTTGGAATAATTTCTCATATTATTACTCAAGAAAGTGGAAAAAAGGAAACATTTGGAACTTTAGGAATAATTTATGCTATATTAACAATTGGATTATTGGGATTTATTGTTTGAGCTCATCATATATTTACAGTAGGTATAGACGTAGATACTCGAGCTTATTTTACTTCAGCAACTATAATTATTGCTGTTCCTACAGGAATTAAAATTTTTAGTTGATTAGCAACTTTACACGGAACTCAATTAACATATAGTCCAGCCCTTCTATGATCATTAGGATTTGTATTTTTATTTACAGTTGGAGGTTTAACAGGAGTAGTATTAGCTAATTCTTCAATTGATATTGTTCTTCATGATACTTATTATGTAGTTGCCCATTTTCATTATGTTTTATCTATAGGAGCTGTATTTGCTATTATAGCAGGATTTATTCATTGATACCCTTTATTAACAGGAATAGTTATAAACCCTTCATGATTAAAGGCTCAATTTAGTATAATATTTATTGGAGTAAATCTAACTTTCTTCCCCCAACATTTTTTAGGATTAGCTGGAATACCTCGACGATACTCGGATTTTCCCGATAGTTACTTAACTTGAAATATTATTTCTTCTTTAGGAAGAACAATTTCATTATTTGCCGTTATTTTCTTTTTATTTATTATTTGAGAAAGTATAATTACTCAACGAACACCTTCTTTC

>Australia-COXI-A01

TTCTGATTCTTTGGACACCCAGAAGTTTATATTTTAATTTTACCCGGATTTGGAATAATTTCTCATATTATTACTCAAGAAAGTGGAAAAAAGGAAACATTTGGAACTTTAGGAATAATTTATGCTATATTAACAATTGGATTATTGGGATTTATTGTTTGAGCTCATCATATATTTACAGTAGGTATAGACGTAGATACTCGAGCTTATTTTACTTCAGCAACTATAATTATTGCTGTTCCTACAGGAATTAAAATTTTTAGTTGATTAGCAACTTTACACGGAACTCAATTAACATATAGTCCAGCCCTTCTATGATCATTAGGATTTGTATTTTTATTTACAGTTGGAGGTTTAACAGGAGTAGTATTAGCTAATTCTTCAATTGATATTGTTCTTCATGATACTTATTATGTAGTTGCCCATTTTCATTATGTTTTATCTATAGGAGCTGTATTTGCTATTATAGCAGGATTTATTCATTGATACCCTTTATTAACAGGAATAGTTATAAACCCTTCATGATTAAAGGCTCAATTTAGTATAATATTTATTGGAGTAAATCTAACTTTCTTCCCCCAACATTTTTTAGGATTAGCTGGAATACCTCGACGATACTCGGATTTTCCCGATAGTTACTTAACTTGAAATATTATTTCTTCTTTAGGAAGAACAATTTCATTATTTGCCGTTATTTTCTTTTTATTTATTATTTGAGAAAGTATAATTACTCAACGAACACCTTCTTTC

>Australia-COXI-A02

TTCTGATTCTTTGGACACCCAGAAGTTTATATTTTAATTTTACCCGGATTTGGAATAATTTCTCATATTATTACTCAAGAAAGTGGAAAAAAGGAAACATTTGGAACTTTAGGAATAATTTATGCTATATTAACAATTGGATTATTGGGATTTATTGTTTGAGCTCATCATATATTTACAGTAGGTATAGACGTAGATACTCGAGCTTATTTTACTTCAGCAACTATAATTATTGCTGTTCCTACAGGAATTAAAATTTTTAGTTGATTAGCAACTTTACACGGAACTCAATTAACATATAGTCCAGCCCTTCTATGATCATTAGGATTTGTATTTTTATTTACAGTTGGAGGTTTAACAGGAGTAGTATTAGCTAATTCTTCAATTGATATTGTTCTTCATGATACTTATTATGTAGTTGCCCATTTTCATTATGTTTTATCTATAGGAGCTGTATTTGCTATTATAGCAGGATTTATTCATTGATACCCTTTATTAACAGGAATAGTTATAAACCCTTCATGATTAAAGGCTCAATTTAGTATAATATTTATTGGAGTAAATCTAACTTTCTTCCCCCAACATTTTTTAGGATTAGCTGGAATACCTCGACGATACTCGGATTTTCCCGATAGTTACTTAACTTGAAATATTATTTCTTCTTTAGGAAGAACAATTTCATTATTTGCCGTTATTTTCTTTTTATTTATTATTTGAGAAAGTATAATTACTCAACGAACACCTTCTTTC

>Australia-COXI-A03

TTCTGATTCTTTGGACACCCAGAAGTTTATATTTTAATTTTACCCGGATTTGGAATAATTTCTCATATTATTACTCAAGAAAGTGGAAAAAAGGAAACATTTGGAACTTTAGGAATAATTTATGCTATATTAACAATTGGATTATTGGGATTTATTGTTTGAGCTCATCATATATTTACAGTAGGTATAGACGTAGATACTCGAGCTTATTTTACTTCAGCAACTATAATTATTGCTGTTCCTACAGGAATTAAAATTTTTAGTTGATTAGCAACTTTACACGGAACTCAATTAACATATAGTCCAGCCCTTCTATGATCATTAGGATTTGTATTTTTATTTACAGTTGGAGGTTTAACAGGAGTAGTATTAGCTAATTCTTCAATTGATATTGTTCTTCATGATACTTATTATGTAGTTGCCCATTTTCATTATGTTTTATCTATAGGAGCTGTATTTGCTATTATAGCAGGATTTATTCATTGATACCCTTTATTAACAGGAATAGTTATAAACCCTTCATGATTAAAAGCTCAATTTAGTATAATATTTATTGGAGTAAATCTAACTTTCTTTCCCCAACATTTTTTAGGATTAGCTGGAATACCTCGACGATACTCAGATTTTCCCGATAGTTACTTAACTTGAAATATTATTTCTTCTTTAGGAAGAACAATTTCATTATTTGCCGTTATTTTCTTTTTATTTATTATTTGAGAAAGTATAATTACTCAACGAACACCGTCTTTC

>Australia-COXI-A04

TTCTGATTCTTTGGACACCCAGAAGTTTATATTTTAATTTTACCCGGATTTGGAATAATTTCTCATATTATTACTCAAGAAAGTGGAAAAAAGGAAACATTTGGAACTTTAGGAATAATTTATGCTATATTAACAATTGGATTATTGGGATTTATTGTTTGAGCTCATCATATATTTACAGTAGGTATAGACGTAGATACTCGAGCTTATTTTACTTCAGCAACTATAATTATTGCTGTTCCTACAGGAATTAAAATTTTTAGTTGATTAGCAACTTTACACGGAACTCAATTAACATATAGTCCAGCCCTTCTATGATCATTAGGATTTGTATTTTTATTTACAGTTGGAGGTTTAACAGGAGTAGTATTAGCTAATTCTTCAATTGATATTGTTCTTCATGATACTTATTATGTAGTTGCCCATTTTCATTATGTTTTATCTATAGGAGCTGTATTTGCTATTATAGCAGGATTTATTCATTGATACCCTTTATTAACAGGAATAGTTATAAACCCTTCATGATTAAAAGCTCAATTTAGTATAATATTTATTGGAGTAAATCTAACTTTCTTTCCCCAACATTTTTTAGGATTAGCTGGAATACCTCGACGATACTCAGATTTTCCCGATAGTTACTTAACTTGAAATATTATTTCTTCTTTAGGAAGAACAATTTCATTATTTGCCGTTATTTTCTTTTTATTTATTATTTGAGAAAGTATAATTACTCAACGAACACCGTCTTTC

>Australia-COXI-A05

TTCTGATTCTTTGGACACCCAGAAGTTTATATTTTAATTTTACCCGGATTTGGAATAATTTCTCATATTATTACTCAAGAAAGTGGAAAAAAGGAAACATTTGGAACTTTAGGAATAATTTATGCTATATTAACAATTGGATTATTGGGATTTATTGTTTGAGCTCATCATATATTTACAGTAGGTATAGACGTAGATACTCGAGCTTATTTTACTTCAGCAACTATAATTATTGCTGTTCCTACAGGAATTAAAATTTTTAGTTGATTAGCAACTTTACACGGAACTCAATTAACATATAGTCCAGCCCTTCTATGATCATTAGGATTTGTATTTTTATTTACAGTTGGAGGTTTAACAGGAGTAGTATTAGCTAATTCTTCAATTGATATTGTTCTTCATGATACTTATTATGTAGTTGCCCATTTTCATTATGTTTTATCTATAGGAGCTGTATTTGCTATTATAGCAGGATTTATTCATTGATACCCTTTATTAACAGGAATAGTTATAAACCCTTCATGATTAAAAGCTCAATTTAGTATAATATTTATTGGAGTAAATCTAACTTTCTTTCCCCAACATTTTTTAGGATTAGCTGGAATACCTCGACGATACTCAGATTTTCCCGATAGTTACTTAACTTGAAATATTATTTCTTCTTTAGGAAGAACAATTTCATTATTTGCCGTTATTTTCTTTTTATTTATTATTTGAGAAAGTATAATTACTCAACGAACACCGTCTTTC

>Australia-COXI-A06

TTCTGATTCTTTGGACACCCAGAAGTTTATATTTTAATTTTACCCGGATTTGGAATAATTTCTCATATTATTACTCAAGAAAGTGGAAAAAAGGAAACATTTGGAACTTTAGGAATAATTTATGCTATATTAACAATTGGATTATTGGGATTTATTGTTTGAGCTCATCATATATTTACAGTAGGTATAGACGTAGATACTCGAGCTTATTTTACTTCAGCAACTATAATTATTGCTGTTCCTACAGGAATTAAAATTTTTAGTTGATTAGCAACTTTACACGGAACTCAATTAACATATAGTCCAGCCCTTCTATGATCATTAGGATTTGTATTTTTATTTACAGTTGGAGGTTTAACAGGAGTAGTATTAGCTAATTCTTCAATTGATATTGTTCTTCATGATACTTATTATGTAGTTGCCCATTTTCATTATGTTTTATCTATAGGAGCTGTATTTGCTATTATAGCAGGATTTATTCATTGATACCCTTTATTAACAGGAATAGTTATAAACCCTTCATGATTAAAGGCTCAATTTAGTATAATATTTATTGGAGTAAATCTAACTTTCTTCCCCCAACATTTTTTAGGATTAGCTGGAATACCTCGACGATACTCGGATTTTCCCGATAGTTACTTAACTTGAAATATTATTTCTTCTTTAGGAAGAACAATTTCATTATTTGCCGTTATTTTCTTTTTATTTATTATTTGAGAAAGTATAATTACTCAACGAACACCTTCTTTC

>Australia-COXI-A07

TTCTGATTCTTTGGACACCCAGAAGTTTATATTTTAATTTTACCCGGATTTGGAATAATTTCTCATATTATTACTCAAGAAAGTGGAAAAAAGGAAACATTTGGAACTTTAGGAATAATTTATGCTATATTAACAATTGGATTATTGGGATTTATTGTTTGAGCTCATCATATATTTACAGTAGGTATAGACGTAGATACTCGAGCTTATTTTACTTCAGCAACTATAATTATTGCTGTTCCTACAGGAATTAAAATTTTTAGTTGATTAGCAACTTTACACGGAACTCAATTAACATATAGTCCAGCCCTTCTATGATCATTAGGATTTGTATTTTTATTTACAGTTGGAGGTTTAACAGGAGTAGTATTAGCTAATTCTTCAATTGATATTGTTCTTCATGATACTTATTATGTAGTTGCCCATTTTCATTATGTTTTATCTATAGGAGCTGTATTTGCTATTATAGCAGGATTTATTCATTGATACCCTTTATTAACAGGAATAGTTATAAACCCTTCATGATTAAAGGCTCAATTTAGTATAATATTTATTGGAGTAAATCTAACTTTCTTTCCCCAACATTTTTTAGGATTAGCTGGAATACCTCGACGATACTCAGATTTTCCCGATAGTTACTTAACTTGAAATATTATTTCTTCTTTAGGAAGAACAATTTCATTATTTGCCGTTATTTTCTTTTTATTTATTATTTGAGAAAGTATAATTACTCAACGAACACCKTCTTTC

>Australia-COXI-A08

TTYTGATTCTTTGGACACCCAGAAGTTTATATTTTAATTTTACCCGGATTTGGAATAATTTCTCATATTATTACTCAAGAAAGTGGAAAAAAGGAAACATTTGGAACTTTAGGAATAATTTATGCTATATTAACAATTGGATTATTGGGATTTATTGTTTGAGCTCATCATATATTTACAGTAGGTATAGATGTAGATACTCGAGCTTATTTTACTTCAGCAACTATAATTATTGCTGTTCCTACAGGAATTAAAATTTTTAGTTGATTAGCAACTTTACACGGAACTCAATTAACATATAGTCCAGCCCTTCTATGATCATTAGGATTTGTATTTTTATTTACAGTTGGAGGTTTAACAGGAGTAGTATTAGCTAATTCTTCAATTGATATTGTTCTTCATGATACTTATTACGTAGTTGCCCATTTTCATTACGTTTTATCTATAGGAGCTGTATTTGCTATTATAGCAGGATTTATTCATTGATACCCTTTATTAACAGGAATAGTTATAAACCCTTCATGATTAAAGGCTCAATTTAGTATAATATTTATTGGAGTAAATCTAACTTTCTTTCCTCAACATTTTTTAGGRTTAGCTGGAATACCTCGACGATACTCAGATTTTCCTGATAGTTACTTAACTTGAAATATTATTTCTTCTTTAGGAAGAACAATTTCACTATTTGCCGTTATTTTCTTTTTATTTATTATTTGAGAAAGTATAATTACTCAACGAACACCTTCTTTC

>Australia-COXI-A09

TTCTGATTCTTTGGACACCCAGAAGTTTATATTTTAATTTTACCCGGATTTGGAATAATTTCTCATATTATTACTCAAGAAAGTGGAAAAAAGGAAACATTTGGAACTTTAGGAATAATTTATGCTATATTAACAATTGGATTATTGGGATTTATTGTTTGAGCTCATCATATATTTACAGTAGGTATAGACGTAGATACTCGAGCTTATTTTACTTCAGCAACTATAATTATTGCTGTTCCTACAGGAATTAAAATTTTTAGTTGATTAGCAACTTTACACGGAACTCAATTAACATATAGTCCAGCCCTTCTATGATCATTAGGATTTGTATTTTTATTTACAGTTGGAGGTTTAACAGGAGTAGTATTAGCTAATTCTTCAATTGATATTGTTCTTCATGATACTTATTATGTAGTTGCCCATTTTCATTATGTTTTATCTATAGGAGCTGTATTTGCTATTATAGCAGGATTTATTCATTGATACCCTTTATTAACAGGAATAGTTATAAACCCTTCATGATTAAAGGCTCAATTTAGTATAATATTTATTGGAGTAAATCTAACTTTCTTCCCCCAACATTTTTTAGGATTAGCTGGAATACCTCGACGATACTCGGATTTTCCCGATAGTTACTTAACTTGAAATATTATTTCTTCTTTAGGAAGAACAATTTCATTATTTGCCGTTATTTTCTTTTTATTTATTATTTGAGAAAGTATAATTACTCAACGAACACCTTCTTTC

>Australia-COXI-A10

TTCTGATTCTTTGGACACCCAGAAGTTTATATTTTAATTTTACCCGGATTTGGAATAATTTCTCATATTATTACTCAAGAAAGTGGAAAAAAGGAAACATTTGGAACTTTAGGAATAATTTATGCTATATTAACAATTGGATTATTGGGATTTATTGTTTGAGCTCATCATATATTTACAGTAGGTATAGACGTAGATACTCGAGCTTATTTTACTTCAGCAACTATAATTATTGCTGTTCCTACAGGAATTAAAATTTTTAGTTGATTAGCAACTTTACACGGAACTCAATTAACATATAGTCCAGCCCTTCTATGATCATTAGGATTTGTATTTTTATTTACAGTTGGAGGTTTAACAGGAGTAGTATTAGCTAATTCTTCAATTGATATTGTTCTTCATGATACTTATTATGTAGTTGCCCATTTTCATTATGTTTTATCTATAGGAGCTGTATTTGCTATTATAGCAGGATTTATTCATTGATACCCTTTATTAACAGGAATAGTTATAAACCCTTCATGATTAAAGGCTCAATTTAGTATAATATTTATTGGAGTAAATCTAACTTTCTTCCCCCAACATTTTTTAGGATTAGCTGGAATACCTCGACGATACTCGGATTTTCCCGATAGTTACTTAACTTGAAATATTATTTCTTCTTTAGGAAGAACAATTTCATTATTTGCCGTTATTTTCTTTTTATTTATTATTTGAGAAAGTATAATTACTCAACGAACACCTTCTTTC

>Australia-COXI-A11

TTCTGATTCTTTGGACACCCAGAAGTTTATATTTTAATTTTACCCGGATTTGGAATAATTTCTCATATTATTACTCAAGAAAGTGGAAAAAAGGAAACATTTGGAACTTTAGGAATAATTTATGCTATATTAACAATTGGATTATTGGGATTTATTGTTTGAGCTCATCATATATTTACAGTAGGTATAGACGTAGATACTCGAGCTTATTTTACTTCAGCAACTATAATTATTGCTGTTCCTACAGGAATTAAAATTTTTAGTTGATTAGCAACTTTACACGGAACTCAATTAACATATAGTCCAGCCCTTCTATGATCATTAGGATTTGTATTTTTATTTACAGTTGGAGGTTTAACAGGAGTAGTATTAGCTAATTCTTCAATTGATATTGTTCTTCATGATACTTATTATGTAGTTGCCCATTTTCATTATGTTTTATCTATAGGAGCTGTATTTGCTATTATAGCAGGATTTATTCATTGATACCCTTTATTAACAGGAATAGTTATAAACCCTTCATGATTAAAGGCTCAATTTAGTATAATATTTATTGGAGTAAATCTAACTTTCTTTCCCCAACATTTTTTAGGATTAGCTGGAATACCTCGACGATACTCAGATTTTCCCGATAGTTACTTAACTTGAAATATTATTTCTTCTTTAGGAAGAACAATTTCATTATTTGCCGTTATTTTCTTTTTATTTATTATTTGAGAAAGTATAATTACTCAACGAACACCTTCTTTC

>Australia-COXI-B02

TTCTGATTCTTTGGACACCCAGAAGTTTATATTTTAATTTTACCCGGATTTGGAATAATTTCTCATATTATTACTCAAGAAAGTGGAAAAAAGGAAACATTTGGAACTTTAGGAATAATTTATGCTATATTAACAATTGGATTATTGGGATTTATTGTTTGAGCTCATCATATATTTACAGTAGGTATAGACGTAGATACTCGAGCTTATTTTACTTCAGCAACTATAATTATTGCTGTTCCTACAGGAATTAAAATTTTTAGTTGATTAGCAACTTTACACGGAACTCAATTAACATATAGTCCAGCCCTTCTATGATCATTAGGATTTGTATTTTTATTTACAGTTGGAGGTTTAACAGGAGTAGTATTAGCTAATTCTTCAATTGATATTGTTCTTCATGATACTTATTATGTAGTTGCCCATTTTCATTATGTTTTATCTATAGGAGCTGTATTTGCTATTATAGCAGGATTTATTCATTGATACCCTTTATTAACAGGAATAGTTATAAACCCTTCATGATTAAAGGCTCAATTTAGTATAATATTTATTGGAGTAAATCTAACTTTCTTCCCCCAACATTTTTTAGGATTAGCTGGAATACCTCGACGATACTCGGATTTTCCCGATAGTTACTTAACTTGAAATATTATTTCTTCTTTAGGAAGAACAATTTCATTATTTGCCGTTATTTTCTTTTTATTTATTATTTGAGAAAGTATAATTACTCAACGAACACCTTCTTTC

>Australia-COXI-B03

TTCTGATTCTTTGGACACCCAGAAGTTTATATTTTAATTTTACCCGGATTTGGAATAATTTCTCATATTATTACTCAAGAAAGTGGAAAAAAGGAAACATTTGGAACTTTAGGAATAATTTATGCTATATTAACAATTGGATTATTGGGATTTATTGTTTGAGCTCATCATATATTTACAGTAGGTATAGACGTAGATACTCGAGCTTATTTTACTTCAGCAACTATAATTATTGCTGTTCCTACAGGAATTAAAATTTTTAGTTGATTAGCAACTTTACACGGAACTCAATTAACATATAGTCCAGCCCTTCTATGATCATTAGGATTTGTATTTTTATTTACAGTTGGAGGTTTAACAGGAGTAGTATTAGCTAATTCTTCAATTGATATTGTTCTTCATGATACTTATTATGTAGTTGCCCATTTTCATTATGTTTTATCTATAGGAGCTGTATTTGCTATTATAGCAGGATTTATTCATTGATACCCTTTATTAACAGGAATAGTTATAAACCCTTCATGATTAAAGGCTCAATTTAGTATAATATTTATTGGAGTAAATCTAACTTTCTTCCCCCAACATTTTTTAGGATTAGCTGGAATACCTCGACGATACTCGGATTTTCCCGATAGTTACTTAACTTGAAATATTATTTCTTCTTTAGGAAGAACAATTTCATTATTTGCCGTTATTTTCTTTTTATTTATTATTTGAGAAAGTATAATTACTCAACGAACACCTTCTTTC

>Australia-COXI-B04

TTCTGATTCTTTGGACACCCAGAAGTTTATATTTTAATTTTACCCGGATTTGGAATAATTTCTCATATTATTACTCAAGAAAGTGGAAAAAAGGAAACATTTGGAACTTTAGGAATAATTTATGCTATATTAACAATTGGATTATTGGGATTTATTGTTTGAGCTCATCATATATTTACAGTAGGTATAGACGTAGATACTCGAGCTTATTTTACTTCAGCAACTATAATTATTGCTGTTCCTACAGGAATTAAAATTTTTAGTTGATTAGCAACTTTACACGGAACTCAATTAACATATAGTCCAGCCCTTCTATGATCATTAGGATTTGTATTTTTATTTACAGTTGGAGGTTTAACAGGAGTAGTATTAGCTAATTCTTCAATTGATATTGTTCTTCATGATACTTATTATGTAGTTGCCCATTTTCATTATGTTTTATCTATAGGAGCTGTATTTGCTATTATAGCAGGATTTATTCATTGATACCCTTTATTAACAGGAATAGTTATAAACCCTTCATGATTAAAGGCTCAATTTAGTATAATATTTATTGGAGTAAATCTAACTTTCTTCCCCCAACATTTTTTAGGATTAGCTGGAATACCTCGACGATACTCGGATTTTCCCGATAGTTACTTAACTTGAAATATTATTTCTTCTTTAGGAAGAACAATTTCATTATTTGCCGTTATTTTCTTTTTATTTATTATTTGAGAAAGTATAATTACTCAACGAACACCTTCTTTC

>Australia-COXI-B05

TTCTGATTCTTTGGACACCCAGAAGTTTATATTTTAATTTTACCCGGATTTGGAATAATTTCTCATATTATTACTCAAGAAAGTGGAAAAAAGGAAACATTTGGAACTTTAGGAATAATTTATGCTATATTAACAATTGGATTATTGGGATTTATTGTTTGAGCTCATCATATATTTACAGTAGGTATAGACGTAGATACTCGAGCTTATTTTACTTCAGCAACTATAATTATTGCTGTTCCTACAGGAATTAAAATTTTTAGTTGATTAGCAACTTTACACGGAACTCAATTAACATATAGTCCAGCCCTTCTATGATCATTAGGATTTGTATTTTTATTTACAGTTGGAGGTTTAACAGGAGTAGTATTAGCTAATTCTTCAATTGATATTGTTCTTCATGATACTTATTATGTAGTTGCCCATTTTCATTATGTTTTATCTATAGGAGCTGTATTTGCTATTATAGCAGGATTTATTCATTGATACCCTTTATTAACAGGAATAGTTATAAACCCTTCATGATTAAAAGCTCAATTTAGTATAATATTTATTGGAGTAAATCTAACTTTCTTTCCCCAACATTTTTTAGGATTAGCTGGAATACCTCGACGATACTCAGATTTTCCCGATAGTTACTTAACTTGAAATATTATTTCTTCTTTAGGAAGAACAATTTCATTATTTGCCGTTATTTTCTTTTTATTTATTATTTGAGAAAGTATAATTACTCAACGAACACCGTCTTTC

>Australia-COXI-B06

TTCTGATTCTTTGGACACCCAGAAGTTTATATTTTAATTTTACCCGGATTTGGAATAATTTCTCATATTATTACTCAAGAAAGTGGAAAAAAGGAAACATTTGGAACTTTAGGAATAATTTATGCTATATTAACAATTGGATTATTGGGATTTATTGTTTGAGCTCATCATATATTTACAGTAGGTATAGACGTAGATACTCGAGCTTATTTTACTTCAGCAACTATAATTATTGCTGTTCCTACAGGAATTAAAATTTTTAGTTGATTAGCAACTTTACACGGAACTCAATTAACATATAGTCCAGCCCTTCTATGATCATTAGGATTTGTATTTTTATTTACAGTTGGAGGTTTAACAGGAGTAGTATTAGCTAATTCTTCAATTGATATTGTTCTTCATGATACTTATTATGTAGTTGCCCATTTTCATTATGTTTTATCTATAGGAGCTGTATTTGCTATTATAGCAGGATTTATTCATTGATACCCTTTATTAACAGGAATAGTTATAAACCCTTCATGATTAAAGGCTCAATTTAGTATAATATTTATTGGAGTAAATCTAACTTTCTTCCCCCAACATTTTTTAGGATTAGCTGGAATACCTCGACGATACTCGGATTTTCCCGATAGTTACTTAACTTGAAATATTATTTCTTCTTTAGGAAGAACAATTTCATTATTTGCCGTTATTTTCTTTTTATTTATTATTTGAGAAAGTATAATTACTCAACGAACACCTTCTTTC

>Australia-COXI-B07

TTCTGATTCTTTGGACACCCAGAAGTTTATATTTTAATTTTACCCGGATTTGGAATAATTTCTCATATTATTACTCAAGAAAGTGGAAAAAAGGAAACATTTGGAACTTTAGGAATAATTTATGCTATATTAACAATTGGATTATTGGGATTTATTGTTTGAGCTCATCATATATTTACAGTAGGTATAGACGTAGATACTCGAGCTTATTTTACTTCAGCAACTATAATTATTGCTGTTCCTACAGGAATTAAAATTTTTAGTTGATTAGCAACTTTACACGGAACTCAATTAACATATAGTCCAGCCCTTCTATGATCATTAGGATTTGTATTTTTATTTACAGTTGGAGGTTTAACAGGAGTAGTATTAGCTAATTCTTCAATTGATATTGTTCTTCATGATACTTATTATGTAGTTGCCCATTTTCATTATGTTTTATCTATAGGAGCTGTATTTGCTATTATAGCAGGATTTATTCATTGATACCCTTTATTAACAGGAATAGTTATAAACCCTTCATGATTAAAGGCTCAATTTAGTATAATATTTATTGGAGTAAATCTAACTTTCTTCCCCCAACATTTTTTAGGATTAGCTGGAATACCTCGACGATACTCGGATTTTCCCGATAGTTACTTAACTTGAAATATTATTTCTTCTTTAGGAAGAACAATTTCATTATTTGCCGTTATTTTCTTTTTATTTATTATTTGAGAAAGTATAATTACTCAACGAACACCTTCTTTC

>Australia-COXI-B08

TTCTGATTCTTTGGACACCCAGAAGTTTATATTTTAATTTTACCCGGATTTGGAATAATTTCTCATATTATTACTCAAGAAAGTGGAAAAAAGGAAACATTTGGAACTTTAGGAATAATTTATGCTATATTAACAATTGGATTATTGGGATTTATTGTTTGAGCTCATCATATATTTACAGTAGGTATAGACGTAGATACTCGAGCTTATTTTACTTCAGCAACTATAATTATTGCTGTTCCTACAGGAATTAAAATTTTTAGTTGATTAGCAACTTTACACGGAACTCAATTAACATATAGTCCAGCCCTTCTATGATCATTAGGATTTGTATTTTTATTTACAGTTGGAGGTTTAACAGGAGTAGTATTAGCTAATTCTTCAATTGATATTGTTCTTCATGATACTTATTATGTAGTTGCCCATTTTCATTATGTTTTATCTATAGGAGCTGTATTTGCTATTATAGCAGGATTTATTCATTGATACCCTTTATTAACAGGAATAGTTATAAACCCTTCATGATTAAAGGCTCAATTTAGTATAATATTTATTGGAGTAAATCTAACTTTCTTCCCCCAACATTTTTTAGGATTAGCTGGAATACCTCGACGATACTCGGATTTTCCCGATAGTTACTTAACTTGAAATATTATTTCTTCTTTAGGAAGAACAATTTCATTATTTGCCGTTATTTTCTTTTTATTTATTATTTGAGAAAGTATAATTACTCAACGAACACCTTCTTTC

>Australia-COXI-B09

TTCTGATTCTTTGGACACCCAGAAGTTTATATTTTAATTTTACCCGGATTTGGAATAATTTCTCATATTATTACTCAAGAAAGTGGAAAAAAGGAAACATTTGGAACTTTAGGAATAATTTATGCTATATTAACAATTGGATTATTGGGATTTATTGTTTGAGCTCATCATATATTTACAGTAGGTATAGACGTAGATACTCGAGCTTATTTTACTTCAGCAACTATAATTATTGCTGTTCCTACAGGAATTAAAATTTTTAGTTGATTAGCAACTTTACACGGAACTCAATTAACATATAGTCCAGCCCTTCTATGATCATTAGGATTTGTATTTTTATTTACAGTTGGAGGTTTAACAGGAGTAGTATTAGCTAATTCTTCAATTGATATTGTTCTTCATGATACTTATTATGTAGTTGCCCATTTTCATTATGTTTTATCTATAGGAGCTGTATTTGCTATTATAGCAGGATTTATTCATTGATACCCTTTATTAACAGGAATAGTTATAAACCCTTCATGATTAAAGGCTCAATTTAGTATAATATTTATTGGAGTAAATCTAACTTTCTTCCCCCAACATTTTTTAGGATTAGCTGGAATACCTCGACGATACTCGGATTTTCCCGATAGTTACTTAACTTGAAATATTATTTCTTCTTTAGGAAGAACAATTTCATTATTTGCCGTTATTTTCTTTTTATTTATTATTTGAGAAAGTATAATTACTCAACGAACACCTTCTTTC

>Australia-COXI-B10

TTCTGATTCTTTGGACACCCAGAAGTTTATATTTTAATTTTACCCGGATTTGGAATAATTTCTCATATTATTACTCAAGAAAGTGGAAAAAAGGAAACATTTGGAACTTTAGGAATAATTTATGCTATATTAACAATTGGATTATTGGGATTTATTGTTTGAGCTCATCATATATTTACAGTAGGTATAGACGTAGATACTCGAGCTTATTTTACTTCAGCAACTATAATTATTGCTGTTCCTACAGGAATTAAAATTTTTAGTTGATTAGCAACTTTACACGGAACTCAATTAACATATAGTCCAGCCCTTCTATGATCATTAGGATTTGTATTTTTATTTACAGTTGGAGGTTTAACAGGAGTAGTATTAGCTAATTCTTCAATTGATATTGTTCTTCATGATACTTATTATGTAGTTGCCCATTTTCATTATGTTTTATCTATAGGAGCTGTATTTGCTATTATAGCAGGATTTATTCATTGATACCCTTTATTAACAGGAATAGTTATAAACCCTTCATGATTAAAGGCTCAATTTAGTATAATATTTATTGGAGTAAATCTAACTTTCTTTCCCCAACATTTTTTAGGATTAGCTGGAATACCTCGACGATACTCAGATTTTCCCGATAGTTACTTAACTTGAAATATTATTTCTTCTTTAGGAAGAACAATTTCATTATTTGCCGTTATTTTCTTTTTATTTATTATTTGAGAAAGTATAATTACTCAACGAACACCGTCTTTC

>Australia-COXI-B11

TTCTGATTCTTTGGACACCCAGAAGTTTATATTTTAATTTTACCCGGATTTGGAATAATTTCTCATATTATTACTCAAGAAAGTGGAAAAAAGGAAACATTTGGAACTTTAGGAATAATTTATGCTATATTAACAATTGGATTATTGGGATTTATTGTTTGAGCTCATCATATATTTACAGTAGGTATAGACGTAGATACTCGAGCTTATTTTACTTCAGCAACTATAATTATTGCTGTTCCTACAGGAATTAAAATTTTTAGTTGATTAGCAACTTTACACGGAACTCAATTAACATATAGTCCAGCCCTTCTATGATCATTAGGATTTGTATTTTTATTTACAGTTGGAGGTTTAACAGGAGTAGTATTAGCTAATTCTTCAATTGATATTGTTCTTCATGATACTTATTATGTAGTTGCCCATTTTCATTATGTTTTATCTATAGGAGCTGTATTTGCTATTATAGCAGGATTTATTCATTGATACCCTTTATTAACAGGAATAGTTATAAACCCTTCATGATTAAAGGCTCAATTTAGTATAATATTTATTGGAGTAAATCTAACTTTCTTCCCCCAACATTTTTTAGGATTAGCTGGAATACCTCGACGATACTCGGATTTTCCCGATAGTTACTTAACTTGAAATATTATTTCTTCTTTAGGAAGAACAATTTCATTATTTGCCGTTATTTTCTTTTTATTTATTATTTGAGAAAGTATAATTACTCAACGAACACCTTCTTTC

>Australia-COXI-B12

TTCTGATTCTTTGGACACCCAGAAGTTTATATTTTAATTTTACCCGGATTTGGAATAATTTCTCATATTATTACTCAAGAAAGTGGAAAAAAGGAAACATTTGGAACTTTAGGAATAATTTATGCTATATTAACAATTGGATTATTGGGATTTATTGTTTGAGCTCATCATATATTTACAGTAGGTATAGACGTAGATACTCGAGCTTATTTTACTTCAGCAACTATAATTATTGCTGTTCCTACAGGAATTAAAATTTTTAGTTGATTAGCAACTTTACACGGAACTCAATTAACATATAGTCCAGCCCTTCTATGATCATTAGGATTTGTATTTTTATTTACAGTTGGAGGTTTAACAGGAGTAGTATTAGCTAATTCTTCAATTGATATTGTTCTTCATGATACTTATTATGTAGTTGCCCATTTTCATTATGTTTTATCTATAGGAGCTGTATTTGCTATTATAGCAGGATTTATTCATTGATACCCTTTATTAACAGGAATAGTTATAAACCCTTCATGATTAAAGGCTCAATTTAGTATAATATTTATTGGAGTAAATCTAACTTTCTTTCCCCAACATTTTTTAGGATTAGCTGGAATACCTCGACGATACTCAGATTTTCCCGATAGTTACTTAACTTGAAATATTATTTCTTCTTTAGGAAGAACAATTTCATTATTTGCCGTTATTTTCTTTTTATTTATTATTTGAGAAAGTATAATTACTCAACGAACACCGTCTTTC

>Australia-COXI-C01

TTCTGATTCTTTGGACACCCAGAAGTTTATATTTTAATTTTACCCGGATTTGGAATAATTTCTCATATTATTACTCAAGAAAGTGGAAAAAAGGAAACATTTGGAACTTTAGGAATAATTTATGCTATATTAACAATTGGATTATTGGGATTTATTGTTTGAGCTCATCATATATTTACAGTAGGTATAGACGTAGATACTCGAGCTTATTTTACTTCAGCAACTATAATTATTGCTGTTCCTACAGGAATTAAAATTTTTAGTTGATTAGCAACTTTACACGGAACTCAATTAACATATAGTCCAGCCCTTCTATGATCATTAGGATTTGTATTTTTATTTACAGTTGGAGGTTTAACAGGAGTAGTATTAGCTAATTCTTCAATTGATATTGTTCTTCATGATACTTATTATGTAGTTGCCCATTTTCATTATGTTTTATCTATAGGAGCTGTATTTGCTATTATAGCAGGATTTATTCATTGATACCCTTTATTAACAGGAATAGTTATAAACCCTTCATGATTAAAGGCTCAATTTAGTATAATATTTATTGGAGTAAATCTAACTTTCTTCCCCCAACATTTTTTAGGATTAGCTGGAATACCTCGACGATACTCGGATTTTCCCGATAGTTACTTAACTTGAAATATTATTTCTTCTTTAGGAAGAACAATTTCATTATTTGCCGTTATTTTCTTTTTATTTATTATTTGAGAAAGTATAATTACTCAACGAACACCTTCTTTC

>Australia-COXI-C02

TTCTGATTCTTTGGACACCCAGAAGTTTATATTTTAATTTTACCCGGATTTGGAATAATTTCTCATATTATTACTCAAGAAAGTGGAAAAAAGGAAACATTTGGAACTTTAGGAATAATTTATGCTATATTAACAATTGGATTATTGGGATTTATTGTTTGAGCTCATCATATATTTACAGTAGGTATAGACGTAGATACTCGAGCTTATTTTACTTCAGCAACTATAATTATTGCTGTTCCTACAGGAATTAAAATTTTTAGTTGATTAGCAACTTTACACGGAACTCAATTAACATATAGTCCAGCCCTTCTATGATCATTAGGATTTGTATTTTTATTTACAGTTGGAGGTTTAACAGGAGTAGTATTAGCTAATTCTTCAATTGATATTGTTCTTCATGATACTTATTATGTAGTTGCCCATTTTCATTATGTTTTATCTATAGGAGCTGTATTTGCTATTATAGCAGGATTTATTCATTGATACCCTTTATTAACAGGAATAGTTATAAACCCTTCATGATTAAAAGCTCAATTTAGTATAATATTTATTGGAGTAAATCTAACTTTCTTTCCCCAACATTTTTTAGGATTAGCTGGAATACCTCGACGATACTCAGATTTTCCCGATAGTTACTTAACTTGAAATATTATTTCTTCTTTAGGAAGAACAATTTCATTATTTGCCGTTATTTTCTTTTTATTTATTATTTGAGAAAGTATAATTACTCAACGAACACCGTCTTTC

>Australia-COXI-C03

TTCTGATTCTTTGGACACCCAGAAGTTTATATTTTAATTTTACCCGGATTTGGAATAATTTCTCATATTATTACTCAAGAAAGTGGAAAAAAGGAAACATTTGGAACTTTAGGAATAATTTATGCTATATTAACAATTGGATTATTGGGATTTATTGTTTGAGCTCATCATATATTTACAGTAGGTATAGACGTAGATACTCGAGCTTATTTTACTTCAGCAACTATAATTATTGCTGTTCCTACAGGAATTAAAATTTTTAGTTGATTAGCAACTTTACACGGAACTCAATTAACATATAGTCCAGCCCTTCTATGATCATTAGGATTTGTATTTTTATTTACAGTTGGAGGTTTAACAGGAGTAGTATTAGCTAATTCTTCAATTGATATTGTTCTTCATGATACTTATTATGTAGTTGCCCATTTTCATTATGTTTTATCTATAGGAGCTGTATTTGCTATTATAGCAGGATTTATTCATTGATACCCTTTATTAACAGGAATAGTTATAAACCCTTCATGATTAAAGGCTCAATTTAGTATAATATTTATTGGAGTAAATCTAACTTTCTTCCCCCAACATTTTTTAGGATTAGCTGGAATACCTCGACGATACTCGGATTTTCCCGATAGTTACTTAACTTGAAATATTATTTCTTCTTTAGGAAGAACAATTTCATTATTTGCCGTTATTTTCTTTTTATTTATTATTTGAGAAAGTATAATTACTCAACGAACACCTTCTTTC

>Australia-COXI-C04

TTCTGATTCTTTGGACACCCAGAAGTTTATATTTTAATTTTACCCGGATTTGGAATAATTTCTCATATTATTACTCAAGAAAGTGGAAAAAAGGAAACATTTGGAACTTTAGGAATAATTTATGCTATATTAACAATTGGATTATTGGGATTTATTGTTTGAGCTCATCATATATTTACAGTAGGTATAGACGTAGATACTCGAGCTTATTTTACTTCAGCAACTATAATTATTGCTGTTCCTACAGGAATTAAAATTTTTAGTTGATTAGCAACTTTACACGGAACTCAATTAACATATAGTCCAGCCCTTCTATGATCATTAGGATTTGTATTTTTATTTACAGTTGGAGGTTTAACAGGAGTAGTATTAGCTAATTCTTCAATTGATATTGTTCTTCATGATACTTATTATGTAGTTGCCCATTTTCATTATGTTTTATCTATAGGAGCTGTATTTGCTATTATAGCAGGATTTATTCATTGATACCCTTTATTAACAGGAATAGTTATAAACCCTTCATGATTAAAGGCTCAATTTAGTATAATATTTATTGGAGTAAATCTAACTTTCTTCCCCCAACATTTTTTAGGATTAGCTGGAATACCTCGACGATACTCGGATTTTCCCGATAGTTACTTAACTTGAAATATTATTTCTTCTTTAGGAAGAACAATTTCATTATTTGCCGTTATTTTCTTTTTATTTATTATTTGAGAAAGTATAATTACTCAACGAACACCTTCTTTC

>Australia-COXI-C05

TTCTGATTCTTTGGACACCCAGAAGTTTATATTTTAATTTTACCCGGATTTGGAATAATTTCTCATATTATTACTCAAGAAAGTGGAAAAAAGGAAACATTTGGAACTTTAGGAATAATTTATGCTATATTAACAATTGGATTATTGGGATTTATTGTTTGAGCTCATCATATATTTACAGTAGGTATAGACGTAGATACTCGAGCTTATTTTACTTCAGCAACTATAATTATTGCTGTTCCTACAGGAATTAAAATTTTTAGTTGATTAGCAACTTTACACGGAACTCAATTAACATATAGTCCAGCCCTTCTATGATCATTAGGATTTGTATTTTTATTTACAGTTGGAGGTTTAACAGGAGTAGTATTAGCTAATTCTTCAATTGATATTGTTCTTCATGATACTTATTATGTAGTTGCCCATTTTCATTATGTTTTATCTATAGGAGCTGTATTTGCTATTATAGCAGGATTTATTCATTGATACCCTTTATTAACAGGAATAGTTATAAACCCTTCATGATTAAAGGCTCAATTTAGTATAATATTTATTGGAGTAAATCTAACTTTCTTCCCCCAACATTTTTTAGGATTAGCTGGAATACCTCGACGATACTCGGATTTTCCCGATAGTTACTTAACTTGAAATATTATTTCTTCTTTAGGAAGAACAATTTCATTATTTGCCGTTATTTTCTTTTTATTTATTATTTGAGAAAGTATAATTACTCAACGAACACCTTCTTTC

>Australia-COXI-C06

TTCTGATTCTTTGGACACCCAGAAGTTTATATTTTAATTTTACCCGGATTTGGAATAATTTCTCATATTATTACTCAAGAAAGTGGAAAAAAGGAAACATTTGGAACTTTAGGAATAATTTATGCTATATTAACAATTGGATTATTGGGATTTATTGTTTGAGCTCATCATATATTTACAGTAGGTATAGACGTAGATACTCGAGCTTATTTTACTTCAGCAACTATAATTATTGCTGTTCCTACAGGAATTAAAATTTTTAGTTGATTAGCAACTTTACACGGAACTCAATTAACATATAGTCCAGCCCTTCTATGATCATTAGGATTTGTATTTTTATTTACAGTTGGAGGTTTAACAGGAGTAGTATTAGCTAATTCTTCAATTGATATTGTTCTTCATGATACTTATTATGTAGTTGCCCATTTTCATTATGTTTTATCTATAGGAGCTGTATTTGCTATTATAGCAGGATTTATTCATTGATACCCTTTATTAACAGGAATAGTTATAAACCCTTCATGATTAAAAGCTCAATTTAGTATAATATTTATTGGAGTAAATCTAACTTTCTTTCCCCAACATTTTTTAGGATTAGCTGGAATACCTCGACGATACTCAGATTTTCCCGATAGTTACTTAACTTGAAATATTATTTCTTCTTTAGGAAGAACAATTTCATTATTTGCCGTTATTTTCTTTTTATTTATTATTTGAGAAAGTATAATTACTCAACGAACACCGTCTTTC

>Australia-COXI-C07

TTCTGATTCTTTGGACACCCAGAAGTTTATATTTTAATTTTACCCGGATTTGGAATAATTTCTCATATTATTACTCAAGAAAGTGGAAAAAAGGAAACATTTGGAACTTTAGGAATAATTTATGCTATATTAACAATTGGATTATTGGGATTTATTGTTTGAGCTCATCATATATTTACAGTAGGTATAGACGTAGATACTCGAGCTTATTTTACTTCAGCAACTATAATTATTGCTGTTCCTACAGGAATTAAAATTTTTAGTTGATTAGCAACTTTACACGGAACTCAATTAACATATAGTCCAGCCCTTCTATGATCATTAGGATTTGTATTTTTATTTACAGTTGGAGGTTTAACAGGAGTAGTATTAGCTAATTCTTCAATTGATATTGTTCTTCATGATACTTATTATGTAGTTGCCCATTTTCATTATGTTTTATCTATAGGAGCTGTATTTGCTATTATAGCAGGATTTATTCATTGATACCCTTTATTAACAGGAATAGTTATAAACCCTTCATGATTAAAGGCTCAATTTAGTATAATATTTATTGGAGTAAATCTAACTTTCTTCCCCCAACATTTTTTAGGATTAGCTGGAATACCTCGACGATACTCGGATTTTCCCGATAGTTACTTAACTTGAAATATTATTTCTTCTTTAGGAAGAACAATTTCATTATTTGCCGTTATTTTCTTTTTATTTATTATTTGAGAAAGTATAATTACTCAACGAACACCTTCTTTC

>Australia-COXI-C08

TTCTGATTCTTTGGACACCCAGAAGTTTATATTTTAATTTTACCCGGATTTGGAATAATTTCTCATATTATTACTCAAGAAAGTGGAAAAAAGGAAACATTTGGAACTTTAGGAATAATTTATGCTATATTAACAATTGGATTATTGGGATTTATTGTTTGAGCTCATCATATATTTACAGTAGGTATAGACGTAGATACTCGAGCTTATTTTACTTCAGCAACTATAATTATTGCTGTTCCTACAGGAATTAAAATTTTTAGTTGATTAGCAACTTTACACGGAACTCAATTAACATATAGTCCAGCCCTTCTATGATCATTAGGATTTGTATTTTTATTTACAGTTGGAGGTTTAACAGGAGTAGTATTAGCTAATTCTTCAATTGATATTGTTCTTCATGATACTTATTATGTAGTTGCCCATTTTCATTATGTTTTATCTATAGGAGCTGTATTTGCTATTATAGCAGGATTTATTCATTGATACCCTTTATTAACAGGAATAGTTATAAACCCTTCATGATTAAAGGCTCAATTTAGTATAATATTTATTGGAGTAAATCTAACTTTCTTCCCCCAACATTTTTTAGGATTAGCTGGAATACCTCGACGATACTCGGATTTTCCCGATAGTTACTTAACTTGAAATATTATTTCTTCTTTAGGAAGAACAATTTCATTATTTGCCGTTATTTTCTTTTTATTTATTATTTGAGAAAGTATAATTACTCAACGAACACCTTCTTTC

>Australia-COXI-C09

TTCTGATTCTTTGGACACCCAGAAGTTTATATTTTAATTTTACCCGGATTTGGAATAATTTCTCATATTATTACTCAAGAAAGTGGAAAAAAGGAAACATTTGGAACTTTAGGAATAATTTATGCTATATTAACAATTGGATTATTGGGATTTATTGTTTGAGCTCATCATATATTTACAGTAGGTATAGACGTAGATACTCGAGCTTATTTTACTTCAGCAACTATAATTATTGCTGTTCCTACAGGAATTAAAATTTTTAGTTGATTAGCAACTTTACACGGAACTCAATTAACATATAGTCCAGCCCTTCTATGATCATTAGGATTTGTATTTTTATTTACAGTTGGAGGTTTAACAGGAGTAGTATTAGCTAATTCTTCAATTGATATTGTTCTTCATGATACTTATTATGTAGTTGCCCATTTTCATTATGTTTTATCTATAGGAGCTGTATTTGCTATTATAGCAGGATTTATTCATTGATACCCTTTATTAACAGGAATAGTTATAAACCCTTCATGATTAAAGGCTCAATTTAGTATAATATTTATTGGAGTAAATCTAACTTTCTTCCCCCAACATTTTTTAGGATTAGCTGGAATACCTCGACGATACTCGGATTTTCCCGATAGTTACTTAACTTGAAATATTATTTCTTCTTTAGGAAGAACAATTTCATTATTTGCCGTTATTTTCTTTTTATTTATTATTTGAGAAAGTATAATTACTCAACGAACACCTTCTTTC

>Australia-COXI-C10_(reversed)

TTCTGATTCTTTGGACACCCAGAAGTTTATATTTTAATTTTACCCGGATTTGGAATAATTTCTCATATTATTACTCAAGAAAGTGGAAAAAAGGAAACATTTGGAACTTTAGGAATAATTTATGCTATATTAACAATTGGATTATTGGGATTTATTGTTTGAGCTCATCATATATTTACAGTAGGTATAGACGTAGATACTCGAGCTTATTTTACTTCAGCAACTATAATTATTGCTGTTCCTACAGGAATTAAAATTTTTAGTTGATTAGCAACTTTACACGGAACTCAATTAACATATAGTCCAGCCCTTCTATGATCATTAGGATTTGTATTTTTATTTACAGTTGGAGGTTTAACAGGAGTAGTATTAGCTAATTCTTCAATTGATATTGTTCTTCATGATACTTATTATGTAGTTGCCCATTTTCATTATGTTTTATCTATAGGAGCTGTATTTGCTATTATAGCAGGATTTATTCATTGATACCCTTTATTAACAGGAATAGTTATAAACCCTTCATGATTAAAGGCTCAATTTAGTATAATATTTATTGGAGTAAATCTAACTTTCTTCCCCCAACATTTTTTAGGATTAGCTGGAATACCTCGACGATACTCGGATTTTCCCGATAGTTACTTAACTTGAAATATTATTTCTTCTTTAGGAAGAACAATTTCATTATTTGCCGTTATTTTCTTTTTATTTATTATTTGAGAAAGTATAATTACTCAACGAACACCTTCTTTC

>Australia-COXI-C11

TTCTGATTCTTTGGACACCCAGAAGTTTATATTTTAATTTTACCCGGATTTGGAATAATTTCTCATATTATTACTCAAGAAAGTGGAAAAAAGGAAACATTTGGAACTTTAGGAATAATTTATGCTATATTAACAATTGGATTATTGGGATTTATTGTTTGAGCTCATCATATATTTACAGTAGGTATAGACGTAGATACTCGAGCTTATTTTACTTCAGCAACTATAATTATTGCTGTTCCTACAGGAATTAAAATTTTTAGTTGATTAGCAACTTTACACGGAACTCAATTAACATATAGTCCAGCCCTTCTATGATCATTAGGATTTGTATTTTTATTTACAGTTGGAGGTTTAACAGGAGTAGTATTAGCTAATTCTTCAATTGATATTGTTCTTCATGATACTTATTATGTAGTTGCCCATTTTCATTATGTTTTATCTATAGGAGCTGTATTTGCTATTATAGCAGGATTTATTCATTGATACCCTTTATTAACAGGAATAGTTATAAACCCTTCATGATTAAAGGCTCAATTTAGTATAATATTTATTGGAGTAAATCTAACTTTCTTTCCCCAACATTTTTTAGGATTAGCTGGAATACCTCGACGATACTCAGATTTTCCCGATAGTTACTTAACTTGAAATATTATTTCTTCTTTAGGAAGAACAATTTCATTATTTGCCGTTATTTTCTTTTTATTTATTATTTGAGAAAGTATAATTACTCAACGAACACCGTCTTTC

>Australia-COXI-C12

TTCTGATTCTTTGGACACCCAGAAGTTTATATTTTAATTTTACCCGGATTTGGAATAATTTCTCATATTATTACTCAAGAAAGTGGAAAAAAGGAAACATTTGGAACTTTAGGAATAATTTATGCTATATTAACAATTGGATTATTGGGATTTATTGTTTGAGCTCATCATATATTTACAGTAGGTATAGACGTAGATACTCGAGCTTATTTTACTTCAGCAACTATAATTATTGCTGTTCCTACAGGAATTAAAATTTTTAGTTGATTAGCAACTTTACACGGAACTCAATTAACATATAGTCCAGCCCTTCTATGATCATTAGGATTTGTATTTTTATTTACAGTTGGAGGTTTAACAGGAGTAGTATTAGCTAATTCTTCAATTGATATTGTTCTTCATGATACTTATTATGTAGTTGCCCATTTTCATTATGTTTTATCTATAGGAGCTGTATTTGCTATTATAGCAGGATTTATTCATTGATACCCTTTATTAACAGGAATAGTTATAAACCCTTCATGATTAAAAGCTCAATTTAGTATAATATTTATTGGAGTAAATCTAACTTTCTTTCCCCAACATTTTTTAGGATTAGCTGGAATACCTCGACGATACTCAGATTTTCCCGATAGTTACTTAACTTGAAATATTATTTCTTCTTTAGGAAGAACAATTTCATTATTTGCCGTTATTTTCTTTTTATTTATTATTTGAGAAAGTATAATTACTCAACGAACACCGTCTTTC

>Australia-COXI-D01

TTCTGATTCTTTGGACACCCAGAAGTTTATATTTTAATTTTACCCGGATTTGGAATAATTTCTCATATTATTACTCAAGAAAGTGGAAAAAAGGAAACATTTGGAACTTTAGGAATAATTTATGCTATATTAACAATTGGATTATTGGGATTTATTGTTTGAGCTCATCATATATTTACAGTAGGTATAGACGTAGATACTCGAGCTTATTTTACTTCAGCAACTATAATTATTGCTGTTCCTACAGGAATTAAAATTTTTAGTTGATTAGCAACTTTACACGGAACTCAATTAACATATAGTCCAGCCCTTCTATGATCATTAGGATTTGTATTTTTATTTACAGTTGGAGGTTTAACAGGAGTAGTATTAGCTAATTCTTCAATTGATATTGTTCTTCATGATACTTATTATGTAGTTGCCCATTTTCATTATGTTTTATCTATAGGAGCTGTATTTGCTATTATAGCAGGATTTATTCATTGATACCCTTTATTAACAGGAATAGTTATAAACCCTTCATGATTAAAGGCTCAATTTAGTATAATATTTATTGGAGTAAATCTAACTTTCTTCCCCCAACATTTTTTAGGATTAGCTGGAATACCTCGACGATACTCGGATTTTCCCGATAGTTACTTAACTTGAAATATTATTTCTTCTTTAGGAAGAACAATTTCATTATTTGCCGTTATTTTCTTTTTATTTATTATTTGAGAAAGTATAATTACTCAACGAACACCTTCTTTC

>Australia-COXI-D02

TTCTGATTCTTTGGACACCCAGAAGTTTATATTTTAATTTTACCCGGATTTGGAATAATTTCTCATATTATTACTCAAGAAAGTGGAAAAAAGGAAACATTTGGAACTTTAGGAATAATTTATGCTATATTAACAATTGGATTATTGGGATTTATTGTTTGAGCTCATCATATATTTACAGTAGGTATAGACGTAGATACTCGAGCTTATTTTACTTCAGCAACTATAATTATTGCTGTTCCTACAGGAATTAAAATTTTTAGTTGATTAGCAACTTTACACGGAACTCAATTAACATATAGTCCAGCCCTTCTATGATCATTAGGATTTGTATTTTTATTTACAGTTGGAGGTTTAACAGGAGTAGTATTAGCTAATTCTTCAATTGATATTGTTCTTCATGATACTTATTATGTAGTTGCCCATTTTCATTATGTTTTATCTATAGGAGCTGTATTTGCTATTATAGCAGGATTTATTCATTGATACCCTTTATTAACAGGAATAGTTATAAACCCTTCATGATTAAAGGCTCAATTTAGTATAATATTTATTGGAGTAAATCTAACTTTCTTCCCCCAACATTTTTTAGGATTAGCTGGAATACCTCGACGATACTCGGATTTTCCCGATAGTTACTTAACTTGAAATATTATTTCTTCTTTAGGAAGAACAATTTCATTATTTGCCGTTATTTTCTTTTTATTTATTATTTGAGAAAGTATAATTACTCAACGAACACCTTCTTTC

>Australia-COXI-D03

TTCTGATTCTTTGGACACCCAGAAGTTTATATTTTAATTTTACCCGGATTTGGAATAATTTCTCATATTATTACTCAAGAAAGTGGAAAAAAGGAAACATTTGGAACTTTAGGAATAATTTATGCTATATTAACAATTGGATTATTGGGATTTATTGTTTGAGCTCATCATATATTTACAGTAGGTATAGACGTAGATACTCGAGCTTATTTTACTTCAGCAACTATAATTATTGCTGTTCCTACAGGAATTAAAATTTTTAGTTGATTAGCAACTTTACACGGAACTCAATTAACATATAGTCCAGCCCTTCTATGATCATTAGGATTTGTATTTTTATTTACAGTTGGAGGTTTAACAGGAGTAGTATTAGCTAATTCTTCAATTGATATTGTTCTTCATGATACTTATTATGTAGTTGCCCATTTTCATTATGTTTTATCTATAGGAGCTGTATTTGCTATTATAGCAGGATTTATTCATTGATACCCTTTATTAACAGGAATAGTTATAAACCCTTCATGATTAAAGGCTCAATTTAGTATAATATTTATTGGAGTAAATCTAACTTTCTTCCCCCAACATTTTTTAGGATTAGCTGGAATACCTCGACGATACTCGGATTTTCCCGATAGTTACTTAACTTGAAATATTATTTCTTCTTTAGGAAGAACAATTTCATTATTTGCCGTTATTTTCTTTTTATTTATTATTTGAGAAAGTATAATTACTCAACGAACACCTTCTTTC

>Australia-COXI-D04

TTCTGATTCTTTGGACACCCAGAAGTTTATATTTTAATTTTACCCGGATTTGGAATAATTTCTCATATTATTACTCAAGAAAGTGGAAAAAAGGAAACATTTGGAACTTTAGGAATAATTTATGCTATATTAACAATTGGATTATTGGGATTTATTGTTTGAGCTCATCATATATTTACAGTAGGTATAGACGTAGATACTCGAGCTTATTTTACTTCAGCAACTATAATTATTGCTGTTCCTACAGGAATTAAAATTTTTAGTTGATTAGCAACTTTACACGGAACTCAATTAACATATAGTCCAGCCCTTCTATGATCATTAGGATTTGTATTTTTATTTACAGTTGGAGGTTTAACAGGAGTAGTATTAGCTAATTCTTCAATTGATATTGTTCTTCATGATACTTATTATGTAGTTGCCCATTTTCATTATGTTTTATCTATAGGAGCTGTATTTGCTATTATAGCAGGATTTATTCATTGATACCCTTTATTAACAGGAATAGTTATAAACCCTTCATGATTAAAGGCTCAATTTAGTATAATATTTATTGGAGTAAATCTAACTTTCTTCCCCCAACATTTTTTAGGATTAGCTGGAATACCTCGACGATACTCGGATTTTCCCGATAGTTACTTAACTTGAAATATTATTTCTTCTTTAGGAAGAACAATTTCATTATTTGCCGTTATTTTCTTTTTATTTATTATTTGAGAAAGTATAATTACTCAACGAACACCTTCTTTC

>Australia-COXI-D05

TTCTGATTCTTTGGACACCCAGAAGTTTATATTTTAATTTTACCCGGATTTGGAATAATTTCTCATATTATTACTCAAGAAAGTGGAAAAAAGGAAACATTTGGAACTTTAGGAATAATTTATGCTATATTAACAATTGGATTATTGGGATTTATTGTTTGAGCTCATCATATATTTACAGTAGGTATAGACGTAGATACTCGAGCTTATTTTACTTCAGCAACTATAATTATTGCTGTTCCTACAGGAATTAAAATTTTTAGTTGATTAGCAACTTTACATGGAACTCAATTAACATATAGTCCAGCCCTTCTATGATCATTAGGATTTGTATTTTTATTTACAGTTGGAGGTTTAACAGGAGTAGTATTAGCTAATTCTTCAATTGATATTGTTCTTCATGATACTTATTATGTAGTTGCCCATTTTCATTATGTTTTATCTATAGGAGCTGTATTTGCTATTATAGCAGGATTTATTCATTGATACCCTTTATTAACAGGAATAGTTATAAACCCTTCATGATTAAAGGCTCAATTTAGTATAATATTTATTGGAGTAAATCTAACTTTCTTTCCCCAACATTTTTTAGGATTAGCTGGAATACCTCGACGATACTCAGATTTTCCCGATAGTTACTTAACTTGAAATATTATTTCTTCTTTAGGAAGAACAATTTCATTATTTGCCGTTATTTTCTTTTTATTTATTATTTGAGAAAGTATAATTACTCAACGAACACCGTCTTTC

>Australia-COXI-D06

TTCTGATTCTTTGGACACCCAGAAGTTTATATTTTAATTTTACCCGGATTTGGAATAATTTCTCATATTATTACTCAAGAAAGTGGAAAAAAGGAAACATTTGGAACTTTAGGAATAATTTATGCTATATTAACAATTGGATTATTGGGATTTATTGTTTGAGCTCATCATATATTTACAGTAGGTATAGACGTAGATACTCGAGCTTATTTTACTTCAGCAACTATAATTATTGCTGTTCCTACAGGAATTAAAATTTTTAGTTGATTAGCAACTTTACACGGAACTCAATTAACATATAGTCCAGCCCTTCTATGATCATTAGGATTTGTATTTTTATTTACAGTTGGAGGTTTAACAGGAGTAGTATTAGCTAATTCTTCAATTGATATTGTTCTTCATGATACTTATTATGTAGTTGCCCATTTTCATTATGTTTTATCTATAGGAGCTGTATTTGCTATTATAGCAGGATTTATTCATTGATACCCTTTATTAACAGGAATAGTTATAAACCCTTCATGATTAAAGGCTCAATTTAGTATAATATTTATTGGAGTAAATCTAACTTTCTTCCCCCAACATTTTTTAGGATTAGCTGGAATACCTCGACGATACTCGGATTTTCCCGATAGTTACTTAACTTGAAATATTATTTCTTCTTTAGGAAGAACAATTTCATTATTTGCCGTTATTTTCTTTTTATTTATTATTTGAGAAAGTATAATTACTCAACGAACACCTTCTTTC

>Australia-COXI-D07

TTCTGATTCTTTGGACACCCAGAAGTTTATATTTTAATTTTACCCGGATTTGGAATAATTTCTCATATTATTACTCAAGAAAGTGGAAAAAAGGAAACATTTGGAACTTTAGGAATAATTTATGCTATATTAACAATTGGATTATTGGGATTTATTGTTTGAGCTCATCATATATTTACAGTAGGTATAGACGTAGATACTCGAGCTTATTTTACTTCAGCAACTATAATTATTGCTGTTCCTACAGGAATTAAAATTTTTAGTTGATTAGCAACTTTACACGGAACTCAATTAACATATAGTCCAGCCCTTCTATGATCATTAGGATTTGTATTTTTATTTACAGTTGGAGGTTTAACAGGAGTAGTATTAGCTAATTCTTCAATTGATATTGTTCTTCATGATACTTATTATGTAGTTGCCCATTTTCATTATGTTTTATCTATAGGAGCTGTATTTGCTATTATAGCAGGATTTATTCATTGATACCCTTTATTAACAGGAATAGTTATAAACCCTTCATGATTAAAAGCTCAATTTAGTATAATATTTATTGGAGTAAATCTAACTTTCTTTCCCCAACATTTTTTAGGATTAGCTGGAATACCTCGACGATACTCAGATTTTCCCGATAGTTACTTAACTTGAAATATTATTTCTTCTTTAGGAAGAACAATTTCATTATTTGCCGTTATTTTCTTTTTATTTATTATTTGAGAAAGTATAATTACTCAACGAACACCGTCTTTC

>Australia-COXI-D08

TTCTGATTCTTTGGACACCCAGAAGTTTATATTTTAATTTTACCCGGATTTGGAATAATTTCTCATATTATTACTCAAGAAAGTGGAAAAAAGGAAACATTTGGAACTTTAGGAATAATTTATGCTATATTAACAATTGGATTATTGGGATTTATTGTTTGAGCTCATCATATATTTACAGTAGGTATAGACGTAGATACTCGAGCTTATTTTACTTCAGCAACTATAATTATTGCTGTTCCTACAGGAATTAAAATTTTTAGTTGATTAGCAACTTTACACGGAACTCAATTAACATATAGTCCAGCCCTTCTATGATCATTAGGATTTGTATTTTTATTTACAGTTGGAGGTTTAACAGGAGTAGTATTAGCTAATTCTTCAATTGATATTGTTCTTCATGATACTTATTATGTAGTTGCCCATTTTCATTATGTTTTATCTATAGGAGCTGTATTTGCTATTATAGCAGGATTTATTCATTGATACCCTTTATTAACAGGAATAGTTATAAACCCTTCATGATTAAAGGCTCAATTTAGTATAATATTTATTGGAGTAAATCTAACTTTCTTCCCCCAACATTTTTTAGGATTAGCTGGAATACCTCGACGATACTCGGATTTTCCCGATAGTTACTTAACTTGAAATATTATTTCTTCTTTAGGAAGAACAATTTCATTATTTGCCGTTATTTTCTTTTTATTTATTATTTGAGAAAGTATAATTACTCAACGAACACCTTCTTTC

>Australia-COXI-D09

TTCTGATTCTTTGGACACCCAGAAGTTTATATTTTAATTTTACCCGGATTTGGAATAATTTCTCATATTATTACTCAAGAAAGTGGAAAAAAGGAAACATTTGGAACTTTAGGAATAATTTATGCTATATTAACAATTGGATTATTGGGATTTATTGTTTGAGCTCATCATATATTTACAGTAGGTATAGACGTAGATACTCGAGCTTATTTTACTTCAGCAACTATAATTATTGCTGTTCCTACAGGAATTAAAATTTTTAGTTGATTAGCAACTTTACACGGAACTCAATTAACATATAGTCCAGCCCTTCTATGATCATTAGGATTTGTATTTTTATTTACAGTTGGAGGTTTAACAGGAGTAGTATTAGCTAATTCTTCAATTGATATTGTTCTTCATGATACTTATTATGTAGTTGCCCATTTTCATTATGTTTTATCTATAGGAGCTGTATTTGCTATTATAGCAGGATTTATTCATTGATACCCTTTATTAACAGGAATAGTTATAAACCCTTCATGATTAAAGGCTCAATTTAGTATAATATTTATTGGAGTAAATCTAACTTTCTTCCCCCAACATTTTTTAGGATTAGCTGGAATACCTCGACGATACTCGGATTTTCCCGATAGTTACTTAACTTGAAATATTATTTCTTCTTTAGGAAGAACAATTTCATTATTTGCCGTTATTTTCTTTTTATTTATTATTTGAGAAAGTATAATTACTCAACGAACACCTTCTTTC

>Australia-COXI-D10

TTCTGATTCTTTGGACACCCAGAAGTTTATATTTTAATTTTACCCGGATTTGGAATAATTTCTCATATTATTACTCAAGAAAGTGGAAAAAAGGAAACATTTGGAACTTTAGGAATAATTTATGCTATATTAACAATTGGATTATTGGGATTTATTGTTTGAGCTCATCATATATTTACAGTAGGTATAGACGTAGATACTCGAGCTTATTTTACTTCAGCAACTATAATTATTGCTGTTCCTACAGGAATTAAAATTTTTAGTTGATTAGCAACTTTACACGGAACTCAATTAACATATAGTCCAGCCCTTCTATGATCATTAGGATTTGTATTTTTATTTACAGTTGGAGGTTTAACAGGAGTAGTATTAGCTAATTCTTCAATTGATATTGTTCTTCATGATACTTATTATGTAGTTGCCCATTTTCATTATGTTTTATCTATAGGAGCTGTATTTGCTATTATAGCAGGATTTATTCATTGATACCCTTTATTAACAGGAATAGTTATAAACCCTTCATGATTAAAGGCTCAATTTAGTATAATATTTATTGGAGTAAATCTAACTTTCTTCCCCCAACATTTTTTAGGATTAGCTGGAATACCTCGACGATACTCGGATTTTCCCGATAGTTACTTAACTTGAAATATTATTTCTTCTTTAGGAAGAACAATTTCATTATTTGCCGTTATTTTCTTTTTATTTATTATTTGAGAAAGTATAATTACTCAACGAACACCTTCTTTC

>Australia-COXI-D11

TTCTGATTCTTTGGACACCCAGAAGTTTATATTTTAATTTTACCCGGATTTGGAATAATTTCTCATATTATTACTCAAGAAAGTGGAAAAAAGGAAACATTTGGAACTTTAGGAATAATTTATGCTATATTAACAATTGGATTATTGGGATTTATTGTTTGAGCTCATCATATATTTACAGTAGGTATAGACGTAGATACTCGAGCTTATTTTACTTCAGCAACTATAATTATTGCTGTTCCTACAGGAATTAAAATTTTTAGTTGATTAGCAACTTTACACGGAACTCAATTAACATATAGTCCAGCCCTTCTATGATCATTAGGATTTGTATTTTTATTTACAGTTGGAGGTTTAACAGGAGTAGTATTAGCTAATTCTTCAATTGATATTGTTCTTCATGATACTTATTATGTAGTTGCCCATTTTCATTATGTTTTATCTATAGGAGCTGTATTTGCTATTATAGCAGGATTTATTCATTGATACCCTTTATTAACAGGAATAGTTATAAACCCTTCATGATTAAAGGCTCAATTTAGTATAATATTTATTGGAGTAAATCTAACTTTCTTTCCCCAACATTTTTTAGGATTAGCTGGAATACCTCGACGATACTCAGATTTTCCCGATAGTTACTTAACTTGAAATATTATTTCTTCTTTAGGAAGAACAATTTCATTATTTGCCGTTATTTTCTTTTTATTTATTATTTGAGAAAGTATAATTACTCAACGAACACCGTCTTTC

>Australia-COXI-D12

TTCTGATTCTTTGGACACCCAGAAGTTTATATTTTAATTTTACCCGGATTTGGAATAATTTCTCATATTATTACTCAAGAAAGTGGAAAAAAGGAAACATTTGGAACTTTAGGAATAATTTATGCTATATTAACAATTGGATTATTGGGATTTATTGTTTGAGCTCATCATATATTTACAGTAGGTATAGACGTAGATACTCGAGCTTATTTTACTTCAGCAACTATAATTATTGCTGTTCCTACAGGAATTAAAATTTTTAGTTGATTAGCAACTTTACACGGAACTCAATTAACATATAGTCCAGCCCTTCTATGATCATTAGGATTTGTATTTTTATTTACAGTTGGAGGTTTAACAGGAGTAGTATTAGCTAATTCTTCAATTGATATTGTTCTTCATGATACTTATTATGTAGTTGCCCATTTTCATTATGTTTTATCTATAGGAGCTGTATTTGCTATTATAGCAGGATTTATTCATTGATACCCTTTATTAACAGGAATAGTTATAAACCCTTCATGATTAAAGGCTCAATTTAGTATAATATTTATTGGAGTAAATCTAACTTTCTTCCCCCAACATTTTTTAGGATTAGCTGGAATACCTCGACGATACTCGGATTTTCCCGATAGTTACTTAACTTGAAATATTATTTCTTCTTTAGGAAGAACAATTTCATTATTTGCCGTTATTTTCTTTTTATTTATTATTTGAGAAAGTATAATTACTCAACGAACACCTTCTTTC

>Australia-COXI-E01

TTCTGATTCTTTGGACACCCAGAAGTTTATATTTTAATTTTACCCGGATTTGGAATAATTTCTCATATTATTACTCAAGAAAGTGGAAAAAAGGAAACATTTGGAACTTTAGGAATAATTTATGCTATATTAACAATTGGATTATTGGGATTTATTGTTTGAGCTCATCATATATTTACAGTAGGTATAGACGTAGATACTCGAGCTTATTTTACTTCAGCAACTATAATTATTGCTGTTCCTACAGGAATTAAAATTTTTAGTTGATTAGCAACTTTACACGGAACTCAATTAACATATAGTCCAGCCCTTCTATGATCATTAGGATTTGTATTTTTATTTACAGTTGGAGGTTTAACAGGAGTAGTATTAGCTAATTCTTCAATTGATATTGTTCTTCATGATACTTATTATGTAGTTGCCCATTTTCATTATGTTTTATCTATAGGAGCTGTATTTGCTATTATAGCAGGATTTATTCATTGATACCCTTTATTAACAGGAATAGTTATAAACCCTTCATGATTAAAGGCTCAATTTAGTATAATATTTATTGGAGTAAATCTAACTTTCTTCCCCCAACATTTTTTAGGATTAGCTGGAATACCTCGACGATACTCGGATTTTCCCGATAGTTACTTAACTTGAAATATTATTTCTTCTTTAGGAAGAACAATTTCATTATTTGCCGTTATTTTCTTTTTATTTATTATTTGAGAAAGTATAATTACTCAACGAACACCTTCTTTC

>Australia-COXI-E02

TTCTGATTCTTTGGACACCCAGAAGTTTATATTTTAATTTTACCCGGATTTGGAATAATTTCTCATATTATTACTCAAGAAAGTGGAAAAAAGGAAACATTTGGAACTTTAGGAATAATTTATGCTATATTAACAATTGGATTATTGGGATTTATTGTTTGAGCTCATCATATATTTACAGTAGGTATAGACGTAGATACTCGAGCTTATTTTACTTCAGCAACTATAATTATTGCTGTTCCTACAGGAATTAAAATTTTTAGTTGATTAGCAACTTTACACGGAACTCAATTAACATATAGTCCAGCCCTTCTATGATCATTAGGATTTGTATTTTTATTTACAGTTGGAGGTTTAACAGGAGTAGTATTAGCTAATTCTTCAATTGATATTGTTCTTCATGATACTTATTATGTAGTTGCCCATTTTCATTATGTTTTATCTATAGGAGCTGTATTTGCTATTATAGCAGGATTTATTCATTGATACCCTTTATTAACAGGAATAGTTATAAACCCTTCATGATTAAAGGCTCAATTTAGTATAATATTTATTGGAGTAAATCTAACTTTCTTCCCCCAACATTTTTTAGGATTAGCTGGAATACCTCGACGATACTCGGATTTTCCCGATAGTTACTTAACTTGAAATATTATTTCTTCTTTAGGAAGAACAATTTCATTATTTGCCGTTATTTTCTTTTTATTTATTATTTGAGAAAGTATAATTACTCAACGAACACCTTCTTTC

>Australia-COXI-E03

TTCTGATTCTTTGGACACCCAGAAGTTTATATTTTAATTTTACCCGGATTTGGAATAATTTCTCATATTATTACTCAAGAAAGTGGAAAAAAGGAAACATTTGGAACTTTAGGAATAATTTATGCTATATTAACAATTGGATTATTGGGATTTATTGTTTGAGCTCATCATATATTTACAGTAGGTATAGACGTAGATACTCGAGCTTATTTTACTTCAGCAACTATAATTATTGCTGTTCCTACAGGAATTAAAATTTTTAGTTGATTAGCAACTTTACACGGAACTCAATTAACATATAGTCCAGCCCTTCTATGATCATTAGGATTTGTATTTTTATTTACAGTTGGAGGTTTAACAGGAGTAGTATTAGCTAATTCTTCAATTGATATTGTTCTTCATGATACTTATTATGTAGTTGCCCATTTTCATTATGTTTTATCTATAGGAGCTGTATTTGCTATTATAGCAGGATTTATTCATTGATACCCTTTATTAACAGGAATAGTTATAAACCCTTCATGATTAAAGGCTCAATTTAGTATAATATTTATTGGAGTAAATCTAACTTTCTTCCCCCAACATTTTTTAGGATTAGCTGGAATACCTCGACGATACTCGGATTTTCCCGATAGTTACTTAACTTGAAATATTATTTCTTCTTTAGGAAGAACAATTTCATTATTTGCCGTTATTTTCTTTTTATTTATTATTTGAGAAAGTATAATTACTCAACGAACACCTTCTTTC

>Australia-COXI-E04

TTCTGATTCTTTGGACACCCAGAAGTTTATATTTTAATTTTACCCGGATTTGGAATAATTTCTCATATTATTACTCAAGAAAGTGGAAAAAAGGAAACATTTGGAACTTTAGGAATAATTTATGCTATATTAACAATTGGATTATTGGGATTTATTGTTTGAGCTCATCATATATTTACAGTAGGTATAGACGTAGATACTCGAGCTTATTTTACTTCAGCAACTATAATTATTGCTGTTCCTACAGGAATTAAAATTTTTAGTTGATTAGCAACTTTACACGGAACTCAATTAACATATAGTCCAGCCCTTCTATGATCATTAGGATTTGTATTTTTATTTACAGTTGGAGGTTTAACAGGAGTAGTATTAGCTAATTCTTCAATTGATATTGTTCTTCATGATACTTATTATGTAGTTGCCCATTTTCATTATGTTTTATCTATAGGAGCTGTATTTGCTATTATAGCAGGATTTATTCATTGATACCCTTTATTAACAGGAATAGTTATAAACCCTTCATGATTAAAAGCTCAATTTAGTATAATATTTATTGGAGTAAATCTAACTTTCTTTCCCCAACATTTTTTAGGATTAGCTGGAATACCTCGACGATACTCAGATTTTCCCGATAGTTACTTAACTTGAAATATTATTTCTTCTTTAGGAAGAACAATTTCATTATTTGCCGTTATTTTCTTTTTATTTATTATTTGAGAAAGTATAATTACTCAACGAACACCGTCTTTC

>Australia-COXI-E05

TTCTGATTCTTTGGACACCCAGAAGTTTATATTTTAATTTTACCCGGATTTGGAATAATTTCTCATATTATTACTCAAGAAAGTGGAAAAAAGGAAACATTTGGAACTTTAGGAATAATTTATGCTATATTAACAATTGGATTATTGGGATTTATTGTTTGAGCTCATCATATATTTACAGTAGGTATAGACGTAGATACTCGAGCTTATTTTACTTCAGCAACTATAATTATTGCTGTTCCTACAGGAATTAAAATTTTTAGTTGATTAGCAACTTTACACGGAACTCAATTAACATATAGTCCAGCCCTTCTATGATCATTAGGATTTGTATTTTTATTTACAGTTGGAGGTTTAACAGGAGTAGTATTAGCTAATTCTTCAATTGATATTGTTCTTCATGATACTTATTATGTAGTTGCCCATTTTCATTATGTTTTATCTATAGGAGCTGTATTTGCTATTATAGCAGGATTTATTCATTGATACCCTTTATTAACAGGAATAGTTATAAACCCTTCATGATTAAAGGCTCAATTTAGTATAATATTTATTGGAGTAAATCTAACTTTCTTCCCCCAACATTTTTTAGGATTAGCTGGAATACCTCGACGATACTCGGATTTTCCCGATAGTTACTTAACTTGAAATATTATTTCTTCTTTAGGAAGAACAATTTCATTATTTGCCGTTATTTTCTTTTTATTTATTATTTGAGAAAGTATAATTACTCAACGAACACCTTCTTTC

>Australia-COXI-E06

TTCTGATTCTTTGGACACCCAGAAGTTTATATTTTAATTTTACCCGGATTTGGAATAATTTCTCATATTATTACTCAAGAAAGTGGAAAAAAGGAAACATTTGGAACTTTAGGAATAATTTATGCTATATTAACAATTGGATTATTGGGATTTATTGTTTGAGCTCATCATATATTTACAGTAGGTATAGACGTAGATACTCGAGCTTATTTTACTTCAGCAACTATAATTATTGCTGTTCCTACAGGAATTAAAATTTTTAGTTGATTAGCAACTTTACACGGAACTCAATTAACATATAGTCCAGCCCTTCTATGATCATTAGGATTTGTATTTTTATTTACAGTTGGAGGTTTAACAGGAGTAGTATTAGCTAATTCTTCAATTGATATTGTTCTTCATGATACTTATTATGTAGTTGCCCATTTTCATTATGTTTTATCTATAGGAGCTGTATTTGCTATTATAGCAGGATTTATTCATTGATACCCTTTATTAACAGGAATAGTTATAAACCCTTCATGATTAAAGGCTCAATTTAGTATAATATTTATTGGAGTAAATCTAACTTTCTTCCCCCAACATTTTTTAGGATTAGCTGGAATACCTCGACGATACTCGGATTTTCCCGATAGTTACTTAACTTGAAATATTATTTCTTCTTTAGGAAGAACAATTTCATTATTTGCCGTTATTTTCTTTTTATTTATTATTTGAGAAAGTATAATTACTCAACGAACACCTTCTTTC

>Australia-COXI-E07

TTCTGATTCTTTGGACACCCAGAAGTTTATATTTTAATTTTACCCGGATTTGGAATAATTTCTCATATTATTACTCAAGAAAGTGGAAAAAAGGAAACATTTGGAACTTTAGGAATAATTTATGCTATATTAACAATTGGATTATTGGGATTTATTGTTTGAGCTCATCATATATTTACAGTAGGTATAGACGTAGATACTCGAGCTTATTTTACTTCAGCAACTATAATTATTGCTGTTCCTACAGGAATTAAAATTTTTAGTTGATTAGCAACTTTACACGGAACTCAATTAACATATAGTCCAGCCCTTCTATGATCATTAGGATTTGTATTTTTATTTACAGTTGGAGGTTTAACAGGAGTAGTATTAGCTAATTCTTCAATTGATATTGTTCTTCATGATACTTATTATGTAGTTGCCCATTTTCATTATGTTTTATCTATAGGAGCTGTATTTGCTATTATAGCAGGATTTATTCATTGATACCCTTTATTAACAGGAATAGTTATAAACCCTTCATGATTAAAGGCTCAATTTAGTATAATATTTATTGGAGTAAATCTAACTTTCTTTCCCCAACATTTTTTAGGATTAGCTGGAATACCTCGACGATACTCAGATTTTCCCGATAGTTACTTAACTTGAAATATTATTTCTTCTTTAGGAAGAACAATTTCATTATTTGCCGTTATTTTCTTTTTATTTATTATTTGAGAAAGTATAATTACTCAACGAACACCGTCTTTC

>Australia-COXI-E08

TTCTGATTCTTTGGACACCCAGAAGTTTATATTTTAATTTTACCCGGATTTGGAATAATTTCTCATATTATTACTCAAGAAAGTGGAAAAAAGGAAACATTTGGAACTTTAGGAATAATTTATGCTATATTAACAATTGGATTATTGGGATTTATTGTTTGAGCTCATCATATATTTACAGTAGGTATAGACGTAGATACTCGAGCTTATTTTACTTCAGCAACTATAATTATTGCTGTTCCTACAGGAATTAAAATTTTTAGTTGATTAGCAACTTTACACGGAACTCAATTAACATATAGTCCAGCCCTTCTATGATCATTAGGATTTGTATTTTTATTTACAGTTGGAGGTTTAACAGGAGTAGTATTAGCTAATTCTTCAATTGATATTGTTCTTCATGATACTTATTATGTAGTTGCCCATTTTCATTATGTTTTATCTATAGGAGCTGTATTTGCTATTATAGCAGGATTTATTCATTGATACCCTTTATTAACAGGAATAGTTATAAACCCTTCATGATTAAAGGCTCAATTTAGTATAATATTTATTGGAGTAAATCTAACTTTCTTCCCCCAACATTTTTTAGGATTAGCTGGAATACCTCGACGATACTCGGATTTTCCCGATAGTTACTTAACTTGAAATATTATTTCTTCTTTAGGAAGAACAATTTCATTATTTGCCGTTATTTTCTTTTTATTTATTATTTGAGAAAGTATAATTACTCAACGAACACCTTCTTTC

>Australia-COXI-E09

TTCTGATTCTTTGGACACCCAGAAGTTTATATTTTAATTTTACCCGGATTTGGAATAATTTCTCATATTATTACTCAAGAAAGTGGAAAAAAGGAAACATTTGGAACTTTAGGAATAATTTATGCTATATTAACAATTGGATTATTGGGATTTATTGTTTGAGCTCATCATATATTTACAGTAGGTATAGACGTAGATACTCGAGCTTATTTTACTTCAGCAACTATAATTATTGCTGTTCCTACAGGAATTAAAATTTTTAGTTGATTAGCAACTTTACACGGAACTCAATTAACATATAGTCCAGCCCTTCTATGATCATTAGGATTTGTATTTTTATTTACAGTTGGAGGTTTAACAGGAGTAGTATTAGCTAATTCTTCAATTGATATTGTTCTTCATGATACTTATTATGTAGTTGCCCATTTTCATTATGTTTTATCTATAGGAGCTGTATTTGCTATTATAGCAGGATTTATTCATTGATACCCTTTATTAACAGGAATAGTTATAAACCCTTCATGATTAAAGGCTCAATTTAGTATAATATTTATTGGAGTAAATCTAACTTTCTTCCCCCAACATTTTTTAGGATTAGCTGGAATACCTCGACGATACTCGGATTTTCCCGATAGTTACTTAACTTGAAATATTATTTCTTCTTTAGGAAGAACAATTTCATTATTTGCCGTTATTTTCTTTTTATTTATTATTTGAGAAAGTATAATTACTCAACGAACACCTTCTTTC

>Australia-COXI-E10

TTCTGATTCTTTGGACACCCAGAAGTTTATATTTTAATTTTACCCGGATTTGGAATAATTTCTCATATTATTACTCAAGAAAGTGGAAAAAAGGAAACATTTGGAACTTTAGGAATAATTTATGCTATATTAACAATTGGATTATTGGGATTTATTGTTTGAGCTCATCATATATTTACAGTAGGTATAGACGTAGATACTCGAGCTTATTTTACTTCAGCAACTATAATTATTGCTGTTCCTACAGGAATTAAAATTTTTAGTTGATTAGCAACTTTACACGGAACTCAATTAACATATAGTCCAGCCCTTCTATGATCATTAGGATTTGTATTTTTATTTACAGTTGGAGGTTTAACAGGAGTAGTATTAGCTAATTCTTCAATTGATATTGTTCTTCATGATACTTATTATGTAGTTGCCCATTTTCATTATGTTTTATCTATAGGAGCTGTATTTGCTATTATAGCAGGATTTATTCATTGATACCCTTTATTAACAGGAATAGTTATAAACCCTTCATGATTAAAGGCTCAATTTAGTATAATATTTATTGGAGTAAATCTAACTTTCTTTCCCCAACATTTTTTAGGATTAGCTGGAATACCTCGACGATACTCAGATTTTCCCGATAGTTACTTAACTTGAAATATTATTTCTTCTTTAGGAAGAACAATTTCATTATTTGCCGTTATTTTCTTTTTATTTATTATTTGAGAAAGTATAATTACTCAACGAACACCKTCTTTC

>Australia-COXI-E11

TTCTGATTCTTTGGACACCCAGAAGTTTATATTTTAATTTTACCCGGATTTGGAATAATTTCTCATATTATTACTCAAGAAAGTGGAAAAAAGGAAACATTTGGAACTTTAGGAATAATTTATGCTATATTAACAATTGGATTATTGGGATTTATTGTTTGAGCTCATCATATATTTACAGTAGGTATAGACGTAGATACTCGAGCTTATTTTACTTCAGCAACTATAATTATTGCTGTTCCTACAGGAATTAAAATTTTTAGTTGATTAGCAACTTTACACGGAACTCAATTAACATATAGTCCAGCCCTTCTATGATCATTAGGATTTGTATTTTTATTTACAGTTGGAGGTTTAACAGGAGTAGTATTAGCTAATTCTTCAATTGATATTGTTCTTCATGATACTTATTATGTAGTTGCCCATTTTCATTATGTTTTATCTATAGGAGCTGTATTTGCTATTATAGCAGGATTTATTCATTGATACCCTTTATTAACAGGAATAGTTATAAACCCTTCATGATTAAAGGCTCAATTTAGTATAATATTTATTGGAGTAAATCTAACTTTCTTTCCCCAACATTTTTTAGGATTAGCTGGAATACCTCGACGATACTCAGATTTTCCCGATAGTTACTTAACTTGAAATATTATTTCTTCTTTAGGAAGAACAATTTCATTATTTGCCGTTATTTTCTTTTTATTTATTATTTGAGAAAGTATAATTACTCAACGAACACCGTCTTTC

>Australia-COXI-E12

TTCTGATTCTTTGGACACCCAGAAGTTTATATTTTAATTTTACCCGGATTTGGAATAATTTCTCATATTATTACTCAAGAAAGTGGAAAAAAGGAAACATTTGGAACTTTAGGAATAATTTATGCTATATTAACAATTGGATTATTGGGATTTATTGTTTGAGCTCATCATATATTTACAGTAGGTATAGACGTAGATACTCGAGCTTATTTTACTTCAGCAACTATAATTATTGCTGTTCCTACAGGAATTAAAATTTTTAGTTGATTAGCAACTTTACACGGAACTCAATTAACATATAGTCCAGCCCTTCTATGATCATTAGGATTTGTATTTTTATTTACAGTTGGAGGTTTAACAGGAGTAGTATTAGCTAATTCTTCAATTGATATTGTTCTTCATGATACTTATTATGTAGTTGCCCATTTTCATTATGTTTTATCTATAGGAGCTGTATTTGCTATTATAGCAGGATTTATTCATTGATACCCTTTATTAACAGGAATAGTTATAAACCCTTCATGATTAAAGGCTCAATTTAGTATAATATTTATTGGAGTAAATCTAACTTTCTTTCCCCAACATTTTTTAGGATTAGCTGGAATACCTCGACGATACTCAGATTTTCCCGATAGTTACTTAACTTGAAATATTATTTCTTCTTTAGGAAGAACAATTTCATTATTTGCCGTTATTTTCTTTTTATTTATTATTTGAGAAAGTATAATTACTCAACGAACACCGTCTTTC

>Australia-COXI-F01

TTCTGATTCTTTGGACACCCAGAAGTTTATATTTTAATTTTACCCGGATTTGGAATAATTTCTCATATTATTACTCAAGAAAGTGGAAAAAAGGAAACATTTGGAACTTTAGGAATAATTTATGCTATATTAACAATTGGATTATTGGGATTTATTGTTTGAGCTCATCATATATTTACAGTAGGTATAGACGTAGATACTCGAGCTTATTTTACTTCAGCAACTATAATTATTGCTGTTCCTACAGGAATTAAAATTTTTAGTTGATTAGCAACTTTACACGGAACTCAATTAACATATAGTCCAGCCCTTCTATGATCATTAGGATTTGTATTTTTATTTACAGTTGGAGGTTTAACAGGAGTAGTATTAGCTAATTCTTCAATTGATATTGTTCTTCATGATACTTATTATGTAGTTGCCCATTTTCATTATGTTTTATCTATAGGAGCTGTATTTGCTATTATAGCAGGATTTATTCATTGATACCCTTTATTAACAGGAATAGTTATAAACCCTTCATGATTAAAGGCTCAATTTAGTATAATATTTATTGGAGTAAATCTAACTTTCTTCCCCCAACATTTTTTAGGATTAGCTGGAATACCTCGACGATACTCGGATTTTCCCGATAGTTACTTAACTTGAAATATTATTTCTTCTTTAGGAAGAACAATTTCATTATTTGCCGTTATTTTCTTTTTATTTATTATTTGAGAAAGTATAATTACTCAACGAACACCTTCTTTC

>Australia-COXI-F02

TTCTGATTCTTTGGACACCCAGAAGTTTATATTTTAATTTTACCCGGATTTGGAATAATTTCTCATATTATTACTCAAGAAAGTGGAAAAAAGGAAACATTTGGAACTTTAGGAATAATTTATGCTATATTAACAATTGGATTATTGGGATTTATTGTTTGAGCTCATCATATATTTACAGTAGGTATAGACGTAGATACTCGAGCTTATTTTACTTCAGCAACTATAATTATTGCTGTTCCTACAGGAATTAAAATTTTTAGTTGATTAGCAACTTTACACGGAACTCAATTAACATATAGTCCAGCCCTTCTATGATCATTAGGATTTGTATTTTTATTTACAGTTGGAGGTTTAACAGGAGTAGTATTAGCTAATTCTTCAATTGATATTGTTCTTCATGATACTTATTATGTAGTTGCCCATTTTCATTATGTTTTATCTATAGGAGCTGTATTTGCTATTATAGCAGGATTTATTCATTGATACCCTTTATTAACAGGAATAGTTATAAACCCTTCATGATTAAAGGCTCAATTTAGTATAATATTTATTGGAGTAAATCTAACTTTCTTCCCCCAACATTTTTTAGGATTAGCTGGAATACCTCGACGATACTCGGATTTTCCCGATAGTTACTTAACTTGAAATATTATTTCTTCTTTAGGAAGAACAATTTCATTATTTGCCGTTATTTTCTTTTTATTTATTATTTGAGAAAGTATAATTACTCAACGAACACCTTCTTTC

>Australia-COXI-F06

TTCTGATTCTTTGGACACCCAGAAGTTTATATTTTAATTTTACCCGGATTTGGAATAATTTCTCATATTATTACTCAAGAAAGTGGAAAAAAGGAAACATTTGGAACTTTAGGAATAATTTATGCTATATTAACAATTGGATTATTGGGATTTATTGTTTGAGCTCATCATATATTTACAGTAGGTATAGACGTAGATACTCGAGCTTATTTTACTTCAGCAACTATAATTATTGCTGTTCCTACAGGAATTAAAATTTTTAGTTGATTAGCAACTTTACACGGAACTCAATTAACATATAGTCCAGCCCTTCTATGATCATTAGGATTTGTATTTTTATTTACAGTTGGAGGTTTAACAGGAGTAGTATTAGCTAATTCTTCAATTGATATTGTTCTTCATGATACTTATTATGTAGTTGCCCATTTTCATTATGTTTTATCTATAGGAGCTGTATTTGCTATTATAGCAGGATTTATTCATTGATACCCTTTATTAACAGGAATAGTTATAAACCCTTCATGATTAAAAGCTCAATTTAGTATAATATTTATTGGAGTAAATCTAACTTTCTTTCCCCAACATTTTTTAGGATTAGCTGGAATACCTCGACGATACTCAGATTTTCCCGATAGTTACTTAACTTGAAATATTATTTCTTCTTTAGGAAGAACAATTTCATTATTTGCCGTTATTTTCTTTTTATTTATTATTTGAGAAAGTATAATTACTCAACGAACACCGTCTTTC

>Australia-COXI-F07

TTCTGATTCTTTGGACACCCAGAAGTTTATATTTTAATTTTACCCGGATTTGGAATAATTTCTCATATTATTACTCAAGAAAGTGGAAAAAAGGAAACATTTGGAACTTTAGGAATAATTTATGCTATATTAACAATTGGATTATTGGGATTTATTGTTTGAGCTCATCATATATTTACAGTAGGTATAGACGTAGATACTCGAGCTTATTTTACTTCAGCAACTATAATTATTGCTGTTCCTACAGGAATTAAAATTTTTAGTTGATTAGCAACTTTACACGGAACTCAATTAACATATAGTCCAGCCCTTCTATGATCATTAGGATTTGTATTTTTATTTACAGTTGGAGGTTTAACAGGAGTAGTATTAGCTAATTCTTCAATTGATATTGTTCTTCATGATACTTATTATGTAGTTGCCCATTTTCATTATGTTTTATCTATAGGAGCTGTATTTGCTATTATAGCAGGATTTATTCATTGATACCCTTTATTAACAGGAATAGTTATAAACCCTTCATGATTAAAGGCTCAATTTAGTATAATATTTATTGGAGTAAATCTAACTTTCTTCCCCCAACATTTTTTAGGATTAGCTGGAATACCTCGACGATACTCGGATTTTCCCGATAGTTACTTAACTTGAAATATTATTTCTTCTTTAGGAAGAACAATTTCATTATTTGCCGTTATTTTCTTTTTATTTATTATTTGAGAAAGTATAATTACTCAACGAACACCTTCTTTC

>Australia-COXI-F08

TTCTGATTCTTTGGACACCCAGAAGTTTATATTTTAATTTTACCCGGATTTGGAATAATTTCTCATATTATTACTCAAGAAAGTGGAAAAAAGGAAACATTTGGAACTTTAGGAATAATTTATGCTATATTAACAATTGGATTATTGGGATTTATTGTTTGAGCTCATCATATATTTACAGTAGGTATAGACGTAGATACTCGAGCTTATTTTACTTCAGCAACTATAATTATTGCTGTTCCTACAGGAATTAAAATTTTTAGTTGATTAGCAACTTTACACGGAACTCAATTAACATATAGTCCAGCCCTTCTATGATCATTAGGATTTGTATTTTTATTTACAGTTGGAGGTTTAACAGGAGTAGTATTAGCTAATTCTTCAATTGATATTGTTCTTCATGATACTTATTATGTAGTTGCCCATTTTCATTATGTTTTATCTATAGGAGCTGTATTTGCTATTATAGCAGGATTTATTCATTGATACCCTTTATTAACAGGAATAGTTATAAACCCTTCATGATTAAAGGCTCAATTTAGTATAATATTTATTGGAGTAAATCTAACTTTCTTCCCCCAACATTTTTTAGGATTAGCTGGAATACCTCGACGATACTCGGATTTTCCCGATAGTTACTTAACTTGAAATATTATTTCTTCTTTAGGAAGAACAATTTCATTATTTGCCGTTATTTTCTTTTTATTTATTATTTGAGAAAGTATAATTACTCAACGAACACCTTCTTTC

>Australia-COXI-F10

TTTTGATTCTTTGGACACCCAGAAGTTTATATTTTAATTTTACCCGGATTTGGAATAATTTCTCATATTATTACTCAAGAAAGTGGAAAAAAGGAAACATTTGGAACTTTAGGAATAATTTATGCTATATTAACAATTGGATTATTGGGATTTATTGTTTGAGCTCATCATATATTTACAGTAGGTATAGATGTAGATACTCGAGCTTATTTTACTTCAGCAACTATAATTATTGCTGTTCCTACAGGAATTAAAATTTTTAGTTGATTAGCAACTTTACACGGAACTCAATTAACATATAGTCCAGCCCTTCTATGATCATTAGGATTTGTATTTTTATTTACAGTTGGAGGTTTAACAGGAGTAGTATTAGCTAATTCTTCAATTGATATTGTTCTTCATGATACTTATTACGTAGTTGCCCATTTTCATTACGTTTTATCTATAGGAGCTGTATTTGCTATTATAGCAGGATTTATTCATTGATACCCTTTATTAACAGGAATAGTTATAAACCCTTCATGATTAAAGGCTCAATTTAGTATAATATTTATTGGAGTAAATCTAACTTTCTTTCCTCAACATTTTTTAGGRTTAGCTGGAATACCTCGACGATACTCAGATTTTCCTGATAGTTACTTAACTTGAAATATTATTTCTTCTTTAGGAAGAACAATTTCACTATTTGCCGTTATTTTCTTTTTATTTATTATTTGAGAAAGTATAATTACTCAACGAACACCTTCTTTC

>Australia-COXI-F11

TTCTGATTCTTTGGACACCCAGAAGTTTATATTTTAATTTTACCCGGATTTGGAATAATTTCTCATATTATTACTCAAGAAAGTGGAAAAAAGGAAACATTTGGAACTTTAGGAATAATTTATGCTATATTAACAATTGGATTATTGGGATTTATTGTTTGAGCTCATCATATATTTACAGTAGGTATAGACGTAGATACTCGAGCTTATTTTACTTCAGCAACTATAATTATTGCTGTTCCTACAGGAATTAAAATTTTTAGTTGATTAGCAACTTTACACGGAACTCAATTAACATATAGTCCAGCCCTTCTATGATCATTAGGATTTGTATTTTTATTTACAGTTGGAGGTTTAACAGGAGTAGTATTAGCTAATTCTTCAATTGATATTGTTCTTCATGATACTTATTATGTAGTTGCCCATTTTCATTATGTTTTATCTATAGGAGCTGTATTTGCTATTATAGCAGGATTTATTCATTGATACCCTTTATTAACAGGAATAGTTATAAACCCTTCATGATTAAAGGCTCAATTTAGTATAATATTTATTGGAGTAAATCTAACTTTCTTCCCCCAACATTTTTTAGGATTAGCTGGAATACCTCGACGATACTCGGATTTTCCCGATAGTTACTTAACTTGAAATATTATTTCTTCTTTAGGAAGAACAATTTCATTATTTGCCGTTATTTTCTTTTTATTTATTATTTGAGAAAGTATAATTACTCAACGAACACCTTCTTTC

>Australia-COXI-F12

TTCTGATTCTTTGGACACCCAGAAGTTTATATTTTAATTTTACCCGGATTTGGAATAATTTCTCATATTATTACTCAAGAAAGTGGAAAAAAGGAAACATTTGGAACTTTAGGAATAATTTATGCTATATTAACAATTGGATTATTGGGATTTATTGTTTGAGCTCATCATATATTTACAGTAGGTATAGACGTAGATACTCGAGCTTATTTTACTTCAGCAACTATAATTATTGCTGTTCCTACAGGAATTAAAATTTTTAGTTGATTAGCAACTTTACACGGAACTCAATTAACATATAGTCCAGCCCTTCTATGATCATTAGGATTTGTATTTTTATTTACAGTTGGAGGTTTAACAGGAGTAGTATTAGCTAATTCTTCAATTGATATTGTTCTTCATGATACTTATTATGTAGTTGCCCATTTTCATTATGTTTTATCTATAGGAGCTGTATTTGCTATTATAGCAGGATTTATTCATTGATACCCTTTATTAACAGGAATAGTTATAAACCCTTCATGATTAAAGGCTCAATTTAGTATAATATTTATTGGAGTAAATCTAACTTTCTTTCCCCAACATTTTTTAGGATTAGCTGGAATACCTCGACGATACTCAGATTTTCCCGATAGTTACTTAACTTGAAATATTATTTCTTCTTTAGGAAGAACAATTTCATTATTTGCCGTTATTTTCTTTTTATTTATTATTTGAGAAAGTATAATTACTCAACGAACACCGTCTTTC

>Australia-COXI-G04

TTCTGATTCTTTGGACACCCAGAAGTTTATATTTTAATTTTACCCGGATTTGGAATAATTTCTCATATTATTACTCAAGAAAGTGGAAAAAAGGAAACATTTGGAACTTTAGGAATAATTTATGCTATATTAACAATTGGATTATTGGGATTTATTGTTTGAGCTCATCATATATTTACAGTAGGTATAGACGTAGATACTCGAGCTTATTTTACTTCAGCAACTATAATTATTGCTGTTCCTACAGGAATTAAAATTTTTAGTTGATTAGCAACTTTACACGGAACTCAATTAACATATAGTCCAGCCCTTCTATGATCATTAGGATTTGTATTTTTATTTACAGTTGGAGGTTTAACAGGAGTAGTATTAGCTAATTCTTCAATTGATATTGTTCTTCATGATACTTATTATGTAGTTGCCCATTTTCATTATGTTTTATCTATAGGAGCTGTATTTGCTATTATAGCAGGATTTATTCATTGATACCCTTTATTAACAGGAATAGTTATAAACCCTTCATGATTAAAGGCTCAATTTAGTATAATATTTATTGGAGTAAATCTAACTTTCTTCCCCCAACATTTTTTAGGATTAGCTGGAATACCTCGACGATACTCGGATTTTCCCGATAGTTACTTAACTTGAAATATTATTTCTTCTTTAGGAAGAACAATTTCATTATTTGCCGTTATTTTCTTTTTATTTATTATTTGAGAAAGTATAATTACTCAACGAACACCTTCTTTC

>Australia-COXI-G05

TTCTGATTCTTTGGACACCCAGAAGTTTATATTTTAATTTTACCCGGATTTGGAATAATTTCTCATATTATTACTCAAGAAAGTGGAAAAAAGGAAACATTTGGAACTTTAGGAATAATTTATGCTATATTAACAATTGGATTATTGGGATTTATTGTTTGAGCTCATCATATATTTACAGTAGGTATAGACGTAGATACTCGAGCTTATTTTACTTCAGCAACTATAATTATTGCTGTTCCTACAGGAATTAAAATTTTTAGTTGATTAGCAACTTTACACGGAACTCAATTAACATATAGTCCAGCCCTTCTATGATCATTAGGATTTGTATTTTTATTTACAGTTGGAGGTTTAACAGGAGTAGTATTAGCTAATTCTTCAATTGATATTGTTCTTCATGATACTTATTATGTAGTTGCCCATTTTCATTATGTTTTATCTATAGGAGCTGTATTTGCTATTATAGCAGGATTTATTCATTGATACCCTTTATTAACAGGAATAGTTATAAACCCTTCATGATTAAAGGCTCAATTTAGTATAATATTTATTGGAGTAAATCTAACTTTCTTCCCCCAACATTTTTTAGGATTAGCTGGAATACCTCGACGATACTCGGATTTTCCCGATAGTTACTTAACTTGAAATATTATTTCTTCTTTAGGAAGAACAATTTCATTATTTGCCGTTATTTTCTTTTTATTTATTATTTGAGAAAGTATAATTACTCAACGAACACCTTCTTTC

>Australia-COXI-G06

TTCTGATTCTTTGGACACCCAGAAGTTTATATTTTAATTTTACCCGGATTTGGAATAATTTCTCATATTATTACTCAAGAAAGTGGAAAAAAGGAAACATTTGGAACTTTAGGAATAATTTATGCTATATTAACAATTGGATTATTGGGATTTATTGTTTGAGCTCATCATATATTTACAGTAGGTATAGACGTAGATACTCGAGCTTATTTTACTTCAGCAACTATAATTATTGCTGTTCCTACAGGAATTAAAATTTTTAGTTGATTAGCAACTTTACACGGAACTCAATTAACATATAGTCCAGCCCTTCTATGATCATTAGGATTTGTATTTTTATTTACAGTTGGAGGTTTAACAGGAGTAGTATTAGCTAATTCTTCAATTGATATTGTTCTTCATGATACTTATTATGTAGTTGCCCATTTTCATTATGTTTTATCTATAGGAGCTGTATTTGCTATTATAGCAGGATTTATTCATTGATACCCTTTATTAACAGGAATAGTTATAAACCCTTCATGATTAAAGGCTCAATTTAGTATAATATTTATTGGAGTAAATCTAACTTTCTTCCCCCAACATTTTTTAGGATTAGCTGGAATACCTCGACGATACTCGGATTTTCCCGATAGTTACTTAACTTGAAATATTATTTCTTCTTTAGGAAGAACAATTTCATTATTTGCCGTTATTTTCTTTTTATTTATTATTTGAGAAAGTATAATTACTCAACGAACACCTTCTTTC

>Australia-COXI-G07

TTCTGATTCTTTGGACACCCAGAAGTTTATATTTTAATTTTACCCGGATTTGGAATAATTTCTCATATTATTACTCAAGAAAGTGGAAAAAAGGAAACATTTGGAACTTTAGGAATAATTTATGCTATATTAACAATTGGATTATTGGGATTTATTGTTTGAGCTCATCATATATTTACAGTAGGTATAGACGTAGATACTCGAGCTTATTTTACTTCAGCAACTATAATTATTGCTGTTCCTACAGGAATTAAAATTTTTAGTTGATTAGCAACTTTACACGGAACTCAATTAACATATAGTCCAGCCCTTCTATGATCATTAGGATTTGTATTTTTATTTACAGTTGGAGGTTTAACAGGAGTAGTATTAGCTAATTCTTCAATTGATATTGTTCTTCATGATACTTATTATGTAGTTGCCCATTTTCATTATGTTTTATCTATAGGAGCTGTATTTGCTATTATAGCAGGATTTATTCATTGATACCCTTTATTAACAGGAATAGTTATAAACCCTTCATGATTAAAGGCTCAATTTAGTATAATATTTATTGGAGTAAATCTAACTTTCTTCCCCCAACATTTTTTAGGATTAGCTGGAATACCTCGACGATACTCGGATTTTCCCGATAGTTACTTAACTTGAAATATTATTTCTTCTTTAGGAAGAACAATTTCATTATTTGCCGTTATTTTCTTTTTATTTATTATTTGAGAAAGTATAATTACTCAACGAACACCTTCTTTC

>Australia-COXI-G08

TTCTGATTCTTTGGACACCCAGAAGTTTATATTTTAATTTTACCCGGATTTGGAATAATTTCTCATATTATTACTCAAGAAAGTGGAAAAAAGGAAACATTTGGAACTTTAGGAATAATTTATGCTATATTAACAATTGGATTATTGGGATTTATTGTTTGAGCTCATCATATATTTACAGTAGGTATAGACGTAGATACTCGAGCTTATTTTACTTCAGCAACTATAATTATTGCTGTTCCTACAGGAATTAAAATTTTTAGTTGATTAGCAACTTTACACGGAACTCAATTAACATATAGTCCAGCCCTTCTATGATCATTAGGATTTGTATTTTTATTTACAGTTGGAGGTTTAACAGGAGTAGTATTAGCTAATTCTTCAATTGATATTGTTCTTCATGATACTTATTATGTAGTTGCCCATTTTCATTATGTTTTATCTATAGGAGCTGTATTTGCTATTATAGCAGGATTTATTCATTGATACCCTTTATTAACAGGAATAGTTATAAACCCTTCATGATTAAAAGCTCAATTTAGTATAATATTTATTGGAGTAAATCTAACTTTCTTTCCCCAACATTTTTTAGGATTAGCTGGAATACCTCGACGATACTCAGATTTTCCCGATAGTTACTTAACTTGAAATATTATTTCTTCTTTAGGAAGAACAATTTCATTATTTGCCGTTATTTTCTTTTTATTTATTATTTGAGAAAGTATAATTACTCAACGAACACCGTCTTTC

>Australia-COXI-G09

TTCTGATTCTTTGGACACCCAGAAGTTTATATTTTAATTTTACCCGGATTTGGAATAATTTCTCATATTATTACTCAAGAAAGTGGAAAAAAGGAAACATTTGGAACTTTAGGAATAATTTATGCTATATTAACAATTGGATTATTGGGATTTATTGTTTGAGCTCATCATATATTTACAGTAGGTATAGACGTAGATACTCGAGCTTATTTTACTTCAGCAACTATAATTATTGCTGTTCCTACAGGAATTAAAATTTTTAGTTGATTAGCAACTTTACACGGAACTCAATTAACATATAGTCCAGCCCTTCTATGATCATTAGGATTTGTATTTTTATTTACAGTTGGAGGTTTAACAGGAGTAGTATTAGCTAATTCTTCAATTGATATTGTTCTTCATGATACTTATTATGTAGTTGCCCATTTTCATTATGTTTTATCTATAGGAGCTGTATTTGCTATTATAGCAGGATTTATTCATTGATACCCTTTATTAACAGGAATAGTTATAAACCCTTCATGATTAAAGGCTCAATTTAGTATAATATTTATTGGAGTAAATCTAACTTTCTTCCCCCAACATTTTTTAGGATTAGCTGGAATACCTCGACGATACTCGGATTTTCCCGATAGTTACTTAACTTGAAATATTATTTCTTCTTTAGGAAGAACAATTTCATTATTTGCCGTTATTTTCTTTTTATTTATTATTTGAGAAAGTATAATTACTCAACGAACACCTTCTTTC

>Australia-COXI-G10

TTCTGATTCTTTGGACACCCAGAAGTTTATATTTTAATTTTACCCGGATTTGGAATAATTTCTCATATTATTACTCAAGAAAGTGGAAAAAAGGAAACATTTGGAACTTTAGGAATAATTTATGCTATATTAACAATTGGATTATTGGGATTTATTGTTTGAGCTCATCATATATTTACAGTAGGTATAGACGTAGATACTCGAGCTTATTTTACTTCAGCAACTATAATTATTGCTGTTCCTACAGGAATTAAAATTTTTAGTTGATTAGCAACTTTACACGGAACTCAATTAACATATAGTCCAGCCCTTCTATGATCATTAGGATTTGTATTTTTATTTACAGTTGGAGGTTTAACAGGAGTAGTATTAGCTAATTCTTCAATTGATATTGTTCTTCATGATACTTATTATGTAGTTGCCCATTTTCATTATGTTTTATCTATAGGAGCTGTATTTGCTATTATAGCAGGATTTATTCATTGATACCCTTTATTAACAGGAATAGTTATAAACCCTTCATGATTAAAGGCTCAATTTAGTATAATATTTATTGGAGTAAATCTAACTTTCTTCCCCCAACATTTTTTAGGATTAGCTGGAATACCTCGACGATACTCGGATTTTCCCGATAGTTACTTAACTTGAAATATTATTTCTTCTTTAGGAAGAACAATTTCATTATTTGCCGTTATTTTCTTTTTATTTATTATTTGAGAAAGTATAATTACTCAACGAACACCTTCTTTC

>Australia-COXI-G11

TTCTGATTCTTTGGACACCCAGAAGTTTATATTTTAATTTTACCCGGATTTGGAATAATTTCTCATATTATTACTCAAGAAAGTGGAAAAAAGGAAACATTTGGAACTTTAGGAATAATTTATGCTATATTAACAATTGGATTATTGGGATTTATTGTTTGAGCTCATCATATATTTACAGTAGGTATAGACGTAGATACTCGAGCTTATTTTACTTCAGCAACTATAATTATTGCTGTTCCTACAGGAATTAAAATTTTTAGTTGATTAGCAACTTTACACGGAACTCAATTAACATATAGTCCAGCCCTTCTATGATCATTAGGATTTGTATTTTTATTTACAGTTGGAGGTTTAACAGGAGTAGTATTAGCTAATTCTTCAATTGATATTGTTCTTCATGATACTTATTATGTAGTTGCCCATTTTCATTATGTTTTATCTATAGGAGCTGTATTTGCTATTATAGCAGGATTTATTCATTGATACCCTTTATTAACAGGAATAGTTATAAACCCTTCATGATTAAAGGCTCAATTTAGTATAATATTTATTGGAGTAAATCTAACTTTCTTCCCCCAACATTTTTTAGGATTAGCTGGAATACCTCGACGATACTCGGATTTTCCCGATAGTTACTTAACTTGAAATATTATTTCTTCTTTAGGAAGAACAATTTCATTATTTGCCGTTATTTTCTTTTTATTTATTATTTGAGAAAGTATAATTACTCAACGAACACCTTCTTTC

>Australia-COXI-G12

TTCTGATTCTTTGGACACCCAGAAGTTTATATTTTAATTTTACCCGGATTTGGAATAATTTCTCATATTATTACTCAAGAAAGTGGAAAAAAGGAAACATTTGGAACTTTAGGAATAATTTATGCTATATTAACAATTGGATTATTGGGATTTATTGTTTGAGCTCATCATATATTTACAGTAGGTATAGACGTAGATACTCGAGCTTATTTTACTTCAGCAACTATAATTATTGCTGTTCCTACAGGAATTAAAATTTTTAGTTGATTAGCAACTTTACACGGAACTCAATTAACATATAGTCCAGCCCTTCTATGATCATTAGGATTTGTATTTTTATTTACAGTTGGAGGTTTAACAGGAGTAGTATTAGCTAATTCTTCAATTGATATTGTTCTTCATGATACTTATTATGTAGTTGCCCATTTTCATTATGTTTTATCTATAGGAGCTGTATTTGCTATTATAGCAGGATTTATTCATTGATACCCTTTATTAACAGGAATAGTTATAAACCCTTCATGATTAAAGGCTCAATTTAGTATAATATTTATTGGAGTAAATCTAACTTTCTTCCCCCAACATTTTTTAGGATTAGCTGGAATACCTCGACGATACTCGGATTTTCCCGATAGTTACTTAACTTGAAATATTATTTCTTCTTTAGGAAGAACAATTTCATTATTTGCCGTTATTTTCTTTTTATTTATTATTTGAGAAAGTATAATTACTCAACGAACACCTTCTTTC

>Australia-COXI-H01

TTCTGATTCTTTGGACACCCAGAAGTTTATATTTTAATTTTACCCGGATTTGGAATAATTTCTCATATTATTACTCAAGAAAGTGGAAAAAAGGAAACATTTGGAACTTTAGGAATAATTTATGCTATATTAACAATTGGATTATTGGGATTTATTGTTTGAGCTCATCATATATTTACAGTAGGTATAGACGTAGATACTCGAGCTTATTTTACTTCAGCAACTATAATTATTGCTGTTCCTACAGGAATTAAAATTTTTAGTTGATTAGCAACTTTACACGGAACTCAATTAACATATAGTCCAGCCCTTCTATGATCATTAGGATTTGTATTTTTATTTACAGTTGGAGGTTTAACAGGAGTAGTATTAGCTAATTCTTCAATTGATATTGTTCTTCATGATACTTATTATGTAGTTGCCCATTTTCATTATGTTTTATCTATAGGAGCTGTATTTGCTATTATAGCAGGATTTATTCATTGATACCCTTTATTAACAGGAATAGTTATAAACCCTTCATGATTAAAGGCTCAATTTAGTATAATATTTATTGGAGTAAATCTAACTTTCTTCCCCCAACATTTTTTAGGATTAGCTGGAATACCTCGACGATACTCGGATTTTCCCGATAGTTACTTAACTTGAAATATTATTTCTTCTTTAGGAAGAACAATTTCATTATTTGCCGTTATTTTCTTTTTATTTATTATTTGAGAAAGTATAATTACTCAACGAACACCTTCTTTC

>Australia-COXI-H02

TTCTGATTCTTTGGACACCCAGAAGTTTATATTTTAATTTTACCCGGATTTGGAATAATTTCTCATATTATTACTCAAGAAAGTGGAAAAAAGGAAACATTTGGAACTTTAGGAATAATTTATGCTATATTAACAATTGGATTATTGGGATTTATTGTTTGAGCTCATCATATATTTACAGTAGGTATAGACGTAGATACTCGAGCTTATTTTACTTCAGCAACTATAATTATTGCTGTTCCTACAGGAATTAAAATTTTTAGTTGATTAGCAACTTTACACGGAACTCAATTAACATATAGTCCAGCCCTTCTATGATCATTAGGATTTGTATTTTTATTTACAGTTGGAGGTTTAACAGGAGTAGTATTAGCTAATTCTTCAATTGATATTGTTCTTCATGATACTTATTATGTAGTTGCCCATTTTCATTATGTTTTATCTATAGGAGCTGTATTTGCTATTATAGCAGGATTTATTCATTGATACCCTTTATTAACAGGAATAGTTATAAACCCTTCATGATTAAAGGCTCAATTTAGTATAATATTTATTGGAGTAAATCTAACTTTCTTTCCCCAACATTTTTTAGGATTAGCTGGAATACCTCGACGATACTCAGATTTTCCCGATAGTTACTTAACTTGAAATATTATTTCTTCTTTAGGAAGAACAATTTCATTATTTGCCGTTATTTTCTTTTTATTTATTATTTGAGAAAGTATAATTACTCAACGAACACCGTCTTTC

>Australia-COXI-H04

TTCTGATTCTTTGGACACCCAGAAGTTTATATTTTAATTTTACCCGGATTTGGAATAATTTCTCATATTATTACTCAAGAAAGTGGAAAAAAGGAAACATTTGGAACTTTAGGAATAATTTATGCTATATTAACAATTGGATTATTGGGATTTATTGTTTGAGCTCATCATATATTTACAGTAGGTATAGACGTAGATACTCGAGCTTATTTTACTTCAGCAACTATAATTATTGCTGTTCCTACAGGAATTAAAATTTTTAGTTGATTAGCAACTTTACACGGAACTCAATTAACATATAGTCCAGCCCTTCTATGATCATTAGGATTTGTATTTTTATTTACAGTTGGAGGTTTAACAGGAGTAGTATTAGCTAATTCTTCAATTGATATTGTTCTTCATGATACTTATTATGTAGTTGCCCATTTTCATTATGTTTTATCTATAGGAGCTGTATTTGCTATTATAGCAGGATTTATTCATTGATACCCTTTATTAACAGGAATAGTTATAAACCCTTCATGATTAAAGGCTCAATTTAGTATAATATTTATTGGAGTAAATCTAACTTTCTTCCCCCAACATTTTTTAGGATTAGCTGGAATACCTCGACGATACTCGGATTTTCCCGATAGTTACTTAACTTGAAATATTATTTCTTCTTTAGGAAGAACAATTTCATTATTTGCCGTTATTTTCTTTTTATTTATTATTTGAGAAAGTATAATTACTCAACGAACACCTTCTTTC

>Australia-COXI-H05

TTCTGATTCTTTGGACACCCAGAAGTTTATATTTTAATTTTACCCGGATTTGGAATAATTTCTCATATTATTACTCAAGAAAGTGGAAAAAAGGAAACATTTGGAACTTTAGGAATAATTTATGCTATATTAACAATTGGATTATTGGGATTTATTGTTTGAGCTCATCATATATTTACAGTAGGTATAGACGTAGATACTCGAGCTTATTTTACTTCAGCAACTATAATTATTGCTGTTCCTACAGGAATTAAAATTTTTAGTTGATTAGCAACTTTACACGGAACTCAATTAACATATAGTCCAGCCCTTCTATGATCATTAGGATTTGTATTTTTATTTACAGTTGGAGGTTTAACAGGAGTAGTATTAGCTAATTCTTCAATTGATATTGTTCTTCATGATACTTATTATGTAGTTGCCCATTTTCATTATGTTTTATCTATAGGAGCTGTATTTGCTATTATAGCAGGATTTATTCATTGATACCCTTTATTAACAGGAATAGTTATAAACCCTTCATGATTAAAGGCTCAATTTAGTATAATATTTATTGGAGTAAATCTAACTTTCTTCCCCCAACATTTTTTAGGATTAGCTGGAATACCTCGACGATACTCGGATTTTCCCGATAGTTACTTAACTTGAAATATTATTTCTTCTTTAGGAAGAACAATTTCATTATTTGCCGTTATTTTCTTTTTATTTATTATTTGAGAAAGTATAATTACTCAACGAACACCTTCTTTC

>Australia-COXI-H06

TTCTGATTCTTTGGACACCCAGAAGTTTATATTTTAATTTTACCCGGATTTGGAATAATTTCTCATATTATTACTCAAGAAAGTGGAAAAAAGGAAACATTTGGAACTTTAGGAATAATTTATGCTATATTAACAATTGGATTATTGGGATTTATTGTTTGAGCTCATCATATATTTACAGTAGGTATAGACGTAGATACTCGAGCTTATTTTACTTCAGCAACTATAATTATTGCTGTTCCTACAGGAATTAAAATTTTTAGTTGATTAGCAACTTTACACGGAACTCAATTAACATATAGTCCAGCCCTTCTATGATCATTAGGATTTGTATTTTTATTTACAGTTGGAGGTTTAACAGGAGTAGTATTAGCTAATTCTTCAATTGATATTGTTCTTCATGATACTTATTATGTAGTTGCCCATTTTCATTATGTTTTATCTATAGGAGCTGTATTTGCTATTATAGCAGGATTTATTCATTGATACCCTTTATTAACAGGAATAGTTATAAACCCTTCATGATTAAAGGCTCAATTTAGTATAATATTTATTGGAGTAAATCTAACTTTCTTCCCCCAACATTTTTTAGGATTAGCTGGAATACCTCGACGATACTCGGATTTTCCCGATAGTTACTTAACTTGAAATATTATTTCTTCTTTAGGAAGAACAATTTCATTATTTGCCGTTATTTTCTTTTTATTTATTATTTGAGAAAGTATAATTACTCAACGAACACCTTCTTTC

>Australia-COXI-H07

TTCTGATTCTTTGGACACCCAGAAGTTTATATTTTAATTTTACCCGGATTTGGAATAATTTCTCATATTATTACTCAAGAAAGTGGAAAAAAGGAAACATTTGGAACTTTAGGAATAATTTATGCTATATTAACAATTGGATTATTGGGATTTATTGTTTGAGCTCATCATATATTTACAGTAGGTATAGACGTAGATACTCGAGCTTATTTTACTTCAGCAACTATAATTATTGCTGTTCCTACAGGAATTAAAATTTTTAGTTGATTAGCAACTTTACACGGAACTCAATTAACATATAGTCCAGCCCTTCTATGATCATTAGGATTTGTATTTTTATTTACAGTTGGAGGTTTAACAGGAGTAGTATTAGCTAATTCTTCAATTGATATTGTTCTTCATGATACTTATTATGTAGTTGCCCATTTTCATTATGTTTTATCTATAGGAGCTGTATTTGCTATTATAGCAGGATTTATTCATTGATACCCTTTATTAACAGGAATAGTTATAAACCCTTCATGATTAAAGGCTCAATTTAGTATAATATTTATTGGAGTAAATCTAACTTTCTTCCCCCAACATTTTTTAGGATTAGCTGGAATACCTCGACGATACTCGGATTTTCCCGATAGTTACTTAACTTGAAATATTATTTCTTCTTTAGGAAGAACAATTTCATTATTTGCCGTTATTTTCTTTTTATTTATTATTTGAGAAAGTATAATTACTCAACGAACACCTTCTTTC

>Australia-COXI-H08

TTCTGATTCTTTGGACACCCAGAAGTTTATATTTTAATTTTACCCGGATTTGGAATAATTTCTCATATTATTACTCAAGAAAGTGGAAAAAAGGAAACATTTGGAACTTTAGGAATAATTTATGCTATATTAACAATTGGATTATTGGGATTTATTGTTTGAGCTCATCATATATTTACAGTAGGTATAGACGTAGATACTCGAGCTTATTTTACTTCAGCAACTATAATTATTGCTGTTCCTACAGGAATTAAAATTTTTAGTTGATTAGCAACTTTACACGGAACTCAATTAACATATAGTCCAGCCCTTCTATGATCATTAGGATTTGTATTTTTATTTACAGTTGGAGGTTTAACAGGAGTAGTATTAGCTAATTCTTCAATTGATATTGTTCTTCATGATACTTATTATGTAGTTGCCCATTTTCATTATGTTTTATCTATAGGAGCTGTATTTGCTATTATAGCAGGATTTATTCATTGATACCCTTTATTAACAGGAATAGTTATAAACCCTTCATGATTAAAGGCTCAATTTAGTATAATATTTATTGGAGTAAATCTAACTTTCTTTCCCCAACATTTTTTAGGATTAGCTGGAATACCTCGACGATACTCAGATTTTCCCGATAGTTACTTAACTTGAAATATTATTTCTTCTTTAGGAAGAACAATTTCATTATTTGCCGTTATTTTCTTTTTATTTATTATTTGAGAAAGTATAATTACTCAACGAACACCGTCTTTC

>Australia-COXI-H09

TTCTGATTCTTTGGACACCCAGAAGTTTATATTTTAATTTTACCCGGATTTGGAATAATTTCTCATATTATTACTCAAGAAAGTGGAAAAAAGGAAACATTTGGAACTTTAGGAATAATTTATGCTATATTAACAATTGGATTATTGGGATTTATTGTTTGAGCTCATCATATATTTACAGTAGGTATAGACGTAGATACTCGAGCTTATTTTACTTCAGCAACTATAATTATTGCTGTTCCTACAGGAATTAAAATTTTTAGTTGATTAGCAACTTTACACGGAACTCAATTAACATATAGTCCAGCCCTTCTATGATCATTAGGATTTGTATTTTTATTTACAGTTGGAGGTTTAACAGGAGTAGTATTAGCTAATTCTTCAATTGATATTGTTCTTCATGATACTTATTATGTAGTTGCCCATTTTCATTATGTTTTATCTATAGGAGCTGTATTTGCTATTATAGCAGGATTTATTCATTGATACCCTTTATTAACAGGAATAGTTATAAACCCTTCATGATTAAAGGCTCAATTTAGTATAATATTTATTGGAGTAAATCTAACTTTCTTCCCCCAACATTTTTTAGGATTAGCTGGAATACCTCGACGATACTCGGATTTTCCCGATAGTTACTTAACTTGAAATATTATTTCTTCTTTAGGAAGAACAATTTCATTATTTGCCGTTATTTTCTTTTTATTTATTATTTGGGAAAGTATAATTACTCAACGAACACCTTCTTTC

>Australia-COXI-H10

TTCTGATTCTTTGGACACCCAGAAGTTTATATTTTAATTTTACCCGGATTTGGAATAATTTCTCATATTATTACTCAAGAAAGTGGAAAAAAGGAAACATTTGGAACTTTAGGAATAATTTATGCTATATTAACAATTGGATTATTGGGATTTATTGTTTGAGCTCATCATATATTTACAGTAGGTATAGACGTAGATACTCGAGCTTATTTTACTTCAGCAACTATAATTATTGCTGTTCCTACAGGAATTAAAATTTTTAGTTGATTAGCAACTTTACACGGAACTCAATTAACATATAGTCCAGCCCTTCTATGATCATTAGGATTTGTATTTTTATTTACAGTTGGAGGTTTAACAGGAGTAGTATTAGCTAATTCTTCAATTGATATTGTTCTTCATGATACTTATTATGTAGTTGCCCATTTTCATTATGTTTTATCTATAGGAGCTGTATTTGCTATTATAGCAGGATTTATTCATTGATACCCTTTATTAACAGGAATAGTTATAAACCCTTCATGATTAAAGGCTCAATTTAGTATAATATTTATTGGAGTAAATCTAACTTTCTTCCCCCAACATTTTTTAGGATTAGCTGGAATACCTCGACGATACTCGGATTTTCCCGATAGTTACTTAACTTGAAATATTATTTCTTCTTTAGGAAGAACAATTTCATTATTTGCCGTTATTTTCTTTTTATTTATTATTTGAGAAAGTATAATTACTCAACGAACACCTTCTTTC

>Australia-COXI-H11

TTCTGATTCTTTGGACACCCAGAAGTTTATATTTTAATTTTACCCGGATTTGGAATAATTTCTCATATTATTACTCAAGAAAGTGGAAAAAAGGAAACATTTGGAACTTTAGGAATAATTTATGCTATATTAACAATTGGATTATTGGGATTTATTGTTTGAGCTCATCATATATTTACAGTAGGTATAGACGTAGATACTCGAGCTTATTTTACTTCAGCAACTATAATTATTGCTGTTCCTACAGGAATTAAAATTTTTAGTTGATTAGCAACTTTACACGGAACTCAATTAACATATAGTCCAGCCCTTCTATGATCATTAGGATTTGTATTTTTATTTACAGTTGGAGGTTTAACAGGAGTAGTATTAGCTAATTCTTCAATTGATATTGTTCTTCATGATACTTATTATGTAGTTGCCCATTTTCATTATGTTTTATCTATAGGAGCTGTATTTGCTATTATAGCAGGATTTATTCATTGATACCCTTTATTAACAGGAATAGTTATAAACCCTTCATGATTAAAGGCTCAATTTAGTATAATATTTATTGGAGTAAATCTAACTTTCTTTCCCCAACATTTTTTAGGATTAGCTGGAATACCTCGACGATACTCAGATTTTCCCGATAGTTACTTAACTTGAAATATTATTTCTTCTTTAGGAAGAACAATTTCATTATTTGCCGTTATTTTCTTTTTATTTATTATTTGAGAAAGTATAATTACTCAACGAACACCGTCTTTC

>Indonesia-COXI-A01

TTCTGATTCTTTGGACACCCAGAAGTTTATATTTTAATTTTACCCGGATTTGGAATAATTTCTCATATTATTACTCAAGAAAGTGGAAAAAAGGAAACATTTGGAACTTTAGGAATAATTTATGCTATATTAACAATTGGATTATTGGGATTTATTGTTTGAGCTCATCATATATTTACAGTAGGTATAGACGTAGATACTCGAGCTTATTTTACTTCAGCAACTATAATTATTGCTGTTCCTACAGGAATTAAAATTTTTAGTTGATTAGCAACTTTACACGGAACTCAATTAACATATAGTCCAGCCCTTCTATGATCATTAGGATTTGTATTTTTATTTACAGTTGGAGGTTTAACAGGAGTAGTATTAGCTAATTCTTCAATTGATATTGTTCTTCATGATACTTATTATGTAGTTGCCCATTTTCATTATGTTTTATCTATAGGAGCTGTATTTGCTATTATAGCAGGATTTATTCATTGATACCCTTTATTAACAGGAATAGTTATAAACCCTTCATGATTAAAGGCTCAATTTAGTATAATATTTATTGGAGTAAATCTAACTTTCTTCCCCCAACATTTTTTAGGATTAGCTGGAATACCTCGACGATACTCAGATTTTCCCGATAGTTACTTAACTTGAAATATTATTTCTTCTTTAGGAAGAACAATTTCATTATTTGCCGTTATTTTCTTTTTATTTATTATTTGAGAAAGTATAATTACTCAACGAACACCTTCTTTC

>Indonesia-COXI-A02

TTCTGATTCTTTGGACACCCAGAAGTTTATATTTTAATTTTACCCGGATTTGGAATAATTTCTCATATTATTACTCAAGAAAGTGGAAAAAAGGAAACATTTGGAACTTTAGGAATAATTTATGCTATATTAACAATTGGATTATTGGGATTTATTGTTTGAGCTCATCATATATTTACAGTAGGTATAGACGTAGATACTCGAGCTTATTTTACTTCAGCAACTATAATTATTGCTGTTCCTACAGGAATTAAAATTTTTAGTTGATTAGCAACTTTACACGGAACTCAATTAACATATAGCCCAGCCCTTCTATGATCATTAGGATTTGTATTTTTATTTACAGTTGGAGGTTTAACAGGAGTAGTATTAGCTAATTCTTCAATTGATATTGTTCTTCATGATACTTATTATGTAGTTGCCCATTTTCATTATGTTTTATCTATAGGAGCTGTATTTGCTATTATAGCTGGATTTATTCATTGATACCCTTTATTAACAGGAATAGTTATAAACCCTTCATGATTAAAGGCTCAATTTAGTATAATATTTATTGGAGTAAATCTAACTTTCTTCCCCCAACATTTTTTAGGATTAGCTGGAATACCTCGACGATACTCAGATTTTCCCGATAGTTACTTAACTTGAAATATTATTTCTTCTTTAGGAAGAACAATTTCATTATTTGCCGTTATTTTCTTTTTATTTATTATTTGAGAAAGTATAATTACTCAACGAACACCTTCTTTC

>Indonesia-COXI-A03

TTCTGATTCTTTGGACACCCAGAAGTTTATATTTTAATTTTACCCGGATTTGGAATAATTTCTCATATTATTACTCAAGAAAGTGGAAAAAAGGAAACATTTGGAACTTTAGGAATAATTTATGCTATATTAACAATTGGATTATTGGGATTTATTGTTTGAGCTCATCATATATTTACAGTAGGTATAGACGTAGATACTCGAGCTTATTTTACTTCAGCAACTATAATTATTGCTGTTCCTACAGGAATTAAAATTTTTAGTTGATTAGCAACTTTACACGGAACTCAATTAACATATAGTCCAGCCCTTCTATGATCATTAGGATTTGTATTTTTATTTACAGTTGGAGGTTTAACAGGAGTAGTATTAGCTAATTCTTCAATTGATATTGTTCTTCATGATACTTATTATGTAGTTGCCCATTTTCATTATGTTTTATCTATAGGAGCTGTATTTGCTATTATAGCAGGATTTATTCATTGATACCCTTTATTAACAGGAATAGTTATAAACCCTTCATGATTAAAGGCTCAATTTAGTATAATATTTATTGGAGTAAATCTAACTTTCTTTCCCCAACATTTTTTAGGATTAGCTGGAATACCTCGACGATACTCAGATTTTCCCGATAGTTACTTAACTTGAAATATTATTTCTTCTTTAGGAAGAACAATTTCATTATTTGCCGTTATTTTCTTTTTATTTATTATTTGAGAAAGTATAATTACTCAACGAACACCTTCTTTC

>Indonesia-COXI-A04

TTCTGATTCTTTGGACACCCAGAAGTTTATATTTTAATTTTACCCGGATTTGGAATAATTTCTCATATTATTACTCAAGAAAGTGGAAAAAAGGAAACATTTGGAACTTTAGGAATAATTTATGCTATATTAACAATTGGATTATTGGGATTTATTGTTTGAGCTCATCATATATTTACAGTAGGTATAGACGTAGATACTCGAGCTTATTTTACTTCAGCAACTATAATTATTGCTGTTCCTACAGGAATTAAAATTTTTAGTTGATTAGCAACTTTACACGGAACTCAATTAACATATAGTCCAGCCCTTCTATGATCATTAGGATTTGTATTTTTATTTACAGTTGGAGGTTTAACAGGAGTAGTATTAGCTAATTCTTCAATTGATATTGTTCTTCATGATACTTATTATGTAGTTGCCCATTTTCATTATGTTTTATCTATAGGAGCTGTATTTGCTATTATAGCAGGATTTATTCATTGATACCCTTTATTAACAGGAATAGTTATAAACCCTTCATGATTAAAGGCTCAATTTAGTATAATATTTATTGGAGTAAATCTAACTTTCTTTCCCCAACATTTTTTAGGATTAGCTGGAATACCTCGACGATACTCAGATTTTCCCGATAGTTACTTAACTTGAAATATTATTTCTTCTTTAGGAAGAACAATTTCATTATTTGCCGTTATTTTCTTTTTATTTATTATTTGAGAAAGTATAATTACTCAACGAACACCTTCTTTC

>Indonesia-COXI-B01

TTCTGATTCTTTGGACACCCAGAAGTTTATATTTTAATTTTACCCGGATTTGGAATAATTTCTCATATTATTACTCAAGAAAGTGGAAAAAAGGAAACATTTGGAACTTTAGGAATAATTTATGCTATATTAACAATTGGATTATTGGGATTTATTGTTTGAGCTCATCATATATTTACAGTAGGTATAGACGTAGATACTCGAGCTTATTTTACTTCAGCAACTATAATTATTGCTGTTCCTACAGGAATTAAAATTTTTAGTTGATTAGCAACTTTACACGGAACTCAATTAACATATAGTCCAGCCCTTCTATGATCATTAGGATTTGTATTTTTATTTACAGTTGGAGGTTTAACAGGAGTAGTATTAGCTAATTCTTCAATTGATATTGTTCTTCATGATACTTATTATGTAGTTGCCCATTTTCATTATGTTTTATCTATAGGAGCTGTATTTGCTATTATAGCAGGATTTATTCATTGATACCCTTTATTAACAGGAATAGTTATAAACCCTTCATGATTAAAGGCTCAATTTAGTATAATATTTATTGGAGTAAATCTAACTTTCTTTCCCCAACATTTTTTAGGATTAGCTGGAATACCTCGACGATACTCAGATTTTCCCGATAGTTACTTAACTTGAAATATTATTTCTTCTTTAGGAAGAACAATTTCATTATTTGCCGTTATTTTCTTTTTATTTATTATTTGAGAAAGTATAATTACTCAACGAACACCTTCTTTC

>Indonesia-COXI-B02

TTCTGATTCTTTGGACACCCAGAAGTTTATATTTTAATTTTACCCGGATTTGGAATAATTTCTCATATTATTACTCAAGAAAGTGGAAAAAAGGAAACATTTGGAACTTTAGGAATAATTTATGCTATATTAACAATTGGATTATTGGGATTTATTGTTTGAGCTCATCATATATTTACAGTAGGTATAGACGTAGATACTCGAGCTTATTTTACTTCAGCAACTATAATTATTGCTGTTCCTACAGGAATTAAAATTTTTAGTTGATTAGCAACTTTACACGGAACTCAATTAACATATAGTCCAGCCCTTCTATGATCATTAGGATTTGTATTTTTATTTACAGTTGGAGGTTTAACAGGAGTAGTATTAGCTAATTCTTCAATTGATATTGTTCTTCATGATACTTATTATGTAGTTGCCCATTTTCATTATGTTTTATCTATAGGAGCTGTATTTGCTATTATAGCAGGATTTATTCATTGATACCCTTTATTAACAGGAATAGTTATAAACCCTTCATGATTAAAGGCTCAATTTAGTATAATATTTATTGGAGTAAATTTAACTTTCTTCCCCCAACATTTTTTAGGATTAGCTGGAATACCTCGACGATACTCAGATTTTCCCGATAGTTACTTAACTTGAAATATTATTTCTTCTTTAGGAAGAACAATTTCATTATTTGCCGTTATTTTCTTTTTATTTATTATTTGAGAAAGTATAATTACTCAACGAACACCTTCTTTC

>Indonesia-COXI-B03

TTCTGGTTCTTTGGACACCCAGAAGTTTATATTTTAATTTTACCCGGATTTGGAATAATTTCTCATATTATTACTCAAGAAAGTGGAAAAAAGGAAACATTTGGAACTTTAGGAATAATTTATGCTATATTAACAATTGGATTATTGGGATTTATTGTTTGAGCTCATCATATATTTACAGTAGGTATAGACGTAGATACTCGAGCTTATTTTACTTCAGCAACTATAATTATTGCTGTTCCTACAGGAATTAAAATTTTTAGTTGATTAGCAACTTTACACGGAACTCAATTAACATATAGTCCAGCCCTTCTATGATCATTAGGATTTGTATTTTTATTTACAGTTGGAGGTTTAACAGGAGTAGTATTAGCTAATTCTTCAATTGATATTGTTCTTCATGATACTTATTATGTAGTTGCCCATTTTCATTATGTTTTATCTATAGGAGCTGTATTTGCTATTATAGCAGGATTTATTCATTGATACCCTTTATTAACAGGAATAGTTATAAACCCTTCATGATTAAAGGCTCAATTTAGTATAATATTTATTGGAGTAAATCTAACTTTCTTTCCCCAACATTTTTTAGGATTAGCTGGAATACCTCGACGATACTCAGATTTTCCCGATAGTTACTTAACTTGAAATATTATTTCTTCTTTAGGAAGAACAATTTCATTATTTGCCGTTATTTTCTTTTTATTTATTATTTGAGAAAGTATAATTACTCAACGAACACCTTCTTTC

>Indonesia-COXI-B04

TTCTGATTCTTTGGACACCCAGAAGTTTATATTTTAATTTTACCCGGATTTGGAATAATTTCTCATATTATTACTCAAGAAAGTGGAAAAAAGGAAACATTTGGAACTTTAGGAATAATTTATGCTATATTAACAATTGGATTATTGGGATTTATTGTTTGAGCTCATCATATATTTACAGTAGGTATAGACGTAGATACTCGAGCTTATTTTACTTCAGCAACTATAATTATTGCTGTTCCTACAGGAATTAAAATTTTTAGTTGATTAGCAACTTTACACGGAACTCAATTAACATATAGTCCAGCCCTTCTATGATCATTAGGATTTGTATTTTTATTTACAGTTGGAGGTTTAACAGGAGTAGTATTAGCTAATTCTTCAATTGATATTGTTCTTCATGATACTTATTATGTAGTTGCCCATTTTCATTATGTTTTATCTATAGGAGCTGTATTTGCTATTATAGCAGGATTTATTCATTGATACCCTTTATTAACAGGAATAGTTATAAACCCTTCATGATTAAAGGCTCAATTTAGTATAATATTTATTGGAGTAAATCTAACTTTCTTTCCCCAACATTTTTTAGGATTAGCTGGAATACCTCGACGATACTCAGATTTTCCCGATAGTTACTTAACTTGAAATATTATTTCTTCTTTAGGAAGAACAATTTCATTATTTGCCGTTATTTTCTTTTTATTTATTATTTGAGAAAGTATAATTACTCAACGAACACCTTCTTTC

>Indonesia-COXI-C01

TTCTGATTCTTTGGACACCCAGAAGTTTATATTTTAATTTTACCCGGATTTGGAATAATTTCTCATATTATTACTCAAGAAAGTGGAAAAAAGGAAACATTTGGAACTTTAGGAATAATTTATGCTATATTAACAATTGGATTATTGGGATTTATTGTTTGAGCTCATCATATATTTACAGTAGGTATAGACGTAGATACTCGAGCTTATTTTACTTCAGCAACTATAATTATTGCTGTTCCTACAGGAATTAAAATTTTTAGTTGATTAGCAACTTTACACGGAACTCAATTAACATATAGTCCAGCCCTTCTATGATCATTAGGATTTGTATTTTTATTTACAGTTGGAGGTTTAACAGGAGTAGTATTAGCTAATTCTTCAATTGATATTGTTCTTCATGATACTTATTATGTAGTTGCCCATTTTCATTATGTTTTATCTATAGGAGCTGTATTTGCTATTATAGCAGGATTTATTCATTGATACCCTTTATTAACAGGAATAGTTATAAACCCTTCATGATTAAAGGCTCAATTTAGTATAATATTTATTGGAGTAAATCTAACTTTCTTTCCCCAACATTTTTTAGGATTAGCTGGAATACCTCGACGATACTCAGATTTTCCCGATAGTTATTTAACTTGAAATATTATTTCTTCTTTAGGAAGAACAATTTCATTATTTGCCGTTATTTTCTTTTTATTTATTATTTGAGAAAGTATAATTACTCAACGAACACCTTCTTTC

>Indonesia-COXI-C02

TTCTGATTCTTTGGACACCCAGAAGTTTATATTTTAATTTTACCCGGATTTGGAATAATTTCTCATATTATTACTCAAGAAAGTGGAAAAAAGGAAACATTTGGAACTTTAGGAATAATTTATGCTATATTAACAATTGGATTATTGGGATTTATTGTTTGAGCTCATCATATATTTACAGTAGGTATAGACGTAGATACTCGAGCTTATTTTACTTCAGCAACTATAATTATTGCTGTTCCTACAGGAATTAAAATTTTTAGTTGATTAGCAACTTTACACGGAACTCAATTAACATATAGTCCAGCCCTTCTATGATCATTAGGATTTGTATTTTTATTTACAGTTGGAGGTTTAACAGGAGTAGTATTAGCTAATTCTTCAATTGATATTGTTCTTCATGATACTTATTATGTAGTTGCCCATTTTCATTATGTTTTATCTATAGGAGCTGTATTTGCTATTATAGCAGGATTTATTCATTGATACCCTTTATTAACAGGAATAGTTATAAACCCTTCATGATTAAAGGCTCAATTTAGTATAATATTTATTGGAGTAAATCTAACTTTCTTTCCCCAACATTTTTTAGGATTAGCTGGAATACCTCGACGATACTCAGATTTTCCCGATAGTTACTTAACTTGAAATATTATTTCTTCTTTAGGAAGAACAATTTCATTATTTGCCGTTATTTTCTTTTTATTTATTATTTGAGAAAGTATAATTACTCAACGAACACCTTCTTTC

>Indonesia-COXI-C03

TTCTGATTCTTTGGACACCCAGAAGTTTATATTTTAATTTTACCCGGATTTGGAATAATTTCTCATATTATTACTCAAGAAAGTGGAAAAAAGGAAACATTTGGAACTTTAGGAATAATTTATGCTATATTAACAATTGGATTATTGGGATTTATTGTTTGAGCTCATCATATATTTACAGTAGGTATAGACGTAGATACTCGAGCTTATTTTACTTCAGCAACTATAATTATTGCTGTTCCTACAGGAATTAAAATTTTTAGTTGATTAGCAACTTTACACGGAACTCAATTAACATATAGTCCAGCCCTTCTATGATCATTAGGATTTGTATTTTTATTTACAGTTGGAGGTTTAACAGGAGTAGTATTAGCTAATTCTTCAATTGATATTGTTCTTCATGATACTTATTATGTAGTTGCCCATTTTCATTATGTTTTATCTATAGGAGCTGTATTTGCTATTATAGCAGGATTTATTCATTGATACCCTTTATTAACAGGAATAGTTATAAACCCTTCATGATTAAAGGCTCAATTTAGTATAATATTTATTGGAGTAAATCTAACTTTCTTTCCCCAACATTTTTTAGGATTAGCTGGAATACCTCGACGATACTCAGATTTTCCCGATAGTTACTTAACTTGAAATATTATTTCTTCTTTAGGAAGAACAATTTCATTATTTGCCATTATTTTCTTTTTATTTATTATTTGAGAAAGTATAATTACTCAACGAACACCTTCTTTC

>Indonesia-COXI-C04

TTCTGATTCTTTGGACACCCAGAAGTTTATATTTTAATTTTACCCGGATTTGGAATAATTTCTCATATTATTACTCAAGAAAGTGGAAAAAAGGAAACATTTGGAACTTTAGGAATAATTTATGCTATATTAACAATTGGATTATTGGGATTTATTGTTTGAGCTCATCATATATTTACAGTAGGTATAGACGTAGATACTCGAGCTTATTTTACTTCAGCAACTATAATTATTGCTGTTCCTACAGGAATTAAAATTTTTAGTTGATTAGCAACTTTACACGGAACTCAATTAACATATAGTCCAGCCCTTCTATGATCATTAGGATTTGTATTTTTATTTACAGTTGGAGGTTTAACAGGAGTAGTATTAGCTAATTCTTCAATTGATATTGTTCTTCATGATACTTATTATGTAGTTGCCCATTTTCATTATGTTTTATCTATAGGAGCTGTATTTGCTATTATAGCAGGATTTATTCATTGATACCCTTTATTAACAGGAATAGTTATAAACCCTTCATGATTAAAGGCTCAATTTAGTATAATATTTATTGGAGTAAATCTAACTTTCTTCCCCCAACATTTTTTAGGATTAGCTGGAATACCTCGACGATACTCAGATTTTCCCGATAGTTACTTAACTTGAAATATTATTTCTTCTTTAGGAAGAACAATTTCATTATTTGCCGTTATTTTCTTTTTATTTATTATTTGAGAAAGTATAATTACTCAACGAACACCTTCTTTC

>Indonesia-COXI-D01

TTCTGATTCTTTGGACACCCAGAAGTTTATATTTTAATTTTACCCGGATTTGGAATAATTTCTCATATTATTACTCAAGAAAGTGGAAAAAAGGAAACATTTGGAACTTTAGGAATAATTTATGCTATATTAACAATTGGATTATTGGGATTTATTGTTTGAGCTCATCATATATTTACAGTAGGTATAGACGTAGATACTCGAGCTTATTTTACTTCAGCAACTATAATTATTGCTGTTCCTACAGGAATTAAAATTTTTAGTTGATTAGCAACTTTACACGGAACTCAATTAACATATAGTCCAGCCCTTCTATGATCATTAGGATTTGTATTTTTATTTACAGTTGGAGGTTTAACAGGAGTAGTATTAGCTAATTCTTCAATTGATATTGTTCTTCATGATACTTATTATGTAGTTGCCCATTTTCATTATGTTTTATCTATAGGAGCTGTATTTGCTATTATAGCAGGATTTATTCATTGATACCCTTTATTAACAGGAATAGTTATAAACCCTTCATGATTAAAGGCTCAATTTAGTATAATATTTATTGGAGTAAATCTAACTTTCTTTCCCCAACATTTTTTAGGATTAGCTGGAATACCTCGACGATACTCAGATTTTCCCGATAGTTACTTAACTTGAAATATTATTTCTTCTTTAGGAAGAACAATTTCATTATTTGCCGTTATTTTCTTTTTATTTATTATTTGAGAAAGTATAATTACTCAACGAACACCTTCTTTC

>Indonesia-COXI-D02

TTCTGATTCTTTGGACACCCAGAAGTTTATATTTTAATTTTACCCGGATTTGGAATAATTTCTCATATTATTACTCAAGAAAGTGGAAAAAAGGAAACATTTGGAACTTTAGGAATAATTTATGCTATATTAACAATTGGATTATTGGGATTTATTGTTTGAGCTCATCATATATTTACAGTAGGTATAGACGTAGATACTCGAGCTTATTTTACTTCAGCAACTATAATTATTGCTGTTCCTACAGGAATTAAAATTTTTAGTTGATTAGCAACTTTACACGGAACTCAATTAACATATAGTCCAGCCCTTCTATGATCATTAGGATTTGTATTTTTATTTACAGTTGGAGGTTTAACAGGAGTAGTATTAGCTAATTCTTCAATTGATATTGTTCTTCATGATACTTATTATGTAGTTGCCCATTTTCATTATGTTTTATCTATAGGAGCTGTATTTGCTATTATAGCAGGATTTATTCATTGATACCCTTTATTAACAGGAATAGTTATAAACCCTTCATGATTAAAGGCTCAATTTAGTATAATATTTATTGGAGTAAATCTAACTTTCTTTCCCCAACATTTTTTAGGATTAGCTGGAATACCTCGACGATACTCAGATTTTCCCGATAGTTACTTAACTTGAAATATTATTTCTTCTTTAGGAAGAACAATTTCATTATTTGCCGTTATTTTCTTTTTATTTATTATTTGAGAAAGTATAATTACTCAACGAACACCTTCTTTC

>Indonesia-COXI-D03

TTCTGATTCTTTGGACACCCAGAAGTTTATATTTTAATTTTACCCGGATTTGGAATAATTTCTCATATTATTACTCAAGAAAGTGGAAAAAAGGAAACATTTGGAACTTTAGGAATAATTTATGCTATATTAACAATTGGATTATTGGGATTTATTGTTTGAGCTCATCATATATTTACAGTAGGTATAGACGTAGATACTCGAGCTTATTTTACTTCAGCAACTATAATTATTGCTGTTCCTACAGGAATTAAAATTTTTAGTTGATTAGCAACTTTACACGGAACTCAATTAACATATAGTCCAGCCCTTCTATGATCATTAGGATTTGTATTTTTATTTACAGTTGGAGGTTTAACAGGAGTAGTATTAGCTAATTCTTCAATTGATATTGTTCTTCATGATACTTATTATGTAGTTGCCCATTTTCATTATGTTTTATCTATAGGAGCTGTATTTGCTATTATAGCAGGATTTATTCATTGATACCCTTTATTAACAGGAATAGTTATAAACCCTTCATGATTAAAGGCTCAATTTAGTATAATATTTATTGGAGTAAATCTAACTTTCTTCCCCCAACATTTTTTAGGATTAGCTGGAATACCTCGACGATACTCAGATTTTCCCGATAGTTACTTAACTTGAAATATTATTTCTTCTTTAGGAAGAACAATTTCATTATTTGCCGTTATTTTCTTTTTATTTATTATTTGAGAAAGTATAATTACTCAACGAACACCTTCTTTC

>Indonesia-COXI-D04

TTCTGATTCTTTGGACACCCAGAAGTTTATATTTTAATTTTACCCGGATTTGGAATAATTTCTCATATTATTACTCAAGAAAGTGGAAAAAAGGAAACATTTGGAACTTTAGGAATAATTTATGCTATATTAACAATTGGATTATTGGGATTTATTGTTTGAGCTCATCATATATTTACAGTAGGTATAGACGTAGATACTCGAGCTTATTTTACTTCAGCAACTATAATTATTGCTGTTCCTACAGGAATTAAAATTTTTAGTTGATTAGCAACTTTACACGGAACTCAATTAACATATAGCCCAGCCCTTCTATGATCATTAGGATTTGTATTTTTATTTACAGTTGGAGGTTTAACAGGAGTAGTATTAGCTAATTCTTCAATTGATATTGTTCTTCATGATACTTATTATGTAGTTGCCCATTTTCATTATGTTTTATCTATAGGAGCTGTATTTGCTATTATAGCTGGATTTATTCATTGATACCCTTTATTAACAGGAATAGTTATAAACCCTTCATGATTAAAGGCTCAATTTAGTATAATATTTATTGGAGTAAATCTAACTTTCTTCCCCCAACATTTTTTAGGATTAGCTGGAATACCTCGACGATACTCAGATTTTCCCGATAGTTACTTAACTTGAAATATTATTTCTTCTTTAGGAAGAACAATTTCATTATTTGCCGTTATTTTCTTTTTATTTATTATTTGAGAAAGTATAATTACTCAACGAACACCTTCTTTC

>Indonesia-COXI-E01

TTCTGATTCTTTGGACACCCAGAAGTTTATATTTTAATTTTACCCGGATTTGGAATAATTTCTCATATTATTACTCAAGAAAGTGGAAAAAAGGAAACATTTGGAACTTTAGGAATAATTTATGCTATATTAACAATTGGATTATTGGGATTTATTGTTTGAGCTCATCATATATTTACAGTAGGTATAGACGTAGATACTCGAGCTTATTTTACTTCAGCAACTATAATTATTGCTGTTCCTACAGGAATTAAAATTTTTAGTTGATTAGCAACTTTACACGGAACTCAATTAACATATAGTCCAGCCCTTCTATGATCATTAGGATTTGTATTTTTATTTACAGTTGGAGGTTTAACAGGAGTAGTATTAGCTAATTCTTCAATTGATATTGTTCTTCATGATACTTATTATGTAGTTGCCCATTTTCATTATGTTTTATCTATAGGAGCTGTATTTGCTATTATAGCAGGATTTATTCATTGATACCCTTTATTAACAGGAATAGTTATAAACCCTTCATGATTAAAGGCTCAATTTAGTATAATATTTATTGGAGTAAATCTAACTTTCTTTCCCCAACATTTTTTAGGATTAGCTGGAATACCTCGACGATACTCAGATTTTCCCGATAGTTACTTAACTTGAAATATTATTTCTTCTTTAGGAAGAACAATTTCATTATTTGCCGTTATTTTCTTTTTATTTATTATTTGAGAAAGTATAATTACTCAACGAACACCTTCTTTC

>Indonesia-COXI-E02

TTTTGATTCTTTGGACACCCAGAAGTTTATATTTTAATTTTACCCGGATTTGGAATAATTTCTCATATTATTACTCAAGAAAGTGGAAAAAAGGAAACATTTGGAACTTTAGGAATAATTTATGCTATATTAACAATTGGATTATTGGGATTTATTGTTTGAGCTCATCATATATTTACAGTAGGTATAGATGTAGATACTCGAGCTTATTTTACTTCAGCAACTATAATTATTGCTGTTCCTACAGGAATTAAAATTTTTAGTTGATTAGCAACTTTACACGGAACTCAATTAACATATAGTCCAGCCCTTCTATGATCATTAGGATTTGTATTTTTATTTACAGTTGGAGGTTTAACAGGAGTAGTATTAGCTAATTCTTCAATTGATATTGTTCTTCATGATACTTATTACGTAGTTGCCCACTTTCATTACGTTTTATCTATAGGAGCTGTATTTGCTATTATAGCAGGATTTATTCATTGATACCCTTTATTAACAGGAATAGTTATAAACCCTTCATGATTAAAGGCTCAATTTAGTATAATATTTATTGGAGTAAATCTAACTTTCTTTCCTCAACATTTTTTAGGATTAGCTGGAATACCTCGACGATACTCAGATTTTCCTGATAGTTACTTAACTTGAAATATTATTTCTTCTTTAGGAAGAACAATTTCACTATTTGCCGTTATTTTCTTTTTATTTATTATTTGAGAAAGTATAATTACTCAACGAACACCTTCTTTC

>Indonesia-COXI-E03

TTCTGATTCTTTGGACACCCAGAAGTTTATATTTTAATTTTACCCGGATTTGGAATAATTTCTCATATTATTACTCAAGAAAGTGGAAAAAAGGAAACATTTGGAACTTTAGGAATAATTTATGCTATATTAACAATTGGATTATTGGGATTTATTGTTTGAGCTCATCATATATTTACAGTAGGTATAGACGTAGATACTCGAGCTTATTTTACTTCAGCAACTATAATTATTGCTGTTCCTACAGGAATTAAAATTTTTAGTTGATTAGCAACTTTACACGGAACTCAATTAACATATAGTCCAGCCCTTCTATGATCATTAGGATTTGTATTTTTATTTACAGTTGGAGGTTTAACAGGAGTAGTATTAGCTAATTCTTCAATTGATATTGTTCTTCATGATACTTATTATGTAGTTGCCCATTTTCATTATGTTTTATCTATAGGAGCTGTATTTGCTATTATAGCAGGATTTATTCATTGATACCCTTTATTAACAGGAATAGTTATAAACCCTTCATGATTAAAGGCTCAATTTAGTATAATATTTATTGGAGTAAATCTAACTTTCTTCCCCCAACATTTTTTAGGATTAGCTGGAATACCTCGACGATACTCAGATTTTCCCGATAGTTACTTAACTTGAAATATTATTTCTTCTTTAGGAAGAACAATTTCATTATTTGCCGTTATTTTCTTTTTATTTATTATTTGAGAAAGTATAATTACTCAACGAACACCTTCTTTC

>Indonesia-COXI-E04

TTCTGATTCTTTGGACACCCAGAAGTTTATATTTTAATTTTACCCGGATTTGGAATAATTTCTCATATTATTACTCAAGAAAGTGGAAAAAAGGAAACATTTGGAACTTTAGGAATAATTTATGCTATATTAACAATTGGATTATTGGGATTTATTGTTTGAGCTCATCATATATTTACAGTAGGTATAGACGTAGATACTCGAGCTTATTTTACTTCAGCAACTATAATTATTGCTGTTCCTACAGGAATTAAAATTTTTAGTTGATTAGCAACTTTACACGGAACTCAATTAACATATAGCCCAGCCCTTCTATGATCATTAGGATTTGTATTTTTATTTACAGTTGGAGGTTTAACAGGAGTAGTATTAGCTAATTCTTCAATTGATATTGTTCTTCATGATACTTATTATGTAGTTGCCCATTTTCATTATGTTTTATCTATAGGAGCTGTATTTGCTATTATAGCTGGATTTATTCATTGATACCCTTTATTAACAGGAATAGTTATAAACCCTTCATGATTAAAGGCTCAATTTAGTATAATATTTATTGGAGTAAATCTAACTTTCTTCCCCCAACATTTTTTAGGATTAGCTGGAATACCTCGACGATACTCAGATTTTCCCGATAGTTACTTAACTTGAAATATTATTTCTTCTTTAGGAAGAACAATTTCATTATTTGCCGTTATTTTCTTTTTATTTATTATTTGAGAAAGTATAATTACTCAACGAACACCTTCTTTC

>Indonesia-COXI-F01

TTCTGATTCTTTGGACACCCAGAAGTTTATATTTTAATTTTACCCGGATTTGGAATAATTTCTCATATTATTACTCAAGAAAGTGGAAAAAAGGAAACATTTGGAACTTTAGGAATAATTTATGCTATATTAACAATTGGATTATTGGGATTTATTGTTTGAGCTCATCATATATTTACAGTAGGTATAGACGTAGATACTCGAGCTTATTTTACTTCAGCAACTATAATTATTGCTGTTCCTACAGGAATTAAAATTTTTAGTTGATTAGCAACTTTACACGGAACTCAATTAACATATAGTCCAGCCCTTCTATGATCATTAGGATTTGTATTTTTATTTACAGTTGGAGGTTTAACAGGAGTAGTATTAGCTAATTCTTCAATTGATATTGTTCTTCATGATACTTATTATGTAGTTGCCCATTTTCATTATGTTTTATCTATAGGAGCTGTATTTGCTATTATAGCAGGATTTATTCATTGATACCCTTTATTAACAGGAATAGTTATAAACCCTTCATGATTAAAGGCTCAATTTAGTATAATATTTATTGGAGTAAATCTAACTTTCTTTCCCCAACATTTTTTAGGATTAGCTGGAATACCTCGACGATACTCAGATTTTCCCGATAGTTACTTAACTTGAAATATTATTTCTTCTTTAGGAAGAACAATTTCATTATTTGCCGTTATTTTCTTTTTATTTATTATTTGAGAAAGTATAATTACTCAACGAACACCTTCTTTC

>Indonesia-COXI-F02

TTCTGATTCTTTGGACACCCAGAAGTTTATATTTTAATTTTACCCGGATTTGGAATAATTTCTCATATTATTACTCAAGAAAGTGGAAAAAAGGAAACATTTGGAACTTTAGGAATAATTTATGCTATATTAACAATTGGATTATTGGGATTTATTGTTTGAGCTCATCATATATTTACAGTAGGTATAGACGTAGATACTCGAGCTTATTTTACTTCAGCAACTATAATTATTGCTGTTCCTACAGGAATTAAAATTTTTAGTTGATTAGCAACTTTACACGGAACTCAATTAACATATAGTCCAGCCCTTCTATGATCATTAGGATTTGTATTTTTATTTACAGTTGGAGGTTTAACAGGAGTAGTATTAGCTAATTCTTCAATTGATATTGTTCTTCATGATACTTATTATGTAGTTGCCCATTTTCATTATGTTTTATCTATAGGAGCTGTATTTGCTATTATAGCAGGATTTATTCATTGATACCCTTTATTAACAGGAATAGTTATAAACCCTTCATGATTAAAGGCTCAATTTAGTATAATATTTATTGGAGTAAATCTAACTTTCTTTCCCCAACATTTTTTAGGATTAGCTGGAATACCTCGACGATACTCAGATTTTCCCGATAGTTACTTAACTTGAAATATTATTTCTTCTTTAGGAAGAACAATTTCATTATTTGCCGTTATTTTCTTTTTATTTATTATTTGAGAAAGTATAATTACTCAACGAACACCTTCTTTC

>Indonesia-COXI-F03

TTCTGATTCTTTGGACACCCAGAAGTTTATATTTTAATTTTACCCGGATTTGGAATAATTTCTCATATTATTACTCAAGAAAGTGGAAAAAAGGAAACATTTGGAACTTTAGGAATAATTTATGCTATATTAACAATTGGATTATTAGGATTTATTGTTTGAGCTCATCATATATTTACAGTAGGTATAGACGTAGATACTCGAGCTTATTTTACTTCAGCAACTATAATTATTGCTGTTCCTACAGGAATTAAAATTTTTAGTTGATTAGCAACTTTACACGGAACTCAATTAACATATAGTCCAGCCCTTCTATGATCATTAGGATTTGTATTTTTATTTACAGTTGGAGGTTTAACAGGAGTAGTATTAGCTAATTCTTCAATTGATATTGTTCTTCATGATACTTATTATGTAGTTGCCCATTTTCATTATGTTTTATCTATAGGAGCTGTATTTGCTATTATAGCAGGATTTATTCATTGATACCCTTTATTAACAGGAATAGTTATAAACCCTTCATGATTAAAGGCTCAATTTAGTATAATATTTATTGGAGTAAATCTAACTTTCTTTCCCCAACATTTTTTAGGATTAGCTGGGATACCTCGACGATACTCAGATTTTCCCGATAGTTACTTAACTTGAAATATTATTTCTTCTTTAGGAAGAACAATTTCATTATTTGCCGTTATTTTCTTTTTATTTATTATTTGAGAAAGTATAATTACTCAACGAACACCTTCTTTC

>Indonesia-COXI-F04

TTCTGATTCTTTGGACACCCAGAAGTTTATATTTTAATTTTACCCGGATTTGGAATAATTTCTCATATTATTACTCAAGAAAGTGGAAAAAAGGAAACATTTGGAACTTTAGGAATAATTTATGCTATATTAACAATTGGATTATTGGGATTTATTGTTTGAGCTCATCATATATTTACAGTAGGTATAGACGTAGATACTCGAGCTTATTTTACTTCAGCAACTATAATTATTGCTGTTCCTACAGGAATTAAAATTTTTAGTTGATTAGCAACTTTACACGGAACTCAATTAACATATAGTCCAGCCCTTCTATGATCATTAGGATTTGTATTTTTATTTACAGTTGGAGGTTTAACAGGAGTAGTATTAGCTAATTCTTCAATTGATATTGTTCTTCATGATACTTATTATGTAGTTGCCCATTTTCATTATGTTTTATCTATAGGAGCTGTATTTGCTATTATAGCAGGATTTATTCATTGATACCCTTTATTAACAGGAATAGTTATAAACCCTTCATGATTAAAGGCTCAATTTAGTATAATATTTATTGGAGTAAATCTAACTTTCTTTCCCCAACATTTTTTAGGATTAGCTGGAATACCTCGACGATACTCAGATTTTCCCGATAGTTACTTAACTTGAAATATTATTTCTTCTTTAGGAAGAACAATTTCATTATTTGCCGTTATTTTCTTTTTATTTATTATTTGAGAAAGTATAATTACTCAACGAACACCTTCTTTC

>Indonesia-COXI-G01

TTCTGATTCTTTGGACACCCAGAAGTTTATATTTTAATTTTACCCGGATTTGGAATAATTTCTCATATTATTACTCAAGAAAGTGGAAAAAAGGAAACATTTGGAACTTTAGGAATAATTTATGCTATATTAACAATTGGATTATTGGGATTTATTGTTTGAGCTCATCATATATTTACAGTAGGTATAGACGTAGATACTCGAGCTTATTTTACTTCAGCAACTATAATTATTGCTGTTCCTACAGGAATTAAAATTTTTAGTTGATTAGCAACTTTACACGGAACTCAATTAACATATAGTCCAGCCCTTCTATGATCATTAGGATTTGTATTTTTATTTACAGTTGGAGGTTTAACAGGAGTAGTATTAGCTAATTCTTCAATTGATATTGTTCTTCATGATACTTATTATGTAGTTGCCCATTTTCATTATGTTTTATCTATAGGAGCTGTATTTGCTATTATAGCAGGATTTATTCATTGATACCCTTTATTAACAGGAATAGTTATAAACCCTTCATGATTAAAGGCTCAATTTAGTATAATATTTATTGGAGTAAATCTAACTTTCTTTCCCCAACATTTTTTAGGATTAGCTGGAATACCTCGACGATACTCAGATTTTCCCGATAGTTACTTAACTTGAAATATTATTTCTTCTTTAGGAAGAACAATTTCATTATTTGCCGTTATTTTCTTTTTATTTATTATTTGAGAAAGTATAATTACTCAACGAACACCTTCTTTC

>Indonesia-COXI-G02

TTCTGATTCTTTGGACACCCAGAAGTTTATATTTTAATTTTACCCGGATTTGGAATAATTTCTCATATTATTACTCAAGAAAGTGGAAAAAAGGAAACATTTGGAACTTTAGGAATAATTTATGCTATATTAACAATTGGATTATTGGGATTTATTGTTTGAGCTCATCATATATTTACAGTAGGTATAGACGTAGATACTCGAGCTTATTTTACTTCAGCAACTATAATTATTGCTGTTCCTACAGGAATTAAAATTTTTAGTTGATTAGCAACTTTACACGGAACTCAATTAACATATAGTCCAGCCCTTCTATGATCATTAGGATTTGTATTTTTATTTACAGTTGGAGGTTTAACAGGAGTAGTATTAGCTAATTCTTCAATTGATATTGTTCTTCATGATACTTATTATGTAGTTGCCCATTTTCATTATGTTTTATCTATAGGAGCTGTATTTGCTATTATAGCAGGATTTATTCATTGATACCCTTTATTAACAGGAATAGTTATAAACCCTTCATGATTAAAGGCTCAATTTAGTATAATATTTATTGGAGTAAATCTAACTTTCTTTCCCCAACATTTTTTAGGATTAGCTGGAATACCTCGACGATACTCAGATTTTCCCGATAGTTACTTAACTTGAAATATTATTTCTTCTTTAGGAAGAACAATTTCATTATTTGCCGTTATTTTCTTTTTATTTATTATTTGAGAAAGTATAATTACTCAACGAACACCTTCTTTC

>Indonesia-COXI-G03

TTCTGATTCTTTGGACACCCAGAAGTTTATATTTTAATTTTACCCGGATTTGGAATAATTTCTCATATTATTACTCAAGAAAGTGGAAAAAAGGAAACATTTGGAACTTTAGGAATAATTTATGCTATATTAACAATTGGATTATTGGGATTTATTGTTTGAGCTCATCATATATTTACAGTAGGTATAGACGTAGATACTCGAGCTTATTTTACTTCAGCAACTATAATTATTGCTGTTCCTACAGGAATTAAAATTTTTAGTTGATTAGCAACTTTACACGGAACTCAATTAACATATAGTCCAGCCCTTCTATGATCATTAGGATTTGTATTTTTATTTACAGTTGGAGGTTTAACAGGAGTAGTATTAGCTAATTCTTCAATTGATATTGTTCTTCATGATACTTATTATGTAGTTGCCCATTTTCATTATGTTTTATCTATAGGAGCTGTATTTGCTATTATAGCAGGATTTATTCATTGATACCCTTTATTAACAGGAATAGTTATAAACCCTTCATGATTAAAGGCTCAATTTAGTATAATATTTATTGGAGTAAATCTAACTTTCTTTCCCCAACATTTTTTAGGATTAGCTGGAATACCTCGACGATACTCAGATTTTCCCGATAGTTACTTAACTTGAAATATTATTTCTTCTTTAGGAAGAACAATTTCATTATTTGCCGTTATTTTCTTTTTATTTATTATTTGAGAAAGTATAATTACTCAACGAACACCTTCTTTC

>Indonesia-COXI-G04

TTCTGATTCTTTGGACACCCAGAAGTTTATATTTTAATTTTACCCGGATTTGGAATAATTTCTCATATTATTACTCAAGAAAGTGGAAAAAAGGAAACATTTGGAACTTTAGGAATAATTTATGCTATATTAACAATTGGATTATTGGGATTTATTGTTTGAGCTCATCATATATTTACAGTAGGTATAGACGTAGATACTCGAGCTTATTTTACTTCAGCAACTATAATTATTGCTGTTCCTACAGGAATTAAAATTTTTAGTTGATTAGCAACTTTACACGGAACTCAATTAACATATAGTCCAGCCCTTCTATGATCATTAGGATTTGTATTTTTATTTACAGTTGGAGGTTTAACAGGAGTAGTATTAGCTAATTCTTCAATTGATATTGTTCTTCATGATACTTATTATGTAGTTGCCCATTTTCATTATGTTTTATCAATAGGAGCTGTATTTGCTATTATAGCAGGATTTATTCATTGATACCCTTTATTAACAGGAATAGTTATAAACCCTTCATGATTAAAGGCTCAATTTAGTATAATATTTATTGGAGTAAATCTAACTTTCTTTCCCCAACATTTTTTAGGATTAGCTGGAATACCTCGACGATACTCAGATTTTCCCGATAGTTACTTAACTTGAAATATTATTTCTTCTTTAGGAAGAACAATTTCATTATTTGCCGTTATTTTCTTTTTATTTATTATTTGAGAAAGTATAATTACTCAACGAACACCTTCTTTC

>Indonesia-COXI-H01

TTCTGATTCTTTGGACACCCAGAAGTTTATATTTTAATTTTACCCGGATTTGGAATAATTTCTCATATTATTACTCAAGAAAGTGGAAAAAAGGAAACATTTGGAACTTTAGGAATAATTTATGCTATATTAACAATTGGATTATTGGGATTTATTGTTTGAGCTCATCATATATTTACAGTAGGTATAGACGTAGATACTCGAGCTTATTTTACTTCAGCAACTATAATTATTGCTGTTCCTACAGGAATTAAAATTTTTAGTTGATTAGCAACTTTACACGGAACTCAATTAACATATAGTCCAGCCCTTCTATGATCATTAGGATTTGTATTTTTATTTACAGTTGGAGGTTTAACAGGAGTAGTATTAGCTAATTCTTCAATTGATATTGTTCTTCATGATACTTATTATGTAGTTGCCCATTTTCATTATGTTTTATCTATAGGAGCTGTATTTGCTATTATAGCAGGATTTATTCATTGATACCCTTTATTAACAGGAATAGTTATAAACCCTTCATGATTAAAGGCTCAATTTAGTATAATATTTATTGGAGTAAATCTAACTTTCTTTCCCCAACATTTTTTAGGATTAGCTGGAATACCTCGACGATACTCAGATTTTCCCGATAGTTACTTAACTTGAAATATTATTTCTTCTTTAGGAAGAACAATTTCATTATTTGCCGTTATTTTCTTTTTATTTATTATTTGAGAAAGTATAATTACTCAACGAACACCTTCTTTC

>Indonesia-COXI-H02

TTCTGATTCTTTGGACACCCAGAAGTTTATATTTTAATTTTACCCGGATTTGGAATAATTTCTCATATTATTACTCAAGAAAGTGGAAAAAAGGAAACATTTGGAACTTTAGGAATAATTTATGCTATATTAACAATTGGATTATTGGGATTTATTGTTTGAGCTCATCATATATTTACAGTAGGTATAGACGTAGATACTCGAGCTTATTTTACTTCAGCAACTATAATTATTGCTGTTCCTACAGGAATTAAAATTTTTAGTTGATTAGCAACTTTACACGGAACTCAATTAACATATAGTCCAGCCCTTCTATGATCATTAGGATTTGTATTTTTATTTACAGTTGGAGGTTTAACAGGAGTAGTATTAGCTAATTCTTCAATTGATATTGTTCTTCATGATACTTATTATGTAGTTGCCCATTTTCATTATGTTTTATCTATAGGAGCTGTATTTGCTATTATAGCAGGATTTATTCATTGATACCCTTTATTAACAGGAATAGTTATAAACCCTTCATGATTAAAGGCTCAATTTAGTATAATATTTATTGGAGTAAATCTAACTTTCTTTCCCCAACATTTTTTAGGATTAGCTGGAATACCTCGACGATACTCAGATTTTCCCGATAGTTACTTAACTTGAAATATTATTTCTTCTTTAGGAAGAACAATTTCATTATTTGCCGTTATTTTCTTTTTATTTATTATTTGAGAAAGTATAATTACTCAACGAACACCTTCTTTC

>Vietnam-COXI-A05

TTCTGATTCTTTGGACACCCAGAAGTTTATATTTTAATTTTACCCGGATTTGGAATAATTTCTCATATTATTACTCAAGAAAGTGGAAAAAAGGAAACATTTGGAACTTTAGGAATAATTTATGCTATATTAACAATTGGATTATTGGGATTTATTGTTTGAGCTCATCATATATTTACAGTAGGTATAGACGTAGATACTCGAGCTTATTTTACTTCAGCAACTATAATTATTGCTGTTCCTACAGGAATTAAAATTTTTAGTTGATTAGCAACTTTACACGGAACTCAATTAACATATAGTCCAGCCCTTCTATGATCATTAGGATTTGTATTTTTATTTACAGTTGGAGGTTTAACAGGAGTAGTATTAGCTAATTCTTCAATTGATATTGTTCTTCATGATACTTATTATGTAGTTGCCCATTTTCATTATGTTTTATCTATAGGAGCTGTATTTGCTATTATAGCAGGATTTATTCATTGATACCCTTTATTAACAGGAATAGTTATAAACCCTTCATGATTAAAGGCTCAATTTAGTATAATATTTATTGGAGTAAATCTAACTTTCTTTCCCCAACATTTTTTAGGATTAGCTGGAATACCTCGACGATACTCAGATTTTCCCGATAGTTACTTAACTTGAAATATTATTTCTTCTTTAGGAAGCACAATTTCATTATTTGCCGTTATTTTCTTTTTATTTATTATTTGAGAAAGTATAATTACTCAACGAACACCTTCTTTC

>Vietnam-COXI-A07

TTCTGATTCTTTGGACACCCAGAAGTTTATATTTTAATTTTACCCGGATTTGGAATAATTTCTCATATTATTACTCAAGAAAGTGGAAAAAAGGAAACATTTGGAACTTTAGGAATAATTTATGCTATATTAACAATTGGATTATTGGGATTTATTGTTTGAGCTCATCATATATTTACAGTAGGTATAGACGTAGATACTCGAGCTTATTTTACTTCAGCAACTATAATTATTGCTGTTCCTACAGGAATTAAAATTTTTAGTTGATTAGCAACTTTACACGGAACTCAATTAACATATAGTCCAGCCCTTCTATGATCATTAGGATTTGTATTTTTATTTACAGTTGGAGGTTTAACAGGAGTAGTATTAGCTAATTCTTCAATTGATATTGTTCTTCATGATACTTATTATGTAGTTGCCCATTTTCATTATGTTTTATCTATAGGAGCTGTATTTGCTATTATAGCAGGATTTATTCATTGATACCCTTTATTAACAGGAATAGTTATAAACCCTTCATGATTAAAGGCTCAATTTAGTATAATATTTATTGGAGTAAATCTAACTTTCTTTCCCCAACATTTTTTAGGATTAGCTGGAATACCTCGACGATACTCAGATTTTCCCGATAGTTACTTAACTTGAAATATTATTTCTTCTTTAGGAAGAACAATTTCATTATTTGCCGTTATTTTCTTTTTATTTATTATTTGAGAAAGTATAATTACTCAACGAACACCTTCTTTC

>Vietnam-COXI-B05

TTCTGATTCTTTGGACACCCAGAAGTTTATATTTTAATTTTACCCGGATTTGGAATAATTTCTCATATTATTACTCAAGAAAGTGGAAAAAAGGAAACATTTGGAACTTTAGGAATAATTTATGCTATATTAACAATTGGATTATTGGGATTTATTGTTTGAGCTCATCATATATTTACAGTAGGTATAGACGTAGATACTCGAGCTTATTTTACTTCAGCAACTATAATTATTGCTGTTCCTACAGGAATTAAAATTTTTAGTTGATTAGCAACTTTACACGGAACTCAATTAACATATAGTCCAGCCCTTCTATGATCATTAGGATTTGTATTTTTATTTACAGTTGGAGGTTTAACAGGAGTAGTATTAGCTAATTCTTCAATTGATATTGTTCTTCATGATACTTATTATGTAGTTGCCCATTTTCATTATGTTTTATCTATAGGAGCTGTATTTGCTATTATAGCAGGATTTATTCATTGATACCCTTTATTAACAGGAATAGTTATAAACCCTTCATGATTAAAGGCTCAATTTAGTATAATATTTATTGGAGTAAATCTAACTTTCTTTCCCCAACATTTTTTAGGATTAGCTGGAATACCTCGACGATACTCAGATTTTCCCGATAGTTACTTAACTTGAAATATTATTTCTTCTTTAGGAAGAACAATTTCATTATTTGCCGTTATTTTCTTTTTATTTATTATTTGAGAAAGTATAATTACTCAACGAACACCTTCTTTC

>Vietnam-COXI-B06

TTCTGATTCTTTGGACACCCAGAAGTTTATATTTTAATTTTACCCGGATTTGGAATAATTTCTCATATTATTACTCAAGAAAGTGGAAAAAAGGAAACATTTGGAACTTTAGGAATAATTTATGCTATATTAACAATTGGATTATTGGGATTTATTGTTTGAGCTCATCATATATTTACAGTAGGTATAGACGTAGATACTCGAGCTTATTTTACTTCAGCAACTATAATTATTGCTGTTCCTACAGGAATTAAAATTTTTAGTTGATTAGCAACTTTACACGGAACTCAATTAACATATAGTCCAGCCCTTCTATGATCATTAGGATTTGTATTTTTATTTACAGTTGGAGGTTTAACAGGAGTAGTATTAGCTAATTCTTCAATTGATATTGTTCTTCATGATACTTATTATGTAGTTGCCCATTTTCATTATGTTTTATCTATAGGAGCTGTATTTGCTATTATAGCAGGATTTATTCATTGATACCCTTTATTAACAGGAATAGTTATAAACCCTTCATGATTAAAGGCTCAATTTAGTATAATATTTATTGGAGTAAATCTAACTTTCTTTCCCCAACATTTTTTAGGATTAGCTGGAATACCTCGACGATACTCAGATTTTCCCGATAGTTACTTAACTTGAAATATTATTTCTTCTTTAGGAAGAACAATTTCATTATTTGCCGTTATTTTCTTTTTATTTATTATTTGAGAAAGTATAATTACTCAACGAACACCTTCTTTC

>Vietnam-COXI-B08

TTCTGATTCTTTGGACACCCAGAAGTTTATATTTTAATTTTACCCGGATTTGGAATAATTTCTCATATTATTACTCAAGAAAGTGGAAAAAAGGAAACATTTGGAACTTTAGGAATAATTTATGCTATATTAACAATTGGATTATTGGGATTTATTGTTTGAGCTCATCATATATTTACAGTAGGTATAGACGTAGATACTCGAGCTTATTTTACTTCAGCAACTATAATTATTGCTGTTCCTACAGGAATTAAAATTTTTAGTTGATTAGCAACTTTACACGGAACTCAATTAACATATAGTCCAGCCCTTCTATGATCATTAGGATTTGTATTTTTATTTACAGTTGGAGGTTTAACAGGAGTAGTATTAGCTAATTCTTCAATTGATATTGTTCTTCATGATACTTATTATGTAGTTGCCCATTTTCATTATGTTTTATCTATAGGAGCTGTATTTGCTATTATAGCAGGATTTATTCATTGATACCCTTTATTAACAGGAATAGTTATAAACCCTTCATGATTAAAGGCTCAATTTAGTATAATATTTATTGGAGTAAATCTAACTTTCTTTCCCCAACATTTTTTAGGATTAGCTGGAATACCTCGACGATACTCAGATTTTCCCGATAGTTACTTAACTTGAAATATTATTTCTTCTTTAGGAAGAACAATTTCATTATTTGCCGTTATTTTCTTTTTATTTATTATTTGAGAAAGTATAATTACTCAACGAACACCTTCTTTC

>Vietnam-COXI-C05

TTCTGATTCTTTGGACACCCAGAAGTTTATATTTTAATTTTACCCGGATTTGGAATAATTTCTCATATTATTACTCAAGAAAGTGGAAAAAAGGAAACATTTGGAACTTTAGGAATAATTTATGCTATATTAACAATTGGATTATTGGGATTTATTGTTTGAGCTCATCATATATTTACAGTAGGTATAGACGTAGATACTCGAGCTTATTTTACTTCAGCAACTATAATTATTGCTGTTCCTACAGGAATTAAAATTTTTAGTTGATTAGCAACTTTACACGGAACTCAATTAACATATAGTCCAGCCCTTCTATGATCATTAGGATTTGTATTTTTATTTACAGTTGGAGGTTTAACAGGAGTAGTATTAGCTAATTCTTCAATTGATATTGTTCTTCATGATACTTATTATGTAGTTGCCCATTTTCATTATGTTTTATCTATAGGAGCTGTATTTGCTATTATAGCAGGATTTATTCATTGATACCCTTTATTAACAGGAATAGTTATAAACCCTTCATGATTAAAGGCTCAATTTAGTATAATATTTATTGGAGTAAATCTAACTTTCTTTCCCCAACATTTTTTAGGATTAGCTGGAATACCTCGACGATACTCAGATTTTCCCGATAGTTACTTAACTTGAAATATTATTTCTTCTTTAGGAAGAACAATTTCATTATTTGCCGTTATTTTCTTTTTATTTATTATTTGAGAAAGTATAATTACTCAACGAACACCTTCTTTC

>Vietnam-COXI-C06

TTTTGATTCTTTGGACACCCAGAAGTTTATATTTTAATTTTACCCGGATTTGGAATAATTTCTCATATTATTACTCAAGAAAGCGGAAAAAAGGAAACATTTGGAACTTTAGGAATAATTTATGCTATATTAACAATTGGATTATTGGGATTTATTGTTTGAGCTCATCATATATTTACAGTAGGTATAGACGTAGATACTCGAGCTTATTTTACTTCAGCAACTATAATTATTGCTGTTCCTACAGGAATTAAAATTTTTAGTTGATTAGCAACTTTACACGGAACTCAATTAACATATAGTCCAGCCCTTCTATGATCATTAGGATTTGTATTTTTATTTACAGTTGGAGGTTTAACAGGAGTAGTATTAGCTAATTCTTCAATTGATATTGTTCTTCATGATACTTATTACGTAGTTGCCCATTTTCATTACGTTTTATCTATAGGAGCTGTATTTGCTATTATAGCAGGATTTATTCATTGATACCCTTTATTAACAGGAATAGTTATAAACCCTTCATGATTAAAGGCTCAATTTAGTATAATATTTATTGGAGTAAATCTAACTTTCTTTCCTCAACATTTTTTAGGGTTAGCTGGAATACCTCGACGATACTCAGATTTTCCTGATAGCTACTTAACTTGAAATATTATTTCTTCTTTAGGAAGAACAATTTCACTATTTGCCGTTATTTTCTTTTTATTTATTATTTGAGAAAGTATAATTACTCAACGAACACCTTCTTTC

>Vietnam-COXI-C07

TTCTGATTCTTTGGACACCCAGAAGTTTATATTTTAATTTTACCCGGATTTGGAATAATTTCTCATATTATTACTCAAGAAAGTGGAAAAAAGGAAACATTTGGAACTTTAGGAATAATTTATGCTATATTAACAATTGGATTATTGGGATTTATTGTTTGAGCTCATCATATATTTACAGTAGGTATAGACGTAGATACTCGAGCTTATTTTACTTCAGCAACTATAATTATTGCTGTTCCTACAGGAATTAAAATTTTTAGTTGATTAGCAACTTTACACGGAACTCAATTAACATATAGTCCAGCCCTTCTATGATCATTAGGATTTGTATTTTTATTTACAGTTGGAGGTTTAACAGGAGTAGTATTAGCTAATTCTTCAATTGATATTGTTCTTCATGATACTTATTATGTAGTTGCCCATTTTCATTATGTTTTATCTATAGGAGCTGTATTTGCTATTATAGCAGGATTTATTCATTGATACCCTTTATTAACAGGAATAGTTATAAACCCTTCATGATTAAAGGCTCAATTTAGTATAATATTTATTGGAGTAAATCTAACTTTCTTTCCCCAACATTTTTTAGGATTAGCTGGAATACCTCGACGATACTCAGATTTTCCCGATAGTTACTTAACTTGAAATATTATTTCTTCTTTAGGAAGAACAATTTCATTATTTGCCGTTATTTTCTTTTTATTTATTATTTGAGAAAGTATAATTACTCAACGAACACCTTCTTTC

>Vietnam-COXI-C08

TTTTGATTCTTTGGACACCCAGAAGTTTATATTTTAATTTTACCCGGATTTGGAATAATTTCTCATATTATTACTCAAGAAAGCGGAAAAAAGGAAACATTTGGAACTTTAGGAATAATTTATGCTATATTAACAATTGGATTATTGGGATTTATTGTTTGAGCTCATCATATATTTACAGTAGGTATAGACGTAGATACTCGAGCTTATTTTACTTCAGCAACTATAATTATTGCTGTTCCTACAGGAATTAAAATTTTTAGTTGATTAGCAACTTTACACGGAACTCAATTAACATATAGTCCAGCCCTTCTATGATCATTAGGATTTGTATTTTTATTTACAGTTGGAGGTTTAACAGGAGTAGTATTAGCTAATTCTTCAATTGATATTGTTCTTCATGATACTTATTACGTAGTTGCCCATTTTCATTACGTTTTATCTATAGGAGCTGTATTTGCTATTATAGCAGGATTTATTCATTGATACCCTTTATTAACAGGAATAGTTATAAACCCTTCATGATTAAAGGCTCAATTTAGTATAATATTTATTGGAGTAAATCTAACTTTCTTTCCTCAACATTTTTTAGGGTTAGCTGGAATACCTCGACGATACTCAGATTTTCCTGATAGCTACTTAACTTGAAATATTATTTCTTCTTTAGGAAGAACAATTTCACTATTTGCCGTTATTTTCTTTTTATTTATTATTTGAGAAAGTATAATTACTCAACGAACACCTTCTTTC

>Vietnam-COXI-D05

TTTTGATTCTTTGGGCACCCAGAAGTTTATATTTTAATTTTACCCGGATTTGGGATAATTTCTCATATTATTACTCAAGAAAGTGGAAAAAAGGAAACATTTGGAACTTTAGGAATAATTTATGCTATATTAACAATTGGATTATTGGGATTTATTGTTTGAGCTCATCATATATTTACAGTAGGTATAGACGTAGATACTCGAGCTTATTTTACTTCAGCAACTATAATTATTGCTGTTCCTACAGGAATTAAAATTTTTAGTTGATTAGCAACTTTACACGGAACTCAATTAACATATAGTCCAGCCCTTCTATGATCATTAGGGTTTGTATTTTTATTTACAGTTGGAGGTTTAACAGGAGTAGTGTTAGCTAATTCTTCAATTGATATTGTTCTTCATGATACTTATTACGTAGTTGCCCATTTTCATTATGTTTTATCTATAGGAGCTGTATTTGCTATTATAGCAGGATTTATTCATTGGTACCCTTTATTAACAGGAATAGTTATGAACCCTTCATGATTAAAGGCTCAATTTAGTATAATATTTATTGGAGTAAATCTAACTTTCTTTCCTCAACATTTTTTAGGATTAGCTGGAATACCTCGACGATACTCAGATTTTCCTGATAGTTACTTAACTTGAAATATTATTTCTTCTTTAGGAAGAACAATTTCATTATTTGCCGTTATTTTCTTTTTATTTATTATTTGAGAAAGTATAATTACTCAACGAACACCTTCTTTC

>Vietnam-COXI-D06

TTCTGATTCTTTGGACACCCAGAAGTTTATATTTTAATTTTACCCGGATTTGGAATAATTTCTCATATTATTACTCAAGAAAGTGGAAAAAAGGAAACATTTGGAACTTTAGGAATAATTTATGCTATATTAACAATTGGATTATTGGGATTTATTGTTTGAGCTCATCATATATTTACAGTAGGTATAGACGTAGATACTCGAGCTTATTTTACTTCAGCAACTATAATTATTGCTGTTCCTACAGGAATTAAAATTTTTAGTTGATTAGCAACTTTACACGGAACTCAATTAACATATAGTCCAGCCCTTCTATGATCATTAGGATTTGTATTTTTATTTACAGTTGGAGGTTTAACAGGAGTAGTATTAGCTAATTCTTCAATTGATATTGTTCTTCATGATACTTATTATGTAGTTGCCCATTTTCATTATGTTTTATCTATAGGAGCTGTATTTGCTATTATAGCAGGATTTATTCATTGATACCCTTTATTAACAGGAATAATTATAAACCCTTCATGATTAAAGGCTCAATTTAGTATAATATTTATTGGAGTAAATCTAACTTTCTTTCCCCAACATTTTTTAGGATTAGCTGGAATACCTCGACGATACTCAGATTTTCCCGATAGTTACTTAACTTGAAATATTATTTCTTCTTTAGGAAGAACAATTTCATTATTTGCCGTTATTTTCTTTTTATTTATTATTTGAGAAAGTATAATTACTCAACGAACACCTTCTTTC

>Vietnam-COXI-D07

TTTTGATTCTTTGGGCACCCAGAAGTTTATATTTTAATTTTACCCGGATTTGGGATAATTTCTCATATTATTACTCAAGAAAGTGGAAAAAAGGAAACATTTGGAACTTTAGGAATAATTTATGCTATATTAACAATTGGATTATTGGGATTTATTGTTTGAGCTCATCATATATTTACAGTAGGTATAGACGTAGATACTCGAGCTTATTTTACTTCAGCAACTATAATTATTGCTGTTCCTACAGGAATTAAAATTTTTAGTTGATTAGCAACTTTACACGGAACTCAATTAACATATAGTCCAGCCCTTCTATGATCATTAGGGTTTGTATTTTTATTTACAGTTGGAGGTTTAACAGGAGTAGTGTTAGCTAATTCTTCAATTGATATTGTTCTTCATGATACTTATTACGTAGTTGCCCATTTTCATTATGTTTTATCTATAGGAGCTGTATTTGCTATTATAGCAGGATTTATTCATTGGTACCCTTTATTAACAGGAATAGTTATGAACCCTTCATGATTAAAGGCTCAATTTAGTATAATATTTATTGGAGTAAATCTAACTTTCTTTCCTCAACATTTTTTAGGATTAGCTGGAATACCTCGACGATACTCAGATTTTCCTGATAGTTACTTAACTTGAAATATTATTTCTTCTTTAGGAAGAACAATTTCATTATTTGCCGTTATTTTCTTTTTATTTATTATTTGAGAAAGTATAATTACTCAACGAACACCTTCTTTC

>Vietnam-COXI-D08

TTCTGATTCTTTGGACACCCAGAAGTTTATATTTTAATTTTACCCGGATTTGGAATAATTTCTCATATTATTACTCAAGAAAGTGGAAAAAAGGAAACATTTGGAACTTTAGGAATAATTTATGCTATATTAACAATTGGATTATTGGGATTTATTGTTTGAGCTCATCATATATTTACAGTAGGTATAGACGTAGATACTCGAGCTTATTTTACTTCAGCAACTATAATTATTGCTGTACCTACAGGAATTAAAATTTTTAGTTGATTAGCAACTTTACACGGAACTCAATTAACATATAGTCCAGCCCTTCTATGATCATTAGGATTTGTATTTTTATTTACAGTTGGAGGTTTAACAGGAGTAGTATTAGCTAATTCTTCAATTGATATTGTTCTTCATGATACTTATTATGTAGTTGCCCATTTTCATTATGTTTTATCTATAGGAGCTGTATTTGCTATTATAGCAGGATTTATTCATTGATACCCTTTATTAACAGGAATAGTTATAAACCCTTCATGATTAAAGGCTCAATTTAGTATAATATTTATTGGAGTAAATCTAACTTTCTTTCCCCAACATTTTTTAGGATTAGCTGGAATACCTCGACGATACTCAGATTTTCCCGATAGTTACTTAACTTGAAATATTATTTCTTCTTTAGGAAGCACAATTTCATTATTTGCCGTTATTTTCTTTTTATTTATTATTTGAGAAAGTATAATTACTCAACGAACACCTTCTTTC

>Vietnam-COXI-E05

TTCTGATTCTTTGGACACCCAGAAGTTTATATTTTAATTTTACCCGGATTTGGAATAATTTCTCATATTATTACTCAAGAAAGTGGAAAAAAGGAAACATTTGGAACTTTAGGAATAATTTATGCTATATTAACAATTGGATTATTGGGATTTATTGTTTGAGCTCATCATATATTTACAGTAGGTATAGACGTAGATACTCGAGCTTATTTTACTTCAGCAACTATAATTATTGCTGTACCTACAGGAATTAAAATTTTTAGTTGATTAGCAACTTTACACGGAACTCAATTAACATATAGTCCAGCCCTTCTATGATCATTAGGATTTGTATTTTTATTTACAGTTGGAGGTTTAACAGGAGTAGTATTAGCTAATTCTTCAATTGATATTGTTCTTCATGATACTTATTATGTAGTTGCCCATTTTCATTATGTTTTATCTATAGGAGCTGTATTTGCTATTATAGCAGGATTTATTCATTGATACCCTTTATTAACAGGAATAGTTATAAACCCTTCATGATTAAAGGCTCAATTTAGTATAATATTTATTGGAGTAAATCTAACTTTCTTTCCCCAACATTTTTTAGGATTAGCTGGAATACCTCGACGATACTCAGATTTTCCCGATAGTTACTTAACTTGAAATATTATTTCTTCTTTAGGAAGCACAATTTCATTATTTGCCGTTATTTTCTTTTTATTTATTATTTGAGAAAGTATAATTACTCAACGAACACCTTCTTTC

>Vietnam-COXI-E06

TTCTGATTCTTTGGACACCCAGAAGTTTATATTTTAATTTTACCCGGATTTGGAATAATTTCTCATATTATTACTCAAGAAAGTGGAAAAAAGGAAACATTTGGAACTTTAGGAATAATTTATGCTATATTAACAATTGGATTATTGGGATTTATTGTTTGAGCTCATCATATATTTACAGTAGGTATAGACGTAGATACTCGAGCTTATTTTACTTCAGCAACTATAATTATTGCTGTTCCTACAGGAATTAAAATTTTTAGTTGATTAGCAACTTTACACGGAACTCAATTAACATATAGTCCAGCCCTTCTATGATCATTAGGATTTGTATTTTTATTTACAGTTGGAGGTTTAACAGGAGTAGTATTAGCTAATTCTTCAATTGATATTGTTCTTCATGATACTTATTATGTAGTTGCCCATTTTCATTATGTTTTATCTATAGGAGCTGTATTTGCTATTATAGCAGGATTTATTCATTGATACCCTTTATTAACAGGAATAGTTATAAACCCTTCATGATTAAAGGCTCAATTTAGTATAATATTTATTGGAGTAAATCTAACTTTCTTTCCCCAACATTTTTTAGGATTAGCTGGAATACCTCGACGATACTCAGATTTTCCCGATAGTTACTTAACTTGAAATATTATTTCTTCTTTAGGAAGAACAATTTCATTATTTGCCGTTATTTTCTTTTTATTTATTATTTGAGAAAGTATAATTACTCAACGAACACCGTCTTTC

>Vietnam-COXI-E07

TTCTGATTCTTTGGACACCCAGAAGTTTATATTTTAATTTTACCCGGATTTGGAATAATTTCTCATATTATTACTCAAGAAAGTGGAAAAAAGGAAACATTTGGAACTTTAGGAATAATTTATGCTATATTAACAATTGGATTATTGGGATTTATTGTTTGAGCTCATCATATATTTACAGTAGGTATAGACGTAGATACTCGAGCTTATTTTACTTCAGCAACTATAATTATTGCTGTTCCTACAGGAATTAAAATTTTTAGTTGATTAGCAACTTTACACGGAACTCAATTAACATATAGTCCAGCCCTTCTATGATCATTAGGATTTGTATTTTTATTTACAGTTGGAGGTTTAACAGGAGTAGTATTAGCTAATTCTTCAATTGATATTGTTCTTCATGATACTTATTATGTAGTTGCCCATTTTCATTATGTTTTATCTATAGGAGCTGTATTTGCTATTATAGCAGGATTTATTCATTGATACCCTTTATTAACAGGAATAGTTATAAACCCTTCATGATTAAAGGCTCAATTTAGTATAATATTTATTGGAGTAAATCTAACTTTCTTTCCCCAACATTTTTTAGGATTAGCTGGAATACCTCGACGATACTCAGATTTTCCCGATAGTTACTTAACTTGAAATATTATTTCTTCTTTAGGAAGAACAATTTCATTATTTGCCGTTATTTTCTTTTTATTTATTATTTGAGAAAGTATAATTACTCAACGAACACCGTCTTTC

>Vietnam-COXI-E08

TTTTGATTCTTTGGGCACCCAGAAGTTTATATTTTAATTTTACCCGGATTTGGGATAATTTCTCATATTATTACTCAAGAAAGTGGAAAAAAGGAAACATTTGGAACTTTAGGAATAATTTATGCTATATTAACAATTGGATTATTGGGATTTATTGTTTGAGCTCATCATATATTTACAGTAGGTATAGACGTAGATACTCGAGCTTATTTTACTTCAGCAACTATAATTATTGCTGTTCCTACAGGAATTAAAATTTTTAGTTGATTAGCAACTTTACACGGAACTCAATTAACATATAGTCCAGCCCTTCTATGATCATTAGGGTTTGTATTTTTATTTACAGTTGGAGGTTTAACAGGAGTAGTGTTAGCTAATTCTTCAATTGATATTGTTCTTCATGATACTTATTACGTAGTTGCCCATTTTCATTATGTTTTATCTATAGGAGCTGTATTTGCTATTATAGCAGGATTTATTCATTGGTACCCTTTATTAACAGGAATAGTTATGAACCCTTCATGATTAAAGGCTCAATTTAGTATAATATTTATTGGAGTAAATCTAACTTTCTTTCCTCAACATTTTTTAGGATTAGCTGGAATACCTCGACGATACTCAGATTTTCCTGATAGTTACTTAACTTGAAATATTATTTCTTCTTTAGGAAGAACAATTTCATTATTTGCCGTTATTTTCTTTTTATTTATTATTTGAGAAAGTATAATTACTCAACGAACACCTTCTTTC

>Vietnam-COXI-F05

TTCTGATTCTTTGGACACCCAGAAGTTTATATTTTAATTTTACCCGGATTTGGAATAATTTCTCATATTATTACTCAAGAAAGTGGAAAAAAGGAAACATTTGGAACTTTAGGAATAATTTATGCTATATTAACAATTGGATTATTGGGATTTATTGTTTGAGCTCATCATATATTTACAGTAGGTATAGACGTAGATACTCGAGCTTATTTTACTTCAGCAACTATAATTATTGCTGTACCTACAGGAATTAAAATTTTTAGTTGATTAGCAACTTTACACGGAACTCAATTAACATATAGTCCAGCCCTTCTATGATCATTAGGATTTGTATTTTTATTTACAGTTGGAGGTTTAACAGGAGTAGTATTAGCTAATTCTTCAATTGATATTGTTCTTCATGATACTTATTATGTAGTTGCCCATTTTCATTATGTTTTATCTATAGGAGCTGTATTTGCTATTATAGCAGGATTTATTCATTGATACCCTTTATTAACAGGAATAGTTATAAACCCTTCATGATTAAAGGCTCAATTTAGTATAATATTTATTGGAGTAAATCTAACTTTCTTTCCCCAACATTTTTTAGGATTAGCTGGAATACCTCGACGATACTCAGATTTTCCCGATAGTTACTTAACTTGAAATATTATTTCTTCTTTAGGAAGCACAATTTCATTATTTGCCGTTATTTTCTTTTTATTTATTATTTGAGAAAGTATAATTACTCAACGAACACCTTCTTTC

>Vietnam-COXI-F06

TTCTGATTCTTTGGACACCCAGAAGTTTATATTTTAATTTTACCCGGATTTGGAATAATTTCTCATATTATTACTCAAGAAAGTGGAAAAAAGGAAACATTTGGAACTTTAGGAATAATTTATGCTATATTAACAATTGGATTATTGGGATTTATTGTTTGAGCTCATCATATATTTACAGTAGGTATAGACGTAGATACTCGAGCTTATTTTACTTCAGCAACTATAATTATTGCTGTACCTACAGGAATTAAAATTTTTAGTTGATTAGCAACTTTACACGGAACTCAATTAACATATAGTCCAGCCCTTCTATGATCATTAGGATTTGTATTTTTATTTACAGTTGGAGGTTTAACAGGAGTAGTATTAGCTAATTCTTCAATTGATATTGTTCTTCATGATACTTATTATGTAGTTGCCCATTTTCATTATGTTTTATCTATAGGAGCTGTATTTGCTATTATAGCAGGATTTATTCATTGATACCCTTTATTAACAGGAATAGTTATAAACCCTTCATGATTAAAGGCTCAATTTAGTATAATATTTATTGGAGTAAATCTAACTTTCTTTCCCCAACATTTTTTAGGATTAGCTGGAATACCTCGACGATACTCAGATTTTCCCGATAGTTACTTAACTTGAAATATTATTTCTTCTTTAGGAAGCACAATTTCATTATTTGCCGTTATTTTCTTTTTATTTATTATTTGAGAAAGTATAATTACTCAACGAACACCTTCTTTC

>Vietnam-COXI-F07

TTTTGATTCTTTGGACACCCAGAAGTTTATATTTTAATTTTACCCGGATTTGGAATAATTTCTCATATTATTACTCAAGAAAGTGGAAAAAAGGAAACATTTGGAACTTTAGGAATAATTTATGCTATATTAACAATTGGATTATTGGGATTTATTGTTTGAGCTCATCATATATTTACAGTAGGTATAGACGTAGATACTCGAGCTTATTTTACTTCAGCAACTATAATTATTGCTGTTCCTACAGGAATTAAAATTTTTAGTTGATTAGCAACTTTACACGGAACTCAATTAACATATAGTCCAGCCCTTCTATGATCATTAGGATTTGTATTTTTATTTACAGTTGGAGGTTTAACAGGAGTAGTATTAGCTAATTCTTCAATTGATATTGTTCTTCATGATACTTATTACGTAGTTGCCCATTTTCATTACGTTTTATCTATAGGAGCTGTATTTGCTATTATAGCAGGATTTATTCATTGATACCCTTTATTAACAGGAATAGTTATAAACCCTTCATGATTAAAGGCTCAATTTAGTATAATATTTATTGGAGTAAATCTAACTTTCTTTCCTCAACATTTTTTAGGRTTAGCTGGAATACCTCGACGATACTCAGATTTTCCTGATAGYTACTTAACTTGAAATATTATTTCTTCTTTAGGAAGAACAATTTCAYTATTTGCCGTTATTTTCTTTTTATTTATTATTTGAGAAAGTATAATTACTCAACGAACACCTTCTTTC

>Vietnam-COXI-F08

TTTTGATTCTTTGGGCACCCAGAAGTTTATATTTTAATTTTACCCGGATTTGGGATAATTTCTCATATTATTACTCAAGAAAGTGGAAAAAAGGAAACATTTGGAACTTTAGGAATAATTTATGCTATATTAACAATTGGATTATTGGGATTTATTGTTTGAGCTCATCATATATTTACAGTAGGTATAGACGTAGATACTCGAGCTTATTTTACTTCAGCAACTATAATTATTGCTGTTCCTACAGGAATTAAAATTTTTAGTTGATTAGCAACTTTACACGGAACTCAATTAACATATAGTCCAGCCCTTCTATGATCATTAGGGTTTGTATTTTTATTTACAGTTGGAGGTTTAACAGGAGTAGTGTTAGCTAATTCTTCAATTGATATTGTTCTTCATGATACTTATTACGTAGTTGCCCATTTTCATTATGTTTTATCTATAGGAGCTGTATTTGCTATTATAGCAGGATTTATTCATTGGTACCCTTTATTAACAGGAATAGTTATGAACCCTTCATGATTAAAGGCTCAATTTAGTATAATATTTATTGGAGTAAATCTAACTTTCTTTCCTCAACATTTTTTAGGATTAGCTGGAATACCTCGACGATACTCAGATTTTCCTGATAGTTACTTAACTTGAAATATTATTTCTTCTTTAGGAAGAACAATTTCATTATTTGCCGTTATTTTCTTTTTATTTATTATTTGAGAAAGTATAATTACTCAACGAACACCTTCTTTC

>Vietnam-COXI-G05

TTTTGATTCTTTGGACACCCAGAAGTTTATATTTTAATTTTACCCGGATTTGGAATAATTTCTCATATTATTACTCAAGAAAGCGGAAAAAAGGAAACATTTGGAACTTTAGGAATAATTTATGCTATATTAACAATTGGATTATTGGGATTTATTGTTTGAGCTCATCATATATTTACAGTAGGTATAGACGTAGATACTCGAGCTTATTTTACTTCAGCAACTATAATTATTGCTGTTCCTACAGGAATTAAAATTTTTAGTTGATTAGCAACTTTACACGGAACTCAATTAACATATAGTCCAGCCCTTCTATGATCATTAGGATTTGTATTTTTATTTACAGTTGGAGGTTTAACAGGAGTAGTATTAGCTAATTCTTCAATTGATATTGTTCTTCATGATACTTATTACGTAGTTGCCCATTTTCATTACGTTTTATCTATAGGAGCTGTATTTGCTATTATAGCAGGATTTATTCATTGATACCCTTTATTAACAGGAATAGTTATAAACCCTTCATGATTAAAGGCTCAATTTAGTATAATATTTATTGGAGTAAATCTAACTTTCTTTCCTCAACATTTTTTAGGGTTAGCTGGAATACCTCGACGATACTCAGATTTTCCTGATAGCTACTTAACTTGAAATATTATTTCTTCTTTAGGAAGAACAATTTCACTATTTGCCGTTATTTTCTTTTTATTTATTATTTGAGAAAGTATAATTACTCAACGAACACCTTCTTTC

>Vietnam-COXI-G06

TTCTGATTCTTTGGACACCCAGAAGTTTATATTTTAATTTTACCCGGATTTGGAATAATTTCTCATATTATTACTCAAGAAAGTGGAAAAAAGGAAACATTTGGAACTTTAGGAATAATTTATGCTATATTAACAATTGGATTATTGGGATTTATTGTTTGAGCTCATCATATATTTACAGTAGGTATAGACGTAGATACTCGAGCTTATTTTACTTCAGCAACTATAATTATTGCTGTACCTACAGGAATTAAAATTTTTAGTTGATTAGCAACTTTACACGGAACTCAATTAACATATAGTCCAGCCCTTCTATGATCATTAGGATTTGTATTTTTATTTACAGTTGGAGGTTTAACAGGAGTAGTATTAGCTAATTCTTCAATTGATATTGTTCTTCATGATACTTATTATGTAGTTGCCCATTTTCATTATGTTTTATCTATAGGAGCTGTATTTGCTATTATAGCAGGATTTATTCATTGATACCCTTTATTAACAGGAATAGTTATAAACCCTTCATGATTAAAGGCTCAATTTAGTATAATATTTATTGGAGTAAATCTAACTTTCTTTCCCCAACATTTTTTAGGATTAGCTGGAATACCTCGACGATACTCAGATTTTCCCGATAGTTACTTAACTTGAAATATTATTTCTTCTTTAGGAAGCACAATTTCATTATTTGCCGTTATTTTCTTTTTATTTATTATTTGAGAAAGTATAATTACTCAACGAACACCTTCTTTC

>Vietnam-COXI-H03

TTCTGATTCTTTGGACACCCAGAAGTTTATATTTTAATTTTACCCGGATTTGGAATAATTTCTCATATTATTACTCAAGAAAGTGGAAAAAAGGAAACATTTGGAACTTTAGGAATAATTTATGCTATATTAACAATTGGATTATTGGGATTTATTGTTTGAGCTCATCATATATTTACAGTAGGTATAGACGTAGATACTCGAGCTTATTTTACTTCAGCAACTATAATTATTGCTGTTCCTACAGGAATTAAAATTTTTAGTTGATTAGCAACTTTACACGGAACTCAATTAACATATAGTCCAGCCCTTCTATGATCATTAGGATTTGTATTTTTATTTACAGTTGGAGGTTTAACAGGAGTAGTATTAGCTAATTCTTCAATTGATATTGTTCTTCATGATACTTATTATGTAGTTGCCCATTTTCATTATGTTTTATCTATAGGAGCTGTATTTGCTATTATAGCAGGATTTATTCATTGATACCCTTTATTAACAGGAATAGTTATAAACCCTTCATGATTAAAGGCTCAATTTAGTATAATATTTATTGGAGTAAATCTAACTTTCTTTCCCCAACATTTTTTAGGATTAGCTGGAATACCTCGACGATACTCAGATTTTCCCGATAGTTACTTAACTTGAAATATTATTTCTTCTTTAGGAAGAACAATTTCATTATTTGCCGTTATTTTCTTTTTATTTATTATTTGAGAAAGTATAATTACTCAACGAACACCKTCTTTC

>Vietnam-COXI-H04

TTTTGATTCTTTGGGCACCCAGAAGTTTATATTTTAATTTTACCCGGATTTGGGATAATTTCTCATATTATTACTCAAGAAAGTGGAAAAAAGGAAACATTTGGAACTTTAGGAATAATTTATGCTATATTAACAATTGGATTATTGGGATTTATTGTTTGAGCTCATCATATATTTACAGTAGGTATAGACGTAGATACTCGAGCTTATTTTACTTCAGCAACTATAATTATTGCTGTTCCTACAGGAATTAAAATTTTTAGTTGATTAGCAACTTTACACGGAACTCAATTAACATATAGTCCAGCCCTTCTATGATCATTAGGRTTTGTATTTTTATTTACAGTTGGAGGTTTAACAGGAGTAGTGTTAGCTAATTCTTCAATTGATATTGTTCTTCATGATACTTATTACGTAGTTGCCCATTTTCATTATGTTTTATCTATAGGAGCTGTATTTGCTATTATAGCAGGATTTATTCATTGRTACCCTTTATTAACAGGAATAGTTATGAACCCTTCATGATTAAAGGCTCAATTTAGTATAATATTTATTGGAGTAAATCTAACTTTCTTTCCTCAACATTTTTTAGGATTAGCTGGAATACCTCGACGATACTCAGATTTTCCTGATAGTTACTTAACTTGAAATATTATTTCTTCTTTAGGAAGAACAATTTCATTATTTGCCGTTATTTTCTTTTTATTTATTATTTGAGAAAGTATAATTACTCAACGAACACCTTCTTTC

>Vietnam-COXI-H05

TTCTGATTCTTTGGACACCCAGAAGTTTATATTTTAATTTTACCCGGATTTGGAATAATTTCTCATATTATTACTCAAGAAAGTGGAAAAAAGGAAACATTTGGAACTTTAGGAATAATTTATGCTATATTAACAATTGGATTATTGGGATTTATTGTTTGAGCTCATCATATATTTACAGTAGGTATAGACGTAGATACTCGAGCTTATTTTACTTCAGCAACTATAATTATTGCTGTTCCTACAGGAATTAAAATTTTTAGTTGATTAGCAACTTTACACGGAACTCAATTAACATATAGTCCAGCCCTTCTATGATCATTAGGATTTGTATTTTTATTTACAGTTGGAGGTTTAACAGGAGTAGTATTAGCTAATTCTTCAATTGATATTGTTCTTCATGATACTTATTATGTAGTTGCCCATTTTCATTATGTTTTATCTATAGGAGCTGTATTTGCTATTATAGCAGGATTTATTCATTGATACCCTTTATTAACAGGAATAGTTATAAACCCTTCATGATTAAAGGCTCAATTTAGTATAATATTTATTGGAGTAAATCTAACTTTCTTTCCCCAACATTTTTTAGGATTAGCTGGAATACCTCGACGATACTCAGATTTTCCCGATAGTTACTTAACTTGAAATATTATTTCTTCTTTAGGAAGAACAATTTCATTATTTGCCGTTATTTTCTTTTTATTTATTATTTGAGAAAGTATAATTACTCAACGAACACCGTCTTTC

>Vietnam-COXI-H06

TTYTGATTCTTTGGACACCCAGAAGTTTATATTTTAATTTTACCCGGATTTGGAATAATTTCTCATATTATTACTCAAGAAAGTGGAAAAAAGGAAACATTTGGAACTTTAGGAATAATTTATGCTATATTAACAATTGGATTATTGGGATTTATTGTTTGAGCTCATCATATATTTACAGTAGGTATAGACGTAGATACTCGAGCTTATTTTACTTCAGCAACTATAATTATTGCTGTTCCTACAGGAATTAAAATTTTTAGTTGATTAGCAACTTTACACGGAACTCAATTAACATATAGTCCAGCCCTTCTATGATCATTAGGATTTGTATTTTTATTTACAGTTGGAGGTTTAACAGGAGTAGTATTAGCTAATTCTTCAATTGATATTGTTCTTCATGATACTTATTATGTAGTTGCCCATTTTCATTATGTTTTATCTATAGGAGCTGTATTTGCTATTATAGCAGGATTTATTCATTGATACCCTTTATTAACAGGAATAGTTATAAACCCTTCATGATTAAAGGCTCAATTTAGTATAATATTTATTGGAGTAAATCTAACTTTCTTTCCCCAACATTTTTTAGGATTAGCTGGAATACCTCGACGATACTCAGATTTTCCYGATAGTTACTTAACTTGAAATATTATTTCTTCTTTAGGAAGAACAATTTCATTATTTGCCGTTATTTTCTTTTTATTTATTATTTGAGAAAGTATAATTACTCAACGAACACCTTCTTTC

>Brazil-ND5-A09

CGATTTAATATTTTATTAGATAATTCTAAGTTAGGACAATTTTTATTATTAGTTTCTGGATTAACAATATTTATAGCTGGATTAGGGGCTAATTTTGAGTTTGATTTAAAAAAAATTATTGCTTTATCTACTTTAAGTCAATTAGGTTTAATAATAAGTATTTTATCTATTGGTTATTATAAGTTAGCTTTTTTTCATTTATTAACACATGCACTCTTTAAGGCTTTATTATTTATATGTGCTGGAGTAATTATTCATAATACAAAAAATGCTCAAGATATTCGATTTATAGGGGGTTTAAGTATAAGAATACCTTTAACATGTAGTTGTTTTAATATTGCTAATTTAGCTTTATGTGGAATACCTTTTTTG

>Brazil-ND5-A10

CGATTTAATATTTTATTAGATAATTCTAAGTTAGGACAATTTTTATTATTAGTTTCTGGATTAACAATATTTATAGCTGGATTAGGGGCTAATTTTGAGTTTGATTTAAAAAAAATTATTGCTTTATCTACTTTAAGTCAATTAGGTTTAATAATAAGTATTTTATCTATTGGTTATTATAAGTTAGCTTTTTTTCATTTATTAACACATGCACTCTTTAAGGCTTTATTATTTATATGTGCTGGAGTAATTATTCATAATACAAAAAATGCTCAAGATATTCGATTTATAGGGGGTTTAAGTATAAGAATACCTTTAACATGTAGTTGTTTTAATATTGCTAATTTAGCTTTATGTGGAATACCTTTTTTG

>Brazil-ND5-A11

CGATTTAATATTTTATTAGATAATTCTAAGTTAGGACAATTTTTATTATTAGTTTCTGGATTAACAATATTTATAGCTGGATTAGGGGCTAATTTTGAGTTTGATTTAAAAAAAATTATTGCTTTATCTACTTTAAGTCAATTAGGTTTAATAATAAGTATTTTATCTATTGGTTATTATAAGTTAGCTTTTTTTCATTTATTAACACATGCACTCTTTAAGGCTTTATTATTTATATGTGCTGGAGTAATTATTCATAATACAAAAAATGCTCAAGATATTCGATTTATAGGGGGTTTAAGTATAAGAATACCTTTAACATGTAGTTGTTTTAATATTGCTAATTTAGCTTTATGTGGAATACCTTTTTTG

>Brazil-ND5-A12

CGATTTAATATTTTATTAGATAATTCTAAGTTAGGACAATTTTTATTATTAGTTTCTGGATTAACAATATTTATAGCTGGATTAGGGGCTAATTTTGAGTTTGATTTAAAAAAAATTATTGCTTTATCTACTTTAAGTCAATTAGGTTTAATAATAAGTATTTTATCTATTGGTTATTATAAGTTAGCTTTTTTTCATTTATTAACACATGCGCTCTTTAAGGCTTTATTATTTATATGTGCTGGAGTAATTATTCATAATACAAAAAATGCTCAAGATATTCGATTTATAGGGGGTTTAAGTATAAGAATACCTTTAACATGTAGTTGTTTTAATATTGCTAATTTAGCTTTATGTGGGATACCTTTTTTG

>Brazil-ND5-B09

CGATTTAATATTTTATTAGATAATTCTAAGTTAGGACAATTTTTATTATTAGTTTCTGGATTAACAATATTTATAGCTGGATTAGGGGCTAATTTTGAGTTTGATTTAAAAAAAATTATTGCTTTATCTACTTTAAGTCAATTAGGTTTAATAATAAGTATTTTATCTATTGGTTATTATAAGTTAGCTTTTTTTCATTTATTAACACATGCACTCTTTAAGGCTTTATTATTTATATGTGCTGGAGTAATTATTCATAATACAAAAAATGCTCAAGATATTCGATTTATAGGGGGTTTAAGTATAAGAATACCTTTAACATGTAGTTGTTTTAATATTGCTAATTTAGCTTTATGTGGAATACCTTTTTTG

>Brazil-ND5-B10

CGATTTAATATTTTATTAGATAATTCTAAGTTAGGACAATTTTTATTATTAGTTTCTGGATTAACAATATTTATAGCTGGATTAGGGGCTAATTTTGAGTTTGATTTAAAAAAAATTATTGCTTTATCTACTTTAAGTCAATTAGGTTTAATAATAAGTATTTTATCTATTGGTTATTATAAGTTAGCTTTTTTTCATTTATTAACACATGCACTCTTTAAGGCTTTATTATTTATATGTGCTGGAGTAATTATTCATAATACAAAAAATGCTCAAGATATTCGATTTATAGGGGGTTTAAGTATAAGAATACCTTTAACATGTAGTTGTTTTAATATTGCTAATTTAGCTTTATGTGGAATACCTTTTTTG

>Brazil-ND5-B11

CGATTTAATATTTTATTAGATAATTCTAAGTTAGGACAATTTTTATTATTAGTTTCTGGATTAACAATATTTATAGCTGGATTAGGGGCTAATTTTGAGTTTGATTTAAAAAAAATTATTGCTTTATCTACTTTAAGTCAATTAGGTTTAATAATAAGTATTTTATCTATTGGTTATTATAAGTTAGCTTTTTTTCATTTATTAACACATGCACTCTTTAAGGCTTTATTATTTATATGTGCTGGAGTAATTATTCATAATACAAAAAATGCTCAAGATATTCGATTTATAGGGGGTTTAAGTATAAGAATACCTTTAACATGTAGTTGTTTTAATATTGCTAATTTAGCTTTATGTGGAATACCTTTTTTG

>Brazil-ND5-B12

CGATTTAATATTTTATTAGATAATTCTAAGTTAGGACAATTTTTATTATTAGTTTCTGGATTAACAATATTTATAGCTGGATTAGGGGCTAATTTTGAGTTTGATTTAAAAAAAATTATTGCTTTATCTACTTTAAGTCAATTAGGTTTAATAATAAGTATTTTATCTATTGGTTATTATAAGTTAGCTTTTTTTCATTTATTAACACATGCACTCTTTAAGGCTTTATTATTTATATGTGCTGGAGTAATTATTCATAATACAAAAAATGCTCAAGATATTCGATTTATAGGGGGTTTAAGTATAAGAATACCTTTAACATGTAGTTGTTTTAATATTGCTAATTTAGCTTTATGTGGAATACCTTTTTTG

>Brazil-ND5-C09

CGATTTAATATTTTATTAGATAATTCTAAGTTAGGACAATTTTTATTATTAGTTTCTGGATTAACAATATTTATAGCTGGATTAGGGGCTAATTTTGAGTTTGATTTAAAAAAAATTATTGCTTTATCTACTTTAAGTCAATTAGGTTTAATAATAAGTATTTTATCTATTGGTTATTATAAGTTAGCTTTTTTTCATTTATTAACACATGCACTCTTTAAGGCTTTATTATTTATATGTGCTGGAGTAATTATTCATAATACAAAAAATGCTCAAGATATTCGATTTATAGGGGGTTTAAGTATAAGAATACCTTTAACATGTAGTTGTTTTAATATTGCTAATTTAGCTTTATGTGGAATACCTTTTTTG

>Brazil-ND5-C10

CGGTTTAATATTTTATTAGATAATTCTAAGTTAGGACAATTTTTATTATTAGTTTCTGGGTTAACAATATTTATAGCTGGATTAGGGGCTAATTTTGAGTTTGATTTAAAAAAAATTATTGCTTTATCTACTTTAAGTCAATTAGGTTTAATAATAAGTATTTTATCTATTGGTTATTATAAGTTAGCTTTTTTTCATTTATTAACACATGCACTCTTTAAGGCTTTATTATTTATATGTGCTGGGGTAATTATTCATAATACAAAAAATGCTCAAGATATTCGATTTATAGGAGGTTTAAGTATAAGAATACCTTTAACATGTAGTTGTTTTAATATTGCTAATTTAGCCTTATGTGGGATGCCTTTTTTG

>Brazil-ND5-C11

CGATTTAATATTTTATTAGATAATTCTAAGTTAGGACAATTTTTATTATTAGTTTCTGGATTAACAATATTTATAGCTGGATTAGGGGCTAATTTTGAGTTTGATTTAAAAAAAATTATTGCTTTATCTACTTTAAGTCAATTAGGTTTAATAATAAGTATTTTATCTATTGGTTATTATAAGTTAGCTTTTTTTCATTTATTAACACATGCACTCTTTAAGGCTTTATTATTTATATGTGCTGGAGTAATTATTCATAATACAAAAAATGCTCAAGATATTCGATTTATAGGGGGTTTAAGTATAAGAATACCTTTAACATGTAGTTGTTTTAATATTGCTAATTTAGCTTTATGTGGAATACCTTTTTTG

>Brazil-ND5-C12

CGATTTAATATTTTATTAGATAATTCTAAGTTAGGACAATTTTTATTATTAGTTTCTGGATTAACAATATTTATAGCTGGATTAGGGGCTAATTTTGAGTTTGATTTAAAAAAAATTATTGCTTTATCTACTTTAAGTCAATTAGGTTTAATAATAAGTATTTTATCTATTGGTTATTATAAGTTAGCTTTTTTTCATTTATTAACACATGCGCTCTTTAAGGCTTTATTATTTATATGTGCTGGAGTAATTATTCATAATACAAAAAATGCTCAAGATATTCGATTTATAGGGGGTTTAAGTATAAGAATACCTTTAACATGTAGTTGTTTTAATATTGCTAATTTAGCTTTATGTGGGATACCTTTTTTG

>Brazil-ND5-D09

CGATTTAATATTTTATTAGATAATTCTAAGTTAGGACAATTTTTATTATTAGTTTCTGGATTAACAATATTTATAGCTGGATTAGGGGCTAATTTTGAGTTTGATTTAAAAAAAATTATTGCTTTATCTACTTTAAGTCAATTAGGTTTAATAATAAGTATTTTATCTATTGGTTATTATAAGTTAGCTTTTTTTCATTTATTAACACATGCACTCTTTAAGGCTTTATTATTTATATGTGCTGGAGTAATTATTCATAATACAAAAAATGCTCAAGATATTCGATTTATAGGGGGTTTAAGTATAAGAATACCTTTAACATGTAGTTGTTTTAATATTGCTAATTTAGCTTTATGTGGAATACCTTTTTTG

>Brazil-ND5-D10

CGATTTAATATTTTATTAGATAATTCTAAGTTAGGACAATTTTTATTATTAGTTTCTGGATTAACAATATTTATAGCTGGATTAGGGGCTAATTTTGAGTTTGATTTAAAAAAAATTATTGCTTTATCTACTTTAAGTCAATTAGGTTTAATAATAAGTATTTTATCTATTGGTTATTATAAGTTAGCTTTTTTTCATTTATTAACACATGCACTCTTTAAGGCTTTATTATTTATATGTGCTGGAGTAATTATTCATAATACAAAAAATGCTCAAGATATTCGATTTATAGGGGGTTTAAGTATAAGAATACCTTTAACATGTAGTTGTTTTAATATTGCTAATTTAGCTTTATGTGGAATACCTTTTTTG

>Brazil-ND5-D11

CGGTTTAATATTTTATTAGATAATTCTAAGTTAGGACAATTTTTATTATTAGTTTCTGGGTTAACAATATTTATAGCTGGATTAGGGGCTAATTTTGAGTTTGATTTAAAAAAAATTATTGCTTTATCTACTTTAAGTCAATTAGGTTTAATAATAAGTATTTTATCTATTGGTTATTATAAGTTAGCTTTTTTTCATTTATTAACACATGCACTCTTTAAGGCTTTATTATTTATATGTGCTGGGGTAATTATTCATAATACAAAAAATGCTCAAGATATTCGATTTATAGGAGGTTTAAGTATAAGAATACCTTTAACATGTAGTTGTTTTAATATTGCTAATTTAGCCTTATGTGGGATGCCTTTTTTG

>Brazil-ND5-D12

CGATTTAATATTTTATTAGATAATTCTAAGTTAGGACAATTTTTATTATTAGTTTCTGGATTAACAATATTTATAGCTGGATTAGGGGCTAATTTTGAGTTTGATTTAAAAAAAATTATTGCTTTATCTACTTTAAGTCAATTAGGTTTAATAATAAGTATTTTATCTATTGGTTATTATAAGTTAGCTTTTTTTCATTTATTAACACATGCACTCTTTAAGGCTTTATTATTTATATGTGCTGGAGTAATTATTCATAATACAAAAAATGCTCAAGATATTCGATTTATAGGGGGTTTAAGTATAAGAATACCTTTAACATGTAGTTGTTTTAATATTGCTAATTTAGCTTTATGTGGAATACCTTTTTTG

>Brazil-ND5-E09

CGATTTAATATTTTATTAGATAATTCTAAGTTAGGACAATTTTTATTATTAGTTTCTGGATTAACAATATTTATAGCTGGATTAGGGGCTAATTTTGAGTTTGATTTAAAAAAAATTATTGCTTTATCTACTTTAAGTCAATTAGGTTTAATAATAAGTATTTTATCTATTGGTTATTATAAGTTAGCTTTTTTTCATTTATTAACACATGCACTCTTTAAGGCTTTATTATTTATATGTGCTGGAGTAATTATTCATAATACAAAAAATGCTCAAGATATTCGATTTATAGGGGGTTTAAGTATAAGAATACCTTTAACATGTAGTTGTTTTAATATTGCTAATTTAGCTTTATGTGGAATACCTTTTTTG

>Brazil-ND5-E10

CGATTTAATATTTTATTAGATAATTCTAAGTTAGGACAATTTTTATTATTAGTTTCTGGATTAACAATATTTATAGCTGGATTAGGGGCTAATTTTGAGTTTGATTTAAAAAAAATTATTGCTTTATCTACTTTAAGTCAATTAGGTTTAATAATAAGTATTTTATCTATTGGTTATTATAAGTTAGCTTTTTTTCATTTATTAACACATGCGCTCTTTAAGGCTTTATTATTTATATGTGCTGGAGTAATTATTCATAATACAAAAAATGCTCAAGATATTCGATTTATAGGGGGTTTAAGTATAAGAATACCTTTAACATGTAGTTGTTTTAATATTGCTAATTTAGCTTTATGTGGGATACCTTTTTTG

>Brazil-ND5-E11

CGATTTAATATTTTATTAGATAATTCTAAGTTAGGACAATTTTTATTATTAGTTTCTGGATTAACAATATTTATAGCTGGATTAGGGGCTAATTTTGAGTTTGATTTAAAAAAAATTATTGCTTTATCTACTTTAAGTCAATTAGGTTTAATAATAAGTATTTTATCTATTGGTTATTATAAGTTAGCTTTTTTTCATTTATTAACACATGCACTCTTTAAGGCTTTATTATTTATATGTGCTGGAGTAATTATTCATAATACAAAAAATGCTCAAGATATTCGATTTATAGGGGGTTTAAGTATAAGAATACCTTTAACATGTAGTTGTTTTAATATTGCTAATTTAGCTTTATGTGGAATACCTTTTTTG

>Brazil-ND5-E12

CGATTTAATATTTTATTAGATAATTCTAAGTTAGGACAATTTTTATTATTAGTTTCTGGATTAACAATATTTATAGCTGGATTAGGGGCTAATTTTGAGTTTGATTTAAAAAAAATTATTGCTTTATCTACTTTAAGTCAATTAGGTTTAATAATAAGTATTTTATCTATTGGTTATTATAAGTTAGCTTTTTTTCATTTATTAACACATGCACTCTTTAAGGCTTTATTATTTATATGTGCTGGAGTAATTATTCATAATACAAAAAATGCTCAAGATATTCGATTTATAGGGGGTTTAAGTATAAGAATACCTTTAACATGTAGTTGTTTTAATATTGCTAATTTAGCTTTATGTGGAATACCTTTTTTG

>Brazil-ND5-F09

CGATTTAATATTTTATTAGATAATTCTAAGTTAGGACAATTTTTATTATTAGTTTCTGGATTAACAATATTTATAGCTGGATTAGGGGCTAATTTTGAGTTTGATTTAAAAAAAATTATTGCTTTATCTACTTTAAGTCAATTAGGTTTAATAATAAGTATTTTATCTATTGGTTATTATAAGTTAGCTTTTTTTCATTTATTAACACATGCACTCTTTAAGGCTTTATTATTTATATGTGCTGGAGTAATTATTCATAATACAAAAAATGCTCAAGATATTCGATTTATAGGGGGTTTAAGTATAAGAATACCTTTAACATGTAGTTGTTTTAATATTGCTAATTTAGCTTTATGTGGAATACCTTTTTTG

>Brazil-ND5-F10

CGATTTAATATTTTATTAGATAATTCTAAGTTAGGACAATTTTTATTATTAGTTTCTGGATTAACAATATTTATAGCTGGATTAGGGGCTAATTTTGAGTTTGATTTAAAAAAAATTATTGCTTTATCTACTTTAAGTCAATTAGGTTTAATAATAAGTATTTTATCTATTGGTTATTATAAGTTAGCTTTTTTTCATTTATTAACACATGCACTCTTTAAGGCTTTATTATTTATATGTGCTGGAGTAATTATTCATAATACAAAAAATGCTCAAGATATTCGATTTATAGGGGGTTTAAGTATAAGAATACCTTTAACATGTAGTTGTTTTAATATTGCTAATTTAGCTTTATGTGGAATACCTTTTTTG

>Brazil-ND5-G07

CGATTTAATATTTTATTAGATAATTCTAAGTTAGGACAATTTTTATTATTAGTTTCTGGATTAACAATATTTATAGCTGGATTAGGGGCTAATTTTGAGTTTGATTTAAAAAAAATTATTGCTTTATCTACTTTAAGTCAATTAGGTTTAATAATAAGTATTTTATCTATTGGTTATTATAAGTTAGCTTTTTTTCATTTATTAACACATGCACTCTTTAAGGCTTTATTATTTATATGTGCTGGAGTAATTATTCATAATACAAAAAATGCTCAAGATATTCGATTTATAGGGGGTTTAAGTATAAGAATACCTTTAACATGTAGTTGTTTTAATATTGCTAATTTAGCTTTATGTGGAATACCTTTTTTG

>Brazil-ND5-G08

CGATTTAATATTTTATTAGATAATTCTAAGTTAGGACAATTTTTATTATTAGTTTCTGGATTAACAATATTTATAGCTGGATTAGGGGCTAATTTTGAGTTTGATTTAAAAAAAATTATTGCTTTATCTACTTTAAGTCAATTAGGTTTAATAATAAGTATTTTATCTATTGGTTATTATAAGTTAGCTTTTTTTCATTTATTAACACATGCACTCTTTAAGGCTTTATTATTTATATGTGCTGGAGTAATTATTCATAATACAAAAAATGCTCAAGATATTCGATTTATAGGGGGTTTAAGTATAAGAATACCTTTAACATGTAGTTGTTTTAATATTGCTAATTTAGCTTTATGTGGAATACCTTTTTTG

>Brazil-ND5-G09

CGATTTAATATTTTATTAGATAATTCTAAGTTAGGACAATTTTTATTATTAGTTTCTGGATTAACAATATTTATAGCTGGATTAGGGGCTAATTTTGAGTTTGATTTAAAAAAAATTATTGCTTTATCTACTTTAAGTCAATTAGGTTTAATAATAAGTATTTTATCTATTGGTTATTATAAGTTAGCTTTTTTTCATTTATTAACACATGCACTCTTTAAGGCTTTATTATTTATATGTGCTGGAGTAATTATTCATAATACAAAAAATGCTCAAGATATTCGATTTATAGGGGGTTTAAGTATAAGAATACCTTTAACATGTAGTTGTTTTAATATTGCTAATTTAGCTTTATGTGGAATACCTTTTTTG

>Brazil-ND5-G10

CGGTTTAATATTTTATTAGATAATTCTAAGTTAGGACAATTTTTATTATTAGTTTCTGGGTTAACAATATTTATAGCTGGATTAGGGGCTAATTTTGAGTTTGATTTAAAAAAAATTATTGCTTTATCTACTTTAAGTCAATTAGGTTTAATAATAAGTATTTTATCTATTGGTTATTATAAGTTAGCTTTTTTTCATTTATTAACACATGCACTCTTTAAGGCTTTATTATTTATATGTGCTGGGGTAATTATTCATAATACAAAAAATGCTCAAGATATTCGATTTATAGGAGGTTTAAGTATAAGAATACCTTTAACATGTAGTTGTTTTAATATTGCTAATTTAGCCTTATGTGGGATGCCTTTTTTG

>Brazil-ND5-H07

CGGTTTAATATTTTATTAGATAATTCTAAGTTAGGACAATTTTTATTATTAGTTTCTGGGTTAACAATATTTATAGCTGGATTAGGGGCTAATTTTGAGTTTGATTTAAAAAAAATTATTGCTTTATCTACTTTAAGTCAATTAGGTTTAATAATAAGTATTTTATCTATTGGTTATTATAAGTTAGCTTTTTTTCATTTATTAACACATGCACTCTTTAAGGCTTTATTATTTATATGTGCTGGGGTAATTATTCATAATACAAAAAATGCTCAAGATATTCGATTTATAGGAGGTTTAAGTATAAGAATACCTTTAACATGTAGTTGTTTTAATATTGCTAATTTAGCCTTATGTGGGATGCCTTTTTTG

>Brazil-ND5-H08

CGGTTTAATATTTTATTAGATAATTCTAAGTTAGGACAATTTTTATTATTAGTTTCTGGGTTAACAATATTTATAGCTGGATTAGGGGCTAATTTTGAGTTTGATTTAAAAAAAATTATTGCTTTATCTACTTTAAGTCAATTAGGTTTAATAATAAGTATTTTATCTATTGGTTATTATAAGTTAGCTTTTTTTCATTTATTAACACATGCACTCTTTAAGGCTTTATTATTTATATGTGCTGGGGTAATTATTCATAATACAAAAAATGCTCAAGATATTCGATTTATAGGAGGTTTAAGTATAAGAATACCTTTAACATGTAGTTGTTTTAATATTGCTAATTTAGCCTTATGTGGGATGCCTTTTTTG

>Brazil-ND5-H09

CGATTTAATATTTTATTAGATAATTCTAAGTTAGGACAATTTTTATTATTAGTTTCTGGATTAACAATATTTATAGCTGGATTAGGGGCTAATTTTGAGTTTGATTTAAAAAAAATTATTGCTTTATCTACTTTAAGTCAATTAGGTTTAATAATAAGTATTTTATCTATTGGTTATTATAAGTTAGCTTTTTTTCATTTATTAACACATGCGCTCTTTAAGGCTTTATTATTTATATGTGCTGGAGTAATTATTCATAATACAAAAAATGCTCAAGATATTCGATTTATAGGGGGTTTAAGTATAAGAATACCTTTAACATGTAGTTGTTTTAATATTGCTAATTTAGCTTTATGTGGGATACCTTTTTTG

>Brazil-ND5-H10

CGATTTAATATTTTATTAGATAATTCTAAGTTAGGACAATTTTTATTATTAGTTTCTGGATTAACAATATTTATAGCTGGATTAGGGGCTAATTTTGAGTTTGATTTAAAAAAAATTATTGCTTTATCTACTTTAAGTCAATTAGGTTTAATAATAAGTATTTTATCTATTGGTTATTATAAGTTAGCTTTTTTTCATTTATTAACACATGCACTCTTTAAGGCTTTATTATTTATATGTGCTGGAGTAATTATTCATAATACAAAAAATGCTCAAGATATTCGATTTATAGGGGGTTTAAGTATAAGAATACCTTTAACATGTAGTTGTTTTAATATTGCTAATTTAGCTTTATGTGGAATACCTTTTTTG

>Australia-ND5-A01

CGGTTTAATATTTTATTAGATAATTCTAAGTTAGGACAATTTTTATTATTAGTTTCTGGGTTAACAATATTTATAGCTGGATTAGGGGCTAATTTTGAGTTTGATTTAAAAAAAATTATTGCTTTATCTACTTTAAGTCAATTAGGTTTAATAATAAGTATTTTATCTATTGGTTATTATAAGTTAGCTTTTTTTCATTTATTAACACATGCACTTTTTAAGGCTTTATTATTTATATGTGCTGGGGTAATTATTCATAATACAAAAAATGCTCAAGATATTCGATTTATAGGGGGTTTAAGTATAAGAATACCTTTAACATGTAGTTGTTTTAATATTGCTAATTTAGCCTTATGTGGGATGCCTTTTTTG

>Australia-ND5-A02

CGGTTTAATATTTTATTAGATAATTCTAAGTTAGGACAATTTTTATTATTAGTTTCTGGGTTAACAATATTTATAGCTGGATTAGGGGCTAATTTTGAGTTTGATTTAAAAAAAATTATTGCTTTATCTACTTTAAGTCAATTAGGTTTAATAATAAGTATTTTATCTATTGGTTATTATAAGTTAGCTTTTTTTCATTTATTAACACATGCACTTTTTAAGGCTTTATTATTTATATGTGCTGGGGTAATTATTCATAATACAAAAAATGCTCAAGATATTCGATTTATAGGGGGTTTAAGTATAAGAATACCTTTAACATGTAGTTGTTTTAATATTGCTAATTTAGCCTTATGTGGGATGCCTTTTTTG

>Australia-ND5-A04

CGGTTTAATATTTTATTAGATAATTCTAAGTTAGGACAATTTTTATTATTAGTTTCTGGGTTAACAATATTTATAGCTGGATTAGGGGCTAATTTTGAGTTTGATTTAAAAAAAATTATTGCTTTATCTACTTTAAGTCAATTAGGTTTAATAATAAGTATTTTATCTATTGGTTATTATAAGTTAGCTTTTTTTCATTTATTAACACATGCACTTTTTAAGGCTTTATTATTTATATGTGCTGGGGTAATTATTCATAATACAAAAAATGCTCAAGATATTCGATTTATAGGGGGTTTAAGTATAAGAATACCTTTAACATGTAGTTGTTTTAATATTGCTAATTTAGCCTTATGTGGGATGCCTTTTTTG

>Australia-ND5-A06

CGGTTTAATATTTTATTAGATAATTCTAAGTTAGGACAATTTTTATTATTAGTTTCTGGGTTAACAATATTTATAGCTGGATTAGGGGCTAATTTTGAGTTTGATTTAAAAAAAATTATTGCTTTATCTACTTTAAGTCAATTAGGTTTAATAATAAGTATTTTATCTATTGGTTATTATAAGTTAGCTTTTTTTCATTTATTAACACATGCACTTTTTAAGGCTTTATTATTTATATGTGCTGGGGTAATTATTCATAATACAAAAAATGCTCAAGATATTCGATTTATAGGGGGTTTAAGTATAAGAATACCTTTAACATGTAGTTGTTTTAATATTGCTAATTTAGCCTTATGTGGGATGCCTTTTTTG

>Australia-ND5-A07

CGGTTTAATATTTTATTAGATAATTCTAAGTTAGGACAATTTTTATTATTAGTTTCTGGGTTAACAATATTTATAGCTGGATTAGGGGCTAATTTTGAGTTTGATTTAAAAAAAATTATTGCTTTATCTACTTTAAGTCAATTAGGTTTAATAATAAGTATTTTATCTATTGGTTATTATAAGTTAGCTTTTTTTCATTTATTAACACATGCACTTTTTAAGGCTTTATTATTTATATGTGCTGGGGTAATTATTCATAATACAAAAAATGCTCAAGATATTCGATTTATAGGGGGTTTAAGTATAAGAATACCTTTAACATGTAGTTGTTTTAATATTGCTAATTTAGCCTTATGTGGGATGCCTTTTTTG

>Australia-ND5-A08

CGGTTTAATATTTTATTAGATAATTCTAAGTTAGGACAATTTTTATTATTAGTTTCTGGGTTAACAATATTTATAGCTGGATTAGGGGCTAATTTTGAGTTTGATTTAAAAAAAATTATTGCTTTATCTACTTTAAGTCAATTAGGTTTAATAATAAGTATTTTATCTATTGGTTATTATAAGTTAGCTTTTTTTCATTTATTAACACATGCACTTTTTAAGGCTTTATTATTTATATGTGCTGGGGTAATTATTCATAATACAAAAAATGCTCAAGATATTCGATTTATAGGGGGTTTAAGTATAAGAATACCTTTAACATGTAGTTGTTTTAATATTGCTAATTTAGCCTTATGTGGGATGCCTTTTTTG

>Australia-ND5-A09

CGGTTTAATATTTTATTAGATAATTCTAAGTTAGGACAATTTTTATTATTAGTTTCTGGGTTAACAATATTTATAGCTGGATTAGGGGCTAATTTTGAGTTTGATTTAAAAAAAATTATTGCTTTATCTACTTTAAGTCAATTAGGTTTAATAATAAGTATTTTATCTATTGGTTATTATAAGTTAGCTTTTTTTCATTTATTAACACATGCACTTTTTAAGGCTTTATTATTTATATGTGCTGGGGTAATTATTCATAATACAAAAAATGCTCAAGATATTCGATTTATAGGGGGTTTAAGTATAAGAATACCTTTAACATGTAGTTGTTTTAATATTGCTAATTTAGCCTTATGTGGGATGCCTTTTTTG

>Australia-ND5-A10

CGGTTTAATATTTTATTAGATAATTCTAAGTTAGGACAATTTTTATTATTAGTTTCTGGGTTAACAATATTTATAGCTGGATTAGGGGCTAATTTTGAGTTTGATTTAAAAAAAATTATTGCTTTATCTACTTTAAGTCAATTAGGTTTAATAATAAGTATTTTATCTATTGGTTATTATAAGTTAGCTTTTTTTCATTTATTAACACATGCACTTTTTAAGGCTTTATTATTTATATGTGCTGGGGTAATTATTCATAATACAAAAAATGCTCAAGATATTCGATTTATAGGGGGTTTAAGTATAAGAATACCTTTAACATGTAGTTGTTTTAATATTGCTAATTTAGCCTTATGTGGGATGCCTTTTTTG

>Australia-ND5-A11

CGGTTTAATATTTTATTAGATAATTCTAAGTTAGGACAATTTTTATTATTAGTTTCTGGGTTAACAATATTTATAGCTGGATTAGGGGCTAATTTTGAGTTTGATTTAAAAAAAATTATTGCTTTATCTACTTTAAGTCAATTAGGTTTAATAATAAGTATTTTATCTATTGGTTATTATAAGTTAGCTTTTTTTCATTTATTAACACATGCACTTTTTAAGGCTTTATTATTTATATGTGCTGGGGTAATTATTCATAATACAAAAAATGCTCAAGATATTCGATTTATAGGGGGTTTAAGTATAAGAATACCTTTAACATGTAGTTGTTTTAATATTGCTAATTTAGCCTTATGTGGGATGCCTTTTTTG

>Australia-ND5-A13

CGGTTTAATATTTTATTAGATAATTCTAAGTTAGGACAATTTTTATTATTAGTTTCTGGGTTAACAATATTTATAGCTGGATTAGGGGCTAATTTTGAGTTTGATTTAAAAAAAATTATTGCTTTATCTACTTTAAGTCAATTAGGTTTAATAATAAGTATTTTATCTATTGGTTATTATAAGTTAGCTTTTTTTCATTTATTAACACATGCACTTTTTAAGGCTTTATTATTTATATGTGCTGGGGTAATTATTCATAATACAAAAAATGCTCAAGATATTCGATTTATAGGGGGTTTAAGTATAAGAATACCTTTAACATGTAGTTGTTTTAATATTGCTAATTTAGCCTTATGTGGGATGCCTTTTTTG

>Australia-ND5-A14

CGGTTTAATATTTTATTAGATAATTCTAAGTTAGGACAATTTTTATTATTAGTTTCTGGGTTAACAATATTTATAGCTGGATTAGGGGCTAATTTTGAGTTTGATTTAAAAAAAATTATTGCTTTATCTACTTTAAGTCAATTAGGTTTAATAATAAGTATTTTATCTATTGGTTATTATAAGTTAGCTTTTTTTCATTTATTAACACATGCACTTTTTAAGGCTTTATTATTTATATGTGCTGGGGTAATTATTCATAATACAAAAAATGCTCAAGATATTCGATTTATAGGGGGTTTAAGTATAAGAATACCTTTAACATGTAGTTGTTTTAATATTGCTAATTTAGCCCTATGTGGGATGCCTTTTTTG

>Australia-ND5-A15

CGGTTTAATATTTTATTAGATAATTCTAAGTTAGGACAATTTTTATTATTAGTTTCTGGGTTAACAATATTTATAGCTGGATTAGGGGCTAATTTTGAGTTTGATTTAAAAAAAATTATTGCTTTATCTACTTTAAGTCAATTAGGTTTAATAATAAGTATTTTATCTATTGGTTATTATAAGTTAGCTTTTTTTCATTTATTAACACATGCACTTTTTAAGGCTTTATTATTTATATGTGCTGGGGTAATTATTCATAATACAAAAAATGCTCAAGATATTCGATTTATAGGGGGTTTAAGTATAAGAATACCTTTAACATGTAGTTGTTTTAATATTGCTAATTTAGCCCTATGTGGGATGCCTTTTTTG

>Australia-ND5-A16

CGGTTTAATATTTTATTAGATAATTCTAAGTTAGGACAATTTTTATTATTAGTTTCTGGGTTAACAATATTTATAGCTGGATTAGGGGCTAATTTTGAGTTTGATTTAAAAAAAATTATTGCTTTATCTACTTTAAGTCAATTAGGTTTAATAATAAGTATTTTATCTATTGGTTATTATAAGTTAGCTTTTTTTCATTTATTAACACATGCACTTTTTAAGGCTTTATTATTTATATGTGCTGGGGTAATTATTCATAATACAAAAAATGCTCAAGATATTCGATTTATAGGGGGTTTAAGTATAAGAATACCTTTAACATGTAGTTGTTTTAATATTGCTAATTTAGCCCTATGTGGGATGCCTTTTTTG

>Australia-ND5-A17

CGGTTTAATATTTTATTAGATAATTCTAAGTTAGGACAATTTTTATTATTAGTTTCTGGGTTAACAATATTTATAGCTGGATTAGGGGCTAATTTTGAGTTTGATTTAAAAAAAATTATTGCTTTATCTACTTTAAGTCAATTAGGTTTAATAATAAGTATTTTATCTATTGGTTATTATAAGTTAGCTTTTTTTCATTTATTAACACATGCACTTTTTAAGGCTTTATTATTTATATGTGCTGGGGTAATTATTCATAATACAAAAAATGCTCAAGATATTCGATTTATAGGGGGTTTAAGTATAAGAATACCTTTAACATGTAGTTGTTTTAATATTGCTAATTTAGCCTTATGTGGGATGCCTTTTTTG

>Australia-ND5-A18

CGGTTTAATATTTTATTAGATAATTCTAAGTTAGGACAATTTTTATTATTAGTTTCTGGGTTAACAATATTTATAGCTGGATTAGGGGCTAATTTTGAGTTTGATTTAAAAAAAATTATTGCTTTATCTACTTTAAGTCAATTAGGTTTAATAATAAGTATTTTATCTATTGGTTATTATAAGTTAGCTTTTTTTCATTTATTAACACATGCACTTTTTAAGGCTTTATTATTTATATGTGCTGGGGTAATTATTCATAATACAAAAAATGCTCAAGATATTCGATTTATAGGGGGTTTAAGTATAAGAATACCTTTAACATGTAGTTGTTTTAATATTGCTAATTTAGCCTTATGTGGGATGCCTTTTTTG

>Australia-ND5-A19

CGGTTTAATATTTTATTAGATAATTCTAAGTTAGGACAATTTTTATTATTAGTTTCTGGGTTAACAATATTTATAGCTGGATTAGGGGCTAATTTTGAGTTTGATTTAAAAAAAATTATTGCTTTATCTACTTTAAGTCAATTAGGTTTAATAATAAGTATTTTATCTATTGGTTATTATAAGTTAGCTTTTTTTCATTTATTAACACATGCACTTTTTAAGGCTTTATTATTTATATGTGCTGGGGTAATTATTCATAATACAAAAAATGCTCAAGATATTCGATTTATAGGGGGTTTAAGTATAAGAATACCTTTAACATGTAGTTGTTTTAATATTGCTAATTTAGCCTTATGTGGGATGCCTTTTTTG

>Australia-ND5-A20

CGGTTTAATATTTTATTAGATAATTCTAAGTTAGGACAATTTTTATTATTAGTTTCTGGGTTAACAATATTTATAGCTGGATTAGGGGCTAATTTTGAGTTTGATTTAAAAAAAATTATTGCTTTATCTACTTTAAGTCAATTAGGTTTAATAATAAGTATTTTATCTATTGGTTATTATAAGTTAGCTTTTTTTCATTTATTAACACATGCACTTTTTAAGGCTTTATTATTTATATGTGCTGGGGTAATTATTCATAATACAAAAAATGCTCAAGATATTCGATTTATAGGGGGTTTAAGTATAAGAATACCTTTAACATGTAGTTGTTTTAATATTGCTAATTTAGCCTTATGTGGGATGCCTTTTTTG

>Australia-ND5-A21

CGGTTTAATATTTTATTAGATAATTCTAAGTTAGGACAATTTTTATTATTAGTTTCTGGGTTAACAATATTTATAGCTGGATTAGGGGCTAATTTTGAGTTTGATTTAAAAAAAATTATTGCTTTATCTACTTTAAGTCAATTAGGTTTAATAATAAGTATTTTATCTATTGGTTATTATAAGTTAGCTTTTTTTCATTTATTAACACATGCACTTTTTAAGGCTTTATTATTTATATGTGCTGGGGTAATTATTCATAATACAAAAAATGCTCAAGATATTCGATTTATAGGGGGTTTAAGTATAAGAATACCTTTAACATGTAGTTGTTTTAATATTGCTAATTTAGCCTTATGTGGGATGCCTTTTTTG

>Australia-ND5-A22

CGGTTTAATATTTTATTAGATAATTCTAAGTTAGGACAATTTTTATTATTAGTTTCTGGGTTAACAATATTTATAGCTGGATTAGGGGCTAATTTTGAGTTTGATTTAAAAAAAATTATTGCTTTATCTACTTTAAGTCAATTAGGTTTAATAATAAGTATTTTATCTATTGGTTATTATAAGTTAGCTTTTTTTCATTTATTAACACATGCACTTTTTAAGGCTTTATTATTTATATGTGCTGGGGTAATTATTCATAATACAAAAAATGCTCAAGATATTCGATTTATAGGGGGTTTAAGTATAAGAATACCTTTAACATGTAGTTGTTTTAATATTGCTAATTTAGCCTTATGTGGGATGCCTTTTTTG

>Australia-ND5-A23

CGGTTTAATATTTTATTAGATAATTCTAAGTTAGGACAATTTTTATTATTAGTTTCTGGGTTAACAATATTTATAGCTGGATTAGGGGCTAATTTTGAGTTTGATTTAAAAAAAATTATTGCTTTATCTACTTTAAGTCAATTAGGTTTAATAATAAGTATTTTATCTATTGGTTATTATAAGTTAGCTTTTTTTCATTTATTAACACATGCACTTTTTAAGGCTTTATTATTTATATGTGCTGGGGTAATTATTCATAATACAAAAAATGCTCAAGATATTCGATTTATAGGGGGTTTAAGTATAAGAATACCTTTAACATGTAGTTGTTTTAATATTGCTAATTTAGCCTTATGTGGGATGCCTTTTTTG

>Australia-ND5-A24

CGGTTTAATATTTTATTAGATAATTCTAAGTTAGGACAATTTTTATTATTAGTTTCTGGGTTAACAATATTTATAGCTGGATTAGGGGCTAATTTTGAGTTTGATTTAAAAAAAATTATTGCTTTATCTACTTTAAGTCAATTAGGTTTAATAATAAGTATTTTATCTATTGGTTATTATAAGTTAGCTTTTTTTCATTTATTAACACATGCACTTTTTAAGGCTTTATTATTTATATGTGCTGGGGTAATTATTCATAATACAAAAAATGCTCAAGATATTCGATTTATAGGGGGTTTAAGTATAAGAATACCTTTAACATGTAGTTGTTTTAATATTGCTAATTTAGCCCTATGTGGGATGCCTTTTTTG

>Australia-ND5-A25

CGGTTTAATATTTTATTAGATAATTCTAAGTTAGGACAATTTTTATTATTAGTTTCTGGGTTAACAATATTTATAGCTGGATTAGGGGCTAATTTTGAGTTTGATTTAAAAAAAATTATTGCTTTATCTACTTTAAGTCAATTAGGTTTAATAATAAGTATTTTATCTATTGGTTATTATAAGTTAGCTTTTTTTCATTTATTAACACATGCACTTTTTAAGGCTTTATTATTTATATGTGCTGGGGTAATTATTCATAATACAAAAAATGCTCAAGATATTCGATTTATAGGGGGTTTAAGTATAAGAATACCTTTAACATGTAGTTGTTTTAATATTGCTAATTTAGCCTTATGTGGGATGCCTTTTTTG

>Australia-ND5-A26

CGGTTTAATATTTTATTAGATAATTCTAAGTTAGGACAATTTTTATTATTAGTTTCTGGGTTAACAATATTTATAGCTGGATTAGGGGCTAATTTTGAGTTTGATTTAAAAAAAATTATTGCTTTATCTACTTTAAGTCAATTAGGTTTAATAATAAGTATTTTATCTATTGGTTATTATAAGTTAGCTTTTTTTCATTTATTAACACATGCACTTTTTAAGGCTTTATTATTTATATGTGCTGGGGTAATTATTCATAATACAAAAAATGCTCAAGATATTCGATTTATAGGGGGTTTAAGTATAAGAATACCTTTAACATGTAGTTGTTTTAATATTGCTAATTTAGCCTTATGTGGGATGCCTTTTTTG

>Australia-ND5-A27

CGGTTTAATATTTTATTAGATAATTCTAAGTTAGGACAATTTTTATTATTAGTTTCTGGGTTAACAATATTTATAGCTGGATTAGGGGCTAATTTTGAGTTTGATTTAAAAAAAATTATTGCTTTATCTACTTTAAGTCAATTAGGTTTAATAATAAGTATTTTATCTATTGGTTATTATAAGTTAGCTTTTTTTCATTTATTAACACATGCACTTTTTAAGGCTTTATTATTTATATGTGCTGGGGTAATTATTCATAATACAAAAAATGCTCAAGATATTCGATTTATAGGGGGTTTAAGTATAAGAATACCTTTAACATGTAGTTGTTTTAATATTGCTAATTTAGCCCTATGTGGGATGCCTTTTTTG

>Australia-ND5-A28

CGGTTTAATATTTTATTAGATAATTCTAAGTTAGGACAATTTTTATTATTAGTTTCTGGGTTAACAATATTTATAGCTGGATTAGGGGCTAATTTTGAGTTTGATTTAAAAAAAATTATTGCTTTATCTACTTTAAGTCAATTAGGTTTAATAATAAGTATTTTATCTATTGGTTATTATAAGTTAGCTTTTTTTCATTTATTAACACATGCACTTTTTAAGGCTTTATTATTTATATGTGCTGGGGTAATTATTCATAATACAAAAAATGCTCAAGATATTCGATTTATAGGGGGTTTAAGTATAAGAATACCTTTAACATGTAGTTGTTTTAATATTGCTAATTTAGCCTTATGTGGGATGCCTTTTTTG

>Australia-ND5-A29

CGGTTTAATATTTTATTAGATAATTCTAAGTTAGGACAATTTTTATTATTAGTTTCTGGGTTAACAATATTTATAGCTGGATTAGGGGCTAATTTTGAGTTTGATTTAAAAAAAATTATTGCTTTATCTACTTTAAGTCAATTAGGTTTAATAATAAGTATTTTATCTATTGGTTATTATAAGTTAGCTTTTTTTCATTTATTAACACATGCACTTTTTAAGGCTTTATTATTTATATGTGCTGGGGTAATTATTCATAATACAAAAAATGCTCAAGATATTCGATTTATAGGGGGTTTAAGTATAAGAATACCTTTAACATGTAGTTGTTTTAATATTGCTAATTTAGCCCTATGTGGGATGCCTTTTTTG

>Australia-ND5-A30

CGGTTTAATATTTTATTAGATAATTCTAAGTTAGGACAATTTTTATTATTAGTTTCTGGGTTAACAATATTTATAGCTGGATTAGGGGCTAATTTTGAGTTTGATTTAAAAAAAATTATTGCTTTATCTACTTTAAGTCAATTAGGTTTAATAATAAGTATTTTATCTATTGGTTATTATAAGTTAGCTTTTTTTCATTTATTAACACATGCACTTTTTAAGGCTTTATTATTTATATGTGCTGGGGTAATTATTCATAATACAAAAAATGCTCAAGATATTCGATTTATAGGGGGTTTAAGTATAAGAATACCTTTAACATGTAGTTGTTTTAATATTGCTAATTTAGCCTTATGTGGGATGCCTTTTTTG

>Australia-ND5-A31

CGGTTTAATATTTTATTAGATAATTCTAAGTTAGGACAATTTTTATTATTAGTTTCTGGGTTAACAATATTTATAGCTGGATTAGGGGCTAATTTTGAGTTTGATTTAAAAAAAATTATTGCTTTATCTACTTTAAGTCAATTAGGTTTAATAATAAGTATTTTATCTATTGGTTATTATAAGTTAGCTTTTTTTCATTTATTAACACATGCACTTTTTAAGGCTTTATTATTTATATGTGCTGGGGTAATTATTCATAATACAAAAAATGCTCAAGATATTCGATTTATAGGGGGTTTAAGTATAAGAATACCTTTAACATGTAGTTGTTTTAATATTGCTAATTTAGCCCTATGTGGGATGCCTTTTTTG

>Australia-ND5-A32

CGGTTTAATATTTTATTAGATAATTCTAAGTTAGGACAATTTTTATTATTAGTTTCTGGGTTAACAATATTTATAGCTGGATTAGGGGCTAATTTTGAGTTTGATTTAAAAAAAATTATTGCTTTATCTACTTTAAGTCAATTAGGTTTAATAATAAGTATTTTATCTATTGGTTATTATAAGTTAGCTTTTTTTCATTTATTAACACATGCACTTTTTAAGGCTTTATTATTTATATGTGCTGGGGTAATTATTCATAATACAAAAAATGCTCAAGATATTCGATTTATAGGGGGTTTAAGTATAAGAATACCTTTAACATGTAGTTGTTTTAATATTGCTAATTTAGCCTTATGTGGGATGCCTTTTTTG

>Australia-ND5-A33

CGGTTTAATATTTTATTAGATAATTCTAAGTTAGGACAATTTTTATTATTAGTTTCTGGGTTAACAATATTTATAGCTGGATTAGGGGCTAATTTTGAGTTTGATTTAAAAAAAATTATTGCTTTATCTACTTTAAGTCAATTAGGTTTAATAATAAGTATTTTATCTATTGGTTATTATAAGTTAGCTTTTTTTCATTTATTAACACATGCACTTTTTAAGGCTTTATTATTTATATGTGCTGGGGTAATTATTCATAATACAAAAAATGCTCAAGATATTCGATTTATAGGGGGTTTAAGTATAAGAATACCTTTAACATGTAGTTGTTTTAATATTGCTAATTTAGCCCTATGTGGGATGCCTTTTTTG

>Australia-ND5-A34

CGGTTTAATATTTTATTAGATAATTCTAAGTTAGGACAATTTTTATTATTAGTTTCTGGGTTAACAATATTTATAGCTGGATTAGGGGCTAATTTTGAGTTTGATTTAAAAAAAATTATTGCTTTATCTACTTTAAGTCAATTAGGTTTAATAATAAGTATTTTATCTATTGGTTATTATAAGTTAGCTTTTTTTCATTTATTAACACATGCACTTTTTAAGGCTTTATTATTTATATGTGCTGGGGTAATTATTCATAATACAAAAAATGCTCAAGATATTCGATTTATAGGGGGTTTAAGTATAAGAATACCTTTAACATGTAGTTGTTTTAATATTGCTAATTTAGCCTTATGTGGGATGCCTTTTTTG

>Australia-ND5-A35

CGGTTTAATATTTTATTAGATAATTCTAAGTTAGGACAATTTTTATTATTAGTTTCTGGGTTAACAATATTTATAGCTGGATTAGGGGCTAATTTTGAGTTTGATTTAAAAAAAATTATTGCTTTATCTACTTTAAGTCAATTAGGTTTAATAATAAGTATTTTATCTATTGGTTATTATAAGTTAGCTTTTTTTCATTTATTAACACATGCACTTTTTAAGGCTTTATTATTTATATGTGCTGGGGTAATTATTCATAATACAAAAAATGCTCAAGATATTCGATTTATAGGGGGTTTAAGTATAAGAATACCTTTAACATGTAGTTGTTTTAATATTGCTAATTTAGCCTTATGTGGGATGCCTTTTTTG

>Australia-ND5-A36

CGGTTTAATATTTTATTAGATAATTCTAAGTTAGGACAATTTTTATTATTAGTTTCTGGGTTAACAATATTTATAGCTGGATTAGGGGCTAATTTTGAGTTTGATTTAAAAAAAATTATTGCTTTATCTACTTTAAGTCAATTAGGTTTAATAATAAGTATTTTATCTATTGGTTATTATAAGTTAGCTTTTTTTCATTTATTAACACATGCACTTTTTAAGGCTTTATTATTTATATGTGCTGGGGTAATTATTCATAATACAAAAAATGCTCAAGATATTCGATTTATAGGGGGTTTAAGTATAAGAATACCTTTAACATGTAGTTGTTTTAATATTGCTAATTTAGCCCTATGTGGGATGCCTTTTTTG

>Australia-ND5-A37

CGGTTTAATATTTTATTAGATAATTCTAAGTTAGGACAATTTTTATTATTAGTTTCTGGGTTAACAATATTTATAGCTGGATTAGGGGCTAATTTTGAGTTTGATTTAAAAAAAATTATTGCTTTATCTACTTTAAGTCAATTAGGTTTAATAATAAGTATTTTATCTATTGGTTATTATAAGTTAGCTTTTTTTCATTTATTAACACATGCACTTTTTAAGGCTTTATTATTTATATGTGCTGGGGTAATTATTCATAATACAAAAAATGCTCAAGATATTCGATTTATAGGGGGTTTAAGTATAAGAATACCTTTAACATGTAGTTGTTTTAATATTGCTAATTTAGCCCTATGTGGGATGCCTTTTTTG

>Australia-ND5-A38

CGGTTTAATATTTTATTAGATAATTCTAAGTTAGGACAATTTTTATTATTAGTTTCTGGGTTAACAATATTTATAGCTGGATTAGGGGCTAATTTTGAGTTTGATTTAAAAAAAATTATTGCTTTATCTACTTTAAGTCAATTAGGTTTAATAATAAGTATTTTATCTATTGGTTATTATAAGTTAGCTTTTTTTCATTTATTAACACATGCACTTTTTAAGGCTTTATTATTTATATGTGCTGGGGTAATTATTCATAATACAAAAAATGCTCAAGATATTCGATTTATAGGGGGTTTAAGTATAAGAATACCTTTAACATGTAGTTGTTTTAATATTGCTAATTTAGCCTTATGTGGGATGCCTTTTTTG

>Australia-ND5-A39

CGGTTTAATATTTTATTAGATAATTCTAAGTTAGGACAATTTTTATTATTAGTTTCTGGGTTAACAATATTTATAGCTGGATTAGGGGCTAATTTTGAGTTTGATTTAAAAAAAATTATTGCTTTATCTACTTTAAGTCAATTAGGTTTAATAATAAGTATTTTATCTATTGGTTATTATAAGTTAGCTTTTTTTCATTTATTAACACATGCACTTTTTAAGGCTTTATTATTTATATGTGCTGGGGTAATTATTCATAATACAAAAAATGCTCAAGATATTCGATTTATAGGGGGTTTAAGTATAAGAATACCTTTAACATGTAGTTGTTTTAATATTGCTAATTTAGCCTTATGTGGGATGCCTTTTTTG

>Australia-ND5-A40

CGGTTTAATATTTTATTAGATAATTCTAAGTTAGGACAATTTTTATTATTAGTTTCTGGGTTAACAATATTTATAGCTGGATTAGGGGCTAATTTTGAGTTTGATTTAAAAAAAATTATTGCTTTATCTACTTTAAGTCAATTAGGTTTAATAATAAGTATTTTATCTATTGGTTATTATAAGTTAGCTTTTTTTCATTTATTAACACATGCACTTTTTAAGGCTTTATTATTTATATGTGCTGGGGTAATTATTCATAATACAAAAAATGCTCAAGATATTCGATTTATAGGGGGTTTAAGTATAAGAATACCTTTAACATGTAGTTGTTTTAATATTGCTAATTTAGCCTTATGTGGGATGCCTTTTTTG

>Australia-ND5-A41

CGGTTTAATATTTTATTAGATAATTCTAAGTTAGGACAATTTTTATTATTAGTTTCTGGGTTAACAATATTTATAGCTGGATTAGGGGCTAATTTTGAGTTTGATTTAAAAAAAATTATTGCTTTATCTACTTTAAGTCAATTAGGTTTAATAATAAGTATTTTATCTATTGGTTATTATAAGTTAGCTTTTTTTCATTTATTAACACATGCACTTTTTAAGGCTTTATTATTTATATGTGCTGGGGTAATTATTCATAATACAAAAAATGCTCAAGATATTCGATTTATAGGGGGTTTAAGTATAAGAATACCTTTAACATGTAGTTGTTTTAATATTGCTAATTTAGCCTTATGTGGGATGCCTTTTTTG

>Australia-ND5-A42

CGGTTTAATATTTTATTAGATAATTCTAAGTTAGGACAATTTTTATTATTAGTTTCTGGGTTAACAATATTTATAGCTGGATTAGGGGCTAATTTTGAGTTTGATTTAAAAAAAATTATTGCTTTATCTACTTTAAGTCAATTAGGTTTAATAATAAGTATTTTATCTATTGGTTATTATAAGTTAGCTTTTTTTCATTTATTAACACATGCACTTTTTAAGGCTTTATTATTTATATGTGCTGGGGTAATTATTCATAATACAAAAAATGCTCAAGATATTCGATTTATAGGGGGTTTAAGTATAAGAATACCTTTAACATGTAGTTGTTTTAATATTGCTAATTTAGCCCTATGTGGGATGCCTTTTTTG

>Australia-ND5-A43

CGGTTTAATATTTTATTAGATAATTCTAAGTTAGGACAATTTTTATTATTAGTTTCTGGGTTAACAATATTTATAGCTGGATTAGGGGCTAATTTTGAGTTTGATTTAAAAAAAATTATTGCTTTATCTACTTTAAGTCAATTAGGTTTAATAATAAGTATTTTATCTATTGGTTATTATAAGTTAGCTTTTTTTCATTTATTAACACATGCACTTTTTAAGGCTTTATTATTTATATGTGCTGGGGTAATTATTCATAATACAAAAAATGCTCAAGATATTCGATTTATAGGGGGTTTAAGTATAAGAATACCTTTAACATGTAGTTGTTTTAATATTGCTAATTTAGCCTTATGTGGGATGCCTTTTTTG

>Australia-ND5-A44

CGGTTTAATATTTTATTAGATAATTCTAAGTTAGGACAATTTTTATTATTAGTTTCTGGGTTAACAATATTTATAGCTGGATTAGGGGCTAATTTTGAGTTTGATTTAAAAAAAATTATTGCTTTATCTACTTTAAGTCAATTAGGTTTAATAATAAGTATTTTATCTATTGGTTATTATAAGTTAGCTTTTTTTCATTTATTAACACATGCACTTTTTAAGGCTTTATTATTTATATGTGCTGGGGTAATTATTCATAATACAAAAAATGCTCAAGATATTCGATTTATAGGGGGTTTAAGTATAAGAATACCTTTAACATGTAGTTGTTTTAATATTGCTAATTTAGCCTTATGTGGGATGCCTTTTTTG

>Australia-ND5-A45

CGGTTTAATATTTTATTAGATAATTCTAAGTTAGGACAATTTTTATTATTAGTTTCTGGGTTAACAATATTTATAGCTGGATTAGGGGCTAATTTTGAGTTTGATTTAAAAAAAATTATTGCTTTATCTACTTTAAGTCAATTAGGTTTAATAATAAGTATTTTATCTATTGGTTATTATAAGTTAGCTTTTTTTCATTTATTAACACATGCACTTTTTAAGGCTTTATTATTTATATGTGCTGGGGTAATTATTCATAATACAAAAAATGCTCAAGATATTCGATTTATAGGGGGTTTAAGTATAAGAATACCTTTAACATGTAGTTGTTTTAATATTGCTAATTTAGCCTTATGTGGGATGCCTTTTTTG

>Australia-ND5-A46

CGGTTTAATATTTTATTAGATAATTCTAAGTTAGGACAATTTTTATTATTAGTTTCTGGGTTAACAATATTTATAGCTGGATTAGGGGCTAATTTTGAGTTTGATTTAAAAAAAATTATTGCTTTATCTACTTTAAGTCAATTAGGTTTAATAATAAGTATTTTATCTATTGGTTATTATAAGTTAGCTTTTTTTCATTTATTAACACATGCACTTTTTAAGGCTTTATTATTTATATGTGCTGGGGTAATTATTCATAATACAAAAAATGCTCAAGATATTCGATTTATAGGGGGTTTAAGTATAAGAATACCTTTAACATGTAGTTGTTTTAATATTGCTAATTTAGCCTTATGTGGGATGCCTTTTTTG

>Australia-ND5-A47

CGGTTTAATATTTTATTAGATAATTCTAAGTTAGGACAATTTTTATTATTAGTTTCTGGGTTAACAATATTTATAGCTGGATTAGGGGCTAATTTTGAGTTTGATTTAAAAAAAATTATTGCTTTATCTACTTTAAGTCAATTAGGTTTAATAATAAGTATTTTATCTATTGGTTATTATAAGTTAGCTTTTTTTCATTTATTAACACATGCACTTTTTAAGGCTTTATTATTTATATGTGCTGGGGTAATTATTCATAATACAAAAAATGCTCAAGATATTCGATTTATAGGGGGTTTAAGTATAAGAATACCTTTAACATGTAGTTGTTTTAATATTGCTAATTTAGCCCTATGTGGGATGCCTTTTTTG

>Australia-ND5-A48

CGATTTAATATTTTATTAGATAATTCTAAGTTAGGACAATTTTTATTATTAGTTTCTGGATTAACAATATTTATAGCTGGATTAGGGGCTAATTTTGAGTTTGATTTAAAAAAAATTATTGCTTTATCTACTTTAAGTCAATTAGGTTTAATAATAAGTATTTTATCTATTGGTTATTATAAGTTAGCTTTTTTTCATTTATTAACACATGCGCTCTTTAAGGCTTTATTATTTATATGTGCTGGAGTAATTATTCATAATACAAAAAATGCTCAAGATATTCGATTTATAGGGGGTTTAAGTATAAGAATACCTTTAACATGTAGTTGTTTTAATATTGCTAATTTAGCTTTATGTGGGATACCTTTTTTG

>Australia-ND5-A49

CGGTTTAATATTTTATTAGATAATTCTAAGTTAGGACAATTTTTATTATTAGTTTCTGGGTTAACAATATTTATAGCTGGATTAGGGGCTAATTTTGAGTTTGATTTAAAAAAAATTATTGCTTTATCTACTTTAAGTCAATTAGGTTTAATAATAAGTATTTTATCTATTGGTTATTATAAGTTAGCTTTTTTTCATTTATTAACACATGCACTTTTTAAGGCTTTATTATTTATATGTGCTGGGGTAATTATTCATAATACAAAAAATGCTCAAGATATTCGATTTATAGGGGGTTTAAGTATAAGAATACCTTTAACATGTAGTTGTTTTAATATTGCTAATTTAGCCTTATGTGGGATGCCTTTTTTG

>Australia-ND5-A50

CGGTTTAATATTTTATTAGATAATTCTAAGTTAGGACAATTTTTATTATTAGTTTCTGGGTTAACAATATTTATAGCTGGATTAGGGGCTAATTTTGAGTTTGATTTAAAAAAAATTATTGCTTTATCTACTTTAAGTCAATTAGGTTTAATAATAAGTATTTTATCTATTGGTTATTATAAGTTAGCTTTTTTTCATTTATTAACACATGCACTTTTTAAGGCTTTATTATTTATATGTGCTGGGGTAATTATTCATAATACAAAAAATGCTCAAGATATTCGATTTATAGGGGGTTTAAGTATAAGAATACCTTTAACATGTAGTTGTTTTAATATTGCTAATTTAGCCTTATGTGGGATGCCTTTTTTG

>Australia-ND5-A51

CGGTTTAATATTTTATTAGATAATTCTAAGTTAGGACAATTTTTATTATTAGTTTCTGGGTTAACAATATTTATAGCTGGATTAGGGGCTAATTTTGAGTTTGATTTAAAAAAAATTATTGCTTTATCTACTTTAAGTCAATTAGGTTTAATAATAAGTATTTTATCTATTGGTTATTATAAGTTAGCTTTTTTTCATTTATTAACACATGCACTTTTTAAGGCTTTATTATTTATATGTGCTGGGGTAATTATTCATAATACAAAAAATGCTCAAGATATTCGATTTATAGGGGGTTTAAGTATAAGAATACCTTTAACATGTAGTTGTTTTAATATTGCTAATTTAGCCTTATGTGGGATGCCTTTTTTG

>Australia-ND5-A55

CGGTTTAATATTTTATTAGATAATTCTAAGTTAGGACAATTTTTATTATTAGTTTCTGGGTTAACAATATTTATAGCTGGATTAGGGGCTAATTTTGAGTTTGATTTAAAAAAAATTATTGCTTTATCTACTTTAAGTCAATTAGGTTTAATAATAAGTATTTTATCTATTGGTTATTATAAGTTAGCTTTTTTTCATTTATTAACACATGCACTTTTTAAGGCTTTATTATTTATATGTGCTGGGGTAATTATTCATAATACAAAAAATGCTCAAGATATTCGATTTATAGGGGGTTTAAGTATAAGAATACCTTTAACATGTAGTTGTTTTAATATTGCTAATTTAGCCTTATGTGGGATGCCTTTTTTG

>Australia-ND5-A56

CGGTTTAATATTTTATTAGATAATTCTAAGTTAGGACAATTTTTATTATTAGTTTCTGGGTTAACAATATTTATAGCTGGATTAGGGGCTAATTTTGAGTTTGATTTAAAAAAAATTATTGCTTTATCTACTTTAAGTCAATTAGGTTTAATAATAAGTATTTTATCTATTGGTTATTATAAGTTAGCTTTTTTTCATTTATTAACACATGCACTTTTTAAGGCTTTATTATTTATATGTGCTGGGGTAATTATTCATAATACAAAAAATGCTCAAGATATTCGATTTATAGGGGGTTTAAGTATAAGAATACCTTTAACATGTAGTTGTTTTAATATTGCTAATTTAGCCTTATGTGGGATGCCTTTTTTG

>Australia-ND5-A57

CGGTTTAATATTTTATTAGATAATTCTAAGTTAGGACAATTTTTATTATTAGTTTCTGGGTTAACAATATTTATAGCTGGATTAGGGGCTAATTTTGAGTTTGATTTAAAAAAAATTATTGCTTTATCTACTTTAAGTCAATTAGGTTTAATAATAAGTATTTTATCTATTGGTTATTATAAGTTAGCTTTTTTTCATTTATTAACACATGCACTTTTTAAGGCTTTATTATTTATATGTGCTGGGGTAATTATTCATAATACAAAAAATGCTCAAGATATTCGATTTATAGGGGGTTTAAGTATAAGAATACCTTTAACATGTAGTTGTTTTAATATTGCTAATTTAGCCTTATGTGGGATGCCTTTTTTG

>Australia-ND5-A58

CGGTTTAATATTTTATTAGATAATTCTAAGTTAGGACAATTTTTATTATTAGTTTCTGGGTTAACAATATTTATAGCTGGATTAGGGGCTAATTTTGAGTTTGATTTAAAAAAAATTATTGCTTTATCTACTTTAAGTCAATTAGGTTTAATAATAAGTATTTTATCTATTGGTTATTATAAGTTAGCTTTTTTTCATTTATTAACACATGCACTTTTTAAGGCTTTATTATTTATATGTGCTGGGGTAATTATTCATAATACAAAAAATGCTCAAGATATTCGATTTATAGGGGGTTTAAGTATAAGAATACCTTTAACATGTAGTTGTTTTAATATTGCTAATTTAGCCTTATGTGGGATGCCTTTTTTG

>Australia-ND5-A59

CGGTTTAATATTTTATTAGATAATTCTAAGTTAGGACAATTTTTATTATTAGTTTCTGGGTTAACAATATTTATAGCTGGATTAGGGGCTAATTTTGAGTTTGATTTAAAAAAAATTATTGCTTTATCTACTTTAAGTCAATTAGGTTTAATAATAAGTATTTTATCTATTGGTTATTATAAGTTAGCTTTTTTTCATTTATTAACACATGCACTTTTTAAGGCTTTATTATTTATATGTGCTGGGGTAATTATTCATAATACAAAAAATGCTCAAGATATTCGATTTATAGGGGGTTTAAGTATAAGAATACCTTTAACATGTAGTTGTTTTAATATTGCTAATTTAGCCCTATGTGGGATGCCTTTTTTG

>Australia-ND5-A60

CGGTTTAATATTTTATTAGATAATTCTAAGTTAGGACAATTTTTATTATTAGTTTCTGGGTTAACAATATTTATAGCTGGATTAGGGGCTAATTTTGAGTTTGATTTAAAAAAAATTATTGCTTTATCTACTTTAAGTCAATTAGGTTTAATAATAAGTATTTTATCTATTGGTTATTATAAGTTAGCTTTTTTTCATTTATTAACACATGCACTTTTTAAGGCTTTATTATTTATATGTGCTGGGGTAATTATTCATAATACAAAAAATGCTCAAGATATTCGATTTATAGGGGGTTTAAGTATAAGAATACCTTTAACATGTAGTTGTTTTAATATTGCTAATTTAGCCTTATGTGGGATGCCTTTTTTG

>Australia-ND5-A61

CGGTTTAATATTTTATTAGATAATTCTAAGTTAGGACAATTTTTATTATTAGTTTCTGGGTTAACAATATTTATAGCTGGATTAGGGGCTAATTTTGAGTTTGATTTAAAAAAAATTATTGCTTTATCTACTTTAAGTCAATTAGGTTTAATAATAAGTATTTTATCTATTGGTTATTATAAGTTAGCTTTTTTTCATTTATTAACACATGCACTTTTTAAGGCTTTATTATTTATATGTGCTGGGGTAATTATTCATAATACAAAAAATGCTCAAGATATTCGATTTATAGGGGGTTTAAGTATAAGAATACCTTTAACATGTAGTTGTTTTAATATTGCTAATTTAGCCCTATGTGGGATGCCTTTTTTG

>Australia-ND5-A62

CGGTTTAATATTTTATTAGATAATTCTAAGTTAGGACAATTTTTATTATTAGTTTCTGGGTTAACAATATTTATAGCTGGATTAGGGGCTAATTTTGAGTTTGATTTAAAAAAAATTATTGCTTTATCTACTTTAAGTCAATTAGGTTTAATAATAAGTATTTTATCTATTGGTTATTATAAGTTAGCTTTTTTTCATTTATTAACACATGCACTTTTTAAGGCTTTATTATTTATATGTGCTGGGGTAATTATTCATAATACAAAAAATGCTCAAGATATTCGATTTATAGGGGGTTTAAGTATAAGAATACCTTTAACATGTAGTTGTTTTAATATTGCTAATTTAGCCTTATGTGGGATGCCTTTTTTG

>Australia-ND5-A63

CGGTTTAATATTTTATTAGATAATTCTAAGTTAGGACAATTTTTATTATTAGTTTCTGGGTTAACAATATTTATAGCTGGATTAGGGGCTAATTTTGAGTTTGATTTAAAAAAAATTATTGCTTTATCTACTTTAAGTCAATTAGGTTTAATAATAAGTATTTTATCTATTGGTTATTATAAGTTAGCTTTTTTTCATTTATTAACACATGCACTTTTTAAGGCTTTATTATTTATATGTGCTGGGGTAATTATTCATAATACAAAAAATGCTCAAGATATTCGATTTATAGGGGGTTTAAGTATAAGAATACCTTTAACATGTAGTTGTTTTAATATTGCTAATTTAGCCTTATGTGGGATGCCTTTTTTG

>Australia-ND5-A64

CGGTTTAATATTTTATTAGATAATTCTAAGTTAGGACAATTTTTATTATTAGTTTCTGGGTTAACAATATTTATAGCTGGATTAGGGGCTAATTTTGAGTTTGATTTAAAAAAAATTATTGCTTTATCTACTTTAAGTCAATTAGGTTTAATAATAAGTATTTTATCTATTGGTTATTATAAGTTAGCTTTTTTTCATTTATTAACACATGCACTTTTTAAGGCTTTATTATTTATATGTGCTGGGGTAATTATTCATAATACAAAAAATGCTCAAGATATTCGATTTATAGGGGGTTTAAGTATAAGAATACCTTTAACATGTAGTTGTTTTAATATTGCTAATTTAGCCTTATGTGGGATGCCTTTTTTG

>Australia-ND5-A65

CGGTTTAATATTTTATTAGATAATTCTAAGTTAGGACAATTTTTATTATTAGTTTCTGGGTTAACAATATTTATAGCTGGATTAGGGGCTAATTTTGAGTTTGATTTAAAAAAAATTATTGCTTTATCTACTTTAAGTCAATTAGGTTTAATAATAAGTATTTTATCTATTGGTTATTATAAGTTAGCTTTTTTTCATTTATTAACACATGCACTTTTTAAGGCTTTATTATTTATATGTGCTGGGGTAATTATTCATAATACAAAAAATGCTCAAGATATTCGATTTATAGGGGGTTTAAGTATAAGAATACCTTTAACATGTAGTTGTTTTAATATTGCTAATTTAGCCCTATGTGGGATGCCTTTTTTG

>Australia-ND5-A66

CGGTTTAATATTTTATTAGATAATTCTAAGTTAGGACAATTTTTATTATTAGTTTCTGGGTTAACAATATTTATAGCTGGATTAGGGGCTAATTTTGAGTTTGATTTAAAAAAAATTATTGCTTTATCTACTTTAAGTCAATTAGGTTTAATAATAAGTATTTTATCTATTGGTTATTATAAGTTAGCTTTTTTTCATTTATTAACACATGCACTTTTTAAGGCTTTATTATTTATATGTGCTGGGGTAATTATTCATAATACAAAAAATGCTCAAGATATTCGATTTATAGGGGGTTTAAGTATAAGAATACCTTTAACATGTAGTTGTTTTAATATTGCTAATTTAGCCTTATGTGGGATGCCTTTTTTG

>Australia-ND5-A69

CGGTTTAATATTTTATTAGATAATTCTAAGTTAGGACAATTTTTATTATTAGTTTCTGGGTTAACAATATTTATAGCTGGATTAGGGGCTAATTTTGAGTTTGATTTAAAAAAAATTATTGCTTTATCTACTTTAAGTCAATTAGGTTTAATAATAAGTATTTTATCTATTGGTTATTATAAGTTAGCTTTTTTTCATTTATTAACACATGCACTTTTTAAGGCTTTATTATTTATATGTGCTGGGGTAATTATTCATAATACAAAAAATGCTCAAGATATTCGATTTATAGGGGGTTTAAGTATAAGAATACCTTTAACATGTAGTTGTTTTAATATTGCTAATTTAGCCTTATGTGGGATGCCTTTTTTG

>Australia-ND5-A70

CGGTTTAATATTTTATTAGATAATTCTAAGTTAGGACAATTTTTATTATTAGTTTCTGGGTTAACAATATTTATAGCTGGATTAGGGGCTAATTTTGAGTTTGATTTAAAAAAAATTATTGCTTTATCTACTTTAAGTCAATTAGGTTTAATAATAAGTATTTTATCTATTGGTTATTATAAGTTAGCTTTTTTTCATTTATTAACACATGCACTTTTTAAGGCTTTATTATTTATATGTGCTGGGGTAATTATTCATAATACAAAAAATGCTCAAGATATTCGATTTATAGGGGGTTTAAGTATAAGAATACCTTTAACATGTAGTTGTTTTAATATTGCTAATTTAGCCTTATGTGGGATGCCTTTTTTG

>Australia-ND5-A71

CGGTTTAATATTTTATTAGATAATTCTAAGTTAGGACAATTTTTATTATTAGTTTCTGGGTTAACAATATTTATAGCTGGATTAGGGGCTAATTTTGAGTTTGATTTAAAAAAAATTATTGCTTTATCTACTTTAAGTCAATTAGGTTTAATAATAAGTATTTTATCTATTGGTTATTATAAGTTAGCTTTTTTTCATTTATTAACACATGCACTTTTTAAGGCTTTATTATTTATATGTGCTGGGGTAATTATTCATAATACAAAAAATGCTCAAGATATTCGATTTATAGGGGGTTTAAGTATAAGAATACCTTTAACATGTAGTTGTTTTAATATTGCTAATTTAGCCCTATGTGGGATGCCTTTTTTG

>Australia-ND5-A72

CGGTTTAATATTTTATTAGATAATTCTAAGTTAGGACAATTTTTATTATTAGTTTCTGGGTTAACAATATTTATAGCTGGATTAGGGGCTAATTTTGAGTTTGATTTAAAAAAAATTATTGCTTTATCTACTTTAAGTCAATTAGGTTTAATAATAAGTATTTTATCTATTGGTTATTATAAGTTAGCTTTTTTTCATTTATTAACACATGCACTTTTTAAGGCTTTATTATTTATATGTGCTGGGGTAATTATTCATAATACAAAAAATGCTCAAGATATTCGATTTATAGGGGGTTTAAGTATAAGAATACCTTTAACATGTAGTTGTTTTAATATTGCTAATTTAGCCTTATGTGGGATGCCTTTTTTG

>Australia-ND5-A73

CGGTTTAATATTTTATTAGATAATTCTAAGTTAGGACAATTTTTATTATTAGTTTCTGGGTTAACAATATTTATAGCTGGATTAGGGGCTAATTTTGAGTTTGATTTAAAAAAAATTATTGCTTTATCTACTTTAAGTCAATTAGGTTTAATAATAAGTATTTTATCTATTGGTTATTATAAGTTAGCTTTTTTTCATTTATTAACACATGCACTTTTTAAGGCTTTATTATTTATATGTGCTGGGGTAATTATTCATAATACAAAAAATGCTCAAGATATTCGATTTATAGGGGGTTTAAGTATAAGAATACCTTTAACATGTAGTTGTTTTAATATTGCTAATTTAGCCTTATGTGGGATGCCTTTTTTG

>Australia-ND5-A74

CGGTTTAATATTTTATTAGATAATTCTAAGTTAGGACAATTTTTATTATTAGTTTCTGGGTTAACAATATTTATAGCTGGATTAGGGGCTAATTTTGAGTTTGATTTAAAAAAAATTATTGCTTTATCTACTTTAAGTCAATTAGGTTTAATAATAAGTATTTTATCTATTGGTTATTATAAGTTAGCTTTTTTTCATTTATTAACACATGCACTTTTTAAGGCTTTATTATTTATATGTGCTGGGGTAATTATTCATAATACAAAAAATGCTCAAGATATTCGATTTATAGGGGGTTTAAGTATAAGAATACCTTTAACATGTAGTTGTTTTAATATTGCTAATTTAGCCTTATGTGGGATGCCTTTTTTG

>Australia-ND5-A75

CGGTTTAATATTTTATTAGATAATTCTAAGTTAGGACAATTTTTATTATTAGTTTCTGGGTTAACAATATTTATAGCTGGATTAGGGGCTAATTTTGAGTTTGATTTAAAAAAAATTATTGCTTTATCTACTTTAAGTCAATTAGGTTTAATAATAAGTATTTTATCTATTGGTTATTATAAGTTAGCTTTTTTTCATTTATTAACACATGCACTTTTTAAGGCTTTATTATTTATATGTGCTGGGGTAATTATTCATAATACAAAAAATGCTCAAGATATTCGATTTATAGGGGGTTTAAGTATAAGAATACCTTTAACATGTAGTTGTTTTAATATTGCTAATTTAGCCTTATGTGGGATGCCTTTTTTG

>Australia-ND5-A76

CGGTTTAATATTTTATTAGATAATTCTAAGTTAGGACAATTTTTATTATTAGTTTCTGGGTTAACAATATTTATAGCTGGATTAGGGGCTAATTTTGAGTTTGATTTAAAAAAAATTATTGCTTTATCTACTTTAAGTCAATTAGGTTTAATAATAAGTATTTTATCTATTGGTTATTATAAGTTAGCTTTTTTTCATTTATTAACACATGCACTTTTTAAGGCTTTATTATTTATATGTGCTGGGGTAATTATTCATAATACAAAAAATGCTCAAGATATTCGATTTATAGGGGGTTTAAGTATAAGAATACCTTTAACATGTAGTTGTTTTAATATTGCTAATTTAGCCTTATGTGGGATGCCTTTTTTG

>Australia-ND5-A77

CGGTTTAATATTTTATTAGATAATTCTAAGTTAGGACAATTTTTATTATTAGTTTCTGGGTTAACAATATTTATAGCTGGATTAGGGGCTAATTTTGAGTTTGATTTAAAAAAAATTATTGCTTTATCTACTTTAAGTCAATTAGGTTTAATAATAAGTATTTTATCTATTGGTTATTATAAGTTAGCTTTTTTTCATTTATTAACACATGCACTTTTTAAGGCTTTATTATTTATATGTGCTGGGGTAATTATTCATAATACAAAAAATGCTCAAGATATTCGATTTATAGGGGGTTTAAGTATAAGAATACCTTTAACATGTAGTTGTTTTAATATTGCTAATTTAGCCTTATGTGGGATGCCTTTTTTG

>Australia-ND5-A78

CGGTTTAATATTTTATTAGATAATTCTAAGTTAGGACAATTTTTATTATTAGTTTCTGGGTTAACAATATTTATAGCTGGATTAGGGGCTAATTTTGAGTTTGATTTAAAAAAAATTATTGCTTTATCTACTTTAAGTCAATTAGGTTTAATAATAAGTATTTTATCTATTGGTTATTATAAGTTAGCTTTTTTTCATTTATTAACACATGCACTTTTTAAGGCTTTATTATTTATATGTGCTGGGGTAATTATTCATAATACAAAAAATGCTCAAGATATTCGATTTATAGGGGGTTTAAGTATAAGAATACCTTTAACATGTAGTTGTTTTAATATTGCTAATTTAGCCTTATGTGGGATGCCTTTTTTG

>Australia-ND5-A79

CGGTTTAATATTTTATTAGATAATTCTAAGTTAGGACAATTTTTATTATTAGTTTCTGGGTTAACAATATTTATAGCTGGATTAGGGGCTAATTTTGAGTTTGATTTAAAAAAAATTATTGCTTTATCTACTTTAAGTCAATTAGGTTTAATAATAAGTATTTTATCTATTGGTTATTATAAGTTAGCTTTTTTTCATTTATTAACACATGCACTTTTTAAGGCTTTATTATTTATATGTGCTGGGGTAATTATTCATAATACAAAAAATGCTCAAGATATTCGATTTATAGGGGGTTTAAGTATAAGAATACCTTTAACATGTAGTTGTTTTAATATTGCTAATTTAGCCCTATGTGGGATGCCTTTTTTG

>Australia-ND5-A80

CGGTTTAATATTTTATTAGATAATTCTAAGTTAGGACAATTTTTATTATTAGTTTCTGGGTTAACAATATTTATAGCTGGATTAGGGGCTAATTTTGAGTTTGATTTAAAAAAAATTATTGCTTTATCTACTTTAAGTCAATTAGGTTTAATAATAAGTATTTTATCTATTGGTTATTATAAGTTAGCTTTTTTTCATTTATTAACACATGCACTTTTTAAGGCTTTATTATTTATATGTGCTGGGGTAATTATTCATAATACAAAAAATGCTCAAGATATTCGATTTATAGGGGGTTTAAGTATAAGAATACCTTTAACATGTAGTTGTTTTAATATTGCTAATTTAGCCCTATGTGGGATGCCTTTTTTG

>Australia-ND5-A81

CGGTTTAATATTTTATTAGATAATTCTAAGTTAGGACAATTTTTATTATTAGTTTCTGGGTTAACAATATTTATAGCTGGATTAGGGGCTAATTTTGAGTTTGATTTAAAAAAAATTATTGCTTTATCTACTTTAAGTCAATTAGGTTTAATAATAAGTATTTTATCTATTGGTTATTATAAGTTAGCTTTTTTTCATTTATTAACACATGCACTTTTTAAGGCTTTATTATTTATATGTGCTGGGGTAATTATTCATAATACAAAAAATGCTCAAGATATTCGATTTATAGGGGGTTTAAGTATAAGAATACCTTTAACATGTAGTTGTTTTAATATTGCTAATTTAGCCCTATGTGGGATGCCTTTTTTG

>Australia-ND5-A82

CGGTTTAATATTTTATTAGATAATTCTAAGTTAGGACAATTTTTATTATTAGTTTCTGGGTTAACAATATTTATAGCTGGATTAGGGGCTAATTTTGAGTTTGATTTAAAAAAAATTATTGCTTTATCTACTTTAAGTCAATTAGGTTTAATAATAAGTATTTTATCTATTGGTTATTATAAGTTAGCTTTTTTTCATTTATTAACACATGCACTTTTTAAGGCTTTATTATTTATATGTGCTGGGGTAATTATTCATAATACAAAAAATGCTCAAGATATTCGATTTATAGGGGGTTTAAGTATAAGAATACCTTTAACATGTAGTTGTTTTAATATTGCTAATTTAGCCTTATGTGGGATGCCTTTTTTG

>Australia-ND5-A83

CGGTTTAATATTTTATTAGATAATTCTAAGTTAGGACAATTTTTATTATTAGTTTCTGGGTTAACAATATTTATAGCTGGATTAGGGGCTAATTTTGAGTTTGATTTAAAAAAAATTATTGCTTTATCTACTTTAAGTCAATTAGGTTTAATAATAAGTATTTTATCTATTGGTTATTATAAGTTAGCTTTTTTTCATTTATTAACACATGCACTTTTTAAGGCTTTATTATTTATATGTGCTGGGGTAATTATTCATAATACAAAAAATGCTCAAGATATTCGATTTATAGGGGGTTTAAGTATAAGAATACCTTTAACATGTAGTTGTTTTAATATTGCTAATTTAGCCCTATGTGGGATGCCTTTTTTG

>Australia-ND5-A84

CGGTTTAATATTTTATTAGATAATTCTAAGTTAGGACAATTTTTATTATTAGTTTCTGGGTTAACAATATTTATAGCTGGATTAGGGGCTAATTTTGAGTTTGATTTAAAAAAAATTATTGCTTTATCTACTTTAAGTCAATTAGGTTTAATAATAAGTATTTTATCTATTGGTTATTATAAGTTAGCTTTTTTTCATTTATTAACACATGCACTTTTTAAGGCTTTATTATTTATATGTGCTGGGGTAATTATTCATAATACAAAAAATGCTCAAGATATTCGATTTATAGGGGGTTTAAGTATAAGAATACCTTTAACATGTAGTTGTTTTAATATTGCTAATTTAGCCCTATGTGGGATGCCTTTTTTG

>Australia-ND5-A85

CGGTTTAATATTTTATTAGATAATTCTAAGTTAGGACAATTTTTATTATTAGTTTCTGGGTTAACAATATTTATAGCTGGATTAGGGGCTAATTTTGAGTTTGATTTAAAAAAAATTATTGCTTTATCTACTTTAAGTCAATTAGGTTTAATAATAAGTATTTTATCTATTGGTTATTATAAGTTAGCTTTTTTTCATTTATTAACACATGCACTTTTTAAGGCTTTATTATTTATATGTGCTGGGGTAATTATTCATAATACAAAAAATGCTCAAGATATTCGATTTATAGGGGGTTTAAGTATAAGAATACCTTTAACATGTAGTTGTTTTAATATTGCTAATTTAGCCTTATGTGGGATGCCTTTTTTG

>Australia-ND5-A86

CGGTTTAATATTTTATTAGATAATTCTAAGTTAGGACAATTTTTATTATTAGTTTCTGGGTTAACAATATTTATAGCTGGATTAGGGGCTAATTTTGAGTTTGATTTAAAAAAAATTATTGCTTTATCTACTTTAAGTCAATTAGGTTTAATAATAAGTATTTTATCTATTGGTTATTATAAGTTAGCTTTTTTTCATTTATTAACACATGCACTTTTTAAGGCTTTATTATTTATATGTGCTGGGGTAATTATTCATAATACAAAAAATGCTCAAGATATTCGATTTATAGGGGGTTTAAGTATAAGAATACCTTTAACATGTAGTTGTTTTAATATTGCTAATTTAGCCCTATGTGGGATGCCTTTTTTG

>Australia-ND5-A87

CGGTTTAATATTTTATTAGATAATTCTAAGTTAGGACAATTTTTATTATTAGTTTCTGGGTTAACAATATTTATAGCTGGATTAGGGGCTAATTTTGAGTTTGATTTAAAAAAAATTATTGCTTTATCTACTTTAAGTCAATTAGGTTTAATAATAAGTATTTTATCTATTGGTTATTATAAGTTAGCTTTTTTTCATTTATTAACACATGCACTTTTTAAGGCTTTATTATTTATATGTGCTGGGGTAATTATTCATAATACAAAAAATGCTCAAGATATTCGATTTATAGGGGGTTTAAGTATAAGAATACCTTTAACATGTAGTTGTTTTAATATTGCTAATTTAGCCTTATGTGGGATGCCTTTTTTG

>Australia-ND5-A88

CGGTTTAATATTTTATTAGATAATTCTAAGTTAGGACAATTTTTATTATTAGTTTCTGGGTTAACAATATTTATAGCTGGATTAGGGGCTAATTTTGAGTTTGATTTAAAAAAAATTATTGCTTTATCTACTTTAAGTCAATTAGGTTTAATAATAAGTATTTTATCTATTGGTTATTATAAGTTAGCTTTTTTTCATTTATTAACACATGCACTTTTTAAGGCTTTATTATTTATATGTGCTGGGGTAATTATTCATAATACAAAAAATGCTCAAGATATTCGATTTATAGGGGGTTTAAGTATAAGAATACCTTTAACATGTAGTTGTTTTAATATTGCTAATTTAGCCTTATGTGGGATGCCTTTTTTG

>Australia-ND5-A89

CGGTTTAATATTTTATTAGATAATTCTAAGTTAGGACAATTTTTATTATTAGTTTCTGGGTTAACAATATTTATAGCTGGATTAGGGGCTAATTTTGAGTTTGATTTAAAAAAAATTATTGCTTTATCTACTTTAAGTCAATTAGGTTTAATAATAAGTATTTTATCTATTGGTTATTATAAGTTAGCTTTTTTTCATTTATTAACACATGCACTTTTTAAGGCTTTATTATTTATATGTGCTGGGGTAATTATTCATAATACAAAAAATGCTCAAGATATTCGATTTATAGGGGGTTTAAGTATAAGAATACCTTTAACATGTAGTTGTTTTAATATTGCTAATTTAGCCCTATGTGGGATGCCTTTTTTG

>Australia-ND5-AF11

CGGTTTAATATTTTATTAGATAATTCTAAGTTAGGACAATTTTTATTATTAGTTTCTGGGTTAACAATATTTATAGCTGGATTAGGGGCTAATTTTGAGTTTGATTTAAAAAAAATTATTGCTTTATCTACTTTAAGTCAATTAGGTTTAATAATAAGTATTTTATCTATTGGTTATTATAAGTTAGCTTTTTTTCATTTATTAACACATGCACTTTTTAAGGCTTTATTATTTATATGTGCTGGGGTAATTATTCATAATACAAAAAATGCTCAAGATATTCGATTTATAGGGGGTTTAAGTATAAGAATACCTTTAACATGTAGTTGTTTTAATATTGCTAATTTAGCCCTATGTGGGATGCCTTTTTTG

>Australia-ND5-AF12

CGGTTTAATATTTTATTAGATAATTCTAAGTTAGGACAATTTTTATTATTAGTTTCTGGGTTAACAATATTTATAGCTGGATTAGGGGCTAATTTTGAGTTTGATTTAAAAAAAATTATTGCTTTATCTACTTTAAGTCAATTAGGTTTAATAATAAGTATTTTATCTATTGGTTATTATAAGTTAGCTTTTTTTCATTTATTAACACATGCACTTTTTAAGGCTTTATTATTTATATGTGCTGGGGTAATTATTCATAATACAAAAAATGCTCAAGATATTCGATTTATAGGGGGTTTAAGTATAAGAATACCTTTAACATGTAGTTGTTTTAATATTGCTAATTTAGCCTTATGTGGGATGCCTTTTTTG

>Australia-ND5-AG11

CGGTTTAATATTTTATTAGATAATTCTAAGTTAGGACAATTTTTATTATTAGTTTCTGGGTTAACAATATTTATAGCTGGATTAGGGGCTAATTTTGAGTTTGATTTAAAAAAAATTATTGCTTTATCTACTTTAAGTCAATTAGGTTTAATAATAAGTATTTTATCTATTGGTTATTATAAGTTAGCTTTTTTTCATTTATTAACACATGCACTTTTTAAGGCTTTATTATTTATATGTGCTGGGGTAATTATTCATAATACAAAAAATGCTCAAGATATTCGATTTATAGGGGGTTTAAGTATAAGAATACCTTTAACATGTAGTTGTTTTAATATTGCTAATTTAGCCTTATGTGGGATGCCTTTTTTG

>Australia-ND5-AG12

CGGTTTAATATTTTATTAGATAATTCTAAGTTAGGACAATTTTTATTATTAGTTTCTGGGTTAACAATATTTATAGCTGGATTAGGGGCTAATTTTGAGTTTGATTTAAAAAAAATTATTGCTTTATCTACTTTAAGTCAATTAGGTTTAATAATAAGTATTTTATCTATTGGTTATTATAAGTTAGCTTTTTTTCATTTATTAACACATGCACTTTTTAAGGCTTTATTATTTATATGTGCTGGGGTAATTATTCATAATACAAAAAATGCTCAAGATATTCGATTTATAGGGGGTTTAAGTATAAGAATACCTTTAACATGTAGTTGTTTTAATATTGCTAATTTAGCCTTATGTGGGATGCCTTTTTTG

>Australia-ND5-AH11

CGGTTTAATATTTTATTAGATAATTCTAAGTTAGGACAATTTTTATTATTAGTTTCTGGGTTAACAATATTTATAGCTGGATTAGGGGCTAATTTTGAGTTTGATTTAAAAAAAATTATTGCTTTATCTACTTTAAGTCAATTAGGTTTAATAATAAGTATTTTATCTATTGGTTATTATAAGTTAGCTTTTTTTCATTTATTAACACATGCACTTTTTAAGGCTTTATTATTTATATGTGCTGGGGTAATTATTCATAATACAAAAAATGCTCAAGATATTCGATTTATAGGGGGTTTAAGTATAAGAATACCTTTAACATGTAGTTGTTTTAATATTGCTAATTTAGCCCTATGTGGGATGCCTTTTTTG

>Australia-ND5-AH12

CGGTTTAATATTTTATTAGATAATTCTAAGTTAGGACAATTTTTATTATTAGTTTCTGGGTTAACAATATTTATAGCTGGATTAGGGGCTAATTTTGAGTTTGATTTAAAAAAAATTATTGCTTTATCTACTTTAAGTCAATTAGGTTTAATAATAAGTATTTTATCTATTGGTTATTATAAGTTAGCTTTTTTTCATTTATTAACACATGCACTTTTTAAGGCTTTATTATTTATATGTGCTGGGGTAATTATTCATAATACAAAAAATGCTCAAGATATTCGATTTATAGGGGGTTTAAGTATAAGAATACCTTTAACATGTAGTTGTTTTAATATTGCTAATTTAGCCTTATGTGGGATGCCTTTTTTG

>Australia-ND5-T21

CGGTTTAATATTTTATTAGATAATTCTAAGTTAGGACAATTTTTATTATTAGTTTCTGGGTTAACAATATTTATAGCTGGATTAGGGGCTAATTTTGAGTTTGATTTAAAAAAAATTATTGCTTTATCTACTTTAAGTCAATTAGGTTTAATAATAAGTATTTTATCTATTGGTTATTATAAGTTAGCTTTTTTTCATTTATTAACACATGCACTTTTTAAGGCTTTATTATTTATATGTGCTGGGGTAATTATTCATAATACAAAAAATGCTCAAGATATTCGATTTATAGGGGGTTTAAGTATAAGAATACCTTTAACATGTAGTTGTTTTAATATTGCTAATTTAGCCTTATGTGGGATGCCTTTTTTG

>Australia-ND5-T22

CGGTTTAATATTTTATTAGATAATTCTAAGTTAGGACAATTTTTATTATTAGTTTCTGGGTTAACAATATTTATAGCTGGATTAGGGGCTAATTTTGAGTTTGATTTAAAAAAAATTATTGCTTTATCTACTTTAAGTCAATTAGGTTTAATAATAAGTATTTTATCTATTGGTTATTATAAGTTAGCTTTTTTTCATTTATTAACACATGCACTTTTTAAGGCTTTATTATTTATATGTGCTGGGGTAATTATTCATAATACAAAAAATGCTCAAGATATTCGATTTATAGGGGGTTTAAGTATAAGAATACCTTTAACATGTAGTTGTTTTAATATTGCTAATTTAGCCCTATGTGGGATGCCTTTTTTG

>Australia-ND5-T23

CGGTTTAATATTTTATTAGATAATTCTAAGTTAGGACAATTTTTATTATTAGTTTCTGGGTTAACAATATTTATAGCTGGATTAGGGGCTAATTTTGAGTTTGATTTAAAAAAAATTATTGCTTTATCTACTTTAAGTCAATTAGGTTTAATAATAAGTATTTTATCTATTGGTTATTATAAGTTAGCTTTTTTTCATTTATTAACACATGCACTTTTTAAGGCTTTATTATTTATATGTGCTGGGGTAATTATTCATAATACAAAAAATGCTCAAGATATTCGATTTATAGGGGGTTTAAGTATAAGAATACCTTTAACATGTAGTTGTTTTAATATTGCTAATTTAGCCTTATGTGGGATGCCTTTTTTG

>Australia-ND5-T26

CGGTTTAATATTTTATTAGATAATTCTAAGTTAGGACAATTTTTATTATTAGTTTCTGGGTTAACAATATTTATAGCTGGATTAGGGGCTAATTTTGAGTTTGATTTAAAAAAAATTATTGCTTTATCTACTTTAAGTCAATTAGGTTTAATAATAAGTATTTTATCTATTGGTTATTATAAGTTAGCTTTTTTTCATTTATTAACACATGCACTTTTTAAGGCTTTATTATTTATATGTGCTGGGGTAATTATTCATAATACAAAAAATGCTCAAGATATTCGATTTATAGGGGGTTTAAGTATAAGAATACCTTTAACATGTAGTTGTTTTAATATTGCTAATTTAGCCTTATGTGGGATGCCTTTTTTG

>Australia-ND5-T27

CGGTTTAATATTTTATTAGATAATTCTAAGTTAGGACAATTTTTATTATTAGTTTCTGGGTTAACAATATTTATAGCTGGATTAGGGGCTAATTTTGAGTTTGATTTAAAAAAAATTATTGCTTTATCTACTTTAAGTCAATTAGGTTTAATAATAAGTATTTTATCTATTGGTTATTATAAGTTAGCTTTTTTTCATTTATTAACACATGCACTTTTTAAGGCTTTATTATTTATATGTGCTGGGGTAATTATTCATAATACAAAAAATGCTCAAGATATTCGATTTATAGGGGGTTTAAGTATAAGAATACCTTTAACATGTAGTTGTTTTAATATTGCTAATTTAGCCTTATGTGGGATGCCTTTTTTG

>Australia-ND5-T28

CGGTTTAATATTTTATTAGATAATTCTAAGTTAGGACAATTTTTATTATTAGTTTCTGGGTTAACAATATTTATAGCTGGATTAGGGGCTAATTTTGAGTTTGATTTAAAAAAAATTATTGCTTTATCTACTTTAAGTCAATTAGGTTTAATAATAAGTATTTTATCTATTGGTTATTATAAGTTAGCTTTTTTTCATTTATTAACACATGCACTTTTTAAGGCTTTATTATTTATATGTGCTGGGGTAATTATTCATAATACAAAAAATGCTCAAGATATTCGATTTATAGGGGGTTTAAGTATAAGAATACCTTTAACATGTAGTTGTTTTAATATTGCTAATTTAGCCTTATGTGGGATGCCTTTTTTG

>Australia-ND5-T29

CGGTTTAATATTTTATTAGATAATTCTAAGTTAGGACAATTTTTATTATTAGTTTCTGGGTTAACAATATTTATAGCTGGATTAGGGGCTAATTTTGAGTTTGATTTAAAAAAAATTATTGCTTTATCTACTTTAAGTCAATTAGGTTTAATAATAAGTATTTTATCTATTGGTTATTATAAGTTAGCTTTTTTTCATTTATTAACACATGCACTTTTTAAGGCTTTATTATTTATATGTGCTGGGGTAATTATTCATAATACAAAAAATGCTCAAGATATTCGATTTATAGGGGGTTTAAGTATAAGAATACCTTTAACATGTAGTTGTTTTAATATTGCTAATTTAGCCTTATGTGGGATGCCTTTTTTG

>Australia-ND5-T30

CGGTTTAATATTTTATTAGATAATTCTAAGTTAGGACAATTTTTATTATTAGTTTCTGGGTTAACAATATTTATAGCTGGATTAGGGGCTAATTTTGAGTTTGATTTAAAAAAAATTATTGCTTTATCTACTTTAAGTCAATTAGGTTTAATAATAAGTATTTTATCTATTGGTTATTATAAGTTAGCTTTTTTTCATTTATTAACACATGCACTTTTTAAGGCTTTATTATTTATATGTGCTGGGGTAATTATTCATAATACAAAAAATGCTCAAGATATTCGATTTATAGGGGGTTTAAGTATAAGAATACCTTTAACATGTAGTTGTTTTAATATTGCTAATTTAGCCTTATGTGGGATGCCTTTTTTG

>Australia-ND5-T31

CGGTTTAATATTTTATTAGATAATTCTAAGTTAGGACAATTTTTATTATTAGTTTCTGGGTTAACAATATTTATAGCTGGATTAGGGGCTAATTTTGAGTTTGATTTAAAAAAAATTATTGCTTTATCTACTTTAAGTCAATTAGGTTTAATAATAAGTATTTTATCTATTGGTTATTATAAGTTAGCTTTTTTTCATTTATTAACACATGCACTTTTTAAGGCTTTATTATTTATATGTGCTGGGGTAATTATTCATAATACAAAAAATGCTCAAGATATTCGATTTATAGGGGGTTTAAGTATAAGAATACCTTTAACATGTAGTTGTTTTAATATTGCTAATTTAGCCTTATGTGGGATGCCTTTTTTG

>Australia-ND5-T32

CGGTTTAATATTTTATTAGATAATTCTAAGTTAGGACAATTTTTATTATTAGTTTCTGGGTTAACAATATTTATAGCTGGATTAGGGGCTAATTTTGAGTTTGATTTAAAAAAAATTATTGCTTTATCTACTTTAAGTCAATTAGGTTTAATAATAAGTATTTTATCTATTGGTTATTATAAGTTAGCTTTTTTTCATTTATTAACACATGCACTTTTTAAGGCTTTATTATTTATATGTGCTGGGGTAATTATTCATAATACAAAAAATGCTCAAGATATTCGATTTATAGGGGGTTTAAGTATAAGAATACCTTTAACATGTAGTTGTTTTAATATTGCTAATTTAGCCTTATGTGGGATGCCTTTTTTG

>Australia-ND5-T33

CGGTTTAATATTTTATTAGATAATTCTAAGTTAGGACAATTTTTATTATTAGTTTCTGGGTTAACAATATTTATAGCTGGATTAGGGGCTAATTTTGAGTTTGATTTAAAAAAAATTATTGCTTTATCTACTTTAAGTCAATTAGGTTTAATAATAAGTATTTTATCTATTGGTTATTATAAGTTAGCTTTTTTTCATTTATTAACACATGCACTTTTTAAGGCTTTATTATTTATATGTGCTGGGGTAATTATTCATAATACAAAAAATGCTCAAGATATTCGATTTATAGGGGGTTTAAGTATAAGAATACCTTTAACATGTAGTTGTTTTAATATTGCTAATTTAGCCCTATGTGGGATGCCTTTTTTG

>Australia-ND5-T34

CGGTTTAATATTTTATTAGATAATTCTAAGTTAGGACAATTTTTATTATTAGTTTCTGGGTTAACAATATTTATAGCTGGATTAGGGGCTAATTTTGAGTTTGATTTAAAAAAAATTATTGCTTTATCTACTTTAAGTCAATTAGGTTTAATAATAAGTATTTTATCTATTGGTTATTATAAGTTAGCTTTTTTTCATTTATTAACACATGCACTTTTTAAGGCTTTATTATTTATATGTGCTGGGGTAATTATTCATAATACAAAAAATGCTCAAGATATTCGATTTATAGGGGGTTTAAGTATAAGAATACCTTTAACATGTAGTTGTTTTAATATTGCTAATTTAGCCCTATGTGGGATGCCTTTTTTG

>Australia-ND5-T35

CGGTTTAATATTTTATTAGATAATTCTAAGTTAGGACAATTTTTATTATTAGTTTCTGGGTTAACAATATTTATAGCTGGATTAGGGGCTAATTTTGAGTTTGATTTAAAAAAAATTATTGCTTTATCTACTTTAAGTCAATTAGGTTTAATAATAAGTATTTTATCTATTGGTTATTATAAGTTAGCTTTTTTTCATTTATTAACACATGCACTTTTTAAGGCTTTATTATTTATATGTGCTGGGGTAATTATTCATAATACAAAAAATGCTCAAGATATTCGATTTATAGGGGGTTTAAGTATAAGAATACCTTTAACATGTAGTTGTTTTAATATTGCTAATTTAGCCTTATGTGGGATGCCTTTTTTG

>Australia-ND5-T36

CGGTTTAATATTTTATTAGATAATTCTAAGTTAGGACAATTTTTATTATTAGTTTCTGGGTTAACAATATTTATAGCTGGATTAGGGGCTAATTTTGAGTTTGATTTAAAAAAAATTATTGCTTTATCTACTTTAAGTCAATTAGGTTTAATAATAAGTATTTTATCTATTGGTTATTATAAGTTAGCTTTTTTTCATTTATTAACACATGCACTTTTTAAGGCTTTATTATTTATATGTGCTGGGGTAATTATTCATAATACAAAAAATGCTCAAGATATTCGATTTATAGGGGGTTTAAGTATAAGAATACCTTTAACATGTAGTTGTTTTAATATTGCTAATTTAGCCTTATGTGGGATGCCTTTTTTG

>Australia-ND5-T37

CGGTTTAATATTTTATTAGATAATTCTAAGTTAGGACAATTTTTATTATTAGTTTCTGGGTTAACAATATTTATAGCTGGATTAGGGGCTAATTTTGAGTTTGATTTAAAAAAAATTATTGCTTTATCTACTTTAAGTCAATTAGGTTTAATAATAAGTATTTTATCTATTGGTTATTATAAGTTAGCTTTTTTTCATTTATTAACACATGCACTTTTTAAGGCTTTATTATTTATATGTGCTGGGGTAATTATTCATAATACAAAAAATGCTCAAGATATTCGATTTATAGGGGGTTTAAGTATAAGAATACCTTTAACATGTAGTTGTTTTAATATTGCTAATTTAGCCTTATGTGGGATGCCTTTTTTG

>Australia-ND5-T38

CGGTTTAATATTTTATTAGATAATTCTAAGTTAGGACAATTTTTATTATTAGTTTCTGGGTTAACAATATTTATAGCTGGATTAGGGGCTAATTTTGAGTTTGATTTAAAAAAAATTATTGCTTTATCTACTTTAAGTCAATTAGGTTTAATAATAAGTATTTTATCTATTGGTTATTATAAGTTAGCTTTTTTTCATTTATTAACACATGCACTTTTTAAGGCTTTATTATTTATATGTGCTGGGGTAATTATTCATAATACAAAAAATGCTCAAGATATTCGATTTATAGGGGGTTTAAGTATAAGAATACCTTTAACATGTAGTTGTTTTAATATTGCTAATTTAGCCTTATGTGGGATGCCTTTTTTG

>Australia-ND5-T39

CGGTTTAATATTTTATTAGATAATTCTAAGTTAGGACAATTTTTATTATTAGTTTCTGGGTTAACAATATTTATAGCTGGATTAGGGGCTAATTTTGAGTTTGATTTAAAAAAAATTATTGCTTTATCTACTTTAAGTCAATTAGGTTTAATAATAAGTATTTTATCTATTGGTTATTATAAGTTAGCTTTTTTTCATTTATTAACACATGCACTTTTTAAGGCTTTATTATTTATATGTGCTGGGGTAATTATTCATAATACAAAAAATGCTCAAGATATTCGATTTATAGGGGGTTTAAGTATAAGAATACCTTTAACATGTAGTTGTTTTAATATTGCTAATTTAGCCTTATGTGGGATGCCTTTTTTG

>Australia-ND5-T40

CGGTTTAATATTTTATTAGATAATTCTAAGTTAGGACAATTTTTATTATTAGTTTCTGGGTTAACAATATTTATAGCTGGATTAGGGGCTAATTTTGAGTTTGATTTAAAAAAAATTATTGCTTTATCTACTTTAAGTCAATTAGGTTTAATAATAAGTATTTTATCTATTGGTTATTATAAGTTAGCTTTTTTTCATTTATTAACACATGCACTTTTTAAGGCTTTATTATTTATATGTGCTGGGGTAATTATTCATAATACAAAAAATGCTCAAGATATTCGATTTATAGGGGGTTTAAGTATAAGAATACCTTTAACATGTAGTTGTTTTAATATTGCTAATTTAGCCTTATGTGGGATGCCTTTTTTG

>Indonesia-ND5-A01

CGGTTTAATATTTTATTAGATAATTCTAAGTTAGGACAATTTTTATTATTAGTTTCTGGGTTAACAATATTTATAGCTGGATTAGGGGCTAATTTTGAGTTTGATTTAAAAAAAATTATTGCTTTATCTACTTTAAGTCAATTAGGTTTAATAATAAGTATTTTATCTATTGGTTATTATAAGTTAGCTTTTTTTCATTTATTAACACATGCACTTTTTAAGGCTTTATTATTTATATGTGCTGGGGTAATTATTCATAATACAAAAAATGCTCAAGATATTCGATTTATAGGGGGTTTAAGTATAAGAATACCTTTAACATGTAGTTGTTTTAATATTGCTAATTTAGCCTTATGTGGGATGCCTTTTTTG

>Indonesia-ND5-A02

CGGTTTAATATTTTATTAGATAATTCTAAGTTAGGACAATTTTTATTATTAGTTTCTGGGTTAACAATATTTATAGCTGGATTAGGGGCTAATTTTGAGTTTGATTTAAAAAAAATTATTGCTTTATCTACTTTAAGTCAATTAGGTTTAATAATAAGTATTTTATCTATTGGTTATTATAAGTTAGCTTTTTTTCATTTATTAACACATGCACTTTTTAAGGCTTTATTATTTATATGTGCTGGGGTAATTATTCATAATACAAAAAATGCTCAAGATATTCGATTTATAGGGGGTTTAAGTATAAGAATACCTTTAACATGTAGTTGTTTTAATATTGCTAATTTAGCCTTATGTGGGATGCCTTTTTTG

>Indonesia-ND5-A03

CGGTTTAATATTTTATTAGATAATTCTAAGTTAGGACAATTTTTATTATTAGTTTCTGGGTTAACAATATTTATAGCTGGATTAGGGGCTAATTTTGAGTTTGATTTAAAAAAAATTATTGCTTTATCTACTTTAAGTCAATTAGGTTTAATAATAAGTATTTTATCTATTGGTTATTATAAGTTAGCTTTTTTTCATTTATTAACACATGCTCTTTTTAAGGCTTTATTATTTATATGTGCTGGGGTAATTATTCATAATACAAAAAATGCTCAAGATATTCGATTTATAGGGGGTTTAAGTATAAGAATACCTTTAACATGTAGTTGTTTTAATATTGCTAATTTAGCCTTATGTGGGATGCCTTTTTTG

>Indonesia-ND5-A04

CGGTTTAATATTTTATTAGATAATTCTAAGTTAGGACAATTTTTATTATTAGTTTCTGGGTTAACAATATTTATAGCTGGATTAGGGGCTAATTTTGAGTTTGATTTAAAAAAAATTATTGCTTTATCTACTTTAAGTCAATTAGGTTTAATAATAAGTATTTTATCTATTGGTTATTATAAGTTAGCTTTTTTTCATTTATTAACACATGCACTTTTTAAGGCTTTATTATTTATATGTGCTGGGGTAATTATTCATAATACAAAAAATGCTCAAGATATTCGATTTATAGGGGGTTTAAGTATAAGAATACCTTTAACATGTAGTTGTTTTAATATTGCTAATTTAGCCTTATGTGGGATGCCTTTTTTG

>Indonesia-ND5-B01

CGGTTTAATATTTTATTAGATAATTCTAAGTTAGGACAATTTTTATTATTAGTTTCTGGGTTAACAATATTTATAGCTGGATTAGGGGCTAATTTTGAGTTTGATTTAAAAAAAATTATTGCTTTATCTACTTTAAGTCAATTAGGTTTAATAATAAGTATTTTATCTATTGGTTATTATAAGTTAGCTTTTTTTCATTTATTAACACATGCACTTTTTAAGGCTTTATTATTTATATGTGCTGGGGTAATTATTCATAATACAAAAAATGCTCAAGATATTCGATTTATAGGGGGTTTAAGTATAAGAATACCTTTAACATGTAGTTGTTTTAATATTGCTAATTTAGCCTTATGTGGGATGCCTTTTTTG

>Indonesia-ND5-B02

CGGTTTAATATTTTATTAGATAATTCTAAGTTAGGACAATTTTTATTATTAGTTTCTGGGTTAACAATATTTATAGCTGGATTAGGGGCTAATTTTGAGTTTGATTTAAAAAAAATTATTGCTTTATCTACTTTAAGTCAATTAGGTTTAATAATAAGTATTTTATCTATTGGTTATTATAAGTTAGCTTTTTTTCATTTATTAACACATGCACTTTTTAAGGCTTTATTATTTATATGTGCTGGGGTAATTATTCATAATACAAAAAATGCTCAAGATATTCGATTTATAGGGGGTTTAAGTATAAGAATACCTTTAACATGTAGTTGTTTTAATATTGCTAATTTAGCCTTATGTGGGATGCCTTTTTTG

>Indonesia-ND5-B03

CGGTTTAATATTTTATTAGATAATTCTAAGTTAGGACAATTTTTATTATTAGTTTCTGGGTTAACAATATTTATAGCTGGATTAGGGGCTAATTTTGAGTTTGATTTAAAAAAAATTATTGCTTTATCTACTTTAAGTCAATTAGGTTTAATAATAAGTATTTTATCTATTGGTTATTATAAGTTAGCTTTTTTTCATTTATTAACACATGCACTTTTTAAGGCTTTATTATTTATATGTGCTGGGGTAATTATTCATAATACAAAAAATGCTCAAGATATTCGATTTATAGGGGGTTTAAGTATAAGAATACCTTTAACATGTAGTTGTTTTAATATTGCTAATTTAGCCTTATGTGGGATGCCTTTTTTG

>Indonesia-ND5-B04

CGGTTTAATATTTTATTAGATAATTCTAAGTTAGGACAATTTTTATTATTAGTTTCTGGGTTAACAATATTTATAGCTGGATTAGGGGCTAATTTTGAGTTTGATTTAAAAAAAATTATTGCTTTATCTACTTTAAGTCAATTAGGTTTAATAATAAGTATTTTATCTATTGGTTATTATAAGTTAGCTTTTTTTCATTTATTAACACATGCACTTTTTAAGGCTTTATTATTTATATGTGCTGGGGTAATTATTCATAATACAAAAAATGCTCAAGATATTCGATTTATAGGGGGTTTAAGTATAAGAATACCTTTAACATGTAGTTGTTTTAATATTGCTAATTTAGCCTTATGTGGGATGCCTTTTTTG

>Indonesia-ND5-C01

CGGTTTAATATTTTATTAGATAATTCTAAGTTAGGACAATTTTTATTATTAGTTTCTGGGTTAACAATATTTATAGCTGGATTAGGGGCTAATTTTGAGTTTGATTTAAAAAAAATTATTGCTTTATCTACTTTAAGTCAATTAGGTTTAATAATAAGTATTTTATCTATTGGTTATTATAAGTTAGCTTTTTTTCATTTATTAACACATGCACTTTTTAAGGCTTTATTATTTATATGTGCTGGGGTAATTATTCATAATACAAAAAATGCTCAAGATATTCGATTTATAGGGGGTTTAAGTATAAGAATACCTTTAACATGTAGTTGTTTTAATATTGCTAATTTAGCCTTATGTGGGATGCCTTTTTTG

>Indonesia-ND5-C02

CGGTTTAATATTTTATTAGATAATTCTAAGTTAGGACAATTTTTATTATTAGTTTCTGGGTTAACAATATTTATAGCTGGATTAGGGGCTAATTTTGAGTTTGATTTAAAAAAAATTATTGCTTTATCTACTTTAAGTCAATTAGGTTTAATAATAAGTATTTTATCTATTGGTTATTATAAGTTAGCTTTTTTTCATTTATTAACACATGCACTTTTTAAGGCTTTATTATTTATATGTGCTGGGGTAATTATTCATAATACAAAAAATGCTCAAGATATTCGATTTATAGGGGGTTTAAGTATAAGAATACCTTTAACATGTAGTTGTTTTAATATTGCTAATTTAGCCTTATGTGGGATGCCTTTTTTG

>Indonesia-ND5-C03

CGGTTTAATATTTTATTAGATAATTCTAAGTTAGGACAATTTTTATTATTAGTTTCTGGGTTAACAATATTTATAGCTGGATTAGGGGCTAATTTTGAGTTTGATTTAAAAAAAATTATTGCTTTATCTACTTTAAGTCAATTAGGTTTAATAATAAGTATTTTATCTATTGGTTATTATAAGTTAGCTTTTTTTCATTTATTAACACATGCACTTTTTAAGGCTTTATTATTTATATGTGCTGGGGTAATTATTCATAATACAAAAAATGCTCAAGATATTCGATTTATAGGGGGTTTAAGTATAAGAATACCTTTAACATGTAGTTGTTTTAATATTGCTAATTTAGCCTTATGTGGGATGCCTTTTTTG

>Indonesia-ND5-C04

CGGTTTAATATTTTATTAGATAATTCTAAGTTAGGACAATTTTTATTATTAGTTTCTGGGTTAACAATATTTATAGCTGGATTAGGGGCTAATTTTGAGTTTGATTTAAAAAAAATTATTGCTTTATCTACTTTAAGTCAATTAGGTTTAATAATAAGTATTTTATCTATTGGTTATTATAAGTTAGCTTTTTTTCATTTATTAACACATGCACTTTTTAAGGCTTTATTATTTATATGTGCTGGGGTAATTATTCATAATACAAAAAATGCTCAAGATATTCGATTTATAGGGGGTTTAAGTATAAGAATACCTTTAACATGTAGTTGTTTTAATATTGCTAATTTAGCCTTATGTGGGATGCCTTTTTTG

>Indonesia-ND5-D01

CGGTTTAATATTTTATTAGATAATTCTAAGTTAGGACAATTTTTATTATTAGTTTCTGGGTTAACAATATTTATAGCTGGATTAGGGGCTAATTTTGAGTTTGATTTAAAAAAAATTATTGCTTTATCTACTTTAAGTCAATTAGGTTTAATAATAAGTATTTTATCTATTGGTTATTATAAGTTAGCTTTTTTTCATTTATTAACACATGCACTTTTTAAGGCTTTATTATTTATATGTGCTGGGGTAATTATTCATAATACAAAAAATGCTCAAGATATTCGATTTATAGGGGGTTTAAGTATAAGAATACCTTTAACATGTAGTTGTTTTAATATTGCTAATTTAGCCCTATGTGGGATGCCTTTTTTG

>Indonesia-ND5-D02

CGGTTTAATATTTTATTAGATAATTCTAAGTTAGGACAATTTTTATTATTAGTTTCTGGGTTAACAATATTTATAGCTGGATTAGGGGCTAATTTTGAGTTTGATTTAAAAAAAATTATTGCTTTATCTACTTTAAGTCAATTAGGTTTAATAATAAGTATTTTATCTATTGGTTATTATAAGTTAGCTTTTTTTCATTTATTAACACATGCACTTTTTAAGGCTTTATTATTTATATGTGCTGGGGTAATTATTCATAATACAAAAAATGCTCAAGATATTCGATTTATAGGGGGTTTAAGTATAAGAATACCTTTAACATGTAGTTGTTTTAATATTGCTAATTTAGCCTTATGTGGGATGCCTTTTTTG

>Indonesia-ND5-D03

CGGTTTAATATTTTATTAGATAATTCTAAGTTAGGACAATTTTTATTATTAGTTTCTGGGTTAACAATATTTATAGCTGGATTAGGGGCTAATTTTGAGTTTGATTTAAAAAAAATTATTGCTTTATCTACTTTAAGTCAATTAGGTTTAATAATAAGTATTTTATCTATTGGTTATTATAAGTTAGCTTTTTTTCATTTATTAACACATGCACTTTTTAAGGCTTTATTATTTATATGTGCTGGGGTAATTATTCATAATACAAAAAATGCTCAAGATATTCGATTTATAGGGGGTTTAAGTATAAGAATACCTTTAACATGTAGTTGTTTTAATATTGCTAATTTAGCCTTATGTGGGATGCCTTTTTTG

>Indonesia-ND5-E01

CGGTTTAATATTTTATTAGATAATTCTAAGTTAGGACAATTTTTATTATTAGTTTCTGGGTTAACAATATTTATAGCTGGATTAGGGGCTAATTTTGAGTTTGATTTAAAAAAAATTATTGCTTTATCTACTTTAAGTCAATTAGGTTTAATAATAAGTATTTTATCTATTGGTTATTATAAGTTAGCTTTTTTTCATTTATTAACACATGCACTTTTTAAGGCTTTATTATTTATATGTGCTGGGGTAATTATTCATAATACAAAAAATGCTCAAGATATTCGATTTATAGGGGGTTTAAGTATAAGAATACCTTTAACATGTAGTTGTTTTAATATTGCTAATTTAGCCTTATGTGGGATGCCTTTTTTG

>Indonesia-ND5-E02

CGATTTAATATTTTATTAGATAATTCTAAGTTAGGACAATTTTTATTATTAGTTTCTGGATTAACAATATTTATGGCTGGATTAGGGGCTAATTTTGAGTTTGATTTAAAAAAAATTATTGCTTTATCTACTTTAAGTCAATTAGGTTTAATAATAAGTATTTTATCTATTGGTTATTATAAGTTAGCTTTTTTTCATTTATTAACACATGCACTTTTTAAGGCTTTATTATTTATATGTGCTGGAGTAATTATTCATAATACAAAAAATGCTCAAGATATTCGATTTATAGGGGGTTTAAGTATAAGAATACCTTTAACATGTAGTTGTTTTAATATTGCTAATTTAGCTTTATGTGGAATACCTTTTTTG

>Indonesia-ND5-E03

CGGTTTAATATTTTATTAGATAATTCTAAGTTAGGACAATTTTTATTATTAGTTTCTGGGTTAACAATATTTATAGCTGGATTAGGGGCTAATTTTGAGTTTGATTTAAAAAAAATTATTGCTTTATCTACTTTAAGTCAATTAGGTTTAATAATAAGTATTTTATCTATTGGTTATTATAAGTTAGCTTTTTTTCATTTATTAACACATGCACTTTTTAAGGCTTTATTATTTATATGTGCTGGGGTAATTATTCATAATACAAAAAATGCTCAAGATATTCGATTTATAGGGGGTTTAAGTATAAGAATACCTTTAACATGTAGTTGTTTTAATATTGCTAATTTAGCCTTATGTGGGATGCCTTTTTTG

>Indonesia-ND5-E04

CGGTTTAATATTTTATTAGATAATTCTAAGTTAGGACAATTTTTATTATTAGTTTCTGGGTTAACAATATTTATAGCTGGATTAGGGGCTAATTTTGAGTTTGATTTAAAAAAAATTATTGCTTTATCTACTTTAAGTCAATTAGGTTTAATAATAAGTATTTTATCTATTGGTTATTATAAGTTAGCTTTTTTTCATTTATTAACACATGCACTTTTTAAGGCTTTATTATTTATATGTGCTGGGGTAATTATTCATAATACAAAAAATGCTCAAGATATTCGATTTATAGGGGGTTTAAGTATAAGAATACCTTTAACATGTAGTTGTTTTAATATTGCTAATTTAGCCTTATGTGGGATGCCTTTTTTG

>Indonesia-ND5-F01

CGGTTTAATATTTTATTAGATAATTCTAAGTTAGGACAATTTTTATTATTAGTTTCTGGGTTAACAATATTTATAGCTGGATTAGGGGCTAATTTTGAGTTTGATTTAAAAAAAATTATTGCTTTATCTACTTTAAGTCAATTAGGTTTAATAATAAGTATTTTATCTATTGGTTATTATAAGTTAGCTTTTTTTCATTTATTAACACATGCACTTTTTAAGGCTTTATTATTTATATGTGCTGGGGTAATTATTCATAATACAAAAAATGCTCAAGATATTCGATTTATAGGGGGTTTAAGTATAAGAATACCTTTAACATGTAGTTGTTTTAATATTGCTAATTTAGCCTTATGTGGGATGCCTTTTTTG

>Indonesia-ND5-F02

CGGTTTAATATTTTATTAGATAATTCTAAGTTAGGACAATTTTTATTATTAGTTTCTGGGTTAACAATATTTATAGCTGGATTAGGGGCTAATTTTGAGTTTGATTTAAAAAAAATTATTGCTTTATCTACTTTAAGTCAATTAGGTTTAATAATAAGTATTTTATCTATTGGTTATTATAAGTTAGCTTTTTTTCATTTATTAACACATGCACTTTTTAAGGCTTTATTATTTATATGTGCTGGGGTAATTATTCATAATACAAAAAATGCTCAAGATATTCGATTTATAGGGGGTTTAAGTATAAGAATACCTTTAACATGTAGTTGTTTTAATATTGCTAATTTAGCCTTATGTGGGATGCCTTTTTTG

>Indonesia-ND5-F03

CGGTTTAATATTTTATTAGATAATTCTAAGTTAGGACAATTTTTATTATTAGTTTCTGGGTTAACAATATTTATAGCTGGATTAGGGGCTAATTTTGAGTTTGATTTAAAAAAAATTATTGCTTTATCTACTTTAAGTCAATTAGGTTTAATAATAAGTATTTTATCTATTGGTTATTATAAGTTAGCTTTTTTTCATTTATTAACACATGCACTTTTTAAGGCTTTATTATTTATATGTGCTGGGGTAATTATTCATAATACAAAAAATGCTCAAGATATTCGATTTATAGGGGGTTTAAGTATAAGAATACCTTTAACATGTAGTTGTTTTAATATTGCTAATTTAGCCTTATGTGGGATGCCTTTTTTG

>Indonesia-ND5-F04

CGGTTTAATATTTTATTAGATAATTCTAAGTTAGGACAATTTTTATTATTAGTTTCTGGGTTAACAATATTTATAGCTGGATTAGGGGCTAATTTTGAGTTTGATTTAAAAAAAATTATTGCTTTATCTACTTTAAGTCAATTAGGTTTAATAATAAGTATTTTATCTATTGGTTATTATAAGTTAGCTTTTTTTCATTTATTAACACATGCACTTTTTAAGGCTTTATTATTTATATGTGCTGGGGTAATTATTCATAATACAAAAAATGCTCAAGATATTCGATTTATAGGGGGTTTAAGTATAAGAATACCTTTAACATGTAGTTGTTTTAATATTGCTAATTTAGCCTTATGTGGGATGCCTTTTTTG

>Indonesia-ND5-G01

CGGTTTAATATTTTATTAGATAATTCTAAGTTAGGACAATTTTTATTATTAGTTTCTGGGTTAACAATATTTATAGCTGGATTAGGGGCTAATTTTGAGTTTGATTTAAAAAAAATTATTGCTTTATCTACTTTAAGTCAATTAGGTTTAATAATAAGTATTTTATCTATTGGTTATTATAAGTTAGCTTTTTTTCATTTATTAACACATGCACTTTTTAAGGCTTTATTATTTATATGTGCTGGGGTAATTATTCATAATACAAAAAATGCTCAAGATATTCGATTTATAGGGGGTTTAAGTATAAGAATACCTTTAACATGTAGTTGTTTTAATATTGCTAATTTAGCCTTATGTGGGATGCCTTTTTTG

>Indonesia-ND5-G02

CGGTTTAATATTTTATTAGATAATTCTAAGTTAGGACAATTTTTATTATTAGTTTCTGGGTTAACAATATTTATAGCTGGATTAGGGGCTAATTTTGAGTTTGATTTAAAAAAAATTATTGCTTTATCTACTTTAAGTCAATTAGGTTTAATAATAAGTATTTTATCTATTGGTTATTATAAGTTAGCTTTTTTTCATTTATTAACACATGCACTTTTTAAGGCTTTATTATTTATATGTGCTGGGGTAATTATTCATAATACAAAAAATGCTCAAGATATTCGATTTATAGGGGGTTTAAGTATAAGAATACCTTTAACATGTAGTTGTTTTAATATTGCTAATTTAGCCCTATGTGGGATGCCTTTTTTG

>Indonesia-ND5-G03

CGGTTTAATATTTTATTAGATAATTCTAAGTTAGGACAATTTTTATTATTAGTTTCTGGGTTAACAATATTTATAGCTGGATTAGGGGCTAATTTTGAGTTTGATTTAAAAAAAATTATTGCTTTATCTACTTTAAGTCAATTAGGTTTAATAATAAGTATTTTATCTATTGGTTATTATAAGTTAGCTTTTTTTCATTTATTAACACATGCACTTTTTAAGGCTTTATTATTTATATGTGCTGGGGTAATTATTCATAATACAAAAAATGCTCAAGATATTCGATTTATAGGGGGTTTAAGTATAAGAATACCTTTAACATGTAGTTGTTTTAATATTGCTAATTTAGCCTTATGTGGGATGCCTTTTTTG

>Indonesia-ND5-G04

CGGTTTAATATTTTATTAGATAATTCTAAGTTAGGACAATTTTTATTATTAGTTTCTGGGTTAACAATATTTATAGCTGGATTAGGGGCTAATTTTGAGTTTGATTTAAAAAAAATTATTGCTTTATCTACTTTAAGTCAATTAGGTTTAATAATAAGTATTTTATCTATTGGTTATTATAAGTTAGCTTTTTTTCATTTATTAACACATGCACTTTTTAAGGCTTTATTATTTATATGTGCTGGGGTAATTATTCATAATACAAAAAATGCTCAAGATATTCGATTTATAGGGGGTTTAAGTATAAGAATACCTTTAACATGTAGTTGTTTTAATATTGCTAATTTAGCCTTATGTGGGATGCCTTTTTTG

>Indonesia-ND5-H01

CGGTTTAATATTTTATTAGATAATTCTAAGTTAGGACAATTTTTATTATTAGTTTCTGGGTTAACAATATTTATAGCTGGATTAGGGGCTAATTTTGAGTTTGATTTAAAAAAAATTATTGCTTTATCTACTTTAAGTCAATTAGGTTTAATAATAAGTATTTTATCTATTGGTTATTATAAGTTAGCTTTTTTTCATTTATTAACACATGCACTTTTTAAGGCTTTATTATTTATATGTGCTGGGGTAATTATTCATAATACAAAAAATGCTCAAGATATTCGATTTATAGGGGGTTTAAGTATAAGAATACCTTTAACATGTAGTTGTTTTAATATTGCTAATTTAGCCTTATGTGGGATGCCTTTTTTG

>Indonesia-ND5-H02

CGGTTTAATATTTTATTAGATAATTCTAAGTTAGGACAATTTTTATTATTAGTTTCTGGGTTAACAATATTTATAGCTGGATTAGGGGCTAATTTTGAGTTTGATTTAAAAAAAATTATTGCTTTATCTACTTTAAGTCAATTAGGTTTAATAATAAGTATTTTATCTATTGGTTATTATAAGTTAGCTTTTTTTCATTTATTAACACATGCACTTTTTAAGGCTTTATTATTTATATGTGCTGGGGTAATTATTCATAATACAAAAAATGCTCAAGATATTCGATTTATAGGGGGTTTAAGTATAAGAATACCTTTAACATGTAGTTGTTTTAATATTGCTAATTTAGCCCTATGTGGGATGCCTTTTTTG

>Vietnam-ND5-A05

CGGTTTAATATTTTATTAGATAATTCTAAGTTAGGACAATTTTTATTATTAGTTTCTGGGTTAACAATATTTATAGCTGGATTAGGGGCTAATTTTGAGTTTGATTTAAAAAAAATTATTGCTTTATCTACTTTAAGTCAATTAGGTTTAATAATAAGTATTTTATCTATTGGTTATTATAAGTTAGCTTTTTTTCATTTATTAACACATGCACTTTTTAAGGCTTTATTATTTATATGTGCTGGGGTAATTATTCATAATACAAAAAATGCTCAAGATATTCGATTTATAGGGGGTTTAAGTATAAGAATACCTTTAACATGTAGTTGTTTTAATATTGCTAATTTAGCCTTATGTGGGATGCCTTTTTTG

>Vietnam-ND5-A06

CGGTTTAATATTTTATTAGATAATTCTAAGTTAGGACAATTTTTATTATTAGTTTCTGGGTTAACAATATTTATAGCTGGATTAGGGGCTAATTTTGAGTTTGATTTAAAAAAAATTATTGCTTTATCTACTTTAAGTCAATTAGGTTTAATAATAAGTATTTTATCTATTGGTTATTATAAGTTAGCTTTTTTTCATTTATTAACACATGCACTTTTTAAGGCTTTATTATTTATATGTGCTGGGGTAATTATTCATAATACAAAAAATGCTCAAGATATTCGATTTATAGGGGGTTTAAGTATAAGAATACCTTTAACATGTAGTTGTTTTAATATTGCTAATTTAGCCTTATGTGGGATGCCTTTTTTG

>Vietnam-ND5-A07

CGGTTTAATATTTTATTAGATAATTCTAAGTTAGGACAATTTTTATTATTAGTTTCTGGGTTAACAATATTTATAGCTGGATTAGGGGCTAATTTTGAGTTTGATTTAAAAAAAATTATTGCTTTATCTACTTTAAGTCAATTAGGTTTAATAATAAGTATTTTATCTATTGGTTATTATAAGTTAGCTTTTTTTCATTTATTAACACATGCACTTTTTAAGGCTTTATTATTTATATGTGCTGGGGTAATTATTCATAATACAAAAAATGCTCAAGATATTCGATTTATAGGGGGTTTAAGTATAAGAATACCTTTAACATGTAGTTGTTTTAATATTGCTAATTTAGCCTTATGTGGGATGCCTTTTTTG

>Vietnam-ND5-A08

CGGTTTAATATTTTATTAGATAATTCTAAGTTAGGACAATTTTTATTATTAGTTTCTGGGTTAACAATATTTATAGCTGGATTAGGGGCTAATTTTGAGTTTGATTTAAAAAAAATTATTGCTTTATCTACTTTAAGTCAATTAGGTTTAATAATAAGTATTTTATCTATTGGTTATTATAAGTTAGCTTTTTTTCATTTATTAACACATGCACTTTTTAAGGCTTTATTATTTATATGTGCTGGGGTAATTATTCATAATACAAAAAATGCTCAAGATATTCGATTTATAGGGGGTTTAAGTATAAGAATACCTTTAACATGTAGTTGTTTTAATATTGCTAATTTAGCCCTATGTGGGATGCCTTTTTTG

>Vietnam-ND5-B05

CGGTTTAATATTTTATTAGATAATTCTAAGTTAGGACAATTTTTATTATTAGTTTCTGGGTTAACAATATTTATAGCTGGATTAGGGGCTAATTTTGAGTTTGATTTAAAAAAAATTATTGCTTTATCTACTTTAAGTCAATTAGGTTTAATAATAAGTATTTTATCTATTGGTTATTATAAGTTAGCTTTTTTTCATTTATTAACACATGCACTTTTTAAGGCTTTATTATTTATATGTGCTGGGGTAATTATTCATAATACAAAAAATGCTCAAGATATTCGATTTATAGGGGGTTTAAGTATAAGAATACCTTTAACATGTAGTTGTTTTAATATTGCTAATTTAGCCTTATGTGGGATGCCTTTTTTG

>Vietnam-ND5-B06

CGGTTTAATATTTTATTAGATAATTCTAAGTTAGGACAATTTTTATTATTAGTTTCTGGGTTAACAATATTTATAGCTGGATTAGGGGCTAATTTTGAGTTTGATTTAAAAAAAATTATTGCTTTATCTACTTTAAGTCAATTAGGTTTAATAATAAGTATTTTATCTATTGGTTATTATAAGTTAGCTTTTTTTCATTTATTAACACATGCACTTTTTAAGGCTTTATTATTTATATGTGCTGGGGTAATTATTCATAATACAAAAAATGCTCAAGATATTCGATTTATAGGGGGTTTAAGTATAAGAATACCTTTAACATGTAGTTGTTTTAATATTGCTAATTTAGCCTTATGTGGGATGCCTTTTTTG

>Vietnam-ND5-B07

CGGTTTAATATTTTATTAGATAATTCTAAGTTAGGACAATTTTTATTATTAGTTTCTGGGTTAACAATATTTATAGCTGGATTAGGGGCTAATTTTGAGTTTGATTTAAAAAAAATTATTGCTTTATCTACTTTAAGTCAATTAGGTTTAATAATAAGTATTTTATCTATTGGTTATTATAAGTTAGCTTTTTTTCATTTATTAACACATGCACTTTTTAAGGCTTTATTATTTATATGTGCTGGGGTAATTATTCATAATACAAAAAATGCTCAAGATATTCGATTTATAGGGGGTTTAAGTATAAGAATACCTTTAACATGTAGTTGTTTTAATATTGCTAATTTAGCCCTATGTGGGATGCCTTTTTTG

>Vietnam-ND5-B08

CGATTTAATATTTTATTAGATAATTCTAAGTTAGGACAATTTTTATTATTAGTTTCTGGATTAACAATATTTATAGCTGGATTAGGGGCTAATTTTGAGTTTGATTTAAAAAAAATTATTGCTTTATCTACTTTAAGTCAATTAGGTTTAATAATAAGTATTTTATCTATTGGTTATTATAAGTTAGCTTTTTTTCATTTATTAACACATGCACTCTTTAAGGCTTTATTATTTATATGTGCTGGAGTAATTATTCATAATACAAAAAATGCTCAAGATATTCGATTTATAGGGGGTTTAAGTATAAGAATACCTTTAACATGTAGTTGTTTTAATATTGCTAATTTAGCTTTATGTGGAATACCTTTTTTG

>Vietnam-ND5-C05

CGGTTTAATATTTTATTAGATAATTCTAAGTTAGGACAATTTTTATTATTAGTTTCTGGGTTAACAATATTTATAGCTGGATTAGGGGCTAATTTTGAGTTTGATTTAAAAAAAATTATTGCTTTATCTACTTTAAGTCAATTAGGTTTAATAATAAGTATTTTATCTATTGGTTATTATAAGTTAGCTTTTTTTCATTTATTAACACATGCACTTTTTAAGGCTTTATTATTTATATGTGCTGGGGTAATTATTCATAATACAAAAAATGCTCAAGATATTCGATTTATAGGGGGTTTAAGTATAAGAATACCTTTAACATGTAGTTGTTTTAATATTGCTAATTTAGCCTTATGTGGGATGCCTTTTTTG

>Vietnam-ND5-C06

CGATTTAATATTTTATTAGATAATTCTAAGTTAGGACAATTTTTATTATTAGTTTCTGGATTAACAATATTTATAGCTGGATTAGGGGCTAATTTTGAGTTTGATTTAAAAAAAATTATTGCTTTATCTACTTTAAGTCAATTAGGTTTAATAATAAGTATTTTATCTATTGGTTATTATAAGTTAGCTTTTTTTCATTTATTAACACATGCACTCTTTAAGGCTTTATTATTTATATGTGCTGGAGTAATTATTCATAATACAAAAAATGCTCAAGATATTCGATTTATAGGAGGTTTAAGTATAAGAATACCTTTAACATGTAGTTGTTTTAATATTGCTAATTTAGCTTTATGTGGAATACCTTTTTTG

>Vietnam-ND5-C07

CGGTTTAATATTTTATTAGATAATTCTAAGTTAGGACAATTTTTATTATTAGTTTCTGGGTTAACAATATTTATAGCTGGATTAGGGGCTAATTTTGAGTTTGATTTAAAAAAAATTATTGCTTTATCTACTTTAAGTCAATTAGGTTTAATAATAAGTATTTTATCTATTGGTTATTATAAGTTAGCTTTTTTTCATTTATTAACACATGCACTTTTTAAGGCTTTATTATTTATATGTGCTGGGGTAATTATTCATAATACAAAAAATGCTCAAGATATTCGATTTATAGGGGGTTTAAGTATAAGAATACCTTTAACATGTAGTTGTTTTAATATTGCTAATTTAGCCCTATGTGGGATGCCTTTTTTG

>Vietnam-ND5-C08

CGATTTAATATTTTATTAGATAATTCTAAGTTAGGACAATTTTTATTATTAGTTTCTGGATTAACAATATTTATAGCTGGATTAGGGGCTAATTTTGAGTTTGATTTAAAAAAAATTATTGCTTTATCTACTTTAAGTCAATTAGGTTTAATAATAAGTATTTTATCTATTGGTTATTATAAGTTAGCTTTTTTTCATTTATTAACACATGCACTCTTTAAGGCTTTATTATTTATATGTGCTGGAGTAATTATTCATAATACAAAAAATGCTCAAGATATTCGATTTATAGGGGGTTTAAGTATAAGAATACCTTTAACATGTAGTTGTTTTAATATTGCTAATTTAGCTTTATGTGGAATACCTTTTTTG

>Vietnam-ND5-D05

CGATTTAATATTTTATTAGATAATTCTAAGTTAGGACAATTTTTATTATTAGTTTCTGGATTAACAATATTTATAGCTGGATTAGGGGCTAATTTTGAATTTGATTTAAAAAAAATTATTGCTTTATCTACTTTAAGTCAATTAGGTTTAATAATAAGTATTTTATCTATTGGTTATTATAAGTTAGCTTTTTTTCATTTATTAACACATGCACTCTTTAAGGCTTTATTATTTATATGTGCTGGAGTAATTATTCATAATACAAAAAATGCTCAAGATATTCGATTTATAGGGGGTTTAAGTATAAGAATACCTTTAACATGTAGTTGTTTTAATATTGCTAATTTAGCTTTATGTGGGATACCTTTTTTG

>Vietnam-ND5-D06

CGGTTTAATATTTTATTAGATAATTCTAAGTTAGGACAATTTTTATTATTAGTTTCTGGGTTAACAATATTTATAGCTGGATTAGGGGCTAATTTTGAGTTTGATTTAAAAAAAATTATTGCTTTATCTACTTTAAGTCAATTAGGTTTAATAATAAGTATTTTATCTATTGGTTATTATAAGTTAGCTTTTTTTCATTTATTAACACATGCACTTTTTAAGGCTTTATTATTTATATGTGCTGGGGTAATTATTCATAATACAAAAAATGCTCAAGATATTCGATTTATAGGGGGTTTAAGTATAAGAATACCTTTAACATGTAGTTGTTTTAATATTGCTAATTTAGCCTTATGTGGGATGCCTTTTTTG

>Vietnam-ND5-D07

CGATTTAATATTTTATTAGATAATTCTAAGTTAGGACAATTTTTATTATTAGTTTCTGGATTAACAATATTTATAGCTGGATTAGGGGCTAATTTTGAATTTGATTTAAAAAAAATTATTGCTTTATCTACTTTAAGTCAATTAGGTTTAATAATAAGTATTTTATCTATTGGTTATTATAAGTTAGCTTTTTTTCATTTATTAACACATGCACTCTTTAAGGCTTTATTATTTATATGTGCTGGAGTAATTATTCATAATACAAAAAATGCTCAAGATATTCGATTTATAGGGGGTTTAAGTATAAGAATACCTTTAACATGTAGTTGTTTTAATATTGCTAATTTAGCTTTATGTGGGATACCTTTTTTG

>Vietnam-ND5-E05

CGGTTTAATATTTTATTAGATAATTCTAAGTTAGGACAATTTTTATTATTAGTTTCTGGGTTAACAATATTTATAGCTGGATTAGGGGCTAATTTTGAGTTTGATTTAAAAAAAATTATTGCTTTATCTACTTTAAGTCAATTAGGTTTAATAATAAGTATTTTATCTATTGGTTATTATAAGTTAGCTTTTTTTCATTTATTAACACATGCACTTTTTAAGGCTTTATTATTTATATGTGCTGGGGTAATTATTCATAATACAAAAAATGCTCAAGATATTCGATTTATAGGGGGTTTAAGTATAAGAATACCTTTAACATGTAGTTGTTTTAATATTGCTAATTTAGCCTTATGTGGGATGCCTTTTTTG

>Vietnam-ND5-E07

CGGTTTAATATTTTATTAGATAATTCTAAGTTAGGACAATTTTTATTATTAGTTTCTGGGTTAACAATATTTATAGCTGGATTAGGGGCTAATTTTGAGTTTGATTTAAAAAAAATTATTGCTTTATCTACTTTAAGTCAATTAGGTTTAATAATAAGTATTTTATCTATTGGTTATTATAAGTTAGCTTTTTTTCATTTATTAACACATGCACTTTTTAAGGCTTTATTATTTATATGTGCTGGGGTAATTATTCATAATACAAAAAATGCTCAAGATATTCGATTTATAGGGGGTTTAAGTATAAGAATACCTTTAACATGTAGTTGTTTTAATATTGCTAATTTAGCCTTATGTGGGATGCCTTTTTTG

>Vietnam-ND5-F05

CGGTTTAATATTTTATTAGATAATTCTAAGTTAGGACAATTTTTATTATTAGTTTCTGGGTTAACAATATTTATAGCTGGATTAGGGGCTAATTTTGAGTTTGATTTAAAAAAAATTATTGCTTTATCTACTTTAAGTCAATTAGGTTTAATAATAAGTATTTTATCTATTGGTTATTATAAGTTAGCTTTTTTTCATTTATTAACACATGCACTTTTTAAGGCTTTATTATTTATATGTGCTGGGGTAATTATTCATAATACAAAAAATGCTCAAGATATTCGATTTATAGGGGGTTTAAGTATAAGAATACCTTTAACATGTAGTTGTTTTAATATTGCTAATTTAGCCTTATGTGGGATGCCTTTTTTG

>Vietnam-ND5-F06

CGGTTTAATATTTTATTAGATAATTCTAAGTTAGGACAATTTTTATTATTAGTTTCTGGGTTAACAATATTTATAGCTGGATTAGGGGCTAATTTTGAGTTTGATTTAAAAAAAATTATTGCTTTATCTACTTTAAGTCAATTAGGTTTAATAATAAGTATTTTATCTATTGGTTATTATAAGTTAGCTTTTTTTCATTTATTAACACATGCACTTTTTAAGGCTTTATTATTTATATGTGCTGGGGTAATTATTCATAATACAAAAAATGCTCAAGATATTCGATTTATAGGGGGTTTAAGTATAAGAATACCTTTAACATGTAGTTGTTTTAATATTGCTAATTTAGCCTTATGTGGGATGCCTTTTTTG

>Vietnam-ND5-F08

CGATTTAATATTTTATTAGATAATTCTAAGTTAGGACAATTTTTATTATTAGTTTCTGGATTAACAATATTTATAGCTGGATTAGGGGCTAATTTTGAATTTGATTTAAAAAAAATTATTGCTTTATCTACTTTAAGTCAATTAGGTTTAATAATAAGTATTTTATCTATTGGTTATTATAAGTTAGCTTTTTTTCATTTATTAACACATGCACTCTTTAAGGCTTTATTATTTATATGTGCTGGAGTAATTATTCATAATACAAAAAATGCTCAAGATATTCGATTTATAGGGGGTTTAAGTATAAGAATACCTTTAACATGTAGTTGTTTTAATATTGCTAATTTAGCTTTATGTGGGATACCTTTTTTG

>Vietnam-ND5-G05

CGATTTAATATTTTATTAGATAATTCTAAGTTAGGACAATTTTTATTATTAGTTTCTGGATTAACAATATTTATAGCTGGATTAGGGGCTAATTTTGAGTTTGATTTAAAAAAAATTATTGCTTTATCTACTTTAAGTCAATTAGGTTTAATAATAAGTATTTTATCTATTGGTTATTATAAGTTAGCTTTTTTTCATTTATTAACACATGCACTCTTTAAGGCTTTATTATTTATATGTGCTGGAGTAATTATTCATAATACAAAAAATGCTCAAGATATTCGATTTATAGGGGGTTTAAGTATAAGAATACCTTTAACATGTAGTTGTTTTAATATTGCTAATTTAGCTTTATGTGGAATACCTTTTTTG

>Vietnam-ND5-G06

CGGTTTAATATTTTATTAGATAATTCTAAGTTAGGACAATTTTTATTATTAGTTTCTGGGTTAACAATATTTATAGCTGGATTAGGGGCTAATTTTGAGTTTGATTTAAAAAAAATTATTGCTTTATCTACTTTAAGTCAATTAGGTTTAATAATAAGTATTTTATCTATTGGTTATTATAAGTTAGCTTTTTTTCATTTATTAACACATGCACTTTTTAAGGCTTTATTATTTATATGTGCTGGGGTAATTATTCATAATACAAAAAATGCTCAAGATATTCGATTTATAGGGGGTTTAAGTATAAGAATACCTTTAACATGTAGTTGTTTTAATATTGCTAATTTAGCCTTATGTGGGATGCCTTTTTTG

>Vietnam-ND5-H03

CGGTTTAATATTTTATTAGATAATTCTAAGTTAGGACAATTTTTATTATTAGTTTCTGGGTTAACAATATTTATAGCTGGATTAGGGGCTAATTTTGAGTTTGATTTAAAAAAAATTATTGCTTTATCTACTTTAAGTCAATTAGGTTTAATAATAAGTATTTTATCTATTGGTTATTATAAGTTAGCTTTTTTTCATTTATTAACACATGCACTTTTTAAGGCTTTATTATTTATATGTGCTGGGGTAATTATTCATAATACAAAAAATGCTCAAGATATTCGATTTATAGGGGGTTTAAGTATAAGAATACCTTTAACATGTAGTTGTTTTAATATTGCTAATTTAGCCTTATGTGGGATGCCTTTTTTG

>Vietnam-ND5-H04

CGATTTAATATTTTATTAGATAATTCTAAGTTAGGACAATTTTTATTATTAGTTTCTGGATTAACAATATTTATAGCTGGATTAGGGGCTAATTTTGAATTTGATTTAAAAAAAATTATTGCTTTATCTACTTTAAGTCAATTAGGTTTAATAATAAGTATTTTATCTATTGGTTATTATAAGTTAGCTTTTTTTCATTTATTAACACATGCACTCTTTAAGGCTTTATTATTTATATGTGCTGGAGTAATTATTCATAATACAAAAAATGCTCAAGATATTCGATTTATAGGGGGTTTAAGTATAAGAATACCTTTAACATGTAGTTGTTTTAATATTGCTAATTTAGCTTTATGTGGGATACCTTTTTTG

>Vietnam-ND5-H05

CGGTTTAATATTTTATTAGATAATTCTAAGTTAGGACAATTTTTATTATTAGTTTCTGGGTTAACAATATTTATAGCTGGATTAGGGGCTAATTTTGAGTTTGATTTAAAAAAAATTATTGCTTTATCTACTTTAAGTCAATTAGGTTTAATAATAAGTATTTTATCTATTGGTTATTATAAGTTAGCTTTTTTTCATTTATTAACACATGCACTTTTTAAGGCTTTATTATTTATATGTGCTGGGGTAATTATTCATAATACAAAAAATGCTCAAGATATTCGATTTATAGGGGGTTTAAGTATAAGAATACCTTTAACATGTAGTTGTTTTAATATTGCTAATTTAGCCCTATGTGGGATGCCTTTTTTG
