# Supplementary material for: Contrasting genetic structure between mitochondrial and nuclear markers in the dengue fever mosquito from Rio de Janeiro: implications for vector control
Source: Evol Appl. 2015 Sep 7;8(9):901–15. doi: 10.1111/eva.12301 (PMC4610386; doi:10.1111/eva.12301)
Supplement: Supplementary file 7 — File S5.RADseq generated mtDNA sequences. [file eva0008-0901-sd7.docx]

>Br22-1

NNNNNNNNNNNNNNNNNNNNNNNNNNNNNNNNNNNNNNNNNNNNNNNNNNNNNNNNNNNNNNNNNNNNNNNNNNNNNNNN

AATTAATCATATTGGTTGAATATTAATAGCTATAATAAATAACGAACTTTTATGATTAACTTATTTTTTATTATATTCAATT

CAACAAATCATAAAGATATTGGAACTTTATATTTCATTTTTGGAGTATGATCTGGAATAGTCGGAACTTCTCTAAGAATT

AATTGGATTATTGGGATTTATTGTTTGAGCTCATCATATATTTACAGTAGGTATAGATGTAGATACTCGAGCTTATTTTACTTCAGCAACTATAATT

CATGATACTTATTACGTAGTTGCCCATTTTCATTACGTTTTATCTATAGGAGCTGTATTTGCTATTATAGCAGGATTTATTCATTGATACCCTTTATTAACAGGAATAGTTATAAACCCTTCATG

CTGGAATACCTCGACGATACTCAGATTTTCCTGATAGTTACTTAACTTGAAATATTATTTCTTCTTTAGGAAGAACAATT

AATTTGTCACACACAACATATATTTGCTCATTTAGTTCCTCAAGGGACTCCTCCTGTTTTAATACCTTTTATAGTATGCATTGAAACTATTAGTAATGTAATCCGACCAGGAACTTTAGCAGTACGATTGACTGCTAATATAATT

GACGAGATATTTCTCGAGAAGGAACTTTTCAAGGACTTCACACTATTCCCGTAACATTAGGATTACGATGAGGAATAATTTTATTTATTATTTCTGAAGTTTTTTTCTTTATTTCCTTCTTTTGAGCTTTTTTCCATAGTAGCTTATCTCCAACAATCGAATT

AATTGTCATTTTTCTAGAAGTCATCATTTTGGATTCGAAGCAGCTGCTTGATATTGACATTTTGTTGATGTAGTTTGACTATTCTTATATATTTCAATT

AATTTTAGGAATATATTCTTATAAAATGTTTGATCAAGGATGAAGTGAATATTTTGGAGGTCAGATATTATATAATCAAT

ATTATTCAATAACAGGGGATTTTAATAGAACTACATTGAATATGTTAAATGATAAGGGGTGAGCTATATCTTTTAGAATT

AATTCCTTTTTCTTCTTGATTACCTGCAGCTATGGCAGCACCGACTCCTGTTTCTGCTTTAGTACATTCTTCTACATTAG

AATTTATTTTCAAAATGTAAAATCTTATAATGCTGGTATATTAACAGCTTTATCTAATCGAATCGGAGATGTTGCCTTAT

CTTTACATTCAATATCTATTGTAATAACTTTTTTATTTGATTGAATAAGTTTAATATTTATATCTTTTGTTTTATTAATT

AATTTTTATTAGATTTACTTTATTTATTTTAAGATTAAAGTTTTTATTAATAGATTTAGTATATTTTATTGAATGAGAAATT

GAGGATATCAACCTGAACGTTTACAGGCGGGGATTTATTTATTATTTTATACTTTACTGGCTTCGTTGCCGTTATTAATT

AATTATGAAAGTTACTTTAGTATATTTTTTTTAACTTTTTGTGTTTGTGAAGGGGTTCTAGGGCTATCTATTTTAGTTTC

AATTATTATATTTATTTTTGGAAGTATTGTTTTTATTTCTAGTCGTAAGCATTTACTTTGTACTTTATTGAGATTAGAGT

AATTTTTATAGGAGGAATATTAGTTTTATTCATTTATGTAACTTCTCTTTCATCTAATGAAATATTTTCATTATCTATAAAACTATTTTTTTTATCTTTAAGTATAATT

AATTTTAACAGGATTATTCTTAGCAATACACTATACTGCTGATATTGAAACAGCTTTTAATAGAGTAAATCACATTTATCGTGATGTTAATAATGGTTGATTCCTACGAATT

CTGTTGATAATGCTACTTTAACTCGATTCTTTACCTTTCATTTTATTCTCCCTTTCATTGTATTAGCTTTAACTATAATT

CTTTTGATTTTGCTGAAGGGGAGTCTGAGTTAGTTTCAGGATTTAATGTAGAGTATAGAAGAGGGGGATTTGCTTTAATT

AATTTTTTTATATATCAGAAATATATATGATTTATTGTATTTTGTTTTCCTTTAGGTCTCGTTTGATTTGCATCTTGTTT

AATTTAAGCTATAAACTTTTTGTAATAAACTAGGATTAGATACCCTATTATTAAAAATAAATATTAAAATGCTAAAGTAG

>Br22-2

AATTTTATCTACTTTATTATTAAAAAGAGGAGCAGCTCCTTTTCATTTTTGATTCCCAGGAGTTATAGAAGGATTAAATT

NNNNNNNNNNNNNNNNNNNNNNNNNNNNNNNNNNNNNNNNNNNNNNNNNNNNNNNNNNNNNNNNNNNNNNNNNNNNNNNNNN

CAACAAATCATAAAGATATTGGAACTTTATATTTCATTTTTGGAGTATGATCTGGAATAGTCGGAACTTCTCTAAGAATT

NNNNNNNNNNNNNNNNNNNNNNNNNNNNNNNNNNNNNNNNNNNNNNNNNNNNNNNNNNNNNNNNNNNNNNNNNNNNNNNNNNNNNNNNNNNNNNNNN

CATGATACTTATTACGTAGTTGCCCATTTTCATTACGTTTTATCTATAGGAGCTGTATTTGCTATTATAGCAGGATTTATTCATTGATACCCTTTATTAACAGGAATAGTTATAAACCCTTCATG

CTGGAATACCTCGACGATACTCAGATTTTCCTGATAGCTACTTAACTTGAAATATTATTTCTTCTTTAGGAAGAACAATT

AATTTGTCACACACAACATATATTTGCTCATTTAGTTCCTCAAGGAACTCCTCCTGTTTTAATACCTTTTATAGTATGCATTGAAACTATTAGTAATGTAATCCGACCAGGAACTTTAGCAGTACGATTGACTGCTAATATAATT

GACGAGATATTTCTCGAGAAGGAACTTTTCAAGGACTTCACACTATTCCCGTAACATTAGGATTACGATGAGGAATAATTTTATTTATTATTTCTGAAGTTTTTTTCTTTATTTCCTTCTTTTGAGCTTTTTTCCATAGTAGCTTATCTCCAACAATCGAATT

AATTGTCATTTTTCTAGAAGTCATCATTTTGGATTCGAAGCAGCTGCTTGATATTGACATTTTGTTGATGTAGTTTGACTNNNNNNNNNNNNNNNNNNN

AATTTTAGGAATATATTCTTATAAAATGTTTGATCAAGGATGAAGTGAATATTTTGGAGGTCAGATATTATATAATCAAT

ATTATTCAATAACAGGGGATTTTAATAGAACTACATTGAATATGTTAGATGATAAGGGGTGAACTATATCTTTTAGAATT

AATTCCTTTTTCTTCTTGATTACCTGCAGCTATGGCAGCACCGACTCCTGTTTCTGCTTTAGTACATTCTTCTACATTAG

AATTTATTTTCAAAATGTAAAATCTTATAATGCTGGTATATTAACAGCTTTATCTAATCGAATCGGGGATGTTGCTTTAT

CTTTACATTCAGTATCTATTGTAATAACTTTTTTATTTGATTGAATAAGTTTAATATTTATATCTTTTGTTTTATTAATT

AATTTTTATTAGATTTACTTTATTTATTTTAAGATTAAAGTTTTTATTAATAGATTTAGTATATTTTATTGAATGAGAAATT

GAGGATATCAACCTGAACGTTTACAGGCGGGGATTTATTTATTATTTTATACTTTACTGGCTTCGTTGCCGTTATTAATT

NNNNNNNNNNNNNNNNNNNNNNNNNNNNNNNNNNNNNNNNNNNNNNNNNNNNNNNNNNNNNNNNNNNNNNNNNNNNNNNN

AATTATTATATTTATTTTTGGAAGTATTGTTTTTATTTCTAGTCGTAAGCATTTACTTTGTACTTTATTGAGATTAGAGT

AATTTTTATAGGAGGAATATTAGTTTTATTCATTTATGTAACTTCTCTTTCATCTAATGAAATATTTTCATTATCTATAAAACTATTTTTTTTATCTTTAAGTATAATT

AATTTTAACAGGATTATTCTTAGCAATACACTATACTGCTGATATTGAAACAGCTTTTAATAGAGTAAATCACATTTATCGTGATGTTAATAATGGTTGATTCCTACGAATT

CTGTTGATAATGCTACTTTAACTCGATTCTTTACATTTCATTTTATTCTCCCTTTCATTGTATTAGCTTTAACTATAATT

CTTTTGATTTTGCTGAAGGGGAGTCTGAGTTAGTTTCAGGATTTAATGTAGAATATAGAAGAGGGGGATTTGCTTTAATT

AATTTTTTTATATATCAGAAATACATATGATTTATTGTATTTTGTTTTCCTTTAGGTCTCGTTTGATTTGCATCTTGTTT

AATTTAAGCTATAAACTTTTTGTAATAAACTAGGATTAGATACCCTATTATTAAAAATAAATATTAAAATGCTAAAGTAG

>Br26-1

NNNNNNNNNNNNNNNNNNNNNNNNNNNNNNNNNNNNNNNNNNNNNNNNNNNNNNNNNNNNNNNNNNNNNNNNNNNNNNNN

AATTAATCATATTGGTTGAATATTAATAGCTATAATAAATAACGAACTTTTATGATTAACTTATTTTTTATTATATTCAATT

CAACAAATCATAAAGATATTGGAACTTTATATTTCATTTTTGGAGTATGATCTGGAATAGTCGGAACTTCTCTAAGAATT

AATTGGATTATTGGGATTTATTGTTTGAGCTCATCATATATTTACAGTAGGTATAGATGTAGATACTCGAGCTTATTTTACTTCAGCAACTATAATT

CATGATACTTATTACGTAGTTGCCCATTTTCATTACGTTTTATCTATAGGAGCTGTATTTGCTATTATAGCAGGATTTATTCATTGATACCCTTTATTAACAGGAATAGTTATAAACCCTTCATG

CTGGAATACCTCGACGATACTCAGATTTTCCTGATAGTTACTTAACTTGAAATATTATTTCTTCTTTAGGAAGAACAATT

AATTTGTCACACACAACATATATTTGCTCATTTAGTTCCTCAAGGGACTCCTCCTGTTTTAATACCTTTTATAGTATGCATTGAAACTATTAGTAATGTAATCCGACCAGGAACTTTAGCAGTACGATTGACTGCTAATATAATT

GACGAGATATTTCTCGAGAAGGAACTTTTCAAGGACTTCACACTATTCCCGTAACATTAGGATTACGATGAGGAATAATTTTATTTATTATTTCTGAAGTTTTTTTCTTTATTTCCTTCTTTTGAGCTTTTTTCCATAGTAGCTTATCTCCAACAATCGAATT

AATTGTCATTTTTCTAGAAGTCATCATTTTGGATTCGAAGCAGCTGCTTGATATTGACATTTTGTTGATGTAGTTTGACTATTCTTATATATTTCAATT

AATTTTAGGAATATATTCTTATAAAATGTTTGATCAAGGATGAAGTGAATATTTTGGAGGTCAGATATTATATAATCAAT

ATTATTCAATAACAGGGGATTTTAATAGAACTACATTGAATATGTTAAATGATAAGGGGTGAGCTATATCTTTTAGAATT

AATTCCTTTTTCTTCTTGATTACCTGCAGCTATGGCAGCACCGACTCCTGTTTCTGCTTTAGTACATTCTTCTACATTAG

AATTTATTTTCAAAATGTAAAATCTTATAATGCTGGTATATTAACAGCTTTATCTAATCGAATCGGAGATGTTGCCTTAT

CTTTACATTCAATATCTATTGTAATAACTTTTTTATTTGATTGAATAAGTTTAATATTTATATCTTTTGTTTTATTAATT

AATTTTTATTAGATTTACTTTATTTATTTTAAGATTAAAGTTTTTATTAATAGATTTAGTATATTTTATTGAATGAGAAATT

GAGGATATCAACCTGAACGTTTACAGGCGGGGATTTATTTATTATTTTATACTTTACTGGCTTCGTTGCCGTTATTAATT

AATTATGAAAGTTACTTTAGTATATTTTTTTTAACTTTTTGTGTTTGTGAAGGGGTTCTAGGGCTATCTATTTTAGTTTC

AATTATTATATTTATTTTTGGAAGTATTGTTTTTATTTCTAGTCGTAAGCATTTACTTTGTACTTTATTGAGATTAGAGT

AATTTTTATAGGAGGAATATTAGTTTTATTCATTTATGTAACTTCTCTTTCATCTAATGAAATATTTTCATTATCTATAAAACTATTTTTTTTATCTTTAAGTATAATT

AATTTTAACAGGATTATTCTTAGCAATACACTATACTGCTGATATTGAAACAGCTTTTAATAGAGTAAATCACATTTATCGTGATGTTAATAATGGTTGATTCCTACGAATT

CTGTTGATAATGCTACTTTAACTCGATTCTTTACCTTTCATTTTATTCTCCCTTTCATTGTATTAGCTTTAACTATAATT

CTTTTGATTTTGCTGAAGGGGAGTCTGAGTTAGTTTCAGGATTTAATGTAGAGTATAGAAGAGGGGGATTTGCTTTAATT

AATTTTTTTATATATCAGAAATATATATGATTTATTGTATTTTGTTTTCCTTTAGGTCTCGTTTGATTTGCATCTTGTTT

AATTTAAGCTATAAACTTTTTGTAATAAACTAGGATTAGATACCCTATTATTAAAAATAAATATTAAAATGCTAAAGTAG

>Br26-2

AATTTTATCTACTTTATTATTAAAAAGAGGGGCAGCTCCTTTTCATTTTTGATTCCCAGGAGTTATAGAAGGATTAAATT

AATTAATCATATTGGTTGAATATTAATAGCTATAATAAATAACGAACTTTTATGATTAACTTATTTTTTATTATATTCAATT

CAACAAATCATAAAGATATTGGAACTTTATATTTCATTTTTGGAGTATGATCTGGAATAGTCGGAACTTCTCTAAGAATT

AATTGGATTATTGGGATTTATTGTTTGAGCTCATCATATATTTACAGTAGGTATAGATGTAGATACTCGAGCTTATTTTACTTCAGCAACTATAATT

CATGATACTTATTACGTAGTTGCCCATTTTCATTACGTTTTATCTATAGGAGCTGTATTTGCTATTATAGCAGGATTTATTCATTGATACCCTTTATTAACAGGAATAGTTATAAACCCTTCATG

CTGGAATACCTCGACGATACTCAGATTTTCCTGATAGTTACTTAACTTGAAATATTATTTCTTCTTTAGGAAGAACAATT

AATTTGTCACACACAACATATATTTGCTCATTTAGTTCCTCAAGGGACTCCTCCTGTTTTAATACCTTTTATAGTATGCATTGAAACTATTAGTAATGTAATCCGACCAGGAACTTTAGCAGTACGATTGACTGCTAATATAATT

GACGAGATATTTCTCGAGAAGGAACTTTTCAAGGACTTCACACTATTCCCGTAACATTAGGATTACGATGAGGAATAATTTTATTTATTATTTCTGAAGTTTTTTTCTTTATTTCCTTCTTTTGAGCTTTTTTCCATAGTAGCTTATCTCCAACAATCGAATT

AATTGTCATTTTTCTAGAAGTCATCATTTTGGATTCGAAGCAGCTGCTTGATATTGACATTTTGTTGATGTAGTTTGACTATTCTTATATATTTCAATT

AATTTTAGGAATATATTCTTATAAAATGTTTGATCAAGGATGAAGTGAATATTTTGGAGGTCAGATATTATATAATCAAT

ATTATTCAATAACAGGGGATTTTAATAGAACTACATTGAATATGTTAAATGATAAGGGGTGAGCTATATCTTTTAGAATT

AATTCCTTTTTCTTCTTGATTACCTGCAGCTATGGCAGCACCGACTCCTGTTTCTGCTTTAGTACATTCTTCTACATTAG

NNNNNNNNNNNNNNNNNNNNNNNNNNNNNNNNNNNNNNNNNNNNNNNNNNNNNNNNNNNNNNNNNNNNNNNNNNNNNNNN

CTTTACATTCAATATCTATTGTAATAACTTTTTTATTTGATTGAATAAGTTTAATATTTATATCTTTTGTTTTATTAATT

AATTTTTATTAGATTTACTTTATTTATTTTAAGATTAAAGTTTTTATTAATAGATTTAGTATATTTTATTGAATGAGAAATT

GAGGATATCAACCTGAACGTTTACAGGCGGGGATTTATTTATTATTTTATACTTTACTGGCTTCGTTGCCGTTATTAATT

AATTATGAAAGTTACTTTAGTATATTTTTTTTAACTTTTTGTGTTTGTGAAGGGGTTCTAGGGCTATCTATTTTAGTTTC

AATTATTATATTTATTTTTGGAAGTATTGTTTTTATTTCTAGTCGTAAGCATTTACTTTGTACTTTATTGAGATTAGAGT

AATTTTTATAGGAGGAATATTAGTTTTATTCATTTATGTAACTTCTCTTTCATCTAATGAAATATTTTCATTATCTATAAAACTATTTTTTTTATCTTTAAGTATAATT

AATTTTAACAGGATTATTCTTAGCAATACACTATACTGCTGATATTGAAACAGCTTTTAATAGAGTAAATCACATTTATCGTGATGTTAATAATGGTTGATTCCTACGAATT

CTGTTGATAATGCTACTTTAACTCGATTCTTTACCTTTCATTTTATTCTCCCTTTCATTGTATTAGCTTTAACTATAATT

CTTTTGATTTTGCTGAAGGGGAGTCTGAGTTAGTTTCAGGATTTAATGTAGAGTATAGAAGAGGGGGATTTGCTTTAATT

AATTTTTTTATATATCAGAAATATATATGATTTATTGTATTTTGTTTTCCTTTAGGTCTCGTTTGATTTGCATCTTGTTT

AATTTAAGCTATAAACTTTTTGTAATAAACTAGGATTAGATACCCTATTATTAAAAATAAATATTAAAATGCTAAAGTAG

>Br30-1

AATTTTATCTACTTTATTATTAAAAAGAGGGGCAGCTCCTTTTCATTTTTGATTCCCAGGAGTTATAGAAGGATTAAATT

AATTAATCATATTGGTTGAATATTAATAGCTATAATAAATAACGAACTTTTATGATTAACTTATTTTTTATTATATTCAATT

CAACAAATCATAAAGATATTGGAACTTTATATTTCATTTTTGGAGTATGATCTGGAATAGTCGGAACTTCTCTAAGAATT

AATTGGATTATTGGGATTTATTGTTTGAGCTCATCATATATTTACAGTAGGTATAGATGTAGATACTCGAGCTTATTTTACTTCAGCAACTATAATT

CATGATACTTATTACGTAGTTGCCCATTTTCATTACGTTTTATCTATAGGAGCTGTATTTGCTATTATAGCAGGATTTATTCATTGATACCCTTTATTAACAGGAATAGTTATAAACCCTTCATG

CTGGAATACCTCGACGATACTCAGATTTTCCTGATAGTTACTTAACTTGAAATATTATTTCTTCTTTAGGAAGAACAATT

AATTTGTCACACACAACATATATTTGCTCATTTAGTTCCTCAAGGGACTCCTCCTGTTTTAATACCTTTTATAGTATGCATTGAAACTATTAGTAATGTAATCCGACCAGGAACTTTAGCAGTACGATTGACTGCTAATATAATT

GACGAGATATTTCTCGAGAAGGAACTTTTCAAGGACTTCACACTATTCCCGTAACATTAGGATTACGATGAGGAATAATTTTATTTATTATTTCTGAAGTTTTTTTCTTTATTTCCTTCTTTTGAGCTTTTTTCCATAGTAGCTTATCTCCAACAATCGAATT

AATTGTCATTTTTCTAGAAGTCATCATTTTGGATTCGAAGCAGCTGCTTGATATTGACATTTTGTTGATGTAGTTTGACTATTCTTATATATTTCAATT

AATTTTAGGAATATATTCTTATAAAATGTTTGATCAAGGATGAAGTGAATATTTTGGAGGTCAGATATTATATAATCAAT

ATTATTCAATAACAGGGGATTTTAATAGAACTACATTGAATATGTTAAATGATAAGGGGTGAGCTATATCTTTTAGAATT

AATTCCTTTTTCTTCTTGATTACCTGCAGCTATGGCAGCACCGACTCCTGTTTCTGCTTTAGTACATTCTTCTACATTAG

AATTTATTTTCAAAATGTAAAATCTTATAATGCTGGTATATTAACAGCTTTATCTAATCGAATCGGAGATGTTGCCTTAT

CTTTACATTCAATATCTATTGTAATAACTTTTTTATTTGATTGAATAAGTTTAATATTTATATCTTTTGTTTTATTAATT

AATTTTTATTAGATTTACTTTATTTATTTTAAGATTAAAGTTTTTATTAATAGATTTAGTATATTTTATTGAATGAGAAATT

GAGGATATCAACCTGAACGTTTACAGGCGGGGATTTATTTATTATTTTATACTTTACTGGCTTCGTTGCCGTTATTAATT

AATTATGAAAGTTACTTTAGTATATTTTTTTTAACTTTTTGTGTTTGTGAAGGGGTTCTAGGGCTATCTATTTTAGTTTC

AATTATTATATTTATTTTTGGAAGTATTGTTTTTATTTCTAGTCGTAAGCATTTACTTTGTACTTTATTGAGATTAGAGT

AATTTTTATAGGAGGAATATTAGTTTTATTCATTTATGTAACTTCTCTTTCATCTAATGAAATATTTTCATTATCTATAAAACTATTTTTTTTATCTTTAAGTATAATT

AATTTTAACAGGATTATTCTTAGCAATACACTATACTGCTGATATTGAAACAGCTTTTAATAGAGTAAATCACATTTATCGTGATGTTAATAATGGTTGATTCCTACGAATT

CTGTTGATAATGCTACTTTAACTCGATTCTTTACCTTTCATTTTATTCTCCCTTTCATTGTATTAGCTTTAACTATAATT

CTTTTGATTTTGCTGAAGGGGAGTCTGAGTTAGTTTCAGGATTTAATGTAGAGTATAGAAGAGGGGGATTTGCTTTAATT

AATTTTTTTATATATCAGAAATATATATGATTTATTGTATTTTGTTTTCCTTTAGGTCTCGTTTGATTTGCATCTTGTTT

AATTTAAGCTATAAACTTTTTGTAATAAACTAGGATTAGATACCCTATTATTAAAAATAAATATTAAAATGCTAAAGTAG

>Br30-2

NNNNNNNNNNNNNNNNNNNNNNNNNNNNNNNNNNNNNNNNNNNNNNNNNNNNNNNNNNNNNNNNNNNNNNNNNNNNNNNN

AATTAATCATATTGGTTGAATATTAATAGCTATAATAAATAACGAACTTTTATGATTAACTTATTTTTTATTATATTCAATT

CAACAAATCATAAAGATATTGGAACTTTATATTTCATTTTTGGAGTATGATCTGGAATAGTCGGAACTTCTCTAAGAATT

AATTGGATTATTGGGATTTATTGTTTGAGCTCATCATATATTTACAGTAGGTATAGATGTAGATACTCGAGCTTATTTTACTTCAGCAACTATAATT

CATGATACTTATTACGTAGTTGCCCATTTTCATTACGTTTTATCTATAGGAGCTGTATTTGCTATTATAGCAGGATTTATTCATTGATACCCTTTATTAACAGGAATAGTTATAAACCCTTCATG

CTGGAATACCTCGACGATACTCAGATTTTCCTGATAGTTACTTAACTTGAAATATTATTTCTTCTTTAGGAAGAACAATT

AATTTGTCACACACAACATATATTTGCTCATTTAGTTCCTCAAGGGACTCCTCCTGTTTTAATACCTTTTATAGTATGCATTGAAACTATTAGTAATGTAATCCGACCAGGAACTTTAGCAGTACGATTGACTGCTAATATAATT

GACGAGATATTTCTCGAGAAGGAACTTTTCAAGGACTTCACACTATTCCCGTAACATTAGGATTACGATGAGGAATAATTTTATTTATTATTTCTGAAGTTTTTTTCTTTATTTCCTTCTTTTGAGCTTTTTTCCATAGTAGCTTATCTCCAACAATCGAATT

AATTGTCATTTTTCTAGAAGTCATCATTTTGGATTCGAAGCAGCTGCTTGATATTGACATTTTGTTGATGTAGTTTGACTATTCTTATATATTTCAATT

AATTTTAGGAATATATTCTTATAAAATGTTTGATCAAGGATGAAGTGAATATTTTGGAGGTCAGATATTATATAATCAAT

ATTATTCAATAACAGGGGATTTTAATAGAACTACATTGAATATGTTAAATGATAAGGGGTGAGCTATATCTTTTAGAATT

AATTCCTTTTTCTTCTTGATTACCTGCAGCTATGGCAGCACCGACTCCTGTTTCTGCTTTAGTACATTCTTCTACATTAG

NNNNNNNNNNNNNNNNNNNNNNNNNNNNNNNNNNNNNNNNNNNNNNNNNNNNNNNNNNNNNNNNNNNNNNNNNNNNNNNN

CTTTACATTCAATATCTATTGTAATAACTTTTTTATTTGATTGAATAAGTTTAATATTTATATCTTTTGTTTTATTAATT

AATTTTTATTAGATTTACTTTATTTATTTTAAGATTAAAGTTTTTATTAATAGATTTAGTATATTTTATTGAATGAGAAATT

GAGGATATCAACCTGAACGTTTACAGGCGGGGATTTATTTATTATTTTATACTTTACTGGCTTCGTTGCCGTTATTAATT

AATTATGAAAGTTACTTTAGTATATTTTTTTTAACTTTTTGTGTTTGTGAAGGGGTTCTAGGGCTATCTATTTTAGTTTC

AATTATTATATTTATTTTTGGAAGTATTGTTTTTATTTCTAGTCGTAAGCATTTACTTTGTACTTTATTGAGATTAGAGT

AATTTTTATAGGAGGAATATTAGTTTTATTCATTTATGTAACTTCTCTTTCATCTAATGAAATATTTTCATTATCTATAAAACTATTTTTTTTATCTTTAAGTATAATT

AATTTTAACAGGATTATTCTTAGCAATACACTATACTGCTGATATTGAAACAGCTTTTAATAGAGTAAATCACATTTATCGTGATGTTAATAATGGTTGATTCCTACGAATT

CTGTTGATAATGCTACTTTAACTCGATTCTTTACCTTTCATTTTATTCTCCCTTTCATTGTATTAGCTTTAACTATAATT

CTTTTGATTTTGCTGAAGGGGAGTCTGAGTTAGTTTCAGGATTTAATGTAGAGTATAGAAGAGGGGGATTTGCTTTAATT

AATTTTTTTATATATCAGAAATATATATGATTTATTGTATTTTGTTTTCCTTTAGGTCTCGTTTGATTTGCATCTTGTTT

AATTTAAGCTATAAACTTTTTGTAATAAACTAGGATTAGATACCCTATTATTAAAAATAAATATTAAAATGCTAAAGTAG

>Br32-1

AATTTTATCTACTTTATTATTAAAAAGAGGGGCAGCTCCTTTTCATTTTTGATTCCCAGGAGTTATAGAAGGATTAAATT

AATTAATCATATTGGTTGAATATTAATAGCTATAATAAATAACGAACTTTTATGATTAACTTATTTTTTATTATATTCAATT

CAACAAATCATAAAGATATTGGAACTTTATATTTCATTTTTGGAGTATGATCTGGAATAGTCGGAACTTCTCTAAGAATT

AATTGGATTATTGGGATTTATTGTTTGAGCTCATCATATATTTACAGTAGGTATAGATGTAGATACTCGAGCTTATTTTACTTCAGCAACTATAATT

CATGATACTTATTACGTAGTTGCCCATTTTCATTACGTTTTATCTATAGGAGCTGTATTTGCTATTATAGCAGGATTTATTCATTGATACCCTTTATTAACAGGAATAGTTATAAACCCTTCATG

CTGGAATACCTCGACGATACTCAGATTTTCCTGATAGTTACTTAACTTGAAATATTATTTCTTCTTTAGGAAGAACAATT

AATTTGTCACACACAACATATATTTGCTCATTTAGTTCCTCAAGGGACTCCTCCTGTTTTAATACCTTTTATAGTATGCATTGAAACTATTAGTAATGTAATCCGACCAGGAACTTTAGCAGTACGATTGACTGCTAATATAATT

GACGAGATATTTCTCGAGAAGGAACTTTTCAAGGACTTCACACTATTCCCGTAACATTAGGATTACGATGAGGAATAATTTTATTTATTATTTCTGAAGTTTTTTTCTTTATTTCCTTCTTTTGAGCTTTTTTCCATAGTAGCTTATCTCCAACAATCGAATT

AATTGTCATTTTTCTAGAAGTCATCATTTTGGATTCGAAGCAGCTGCTTGATATTGACATTTTGTTGATGTAGTTTGACTATTCTTATATATTTCAATT

AATTTTAGGAATATATTCTTATAAAATGTTTGATCAAGGATGAAGTGAATATTTTGGAGGTCAGATATTATATAATCAAT

ATTATTCAATAACAGGGGATTTTAATAGAACTACATTGAATATGTTAAATGATAAGGGGTGAGCTATATCTTTTAGAATT

AATTCCTTTTTCTTCTTGATTACCTGCAGCTATGGCAGCACCGACTCCTGTTTCTGCTTTAGTACATTCTTCTACATTAG

AATTTATTTTCAAAATGTAAAATCTTATAATGCTGGTATATTAACAGCTTTATCTAATCGAATCGGAGATGTTGCTTTAT

CTTTACATTCAATATCTATTGTAATAACTTTTTTATTTGATTGAATAAGTTTAATATTTATATCTTTTGTTTTATTAATT

AATTTTTATTAGATTTACTTTATTTATTTTAAGATTAAAGTTTTTATTAATAGATTTAGTATATTTTATTGAATGAGAAATT

GAGGATATCAACCTGAACGTTTACAGGCGGGGATTTATTTATTATTTTATACTTTACTGGCTTCGTTGCCGTTATTAATT

AATTATGAAAGTTACTTTAGTATATTTTTTTTAACTTTTTGTGTTTGTGAAGGGGTTCTAGGGCTATCTATTTTAGTTTC

AATTATTATATTTATTTTTGGAAGTATTGTTTTTATTTCTAGTCGTAAGCATTTACTTTGTACTTTATTGAGATTAGAGT

AATTTTTATAGGAGGAATATTAGTTTTATTCATTTATGTAACTTCTCTTTCATCTAATGAAATATTTTCATTATCTATAAAACTATTTTTTTTATCTTTAAGTATAATT

AATTTTAACAGGATTATTCTTAGCAATACACTATACTGCTGATATTGAAACAGCTTTTAATAGAGTAAATCACATTTATCGTGATGTTAATAATGGTTGATTCCTACGAATT

CTGTTGATAATGCTACTTTAACTCGATTCTTTACCTTTCATTTTATTCTCCCTTTCATTGTATTAGCTTTAACTATAATT

CTTTTGATTTTGCTGAAGGGGAGTCTGAGTTAGTTTCAGGATTTAATGTAGAGTATAGAAGAGGGGGATTTGCTTTAATT

AATTTTTTTATATATCAGAAATATATATGATTTATTGTATTTTGTTTTCCTTTAGGTCTCGTTTGATTTGCATCTTGTTT

AATTTAAGCTATAAACTTTTTGTAATAAACTAGGATTAGATACCCTATTATTAAAAATAAATATTAAAATGCTAAAGTAG

>Br32-2

NNNNNNNNNNNNNNNNNNNNNNNNNNNNNNNNNNNNNNNNNNNNNNNNNNNNNNNNNNNNNNNNNNNNNNNNNNNNNNNN

AATTAATCATATTGGTTGAATATTAATAGCTATAATAAATAACGAACTTTTATGATTAACTTATTTTTTATTATATTCAATT

CAACAAATCATAAAGATATTGGAACTTTATATTTCATTTTTGGAGTATGATCTGGAATAGTCGGAACTTCTCTAAGAATT

AATTGGATTATTGGGATTTATTGTTTGAGCTCATCATATATTTACAGTAGGTATAGATGTAGATACTCGAGCTTATTTTACTTCAGCAACTATAATT

CATGATACTTATTACGTAGTTGCCCATTTTCATTACGTTTTATCTATAGGAGCTGTATTTGCTATTATAGCAGGATTTATTCATTGATACCCTTTATTAACAGGAATAGTTATAAACCCTTCATG

CTGGAATACCTCGACGATACTCAGATTTTCCTGATAGTTACTTAACTTGAAATATTATTTCTTCTTTAGGAAGAACAATT

AATTTGTCACACACAACATATATTTGCTCATTTAGTTCCTCAAGGGACTCCTCCTGTTTTAATACCTTTTATAGTATGCATTGAAACTATTAGTAATGTAATCCGACCAGGAACTTTAGCAGTACGATTGACTGCTAATATAATT

GACGAGATATTTCTCGAGAAGGAACTTTTCAAGGACTTCACACTATTCCCGTAACATTAGGATTACGATGAGGAATAATTTTATTTATTATTTCTGAAGTTTTTTTCTTTATTTCCTTCTTTTGAGCTTTTTTCCATAGTAGCTTATCTCCAACAATCGAATT

AATTGTCATTTTTCTAGAAGTCATCATTTTGGATTTGAAGCAGCTGCTTGATATTGACATTTTGTTGATGTAGTTTGACTATTCTTATATATTTCAATT

AATTTTAGGAATATATTCTTATAAAATGTTTGATCAAGGATGAAGTGAATATTTTGGAGGTCAGATATTATATAATCAAT

ATTATTCAATAACAGGGGATTTTAATAGAACTACATTGAATATGTTAAATGATAAGGGGTGAGCTATATCTTTTAGAATT

AATTCCTTTTTCTTCTTGATTACCTGCAGCTATGGCAGCACCGACTCCTGTTTCTGCTTTAGTACATTCTTCTACATTAG

AATTTATTTTCAAAATGTAAAATCTTATAATGCTGGTATATTAACAGCTTTATCTAATCGAATCGGAGATGTTGCCTTAT

CTTTACATTCAATATCTATTGTAATAACTTTTTTATTTGATTGAATAAGTTTAATATTTATATCTTTTGTTTTATTAATT

AATTTTTATTAGATTTACTTTATTTATTTTAAGATTAAAGTTTTTATTAATAGATTTAGTATATTTTATTGAATGAGAAATT

GAGGATATCAACCTGAACGTTTACAGGCGGGGATTTATTTATTATTTTATACTTTACTGGCTTCGTTGCCGTTATTAATT

AATTATGAAAGTTACTTTAGTATATTTTTTTTAACTTTTTGTGTTTGTGAAGGGGTTCTAGGGCTATCTATTTTAGTTTC

AATTATTATATTTATTTTTGGAAGTATTGTTTTTATTTCTAGTCGTAAGCATTTACTTTGTACTTTATTGAGATTAGAGT

AATTTTTATAGGAGGAATATTAGTTTTATTCATTTATGTAACTTCTCTTTCATCTAATGAAATATTTTCATTATCTATAAAACTATTTTTTTTATCTTTAAGTATAATT

AATTTTAACAGGATTATTCTTAGCAATACACTATACTGCTGATATTGAAACAGCTTTTAATAGAGTAAATCACATTTATCGTGATGTTAATAATGGTTGATTCCTACGAATT

CTGTTGATAATGCTACTTTAACTCGATTCTTTACCTTTCATTTTATTCTCCCTTTCATTGTATTAGCTTTAACTATAATT

CTTTTGATTTTGCTGAAGGGGAGTCTGAGTTAGTTTCAGGATTTAATGTAGAGTATAGAAGAGGGGGATTTGCTTTAATT

AATTTTTTTATATATCAGAAATATATATGATTTATTGTATTTTGTTTTCCTTTAGGTCTCGTTTGATTTGCATCTTGTTT

AATTTAAGCTATAAACTTTTTGTAATAAACTAGGATTAGATACCCTATTATTAAAAATAAATATTAAAATGCTAAAGTAG

>Br46-1

AATTTTATCTACTTTATTATTAAAAAGAGGGGCAGCTCCTTTTCATTTTTGATTCCCAGGAGTTATAGAAGGATTAAATT

AATTAATCATATTGGTTGAATATTAATAGCTATAATAAATAACGAACTTTTATGATTAACTTATTTTTTATTATATTCAATT

CAACAAATCATAAAGATATTGGAACTTTATATTTCATTTTTGGAGTATGATCTGGAATAGTCGGAACTTCTCTAAGAATT

AATTGGATTATTGGGATTTATTGTTTGAGCTCATCATATATTTACAGTAGGTATAGATGTAGATACTCGAGCTTATTTTACTTCAGCAACTATAATT

CATGATACTTATTACGTAGTTGCCCATTTTCATTACGTTTTATCTATAGGAGCTGTATTTGCTATTATAGCAGGATTTATTCATTGATACCCTTTATTAACAGGAATAGTTATAAACCCTTCATG

CTGGAATACCTCGACGATACTCAGATTTTCCTGATAGTTACTTAACTTGAAATATTATTTCTTCTTTAGGAAGAACAATT

AATTTGTCACACACAACATATATTTGCTCATTTAGTTCCTCAAGGGACTCCTCCTGTTTTAATACCTTTTATAGTATGCATTGAAACTATTAGTAATGTAATCCGACCAGGAACTTTAGCAGTACGATTGACTGCTAATATAATT

GACGAGATATTTCTCGAGAAGGAACTTTTCAAGGACTTCACACTATTCCCGTAACATTAGGATTACGATGAGGAATAATTTTATTTATTATTTCTGAAGTTTTTTTCTTTATTTCCTTCTTTTGAGCTTTTTTCCATAGTAGCTTATCTCCAACAATCGAATT

AATTGTCATTTTTCTAGAAGTCATCATTTTGGATTCGAAGCAGCTGCTTGATATTGACATTTTGTTGATGTAGTTTGACTATTCTTATATATTTCAATT

AATTTTAGGAATATATTCTTATAAAATGTTTGATCAAGGATGAAGTGAATATTTTGGAGGTCAGATATTATATAATCAAT

ATTATTCAATAACAGGGGATTTTAATAGAACTACATTGAATATGTTAAATGATAAGGGGTGAGCTATATCTTTTAGAATT

AATTCCTTTTTCTTCTTGATTACCTGCAGCTATGGCAGCACCGACTCCTGTTTCTGCTTTAGTACATTCTTCTACATTAG

AATTTATTTTCAAAATGTAAAATCTTATAATGCTGGTATATTAACAGCTTTATCTAATCGAATCGGAGATGTTGCCTTAT

CTTTACATTCAATATCTATTGTAATAACTTTTTTATTTGATTGAATAAGTTTAATATTTATATCTTTTGTTTTATTAATT

AATTTTTATTAGATTTACTTTATTTATTTTAAGATTAAAGTTTTTATTAATAGATTTAGTATATTTTATTGAATGAGAAATT

GAGGATATCAACCTGAACGTTTACAGGCGGGGATTTATTTATTATTTTATACTTTACTGGCTTCGTTGCCGTTATTAATT

AATTATGAAAGTTACTTTAGTATATTTTTTTTAACTTTTTGTGTTTGTGAAGGGGTTCTAGGGCTATCTATTTTAGTTTC

AATTATTATATTTATTTTTGGAAGTATTGTTTTTATTTCTAGTCGTAAGCATTTACTTTGTACTTTATTGAGATTAGAGT

AATTTTTATAGGAGGAATATTAGTTTTATTCATTTATGTAACTTCTCTTTCATCTAATGAAATATTTTCATTATCTATAAAACTATTTTTTTTATCTTTAAGTATAATT

AATTTTAACAGGATTATTCTTAGCAATACACTATACTGCTGATATTGAAACAGCTTTTAATAGAGTAAATCACATTTATCGTGATGTTAATAATGGTTGATTCCTACGAATT

CTGTTGATAATGCTACTTTAACTCGATTCTTTACCTTTCATTTTATTCTCCCTTTCATTGTATTAGCTTTAACTATAATT

CTTTTGATTTTGCTGAAGGGGAGTCTGAGTTAGTTTCAGGATTTAATGTAGAGTATAGAAGAGGGGGATTTGCTTTAATT

AATTTTTTTATATATCAGAAATATATATGATTTATTGTATTTTGTTTTCCTTTAGGTCTCGTTTGATTTGCATCTTGTTT

AATTTAAGCTATAAACTTTTTGTAATAAACTAGGATTAGATACCCTATTATTAAAAATAAATATTAAAATGCTAAAGTAG

>Br46-2

AATTTTATCTACTTTATTATTAAAAAGAGGGGCAGCTCCTTTTCATTTTTGATTCCCAGGAGTTATAGAAGGATTAAATT

AATTAATCATATTGGTTGAATATTAATAGCTATAATAAATAACGAACTTTTATGATTAACTTATTTTTTATTATATTCAATT

CAACAAATCATAAAGATATTGGAACTTTATATTTCATTTTTGGAGTATGATCTGGAATAGTCGGAACTTCTCTAAGAATT

AATTGGATTATTGGGATTTATTGTTTGAGCTCATCATATATTTACAGTAGGTATAGATGTAGATACTCGAGCTTATTTTACTTCAGCAACTATAATT

CATGATACTTATTACGTAGTTGCCCATTTTCATTACGTTTTATCTATAGGAGCTGTATTTGCTATTATAGCAGGATTTATTCATTGATACCCTTTATTAACAGGAATAGTTATAAACCCTTCATG

CTGGAATACCTCGACGATACTCAGATTTTCCTGATAGTTACTTAACTTGAAATATTATTTCTTCTTTAGGAAGAACAATT

AATTTGTCACACACAACATATATTTGCTCATTTAGTTCCTCAAGGGACTCCTCCTGTTTTAATACCTTTTATAGTATGCATTGAAACTATTAGTAATGTAATCCGACCAGGAACTTTAGCAGTACGATTGACTGCTAATATAATT

GACGAGATATTTCTCGAGAAGGAACTTTTCAAGGACTTCACACTATTCCCGTAACATTAGGATTACGATGAGGAATAATTTTATTTATTATTTCTGAAGTTTTTTTCTTTATTTCCTTCTTTTGAGCTTTTTTCCATAGTAGCTTATCTCCAACAATCGAATT

AATTGTCATTTTTCTAGAAGTCATCATTTTGGATTCGAAGCAGCTGCTTGATATTGACATTTTGTTGATGTAGTTTGACTATTCTTATATATTTCAATT

AATTTTAGGAATATATTCTTATAAAATGTTTGATCAAGGATGAAGTGAATATTTTGGAGGTCAGATATTATATAATCAAT

ATTATTCAATAACAGGGGATTTTAATAGAACTACATTGAATATGTTAAATGATAAGGGGTGAGCTATATCTTTTAGAATT

AATTCCTTTTTCTTCTTGATTACCTGCAGCTATGGCAGCACCGACTCCTGTTTCTGCTTTAGTACATTCTTCTACATTAG

AATTTATTTTCAAAATGTAAAATCTTATAATGCTGGTATATTAACAGCTTTATCTAATCGAATCGGAGATGTTGCCTTAT

CTTTACATTCAATATCTATTGTAATAACTTTTTTATTTGATTGAATAAGTTTAATATTTATATCTTTTGTTTTATTAATT

AATTTTTATTAGATTTACTTTATTTATTTTAAGATTAAAGTTTTTATTAATAGATTTAGTATATTTTATTGAATGAGAAATT

GAGGATATCAACCTGAACGTTTACAGGCGGGGATTTATTTATTATTTTATACTTTACTGGCTTCGTTGCCGTTATTAATT

AATTATGAAAGTTACTTTAGTATATTTTTTTTAACTTTTTGTGTTTGTGAAGGGGTTCTAGGGCTATCTATTTTAGTTTC

AATTATTATATTTATTTTTGGAAGTATTGTTTTTATTTCTAGTCGTAAGCATTTACTTTGTACTTTATTGAGATTAGAGT

AATTTTTATAGGAGGAATATTAGTTTTATTCATTTATGTAACTTCTCTTTCATCTAATGAAATATTTTCATTATCTATAAAACTATTTTTTTTATCTTTAAGTATAATT

AATTTTAACAGGATTATTCTTAGCAATACACTATACTGCTGATATTGAAACAGCTTTTAATAGAGTAAATCACATTTATCGTGATGTTAATAATGGTTGATTCCTACGAATT

CTGTTGATAATGCTACTTTAACTCGATTCTTTACCTTTCATTTTATTCTCCCTTTCATTGTATTAGCTTTAACTATAATT

CTTTTGATTTTGCTGAAGGGGAGTCTGAGTTAGTTTCAGGATTTAATGTAGAGTATAGAAGAGGGGGATTTGCTTTAATT

AATTTTTTTATATATCAGAAATATATATGATTTATTGTATTTTGTTTTCCTTTAGGTCTCGTTTGATTTGCATCTTGTTT

AATTTAAGCTATAAACTTTTTGTAATAAACTAGGATTAGATACCCTATTATTAAAAATAAATATTAAAATGCTAAAGTAG

>Br47-1

AATTTTATCTACTTTATTATTAAAAAGAGGGGCAGCTCCTTTTCATTTTTGATTCCCAGGAGTTATAGAAGGATTAAATT

NNNNNNNNNNNNNNNNNNNNNNNNNNNNNNNNNNNNNNNNNNNNNNNNNNNNNNNNNNNNNNNNNNNNNNNNNNNNNNNNNN

CAACAAATCATAAAGATATTGGAACTTTATATTTCATTTTTGGAGTATGATCCGGAATAGTCGGAACTTCTCTAAGAATT

AATTGGATTATTGGGATTTATTGTTTGAGCTCATCATATATTTACAGTAGGTATAGACGTAGATACTCGAGCTTATTTTACTTCAGCAACTATAATT

CATGATACTTATTACGTAGTTGCCCATTTTCATTATGTTTTATCTATAGGAGCTGTATTTGCTATTATAGCAGGATTTATTCATTGATACCCTTTATTAACAGGAATAGTTATAAACCCTTCATG

CTGGAATACCTCGACGATACTCAGATTTTCCCGATAGTTACTTAACTTGAAATATTATTTCTTCTTTAGGAAGAACAATT

AATTTGTCACACACAACATATATTTGCTCATTTAGTCCCTCAAGGAACTCCCCCTGTTTTAATACCTTTTATAGTATGCATTGAAACTATTAGTAATGTAATCCGACCAGGAACTTTAGCAGTACGATTAACTGCTAATATAATT

GACGAGATATTTCTCGAGAAGGAACTTTTCAAGGACTTCATACTATTCCCGTAACATTAGGATTACGATGAGGAATAATTTTATTTATTATTTCTGAAGTTTTTTTCTTTATTTCCTTCTTTTGAGCTTTTTTCCATAGTAGCTTATCCCCAACAATCGAATT

AATTGTCATTTTTCTAGAAGTCATCATTTTGGATTTGAAGCAGCTGCTTGATATTGACATTTTGTTGATGTAGTTTGACTATTCTTATATATTTCAATT

AATTTTAGGAATATATTCTTATAAAATGTTTGATCAAGGATGAAGTGAATATTTTGGAGGTCAGATATTATATAATCAAT

ATTATTCAATAACAGGGGATTTTAATAGAACTACATTGAATATATTAAATGATAAGGGGTGAACTATATCTTTTAGAATT

NNNNNNNNNNNNNNNNNNNNNNNNNNNNNNNNNNNNNNNNNNNNNNNNNNNNNNNNNNNNNNNNNNNNNNNNNNNNNNNN

AATTTATTTTCAAAATGTAAAATCTTATAATGCTGGTATATTAACAGCTTTATCTAATCGAATCGGAGATGTTGCCTTAT

CTTTACATTCAATATCTATTGTAATAACTTTTTTATTTGATTGAATAAGTTTAATATTTATATCTTTTGTTTTATTAATT

AATTTTTATTAGATTTACTTTATTTATTTTGAGATTAAAGTTTTTATTAATAGATTTAGTATATTTTATTGAATGAGAAATT

GAGGGTATCAACCTGAACGTTTACAGGCGGGGATTTATTTATTATTTTATACTTTACTGGCTTCGTTGCCGTTATTAATT

AATTATGAAAGTTACTTTAGTATATTTTTTTTAACTTTTTGTGTTTGTGAAGGGGTTCTAGGGTTATCTATTTTAGTTTC

AATTATTATATTTATTTTTGGAAGTATTGTTTTTATTTCTAGTCGTAAGCATTTACTTTGTACTTTATTGAGATTAGAGT

AATTTTTATAGGAGGAATATTAGTTTTATTCATTTATGTTACTTCTCTTTCATCTAATGAAATATTTTCATTATCTATAAAACTATTTTTTTTATCTTTAAGTATAATT

AATTTTAACAGGATTATTCTTAGCAATACACTATACTGCTGATATTGAAACAGCTTTTAATAGAGTAAATCACATTTATCGTGATGTTAATAATGGTTGATTCCTACGAATT

CTGTTGATAATGCTACTTTAACTCGATTCTTTACATTTCATTTTATTTTACCTTTCATTGTATTAGCTTTAACTATAATT

CTTTTGATTTTGCTGAAGGGGAGTCTGAGTTAGTTTCAGGATTTAATGTAGAATATAGAAGAGGGGGATTTGCTTTAATT

AATTTTTTTATATATCAGAAATATATATGATTTATTGTATTTTGTTTTCCCTTAGGTCTCGTTTGATTTGCATCTTGTTT

AATTTAAGCTATAAACTTTTTGTAATAAACTAGGATTAGATACCCTATTATTAAAAATAAATATTAAAATGCTAAAGTAG

>Br47-2

AATTTTATCTACTTTATTATTAAAAAGAGGGGCAGCTCCTTTTCATTTTTGATTCCCAGGAGTTATAGAAGGATTAAATT

AATTAATCATATTGGTTGAATATTAATATCTATAATAAATAACGAACTTTTATGGCTAGCTTATTTTTTATTATATTCAATT

NNNNNNNNNNNNNNNNNNNNNNNNNNNNNNNNNNNNNNNNNNNNNNNNNNNNNNNNNNNNNNNNNNNNNNNNNNNNNNNN

AATTGGATTATTGGGATTTATTGTTTGAGCTCATCATATATTTACAGTAGGTATAGACGTAGATACTCGAGCTTATTTTANNNNNNNNNNNNNNNNN

CATGATACTTATTACGTAGTTGCCCATTTTCATTATGTTTTATCTATAGGAGCTGTATTTGCTATTATAGCAGGATTTATTCATTGATACCCTTTATTAACAGGAATAGTTATAAACCCTTCATG

CTGGAATACCTCGACGATACTCAGATTTTCCCGATAGTTACTTAACTTGAAATATTATTTCTTCTTTAGGAAGAACAATT

AATTTGTCACACACAACATATATTTGCTCATTTAGTCCCTCAAGGAACTCCCCCTGTTTTAATACCTTTTATAGTATGCATTGAAACTATTAGTAATGTAATCCGACCAGGAACTTTAGCAGTACGATTAACTGCTAATATAATT

GACGAGATATTTCTCGAGAAGGAACTTTTCAAGGACTTCATACTATTCCCGTAACATTAGGATTACGATGAGGAATAATTTTATTTATTATTTCTGAAGTTTTTTTCTTTATTTCCTTCTTTTGAGCTTTTTTCCATAGTAGCTTATCCCCAACAATCGAATT

AATTGTCATTTTTCTAGAAGTCATCATTTTGGATTTGAAGCAGCTGCTTGATATTGACATTTTGTTGATGTAGTTTGACTATTCTTATATATTTCAATT

AATTTTAGGAATATATTCTTATAAAATGTTTGATCAAGGATGAAGTGAATATTTTGGAGGTCAGATATTATATAATCAAT

ATTATTCAATAACAGGGGATTTTAATAGAACTACATTGAATATATTAAATGATAAGGGGTGAACTATATCTTTTAGAATT

NNNNNNNNNNNNNNNNNNNNNNNNNNNNNNNNNNNNNNNNNNNNNNNNNNNNNNNNNNNNNNNNNNNNNNNNNNNNNNNN

AATTTATTTTCAAAATGTAAAATCTTATAATGCTGGTATATTAACAGCTTTATCTAATCGAATCGGAGATGTTGCCTTAT

NNNNNNNNNNNNNNNNNNNNNNNNNNNNNNNNNNNNNNNNNNNNNNNNNNNNNNNNNNNNNNNNNNNNNNNNNNNNNNNN

AATTTTTATTAGATTTACTTTATTTATTTTGAGATTAAAGTTTTTATTAATAGATTTAGTATATTTTATTGAATGAGAAATT

GAGGGTATCAACCTGAACGTTTACAGGCGGGGATTTATTTATTATTTTATACTTTACTGGCTTCGTTGCCGTTATTAATT

NNNNNNNNNNNNNNNNNNNNNNNNNNNNNNNNNNNNNNNNNNNNNNNNNNNNNNNNNNNNNNNNNNNNNNNNNNNNNNNN

NNNNNNNNNNNNNNNNNNNNNNNNNNNNNNNNNNNNNNNNNNNNNNNNNNNNNNNNNNNNNNNNNNNNNNNNNNNNNNNN

AATTTTTATAGGAGGAATATTAGTTTTATTCATTTATGTTACTTCTCTTTCATCTAATGAAATATTTTCATTATCTATAAAACTATTTTTTTTATCTTTAAGTATAATT

AATTTTAACAGGATTATTCTTAGCAATACACTATACTGCTGATATTGAAACAGCTTTTAATAGAGTAAATCACATTTATCGTGATGTTAATAATGGTTGATTCCTACGAATT

CTGTTGATAATGCTACTTTAACTCGATTCTTTACATTTCATTTTATTTTACCTTTCATTGTATTAGCTTTAACTATAATT

CTTTTGATTTTGCTGAAGGGGAGTCTGAGTTAGTTTCAGGATTTAATGTAGAATATAGAAGAGGGGGATTTGCTTTAATT

AATTTTTTTATATATCAGAAATATATATGATTTATTGTATTTTGTTTTCCCTTAGGTCTCGTTTGATTTGCATCTTGTTT

AATTTAAGCTATAAACTTTTTGTAATAAACTAGGATTAGATACCCTATTATTAAAAATAAATATTAAAATGCTAAAGTAG

>Br48-1

AATTTTATCTACTTTATTATTAAAAAGAGGAGCAGCTCCTTTTCATTTTTGATTCCCAGGAGTTATAGAAGGATTAAATT

AATTAATCATATTGGTTGAATATTAATAGCTATAATAAATAACGAACTTTTATGATTAACTTATTTTTTATTATATTCAATT

CAACAAATCATAAAGATATTGGAACTTTATATTTCATTTTTGGAGTATGATCTGGAATAGTCGGAACTTCTCTAAGAATT

AATTGGATTATTAGGATTTATTGTTTGAGCTCATCATATATTTACAGTAGGTATAGACGTAGATACTCGAGCTTATTTTACTTCAGCAACTATAATT

CATGATACTTATTACGTAGTTGCCCATTTTCATTACGTTTTATCTATAGGAGCTGTATTTGCTATTATAGCAGGATTTATTCATTGATACCCTTTATTAACAGGAATAGTTATAAACCCTTCATG

CTGGAATACCTCGACGATACTCAGATTTTCCTGATAGCTACTTAACTTGAAATATTATTTCTTCTTTAGGAAGAACAATT

AATTTGTCACACACAACATATATTTGCTCATTTAGTTCCTCAAGGAACTCCTCCTGTTTTAATACCTTTTATAGTATGCATTGAAACTATTAGTAATGTAATCCGACCAGGAACTTTAGCAGTACGATTGACTGCTAATATAATT

GACGAGATATTTCTCGAGAAGGAACTTTTCAAGGACTTCACACTATTCCCGTAACATTAGGATTACGATGAGGAATAATTTTATTTATTATTTCTGAAGTTTTTTTCTTTATTTCCTTCTTTTGAGCTTTTTTCCATAGTAGCTTATCTCCAACAATCGAATT

AATTGTCATTTTTCTAGAAGTCATCATTTTGGATTCGAAGCAGCTGCTTGATATTGACATTTTGTTGATGTAGTTTGACTATTCTTATATATTTCAATT

AATTTTAGGAATATATTCTTATAAAATGTTTGATCAAGGATGAAGTGAATATTTTGGAGGTCAGATATTATATAATCAAT

ATTATTCAATAACAGGGGATTTTAATAGAACTACATTGAATATGTTAGATGATAAGGGGTGAACTATATCTTTTAGAATT

AATTCCTTTTTCTTCTTGATTACCTGCAGCTATGGCAGCACCGACTCCTGTTTCTGCTTTAGTACATTCTTCTACATTAG

AATTTATTTTCAAAATGTAAAATCTTATAATGCTGGTATATTAACAGCTTTATCTAATCGAATCGGGGATGTTGCTTTAT

CTTTACATTCAGTATCTATTGTAATAACTTTTTTATTTGATTGAATAAGTTTAATATTTATATCTTTTGTTTTATTAATT

AATTTTTATTAGATTTACTTTATTTATTTTAAGATTAAAGTTTTTATTAATAGATTTAGTATATTTTATTGAATGAGAAATT

GAGGATATCAACCTGAACGTTTACAGGCGGGGATTTATTTATTATTTTATACTTTACTGGCTTCGTTGCCGTTATTAATT

AATTATGAAAGTTACTTTAGTATATTTTTTTTAACTTTTTGTGTTTGTGAAGGGGTTCTAGGGCTGTCCATTTTAGTTTC

AATTATTATATTTATTTTTGGAAGTATTGTTTTTATTTCTAGTCGTAAGCATTTACTTTGTACTTTATTGAGATTAGAGT

AATTTTTATAGGAGGAATATTAGTTTTATTCATTTATGTAACTTCTCTTTCATCTAATGAAATATTTTCATTATCTATAAAACTATTTTTTTTATCTTTAAGTATAATT

AATTTTAACAGGATTATTCTTAGCAATACACTATACTGCTGATATTGAAACAGCTTTTAATAGAGTAAATCACATTTATCGTGATGTTAATAATGGTTGATTCCTACGAATT

CTGTTGATAATGCTACTTTAACTCGATTCTTTACATTTCATTTTATTCTCCCTTTCATTGTATTAGCTTTAACTATAATT

CTTTTGATTTTGCTGAAGGGGAGTCTGAGTTAGTTTCAGGATTTAATGTAGAATATAGAAGAGGGGGATTTGCTTTAATT

AATTTTTTTATATATCAGAAATATATATGATTTATTGTATTTTGTTTTCCTTTAGGTCTCGTTTGATTTGCATCTTGTTT

AATTTAAGCTATAAACTTTTTGTAATAAACTAGGATTAGATACCCTATTATTAAAAATAAATATTAAAATGCTAAAGTAG

>Br48-2

AATTTTATCTACTTTATTATTAAAAAGAGGAGCAGCTCCTTTTCATTTTTGATTCCCAGGAGTTATAGAAGGATTAAATT

AATTAATCATATTGGTTGAATATTAATAGCTATAATAAATAACGAACTTTTATGATTAACTTATTTTTTATTATATTCAATT

CAACAAATCATAAAGATATTGGAACTTTATATTTCATTTTTGGAGTATGATCTGGAATAGTCGGAACTTCTCTAAGAATT

AATTGGATTATTAGGATTTATTGTTTGAGCTCATCATATATTTACAGTAGGTATAGACGTAGATACTCGAGCTTATTTTACTTCAGCAACTATAATT

CATGATACTTATTACGTAGTTGCCCATTTTCATTACGTTTTATCTATAGGAGCTGTATTTGCTATTATAGCAGGATTTATTCATTGATACCCTTTATTAACAGGAATAGTTATAAACCCTTCATG

CTGGAATACCTCGACGATACTCAGATTTTCCTGATAGCTACTTAACTTGAAATATTATTTCTTCTTTAGGAAGAACAATT

AATTTGTCACACACAACATATATTTGCTCATTTAGTTCCTCAAGGAACTCCTCCTGTTTTAATACCTTTTATAGTATGCATTGAAACTATTAGTAATGTAATCCGACCAGGAACTTTAGCAGTACGATTGACTGCTAATATAATT

GACGAGATATTTCTCGAGAAGGAACTTTTCAAGGACTTCACACTATTCCCGTAACATTAGGATTACGATGAGGAATAATTTTATTTATTATTTCTGAAGTTTTTTTCTTTATTTCCTTCTTTTGAGCTTTTTTCCATAGTAGCTTATCTCCAACAATCGAATT

AATTGTCATTTTTCTAGAAGTCATCATTTTGGATTCGAAGCAGCTGCTTGATATTGACATTTTGTTGATGTAGTTTGACTATTCTTATATATTTCAATT

AATTTTAGGAATATATTCTTATAAAATGTTTGATCAAGGATGAAGTGAATATTTTGGAGGTCAGATATTATATAATCAAT

ATTATTCAATAACAGGGGATTTTAATAGAACTACATTGAATATGTTAGATGATAAGGGGTGAACTATATCTTTTAGAATT

AATTCCTTTTTCTTCTTGATTACCTGCAGCTATGGCAGCACCGACTCCTGTTTCTGCTTTAGTACATTCTTCTACATTAG

AATTTATTTTCAAAATGTAAAATCTTATAATGCTGGTATATTAACAGCTTTATCTAATCGAATCGGGGATGTTGCTTTAT

CTTTACATTCAGTATCTATTGTAATAACTTTTTTATTTGATTGAATAAGTTTAATATTTATATCTTTTGTTTTATTAATT

AATTTTTATTAGATTTACTTTATTTATTTTAAGATTAAAGTTTTTATTAATAGATTTAGTATATTTTATTGAATGAGAAATT

GAGGATATCAACCTGAACGTTTACAGGCGGGGATTTATTTATTATTTTATACTTTACTGGCTTCGTTGCCGTTATTAATT

AATTATGAAAGTTACTTTAGTATATTTTTTTTAACTTTTTGTGTTTGTGAAGGGGTTCTAGGGCTGTCCATTTTAGTTTC

AATTATTATATTTATTTTTGGAAGTATTGTTTTTATTTCTAGTCGTAAGCATTTACTTTGTACTTTATTGAGATTAGAGT

AATTTTTATAGGAGGAATATTAGTTTTATTCATTTATGTAACTTCTCTTTCATCTAATGAAATATTTTCATTATCTATAAAACTATTTTTTTTATCTTTAAGTATAATT

AATTTTAACAGGATTATTCTTAGCAATACACTATACTGCTGATATTGAAACAGCTTTTAATAGAGTAAATCACATTTATCGTGATGTTAATAATGGTTGATTCCTACGAATT

CTGTTGATAATGCTACTTTAACTCGATTCTTTACATTTCATTTTATTCTCCCTTTCATTGTATTAGCTTTAACTATAATT

CTTTTGATTTTGCTGAAGGGGAGTCTGAGTTAGTTTCAGGATTTAATGTAGAATATAGAAGAGGGGGATTTGCTTTAATT

AATTTTTTTATATATCAGAAATATATATGATTTATTGTATTTTGTTTTCCTTTAGGTCTCGTTTGATTTGCATCTTGTTT

AATTTAAGCTATAAACTTTTTGTAATAAACTAGGATTAGATACCCTATTATTAAAAATAAATATTAAAATGCTAAAGTAG

>Br53-1

AATTTTATCTACTTTATTATTAAAAAGAGGGGCAGCTCCTTTTCATTTTTGATTCCCAGGAGTTATAGAAGGATTAAATT

AATTAATCATATTGGTTGAATATTAATAGCTATAATAAATAACGAACTTTTATGATTAACTTATTTTTTATTATATTCAATT

CAACAAATCATAAAGATATTGGAACTTTATATTTCATTTTTGGAGTATGATCTGGAATAGTCGGAACTTCTCTAAGAATT

AATTGGATTATTGGGATTTATTGTTTGAGCTCATCATATATTTACAGTAGGTATAGACGTAGATACTCGAGCTTATTTTACTTCAGCAACTATAATT

CATGATACTTATTACGTAGTTGCCCATTTTCATTATGTTTTATCTATAGGAGCTGTATTTGCTATTATAGCAGGATTTATTCATTGATACCCTTTATTAACAGGAATAGTTATAAACCCTTCATG

CTGGAATACCTCGACGATACTCAGATTTTCCTGATAGTTACTTAACTTGAAATATTATTTCTTCTTTAGGAAGAACAATT

AATTTGTCACACACAACATATATTTGCTCATTTAGTTCCTCAAGGAACTCCTCCTGTTTTAATACCTTTTATAGTATGCATTGAAACTATTAGTAATGTAATCCGACCAGGAACTTTAGCAGTACGATTGACTGCTAATATAATT

GACGAGATATTTCTCGAGAAGGAACTTTTCAAGGACTTCACACTATTCCCGTAACATTAGGATTACGATGAGGAATAATTTTATTTATTATTTCTGAAGTTTTTTTCTTTATTTCCTTCTTTTGAGCTTTTTTCCACAGTAGCTTATCTCCAACAATCGAATT

AATTGTCATTTTTCTAGAAGTCATCATTTTGGATTCGAAGCAGCTGCTTGATATTGACATTTTGTTGATGTAGTTTGACTATTCTTATATATTTCAATT

AATTTTAGGAATATATTCTTATAAAATGTTTGATCAAGGATGAAGTGAATATTTTGGAGGTCAGATATTATATAATCAAT

ATTATTCAATAACAGGAGATTTTAATAGAACTACATTGAATATATTAAATGATAAGGGGTGAACTATATCTTTTAGAATT

AATTCCTTTTTCTTCTTGATTACCTGCAGCTATGGCAGCGCCGACTCCTGTCTCTGCTTTAGTACATTCTTCTACATTAG

AATTTATTTTCAAAATGTAAAATCTTATAATGCTGGTATATTAACAGCTTTATCTAATCGAATCGGAGATGTTGCTTTAT

CTTTACATTCAATATCTATTGTAATAACTTTTTTATTTGATTGAATAAGTTTAATATTTATATCTTTTGTTTTATTAATT

AATTTTTATTAGATTTACTTTATTTATTTTAAGATTAAAGTTTTTATTAATAGATTTAGTATATTTTATTGAATGAGAAATT

GAGGGTATCAACCTGAACGTTTACAGGCGGGGATTTATTTATTATTTTATACTTTATTGGCTTCGTTGCCGTTATTAATT

AATTATGAAAGTTACTTTAGTATATTTTTTTTAACTTTTTGTGTTTGTGAAGGGGTTCTAGGGCTATCTATTTTAGTTTC

AATTATTATATTTATTTTTGGAAGTATTGTTTTTATTTCTAGTCGTAAGCATTTACTTTGTACTTTATTGAGATTAGAGT

AATTTTTATAGGAGGAATATTAGTTTTATTCATTTATGTAACTTCTCTTTCATCTAATGAAATATTTTCATTATCTATAAAACTATTTTTTTTATCTTTAAGTATAATT

AATTTTAACAGGATTATTTTTAGCAATACACTATACTGCTGATATTGAAACAGCTTTTAATAGAGTAAATCACATTTATCGTGATGTTAATAATGGTTGATTCCTACGAATT

CTGTTGATAATGCTACTTTAACTCGATTCTTTACATTTCATTTTATTCTACCTTTCATTGTATTAGCTTTAACTATAATT

CTTTTGATTTTGCTGAAGGGGAGTCTGAGTTAGTTTCAGGATTTAATGTAGAATACAGAAGAGGGGGATTTGCTTTAATT

AATTTTTTTATATATCAGAAATATATATGATTTATTGTATTTTGTTTTCCTTTAGGTCTCGTTTGATTTGCATCTTGTTT

AATTTAAGCTATAAACTTTTTGTAATAAACTAGGATTAGATACCCTATTATTAAAAATAAATATTAAAATGCTAAAGTAG

>Br57-1

NNNNNNNNNNNNNNNNNNNNNNNNNNNNNNNNNNNNNNNNNNNNNNNNNNNNNNNNNNNNNNNNNNNNNNNNNNNNNNNN

AATTAATCATATTGGTTGAATATTAATAGCTATAATAAATAACGAACTTTTATGATTAACTTATTTTTTATTATATTCAATT

CAACAAATCATAAAGATATTGGAACTTTATATTTCATTTTTGGAGTATGATCTGGAATAGTCGGAACTTCTCTAAGAATT

AATTGGATTATTGGGATTTATTGTTTGAGCTCATCATATATTTACAGTAGGTATAGATGTAGATACTCGAGCTTATTTTANNNNNNNNNNNNNNNNN

CATGATACTTATTACGTAGTTGCCCATTTTCATTACGTTTTATCTATAGGAGCTGTATTTGCTATTATAGCAGGATTTATTCATTGATACCCTTTATTAACAGGAATAGTTATAAACCCTTCATG

CTGGAATACCTCGACGATACTCAGATTTTCCTGATAGTTACTTAACTTGAAATATTATTTCTTCTTTAGGAAGAACAATT

AATTTGTCACACACAACATATATTTGCTCATTTAGTTCCTCAAGGGACTCCTCCTGTTTTAATACCTTTTATAGTATGCATTGAAACTATTAGTAATGTAATCCGACCAGGAACTTTAGCAGTACGATTGACTGCTAATATAATT

GACGAGATATTTCTCGAGAAGGAACTTTTCAAGGACTTCACACTATTCCCGTAACATTAGGATTACGATGAGGAATAATTTTATTTATTATTTCTGAAGTTTTTTTCTTTATTTCCTTCTTTTGAGCTTTTTTCCATAGTAGCTTATCTCCAACAATCGAATT

AATTGTCATTTTTCTAGAAGTCATCATTTTGGATTCGAAGCAGCTGCTTGATATTGACATTTTGTTGATGTATTTTGACTATTCTTATATATTTCAATT

AATTTTAGGAATATATTCTTATAAAATGTTTGATCAAGGATGAAGTGAATATTTTGGAGGTCAGATATTATATAATCAAT

ATTATTCAATAACAGGGGATTTTAATAGAACTACATTGAATATGTTAAATGATAAGGGGTGAGCTATATCTTTTAGAATT

AATTCCTTTTTCTTCTTGATTACCTGCAGCTATGGCAGCACCGACTCCTGTTTCTGCTTTAGTACATTCTTCTACATTAG

AATTTATTTTCAAAATGTAAAATCTTATAATGCTGGTATATTAACAGCTTTATCTAATCGAATCGGAGATGTTGCCTTAT

CTTTACATTCAATATCTATTGTAATAACTTTTTTATTTGATTGAATAAGTTTAATATTTATATCTTTTGTTTTATTAATT

AATTTTTATTAGATTTACTTTATTTATTTTAAGATTAAAGTTTTTATTAATAGATTTAGTATATTTTATTGAATGAGAAATT

GAGGATATCAACCTGAACGTTTACAGGCGGGGATTTATTTATTATTTTATACTTTACTGGCTTCGTTGCCGTTATTAATT

AATTATGAAAGTTACTTTAGTATATTTTTTTTAACTTTTTGTGTTTGTGAAGGGGTTCTAGGGCTATCTATTTTAGTTTC

AATTATTATATTTATTTTTGGAAGTATTGTTTTTATTTCTAGTCGTAAGCATTTACTTTGTACTTTATTGAGATTAGAGT

AATTTTTATAGGAGGAATATTAGTTTTATTCATTTATGTAACTTCTCTTTCATCTAATGAAATATTTTCATTATCTATAAAACTATTTTTTTTATCTTTAAGTATAATT

AATTTTAACAGGATTATTCTTAGCAATACACTATACTGCTGATATTGAAACAGCTTTTAATAGAGTAAATCACATTTATCGTGATGTTAATAATGGTTGATTCCTACGAATT

CTGTTGATAATGCTACTTTAACTCGATTCTTTACCTTTCATTTTATTCTCCCTTTCATTGTATTAGCTTTAACTATAATT

CTTTTGATTTTGCTGAAGGGGAGTCTGAGTTAGTTTCAGGATTTAATGTAGAGTATAGAAGAGGGGGATTTGCTTTAATT

AATTTTTTTATATATCAGAAATATATATGATTTATTGTATTTTGTTTTCCTTTAGGTCTCGTTTGATTTGCATCTTGTTT

AATTTAAGCTATAAACTTTTTGTAATAAACTAGGATTAGATACCCTATTATTAAAAATAAATATTAAAATGCTAAAGTAG

>Br57-2

NNNNNNNNNNNNNNNNNNNNNNNNNNNNNNNNNNNNNNNNNNNNNNNNNNNNNNNNNNNNNNNNNNNNNNNNNNNNNNNN

AATTAATCATATTGGTTGAATATTAATAGCTATAATAAATAACGAACTTTTATGATTAACTTATTTTTTATTATATTCAATT

CAACAAATCATAAAGATATTGGAACTTTATATTTCATTTTTGGAGTATGATCTGGAATAGTCGGAACTTCTCTAAGAATT

AATTGGATTATTGGGATTTATTGTTTGAGCTCATCATATATTTACAGTAGGTATAGATGTAGATACTCGAGCTTATTTTACTTCAGCAACTATAATT

CATGATACTTATTACGTAGTTGCCCATTTTCATTACGTTTTATCTATAGGAGCTGTATTTGCTATTATAGCAGGATTTATTCATTGATACCCTTTATTAACAGGAATAGTTATAAACCCTTCATG

CTGGAATACCTCGACGATACTCAGATTTTCCTGATAGTTACTTAACTTGAAATATTATTTCTTCTTTAGGAAGAACAATT

AATTTGTCACACACAACATATATTTGCTCATTTAGTTCCTCAAGGGACTCCTCCTGTTTTAATACCTTTTATAGTATGCATTGAAACTATTAGTAATGTAATCCGACCAGGAACTTTAGCAGTACGATTGACTGCTAATATAATT

GACGAGATATTTCTCGAGAAGGAACTTTTCAAGGACTTCACACTATTCCCGTAACATTAGGATTACGATGAGGAATAATTTTATTTATTATTTCTGAAGTTTTTTTCTTTATTTCCTTCTTTTGAGCTTTTTTCCATAGTAGCTTATCTCCAACAATCGAATT

AATTGTCATTTTTCTAGAAGTCATCATTTTGGATTCGAAGCAGCTGCTTGATATTGACATTTTGTTGATGTAGTTTGACTATTCTTATATATTTCAATT

AATTTTAGGAATATATTCTTATAAAATGTTTGATCAAGGATGAAGTGAATATTTTGGAGGTCAGATATTATATAATCAAT

ATTATTCAATAACAGGGGATTTTAATAGAACTACATTGAATATGTTAAATGATAAGGGGTGAGCTATATCTTTTAGAATT

AATTCCTTTTTCTTCTTGATTACCTGCAGCTATGGCAGCACCGACTCCTGTTTCTGCTTTAGTACATTCTTCTACATTAG

AATTTATTTTCAAAATGTAAAATCTTATAATGCTGGTATATTAACAGCTTTATCTAATCGAATCGGAGATGTTGCCTTAT

CTTTACATTCAATATCTATTGTAATAACTTTTTTATTTGATTGAATAAGTTTAATATTTATATCTTTTGTTTTATTAATT

AATTTTTATTAGATTTACTTTATTTATTTTAAGATTAAAGTTTTTATTAATAGATTTAGTATATTTTATTGAATGAGAAATT

GAGGATATCAACCTGAACGTTTACAGGCGGGGATTTATTTATTATTTTATACTTTACTGGCTTCGTTGCCGTTATTAATT

AATTATGAAAGTTACTTTAGTATATTTTTTTTAACTTTTTGTGTTTGTGAAGGGGTTCTAGGGCTATCTATTTTAGTTTC

AATTATTATATTTATTTTTGGAAGTATTGTTTTTATTTCTAGTCGTAAGCATTTACTTTGTACTTTATTGAGATTAGAGT

AATTTTTATAGGAGGAATATTAGTTTTATTCATTTATGTAACTTCTCTTTCATCTAATGAAATATTTTCATTATCTATAAAACTATTTTTTTTATCTTTAAGTATAATT

AATTTTAACAGGATTATTCTTAGCAATACACTATACTGCTGATATTGAAACAGCTTTTAATAGAGTAAATCACATTTATCGTGATGTTAATAATGGTTGATTCCTACGAATT

CTGTTGATAATGCTACTTTAACTCGATTCTTTACCTTTCATTTTATTCTCCCTTTCATTGTATTAGCTTTAACTATAATT

CTTTTGATTTTGCTGAAGGGGAGTCTGAGTTAGTTTCAGGATTTAATGTAGAGTATAGAAGAGGGGGATTTGCTTTAATT

AATTTTTTTATATATCAGAAATATATATGATTTATTGTATTTTGTTTTCCTTTAGGTCTCGTTTGATTTGCATCTTGTTT

AATTTAAGCTATAAACTTTTTGTAATAAACTAGGATTAGATACCCTATTATTAAAAATAAATATTAAAATGCTAAAGTAG

>BrK01-1

AATTTTATCTACTTTATTATTAAAAAGAGGAGCAGCTCCTTTTCATTTTTGATTCCCAGGAGTTATAGAAGGATTAAATT

AATTAATCATATTGGTTGAATATTAATAGCTATAATAAATAACGAACTTTTATGATTAACTTATTTTTTATTATATTCAATT

CAACAAATCATAAAGATATTGGAACTTTATATTTCATTTTTGGAGTATGATCTGGAATAGTCGGAACTTCTCTAAGAATT

AATTGGATTATTAGGATTTATTGTTTGAGCTCATCATATATTTACAGTAGGTATAGACGTAGATACTCGAGCTTATTTTACTTCAGCAACTATAATT

CATGATACTTATTACGTAGTTGCCCATTTTCATTACGTTTTATCTATAGGAGCTGTATTTGCTATTATAGCAGGATTTATTCATTGATACCCTTTATTAACAGGAATAGTTATAAACCCTTCATG

CTGGAATACCTCGACGATACTCAGATTTTCCTGATAGCTACTTAACTTGAAATATTATTTCTTCTTTAGGAAGAACAATT

AATTTGTCACACACAACATATATTTGCTCATTTAGTTCCTCAAGGAACTCCTCCTGTTTTAATACCTTTTATAGTATGCATTGAAACTATTAGTAATGTAATCCGACCAGGAACTTTAGCAGTACGATTGACTGCTAATATAATT

GACGAGATATTTCTCGAGAAGGAACTTTTCAAGGACTTCACACTATTCCCGTAACATTAGGATTACGATGAGGAATAATTTTATTTATTATTTCTGAAGTTTTTTTCTTTATTTCCTTCTTTTGAGCTTTTTTCCATAGTAGCTTATCTCCAACAATCGAATT

AATTGTCATTTTTCTAGAAGTCATCATTTTGGATTCGAAGCAGCTGCTTGATATTGACATTTTGTTGATGTAGTTTGACTATTCTTATATATTTCAATT

AATTTTAGGAATATATTCTTATAAAATGTTTGATCAAGGATGAAGTGAATATTTTGGAGGTCAGATATTATATAATCAAT

ATTATTCAATAACAGGGGATTTTAATAGAACTACATTGAATATGTTAGATGATAAGGGGTGAACTATATCTTTTAGAATT

AATTCCTTTTTCTTCTTGATTACCTGCAGCTATGGCAGCACCGACTCCTGTTTCTGCTTTAGTACATTCTTCTACATTAG

AATTTATTTTCAAAATGTAAAATCTTATAATGCTGGTATATTAACAGCTTTATCTAATCGAATCGGGGATGTTGCTTTAT

CTTTACATTCAGTATCTATTGTAATAACTTTTTTATTTGATTGAATAAGTTTAATATTTATATCTTTTGTTTTATTAATT

AATTTTTATTAGATTTACTTTATTTATTTTAAGATTAAAGTTTTTATTAATAGATTTAGTATATTTTATTGAATGAGAAATT

GAGGATATCAACCTGAACGTTTACAGGCGGGGATTTATTTATTATTTTATACTTTACTGGCTTCGTTGCCGTTATTAATT

AATTATGAAAGTTACTTTAGTATATTTTTTTTAACTTTTTGTGTTTGTGAAGGGGTTCTAGGGCTGTCCATTTTAGTTTC

AATTATTATATTTATTTTTGGAAGTATTGTTTTTATTTCTAGTCGTAAGCATTTACTTTGTACTTTATTGAGATTAGAGT

AATTTTTATAGGAGGAATATTAGTTTTATTCATTTATGTAACTTCTCTTTCATCTAATGAAATATTTTCATTATCTATAAAACTATTTTTTTTATCTTTAAGTATAATT

AATTTTAACAGGATTATTCTTAGCAATACACTATACTGCTGATATTGAAACAGCTTTTAATAGAGTAAATCACATTTATCGTGATGTTAATAATGGTTGATTCCTACGAATT

CTGTTGATAATGCTACTTTAACTCGATTCTTTACATTTCATTTTATTCTCCCTTTCATTGTATTAGCTTTAACTATAATT

CTTTTGATTTTGCTGAAGGGGAGTCTGAGTTAGTTTCAGGATTTAATGTAGAATATAGAAGAGGGGGATTTGCTTTAATT

AATTTTTTTATATATCAGAAATATATATGATTTATTGTATTTTGTTTTCCTTTAGGTCTCGTTTGATTTGCATCTTGTTT

AATTTAAGCTATAAACTTTTTGTAATAAACTAGGATTAGATACCCTATTATTAAAAATAAATATTAAAATGCTAAAGTAG

>BrK02-1

AATTTTATCTACTTTATTATTAAAAAGAGGAGCAGCTCCTTTTCATTTTTGATTCCCAGGAGTTATAGAAGGATTAAATT

AATTAATCATATTGGTTGAATATTAATAGCTATAATAAATAACGAACTTTTATGATTAACTTATTTTTTATTATATTCAATT

CAACAAATCATAAAGATATTGGAACTTTATATTTCATTTTTGGAGTATGATCTGGAATAGTCGGAACTTCTCTAAGAATT

AATTGGATTATTAGGATTTATTGTTTGAGCTCATCATATATTTACAGTAGGTATAGACGTAGATACTCGAGCTTATTTTACTTCAGCAACTATAATN

CATGATACTTATTACGTAGTTGCCCATTTTCATTACGTTTTATCTATAGGAGCTGTATTTGCTATTATAGCAGGATTTATTCATTGATACCCTTTATTAACAGGAATAGTTATAAACCCTTCATG

CTGGAATACCTCGACGATACTCAGATTTTCCTGATAGCTACTTAACTTGAAATATTATTTCTTCTTTAGGAAGAACAATT

AATTTGTCACACACAACATATATTTGCTCATTTAGTTCCTCAAGGAACTCCTCCTGTTTTAATACCTTTTATAGTATGCATTGAAACTATTAGTAATGTAATCCGACCAGGAACTTTAGCAGTACGATTGACTGCTAATATAATT

GACGAGATATTTCTCGAGAAGGAACTTTTCAAGGACTTCACACTATTCCCGTAACATTAGGATTACGATGAGGAATAATTTTATTTATTATTTCTGAAGTTTTTTTCTTTATTTCCTTCTTTTGAGCTTTTTTCCATAGTAGCTTATCTCCAACAATCGAATT

AATTGTCATTTTTCTAGAAGTCATCATTTTGGATTCGAAGCAGCTGCTTGATATTGACATTTTGTTGATGTAGTTTGACTNNNNNNNNNNNNNNNNNNN

NNNNNNNNNNNNNNNNNNNNNNNNNNNNNNNNNNNNNNNNNNNNNNNNNNNNNNNNNNNNNNNNNNNNNNNNNNNNNNNN

ATTATTCAATAACAGGGGATTTTAATAGAACTACATTGAATATGTTAGATGATAAGGGGTGAACTATATCTTTTAGAATT

AATTCCTTTTTCTTCTTGATTACCTGCAGCTATGGCAGCACCGACTCCTGTTTCTGCTTTAGTACATTCTTCTACATTAG

AATTTATTTTCAAAATGTAAAATCTTATAATGCTGGTATATTAACAGCTTTATCTAATCGAATCGGGGATGTTGCTTTAT

NNNNNNNNNNNNNNNNNNNNNNNNNNNNNNNNNNNNNNNNNNNNNNNNNNNNNNNNNNNNNNNNNNNNNNNNNNNNNNNN

AATTTTTATTAGATTTACTTTATTTATTTTAAGATTAAAGTTTTTATTAATAGATTTAGTATATTTTATTGAATGAGAAATT

GAGGATATCAACCTGAACGTTTACAGGCGGGGATTTATTTATTATTTTATACTTTACTGGCTTCGTTGCCGTTATTAATT

AATTATGAAAGTTACTTTAGTATATTTTTTTTAACTTTTTGTGTTTGTGAAGGGGTTCTAGGGCTGTCCATTTTAGTTTC

AATTATTATATTTATTTTTGGAAGTATTGTTTTTATTTCTAGTCGTAAGCATTTACTTTGTACTTTATTGAGATTAGAGT

AATTTTTATAGGAGGAATATTAGTTTTATTCATTTATGTAACTTCTCTTTCATCTAATGAAATATTTTCATTATCTATAAAACTATTTTTTTTATCTTTAAGTATAATT

AATTTTAACAGGATTATTCTTAGCAATACACTATACTGCTGATATTGAAACAGCTTTTAATAGAGTAAATCACATTTATCGTGATGTTAATAATGGTTGATTCCTACGAATT

CTGTTGATAATGCTACTTTAACTCGATTCTTTACATTTCATTTTATTCTCCCTTTCATTGTATTAGCTTTAACTATAATT

CTTTTGATTTTGCTGAAGGGGAGTCTGAGTTAGTTTCAGGATTTAATGTAGAATATAGAAGAGGGGGATTTGCTTTAATT

AATTTTTTTATATATCAGAAATATATATGATTTATTGTATTTTGTTTTCCTTTAGGTCTCGTTTGATTTGCATCTTGTTT

AATTTAAGCTATAAACTTTTTGTAATAAACTAGGATTAGATACCCTATTATTAAAAATAAATATTAAAATGCTAAAGTAG

>BrK02-2

AATTTTATCTACTTTATTATTAAAAAGAGGAGCAGCTCCTTTTCATTTTTGATTCCCAGGAGTTATAGAAGGATTAAATT

AATTAATCATATTGGTTGAATATTAATAGCTATAATAAATAACGAACTTTTATGATTAACTTATTTTTTATTATATTCAATT

CAACAAATCATAAAGATATTGGAACTTTATATTTCATTTTTGGAGTATGATCTGGAATAGTCGGAACTTCTCTAAGAATT

AATTGGATTATTAGGATTTATTGTTTGAGCTCATCATATATTTACAGTAGGTATAGACGTAGATACTCGAGCTTATTTTACTTCAGCAACTATAATT

CATGATACTTATTACGTAGTTGCCCATTTTCATTACGTTTTATCTATAGGAGCTGTATTTGCTATTATAGCAGGATTTATTCATTGATACCCTTTATTAACAGGAATAGTTATAAACCCTTCATG

CTGGAATACCTCGACGATACTCAGATTTTCCTGATAGCTACTTAACTTGAAATATTATTTCTTCTTTAGGAAGAACAATT

AATTTGTCACACACAACATATATTTGCTCATTTAGTTCCTCAAGGAACTCCTCCTGTTTTAATACCTTTTATAGTATGCATTGAAACTATTAGTAATGTAATCCGACCAGGAACTTTAGCAGTACGATTGACTGCTAATATAATT

GACGAGATATTTCTCGAGAAGGAACTTTTCAAGGACTTCACACTATTCCCGTAACATTAGGATTACGATGAGGAATAATTTTATTTATTATTTCTGAAGTTTTTTTCTTTATTTCCTTCTTTTGAGCTTTTTTCCATAGTAGCTTATCTCCAACAATCGAATT

AATTGTCATTTTTCTAGAAGTCATCATTTTGGATTCGAAGCAGCTGCTTGATATTGACATTTTGTTGATGTAGTTTGACTATTCTTATATATTTCAATT

AATTTTAGGAATATATTCTTATAAAATGTTTGATCAAGGATGAAGTGAATATTTTGGAGGTCAGATATTATATAATCAAT

ATTATTCAATAACAGGGGATTTTAATAGAACTACATTGAATATGTTAGATGATAAGGGGTGAACTATATCTTTTAGAATT

AATTCCTTTTTCTTCTTGATTACCTGCAGCTATGGCAGCACCGACTCCTGTTTCTGCTTTAGTACATTCTTCTACATTAG

AATTTATTTTCAAAATGTAAAATCTTATAATGCTGGTATATTAACAGCTTTATCTAATCGAATCGGGGATGTTGCTTTAT

CTTTACATTCAGTATCTATTGTAATAACTTTTTTATTTGATTGAATAAGTTTAATATTTATATCTTTTGTTTTATTAATT

AATTTTTATTAGATTTACTTTATTTATTTTAAGATTAAAGTTTTTATTAATAGATTTAGTATATTTTATTGAATGAGAAATT

GAGGATATCAACCTGAACGTTTACAGGCGGGGATTTATTTATTATTTTATACTTTACTGGCTTCGTTGCCGTTATTAATT

AATTATGAAAGTTACTTTAGTATATTTTTTTTAACTTTTTGTGTTTGTGAAGGGGTTCTAGGGCTGTCCATTTTAGTTTC

AATTATTATATTTATTTTTGGAAGTATTGTTTTTATTTCTAGTCGTAAGCATTTACTTTGTACTTTATTGAGATTAGAGT

AATTTTTATAGGAGGAATATTAGTTTTATTCATTTATGTAACTTCTCTTTCATCTAATGAAATATTTTCATTATCTATAAAACTATTTTTTTTATCTTTAAGTATAATT

AATTTTAACAGGATTATTCTTAGCAATACACTATACTGCTGATATTGAAACAGCTTTTAATAGAGTAAATCACATTTATCGTGATGTTAATAATGGTTGATTCCTACGAATT

CTGTTGATAATGCTACTTTAACTCGATTCTTTACATTTCATTTTATTCTCCCTTTCATTGTATTAGCTTTAACTATAATT

CTTTTGATTTTGCTGAAGGGGAGTCTGAGTTAGTTTCAGGATTTAATGTAGAATATAGAAGAGGGGGATTTGCTTTAATT

AATTTTTTTATATATCAGAAATATATATGATTTATTGTATTTTGTTTTCCTTTAGGTCTCGTTTGATTTGCATCTTGTTT

AATTTAAGCTATAAACTTTTTGTAATAAACTAGGATTAGATACCCTATTATTAAAAATAAATATTAAAATGCTAAAGTAG

>BrK04-1

AATTTTATCTACTTTATTATTAAAAAGAGGAGCAGCTCCTTTTCATTTTTGATTCCCAGGAGTTATAGAAGGATTAAATT

AATTAATCATATTGGTTGAATATTAATAGCTATAATAAATAACGAACTTTTATGATTAACTTATTTTTTATTATATTCAATT

CAACAAATCATAAAGATATTGGAACTTTATATTTCATTTTTGGAGTATGATCTGGAATAGTCGGAACTTCTCTAAGAATT

AATTGGATTATTAGGATTTATTGTTTGAGCTCATCATATATTTACAGTAGGTATAGACGTAGATACTCGAGCTTATTTTACTTCAGCAACTATAATT

CATGATACTTATTACGTAGTTGCCCATTTTCATTACGTTTTATCTATAGGAGCTGTATTTGCTATTATAGCAGGATTTATTCATTGATACCCTTTATTAACAGGAATAGTTATAAACCCTTCATG

CTGGAATACCTCGACGATACTCAGATTTTCCTGATAGCTACTTAACTTGAAATATTATTTCTTCTTTAGGAAGAACAATT

AATTTGTCACACACAACATATATTTGCTCATTTAGTTCCTCAAGGAACTCCTCCTGTTTTAATACCTTTTATAGTATGCATTGAAACTATTAGTAATGTAATCCGACCAGGAACTTTAGCAGTACGATTGACTGCTAATATAATT

GACGAGATATTTCTCGAGAAGGAACTTTTCAAGGACTTCACACTATTCCCGTAACATTAGGATTACGATGAGGAATAATTTTATTTATTATTTCTGAAGTTTTTTTCTTTATTTCCTTCTTTTGAGCTTTTTTCCATAGTAGCTTATCTCCAACAATCGAATT

AATTGTCATTTTTCTAGAAGTCATCATTTTGGATTCGAAGCAGCTGCTTGATATTGACATTTTGTTGATGTAGTTTGACTATTCTTATATATTTCAATT

NATTTTAGGAATATATTCTTATAAAATGTTTGATCAAGGATGAAGTGAATATTTTGGAGGTCAGATATTATATAATCAAT

ATTATTCAATAACAGGGGATTTTAATAGAACTACATTGAATATGTTAGATGATAAGGGGTGAACTATATCTTTTAGAATT

AATTCCTTTTTCTTCTTGATTACCTGCAGCTATGGCAGCACCGACTCCTGTTTCTGCTTTAGTACATTCTTCTACATTAG

AATTTATTTTCAAAATGTAAAATCTTATAATGCTGGTATATTAACAGCTTTATCTAATCGAATCGGGGATGTTGCTTTAT

CTTTACATTCAGTATCTATTGTAATAACTTTTTTATTTGATTGAATAAGTTTAATATTTATATCTTTTGTTTTATTAATT

AATTTTTATTAGATTTACTTTATTTATTTTAAGATTAAAGTTTTTATTAATAGATTTAGTATATTTTATTGAATGAGAAATT

GAGGATATCAACCTGAACGTTTACAGGCGGGGATTTATTTATTATTTTATACTTTACTGGCTTCGTTGCCGTTATTAATT

AATTATGAAAGTTACTTTAGTATATTTTTTTTAACTTTTTGTGTTTGTGAAGGGGTTCTAGGGCTGTCCATTTTAGTTTC

AATTATTATATTTATTTTTGGAAGTATTGTTTTTATTTCTAGTCGTAAGCATTTACTTTGTACTTTATTGAGATTAGAGT

AATTTTTATAGGAGGAATATTAGTTTTATTCATTTATGTAACTTCTCTTTCATCTAATGAAATATTTTCATTATCTATAAAACTATTTTTTTTATCTTTAAGTATAATT

AATTTTAACAGGATTATTCTTAGCAATACACTATACTGCTGATATTGAAACAGCTTTTAATAGAGTAAATCACATTTATCGTGATGTTAATAATGGTTGATTCCTACGAATT

CTGTTGATAATGCTACTTTAACTCGATTCTTTACATTTCATTTTATTCTCCCTTTCATTGTATTAGCTTTAACTATAATT

CTTTTGATTTTGCTGAAGGGGAGTCTGAGTTAGTTTCAGGATTTAATGTAGAATATAGAAGAGGGGGATTTGCTTTAATT

AATTTTTTTATATATCAGAAATATATATGATTTATTGTATTTTGTTTTCCTTTAGGTCTCGTTTGATTTGCATCTTGTTT

AATTTAAGCTATAAACTTTTTGTAATAAACTAGGATTAGATACCCTATTATTAAAAATAAATATTAAAATGCTAAAGTAG

>BrK04-2

AATTTTATCTACTTTATTATTAAAAAGAGGAGCAGCTCCTTTTCATTTTTGATTCCCAGGAGTTATAGAAGGATTAAATT

AATTAATCATATTGGTTGAATATTAATAGCTATAATAAATAACGAACTTTTATGATTAACTTATTTTTTATTATATTCAATT

CAACAAATCATAAAGATATTGGAACTTTATATTTCATTTTTGGAGTATGATCTGGAATAGTCGGAACTTCTCTAAGAATT

AATTGGATTATTAGGATTTATTGTTTGAGCTCATCATATATTTACAGTAGGTATAGACGTAGATACTCGAGCTTATTTTACTTCAGCAACTATAATT

CATGATACTTATTACGTAGTTGCCCATTTTCATTACGTTTTATCTATAGGAGCTGTATTTGCTATTATAGCAGGATTTATTCATTGATACCCTTTATTAACAGGAATAGTTATAAACCCTTCATG

CTGGAATACCTCGACGATACTCAGATTTTCCTGATAGCTACTTAACTTGAAATATTATTTCTTCTTTAGGAAGAACAATT

AATTTGTCACACACAACATATATTTGCTCATTTAGTTCCTCAAGGAACTCCTCCTGTTTTAATACCTTTTATAGTATGCATTGAAACTATTAGTAATGTAATCCGACCAGGAACTTTAGCAGTACGATTGACTGCTAATATAATT

GACGAGATATTTCTCGAGAAGGAACTTTTCAAGGACTTCACACTATTCCCGTAACATTAGGATTACGATGAGGAATAATTTTATTTATTATTTCTGAAGTTTTTTTCTTTATTTCCTTCTTTTGAGCTTTTTTCCATAGTAGCTTATCTCCAACAATCGAATT

AATTGTCATTTTTCTAGAAGTCATCATTTTGGATTCGAAGCAGCTGCTTGATATTGACATTTTGTTGATGTAGTTTGACTATTCTTATATATTTCAATT

AATTTTAGGAATATATTCTTATAAAATGTTTGATCAAGGATGAAGTGAATATTTTGGAGGTCAGATATTATATAATCAAT

ATTATTCAATAACAGGGGATTTTAATAGAACTACATTGAATATGTTAGATGATAAGGGGTGAACTATATCTTTTAGAATT

AATTCCTTTTTCTTCTTGATTACCTGCAGCTATGGCAGCACCGACTCCTGTTTCTGCTTTAGTACATTCTTCTACATTAG

AATTTATTTTCAAAATGTAAAATCTTATAATGCTGGTATATTAACAGCTTTATCTAATCGAATCGGGGATGTTGCTTTAT

CTTTACATTCAGTATCTATTGTAATAACTTTTTTATTTGATTGAATAAGTTTAATATTTATATCTTTTGTTTTATTAATT

AATTTTTATTAGATTTACTTTATTTATTTTAAGATTAAAGTTTTTATTAATAGATTTAGTATATTTTATTGAATGAGAAATT

GAGGATATCAACCTGAACGTTTACAGGCGGGGATTTATTTATTATTTTATACTTTACTGGCTTCGTTGCCGTTATTAATT

AATTATGAAAGTTACTTTAGTATATTTTTTTTAACTTTTTGTGTTTGTGAAGGGGTTCTAGGGCTGTCCATTTTAGTTTC

AATTATTATATTTATTTTTGGAAGTATTGTTTTTATTTCTAGTCGTAAGCATTTACTTTGTACTTTATTGAGATTAGAGT

AATTTTTATAGGAGGAATATTAGTTTTATTCATTTATGTAACTTCTCTTTCATCTAATGAAATATTTTCATTATCTATAAAACTATTTTTTTTATCTTTAAGTATAATT

AATTTTAACAGGATTATTCTTAGCAATACACTATACTGCTGATATTGAAACAGCTTTTAATAGAGTAAATCACATTTATCGTGATGTTAATAATGGTTGATTCCTACGAATT

CTGTTGATAATGCTACTTTAACTCGATTCTTTACATTTCATTTTATTCTCCCTTTCATTGTATTAGCTTTAACTATAATT

CTTTTGATTTTGCTGAAGGGGAGTCTGAGTTAGTTTCAGGATTTAATGTAGAATATAGAAGAGGGGGATTTGCTTTAATT

AATTTTTTTATATATCAGAAATATATATGATTTATTGTATTTTGTTTTCCTTTAGGTCTCGTTTGATTTGCATCTTGTTT

AATTTAAGCTATAAACTTTTTGTAATAAACTAGGATTAGATACCCTATTATTAAAAATAAATATTAAAATGCTAAAGTAG

>BrK05-1

AATTTTATCTACTTTATTATTAAAAAGAGGAGCAGCTCCTTTTCATTTTTGATTCCCAGGAGTTATAGAAGGATTAAATT

AATTAATCATATTGGTTGAATATTAATAGCTATAATAAATAACGAACTTTTATGATTAACTTATTTTTTATTATATTCAATT

CAACAAATCATAAAGATATTGGAACTTTATATTTCATTTTTGGAGTATGATCTGGAATAGTCGGAACTTCTCTAAGAATT

AATTGGATTATTAGGATTTATTGTTTGAGCTCATCATATATTTACAGTAGGTATAGACGTAGATACTCGAGCTTATTTTACTTCAGCAACTATAATT

CATGATACTTATTACGTAGTTGCCCATTTTCATTACGTTTTATCTATAGGAGCTGTATTTGCTATTATAGCAGGATTTATTCATTGATACCCTTTATTAACAGGAATAGTTATAAACCCTTCATG

CTGGAATACCTCGACGATACTCAGATTTTCCTGATAGCTACTTAACTTGAAATATTATTTCTTCTTTAGGAAGAACAATT

AATTTGTCACACACAACATATATTTGCTCATTTAGTTCCTCAAGGAACTCCTCCTGTTTTAATACCTTTTATAGTATGCATTGAAACTATTAGTAATGTAATCCGACCAGGAACTTTAGCAGTACGATTGACTGCTAATATAATT

GACGAGATATTTCTCGAGAAGGAACTTTTCAAGGACTTCACACTATTCCCGTAACATTAGGATTACGATGAGGAATAATTTTATTTATTATTTCTGAAGTTTTTTTCTTTATTTCCTTCTTTTGAGCTTTTTTCCATAGTAGCTTATCTCCAACAATCGAATT

AATTGTCATTTTTCTAGAAGTCATCATTTTGGATTCGAAGCAGCTGCTTGATATTGACATTTTGTTGATGTAGTTTGACTATTCTTATATATTTCAATT

AATTTTAGGAATATATTCTTATAAAATGTTTGATCAAGGATGAAGTGAATATTTTGGAGGTCAGATATTATATAATCAAT

ATTATTCAATAACAGGGGATTTTAATAGAACTACATTGAATATGTTAGATGATAAGGGGTGAACTATATCTTTTAGAATT

AATTCCTTTTTCTTCTTGATTACCTGCAGCTATGGCAGCACCGACTCCTGTTTCTGCTTTAGTACATTCTTCTACATTAG

AATTTATTTTCAAAATGTAAAATCTTATAATGCTGGTATATTAACAGCTTTATCTAATCGAATCGGGGATGTTGCTTTAT

CTTTACATTCAGTATCTATTGTAATAACTTTTTTATTTGATTGAATAAGTTTAATATTTATATCTTTTGTTTTATTAATT

AATTTTTATTAGATTTACTTTATTTATTTTAAGATTAAAGTTTTTATTAATAGATTTAGTATATTTTATTGAATGAGAAATT

GAGGATATCAACCTGAACGTTTACAGGCGGGGATTTATTTATTATTTTATACTTTACTGGCTTCGTTGCCGTTATTAATT

AATTATGAAAGTTACTTTAGTATATTTTTTTTAACTTTTTGTGTTTGTGAAGGGGTTCTAGGGCTGTCCATTTTAGTTTC

AATTATTATATTTATTTTTGGAAGTATTGTTTTTATTTCTAGTCGTAAGCATTTACTTTGTACTTTATTGAGATTAGAGT

AATTTTTATAGGAGGAATATTAGTTTTATTCATTTATGTAACTTCTCTTTCATCTAATGAAATATTTTCATTATCTATAAAACTATTTTTTTTATCTTTAAGTATAATT

AATTTTAACAGGATTATTCTTAGCAATACACTATACTGCTGATATTGAAACAGCTTTTAATAGAGTAAATCACATTTATCGTGATGTTAATAATGGTTGATTCCTACGAATT

CTGTTGATAATGCTACTTTAACTCGATTCTTTACATTTCATTTTATTCTCCCTTTCATTGTATTAGCTTTAACTATAATT

CTTTTGATTTTGCTGAAGGGGAGTCTGAGTTAGTTTCAGGATTTAATGTAGAATATAGAAGAGGGGGATTTGCTTTAATT

AATTTTTTTATATATCAGAAATATATATGATTTATTGTATTTTGTTTTCCTTTAGGTCTCGTTTGATTTGCATCTTGTTT

AATTTAAGCTATAAACTTTTTGTAATAAACTAGGATTAGATACCCTATTATTAAAAATAAATATTAAAATGCTAAAGTAG

>BrK05-2

AATTTTATCTACTTTATTATTAAAAAGAGGAGCAGCTCCTTTTCATTTTTGATTCCCAGGAGTTATAGAAGGATTAAATT

AATTAATCATATTGGTTGAATATTAATAGCTATAATAAATAACGAACTTTTATGATTAACTTATTTTTTATTATATTCAATT

CAACAAATCATAAAGATATTGGAACTTTATATTTCATTTTTGGAGTATGATCTGGAATAGTCGGAACTTCTCTAAGAATT

AATTGGATTATTAGGATTTATTGTTTGAGCTCATCATATATTTACAGTAGGTATAGACGTAGATACTCGAGCTTATTTTACTTCAGCAACTATAATT

CATGATACTTATTACGTAGTTGCCCATTTTCATTACGTTTTATCTATAGGAGCTGTATTTGCTATTATAGCAGGATTTATTCATTGATACCCTTTATTAACAGGAATAGTTATAAACCCTTCATG

CTGGAATACCTCGACGATACTCAGATTTTCCTGATAGCTACTTAACTTGAAATATTATTTCTTCTTTAGGAAGAACAATT

AATTTGTCACACACAACATATATTTGCTCATTTAGTTCCTCAAGGAACTCCTCCTGTTTTAATACCTTTTATAGTATGCATTGAAACTATTAGTAATGTAATCCGACCAGGAACTTTAGCAGTACGATTGACTGCTAATATAATT

GACGAGATATTTCTCGAGAAGGAACTTTTCAAGGACTTCACACTATTCCCGTAACATTAGGATTACGATGAGGAATAATTTTATTTATTATTTCTGAAGTTTTTTTCTTTATTTCCTTCTTTTGAGCTTTTTTCCATAGTAGCTTATCTCCAACAATCGAATT

AATTGTCATTTTTCTAGAAGTCATCATTTTGGATTCGAAGCAGCTGCTTGATATTGACATTTTGTTGATGTAGTTTGACTATTCTTATATATTTCAATT

AATTTTAGGAATATATTCTTATAAAATGTTTGATCAAGGATGAAGTGAATATTTTGGAGGTCAGATATTATATAATCAAT

ATTATTCAATAACAGGGGATTTTAATAGAACTACATTGAATATGTTAGATGATAAGGGGTGAACTATATCTTTTAGAATT

AATTCCTTTTTCTTCTTGATTACCTGCAGCTATGGCAGCACCGACTCCTGTTTCTGCTTTAGTACATTCTTCTACATTAG

AATTTATTTTCAAAATGTAAAATCTTATAATGCTGGTATATTAACAGCTTTATCTAATCGAATCGGGGATGTTGCTTTAT

CTTTACATTCAGTATCTATTGTAATAACTTTTTTATTTGATTGAATAAGTTTAATATTTATATCTTTTGTTTTATTAATT

AATTTTTATTAGATTTACTTTATTTATTTTAAGATTAAAGTTTTTATTAATAGATTTAGTATATTTTATTGAATGAGAAATT

GAGGATATCAACCTGAACGTTTACAGGCGGGGATTTATTTATTATTTTATACTTTACTGGCTTCGTTGCCGTTATTAATT

AATTATGAAAGTTACTTTAGTATATTTTTTTTAACTTTTTGTGTTTGTGAAGGGGTTCTAGGGCTGTCCATTTTAGTTTC

AATTATTATATTTATTTTTGGAAGTATTGTTTTTATTTCTAGTCGTAAGCATTTACTTTGTACTTTATTGAGATTAGAGT

AATTTTTATAGGAGGAATATTAGTTTTATTCATTTATGTAACTTCTCTTTCATCTAATGAAATATTTTCATTATCTATAAAACTATTTTTTTTATCTTTAAGTATAATT

AATTTTAACAGGATTATTCTTAGCAATACACTATACTGCTGATATTGAAACAGCTTTTAATAGAGTAAATCACATTTATCGTGATGTTAATAATGGTTGATTCCTACGAATT

CTGTTGATAATGCTACTTTAACTCGATTCTTTACATTTCATTTTATTCTCCCTTTCATTGTATTAGCTTTAACTATAATT

CTTTTGATTTTGCTGAAGGGGAGTCTGAGTTAGTTTCAGGATTTAATGTAGAATATAGAAGAGGGGGATTTGCTTTAATT

AATTTTTTTATATATCAGAAATATATATGATTTATTGTATTTTGTTTTCCTTTAGGTCTCGTTTGATTTGCATCTTGTTT

AATTTAAGCTATAAACTTTTTGTAATAAACTAGGATTAGATACCCTATTATTAAAAATAAATATTAAAATGCTAAAGTAG

>BrK06-1

AATTTTATCTACTTTATTATTAAAAAGAGGAGCAGCTCCTTTTCATTTTTGATTCCCAGGAGTTATAGAAGGATTAAATT

AATTAATCATATTGGTTGAATATTAATAGCTATAATAAATAACGAACTTTTATGATTAACTTATTTTTTATTATATTCAATT

CAACAAATCATAAAGATATTGGAACTTTATATTTCATTTTTGGAGTATGATCTGGAATAGTCGGAACTTCTCTAAGAATT

AATTGGATTATTAGGATTTATTGTTTGAGCTCATCATATATTTACAGTAGGTATAGACGTAGATACTCGAGCTTATTTTACTTCAGCAACTATAATT

CATGATACTTATTACGTAGTTGCCCATTTTCATTACGTTTTATCTATAGGAGCTGTATTTGCTATTATAGCAGGATTTATTCATTGATACCCTTTATTAACAGGAATAGTTATAAACCCTTCATG

CTGGAATACCTCGACGATACTCAGATTTTCCTGATAGCTACTTAACTTGAAATATTATTTCTTCTTTAGGAAGAACAATT

AATTTGTCACACACAACATATATTTGCTCATTTAGTTCCTCAAGGAACTCCTCCTGTTTTAATACCTTTTATAGTATGCATTGAAACTATTAGTAATGTAATCCGACCAGGAACTTTAGCAGTACGATTGACTGCTAATATAATT

GACGAGATATTTCTCGAGAAGGAACTTTTCAAGGACTTCACACTATTCCCGTAACATTAGGATTACGATGAGGAATAATTTTATTTATTATTTCTGAAGTTTTTTTCTTTATTTCCTTCTTTTGAGCTTTTTTCCATAGTAGCTTATCTCCAACAATCGAATT

AATTGTCATTTTTCTAGAAGTCATCATTTTGGATTCGAAGCAGCTGCTTGATATTGACATTTTGTTGATGTAGTTTGACTATTCTTATATATTTCAATT

AATTTTAGGAATATATTCTTATAAAATGTTTGATCAAGGATGAAGTGAATATTTTGGAGGTCAGATATTATATAATCAAT

ATTATTCAATAACAGGGGATTTTAATAGAACTACATTGAATATGTTAGATGATAAGGGGTGAACTATATCTTTTAGAATT

AATTCCTTTTTCTTCTTGATTACCTGCAGCTATGGCAGCACCGACTCCTGTTTCTGCTTTAGTACATTCTTCTACATTAG

AATTTATTTTCAAAATGTAAAATCTTATAATGCTGGTATATTAACAGCTTTATCTAATCGAATCGGGGATGTTGCTTTAT

CTTTACATTCAGTATCTATTGTAATAACTTTTTTATTTGATTGAATAAGTTTAATATTTATATCTTTTGTTTTATTAATT

AATTTTTATTAGATTTACTTTATTTATTTTAAGATTAAAGTTTTTATTAATAGATTTAGTATATTTTATTGAATGAGAAATT

GAGGATATCAACCTGAACGTTTACAGGCGGGGATTTATTTATTATTTTATACTTTACTGGCTTCGTTGCCGTTATTAATT

AATTATGAAAGTTACTTTAGTATATTTTTTTTAACTTTTTGTGTTTGTGAAGGGGTTCTAGGGCTGTCCATTTTAGTTTC

AATTATTATATTTATTTTTGGAAGTATTGTTTTTATTTCTAGTCGTAAGCATTTACTTTGTACTTTATTGAGATTAGAGT

AATTTTTATAGGAGGAATATTAGTTTTATTCATTTATGTAACTTCTCTTTCATCTAATGAAATATTTTCATTATCTATAAAACTATTTTTTTTATCTTTAAGTATAATT

AATTTTAACAGGATTATTCTTAGCAATACACTATACTGCTGATATTGAAACAGCTTTTAATAGAGTAAATCACATTTATCGTGATGTTAATAATGGTTGATTCCTACGAATT

CTGTTGATAATGCTACTTTAACTCGATTCTTTACATTTCATTTTATTCTCCCTTTCATTGTATTAGCTTTAACTATAATT

CTTTTGATTTTGCTGAAGGGGAGTCTGAGTTAGTTTCAGGATTTAATGTAGAATATAGAAGAGGGGGATTTGCTTTAATT

AATTTTTTTATATATCAGAAATATATATGATTTATTGTATTTTGTTTTCCTTTAGGTCTCGTTTGATTTGCATCTTGTTT

AATTTAAGCTATAAACTTTTTGTAATAAACTAGGATTAGATACCCTATTATTAAAAATAAATATTAAAATGCTAAAGTAG

>BrK06-2

AATTTTATCTACTTTATTATTAAAAAGAGGAGCAGCTCCTTTTCATTTTTGATTCCCAGGAGTTATAGAAGGATTAAATT

AATTAATCATATTGGTTGAATATTAATAGCTATAATAAATAACGAACTTTTATGATTAACTTATTTTTTATTATATTCAATT

CAACAAATCATAAAGATATTGGAACTTTATATTTCATTTTTGGAGTATGATCTGGAATAGTCGGAACTTCTCTAAGAATT

AATTGGATTATTAGGATTTATTGTTTGAGCTCATCATATATTTACAGTAGGTATAGACGTAGATACTCGAGCTTATTTTACTTCAGCAACTATAATT

CATGATACTTATTACGTAGTTGCCCATTTTCATTACGTTTTATCTATAGGAGCTGTATTTGCTATTATAGCAGGATTTATTCATTGATACCCTTTATTAACAGGAATAGTTATAAACCCTTCATG

CTGGAATACCTCGACGATACTCAGATTTTCCTGATAGCTACTTAACTTGAAATATTATTTCTTCTTTAGGAAGAACAATT

AATTTGTCACACACAACATATATTTGCTCATTTAGTTCCTCAAGGAACTCCTCCTGTTTTAATACCTTTTATAGTATGCATTGAAACTATTAGTAATGTAATCCGACCAGGAACTTTAGCAGTACGATTGACTGCTAATATAATT

GACGAGATATTTCTCGAGAAGGAACTTTTCAAGGACTTCACACTATTCCCGTAACATTAGGATTACGATGAGGAATAATTTTATTTATTATTTCTGAAGTTTTTTTCTTTATTTCCTTCTTTTGAGCTTTTTTCCATAGTAGCTTATCTCCAACAATCGAATT

AATTGTCATTTTTCTAGAAGTCATCATTTTGGATTCGAAGCAGCTGCTTGATATTGACATTTTGTTGATGTAGTTTGACTATTCTTATATATTTCAATT

AATTTTAGGAATATATTCTTATAAAATGTTTGATCAAGGATGAAGTGAATATTTTGGAGGTCAGATATTATATAATCAAT

ATTATTCAATAACAGGGGATTTTAATAGAACTACATTGAATATGTTAGATGATAAGGGGTGAACTATATCTTTTAGAATT

AATTCCTTTTTCTTCTTGATTACCTGCAGCTATGGCAGCACCGACTCCTGTTTCTGCTTTAGTACATTCTTCTACATTAG

AATTTATTTTCAAAATGTAAAATCTTATAATGCTGGTATATTAACAGCTTTATCTAATCGAATCGGGGATGTTGCTTTAT

CTTTACATTCAGTATCTATTGTAATAACTTTTTTATTTGATTGAATAAGTTTAATATTTATATCTTTTGTTTTATTAATT

AATTTTTATTAGATTTACTTTATTTATTTTAAGATTAAAGTTTTTATTAATAGATTTAGTATATTTTATTGAATGAGAAATT

GAGGATATCAACCTGAACGTTTACAGGCGGGGATTTATTTATTATTTTATACTTTACTGGCTTCGTTGCCGTTATTAATT

NATTATGAAAGTTACTTTAGTATATTTTTTTTAACTTTTTGTGTTTGTGAAGGGGTTCTAGGGCTGTCCATTTTAGTTTC

AATTATTATATTTATTTTTGGAAGTATTGTTTTTATTTCTAGTCGTAAGCATTTACTTTGTACTTTATTGAGATTAGAGT

AATTTTTATAGGAGGAATATTAGTTTTATTCATTTATGTAACTTCTCTTTCATCTAATGAAATATTTTCATTATCTATAAAACTATTTTTTTTATCTTTAAGTATAATT

AATTTTAACAGGATTATTCTTAGCAATACACTATACTGCTGATATTGAAACAGCTTTTAATAGAGTAAATCACATTTATCGTGATGTTAATAATGGTTGATTCCTACGAATT

CTGTTGATAATGCTACTTTAACTCGATTCTTTACATTTCATTTTATTCTCCCTTTCATTGTATTAGCTTTAACTATAATT

CTTTTGATTTTGCTGAAGGGGAGTCTGAGTTAGTTTCAGGATTTAATGTAGAATATAGAAGAGGGGGATTTGCTTTAATT

AATTTTTTTATATATCAGAAATATATATGATTTATTGTATTTTGTTTTCCTTTAGGTCTCGTTTGATTTGCATCTTGTTT

AATTTAAGCTATAAACTTTTTGTAATAAACTAGGATTAGATACCCTATTATTAAAAATAAATATTAAAATGCTAAAGTAG

>BrK09-1

AATTTTATCTACTTTATTATTAAAAAGAGGAGCAGCTCCTTTTCATTTTTGATTCCCAGGAGTTATAGAAGGATTAAATT

AATTAATCATATTGGTTGAATATTAATAGCTATAATAAATAACGAACTTTTATGATTAACTTATTTTTTATTATATTCAATT

CAACAAATCATAAAGATATTGGAACTTTATATTTCATTTTTGGAGTATGATCTGGAATAGTCGGAACTTCTCTAAGAATT

AATTGGATTATTAGGATTTATTGTTTGAGCTCATCATATATTTACAGTAGGTATAGACGTAGATACTCGAGCTTATTTTACTTCAGCAACTATAATT

CATGATACTTATTACGTAGTTGCCCATTTTCATTACGTTTTATCTATAGGAGCTGTATTTGCTATTATAGCAGGATTTATTCATTGATACCCTTTATTAACAGGAATAGTTATAAACCCTTCATG

CTGGAATACCTCGACGATACTCAGATTTTCCTGATAGCTACTTAACTTGAAATATTATTTCTTCTTTAGGAAGAACAATT

AATTTGTCACACACAACATATATTTGCTCATTTAGTTCCTCAAGGAACTCCTCCTGTTTTAATACCTTTTATAGTATGCATTGAAACTATTAGTAATGTAATCCGACCAGGAACTTTAGCAGTACGATTGACTGCTAATATAATT

GACGAGATATTTCTCGAGAAGGAACTTTTCAAGGACTTCACACTATTCCCGTAACATTAGGATTACGATGAGGAATAATTTTATTTATTATTTCTGAAGTTTTTTTCTTTATTTCCTTCTTTTGAGCTTTTTTCCATAGTAGCTTATCTCCAACAATCGAATT

AATTGTCATTTTTCTAGAAGTCATCATTTTGGATTCGAAGCAGCTGCTTGATATTGACATTTTGTTGATGTAGTTTGACTATTCTTATATATTTCAATT

AATTTTAGGAATATATTCTTATAAAATGTTTGATCAAGGATGAAGTGAATATTTTGGAGGTCAGATATTATATAATCAAT

ATTATTCAATAACAGGGGATTTTAATAGAACTACATTGAATATGTTAGATGATAAGGGGTGAACTATATCTTTTAGAATT

AATTCCTTTTTCTTCTTGATTACCTGCAGCTATGGCAGCACCGACTCCTGTTTCTGCTTTAGTACATTCTTCTACATTAG

AATTTATTTTCAAAATGTAAAATCTTATAATGCTGGTATATTAACAGCTTTATCTAATCGAATCGGGGATGTTGCTTTAT

CTTTACATTCAGTATCTATTGTAATAACTTTTTTATTTGATTGAATAAGTTTAATATTTATATCTTTTGTTTTATTAATT

AATTTTTATTAGATTTACTTTATTTATTTTAAGATTAAAGTTTTTATTAATAGATTTAGTATATTTTATTGAATGAGAAATT

GAGGATATCAACCTGAACGTTTACAGGCGGGGATTTATTTATTATTTTATACTTTACTGGCTTCGTTGCCGTTATTAATT

AATTATGAAAGTTACTTTAGTATATTTTTTTTAACTTTTTGTGTTTGTGAAGGGGTTCTAGGGCTGTCCATTTTAGTTTC

AATTATTATATTTATTTTTGGAAGTATTGTTTTTATTTCTAGTCGTAAGCATTTACTTTGTACTTTATTGAGATTAGAGT

AATTTTTATAGGAGGAATATTAGTTTTATTCATTTATGTAACTTCTCTTTCATCTAATGAAATATTTTCATTATCTATAAAACTATTTTTTTTATCTTTAAGTATAATT

AATTTTAACAGGATTATTCTTAGCAATACACTATACTGCTGATATTGAAACAGCTTTTAATAGAGTAAATCACATTTATCGTGATGTTAATAATGGTTGATTCCTACGAATT

CTGTTGATAATGCTACTTTAACTCGATTCTTTACATTTCATTTTATTCTCCCTTTCATTGTATTAGCTTTAACTATAATT

CTTTTGATTTTGCTGAAGGGGAGTCTGAGTTAGTTTCAGGATTTAATGTAGAATATAGAAGAGGGGGATTTGCTTTAATT

AATTTTTTTATATATCAGAAATATATATGATTTATTGTATTTTGTTTTCCTTTAGGTCTCGTTTGATTTGCATCTTGTTT

AATTTAAGCTATAAACTTTTTGTAATAAACTAGGATTAGATACCCTATTATTAAAAATAAATATTAAAATGCTAAAGTAG

>BrK09-2

AATTTTATCTACTTTATTATTAAAAAGAGGAGCAGCTCCTTTTCATTTTTGATTCCCAGGAGTTATAGAAGGATTAAATT

AATTAATCATATTGGTTGAATATTAATAGCTATAATAAATAACGAACTTTTATGATTAACTTATTTTTTATTATATTCAATT

CAACAAATCATAAAGATATTGGAACTTTATATTTCATTTTTGGAGTATGATCTGGAATAGTCGGAACTTCTCTAAGAATT

AATTGGATTATTAGGATTTATTGTTTGAGCTCATCATATATTTACAGTAGGTATAGACGTAGATACTCGAGCTTATTTTACTTCAGCAACTATAATT

CATGATACTTATTACGTAGTTGCCCATTTTCATTACGTTTTATCTATAGGAGCTGTATTTGCTATTATAGCAGGATTTATTCATTGATACCCTTTATTAACAGGAATAGTTATAAACCCTTCATG

CTGGAATACCTCGACGATACTCAGATTTTCCTGATAGCTACTTAACTTGAAATATTATTTCTTCTTTAGGAAGAACAATT

AATTTGTCACACACAACATATATTTGCTCATTTAGTTCCTCAAGGAACTCCTCCTGTTTTAATACCTTTTATAGTATGCATTGAAACTATTAGTAATGTAATCCGACCAGGAACTTTAGCAGTACGATTGACTGCTAATATAATT

GACGAGATATTTCTCGAGAAGGAACTTTTCAAGGACTTCACACTATTCCCGTAACATTAGGATTACGATGAGGAATAATTTTATTTATTATTTCTGAAGTTTTTTTCTTTATTTCCTTCTTTTGAGCTTTTTTCCATAGTAGCTTATCTCCAACAATCGAATT

AATTGTCATTTTTCTAGAAGTCATCATTTTGGATTCGAAGCAGCTGCTTGATATTGACATTTTGTTGATGTAGTTTGACTATTCTTATATATTTCAATT

AATTTTAGGAATATATTCTTATAAAATGTTTGATCAAGGATGAAGTGAATATTTTGGAGGTCAGATATTATATAATCAAT

ATTATTCAATAACAGGGGATTTTAATAGAACTACATTGAATATGTTAGATGATAAGGGGTGAACTATATCTTTTAGAATT

AATTCCTTTTTCTTCTTGATTACCTGCAGCTATGGCAGCACCGACTCCTGTTTCTGCTTTAGTACATTCTTCTACATTAG

AATTTATTTTCAAAATGTAAAATCTTATAATGCTGGTATATTAACAGCTTTATCTAATCGAATCGGGGATGTTGCTTTAT

CTTTACATTCAGTATCTATTGTAATAACTTTTTTATTTGATTGAATAAGTTTAATATTTATATCTTTTGTTTTATTAATT

AATTTTTATTAGATTTACTTTATTTATTTTAAGATTAAAGTTTTTATTAATAGATTTAGTATATTTTATTGAATGAGAAATT

GAGGATATCAACCTGAACGTTTACAGGCGGGGATTTATTTATTATTTTATACTTTACTGGCTTCGTTGCCGTTATTAATT

AATTATGAAAGTTACTTTAGTATATTTTTTTTAACTTTTTGTGTTTGTGAAGGGGTTCTAGGGCTGTCCATTTTAGTTTC

AATTATTATATTTATTTTTGGAAGTATTGTTTTTATTTCTAGTCGTAAGCATTTACTTTGTACTTTATTGAGATTAGAGT

AATTTTTATAGGAGGAATATTAGTTTTATTCATTTATGTAACTTCTCTTTCATCTAATGAAATATTTTCATTATCTATAAAACTATTTTTTTTATCTTTAAGTATAATT

AATTTTAACAGGATTATTCTTAGCAATACACTATACTGCTGATATTGAAACAGCTTTTAATAGAGTAAATCACATTTATCGTGATGTTAATAATGGTTGATTCCTACGAATT

CTGTTGATAATGCTACTTTAACTCGATTCTTTACATTTCATTTTATTCTCCCTTTCATTGTATTAGCTTTAACTATAATT

CTTTTGATTTTGCTGAAGGGGAGTCTGAGTTAGTTTCAGGATTTAATGTAGAATATAGAAGAGGGGGATTTGCTTTAATT

AATTTTTTTATATATCAGAAATATATATGATTTATTGTATTTTGTTTTCCTTTAGGTCTCGTTTGATTTGCATCTTGTTT

AATTTAAGCTATAAACTTTTTGTAATAAACTAGGATTAGATACCCTATTATTAAAAATAAATATTAAAATGCTAAAGTAG

>BrK09-3

AATTTTATCTACTTTATTATTAAAAAGAGGAGCAGCTCCTTTTCATTTTTGATTCCCAGGAGTTATAGAAGGATTAAATT

AATTAATCATATTGGTTGAATATTAATAGCTATAATAAATAACGAACTTTTATGATTAACTTATTTTTTATTATATTCAATT

CAACAAATCATAAAGATATTGGAACTTTATATTTCATTTTTGGAGTATGATCTGGAATAGTCGGAACTTCTCTAAGAATT

AATTGGATTATTAGGATTTATTGTTTGAGCTCATCATATATTTACAGTAGGTATAGACGTAGATACTCGAGCTTATTTTACTTCAGCAACTATAATT

CATGATACTTATTACGTAGTTGCCCATTTTCATTACGTTTTATCTATAGGAGCTGTATTTGCTATTATAGCAGGATTTATTCATTGATACCCTTTATTAACAGGAATAGTTATAAACCCTTCATG

CTGGAATACCTCGACGATACTCAGATTTTCCTGATAGCTACTTAACTTGAAATATTATTTCTTCTTTAGGAAGAACAATT

AATTTGTCACACACAACATATATTTGCTCATTTAGTTCCTCAAGGAACTCCTCCTGTTTTAATACCTTTTATAGTATGCATTGAAACTATTAGTAATGTAATCCGACCAGGAACTTTAGCAGTACGATTGACTGCTAATATAATT

GACGAGATATTTCTCGAGAAGGAACTTTTCAAGGACTTCACACTATTCCCGTAACATTAGGATTACGATGAGGAATAATTTTATTTATTATTTCTGAAGTTTTTTTCTTTATTTCCTTCTTTTGAGCTTTTTTCCATAGTAGCTTATCTCCAACAATCGAATT

AATTGTCATTTTTCTAGAAGTCATCATTTTGGATTCGAAGCAGCTGCTTGATATTGACATTTTGTTGATGTAGTTTGACTATTCTTATATATTTCAATT

AATTTTAGGAATATATTCTTATAAAATGTTTGATCAAGGATGAAGTGAATATTTTGGAGGTCAGATATTATATAATCAAT

ATTATTCAATAACAGGGGATTTTAATAGAACTACATTGAATATGTTAGATGATAAGGGGTGAACTATATCTTTTAGAATT

AATTCCTTTTTCTTCTTGATTACCTGCAGCTATGGCAGCACCGACTCCTGTTTCTGCTTTAGTACATTCTTCTACATTAG

AATTTATTTTCAAAATGTAAAATCTTATAATGCTGGTATATTAACAGCTTTATCTAATCGAATCGGGGATGTTGCTTTAT

CTTTACATTCAGTATCTATTGTAATAACTTTTTTATTTGATTGAATAAGTTTAATATTTATATCTTTTGTTTTATTAATT

AATTTTTATTAGATTTACTTTATTTATTTTAAGATTAAAGTTTTTATTAATAGATTTAGTATATTTTATTGAATGAGAAATT

GAGGATATCAACCTGAACGTTTACAGGCGGGGATTTATTTATTATTTTATACTTTACTGGCTTCGTTGCCGTTATTAATT

AATTATGAAAGTTACTTTAGTATATTTTTTTTAACTTTTTGTGTTTGTGAAGGGGTTCTAGGGCTGTCCATTTTAGTTTC

AATTATTATATTTATTTTTGGAAGTATTGTTTTTATTTCTAGTCGTAAGCATTTACTTTGTACTTTATTGAGATTAGAGT

AATTTTTATAGGAGGAATATTAGTTTTATTCATTTATGTAACTTCTCTTTCATCTAATGAAATATTTTCATTATCTATAAAACTATTTTTTTTATCTTTAAGTATAATT

AATTTTAACAGGATTATTCTTAGCAATACACTATACTGCTGATATTGAAACAGCTTTTAATAGAGTAAATCACATTTATCGTGATGTTAATAATGGTTGATTCCTACGAATT

CTGTTGATAATGCTACTTTAACTCGATTCTTTACATTTCATTTTATTCTCCCTTTCATTGTATTAGCTTTAACTATAATT

CTTTTGATTTTGCTGAAGGGGAGTCTGAGTTAGTTTCAGGATTTAATGTAGAATATAGAAGAGGGGGATTTGCTTTAATT

AATTTTTTTATATATCAGAAATATATATGATTTATTGTATTTTGTTTTCCTTTAGGTCTCGTTTGATTTGCATCTTGTTT

AATTTAAGCTATAAACTTTTTGTAATAAACTAGGATTAGATACCCTATTATTAAAAATAAATATTAAAATGCTAAAGTAG

>BrK09-4

AATTTTATCTACTTTATTATTAAAAAGAGGAGCAGCTCCTTTTCATTTTTGATTCCCAGGAGTTATAGAAGGATTAAATT

AATTAATCATATTGGTTGAATATTAATAGCTATAATAAATAACGAACTTTTATGATTAACTTATTTTTTATTATATTCAATT

CAACAAATCATAAAGATATTGGAACTTTATATTTCATTTTTGGAGTATGATCTGGAATAGTCGGAACTTCTCTAAGAATT

AATTGGATTATTAGGATTTATTGTTTGAGCTCATCATATATTTACAGTAGGTATAGACGTAGATACTCGAGCTTATTTTACTTCAGCAACTATAATT

CATGATACTTATTACGTAGTTGCCCATTTTCATTACGTTTTATCTATAGGAGCTGTATTTGCTATTATAGCAGGATTTATTCATTGATACCCTTTATTAACAGGAATAGTTATAAACCCTTCATG

CTGGAATACCTCGACGATACTCAGATTTTCCTGATAGCTACTTAACTTGAAATATTATTTCTTCTTTAGGAAGAACAATT

AATTTGTCACACACAACATATATTTGCTCATTTAGTTCCTCAAGGAACTCCTCCTGTTTTAATACCTTTTATAGTATGCATTGAAACTATTAGTAATGTAATCCGACCAGGAACTTTAGCAGTACGATTGACTGCTAATATAATT

GACGAGATATTTCTCGAGAAGGAACTTTTCAAGGACTTCACACTATTCCCGTAACATTAGGATTACGATGAGGAATAATTTTATTTATTATTTCTGAAGTTTTTTTCTTTATTTCCTTCTTTTGAGCTTTTTTCCATAGTAGCTTATCTCCAACAATCGAATT

AATTGTCATTTTTCTAGAAGTCATCATTTTGGATTCGAAGCAGCTGCTTGATATTGACATTTTGTTGATGTAGTTTGACTATTCTTATATATTTCAATT

AATTTTAGGAATATATTCTTATAAAATGTTTGATCAAGGATGAAGTGAATATTTTGGAGGTCAGATATTATATAATCAAT

ATTATTCAATAACAGGGGATTTTAATAGAACTACATTGAATATGTTAGATGATAAGGGGTGAACTATATCTTTTAGAATT

AATTCCTTTTTCTTCTTGATTACCTGCAGCTATGGCAGCACCGACTCCTGTTTCTGCTTTAGTACATTCTTCTACATTAG

AATTTATTTTCAAAATGTAAAATCTTATAATGCTGGTATATTAACAGCTTTATCTAATCGAATCGGGGATGTTGCTTTAT

CTTTACATTCAGTATCTATTGTAATAACTTTTTTATTTGATTGAATAAGTTTAATATTTATATCTTTTGTTTTATTAATT

AATTTTTATTAGATTTACTTTATTTATTTTAAGATTAAAGTTTTTATTAATAGATTTAGTATATTTTATTGAATGAGAAATT

GAGGATATCAACCTGAACGTTTACAGGCGGGGATTTATTTATTATTTTATACTTTACTGGCTTCGTTGCCGTTATTAATT

AATTATGAAAGTTACTTTAGTATATTTTTTTTAACTTTTTGTGTTTGTGAAGGGGTTCTAGGGCTGTCCATTTTAGTTTC

AATTATTATATTTATTTTTGGAAGTATTGTTTTTATTTCTAGTCGTAAGCATTTACTTTGTACTTTATTGAGATTAGAGT

AATTTTTATAGGAGGAATATTAGTTTTATTCATTTATGTAACTTCTCTTTCATCTAATGAAATATTTTCATTATCTATAAAACTATTTTTTTTATCTTTAAGTATAATT

AATTTTAACAGGATTATTCTTAGCAATACACTATACTGCTGATATTGAAACAGCTTTTAATAGAGTAAATCACATTTATCGTGATGTTAATAATGGTTGATTCCTACGAATT

CTGTTGATAATGCTACTTTAACTCGATTCTTTACATTTCATTTTATTCTCCCTTTCATTGTATTAGCTTTAACTATAATT

CTTTTGATTTTGCTGAAGGGGAGTCTGAGTTAGTTTCAGGATTTAATGTAGAATATAGAAGAGGGGGATTTGCTTTAATT

AATTTTTTTATATATCAGAAATATATATGATTTATTGTATTTTGTTTTCCTTTAGGTCTCGTTTGATTTGCATCTTGTTT

AATTTAAGCTATAAACTTTTTGTAATAAACTAGGATTAGATACCCTATTATTAAAAATAAATATTAAAATGCTAAAGTAG

>BrK11-1

AATTTTATCTACTTTATTATTAAAAAGAGGAGCAGCTCCTTTTCATTTTTGATTCCCAGGAGTTATAGAAGGATTAAATT

AATTAATCATATTGGTTGAATATTAATAGCTATAATAAATAACGAACTTTTATGATTAACTTATTTTTTATTATATTCAATT

CAACAAATCATAAAGATATTGGAACTTTATATTTCATTTTTGGAGTATGATCTGGAATAGTCGGAACTTCTCTAAGAATT

AATTGGATTATTAGGATTTATTGTTTGAGCTCATCATATATTTACAGTAGGTATAGACGTAGATACTCGAGCTTATTTTACTTCAGCAACTATAATT

CATGATACTTATTACGTAGTTGCCCATTTTCATTACGTTTTATCTATAGGAGCTGTATTTGCTATTATAGCAGGATTTATTCATTGATACCCTTTATTAACAGGAATAGTTATAAACCCTTCATG

CTGGAATACCTCGACGATACTCAGATTTTCCTGATAGCTACTTAACTTGAAATATTATTTCTTCTTTAGGAAGAACAATT

AATTTGTCACACACAACATATATTTGCTCATTTAGTTCCTCAAGGAACTCCTCCTGTTTTAATACCTTTTATAGTATGCATTGAAACTATTAGTAATGTAATCCGACCAGGAACTTTAGCAGTACGATTGACTGCTAATATAATT

GACGAGATATTTCTCGAGAAGGAACTTTTCAAGGACTTCACACTATTCCCGTAACATTAGGATTACGATGAGGAATAATTTTATTTATTATTTCTGAAGTTTTTTTCTTTATTTCCTTCTTTTGAGCTTTTTTCCATAGTAGCTTATCTCCAACAATCGAATT

AATTGTCATTTTTCTAGAAGTCATCATTTTGGATTCGAAGCAGCTGCTTGATATTGACATTTTGTTGATGTAGTTTGACTATTCTTATATATTTCAATT

AATTTTAGGAATATATTCTTATAAAATGTTTGATCAAGGATGAAGTGAATATTTTGGAGGTCAGATATTATATAATCAAT

ATTATTCAATAACAGGGGATTTTAATAGAACTACATTGAATATGTTAGATGATAAGGGGTGAACTATATCTTTTAGAATT

AATTCCTTTTTCTTCTTGATTACCTGCAGCTATGGCAGCACCGACTCCTGTTTCTGCTTTAGTACATTCTTCTACATTAG

AATTTATTTTCAAAATGTAAAATCTTATAATGCTGGTATATTAACAGCTTTATCTAATCGAATCGGGGATGTTGCTTTAT

CTTTACATTCAGTATCTATTGTAATAACTTTTTTATTTGATTGAATAAGTTTAATATTTATATCTTTTGTTTTATTAATT

AATTTTTATTAGATTTACTTTATTTATTTTAAGATTAAAGTTTTTATTAATAGATTTAGTATATTTTATTGAATGAGAAATT

GAGGATATCAACCTGAACGTTTACAGGCGGGGATTTATTTATTATTTTATACTTTACTGGCTTCGTTGCCGTTATTAATT

AATTATGAAAGTTACTTTAGTATATTTTTTTTAACTTTTTGTGTTTGTGAAGGGGTTCTAGGGCTATCCATTTTAGTTTC

AATTATTATATTTATTTTTGGAAGTATTGTTTTTATTTCTAGTCGTAAGCATTTACTTTGTACTTTATTGAGATTAGAGT

AATTTTTATAGGAGGAATATTAGTTTTATTCATTTATGTAACTTCTCTTTCATCTAATGAAATATTTTCATTATCTATAAAACTATTTTTTTTATCTTTAAGTATAATT

AATTTTAACAGGATTATTCTTAGCAATACACTATACTGCTGATATTGAAACAGCTTTTAATAGAGTAAATCACATTTATCGTGATGTTAATAATGGTTGATTCCTACGAATT

CTGTTGATAATGCTACTTTAACTCGATTCTTTACATTTCATTTTATTCTCCCTTTCATTGTATTAGCTTTAACTATAATT

CTTTTGATTTTGCTGAAGGGGAGTCTGAGTTAGTTTCAGGATTTAATGTAGAATATAGAAGAGGGGGATTTGCTTTAATT

AATTTTTTTATATATCAGAAATATATATGATTTATTGTATTTTGTTTTCCTTTAGGTCTCGTTTGATTTGCATCTTGTTT

AATTTAAGCTATAAACTTTTTGTAATAAACTAGGATTAGATACCCTATTATTAAAAATAAATATTAAAATGCTAAAGTAG

>BrK11-2

AATTTTATCTACTTTATTATTAAAAAGAGGAGCAGCTCCTTTTCATTTTTGATTCCCAGGAGTTATAGAAGGATTAAATT

AATTAATCATATTGGTTGAATATTAATAGCTATAATAAATAACGAACTTTTATGATTAACTTATTTTTTATTATATTCAATT

CAACAAATCATAAAGATATTGGAACTTTATATTTCATTTTTGGAGTATGATCTGGAATAGTCGGAACTTCTCTAAGAATT

AATTGGATTATTAGGATTTATTGTTTGAGCTCATCATATATTTACAGTAGGTATAGACGTAGATACTCGAGCTTATTTTACTTCAGCAACTATAATT

CATGATACTTATTACGTAGTTGCCCATTTTCATTACGTTTTATCTATAGGAGCTGTATTTGCTATTATAGCAGGATTTATTCATTGATACCCTTTATTAACAGGAATAGTTATAAACCCTTCATG

CTGGAATACCTCGACGATACTCAGATTTTCCTGATAGCTACTTAACTTGAAATATTATTTCTTCTTTAGGAAGAACAATT

AATTTGTCACACACAACATATATTTGCTCATTTAGTTCCTCAAGGAACTCCTCCTGTTTTAATACCTTTTATAGTATGCATTGAAACTATTAGTAATGTAATCCGACCAGGAACTTTAGCAGTACGATTGACTGCTAATATAATT

GACGAGATATTTCTCGAGAAGGAACTTTTCAAGGACTTCACACTATTCCCGTAACATTAGGATTACGATGAGGAATAATTTTATTTATTATTTCTGAAGTTTTTTTCTTTATTTCCTTCTTTTGAGCTTTTTTCCATAGTAGCTTATCTCCAACAATCGAATT

AATTGTCATTTTTCTAGAAGTCATCATTTTGGATTCGAAGCAGCTGCTTGATATTGACATTTTGTTGATGTAGTTTGACTATTCTTATATATTTCAATT

AATTTTAGGAATATATTCTTATAAAATGTTTGATCAAGGATGAAGTGAATATTTTGGAGGTCAGATATTATATAATCAAT

ATTATTCAATAACAGGGGATTTTAATAGAACTACATTGAATATGTTAGATGATAAGGGGTGAACTATATCTTTTAGAATT

AATTCCTTTTTCTTCTTGATTACCTGCAGCTATGGCAGCACCGACTCCTGTTTCTGCTTTAGTACATTCTTCTACATTAG

AATTTATTTTCAAAATGTAAAATCTTATAATGCTGGTATATTAACAGCTTTATCTAATCGAATCGGGGATGTTGCTTTAT

CTTTACATTCAGTATCTATTGTAATAACTTTTTTATTTGATTGAATAAGTTTAATATTTATATCTTTTGTTTTATTAATT

AATTTTTATTAGATTTACTTTATTTATTTTAAGATTAAAGTTTTTATTAATAGATTTAGTATATTTTATTGAATGAGAAATT

GAGGATATCAACCTGAACGTTTACAGGCGGGGATTTATTTATTATTTTATACTTTACTGGCTTCGTTGCCGTTATTAATT

AATTATGAAAGTTACTTTAGTATATTTTTTTTAACTTTTTGTGTTTGTGAAGGGGTTCTAGGGCTGTCCATTTTAGTTTC

AATTATTATATTTATTTTTGGAAGTATTGTTTTTATTTCTAGTCGTAAGCATTTACTTTGTACTTTATTGAGATTAGAGT

AATTTTTATAGGAGGAATATTAGTTTTATTCATTTATGTAACTTCTCTTTCATCTAATGAAATATTTTCATTATCTATAAAACTATTTTTTTTATCTTTAAGTATAATT

AATTTTAACAGGATTATTCTTAGCAATACACTATACTGCTGATATTGAAACAGCTTTTAATAGAGTAAATCACATTTATCGTGATGTTAATAATGGTTGATTCCTACGAATT

CTGTTGATAATGCTACTTTAACTCGATTCTTTACATTTCATTTTATTCTCCCTTTCATTGTATTAGCTTTAACTATAATT

CTTTTGATTTTGCTGAAGGGGAGTCTGAGTTAGTTTCAGGATTTAATGTAGAATATAGAAGAGGGGGATTTGCTTTAATT

AATTTTTTTATATATCAGAAATATATATGATTTATTGTATTTTGTTTTCCTTTAGGTCTCGTTTGATTTGCATCTTGTTT

AATTTAAGCTATAAACTTTTTGTAATAAACTAGGATTAGATACCCTATTATTAAAAATAAATATTAAAATGCTAAAGTAG

>BrK13-1

AATTTTATCTACTTTATTATTAAAAAGAGGAGCAGCTCCTTTTCATTTTTGATTCCCAGGAGTTATAGAAGGATTAAATT

AATTAATCATATTGGTTGAATATTAATAGCTATAATAAATAACGAACTTTTATGATTAACTTATTTTTTATTATATTCAATT

CAACAAATCATAAAGATATTGGAACTTTATATTTCATTTTTGGAGTATGATCTGGAATAGTCGGAACTTCTCTAAGAATT

AATTGGATTATTAGGATTTATTGTTTGAGCTCATCATATATTTACAGTAGGTATAGACGTAGATACTCGAGCTTATTTTACTTCAGCAACTATAATT

CATGATACTTATTACGTAGTTGCCCATTTTCATTACGTTTTATCTATAGGAGCTGTATTTGCTATTATAGCAGGATTTATTCATTGATACCCTTTATTAACAGGAATAGTTATAAACCCTTCATG

CTGGAATACCTCGACGATACTCAGATTTTCCTGATAGCTACTTAACTTGAAATATTATTTCTTCTTTAGGAAGAACAATT

AATTTGTCACACACAACATATATTTGCTCATTTAGTTCCTCAAGGAACTCCTCCTGTTTTAATACCTTTTATAGTATGCATTGAAACTATTAGTAATGTAATCCGACCAGGAACTTTAGCAGTACGATTGACTGCTAATATAATT

GACGAGATATTTCTCGAGAAGGAACTTTTCAAGGACTTCACACTATTCCCGTAACATTAGGATTACGATGAGGAATAATTTTATTTATTATTTCTGAAGTTTTTTTCTTTATTTCCTTCTTTTGAGCTTTTTTCCATAGTAGCTTATCTCCAACAATCGAATT

AATTGTCATTTTTCTAGAAGTCATCATTTTGGATTCGAAGCAGCTGCTTGATATTGACATTTTGTTGATGTAGTTTGACTATTCTTATATATTTCAATT

AATTTTAGGAATATATTCTTATAAAATGTTTGATCAAGGATGAAGTGAATATTTTGGAGGTCAGATATTATATAATCAAT

ATTATTCAATAACAGGGGATTTTAATAGAACTACATTGAATATGTTAGATGATAAGGGGTGAACTATATCTTTTAGAATT

AATTCCTTTTTCTTCTTGATTACCTGCAGCTATGGCAGCACCGACTCCTGTTTCTGCTTTAGTACATTCTTCTACATTAG

AATTTATTTTCAAAATGTAAAATCTTATAATGCTGGTATATTAACAGCTTTATCTAATCGAATCGGGGATGTTGCTTTAT

CTTTACATTCAGTATCTATTGTAATAACTTTTTTATTTGATTGAATAAGTTTAATATTTATATCTTTTGTTTTATTAATT

AATTTTTATTAGATTTACTTTATTTATTTTAAGATTAAAGTTTTTATTAATAGATTTAGTATATTTTATTGAATGAGAAATT

GAGGATATCAACCTGAACGTTTACAGGCGGGGATTTATTTATTATTTTATACTTTACTGGCTTCGTTGCCGTTATTAATT

AATTATGAAAGTTACTTTAGTATATTTTTTTTAACTTTTTGTGTTTGTGAAGGGGTTCTAGGGCTGTCCATTTTAGTTTC

AATTATTATATTTATTTTTGGAAGTATTGTTTTTATTTCTAGTCGTAAGCATTTACTTTGTACTTTATTGAGATTAGAGT

AATTTTTATAGGAGGAATATTAGTTTTATTCATTTATGTAACTTCTCTTTCATCTAATGAAATATTTTCATTATCTATAAAACTATTTTTTTTATCTTTAAGTATAATT

AATTTTAACAGGATTATTCTTAGCAATACACTATACTGCTGATATTGAAACAGCTTTTAATAGAGTAAATCACATTTATCGTGATGTTAATAATGGTTGATTCCTACGAATT

CTGTTGATAATGCTACTTTAACTCGATTCTTTACATTTCATTTTATTCTCCCTTTCATTGTATTAGCTTTAACTATAATT

CTTTTGATTTTGCTGAAGGGGAGTCTGAGTTAGTTTCAGGATTTAATGTAGAATATAGAAGAGGGGGATTTGCTTTAATT

AATTTTTTTATATATCAGAAATATATATGATTTATTGTATTTTGTTTTCCTTTAGGTCTCGTTTGATTTGCATCTTGTTT

AATTTAAGCTATAAACTTTTTGTAATAAACTAGGATTAGATACCCTATTATTAAAAATAAATATTAAAATGCTAAAGTAG

>BrK13-2

AATTTTATCTACTTTATTATTAAAAAGAGGAGCAGCTCCTTTTCATTTTTGATTCCCAGGAGTTATAGAAGGATTAAATT

AATTAATCATATTGGTTGAATATTAATAGCTATAATAAATAACGAACTTTTATGATTAACTTATTTTTTATTATATTCAATT

CAACAAATCATAAAGATATTGGAACTTTATATTTCATTTTTGGAGTATGATCTGGAATAGTCGGAACTTCTCTAAGAATT

AATTGGATTATTAGGATTTATTGTTTGAGCTCATCATATATTTACAGTAGGTATAGACGTAGATACTCGAGCTTATTTTACTTCAGCAACTATAATT

CATGATACTTATTACGTAGTTGCCCATTTTCATTACGTTTTATCTATAGGAGCTGTATTTGCTATTATAGCAGGATTTATTCATTGATACCCTTTATTAACAGGAATAGTTATAAACCCTTCATG

CTGGAATACCTCGACGATACTCAGATTTTCCTGATAGCTACTTAACTTGAAATATTATTTCTTCTTTAGGAAGAACAATT

AATTTGTCACACACAACATATATTTGCTCATTTAGTTCCTCAAGGAACTCCTCCTGTTTTAATACCTTTTATAGTATGCATTGAAACTATTAGTAATGTAATCCGACCAGGAACTTTAGCAGTACGATTGACTGCTAATATAATT

GACGAGATATTTCTCGAGAAGGAACTTTTCAAGGACTTCACACTATTCCCGTAACATTAGGATTACGATGAGGAATAATTTTATTTATTATTTCTGAAGTTTTTTTCTTTATTTCCTTCTTTTGAGCTTTTTTCCATAGTAGCTTATCTCCAACAATCGAATT

AATTGTCATTTTTCTAGAAGTCATCATTTTGGATTCGAAGCAGCTGCTTGATATTGACATTTTGTTGATGTAGTTTGACTATTCTTATATATTTCAATT

AATTTTAGGAATATATTCTTATAAAATGTTTGATCAAGGATGAAGTGAATATTTTGGAGGTCAGATATTATATAATCAAT

ATTATTCAATAACAGGGGATTTTAATAGAACTACATTGAATATGTTAGATGATAAGGGGTGAACTATATCTTTTAGAATT

AATTCCTTTTTCTTCTTGATTACCTGCAGCTATGGCAGCACCGACTCCTGTTTCTGCTTTAGTACATTCTTCTACATTAG

AATTTATTTTCAAAATGTAAAATCTTATAATGCTGGTATATTAACAGCTTTATCTAATCGAATCGGGGATGTTGCTTTAT

CTTTACATTCAGTATCTATTGTAATAACTTTTTTATTTGATTGAATAAGTTTAATATTTATATCTTTTGTTTTATTAATT

AATTTTTATTAGATTTACTTTATTTATTTTAAGATTAAAGTTTTTATTAATAGATTTAGTATATTTTATTGAATGAGAAATT

GAGGATATCAACCTGAACGTTTACAGGCGGGGATTTATTTATTATTTTATACTTTACTGGCTTCGTTGCCGTTATTAATT

AATTATGAAAGTTACTTTAGTATATTTTTTTTAACTTTTTGTGTTTGTGAAGGGGTTCTAGGGCTGTCCATTTTAGTTTC

AATTATTATATTTATTTTTGGAAGTATTGTTTTTATTTCTAGTCGTAAGCATTTACTTTGTACTTTATTGAGATTAGAGT

AATTTTTATAGGAGGAATATTAGTTTTATTCATTTATGTAACTTCTCTTTCATCTAATGAAATATTTTCATTATCTATAAAACTATTTTTTTTATCTTTAAGTATAATT

AATTTTAACAGGATTATTCTTAGCAATACACTATACTGCTGATATTGAAACAGCTTTTAATAGAGTAAATCACATTTATCGTGATGTTAATAATGGTTGATTCCTACGAATT

CTGTTGATAATGCTACTTTAACTCGATTCTTTACATTTCATTTTATTCTCCCTTTCATTGTATTAGCTTTAACTATAATT

CTTTTGATTTTGCTGAAGGGGAGTCTGAGTTAGTTTCAGGATTTAATGTAGAATATAGAAGAGGGGGATTTGCTTTAATT

AATTTTTTTATATATCAGAAATATATATGATTTATTGTATTTTGTTTTCCTTTAGGTCTCGTTTGATTTGCATCTTGTTT

AATTTAAGCTATAAACTTTTTGTAATAAACTAGGATTAGATACCCTATTATTAAAAATAAATATTAAAATGCTAAAGTAG

>BrK16-1

AATTTTATCTACTTTATTATTAAAAAGAGGAGCAGCTCCTTTTCATTTTTGATTCCCAGGAGTTATAGAAGGATTAAATT

AATTAATCATATTGGTTGAATATTAATAGCTATAATAAATAACGAACTTTTATGATTAACTTATTTTTTATTATATTCAATT

CAACAAATCATAAAGATATTGGAACTTTATATTTCATTTTTGGAGTATGATCTGGAATAGTCGGAACTTCTCTAAGAATT

AATTGGATTATTAGGATTTATTGTTTGAGCTCATCATATATTTACAGTAGGTATAGACGTAGATACTCGAGCTTATTTTACTTCAGCAACTATAATT

CATGATACTTATTACGTAGTTGCCCATTTTCATTACGTTTTATCTATAGGAGCTGTATTTGCTATTATAGCAGGATTTATTCATTGATACCCTTTATTAACAGGAATAGTTATAAACCCTTCATG

CTGGAATACCTCGACGATACTCAGATTTTCCTGATAGCTACTTAACTTGAAATATTATTTCTTCTTTAGGAAGAACAATT

AATTTGTCACACACAACATATATTTGCTCATTTAGTTCCTCAAGGAACTCCTCCTGTTTTAATACCTTTTATAGTATGCATTGAAACTATTAGTAATGTAATCCGACCAGGAACTTTAGCAGTACGATTGACTGCTAATATAATT

GACGAGATATTTCTCGAGAAGGAACTTTTCAAGGACTTCACACTATTCCCGTAACATTAGGATTACGATGAGGAATAATTTTATTTATTATTTCTGAAGTTTTTTTCTTTATTTCCTTCTTTTGAGCTTTTTTCCATAGTAGCTTATCTCCAACAATCGAATT

AATTGTCATTTTTCTAGAAGTCATCATTTTGGATTCGAAGCAGCTGCTTGATATTGACATTTTGTTGATGTAGTTTGACTATTCTTATATATTTCAATT

AATTTTAGGAATATATTCTTATAAAATGTTTGATCAAGGATGAAGTGAATATTTTGGAGGTCAGATATTATATAATCAAT

ATTATTCAATAACAGGGGATTTTAATAGAACTACATTGAATATGTTAGATGATAAGGGGTGAACTATATCTTTTAGAATT

AATTCCTTTTTCTTCTTGATTACCTGCAGCTATGGCAGCACCGACTCCTGTTTCTGCTTTAGTACATTCTTCTACATTAG

AATTTATTTTCAAAATGTAAAATCTTATAATGCTGGTATATTAACAGCTTTATCTAATCGAATCGGGGATGTTGCTTTAT

CTTTACATTCAGTATCTATTGTAATAACTTTTTTATTTGATTGAATAAGTTTAATATTTATATCTTTTGTTTTATTAATT

AATTTTTATTAGATTTACTTTATTTATTTTAAGATTAAAGTTTTTATTAATAGATTTAGTATATTTTATTGAATGAGAAATT

GAGGATATCAACCTGAACGTTTACAGGCGGGGATTTATTTATTATTTTATACTTTACTGGCTTCGTTGCCGTTATTAATT

AATTATGAAAGTTACTTTAGTATATTTTTTTTAACTTTTTGTGTTTGTGAAGGGGTTCTAGGGCTGTCCATTTTAGTTTC

AATTATTATATTTATTTTTGGAAGTATTGTTTTTATTTCTAGTCGTAAGCATTTACTTTGTACTTTATTGAGATTAGAGT

AATTTTTATAGGAGGAATATTAGTTTTATTCATTTATGTAACTTCTCTTTCATCTAATGAAATATTTTCATTATCTATAAAACTATTTTTTTTATCTTTAAGTATAATT

AATTTTAACAGGATTATTCTTAGCAATACACTATACTGCTGATATTGAAACAGCTTTTAATAGAGTAAATCACATTTATCGTGATGTTAATAATGGTTGATTCCTACGAATT

CTGTTGATAATGCTACTTTAACTCGATTCTTTACATTTCATTTTATTCTCCCTTTCATTGTATTAGCTTTAACTATAATT

CTTTTGATTTTGCTGAAGGGGAGTCTGAGTTAGTTTCAGGATTTAATGTAGAATATAGAAGAGGGGGATTTGCTTTAATT

AATTTTTTTATATATCAGAAATATATATGATTTATTGTATTTTGTTTTCCTTTAGGTCTCGTTTGATTTGCATCTTGTTT

AATTTAAGCTATAAACTTTTTGTAATAAACTAGGATTAGATACCCTATTATTAAAAATAAATATTAAAATGCTAAAGTAG

>BrK16-2

AATTTTATCTACTTTATTATTAAAAAGAGGAGCAGCTCCTTTTCATTTTTGATTCCCAGGAGTTATAGAAGGATTAAATT

AATTAATCATATTGGTTGAATATTAATAGCTATAATAAATAACGAACTTTTATGATTAACTTATTTTTTATTATATTCAATT

CAACAAATCATAAAGATATTGGAACTTTATATTTCATTTTTGGAGTATGATCTGGAATAGTCGGAACTTCTCTAAGAATT

AATTGGATTATTAGGATTTATTGTTTGAGCTCATCATATATTTACAGTAGGTATAGACGTAGATACTCGAGCTTATTTTACTTCAGCAACTATAATT

CATGATACTTATTACGTAGTTGCCCATTTTCATTACGTTTTATCTATAGGAGCTGTATTTGCTATTATAGCAGGATTTATTCATTGATACCCTTTATTAACAGGAATAGTTATAAACCCTTCATG

CTGGAATACCTCGACGATACTCAGATTTTCCTGATAGCTACTTAACTTGAAATATTATTTCTTCTTTAGGAAGAACAATT

AATTTGTCACACACAACATATATTTGCTCATTTAGTTCCTCAAGGAACTCCTCCTGTTTTAATACCTTTTATAGTATGCATTGAAACTATTAGTAATGTAATCCGACCAGGAACTTTAGCAGTACGATTGACTGCTAATATAATT

GACGAGATATTTCTCGAGAAGGAACTTTTCAAGGACTTCACACTATTCCCGTAACATTAGGATTACGATGAGGAATAATTTTATTTATTATTTCTGAAGTTTTTTTCTTTATTTCCTTCTTTTGAGCTTTTTTCCATAGTAGCTTATCTCCAACAATCGAATT

AATTGTCATTTTTCTAGAAGTCATCATTTTGGATTCGAAGCAGCTGCTTGATATTGACATTTTGTTGATGTAGTTTGACTATTCTTATATATTTCAATT

AATTTTAGGAATATATTCTTATAAAATGTTTGATCAAGGATGAAGTGAATATTTTGGAGGTCAGATATTATATAATCAAT

ATTATTCAATAACAGGGGATTTTAATAGAACTACATTGAATATGTTAGATGATAAGGGGTGAACTATATCTTTTAGAATT

AATTCCTTTTTCTTCTTGATTACCTGCAGCTATGGCAGCACCGACTCCTGTTTCTGCTTTAGTACATTCTTCTACATTAG

AATTTATTTTCAAAATGTAAAATCTTATAATGCTGGTATATTAACAGCTTTATCTAATCGAATCGGGGATGTTGCTTTAT

CTTTACATTCAGTATCTATTGTAATAACTTTTTTATTTGATTGAATAAGTTTAATATTTATATCTTTTGTTTTATTAATT

AATTTTTATTAGATTTACTTTATTTATTTTAAGATTAAAGTTTTTATTAATAGATTTAGTATATTTTATTGAATGAGAAATT

GAGGATATCAACCTGAACGTTTACAGGCGGGGATTTATTTATTATTTTATACTTTACTGGCTTCGTTGCCGTTATTAATT

AATTATGAAAGTTACTTTAGTATATTTTTTTTAACTTTTTGTGTTTGTGAAGGGGTTCTAGGGCTGTCCATTTTAGTTTC

AATTATTATATTTATTTTTGGAAGTATTGTTTTTATTTCTAGTCGTAAGCATTTACTTTGTACTTTATTGAGATTAGAGT

AATTTTTATAGGAGGAATATTAGTTTTATTCATTTATGTAACTTCTCTTTCATCTAATGAAATATTTTCATTATCTATAAAACTATTTTTTTTATCTTTAAGTATAATT

AATTTTAACAGGATTATTCTTAGCAATACACTATACTGCTGATATTGAAACAGCTTTTAATAGAGTAAATCACATTTATCGTGATGTTAATAATGGTTGATTCCTACGAATT

CTGTTGATAATGCTACTTTAACTCGATTCTTTACATTTCATTTTATTCTCCCTTTCATTGTATTAGCTTTAACTATAATT

CTTTTGATTTTGCTGAAGGGGAGTCTGAGTTAGTTTCAGGATTTAATGTAGAATATAGAAGAGGGGGATTTGCTTTAATT

AATTTTTTTATATATCAGAAATATATATGATTTATTGTATTTTGTTTTCCTTTAGGTCTCGTTTGATTTGCATCTTGTTT

AATTTAAGCTATAAACTTTTTGTAATAAACTAGGATTAGATACCCTATTATTAAAAATAAATATTAAAATGCTAAAGTAG

>BrK18-1

AATTTTATCTACTTTATTATTAAAAAGAGGAGCAGCTCCTTTTCATTTTTGATTCCCAGGAGTTATAGAAGGATTAAATT

AATTAATCATATTGGTTGAATATTAATAGCTATAATAAATAACGAACTTTTATGATTAACTTATTTTTTATTATATTCAATT

CAACAAATCATAAAGATATTGGAACTTTATATTTCATTTTTGGAGTATGATCTGGAATAGTCGGAACTTCTCTAAGAATT

AATTGGATTATTAGGATTTATTGTTTGAGCTCATCATATATTTACAGTAGGTATAGACGTAGATACTCGAGCTTATTTTACTTCAGCAACTATAATT

CATGATACTTATTACGTAGTTGCCCATTTTCATTACGTTTTATCTATAGGAGCTGTATTTGCTATTATAGCAGGATTTATTCATTGATACCCTTTATTAACAGGAATAGTTATAAACCCTTCATG

CTGGAATACCTCGACGATACTCAGATTTTCCTGATAGCTACTTAACTTGAAATATTATTTCTTCTTTAGGAAGAACAATT

AATTTGTCACACACAACATATATTTGCTCATTTAGTTCCTCAAGGAACTCCTCCTGTTTTAATACCTTTTATAGTATGCATTGAAACTATTAGTAATGTAATCCGACCAGGAACTTTAGCAGTACGATTGACTGCTAATATAATT

GACGAGATATTTCTCGAGAAGGAACTTTTCAAGGACTTCACACTATTCCCGTAACATTAGGATTACGATGAGGAATAATTTTATTTATTATTTCTGAAGTTTTTTTCTTTATTTCCTTCTTTTGAGCTTTTTTCCATAGTAGCTTATCTCCAACAATCGAATT

AATTGTCATTTTTCTAGAAGTCATCATTTTGGATTCGAAGCAGCTGCTTGATATTGACATTTTGTTGATGTAGTTTGACTATTCTTATATATTTCAATT

AATTTTAGGAATATATTCTTATAAAATGTTTGATCAAGGATGAAGTGAATATTTTGGAGGTCAGATATTATATAATCAAT

ATTATTCAATAACAGGGGATTTTAATAGAACTACATTGAATATGTTAGATGATAAGGGGTGAACTATATCTTTTAGAATT

AATTCCTTTTTCTTCTTGATTACCTGCAGCTATGGCAGCACCGACTCCTGTTTCTGCTTTAGTACATTCTTCTACATTAG

AATTTATTTTCAAAATGTAAAATCTTATAATGCTGGTATATTAACAGCTTTATCTAATCGAATCGGGGATGTTGCTTTAT

CTTTACATTCAGTATCTATTGTAATAACTTTTTTATTTGATTGAATAAGTTTAATATTTATATCTTTTGTTTTATTAATT

AATTTTTATTAGATTTACTTTATTTATTTTAAGATTAAAGTTTTTATTAATAGATTTAGTATATTTTATTGAATGAGAAATT

GAGGATATCAACCTGAACGTTTACAGGCGGGGATTTATTTATTATTTTATACTTTACTGGCTTCGTTGCCGTTATTAATT

AATTATGAAAGTTACTTTAGTATATTTTTTTTAACTTTTTGTGTTTGTGAAGGGGTTCTAGGGCTGTCCATTTTAGTTTC

AATTATTATATTTATTTTTGGAAGTATTGTTTTTATTTCTAGTCGTAAGCATTTACTTTGTACTTTATTGAGATTAGAGT

AATTTTTATAGGAGGAATATTAGTTTTATTCATTTATGTAACTTCTCTTTCATCTAATGAAATATTTTCATTATCTATAAAACTATTTTTTTTATCTTTAAGTATAATT

AATTTTAACAGGATTATTCTTAGCAATACACTATACTGCTGATATTGAAACAGCTTTTAATAGAGTAAATCACATTTATCGTGATGTTAATAATGGTTGATTCCTACGAATT

CTGTTGATAATGCTACTTTAACTCGATTCTTTACATTTCATTTTATTCTCCCTTTCATTGTATTAGCTTTAACTATAATT

CTTTTGATTTTGCTGAAGGGGAGTCTGAGTTAGTTTCAGGATTTAATGTAGAATATAGAAGAGGGGGATTTGCTTTAATT

AATTTTTTTATATATCAGAAATATATATGATTTATTGTATTTTGTTTTCCTTTAGGTCTCGTTTGATTTGCATCTTGTTT

AATTTAAGCTATAAACTTTTTGTAATAAACTAGGATTAGATACCCTATTATTAAAAATAAATATTAAAATGCTAAAGTAG

>BrK18-2

AATTTTATCTACTTTATTATTAAAAAGAGGAGCAGCTCCTTTTCATTTTTGATTCCCAGGAGTTATAGAAGGATTAAATT

AATTAATCATATTGGTTGAATATTAATAGCTATAATAAATAACGAACTTTTATGATTAACTTATTTTTTATTATATTCAATT

CAACAAATCATAAAGATATTGGAACTTTATATTTCATTTTTGGAGTATGATCTGGAATAGTCGGAACTTCTCTAAGAATT

AATTGGATTATTAGGATTTATTGTTTGAGCTCATCATATATTTACAGTAGGTATAGACGTAGATACTCGAGCTTATTTTACTTCAGCAACTATAATT

CATGATACTTATTACGTAGTTGCCCATTTTCATTACGTTTTATCTATAGGAGCTGTATTTGCTATTATAGCAGGATTTATTCATTGATACCCTTTATTAACAGGAATAGTTATAAACCCTTCATG

CTGGAATACCTCGACGATACTCAGATTTTCCTGATAGCTACTTAACTTGAAATATTATTTCTTCTTTAGGAAGAACAATT

AATTTGTCACACACAACATATATTTGCTCATTTAGTTCCTCAAGGAACTCCTCCTGTTTTAATACCTTTTATAGTATGCATTGAAACTATTAGTAATGTAATCCGACCAGGAACTTTAGCAGTACGATTGACTGCTAATATAATT

GACGAGATATTTCTCGAGAAGGAACTTTTCAAGGACTTCACACTATTCCCGTAACATTAGGATTACGATGAGGAATAATTTTATTTATTATTTCTGAAGTTTTTTTCTTTATTTCCTTCTTTTGAGCTTTTTTCCATAGTAGCTTATCTCCAACAATCGAATT

AATTGTCATTTTTCTAGAAGTCATCATTTTGGATTCGAAGCAGCTGCTTGATATTGACATTTTGTTGATGTAGTTTGACTATTCTTATATATTTCAATT

AATTTTAGGAATATATTCTTATAAAATGTTTGATCAAGGATGAAGTGAATATTTTGGAGGTCAGATATTATATAATCAAT

ATTATTCAATAACAGGGGATTTTAATAGAACTACATTGAATATGTTAGATGATAAGGGGTGAACTATATCTTTTAGAATT

AATTCCTTTTTCTTCTTGATTACCTGCAGCTATGGCAGCACCGACTCCTGTTTCTGCTTTAGTACATTCTTCTACATTAG

AATTTATTTTCAAAATGTAAAATCTTATAATGCTGGTATATTAACAGCTTTATCTAATCGAATCGGGGATGTTGCTTTAT

CTTTACATTCAGTATCTATTGTAATAACTTTTTTATTTGATTGAATAAGTTTAATATTTATATCTTTTGTTTTATTAATT

AATTTTTATTAGATTTACTTTATTTATTTTAAGATTAAAGTTTTTATTAATAGATTTAGTATATTTTATTGAATGAGAAATT

GAGGATATCAACCTGAACGTTTACAGGCGGGGATTTATTTATTATTTTATACTTTACTGGCTTCGTTGCCGTTATTAATT

AATTATGAAAGTTACTTTAGTATATTTTTTTTAACTTTTTGTGTTTGTGAAGGGGTTCTAGGGCTGTCCATTTTAGTTTC

AATTATTATATTTATTTTTGGAAGTATTGTTTTTATTTCTAGTCGTAAGCATTTACTTTGTACTTTATTGAGATTAGAGT

AATTTTTATAGGAGGAATATTAGTTTTATTCATTTATGTAACTTCTCTTTCATCTAATGAAATATTTTCATTATCTATAAAACTATTTTTTTTATCTTTAAGTATAATT

AATTTTAACAGGATTATTCTTAGCAATACACTATACTGCTGATATTGAAACAGCTTTTAATAGAGTAAATCACATTTATCGTGATGTTAATAATGGTTGATTCCTACGAATT

CTGTTGATAATGCTACTTTAACTCGATTCTTTACATTTCATTTTATTCTCCCTTTCATTGTATTAGCTTTAACTATAATT

CTTTTGATTTTGCTGAAGGGGAGTCTGAGTTAGTTTCAGGATTTAATGTAGAATATAGAAGAGGGGGATTTGCTTTAATT

AATTTTTTTATATATCAGAAATATATATGATTTATTGTATTTTGTTTTCCTTTAGGTCTCGTTTGATTTGCATCTTGTTT

AATTTAAGCTATAAACTTTTTGTAATAAACTAGGATTAGATACCCTATTATTAAAAATAAATATTAAAATGCTAAAGTAG

>BrK21-1

AATTTTATCTACTTTATTATTAAAAAGAGGAGCAGCTCCTTTTCATTTTTGATTCCCAGGAGTTATAGAAGGATTAAATT

AATTAATCATATTGGTTGAATATTAATAGCTATAATAAATAACGAACTTTTATGATTAACTTATTTTTTATTATATTCAATT

CAACAAATCATAAAGATATTGGAACTTTATATTTCATTTTTGGAGTATGATCTGGAATAGTCGGAACTTCTCTAAGAATT

AATTGGATTATTAGGATTTATTGTTTGAGCTCATCATATATTTACAGTAGGTATAGACGTAGATACTCGAGCTTATTTTACTTCAGCAACTATAATT

CATGATACTTATTACGTAGTTGCCCATTTTCATTACGTTTTATCTATAGGAGCTGTATTTGCTATTATAGCAGGATTTATTCATTGATACCCTTTATTAACAGGAATAGTTATAAACCCTTCATG

CTGGAATACCTCGACGATACTCAGATTTTCCTGATAGCTACTTAACTTGAAATATTATTTCTTCTTTAGGAAGAACAATT

AATTTGTCACACACAACATATATTTGCTCATTTAGTTCCTCAAGGAACTCCTCCTGTTTTAATACCTTTTATAGTATGCATTGAAACTATTAGTAATGTAATCCGACCAGGAACTTTAGCAGTACGATTGACTGCTAATATAATT

GACGAGATATTTCTCGAGAAGGAACTTTTCAAGGACTTCACACTATTCCCGTAACATTAGGATTACGATGAGGAATAATTTTATTTATTATTTCTGAAGTTTTTTTCTTTATTTCCTTCTTTTGAGCTTTTTTCCATAGTAGCTTATCTCCAACAATCGAATT

AATTGTCATTTTTCTAGAAGTCATCATTTTGGATTCGAAGCAGCTGCTTGATATTGACATTTTGTTGATGTAGTTTGACTATTCTTATATATTTCAATT

AATTTTAGGAATATATTCTTATAAAATGTTTGATCAAGGATGAAGTGAATATTTTGGAGGTCAGATATTATATAATCAAT

ATTATTCAATAACAGGGGATTTTAATAGAACTACATTGAATATGTTAGATGATAAGGGGTGAACTATATCTTTTAGAATT

AATTCCTTTTTCTTCTTGATTACCTGCAGCTATGGCAGCACCGACTCCTGTTTCTGCTTTAGTACATTCTTCTACATTAG

AATTTATTTTCAAAATGTAAAATCTTATAATGCTGGTATATTAACAGCTTTATCTAATCGAATCGGGGATGTTGCTTTAT

CTTTACATTCAGTATCTATTGTAATAACTTTTTTATTTGATTGAATAAGTTTAATATTTATATCTTTTGTTTTATTAATT

AATTTTTATTAGATTTACTTTATTTATTTTAAGATTAAAGTTTTTATTAATAGATTTAGTATATTTTATTGAATGAGAAATT

GAGGATATCAACCTGAACGTTTACAGGCGGGGATTTATTTATTATTTTATACTTTACTGGCTTCGTTGCCGTTATTAATT

AATTATGAAAGTTACTTTAGTATATTTTTTTTAACTTTTTGTGTTTGTGAAGGGGTTCTAGGGCTGTCCATTTTAGTTTC

AATTATTATATTTATTTTTGGAAGTATTGTTTTTATTTCTAGTCGTAAGCATTTACTTTGTACTTTATTGAGATTAGAGT

AATTTTTATAGGAGGAATATTAGTTTTATTCATTTATGTAACTTCTCTTTCATCTAATGAAATATTTTCATTATCTATAAAACTATTTTTTTTATCTTTAAGTATAATT

AATTTTAACAGGATTATTCTTAGCAATACACTATACTGCTGATATTGAAACAGCTTTTAATAGAGTAAATCACATTTATCGTGATGTTAATAATGGTTGATTCCTACGAATT

CTGTTGATAATGCTACTTTAACTCGATTCTTTACATTTCATTTTATTCTCCCTTTCATTGTATTAGCTTTAACTATAATT

CTTTTGATTTTGCTGAAGGGGAGTCTGAGTTAGTTTCAGGATTTAATGTAGAATATAGAAGAGGGGGATTTGCTTTAATT

AATTTTTTTATATATCAGAAATATATATGATTTATTGTATTTTGTTTTCCTTTAGGTCTCGTTTGATTTGCATCTTGTTT

AATTTAAGCTATAAACTTTTTGTAATAAACTAGGATTAGATACCCTATTATTAAAAATAAATATTAAAATGCTAAAGTAG

>BrK21-2

AATTTTATCTACTTTATTATTAAAAAGAGGAGCAGCTCCTTTTCATTTTTGATTCCCAGGAGTTATAGAAGGATTAAATT

AATTAATCATATTGGTTGAATATTAATAGCTATAATAAATAACGAACTTTTATGATTAACTTATTTTTTATTATATTCAATT

CAACAAATCATAAAGATATTGGAACTTTATATTTCATTTTTGGAGTATGATCTGGAATAGTCGGAACTTCTCTAAGAATT

AATTGGATTATTAGGATTTATTGTTTGAGCTCATCATATATTTACAGTAGGTATAGACGTAGATACTCGAGCTTATTTTACTTCAGCAACTATAATT

CATGATACTTATTACGTAGTTGCCCATTTTCATTACGTTTTATCTATAGGAGCTGTATTTGCTATTATAGCAGGATTTATTCATTGATACCCTTTATTAACAGGAATAGTTATAAACCCTTCATG

CTGGAATACCTCGACGATACTCAGATTTTCCTGATAGCTACTTAACTTGAAATATTATTTCTTCTTTAGGAAGAACAATT

AATTTGTCACACACAACATATATTTGCTCATTTAGTTCCTCAAGGAACTCCTCCTGTTTTAATACCTTTTATAGTATGCATTGAAACTATTAGTAATGTAATCCGACCAGGAACTTTAGCAGTACGATTGACTGCTAATATAATT

GACGAGATATTTCTCGAGAAGGAACTTTTCAAGGACTTCACACTATTCCCGTAACATTAGGATTACGATGAGGAATAATTTTATTTATTATTTCTGAAGTTTTTTTCTTTATTTCCTTCTTTTGAGCTTTTTTCCATAGTAGCTTATCTCCAACAATCGAATT

AATTGTCATTTTTCTAGAAGTCATCATTTTGGATTCGAAGCAGCTGCTTGATATTGACATTTTGTTGATGTAGTTTGACTATTCTTATATATTTCAATT

AATTTTAGGAATATATTCTTATAAAATGTTTGATCAAGGATGAAGTGAATATTTTGGAGGTCAGATATTATATAATCAAT

ATTATTCAATAACAGGGGATTTTAATAGAACTACATTGAATATGTTAGATGATAAGGGGTGAACTATATCTTTTAGAATT

AATTCCTTTTTCTTCTTGATTACCTGCAGCTATGGCAGCACCGACTCCTGTTTCTGCTTTAGTACATTCTTCTACATTAG

AATTTATTTTCAAAATGTAAAATCTTATAATGCTGGTATATTAACAGCTTTATCTAATCGAATCGGGGATGTTGCTTTAT

CTTTACATTCAGTATCTATTGTAATAACTTTTTTATTTGATTGAATAAGTTTAATATTTATATCTTTTGTTTTATTAATT

AATTTTTATTAGATTTACTTTATTTATTTTAAGATTAAAGTTTTTATTAATAGATTTAGTATATTTTATTGAATGAGAAATT

GAGGATATCAACCTGAACGTTTACAGGCGGGGATTTATTTATTATTTTATACTTTACTGGCTTCGTTGCCGTTATTAATT

AATTATGAAAGTTACTTTAGTATATTTTTTTTAACTTTTTGTGTTTGTGAAGGGGTTCTAGGGCTGTCCATTTTAGTTTC

AATTATTATATTTATTTTTGGAAGTATTGTTTTTATTTCTAGTCGTAAGCATTTACTTTGTACTTTATTGAGATTAGAGT

AATTTTTATAGGAGGAATATTAGTTTTATTCATTTATGTAACTTCTCTTTCATCTAATGAAATATTTTCATTATCTATAAAACTATTTTTTTTATCTTTAAGTATAATT

AATTTTAACAGGATTATTCTTAGCAATACACTATACTGCTGATATTGAAACAGCTTTTAATAGAGTAAATCACATTTATCGTGATGTTAATAATGGTTGATTCCTACGAATT

CTGTTGATAATGCTACTTTAACTCGATTCTTTACATTTCATTTTATTCTCCCTTTCATTGTATTAGCTTTAACTATAATT

CTTTTGATTTTGCTGAAGGGGAGTCTGAGTTAGTTTCAGGATTTAATGTAGAATATAGAAGAGGGGGATTTGCTTTAATT

AATTTTTTTATATATCAGAAATATATATGATTTATTGTATTTTGTTTTCCTTTAGGTCTCGTTTGATTTGCATCTTGTTT

AATTTAAGCTATAAACTTTTTGTAATAAACTAGGATTAGATACCCTATTATTAAAAATAAATATTAAAATGCTAAAGTAG

>BrK21-3

AATTTTATCTACTTTATTATTAAAAAGAGGAGCAGCTCCTTTTCATTTTTGATTCCCAGGAGTTATAGAAGGATTAAATT

AATTAATCATATTGGTTGAATATTAATAGCTATAATAAATAACGAACTTTTATGATTAACTTATTTTTTATTATATTCAATT

CAACAAATCATAAAGATATTGGAACTTTATATTTCATTTTTGGAGTATGATCTGGAATAGTCGGAACTTCTCTAAGAATT

AATTGGATTATTAGGATTTATTGTTTGAGCTCATCATATATTTACAGTAGGTATAGACGTAGATACTCGAGCTTATTTTACTTCAGCAACTATAATT

CATGATACTTATTACGTAGTTGCCCATTTTCATTACGTTTTATCTATAGGAGCTGTATTTGCTATTATAGCAGGATTTATTCATTGATACCCTTTATTAACAGGAATAGTTATAAACCCTTCATG

CTGGAATACCTCGACGATACTCAGATTTTCCTGATAGCTACTTAACTTGAAATATTATTTCTTCTTTAGGAAGAACAATT

AATTTGTCACACACAACATATATTTGCTCATTTAGTTCCTCAAGGAACTCCTCCTGTTTTAATACCTTTTATAGTATGCATTGAAACTATTAGTAATGTAATCCGACCAGGAACTTTAGCAGTACGATTGACTGCTAATATAATT

GACGAGATATTTCTCGAGAAGGAACTTTTCAAGGACTTCACACTATTCCCGTAACATTAGGATTACGATGAGGAATAATTTTATTTATTATTTCTGAAGTTTTTTTCTTTATTTCCTTCTTTTGAGCTTTTTTCCATAGTAGCTTATCTCCAACAATCGAATT

AATTGTCATTTTTCTAGAAGTCATCATTTTGGATTCGAAGCAGCTGCTTGATATTGACATTTTGTTGATGTAGTTTGACTATTCTTATATATTTCAATT

AATTTTAGGAATATATTCTTATAAAATGTTTGATCAAGGATGAAGTGAATATTTTGGAGGTCAGATATTATATAATCAAT

ATTATTCAATAACAGGGGATTTTAATAGAACTACATTGAATATGTTAGATGATAAGGGGTGAACTATATCTTTTAGAATT

AATTCCTTTTTCTTCTTGATTACCTGCAGCTATGGCAGCACCGACTCCTGTTTCTGCTTTAGTACATTCTTCTACATTAG

AATTTATTTTCAAAATGTAAAATCTTATAATGCTGGTATATTAACAGCTTTATCTAATCGAATCGGGGATGTTGCTTTAT

CTTTACATTCAGTATCTATTGTAATAACTTTTTTATTTGATTGAATAAGTTTAATATTTATATCTTTTGTTTTATTAATT

AATTTTTATTAGATTTACTTTATTTATTTTAAGATTAAAGTTTTTATTAATAGATTTAGTATATTTTATTGAATGAGAAATT

GAGGATATCAACCTGAACGTTTACAGGCGGGGATTTATTTATTATTTTATACTTTACTGGCTTCGTTGCCGTTATTAATT

AATTATGAAAGTTACTTTAGTATATTTTTTTTAACTTTTTGTGTTTGTGAAGGGGTTCTAGGGCTGTCCATTTTAGTTTC

AATTATTATATTTATTTTTGGAAGTATTGTTTTTATTTCTAGTCGTAAGCATTTACTTTGTACTTTATTGAGATTAGAGT

AATTTTTATAGGAGGAATATTAGTTTTATTCATTTATGTAACTTCTCTTTCATCTAATGAAATATTTTCATTATCTATAAAACTATTTTTTTTATCTTTAAGTATAATT

AATTTTAACAGGATTATTCTTAGCAATACACTATACTGCTGATATTGAAACAGCTTTTAATAGAGTAAATCACATTTATCGTGATGTTAATAATGGTTGATTCCTACGAATT

CTGTTGATAATGCTACTTTAACTCGATTCTTTACATTTCATTTTATTCTCCCTTTCATTGTATTAGCTTTAACTATAATT

CTTTTGATTTTGCTGAAGGGGAGTCTGAGTTAGTTTCAGGATTTAATGTAGAATATAGAAGAGGGGGATTTGCTTTAATT

AATTTTTTTATATATCAGAAATATATATGATTTATTGTATTTTGTTTTCCTTTAGGTCTCGTTTGATTTGCATCTTGTTT

AATTTAAGCTATAAACTTTTTGTAATAAACTAGGATTAGATACCCTATTATTAAAAATAAATATTAAAATGCTAAAGTAG

>BrK23-1

AATTTTATCTACTTTATTATTAAAAAGAGGAGCAGCTCCTTTTCATTTTTGATTCCCAGGAGTTATAGAAGGATTAAATT

AATTAATCATATTGGTTGAATATTAATAGCTATAATAAATAACGAACTTTTATGATTAACTTATTTTTTATTATATTCAATT

CAACAAATCATAAAGATATTGGAACTTTATATTTCATTTTTGGAGTATGATCTGGAATAGTCGGAACTTCTCTAAGAATT

AATTGGATTATTAGGATTTATTGTTTGAGCTCATCATATATTTACAGTAGGTATAGACGTAGATACTCGAGCTTATTTTACTTCAGCAACTATAATT

CATGATACTTATTACGTAGTTGCCCATTTTCATTACGTTTTATCTATAGGAGCTGTATTTGCTATTATAGCAGGATTTATTCATTGATACCCTTTATTAACAGGAATAGTTATAAACCCTTCATG

CTGGAATACCTCGACGATACTCAGATTTTCCTGATAGCTACTTAACTTGAAATATTATTTCTTCTTTAGGAAGAACAATT

AATTTGTCACACACAACATATATTTGCTCATTTAGTTCCTCAAGGAACTCCTCCTGTTTTAATACCTTTTATAGTATGCATTGAAACTATTAGTAATGTAATCCGACCAGGAACTTTAGCAGTACGATTGACTGCTAATATAATT

GACGAGATATTTCTCGAGAAGGAACTTTTCAAGGACTTCACACTATTCCCGTAACATTAGGATTACGATGAGGAATAATTTTATTTATTATTTCTGAAGTTTTTTTCTTTATTTCCTTCTTTTGAGCTTTTTTCCATAGTAGCTTATCTCCAACAATCGAATT

AATTGTCATTTTTCTAGAAGTCATCATTTTGGATTCGAAGCAGCTGCTTGATATTGACATTTTGTTGATGTAGTTTGACTATTCTTATATATTTCAATT

AATTTTAGGAATATATTCTTATAAAATGTTTGATCAAGGATGAAGTGAATATTTTGGAGGTCAGATATTATATAATCAAT

ATTATTCAATAACAGGGGATTTTAATAGAACTACATTGAATATGTTAGATGATAAGGGGTGAACTATATCTTTTAGAATT

AATTCCTTTTTCTTCTTGATTACCTGCAGCTATGGCAGCACCGACTCCTGTTTCTGCTTTAGTACATTCTTCTACATTAG

AATTTATTTTCAAAATGTAAAATCTTATAATGCTGGTATATTAACAGCTTTATCTAATCGAATCGGGGATGTTGCTTTAT

CTTTACATTCAGTATCTATTGTAATAACTTTTTTATTTGATTGAATAAGTTTAATATTTATATCTTTTGTTTTATTAATT

AATTTTTATTAGATTTACTTTATTTATTTTAAGATTAAAGTTTTTATTAATAGATTTAGTATATTTTATTGAATGAGAAATT

GAGGATATCAACCTGAACGTTTACAGGCGGGGATTTATTTATTATTTTATACTTTACTGGCTTCGTTGCCGTTATTAATT

AATTATGAAAGTTACTTTAGTATATTTTTTTTAACTTTTTGTGTTTGTGAAGGGGTTCTAGGGCTGTCCATTTTAGTTTC

AATTATTATATTTATTTTTGGAAGTATTGTTTTTATTTCTAGTCGTAAGCATTTACTTTGTACTTTATTGAGATTAGAGT

AATTTTTATAGGAGGAATATTAGTTTTATTCATTTATGTAACTTCTCTTTCATCTAATGAAATATTTTCATTATCTATAAAACTATTTTTTTTATCTTTAAGTATAATT

AATTTTAACAGGATTATTCTTAGCAATACACTATACTGCTGATATTGAAACAGCTTTTAATAGAGTAAATCACATTTATCGTGATGTTAATAATGGTTGATTCCTACGAATT

CTGTTGATAATGCTACTTTAACTCGATTCTTTACATTTCATTTTATTCTCCCTTTCATTGTATTAGCTTTAACTATAATT

CTTTTGATTTTGCTGAAGGGGAGTCTGAGTTAGTTTCAGGATTTAATGTAGAATATAGAAGAGGGGGATTTGCTTTAATT

AATTTTTTTATATATCAGAAATATATATGATTTATTGTATTTTGTTTTCCTTTAGGTCTCGTTTGATTTGCATCTTGTTT

AATTTAAGCTATAAACTTTTTGTAATAAACTAGGATTAGATACCCTATTATTAAAAATAAATATTAAAATGCTAAAGTAG

>BrK24-1

AATTTTATCTACTTTATTATTAAAAAGAGGAGCAGCTCCTTTTCATTTTTGATTCCCAGGAGTTATAGAAGGATTAAATT

AATTAATCATATTGGTTGAATATTAATAGCTATAATAAATAACGAACTTTTATGATTAACTTATTTTTTATTATATTCAATT

CAACAAATCATAAAGATATTGGAACTTTATATTTCATTTTTGGAGTATGATCTGGAATAGTCGGAACTTCTCTAAGAATT

AATTGGATTATTAGGATTTATTGTTTGAGCTCATCATATATTTACAGTAGGTATAGACGTAGATACTCGAGCTTATTTTACTTCAGCAACTATAATT

CATGATACTTATTACGTAGTTGCCCATTTTCATTACGTTTTATCTATAGGAGCTGTATTTGCTATTATAGCAGGATTTATTCATTGATACCCTTTATTAACAGGAATAGTTATAAACCCTTCATG

CTGGAATACCTCGACGATACTCAGATTTTCCTGATAGCTACTTAACTTGAAATATTATTTCTTCTTTAGGAAGAACAATT

AATTTGTCACACACAACATATATTTGCTCATTTAGTTCCTCAAGGAACTCCTCCTGTTTTAATACCTTTTATAGTATGCATTGAAACTATTAGTAATGTAATCCGACCAGGAACTTTAGCAGTACGATTGACTGCTAATATAATT

GACGAGATATTTCTCGAGAAGGAACTTTTCAAGGACTTCACACTATTCCCGTAACATTAGGATTACGATGAGGAATAATTTTATTTATTATTTCTGAAGTTTTTTTCTTTATTTCCTTCTTTTGAGCTTTTTTCCATAGTAGCTTATCTCCAACAATCGAATT

AATTGTCATTTTTCTAGAAGTCATCATTTTGGATTCGAAGCAGCTGCTTGATATTGACATTTTGTTGATGTAGTTTGACTATTCTTATATATTTCAATT

AATTTTAGGAATATATTCTTATAAAATGTTTGATCAAGGATGAAGTGAATATTTTGGAGGTCAGATATTATATAATCAAT

ATTATTCAATAACAGGGGATTTTAATAGAACTACATTGAATATGTTAGATGATAAGGGGTGAACTATATCTTTTAGAATT

AATTCCTTTTTCTTCTTGATTACCTGCAGCTATGGCAGCACCGACTCCTGTTTCTGCTTTAGTACATTCTTCTACATTAG

AATTTATTTTCAAAATGTAAAATCTTATAATGCTGGTATATTAACAGCTTTATCTAATCGAATCGGGGATGTTGCTTTAT

CTTTACATTCAGTATCTATTGTAATAACTTTTTTATTTGATTGAATAAGTTTAATATTTATATCTTTTGTTTTATTAATT

AATTTTTATTAGATTTACTTTATTTATTTTAAGATTAAAGTTTTTATTAATAGATTTAGTATATTTTATTGAATGAGAAATT

GAGGATATCAACCTGAACGTTTACAGGCGGGGATTTATTTATTATTTTATACTTTACTGGCTTCGTTGCCGTTATTAATT

AATTATGAAAGTTACTTTAGTATATTTTTTTTAACTTTTTGTGTTTGTGAAGGGGTTCTAGGGCTGTCCATTTTAGTTTC

AATTATTATATTTATTTTTGGAAGTATTGTTTTTATTTCTAGTCGTAAGCATTTACTTTGTACTTTATTGAGATTAGAGT

AATTTTTATAGGAGGAATATTAGTTTTATTCATTTATGTAACTTCTCTTTCATCTAATGAAATATTTTCATTATCTATAAAACTATTTTTTTTATCTTTAAGTATAATT

AATTTTAACAGGATTATTCTTAGCAATACACTATACTGCTGATATTGAAACAGCTTTTAATAGAGTAAATCACATTTATCGTGATGTTAATAATGGTTGATTCCTACGAATT

CTGTTGATAATGCTACTTTAACTCGATTCTTTACATTTCATTTTATTCTCCCTTTCATTGTATTAGCTTTAACTATAATT

CTTTTGATTTTGCTGAAGGGGAGTCTGAGTTAGTTTCAGGATTTAATGTAGAATATAGAAGAGGGGGATTTGCTTTAATT

AATTTTTTTATATATCAGAAATATATATGATTTATTGTATTTTGTTTTCCTTTAGGTCTCGTTTGATTTGCATCTTGTTT

AATTTAAGCTATAAACTTTTTGTAATAAACTAGGATTAGATACCCTATTATTAAAAATAAATATTAAAATGCTAAAGTAG

>BrK24-2

AATTTTATCTACTTTATTATTAAAAAGAGGAGCAGCTCCTTTTCATTTTTGATTCCCAGGAGTTATAGAAGGATTAAATT

AATTAATCATATTGGTTGAATATTAATAGCTATAATAAATAACGAACTTTTATGATTAACTTATTTTTTATTATATTCAATT

CAACAAATCATAAAGATATTGGAACTTTATATTTCATTTTTGGAGTATGATCTGGAATAGTCGGAACTTCTCTAAGAATT

AATTGGATTATTAGGATTTATTGTTTGAGCTCATCATATATTTACAGTAGGTATAGACGTAGATACTCGAGCTTATTTTACTTCAGCAACTATAATT

CATGATACTTATTACGTAGTTGCCCATTTTCATTACGTTTTATCTATAGGAGCTGTATTTGCTATTATAGCAGGATTTATTCATTGATACCCTTTATTAACAGGAATAGTTATAAACCCTTCATG

CTGGAATACCTCGACGATACTCAGATTTTCCTGATAGCTACTTAACTTGAAATATTATTTCTTCTTTAGGAAGAACAATT

AATTTGTCACACACAACATATATTTGCTCATTTAGTTCCTCAAGGAACTCCTCCTGTTTTAATACCTTTTATAGTATGCATTGAAACTATTAGTAATGTAATCCGACCAGGAACTTTAGCAGTACGATTGACTGCTAATATAATT

GACGAGATATTTCTCGAGAAGGAACTTTTCAAGGACTTCACACTATTCCCGTAACATTAGGATTACGATGAGGAATAATTTTATTTATTATTTCTGAAGTTTTTTTCTTTATTTCCTTCTTTTGAGCTTTTTTCCATAGTAGCTTATCTCCAACAATCGAATT

AATTGTCATTTTTCTAGAAGTCATCATTTTGGATTCGAAGCAGCTGCTTGATATTGACATTTTGTTGATGTAGTTTGACTATTCTTATATATTTCAATT

AATTTTAGGAATATATTCTTATAAAATGTTTGATCAAGGATGAAGTGAATATTTTGGAGGTCAGATATTATATAATCAAT

ATTATTCAATAACAGGGGATTTTAATAGAACTACATTGAATATGTTAGATGATAAGGGGTGAACTATATCTTTTAGAATT

AATTCCTTTTTCTTCTTGATTACCTGCAGCTATGGCAGCACCGACTCCTGTTTCTGCTTTAGTACATTCTTCTACATTAG

AATTTATTTTCAAAATGTAAAATCTTATAATGCTGGTATATTAACAGCTTTATCTAATCGAATCGGGGATGTTGCTTTAT

CTTTACATTCAGTATCTATTGTAATAACTTTTTTATTTGATTGAATAAGTTTAATATTTATATCTTTTGTTTTATTAATT

AATTTTTATTAGATTTACTTTATTTATTTTAAGATTAAAGTTTTTATTAATAGATTTAGTATATTTTATTGAATGAGAAATT

GAGGATATCAACCTGAACGTTTACAGGCGGGGATTTATTTATTATTTTATACTTTACTGGCTTCGTTGCCGTTATTAATT

AATTATGAAAGTTACTTTAGTATATTTTTTTTAACTTTTTGTGTTTGTGAAGGGGTTCTAGGGCTGTCCATTTTAGTTTC

AATTATTATATTTATTTTTGGAAGTATTGTTTTTATTTCTAGTCGTAAGCATTTACTTTGTACTTTATTGAGATTAGAGT

AATTTTTATAGGAGGAATATTAGTTTTATTCATTTATGTAACTTCTCTTTCATCTAATGAAATATTTTCATTATCTATAAAACTATTTTTTTTATCTTTAAGTATAATT

AATTTTAACAGGATTATTCTTAGCAATACACTATACTGCTGATATTGAAACAGCTTTTAATAGAGTAAATCACATTTATCGTGATGTTAATAATGGTTGATTCCTACGAATT

CTGTTGATAATGCTACTTTAACTCGATTCTTTACATTTCATTTTATTCTCCCTTTCATTGTATTAGCTTTAACTATAATT

CTTTTGATTTTGCTGAAGGGGAGTCTGAGTTAGTTTCAGGATTTAATGTAGAATATAGAAGAGGGGGATTTGCTTTAATT

AATTTTTTTATATATCAGAAATATATATGATTTATTGTATTTTGTTTTCCTTTAGGTCTCGTTTGATTTGCATCTTGTTT

AATTTAAGCTATAAACTTTTTGTAATAAACTAGGATTAGATACCCTATTATTAAAAATAAATATTAAAATGCTAAAGTAG

>BrK25-1

AATTTTATCTACTTTATTATTAAAAAGAGGAGCAGCTCCTTTTCATTTTTGATTCCCAGGAGTTATAGAAGGATTAAATT

AATTAATCATATTGGTTGAATATTAATAGCTATAATAAATAACGAACTTTTATGATTAACTTATTTTTTATTATATTCAATT

CAACAAATCATAAAGATATTGGAACTTTATATTTCATTTTTGGAGTATGATCTGGAATAGTCGGAACTTCTCTAAGAATT

AATTGGATTATTAGGATTTATTGTTTGAGCTCATCATATATTTACAGTAGGTATAGACGTAGATACTCGAGCTTATTTTACTTCAGCAACTATAATT

CATGATACTTATTACGTAGTTGCCCATTTTCATTACGTTTTATCTATAGGAGCTGTATTTGCTATTATAGCAGGATTTATTCATTGATACCCTTTATTAACAGGAATAGTTATAAACCCTTCATG

CTGGAATACCTCGACGATACTCAGATTTTCCTGATAGCTACTTAACTTGAAATATTATTTCTTCTTTAGGAAGAACAATT

AATTTGTCACACACAACATATATTTGCTCATTTAGTTCCTCAAGGAACTCCTCCTGTTTTAATACCTTTTATAGTATGCATTGAAACTATTAGTAATGTAATCCGACCAGGAACTTTAGCAGTACGATTGACTGCTAATATAATT

GACGAGATATTTCTCGAGAAGGAACTTTTCAAGGACTTCACACTATTCCCGTAACATTAGGATTACGATGAGGAATAATTTTATTTATTATTTCTGAAGTTTTTTTCTTTATTTCCTTCTTTTGAGCTTTTTTCCATAGTAGCTTATCTCCAACAATCGAATT

AATTGTCATTTTTCTAGAAGTCATCATTTTGGATTCGAAGCAGCTGCTTGATATTGACATTTTGTTGATGTAGTTTGACTATTCTTATATATTTCAATT

AATTTTAGGAATATATTCTTATAAAATGTTTGATCAAGGATGAAGTGAATATTTTGGAGGTCAGATATTATATAATCAAT

ATTATTCAATAACAGGGGATTTTAATAGAACTACATTGAATATGTTAGATGATAAGGGGTGAACTATATCTTTTAGAATT

AATTCCTTTTTCTTCTTGATTACCTGCAGCTATGGCAGCACCGACTCCTGTTTCTGCTTTAGTACATTCTTCTACATTAG

AATTTATTTTCAAAATGTAAAATCTTATAATGCTGGTATATTAACAGCTTTATCTAATCGAATCGGGGATGTTGCTTTAT

CTTTACATTCAGTATCTATTGTAATAACTTTTTTATTTGATTGAATAAGTTTAATATTTATATCTTTTGTTTTATTAATT

AATTTTTATTAGATTTACTTTATTTATTTTAAGATTAAAGTTTTTATTAATAGATTTAGTATATTTTATTGAATGAGAAATT

GAGGATATCAACCTGAACGTTTACAGGCGGGGATTTATTTATTATTTTATACTTTACTGGCTTCGTTGCCGTTATTAATT

AATTATGAAAGTTACTTTAGTATATTTTTTTTAACTTTTTGTGTTTGTGAAGGGGTTCTAGGGCTGTCCATTTTAGTTTC

AATTATTATATTTATTTTTGGAAGTATTGTTTTTATTTCTAGTCGTAAGCATTTACTTTGTACTTTATTGAGATTAGAGT

AATTTTTATAGGAGGAATATTAGTTTTATTCATTTATGTAACTTCTCTTTCATCTAATGAAATATTTTCATTATCTATAAAACTATTTTTTTTATCTTTAAGTATAATT

AATTTTAACAGGATTATTCTTAGCAATACACTATACTGCTGATATTGAAACAGCTTTTAATAGAGTAAATCACATTTATCGTGATGTTAATAATGGTTGATTCCTACGAATT

CTGTTGATAATGCTACTTTAACTCGATTCTTTACATTTCATTTTATTCTCCCTTTCATTGTATTAGCTTTAACTATAATT

CTTTTGATTTTGCTGAAGGGGAGTCTGAGTTAGTTTCAGGATTTAATGTAGAATATAGAAGAGGGGGATTTGCTTTAATT

AATTTTTTTATATATCAGAAATATATATGATTTATTGTATTTTGTTTTCCTTTAGGTCTCGTTTGATTTGCATCTTGTTT

AATTTAAGCTATAAACTTTTTGTAATAAACTAGGATTAGATACCCTATTATTAAAAATAAATATTAAAATGCTAAAGTAG

>BrK25-2

AATTTTATCTACTTTATTATTAAAAAGAGGAGCAGCTCCTTTTCATTTTTGATTCCCAGGAGTTATAGAAGGATTAAATT

AATTAATCATATTGGTTGAATATTAATAGCTATAATAAATAACGAACTTTTATGATTAACTTATTTTTTATTATATTCAATT

CAACAAATCATAAAGATATTGGAACTTTATATTTCATTTTTGGAGTATGATCTGGAATAGTCGGAACTTCTCTAAGAATT

AATTGGATTATTAGGATTTATTGTTTGAGCTCATCATATATTTACAGTAGGTATAGACGTAGATACTCGAGCTTATTTTACTTCAGCAACTATAATT

CATGATACTTATTACGTAGTTGCCCATTTTCATTACGTTTTATCTATAGGAGCTGTATTTGCTATTATAGCAGGATTTATTCATTGATACCCTTTATTAACAGGAATAGTTATAAACCCTTCATG

CTGGAATACCTCGACGATACTCAGATTTTCCTGATAGCTACTTAACTTGAAATATTATTTCTTCTTTAGGAAGAACAATT

AATTTGTCACACACAACATATATTTGCTCATTTAGTTCCTCAAGGAACTCCTCCTGTTTTAATACCTTTTATAGTATGCATTGAAACTATTAGTAATGTAATCCGACCAGGAACTTTAGCAGTACGATTGACTGCTAATATAATT

GACGAGATATTTCTCGAGAAGGAACTTTTCAAGGACTTCACACTATTCCCGTAACATTAGGATTACGATGAGGAATAATTTTATTTATTATTTCTGAAGTTTTTTTCTTTATTTCCTTCTTTTGAGCTTTTTTCCATAGTAGCTTATCTCCAACAATCGAATT

AATTGTCATTTTTCTAGAAGTCATCATTTTGGATTCGAAGCAGCTGCTTGATATTGACATTTTGTTGATGTAGTTTGACTATTCTTATATATTTCAATT

AATTTTAGGAATATATTCTTATAAAATGTTTGATCAAGGATGAAGTGAATATTTTGGAGGTCAGATATTATATAATCAAT

ATTATTCAATAACAGGGGATTTTAATAGAACTACATTGAATATGTTAGATGATAAGGGGTGAACTATATCTTTTAGAATT

AATTCCTTTTTCTTCTTGATTACCTGCAGCTATGGCAGCACCGACTCCTGTTTCTGCTTTAGTACATTCTTCTACATTAG

AATTTATTTTCAAAATGTAAAATCTTATAATGCTGGTATATTAACAGCTTTATCTAATCGAATCGGGGATGTTGCTTTAT

CTTTACATTCAGTATCTATTGTAATAACTTTTTTATTTGATTGAATAAGTTTAATATTTATATCTTTTGTTTTATTAATT

AATTTTTATTAGATTTACTTTATTTATTTTAAGATTAAAGTTTTTATTAATAGATTTAGTATATTTTATTGAATGAGAAATT

GAGGATATCAACCTGAACGTTTACAGGCGGGGATTTATTTATTATTTTATACTTTACTGGCTTCGTTGCCGTTATTAATT

AATTATGAAAGTTACTTTAGTATATTTTTTTTAACTTTTTGTGTTTGTGAAGGGGTTCTAGGGCTGTCCATTTTAGTTTC

AATTATTATATTTATTTTTGGAAGTATTGTTTTTATTTCTAGTCGTAAGCATTTACTTTGTACTTTATTGAGATTAGAGT

AATTTTTATAGGAGGAATATTAGTTTTATTCATTTATGTAACTTCTCTTTCATCTAATGAAATATTTTCATTATCTATAAAACTATTTTTTTTATCTTTAAGTATAATT

AATTTTAACAGGATTATTCTTAGCAATACACTATACTGCTGATATTGAAACAGCTTTTAATAGAGTAAATCACATTTATCGTGATGTTAATAATGGTTGATTCCTACGAATT

CTGTTGATAATGCTACTTTAACTCGATTCTTTACATTTCATTTTATTCTCCCTTTCATTGTATTAGCTTTAACTATAATT

CTTTTGATTTTGCTGAAGGGGAGTCTGAGTTAGTTTCAGGATTTAATGTAGAATATAGAAGAGGGGGATTTGCTTTAATT

AATTTTTTTATATATCAGAAATATATATGATTTATTGTATTTTGTTTTCCTTTAGGTCTCGTTTGATTTGCATCTTGTTT

AATTTAAGCTATAAACTTTTTGTAATAAACTAGGATTAGATACCCTATTATTAAAAATAAATATTAAAATGCTAAAGTAG

>BrK26-1

AATTTTATCTACTTTATTATTAAAAAGAGGAGCAGCTCCTTTTCATTTTTGATTCCCAGGAGTTATAGAAGGATTAAATT

AATTAATCATATTGGTTGAATATTAATAGCTATAATAAATAACGAACTTTTATGATTAACTTATTTTTTATTATATTCAATT

CAACAAATCATAAAGATATTGGAACTTTATATTTCATTTTTGGAGTATGATCTGGAATAGTCGGAACTTCTCTAAGAATT

AATTGGATTATTAGGATTTATTGTTTGAGCTCATCATATATTTACAGTAGGTATAGACGTAGATACTCGAGCTTATTTTACTTCAGCAACTATAATT

CATGATACTTATTACGTAGTTGCCCATTTTCATTACGTTTTATCTATAGGAGCTGTATTTGCTATTATAGCAGGATTTATTCATTGATACCCTTTATTAACAGGAATAGTTATAAACCCTTCATG

CTGGAATACCTCGACGATACTCAGATTTTCCTGATAGCTACTTAACTTGAAATATTATTTCTTCTTTAGGAAGAACAATT

AATTTGTCACACACAACATATATTTGCTCATTTAGTTCCTCAAGGAACTCCTCCTGTTTTAATACCTTTTATAGTATGCATTGAAACTATTAGTAATGTAATCCGACCAGGAACTTTAGCAGTACGATTGACTGCTAATATAATT

GACGAGATATTTCTCGAGAAGGAACTTTTCAAGGACTTCACACTATTCCCGTAACATTAGGATTACGATGAGGAATAATTTTATTTATTATTTCTGAAGTTTTTTTCTTTATTTCCTTCTTTTGAGCTTTTTTCCATAGTAGCTTATCTCCAACAATCGAATT

AATTGTCATTTTTCTAGAAGTCATCATTTTGGATTCGAAGCAGCTGCTTGATATTGACATTTTGTTGATGTAGTTTGACTATTCTTATATATTTCAATT

AATTTTAGGAATATATTCTTATAAAATGTTTGATCAAGGATGAAGTGAATATTTTGGAGGTCAGATATTATATAATCAAT

ATTATTCAATAACAGGGGATTTTAATAGAACTACATTGAATATGTTAGATGATAAGGGGTGAACTATATCTTTTAGAATT

AATTCCTTTTTCTTCTTGATTACCTGCAGCTATGGCAGCACCGACTCCTGTTTCTGCTTTAGTACATTCTTCTACATTAG

AATTTATTTTCAAAATGTAAAATCTTATAATGCTGGTATATTAACAGCTTTATCTAATCGAATCGGGGATGTTGCTTTAT

CTTTACATTCAGTATCTATTGTAATAACTTTTTTATTTGATTGAATAAGTTTAATATTTATATCTTTTGTTTTATTAATT

AATTTTTATTAGATTTACTTTATTTATTTTAAGATTAAAGTTTTTATTAATAGATTTAGTATATTTTATTGAATGAGAAATT

GAGGATATCAACCTGAACGTTTACAGGCGGGGATTTATTTATTATTTTATACTTTACTGGCTTCGTTGCCGTTATTAATT

AATTATGAAAGTTACTTTAGTATATTTTTTTTAACTTTTTGTGTTTGTGAAGGGGTTCTAGGGCTGTCCATTTTAGTTTC

AATTATTATATTTATTTTTGGAAGTATTGTTTTTATTTCTAGTCGTAAGCATTTACTTTGTACTTTATTGAGATTAGAGT

AATTTTTATAGGAGGAATATTAGTTTTATTCATTTATGTAACTTCTCTTTCATCTAATGAAATATTTTCATTATCTATAAAACTATTTTTTTTATCTTTAAGTATAATT

AATTTTAACAGGATTATTCTTAGCAATACACTATACTGCTGATATTGAAACAGCTTTTAATAGAGTAAATCACATTTATCGTGATGTTAATAATGGTTGATTCCTACGAATT

CTGTTGATAATGCTACTTTAACTCGATTCTTTACATTTCATTTTATTCTCCCTTTCATTGTATTAGCTTTAACTATAATT

CTTTTGATTTTGCTGAAGGGGAGTCTGAGTTAGTTTCAGGATTTAATGTAGAATATAGAAGAGGGGGATTTGCTTTAATT

AATTTTTTTATATATCAGAAATATATATGATTTATTGTATTTTGTTTTCCTTTAGGTCTCGTTTGATTTGCATCTTGTTT

AATTTAAGCTATAAACTTTTTGTAATAAACTAGGATTAGATACCCTATTATTAAAAATAAATATTAAAATGCTAAAGTAG

>BrK26-2

AATTTTATCTACTTTATTATTAAAAAGAGGAGCAGCTCCTTTTCATTTTTGATTCCCAGGAGTTATAGAAGGATTAAATT

AATTAATCATATTGGTTGAATATTAATAGCTATAATAAATAACGAACTTTTATGATTAACTTATTTTTTATTATATTCAATT

CAACAAATCATAAAGATATTGGAACTTTATATTTCATTTTTGGAGTATGATCTGGAATAGTCGGAACTTCTCTAAGAATT

AATTGGATTATTAGGATTTATTGTTTGAGCTCATCATATATTTACAGTAGGTATAGACGTAGATACTCGAGCTTATTTTACTTCAGCAACTATAATT

CATGATACTTATTACGTAGTTGCCCATTTTCATTACGTTTTATCTATAGGAGCTGTATTTGCTATTATAGCAGGATTTATTCATTGATACCCTTTATTAACAGGAATAGTTATAAACCCTTCATG

CTGGAATACCTCGACGATACTCAGATTTTCCTGATAGCTACTTAACTTGAAATATTATTTCTTCTTTAGGAAGAACAATT

AATTTGTCACACACAACATATATTTGCTCATTTAGTTCCTCAAGGAACTCCTCCTGTTTTAATACCTTTTATAGTATGCATTGAAACTATTAGTAATGTAATCCGACCAGGAACTTTAGCAGTACGATTGACTGCTAATATAATT

GACGAGATATTTCTCGAGAAGGAACTTTTCAAGGACTTCACACTATTCCCGTAACATTAGGATTACGATGAGGAATAATTTTATTTATTATTTCTGAAGTTTTTTTCTTTATTTCCTTCTTTTGAGCTTTTTTCCATAGTAGCTTATCTCCAACAATCGAATT

AATTGTCATTTTTCTAGAAGTCATCATTTTGGATTCGAAGCAGCTGCTTGATATTGACATTTTGTTGATGTAGTTTGACTATTCTTATATATTTCAATT

AATTTTAGGAATATATTCTTATAAAATGTTTGATCAAGGATGAAGTGAATATTTTGGAGGTCAGATATTATATAATCAAT

ATTATTCAATAACAGGGGATTTTAATAGAACTACATTGAATATGTTAGATGATAAGGGGTGAACTATATCTTTTAGAATT

AATTCCTTTTTCTTCTTGATTACCTGCAGCTATGGCAGCACCGACTCCTGTTTCTGCTTTAGTACATTCTTCTACATTAG

AATTTATTTTCAAAATGTAAAATCTTATAATGCTGGTATATTAACAGCTTTATCTAATCGAATCGGGGATGTTGCTTTAT

CTTTACATTCAGTATCTATTGTAATAACTTTTTTATTTGATTGAATAAGTTTAATATTTATATCTTTTGTTTTATTAATT

AATTTTTATTAGATTTACTTTATTTATTTTAAGATTAAAGTTTTTATTAATAGATTTAGTATATTTTATTGAATGAGAAATT

GAGGATATCAACCTGAACGTTTACAGGCGGGGATTTATTTATTATTTTATACTTTACTGGCTTCGTTGCCGTTATTAATT

AATTATGAAAGTTACTTTAGTATATTTTTTTTAACTTTTTGTGTTTGTGAAGGGGTTCTAGGGCTGTCCATTTTAGTTTC

AATTATTATATTTATTTTTGGAAGTATTGTTTTTATTTCTAGTCGTAAGCATTTACTTTGTACTTTATTGAGATTAGAGT

AATTTTTATAGGAGGAATATTAGTTTTATTCATTTATGTAACTTCTCTTTCATCTAATGAAATATTTTCATTATCTATAAAACTATTTTTTTTATCTTTAAGTATAATT

AATTTTAACAGGATTATTCTTAGCAATACACTATACTGCTGATATTGAAACAGCTTTTAATAGAGTAAATCACATTTATCGTGATGTTAATAATGGTTGATTCCTACGAATT

CTGTTGATAATGCTACTTTAACTCGATTCTTTACATTTCATTTTATTCTCCCTTTCATTGTATTAGCTTTAACTATAATT

CTTTTGATTTTGCTGAAGGGGAGTCTGAGTTAGTTTCAGGATTTAATGTAGAATATAGAAGAGGGGGATTTGCTTTAATT

AATTTTTTTATATATCAGAAATATATATGATTTATTGTATTTTGTTTTCCTTTAGGTCTCGTTTGATTTGCATCTTGTTT

AATTTAAGCTATAAACTTTTTGTAATAAACTAGGATTAGATACCCTATTATTAAAAATAAATATTAAAATGCTAAAGTAG

>BrK27-1

AATTTTATCTACTTTATTATTAAAAAGAGGAGCAGCTCCTTTTCATTTTTGATTCCCAGGAGTTATAGAAGGATTAAATT

AATTAATCATATTGGTTGAATATTAATAGCTATAATAAATAACGAACTTTTATGATTAACTTATTTTTTATTATATTCAATT

CAACAAATCATAAAGATATTGGAACTTTATATTTCATTTTTGGAGTATGATCTGGAATAGTCGGAACTTCTCTAAGAATT

AATTGGATTATTAGGATTTATTGTTTGAGCTCATCATATATTTACAGTAGGTATAGACGTAGATACTCGAGCTTATTTTACTTCAGCAACTATAATT

CATGATACTTATTACGTAGTTGCCCATTTTCATTACGTTTTATCTATAGGAGCTGTATTTGCTATTATAGCAGGATTTATTCATTGATACCCTTTATTAACAGGAATAGTTATAAACCCTTCATG

CTGGAATACCTCGACGATACTCAGATTTTCCTGATAGCTACTTAACTTGAAATATTATTTCTTCTTTAGGAAGAACAATT

AATTTGTCACACACAACATATATTTGCTCATTTAGTTCCTCAAGGAACTCCTCCTGTTTTAATACCTTTTATAGTATGCATTGAAACTATTAGTAATGTAATCCGACCAGGAACTTTAGCAGTACGATTGACTGCTAATATAATT

GACGAGATATTTCTCGAGAAGGAACTTTTCAAGGACTTCACACTATTCCCGTAACATTAGGATTACGATGAGGAATAATTTTATTTATTATTTCTGAAGTTTTTTTCTTTATTTCCTTCTTTTGAGCTTTTTTCCATAGTAGCTTATCTCCAACAATCGAATT

AATTGTCATTTTTCTAGAAGTCATCATTTTGGATTCGAAGCAGCTGCTTGATATTGACATTTTGTTGATGTAGTTTGACTATTCTTATATATTTCAATT

AATTTTAGGAATATATTCTTATAAAATGTTTGATCAAGGATGAAGTGAATATTTTGGAGGTCAGATATTATATAATCAAT

ATTATTCAATAACAGGGGATTTTAATAGAACTACATTGAATATGTTAGATGATAAGGGGTGAACTATATCTTTTAGAATT

AATTCCTTTTTCTTCTTGATTACCTGCAGCTATGGCAGCACCGACTCCTGTTTCTGCTTTAGTACATTCTTCTACATTAG

AATTTATTTTCAAAATGTAAAATCTTATAATGCTGGTATATTAACAGCTTTATCTAATCGAATCGGGGATGTTGCTTTAT

CTTTACATTCAGTATCTATTGTAATAACTTTTTTATTTGATTGAATAAGTTTAATATTTATATCTTTTGTTTTATTAATT

AATTTTTATTAGATTTACTTTATTTATTTTAAGATTAAAGTTTTTATTAATAGATTTAGTATATTTTATTGAATGAGAAATT

GAGGATATCAACCTGAACGTTTACAGGCGGGGATTTATTTATTATTTTATACTTTACTGGCTTCGTTGCCGTTATTAATT

AATTATGAAAGTTACTTTAGTATATTTTTTTTAACTTTTTGTGTTTGTGAAGGGGTTCTAGGGCTGTCCATTTTAGTTTC

AATTATTATATTTATTTTTGGAAGTATTGTTTTTATTTCTAGTCGTAAGCATTTACTTTGTACTTTATTGAGATTAGAGT

AATTTTTATAGGAGGAATATTAGTTTTATTCATTTATGTAACTTCTCTTTCATCTAATGAAATATTTTCATTATCTATAAAACTATTTTTTTTATCTTTAAGTATAATT

AATTTTAACAGGATTATTCTTAGCAATACACTATACTGCTGATATTGAAACAGCTTTTAATAGAGTAAATCACATTTATCGTGATGTTAATAATGGTTGATTCCTACGAATT

CTGTTGATAATGCTACTTTAACTCGATTCTTTACATTTCATTTTATTCTCCCTTTCATTGTATTAGCTTTAACTATAATT

CTTTTGATTTTGCTGAAGGGGAGTCTGAGTTAGTTTCAGGATTTAATGTAGAATATAGAAGAGGGGGATTTGCTTTAATT

AATTTTTTTATATATCAGAAATATATATGATTTATTGTATTTTGTTTTCCTTTAGGTCTCGTTTGATTTGCATCTTGTTT

AATTTAAGCTATAAACTTTTTGTAATAAACTAGGATTAGATACCCTATTATTAAAAATAAATATTAAAATGCTAAAGTAG

>BrK27-2

AATTTTATCTACTTTATTATTAAAAAGAGGAGCAGCTCCTTTTCATTTTTGATTCCCAGGAGTTATAGAAGGATTAAATT

AATTAATCATATTGGTTGAATATTAATAGCTATAATAAATAACGAACTTTTATGATTAACTTATTTTTTATTATATTCAATT

CAACAAATCATAAAGATATTGGAACTTTATATTTCATTTTTGGAGTATGATCTGGAATAGTCGGAACTTCTCTAAGAATT

AATTGGATTATTAGGATTTATTGTTTGAGCTCATCATATATTTACAGTAGGTATAGACGTAGATACTCGAGCTTATTTTACTTCAGCAACTATAATT

CATGATACTTATTACGTAGTTGCCCATTTTCATTACGTTTTATCTATAGGAGCTGTATTTGCTATTATAGCAGGATTTATTCATTGATACCCTTTATTAACAGGAATAGTTATAAACCCTTCATG

CTGGAATACCTCGACGATACTCAGATTTTCCTGATAGCTACTTAACTTGAAATATTATTTCTTCTTTAGGAAGAACAATT

AATTTGTCACACACAACATATATTTGCTCATTTAGTTCCTCAAGGAACTCCTCCTGTTTTAATACCTTTTATAGTATGCATTGAAACTATTAGTAATGTAATCCGACCAGGAACTTTAGCAGTACGATTGACTGCTAATATAATT

GACGAGATATTTCTCGAGAAGGAACTTTTCAAGGACTTCACACTATTCCCGTAACATTAGGATTACGATGAGGAATAATTTTATTTATTATTTCTGAAGTTTTTTTCTTTATTTCCTTCTTTTGAGCTTTTTTCCATAGTAGCTTATCTCCAACAATCGAATT

AATTGTCATTTTTCTAGAAGTCATCATTTTGGATTCGAAGCAGCTGCTTGATATTGACATTTTGTTGATGTAGTTTGACTATTCTTATATATTTCAATT

AATTTTAGGAATATATTCTTATAAAATGTTTGATCAAGGATGAAGTGAATATTTTGGAGGTCAGATATTATATAATCAAT

ATTATTCAATAACAGGGGATTTTAATAGAACTACATTGAATATGTTAGATGATAAGGGGTGAACTATATCTTTTAGAATT

AATTCCTTTTTCTTCTTGATTACCTGCAGCTATGGCAGCACCGACTCCTGTTTCTGCTTTAGTACATTCTTCTACATTAG

AATTTATTTTCAAAATGTAAAATCTTATAATGCTGGTATATTAACAGCTTTATCTAATCGAATCGGGGATGTTGCTTTAT

CTTTACATTCAGTATCTATTGTAATAACTTTTTTATTTGATTGAATAAGTTTAATATTTATATCTTTTGTTTTATTAATT

AATTTTTATTAGATTTACTTTATTTATTTTAAGATTAAAGTTTTTATTAATAGATTTAGTATATTTTATTGAATGAGAAATT

GAGGATATCAACCTGAACGTTTACAGGCGGGGATTTATTTATTATTTTATACTTTACTGGCTTCGTTGCCGTTATTAATT

NNNNNNNNNNNNNNNNNNNNNNNNNNNNNNNNNNNNNNNNNNNNNNNNNNNNNNNNNNNNNNNNNNNNNNNNNNNNNNNN

AATTATTATATTTATTTTTGGAAGTATTGTTTTTATTTCTAGTCGTAAGCATTTACTTTGTACTTTATTGAGATTAGAGT

AATTTTTATAGGAGGAATATTAGTTTTATTCATTTATGTAACTTCTCTTTCATCTAATGAAATATTTTCATTATCTATAAAACTATTTTTTTTATCTTTAAGTATAATT

AATTTTAACAGGATTATTCTTAGCAATACACTATACTGCTGATATTGAAACAGCTTTTAATAGAGTAAATCACATTTATCGTGATGTTAATAATGGTTGATTCCTACGAATT

CTGTTGATAATGCTACTTTAACTCGATTCTTTACATTTCATTTTATTCTCCCTTTCATTGTATTAGCTTTAACTATAATT

CTTTTGATTTTGCTGAAGGGGAGTCTGAGTTAGTTTCAGGATTTAATGTAGAATATAGAAGAGGGGGATTTGCTTTAATT

AATTTTTTTATATATCAGAAATATATATGATTTATTGTATTTTGTTTTCCTTTAGGTCTCGTTTGATTTGCATCTTGTTT

AATTTAAGCTATAAACTTTTTGTAATAAACTAGGATTAGATACCCTATTATTAAAAATAAATATTAAAATGCTAAAGTAG

>BrK30-1

NNNNNNNNNNNNNNNNNNNNNNNNNNNNNNNNNNNNNNNNNNNNNNNNNNNNNNNNNNNNNNNNNNNNNNNNNNNNNNNN

NNNNNNNNNNNNNNNNNNNNNNNNNNNNNNNNNNNNNNNNNNNNNNNNNNNNNNNNNNNNNNNNNNNNNNNNNNNNNNNNNN

NNNNNNNNNNNNNNNNNNNNNNNNNNNNNNNNNNNNNNNNNNNNNNNNNNNNNNNNNNNNNNNNNNNNNNNNNNNNNNNN

NNNNNNNNNNNNNNNNNNNNNNNNNNNNNNNNNNNNNNNNNNNNNNNNNNNNNNNNNNNNNNNNNNNNNNNNNNNNNNNNNNNNNNNNNNNNNNNNN

NNNNNNNNNNNNNNNNNNNNNNNNNNNNNNNNNNNNNNNNNNNNNATAGGAGCTGTATTTGCTATTATAGCAGGATTTATTCATTGATACCCTTTATTAACAGGAATAGTTATAAACCCTTCATG

NNNNNNNNNNNNNNNNNNNNNNNNNNNNNNNNNNNNNNNNNNNNNNNNNNNNNNNNNNNNNNNNNNNNNNNNNNNNNNNN

NNNNNNNNNNNNNNNNNNNNNNNNNNNNNNNNNNNNNNNNNNNNNNNNNNNNNNNNNNNNNNNNNNNNNNNNNNNNNNNNNNNNNNNNNNNNNNNNNNNNNNNNNNNNNNNNNNNNNNNNNNNNNNNNNNNNNNNNNNNNNNNNN

NNNNNNNNNNNNNNNNNNNNNNNNNNNNNNNNNNNNNNNNNNNNNNNNNNNNNNNNNNNNNNNNNNNNNNNNNNNNNNNNNNNNNNNNNNNNNNNNNNNNNNNNNNNNNNNNNNNNNNNNNNNNNNNNNNNNNNNNNNNNNNNNNNNNNNNNNNNNNNNNNNN

NNNNNNNNNNNNNNNNNNNNNNNNNNNNNNNNNNNNNNNNNNNNNNNNNNNNNNNNNNNNNNNNNNNNNNNNNNNNNNNNNNNNNNNNNNNNNNNNNNN

NNNNNNNNNNNNNNNNNNNNNNNNNNNNNNNNNNNNNNNNNNNNNNNNNNNNNNNNNNNNNNNNNNNNNNNNNNNNNNNN

NNNNNNNNNNNNNNNNNNNNNNNNNNNNNNNNNNNNNNNNNNNNNNNNNNNNNNNNNNNNNNNNNNNNNNNNNNNNNNNN

NNNNNNNNNNNNNNNNNNNNNNNNNNNNNNNNNNNNNNNNNNNNNNNNNNNNNNNNNNNNNNNNNNNNNNNNNNNNNNNN

NNNNNNNNNNNNNNNNNNNNNNNNNNNNNNNNNNNNNNNNNNNNNNNNNNNNNNNNNNNNNNNNNNNNNNNNNNNNNNNN

NNNNNNNNNNNNNNNNNNNNNNNNNNNNNNNNNNNNNNNNNNNNNNNNNNNNNNNNNNNNNNNNNNNNNNNNNNNNNNNN

NNNNNNNNNNNNNNNNNNNNNNNNNNNNNNNNNNNNNNNNNNNNNNNNNNNNNNNNNNNNNNNNNNNNNNNNNNNNNNNNNN

NNNNNNNNNNNNNNNNNNNNNNNNNNNNNNNNNNNNNNNNNNNNNNNNNNNNNNNNNNNNNNNNNNNNNNNNNNNNNNNN

NNNNNNNNNNNNNNNNNNNNNNNNNNNNNNNNNNNNNNNNNNNNNNNNNNNNNNNNNNNNNNNNNNNNNNNNNNNNNNNN

NNNNNNNNNNNNNNNNNNNNNNNNNNNNNNNNNNNNNNNNNNNNNNNNNNNNNNNNNNNNNNNNNNNNNNNNNNNNNNNN

NNNNNNNNNNNNNNNNNNNNNNNNNNNNNNNNNNNNNNNNNNNNNNNNNNNNNNNNNNNNNNNNNNNNNNNNNNNNNNNNNNNNNNNNNNNNNNNNNNNNNNNNNNNNN

NNNNNNNNNNNNNNNNNNNNNNNNNNNNNNNNNNNNNNNNNNNNNNNNNNNNNNNNNNNNNNNNNNNNNNNNNNNNNNNNNNNNNNNNNNNNNNNNNNNNNNNNNNNNNNNN

NNNNNNNNNNNNNNNNNNNNNNNNNNNNNNNNNNNNNNNNNNNNNNNNNNNNNNNNNNNNNNNNNNNNNNNNNNNNNNNN

NNNNNNNNNNNNNNNNNNNNNNNNNNNNNNNNNNNNNNNNNNNNNNNNNNNNNNNNNNNNNNNNNNNNNNNNNNNNNNNN

NNNNNNNNNNNNNNNNNNNNNNNNNNNNNNNNNNNNNNNNNNNNNNNNNNNNNNNNNNNNNNNNNNNNNNNNNNNNNNNN

AATTTAAGCTATAAACTTTTTATAATAAACTAGGATTAGATACCCTATTATTAAAAATAAATATTTAAATGCTAAAGTAG

>BrK30-2

AATTTTATCTACTTTATTATTAAAAAGAGGAGCAGCTCCTTTTCATTTTTGATTCCCAGGAGTTATAGAAGGATTAAATT

AATTAATCATATTGGTTGAATATTAATAGCTATAATAAATAACGAACTTTTATGATTAACTTATTTTTTATTATATTCAATT

CAACAAATCATAAAGATATTGGAACTTTATATTTCATTTTTGGAGTATGATCTGGAATAGTCGGAACTTCTCTAAGAATT

AATTGGATTATTAGGATTTATTGTTTGAGCTCATCATATATTTACAGTAGGTATAGACGTAGATACTCGAGCTTATTTTACTTCAGCAACTATAATT

CATGATACTTATTACGTAGTTGCCCATTTTCATTACGTTTTATCTATAGGAGCTGTATTTGCTATTATAGCAGGATTTATTCATTGATACCCTTTATTAACAGGAATAGTTATAAACCCTTCATG

CTGGAATACCTCGACGATACTCAGATTTTCCTGATAGCTACTTAACTTGAAATATTATTTCTTCTTTAGGAAGAACAATT

AATTTGTCACACACAACATATATTTGCTCATTTAGTTCCTCAAGGAACTCCTCCTGTTTTAATACCTTTTATAGTATGCATTGAAACTATTAGTAATGTAATCCGACCAGGAACTTTAGCAGTACGATTGACTGCTAATATAATT

GACGAGATATTTCTCGAGAAGGAACTTTTCAAGGACTTCACACTATTCCCGTAACATTAGGATTACGATGAGGAATAATTTTATTTATTATTTCTGAAGTTTTTTTCTTTATTTCCTTCTTTTGAGCTTTTTTCCATAGTAGCTTATCTCCAACAATCGAATT

AATTGTCATTTTTCTAGAAGTCATCATTTTGGATTCGAAGCAGCTGCTTGATATTGACATTTTGTTGATGTAGTTTGACTATTCTTATATATTTCAATT

AATTTTAGGAATATATTCTTATAAAATGTTTGATCAAGGATGAAGTGAATATTTTGGAGGTCAGATATTATATAATCAAT

ATTATTCAATAACAGGGGATTTTAATAGAACTACATTGAATATGTTAGATGATAAGGGGTGAACTATATCTTTTAGAATT

AATTCCTTTTTCTTCTTGATTACCTGCAGCTATGGCAGCACCGACTCCTGTTTCTGCTTTAGTACATTCTTCTACATTAG

AATTTATTTTCAAAATGTAAAATCTTATAATGCTGGTATATTAACAGCTTTATCTAATCGAATCGGGGATGTTGCTTTAT

CTTTACATTCAGTATCTATTGTAATAACTTTTTTATTTGATTGAATAAGTTTAATATTTATATCTTTTGTTTTATTAATT

AATTTTTATTAGATTTACTTTATTTATTTTAAGATTAAAGTTTTTATTAATAGATTTAGTATATTTTATTGAATGAGAAATT

GAGGATATCAACCTGAACGTTTACAGGCGGGGATTTATTTATTATTTTATACTTTACTGGCTTCGTTGCCGTTATTAATT

AATTATGAAAGTTACTTTAGTATATTTTTTTTAACTTTTTGTGTTTGTGAAGGGGTTCTAGGGCTGTCCATTTTAGTTTC

AATTATTATATTTATTTTTGGAAGTATTGTTTTTATTTCTAGTCGTAAGCATTTACTTTGTACTTTATTGAGATTAGAGT

AATTTTTATAGGAGGAATATTAGTTTTATTCATTTATGTAACTTCTCTTTCATCTAATGAAATATTTTCATTATCTATAAAACTATTTTTTTTATCTTTAAGTATAATT

AATTTTAACAGGATTATTCTTAGCAATACACTATACTGCTGATATTGAAACAGCTTTTAATAGAGTAAATCACATTTATCGTGATGTTAATAATGGTTGATTCCTACGAATT

CTGTTGATAATGCTACTTTAACTCGATTCTTTACATTTCATTTTATTCTCCCTTTCATTGTATTAGCTTTAACTATAATT

CTTTTGATTTTGCTGAAGGGGAGTCTGAGTTAGTTTCAGGATTTAATGTAGAATATAGAAGAGGGGGATTTGCTTTAATT

AATTTTTTTATATATCAGAAATATATATGATTTATTGTATTTTGTTTTCCTTTAGGTCTCGTTTGATTTGCATCTTGTTT

AATTTAAGCTATAAACTTTTTGTAATAAACTAGGATTAGATACCCTATTATTAAAAATAAATATTAAAATGCTAAAGTAG

>BrK34-1

AATTTTATCTACTTTATTATTAAAAAGAGGAGCAGCTCCTTTTCATTTTTGATTCCCAGGAGTTATAGAAGGATTAAATT

AATTAATCATATTGGTTGAATATTAATAGCTATAATAAATAACGAACTTTTATGATTAACTTATTTTTTATTATATTCAATT

CAACAAATCATAAAGATATTGGAACTTTATATTTCATTTTTGGAGTATGATCTGGAATAGTCGGAACTTCTCTAAGAATT

AATTGGATTATTAGGATTTATTGTTTGAGCTCATCATATATTTACAGTAGGTATAGACGTAGATACTCGAGCTTATTTTACTTCAGCAACTATAATT

CATGATACTTATTACGTAGTTGCCCATTTTCATTACGTTTTATCTATAGGAGCTGTATTTGCTATTATAGCAGGATTTATTCATTGATACCCTTTATTAACAGGAATAGTTATAAACCCTTCATG

CTGGAATACCTCGACGATACTCAGATTTTCCTGATAGCTACTTAACTTGAAATATTATTTCTTCTTTAGGAAGAACAATT

AATTTGTCACACACAACATATATTTGCTCATTTAGTTCCTCAAGGAACTCCTCCTGTTTTAATACCTTTTATAGTATGCATTGAAACTATTAGTAATGTAATCCGACCAGGAACTTTAGCAGTACGATTGACTGCTAATATAATT

GACGAGATATTTCTCGAGAAGGAACTTTTCAAGGACTTCACACTATTCCCGTAACATTAGGATTACGATGAGGAATAATTTTATTTATTATTTCTGAAGTTTTTTTCTTTATTTCCTTCTTTTGAGCTTTTTTCCATAGTAGCTTATCTCCAACAATCGAATT

AATTGTCATTTTTCTAGAAGTCATCATTTTGGATTCGAAGCAGCTGCTTGATATTGACATTTTGTTGATGTAGTTTGACTATTCTTATATATTTCAATT

AATTTTAGGAATATATTCTTATAAAATGTTTGATCAAGGATGAAGTGAATATTTTGGAGGTCAGATATTATATAATCAAT

ATTATTCAATAACAGGGGATTTTAATAGAACTACATTGAATATGTTAGATGATAAGGGGTGAACTATATCTTTTAGAATT

AATTCCTTTTTCTTCTTGATTACCTGCAGCTATGGCAGCACCGACTCCTGTTTCTGCTTTAGTACATTCTTCTACATTAG

AATTTATTTTCAAAATGTAAAATCTTATAATGCTGGTATATTAACAGCTTTATCTAATCGAATCGGGGATGTTGCTTTAT

CTTTACATTCAGTATCTATTGTAATAACTTTTTTATTTGATTGAATAAGTTTAATATTTATATCTTTTGTTTTATTAATT

AATTTTTATTAGATTTACTTTATTTATTTTAAGATTAAAGTTTTTATTAATAGATTTAGTATATTTTATTGAATGAGAAATT

GAGGATATCAACCTGAACGTTTACAGGCGGGGATTTATTTATTATTTTATACTTTACTGGCTTCGTTGCCGTTATTAATT

AATTATGAAAGTTACTTTAGTATATTTTTTTTAACTTTTTGTGTTTGTGAAGGGGTTCTAGGGCTGTCCATTTTAGTTTC

AATTATTATATTTATTTTTGGAAGTATTGTTTTTATTTCTAGTCGTAAGCATTTACTTTGTACTTTATTGAGATTAGAGT

AATTTTTATAGGAGGAATATTAGTTTTATTCATTTATGTAACTTCTCTTTCATCTAATGAAATATTTTCATTATCTATAAAACTATTTTTTTTATCTTTAAGTATAATT

AATTTTAACAGGATTATTCTTAGCAATACACTATACTGCTGATATTGAAACAGCTTTTAATAGAGTAAATCACATTTATCGTGATGTTAATAATGGTTGATTCCTACGAATT

CTGTTGATAATGCTACTTTAACTCGATTCTTTACATTTCATTTTATTCTCCCTTTCATTGTATTAGCTTTAACTATAATT

CTTTTGATTTTGCTGAAGGGGAGTCTGAGTTAGTTTCAGGATTTAATGTAGAATATAGAAGAGGGGGATTTGCTTTAATT

AATTTTTTTATATATCAGAAATATATATGATTTATTGTATTTTGTTTTCCTTTAGGTCTCGTTTGATTTGCATCTTGTTT

AATTTAAGCTATAAACTTTTTGTAATAAACTAGGATTAGATACCCTATTATTAAAAATAAATATTAAAATGCTAAAGTAG

>BrK34-2

AATTTTATCTACTTTATTATTAAAAAGAGGGGCAGCTCCTTTTCATTTTTGATTCCCAGGGGTTATAGAAGGATTAAATT

AATTAATCATATTGGTTGAATATTAATAGCTATAATAAATAACGAACTTTTATGATTAACTTATTTTTTATTATATTCAATT

CAACAAATCATAAAGATATTGGAACTTTATATTTCATTTTTGGAGTATGATCTGGAATAGTCGGAACTTCTCTAAGAATT

AATTGGATTATTGGGATTTATTGTTTGAGCTCATCATATATTTACAGTAGGTATAGATGTAGATACTCGAGCTTATTTTACTTCAGCAACTATAATT

CATGATACTTATTACGTAGTTGCCCATTTTCATTACGTTTTATCTATAGGAGCTGTATTTGCTATTATAGCAGGATTTATTCATTGATACCCTTTATTAACAGGAATAGTTATAAACCCTTCATG

CTGGAATACCTCGACGATACTCAGATTTTCCTGATAGTTACTTAACTTGAAATATTATTTCTTCTTTAGGAAGAACAATT

AATTTGTCACACACAACATATATTTGCTCATTTAGTTCCTCAAGGGACTCCTCCTGTTTTAATACCTTTTATAGTATGCATTGAAACTATTAGTAATGTAATCCGACCAGGAACTTTAGCAGTACGATTGACTGCTAATATAATT

GACGAGATATTTCTCGAGAAGGAACTTTTCAAGGACTTCACACTATTCCCGTAACATTAGGATTACGATGAGGAATAATTTTATTTATTATTTCTGAAGTTTTTTTCTTTATTTCCTTCTTTTGAGCTTTTTTCCATAGTAGCTTATCTCCAACAATCGAATT

AATTGTCATTTTTCTAGAAGTCATCATTTTGGATTCGAAGCAGCTGCTTGATATTGACATTTTGTTGATGTAGTTTGACTATTCTTATATATTTCAATT

AATTTTAGGAATATATTCTTATAAAATGTTTGATCAAGGATGAAGTGAATATTTTGGAGGTCAGATATTATATAATCAAT

ATTATTCAATAACAGGGGATTTTAATAGAACTACATTGAATATGTTAAATGATAAGGGGTGAGCTATATCTTTTAGAATT

AATTCCTTTTTCTTCTTGATTACCTGCAGCTATGGCAGCACCGACTCCTGTTTCTGCTTTAGTACATTCTTCTACATTAG

AATTTATTTTCAAAATGTAAAATCTTATAATGCTGGTATATTAACAGCTTTATCTAATCGAATCGGAGATGTTGCCTTAT

CTTTACATTCAATATCTATTGTAATAACTTTTTTATTTGATTGAATAAGTTTAATATTTATATCTTTTGTTTTATTAATT

AATTTTTATTAGATTTACTTTATTTATTTTAAGATTAAAGTTTTTATTAATAGATTTAGTATATTTTATTGAATGAGAAATT

GAGGATATCAACCTGAACGTTTACAGGCGGGGATTTATTTATTATTTTATACTTTACTGGCTTCGTTGCCGTTATTAATT

AATTATGAAAGTTACTTTAGTATATTTTTTTTAACTTTTTGTGTTTGTGAAGGGGTTCTAGGGCTATCTATTTTAGTTTC

AATTATTATATTTATTTTTGGAAGTATTGTTTTTATTTCTAGTCGTAAGCATTTACTTTGTACTTTATTGAGATTAGAGT

AATTTTTATAGGAGGAATATTAGTTTTATTCATTTATGTAACTTCTCTTTCATCTAATGAAATATTTTCATTATCTATAAAACTATTTTTTTTATCTTTAAGTATAATT

AATTTTAACAGGATTATTCTTAGCAATACACTATACTGCTGATATTGAAACAGCTTTTAATAGAGTAAATCACATTTATCGTGATGTTAATAATGGTTGATTCCTACGAATT

CTGTTGATAATGCTACTTTAACTCGATTCTTTACCTTTCATTTTATTCTCCCTTTCATTGTATTAGCTTTAACTATAATT

CTTTTGATTTTGCTGAAGGGGAGTCTGAGTTAGTTTCAGGATTTAATGTAGAGTATAGAAGAGGGGGATTTGCTTTAATT

AATTTTTTTATATATCAGAAATATATATGATTTATTGTATTTTGTTTTCCTTTAGGTCTCGTTTGATTTGCATCTTGTTT

AATTTAAGCTATAAACTTTTTGTAATAAACTAGGATTAGATACCCTATTATTAAAAATAAATATTAAAATGCTAAAGTAG

>BrK43-1

AATTTTATCTACTTTATTATTAAAAAGAGGAGCAGCTCCTTTTCATTTTTGATTCCCAGGAGTTATAGAAGGATTAAATT

AATTAATCATATTGGTTGAATATTAATAGCTATAATAAATAACGAACTTTTATGATTAACTTATTTTTTATTATATTCAATT

CAACAAATCATAAAGATATTGGAACTTTATATTTCATTTTTGGAGTATGATCTGGAATAGTCGGAACTTCTCTAAGAATT

AATTGGATTATTAGGATTTATTGTTTGAGCTCATCATATATTTACAGTAGGTATAGACGTAGATACTCGAGCTTATTTTACTTCAGCAACTATAATT

CATGATACTTATTACGTAGTTGCCCATTTTCATTACGTTTTATCTATAGGAGCTGTATTTGCTATTATAGCAGGATTTATTCATTGATACCCTTTATTAACAGGAATAGTTATAAACCCTTCATG

CTGGAATACCTCGACGATACTCAGATTTTCCTGATAGCTACTTAACTTGAAATATTATTTCTTCTTTAGGAAGAACAATT

AATTTGTCACACACAACATATATTTGCTCATTTAGTTCCTCAAGGAACTCCTCCTGTTTTAATACCTTTTATAGTATGCATTGAAACTATTAGTAATGTAATCCGACCAGGAACTTTAGCAGTACGATTGACTGCTAATATAATT

GACGAGATATTTCTCGAGAAGGAACTTTTCAAGGACTTCACACTATTCCCGTAACATTAGGATTACGATGAGGAATAATTTTATTTATTATTTCTGAAGTTTTTTTCTTTATTTCCTTCTTTTGAGCTTTTTTCCATAGTAGCTTATCTCCAACAATCGAATT

AATTGTCATTTTTCTAGAAGTCATCATTTTGGATTCGAAGCAGCTGCTTGATATTGACATTTTGTTGATGTAGTTTGACTATTCTTATATATTTCAATT

AATTTTAGGAATATATTCTTATAAAATGTTTGATCAAGGATGAAGTGAATATTTTGGAGGTCAGATATTATATAATCAAT

ATTATTCAATAACAGGGGATTTTAATAGAACTACATTGAATATGTTAGATGATAAGGGGTGAACTATATCTTTTAGAATT

AATTCCTTTTTCTTCTTGATTACCTGCAGCTATGGCAGCACCGACTCCTGTTTCTGCTTTAGTACATTCTTCTACATTAG

AATTTATTTTCAAAATGTAAAATCTTATAATGCTGGTATATTAACAGCTTTATCTAATCGAATCGGGGATGTTGCTTTAT

CTTTACATTCAGTATCTATTGTAATAACTTTTTTATTTGATTGAATAAGTTTAATATTTATATCTTTTGTTTTATTAATT

AATTTTTATTAGATTTACTTTATTTATTTTAAGATTAAAGTTTTTATTAATAGATTTAGTATATTTTATTGAATGAGAAATT

GAGGATATCAACCTGAACGTTTACAGGCGGGGATTTATTTATTATTTTATACTTTACTGGCTTCGTTGCCGTTATTAATT

AATTATGAAAGTTACTTTAGTATATTTTTTTTAACTTTTTGTGTTTGTGAAGGGGTTCTAGGGCTGTCCATTTTAGTTTC

AATTATTATATTTATTTTTGGAAGTATTGTTTTTATTTCTAGTCGTAAGCATTTACTTTGTACTTTATTGAGATTAGAGT

AATTTTTATAGGAGGAATATTAGTTTTATTCATTTATGTAACTTCTCTTTCATCTAATGAAATATTTTCATTATCTATAAAACTATTTTTTTTATCTTTAAGTATAATT

AATTTTAACAGGATTATTCTTAGCAATACACTATACTGCTGATATTGAAACAGCTTTTAATAGAGTAAATCACATTTATCGTGATGTTAATAATGGTTGATTCCTACGAATT

CTGTTGATAATGCTACTTTAACTCGATTCTTTACATTTCATTTTATTCTCCCTTTCATTGTATTAGCTTTAACTATAATT

CTTTTGATTTTGCTGAAGGGGAGTCTGAGTTAGTTTCAGGATTTAATGTAGAATATAGAAGAGGGGGATTTGCTTTAATT

AATTTTTTTATATATCAGAAATATATATGATTTATTGTATTTTGTTTTCCTTTAGGTCTCGTTTGATTTGCATCTTGTTT

AATTTAAGCTATAAACTTTTTGTAATAAACTAGGATTAGATACCCTATTATTAAAAATAAATATTAAAATGCTAAAGTAG

>BrK45-1

AATTTTATCTACTTTATTATTAAAAAGAGGAGCAGCTCCTTTTCATTTTTGATTCCCAGGAGTTATAGAAGGATTAAATT

AATTAATCATATTGGTTGAATATTAATAGCTATAATAAATAACGAACTTTTATGATTAACTTATTTTTTATTATATTCAATT

CAACAAATCATAAAGATATTGGAACTTTATATTTCATTTTTGGAGTATGATCTGGAATAGTCGGAACTTCTCTAAGAATT

AATTGGATTATTAGGATTTATTGTTTGAGCTCATCATATATTTACAGTAGGTATAGACGTAGATACTCGAGCTTATTTTACTTCAGCAACTATAATT

CATGATACTTATTACGTAGTTGCCCATTTTCATTACGTTTTATCTATAGGAGCTGTATTTGCTATTATAGCAGGATTTATTCATTGATACCCTTTATTAACAGGAATAGTTATAAACCCTTCATG

CTGGAATACCTCGACGATACTCAGATTTTCCTGATAGCTACTTAACTTGAAATATTATTTCTTCTTTAGGAAGAACAATT

AATTTGTCACACACAACATATATTTGCTCATTTAGTTCCTCAAGGAACTCCTCCTGTTTTAATACCTTTTATAGTATGCATTGAAACTATTAGTAATGTAATCCGACCAGGAACTTTAGCAGTACGATTGACTGCTAATATAATT

GACGAGATATTTCTCGAGAAGGAACTTTTCAAGGACTTCACACTATTCCCGTAACATTAGGATTACGATGAGGAATAATTTTATTTATTATTTCTGAAGTTTTTTTCTTTATTTCCTTCTTTTGAGCTTTTTTCCATAGTAGCTTATCTCCAACAATCGAATT

AATTGTCATTTTTCTAGAAGTCATCATTTTGGATTCGAAGCAGCTGCTTGATATTGACATTTTGTTGATGTAGTTTGACTATTCTTATATATTTCAATT

AATTTTAGGAATATATTCTTATAAAATGTTTGATCAAGGATGAAGTGAATATTTTGGAGGTCAGATATTATATAATCAAT

ATTATTCAATAACAGGGGATTTTAATAGAACTACATTGAATATGTTAGATGATAAGGGGTGAACTATATCTTTTAGAATT

AATTCCTTTTTCTTCTTGATTACCTGCAGCTATGGCAGCACCGACTCCTGTTTCTGCTTTAGTACATTCTTCTACATTAG

AATTTATTTTCAAAATGTAAAATCTTATAATGCTGGTATATTAACAGCTTTATCTAATCGAATCGGGGATGTTGCTTTAT

CTTTACATTCAGTATCTATTGTAATAACTTTTTTATTTGATTGAATAAGTTTAATATTTATATCTTTTGTTTTATTAATT

AATTTTTATTAGATTTACTTTATTTATTTTAAGATTAAAGTTTTTATTAATAGATTTAGTATATTTTATTGAATGAGAAATT

GAGGATATCAACCTGAACGTTTACAGGCGGGGATTTATTTATTATTTTATACTTTACTGGCTTCGTTGCCGTTATTAATT

AATTATGAAAGTTACTTTAGTATATTTTTTTTAACTTTTTGTGTTTGTGAAGGGGTTCTAGGGCTGTCCATTTTAGTTTC

AATTATTATATTTATTTTTGGAAGTATTGTTTTTATTTCTAGTCGTAAGCATTTACTTTGTACTTTATTGAGATTAGAGT

AATTTTTATAGGAGGAATATTAGTTTTATTCATTTATGTAACTTCTCTTTCATCTAATGAAATATTTTCATTATCTATAAAACTATTTTTTTTATCTTTAAGTATAATT

AATTTTAACAGGATTATTCTTAGCAATACACTATACTGCTGATATTGAAACAGCTTTTAATAGAGTAAATCACATTTATCGTGATGTTAATAATGGTTGATTCCTACGAATT

CTGTTGATAATGCTACTTTAACTCGATTCTTTACATTTCATTTTATTCTCCCTTTCATTGTATTAGCTTTAACTATAATT

CTTTTGATTTTGCTGAAGGGGAGTCTGAGTTAGTTTCAGGATTTAATGTAGAATATAGAAGAGGGGGATTTGCTTTAATT

AATTTTTTTATATATCAGAAATATATATGATTTATTGTATTTTGTTTTCCTTTAGGTCTCGTTTGATTTGCATCTTGTTT

AATTTAAGCTATAAACTTTTTGTAATAAACTAGGATTAGATACCCTATTATTAAAAATAAATATTAAAATGCTAAAGTAG

>BrK45-2

AATTTTATCTACTTTATTATTAAAAAGAGGAGCAGCTCCTTTTCATTTTTGATTCCCAGGAGTTATAGAAGGATTAAATT

AATTAATCATATTGGTTGAATATTAATAGCTATAATAAATAACGAACTTTTATGATTAACTTATTTTTTATTATATTCAATT

CAACAAATCATAAAGATATTGGAACTTTATATTTCATTTTTGGAGTATGATCTGGAATAGTCGGAACTTCTCTAAGAATT

AATTGGATTATTAGGATTTATTGTTTGAGCTCATCATATATTTACAGTAGGTATAGACGTAGATACTCGAGCTTATTTTACTTCAGCAACTATAATT

CATGATACTTATTACGTAGTTGCCCATTTTCATTACGTTTTATCTATAGGAGCTGTATTTGCTATTATAGCAGGATTTATTCATTGATACCCTTTATTAACAGGAATAGTTATAAACCCTTCATG

CTGGAATACCTCGACGATACTCAGATTTTCCTGATAGCTACTTAACTTGAAATATTATTTCTTCTTTAGGAAGAACAATT

AATTTGTCACACACAACATATATTTGCTCATTTAGTTCCTCAAGGAACTCCTCCTGTTTTAATACCTTTTATAGTATGCATTGAAACTATTAGTAATGTAATCCGACCAGGAACTTTAGCAGTACGATTGACTGCTAATATAATT

GACGAGATATTTCTCGAGAAGGAACTTTTCAAGGACTTCACACTATTCCCGTAACATTAGGATTACGATGAGGAATAATTTTATTTATTATTTCTGAAGTTTTTTTCTTTATTTCCTTCTTTTGAGCTTTTTTCCATAGTAGCTTATCTCCAACAATCGAATT

AATTGTCATTTTTCTAGAAGTCATCATTTTGGATTCGAAGCAGCTGCTTGATATTGACATTTTGTTGATGTAGTTTGACTATTCTTATATATTTCAATT

AATTTTAGGAATATATTCTTATAAAATGTTTGATCAAGGATGAAGTGAATATTTTGGAGGTCAGATATTATATAATCAAT

ATTATTCAATAACAGGGGATTTTAATAGAACTACATTGAATATGTTAGATGATAAGGGGTGAACTATATCTTTTAGAATT

AATTCCTTTTTCTTCTTGATTACCTGCAGCTATGGCAGCACCGACTCCTGTTTCTGCTTTAGTACATTCTTCTACATTAG

AATTTATTTTCAAAATGTAAAATCTTATAATGCTGGTATATTAACAGCTTTATCTAATCGAATCGGGGATGTTGCTTTAT

CTTTACATTCAGTATCTATTGTAATAACTTTTTTATTTGATTGAATAAGTTTAATATTTATATCTTTTGTTTTATTAATT

AATTTTTATTAGATTTACTTTATTTATTTTAAGATTAAAGTTTTTATTAATAGATTTAGTATATTTTATTGAATGAGAAATT

GAGGATATCAACCTGAACGTTTACAGGCGGGGATTTATTTATTATTTTATACTTTACTGGCTTCGTTGCCGTTATTAATT

AATTATGAAAGTTACTTTAGTATATTTTTTTTAACTTTTTGTGTTTGTGAAGGGGTTCTAGGGCTGTCCATTTTAGTTTC

AATTATTATATTTATTTTTGGAAGTATTGTTTTTATTTCTAGTCGTAAGCATTTACTTTGTACTTTATTGAGATTAGAGT

AATTTTTATAGGAGGAATATTAGTTTTATTCATTTATGTAACTTCTCTTTCATCTAATGAAATATTTTCATTATCTATAAAACTATTTTTTTTATCTTTAAGTATAATT

AATTTTAACAGGATTATTCTTAGCAATACACTATACTGCTGATATTGAAACAGCTTTTAATAGAGTAAATCACATTTATCGTGATGTTAATAATGGTTGATTCCTACGAATT

CTGTTGATAATGCTACTTTAACTCGATTCTTTACATTTCATTTTATTCTCCCTTTCATTGTATTAGCTTTAACTATAATT

CTTTTGATTTTGCTGAAGGGGAGTCTGAGTTAGTTTCAGGATTTAATGTAGAATATAGAAGAGGGGGATTTGCTTTAATT

AATTTTTTTATATATCAGAAATATATATGATTTATTGTATTTTGTTTTCCTTTAGGTCTCGTTTGATTTGCATCTTGTTT

AATTTAAGCTATAAACTTTTTGTAATAAACTAGGATTAGATACCCTATTATTAAAAATAAATATTAAAATGCTAAAGTAG

>BrK47-1

AATTTTATCTACTTTATTATTAAAAAGAGGAGCAGCTCCTTTTCATTTTTGATCCCCAGGAGTTATAGAAGGATTAAATT

AATTAATCATATTGGTTGAATATTAATAGCTATAATAAATAACGAACTTTTATGATTAACTTATTTTTTATTATATTCAATT

CAACAAATCATAAAGATATTGGAACTTTATATTTCATTTTTGGAGTATGATCTGGAATAGTCGGAACTTCTCTAAGAATT

AATTGGATTATTAGGATTTATTGTTTGAGCTCATCATATATTTACAGTAGGTATAGACGTAGATACTCGAGCTTATTTTACTTCAGCAACTATAATT

CATGATACTTATTACGTAGTTGCCCATTTTCATTACGTTTTATCTATAGGAGCTGTATTTGCTATTATAGCAGGATTTATTCATTGATACCCTTTATTAACAGGAATAGTTATAAACCCTTCATG

CTGGAATACCTCGACGATACTCAGATTTTCCTGATAGCTACTTAACTTGAAATATTATTTCTTCTTTAGGAAGAACAATT

AATTTGTCACACACAACATATATTTGCTCATTTAGTTCCTCAAGGAACTCCTCCTGTTTTAATACCTTTTATAGTATGCATTGAAACTATTAGTAATGTAATCCGACCAGGAACTTTAGCAGTACGATTGACTGCTAATATAATT

GACGAGATATTTCTCGAGAAGGAACTTTTCAAGGACTTCACACTATTCCCGTAACATTAGGATTACGATGAGGAATAATTTTATTTATTATTTCTGAAGTTTTTTTCTTTATTTCCTTCTTTTGAGCTTTTTTCCATAGTAGCTTATCTCCAACAATCGAATT

AATTGTCATTTTTCTAGAAGTCATCATTTTGGATTCGAAGCAGCTGCTTGATATTGACATTTTGTTGATGTAGTTTGACTATTCTTATATATTTCAATT

AATTTTAGGAATATATTCTTATAAAATGTTTGATCAAGGATGAAGTGAATATTTTGGAGGTCAGATATTATATAATCAAT

ATTATTCAATAACAGGGGATTTTAATAGAACTACATTGAATATGTTAGATGATAAGGGGTGAACTATATCTTTTAGAATT

AATTCCTTTTTCTTCTTGATTACCTGCAGCTATGGCAGCACCGACTCCTGTTTCTGCTTTAGTACATTCTTCTACATTAG

AATTTATTTTCAAAATGTAAAATCTTATAATGCTGGTATATTAACAGCTTTATCTAATCGAATCGGGGATGTTGCTTTAT

CTTTACATTCAGTATCTATTGTAATAACTTTTTTATTTGATTGAATAAGTTTAATATTTATATCTTTTGTTTTATTAATT

AATTTTTATTAGATTTACTTTATTTATTTTAAGATTAAAGTTTTTATTAATAGATTTAGTATATTTTATTGAATGAGAAATT

GAGGATATCAACCTGAACGTTTACAGGCGGGGATTTATTTATTATTTTATACTTTACTGGCTTCGTTGCCGTTATTAATT

AATTATGAAAGTTACTTTAGTATATTTTTTTTAACTTTTTGTGTTTGTGAAGGGGTTCTAGGGCTGTCCATTTTAGTTTC

AATTATTATATTTATTTTTGGAAGTATTGTTTTTATTTCTAGTCGTAAGCATTTACTTTGTACTTTATTGAGATTAGAGT

AATTTTTATAGGAGGAATATTAGTTTTATTCATTTATGTAACTTCTCTTTCATCTAATGAAATATTTTCATTATCTATAAAACTATTTTTTTTATCTTTAAGTATAATT

AATTTTAACAGGATTATTCTTAGCAATACACTATACTGCTGATATTGAAACAGCTTTTAATAGAGTAAATCACATTTATCGTGATGTTAATAATGGTTGATTCCTACGAATT

CTGTTGATAATGCTACTTTAACTCGATTCTTTACATTTCATTTTATTCTCCCTTTCATTGTATTAGCTTTAACTATAATT

CTTTTGATTTTGCTGAAGGGGAGTCTGAGTTAGTTTCAGGATTTAATGTAGAATATAGAAGAGGGGGATTTGCTTTAATT

AATTTTTTTATATATCAGAAATATATATGATTTATTGTATTTTGTTTTCCTTTAGGTCTCGTTTGATTTGCATCTTGTTT

AATTTAAGCTATAAACTTTTTGTAATAAACTAGGATTAGATACCCTATTATTAAAAATAAATATTAAAATGCTAAAGTAG

>BrK47-2

AATTTTATCTACTTTATTATTAAAAAGAGGAGCAGCTCCTTTTCATTTTTGATTCCCAGGAGTTATAGAAGGATTAAATT

AATTAATCATATTGGTTGAATATTAATAGCTATAATAAATAACGAACTTTTATGATTAACTTATTTTTTATTATATTCAATT

CAACAAATCATAAAGATATTGGAACTTTATATTTCATTTTTGGAGTATGATCTGGAATAGTCGGAACTTCTCTAAGAATT

AATTGGATTATTAGGATTTATTGTTTGAGCTCATCATATATTTACAGTAGGTATAGACGTAGATACTCGAGCTTATTTTACTTCAGCAACTATAATT

CATGATACTTATTACGTAGTTGCCCATTTTCATTACGTTTTATCTATAGGAGCTGTATTTGCTATTATAGCAGGATTTATTCATTGATACCCTTTATTAACAGGAATAGTTATAAACCCTTCATG

CTGGAATACCTCGACGATACTCAGATTTTCCTGATAGCTACTTAACTTGAAATATTATTTCTTCTTTAGGAAGAACAATT

AATTTGTCACACACAACATATATTTGCTCATTTAGTTCCTCAAGGAACTCCTCCTGTTTTAATACCTTTTATAGTATGCATTGAAACTATTAGTAATGTAATCCGACCAGGAACTTTAGCAGTACGATTGACTGCTAATATAATT

GACGAGATATTTCTCGAGAAGGAACTTTTCAAGGACTTCACACTATTCCCGTAACATTAGGATTACGATGAGGAATAATTTTATTTATTATTTCTGAAGTTTTTTTCTTTATTTCCTTCTTTTGAGCTTTTTTCCATAGTAGCTTATCTCCAACAATCGAATT

AATTGTCATTTTTCTAGAAGTCATCATTTTGGATTCGAAGCAGCTGCTTGATATTGACATTTTGTTGATGTAGTTTGACTATTCTTATATATTTCAATT

AATTTTAGGAATATATTCTTATAAAATGTTTGATCAAGGATGAAGTGAATATTTTGGAGGTCAGATATTATATAATCAAT

ATTATTCAATAACAGGGGATTTTAATAGAACTACATTGAATATGTTAGATGATAAGGGGTGAACTATATCTTTTAGAATT

AATTCCTTTTTCTTCTTGATTACCTGCAGCTATGGCAGCACCGACTCCTGTTTCTGCTTTAGTACATTCTTCTACATTAG

AATTTATTTTCAAAATGTAAAATCTTATAATGCTGGTATATTAACAGCTTTATCTAATCGAATCGGGGATGTTGCTTTAT

CTTTACATTCAGTATCTATTGTAATAACTTTTTTATTTGATTGAATAAGTTTAATATTTATATCTTTTGTTTTATTAATT

AATTTTTATTAGATTTACTTTATTTATTTTAAGATTAAAGTTTTTATTAATAGATTTAGTATATTTTATTGAATGAGAAATT

GAGGATATCAACCTGAACGTTTACAGGCGGGGATTTATTTATTATTTTATACTTTACTGGCTTCGTTGCCGTTATTAATT

AATTATGAAAGTTACTTTAGTATATTTTTTTTAACTTTTTGTGTTTGTGAAGGGGTTCTAGGGCTGTCCATTTTAGTTTC

AATTATTATATTTATTTTTGGAAGTATTGTTTTTATTTCTAGTCGTAAGCATTTACTTTGTACTTTATTGAGATTAGAGT

AATTTTTATAGGAGGAATATTAGTTTTATTCATTTATGTAACTTCTCTTTCATCTAATGAAATATTTTCATTATCTATAAAACTATTTTTTTTATCTTTAAGTATAATT

AATTTTAACAGGATTATTCTTAGCAATACACTATACTGCTGATATTGAAACAGCTTTTAATAGAGTAAATCACATTTATCGTGATGTTAATAATGGTTGATTCCTACGAATT

CTGTTGATAATGCTACTTTAACTCGATTCTTTACATTTCATTTTATTCTCCCTTTCATTGTATTAGCTTTAACTATAATT

CTTTTGATTTTGCTGAAGGGGAGTCTGAGTTAGTTTCAGGATTTAATGTAGAATATAGAAGAGGGGGATTTGCTTTAATT

AATTTTTTTATATATCAGAAATATATATGATTTATTGTATTTTGTTTTCCTTTAGGTCTCGTTTGATTTGCATCTTGTTT

AATTTAAGCTATAAACTTTTTGTAATAAACTAGGATTAGATACCCTATTATTAAAAATAAATATTAAAATGCTAAAGTAG

>BrK48-1

AATTTTATCTACTTTATTATTAAAAAGAGGAGCAGCTCCTTTTCATTTTTGATTCCCAGGAGTTATAGAAGGATTAAATT

AATTAATCATATTGGTTGAATATTAATAGCTATAATAAATAACGAACTTTTATGATTAACTTATTTTTTATTATATTCAATT

CAACAAATCATAAAGATATTGGAACTTTATATTTCATTTTTGGAGTATGATCTGGAATAGTCGGAACTTCTCTAAGAATT

AATTGGATTATTAGGATTTATTGTTTGAGCTCATCATATATTTACAGTAGGTATAGACGTAGATACTCGAGCTTATTTTACTTCAGCAACTATAATT

CATGATACTTATTACGTAGTTGCCCATTTTCATTACGTTTTATCTATAGGAGCTGTATTTGCTATTATAGCAGGATTTATTCATTGATACCCTTTATTAACAGGAATAGTTATAAACCCTTCATG

CTGGAATACCTCGACGATACTCAGATTTTCCTGATAGCTACTTAACTTGAAATATTATTTCTTCTTTAGGAAGAACAATT

AATTTGTCACACACAACATATATTTGCTCATTTAGTTCCTCAAGGAACTCCTCCTGTTTTAATACCTTTTATAGTATGCATTGAAACTATTAGTAATGTAATCCGACCAGGAACTTTAGCAGTACGATTGACTGCTAATATAATT

GACGAGATATTTCTCGAGAAGGAACTTTTCAAGGACTTCACACTATTCCCGTAACATTAGGATTACGATGAGGAATAATTTTATTTATTATTTCTGAAGTTTTTTTCTTTATTTCCTTCTTTTGAGCTTTTTTCCATAGTAGCTTATCTCCAACAATCGAATT

AATTGTCATTTTTCTAGAAGTCATCATTTTGGATTCGAAGCAGCTGCTTGATATTGACATTTTGTTGATGTAGTTTGACTATTCTTATATATTTCAATT

AATTTTAGGAATATATTCTTATAAAATGTTTGATCAAGGATGAAGTGAATATTTTGGAGGTCAGATATTATATAATCAAT

ATTATTCAATAACAGGGGATTTTAATAGAACTACATTGAATATGTTAGATGATAAGGGGTGAACTATATCTTTTAGAATT

AATTCCTTTTTCTTCTTGATTACCTGCAGCTATGGCAGCACCGACTCCTGTTTCTGCTTTAGTACATTCTTCTACATTAG

AATTTATTTTCAAAATGTAAAATCTTATAATGCTGGTATATTAACAGCTTTATCTAATCGAATCGGGGATGTTGCTTTAT

CTTTACATTCAGTATCTATTGTAATAACTTTTTTATTTGATTGAATAAGTTTAATATTTATATCTTTTGTTTTATTAATT

AATTTTTATTAGATTTACTTTATTTATTTTAAGATTAAAGTTTTTATTAATAGATTTAGTATATTTTATTGAATGAGAAATT

GAGGATATCAACCTGAACGTTTACAGGCGGGGATTTATTTATTATTTTATACTTTACTGGCTTCGTTGCCGTTATTAATT

AATTATGAAAGTTACTTTAGTATATTTTTTTTAACTTTTTGTGTTTGTGAAGGGGTTCTAGGGCTGTCCATTTTAGTTTC

AATTATTATATTTATTTTTGGAAGTATTGTTTTTATTTCTAGTCGTAAGCATTTACTTTGTACTTTATTGAGATTAGAGT

AATTTTTATAGGAGGAATATTAGTTTTATTCATTTATGTAACTTCTCTTTCATCTAATGAAATATTTTCATTATCTATAAAACTATTTTTTTTATCTTTAAGTATAATT

AATTTTAACAGGATTATTCTTAGCAATACACTATACTGCTGATATTGAAACAGCTTTTAATAGAGTAAATCACATTTATCGTGATGTTAATAATGGTTGATTCCTACGAATT

CTGTTGATAATGCTACTTTAACTCGATTCTTTACATTTCATTTTATTCTCCCTTTCATTGTATTAGCTTTAACTATAATT

CTTTTGATTTTGCTGAAGGGGAGTCTGAGTTAGTTTCAGGATTTAATGTAGAATATAGAAGAGGGGGATTTGCTTTAATT

AATTTTTTTATATATCAGAAATATATATGATTTATTGTATTTTGTTTTCCTTTAGGTCTCGTTTGATTTGCATCTTGTTT

AATTTAAGCTATAAACTTTTTGTAATAAACTAGGATTAGATACCCTATTATTAAAAATAAATATTAAAATGCTAAAGTAG

>BrK48-2

AATTTTATCTACTTTATTATTAAAAAGAGGAGCAGCTCCTTTTCATTTTTGATTCCCAGGAGTTATAGAAGGATTAAATT

AATTAATCATATTGGTTGAATATTAATAGCTATAATAAATAACGAACTTTTATGATTAACTTATTTTTTATTATATTCAATT

CAACAAATCATAAAGATATTGGAACTTTATATTTCATTTTTGGAGTATGATCTGGAATAGTCGGAACTTCTCTAAGAATT

AATTGGATTATTAGGATTTATTGTTTGAGCTCATCATATATTTACAGTAGGTATAGACGTAGATACTCGAGCTTATTTTACTTCAGCAACTATAATT

CATGATACTTATTACGTAGTTGCCCATTTTCATTACGTTTTATCTATAGGAGCTGTATTTGCTATTATAGCAGGATTTATTCATTGATACCCTTTATTAACAGGAATAGTTATAAACCCTTCATG

CTGGAATACCTCGACGATACTCAGATTTTCCTGATAGCTACTTAACTTGAAATATTATTTCTTCTTTAGGAAGAACAATT

AATTTGTCACACACAACATATATTTGCTCATTTAGTTCCTCAAGGAACTCCTCCTGTTTTAATACCTTTTATAGTATGCATTGAAACTATTAGTAATGTAATCCGACCAGGAACTTTAGCAGTACGATTGACTGCTAATATAATT

GACGAGATATTTCTCGAGAAGGAACTTTTCAAGGACTTCACACTATTCCCGTAACATTAGGATTACGATGAGGAATAATTTTATTTATTATTTCTGAAGTTTTTTTCTTTATTTCCTTCTTTTGAGCTTTTTTCCATAGTAGCTTATCTCCAACAATCGAATT

AATTGTCATTTTTCTAGAAGTCATCATTTTGGATTCGAAGCAGCTGCTTGATATTGACATTTTGTTGATGTAGTTTGACTATTCTTATATATTTCAATT

AATTTTAGGAATATATTCTTATAAAATGTTTGATCAAGGATGAAGTGAATATTTTGGAGGTCAGATATTATATAATCAAT

ATTATTCAATAACAGGGGATTTTAATAGAACTACATTGAATATGTTAGATGATAAGGGGTGAACTATATCTTTTAGAATT

AATTCCTTTTTCTTCTTGATTACCTGCAGCTATGGCAGCACCGACTCCTGTTTCTGCTTTAGTACATTCTTCTACATTAG

AATTTATTTTCAAAATGTAAAATCTTATAATGCTGGTATATTAACAGCTTTATCTAATCGAATCGGGGATGTTGCTTTAT

CTTTACATTCAGTATCTATTGTAATAACTTTTTTATTTGATTGAATAAGTTTAATATTTATATCTTTTGTTTTATTAATT

AATTTTTATTAGATTTACTTTATTTATTTTAAGATTAAAGTTTTTATTAATAGATTTAGTATATTTTATTGAATGAGAAATT

GAGGATATCAACCTGAACGTTTACAGGCGGGGATTTATTTATTATTTTATACTTTACTGGCTTCGTTGCCGTTATTAATT

AATTATGAAAGTTACTTTAGTATATTTTTTTTAACTTTTTGTGTTTGTGAAGGGGTTCTAGGGCTGTCCATTTTAGTTTC

AATTATTATATTTATTTTTGGAAGTATTGTTTTTATTTCTAGTCGTAAGCATTTACTTTGTACTTTATTGAGATTAGAGT

AATTTTTATAGGAGGAATATTAGTTTTATTCATTTATGTAACTTCTCTTTCATCTAATGAAATATTTTCATTATCTATAAAACTATTTTTTTTATCTTTAAGTATAATT

AATTTTAACAGGATTATTCTTAGCAATACACTATACTGCTGATATTGAAACAGCTTTTAATAGAGTAAATCACATTTATCGTGATGTTAATAATGGTTGATTCCTACGAATT

CTGTTGATAATGCTACTTTAACTCGATTCTTTACATTTCATTTTATTCTCCCTTTCATTGTATTAGCTTTAACTATAATT

CTTTTGATTTTGCTGAAGGGGAGTCTGAGTTAGTTTCAGGATTTAATGTAGAATATAGAAGAGGGGGATTTGCTTTAATT

AATTTTTTTATATATCAGAAATATATATGATTTATTGTATTTTGTTTTCCTTTAGGTCTCGTTTGATTTGCATCTTGTTT

AATTTAAGCTATAAACTTTTTGTAATAAACTAGGATTAGATACCCTATTATTAAAAATAAATATTAAAATGCTAAAGTAG

>BrK53-1

AATTTTATCTACTTTATTATTAAAAAGAGGAGCAGCTCCTTTTCATTTTTGATTCCCAGGAGTTATAGAAGGATTAAATT

AATTAATCATATTGGTTGAATATTAATAGCTATAATAAATAACGAACTTTTATGATTAACTTATTTTTTATTATATTCAATT

CAACAAATCATAAAGATATTGGAACTTTATATTTCATTTTTGGAGTATGATCTGGAATAGTCGGAACTTCTCTAAGAATT

AATTGGATTATTAGGATTTATTGTTTGAGCTCATCATATATTTACAGTAGGTATAGACGTAGATACTCGAGCTTATTTTACTTCAGCAACTATAATT

CATGATACTTATTACGTAGTTGCCCATTTTCATTACGTTTTATCTATAGGAGCTGTATTTGCTATTATAGCAGGATTTATTCATTGATACCCTTTATTAACAGGAATAGTTATAAACCCTTCATG

CTGGAATACCTCGACGATACTCAGATTTTCCTGATAGCTACTTAACTTGAAATATTATTTCTTCTTTAGGAAGAACAATT

AATTTGTCACACACAACATATATTTGCTCATTTAGTTCCTCAAGGAACTCCTCCTGTTTTAATACCTTTTATAGTATGCATTGAAACTATTAGTAATGTAATCCGACCAGGAACTTTAGCAGTACGATTGACTGCTAATATAATT

GACGAGATATTTCTCGAGAAGGAACTTTTCAAGGACTTCACACTATTCCCGTAACATTAGGATTACGATGAGGAATAATTTTATTTATTATTTCTGAAGTTTTTTTCTTTATTTCCTTCTTTTGAGCTTTTTTCCATAGTAGCTTATCTCCAACAATCGAATT

AATTGTCATTTTTCTAGAAGTCATCATTTTGGATTCGAAGCAGCTGCTTGATATTGACATTTTGTTGATGTAGTTTGACTATTCTTATATATTTCAATT

AATTTTAGGAATATATTCTTATAAAATGTTTGATCAAGGATGAAGTGAATATTTTGGAGGTCAGATATTATATAATCAAT

ATTATTCAATAACAGGGGATTTTAATAGAACTACATTGAATATGTTAGATGATAAGGGGTGAACTATATCTTTTAGAATT

AATTCCTTTTTCTTCTTGATTACCTGCAGCTATGGCAGCACCGACTCCTGTTTCTGCTTTAGTACATTCTTCTACATTAG

AATTTATTTTCAAAATGTAAAATCTTATAATGCTGGTATATTAACAGCTTTATCTAATCGAATCGGGGATGTTGCTTTAT

CTTTACATTCAGTATCTATTGTAATAACTTTTTTATTTGATTGAATAAGTTTAATATTTATATCTTTTGTTTTATTAATT

AATTTTTATTAGATTTACTTTATTTATTTTAAGATTAAAGTTTTTATTAATAGATTTAGTATATTTTATTGAATGAGAAATT

GAGGATATCAACCTGAACGTTTACAGGCGGGGATTTATTTATTATTTTATACTTTACTGGCTTCGTTGCCGTTATTAATT

AATTATGAAAGTTACTTTAGTATATTTTTTTTAACTTTTTGTGTTTGTGAAGGGGTTCTAGGGCTGTCCATTTTAGTTTC

AATTATTATATTTATTTTTGGAAGTATTGTTTTTATTTCTAGTCGTAAGCATTTACTTTGTACTTTATTGAGATTAGAGT

AATTTTTATAGGAGGAATATTAGTTTTATTCATTTATGTAACTTCTCTTTCATCTAATGAAATATTTTCATTATCTATAAAACTATTTTTTTTATCTTTAAGTATAATT

AATTTTAACAGGATTATTCTTAGCAATACACTATACTGCTGATATTGAAACAGCTTTTAATAGAGTAAATCACATTTATCGTGATGTTAATAATGGTTGATTCCTACGAATT

CTGTTGATAATGCTACTTTAACTCGATTCTTTACATTTCATTTTATTCTCCCTTTCATTGTATTAGCTTTAACTATAATT

CTTTTGATTTTGCTGAAGGGGAGTCTGAGTTAGTTTCAGGATTTAATGTAGAATATAGAAGAGGGGGATTTGCTTTAATT

AATTTTTTTATATATCAGAAATATATATGATTTATTGTATTTTGTTTTCCTTTAGGTCTCGTTTGATTTGCATCTTGTTT

AATTTAAGCTATAAACTTTTTGTAATAAACTAGGATTAGATACCCTATTATTAAAAATAAATATTAAAATGCTAAAGTAG

>BrK53-2

AATTTTATCTACTTTATTATTAAAAAGAGGAGCAGCTCCTTTTCATTTTTGATTCCCAGGAGTTATAGAAGGATTAAATT

AATTAATCATATTGGTTGAATATTAATAGCTATAATAAATAACGAACTTTTATGATTAACTTATTTTTTATTATATTCAATT

CAACAAATCATAAAGATATTGGAACTTTATATTTCATTTTTGGAGTATGATCTGGAATAGTCGGAACTTCTCTAAGAATT

AATTGGATTATTAGGATTTATTGTTTGAGCTCATCATATATTTACAGTAGGTATAGACGTAGATACTCGAGCTTATTTTACTTCAGCAACTATAATT

CATGATACTTATTACGTAGTTGCCCATTTTCATTACGTTTTATCTATAGGAGCTGTATTTGCTATTATAGCAGGATTTATTCATTGATACCCTTTATTAACAGGAATAGTTATAAACCCTTCATG

CTGGAATACCTCGACGATACTCAGATTTTCCTGATAGCTACTTAACTTGAAATATTATTTCTTCTTTAGGAAGAACAATT

AATTTGTCACACACAACATATATTTGCTCATTTAGTTCCTCAAGGAACTCCTCCTGTTTTAATACCTTTTATAGTATGCATTGAAACTATTAGTAATGTAATCCGACCAGGAACTTTAGCAGTACGATTGACTGCTAATATAATT

GACGAGATATTTCTCGAGAAGGAACTTTTCAAGGACTTCACACTATTCCCGTAACATTAGGATTACGATGAGGAATAATTTTATTTATTATTTCTGAAGTTTTTTTCTTTATTTCCTTCTTTTGAGCTTTTTTCCATAGTAGCTTATCTCCAACAATCGAATT

AATTGTCATTTTTCTAGAAGTCATCATTTTGGATTCGAAGCAGCTGCTTGATATTGACATTTTGTTGATGTAGTTTGACTATTCTTATATATTTCAATT

AATTTTAGGAATATATTCTTATAAAATGTTTGATCAAGGATGAAGTGAATATTTTGGAGGTCAGATATTATATAATCAAT

ATTATTCAATAACAGGGGATTTTAATAGAACTACATTGAATATGTTAGATGATAAGGGGTGAACTATATCTTTTAGAATT

AATTCCTTTTTCTTCTTGATTACCTGCAGCTATGGCAGCACCGACTCCTGTTTCTGCTTTAGTACATTCTTCTACATTAG

AATTTATTTTCAAAATGTAAAATCTTATAATGCTGGTATATTAACAGCTTTATCTAATCGAATCGGGGATGTTGCTTTAT

CTTTACATTCAGTATCTATTGTAATAACTTTTTTATTTGATTGAATAAGTTTAATATTTATATCTTTTGTTTTATTAATT

AATTTTTATTAGATTTACTTTATTTATTTTAAGATTAAAGTTTTTATTAATAGATTTAGTATATTTTATTGAATGAGAAATT

GAGGATATCAACCTGAACGTTTACAGGCGGGGATTTATTTATTATTTTATACTTTACTGGCTTCGTTGCCGTTATTAATT

AATTATGAAAGTTACTTTAGTATATTTTTTTTAACTTTTTGTGTTTGTGAAGGGGTTCTAGGGCTGTCCATTTTAGTTTC

AATTATTATATTTATTTTTGGAAGTATTGTTTTTATTTCTAGTCGTAAGCATTTACTTTGTACTTTATTGAGATTAGAGT

AATTTTTATAGGAGGAATATTAGTTTTATTCATTTATGTAACTTCTCTTTCATCTAATGAAATATTTTCATTATCTATAAAACTATTTTTTTTATCTTTAAGTATAATT

AATTTTAACAGGATTATTCTTAGCAATACACTATACTGCTGATATTGAAACAGCTTTTAATAGAGTAAATCACATTTATCGTGATGTTAATAATGGTTGATTCCTACGAATT

CTGTTGATAATGCTACTTTAACTCGATTCTTTACATTTCATTTTATTCTCCCTTTCATTGTATTAGCTTTAACTATAATT

CTTTTGATTTTGCTGAAGGGGAGTCTGAGTTAGTTTCAGGATTTAATGTAGAATATAGAAGAGGGGGATTTGCTTTAATT

AATTTTTTTATATATCAGAAATATATATGATTTATTGTATTTTGTTTTCCTTTAGGTCTCGTTTGATTTGCATCTTGTTT

AATTTAAGCTATAAACTTTTTGTAATAAACTAGGATTAGATACCCTATTATTAAAAATAAATATTAAAATGCTAAAGTAG

>BrK54-1

AATTTTATCTACTTTATTATTAAAAAGAGGAGCAGCTCCTTTTCATTTTTGATTCCCAGGAGTTATAGAAGGATTAAATT

AATTAATCATATTGGTTGAATATTAATAGCTATAATAAATAACGAACTTTTATGATTAACTTATTTTTTATTATATTCAATT

CAACAAATCATAAAGATATTGGAACTTTATATTTCATTTTTGGAGTATGATCTGGAATAGTCGGAACTTCTCTAAGAATT

AATTGGATTATTAGGATTTATTGTTTGAGCTCATCATATATTTACAGTAGGTATAGACGTAGATACTCGAGCTTATTTTACTTCAGCAACTATAATT

CATGATACTTATTACGTAGTTGCCCATTTTCATTACGTTTTATCTATAGGAGCTGTATTTGCTATTATAGCAGGATTTATTCATTGATACCCTTTATTAACAGGAATAGTTATAAACCCTTCATG

CTGGAATACCTCGACGATACTCAGATTTTCCTGATAGCTACTTAACTTGAAATATTATTTCTTCTTTAGGAAGAACAATT

AATTTGTCACACACAACATATATTTGCTCATTTAGTTCCTCAAGGAACTCCTCCTGTTTTAATACCTTTTATAGTATGCATTGAAACTATTAGTAATGTAATCCGACCAGGAACTTTAGCAGTACGATTGACTGCTAATATAATT

GACGAGATATTTCTCGAGAAGGAACTTTTCAAGGACTTCACACTATTCCCGTAACATTAGGATTACGATGAGGAATAATTTTATTTATTATTTCTGAAGTTTTTTTCTTTATTTCCTTCTTTTGAGCTTTTTTCCATAGTAGCTTATCTCCAACAATCGAATT

AATTGTCATTTTTCTAGAAGTCATCATTTTGGATTCGAAGCAGCTGCTTGATATTGACATTTTGTTGATGTAGTTTGACTATTCTTATATATTTCAATT

AATTTTAGGAATATATTCTTATAAAATGTTTGATCAAGGATGAAGTGAATATTTTGGAGGTCAGATATTATATAATCAAT

ATTATTCAATAACAGGGGATTTTAATAGAACTACATTGAATATGTTAGATGATAAGGGGTGAACTATATCTTTTAGAATT

AATTCCTTTTTCTTCTTGATTACCTGCAGCTATGGCAGCACCGACTCCTGTTTCTGCTTTAGTACATTCTTCTACATTAG

AATTTATTTTCAAAATGTAAAATCTTATAATGCTGGTATATTAACAGCTTTATCTAATCGAATCGGGGATGTTGCTTTAT

CTTTACATTCAGTATCTATTGTAATAACTTTTTTATTTGATTGAATAAGTTTAATATTTATATCTTTTGTTTTATTAATT

AATTTTTATTAGATTTACTTTATTTATTTTAAGATTAAAGTTTTTATTAATAGATTTAGTATATTTTATTGAATGAGAAATT

GAGGATATCAACCTGAACGTTTACAGGCGGGGATTTATTTATTATTTTATACTTTACTGGCTTCGTTGCCGTTATTAATT

AATTATGAAAGTTACTTTAGTATATTTTTTTTAACTTTTTGTGTTTGTGAAGGGGTTCTAGGGCTGTCCATTTTAGTTTC

AATTATTATATTTATTTTTGGAAGTATTGTTTTTATTTCTAGTCGTAAGCATTTACTTTGTACTTTATTGAGATTAGAGT

AATTTTTATAGGAGGAATATTAGTTTTATTCATTTATGTAACTTCTCTTTCATCTAATGAAATATTTTCATTATCTATAAAACTATTTTTTTTATCTTTAAGTATAATT

AATTTTAACAGGATTATTCTTAGCAATACACTATACTGCTGATATTGAAACAGCTTTTAATAGAGTAAATCACATTTATCGTGATGTTAATAATGGTTGATTCCTACGAATT

CTGTTGATAATGCTACTTTAACTCGATTCTTTACATTTCATTTTATTCTCCCTTTCATTGTATTAGCTTTAACTATAATT

CTTTTGATTTTGCTGAAGGGGAGTCTGAGTTAGTTTCAGGATTTAATGTAGAATATAGAAGAGGGGGATTTGCTTTAATT

AATTTTTTTATATATCAGAAATATATATGATTTATTGTATTTTGTTTTCCTTTAGGTCTCGTTTGATTTGCATCTTGTTT

AATTTAAGCTATAAACTTTTTGTAATAAACTAGGATTAGATACCCTATTATTAAAAATAAATATTAAAATGCTAAAGTAG

>BrK54-2

AATTTTATCTACTTTATTATTAAAAAGAGGAGCAGCTCCTTTTCATTTTTGATTCCCAGGAGTTATAGAAGGATTAAATT

AATTAATCATATTGGTTGAATATTAATAGCTATAATAAATAACGAACTTTTATGATTAACTTATTTTTTATTATATTCAATT

CAACAAATCATAAAGATATTGGAACTTTATATTTCATTTTTGGAGTATGATCTGGAATAGTCGGAACTTCTCTAAGAATT

AATTGGATTATTAGGATTTATTGTTTGAGCTCATCATATATTTACAGTAGGTATAGACGTAGATACTCGAGCTTATTTTACTTCAGCAACTATAATT

CATGATACTTATTACGTAGTTGCCCATTTTCATTACGTTTTATCTATAGGAGCTGTATTTGCTATTATAGCAGGATTTATTCATTGATACCCTTTATTAACAGGAATAGTTATAAACCCTTCATG

CTGGAATACCTCGACGATACTCAGATTTTCCTGATAGCTACTTAACTTGAAATATTATTTCTTCTTTAGGAAGAACAATT

AATTTGTCACACACAACATATATTTGCTCATTTAGTTCCTCAAGGAACTCCTCCTGTTTTAATACCTTTTATAGTATGCATTGAAACTATTAGTAATGTAATCCGACCAGGAACTTTAGCAGTACGATTGACTGCTAATATAATT

GACGAGATATTTCTCGAGAAGGAACTTTTCAAGGACTTCACACTATTCCCGTAACATTAGGATTACGATGAGGAATAATTTTATTTATTATTTCTGAAGTTTTTTTCTTTATTTCCTTCTTTTGAGCTTTTTTCCATAGTAGCTTATCTCCAACAATCGAATT

AATTGTCATTTTTCTAGAAGTCATCATTTTGGATTCGAAGCAGCTGCTTGATATTGACATTTTGTTGATGTAGTTTGACTATTCTTATATATTTCAATT

AATTTTAGGAATATATTCTTATAAAATGTTTGATCAAGGATGAAGTGAATATTTTGGAGGTCAGATATTATATAATCAAT

ATTATTCAATAACAGGGGATTTTAATAGAACTACATTGAATATGTTAGATGATAAGGGGTGAACTATATCTTTTAGAATT

AATTCCTTTTTCTTCTTGATTACCTGCAGCTATGGCAGCACCGACTCCTGTTTCTGCTTTAGTACATTCTTCTACATTAG

AATTTATTTTCAAAATGTAAAATCTTATAATGCTGGTATATTAACAGCTTTATCTAATCGAATCGGGGATGTTGCTTTAT

CTTTACATTCAGTATCTATTGTAATAACTTTTTTATTTGATTGAATAAGTTTAATATTTATATCTTTTGTTTTATTAATT

AATTTTTATTAGATTTACTTTATTTATTTTAAGATTAAAGTTTTTATTAATAGATTTAGTATATTTTATTGAATGAGAAATT

GAGGATATCAACCTGAACGTTTACAGGCGGGGATTTATTTATTATTTTATACTTTACTGGCTTCGTTGCCGTTATTAATT

AATTATGAAAGTTACTTTAGTATATTTTTTTTAACTTTTTGTGTTTGTGAAGGGGTTCTAGGGCTGTCCATTTTAGTTTC

AATTATTATATTTATTTTTGGAAGTATTGTTTTTATTTCTAGTCGTAAGCATTTACTTTGTACTTTATTGAGATTAGAGT

AATTTTTATAGGAGGAATATTAGTTTTATTCATTTATGTAACTTCTCTTTCATCTAATGAAATATTTTCATTATCTATAAAACTATTTTTTTTATCTTTAAGTATAATT

AATTTTAACAGGATTATTCTTAGCAATACACTATACTGCTGATATTGAAACAGCTTTTAATAGAGTAAATCACATTTATCGTGATGTTAATAATGGTTGATTCCTACGAATT

CTGTTGATAATGCTACTTTAACTCGATTCTTTACATTTCATTTTATTCTCCCTTTCATTGTATTAGCTTTAACTATAATT

CTTTTGATTTTGCTGAAGGGGAGTCTGAGTTAGTTTCAGGATTTAATGTAGAATATAGAAGAGGGGGATTTGCTTTAATT

AATTTTTTTATATATCAGAAATATATATGATTTATTGTATTTTGTTTTCCTTTAGGTCTCGTTTGATTTGCATCTTGTTT

AATTTAAGCTATAAACTTTTTGTAATAAACTAGGATTAGATACCCTATTATTAAAAATAAATATTAAAATGCTAAAGTAG

>BrK55-1

AATTTTATCTACTTTATTATTAAAAAGAGGGGCAGCTCCTTTTCATTTTTGATTCCCAGGGGTTATAGAAGGATTAAATT

AATTAATCATATTGGTTGAATATTAATAGCTATAATAAATAACGAACTTTTATGATTAACTTATTTTTTATTATATTCAATT

CAACAAATCATAAAGATATTGGAACTTTATATTTCATTTTTGGAGTATGATCTGGAATAGTCGGAACTTCTCTAAGAATT

AATTGGATTATTGGGATTTATTGTTTGAGCTCATCATATATTTACAGTAGGTATAGATGTAGATACTCGAGCTTATTTTACTTCAGCAACTATAATT

CATGATACTTATTACGTAGTTGCCCATTTTCATTACGTTTTATCTATAGGAGCTGTATTTGCTATTATAGCAGGATTTATTCATTGATACCCTTTATTAACAGGAATAGTTATAAACCCTTCATG

CTGGAATACCTCGACGATACTCAGATTTTCCTGATAGTTACTTAACTTGAAATATTATTTCTTCTTTAGGAAGAACAATT

AATTTGTCACACACAACATATATTTGCTCATTTAGTTCCTCAAGGGACTCCTCCTGTTTTAATACCTTTTATAGTATGCATTGAAACTATTAGTAATGTAATCCGACCAGGAACTTTAGCAGTACGATTGACTGCTAATATAATT

GACGAGATATTTCTCGAGAAGGAACTTTTCAAGGACTTCACACTATTCCCGTAACATTAGGATTACGATGAGGAATAATTTTATTTATTATTTCTGAAGTTTTTTTCTTTATTTCCTTCTTTTGAGCTTTTTTCCATAGTAGCTTATCTCCAACAATCGAATT

AATTGTCATTTTTCTAGAAGTCATCATTTTGGATTCGAAGCAGCTGCTTGATATTGACATTTTGTTGATGTAGTTTGACTATTCTTATATATTTCAATT

AATTTTAGGAATATATTCTTATAAAATGTTTGATCAAGGATGAAGTGAATATTTTGGAGGTCAGATATTATATAATCAAT

ATTATTCAATAACAGGGGATTTTAATAGAACTACATTGAATATGTTAAATGATAAGGGGTGAGCTATATCTTTTAGAATT

AATTCCTTTTTCTTCTTGATTACCTGCAGCTATGGCAGCACCGACTCCTGTTTCTGCTTTAGTACATTCTTCTACATTAG

NNNNNNNNNNNNNNNNNNNNNNNNNNNNNNNNNNNNNNNNNNNNNNNNNNNNNNNNNNNNNNNNNNNNNNNNNNNNNNNN

CTTTACATTCAATATCTATTGTAATAACTTTTTTATTTGATTGAATAAGTTTAATATTTATATCTTTTGTTTTATTAATT

AATTTTTATTAGATTTACTTTATTTATTTTAAGATTAAAGTTTTTATTAATAGATTTAGTATATTTTATTGAATGAGAAATT

GAGGATATCAACCTGAACGTTTACAGGCGGGGATTTATTTATTATTTTATACTTTACTGGCTTCGTTGCCGTTATTAATT

AATTATGAAAGTTACTTTAGTATATTTTTTTTAACTTTTTGTGTTTGTGAAGGGGTTCTAGGGCTATCTATTTTAGTTTC

AATTATTATATTTATTTTTGGAAGTATTGTTTTTATTTCTAGTCGTAAGCATTTACTTTGTACTTTATTGAGATTAGAGT

AATTTTTATAGGAGGAATATTAGTTTTATTCATTTATGTAACTTCTCTTTCATCTAATGAAATATTTTCATTATCTATAAAACTATTTTTTTTATCTTTAAGTATAATT

AATTTTAACAGGATTATTCTTAGCAATACACTATACTGCTGATATTGAAACAGCTTTTAATAGAGTAAATCACATTTATCGTGATGTTAATAATGGTTGATTCCTACGAATT

CTGTTGATAATGCTACTTTAACTCGATTCTTTACCTTTCATTTTATTCTCCCTTTCATTGTATTAGCTTTAACTATAATT

CTTTTGATTTTGCTGAAGGGGAGTCTGAGTTAGTTTCAGGATTTAATGTAGAGTATAGAAGAGGGGGATTTGCTTTAATT

AATTTTTTTATATATCAGAAATATATATGATTTATTGTATTTTGTTTTCCTTTAGGTCTCGTTTGATTTGCATCTTGTTT

AATTTAAGCTATAAACTTTTTGTAATAAACTAGGATTAGATACCCTATTATTAAAAATAAATATTAAAATGCTAAAGTAG

>BrK55-2

AATTTTATCTACTTTATTATTAAAAAGAGGAGCAGCTCCTTTTCATTTTTGATTCCCAGGAGTTATAGAAGGATTAAATT

AATTAATCATATTGGTTGAATATTAATAGCTATAATAAATAACGAACTTTTATGATTAACTTATTTTTTATTATATTCAATT

CAACAAATCATAAAGATATTGGAACTTTATATTTCATTTTTGGAGTATGATCTGGAATAGTCGGAACTTCTCTAAGAATT

AATTGGATTATTAGGATTTATTGTTTGAGCTCATCATATATTTACAGTAGGTATAGACGTAGATACTCGAGCTTATTTTACTTCAGCAACTATAATT

CATGATACTTATTACGTAGTTGCCCATTTTCATTACGTTTTATCTATAGGAGCTGTATTTGCTATTATAGCAGGATTTATTCATTGATACCCTTTATTAACAGGAATAGTTATAAACCCTTCATG

CTGGAATACCTCGACGATACTCAGATTTTCCTGATAGCTACTTAACTTGAAATATTATTTCTTCTTTAGGAAGAACAATT

AATTTGTCACACACAACATATATTTGCTCATTTAGTTCCTCAAGGAACTCCTCCTGTTTTAATACCTTTTATAGTATGCATTGAAACTATTAGTAATGTAATCCGACCAGGAACTTTAGCAGTACGATTGACTGCTAATATAATT

GACGAGATATTTCTCGAGAAGGAACTTTTCAAGGACTTCACACTATTCCCGTAACATTAGGATTACGATGAGGAATAATTTTATTTATTATTTCTGAAGTTTTTTTCTTTATTTCCTTCTTTTGAGCTTTTTTCCATAGTAGCTTATCTCCAACAATCGAATT

AATTGTCATTTTTCTAGAAGTCATCATTTTGGATTCGAAGCAGCTGCTTGATATTGACATTTTGTTGATGTAGTTTGACTATTCTTATATATTTCAATT

AATTTTAGGAATATATTCTTATAAAATGTTTGATCAAGGATGAAGTGAATATTTTGGAGGTCAGATATTATATAATCAAT

ATTATTCAATAACAGGGGATTTTAATAGAACTACATTGAATATGTTAGATGATAAGGGGTGAACTATATCTTTTAGAATT

AATTCCTTTTTCTTCTTGATTACCTGCAGCTATGGCAGCACCGACTCCTGTTTCTGCTTTAGTACATTCTTCTACATTAG

AATTTATTTTCAAAATGTAAAATCTTATAATGCTGGTATATTAACAGCTTTATCTAATCGAATCGGGGATGTTGCTTTAT

CTTTACATTCAGTATCTATTGTAATAACTTTTTTATTTGATTGAATAAGTTTAATATTTATATCTTTTGTTTTATTAATT

AATTTTTATTAGATTTACTTTATTTATTTTAAGATTAAAGTTTTTATTAATAGATTTAGTATATTTTATTGAATGAGAAATT

GAGGATATCAACCTGAACGTTTACAGGCGGGGATTTATTTATTATTTTATACTTTACTGGCTTCGTTGCCGTTATTAATT

AATTATGAAAGTTACTTTAGTATATTTTTTTTAACTTTTTGTGTTTGTGAAGGGGTTCTAGGGCTGTCCATTTTAGTTTC

AATTATTATATTTATTTTTGGAAGTATTGTTTTTATTTCTAGTCGTAAGCATTTACTTTGTACTTTATTGAGATTAGAGT

AATTTTTATAGGAGGAATATTAGTTTTATTCATTTATGTAACTTCTCTTTCATCTAATGAAATATTTTCATTATCTATAAAACTATTTTTTTTATCTTTAAGTATAATT

AATTTTAACAGGATTATTCTTAGCAATACACTATACTGCTGATATTGAAACAGCTTTTAATAGAGTAAATCACATTTATCGTGATGTTAATAATGGTTGATTCCTACGAATT

CTGTTGATAATGCTACTTTAACTCGATTCTTTACATTTCATTTTATTCTCCCTTTCATTGTATTAGCTTTAACTATAATT

CTTTTGATTTTGCTGAAGGGGAGTCTGAGTTAGTTTCAGGATTTAATGTAGAATATAGAAGAGGGGGATTTGCTTTAATT

AATTTTTTTATATATCAGAAATATATATGATTTATTGTATTTTGTTTTCCTTTAGGTCTCGTTTGATTTGCATCTTGTTT

AATTTAAGCTATAAACTTTTTGTAATAAACTAGGATTAGATACCCTATTATTAAAAATAAATATTAAAATGCTAAAGTAG

>BrK58-1

AATTTTATCTACTTTATTATTAAAAAGAGGAGCAGCTCCTTTTCATTTTTGATTCCCAGGAGTTATAGAAGGATTAAATT

AATTAATCATATTGGTTGAATATTAATAGCTATAATAAATAACGAACTTTTATGATTAACTTATTTTTTATTATATTCAATT

CAACAAATCATAAAGATATTGGAACTTTATATTTCATTTTTGGAGTATGATCTGGAATAGTCGGAACTTCTCTAAGAATT

AATTGGATTATTAGGATTTATTGTTTGAGCTCATCATATATTTACAGTAGGTATAGACGTAGATACTCGAGCTTATTTTACTTCAGCAACTATAATT

CATGATACTTATTACGTAGTTGCCCATTTTCATTACGTTTTATCTATAGGAGCTGTATTTGCTATTATAGCAGGATTTATTCATTGATACCCTTTATTAACAGGAATAGTTATAAACCCTTCATG

CTGGAATACCTCGACGATACTCAGATTTTCCTGATAGCTACTTAACTTGAAATATTATTTCTTCTTTAGGAAGAACAATT

AATTTGTCACACACAACATATATTTGCTCATTTAGTTCCTCAAGGAACTCCTCCTGTTTTAATACCTTTTATAGTATGCATTGAAACTATTAGTAATGTAATCCGACCAGGAACTTTAGCAGTACGATTGACTGCTAATATAATT

GACGAGATATTTCTCGAGAAGGAACTTTTCAAGGACTTCACACTATTCCCGTAACATTAGGATTACGATGAGGAATAATTTTATTTATTATTTCTGAAGTTTTTTTCTTTATTTCCTTCTTTTGAGCTTTTTTCCATAGTAGCTTATCTCCAACAATCGAATT

AATTGTCATTTTTCTAGAAGTCATCATTTTGGATTCGAAGCAGCTGCTTGATATTGACATTTTGTTGATGTAGTTTGACTATTCTTATATATTTCAATT

AATTTTAGGAATATATTCTTATAAAATGTTTGATCAAGGATGAAGTGAATATTTTGGAGGTCAGATATTATATAATCAAT

ATTATTCAATAACAGGGGATTTTAATAGAACTACATTGAATATGTTAGATGATAAGGGGTGAACTATATCTTTTAGAATT

AATTCCTTTTTCTTCTTGATTACCTGCAGCTATGGCAGCACCGACTCCTGTTTCTGCTTTAGTACATTCTTCTACATTAG

AATTTATTTTCAAAATGTAAAATCTTATAATGCTGGTATATTAACAGCTTTATCTAATCGAATCGGGGATGTTGCTTTAT

CTTTACATTCAGTATCTATTGTAATAACTTTTTTATTTGATTGAATAAGTTTAATATTTATATCTTTTGTTTTATTAATT

AATTTTTATTAGATTTACTTTATTTATTTTAAGATTAAAGTTTTTATTAATAGATTTAGTATATTTTATTGAATGAGAAATT

GAGGATATCAACCTGAACGTTTACAGGCGGGGATTTATTTATTATTTTATACTTTACTGGCTTCGTTGCCGTTATTAATT

AATTATGAAAGTTACTTTAGTATATTTTTTTTAACTTTTTGTGTTTGTGAAGGGGTTCTAGGGCTGTCCATTTTAGTTTC

AATTATTATATTTATTTTTGGAAGTATTGTTTTTATTTCTAGTCGTAAGCATTTACTTTGTACTTTATTGAGATTAGAGT

AATTTTTATAGGAGGAATATTAGTTTTATTCATTTATGTAACTTCTCTTTCATCTAATGAAATATTTTCATTATCTATAAAACTATTTTTTTTATCTTTAAGTATAATT

AATTTTAACAGGATTATTCTTAGCAATACACTATACTGCTGATATTGAAACAGCTTTTAATAGAGTAAATCACATTTATCGTGATGTTAATAATGGTTGATTCCTACGAATT

CTGTTGATAATGCTACTTTAACTCGATTCTTTACATTTCATTTTATTCTCCCTTTCATTGTATTAGCTTTAACTATAATT

CTTTTGATTTTGCTGAAGGGGAGTCTGAGTTAGTTTCAGGATTTAATGTAGAATATAGAAGAGGGGGATTTGCTTTAATT

AATTTTTTTATATATCAGAAATATATATGATTTATTGTATTTTGTTTTCCTTTAGGTCTCGTTTGATTTGCATCTTGTTT

AATTTAAGCTATAAACTTTTTGTAATAAACTAGGATTAGATACCCTATTATTAAAAATAAATATTAAAATGCTAAAGTAG

>BrK58-2

AATTTTATCTACTTTATTATTAAAAAGAGGAGCAGCTCCTTTTCATTTTTGATTCCCAGGAGTTATAGAAGGATTAAATT

AATTAATCATATTGGTTGAATATTAATAGCTATAATAAATAACGAACTTTTATGATTAACTTATTTTTTATTATATTCAATT

CAACAAATCATAAAGATATTGGAACTTTATATTTCATTTTTGGAGTATGATCTGGAATAGTCGGAACTTCTCTAAGAATT

AATTGGATTATTAGGATTTATTGTTTGAGCTCATCATATATTTACAGTAGGTATAGACGTAGATACTCGAGCTTATTTTACTTCAGCAACTATAATT

CATGATACTTATTACGTAGTTGCCCATTTTCATTACGTTTTATCTATAGGAGCTGTATTTGCTATTATAGCAGGATTTATTCATTGATACCCTTTATTAACAGGAATAGTTATAAACCCTTCATG

CTGGAATACCTCGACGATACTCAGATTTTCCTGATAGCTACTTAACTTGAAATATTATTTCTTCTTTAGGAAGAACAATT

AATTTGTCACACACAACATATATTTGCTCATTTAGTTCCTCAAGGAACTCCTCCTGTTTTAATACCTTTTATAGTATGCATTGAAACTATTAGTAATGTAATCCGACCAGGAACTTTAGCAGTACGATTGACTGCTAATATAATT

GACGAGATATTTCTCGAGAAGGAACTTTTCAAGGACTTCACACTATTCCCGTAACATTAGGATTACGATGAGGAATAATTTTATTTATTATTTCTGAAGTTTTTTTCTTTATTTCCTTCTTTTGAGCTTTTTTCCATAGTAGCTTATCTCCAACAATCGAATT

AATTGTCATTTTTCTAGAAGTCATCATTTTGGATTCGAAGCAGCTGCTTGATATTGACATTTTGTTGATGTAGTTTGACTATTCTTATATATTTCAATT

AATTTTAGGAATATATTCTTATAAAATGTTTGATCAAGGATGAAGTGAATATTTTGGAGGTCAGATATTATATAATCAAT

ATTATTCAATAACAGGGGATTTTAATAGAACTACATTGAATATGTTAGATGATAAGGGGTGAACTATATCTTTTAGAATT

AATTCCTTTTTCTTCTTGATTACCTGCAGCTATGGCAGCACCGACTCCTGTTTCTGCTTTAGTACATTCTTCTACATTAG

AATTTATTTTCAAAATGTAAAATCTTATAATGCTGGTATATTAACAGCTTTATCTAATCGAATCGGGGATGTTGCTTTAT

NNNNNNNNNNNNNNNNNNNNNNNNNNNNNNNNNNNNNNNNNNNNNNNNNNNNNNNNNNNNNNNNNNNNNNNNNNNNNNNN

AATTTTTATTAGATTTACTTTATTTATTTTAAGATTAAAGTTTTTATTAATAGATTTAGTATATTTTATTGAATGAGAAATT

GAGGATATCAACCTGAACGTTTACAGGCGGGGATTTATTTATTATTTTATACTTTACTGGCTTCGTTGCCGTTATTAATT

NATTATGAAAGTTACTTTAGTATATTTTTTTTAACTTTTTGTGTTTGTGAAGGGGTTCTAGGGCTGTCCATTTTAGTTTC

AATTATTATATTTATTTTTGGAAGTATTGTTTTTATTTCTAGTCGTAAGCATTTACTTTGTACTTTATTGAGATTAGAGT

AATTTTTATAGGAGGAATATTAGTTTTATTCATTTATGTAACTTCTCTTTCATCTAATGAAATATTTTCATTATCTATAAAACTATTTTTTTTATCTTTAAGTATAATT

AATTTTAACAGGATTATTCTTAGCAATACACTATACTGCTGATATTGAAACAGCTTTTAATAGAGTAAATCACATTTATCGTGATGTTAATAATGGTTGATTCCTACGAATT

CTGTTGATAATGCTACTTTAACTCGATTCTTTACATTTCATTTTATTCTCCCTTTCATTGTATTAGCTTTAACTATAATT

CTTTTGATTTTGCTGAAGGGGAGTCTGAGTTAGTTTCAGGATTTAATGTAGAATATAGAAGAGGGGGATTTGCTTTAATT

AATTTTTTTATATATCAGAAATATATATGATTTATTGTATTTTGTTTTCCTTTAGGTCTCGTTTGATTTGCATCTTGTTT

AATTTAAGCTATAAACTTTTTGTAATAAACTAGGATTAGATACCCTATTATTAAAAATAAATATTAAAATGCTAAAGTAG

>BrK60-1

AATTTTATCTACTTTATTATTAAAAAGAGGAGCAGCTCCTTTTCATTTTTGATTCCCAGGAGTTATAGAAGGATTAAATT

AATTAATCATATTGGTTGAATATTAATAGCTATAATAAATAACGAACTTTTATGATTAACTTATTTTTTATTATATTCAATT

CAACAAATCATAAAGATATTGGAACTTTATATTTCATTTTTGGAGTATGATCTGGAATAGTCGGAACTTCTCTAAGAATT

AATTGGATTATTAGGATTTATTGTTTGAGCTCATCATATATTTACAGTAGGTATAGACGTAGATACTCGAGCTTATTTTANNNNNNNNNNNNNNNNN

CATGATACTTATTACGTAGTTGCCCATTTTCATTACGTTTTATCTATAGGAGCTGTATTTGCTATTATAGCAGGATTTATTCATTGATACCCTTTATTAACAGGAATAGTTATAAACCCTTCATG

CTGGAATACCTCGACGATACTCAGATTTTCCTGATAGCTACTTAACTTGAAATATTATTTCTTCTTTAGGAAGAACAATT

AATTTGTCACACACAACATATATTTGCTCATTTAGTTCCTCAAGGAACTCCTCCTGTTTTAATACCTTTTATAGTATGCATTGAAACTATTAGTAATGTAATCCGACCAGGAACTTTAGCAGTACGATTGACTGCTAATATAATT

GACGAGATATTTCTCGAGAAGGAACTTTTCAAGGACTTCACACTATTCCCGTAACATTAGGATTACGATGAGGAATAATTTTATTTATTATTTCTGAAGTTTTTTTCTTTATTTCCTTCTTTTGAGCTTTTTTCCATAGTAGCTTATCTCCAACAATCGAATT

AATTGTCATTTTTCTAGAAGTCATCATTTTGGATTCGAAGCAGCTGCTTGATATTGACATTTTGTTGATGTAGTTTGACTATTCTTATATATTTCAATN

AATTTTAGGAATATATTCTTATAAAATGTTTGATCAAGGATGAAGTGAATATTTTGGAGGTCAGATATTATATAATCAAT

ATTATTCAATAACAGGGGATTTTAATAGAACTACATTGAATATGTTAGATGATAAGGGGTGAACTATATCTTTTAGAATT

AATTCCTTTTTCTTCTTGATTACCTGCAGCTATGGCAGCACCGACTCCTGTTTCTGCTTTAGTACATTCTTCTACATTAG

AATTTATTTTCAAAATGTAAAATCTTATAATGCTGGTATATTAACAGCTTTATCTAATCGAATCGGGGATGTTGCTTTAT

CTTTACATTCAGTATCTATTGTAATAACTTTTTTATTTGATTGAATAAGTTTAATATTTATATCTTTTGTTTTATTAATT

AATTTTTATTAGATTTACTTTATTTATTTTAAGATTAAAGTTTTTATTAATAGATTTAGTATATTTTATTGAATGAGAAATT

GAGGATATCAACCTGAACGTTTACAGGCGGGGATTTATTTATTATTTTATACTTTACTGGCTTCGTTGCCGTTATTAATT

AATTATGAAAGTTACTTTAGTATATTTTTTTTAACTTTTTGTGTTTGTGAAGGGGTTCTAGGGCTATCCATTTTAGTTTC

AATTATTATATTTATTTTTGGAAGTATTGTTTTTATTTCTAGTCGTAAGCATTTACTTTGTACTTTATTGAGATTAGAGT

AATTTTTATAGGAGGAATATTAGTTTTATTCATTTATGTAACTTCTCTTTCATCTAATGAAATATTTTCATTATCTATAAAACTATTTTTTTTATCTTTAAGTATAATT

AATTTTAACAGGATTATTCTTAGCAATACACTATACTGCTGATATTGAAACAGCTTTTAATAGAGTAAATCACATTTATCGTGATGTTAATAATGGTTGATTCCTACGAATT

CTGTTGATAATGCTACTTTAACTCGATTCTTTACATTTCATTTTATTCTCCCTTTCATTGTATTAGCTTTAACTATAATT

CTTTTGATTTTGCTGAAGGGGAGTCTGAGTTAGTTTCAGGATTTAATGTAGAATATAGAAGAGGGGGATTTGCTTTAATT

AATTTTTTTATATATCAGAAATATATATGATTTATTGTATTTTGTTTTCCTTTAGGTCTCGTTTGATTTGCATCTTGTTT

AATTTAAGCTATAAACTTTTTGTAATAAACTAGGATTAGATACCCTATTATTAAAAATAAATATTAAAATGCTAAAGTAG

>BrK60-2

AATTTTATCTACTTTATTATTAAAAAGAGGAGCAGCTCCTTTTCATTTTTGATTCCCAGGAGTTATAGAAGGATTAAATT

AATTAATCATATTGGTTGAATATTAATAGCTATAATAAATAACGAACTTTTATGATTAACTTATTTTTTATTATATTCAATT

CAACAAATCATAAAGATATTGGAACTTTATATTTCATTTTTGGAGTATGATCTGGAATAGTCGGAACTTCTCTAAGAATT

AATTGGATTATTAGGATTTATTGTTTGAGCTCATCATATATTTACAGTAGGTATAGACGTAGATACTCGAGCTTATTTTACTTCAGCAACTATAATT

CATGATACTTATTACGTAGTTGCCCATTTTCATTACGTTTTATCTATAGGAGCTGTATTTGCTATTATAGCAGGATTTATTCATTGATACCCTTTATTAACAGGAATAGTTATAAACCCTTCATG

CTGGAATACCTCGACGATACTCAGATTTTCCTGATAGCTACTTAACTTGAAATATTATTTCTTCTTTAGGAAGAACAATT

AATTTGTCACACACAACATATATTTGCTCATTTAGTTCCTCAAGGAACTCCTCCTGTTTTAATACCTTTTATAGTATGCATTGAAACTATTAGTAATGTAATCCGACCAGGAACTTTAGCAGTACGATTGACTGCTAATATAATT

GACGAGATATTTCTCGAGAAGGAACTTTTCAAGGACTTCACACTATTCCCGTAACATTAGGATTACGATGAGGAATAATTTTATTTATTATTTCTGAAGTTTTTTTCTTTATTTCCTTCTTTTGAGCTTTTTTCCATAGTAGCTTATCTCCAACAATCGAATT

AATTGTCATTTTTCTAGAAGTCATCATTTTGGATTCGAAGCAGCTGCTTGATATTGACATTTTGTTGATGTAGTTTGACTATTCTTATATATTTCAATT

AATTTTAGGAATATATTCTTATAAAATGTTTGATCAAGGATGAAGTGAATATTTTGGAGGTCAGATATTATATAATCAAT

ATTATTCAATAACAGGGGATTTTAATAGAACTACATTGAATATGTTAGATGATAAGGGGTGAACTATATCTTTTAGAATT

AATTCCTTTTTCTTCTTGATTACCTGCAGCTATGGCAGCACCGACTCCTGTTTCTGCTTTAGTACATTCTTCTACATTAG

AATTTATTTTCAAAATGTAAAATCTTATAATGCTGGTATATTAACAGCTTTATCTAATCGAATCGGGGATGTTGCTTTAT

CTTTACATTCAGTATCTATTGTAATAACTTTTTTATTTGATTGAATAAGTTTAATATTTATATCTTTTGTTTTATTAATT

AATTTTTATTAGATTTACTTTATTTATTTTAAGATTAAAGTTTTTATTAATAGATTTAGTATATTTTATTGAATGAGAAATT

GAGGATATCAACCTGAACGTTTACAGGCGGGGATTTATTTATTATTTTATACTTTACTGGCTTCGTTGCCGTTATTAATT

AATTATGAAAGTTACTTTAGTATATTTTTTTTAACTTTTTGTGTTTGTGAAGGGGTTCTAGGGCTGTCCATTTTAGTTTC

AATTATTATATTTATTTTTGGAAGTATTGTTTTTATTTCTAGTCGTAAGCATTTACTTTGTACTTTATTGAGATTAGAGT

AATTTTTATAGGAGGAATATTAGTTTTATTCATTTATGTAACTTCTCTTTCATCTAATGAAATATTTTCATTATCTATAAAACTATTTTTTTTATCTTTAAGTATAATT

AATTTTAACAGGATTATTCTTAGCAATACACTATACTGCTGATATTGAAACAGCTTTTAATAGAGTAAATCACATTTATCGTGATGTTAATAATGGTTGATTCCTACGAATT

CTGTTGATAATGCTACTTTAACTCGATTCTTTACATTTCATTTTATTCTCCCTTTCATTGTATTAGCTTTAACTATAATT

CTTTTGATTTTGCTGAAGGGGAGTCTGAGTTAGTTTCAGGATTTAATGTAGAATATAGAAGAGGGGGATTTGCTTTAATT

AATTTTTTTATATATCAGAAATATATATGATTTATTGTATTTTGTTTTCCTTTAGGTCTCGTTTGATTTGCATCTTGTTT

AATTTAAGCTATAAACTTTTTGTAATAAACTAGGATTAGATACCCTATTATTAAAAATAAATATTAAAATGCTAAAGTAG

>BrM02-1

AATTTTATCTACTTTATTATTAAAAAGAGGAGCAGCTCCTTTTCATTTTTGATTCCCAGGAGTTATAGAAGGATTAAATT

AATTAATCATATTGGTTGAATATTAATAGCTATAATAAATAACGAACTTTTATGATTAACTTATTTTTTATTATATTCAATT

CAACAAATCATAAAGATATTGGAACTTTATATTTCATTTTTGGAGTATGATCTGGAATAGTCGGAACTTCTCTAAGAATT

AATTGGATTATTAGGATTTATTGTTTGAGCTCATCATATATTTACAGTAGGTATAGACGTAGATACTCGAGCTTATTTTACTTCAGCAACTATAATT

CATGATACTTATTACGTAGTTGCCCATTTTCATTACGTTTTATCTATAGGAGCTGTATTTGCTATTATAGCAGGATTTATTCATTGATACCCTTTATTAACAGGAATAGTTATAAACCCTTCATG

CTGGAATACCTCGACGATACTCAGATTTTCCTGATAGCTACTTAACTTGAAATATTATTTCTTCTTTAGGAAGAACAATT

AATTTGTCACACACAACATATATTTGCTCATTTAGTTCCTCAAGGAACTCCTCCTGTTTTAATACCTTTTATAGTATGCATTGAAACTATTAGTAATGTAATCCGACCAGGAACTTTAGCAGTACGATTGACTGCTAATATAATT

GACGAGATATTTCTCGAGAAGGAACTTTTCAAGGACTTCACACTATTCCCGTAACATTAGGATTACGATGAGGAATAATTTTATTTATTATTTCTGAAGTTTTTTTCTTTATTTCCTTCTTTTGAGCTTTTTTCCATAGTAGCTTATCTCCAACAATCGAATT

AATTGTCATTTTTCTAGAAGTCATCATTTTGGATTCGAAGCAGCTGCTTGATATTGACATTTTGTTGATGTAGTTTGACTATTCTTATATATTTCAATT

AATTTTAGGAATATATTCTTATAAAATGTTTGATCAAGGATGAAGTGAATATTTTGGAGGTCAGATATTATATAATCAAT

ATTATTCAATAACAGGGGATTTTAATAGAACTACATTGAATATGTTAGATGATAAGGGGTGAACTATATCTTTTAGAATT

AATTCCTTTTTCTTCTTGATTACCTGCAGCTATGGCAGCACCGACTCCTGTTTCTGCTTTAGTACATTCTTCTACATTAG

AATTTATTTTCAAAATGTAAAATCTTATAATGCTGGTATATTAACAGCTTTATCTAATCGAATCGGGGATGTTGCTTTAT

CTTTACATTCAGTATCTATTGTAATAACTTTTTTATTTGATTGAATAAGTTTAATATTTATATCTTTTGTTTTATTAATT

AATTTTTATTAGATTTACTTTATTTATTTTAAGATTAAAGTTTTTATTAATAGATTTAGTATATTTTATTGAATGAGAAATT

GAGGATATCAACCTGAACGTTTACAGGCGGGGATTTATTTATTATTTTATACTTTACTGGCTTCGTTGCCGTTATTAATT

AATTATGAAAGTTACTTTAGTATATTTTTTTTAACTTTTTGTGTTTGTGAAGGGGTTCTAGGGCTGTCCATTTTAGTTTC

AATTATTATATTTATTTTTGGAGGTATTGTTTTTATTTCTAGTCGTAAGCATTTACTTTGTACTTTATTGAGATTAGAGT

AATTTTTATAGGAGGAATATTAGTTTTATTCATTTATGTAACTTCTCTTTCATCTAATGAAATATTTTCATTATCTATAAAACTATTTTTTTTATCTTTAAGTATAATT

AATTTTAACAGGATTATTCTTAGCAATACACTATACTGCTGATATTGAAACAGCTTTTAATAGAGTAAATCACATTTATCGTGATGTTAATAATGGTTGATTCCTACGAATT

CTGTTGATAATGCTACTTTAACTCGATTCTTTACATTTCATTTTATTCTCCCTTTCATTGTATTAGCTTTAACTATAATT

CTTTTGATTTTGCTGAAGGGGAGTCTGAGTTAGTTTCAGGATTTAATGTAGAATATAGAAGAGGGGGATTTGCTTTAATT

AATTTTTTTATATATCAGAAATATATATGATTTATTGTATTTTGTTTTCCTTTAGGTCTCGTTTGATTTGCATCTTGTTT

AATTTAAGCTATAAACTTTTTGTAATAAACTAGGATTAGATACCCTATTATTAAAAATAAATATTAAAATGCTAAAGTAG

>BrM03-1

AATTTTATCTACTTTATTATTAAAAAGAGGAGCAGCTCCTTTTCATTTTTGATTCCCAGGAGTTATAGAAGGATTAAATT

AATTAATCATATTGGTTGAATATTAATAGCTATAATAAATAACGAACTTTTATGATTAACTTATTTTTTATTATATTCAATT

CAACAAATCATAAAGATATTGGAACTTTATATTTCATTTTTGGAGTATGATCTGGAATAGTCGGAACTTCTCTAAGAATT

AATTGGATTATTAGGATTTATTGTTTGAGCTCATCATATATTTACAGTAGGTATAGACGTAGATACTCGAGCTTATTTTANNNNNNNNNNNNNNNNN

CATGATACTTATTACGTAGTTGCCCATTTTCATTACGTTTTATCTATAGGAGCTGTATTTGCTATTATAGCAGGATTTATTCATTGATACCCTTTATTAACAGGAATAGTTATAAACCCTTCATG

CTGGAATACCTCGACGATACTCAGATTTTCCTGATAGCTACTTAACTTGAAATATTATTTCTTCTTTAGGAAGAACAATT

AATTTGTCACACACAACATATATTTGCTCATTTAGTTCCTCAAGGAACTCCTCCTGTTTTAATACCTTTTATAGTATGCATTGAAACTATTAGTAATGTAATCCGACCAGGAACTTTAGCAGTACGATTGACTGCTAATATAATT

GACGAGATATTTCTCGAGAAGGAACTTTTCAAGGACTTCACACTATTCCCGTAACATTAGGATTACGATGAGGAATAATTTTATTTATTATTTCTGAAGTTTTTTTCTTTATTTCCTTCTTTTGAGCTTTTTTCCATAGTAGCTTATCTCCAACAATCGAATT

NNNNNNNNNNNNNNNNNNNGTCATCATTTTGGATTCGAAGCAGCTGCTTGATATTGACATTTTGTTGATGTAGTTTGACTATTCTTATATATTTCAATT

NNNNNNNNNNNNNNNNNNNNNNNNNNNNNNNNNNNNNNNNNNNNNNNNNNNNNNNNNNNNNNNNNNNNNNNNNNNNNNNN

ATTATTCAATAACAGGGGATTTTAATAGAACTACATTGAATATGTTAGATGATAAGGGGTGAACTATATCTTTTAGAATT

AATTCCTTTTTCTTCTTGATTACCTGCAGCTATGGCAGCACCGACTCCTGTTTCTGCTTTAGTACATTCTTCTACATTAG

AATTTATTTTCAAAATGTAAAATCTTATAATGCTGGTATATTAACAGCTTTATCTAATCGAATCGGGGATGTTGCTTTAT

NNNNNNNNNNNNNNNNNNNNNNNNNNNNNNNNNNNNNNNNNNNNNNNNNNNNNNNNNNNNNNNNNNNNNNNNNNNNNNNN

AATTTTTATTAGATTTACTTTATTTATTTTAAGATTAAAGTTTTTATTAATAGATTTAGTATATTTTATTGAATGAGAAATT

GAGGATATCAACCTGAACGTTTACAGGCGGGGATTTATTTATTATTTTATACTTTACTGGCTTCGTTGCCGTTATTAATT

ANTTATGAAAGTTACTTTAGTATATTTTTTTTAACTTTTTGTGTTTGTGAAGGGGTTCTAGGGCTGTCCATTTTAGTTTC

AATTATTATATTTATTTTTGGAAGTATTGTTTTTATTTCTAGTCGTAAGCATTTACTTTGTACTTTATTGAGATTAGAGT

AATTTTTATAGGAGGAATATTAGTTTTATTCATTTATGTAACTTCTCTTTCATCTAATGAAATATTTTCATTATCTATAAAACTATTTTTTTTATCTTTAAGTATAATT

AATTTTAACAGGATTATTCTTAGCAATACACTATACTGCTGATATTGAAACAGCTTTTAATAGAGTAAATCACATTTATCGTGATGTTAATAATGGTTGATTCCTACGAATT

CTGTTGATAATGCTACTTTAACTCGATTCTTTACATTTCATTTTATTCTCCCTTTCATTGTATTAGCTTTAACTATAATT

CTTTTGATTTTGCTGAAGGGGAGTCTGAGTTAGTTTCAGGATTTAATGTAGAATATAGAAGAGGGGGATTTGCTTTAATT

AATTTTTTTATATATCAGAAATATATATGATTTATTGTATTTTGTTTTCCTTTAGGTCTCGTTTGATTTGCATCTTGTTT

AATTTAAGCTATAAACTTTTTGTAATAAACTAGGATTAGATACCCTATTATTAAAAATAAATATTAAAATGCTAAAGTAG

>BrM11-1

AATTTTATCTACTTTATTATTAAAAAGAGGAGCAGCTCCTTTTCATTTTTGATTCCCAGGAGTTATAGAAGGATTAAATT

AATTAATCATATTGGTTGAATATTAATAGCTATAATAAATAACGAACTTTTATGATTAACTTATTTTTTATTATATTCAATT

CAACAAATCATAAAGATATTGGAACTTTATATTTCATTTTTGGAGTATGATCTGGAATAGTCGGAACTTCTCTAAGAATT

AATTGGATTATTAGGATTTATTGTTTGAGCTCATCATATATTTACAGTAGGTATAGACGTAGATACTCGAGCTTATTTTANNNNNNNNNNNNNNNNN

CATGATACTTATTACGTAGTTGCCCATTTTCATTACGTTTTATCTATAGGAGCTGTATTTGCTATTATAGCAGGATTTATTCATTGATACCCTTTATTAACAGGAATAGTTATAAACCCTTCATG

CTGGAATACCTCGACGATACTCAGATTTTCCTGATAGCTACTTAACTTGAAATATTATTTCTTCTTTAGGAAGAACAATT

AATTTGTCACACACAACATATATTTGCTCATTTAGTTCCTCAAGGAACTCCTCCTGTTTTAATACCTTTTATAGTATGCATTGAAACTATTAGTAATGTAATCCGACCAGGAACTTTAGCAGTACGATTGACTGCTAATATAATT

GACGAGATATTTCTCGAGAAGGAACTTTTCAAGGACTTCACACTATTCCCGTAACATTAGGATTACGATGAGGAATAATTTTATTTATTATTTCTGAAGTTTTTTTCTTTATTTCCTTCTTTTGAGCTTTTTTCCATAGTAGCTTATCTCCAACAATCGAATT

AATTGTCATTTTTCTAGAAGTCATCATTTTGGATTCGAAGCAGCTGCTTGATATTGACATTTTGTTGATGTAGTTTGACTATTCTTATATATTTCAATT

AATTTTAGGAATATATTCTTATAAAATGTTTGATCAAGGATGAAGTGAATATTTTGGAGGTCAGATATTATATAATCAAT

ATTATTCAATAACAGGGGATTTTAATAGAACTACATTGAATATGTTAGATGATAAGGGGTGAACTATATCTTTTAGAATT

AATTCCTTTTTCTTCTTGATTACCTGCAGCTATGGCAGCACCGACTCCTGTTTCTGCTTTAGTACATTCTTCTACATTAG

AATTTATTTTCAAAATGTAAAATCTTATAATGCTGGTATATTAACAGCTTTATCTAATCGAATCGGGGATGTTGCTTTAT

CTTTACATTCAGTATCTATTGTAATAACTTTTTTATTTGATTGAATAAGTTTAATATTTATATCTTTTGTTTTATTAATT

AATTTTTATTAGATTTACTTTATTTATTTTAAGATTAAAGTTTTTATTAATAGATTTAGTATATTTTATTGAATGAGAAATT

GAGGATATCAACCTGAACGTTTACAGGCGGGGATTTATTTATTATTTTATACTTTACTGGCTTCGTTGCCGTTATTAATT

AATTATGAAAGTTACTTTAGCATATTTTTTTTAACTTTTTGTGTTTGTGAAGGGGTTCTAGGGCTGTCCATTTTAGTTTC

AATTATTATATTTATTTTTGGAAGTATTGTTTTTATTTCTAGTCGTAAGCATTTACTTTGTACTTTATTGAGATTAGAGT

AATTTTTATAGGAGGAATATTAGTTTTATTCATTTATGTAACTTCTCTTTCATCTAATGAAATATTTTCATTATCTATAAAACTATTTTTTTTATCTTTAAGTATAATT

AATTTTAACAGGATTATTCTTAGCAATACACTATACTGCTGATATTGAAACAGCTTTTAATAGAGTAAATCACATTTATCGTGATGTTAATAATGGTTGATTCCTACGAATT

CTGTTGATAATGCTACTTTAACTCGATTCTTTACATTTCATTTTATTCTCCCTTTCATTGTATTAGCTTTAACTATAATT

CTTTTGATTTTGCTGAAGGGGAGTCTGAGTTAGTTTCAGGATTTAATGTAGAATATAGAAGAGGGGGATTTGCTTTAATT

AATTTTTTTATATATCAGAAATATATATGATTTATTGTATTTTGTTTTCCTTTAGGTCTCGTTTGATTTGCATCTTGTTT

AATTTAAGCTATAAACTTTTTGTAATAAACTAGGATTAGATACCCTATTATTAAAAATAAATATTAAAATGCTAAAGTAG

>BrM17-1

AATTTTATCTACTTTATTATTAAAAAGAGGAGCAGCTCCTTTTCATTTTTGATTCCCAGGAGTTATAGAAGGATTAAATT

AATTAATCATATTGGTTGAATATTAATAGCTATAATAAATAACGAACTTTTATGATTAACTTATTTTTTATTATATTCAATT

CAACAAATCATAAAGATATTGGAACTTTATATTTCATTTTTGGAGTATGATCTGGAATAGTCGGAACTTCTCTAAGAATT

NNNNNNNNNNNNNNNNNNNNNNNNNNNNNNNNNNNNNNNNNNNNNNNNNNNNNNNNNNNNNNNNNNNNNNNNNNNNNNNNNNNNNNNNNNNNNNNNN

CATGATACTTATTACGTAGTTGCCCATTTTCATTACGTTTTATCTATAGGAGCTGTATTTGCTATTATAGCAGGATTTATTCATTGATACCCTTTATTAACAGGAATAGTTATAAACCCTTCATG

CTGGAATACCTCGACGATACTCAGATTTTCCTGATAGCTACTTAACTTGAAATATTATTTCTTCTTTAGGAAGAACAATT

AATTTGTCACACACAACATATATTTGCTCATTTAGTTCCTCAAGGAACTCCTCCTGTTTTAATACCTTTTATAGTATGCATTGAAACTATTAGTAATGTAATCCGACCAGGAACTTTAGCAGTACGATTGACTGCTAATATAATT

GACGAGATATTTCTCGAGAAGGAACTTTTCAAGGACTTCACACTATTCCCGTAACATTAGGATTACGATGAGGAATAATTTTATTTATTATTTCTGAAGTTTTTTTCTTTATTTCCTTCTTTTGAGCTTTTTTCCATAGTAGCTTATCTCCAACAATCGAATT

AATTGTCATTTTTCTAGAAGTCATCATTTTGGATTCGAAGCAGCTGCTTGATATTGACATTTTGTTGATGTAGTTTGACTATTCTTATATATTTCAATT

AATTTTAGGAATATATTCTTATAAAATGTTTGATCAAGGATGAAGTGAATATTTTGGAGGTCAGATATTATATAATCAAT

ATTATTCAATAACAGGGGATTTTAATAGAACTACATTGAATATGTTAGATGATAAGGGGTGAACTATATCTTTTAGAATT

AATTCCTTTTTCTTCTTGATTACCTGCAGCTATGGCAGCACCGACTCCTGTTTCTGCTTTAGTACATTCTTCTACATTAG

AATTTATTTTCAAAATGTAAAATCTTATAATGCTGGTATATTAACAGCTTTATCTAATCGAATCGGGGATGTTGCTTTAT

CTTTACATTCAGTATCTATTGTAATAACTTTTTTATTTGATTGAATAAGTTTAATATTTATATCTTTTGTTTTATTAATT

AATTTTTATTAGATTTACTTTATTTATTTTAAGATTAAAGTTTTTATTAATAGATTTAGTATATTTTATTGAATGAGAAATT

GAGGATATCAACCTGAACGTTTACAGGCGGGGATTTATTTATTATTTTATACTTTACTGGCTTCGTTGCCGTTATTAATT

ANTTATGAAAGTTACTTTAGTATATTTTTTTTAACTTTTTGTGTTTGTGAAGGGGTTCTAGGGCTATCTATTTTAGTTTC

AATTATTATATTTATTTTTGGAAGTATTGTTTTTATTTCTAGTCGTAAGCATTTACTTTGTACTTTATTGAGATTAGAGT

AATTTTTATAGGAGGAATATTAGTTTTATTCATTTATGTAACTTCTCTTTCATCTAATGAAATATTTTCATTATCTATAAAACTATTTTTTTTATCTTTAAGTATAATT

AATTTTAACAGGATTATTCTTAGCAATACACTATACTGCTGATATTGAAACAGCTTTTAATAGAGTAAATCACATTTATCGTGATGTTAATAATGGTTGATTCCTACGAATT

CTGTTGATAATGCTACTTTAACTCGATTCTTTACATTTCATTTTATTCTCCCTTTCATTGTATTAGCTTTAACTATAATT

CTTTTGATTTTGCTGAAGGGGAGTCTGAGTTAGTTTCAGGATTTAATGTAGAATATAGAAGAGGGGGATTTGCTTTAATT

AATTTTTTTATATATCAGAAATATATATGATTTATTGTATTTTGTTTTCCTTTAGGTCTCGTTTGATTTGCATCTTGTTT

AATTTAAGCTATAAACTTTTTGTAATAAACTAGGATTAGATACCCTATTATTAAAAATAAATATTAAAATGCTAAAGTAG

>BrM21-1

AATTTTATCTACTTTATTATTAAAAAGAGGGGCAGCTCCTTTTCATTTTTGATTCCCAGGAGTTATAGAAGGATTAAATT

AATTAATCATATTGGTTGAATATTAATAGCTATAATAAATAACGAACTTTTATGATTAACTTATTTTTTATTATATTCAATT

CAACAAATCATAAAGATATTGGAACTTTATATTTCATTTTTGGAGTATGATCCGGAATAGTCGGAACTTCTCTAAGAATT

AATTGGATTATTGGGATTTATTGTTTGAGCTCATCATATATTTACAGTAGGTATAGACGTAGATACTCGAGCTTATTTTACTTCAGCAACTATAATT

CATGATACTTATTACGTAGTTGCCCATTTTCATTATGTTTTATCTATAGGAGCTGTATTTGCTATTATAGCAGGATTTATTCATTGATACCCTTTATTAACAGGAATAGTTATAAACCCTTCATG

CTGGAATACCTCGACGATACTCAGATTTTCCCGATAGTTACTTAACTTGAAATATTATTTCTTCTTTAGGAAGAACAATT

AATTTGTCACACACAACATATATTTGCTCATTTAGTCCCTCAAGGAACTCCCCCTGTTTTAATACCTTTTATAGTATGCATTGAAACTATTAGTAATGTAATCCGACCAGGAACTTTAGCAGTACGATTAACTGCTAATATAATT

GACGAGATATTTCTCGAGAAGGAACTTTTCAAGGACTTCATACTATTCCCGTAACATTAGGATTACGATGAGGAATAATTTTATTTATTATTTCTGAAGTTTTTTTCTTTATTTCCTTCTTTTGAGCTTTTTTCCATAGTAGCTTATCCCCAACAATCGAATT

NNNNNNNNNNNNNNNNNNNGTCATCATTTTGGATTTGAAGCAGCTGCTTGATATTGACATTTTGTTGATGTAGTTTGACTATTCTTATATATTTCAATT

AATTTTAGGAATATATTCTTATAAAATGTTTGATCAAGGATGAAGTGAATATTTTGGAGGTCAGATATTATATAATCAAT

ATTATTCAATAACAGGGGATTTTAATAGAACTACATTGAATATATTAAATGATAAGGGGTGAACTATATCTTTTAGAATT

AATTCCTTTTTCTTCTTGATTACCTGCAGCTATGGCAGCACCGACTCCTGTTTCTGCTTTAGTACATTCTTCTACATTAG

AATTTATTTTCAAAATGTAAAATCTTATAATGCTGGTATATTAACAGCTTTATCTAATCGAATCGGAGATGTTGCCTTAT

CTTTACATTCAATATCTATTGTAATAACTTTTTTATTTGATTGAATAAGTTTAATATTTATATCTTTTGTTTTATTAANT

AATTTTTATTAGATTTACTTTATTTATTTTGAGATTAAAGTTTTTATTAATAGATTTAGTATATTTTATTGAATGAGAAATT

GAGGGTATCAACCTGAACGTTTACAGGCGGGGATTTATTTATTATTTTATACTTTACTGGCTTCGTTGCCGTTATTAATT

AATTATGAAAGTTACTTTAGTATATTTTTTTTAACTTTTTGTGTTTGTGAAGGGGTTCTAGGGTTATCTATTTTAGTTTC

AATTATTATATTTATTTTTGGAAGTATTGTTTTTATTTCTAGTCGTAAGCATTTACTTTGTACTTTATTGAGATTAGAGT

AATTTTTATAGGAGGAATATTAGTTTTATTCATTTATGTTACTTCTCTTTCATCTAATGAAATATTTTCATTATCTATAAAACTATTTTTTTTATCTTTAAGTATAATT

AATTTTAACAGGATTATTCTTAGCAATACACTATACTGCTGATATTGAAACAGCTTTTAATAGAGTAAATCACATTTATCGTGATGTTAATAATGGTTGATTCCTACGAATT

CTGTTGATAATGCTACTTTAACTCGATTCTTTACATTTCATTTTATTTTACCTTTCATTGTATTAGCTTTAACTATAATT

CTTTTGATTTTGCTGAAGGGGAGTCTGAGTTAGTTTCAGGATTTAATGTAGAATATAGAAGAGGGGGATTTGCTTTAATT

AATTTTTTTATATATCAGAAATATATATGATTTATTGTATTTTGTTTTCCCTTAGGTCTCGTTTGATTTGCATCTTGTTT

AATTTAAGCTATAAACTTTTTGTAATAAACTAGGATTAGATACCCTATTATTAAAAATAAATATTAAAATGCTAAAGTAG

>BrM22-1

AATTTTATCTACTTTATTATTAAAAAGAGGGGCAGCTCCTTTTCATTTTTGATTCCCAGGAGTTATAGAAGGATTAAATT

NNNNNNNNNNNNNNNNNNNNNNNNNNNNNNNNNNNNNNNNNNNNNNNNNNNNNNNNNNNNNNNNNNNNNNNNNNNNNNNNNN

CAACAAATCATAAAGATATTGGAACTTTATATTTCATTTTTGGAGTATGATCCGGAATAGTCGGAACTTCTCTAAGAATT

AATTGGATTATTGGGATTTATTGTTTGAGCTCATCATATATTTACAGTAGGTATAGACGTAGATACTCGAGCTTATTTTACTTCAGCAACTATAATT

CATGATACTTATTACGTAGTTGCCCATTTTCATTATGTTTTATCTATAGGAGCTGTATTTGCTATTATAGCAGGATTTATTCATTGATACCCTTTATTAACAGGAATAGTTATAAACCCTTCATG

CTGGAATACCTCGACGATACTCAGATTTTCCCGATAGTTACTTAACTTGAAATATTATTTCTTCTTTAGGAAGAACAATT

AATTTGTCACACACAACATATATTTGCTCATTTAGTCCCTCAAGGAACTCCCCCTGTTTTAATACCTTTTATAGTATGCATTGAAACTATTAGTAATGTAATCCGACCAGGAACTTTAGCAGTACGATTAACTGCTAATATAATT

GACGAGATATTTCTCGAGAAGGAACTTTTCAAGGACTTCATACTATTCCCGTAACATTAGGATTACGATGAGGAATAATTTTATTTATTATTTCTGAAGTTTTTTTCTTTATTTCCTTCTTTTGAGCTTTTTTCCATAGTAGCTTATCCCCAACAATCGAATT

AATTGTCATTTTTCTAGAAGTCATCATTTTGGATTTGAAGCAGCTGCTTGATATTGACATTTTGTTGATGTAGTTTGACTATTCTTATATATTTCAATT

AATTTTAGGAATATATTCTTATAAAATGTTTGATCAAGGATGAAGTGAATATTTTGGAGGTCAGATACTATATAATCAAT

ATTATTCAATAACAGGGGATTTTAATAGAACTACATTGAATATATTAAATGATAAGGGGTGAACTATATCTTTTAGAATT

AATTCCTTTTTCTTCTTGATTACCTGCAGCTATGGCAGCTCCGACTCCTGTTTCTGCTTTAGTACATTCTTCTACATTAG

AATTTATTTTCAAAATGTAAAATCTTATAATGCTGGTATATTAACAGCTTTATCTAATCGAATCGGAGATGTTGCCTTAT

CTTTACATTCAATATCTATTGTAATAACTTTTTTATTTGATTGAATAAGTTTAATATTTATATCTTTTGTTTTATTAATT

AATTTTTATTAGATTTACTTTATTTATTTTGAGATTAAAGTTTTTATTAATAGATTTAGTATATTTTATTGAATGAGAAATT

GAGGGTATCAACCTGAACGTTTACAGGCGGGGATTTATTTATTATTTTATACTTTACTGGCTTCGTTGCCGTTATTAATT

AATTATGAAAGTTACTTTAGTATATTTTTTTTAACTTTTTGTGTTTGTGAAGGGGTTCTAGGGTTATCTATTTTAGTTTC

AATTATTATATTTATTTTTGGAAGTATTGTTTTTATTTCTAGTCGTAAGCATTTACTTTGTACTTTATTGAGATTAGAGT

AATTTTTATAGGAGGAATATTAGTTTTATTCATTTATGTTACTTCTCTTTCATCTAATGAAATATTTTCATTATCTATAAAACTATTTTTTTTATCTTTAAGTATAATT

AATTTTAACAGGATTATTCTTAGCAATACACTATACTGCTGATATTGAAACAGCTTTTAATAGAGTAAATCACATTTATCGTGATGTTAATAATGGTTGATTCCTACGAATT

CTGTTGATAATGCTACTTTAACTCGATTCTTTACATTTCATTTTATTTTACCTTTCATTGTATTAGCTTTAACTATAATT

CTTTTGATTTTGCTGAAGGGGAGTCTGAGTTAGTTTCAGGATTTAATGTAGAATATAGAAGAGGGGGATTTGCTTTAATT

AATTTTTTTATATATCAGAAATATATATGATTTATTGTATTTTGTTTTCCCTTAGGTCTCGTTTGATTTGCATCTTGTTT

AATTTAAGCTATAAACTTTTTGTAATAAACTAGGATTAGATACCCTATTATTAAAAATAAATATTAAAATGCTAAAGTAG

>BrM22-2

NATTTTATCTACTTTATTATTAAAAAGAGGAGCAGCTCCTTTTCATTTTTGATTCCCAGGAGTTATAGAAGGATTAAATT

AATTAATCATATTGGTTGAATATTAATAGCTATAATAAATAACGAACTTTTATGATTAACTTATTTTTTATTATATTCAATN

CAACAAATCATAAAGATATTGGAACTTTATATTTCATTTTTGGAGTATGATCTGGAATAGTCGGAACTTCTCTAAGAATT

NATTGGATTATTAGGATTTATTGTTTGAGCTCATCATATATTTACAGTAGGTATAGACGTAGATACTCGAGCTTATTTTANNNNNNNNNNNNNNNNN

CATGATACTTATTACGTAGTTGCCCATTTTCATTACGTTTTATCTATAGGAGCTGTATTTGCTATTATAGCAGGATTTATTCATTGATACCCTTTATTAACAGGAATAGTTATAAACCCTTCATG

CTGGAATACCTCGACGATACTCAGATTTTCCTGATAGCTACTTAACTTGAAATATTATTTCTTCTTTAGGAAGAACAATT

AATTTGTCACACACAACATATATTTGCTCATTTAGTTCCTCAAGGAACTCCTCCTGTTTTAATACCTTTTATAGTATGCATTGAAACTATTAGTAATGTAATCCGACCAGGAACTTTAGCAGTACGATTGACTGCTAATATAATT

GACGAGATATTTCTCGAGAAGGAACTTTTCAAGGACTTCACACTATTCCCGTAACATTAGGATTACGATGAGGAATAATTTTATTTATTATTTCTGAAGTTTTTTTCTTTATTTCCTTCTTTTGAGCTTTTTTCCATAGTAGCTTATCTCCAACAATCGAATT

NATTGTCATTTTTCTAGAAGTCATCATTTTGGATTCGAAGCAGCTGCTTGATATTGACATTTTGTTGATGTAGTTTGACTNNNNNNNNNNNNNNNNNNN

NNNNNNNNNNNNNNNNNNNNNNNNNNNNNNNNNNNNNNNNNNNNNNNNNNNNNNNNNNNNNNNNNNNNNNNNNNNNNNNN

ATTATTCAATAACAGGGGATTTTAATAGAACTACATTGAATATGTTAGATGATAAGGGGTGAACTATATCTTTTAGAATT

AATTCCTTTTTCTTCTTGATTACCTGCAGCTATGGCAGCACCGACTCCTGTTTCTGCTTTAGTACATTCTTCTACATTAG

AATTTATTTTCAAAATGTAAAATCTTATAATGCTGGTATATTAACAGCTTTATCTAATCGAATCGGGGATGTTGCTTTAT

NNNNNNNNNNNNNNNNNNNNNNNNNNNNNNNNNNNNNNNNNNNNNNNNNNNNNNNNNNNNNNNNNNNNNNNNNNNNNNNN

AATTTTTATTAGATTTACTTTATTTATTTTAAGATTAAAGTTTTTATTAATAGATTTAGTATATTTTATTGAATGAGAAATT

GAGGATATCAACCTGAACGTTTACAGGCGGGGATTTATTTATTATTTTATACTTTACTGGCTTCGTTGCCGTTATTAATT

AATTATGAAAGTTACTTTAGTATATTTTTTTTAACTTTTTGTGTTTGTGAAGGGGTTCTAGGGCTGTCCATTTTAGTTTC

AATTATTATATTTATTTTTGGAAGTATTGTTTTTATTTCTAGTCGTAAGCATTTACTTTGTACTTTATTGAGATTAGAGT

AATTTTTATAGGAGGAATATTAGTTTTATTCATTTATGTAACTTCTCTTTCATCTAATGAAATATTTTCATTATCTATAAAACTATTTTTTTTATCTTTAAGTATAATT

AATTTTAACAGGATTATTCTTAGCAATACACTATACTGCTGATATTGAAACAGCTTTTAATAGAGTAAATCACATTTATCGTGATGTTAATAATGGTTGATTCCTACGAATT

CTGTTGATAATGCTACTTTAACTCGATTCTTTACATTTCATTTTATTCTCCCTTTCATTGTATTAGCTTTAACTATAATT

CTTTTGATTTTGCTGAAGGGGAGTCTGAGTTAGTTTCAGGATTTAATGTAGAATATAGAAGAGGGGGATTTGCTTTAATT

AATTTTTTTATATATCAGAAATATATATGATTTATTGTATTTTGTTTTCCTTTAGGTCTCGTTTGATTTGCATCTTGTTT

AATTTAAGCTATAAACTTTTTGTAATAAACTAGGATTAGATACCCTATTATTAAAAATAAATATTAAAATGCTAAAGTAG

>BrM22-3

AATTTTATCTACTTTATTATTAAAAAGAGGGGCAGCTCCTTTTCATTTTTGATTCCCAGGAGTTATAGAAGGATTAAATT

NNNNNNNNNNNNNNNNNNNNNNNNNNNNNNNNNNNNNNNNNNNNNNNNNNNNNNNNNNNNNNNNNNNNNNNNNNNNNNNNNN

CAACAAATCATAAAGATATTGGAACTTTATATTTCATTTTTGGAGTATGATCCGGAATAGTCGGAACTTCTCTAAGAATT

NNNNNNNNNNNNNNNNNNNNNNNNNNNNNNNNNNNNNNNNNNNNNNNNNNNNNNNNNNNNNNNNNNNNNNNNNNNNNNNNNNNNNNNNNNNNNNNNN

CATGATACTTATTACGTAGTTGCCCATTTTCATTATGTTTTATCTATAGGAGCTGTATTTGCTATTATAGCAGGATTTATTCATTGATACCCTTTATTAACAGGAATAGTTATAAACCCTTCATG

CTGGAATACCTCGACGATACTCAGATTTTCCCGATAGTTACTTAACTTGAAATATTATTTCTTCTTTAGGAAGAACAATT

AATTTGTCACACACAACATATATTTGCTCATTTAGTCCCTCAAGGAACTCCCCCTGTTTTAATACCTTTTATAGTATGCATTGAAACTATTAGTAATGTAATCCGACCAGGAACTTTAGCAGTACGATTAACTGCTAATATAATT

GACGAGATATTTCTCGAGAAGGAACTTTTCAAGGACTTCATACTATTCCCGTAACATTAGGATTACGATGAGGAATAATTTTATTTATTATTTCTGAAGTTTTTTTCTTTATTTCCTTCTTTTGAGCTTTTTTCCATAGTAGCTTATCCCCAACAATCGAATT

ANTTGTCATTTTTCTAGAAGTCATCATTTTGGATTTGAAGCAGCTGCTTGATATTGACATTTTGTTGATGTAGTTTGACTATTCTTATATATTTCAANT

ANTTTTAGGAATATATTCTTATAAAATGTTTGATCAAGGATGAAGTGAATATTTTGGAGGTCAGATATTATATAATCAAT

ATTATTCAATAACAGGGGATTTTAATAGAACTACATTGAATATATTAAATGATAAGGGGTGAACTATATCTTTTAGAATT

AATTCCTTTTTCTTCTTGATTACCTGCAGCTATGGCAGCACCGACTCCTGTTTCTGCTTTAGTACATTCTTCTACATTAG

AATTTATTTTCAAAATGTAAAATCTTATAATGCTGGTATATTAACAGCTTTATCTAATCGAATCGGAGATGTTGCCTTAT

CTTTACATTCAATATCTATTGTAATAACTTTTTTATTTGATTGAATAAGTTTAATATTTATATCTTTTGTTTTATTAANT

AATTTTTATTAGATTTACTTTATTTATTTTGAGATTAAAGTTTTTATTAATAGATTTAGTATATTTTATTGAATGAGAAATT

GAGGGTATCAACCTGAACGTTTACAGGCGGGGATTTATTTATTATTTTATACTTTACTGGCTTCGTTGCCGTTATTAATT

AATTATGAAAGTTACTTTAGTATATTTTTTTTAACTTTTTGTGTTTGTGAAGGGGTTCTAGGGTTATCTATTTTAGTTTC

AATTATTATATTTATTTTTGGAAGTATTGTTTTTATTTCTAGTCGTAAGCATTTACTTTGTACTTTATTGAGATTAGAGT

AATTTTTATAGGAGGAATATTAGTTTTATTCATTTATGTTACTTCTCTTTCATCTAATGAAATATTTTCATTATCTATAAAACTATTTTTTTTATCTTTAAGTATAATT

AATTTTAACAGGATTATTCTTAGCAATACACTATACTGCTGATATTGAAACAGCTTTTAATAGAGTAAATCACATTTATCGTGATGTTAATAATGGTTGATTCCTACGAATT

CTGTTGATAATGCTACTTTAACTCGATTCTTTACATTTCATTTTATTTTACCTTTCATTGTATTAGCTTTAACTATAATT

CTTTTGATTTTGCTGAAGGGGAGTCTGAGTTAGTTTCAGGATTTAATGTAGAATATAGAAGAGGGGGATTTGCTTTAATT

AATTTTTTTATATATCAGAAATATATATGATTTATTGTATTTTGTTTTCCCTTAGGTCTCGTTTGATTTGCATCTTGTTT

AATTTAAGCTATAAACTTTTTGTAATAAACTAGGATTAGATACCCTATTATTAAAAATAAATATTAAAATGCTAAAGTAG

>BrM23-1

AATTTTATCTACTTTATTATTAAAAAGAGGAGCAGCTCCTTTTCATTTTTGATTCCCAGGAGTTATAGAAGGATTAAATT

AATTAATCATATTGGTTGAATATTAATAGCTATAATAAATAACGAACTTTTATGATTAACTTATTTTTTATTATATTCAATT

CAACAAATCATAAAGATATTGGAACTTTATATTTCATTTTTGGAGTATGATCTGGAATAGTCGGAACTTCTCTAAGAATT

AATTGGATTATTGGGATTTATTGTTTGAGCTCATCATATATTTACAGTAGGTATAGATGTAGATACTCGAGCTTATTTTANNNNNNNNNNNNNNNNN

CATGATACTTATTACGTAGTTGCCCATTTTCATTACGTTTTATCTATAGGAGCTGTATTTGCTATTATAGCAGGATTTATTCATTGATACCCTTTATTAACAGGAATAGTTATAAACCCTTCATG

CTGGAATACCTCGACGATACTCAGATTTTCCTGATAGTTACTTAACTTGAAATATTATTTCTTCTTTAGGAAGAACAATT

AATTTGTCACACACAACATATATTTGCTCATTTAGTTCCTCAAGGGACTCCTCCTGTTTTAATACCTTTTATAGTATGCATTGAAACTATTAGTAATGTAATCCGACCAGGAACTTTAGCAGTACGATTGACTGCTAATATAATT

GACGAGATATTTCTCGAGAAGGAACTTTTCAAGGACTTCACACTATTCCCGTAACATTAGGATTACGATGAGGAATAATTTTATTTATTATTTCTGAAGTTTTTTTCTTTATTTCCTTCTTTTGAGCTTTTTTCCATAGTAGCTTATCTCCAACAATCGAATT

AATTGTCATTTTTCTAGAAGTCATCATTTTGGATTCGAAGCAGCTGCTTGATATTGACATTTTGTTGATGTAGTTTGACTATTCTTATATATTTCAATT

AATTTTAGGAATATATTCTTATAAAATGTTTGATCAAGGATGAAGTGAATATTTTGGAGGTCAGATATTATATAATCAAT

ATTATTCAATAACAGGGGATTTTAATAGAACTACATTGAATATGTTAAATGATAAGGGGTGAGCTATATCTTTTAGAATT

AATTCCTTTTTCTTCTTGATTACCTGCAGCTATGGCAGCACCGACTCCTGTTTCTGCTTTAGTACATTCTTCTACATTAG

AATTTATTTTCAAAATGTAAAATCTTATAATGCTGGTATATTAACAGCTTTATCTAATCGAATCGGGGATGTTGCTTTAT

CTTTACATTCAATATCTATTGTAATAACTTTTTTATTTGATTGAATAAGTTTAATATTTATATCTTTTGTTTTATTAATN

AATTTTTATTAGATTTACTTTATTTATTTTAAGATTAAAGTTTTTATTAATAGATTTAGTATATTTTATTGAATGAGAAATT

GAGGATATCAACCTGAACGTTTACAGGCGGGGATTTATTTATTATTTTATACTTTACTGGCTTCGTTGCCGTTATTAATT

AATTATGAAAGTTACTTTAGTATATTTTTTTTAACTTTTTGTGTTTGTGAAGGGGTTCTAGGGCTATCTATTTTAGTTTC

AATTATTATATTTATTTTTGGAAGTATTGTTTTTATTTCTAGTCGTAAGCATTTACTTTGTACTTTATTGAGATTAGAGT

AATTTTTATAGGAGGAATATTAGTTTTATTCATTTATGTAACTTCTCTTTCATCTAATGAAATATTTTCATTATCTATAAAACTATTTTTTTTATCTTTAAGTATAATT

AATTTTAACAGGATTATTCTTAGCAATACACTATACTGCTGATATTGAAACAGCTTTTAATAGAGTAAATCACATTTATCGTGATGTTAATAATGGTTGATTCCTACGAATT

CTGTTGATAATGCTACTTTAACTCGATTCTTTACCTTTCATTTTATTCTCCCTTTCATTGTATTAGCTTTAACTATAATT

CTTTTGATTTTGCTGAAGGGGAGTCTGAGTTAGTTTCAGGATTTAATGTAGAGTATAGAAGAGGGGGATTTGCTTTAATT

AATTTTTTTATATATCAGAAATATATATGATTTATTGTATTTTGTTTTCCTTTAGGTCTCGTTTGATTTGCATCTTGTTT

AATTTAAGCTATAAACTTTTTGTAATAAACTAGGATTAGATACCCTATTATTAAAAATAAATATTAAAATGCTAAAGTAG

>BrM23-2

AATTTTATCTACTTTATTATTAAAAAGAGGGGCAGCTCCTTTTCATTTTTGATTCCCAGGAGTTATAGAAGGATTAAATT

AATTAATCATATTGGTTGAATATTAATAGCTATAATAAATAACGAACTTTTATGATTAACTTATTTTTTATTATATTCAATT

CAACAAATCATAAAGATATTGGAACTTTATATTTCATTTTTGGAGTATGATCTGGAATAGTCGGAACTTCTCTAAGAATT

AATTGGATTATTGGGATTTATTGTTTGAGCTCATCATATATTTACAGTAGGTATAGATGTAGATACTCGAGCTTATTTTACTTCAGCAACTATAATT

CATGATACTTATTACGTAGTTGCCCATTTTCATTACGTTTTATCTATAGGAGCTGTATTTGCTATTATAGCAGGATTTATTCATTGATACCCTTTATTAACAGGAATAGTTATAAACCCTTCATG

CTGGAATACCTCGACGATACTCAGATTTTCCTGATAGTTACTTAACTTGAAATATTATTTCTTCTTTAGGAAGAACAATT

AATTTGTCACACACAACATATATTTGCTCATTTAGTTCCTCAAGGGACTCCTCCTGTTTTAATACCTTTTATAGTATGCATTGAAACTATTAGTAATGTAATCCGACCAGGAACTTTAGCAGTACGATTGACTGCTAATATAATT

GACGAGATATTTCTCGAGAAGGAACTTTTCAAGGACTTCACACTATTCCCGTAACATTAGGATTACGATGAGGAATAATTTTATTTATTATTTCTGAAGTTTTTTTCTTTATTTCCTTCTTTTGAGCTTTTTTCCATAGTAGCTTATCTCCAACAATCGAATT

AATTGTCATTTTTCTAGAAGTCATCATTTTGGATTTGAAGCAGCTGCTTGATATTGACATTTTGTTGATGTAGTTTGACTATTCTTATATATTTCAATT

AATTTTAGGAATATATTCTTATAAAATGTTTGATCAAGGATGAAGTGAATATTTTGGAGGTCAGATATTATATAATCAAT

ATTATTCAATAACAGGGGATTTTAATAGAACTACATTGAATATGTTAAATGATAAGGGGTGAGCTATATCTTTTAGAATT

AATTCCTTTTTCTTCTTGATTACCTGCAGCTATGGCAGCACCGACTCCTGTTTCTGCTTTAGTACATTCTTCTACATTAG

AATTTATTTTCAAAATGTAAAATCTTATAATGCTGGTATATTAACAGCTTTATCTAATCGAATCGGAGATGTTGCTTTAT

CTTTACATTCAATATCTATTGTAATAACTTTTTTATTTGATTGAATAAGTTTAATATTTATATCTTTTGTTTTATTAATT

AATTTTTATTAGATTTACTTTATTTATTTTAAGATTAAAGTTTTTATTAATAGATTTAGTATATTTTATTGAATGAGAAATT

GAGGATATCAACCTGAACGTTTACAGGCGGGGATTTATTTATTATTTTATACTTTACTGGCTTCGTTGCCGTTATTAATT

AATTATGAAAGTTACTTTAGTATATTTTTTTTAACTTTTTGTGTTTGTGAAGGGGTTCTAGGGCTATCTATTTTAGTTTC

AATTATTATATTTATTTTTGGAAGTATTGTTTTTATTTCTAGTCGTAAGCATTTACTTTGTACTTTATTGAGATTAGAGT

AATTTTTATAGGAGGAATATTAGTTTTATTCATTTATGTAACTTCTCTTTCATCTAATGAAATATTTTCATTATCTATAAAACTATTTTTTTTATCTTTAAGTATAATT

AATTTTAACAGGATTATTCTTAGCAATACACTATACTGCTGATATTGAAACAGCTTTTAATAGAGTAAATCACATTTATCGTGATGTTAATAATGGTTGATTCCTACGAATT

CTGTTGATAATGCTACTTTAACTCGATTCTTTACCTTTCATTTTATTCTCCCTTTCATTGTATTAGCTTTAACTATAATT

CTTTTGATTTTGCTGAAGGGGAGTCTGAGTTAGTTTCAGGATTTAATGTAGAGTATAGAAGAGGGGGATTTGCTTTAATT

AATTTTTTTATATATCAGAAATATATATGATTTATTGTATTTTGTTTTCCTTTAGGTCTCGTTTGATTTGCATCTTGTTT

AATTTAAGCTATAAACTTTTTGTAATAAACTAGGATTAGATACCCTATTATTAAAAATAAATATTAAAATGCTAAAGTAG

>BrM24-1

AATTTTATCTACTTTATTATTAAAAAGAGGAGCAGCTCCTTTTCATTTTTGATTCCCAGGAGTTATAGAAGGATTAAATT

AATTAATCATATTGGTTGAATATTAATAGCTATAATAAATAACGAACTTTTATGATTAACTTATTTTTTATTATATTCAATT

CAACAAATCATAAAGATATTGGAACTTTATATTTCATTTTTGGAGTATGATCTGGAATAGTCGGAACTTCTCTAAGAATT

AATTGGATTATTAGGATTTATTGTTTGAGCTCATCATATATTTACAGTAGGTATAGACGTAGATACTCGAGCTTATTTTACTTCAGCAACTATAATT

CATGATACTTATTACGTAGTTGCCCATTTTCATTACGTTTTATCTATAGGAGCTGTATTTGCTATTATAGCAGGATTTATTCATTGATACCCTTTATTAACAGGAATAGTTATAAACCCTTCATG

CTGGAATACCTCGACGATACTCAGATTTTCCTGATAGCTACTTAACTTGAAATATTATTTCTTCTTTAGGAAGAACAATT

AATTTGTCACACACAACATATATTTGCTCATTTAGTTCCTCAAGGAACTCCTCCTGTTTTAATACCTTTTATAGTATGCATTGAAACTATTAGTAATGTAATCCGACCAGGAACTTTAGCAGTACGATTGACTGCTAATATAATT

GACGAGATATTTCTCGAGAAGGAACTTTTCAAGGACTTCACACTATTCCCGTAACATTAGGATTACGATGAGGAATAATTTTATTTATTATTTCTGAAGTTTTTTTCTTTATTTCCTTCTTTTGAGCTTTTTTCCATAGTAGCTTATCTCCAACAATCGAATT

AATTGTCATTTTTCTAGAAGTCATCATTTTGGATTCGAAGCAGCTGCTTGATATTGACATTTTGTTGATGTAGTTTGACTATTCTTATATATTTCAATT

AATTTTAGGAATATATTCTTATAAAATGTTTGATCAAGGATGAAGTGAATATTTTGGAGGTCAGATATTATATAATCAAT

ATTATTCAATAACAGGGGATTTTAATAGAACTACATTGAATATGTTAGATGATAAGGGGTGAACTATATCTTTTAGAATT

AATTCCTTTTTCTTCTTGATTACCTGCAGCTATGGCAGCACCGACTCCTGTTTCTGCTTTAGTACATTCTTCTACATTAG

AATTTATTTTCAAAATGTAAAATCTTATAATGCTGGTATATTAACAGCTTTATCTAATCGAATCGGGGATGTTGCTTTAT

CTTTACATTCAGTATCTATTGTAATAACTTTTTTATTTGATTGAATAAGTTTAATATTTATATCTTTTGTTTTATTAATT

AATTTTTATTAGATTTACTTTATTTATTTTAAGATTAAAGTTTTTATTAATAGATTTAGTATATTTTATTGAATGAGAAATT

GAGGATATCAACCTGAACGTTTACAGGCGGGGATTTATTTATTATTTTATACTTTACTGGCTTCGTTGCCGTTATTAATT

AATTATGAAAGTTACTTTAGTATATTTTTTTTAACTTTTTGTGTTTGTGAAGGGGTTCTAGGGCTGTCCATTTTAGTTTC

AATTATTATATTTATTTTTGGAAGTATTGTTTTTATTTCTAGTCGTAAGCATTTACTTTGTACTTTATTGAGATTAGAGT

AATTTTTATAGGAGGAATATTAGTTTTATTCATTTATGTAACTTCTCTTTCATCTAATGAAATATTTTCATTATCTATAAAACTATTTTTTTTATCTTTAAGTATAATT

AATTTTAACAGGATTATTCTTAGCAATACACTATACTGCTGATATTGAAACAGCTTTTAATAGAGTAAATCACATTTATCGTGATGTTAATAATGGTTGATTCCTACGAATT

CTGTTGATAATGCTACTTTAACTCGATTCTTTACATTTCATTTTATTCTCCCTTTCATTGTATTAGCTTTAACTATAATT

CTTTTGATTTTGCTGAAGGGGAGTCTGAGTTAGTTTCAGGATTTAATGTAGAATATAGAAGAGGGGGATTTGCTTTAATT

AATTTTTTTATATATCAGAAATATATATGATTTATTGTATTTTGTTTTCCTTTAGGTCTCGTTTGATTTGCATCTTGTTT

AATTTAAGCTATAAACTTTTTGTAATAAACTAGGATTAGATACCCTATTATTAAAAATAAATATTAAAATGCTAAAGTAG

>BrM24-2

AATTTTATCTACTTTATTATTAAAAAGAGGAGCAGCTCCTTTTCATTTTTGATTCCCAGGAGTTATAGAAGGATTAAATT

AATTAATCATATTGGTTGAATATTAATAGCTATAATAAATAACGAACTTTTATGATTAACTTATTTTTTATTATATTCAATT

CAACAAATCATAAAGATATTGGAACTTTATATTTCATTTTTGGAGTATGATCTGGAATAGTCGGAACTTCTCTAAGAATT

AATTGGATTATTAGGATTTATTGTTTGAGCTCATCATATATTTACAGTAGGTATAGACGTAGATACTCGAGCTTATTTTACTTCAGCAACTATAATT

CATGATACTTATTACGTAGTTGCCCATTTTCATTACGTTTTATCTATAGGAGCTGTATTTGCTATTATAGCAGGATTTATTCATTGATACCCTTTATTAACAGGAATAGTTATAAACCCTTCATG

CTGGAATACCTCGACGATACTCAGATTTTCCTGATAGCTACTTAACTTGAAATATTATTTCTTCTTTAGGAAGAACAATT

AATTTGTCACACACAACATATATTTGCTCATTTAGTTCCTCAAGGAACTCCTCCTGTTTTAATACCTTTTATAGTATGCATTGAAACTATTAGTAATGTAATCCGACCAGGAACTTTAGCAGTACGATTGACTGCTAATATAATT

GACGAGATATTTCTCGAGAAGGAACTTTTCAAGGACTTCACACTATTCCCGTAACATTAGGATTACGATGAGGAATAATTTTATTTATTATTTCTGAAGTTTTTTTCTTTATTTCCTTCTTTTGAGCTTTTTTCCATAGTAGCTTATCTCCAACAATCGAATT

AATTGTCATTTTTCTAGAAGTCATCATTTTGGATTCGAAGCAGCTGCTTGATATTGACATTTTGTTGATGTAGTTTGACTATTCTTATATATTTCAATT

AATTTTAGGAATATATTCTTATAAAATGTTTGATCAAGGATGAAGTGAATATTTTGGAGGTCAGATATTATATAATCAAT

ATTATTCAATAACAGGGGATTTTAATAGAACTACATTGAATATGTTAGATGATAAGGGGTGAACTATATCTTTTAGAATT

AATTCCTTTTTCTTCTTGATTACCTGCAGCTATGGCAGCACCGACTCCTGTTTCTGCTTTAGTACATTCTTCTACATTAG

AATTTATTTTCAAAATGTAAAATCTTATAATGCTGGTATATTAACAGCTTTATCTAATCGAATCGGGGATGTTGCTTTAT

CTTTACATTCAGTATCTATTGTAATAACTTTTTTATTTGATTGAATAAGTTTAATATTTATATCTTTTGTTTTATTAATT

AATTTTTATTAGATTTACTTTATTTATTTTAAGATTAAAGTTTTTATTAATAGATTTAGTATATTTTATTGAATGAGAAATT

GAGGATATCAACCTGAACGTTTACAGGCGGGGATTTATTTATTATTTTATACTTTACTGGCTTCGTTGCCGTTATTAATT

AATTATGAAAGTTACTTTAGTATATTTTTTTTAACTTTTTGTGTTTGTGAAGGGGTTCTAGGGCTGTCCATTTTAGTTTC

AATTATTATATTTATTTTTGGAAGTATTGTTTTTATTTCTAGTCGTAAGCATTTACTTTGTACTTTATTGAGATTAGAGT

AATTTTTATAGGAGGAATATTAGTTTTATTCATTTATGTAACTTCTCTTTCATCTAATGAAATATTTTCATTATCTATAAAACTATTTTTTTTATCTTTAAGTATAATT

AATTTTAACAGGATTATTCTTAGCAATACACTATACTGCTGATATTGAAACAGCTTTTAATAGAGTAAATCACATTTATCGTGATGTTAATAATGGTTGATTCCTACGAATT

CTGTTGATAATGCTACTTTAACTCGATTCTTTACATTTCATTTTATTCTCCCTTTCATTGTATTAGCTTTAACTATAATT

CTTTTGATTTTGCTGAAGGGGAGTCTGAGTTAGTTTCAGGATTTAATGTAGAATATAGAAGAGGGGGATTTGCTTTAATT

AATTTTTTTATATATCAGAAATATATATGATTTATTGTATTTTGTTTTCCTTTAGGTCTCGTTTGATTTGCATCTTGTTT

AATTTAAGCTATAAACTTTTTGTAATAAACTAGGATTAGATACCCTATTATTAAAAATAAATATTAAAATGCTAAAGTAG

>BrM26-1

AATTTTATCTACTTTATTATTAAAAAGAGGAGCAGCTCCTTTTCATTTTTGATTCCCAGGAGTTATAGAAGGATTAAATT

AATTAATCATATTGGTTGAATATTAATAGCTATAATAAATAACGAACTTTTATGATTAACTTATTTTTTATTATATTCAATT

CAACAAATCATAAAGATATTGGAACTTTATATTTCATTTTTGGAGTATGATCTGGAATAGTCGGAACTTCTCTAAGAATT

AATTGGATTATTAGGATTTATTGTTTGAGCTCATCATATATTTACAGTAGGTATAGACGTAGATACTCGAGCTTATTTTACTTCAGCAACTATAATT

CATGATACTTATTACGTAGTTGCCCATTTTCATTACGTTTTATCTATAGGAGCTGTATTTGCTATTATAGCAGGATTTATTCATTGATACCCTTTATTAACAGGAATAGTTATAAACCCTTCATG

CTGGAATACCTCGACGATACTCAGATTTTCCTGATAGCTACTTAACTTGAAATATTATTTCTTCTTTAGGAAGAACAATT

AATTTGTCACACACAACATATATTTGCTCATTTAGTTCCTCAAGGAACTCCTCCTGTTTTAATACCTTTTATAGTATGCATTGAAACTATTAGTAATGTAATCCGACCAGGAACTTTAGCAGTACGATTGACTGCTAATATAATT

GACGAGATATTTCTCGAGAAGGAACTTTTCAAGGACTTCACACTATTCCCGTAACATTAGGATTACGATGAGGAATAATTTTATTTATTATTTCTGAAGTTTTTTTCTTTATTTCCTTCTTTTGAGCTTTTTTCCATAGTAGCTTATCTCCAACAATCGAATT

AATTGTCATTTTTCTAGAAGTCATCATTTTGGATTCGAAGCAGCTGCTTGATATTGACATTTTGTTGATGTAGTTTGACTATTCTTATATATTTCAATT

AATTTTAGGAATATATTCTTATAAAATGTTTGATCAAGGATGAAGTGAATATTTTGGAGGTCAGATATTATATAATCAAT

ATTATTCAATAACAGGGGATTTTAATAGAACTACATTGAATATGTTAGATGATAAGGGGTGAACTATATCTTTTAGAATT

AATTCCTTTTTCTTCTTGATTACCTGCAGCTATGGCAGCACCGACTCCTGTTTCTGCTTTAGTACATTCTTCTACATTAG

AATTTATTTTCAAAATGTAAAATCTTATAATGCTGGTATATTAACAGCTTTATCTAATCGAATCGGGGATGTTGCTTTAT

CTTTACATTCAGTATCTATTGTAATAACTTTTTTATTTGATTGAATAAGTTTAATATTTATATCTTTTGTTTTATTAATT

AATTTTTATTAGATTTACTTTATTTATTTTAAGATTAAAGTTTTTATTAATAGATTTAGTATATTTTATTGAATGAGAAATT

GAGGATATCAACCTGAACGTTTACAGGCGGGGATTTATTTATTATTTTATACTTTACTGGCTTCGTTGCCGTTATTAATT

AATTATGAAAGTTACTTTAGTATATTTTTTTTAACTTTGTGTGTTTGTGAAGGGGTTCTAGGGCTGTCCATTTTAGTTTC

AATTATTATATTTATTTTTGGAAGTATTGTTTTTATTTCTAGTCGTAAGCATTTACTTTGTACTTTATTGAGATTAGAGT

AATTTTTATAGGAGGAATATTAGTTTTATTCATTTATGTAACTTCTCTTTCATCTAATGAAATATTTTCATTATCTATAAAACTATTTTTTTTATCTTTAAGTATAATT

AATTTTAACAGGATTATTCTTAGCAATACACTATACTGCTGATATTGAAACAGCTTTTAATAGAGTAAATCACATTTATCGTGATGTTAATAATGGTTGATTCCTACGAATT

CTGTTGATAATGCTACTTTAACTCGATTCTTTACATTTCATTTTATTCTCCCTTTCATTGTATTAGCTTTAACTATAATT

CTTTTGATTTTGCTGAAGGGGAGTCTGAGTTAGTTTCAGGATTTAATGTAGAATATAGAAGAGGGGGATTTGCTTTAATT

AATTTTTTTATATATCAGAAATATATATGATTTATTGTATTTTGTTTTCCTTTAGGTCTCGTTTGATTTGCATCTTGTTT

AATTTAAGCTATAAACTTTTTGTAATAAACTAGGATTAGATACCCTATTATTAAAAATAAATATTAAAATGCTAAAGTAG

>BrM26-2

AATTTTATCTACTTTATTATTAAAAAGAGGAGCAGCTCCTTTTCATTTTTGATTCCCAGGAGTTATAGAAGGATTAAATT

AATTAATCATATTGGTTGAATATTAATAGCTATAATAAATAACGAACTTTTATGATTAACTTATTTTTTATTATATTCAATT

CAACAAATCATAAAGATATTGGAACTTTATATTTCATTTTTGGAGTATGATCTGGAATAGTCGGAACTTCTCTAAGAATT

AATTGGATTATTAGGATTTATTGTTTGAGCTCATCATATATTTACAGTAGGTATAGACGTAGATACTCGAGCTTATTTTACTTCAGCAACTATAATT

CATGATACTTATTACGTAGTTGCCCATTTTCATTACGTTTTATCTATAGGAGCTGTATTTGCTATTATAGCAGGATTTATTCATTGATACCCTTTATTAACAGGAATAGTTATAAACCCTTCATG

CTGGAATACCTCGACGATACTCAGATTTTCCTGATAGCTACTTAACTTGAAATATTATTTCTTCTTTAGGAAGAACAATT

AATTTGTCACACACAACATATATTTGCTCATTTAGTTCCTCAAGGAACTCCTCCTGTTTTAATACCTTTTATAGTATGCATTGAAACTATTAGTAATGTAATCCGACCAGGAACTTTAGCAGTACGATTGACTGCTAATATAATT

GACGAGATATTTCTCGAGAAGGAACTTTTCAAGGACTTCACACTATTCCCGTAACATTAGGATTACGATGAGGAATAATTTTATTTATTATTTCTGAAGTTTTTTTCTTTATTTCCTTCTTTTGAGCTTTTTTCCATAGTAGCTTATCTCCAACAATCGAATT

AATTGTCATTTTTCTAGAAGTCATCATTTTGGATTCGAAGCAGCTGCTTGATATTGACATTTTGTTGATGTAGTTTGACTATTCTTATATATTTCAATT

AATTTTAGGAATATATTCTTATAAAATGTTTGATCAAGGATGAAGTGAATATTTTGGAGGTCAGATATTATATAATCAAT

ATTATTCAATAACAGGGGATTTTAATAGAACTACATTGAATATGTTAGATGATAAGGGGTGAACTATATCTTTTAGAATT

AATTCCTTTTTCTTCTTGATTACCTGCAGCTATGGCAGCACCGACTCCTGTTTCTGCTTTAGTACATTCTTCTACATTAG

AATTTATTTTCAAAATGTAAAATCTTATAATGCTGGTATATTAACAGCTTTATCTAATCGAATCGGGGATGTTGCTTTAT

CTTTACATTCAGTATCTATTGTAATAACTTTTTTATTTGATTGAATAAGTTTAATATTTATATCTTTTGTTTTATTAATT

AATTTTTATTAGATTTACTTTATTTATTTTAAGATTAAAGTTTTTATTAATAGATTTAGTATATTTTATTGAATGAGAAATT

GAGGATATCAACCTGAACGTTTACAGGCGGGGATTTATTTATTATTTTATACTTTACTGGCTTCGTTGCCGTTATTAATT

AATTATGAAAGTTACTTTAGTATATTTTTTTTAACTTTTTGTGTTTGTGAAGGGGTTCTAGGGCTGTCCATTTTAGTTTC

AATTATTATATTTATTTTTGGAAGTATTGTTTTTATTTCTAGTCGTAAGCATTTACTTTGTACTTTATTGAGATTAGAGT

AATTTTTATAGGAGGAATATTAGTTTTATTCATTTATGTAACTTCTCTTTCATCTAATGAAATATTTTCATTATCTATAAAACTATTTTTTTTATCTTTAAGTATAATT

AATTTTAACAGGATTATTCTTAGCAATACACTATACTGCTGATATTGAAACAGCTTTTAATAGAGTAAATCACATTTATCGTGATGTTAATAATGGTTGATTCCTACGAATT

CTGTTGATAATGCTACTTTAACTCGATTCTTTACATTTCATTTTATTCTCCCTTTCATTGTATTAGCTTTAACTATAATT

CTTTTGATTTTGCTGAAGGGGAGTCTGAGTTAGTTTCAGGATTTAATGTAGAATATAGAAGAGGGGGATTTGCTTTAATT

AATTTTTTTATATATCAGAAATATATATGATTTATTGTATTTTGTTTTCCTTTAGGTCTCGTTTGATTTGCATCTTGTTT

AATTTAAGCTATAAACTTTTTGTAATAAACTAGGATTAGATACCCTATTATTAAAAATAAATATTAAAATGCTAAAGTAG

>BrM27-1

AATTTTATCTACTTTATTATTAAAAAGAGGAGCAGCTCCTTTTCATTTTTGATTCCCAGGAGTTATAGAAGGATTAAATT

NATTAATCATATTGGTTGAATATTAATAGCTATAATAAATAACGAACTTTTATGATTAACTTATTTTTTATTATATTCAANN

CAACAAATCATAAAGATATTGGAACTTTATATTTCATTTTTGGAGTATGATCTGGAATAGTCGGAACTTCTCTAAGAATT

NATTGGATTATTAGGATTTATTGTTTGAGCTCATCATATATTTACAGTAGGTATAGACGTAGATACTCGAGCTTATTTTANNNNNNNNNNNNNNNNN

CATGATACTTATTACGTAGTTGCCCATTTTCATTACGTTTTATCTATAGGAGCTGTATTTGCTATTATAGCAGGATTTATTCATTGATACCCTTTATTAACAGGAATAGTTATAAACCCTTCATG

CTGGAATACCTCGACGATACTCAGATTTTCCTGATAGCTACTTAACTTGAAATATTATTTCTTCTTTAGGAAGAACAATT

AATTTGTCACACACAACATATATTTGCTCATTTAGTTCCTCAAGGAACTCCTCCTGTTTTAATACCTTTTATAGTATGCATTGAAACTATTAGTAATGTAATCCGACCAGGAACTTTAGCAGTACGATTGACTGCTAATATAATT

GACGAGATATTTCTCGAGAAGGAACTTTTCAAGGACTTCACACTATTCCCGTAACATTAGGATTACGATGAGGAATAATTTTATTTATTATTTCTGAAGTTTTTTTCTTTATTTCCTTCTTTTGAGCTTTTTTCCATAGTAGCTTATCTCCAACAATCGAATT

AATTGTCATTTTTCTAGAAGTCATCATTTTGGATTCGAAGCAGCTGCTTGATATTGACATTTTGTTGATGTAGTTTGACTATTCTTATATATTTCAATT

AATTTTAGGAATATATTCTTATAAAATGTTTGATCAAGGATGAAGTGAATATTTTGGAGGTCAGATATTATATAATCAAT

ATTATTCAATAACAGGGGATTTTAATAGAACTACATTGAATATGTTAGATGATAAGGGGTGAACTATATCTTTTAGAATT

AATTCCTTTTTCTTCTTGATTACCTGCAGCTATGGCAGCACCGACTCCTGTTTCTGCTTTAGTACATTCTTCTACATTAG

AATTTATTTTCAAAATGTAAAATCTTATAATGCTGGTATATTAACAGCTTTATCTAATCGAATCGGGGATGTTGCTTTAT

CTTTACATTCAGTATCTATTGTAATAACTTTTTTATTTGATTGAATAAGTTTAATATTTATATCTTTTGTTTTATTAATT

AATTTTTATTAGATTTACTTTATTTATTTTAAGATTAAAGTTTTTATTAATAGATTTAGTATATTTTATTGAATGAGAAATT

GAGGATATCAACCTGAACGTTTACAGGCGGGGATTTATTTATTATTTTATACTTTACTGGCTTCGTTGCCGTTATTAATT

NATTATGAAAGTTACTTTAGTATATTTTTTTTAACTTTTTGTGTTTGTGAAGGGGTTCTAGGGCTGTCCATTTTAGTTTC

AATTATTATATTTATTTTTGGAAGTATTGTTTTTATTTCTAGTCGTAAGCATTTACTTTGTACTTTATTGAGATTAGAGT

AATTTTTATAGGAGGAATATTAGTTTTATTCATTTATGTAACTTCTCTTTCATCTAATGAAATATTTTCATTATCTATAAAACTATTTTTTTTATCTTTAAGTATAATT

AATTTTAACAGGATTATTCTTAGCAATACACTATACTGCTGATATTGAAACAGCTTTTAATAGAGTAAATCACATTTATCGTGATGTTAATAATGGTTGATTCCTACGAATT

CTGTTGATAATGCTACTTTAACTCGATTCTTTACATTTCATTTTATTCTCCCTTTCATTGTATTAGCTTTAACTATAATT

CTTTTGATTTTGCTGAAGGGGAGTCTGAGTTAGTTTCAGGATTTAATGTAGAATATAGAAGAGGGGGATTTGCTTTAATT

AATTTTTTTATATATCAGAAATATATATGATTTATTGTATTTTGTTTTCCTTTAGGTCTCGTTTGATTTGCATCTTGTTT

AATTTAAGCTATAAACTTTTTGTAATAAACTAGGATTAGATACCCTATTATTAAAAATAAATATTAAAATGCTAAAGTAG

>BrM28-1

AATTTTATCTACTTTATTATTAAAAAGAGGAGCAGCTCCTTTTCATTTTTGATTCCCAGGAGTTATAGAAGGATTAAATT

AATTAATCATATTGGTTGAATATTAATAGCTATAATAAATAACGAACTTTTATGATTAACTTATTTTTTATTATATTCAATT

CAACAAATCATAAAGATATTGGAACTTTATATTTCATTTTTGGAGTATGATCTGGAATAGTCGGAACTTCTCTAAGAATT

AATTGGATTATTAGGATTTATTGTTTGAGCTCATCATATATTTACAGTAGGTATAGACGTAGATACTCGAGCTTATTTTACTTCAGCAACTATAATT

CATGATACTTATTACGTAGTTGCCCATTTTCATTACGTTTTATCTATAGGAGCTGTATTTGCTATTATAGCAGGATTTATTCATTGATACCCTTTATTAACAGGAATAGTTATAAACCCTTCATG

CTGGAATACCTCGACGATACTCAGATTTTCCTGATAGCTACTTAACTTGAAATATTATTTCTTCTTTAGGAAGAACAATT

AATTTGTCACACACAACATATATTTGCTCATTTAGTTCCTCAAGGAACTCCTCCTGTTTTAATACCTTTTATAGTATGCATTGAAACTATTAGTAATGTAATCCGACCAGGAACTTTAGCAGTACGATTGACTGCTAATATAATT

GACGAGATATTTCTCGAGAAGGAACTTTTCAAGGACTTCACACTATTCCCGTAACATTAGGATTACGATGAGGAATAATTTTATTTATTATTTCTGAAGTTTTTTTCTTTATTTCCTTCTTTTGAGCTTTTTTCCATAGTAGCTTATCTCCAACAATCGAATT

AATTGTCATTTTTCTAGAAGTCATCATTTTGGATTCGAAGCAGCTGCTTGATATTGACATTTTGTTGATGTAGTTTGACTATTCTTATATATTTCAATT

AATTTTAGGAATATATTCTTATAAAATGTTTGATCAAGGATGAAGTGAATATTTTGGAGGTCAGATATTATATAATCAAT

ATTATTCAATAACAGGGGATTTTAATAGAACTACATTGAATATGTTAGATGATAAGGGGTGAACTATATCTTTTAGAATT

AATTCCTTTTTCTTCTTGATTACCTGCAGCTATGGCAGCACCGACTCCTGTTTCTGCTTTAGTACATTCTTCTACATTAG

AATTTATTTTCAAAATGTAAAATCTTATAATGCTGGTATATTAACAGCTTTATCTAATCGAATCGGGGATGTTGCTTTAT

CTTTACATTCAGTATCTATTGTAATAACTTTTTTATTTGATTGAATAAGTTTAATATTTATATCTTTTGTTTTATTAATT

AATTTTTATTAGATTTACTTTATTTATTTTAAGATTAAAGTTTTTATTAATAGATTTAGTATATTTTATTGAATGAGAAATT

GAGGATATCAACCTGAACGTTTACAGGCGGGGATTTATTTATTATTTTATACTTTACTGGCTTCGTTGCCGTTATTAATT

AATTATGAAAGTTACTTTAGTATATTTTTTTTAACTTTTTGTGTTTGTGAAGGGGTTCTAGGGCTGTCCATTTTAGTTTC

AATTATTATATTTATTTTTGGAAGTATTGTTTTTATTTCTAGTCGTAAGCATTTACTTTGTACTTTATTGAGATTAGAGT

AATTTTTATAGGAGGAATATTAGTTTTATTCATTTATGTAACTTCTCTTTCATCTAATGAAATATTTTCATTATCTATAAAACTATTTTTTTTATCTTTAAGTATAATT

AATTTTAACAGGATTATTCTTAGCAATACACTATACTGCTGATATTGAAACAGCTTTTAATAGAGTAAATCACATTTATCGTGATGTTAATAATGGTTGATTCCTACGAATT

CTGTTGATAATGCTACTTTAACTCGATTCTTTACATTTCATTTTATTCTCCCTTTCATTGTATTAGCTTTAACTATAATT

CTTTTGATTTTGCTGAAGGGGAGTCTGAGTTAGTTTCAGGATTTAATGTAGAATATAGAAGAGGGGGATTTGCTTTAATT

AATTTTTTTATATATCAGAAATATATATGATTTATTGTATTTTGTTTTCCTTTAGGTCTCGTTTGATTTGCATCTTGTTT

AATTTAAGCTATAAACTTTTTGTAATAAACTAGGATTAGATACCCTATTATTAAAAATAAATATTAAAATGCTAAAGTAG

>BrM28-2

AATTTTATCTACTTTATTATTAAAAAGAGGAGCAGCTCCTTTTCATTTTTGATTCCCAGGAGTTATAGAAGGATTAAATT

AATTAATCATATTGGTTGAATATTAATAGCTATAATAAATAACGAACTTTTATGATTAACTTATTTTTTATTATATTCAATT

CAACAAATCATAAAGATATTGGAACTTTATATTTCATTTTTGGAGTATGATCTGGAATAGTCGGAACTTCTCTAAGAATT

AATTGGATTATTAGGATTTATTGTTTGAGCTCATCATATATTTACAGTAGGTATAGACGTAGATACTCGAGCTTATTTTACTTCAGCAACTATAATT

CATGATACTTATTACGTAGTTGCCCATTTTCATTACGTTTTATCTATAGGAGCTGTATTTGCTATTATAGCAGGATTTATTCATTGATACCCTTTATTAACAGGAATAGTTATAAACCCTTCATG

CTGGAATACCTCGACGATACTCAGATTTTCCTGATAGCTACTTAACTTGAAATATTATTTCTTCTTTAGGAAGAACAATT

AATTTGTCACACACAACATATATTTGCTCATTTAGTTCCTCAAGGAACTCCTCCTGTTTTAATACCTTTTATAGTATGCATTGAAACTATTAGTAATGTAATCCGACCAGGAACTTTAGCAGTACGATTGACTGCTAATATAATT

GACGAGATATTTCTCGAGAAGGAACTTTTCAAGGACTTCACACTATTCCCGTAACATTAGGATTACGATGAGGAATAATTTTATTTATTATTTCTGAAGTTTTTTTCTTTATTTCCTTCTTTTGAGCTTTTTTCCATAGTAGCTTATCTCCAACAATCGAATT

AATTGTCATTTTTCTAGAAGTCATCATTTTGGATTCGAAGCAGCTGCTTGATATTGACATTTTGTTGATGTAGTTTGACTATTCTTATATATTTCAATT

AATTTTAGGAATATATTCTTATAAAATGTTTGATCAAGGATGAAGTGAATATTTTGGAGGTCAGATATTATATAATCAAT

ATTATTCAATAACAGGGGATTTTAATAGAACTACATTGAATATGTTAGATGATAAGGGGTGAACTATATCTTTTAGAATT

AATTCCTTTTTCTTCTTGATTACCTGCAGCTATGGCAGCACCGACTCCTGTTTCTGCTTTAGTACATTCTTCTACATTAG

AATTTATTTTCAAAATGTAAAATCTTATAATGCTGGTATATTAACAGCTTTATCTAATCGAATCGGGGATGTTGCTTTAT

CTTTACATTCAGTATCTATTGTAATAACTTTTTTATTTGATTGAATAAGTTTAATATTTATATCTTTTGTTTTATTAATT

AATTTTTATTAGATTTACTTTATTTATTTTAAGATTAAAGTTTTTATTAATAGATTTAGTATATTTTATTGAATGAGAAATT

GAGGATATCAACCTGAACGTTTACAGGCGGGGATTTATTTATTATTTTATACTTTACTGGCTTCGTTGCCGTTATTAATT

AATTATGAAAGTTACTTTAGTATATTTTTTTTAACTTTTTGTGTTTGTGAAGGGGTTCTAGGGCTGTCCATTTTAGTTTC

AATTATTATATTTATTTTTGGAAGTATTGTTTTTATTTCTAGTCGTAAGCATTTACTTTGTACTTTATTGAGATTAGAGT

AATTTTTATAGGAGGAATATTAGTTTTATTCATTTATGTAACTTCTCTTTCATCTAATGAAATATTTTCATTATCTATAAAACTATTTTTTTTATCTTTAAGTATAATT

AATTTTAACAGGATTATTCTTAGCAATACACTATACTGCTGATATTGAAACAGCTTTTAATAGAGTAAATCACATTTATCGTGATGTTAATAATGGTTGATTCCTACGAATT

CTGTTGATAATGCTACTTTAACTCGATTCTTTACATTTCATTTTATTCTCCCTTTCATTGTATTAGCTTTAACTATAATT

CTTTTGATTTTGCTGAAGGGGAGTCTGAGTTAGTTTCAGGATTTAATGTAGAATATAGAAGAGGGGGATTTGCTTTAATT

AATTTTTTTATATATCAGAAATATATATGATTTATTGTATTTTGTTTTCCTTTAGGTCTCGTTTGATTTGCATCTTGTTT

AATTTAAGCTATAAACTTTTTGTAATAAACTAGGATTAGATACCCTATTATTAAAAATAAATATTAAAATGCTAAAGTAG

>BrM29-1

AATTTTATCTACTTTATTATTAAAAAGAGGAGCAGCTCCTTTTCATTTTTGATTCCCAGGAGTTATAGAAGGATTAAATT

AATTAATCATATTGGTTGAATATTAATAGCTATAATAAATAACGAACTTTTATGATTAACTTATTTTTTATTATATTCAATT

CAACAAATCATAAAGATATTGGAACTTTATATTTCATTTTTGGAGTATGATCTGGAATAGTCGGAACTTCTCTAAGAATT

AATTGGATTATTAGGATTTATTGTTTGAGCTCATCATATATTTACAGTAGGTATAGACGTAGATACTCGAGCTTATTTTACTTCAGCAACTATAATT

CATGATACTTATTACGTAGTTGCCCATTTTCATTACGTTTTATCTATAGGAGCTGTATTTGCTATTATAGCAGGATTTATTCATTGATACCCTTTATTAACAGGAATAGTTATAAACCCTTCATG

CTGGAATACCTCGACGATACTCAGATTTTCCTGATAGCTACTTAACTTGAAATATTATTTCTTCTTTAGGAAGAACAATT

AATTTGTCACACACAACATATATTTGCTCATTTAGTTCCTCAAGGAACTCCTCCTGTTTTAATACCTTTTATAGTATGCATTGAAACTATTAGTAATGTAATCCGACCAGGAACTTTAGCAGTACGATTGACTGCTAATATAATT

GACGAGATATTTCTCGAGAAGGAACTTTTCAAGGACTTCACACTATTCCCGTAACATTAGGATTACGATGAGGAATAATTTTATTTATTATTTCTGAAGTTTTTTTCTTTATTTCCTTCTTTTGAGCTTTTTTCCATAGTAGCTTATCTCCAACAATCGAATT

AATTGTCATTTTTCTAGAAGTCATCATTTTGGATTCGAAGCAGCTGCTTGATATTGACATTTTGTTGATGTAGTTTGACTATTCTTATATATTTCAATT

AATTTTAGGAATATATTCTTATAAAATGTTTGATCAAGGATGAAGTGAATATTTTGGAGGTCAGATATTATATAATCAAT

ATTATTCAATAACAGGGGATTTTAATAGAACTACATTGAATATGTTAGATGATAAGGGGTGAACTATATCTTTTAGAATT

AATTCCTTTTTCTTCTTGATTACCTGCAGCTATGGCAGCACCGACTCCTGTTTCTGCTTTAGTACATTCTTCTACATTAG

AATTTATTTTCAAAATGTAAAATCTTATAATGCTGGTATATTAACAGCTTTATCTAATCGAATCGGGGATGTTGCTTTAT

CTTTACATTCAGTATCTATTGTAATAACTTTTTTATTTGATTGAATAAGTTTAATATTTATATCTTTTGTTTTATTAATT

AATTTTTATTAGATTTACTTTATTTATTTTAAGATTAAAGTTTTTATTAATAGATTTAGTATATTTTATTGAATGAGAAATT

GAGGATATCAACCTGAACGTTTACAGGCGGGGATTTATTTATTATTTTATACTTTACTGGCTTCGTTGCCGTTATTAATT

AATTATGAAAGTTACTTTAGTATATTTTTTTTAACTTTTTGTGTTTGTGAAGGGGTTCTAGGGCTGTCCATTTTAGTTTC

AATTATTATATTTATTTTTGGAAGTATTGTTTTTATTTCTAGTCGTAAGCATTTACTTTGTACTTTATTGAGATTAGAGT

AATTTTTATAGGAGGAATATTAGTTTTATTCATTTATGTAACTTCTCTTTCATCTAATGAAATATTTTCATTATCTATAAAACTATTTTTTTTATCTTTAAGTATAATT

AATTTTAACAGGATTATTCTTAGCAATACACTATACTGCTGATATTGAAACAGCTTTTAATAGAGTAAATCACATTTATCGTGATGTTAATAATGGTTGATTCCTACGAATT

CTGTTGATAATGCTACTTTAACTCGATTCTTTACATTTCATTTTATTCTCCCTTTCATTGTATTAGCTTTAACTATAATT

CTTTTGATTTTGCTGAAGGGGAGTCTGAGTTAGTTTCAGGATTTAATGTAGAATATAGAAGAGGGGGATTTGCTTTAATT

AATTTTTTTATATATCAGAAATATATATGATTTATTGTATTTTGTTTTCCTTTAGGTCTCGTTTGATTTGCATCTTGTTT

AATTTAAGCTATAAACTTTTTGTAATAAACTAGGATTAGATACCCTATTATTAAAAATAAATATTAAAATGCTAAAGTAG

>BrM29-2

AATTTTATCTACTTTATTATTAAAAAGAGGAGCAGCTCCTTTTCATTTTTGATTCCCAGGAGTTATAGAAGGATTAAATT

AATTAATCATATTGGTTGAATATTAATAGCTATAATAAATAACGAACTTTTATGATTAACTTATTTTTTATTATATTCAATT

CAACAAATCATAAAGATATTGGAACTTTATATTTCATTTTTGGAGTATGATCTGGAATAGTCGGAACTTCTCTAAGAATT

AATTGGATTATTAGGATTTATTGTTTGAGCTCATCATATATTTACAGTAGGTATAGACGTAGATACTCGAGCTTATTTTACTTCAGCAACTATAATT

CATGATACTTATTACGTAGTTGCCCATTTTCATTACGTTTTATCTATAGGAGCTGTATTTGCTATTATAGCAGGATTTATTCATTGATACCCTTTATTAACAGGAATAGTTATAAACCCTTCATG

CTGGAATACCTCGACGATACTCAGATTTTCCTGATAGCTACTTAACTTGAAATATTATTTCTTCTTTAGGAAGAACAATT

AATTTGTCACACACAACATATATTTGCTCATTTAGTTCCTCAAGGAACTCCTCCTGTTTTAATACCTTTTATAGTATGCATTGAAACTATTAGTAATGTAATCCGACCAGGAACTTTAGCAGTACGATTGACTGCTAATATAATT

GACGAGATATTTCTCGAGAAGGAACTTTTCAAGGACTTCACACTATTCCCGTAACATTAGGATTACGATGAGGAATAATTTTATTTATTATTTCTGAAGTTTTTTTCTTTATTTCCTTCTTTTGAGCTTTTTTCCATAGTAGCTTATCTCCAACAATCGAATT

AATTGTCATTTTTCTAGAAGTCATCATTTTGGATTCGAAGCAGCTGCTTGATATTGACATTTTGTTGATGTAGTTTGACTATTCTTATATATTTCAATT

AATTTTAGGAATATATTCTTATAAAATGTTTGATCAAGGATGAAGTGAATATTTTGGAGGTCAGATATTATATAATCAAT

ATTATTCAATAACAGGGGATTTTAATAGAACTACATTGAATATGTTAGATGATAAGGGGTGAACTATATCTTTTAGAATT

AATTCCTTTTTCTTCTTGATTACCTGCAGCTATGGCAGCACCGACTCCTGTTTCTGCTTTAGTACATTCTTCTACATTAG

AATTTATTTTCAAAATGTAAAATCTTATAATGCTGGTATATTAACAGCTTTATCTAATCGAATCGGGGATGTTGCTTTAT

CTTTACATTCAGTATCTATTGTAATAACTTTTTTATTTGATTGAATAAGTTTAATATTTATATCTTTTGTTTTATTAATT

AATTTTTATTAGATTTACTTTATTTATTTTAAGATTAAAGTTTTTATTAATAGATTTAGTATATTTTATTGAATGAGAAATT

GAGGATATCAACCTGAACGTTTACAGGCGGGGATTTATTTATTATTTTATACTTTACTGGCTTCGTTGCCGTTATTAATT

AATTATGAAAGTTACTTTAGTATATTTTTTTTAACTTTTTGTGTTTGTGAAGGGGTTCTAGGGCTGTCCATTTTAGTTTC

AATTATTATATTTATTTTTGGAAGTATTGTTTTTATTTCTAGTCGTAAGCATTTACTTTGTACTTTATTGAGATTAGAGT

AATTTTTATAGGAGGAATATTAGTTTTATTCATTTATGTAACTTCTCTTTCATCTAATGAAATATTTTCATTATCTATAAAACTATTTTTTTTATCTTTAAGTATAATT

AATTTTAACAGGATTATTCTTAGCAATACACTATACTGCTGATATTGAAACAGCTTTTAATAGAGTAAATCACATTTATCGTGATGTTAATAATGGTTGATTCCTACGAATT

CTGTTGATAATGCTACTTTAACTCGATTCTTTACATTTCATTTTATTCTCCCTTTCATTGTATTAGCTTTAACTATAATT

CTTTTGATTTTGCTGAAGGGGAGTCTGAGTTAGTTTCAGGATTTAATGTAGAATATAGAAGAGGGGGATTTGCTTTAATT

AATTTTTTTATATATCAGAAATATATATGATTTATTGTATTTTGTTTTCCTTTAGGTCTCGTTTGATTTGCATCTTGTTT

AATTTAAGCTATAAACTTTTTGTAATAAACTAGGATTAGATACCCTATTATTAAAAATAAATATTAAAATGCTAAAGTAG

>BrM30-1

AATTTTATCTACTTTATTATTAAAAAGAGGAGCAGCTCCTTTTCATTTTTGATTCCCAGGAGTTATAGAAGGATTAAATT

AATTAATCATATTGGTTGAATATTAATAGCTATAATAAATAACGAACTTTTATGATTAACTTATTTTTTATTATATTCAATT

CAACAAATCATAAAGATATTGGAACTTTATATTTCATTTTTGGAGTATGATCTGGAATAGTCGGAACTTCTCTAAGAATT

AATTGGATTATTAGGATTTATTGTTTGAGCTCATCATATATTTACAGTAGGTATAGACGTAGATACTCGAGCTTATTTTACTTCAGCAACTATAATT

CATGATACTTATTACGTAGTTGCCCATTTTCATTACGTTTTATCTATAGGAGCTGTATTTGCTATTATAGCAGGATTTATTCATTGATACCCTTTATTAACAGGAATAGTTATAAACCCTTCATG

CTGGAATACCTCGACGATACTCAGATTTTCCTGATAGCTACTTAACTTGAAATATTATTTCTTCTTTAGGAAGAACAATT

AATTTGTCACACACAACATATATTTGCTCATTTAGTTCCTCAAGGAACTCCTCCTGTTTTAATACCTTTTATAGTATGCATTGAAACTATTAGTAATGTAATCCGACCAGGAACTTTAGCAGTACGATTGACTGCTAATATAATT

GACGAGATATTTCTCGAGAAGGAACTTTTCAAGGACTTCACACTATTCCCGTAACATTAGGATTACGATGAGGAATAATTTTATTTATTATTTCTGAAGTTTTTTTCTTTATTTCCTTCTTTTGAGCTTTTTTCCATAGTAGCTTATCTCCAACAATCGAATT

AATTGTCATTTTTCTAGAAGTCATCATTTTGGATTCGAAGCAGCTGCTTGATATTGACATTTTGTTGATGTAGTTTGACTATTCTTATATATTTCAATT

AATTTTAGGAATATATTCTTATAAAATGTTTGATCAAGGATGAAGTGAATATTTTGGAGGTCAGATATTATATAATCAAT

ATTATTCAATAACAGGGGATTTTAATAGAACTACATTGAATATGTTAGATGATAAGGGGTGAACTATATCTTTTAGAATT

AATTCCTTTTTCTTCTTGATTACCTGCAGCTATGGCAGCACCGACTCCTGTTTCTGCTTTAGTACATTCTTCTACATTAG

AATTTATTTTCAAAATGTAAAATCTTATAATGCTGGTATATTAACAGCTTTATCTAATCGAATCGGGGATGTTGCTTTAT

CTTTACATTCAGTATCTATTGTAATAACTTTTTTATTTGATTGAATAAGTTTAATATTTATATCTTTTGTTTTATTAATT

AATTTTTATTAGATTTACTTTATTTATTTTAAGATTAAAGTTTTTATTAATAGATTTAGTATATTTTATTGAATGAGAAATT

GAGGATATCAACCTGAACGTTTACAGGCGGGGATTTATTTATTATTTTATACTTTACTGGCTTCGTTGCCGTTATTAATT

NNNNNNNNNNNNNNNNNNNNNNNNNNNNNNNNNNNNNNNNNNNNNNNNNNNNNNNNNNNNNNNNNNNNNNNNNNNNNNNN

AATTATTATATTTATTTTTGGAAGTATTGTTTTTATTTCTAGTCGTAAGCATTTACTTTGTACTTTATTGAGATTAGAGT

AATTTTTATAGGAGGAATATTAGTTTTATTCATTTATGTAACTTCTCTTTCATCTAATGAAATATTTTCATTATCTATAAAACTATTTTTTTTATCTTTAAGTATAATT

AATTTTAACAGGATTATTCTTAGCAATACACTATACTGCTGATATTGAAACAGCTTTTAATAGAGTAAATCACATTTATCGTGATGTTAATAATGGTTGATTCCTACGAATT

CTGTTGATAATGCTACTTTAACTCGATTCTTTACATTTCATTTTATTCTCCCTTTCATTGTATTAGCTTTAACTATAATT

CTTTTGATTTTGCTGAAGGGGAGTCTGAGTTAGTTTCAGGATTTAATGTAGAATATAGAAGAGGGGGATTTGCTTTAATT

AATTTTTTTATATATCAGAAATATATATGATTTATTGTATTTTGTTTTCCTTTAGGTCTCGTTTGATTTGCATCTTGTTT

AATTTAAGCTATAAACTTTTTGTAATAAACTAGGATTAGATACCCTATTATTAAAAATAAATATTAAAATGCTAAAGTAG

>BrM30-2

AATTTTATCTACTTTATTATTAAAAAGAGGAGCAGCTCCTTTTCATTTTTGATTCCCAGGAGTTATAGAAGGATTAAATT

AATTAATCATATTGGTTGAATATTAATAGCTATAATAAATAACGAACTTTTATGATTAACTTATTTTTTATTATATTCAATT

CAACAAATCATAAAGATATTGGAACTTTATATTTCATTTTTGGAGTATGATCTGGAATAGTCGGAACTTCTCTAAGAATT

AATTGGATTATTAGGATTTATTGTTTGAGCTCATCATATATTTACAGTAGGTATAGACGTAGATACTCGAGCTTATTTTACTTCAGCAACTATAATT

CATGATACTTATTACGTAGTTGCCCATTTTCATTACGTTTTATCTATAGGAGCTGTATTTGCTATTATAGCAGGATTTATTCATTGATACCCTTTATTAACAGGAATAGTTATAAACCCTTCATG

CTGGAATACCTCGACGATACTCAGATTTTCCTGATAGCTACTTAACTTGAAATATTATTTCTTCTTTAGGAAGAACAATT

AATTTGTCACACACAACATATATTTGCTCATTTAGTTCCTCAAGGAACTCCTCCTGTTTTAATACCTTTTATAGTATGCATTGAAACTATTAGTAATGTAATCCGACCAGGAACTTTAGCAGTACGATTGACTGCTAATATAATT

GACGAGATATTTCTCGAGAAGGAACTTTTCAAGGACTTCACACTATTCCCGTAACATTAGGATTACGATGAGGAATAATTTTATTTATTATTTCTGAAGTTTTTTTCTTTATTTCCTTCTTTTGAGCTTTTTTCCATAGTAGCTTATCTCCAACAATCGAATT

AATTGTCATTTTTCTAGAAGTCATCATTTTGGATTCGAAGCAGCTGCTTGATATTGACATTTTGTTGATGTAGTTTGACTATTCTTATATATTTCAATT

AATTTTAGGAATATATTCTTATAAAATGTTTGATCAAGGATGAAGTGAATATTTTGGAGGTCAGATATTATATAATCAAT

ATTATTCAATAACAGGGGATTTTAATAGAACTACATTGAATATGTTAGATGATAAGGGGTGAACTATATCTTTTAGAATT

AATTCCTTTTTCTTCTTGATTACCTGCAGCTATGGCAGCACCGACTCCTGTTTCTGCTTTAGTACATTCTTCTACATTAG

AATTTATTTTCAAAATGTAAAATCTTATAATGCTGGTATATTAACAGCTTTATCTAATCGAATCGGGGATGTTGCTTTAT

CTTTACATTCAGTATCTATTGTAATAACTTTTTTATTTGATTGAATAAGTTTAATATTTATATCTTTTGTTTTATTAATT

AATTTTTATTAGATTTACTTTATTTATTTTAAGATTAAAGTTTTTATTAATAGATTTAGTATATTTTATTGAATGAGAAATT

GAGGATATCAACCTGAACGTTTACAGGCGGGGATTTATTTATTATTTTATACTTTACTGGCTTCGTTGCCGTTATTAATT

AATTATGAAAGTTACTTTAGTATATTTTTTTTAACTTTTTGTGTTTGTGAAGGGGTTCTAGGGCTGTCCATTTTAGTTTC

AATTATTATATTTATTTTTGGAAGTATTGTTTTTATTTCTAGTCGTAAGCATTTACTTTGTACTTTATTGAGATTAGAGT

AATTTTTATAGGAGGAATATTAGTTTTATTCATTTATGTAACTTCTCTTTCATCTAATGAAATATTTTCATTATCTATAAAACTATTTTTTTTATCTTTAAGTATAATT

AATTTTAACAGGATTATTCTTAGCAATACACTATACTGCTGATATTGAAACAGCTTTTAATAGAGTAAATCACATTTATCGTGATGTTAATAATGGTTGATTCCTACGAATT

CTGTTGATAATGCTACTTTAACTCGATTCTTTACATTTCATTTTATTCTCCCTTTCATTGTATTAGCTTTAACTATAATT

CTTTTGATTTTGCTGAAGGGGAGTCTGAGTTAGTTTCAGGATTTAATGTAGAATATAGAAGAGGGGGATTTGCTTTAATT

AATTTTTTTATATATCAGAAATATATATGATTTATTGTATTTTGTTTTCCTTTAGGTCTCGTTTGATTTGCATCTTGTTT

AATTTAAGCTATAAACTTTTTGTAATAAACTAGGATTAGATACCCTATTATTAAAAATAAATATTAAAATGCTAAAGTAG

>BrM31-1

AATTTTATCTACTTTATTATTAAAAAGAGGAGCAGCTCCTTTTCATTTTTGATTCCCAGGAGTTATAGAAGGATTAAATT

AATTAATCATATTGGTTGAATATTAATAGCTATAATAAATAACGAACTTTTATGATTAACTTATTTTTTATTATATTCAATT

CAACAAATCATAAAGATATTGGAACTTTATATTTCATTTTTGGAGTATGATCTGGAATAGTCGGAACTTCTCTAAGAATT

AATTGGATTATTAGGATTTATTGTTTGAGCTCATCATATATTTACAGTAGGTATAGACGTAGATACTCGAGCTTATTTTACTTCAGCAACTATAATT

CATGATACTTATTACGTAGTTGCCCATTTTCATTACGTTTTATCTATAGGAGCTGTATTTGCTATTATAGCAGGATTTATTCATTGATACCCTTTATTAACAGGAATAGTTATAAACCCTTCATG

CTGGAATACCTCGACGATACTCAGATTTTCCTGATAGCTACTTAACTTGAAATATTATTTCTTCTTTAGGAAGAACAATT

AATTTGTCACACACAACATATATTTGCTCATTTAGTTCCTCAAGGAACTCCTCCTGTTTTAATACCTTTTATAGTATGCATTGAAACTATTAGTAATGTAATCCGACCAGGAACTTTAGCAGTACGATTGACTGCTAATATAATT

GACGAGATATTTCTCGAGAAGGAACTTTTCAAGGACTTCACACTATTCCCGTAACATTAGGATTACGATGAGGAATAATTTTATTTATTATTTCTGAAGTTTTTTTCTTTATTTCCTTCTTTTGAGCTTTTTTCCATAGTAGCTTATCTCCAACAATCGAATT

AATTGTCATTTTTCTAGAAGTCATCATTTTGGATTCGAAGCAGCTGCTTGATATTGACATTTTGTTGATGTAGTTTGACTATTCTTATATATTTCAATT

AATTTTAGGAATATATTCTTATAAAATGTTTGATCAAGGATGAAGTGAATATTTTGGAGGTCAGATATTATATAATCAAT

ATTATTCAATAACAGGGGATTTTAATAGAACTACATTGAATATGTTAGATGATAAGGGGTGAACTATATCTTTTAGAATT

AATTCCTTTTTCTTCTTGATTACCTGCAGCTATGGCAGCACCGACTCCTGTTTCTGCTTTAGTACATTCTTCTACATTAG

AATTTATTTTCAAAATGTAAAATCTTATAATGCTGGTATATTAACAGCTTTATCTAATCGAATCGGGGATGTTGCTTTAT

CTTTACATTCAGTATCTATTGTAATAACTTTTTTATTTGATTGAATAAGTTTAATATTTATATCTTTTGTTTTATTAATT

AATTTTTATTAGATTTACTTTATTTATTTTAAGATTAAAGTTTTTATTAATAGATTTAGTATATTTTATTGAATGAGAAATT

GAGGATATCAACCTGAACGTTTACAGGCGGGGATTTATTTATTATTTTATACTTTACTGGCTTCGTTGCCGTTATTAATT

AATTATGAAAGTTACTTTAGTATATTTTTTTTAACTTTTTGTGTTTGTGAAGGGGTTCTAGGGCTGCCCATTTTAGTTTC

AATTATTATATTTATTTTTGGAAGTATTGTTTTTATTTCTAGTCGTAAGCATTTACTTTGTACTTTATTGAGATTAGAGT

AATTTTTATAGGAGGAATATTAGTTTTATTCATTTATGTAACTTCTCTTTCATCTAATGAAATATTTTCATTATCTATAAAACTATTTTTTTTATCTTTAAGTATAATT

AATTTTAACAGGATTATTCTTAGCAATACACTATACTGCTGATATTGAAACAGCTTTTAATAGAGTAAATCACATTTATCGTGATGTTAATAATGGTTGATTCCTACGAATT

CTGTTGATAATGCTACTTTAACTCGATTCTTTACATTTCATTTTATTCTCCCTTTCATTGTATTAGCTTTAACTATAATT

CTTTTGATTTTGCTGAAGGGGAGTCTGAGTTAGTTTCAGGATTTAATGTAGAATATAGAAGAGGGGGATTTGCTTTAATT

AATTTTTTTATATATCAGAAATATATATGATTTATTGTATTTTGTTTTCCTTTAGGTCTCGTTTGATTTGCATCTTGTTT

AATTTAAGCTATAAACTTTTTGTAATAAACTAGGATTAGATACCCTATTATTAAAAATAAATATTAAAATGCTAAAGTAG

>BrM31-2

AATTTTATCTACTTTATTATTAAAAAGAGGAGCAGCTCCTTTTCATTTTTGATTCCCAGGAGTTATAGAAGGATTAAATT

AATTAATCATATTGGTTGAATATTAATAGCTATAATAAATAACGAACTTTTATGATTAACTTATTTTTTATTATATTCAATT

CAACAAATCATAAAGATATTGGAACTTTATATTTCATTTTTGGAGTATGATCTGGAATAGTCGGAACTTCTCTAAGAATT

AATTGGATTATTAGGATTTATTGTTTGAGCTCATCATATATTTACAGTAGGTATAGACGTAGATACTCGAGCTTATTTTACTTCAGCAACTATAATT

CATGATACTTATTACGTAGTTGCCCATTTTCATTACGTTTTATCTATAGGAGCTGTATTTGCTATTATAGCAGGATTTATTCATTGATACCCTTTATTAACAGGAATAGTTATAAACCCTTCATG

CTGGAATACCTCGACGATACTCAGATTTTCCTGATAGCTACTTAACTTGAAATATTATTTCTTCTTTAGGAAGAACAATT

AATTTGTCACACACAACATATATTTGCTCATTTAGTTCCTCAAGGAACTCCTCCTGTTTTAATACCTTTTATAGTATGCATTGAAACTATTAGTAATGTAATCCGACCAGGAACTTTAGCAGTACGATTGACTGCTAATATAATT

GACGAGATATTTCTCGAGAAGGAACTTTTCAAGGACTTCACACTATTCCCGTAACATTAGGATTACGATGAGGAATAATTTTATTTATTATTTCTGAAGTTTTTTTCTTTATTTCCTTCTTTTGAGCTTTTTTCCATAGTAGCTTATCTCCAACAATCGAATT

AATTGTCATTTTTCTAGAAGTCATCATTTTGGATTCGAAGCAGCTGCTTGATATTGACATTTTGTTGATGTAGTTTGACTATTCTTATATATTTCAATT

AATTTTAGGAATATATTCTTATAAAATGTTTGATCAAGGATGAAGTGAATATTTTGGAGGTCAGATATTATATAATCAAT

ATTATTCAATAACAGGGGATTTTAATAGAACTACATTGAATATGTTAGATGATAAGGGGTGAACTATATCTTTTAGAATT

AATTCCTTTTTCTTCTTGATTACCTGCAGCTATGGCAGCACCGACTCCTGTTTCTGCTTTAGTACATTCTTCTACATTAG

AATTTATTTTCAAAATGTAAAATCTTATAATGCTGGTATATTAACAGCTTTATCTAATCGAATCGGGGATGTTGCTTTAT

CTTTACATTCAGTATCTATTGTAATAACTTTTTTATTTGATTGAATAAGTTTAATATTTATATCTTTTGTTTTATTAATT

AATTTTTATTAGATTTACTTTATTTATTTTAAGATTAAAGTTTTTATTAATAGATTTAGTATATTTTATTGAATGAGAAATT

GAGGATATCAACCTGAACGTTTACAGGCGGGGATTTATTTATTATTTTATACTTTACTGGCTTCGTTGCCGTTATTAATT

AATTATGAAAGTTACTTTAGTATATTTTTTTTAACTTTTTGTGTTTGTGAAGGGGTTCTAGGGCTGTCCATTTTAGTTTC

AATTATTATATTTATTTTTGGAAGTATTGTTTTTATTTCTAGTCGTAAGCATTTACTTTGTACTTTATTGAGATTAGAGT

AATTTTTATAGGAGGAATATTAGTTTTATTCATTTATGTAACTTCTCTTTCATCTAATGAAATATTTTCATTATCTATAAAACTATTTTTTTTATCTTTAAGTATAATT

AATTTTAACAGGATTATTCTTAGCAATACACTATACTGCTGATATTGAAACAGCTTTTAATAGAGTAAATCACATTTATCGTGATGTTAATAATGGTTGATTCCTACGAATT

CTGTTGATAATGCTACTTTAACTCGATTCTTTACATTTCATTTTATTCTCCCTTTCATTGTATTAGCTTTAACTATAATT

CTTTTGATTTTGCTGAAGGGGAGTCTGAGTTAGTTTCAGGATTTAATGTAGAATATAGAAGAGGGGGATTTGCTTTAATT

AATTTTTTTATATATCAGAAATATATATGATTTATTGTATTTTGTTTTCCTTTAGGTCTCGTTTGATTTGCATCTTGTTT

AATTTAAGCTATAAACTTTTTGTAATAAACTAGGATTAGATACCCTATTATTAAAAATAAATATTAAAATGCTAAAGTAG

>BrM35-1

AATTTTATCTACTTTATTATTAAAAAGAGGAGCAGCTCCTTTTCATTTTTGATTCCCAGGAGTTATAGAAGGATTAAATT

AATTAATCATATTGGTTGAATATTAATAGCTATAATAAATAACGAACTTTTATGATTAACTTATTTTTTATTATATTCAATT

CAACAAATCATAAAGATATTGGAACTTTATATTTCATTTTTGGAGTATGATCTGGAATAGTCGGAACTTCTCTAAGAATT

AATTGGATTATTAGGATTTATTGTTTGAGCTCATCATATATTTACAGTAGGTATAGACGTAGATACTCGAGCTTATTTTACTTCAGCAACTATAATT

CATGATACTTATTACGTAGTTGCCCATTTTCATTACGTTTTATCTATAGGAGCTGTATTTGCTATTATAGCAGGATTTATTCATTGATACCCTTTATTAACAGGAATAGTTATAAACCCTTCATG

CTGGAATACCTCGACGATACTCAGATTTTCCTGATAGCTACTTAACTTGAAATATTATTTCTTCTTTAGGAAGAACAATT

AATTTGTCACACACAACATATATTTGCTCATTTAGTTCCTCAAGGAACTCCTCCTGTTTTAATACCTTTTATAGTATGCATTGAAACTATTAGTAATGTAATCCGACCAGGAACTTTAGCAGTACGATTGACTGCTAATATAATT

GACGAGATATTTCTCGAGAAGGAACTTTTCAAGGACTTCACACTATTCCCGTAACATTAGGATTACGATGAGGAATAATTTTATTTATTATTTCTGAAGTTTTTTTCTTTATTTCCTTCTTTTGAGCTTTTTTCCATAGTAGCTTATCTCCAACAATCGAATT

AATTGTCATTTTTCTAGAAGTCATCATTTTGGATTCGAAGCAGCTGCTTGATATTGACATTTTGTTGATGTAGTTTGACTATTCTTATATATTTCAATT

AATTTTAGGAATATATTCTTATAAAATGTTTGATCAAGGATGAAGTGAATATTTTGGAGGTCAGATATTATATAATCAAT

ATTATTCAATAACAGGGGATTTTAATAGAACTACATTGAATATGTTAGATGATAAGGGGTGAACTATATCTTTTAGAATT

AATTCCTTTTTCTTCTTGATTACCTGCAGCTATGGCAGCACCGACTCCTGTTTCTGCTTTAGTACATTCTTCTACATTAG

AATTTATTTTCAAAATGTAAAATCTTATAATGCTGGTATATTAACAGCTTTATCTAATCGAATCGGGGATGTTGCTTTAT

CTTTACATTCAGTATCTATTGTAATAACTTTTTTATTTGATTGAATAAGTTTAATATTTATATCTTTTGTTTTATTAATT

AATTTTTATTAGATTTACTTTATTTATTTTAAGATTAAAGTTTTTATTAATAGATTTAGTATATTTTATTGAATGAGAAATT

GAGGATATCAACCTGAACGTTTACAGGCGGGGATTTATTTATTATTTTATACTTTACTGGCTTCGTTGCCGTTATTAATT

AATTATGAAAGTTACTTTAGTATATTTTTTTTAACTTTTTGTGTTTGTGAAGGGGTTCTAGGGCTGTCCATTTTAGTTTC

AATTATTATATTTATTTTTGGAAGTATTGTTTTTATTTCTAGTCGTAAGCATTTACTTTGTACTTTATTGAGATTAGAGT

AATTTTTATAGGAGGAATATTAGTTTTATTCATTTATGTAACTTCTCTTTCATCTAATGAAATATTTTCATTATCTATAAAACTATTTTTTTTATCTTTAAGTATAATT

AATTTTAACAGGATTATTCTTAGCAATACACTATACTGCTGATATTGAAACAGCTTTTAATAGAGTAAATCACATTTATCGTGATGTTAATAATGGTTGATTCCTACGAATT

CTGTTGATAATGCTACTTTAACTCGATTCTTTACATTTCATTTTATTCTCCCTTTCATTGTATTAGCTTTAACTATAATT

CTTTTGATTTTGCTGAAGGGGAGTCTGAGTTAGTTTCAGGATTTAATGTAGAATATAGAAGAGGGGGATTTGCTTTAATT

AATTTTTTTATATATCAGAAATATATATGATTTATTGTATTTTGTTTTCCTTTAGGTCTCGTTTGATTTGCATCTTGTTT

AATTTAAGCTATAAACTTTTTGTAATAAACTAGGATTAGATACCCTATTATTAAAAATAAATATTAAAATGCTAAAGTAG

>BrM35-2

AATTTTATCTACTTTATTATTAAAAAGAGGAGCAGCTCCTTTTCATTTTTGATTCCCAGGAGTTATAGAAGGATTAAATT

AATTAATCATATTGGTTGAATATTAATAGCTATAATAAATAACGAACTTTTATGATTAACTTATTTTTTATTATATTCAATT

CAACAAATCATAAAGATATTGGAACTTTATATTTCATTTTTGGAGTATGATCTGGAATAGTCGGAACTTCTCTAAGAATT

AATTGGATTATTAGGATTTATTGTTTGAGCTCATCATATATTTACAGTAGGTATAGACGTAGATACTCGAGCTTATTTTACTTCAGCAACTATAATT

CATGATACTTATTACGTAGTTGCCCATTTTCATTACGTTTTATCTATAGGAGCTGTATTTGCTATTATAGCAGGATTTATTCATTGATACCCTTTATTAACAGGAATAGTTATAAACCCTTCATG

CTGGAATACCTCGACGATACTCAGATTTTCCTGATAGCTACTTAACTTGAAATATTATTTCTTCTTTAGGAAGAACAATT

AATTTGTCACACACAACATATATTTGCTCATTTAGTTCCTCAAGGAACTCCTCCTGTTTTAATACCTTTTATAGTATGCATTGAAACTATTAGTAATGTAATCCGACCAGGAACTTTAGCAGTACGATTGACTGCTAATATAATT

GACGAGATATTTCTCGAGAAGGAACTTTTCAAGGACTTCACACTATTCCCGTAACATTAGGATTACGATGAGGAATAATTTTATTTATTATTTCTGAAGTTTTTTTCTTTATTTCCTTCTTTTGAGCTTTTTTCCATAGTAGCTTATCTCCAACAATCGAATT

AATTGTCATTTTTCTAGAAGTCATCATTTTGGATTCGAAGCAGCTGCTTGATATTGACATTTTGTTGATGTAGTTTGACTATTCTTATATATTTCAATT

AATTTTAGGAATATATTCTTATAAAATGTTTGATCAAGGATGAAGTGAATATTTTGGAGGTCAGATATTATATAATCAAT

ATTATTCAATAACAGGGGATTTTAATAGAACTACATTGAATATGTTAGATGATAAGGGGTGAACTATATCTTTTAGAATT

AATTCCTTTTTCTTCTTGATTACCTGCAGCTATGGCAGCACCGACTCCTGTTTCTGCTTTAGTACATTCTTCTACATTAG

AATTTATTTTCAAAATGTAAAATCTTATAATGCTGGTATATTAACAGCTTTATCTAATCGAATCGGGGATGTTGCTTTAT

CTTTACATTCAGTATCTATTGTAATAACTTTTTTATTTGATTGAATAAGTTTAATATTTATATCTTTTGTTTTATTAATT

AATTTTTATTAGATTTACTTTATTTATTTTAAGATTAAAGTTTTTATTAATAGATTTAGTATATTTTATTGAATGAGAAATT

GAGGATATCAACCTGAACGTTTACAGGCGGGGATTTATTTATTATTTTATACTTTACTGGCTTCGTTGCCGTTATTAATT

AATTATGAAAGTTACTTTAGTATATTTTTTTTAACTTTTTGTGTTTGTGAAGGGGTTCTAGGGCTATCTATTTTAGTTTC

AATTATTATATTTATTTTTGGAAGTATTGTTTTTATTTCTAGTCGTAAGCATTTACTTTGTACTTTATTGAGATTAGAGT

AATTTTTATAGGAGGAATATTAGTTTTATTCATTTATGTAACTTCTCTTTCATCTAATGAAATATTTTCATTATCTATAAAACTATTTTTTTTATCTTTAAGTATAATT

AATTTTAACAGGATTATTCTTAGCAATACACTATACTGCTGATATTGAAACAGCTTTTAATAGAGTAAATCACATTTATCGTGATGTTAATAATGGTTGATTCCTACGAATT

CTGTTGATAATGCTACTTTAACTCGATTCTTTACATTTCATTTTATTCTCCCTTTCATTGTATTAGCTTTAACTATAATT

CTTTTGATTTTGCTGAAGGGGAGTCTGAGTTAGTTTCAGGATTTAATGTAGAATATAGAAGAGGGGGATTTGCTTTAATT

AATTTTTTTATATATCAGAAATATATATGATTTATTGTATTTTGTTTTCCTTTAGGTCTCGTTTGATTTGCATCTTGTTT

AATTTAAGCTATAAACTTTTTGTAATAAACTAGGATTAGATACCCTATTATTAAAAATAAATATTAAAATGCTAAAGTAG

>BrM39-1

AATTTTATCTACTTTATTATTAAAAAGAGGAGCAGCTCCTTTTCATTTTTGATTCCCAGGAGTTATAGAAGGATTAAATT

AATTAATCATATTGGTTGAATATTAATAGCTATAATAAATAACGAACTTTTATGATTAACTTATTTTTTATTATATTCAATT

CAACAAATCATAAAGATATTGGAACTTTATATTTCATTTTTGGAGTATGATCTGGAATAGTCGGAACTTCTCTAAGAATT

AATTGGATTATTAGGATTTATTGTTTGAGCTCATCATATATTTACAGTAGGTATAGACGTAGATACTCGAGCTTATTTTACTTCAGCAACTATAATT

CATGATACTTATTACGTAGTTGCCCATTTTCATTACGTTTTATCTATAGGAGCTGTATTTGCTATTATAGCAGGATTTATTCATTGATACCCTTTATTAACAGGAATAGTTATAAACCCTTCATG

CTGGAATACCTCGACGATACTCAGATTTTCCTGATAGCTACTTAACTTGAAATATTATTTCTTCTTTAGGAAGAACAATT

AATTTGTCACACACAACATATATTTGCTCATTTAGTTCCTCAAGGAACTCCTCCTGTTTTAATACCTTTTATAGTATGCATTGAAACTATTAGTAATGTAATCCGACCAGGAACTTTAGCAGTACGATTGACTGCTAATATAATT

GACGAGATATTTCTCGAGAAGGAACTTTTCAAGGACTTCACACTATTCCCGTAACATTAGGATTACGATGAGGAATAATTTTATTTATTATTTCTGAAGTTTTTTTCTTTATTTCCTTCTTTTGAGCTTTTTTCCATAGTAGCTTATCTCCAACAATCGAATT

AATTGTCATTTTTCTAGAAGTCATCATTTTGGATTCGAAGCAGCTGCTTGATATTGACATTTTGTTGATGTAGTTTGACTATTCTTATATATTTCAATT

AATTTTAGGAATATATTCTTATAAAATGTTTGATCAAGGATGAAGTGAATATTTTGGAGGTCAGATATTATATAATCAAT

ATTATTCAATAACAGGGGATTTTAATAGAACTACATTGAATATGTTAGATGATAAGGGGTGAACTATATCTTTTAGAATT

AATTCCTTTTTCTTCTTGATTACCTGCAGCTATGGCAGCACCGACTCCTGTTTCTGCTTTAGTACATTCTTCTACATTAG

AATTTATTTTCAAAATGTAAAATCTTATAATGCTGGTATATTAACAGCTTTATCTAATCGAATCGGGGATGTTGCTTTAT

CTTTACATTCAGTATCTATTGTAATAACTTTTTTATTTGATTGAATAAGTTTAATATTTATATCTTTTGTTTTATTAATT

AATTTTTATTAGATTTACTTTATTTATTTTAAGATTAAAGTTTTTATTAATAGATTTAGTATATTTTATTGAATGAGAAATT

GAGGATATCAACCTGAACGTTTACAGGCGGGGATTTATTTATTATTTTATACTTTACTGGCTTCGTTGCCGTTATTAATT

AATTATGAAAGTTACTTTAGTATATTTTTTTTAACTTTTTGTGTTTGTGAAGGGGTTCTAGGGCTGTCCATTTTAGTTTC

AATTATTATATTTATTTTTGGAAGTATTGTTTTTATTTCTAGTCGTAAGCATTTACTTTGTACTTTATTGAGATTAGAGT

AATTTTTATAGGAGGAATATTAGTTTTATTCATTTATGTAACTTCTCTTTCATCTAATGAAATATTTTCATTATCTATAAAACTATTTTTTTTATCTTTAAGTATAATT

AATTTTAACAGGATTATTCTTAGCAATACACTATACTGCTGATATTGAAACAGCTTTTAATAGAGTAAATCACATTTATCGTGATGTTAATAATGGTTGATTCCTACGAATT

CTGTTGATAATGCTACTTTAACTCGATTCTTTACATTTCATTTTATTCTCCCTTTCATTGTATTAGCTTTAACTATAATT

CTTTTGATTTTGCTGAAGGGGAGTCTGAGTTAGTTTCAGGATTTAATGTAGAATATAGAAGAGGGGGATTTGCTTTAATT

AATTTTTTTATATATCAGAAATATATATGATTTATTGTATTTTGTTTTCCTTTAGGTCTCGTTTGATTTGCATCTTGTTT

AATTTAAGCTATAAACTTTTTGTAATAAACTAGGATTAGATACCCTATTATTAAAAATAAATATTAAAATGCTAAAGTAG

>BrM39-2

AATTTTATCTACTTTATTATTAAAAAGAGGAGCAGCTCCTTTTCATTTTTGATTCCCAGGAGTTATAGAAGGATTAAATT

AATTAATCATATTGGTTGAATATTAATAGCTATAATAAATAACGAACTTTTATGATTAACTTATTTTTTATTATATTCAATT

CAACAAATCATAAAGATATTGGAACTTTATATTTCATTTTTGGAGTATGATCTGGAATAGTCGGAACTTCTCTAAGAATT

AATTGGATTATTAGGATTTATTGTTTGAGCTCATCATATATTTACAGTAGGTATAGACGTAGATACTCGAGCTTATTTTACTTCAGCAACTATAATT

CATGATACTTATTACGTAGTTGCCCATTTTCATTACGTTTTATCTATAGGAGCTGTATTTGCTATTATAGCAGGATTTATTCATTGATACCCTTTATTAACAGGAATAGTTATAAACCCTTCATG

CTGGAATACCTCGACGATACTCAGATTTTCCTGATAGCTACTTAACTTGAAATATTATTTCTTCTTTAGGAAGAACAATT

AATTTGTCACACACAACATATATTTGCTCATTTAGTTCCTCAAGGAACTCCTCCTGTTTTAATACCTTTTATAGTATGCATTGAAACTATTAGTAATGTAATCCGACCAGGAACTTTAGCAGTACGATTGACTGCTAATATAATT

GACGAGATATTTCTCGAGAAGGAACTTTTCAAGGACTTCACACTATTCCCGTAACATTAGGATTACGATGAGGAATAATTTTATTTATTATTTCTGAAGTTTTTTTCTTTATTTCCTTCTTTTGAGCTTTTTTCCATAGTAGCTTATCTCCAACAATCGAATT

AATTGTCATTTTTCTAGAAGTCATCATTTTGGATTCGAAGCAGCTGCTTGATATTGACATTTTGTTGATGTAGTTTGACTATTCTTATATATTTCAATT

AATTTTAGGAATATATTCTTATAAAATGTTTGATCAAGGATGAAGTGAATATTTTGGAGGTCAGATATTATATAATCAAT

ATTATTCAATAACAGGGGATTTTAATAGAACTACATTGAATATGTTAGATGATAAGGGGTGAACTATATCTTTTAGAATT

AATTCCTTTTTCTTCTTGATTACCTGCAGCTATGGCAGCACCGACTCCTGTTTCTGCTTTAGTACATTCTTCTACATTAG

AATTTATTTTCAAAATGTAAAATCTTATAATGCTGGTATATTAACAGCTTTATCTAATCGAATCGGGGATGTTGCTTTAT

CTTTACATTCAGTATCTATTGTAATAACTTTTTTATTTGATTGAATAAGTTTAATATTTATATCTTTTGTTTTATTAATT

AATTTTTATTAGATTTACTTTATTTATTTTAAGATTAAAGTTTTTATTAATAGATTTAGTATATTTTATTGAATGAGAAATT

GAGGATATCAACCTGAACGTTTACAGGCGGGGATTTATTTATTATTTTATACTTTACTGGCTTCGTTGCCGTTATTAATT

AATTATGAAAGTTACTTTAGTATATTTTTTTTAACTTTTTGTGTTTGTGAAGGGGTTCTAGGGCTATCCATTTTAGTTTC

AATTATTATATTTATTTTTGGAAGTATTGTTTTTATTTCTAGTCGTAAGCATTTACTTTGTACTTTATTGAGATTAGAGT

AATTTTTATAGGAGGAATATTAGTTTTATTCATTTATGTAACTTCTCTTTCATCTAATGAAATATTTTCATTATCTATAAAACTATTTTTTTTATCTTTAAGTATAATT

AATTTTAACAGGATTATTCTTAGCAATACACTATACTGCTGATATTGAAACAGCTTTTAATAGAGTAAATCACATTTATCGTGATGTTAATAATGGTTGATTCCTACGAATT

CTGTTGATAATGCTACTTTAACTCGATTCTTTACATTTCATTTTATTCTCCCTTTCATTGTATTAGCTTTAACTATAATT

CTTTTGATTTTGCTGAAGGGGAGTCTGAGTTAGTTTCAGGATTTAATGTAGAATATAGAAGAGGGGGATTTGCTTTAATT

AATTTTTTTATATATCAGAAATATATATGATTTATTGTATTTTGTTTTCCTTTAGGTCTCGTTTGATTTGCATCTTGTTT

AATTTAAGCTATAAACTTTTTGTAATAAACTAGGATTAGATACCCTATTATTAAAAATAAATATTAAAATGCTAAAGTAG

>BrM42-1

AATTTTATCTACTTTATTATTAAAAAGAGGAGCAGCTCCTTTTCATTTTTGATTCCCAGGAGTTATAGAAGGATTAAATT

AATTAATCATATTGGTTGAATATTAATAGCTATAATAAATAACGAACTTTTATGATTAACTTATTTTTTATTATATTCAATT

CAACAAATCATAAAGATATTGGAACTTTATATTTCATTTTTGGAGTATGATCTGGAATAGTCGGAACTTCTCTAAGAATT

AATTGGATTATTAGGATTTATTGTTTGAGCTCATCATATATTTACAGTAGGTATAGACGTAGATACTCGAGCTTATTTTACTTCAGCAACTATAATT

CATGATACTTATTACGTAGTTGCCCATTTTCATTACGTTTTATCTATAGGAGCTGTATTTGCTATTATAGCAGGATTTATTCATTGATACCCTTTATTAACAGGAATAGTTATAAACCCTTCATG

CTGGAATACCTCGACGATACTCAGATTTTCCTGATAGCTACTTAACTTGAAATATTATTTCTTCTTTAGGAAGAACAATT

AATTTGTCACACACAACATATATTTGCTCATTTAGTTCCTCAAGGAACTCCTCCTGTTTTAATACCTTTTATAGTATGCATTGAAACTATTAGTAATGTAATCCGACCAGGAACTTTAGCAGTACGATTGACTGCTAATATAATT

GACGAGATATTTCTCGAGAAGGAACTTTTCAAGGACTTCACACTATTCCCGTAACATTAGGATTACGATGAGGAATAATTTTATTTATTATTTCTGAAGTTTTTTTCTTTATTTCCTTCTTTTGAGCTTTTTTCCATAGTAGCTTATCTCCAACAATCGAATT

AATTGTCATTTTTCTAGAAGTCATCATTTTGGATTCGAAGCAGCTGCTTGATATTGACATTTTGTTGATGTAGTTTGACTATTCTTATATATTTCAATT

AATTTTAGGAATATATTCTTATAAAATGTTTGATCAAGGATGAAGTGAATATTTTGGAGGTCAGATATTATATAATCAAT

ATTATTCAATAACAGGGGATTTTAATAGAACTACATTGAATATGTTAGATGATAAGGGGTGAACTATATCTTTTAGAATT

AATTCCTTTTTCTTCTTGATTACCTGCAGCTATGGCAGCACCGACTCCTGTTTCTGCTTTAGTACATTCTTCTACATTAG

AATTTATTTTCAAAATGTAAAATCTTATAATGCTGGTATATTAACAGCTTTATCTAATCGAATCGGGGATGTTGCTTTAT

CTTTACATTCAGTATCTATTGTAATAACTTTTTTATTTGATTGAATAAGTTTAATATTTATATCTTTTGTTTTATTAATT

AATTTTTATTAGATTTACTTTATTTATTTTAAGATTAAAGTTTTTATTAATAGATTTAGTATATTTTATTGAATGAGAAATT

GAGGATATCAACCTGAACGTTTACAGGCGGGGATTTATTTATTATTTTATACTTTACTGGCTTCGTTGCCGTTATTAATT

AATTATGAAAGTTACTTTAGTATATTTTTTTTAACTTTTTGTGTTTGTGAAGGGGTTCTAGGGCTGTCCATTTTAGTTTC

AATTATTATATTTATTTTTGGAAGTATTGTTTTTATTTCTAGTCGTAAGCATTTACTTTGTACTTTATTGAGATTAGAGT

AATTTTTATAGGAGGAATATTAGTTTTATTCATTTATGTAACTTCTCTTTCATCTAATGAAATATTTTCATTATCTATAAAACTATTTTTTTTATCTTTAAGTATAATT

AATTTTAACAGGATTATTCTTAGCAATACACTATACTGCTGATATTGAAACAGCTTTTAATAGAGTAAATCACATTTATCGTGATGTTAATAATGGTTGATTCCTACGAATT

CTGTTGATAATGCTACTTTAACTCGATTCTTTACATTTCATTTTATTCTCCCTTTCATTGTATTAGCTTTAACTATAATT

CTTTTGATTTTGCTGAAGGGGAGTCTGAGTTAGTTTCAGGATTTAATGTAGAATATAGAAGAGGGGGATTTGCTTTAATT

AATTTTTTTATATATCAGAAATATATATGATTTATTGTATTTTGTTTTCCTTTAGGTCTCGTTTGATTTGCATCTTGTTT

AATTTAAGCTATAAACTTTTTGTAATAAACTAGGATTAGATACCCTATTATTAAAAATAAATATTAAAATGCTAAAGTAG

>BrM42-2

AATTTTATCTACTTTATTATTAAAAAGAGGAGCAGCTCCTTTTCATTTTTGATTCCCAGGAGTTATAGAAGGATTAAATT

AATTAATCATATTGGTTGAATATTAATAGCTATAATAAATAACGAACTTTTATGATTAACTTATTTTTTATTATATTCAATT

CAACAAATCATAAAGATATTGGAACTTTATATTTCATTTTTGGAGTATGATCTGGAATAGTCGGAACTTCTCTAAGAATT

AATTGGATTATTAGGATTTATTGTTTGAGCTCATCATATATTTACAGTAGGTATAGACGTAGATACTCGAGCTTATTTTACTTCAGCAACTATAATT

CATGATACTTATTACGTAGTTGCCCATTTTCATTACGTTTTATCTATAGGAGCTGTATTTGCTATTATAGCAGGATTTATTCATTGATACCCTTTATTAACAGGAATAGTTATAAACCCTTCATG

CTGGAATACCTCGACGATACTCAGATTTTCCTGATAGCTACTTAACTTGAAATATTATTTCTTCTTTAGGAAGAACAATT

AATTTGTCACACACAACATATATTTGCTCATTTAGTTCCTCAAGGAACTCCTCCTGTTTTAATACCTTTTATAGTATGCATTGAAACTATTAGTAATGTAATCCGACCAGGAACTTTAGCAGTACGATTGACTGCTAATATAATT

GACGAGATATTTCTCGAGAAGGAACTTTTCAAGGACTTCACACTATTCCCGTAACATTAGGATTACGATGAGGAATAATTTTATTTATTATTTCTGAAGTTTTTTTCTTTATTTCCTTCTTTTGAGCTTTTTTCCATAGTAGCTTATCTCCAACAATCGAATT

AATTGTCATTTTTCTAGAAGTCATCATTTTGGATTCGAAGCAGCTGCTTGATATTGACATTTTGTTGATGTAGTTTGACTATTCTTATATATTTCAATT

AATTTTAGGAATATATTCTTATAAAATGTTTGATCAAGGATGAAGTGAATATTTTGGAGGTCAGATATTATATAATCAAT

ATTATTCAATAACAGGGGATTTTAATAGAACTACATTGAATATGTTAGATGATAAGGGGTGAACTATATCTTTTAGAATT

AATTCCTTTTTCTTCTTGATTACCTGCAGCTATGGCAGCACCGACTCCTGTTTCTGCTTTAGTACATTCTTCTACATTAG

AATTTATTTTCAAAATGTAAAATCTTATAATGCTGGTATATTAACAGCTTTATCTAATCGAATCGGGGATGTTGCTTTAT

CTTTACATTCAGTATCTATTGTAATAACTTTTTTATTTGATTGAATAAGTTTAATATTTATATCTTTTGTTTTATTAATT

AATTTTTATTAGATTTACTTTATTTATTTTAAGATTAAAGTTTTTATTAATAGATTTAGTATATTTTATTGAATGAGAAATT

GAGGATATCAACCTGAACGTTTACAGGCGGGGATTTATTTATTATTTTATACTTTACTGGCTTCGTTGCCGTTATTAATT

AATTATGAAAGTTACTTTAGTATATTTTTTTTAACTTTTTGTGTTTGTGAAGGGGTTCTAGGGCTGTCCATTTTAGTTTC

AATTATTATATTTATTTTTGGAAGTATTGTTTTTATTTCTAGTCGTAAGCATTTACTTTGTACTTTATTGAGATTAGAGT

AATTTTTATAGGAGGAATATTAGTTTTATTCATTTATGTAACTTCTCTTTCATCTAATGAAATATTTTCATTATCTATAAAACTATTTTTTTTATCTTTAAGTATAATT

AATTTTAACAGGATTATTCTTAGCAATACACTATACTGCTGATATTGAAACAGCTTTTAATAGAGTAAATCACATTTATCGTGATGTTAATAATGGTTGATTCCTACGAATT

CTGTTGATAATGCTACTTTAACTCGATTCTTTACATTTCATTTTATTCTCCCTTTCATTGTATTAGCTTTAACTATAATT

CTTTTGATTTTGCTGAAGGGGAGTCTGAGTTAGTTTCAGGATTTAATGTAGAATATAGAAGAGGGGGATTTGCTTTAATT

AATTTTTTTATATATCAGAAATATATATGATTTATTGTATTTTGTTTTCCTTTAGGTCTCGTTTGATTTGCATCTTGTTT

AATTTAAGCTATAAACTTTTTGTAATAAACTAGGATTAGATACCCTATTATTAAAAATAAATATTAAAATGCTAAAGTAG

>BrM44-1

AATTTTATCTACTTTATTATTAAAAAGAGGAGCAGCTCCTTTTCATTTTTGATTCCCAGGAGTTATAGAAGGATTAAATT

AATTAATCATATTGGTTGAATATTAATAGCTATAATAAATAACGAACTTTTATGATTAACTTATTTTTTATTATATTCAATT

CAACAAATCATAAAGATATTGGAACTTTATATTTCATTTTTGGAGTATGATCTGGAATAGTCGGAACTTCTCTAAGAATT

AATTGGATTATTAGGATTTATTGTTTGAGCTCATCATATATTTACAGTAGGTATAGACGTAGATACTCGAGCTTATTTTACTTCAGCAACTATAATT

CATGATACTTATTACGTAGTTGCCCATTTTCATTACGTTTTATCTATAGGAGCTGTATTTGCTATTATAGCAGGATTTATTCATTGATACCCTTTATTAACAGGAATAGTTATAAACCCTTCATG

CTGGAATACCTCGACGATACTCAGATTTTCCTGATAGCTACTTAACTTGAAATATTATTTCTTCTTTAGGAAGAACAATT

AATTTGTCACACACAACATATATTTGCTCATTTAGTTCCTCAAGGAACTCCTCCTGTTTTAATACCTTTTATAGTATGCATTGAAACTATTAGTAATGTAATCCGACCAGGAACTTTAGCAGTACGATTGACTGCTAATATAATT

GACGAGATATTTCTCGAGAAGGAACTTTTCAAGGACTTCACACTATTCCCGTAACATTAGGATTACGATGAGGAATAATTTTATTTATTATTTCTGAAGTTTTTTTCTTTATTTCCTTCTTTTGAGCTTTTTTCCATAGTAGCTTATCTCCAACAATCGAATT

AATTGTCATTTTTCTAGAAGTCATCATTTTGGATTCGAAGCAGCTGCTTGATATTGACATTTTGTTGATGTAGTTTGACTATTCTTATATATTTCAATT

AATTTTAGGAATATATTCTTATAAAATGTTTGATCAAGGATGAAGTGAATATTTTGGAGGTCAGATATTATATAATCAAT

ATTATTCAATAACAGGGGATTTTAATAGAACTACATTGAATATGTTAGATGATAAGGGGTGAACTATATCTTTTAGAATT

AATTCCTTTTTCTTCTTGATTACCTGCAGCTATGGCAGCACCGACTCCTGTTTCTGCTTTAGTACATTCTTCTACATTAG

AATTTATTTTCAAAATGTAAAATCTTATAATGCTGGTATATTAACAGCTTTATCTAATCGAATCGGGGATGTTGCTTTAT

CTTTACATTCAGTATCTATTGTAATAACTTTTTTATTTGATTGAATAAGTTTAATATTTATATCTTTTGTTTTATTAATT

AATTTTTATTAGATTTACTTTATTTATTTTAAGATTAAAGTTTTTATTAATAGATTTAGTATATTTTATTGAATGAGAAATT

GAGGATATCAACCTGAACGTTTACAGGCGGGGATTTATTTATTATTTTATACTTTACTGGCTTCGTTGCCGTTATTAATT

AATTATGAAAGTTACTTTAGTATATTTTTTTTAACTTTTTGTGTTTGTGAAGGGGTTCTAGGGCTGTCCATTTTAGTTTC

AATTATTATATTTATTTTTGGAAGTATTGTTTTTATTTCTAGTCGTAAGCATTTACTTTGTACTTTATTGAGATTAGAGT

AATTTTTATAGGAGGAATATTAGTTTTATTCATTTATGTAACTTCTCTTTCATCTAATGAAATATTTTCATTATCTATAAAACTATTTTTTTTATCTTTAAGTATAATT

AATTTTAACAGGATTATTCTTAGCAATACACTATACTGCTGATATTGAAACAGCTTTTAATAGAGTAAATCACATTTATCGTGATGTTAATAATGGTTGATTCCTACGAATT

CTGTTGATAATGCTACTTTAACTCGATTCTTTACATTTCATTTTATTCTCCCTTTCATTGTATTAGCTTTAACTATAATT

CTTTTGATTTTGCTGAAGGGGAGTCTGAGTTAGTTTCAGGATTTAATGTAGAATATAGAAGAGGGGGATTTGCTTTAATT

AATTTTTTTATATATCAGAAATATATATGATTTATTGTATTTTGTTTTCCTTTAGGTCTCGTTTGATTTGCATCTTGTTT

AATTTAAGCTATAAACTTTTTGTAATAAACTAGGATTAGATACCCTATTATTAAAAATAAATATTAAAATGCTAAAGTAG

>BrM44-2

AATTTTATCTACTTTATTATTAAAAAGAGGAGCAGCTCCTTTTCATTTTTGATTCCCAGGAGTTATAGAAGGATTAAATT

AATTAATCATATTGGTTGAATATTAATAGCTATAATAAATAACGAACTTTTATGATTAACTTATTTTTTATTATATTCAATT

CAACAAATCATAAAGATATTGGAACTTTATATTTCATTTTTGGAGTATGATCTGGAATAGTCGGAACTTCTCTAAGAATT

AATTGGATTATTAGGATTTATTGTTTGAGCTCATCATATATTTACAGTAGGTATAGACGTAGATACTCGAGCTTATTTTACTTCAGCAACTATAATT

CATGATACTTATTACGTAGTTGCCCATTTTCATTACGTTTTATCTATAGGAGCTGTATTTGCTATTATAGCAGGATTTATTCATTGATACCCTTTATTAACAGGAATAGTTATAAACCCTTCATG

CTGGAATACCTCGACGATACTCAGATTTTCCTGATAGCTACTTAACTTGAAATATTATTTCTTCTTTAGGAAGAACAATT

AATTTGTCACACACAACATATATTTGCTCATTTAGTTCCTCAAGGAACTCCTCCTGTTTTAATACCTTTTATAGTATGCATTGAAACTATTAGTAATGTAATCCGACCAGGAACTTTAGCAGTACGATTGACTGCTAATATAATT

GACGAGATATTTCTCGAGAAGGAACTTTTCAAGGACTTCACACTATTCCCGTAACATTAGGATTACGATGAGGAATAATTTTATTTATTATTTCTGAAGTTTTTTTCTTTATTTCCTTCTTTTGAGCTTTTTTCCATAGTAGCTTATCTCCAACAATCGAATT

AATTGTCATTTTTCTAGAAGTCATCATTTTGGATTCGAAGCAGCTGCTTGATATTGACATTTTGTTGATGTAGTTTGACTATTCTTATATATTTCAATT

AATTTTAGGAATATATTCTTATAAAATGTTTGATCAAGGATGAAGTGAATATTTTGGAGGTCAGATATTATATAATCAAT

ATTATTCAATAACAGGGGATTTTAATAGAACTACATTGAATATGTTAGATGATAAGGGGTGAACTATATCTTTTAGAATT

AATTCCTTTTTCTTCTTGATTACCTGCAGCTATGGCAGCACCGACTCCTGTTTCTGCTTTAGTACATTCTTCTACATTAG

AATTTATTTTCAAAATGTAAAATCTTATAATGCTGGTATATTAACAGCTTTATCTAATCGAATCGGGGATGTTGCTTTAT

CTTTACATTCAGTATCTATTGTAATAACTTTTTTATTTGATTGAATAAGTTTAATATTTATATCTTTTGTTTTATTAATT

AATTTTTATTAGATTTACTTTATTTATTTTAAGATTAAAGTTTTTATTAATAGATTTAGTATATTTTATTGAATGAGAAATT

GAGGATATCAACCTGAACGTTTACAGGCGGGGATTTATTTATTATTTTATACTTTACTGGCTTCGTTGCCGTTATTAATT

AATTATGAAAGTTACTTTAGTATATTTTTTTTAACTTTTTGTGTTTGTGAAGGGGTTCTAGGGCTGTCCATTTTAGTTTC

AATTATTATATTTATTTTTGGAAGTATTGTTTTTATTTCTAGTCGTAAGCATTTACTTTGTACTTTATTGAGATTAGAGT

AATTTTTATAGGAGGAATATTAGTTTTATTCATTTATGTAACTTCTCTTTCATCTAATGAAATATTTTCATTATCTATAAAACTATTTTTTTTATCTTTAAGTATAATT

AATTTTAACAGGATTATTCTTAGCAATACACTATACTGCTGATATTGAAACAGCTTTTAATAGAGTAAATCACATTTATCGTGATGTTAATAATGGTTGATTCCTACGAATT

CTGTTGATAATGCTACTTTAACTCGATTCTTTACATTTCATTTTATTCTCCCTTTCATTGTATTAGCTTTAACTATAATT

CTTTTGATTTTGCTGAAGGGGAGTCTGAGTTAGTTTCAGGATTTAATGTAGAATATAGAAGAGGGGGATTTGCTTTAATT

AATTTTTTTATATATCAGAAATATATATGATTTATTGTATTTTGTTTTCCTTTAGGTCTCGTTTGATTTGCATCTTGTTT

AATTTAAGCTATAAACTTTTTGTAATAAACTAGGATTAGATACCCTATTATTAAAAATAAATATTAAAATGCTAAAGTAG

>BrM45-1

ANTTTTATCTACTTTATTATTAAAAAGAGGAGCAGCTCCTTTTCATTTTTGATTCCCAGGAGTTATAGAAGGATTAAATT

ANTTAATCATATTGGTTGAATATTAATAGCTATAATAAATAACGAACTTTTATGATTAACTTATTTTTTATTATATTCAATT

CAACAAATCATAAAGATATTGGAACTTTATATTTCATTTTTGGAGTATGATCCGGAATAGTCGGAACTTCTCTAAGAATT

NNNNNNNNNNNNNNNNNNNNNNNNNNNNNNNNNNNNNNNNNNNNNNNNNNNNNNNNNNNNNNNNNNNNNNNNNNNNNNNNNNNNNNNNNNNNNNNNN

CATGATACTTATTACGTAGTTGCCCATTTTCATTACGTTTTATCTATAGGAGCTGTATTTGCTATTATAGCAGGATTTATTCATTGATACCCTTTATTAACAGGAATAGTTATAAACCCTTCATG

CTGGAATACCTCGACGATACTCAGATTTTCCTGATAGCTACTTAACTTGAAATATTATTTCTTCTTTAGGAAGAACAATT

AATTTGTCACACACAACATATATTTGCTCATTTAGTTCCTCAAGGAACTCCTCCTGTTTTAATACCTTTTATAGTATGCATTGAAACTATTAGTAATGTAATCCGACCAGGAACTTTAGCAGTACGATTGACTGCTAATATAATT

GACGAGATATTTCTCGAGAAGGAACTTTTCAAGGACTTCACACTATTCCCGTAACATTAGGATTACGATGAGGAATAATTTTATTTATTATTTCTGAAGTTTTTTTCTTTATTTCCTTCTTTTGAGCTTTTTTCCATAGTAGCTTATCTCCAACAATCGAATT

ANTTGTCATTTTTCTAGAAGTCATCATTTTGGATTTGAAGCAGCTGCTTGATATTGACATTTTGTTGATGTAGTTTGACTNNNNNNNNNNNNNNNNNNN

AATTTTAGGAATATATTCTTATAAAATGTTTGATCAAGGATGAAGTGAATATTTTGGAGGTCAGATATTATATAATCAAT

ATTATTCAATAACAGGGGATTTTAATAGAACTACATTGAATATGTTAGATGATAAGGGGTGAACTATATCTTTTAGAATT

AATTCCTTTTTCTTCTTGATTACCTGCAGCTATGGCAGCACCGACTCCTGTTTCTGCTTTAGTACATTCTTCTACATTAG

AATTTATTTTCAAAATGTAAAATCTTATAATGCTGGTATATTAACAGCTTTATCTAATCGAATCGGGGATGTTGCTTTAT

CTTTACATTCAGTATCTATTGTAATAACTTTTTTATTTGATTGAATAAGTTTAATATTTATATCTTTTGTTTTATTAATT

ANTTTTTATTAGATTTACTTTATTTATTTTAAGATTAAAGTTTTTATTAATAGATTTAGTATATTTTATTGAATGAGAAATT

GAGGATATCAACCTGAACGTTTACAGGCGGGGATTTATTTATTATTTTATACTTTACTGGCTTCGTTGCCGTTATTAATT

ANTTATGAAAGTTACTTTAGTATATTTTTTTTAACTTTTTGTGTTTGTGAAGGGGTTCTAGGGCTATCTATTTTAGTTTC

AATTATTATATTTATTTTTGGAAGTATTGTTTTTATTTCTAGTCGTAAGCATTTACTTTGTACTTTATTGAGATTAGAGT

ANTTTTTATAGGAGGAATATTAGTTTTATTCATTTATGTAACTTCTCTTTCATCTAATGAAATATTTTCATTATCTATAAAACTATTTTTTTTATCTTTAAGTATAATT

AATTTTAACAGGATTATTCTTAGCAATACACTATACTGCTGATATTGAAACAGCTTTTAATAGAGTAAATCACATTTATCNNNNNNNNNNNNNNNNNNNNNNNNNNNNNNNN

CTGTTGATAATGCTACTTTAACTCGATTCTTTACATTTCATTTTATTTTCCCTTTCATTGTATTAGCTTTAACTATAATT

CTTTTGATTTTGCTGAAGGGGAGTCTGAGTTAGTTTCAGGATTTAATGTAGAATATAGAAGAGGGGGATTTGCTTTAATT

AATTTTTTTATATATCAGAAATATATATGATTTATTGTATTTTGTTTTCCTTTAGGTCTCGTTTGATTTGCATCTTGTTT

AATTTAAGCTATAAACTTTTTGTAATAAACTAGGATTAGATACCCTATTATTAAAAATAAATATTAAAATGCTAAAGTAG

>BrM46-1

AATTTTATCTACTTTATTATTAAAAAGAGGAGCAGCTCCTTTTCATTTTTGATTCCCAGGAGTTATAGAAGGATTAAATT

AATTAATCATATTGGTTGAATATTAATAGCTATAATAAATAACGAACTTTTATGATTAACTTATTTTTTATTATATTCAATT

CAACAAATCATAAAGATATTGGAACTTTATATTTCATTTTTGGAGTATGATCTGGAATAGTCGGAACTTCTCTAAGAATT

AATTGGATTATTAGGATTTATTGTTTGAGCTCATCATATATTTACAGTAGGTATAGACGTAGATACTCGAGCTTATTTTACTTCAGCAACTATAATT

CATGATACTTATTACGTAGTTGCCCATTTTCATTACGTTTTATCTATAGGAGCTGTATTTGCTATTATAGCAGGATTTATTCATTGATACCCTTTATTAACAGGAATAGTTATAAACCCTTCATG

CTGGAATACCTCGACGATACTCAGATTTTCCTGATAGCTACTTAACTTGAAATATTATTTCTTCTTTAGGAAGAACAATT

AATTTGTCACACACAACATATATTTGCTCATTTAGTTCCTCAAGGAACTCCTCCTGTTTTAATACCTTTTATAGTATGCATTGAAACTATTAGTAATGTAATCCGACCAGGAACTTTAGCAGTACGATTGACTGCTAATATAATT

GACGAGATATTTCTCGAGAAGGAACTTTTCAAGGACTTCACACTATTCCCGTAACATTAGGATTACGATGAGGAATAATTTTATTTATTATTTCTGAAGTTTTTTTCTTTATTTCCTTCTTTTGAGCTTTTTTCCATAGTAGCTTATCTCCAACAATCGAATT

AATTGTCATTTTTCTAGAAGTCATCATTTTGGATTCGAAGCAGCTGCTTGATATTGACATTTTGTTGATGTAGTTTGACTATTCTTATATATTTCAATT

AATTTTAGGAATATATTCTTATAAAATGTTTGATCAAGGATGAAGTGAATATTTTGGAGGTCAGATATTATATAATCAAT

ATTATTCAATAACAGGGGATTTTAATAGAACTACATTGAATATGTTAGATGATAAGGGGTGAACTATATCTTTTAGAATT

AATTCCTTTTTCTTCTTGATTACCTGCAGCTATGGCAGCACCGACTCCTGTTTCTGCTTTAGTACATTCTTCTACATTAG

AATTTATTTTCAAAATGTAAAATCTTATAATGCTGGTATATTAACAGCTTTATCTAATCGAATCGGGGATGTTGCTTTAT

CTTTACATTCAGTATCTATTGTAATAACTTTTTTATTTGATTGAATAAGTTTAATATTTATATCTTTTGTTTTATTAATT

AATTTTTATTAGATTTACTTTATTTATTTTAAGATTAAAGTTTTTATTAATAGATTTAGTATATTTTATTGAATGAGAAATT

GAGGATATCAACCTGAACGTTTACAGGCGGGGATTTATTTATTATTTTATACTTTACTGGCTTCGTTGCCGTTATTAATT

AATTATGAAAGTTACTTTAGTATATTTTTTTTAACTTTTTGTGTTTGTGAAGGGGTTCTAGGGCTATCCATTTTAGTTTC

AATTATTATATTTATTTTTGGAAGTATTGTTTTTATTTCTAGTCGTAAGCATTTACTTTGTACTTTATTGAGATTAGAGT

AATTTTTATAGGAGGAATATTAGTTTTATTCATTTATGTAACTTCTCTTTCATCTAATGAAATATTTTCATTATCTATAAAACTATTTTTTTTATCTTTAAGTATAATT

AATTTTAACAGGATTATTCTTAGCAATACACTATACTGCTGATATTGAAACAGCTTTTAATAGAGTAAATCACATTTATCGTGATGTTAATAATGGTTGATTCCTACGAATT

CTGTTGATAATGCTACTTTAACTCGATTCTTTACATTTCATTTTATTCTCCCTTTCATTGTATTAGCTTTAACTATAATT

CTTTTGATTTTGCTGAAGGGGAGTCTGAGTTAGTTTCAGGATTTAATGTAGAATATAGAAGAGGGGGATTTGCTTTAATT

AATTTTTTTATATATCAGAAATATATATGATTTATTGTATTTTGTTTTCCTTTAGGTCTCGTTTGATTTGCATCTTGTTT

AATTTAAGCTATAAACTTTTTGTAATAAACTAGGATTAGATACCCTATTATTAAAAATAAATATTAAAATGCTAAAGTAG

>BrM46-2

AATTTTATCTACTTTATTATTAAAAAGAGGAGCAGCTCCTTTTCATTTTTGATTCCCAGGAGTTATAGAAGGATTAAATT

AATTAATCATATTGGTTGAATATTAATAGCTATAATAAATAACGAACTTTTATGATTAACTTATTTTTTATTATATTCAATT

CAACAAATCATAAAGATATTGGAACTTTATATTTCATTTTTGGAGTATGATCTGGAATAGTCGGAACTTCTCTAAGAATT

AATTGGATTATTAGGATTTATTGTTTGAGCTCATCATATATTTACAGTAGGTATAGACGTAGATACTCGAGCTTATTTTACTTCAGCAACTATAATN

CATGATACTTATTACGTAGTTGCCCATTTTCATTACGTTTTATCTATAGGAGCTGTATTTGCTATTATAGCAGGATTTATTCATTGATACCCTTTATTAACAGGAATAGTTATAAACCCTTCATG

CTGGAATACCTCGACGATACTCAGATTTTCCTGATAGCTACTTAACTTGAAATATTATTTCTTCTTTAGGAAGAACAATT

AATTTGTCACACACAACATATATTTGCTCATTTAGTTCCTCAAGGAACTCCTCCTGTTTTAATACCTTTTATAGTATGCATTGAAACTATTAGTAATGTAATCCGACCAGGAACTTTAGCAGTACGATTGACTGCTAATATAATT

GACGAGATATTTCTCGAGAAGGAACTTTTCAAGGACTTCACACTATTCCCGTAACATTAGGATTACGATGAGGAATAATTTTATTTATTATTTCTGAAGTTTTTTTCTTTATTTCCTTCTTTTGAGCTTTTTTCCATAGTAGCTTATCTCCAACAATCGAATT

AATTGTCATTTTTCTAGAAGTCATCATTTTGGATTCGAAGCAGCTGCTTGATATTGACATTTTGTTGATGTAGTTTGACTATTCTTATATATTTCAATT

NATTTTAGGAATATATTCTTATAAAATGTTTGATCAAGGATGAAGTGAATATTTTGGAGGTCAGATATTATATAATCAAT

ATTATTCAATAACAGGGGATTTTAATAGAACTACATTGAATATGTTAGATGATAAGGGGTGAACTATATCTTTTAGAATT

AATTCCTTTTTCTTCTTGATTACCTGCAGCTATGGCAGCACCGACTCCTGTTTCTGCTTTAGTACATTCTTCTACATTAG

AATTTATTTTCAAAATGTAAAATCTTATAATGCTGGTATATTAACAGCTTTATCTAATCGAATCGGGGATGTTGCTTTAT

CTTTACATTCAGTATCTATTGTAATAACTTTTTTATTTGATTGAATAAGTTTAATATTTATATCTTTTGTTTTATTAATT

AATTTTTATTAGATTTACTTTATTTATTTTAAGATTAAAGTTTTTATTAATAGATTTAGTATATTTTATTGAATGAGAAATT

GAGGATATCAACCTGAACGTTTACAGGCGGGGATTTATTTATTATTTTATACTTTACTGGCTTCGTTGCCGTTATTAATT

AATTATGAAAGTTACTTTAGTATATTTTTTTTAACTTTTTGTGTTTGTGAAGGGGTTCTAGGGCTGTCCATTTTAGTTTC

AATTATTATATTTATTTTTGGAAGTATTGTTTTTATTTCTAGTCGTAAGCATTTACTTTGTACTTTATTGAGATTAGAGT

AATTTTTATAGGAGGAATATTAGTTTTATTCATTTATGTAACTTCTCTTTCATCTAATGAAATATTTTCATTATCTATAAAACTATTTTTTTTATCTTTAAGTATAATT

AATTTTAACAGGATTATTCTTAGCAATACACTATACTGCTGATATTGAAACAGCTTTTAATAGAGTAAATCACATTTATCGTGATGTTAATAATGGTTGATTCCTACGAATT

CTGTTGATAATGCTACTTTAACTCGATTCTTTACATTTCATTTTATTCTCCCTTTCATTGTATTAGCTTTAACTATAATT

CTTTTGATTTTGCTGAAGGGGAGTCTGAGTTAGTTTCAGGATTTAATGTAGAATATAGAAGAGGGGGATTTGCTTTAATT

AATTTTTTTATATATCAGAAATATATATGATTTATTGTATTTTGTTTTCCTTTAGGTCTCGTTTGATTTGCATCTTGTTT

AATTTAAGCTATAAACTTTTTGTAATAAACTAGGATTAGATACCCTATTATTAAAAATAAATATTAAAATGCTAAAGTAG

>BrM48-1

AATTTTATCTACTTTATTATTAAAAAGAGGAGCAGCTCCTTTTCATTTTTGATTCACAGGAGTTATAGAAGGATTAAATT

AATTAATCATATTGGTTGAATATTAATAGCTATAATAAATAACGAACTTTTATGATTAACTTATTTTTTATTATATTCAATT

CAACAAATCATAAAGATATTGGAACTTTATATTTCATTTTTGGAGTATGATCTGGAATAGTCGGAACTTCTCTAAGAATT

NNNNNNNNNNNNNNNNNNNNNNNNNNNNNNNNNNNNNNNNNNNNNNNNNNNNNNNNNNNNNNNNNNNNNNNNNNNNNNNNNNNNNNNNNNNNNNNNN

CATGATACTTATTACGTAGTTGCCCATTTTCATTACGTTTTATCTATAGGAGCTGTATTTGCTATTATAGCAGGATTTATTCATTGATACCCTTTATTAACAGGAATAGTTATAAACCCTTCATG

CTGGAATACCTCGACGATACTCAGATTTTCCTGATAGCTACTTAACTTGAAATATTATTTCTTCTTTAGGAAGAACAATT

NNNNNNNNNNNNNNNNNNNNNNNNNNNNNNNNNNNNNNNNNNNNNNNNNNNNNNNNNNNNNNNNNCTTTTATAGTATGCATTGAAACTATTAGTAATGTAATCCGACCAGGAACTTTAGCAGTACGATTGACTGCTAATATAATT

GACGAGATATTTCTCGAGAAGGAACTTTTCAAGGACTTCACACTATTCCCGTAACATTAGGATTACGATGAGGAATAATTTTATTTATTATTTCTGAAGTTTTTTTCTTTATTTCCTTCTTTTGAGCTTTTTTCCATAGTAGCTTATCTCCAACAATCGAATT

AATTGTCATTTTTCTAGAAGTCATCATTTTGGATTCGAAGCAGCTGCTTGATATTGACATTTTGTTGATGTAGTTTGACTNNNNNNNNNNNNNNNNNNN

AATTTTAGGAATATATTCTTATAAAATGTTTGATCAAGGATGAAGTGAATATTTTGGAGGTCAGATATTATATAATCAAT

ATTATTCAATAACAGGGGATTTTAATAGAACTACATTGAATATGTTAGATGATAAGGGGTGAACTATATCTTTTAGAATT

AATTCCTTTTTCTTCTTGATTACCTGCAGCTATGGCAGCACCGACTCCTGTTTCTGCTTTAGTACATTCTTCTACATTAG

AATTTATTTTCAAAATGTAAAATCTTATAATGCTGGTATATTAACAGCTTTATCTAATCGAATCGGGGATGTTGCTTTAT

CTTTACATTCAGTATCTATTGTAATAACTTTTTTATTTGATTGAATAAGTTTAATATTTATATCTTTTGTTTTATTAATT

AATTTTTATTAGATTTACTTTATTTATTTTAAGATTAAAGTTTTTATTAATAGATTTAGTATATTTTATTGAATGAGAAATT

GAGGATATCAACCTGAACGTTTACAGGCGGGGATTTATTTATTATTTTATACTTTACTGGCTTCGTTGCCGTTATTAATT

NATTATGAAAGTTACTTTAGTATATTTTTTTTAACTTTTTGTGTTTGTGAAGGGGTTCTAGGGCTGTCCATTTTAGTTTC

NATTATTATATTTAGTTTTGGAAGTATTGTTTTTATTTCTAGTCGTAAGCATTTACTTTGTACTTTATTGAGATTAGAGT

AATTTTTATAGGAGGAATATTAGTTTTATTCATTTATGTAACTTCTCTTTCATCTAATGAAATATTTTCATTATCTATAAAACTATTTTTTTTATCTTTAAGTATAATT

AATTTTAACAGGATTATTCTTAGCAATACACTATACTGCTGATATTGAAACAGCTTTTAATAGAGTAAATCACATTTATCGTGATGTTAATAATGGTTGATTCCTACGAATT

CTGTTGATAATGCTACTTTAACTCGATTCTTTACATTTCATTTTATTCTCCCTTTCATTGTATTAGCTTTAACTATAATT

CTTTTGATTTTGCTGAAGGGGAGTCTGAGTTAGTTTCAGGATTTAATGTAGAATATAGAAGAGGGGGATTTGCTTTAATT

AATTTTTTTATATATCAGAAATATATATGATTTATTGTATTTTGTTTTCCTTTAGGTCTCGTTTGATTTGCATCTTGTTT

AATTTAAGCTATAAACTTTTTGTAATAAACTAGGATTAGATACCCTATTATTAAAAATAAATATTAAAATGCTAAAGTAG

>BrM48-2

AATTTTATCTACTTTATTATTAAAAAGAGGGGCAGCTCCTTTTCATTTTTGATTCCCAGGGGTTATAGAAGGATTAAATT

AATTAATCATATTGGTTGAATATTAATAGCTATAATAAATAACGAACTTTTATGATTAACTTATTTTTTATTATATTCAATT

CAACAAATCATAAAGATATTGGAACTTTATATTTCATTTTTGGAGTATGATCTGGAATAGTCGGAACTTCTCTAAGAATT

AATTGGATTATTGGGATTTATTGTTTGAGCTCATCATATATTTACAGTAGGTATAGATGTAGATACTCGAGCTTATTTTACTTCAGCAACTATAATT

CATGATACTTATTACGTAGTTGCCCATTTTCATTACGTTTTATCTATAGGAGCTGTATTTGCTATTATAGCAGGATTTATTCATTGATACCCTTTATTAACAGGAATAGTTATAAACCCTTCATG

CTGGAATACCTCGACGATACTCAGATTTTCCTGATAGTTACTTAACTTGAAATATTATTTCTTCTTTAGGAAGAACAATT

AATTTGTCACACACAACATATATTTACTCATTTAGTTCCTCAAGGGACTCCTCCTGTTTTAATACCTTTTATAGTATGCATTGAAACTATTAGTAATGTAATCCGACCAGGAACTTTAGCAGTACGATTGACTGCTAATATAATT

GACGAGATATTTCTCGAGAAGGAACTTTTCAAGGACTTCACACTATTCCCGTAACATTAGGATTACGATGAGGAATAATTTTATTTATTATTTCTGAAGTTTTTTTCTTTATTTCCTTCTTTTGAGCTTTTTTCCATAGTAGCTTATCTCCAACAATCGAATT

AATTGTCATTTTTCTAGAAGTCATCATTTTGGATTCGAAGCAGCTGCTTGATATTGACATTTTGTTGATGTAGTTTGACTATTCTTATATATTTCAATT

AATTTTAGGAATATATTCTTATAAAATGTTTGATCAAGGATGAAGTGAATATTTTGGAGGTCAGATATTATATAATCAAT

ATTATTCAATAACAGGGGATTTTAATAGAACTACATTGAATATGTTAAATGATAAGGGGTGAGCTATATCTTTTAGAATT

AATTCCTTTTTCTTCTTGATTACCTGCAGCTATGGCAGCACCGACTCCTGTTTCTGCTTTAGTACATTCTTCTACATTAG

NNNNNNNNNNNNNNNNNNNNNNNNNNNNNNNNNNNNNNNNNNNNNNNNNNNNNNNNNNNNNNNNNNNNNNNNNNNNNNNN

NNNNNNNNNNNNNNNNNNNNNNNNNNNNNNNNNNNNNNNNNNNNNNNNNNNNNNNNNNNNNNNNNNNNNNNNNNNNNNNN

AATTTTTATTAGATTTACTTTATTTATTTTAAGATTAAAGTTTTTATTAATAGATTTAGTATATTTTATTGAATGAGAAATT

GAGGATATCAACCTGAACGTTTACAGGCGGGGATTTATTTATTATTTTATACTTTACTGGCTTCGTTGCCGTTATTAATT

AATTATGAAAGTTACTTTAGTATATTTTTTTTAACTTTTTGTGTTTGTGAAGGGGTTCTAGGGCTATCTATTTTAGTTTC

AATTATTATATTTATTTTTGGAAGTATTGTTTTTATTTCTAGTCGTAAGCATTTACTTTGTACTTTATTGAGATTAGAGT

AATTTTTATAGGAGGAATATTAGTTTTATTCATTTATGTAACTTCTCTTTCATCTAATGAAATATTTTCATTATCTATAAAACTATTTTTTTTATCTTTAAGTATAATT

AATTTTAACAGGATTATTCTTAGCAATACACTATACTGCTGATATTGAAACAGCTTTTAATAGAGTAAATCACATTTATCGTGATGTTAATAATGGTTGATTCCTACGAATT

CTGTTGATAATGCTACTTTAACTCGATTCTTTACCTTTCATTTTATTCTCCCTTTCATTGTATTAGCTTTAACTATAATT

CTTTTGATTTTGCTGAAGGGGAGTCTGAGTTAGTTTCAGGATTTAATGTAGAGTATAGAAGAGGGGGATTTGCTTTAATT

AATTTTTTTATATATCAGAAATATATATGATTTATTGTATTTTGTTTTCCTTTAGGTCTCGTTTGATTTGCATCTTGTTT

AATTTAAGCTATAAACTTTTTGTAATAAACTAGGATTAGATACCCTATTATTAAAAATAAATATTAAAATGCTAAAGTAG

>BrM50-1

AATTTTATCTACTTTATTATTAAAAAGAGGAGCAGCTCCTTTTCATTTTTGATTCCCAGGAGTTATAGAAGGATTAAATT

AATTAATCATATTGGTTGAATATTAATAGCTATAATAAATAACGAACTTTTATGATTAACTTATTTTTTATTATATTCAATN

CAACAAATCATAAAGATATTGGAACTTTATATTTCATTTTTGGAGTATGATCTGGAATAGTCGGAACTTCTCTAAGAATT

AATTGGATTATTAGGATTTATTGTTTGAGCTCATCATATATTTACAGTAGGTATAGACGTAGATACTCGAGCTTATTTTACTTCAGCAACTATAATT

CATGATACTTATTACGTAGTTGCCCATTTTCATTACGTTTTATCTATAGGAGCTGTATTTGCTATTATAGCAGGATTTATTCATTGATACCCTTTATTAACAGGAATAGTTATAAACCCTTCATG

CTGGAATACCTCGACGATACTCAGATTTTCCTGATAGCTACTTAACTTGAAATATTATTTCTTCTTTAGGAAGAACAATT

AATTTGTCACACACAACATATATTTGCTCATTTAGTTCCTCAAGGAACTCCTCCTGTTTTAATACCTTTTATAGTATGCATTGAAACTATTAGTAATGTAATCCGACCAGGAACTTTAGCAGTACGATTGACTGCTAATATAATT

GACGAGATATTTCTCGAGAAGGAACTTTTCAAGGACTTCACACTATTCCCGTAACATTAGGATTACGATGAGGAATAATTTTATTTATTATTTCTGAAGTTTTTTTCTTTATTTCCTTCTTTTGAGCTTTTTTCCATAGTAGCTTATCTCCAACAATCGAATT

AATTGTCATTTTTCTAGAAGTCATCATTTTGGATTCGAAGCAGCTGCTTGATATTGACATTTTGTTGATGTAGTTTGACTATTCTTATATATTTCAATT

AATTTTAGGAATATATTCTTATAAAATGTTTGATCAAGGATGAAGTGAATATTTTGGAGGTCAGATATTATATAATCAAT

ATTATTCAATAACAGGGGATTTTAATAGAACTACATTGAATATGTTAGATGATAAGGGGTGAACTATATCTTTTAGAATT

AATTCCTTTTTCTTCTTGATTACCTGCAGCTATGGCAGCACCGACTCCTGTTTCTGCTTTAGTACATTCTTCTACATTAG

AATTTATTTTCAAAATGTAAAATCTTATAATGCTGGTATATTAACAGCTTTATCTAATCGAATCGGGGATGTTGCTTTAT

CTTTACATTCAGTATCTATTGTAATAACTTTTTTATTTGATTGAATAAGTTTAATATTTATATCTTTTGTTTTATTAANT

AATTTTTATTAGATTTACTTTATTTATTTTAAGATTAAAGTTTTTATTAATAGATTTAGTATATTTTATTGAATGAGAAATT

GAGGATATCAACCTGAACGTTTACAGGCGGGGATTTATTTATTATTTTATACTTTACTGGCTTCGTTGCCGTTATTAATT

AATTATGAAAGTTACTTTAGTATATTTTTTTTAACTTTTTGTGTTTGTGAAGGGGTTCTAGGGCTGTCCATTTTAGTTTC

AATTATTATATTTATTTTTGGAAGTATTGTTTTTATTTCTAGTCGTAAGCATTTACTTTGTACTTTATTGAGATTAGAGT

AATTTTTATAGGAGGAATATTAGTTTTATTCATTTATGTAACTTCTCTTTCATCTAATGAAATATTTTCATTATCTATAAAACTATTTTTTTTATCTTTAAGTATAATT

AATTTTAACAGGATTATTCTTAGCAATACACTATACTGCTGATATTGAAACAGCTTTTAATAGAGTAAATCACATTTATCGTGATGTTAATAATGGTTGATTCCTACGAATT

CTGTTGATAATGCTACTTTAACTCGATTCTTTACATTTCATTTTATTCTCCCTTTCATTGTATTAGCTTTAACTATAATT

CTTTTGATTTTGCTGAAGGGGAGTCTGAGTTAGTTTCAGGATTTAATGTAGAATATAGAAGAGGGGGATTTGCTTTAATT

AATTTTTTTATATATCAGAAATATATATGATTTATTGTATTTTGTTTTCCTTTAGGTCTCGTTTGATTTGCATCTTGTTT

AATTTAAGCTATAAACTTTTTGTAATAAACTAGGATTAGATACCCTATTATTAAAAATAAATATTAAAATGCTAAAGTAG

>BrM50-2

AATTTTATCTACTTTATTATTAAAAAGAGGAGCAGCTCCTTTTCATTTTTGATTCCCAGGAGTTATAGAAGGATTAAATT

ANTTAATCATATTGGTTGAATATTAATAGCTATAATAAATAACGAACTTTTATGATTAACTTATTTTTTATTATATTCAATT

CAACAAATCATAAAGATATTGGAACTTTATATTTCATTTTTGGAGTATGATCTGGAATAGTCGGAACTTCTCTAAGAATT

NNNNNNNNNNNNNNNNNNNNNNNNNNNNNNNNNNNNNNNNNNNNNNNNNNNNNNNNNNNNNNNNNNNNNNNNNNNNNNNNNNNNNNNNNNNNNNNNN

CATGATACTTATTACGTAGTTGCCCATTTTCATTACGTTTTATCTATAGGAGCTGTATTTGCTATTATAGCAGGATTTATTCATTGATACCCTTTATTAACAGGAATAGTTATAAACCCTTCATG

CTGGAATACCTCGACGATACTCAGATTTTCCTGATAGCTACTTAACTTGAAATATTATTTCTTCTTTAGGAAGAACAATT

AATTTGTCACACACAACATATATTTGCTCATTTAGTTCCTCAAGGAACTCCTCCTGTTTTAATACCTTTTATAGTATGCATTGAAACTATTAGTAATGTAATCCGACCAGGAACTTTAGCAGTACGATTGACTGCTAATATAATT

GACGAGATATTTCTCGAGAAGGAACTTTTCAAGGACTTCACACTATTCCCGTAACATTAGGATTACGATGAGGAATAATTTTATTTATTATTTCTGAAGTTTTTTTCTTTATTTCCTTCTTTTGAGCTTTTTTCCATAGTAGCTTATCTCCAACAATCGAATT

AATTGTCATTTTTCTAGAAGTCATCATTTTGGATTCGAAGCAGCTGCTTGATATTGACATTTTGTTGATGTAGTTTGACTATTCTTATATATTTCAATT

AATTTTAGGAATATATTCTTATAAAATGTTTGATCAAGGATGAAGTGAATATTTTGGAGGTCAGATATTATATAATCAAT

ATTATTCAATAACAGGGGATTTTAATAGAACTACATTGAATATGTTAGATGATAAGGGGTGAACTATATCTTTTAGAATT

AATTCCTTTTTCTTCTTGATTACCTGCAGCTATGGCAGCACCGACTCCTGTTTCTGCTTTAGTACATTCTTCTACATTAG

AATTTATTTTCAAAATGTAAAATCTTATAATGCTGGTATATTAACAGCTTTATCTAATCGAATCGGGGATGTTGCTTTAT

CTTTACATTCAGTATCTATTGTAATAACTTTTTTATTTGATTGAATAAGTTTAATATTTATATCTTTTGTTTTATTAATN

AATTTTTATTAGATTTACTTTATTTATTTTAAGATTAAAGTTTTTATTAATAGATTTAGTATATTTTATTGAATGAGAAATT

GAGGATATCAACCTGAACGTTTACAGGCGGGGATTTATTTATTATTTTATACTTTACTGGCTTCGTTGCCGTTATTAATT

AATTATGAAAGTTACTTTAGTATATTTTTTTTAACTTTTTGTGTTTGTGAAGGGGTTCTAGGGCTGTCCATTTTAGTTTC

AATTATTATATTTATTTTTGGAAGTATTGTTTTTATTTCTAGTCGTAAGCATTTACTTTGTACTTTATTGAGATTAGAGT

AATTTTTATAGGAGGAATATTAGTTTTATTCATTTATGTAACTTCTCTTTCATCTAATGAAATATTTTCATTATCTATAAAACTATTTTTTTTATCTTTAAGTATAATT

AATTTTAACAGGATTATTCTTAGCAATACACTATACTGCTGATATTGAAACAGCTTTTAATAGAGTAAATCACATTTATCGTGATGTTAATAATGGTTGATTCCTACGAATT

CTGTTGATAATGCTACTTTAACTCGATTCTTTACATTTCATTTTATTCTCCCTTTCATTGTATTAGCTTTAACTATAATT

CTTTTGATTTTGCTGAAGGGGAGTCTGAGTTAGTTTCAGGATTTAATGTAGAATATAGAAGAGGGGGATTTGCTTTAATT

AATTTTTTTATATATCAGAAATATATATGATTTATTGTATTTTGTTTTCCTTTAGGTCTCGTTTGATTTGCATCTTGTTT

AATTTAAGCTATAAACTTTTTGTAATAAACTAGGATTAGATACCCTATTATTAAAAATAAATATTAAAATGCTAAAGTAG

>BrM51-1

NATTTTATCTACTTTATTATTAAAAAGAGGAGCAGCTCCTTTTCATTTTTGATTCCCAGGAGTTATAGAAGGATTAAATT

AATTAATCATATTGGTTGAATATTAATAGCTATAATAAATAACGAACTTTTATGATTAACTTATTTTTTATTATATACAATT

CAACAAATCATAAAGATATTGGAACTTTATATTTCATTTTTGGAGTATGATCTGGAATAGTCGGAACTTCTCTAAGAATT

AATTGGATTATTAGGATTTATTGTTTGAGCTCATCATATATTTACAGTAGGTATAGACGTAGATACTCGAGCTTATTTTACTTCAGCAACTATAATT

CATGATACTTATTACGTAGTTGCCCATTTTCATTACGTTTTATCTATAGGAGCTGTATTTGCTATTATAGCAGGATTTATTCATTGATACCCTTTATTAACAGGAATAGTTATAAACCCTTCATG

CTGGAATACCTCGACGATACTCAGATTTTCCTGATAGCTACTTAACTTGAAATATTATTTCTTCTTTAGGAAGAACAATT

AATTTGTCACACACAACATATATTTGCTCATTTAGTTCCTCAAGGAACTCCTCCTGTTTTAATACCTTTTATAGTATGCATTGAAACTATTAGTAATGTAATCCGACCAGGAACTTTAGCAGTACGATTGACTGCTAATATAATT

GACGAGATATTTCTCGAGAAGGAACTTTTCAAGGACTTCACACTATTCCCGTAACATTAGGATTACGATGAGGAATAATTTTATTTATTATTTCTGAAGTTTTTTTCTTTATTTCCTTCTTTTGAGCTTTTTTCCATAGTAGCTTATCTCCAACAATCGAATT

AATTGTCATTTTTCTAGAAGTCATCATTTTGGATTCGAAGCAGCTGCTTGATATTGACATTTTGTTGATGTAGTTTGACTATTCTTATATATTTCAATT

AATTTTAGGAATATATTCTTATAAAATGTTTGATCAAGGATGAAGTGAATATTTTGGAGGTCAGATATTATATAATCAAT

ATTATTCAATAACAGGGGATTTTAATAGAACTACATTGAATATGTTAGATGATAAGGGGTGAACTATATCTTTTAGAATT

AATTCCTTTTTCTTCTTGATTACCTGCAGCTATGGCAGCACCGACTCCTGTTTCTGCTTTAGTACATTCTTCTACATTAG

AATTTATTTTCAAAATGTAAAATCTTATAATGCTGGTATATTAACAGCTTTATCTAATCGAATCGGGGATGTTGCTTTAT

CTTTACATTCAGTATCTATTGTAATAACTTTTTTATTTGATTGAATAAGTTTAATATTTATATCTTTTGTTTTATTAATT

AATTTTTATTAGATTTACTTTATTTATTTTAAGATTAAAGTTTTTATTAATAGATTTAGTATATTTTATTGAATGAGAAATT

GAGGATATCAACCTGAACGTTTACAGGCGGGGATTTATTTATTATTTTATACTTTACTGGCTTCGTTGCCGTTATTAATT

AATTATGAAAGTTACTTTAGTATATTTTTTTTAACTTTTTGTGTTTGTGAAGGGGTTCTAGGGCTGTCCATTTTAGTTTC

AATTATTATATTTATTTTTGGAAGTATTGTTTTTATTTCTAGTCGTAAGCATTTACTTTGTACTTTATTGAGATTAGAGT

AATTTTTATAGGAGGAATATTAGTTTTATTCATTTATGTAACTTCTCTTTCATCTAATGAAATATTTTCATTATCTATAAAACTATTTTTTTTATCTTTAAGTATAATT

AATTTTAACAGGATTATTCTTAGCAATACACTATACTGCTGATATTGAAACAGCTTTTAATAGAGTAAATCACATTTATCGTGATGTTAATAATGGTTGATTCCTACGAATT

CTGTTGATAATGCTACTTTAACTCGATTCTTTACATTTCATTTTATTCTCCCTTTCATTGTATTAGCTTTAACTATAATT

CTTTTGATTTTGCTGAAGGGGAGTCTGAGTTAGTTTCAGGATTTAATGTAGAATATAGAAGAGGGGGATTTGCTTTAATT

AATTTTTTTATATATCAGAAATATATATGATTTATTGTATTTTGTTTTCCTTTAGGTCTCGTTTGATTTGCATCTTGTTT

AATTTAAGCTATAAACTTTTTGTAATAAACTAGGATTAGATACCCTATTATTAAAAATAAATATTAAAATGCTAAAGTAG

>BrM51-2

AATTTTATCTACTTTATTATTAAAAAGAGGAGCAGCTCCTTTTCATTTTTGATTCCCAGGAGTTATAGAAGGATTAAATT

AATTAATCATATTGGTTGAATATTAATAGCTATAATAAATAACGAACTTTTATGATTAACTTATTTTTTATTATATTCAATT

CAACAAATCATAAAGATATTGGAACTTTATATTTCATTTTTGGAGTATGATCTGGAATAGTCGGAACTTCTCTAAGAATT

AATTGGATTATTAGGATTTATTGTTTGAGCTCATCATATATTTACAGTAGGTATAGACGTAGATACTCGAGCTTATTTTACTTCAGCAACTATAATT

CATGATACTTATTACGTAGTTGCCCATTTTCATTACGTTTTATCTATAGGAGCTGTATTTGCTATTATAGCAGGATTTATTCATTGATACCCTTTATTAACAGGAATAGTTATAAACCCTTCATG

CTGGAATACCTCGACGATACTCAGATTTTCCTGATAGCTACTTAACTTGAAATATTATTTCTTCTTTAGGAAGAACAATT

AATTTGTCACACACAACATATATTTGCTCATTTAGTTCCTCAAGGAACTCCTCCTGTTTTAATACCTTTTATAGTATGCATTGAAACTATTAGTAATGTAATCCGACCAGGAACTTTAGCAGTACGATTGACTGCTAATATAATT

GACGAGATATTTCTCGAGAAGGAACTTTTCAAGGACTTCACACTATTCCCGTAACATTAGGATTACGATGAGGAATAATTTTATTTATTATTTCTGAAGTTTTTTTCTTTATTTCCTTCTTTTGAGCTTTTTTCCATAGTAGCTTATCTCCAACAATCGAATT

AATTGTCATTTTTCTAGAAGTCATCATTTTGGATTCGAAGCAGCTGCTTGATATTGACATTTTGTTGATGTAGTTTGACTATTCTTATATATTTCAATT

AATTTTAGGAATATATTCTTATAAAATGTTTGATCAAGGATGAAGTGAATATTTTGGAGGTCAGATATTATATAATCAAT

ATTATTCAATAACAGGGGATTTTAATAGAACTACATTGAATATGTTAGATGATAAGGGGTGAACTATATCTTTTAGAATT

AATTCCTTTTTCTTCTTGATTACCTGCAGCTATGGCAGCACCGACTCCTGTTTCTGCTTTAGTACATTCTTCTACATTAG

AATTTATTTTCAAAATGTAAAATCTTATAATGCTGGTATATTAACAGCTTTATCTAATCGAATCGGGGATGTTGCTTTAT

CTTTACATTCAGTATCTATTGTAATAACTTTTTTATTTGATTGAATAAGTTTAATATTTATATCTTTTGTTTTATTAATT

AATTTTTATTAGATTTACTTTATTTATTTTAAGATTAAAGTTTTTATTAATAGATTTAGTATATTTTATTGAATGAGAAATT

GAGGATATCAACCTGAACGTTTACAGGCGGGGATTTATTTATTATTTTATACTTTACTGGCTTCGTTGCCGTTATTAATT

AATTATGAAAGTTACTTTAGTATATTTTTTTTAACTTTTTGTGTTTGTGAAGGGGTTCTAGGGCTGTCCATTTTAGTTTC

AATTATTATATTTATTTTTGGAAGTATTGTTTTTATTTCTAGTCGTAAGCATTTACTTTGTACTTTATTGAGATTAGAGT

NNNNNNNNNNNNNNNNNNNNNNNNNNNNNTCATTTATGTAACTTCTCTTTCATCTAATGAAATATTTTCATTATCTATAAAACTATTTTTTTTATCTTTAAGTATAATT

AATTTTAACAGGATTATTCTTAGCAATACACTATACTGCTGATATTGAAACAGCTTTTAATAGAGTAAATCACATTTATCGTGATGTTAATAATGGTTGATTCCTACGAATT

CTGTTGATAATGCTACTTTAACTCGATTCTTTACATTTCATTTTATTCTCCCTTTCATTGTATTAGCTTTAACTATAATT

CTTTTGATTTTGCTGAAGGGGAGTCTGAGTTAGTTTCAGGATTTAATGTAGAATATAGAAGAGGGGGATTTGCTTTAATT

NATTTTTTTATATATCAGAAATATATATGATTTATTGTATTTTGTTTTCCTTTAGGTCTCGTTTGATTTGCATCTTGTTT

AATTTAAGCTATAAACTTTTTGTAATAAACTAGGATTAGATACCCTATTATTAAAAATAAATATTAAAATGCTAAAGTAG

>BrM52-1

AATTTTATCTACTTTATTATTAAAAAGAGGAGCAGCTCCTTTTCATTTTTGATTCCCAGGAGTTATAGAAGGATTAAATT

AATTAATCATATTGGTTGAATATTAATAGCTATAATAAATAACGAACTTTTATGATTAACTTATTTTTTATTATATTCAATT

CAACAAATCATAAAGATATTGGAACTTTATATTTCATTTTTGGAGTATGATCTGGAATAGTCGGAACTTCTCTAAGAATT

AATTGGATTATTAGGATTTATTGTTTGAGCTCATCATATATTTACAGTAGGTATAGACGTAGATACTCGAGCTTATTTTACTTCAGCAACTATAATT

CATGATACTTATTACGTAGTTGCCCATTTTCATTACGTTTTATCTATAGGAGCTGTATTTGCTATTATAGCAGGATTTATTCATTGATACCCTTTATTAACAGGAATAGTTATAAACCCTTCATG

CTGGAATACCTCGACGATACTCAGATTTTCCTGATAGCTACTTAACTTGAAATATTATTTCTTCTTTAGGAAGAACAATT

AATTTGTCACACACAACATATATTTGCTCATTTAGTTCCTCAAGGAACTCCTCCTGTTTTAATACCTTTTATAGTATGCATTGAAACTATTAGTAATGTAATCCGACCAGGAACTTTAGCAGTACGATTGACTGCTAATATAATT

GACGAGATATTTCTCGAGAAGGAACTTTTCAAGGACTTCACACTATTCCCGTAACATTAGGATTACGATGAGGAATAATTTTATTTATTATTTCTGAAGTTTTTTTCTTTATTTCCTTCTTTTGAGCTTTTTTCCATAGTAGCTTATCTCCAACAATCGAATT

AATTGTCATTTTTCTAGAAGTCATCATTTTGGATTCGAAGCAGCTGCTTGATATTGACATTTTGTTGATGTAGTTTGACTATTCTTATATATTTCAATT

AATTTTAGGAATATATTCTTATAAAATGTTTGATCAAGGATGAAGTGAATATTTTGGAGGTCAGATATTATATAATCAAT

ATTATTCAATAACAGGGGATTTTAATAGAACTACATTGAATATGTTAGATGATAAGGGGTGAACTATATCTTTTAGAATT

AATTCCTTTTTCTTCTTGATTACCTGCAGCTATGGCAGCACCGACTCCTGTTTCTGCTTTAGTACATTCTTCTACATTAG

AATTTATTTTCAAAATGTAAAATCTTATAATGCTGGTATATTAACAGCTTTATCTAATCGAATCGGGGATGTTGCTTTAT

CTTTACATTCAGTATCTATTGTAATAACTTTTTTATTTGATTGAATAAGTTTAATATTTATATCTTTTGTTTTATTAATN

AATTTTTATTAGATTTACTTTATTTATTTTAAGATTAAAGTTTTTATTAATAGATTTAGTATATTTTATTGAATGAGAAATT

GAGGATATCAACCTGAACGTTTACAGGCGGGGATTTATTTATTATTTTATACTTTACTGGCTTCGTTGCCGTTATTAATT

NATTATGAAAGTTACTTTAGTATATTTTTTTTAACTTTTTGTGTTTGTGAAGGGGTTCTAGGGCTGTCCATTTTAGTTTC

AATTATTATATTTATTTTTGGAAGTATTGTTTTTATTTCTAGTCGTAAGCATTTACTTTGTACTTTATTGAGATTAGAGT

AATTTTTATAGGAGGAATATTAGTTTTATTCATTTATGTAACTTCTCTTTCATCTAATGAAATATTTTCATTATCTATAAAACTATTTTTTTTATCTTTAAGTATAATT

AATTTTAACAGGATTATTCTTAGCAATACACTATACTGCTGATATTGAAACAGCTTTTAATAGAGTAAATCACATTTATCGTGATGTTAATAATGGTTGATTCCTACGAATT

CTGTTGATAATGCTACTTTAACTCGATTCTTTACATTTCATTTTATTCTCCCTTTCATTGTATTAGCTTTAACTATAATT

CTTTTGATTTTGCTGAAGGGGAGTCTGAGTTAGTTTCAGGATTTAATGTAGAATATAGAAGAGGGGGATTTGCTTTAATT

AATTTTTTTATATATCAGAAATATATATGATTTATTGTATTTTGTTTTCCTTTAGGTCTCGTTTGATTTGCATCTTGTTT

AATTTAAGCTATAAACTTTTTGTAATAAACTAGGATTAGATACCCTATTATTAAAAATAAATATTAAAATGCTAAAGTAG

>BrM52-2

AATTTTATCTACTTTATTATTAAAAAGAGGAGCAGCTCCTTTTCATTTTTGATTCCCAGGAGTTATAGAAGGATTAAATT

NATTAATCATATTGGTTGAATATTAATAGCTATAATAAATAACGAACTTTTATGATTAACTTATTTTTTATTATATTCAATT

CAACAAATCATAAAGATATTGGAACTTTATATTTCATTTTTGGAGTATGATCTGGAATAGTCGGAACTTCTCTAAGAATT

AATTGGATTATTAGGATTTATTGTTTGAGCTCATCATATATTTACAGTAGGTATAGACGTAGATACTCGAGCTTATTTTACTTCAGCAACTATAATT

CATGATACTTATTACGTAGTTGCCCATTTTCATTACGTTTTATCTATAGGAGCTGTATTTGCTATTATAGCAGGATTTATTCATTGATACCCTTTATTAACAGGAATAGTTATAAACCCTTCATG

CTGGAATACCTCGACGATACTCAGATTTTCCTGATAGCTACTTAACTTGAAATATTATTTCTTCTTTAGGAAGAACAATT

AATTTGTCACACACAACATATATTTGCTCATTTAGTTCCTCAAGGAACTCCTCCTGTTTTAATACCTTTTATAGTATGCATTGAAACTATTAGTAATGTAATCCGACCAGGAACTTTAGCAGTACGATTGACTGCTAATATAATT

GACGAGATATTTCTCGAGAAGGAACTTTTCAAGGACTTCACACTATTCCCGTAACATTAGGATTACGATGAGGAATAATTTTATTTATTATTTCTGAAGTTTTTTTCTTTATTTCCTTCTTTTGAGCTTTTTTCCATAGTAGCTTATCTCCAACAATCGAATT

AATTGTCATTTTTCTAGAAGTCATCATTTTGGATTCGAAGCAGCTGCTTGATATTGACATTTTGTTGATGTAGTTTGACTATTCTTATATATTTCAATN

AATTTTAGGAATATATTCTTATAAAATGTTTGATCAAGGATGAAGTGAATATTTTGGAGGTCAGATATTATATAATCAAT

ATTATTCAATAACAGGGGATTTTAATAGAACTACATTGAATATGTTAGATGATAAGGGGTGAACTATATCTTTTAGAATT

AATTCCTTTTTCTTCTTGATTACCTGCAGCTATGGCAGCACCGACTCCTGTTTCTGCTTTAGTACATTCTTCTACATTAG

AATTTATTTTCAAAATGTAAAATCTTATAATGCTGGTATATTAACAGCTTTATCTAATCGAATCGGGGATGTTGCTTTAT

CTTTACATTCAGTATCTATTGTAATAACTTTTTTATTTGATTGAATAAGTTTAATATTTATATCTTTTGTTTTATTAATT

AATTTTTATTAGATTTACTTTATTTATTTTAAGATTAAAGTTTTTATTAATAGATTTAGTATATTTTATTGAATGAGAAATT

GAGGATATCAACCTGAACGTTTACAGGCGGGGATTTATTTATTATTTTATACTTTACTGGCTTCGTTGCCGTTATTAATT

AATTATGAAAGTTACTTTAGTATATTTTTTTTAACTTTTTGTGTTTGTGAAGGGGTTCTAGGGCTGTCCATTTTAGTTTC

AATTATTATATTTATTTTTGGAAGTATTGTTTTTATTTCTAGTCGTAAGCATTTACTTTGTACTTTATTGAGATTAGAGT

AATTTTTATAGGAGGAATATTAGTTTTATTCATTTATGTAACTTCTCTTTCATCTAATGAAATATTTTCATTATCTATAAAACTATTTTTTTTATCTTTAAGTATAATT

AATTTTAACAGGATTATTCTTAGCAATACACTATACTGCTGATATTGAAACAGCTTTTAATAGAGTAAATCACATTTATCGTGATGTTAATAATGGTTGATTCCTACGAATT

CTGTTGATAATGCTACTTTAACTCGATTCTTTACATTTCATTTTATTCTCCCTTTCATTGTATTAGCTTTAACTATAATT

CTTTTGATTTTGCTGAAGGGGAGTCTGAGTTAGTTTCAGGATTTAATGTAGAATATAGAAGAGGGGGATTTGCTTTAATT

AATTTTTTTATATATCAGAAATATATATGATTTATTGTATTTTGTTTTCCTTTAGGTCTCGTTTGATTTGCATCTTGTTT

AATTTAAGCTATAAACTTTTTGTAATAAACTAGGATTAGATACCCTATTATTAAAAATAAATATTAAAATGCTAAAGTAG

>BrM54-1

AATTTTATCTACTTTATTATTAAAAAGAGGGGCAGCTCCTTTTCATTTTTGATTCCCAGGAGTTATAGAAGGATTAAATT

NNNNNNNNNNNNNNNNNNNNNNNNNNNNNNNNNNNNNNNNNNNNNNNNNNNNNNNNNNNNNNNNNNNNNNNNNNNNNNNNNN

CAACAAATCATAAAGATATTGGAACTTTATATTTCATTTTTGGAGTATGATCCGGAATAGTCGGAACTTCTCTAAGAATT

AATTGGATTATTGGGATTTATTGTTTGAGCTCATCATATATTTACAGTAGGTATAGACGTAGATACTCGAGCTTATTTTACTTCAGCAACTATAANT

CATGATACTTATTACGTAGTTGCCCATTTTCATTATGTTTTATCTATAGGAGCTGTATTTGCTATTATAGCAGGATTTATTCATTGATACCCTTTATTAACAGGAATAGTTATAAACCCTTCATG

CTGGAATACCTCGACGATACTCAGATTTTCCCGATAGTTACTTAACTTGAAATATTATTTCTTCTTTAGGAAGAACAATT

AATTTGTCACACACAACATATATTTGCTCATTTAGTCCCTCAAGGAACTCCCCCTGTTTTAATACCTTTTATAGTATGCATTGAAACTATTAGTAATGTAATCCGACCAGGAACTTTAGCAGTACGATTAACTGCTAATATAATT

GACGAGATATTTCTCGAGAAGGAACTTTTCAAGGACTTCATACTATTCCCGTAACATTAGGATTACGATGAGGAATAATTTTATTTATTATTTCTGAAGTTTTTTTCTTTATTTCCTTCTTTTGAGCTTTTTTCCATAGTAGCTTATCCCCAACAATCGAATT

ANTTGTCATTTTTCTAGAAGTCATCATTTTGGATTTGAAGCAGCTGCTTGATATTGACATTTTGTTGATGTAGTTTGACTATTCTTATATATTTCAATT

AATTTTAGGAATATATTCTTATAAAATGTTTGATCAAGGATGAAGTGAATATTTTGGAGGTCAGATATTATATAATCAAT

ATTATTCAATAACAGGGGATTTTAATAGAACTACATTGAATATATTAAATGATAAGGGGTGAACTATATCTTTTAGAATT

NNNNNNNNNNNNNNNNNNNNNNNNNNNNNNNNNNNNNNNNNNNNNNNNNNNNNNNNNNNNNNNNNNNNNNNNNNNNNNNN

AATTTATTTTCAAAATGTAAAATCTTATAATGCTGGTATATTAACAGCTTTATCTAATCGAATCGGAGATGTTGCCTTAT

NNNNNNNNNNNNNNNNNNNNNNNNNNNNNNNNNNNNNNNNNNNNNNNNNNNNNNNNNNNNNNNNNNNNNNNNNNNNNNNN

AATTTTTATTAGATTTACTTTATTTATTTTGAGATTAAAGTTTTTATTAATAGATTTAGTATATTTTATTGAATGAGAAATT

GAGGGTATCAACCTGAACGTTTACAGGCGGGGATTTATTTATTATTTTATACTTTACTGGCTTCGTTGCCGTTATTAATT

ANTTATGAAAGTTACTTTAGTATATTTTTTTTAACTTTTTGTGTTTGTGAAGGGGTTCTAGGGTTATCTATTTTAGTTTC

AATTATTATATTTATTTTTGGAAGTATTGTTTTTATTTCTAGTCGTAAGCATTTACTTTGTACTTTATTGAGATTAGAGT

AATTTTTATAGGAGGAATATTAGTTTTATTCATTTATGTTACTTCTCTTTCATCTAATGAAATATTTTCATTATCTATAAAACTATTTTTTTTATCTTTAAGTATAATT

AATTTTAACAGGATTATTCTTAGCAATACACTATACTGCTGATATTGAAACAGCTTTTAATAGAGTAAATCACATTTATCGTGATGTTAATAATGGTTGATTCCTACGAATT

CTGTTGATAATGCTACTTTAACTCGATTCTTTACATTTCATTTTATTTTACCTTTCATTGTATTAGCTTTAACTATAATT

CTTTTGATTTTGCTGAAGGGGAGTCTGAGTTAGTTTCAGGATTTAATGTAGAATATAGAAGAGGGGGATTTGCTTTAATT

NNNNNNNNNNNNNNNNNNNNNNNNNNNNNNNNNNNNNNNNNNNNNNNNNNNNNNNNNNNNNNNNNNNNNNNNNNNNNNNN

AATTTAAGCTATAAACTTTTTGTAATAAACTAGGATTAGATACCCTATTATTAAAAATAAATATTAAAATGCTAAAGTAG

>BrM55-1

AATTTTATCTACTTTATTATTAAAAAGAGGAGCAGCTCCTTTTCATTTTTGATTCCCAGGAGTTATAGAAGGATTAAATT

AATTAATCATATTGGTTGAATATTAATAGCTATAATAAATAACGAACTTTTATGATTAACTTATTTTTTATTATATTCAATT

CAACAAATCATAAAGATATTGGAACTTTATATTTCATTTTTGGAGTATGATCTGGAATAGTCGGAACTTCTCTAAGAATT

ANTTGGATTATTAGGATTTATTGTTTGAGCTCATCATATATTTACAGTAGGTATAGACGTAGATACTCGAGCTTATTTTACTTCAGCAACTATAANT

CATGATACTTATTACGTAGTTGCCCATTTTCATTACGTTTTATCTATAGGAGCTGTATTTGCTATTATAGCAGGATTTATTCATTGATACCCTTTATTAACAGGAATAGTTATAAACCCTTCATG

CTGGAATACCTCGACGATACTCAGATTTTCCTGATAGCTACTTAACTTGAAATATTATTTCTTCTTTAGGAAGAACAATT

AATTTGTCACACACAACATATATTTGCTCATTTAGTTCCTCAAGGAACTCCTCCTGTTTTAATACCTTTTATAGTATGCATTGAAACTATTAGTAATGTAATCCGACCAGGAACTTTAGCAGTACGATTGACTGCTAATATAATT

GACGAGATATTTCTCGAGAAGGAACTTTTCAAGGACTTCACACTATTCCCGTAACATTAGGATTACGATGAGGAATAATTTTATTTATTATTTCTGAAGTTTTTTTCTTTATTTCCTTCTTTTGAGCTTTTTTCCATAGTAGCTTATCTCCAACAATCGAATT

AATTGTCATTTTTCTAGAAGTCATCATTTTGGATTCGAAGCAGCTGCTTGATATTGACATTTTGTTGATGTAGTTTGACTATTCTTATATATTTCAATT

NNNNNNNNNNNNNNNNNNNNNNNNNNNNNNNNNNNNNNNNNNNNNNNNNNNNNNNNNNNNNNNNNNNNNNNNNNNNNNNN

ATTATTCAATAACAGGGGATTTTAATAGAACTACATTGAATATGTTAGATGATAAGGGGTGAACTATATCTTTTAGAATT

AATTCCTTTTTCTTCTTGATTACCTGCAGCTATGGCAGCACCGACTCCTGTTTCTGCTTTAGTACATTCTTCTACATTAG

AATTTATTTTCAAAATGTAAAATCTTATAATGCTGGTATATTAACAGCTTTATCTAATCGAATCGGGGATGTTGCTTTAT

CTTTACATTCAGTATCTATTGTAATAACTTTTTTATTTGATTGAATAAGTTTAATATTTATATCTTTTGTTTTATTAATN

AATTTTTATTAGATTTACTTTATTTATTTTAAGATTAAAGTTTTTATTAATAGATTTAGTATATTTTATTGAATGAGAAATT

GAGGATATCAACCTGAACGTTTACAGGCGGGGATTTATTTATTATTTTATACTTTACTGGCTTCGTTGCCGTTATTAATT

ANTTATGAAAGTTACTTTAGTATATTTTTTTTAACTTTTTGTGTTTGTGAAGGGGTTCTAGGGCTGTCCATTTTAGTTTC

AATTATTATATTTATTTTTGGAAGTATTGTTTTTATTTCTAGTCGTAAGCATTTACTTTGTACTTTATTGAGATTAGAGT

AATTTTTATAGGAGGAATATTAGTTTTATTCATTTATGTAACTTCTCTTTCATCTAATGAAATATTTTCATTATCTATAAAACTATTTTTTTTATCTTTAAGTATAATT

AATTTTAACAGGATTATTCTTAGCAATACACTATACTGCTGATATTGAAACAGCTTTTAATAGAGTAAATCACATTTATCGTGATGTTAATAATGGTTGATTCCTACGAATT

CTGTTGATAATGCTACTTTAACTCGATTCTTTACATTTCATTTTATTCTCCCTTTCATTGTATTAGCTTTAACTATAATT

CTTTTGATTTTGCTGAAGGGGAGTCTGAGTTAGTTTCAGGATTTAATGTAGAATATAGAAGAGGGGGATTTGCTTTAATT

AATTTTTTTATATATCAGAAATATATATGATTTATTGTATTTTGTTTTCCTTTAGGTCTCGTTTGATTTGCATCTTGTTT

AATTTAAGCTATAAACTTTTTGTAATAAACTAGGATTAGATACCCTATTATTAAAAATAAATATTAAAATGCTAAAGTAG

>BrM55-2

AATTTTATCTACTTTATTATTAAAAAGAGGAGCAGCTCCTTTTCATTTTTGATTCCCAGGAGTTATAGAAGGATTAAATT

AATTAATCATATTGGTTGAATATTAATAGCTATAATAAATAACGAACTTTTATGATTAACTTATTTTTTATTATATTCAATT

CAACAAATCATAAAGATATTGGAACTTTATATTTCATTTTTGGAGTATGATCTGGAATAGTCGGAACTTCTCTAAGAATT

AATTGGATTATTAGGATTTATTGTTTGAGCTCATCATATATTTACAGTAGGTATAGACGTAGATACTCGAGCTTATTTTACTTCAGCAACTATAATT

CATGATACTTATTACGTAGTTGCCCATTTTCATTACGTTTTATCTATAGGAGCTGTATTTGCTATTATAGCAGGATTTATTCATTGATACCCTTTATTAACAGGAATAGTTATAAACCCTTCATG

CTGGAATACCTCGACGATACTCAGATTTTCCTGATAGCTACTTAACTTGAAATATTATTTCTTCTTTAGGAAGAACAATT

AATTTGTCACACACAACATATATTTGCTCATTTAGTTCCTCAAGGAACTCCTCCTGTTTTAATACCTTTTATAGTATGCATTGAAACTATTAGTAATGTAATCCGACCAGGAACTTTAGCAGTACGATTGACTGCTAATATAATT

GACGAGATATTTCTCGAGAAGGAACTTTTCAAGGACTTCACACTATTCCCGTAACATTAGGATTACGATGAGGAATAATTTTATTTATTATTTCTGAAGTTTTTTTCTTTATTTCCTTCTTTTGAGCTTTTTTCCATAGTAGCTTATCTCCAACAATCGAATT

AATTGTCATTTTTCTAGAAGTCATCATTTTGGATTCGAAGCAGCTGCTTGATATTGACATTTTGTTGATGTAGTTTGACTATTCTTATATATTTCAATT

AATTTTAGGAATATATTCTTATAAAATGTTTGATCAAGGATGAAGTGAATATTTTGGAGGTCAGATATTATATAATCAAT

ATTATTCAATAACAGGGGATTTTAATAGAACTACATTGAATATGTTAGATGATAAGGGGTGAACTATATCTTTTAGAATT

AATTCCTTTTTCTTCTTGATTACCTGCAGCTATGGCAGCACCGACTCCTGTTTCTGCTTTAGTACATTCTTCTACATTAG

AATTTATTTTCAAAATGTAAAATCTTATAATGCTGGTATATTAACAGCTTTATCTAATCGAATCGGGGATGTTGCTTTAT

CTTTACATTCAGTATCTATTGTAATAACTTTTTTATTTGATTGAATAAGTTTAATATTTATATCTTTTGTTTTATTAATT

AATTTTTATTAGATTTACTTTATTTATTTTAAGATTAAAGTTTTTATTAATAGATTTAGTATATTTTATTGAATGAGAAATT

GAGGATATCAACCTGAACGTTTACAGGCGGGGATTTATTTATTATTTTATACTTTACTGGCTTCGTTGCCGTTATTAATT

AATTATGAAAGTTACTTTAGTATATTTTTTTTAACTTTTTGTGTTTGTGAAGGGGTTCTAGGGCTGTCCATTTTAGTTTC

AATTATTATATTTATTTTTGGAAGTATTGTTTTTATTTCTAGTCGTAAGCATTTACTTTGTACTTTATTGAGATTAGAGT

AATTTTTATAGGAGGAATATTAGTTTTATTCATTTATGTAACTTCTCTTTCATCTAATGAAATATTTTCATTATCTATAAAACTATTTTTTTTATCTTTAAGTATAATT

AATTTTAACAGGATTATTCTTAGCAATACACTATACTGCTGATATTGAAACAGCTTTTAATAGAGTAAATCACATTTATCGTGATGTTAATAATGGTTGATTCCTACGAATT

CTGTTGATAATGCTACTTTAACTCGATTCTTTACATTTCATTTTATTCTCCCTTTCATTGTATTAGCTTTAACTATAATT

CTTTTGATTTTGCTGAAGGGGAGTCTGAGTTAGTTTCAGGATTTAATGTAGAATATAGAAGAGGGGGATTTGCTTTAATT

AATTTTTTTATATATCAGAAATATATATGATTTATTGTATTTTGTTTTCCTTTAGGTCTCGTTTGATTTGCATCTTGTTT

AATTTAAGCTATAAACTTTTTGTAATAAACTAGGATTAGATACCCTATTATTAAAAATAAATATTAAAATGCTAAAGTAG

>BrM56-1

AATTTTATCTACTTTATTATTAAAAAGAGGAGCAGCTCCTTTTCATTTTTGATTCCCAGGAGTTATAGAAGGATTAAATT

NNNNNNNNNNNNNNNNNNNNNNNNNNNNNNNNNNNNNNNNNNNNNNNNNNNNNNNNNNNNNNNNNNNNNNNNNNNNNNNNNN

CAACAAATCATAAAGATATTGGAACTTTATATTTCATTTTTGGAGTATGATCTGGAATAGTCGGAACTTCTCTAAGAATT

AATTGGATTATTAGGATTTATTGTTTGAGCTCATCATATATTTACAGTAGGTATAGACGTAGATACTCGAGCTTATTTTACTTCAGCAACTATAATT

CATGATACTTATTACGTAGTTGCCCATTTTCATTACGTTTTATCTATAGGAGCTGTATTTGCTATTATAGCAGGATTTATTCATTGATACCCTTTATTAACAGGAATAGTTATAAACCCTTCATG

CTGGAATACCTCGACGATACTCAGATTTTCCTGATAGCTACTTAACTTGAAATATTATTTCTTCTTTAGGAAGAACAATT

AATTTGTCACACACAACATATATTTGCTCATTTAGTTCCTCAAGGAACTCCTCCTGTTTTAATACCTTTTATAGTATGCATTGAAACTATTAGTAATGTAATCCGACCAGGAACTTTAGCAGTACGATTGACTGCTAATATAATT

GACGAGATATTTCTCGAGAAGGAACTTTTCAAGGACTTCACACTATTCCCGTAACATTAGGATTACGATGAGGAATAATTTTATTTATTATTTCTGAAGTTTTTTTCTTTATTTCCTTCTTTTGAGCTTTTTTCCATAGTAGCTTATCTCCAACAATCGAATT

AATTGTCATTTTTCTAGAAGTCATCATTTTGGATTCGAAGCAGCTGCTTGATATTGACATTTTGTTGATGTAGTTTGACTATTCTTATATATTTCAANT

AATTTTAGGAATATATTCTTATAAAATGTTTGATCAAGGATGAAGTGAATATTTTGGAGGTCAGATATTATATAATCAAT

ATTATTCAATAACAGGGGATTTTAATAGAACTACATTGAATATGTTAGATGATAAGGGGTGAACTATATCTTTTAGAATT

AATTCCTTTTTCTTCTTGATTACCTGCAGCTATGGCAGCACCGACTCCTGTTTCTGCTTTAGTACATTCTTCTACATTAG

AATTTATTTTCAAAATGTAAAATCTTATAATGCTGGTATATTAACAGCTTTATCTAATCGAATCGGGGATGTTGCTTTAT

NNNNNNNNNNNNNNNNNNNNNNNNNNNNNNNNNNNNNNNNNNNNNNNNNNNNNNNNNNNNNNNNNNNNNNNNNNNNNNNN

AATTTTTATTAGATTTACTTTATTTATTTTAAGATTAAAGTTTTTATTAATAGATTTAGTATATTTTATTGAATGAGAAATT

GAGGATATCAACCTGAACGTTTACAGGCGGGGATTTATTTATTATTTTATACTTTACTGGCTTCGTTGCCGTTATTAATT

AATTATGAAAGTTACTTTAGTATATTTTTTTTAACTTTTTGTGTTTGTGAAGGGGTTCTAGGGCTATCCATTTTAGTTTC

AATTATTATATTTATTTTTGGAAGTATTGTTTTTATTTCTAGTCGTAAGCATTTACTTTGTACTTTATTGAGATTAGAGT

AATTTTTATAGGAGGAATATTAGTTTTATTCATTTATGTAACTTCTCTTTCATCTAATGAAATATTTTCATTATCTATAAAACTATTTTTTTTATCTTTAAGTATAATT

AATTTTAACAGGATTATTCTTAGCAATACACTATACTGCTGATATTGAAACAGCTTTTAATAGAGTAAATCACATTTATCGTAATGTTAATAATGGTTGATTCCTACGAANT

CTGTTGATAATGCTACTTTAACTCGATTCTTTACATTTCATTTTATTCTCCCTTTCATTGTATTAGCTTTAACTATAATT

CTTTTGATTTTGCTGAAGGGGAGTCTGAGTTAGTTTCAGGATTTAATGTAGAATATAGAAGAGGGGGATTTGCTTTAATT

AATTTTTTTATATATCAGAAATATATATGATTTATTGTATTTTGTTTTCCTTTAGGTCTCGTTTGATTTGCATCTTGTTT

AATTTAAGCTATAAACTTTTTGTAATAAACTAGGATTAGATACCCTATTATTAAAAATAAATATTAAAATGCTAAAGTAG

>BrM56-2

AATTTTATCTACTTTATTATTAAAAAGAGGAGCAGCTCCTTTTCATTTTTGATTCCCAGGAGTTATAGAAGGATTAAATT

AATTAATCATATTGGTTGAATATTAATAGCTATAATAAATAACGAACTTTTATGATTAACTTATTTTTTATTATATTCAATT

CAACAAATCATAAAGATATTGGAACTTTATATTTCATTTTTGGAGTATGATCTGGAATAGTCGGAACTTCTCTAAGAATT

AATTGGATTATTAGGATTTATTGTTTGAGCTCATCATATATTTACAGTAGGTATAGACGTAGATACTCGAGCTTATTTTANNNNNNNNNNNNNNNNN

CATGATACTTATTACGTAGTTGCCCATTTTCATTACGTTTTATCTATAGGAGCTGTATTTGCTATTATAGCAGGATTTATTCATTGATACCCTTTATTAACAGGAATAGTTATAAACCCTTCATG

CTGGAATACCTCGACGATACTCAGATTTTCCTGATAGCTACTTAACTTGAAATATTATTTCTTCTTTAGGAAGAACAATT

ANTTTGTCACACACAACATATATTTGCTCATTTAGTTCCTCAAGGAACTCCTCCTGTTTTAATACCTTTTATAGTATGCATTGAAACTATTAGTAATGTAATCCGACCAGGAACTTTAGCAGTACGATTGACTGCTAATATAATT

GACGAGATATTTCTCGAGAAGGAACTTTTCAAGGACTTCACACTATTCCCGTAACATTAGGATTACGATGAGGAATAATTTTATTTATTATTTCTGAAGTTTTTTTCTTTATTTCCTTCTTTTGAGCTTTTTTCCATAGTAGCTTATCTCCAACAATCGAATT

AATTGTCATTTTTCTAGAAGTCATCATTTTGGATTCGAAGCAGCTGCTTGATATTGACATTTTGTTGATGTAGTTTGACTATTCTTATATATTTCAATT

AATTTTAGGAATATATTCTTATAAAATGTTTGATCAAGGATGAAGTGAATATTTTGGAGGTCAGATATTATATAATCAAT

ATTATTCAATAACAGGGGATTTTAATAGAACTACATTGAATATGTTAGATGATAAGGGGTGAACTATATCTTTTAGAATT

AATTCCTTTTTCTTCTTGATTACCTGCAGCTATGGCAGCACCGACTCCTGTTTCTGCTTTAGTACATTCTTCTACATTAG

AATTTATTTTCAAAATGTAAAATCTTATAATGCTGGTATATTAACAGCTTTATCTAATCGAATCGGGGATGTTGCTTTAT

CTTTACATTCAGTATCTATTGTAATAACTTTTTTATTTGATTGAATAAGTTTAATATTTATATCTTTTGTTTTATTAATT

ANTTTTTATTAGATTTACTTTATTTATTTTAAGATTAAAGTTTTTATTAATAGATTTAGTATATTTTATTGAATGAGAAANT

GAGGATATCAACCTGAACGTTTACAGGCGGGGATTTATTTATTATTTTATACTTTACTGGCTTCGTTGCCGTTATTAATT

NNNNNNNNNNNNNNNNNNNNNNNNNNNNNNNNNNNNNNNNNNNNNNNNNNNNNNNNNNNNNNNNNNNNNNNNNNNNNNNN

AATTATTATATTTATTTTTGGAAGTATTGTTTTTATTTCTAGTCGTAAGCATTTACTTTGTACTTTATTGAGATTAGAGT

AATTTTTATAGGAGGAATATTAGTTTTATTCATTTATGTAACTTCTCTTTCATCTAATGAAATATTTTCATTATCTATAAAACTATTTTTTTTATCTTTAAGTATAATT

AATTTTAACAGGATTATTCTTAGCAATACACTATACTGCTGATATTGAAACAGCTTTTAATAGAGTAAATCACATTTATCGTAATGTTAATAATGGTTGATTCCTACGAATT

CTGTTGATAATGCTACTTTAACTCGATTCTTTACATTTCATTTTATTCTCCCTTTCATTGTATTAGCTTTAACTATAATT

CTTTTGATTTTGCTGAAGGGGAGTCTGAGTTAGTTTCAGGATTTAATGTAGAATATAGAAGAGGGGGATTTGCTTTAATT

AATTTTTTTATATATCAGAAATATATATGATTTATTGTATTTTGTTTTCCTTTAGGTCTCGTTTGATTTGCATCTTGTTT

AATTTAAGCTATAAACTTTTTGTAATAAACTAGGATTAGATACCCTATTATTAAAAATAAATATTAAAATGCTAAAGTAG

>BrM57-1

AATTTTATCTACTTTATTATTAAAAAGAGGAGCAGCTCCTTTTCATTTTTGATTCCCAGGAGTTATAGAAGGATTAAATT

ANTTAATCATATTGGTTGAATATTAATAGCTATAATAAATAACGAACTTTTATGATTAACTTATTTTTTATTATATTCAANT

CAACAAATCATAAAGATATTGGAACTTTATATTTCATTTTTGGAGTATGATCTGGAATAGTCGGAACTTCTCTAAGAATT

AATTGGATTATTAGGATTTATTGTTTGAGCTCATCATATATTTACAGTAGGTATAGACGTAGATACTCGAGCTTATTTTACTTCAGCAACTATAATT

CATGATACTTATTACGTAGTTGCCCATTTTCATTACGTTTTATCTATAGGAGCTGTATTTGCTATTATAGCAGGATTTATTCATTGATACCCTTTATTAACAGGAATAGTTATAAACCCTTCATG

CTGGAATACCTCGACGATACTCAGATTTTCCTGATAGCTACTTAACTTGAAATATTATTTCTTCTTTAGGAAGAACAATT

AATTTGTCACACACAACATATATTTGCTCATTTAGTTCCTCAAGGAACTCCTCCTGTTTTAATACCTTTTATAGTATGCATTGAAACTATTAGTAATGTAATCCGACCAGGAACTTTAGCAGTACGATTGACTGCTAATATAATT

GACGAGATATTTCTCGAGAAGGAACTTTTCAAGGACTTCACACTATTCCCGTAACATTAGGATTACGATGAGGAATAATTTTATTTATTATTTCTGAAGTTTTTTTCTTTATTTCCTTCTTTTGAGCTTTTTTCCATAGTAGCTTATCTCCAACAATCGAATT

AATTGTCATTTTTCTAGAAGTCATCATTTTGGATTCGAAGCAGCTGCTTGATATTGACATTTTGTTGATGTAGTTTGACTATTCTTATATATTTCAANT

AATTTTAGGAATATATTCTTATAAAATGTTTGATCAAGGATGAAGTGAATATTTTGGAGGTCAGATATTATATAATCAAT

ATTATTCAATAACAGGGGATTTTAATAGAACTACATTGAATATGTTAGATGATAAGGGGTGAACTATATCTTTTAGAATT

AATTCCTTTTTCTTCTTGATTACCTGCAGCTATGGCAGCACCGACTCCTGTTTCTGCTTTAGTACATTCTTCTACATTAG

AATTTATTTTCAAAATGTAAAATCTTATAATGCTGGTATATTAACAGCTTTATCTAATCGAATCGGGGATGTTGCTTTAT

CTTTACATTCAGTATCTATTGTAATAACTTTTTTATTTGATTGAATAAGTTTAATATTTATATCTTTTGTTTTATTAATT

AATTTTTATTAGATTTACTTTATTTATTTTAAGATTAAAGTTTTTATTAATAGATTTAGTATATTTTATTGAATGAGAAATT

GAGGATATCAACCTGAACGTTTACAGGCGGGGATTTATTTATTATTTTATACTTTACTGGCTTCGTTGCCGTTATTAATT

AATTATGAAAGTTACTTTAGTATATTTTTTTTAACTTTTTGTGTTTGTGAAGGGGTTCTAGGGCTGTCCATTTTAGTTTC

AATTATTATATTTATTTTTGGAAGTATTGTTTTTATTTCTAGTCGTAAGCATTTACTTTGTACTTTATTGAGATTAGAGT

AATTTTTATAGGAGGAATATTAGTTTTATTCATTTATGTAACTTCTCTTTCATCTAATGAAATATTTTCATTATCTATAAAACTATTTTTTTTATCTTTAAGTATAATT

AATTTTAACAGGATTATTCTTAGCAATACACTATACTGCTGATATTGAAACAGCTTTTAATAGAGTAAATCACATTTATCGTGATGTTAATAATGGTTGATTCCTACGAATT

CTGTTGATAATGCTACTTTAACTCGATTCTTTACATTTCATTTTATTCTCCCTTTCATTGTATTAGCTTTAACTATAATT

CTTTTGATTTTGCTGAAGGGGAGTCTGAGTTAGTTTCAGGATTTAATGTAGAATATAGAAGAGGGGGATTTGCTTTAATT

AATTTTTTTATATATCAGAAATATATATGATTTATTGTATTTTGTTTTCCTTTAGGTCTCGTTTGATTTGCATCTTGTTT

AATTTAAGCTATAAACTTTTTGTAATAAACTAGGATTAGATACCCTATTATTAAAAATAAATATTAAAATGCTAAAGTAG

>BrM57-2

AATTTTATCTACTTTATTATTAAAAAGAGGAGCAGCTCCTTTTCATTTTTGATTCCCAGGAGTTATAGAAGGATTAAATT

AATTAATCATATTGGTTGAATATTAATAGCTATAATAAATAACGAACTTTTATGATTAACTTATTTTTTATTATATTCAATT

CAACAAATCATAAAGATATTGGAACTTTATATTTCATTTTTGGAGTATGATCTGGAATAGTCGGAACTTCTCTAAGAATT

AATTGGATTATTAGGATTTATTGTTTGAGCTCATCATATATTTACAGTAGGTATAGACGTAGATACTCGAGCTTATTTTACTTCAGCAACTATAATT

CATGATACTTATTACGTAGTTGCCCATTTTCATTACGTTTTATCTATAGGAGCTGTATTTGCTATTATAGCAGGATTTATTCATTGATACCCTTTATTAACAGGAATAGTTATAAACCCTTCATG

CTGGAATACCTCGACGATACTCAGATTTTCCTGATAGCTACTTAACTTGAAATATTATTTCTTCTTTAGGAAGAACAATT

AATTTGTCACACACAACATATATTTGCTCATTTAGTTCCTCAAGGAACTCCTCCTGTTTTAATACCTTTTATAGTATGCATTGAAACTATTAGTAATGTAATCCGACCAGGAACTTTAGCAGTACGATTGACTGCTAATATAATT

GACGAGATATTTCTCGAGAAGGAACTTTTCAAGGACTTCACACTATTCCCGTAACATTAGGATTACGATGAGGAATAATTTTATTTATTATTTCTGAAGTTTTTTTCTTTATTTCCTTCTTTTGAGCTTTTTTCCATAGTAGCTTATCTCCAACAATCGAATT

AATTGTCATTTTTCTAGAAGTCATCATTTTGGATTCGAAGCAGCTGCTTGATATTGACATTTTGTTGATGTAGTTTGACTATTCTTATATATTTCAATT

AATTTTAGGAATATATTCTTATAAAATGTTTGATCAAGGATGAAGTGAATATTTTGGAGGTCAGATATTATATAATCAAT

ATTATTCAATAACAGGGGATTTTAATAGAACTACATTGAATATGTTAGATGATAAGGGGTGAACTATATCTTTTAGAATT

AATTCCTTTTTCTTCTTGATTACCTGCAGCTATGGCAGCTCCGACTCCTGTTTCTGCTTTAGTACATTCTTCTACATTAG

AATTTATTTTCAAAATGTAAAATCTTATAATGCTGGTATATTAACAGCTTTATCTAATCGAATCGGGGATGTTGCTTTAT

CTTTACATTCAGTATCTATTGTAATAACTTTTTTATTTGATTGAATAAGTTTAATATTTATATCTTTTGTTTTATTAATT

AATTTTTATTAGATTTACTTTATTTATTTTAAGATTAAAGTTTTTATTAATAGATTTAGTATATTTTATTGAATGAGAAATT

GAGGATATCAACCTGAACGTTTACAGGCGGGGATTTATTTATTATTTTATACTTTACTGGCTTCGTTGCCGTTATTAATT

AATTATGAAAGTTACTTTAGTATATTTTTTTTAACTTTTTGTGTTTGTGAAGGGGTTCTAGGGCTGTCCATTTTAGTTTC

AATTATTATATTTATTTTTGGAAGTATTGTTTTTATTTCTAGTCGTAAGCATTTACTTTGTACTTTATTGAGATTAGAGT

AATTTTTATAGGAGGAATATTAGTTTTATTCATTTATGTAACTTCTCTTTCATCTAATGAAATATTTTCATTATCTATAAAACTATTTTTTTTATCTTTAAGTATAATT

AATTTTAACAGGATTATTCTTAGCAATACACTATACTGCTGATATTGAAACAGCTTTTAATAGAGTAAATCACATTTATCGTGATGTTAATAATGGTTGATTCCTACGAATT

CTGTTGATAATGCTACTTTAACTCGATTCTTTACATTTCATTTTATTCTCCCTTTCATTGTATTAGCTTTAACTATAATT

CTTTTGATTTTGCTGAAGGGGAGTCTGAGTTAGTTTCAGGATTTAATGTAGAATATAGAAGAGGGGGATTTGCTTTAATT

AATTTTTTTATATATCAGAAATATATATGATTTATTGTATTTTGTTTTCCTTTAGGTCTCGTTTGATTTGCATCTTGTTT

AATTTAAGCTATAAACTTTTTGTAATAAACTAGGATTAGATACCCTATTATTAAAAATAAATATTAAAATGCTAAAGTAG

>BrM59-1

AATTTTATCTACTTTATTATTAAAAAGAGGGGCAGCTCCTTTTCATTTTTGATTCCCAGGGGTTATAGAAGGATTAAATT

AATTAATCATATTGGTTGAATATTAATAGCTATAATAAATAACGAACTTTTATGATTAACTTATTTTTTATTATATTCAATT

CAACAAATCATAAAGATATTGGAACTTTATATTTCATTTTTGGAGTATGATCTGGAATAGTCGGAACTTCTCTAAGAANT

NNNNNNNNNNNNNNNNNTTATTGTTTGAGCTCATCATATATTTACAGTAGGTATAGATGTAGATACTCGAGCTTATTTTACTTCAGCAACTATAATT

CATGATACTTATTACGTAGTTGCCCATTTTCATTACGTTTTATCTATAGGAGCTGTATTTGCTATTATAGCAGGATTTATTCATTGATACCCTTTATTAACAGGAATAGTTATAAACCCTTCATG

CTGGAATACCTCGACGATACTCAGATTTTCCTGATAGTTACTTAACTTGAAATATTATTTCTTCTTTAGGAAGAACAATT

AATTTGTCACACACAACATATATTTGCTCATTTAGTTCCTCAAGGGACTCCTCCTGTTTTAATACCTTTTATAGTATGCATTGAAACTATTAGTAATGTAATCCGACCAGGAACTTTAGCAGTACGATTGACTGCTAATATAATT

GACGAGATATTTCTCGAGAAGGAACTTTTCAAGGACTTCACACTATTCCCGTAACATTAGGATTACGATGAGGAATANTTTTATTTATTATTTCTGAAGTTTTTTTCTTTATTTCCTTCTTTTGAGCTTTTTTCCATAGTAGCTTATCTCCAACAATCGAANT

AATTGTCATTTTTCTAGAAGTCATCATTTTGGATTCGAAGCAGCTGCTTGATATTGACATTTTGTTGATGTAGTTTGACTATTCTTATATATTTCAATT

AATTTTAGGAATATATTCTTATAAAATGTTTGACCAAGGATGAAGTGAATATTTTGGAGGTCAGATATTATATAATCAAT

ATTATTCAATAACAGGGGATTTTAATAGAACTACATTGAATATGTTAAATGATAAGGGGTGAGCTATATCTTTTAGAATT

AATTCCTTTTTCTTCTTGATTACCTGCAGCTATGGCAGCACCGACTCCTGTTTCTGCTTTAGTACATTCTTCTACATTAG

ANTTTATTTTCAAAATGTAAAATCTTATAATGCTGGTATATTAACAGCTTTATCTAATCGAATCGGAGATGTTGCCTTAT

NNNNNNNNNNNNNNNNNNNNNNNNNNNNNNNNNNNNNNNNNNNNNNNNNNNNNNNNNNNNNNNNNNNNNNNNNNNNNNNN

AATTTTTATTAGATTTACTTTATTTATTTTGAGATTAAAGTTTTTATTAATAGATTTAGTATATTTTATTGAATGAGAAATT

GAGGATATCAACCTGAACGTTTACAGGCGGGGATTTATTTATTATTTTATACTTTACTGGCTTCGTTGCCGTTATTAATT

AATTATGAAAGTTACTTTAGTATATTTTTTTTAACTTTTTGTGTTTGTGAAGGGGTTCTAGGGCTATCTATTTTAGTTTC

AATTATTATATTTATTTTTGGAAGTATTGTTTTTATTTCTAGTCGTAAGCATTTACTTTGTACTTTATTGAGATTAGAGT

AATTTTTATAGGAGGAATATTAGTTTTATTCATTTATGTAACTTCTCTTTCATCTAATGAAATATTTTCATTATCTATAAAACTATTTTTTTTATCTTTAAGTATAANT

AATTTTAACAGGATTATTCTTAGCAATACACTATACTGCTGATATTGAAACAGCTTTTAATAGAGTAAATCACATTTATCGTGATGTTAATAATGGTTGATTCCTACGAATT

CTGTTGATAATGCTACTTTAACTCGATTCTTTACCTTTCATTTTATTCTCCCTTTCATTGTATTAGCTTTAACTATAATT

CTTTTGATTTTGCTGAAGGGGAGTCTGAGTTAGTTTCAGGATTTAATGTAGAGTATAGAAGAGGGGGATTTGCTTTAATT

AATTTTTTTATATATCAGAAATATATATGATTTATTGTATTTTGTTTTCCTTTAGGTCTCGTTTGATTTGCATCTTGTTT

AATTTAAGCTATAAACTTTTTGTAATAAACTAGGATTAGATACCCTATTATTAAAAATAAATATTAAAATGCTAAAGTAG

>BrM59-2

AATTTTATCTACTTTATTATTAAAAAGAGGAGCAGCTCCTTTTCATTTTTGATTCCCAGGAGTTATAGAAGGATTAAATT

AATTAATCATATTGGTTGAATATTAATAGCTATAATAAATAACGAACTTTTATGATTAACTTATTTTTTATTATATTCAATT

CAACAAATCATAAAGATATTGGAACTTTATATTTCATTTTTGGAGTATGATCTGGAATAGTCGGAACTTCTCTAAGAATT

AATTGGATTATTAGGATTTATTGTTTGAGCTCATCATATATTTACAGTAGGTATAGACGTAGATACTCGAGCTTATTTTACTTCAGCAACTATAATT

CATGATACTTATTACGTAGTTGCCCATTTTCATTACGTTTTATCTATAGGAGCTGTATTTGCTATTATAGCAGGATTTATTCATTGATACCCTTTATTAACAGGAATAGTTATAAACCCTTCATG

CTGGAATACCTCGACGATACTCAGATTTTCCTGATAGCTACTTAACTTGAAATATTATTTCTTCTTTAGGAAGAACAATT

AATTTGTCACACACAACATATATTTGCTCATTTAGTTCCTCAAGGAACTCCTCCTGTTTTAATACCTTTTATAGTATGCATTGAAACTATTAGTAATGTAATCCGACCAGGAACTTTAGCAGTACGATTGACTGCTAATATAATT

GACGAGATATTTCTCGAGAAGGAACTTTTCAAGGACTTCACACTATTCCCGTAACATTAGGATTACGATGAGGAATAATTTTATTTATTATTTCTGAAGTTTTTTTCTTTATTTCCTTCTTTTGAGCTTTTTTCCATAGTAGCTTATCTCCAACAATCGAATT

AATTGTCATTTTTCTAGAAGTCATCATTTTGGATTCGAAGCAGCTGCTTGATATTGACATTTTGTTGATGTAGTTTGACTATTCTTATATATTTCAATT

AATTTTAGGAATATATTCTTATAAAATGTTTGATCAAGGATGAAGTGAATATTTTGGAGGTCAGATATTATATAATCAAT

ATTATTCAATAACAGGGGATTTTAATAGAACTACATTGAATATGTTAGATGATAAGGGGTGAACTATATCTTTTAGAATT

AATTCCTTTTTCTTCTTGATTACCTGCAGCTATGGCAGCACCGACTCCTGTTTCTGCTTTAGTACATTCTTCTACATTAG

AATTTATTTTCAAAATGTAAAATCTTATAATGCTGGTATATTAACAGCTTTATCTAATCGAATCGGGGATGTTGCTTTAT

CTTTACATTCAGTATCTATTGTAATAACTTTTTTATTTGATTGAATAAGTTTAATATTTATATCTTTTGTTTTATTAATT

AATTTTTATTAGATTTACTTTATTTATTTTAAGATTAAAGTTTTTATTAATAGATTTAGTATATTTTATTGAATGAGAAATT

GAGGATATCAACCTGAACGTTTACAGGCGGGGATTTATTTATTATTTTATACTTTACTGGCTTCGTTGCCGTTATTAATT

AATTATGAAAGTTACTTTAGTATATTTTTTTTAACTTTTTGTGTTTGTGAAGGGGTTCTAGGGCTGTCCATTTTAGTTTC

AATTATTATATTTATTTTTGGAAGTATTGTTTTTATTTCTAGTCGTAAGCATTTACTTTGTACTTTATTGAGATTAGAGT

AATTTTTATAGGAGGAATATTAGTTTTATTCATTTATGTAACTTCTCTTTCATCTAATGAAATATTTTCATTATCTATAAAACTATTTTTTTTATCTTTAAGTATAATT

AATTTTAACAGGATTATTCTTAGCAATACACTATACTGCTGATATTGAAACAGCTTTTAATAGAGTAAATCACATTTATCGTGATGTTAATAATGGTTGATTCCTACGAATT

CTGTTGATAATGCTACTTTAACTCGATTCTTTACATTTCATTTTATTCTCCCTTTCATTGTATTAGCTTTAACTATAATT

CTTTTGATTTTGCTGAAGGGGAGTCTGAGTTAGTTTCAGGATTTAATGTAGAATATAGAAGAGGGGGATTTGCTTTAATT

ANTTTTTTTATATATCAGAAATATATATGATTTATTGTATTTTGTTTTCCTTTAGGTCTCGTTTGATTTGCATCTTGTTT

AATTTAAGCTATAAACTTTTTGTAATAAACTAGGATTAGATACCCTATTATTAAAAATAAATATTAAAATGCTAAAGTAG

>BrM60-1

AATTTTATCTACTTTATTATTAAAAAGAGGAGCAGCTCCTTTTCATTTTTGATTCCCAGGAGTTATAGAAGGATTAAATT

AATTAATCATATTGGTTGAATATTAATAGCTATAATAAATAACGAACTTTTATGATTAACTTATTTTTTATTATATTCAATT

CAACAAATCATAAAGATATTGGAACTTTATATTTCATTTTTGGAGTATGATCTGGAATAGTCGGAACTTCTCTAAGAATT

AATTGGATTATTAGGATTTATTGTTTGAGCTCATCATATATTTACAGTAGGTATAGACGTAGATACTCGAGCTTATTTTACTTCAGCAACTATAATT

CATGATACTTATTACGTAGTTGCCCATTTTCATTACGTTTTATCTATAGGAGCTGTATTTGCTATTATAGCAGGATTTATTCATTGATACCCTTTATTAACAGGAATAGTTATAAACCCTTCATG

CTGGAATACCTCGACGATACTCAGATTTTCCTGATAGCTACTTAACTTGAAATATTATTTCTTCTTTAGGAAGAACAATT

AATTTGTCACACACAACATATATTTGCTCATTTAGTTCCTCAAGGAACTCCTCCTGTTTTAATACCTTTTATAGTATGCATTGAAACTATTAGTAATGTAATCCGACCAGGAACTTTAGCAGTACGATTGACTGCTAATATAATT

GACGAGATATTTCTCGAGAAGGAACTTTTCAAGGACTTCACACTATTCCCGTAACATTAGGATTACGATGAGGAATAATTTTATTTATTATTTCTGAAGTTTTTTTCTTTATTTCCTTCTTTTGAGCTTTTTTCCATAGTAGCTTATCTCCAACAATCGAATT

AATTGTCATTTTTCTAGAAGTCATCATTTTGGATTCGAAGCAGCTGCTTGATATTGACATTTTGTTGATGTAGTTTGACTATTCTTATATATTTCAATT

AATTTTAGGAATATATTCTTATAAAATGTTTGATCAAGGATGAAGTGAATATTTTGGAGGTCAGATATTATATAATCAAT

ATTATTCAATAACAGGGGATTTTAATAGAACTACATTGAATATGTTAGATGATAAGGGGTGAACTATATCTTTTAGAATT

AATTCCTTTTTCTTCTTGATTACCTGCAGCTATGGCAGCACCGACTCCTGTTTCTGCTTTAGTACATTCTTCTACATTAG

AATTTATTTTCAAAATGTAAAATCTTATAATGCTGGTATATTAACAGCTTTATCTAATCGAATCGGGGATGTTGCTTTAT

CTTTACATTCAGTATCTATTGTAATAACTTTTTTATTTGATTGAATAAGTTTAATATTTATATCTTTTGTTTTATTAATT

AATTTTTATTAGATTTACTTTATTTATTTTAAGATTAAAGTTTTTATTAATAGATTTAGTATATTTTATTGAATGAGAAATT

GAGGATATCAACCTGAACGTTTACAGGCGGGGATTTATTTATTATTTTATACTTTACTGGCTTCGTTGCCGTTATTAATT

AATTATGAAAGTTACTTTAGTATATTTTTTTTAACTTTTTGTGTTTGTGAAGGGGTTCTAGGGCTGTCCATTTTAGTTTC

AATTATTATATTTATTTTTGGAAGTATTGTTTTTATTTCTAGTCGTAAGCATTTACTTTGTACTTTATTGAGATTAGAGT

AATTTTTATAGGAGGAATATTAGTTTTATTCATTTATGTAACTTCTCTTTCATCTAATGAAATATTTTCATTATCTATAAAACTATTTTTTTTATCTTTAAGTATAATT

AATTTTAACAGGATTATTCTTAGCAATACACTATACTGCTGATATTGAAACAGCTTTTAATAGAGTAAATCACATTTATCGTGATGTTAATAATGGTTGATTCCTACGAATT

CTGTTGATAATGCTACTTTAACTCGATTCTTTACATTTCATTTTATTCTCCCTTTCATTGTATTAGCTTTAACTATAATT

CTTTTGATTTTGCTGAAGGGGAGTCTGAGTTAGTTTCAGGATTTAATGTAGAATATAGAAGAGGGGGATTTGCTTTAATT

AATTTTTTTATATATCAGAAATATATATGATTTATTGTATTTTGTTTTCCTTTAGGTCTCGTTTGATTTGCATCTTGTTT

AATTTAAGCTATAAACTTTTTGTAATAAACTAGGATTAGATACCCTATTATTAAAAATAAATATTAAAATGCTAAAGTAG

>BrM60-2

AATTTTATCTACTTTATTATTAAAAAGAGGAGCAGCTCCTTTTCATTTTTGATTCCCAGGAGTTATAGAAGGATTAAATT

AATTAATCATATTGGTTGAATATTAATAGCTATAATAAATAACGAACTTTTATGATTAACTTATTTTTTATTATATTCAATT

CAACAAATCATAAAGATATTGGAACTTTATATTTCATTTTTGGAGTATGATCTGGAATAGTCGGAACTTCTCTAAGAATT

AATTGGATTATTAGGATTTATTGTTTGAGCTCATCATATATTTACAGTAGGTATAGACGTAGATACTCGAGCTTATTTTACTTCAGCAACTATAATT

CATGATACTTATTACGTAGTTGCCCATTTTCATTACGTTTTATCTATAGGAGCTGTATTTGCTATTATAGCAGGATTTATTCATTGATACCCTTTATTAACAGGAATAGTTATAAACCCTTCATG

CTGGAATACCTCGACGATACTCAGATTTTCCTGATAGCTACTTAACTTGAAATATTATTTCTTCTTTAGGAAGAACAATT

AATTTGTCACACACAACATATATTTGCTCATTTAGTTCCTCAAGGAACTCCTCCTGTTTTAATACCTTTTATAGTATGCATTGAAACTATTAGTAATGTAATCCGACCAGGAACTTTAGCAGTACGATTGACTGCTAATATAATT

GACGAGATATTTCTCGAGAAGGAACTTTTCAAGGACTTCACACTATTCCCGTAACATTAGGATTACGATGAGGAATAATTTTATTTATTATTTCTGAAGTTTTTTTCTTTATTTCCTTCTTTTGAGCTTTTTTCCATAGTAGCTTATCTCCAACAATCGAATT

AATTGTCATTTTTCTAGAAGTCATCATTTTGGATTCGAAGCAGCTGCTTGATATTGACATTTTGTTGATGTAGTTTGACTATTCTTATATATTTCAATT

AATTTTAGGAATATATTCTTATAAAATGTTTGATCAAGGATGAAGTGAATATTTTGGAGGTCAGATATTATATAATCAAT

ATTATTCAATAACAGGGGATTTTAATAGAACTACATTGAATATGTTAGATGATAAGGGGTGAACTATATCTTTTAGAATT

AATTCCTTTTTCTTCTTGATTACCTGCAGCTATGGCAGCACCGACTCCTGTTTCTGCTTTAGTACATTCTTCTACATTAG

AATTTATTTTCAAAATGTAAAATCTTATAATGCTGGTATATTAACAGCTTTATCTAATCGAATCGGGGATGTTGCTTTAT

CTTTACATTCAGTATCTATTGTAATAACTTTTTTATTTGATTGAATAAGTTTAATATTTATATCTTTTGTTTTATTAATT

AATTTTTATTAGATTTACTTTATTTATTTTAAGATTAAAGTTTTTATTAATAGATTTAGTATATTTTATTGAATGAGAAATT

GAGGATATCAACCTGAACGTTTACAGGCGGGGATTTATTTATTATTTTATACTTTACTGGCTTCGTTGCCGTTATTAATT

AATTATGAAAGTTACTTTAGTATATTTTTTTTAACTTTTTGTGTTTGTGAAGGGGTTCTAGGGCTGTCCATTTTAGTTTC

AATTATTATATTTATTTTTGGAAGTATTGTTTTTATTTCTAGTCGTAAGCATTTACTTTGTACTTTATTGAGATTAGAGT

AATTTTTATAGGAGGAATATTAGTTTTATTCATTTATGTAACTTCTCTTTCATCTAATGAAATATTTTCATTATCTATAAAACTATTTTTTTTATCTTTAAGTATAATT

AATTTTAACAGGATTATTCTTAGCAATACACTATACTGCTGATATTGAAACAGCTTTTAATAGAGTAAATCACATTTATCGTGATGTTAATAATGGTTGATTCCTACGAATT

CTGTTGATAATGCTACTTTAACTCGATTCTTTACATTTCATTTTATTCTCCCTTTCATTGTATTAGCTTTAACTATAATT

CTTTTGATTTTGCTGAAGGGGAGTCTGAGTTAGTTTCAGGATTTAATGTAGAATATAGAAGAGGGGGATTTGCTTTAATT

AATTTTTTTATATATCAGAAATATATATGATTTATTGTATTTTGTTTTCCTTTAGGTCTCGTTTGATTTGCATCTTGTTT

AATTTAAGCTATAAACTTTTTGTAATAAACTAGGATTAGATACCCTATTATTAAAAATAAATATTAAAATGCTAAAGTAG

>BrM60-3

ANTTTTATCTACTTTATTATTAAAAAGAGGAGCAGCTCCTTTTCATTTTTGATTCCCAGGAGTTATAGAAGGATTAAATT

AATTAATCATATTGGTTGAATATTAATAGCTATAATAAATAACGAACTTTTATGATTAACTTATTTTTTATTATATTCAATT

CAACAAATCATAAAGATATTGGAACTTTATATTTCATTTTTGGAGTATGATCTGGAATAGTCGGAACTTCTCTAAGAATT

AATTGGATTATTAGGATTTATTGTTTGAGCTCATCATATATTTACAGTAGGTATAGACGTAGATACTCGAGCTTATTTTACTTCAGCAACTATAANT

CATGATACTTATTACGTAGTTGCCCATTTTCATTACGTTTTATCTATAGGAGCTGTATTTGCTATTATAGCAGGATTTATTCATTGATACCCTTTATTAACAGGAATAGTTATAAACCCTTCATG

CTGGAATACCTCGACGATACTCAGATTTTCCTGATAGCTACTTAACTTGAAATATTATTTCTTCTTTAGGAAGAACAATT

AATTTGTCACACACAACATATATTTGCTCATTTAGTTCCTCAAGGAACTCCTCCTGTTTTAATACCTTTTATAGTATGCATTGAAACTATTAGTAATGTAATCCGACCAGGAACTTTAGCAGTACGATTGACTGCTAATATAATT

GACGAGATATTTCTCGAGAAGGAACTTTTCAAGGACTTCACACTATTCCCGTAACATTAGGATTACGATGAGGAATAATTTTATTTATTATTTCTGAAGTTTTTTTCTTTATTTCCTTCTTTTGAGCTTTTTTCCATAGTAGCTTATCTCCAACAATCGAATT

AATTGTCATTTTTCTAGAAGTCATCATTTTGGATTCGAAGCAGCTGCTTGATATTGACATTTTGTTGATGTAGTTTGACTATTCTTATATATTTCAATT

AATTTTAGGAATATATTCTTATAAAATGTTTGATCAAGGATGAAGTGAATATTTTGGAGGTCAGATATTATATAATCAAT

ATTATTCAATAACAGGGGATTTTAATAGAACTACATTGAATATGTTAGATGATAAGGGGTGAACTATATCTTTTAGAATT

NNNNNNNNNNNNNNNNNNNNNNNNNNNNNNNNNNNNNNNNNNNNNNNNNNNNNNNNNNNNNNNNNNNNNNNNNNNNNNNN

AATTTATTTTCAAAATGTAAAATCTTATAATGCTGGTATATTAACAGCTTTATCTAATCGAATCGGGGATGTTGCTTTAT

CTTTACATTCAGTATCTATTGTAATAACTTTTTTATTTGATTGAATAAGTTTAATATTTATATCTTTTGTTTTATTAATT

AATTTTTATTAGATTTACTTTATTTATTTTAAGATTAAAGTTTTTATTAATAGATTTAGTATATTTTATTGAATGAGAAATT

GAGGATATCAACCTGAACGTTTACAGGCGGGGATTTATTTATTATTTTATACTTTACTGGCTTCGTTGCCGTTATTAATT

AATTATGAAAGTTACTTTAGTATATTTTTTTTAACTTTTTGTGTTTGTGAAGGGGTTCTAGGGCTGTCCATTTTAGTTTC

AATTATTATATTTATTTTTGGAAGTATTGTTTTTATTTCTAGTCGTAAGCATTTACTTTGTACTTTATTGAGATTAGAGT

AATTTTTATAGGAGGAATATTAGTTTTATTCATTTATGTAACTTCTCTTTCATCTAATGAAATATTTTCATTATCTATAAAACTATTTTTTTTATCTTTAAGTATAATT

AATTTTAACAGGATTATTCTTAGCAATACACTATACTGCTGATATTGAAACAGCTTTTAATAGAGTAAATCACATTTATCGTGATGTTAATAATGGTTGATTCCTACGAATT

CTGTTGATAATGCTACTTTAACTCGATTCTTTACATTTCATTTTATTCTCCCTTTCATTGTATTAGCTTTAACTATAATT

CTTTTGATTTTGCTGAAGGGGAGTCTGAGTTAGTTTCAGGATTTAATGTAGAATATAGAAGAGGGGGATTTGCTTTAATT

AATTTTTTTATATATCAGAAATATATATGATTTATTGTATTTTGTTTTCCTTTAGGTCTCGTTTGATTTGCATCTTGTTT

AATTTAAGCTATAAACTTTTTGTAATAAACTAGGATTAGATACCCTATTATTAAAAATAAATATTAAAATGCTAAAGTAG

>Gv10-A-01

ANTTTTATCTACTTTATTATTAAAAAGAGGGGCAGCTCCTTTTCATTTTTGATTCCCAGGAGTTATAGAAGGATTAAATT

AATTAATCATATTGGTTGAATATTAATAGCTATAATAAATAACGAACTTTTATGACTAACTTATTTTTTATTATATTCAATT

CAACAAATCATAAAGATATTGGAACTTTATATTTCATTTTTGGAGTATGATCCGGAATAGTCGGAACTTCTCTAAGAATT

AATTGGATTATTGGGATTTATTGTTTGAGCTCATCATATATTTACAGTAGGTATAGACGTAGATACTCGAGCTTATTTTACTTCAGCAACTATAATT

CATGATACTTATTATGTAGTTGCCCATTTTCATTATGTTTTATCTATAGGAGCTGTATTTGCTATTATAGCAGGATTTATTCATTGATACCCTTTATTAACAGGAATAGTTATAAACCCTTCATG

CTGGAATACCTCGACGATACTCAGATTTTCCCGATAGTTACTTAACTTGAAATATTATTTCTTCTTTAGGAAGAACAANT

AATTTGTCACACACAACATATATTTGCTCATTTAGTTCCTCAAGGAACTCCCCCTGTTTTAATACCTTTTATAGTATGCATTGAAACTATCAGTAATGTAATCCGACCAGGAACTTTAGCAGTACGATTAACTGCTAATATAATT

GACGAGATATTTCTCGAGAAGGAACTTTTCAAGGACTTCACACTATTCCCGTAACATTAGGATTACGATGAGGAATAATTTTATTTATTATTTCTGAAGTTTTTTTCTTTATTTCCTTCTTTTGAGCTTTTTTCCATAGTAGCTTATCCCCAACAATCGAATT

AATTGTCATTTTTCTAGAAGTCATCATTTTGGATTTGAAGCAGCTGCTTGATATTGACATTTTGTTGATGTAGTTTGACTATTCTTATATATTTCAATT

AATTTTAGGAATATATTCTTATAAAATGTTTGATCAAGGATGAAGTGAATATTTTGGAGGTCAGATATTATATAATCAAT

ATTATTCAATAACAGGGGATTTTAATAGAACTACATTGAATATACTAAATGATAAGGGGTGAACTATATCTTTTAGAATT

AATTCCTTTTTCTTCTTGATTACCTGCAGCTATGGCAGCTCCGACTCCTGTTTCTGCTTTAGTACATTCTTCTACATTAG

AATTTATTTTCAAAATGTAAAATCTTATAATGCTGGTATATTAACAGCTTTATCTAATCGAATCGGAGATGTTGCCTTAT

NNNNNNNNNNNNNNNNNNNNNNNNNNNNNNNNNNNNNNNNNNNNNNNNNNNNNNNNNNNNNNNNNNNNNNNNNNNNNNNN

AATTTTTATTAGATTTACTTTATTTATTTTGAGATTAAAGTTTTTATTAATAGATTTAGTATATTTTATTGAATGAGAAATT

GAGGGTATCAACCTGAACGTTTACAGGCGGGGATTTATTTATTATTTTATACTTTACTGGCTTCGTTGCCGTTATTAATT

AATTATGAAAGTTACTTTAGTATATTTTTTTTAACTTTTTGTGTTTGTGAAGGGGTTCTAGGGCTATCTATTTTAGTTTC

AATTATTATATTTATTTTTGGAAGTATTGTTTTTATTTCTAGTCGTAAGCATTTACTTTGTACTTTATTGAGATTAGAAT

ANTTTTTATAGGAGGAATATTAGTTTTATTCATTTATGTTACTTCTCTTTCATCTAATGAAATATTTTCATTATCTATAAAACTATTTTTTTTATCTTTAAGTATAATT

AATTTTAACAGGATTATTCTTAGCAATACACTATACTGCTGATATTGAAACAGCTTTTAATAGAGTAAATCACATTTATCGTGATGTTAATAATGGTTGATTCCTACGAATT

CTGTTGATAATGCTACTTTAACTCGATTCTTTACATTTCATTTTATTTTACCTTTCATTGTATTAGCTTTAACTATAATT

CTTTTGATTTTGCTGAAGGGGAGTCTGAGTTAGTTTCAGGATTTAATGTAGAATATAGAAGAGGGGGATTTGCTTTAATT

AATTTTTTTATATATCAGAAATACATATGATTTATTGTATTTTGTTTTCCTTTAGGTCTCGTTTGATTTGCATCTTGTTT

AATTTAAGCTATAAACTTTTTGTAATAAACTAGGATTAGATACCCTATTATTAAAAATAAATATTAAAATGCTAAAGTAG

>Gv10-A-02

AATTTTATCTACTTTATTATTAAAAAGAGGGGCAGCTCCTTTTCATTTTTGATTCCCAGGAGTTATAGAAGGATTAAATT

AATTAATCATATTGGTTGAATATTAATAGCTATAATAAATAACGAACTTTTATGACTAACTTATTTTTTATTATATTCAATT

CAACAAATCATAAAGATATTGGAACTTTATATTTCATTTTTGGAGTATGATCCGGAATAGTCGGAACTTCTCTAAGAATT

AATTGGATTATTGGGATTTATTGTTTGAGCTCATCATATATTTACAGTAGGTATAGACGTAGATACTCGAGCTTATTTTACTTCAGCAACTATAATT

CATGATACTTATTATGTAGTTGCCCATTTTCATTATGTTTTATCTATAGGAGCTGTATTTGCTATTATAGCAGGATTTATTCATTGATACCCTTTATTAACAGGAATAGTTATAAACCCTTCATG

CTGGAATACCTCGACGATACTCGGATTTTCCCGATAGTTACTTAACTTGAAATATTATTTCTTCTTTAGGAAGAACAANT

AATTTGTCACACACAACATATATTTGCTCATTTAGTTCCTCAAGGAACTCCCCCTGTTTTAATACCTTTTATAGTATGCATTGAAACTATCAGTAATGTAATCCGACCAGGAACTTTAGCAGTACGATTAACTGCTAATATAATT

GACGAGATATTTCTCGAGAAGGAACTTTTCAAGGACTTCACACTATTCCCGTAACATTAGGATTACGATGAGGAATAATTTTATTTATTATTTCTGAAGTTTTTTTCTTTATTTCCTTCTTTTGAGCTTTTTTCCATAGTAGCTTATCCCCAACAATCGAATT

AATTGTCATTTTTCTAGAAGTCATCATTTTGGATTTGAAGCAGCTGCTTGATATTGACATTTTGTTGATGTAGTTTGACTATTCTTATATATTTCAATT

AATTTTAGGAATATATTCTTATAAAATGTTTGATCAAGGATGAAGTGAATATTTTGGAGGTCAGATATTATATAATCAAT

ATTATTCAATAACAGGGGATTTTAATAGAACTACATTGAATATACTAAATGATAAGGGGTGAACTATATCTTTTAGAATT

AATTCCTTTTTCTTCTTGATTACCTGCAGCTATGGCAGCTCCGACTCCTGTTTCTGCTTTAGTACATTCTTCTACATTAG

AATTTATTTTCAAAATGTAAAATCTTATAATGCTGGTATATTAACAGCTTTATCTAATCGAATCGGAGATGTTGCCTTAT

CTTTACATTCAATATCTATTGTAATAACTTTTTTATTTGATTGAATAAGTTTAATATTTATATCTTTTGTTTTATTAATT

AATTTTTATTAGATTTACTTTATTTATTTTGAGATTAAAGTTTTTATTAATAGATTTAGTATATTTTATTGAATGAGAAATT

GAGGGTATCAACCTGAACGTTTACAGGCGGGGATTTATTTATTATTTTATACTTTACTGGCTTCGTTGCCGTTATTAATT

AATTATGAAAGTTACTTTAGTATATTTTTTTTAACTTTTTGTGTTTGTGAAGGGGTTCTAGGGCTATCTATTTTAGTTTC

AATTATTATATTTATTTTTGGAAGTATTGTTTTTATTTCTAGTCGTAAGCATTTACTTTGTACTTTATTGAGATTAGAAT

AATTTTTATAGGAGGAATATTAGTTTTATTCATTTATGTTACTTCTCTTTCATCTAATGAAATATTTTCATTATCTATAAAACTATTTTTTTTATCTTTAAGTATAATT

AATTTTAACAGGATTATTCTTAGCAATACACTATACTGCTGATATTGAAACAGCTTTTAATAGAGTAAATCACATTTATCGTGATGTTAATAATGGTTGATTCCTACGAATT

CTGTTGATAATGCTACTTTAACTCGATTCTTTACATTTCATTTTATTTTACCTTTCATTGTATTAGCTTTAACTATAATT

CTTTTGATTTTGCTGAAGGGGAGTCTGAGTTAGTTTCAGGATTTAATGTAGAATATAGAAGAGGGGGATTTGCTTTAATT

AATTTTTTTATATATCAGAAATACATATGATTTATTGTATTTTGTTTTCCTTTAGGTCTCGTTTGATTTGCATCTTGTTT

AATTTAAGCTATAAACTTTTTGTAATAAACTAGGATTAGATACCCTATTATTAAAAATAAATATTAAAATGCTAAAGTAG

>Gv10-A-03

NNNNNNNNNNNNNNNNNNNNNNNNNNNNNNNNNNNNNNNNNNNNNNNNNNNNNNNNNNNNNNNNNNNNNNNNNNNNNNNN

AATTAATCATATTGGTTGAATATTAATAGCTATAATAAATAACGAACTTTTATGACTAACTTATTTTTTATTATATTCAANN

CAACAAATCATAAAGATATTGGAACTTTATATTTCATTTTTGGAGTATGATCCGGAATAGTCGGAACTTCTCTAAGAATT

ANTTGGATTATTGGGATTTATTGTTTGAGCTCATCATATATTTACAGTAGGTATAGACGTAGATACTCGAGCTTATTTTANNNNNNNNNNNNNNNNN

CATGATACTTATTATGTAGTTGCCCATTTTCATTATGTTTTATCTATAGGAGCTGTATTTGCTATTATAGCAGGATTTATTCATTGATACCCTTTATTAACAGGAATAGTTATAAACCCTTCATG

CTGGAATACCTCGACGATACTCAGATTTTCCCGATAGTTACTTAACTTGAAATATTATTTCTTCTTTAGGAAGAACAATT

AATTTGTCACACACAACATATATTTGCTCATTTAGTTCCTCAAGGAACTCCCCCTGTTTTAATACCTTTTATAGTATGCATTGAAACTATTAGTAATGTAATCCGACCAGGAACTTTAGCAGTACGATTAACTGCTAATATAATT

GACGAGATATTTCTCGAGAAGGAACTTTTCAAGGACTTCACACTATTCCCGTAACATTAGGATTACGATGAGGAATAATTTTATTTATTATTTCTGAAGTTTTTTTCTTTATTTCCTTCTTTTGAGCTTTTTTCCATAGTAGCTTATCCCCAACAATCGAATT

NNNNNNNNNNNNNNNNNNNGTCATCATTTTGGATTTGAAGCAGCTGCTTGATATTGACATTTTGTTGATGTAGTTTGACTATTCTTATATATTTCAATT

ANTTTTAGGAATATATTCTTATAAAATGTTTGATCAAGGATGAAGTGAATATTTTGGAGGTCAGATATTATATAATCAAT

ATTATTCAATAACAGGGGATTTTAATAGAACTACATTGAATATACTAAATGATAAGGGGTGAACTATATCTTTTAGAATT

AATTCCTTTTTCTTCTTGATTACCTGCAGCTATGGCAGCTCCGACTCCTGTTTCTGCTTTAGTACATTCTTCTACATTAG

AATTTATTTTCAAAATGTAAAATCTTATAATGCTGGTATATTAACAGCTTTATCTAATCGAATCGGAGATGTTGCCTTAT

NNNNNNNNNNNNNNNNNNNNNNNNNNNNNNNNNNNNNNNNNNNNNNNNNNNNNNNNNNNNNNNNNNNNNNNNNNNNNNNN

NNTTTTTATTAGATTTACTTTATTTATTTTGAGATTAAAGTTTTTATTAATAGATTTAGTATATTTTATTGAATGAGAAATT

GAGGGTATCAACCTGAACGTTTACAGGCGGGGATTTATTTATTATTTTATACTTTACTGGCTTCGTTGCCGTTATTAATT

ANTTATGAAAGTTACTTTAGTATATTTTTTTTAACTTTTTGTGTTTGTGAAGGGGTTCTAGGGCTATCTATTTTAGTTTC

ANTTATTATATTTATTTTTGGAAGTATTGTTTTTATTTCTAGTCGTAAGCATTTACTTTGTACTTTATTGAGATTAGAGT

NNNNNNNNNNNNNNNNNNNNNNNNNNNNNTCATTTATGTTACTTCTCTTTCATCTAATGAAATATTTTCATTATCTATAAAACTATTTTTTTTATCTTTAAGTATAATT

AATTTTAACAGGATTATTCTTAGCAATACACTATACTGCTGATATTGAAACAGCTTTTAATAGAGTAAATCACATTTATCGTGATGTTAATAATGGTTGATTCCTACGAATT

CTGTTGATAATGCTACTTTAACTCGATTCTTTACATTTCATTTTATTTTACCTTTCATTGTATTAGCTTTAACTATAATT

CTTTTGATTTTGCTGAAGGGGAGTCTGAGTTAGTTTCAGGATTTAATGTAGAATATAGAAGAGGGGGATTTGCTTTAATT

AATTTTTTTATATATCAGAAATACATATGATTTATTGTATTTTGTTTTCCTTTAGGTCTCGTTTGATTTGCATCTTGTTT

AATTTAAGCTATAAACTTTTTGTAATAAACTAGGATTAGATACCCTATTATTAAAAATAAATATTAAAATGCTAAAGTAG

>Gv10-A-04

AATTTTATCTACTTTATTATTAAAAAGAGGAGCAGCTCCTTTTCATTTTTGATTCCCAGGAGTTATAGAAGGATTAAATT

AATTAATCATATTGGTTGAATATTAATAGCTATAATAAATAACGAACTTTTATGACTAACTTATTTTTTATTATATTCAATT

CAACAAATCATAAAGATATTGGAACTTTATATTTCATTTTTGGAGTATGATCCGGAATAGTCGGAACTTCTCTAAGAATT

AATTGGATTATTGGGATTTATTGTTTGAGCTCATCATATATTTACAGTAGGTATAGACGTAGATACTCGAGCTTATTTTACTTCAGCAACTATAATT

CATGATACTTATTATGTAGTTGCCCATTTTCATTATGTTTTATCTATAGGAGCTGTATTTGCTATTATAGCAGGATTTATTCATTGATACCCTTTATTAACAGGAATAGTTATAAACCCTTCATG

CTGGAATACCTCGACGATACTCAGATTTTCCCGATAGTTACTTAACTTGAAATATTATTTCTTCTTTAGGAAGAACAATT

AATTTGTCACACACAACATATATTTGCTCATTTAGTTCCTCAAGGAACTCCCCCTGTTTTAATACCTTTTATAGTATGCATTGAAACTATTAGTAATGTAATCCGACCAGGAACTTTAGCAGTACGATTAACTGCTAATATAATT

GACGAGATATTTCTCGAGAAGGAACTTTTCAAGGACTTCACACTATTCCCGTAACATTAGGATTACGATGAGGAATAATTTTATTTATTATTTCTGAAGTTTTTTTCTTTATTTCCTTCTTTTGAGCTTTTTTCCATAGTAGCTTATCCCCAACAATCGAATT

AATTGTCATTTTTCTAGAAGTCATCATTTTGGATTTGAAGCAGCTGCTTGATATTGACATTTTGTTGATGTAGTTTGACTATTCTTATATATTTCAATT

AATTTTAGGAATATATTCTTATAAAATGTTTGATCAAGGATGAAGTGAATATTTTGGAGGTCAGATATTATATAATCAAT

ATTATTCAATAACAGGGGATTTTAATAGAACTACATTGAATATACTAAATGATAAGGGGTGAACTATATCTTTTAGAATT

AATTCCTTTTTCTTCTTGATTACCTGCAGCTATGGCAGCTCCGACTCCTGTTTCTGCTTTAGTACATTCTTCTACATTAG

AATTTATTTTCAAAATGTAAAATCTTATAATGCTGGTATATTAACAGCTTTATCTAATCGAATCGGAGATGTTGCCTTAT

CTTTACATTCAATATCTATTGTAATAACTTTTTTATTTGATTGAATAAGTTTAATATTTATATCTTTTGTTTTATTAATT

AATTTTTATTAGATTTACTTTATTTATTTTGAGATTAAAGTTTTTATTAATAGATTTAGTATATTTTATTGAATGAGAAATT

GAGGGTATCAACCTGAACGTTTACAGGCGGGGATTTATTTATTATTTTATACTTTACTGGCTTCGTTGCCGTTATTAATT

AATTATGAAAGTTACTTTAGTATATTTTTTTTAACTTTTTGTGTTTGTGAAGGGGTTCTAGGGCTATCTATTTTAGTTTC

AATTATTATATTTATTTTTGGAAGTATTGTTTTTATTTCTAGTCGTAAGCATTTACTTTGTACTTTATTGAGATTAGAGT

AATTTTTATAGGAGGAATATTAGTTTTATTCATTTATGTTACTTCTCTTTCATCTAATGAAATATTTTCATTATCTATAAAACTATTTTTTTTATCTTTAAGTATAATT

AATTTTAACAGGATTATTCTTAGCAATACACTATACTGCTGATATTGAAACAGCTTTTAATAGAGTAAATCACATTTATCGTGATGTTAATAATGGTTGATTCCTACGAATT

CTGTTGATAATGCTACTTTAACTCGATTCTTTACATTTCATTTTATTTTACCTTTCATTGTATTAGCTTTAACTATAATT

CTTTTGATTTTGCTGAAGGGGAGTCTGAGTTAGTTTCAGGATTTAATGTAGAATATAGAAGAGGGGGATTTGCTTTAATT

AATTTTTTTATATATCAGAAATACATATGATTTATTGTATTTTGTTTTCCTTTAGGTCTCGTTTGATTTGCATCTTGTTT

AATTTAAGCTATAAACTTTTTGTAATAAACTAGGATTAGATACCCTATTATTAAAAATAAATATTAAAATGCTAAAGTAG

>Gv10-A-05

AATTTTATCTACTTTATTATTAAAAAGAGGAGCAGCTCCTTTTCATTTTTGATTCCCAGGAGTTATAGAAGGATTAAATT

AATTAATCATATTGGTTGAATATTAATAGCTATAATAAATAACGAACTTTTATGACTAACTTATTTTTTATTATATTCAANT

CAACAAATCATAAAGATATTGGAACTTTATATTTCATTTTTGGAGTATGATCCGGAATAGTCGGAACTTCTCTAAGAATT

AATTGGATTATTGGGATTTATTGTTTGAGCTCATCATATATTTACAGTAGGTATAGACGTAGATACTCGAGCTTATTTTACTTCAGCAACTATAATT

CATGATACTTATTATGTAGTTGCCCATTTTCATTATGTTTTATCTATAGGAGCTGTATTTGCTATTATAGCAGGATTTATTCATTGATACCCTTTATTAACAGGAATAGTTATAAACCCTTCATG

CTGGAATACCTCGACGATACTCAGATTTTCCCGATAGTTACTTAACTTGAAATATTATTTCTTCTTTAGGAAGAACAATT

AATTTGTCACACACAACATATATTTGCTCATTTAGTTCCTCAAGGAACTCCCCCTGTTTTAATACCTTTTATAGTATGCATTGAAACTATTAGTAATGTAATCCGACCAGGAACTTTAGCAGTACGATTAACTGCTAATATAATT

GACGAGATATTTCTCGAGAAGGAACTTTTCAAGGACTTCACACTATTCCCGTAACATTAGGATTACGATGAGGAATAATTTTATTTATTATTTCTGAAGTTTTTTTCTTTATTTCCTTCTTTTGAGCTTTTTTCCATAGTAGCTTATCCCCAACAATCGAATT

AATTGTCATTTTTCTAGAAGTCATCATTTTGGATTTGAAGCAGCTGCTTGATATTGACATTTTGTTGATGTAGTTTGACTATTCTTATATATTTCAATT

AATTTTAGGAATATATTCTTATAAAATGTTTGATCAAGGATGAAGTGAATATTTTGGAGGTCAGATATTATATAATCAAT

ATTATTCAATAACAGGGGATTTTAATAGAACTACATTGAATATACTAAATGATAAGGGGTGAACTATATCTTTTAGAATT

AATTCCTTTTTCTTCTTGATTACCTGCAGCTATGGCAGCTCCGACTCCTGTTTCTGCTTTAGTACATTCTTCTACATTAG

AATTTATTTTCAAAATGTAAAATCTTATAATGCTGGTATATTAACAGCTTTATCTAATCGAATCGGAGATGTTGCCTTAT

CTTTACATTCAATATCTATTGTAATAACTTTTTTATTTGATTGAATAAGTTTAATATTTATATCTTTTGTTTTATTAANT

AATTTTTATTAGATTTACTTTATTTATTTTGAGATTAAAGTTTTTATTAATAGATTTAGTATATTTTATTGAATGAGAAATT

GAGGGTATCAACCTGAACGTTTACAGGCGGGGATTTATTTATTATTTTATACTTTACTGGCTTCGTTGCCGTTATTAATT

AATTATGAAAGTTACTTTAGTATATTTTTTTTAACTTTTTGTGTTTGTGAAGGGGTTCTAGGGCTATCTATTTTAGTTTC

AATTATTATATTTATTTTTGGAAGTATTGTTTTTATTTCTAGTCGTAAGCATTTACTTTGTACTTTATTGAGATTAGAGT

AATTTTTATAGGAGGAATATTAGTTTTATTCATTTATGTTACTTCTCTTTCATCTAATGAAATATTTTCATTATCTATAAAACTATTTTTTTTATCTTTAAGTATAATT

AATTTTAACAGGATTATTCTTAGCAATACACTATACTGCTGATATTGAAACAGCTTTTAATAGAGTAAATCACATTTATCGTGATGTTAATAATGGTTGATTCCTACGAATT

CTGTTGATAATGCTACTTTAACTCGATTCTTTACATTTCATTTTATTTTACCTTTCATTGTATTAGCTTTAACTATAATT

CTTTTGATTTTGCTGAAGGGGAGTCTGAGTTAGTTTCAGGATTTAATGTAGAATATAGAAGAGGGGGATTTGCTTTAATT

AATTTTTTTATATATCAGAAATACATATGATTTATTGTATTTTGTTTTCCTTTAGGTCTCGTTTGATTTGCATCTTGTTT

AATTTAAGCTATAAACTTTTTGTAATAAACTAGGATTAGATACCCTATTATTAAAAATAAATATTAAAATGCTAAAGTAG

>Gv10-A-06

AATTTTATCTACTTTATTATTAAAAAGAGGGGCAGCTCCTTTTCATTTTTGATTCCCAGGAGTTATAGAAGGATTAAATT

AATTAATCATATTGGTTGAATATTAATAGCTATAATAAATAACGAACTTTTATGACTAACTTATTTTTTATTATATTCAANT

CAACAAATCATAAAGATATTGGAACTTTATATTTCATTTTTGGAGTATGATCCGGAATAGTCGGAACTTCTCTAAGAATT

AATTGGATTATTGGGATTTATTGTTTGAGCTCATCATATATTTACAGTAGGTATAGACGTAGATACTCGAGCTTATTTTACTTCAGCAACTATAATT

CATGATACTTATTATGTAGTTGCCCATTTTCATTATGTTTTATCTATAGGAGCTGTATTTGCTATTATAGCAGGATTTATTCATTGATACCCTTTATTAACAGGAATAGTTATAAACCCTTCATG

CTGGAATACCTCGACGATACTCAGATTTTCCCGATAGTTACTTAACTTGAAATATTATTTCTTCTTTAGGAAGAACAATT

AATTTGTCACACACAACATATATTTGCTCATTTAGTTCCTCAAGGAACTCCCCCTGTTTTAATACCTTTTATAGTATGCATTGAAACTATCAGTAATGTAATCCGACCAGGAACTTTAGCAGTACGATTAACTGCTAATATAATT

GACGAGATATTTCTCGAGAAGGAACTTTTCAAGGACTTCACACTATTCCCGTAACATTAGGATTACGATGAGGAATAATTTTATTTATTATTTCTGAAGTTTTTTTCTTTATTTCCTTCTTTTGAGCTTTTTTCCATAGTAGCTTATCCCCAACAATCGAATT

AATTGTCATTTTTCTAGAAGTCATCATTTTGGATTTGAAGCAGCTGCTTGATATTGACATTTTGTTGATGTAGTTTGACTATTCTTATATATTTCAATT

ANTTTTAGGAATATATTCTTATAAAATGTTTGATCAAGGATGAAGTGAATATTTTGGAGGTCAGATATTATATAATCAAT

ATTATTCAATAACAGGGGATTTTAATAGAACTACATTGAATATACTAAATGATAAGGGGTGAACTATATCTTTTAGAATT

AATTCCTTTTTCTTCTTGATTACCTGCAGCTATGGCAGCTCCGACTCCTGTTTCTGCTTTAGTACATTCTTCTACATTAG

AATTTATTTTCAAAATGTAAAATCTTATAATGCTGGTATATTAACAGCTTTATCTAATCGAATCGGAGATGTTGCCTTAT

CTTTACATTCAATATCTATTGTAATAACTTTTTTATTTGATTGAATAAGTTTAATATTTATATCTTTTGTTTTATTAATT

AATTTTTATTAGATTTACTTTATTTATTTTGAGATTAAAGTTTTTATTAATAGATTTAGTATATTTTATTGAATGAGAAATT

GAGGGTATCAACCTGAACGTTTACAGGCGGGGATTTATTTATTATTTTATACTTTACTGGCTTCGTTGCCGTTATTAATT

AATTATGAAAGTTACTTTAGTATATTTTTTTTAACTTTTTGTGTTTGTGAAGGGGTTCTAGGGCTATCTATTTTAGTTTC

ANTTATTATATTTATTTTTGGAAGTATTGTTTTTATTTCTAGTCGTAAGCATTTACTTTGTACTTTATTGAGATTAGAAT

AATTTTTATAGGAGGAATATTAGTTTTATTCATTTATGTTACTTCTCTTTCATCTAATGAAATATTTTCATTATCTATAAAACTATTTTTTTTATCTTTAAGTATAATT

AATTTTAACAGGATTATTCTTAGCAATACACTATACTGCTGATATTGAAACAGCTTTTAATAGAGTAAATCACATTTATCGTGATGTTAATAATGGTTGATTCCTACGAATT

CTGTTGATAATGCTACTTTAACTCGATTCTTTACATTTCATTTTATTTTACCTTTCATTGTATTAGCTTTAACTATAATT

CTTTTGATTTTGCTGAAGGGGAGTCTGAGTTAGTTTCAGGATTTAATGTAGAATATAGAAGAGGGGGATTTGCTTTAATT

AATTTTTTTATATATCAGAAATACATATGATTTATTGTATTTTGTTTTCCTTTAGGTCTCGTTTGATTTGCATCTTGTTT

AATTTAAGCTATAAACTTTTTGTAATAAACTAGGATTAGATACCCTATTATTAAAAATAAATATTAAAATGCTAAAGTAG

>Gv10-A-07

AATTTTATCTACTTTATTATTAAAAAGAGGAGCAGCTCCTTTTCATTTTTGATTCCCAGGAGTTATAGAAGGATTAAATT

AATTAATCATATTGGTTGAATATTAATAGCTATAATAAATAACGAACTTTTATGGCTAACTTATTTTTTATTATATTCAANT

CAACAAATCATAAAGATATTGGAACTTTATATTTCATTTTTGGAGTATGATCCGGAATAGTCGGAACTTCTCTAAGAATT

AATTGGATTATTGGGATTTATTGTTTGAGCTCATCATATATTTACAGTAGGTATAGACGTAGATACTCGAGCTTATTTTACTTCAGCAACTATAATT

CATGATACTTATTATGTAGTTGCCCATTTTCATTATGTTTTATCTATAGGAGCTGTATTTGCTATTATAGCAGGATTTATTCATTGATACCCTTTATTAACAGGAATAGTTATAAACCCTTCATG

CTGGAATACCTCGACGATACTCAGATTTTCCCGATAGTTACTTAACTTGAAATATTATTTCTTCTTTAGGAAGAACAATT

AATTTGTCACACACAACATATATTTGCTCATTTAGTTCCTCAAGGAACTCCCCCTGTTTTAATACCTTTTATAGTATGCATTGAAACTATTAGTAATGTAATCCGACCAGGAACTTTAGCAGTACGATTAACTGCTAATATAATT

GACGAGATATTTCTCGAGAAGGAACTTTTCAAGGACTTCACACTATTCCCGTAACATTAGGATTACGATGAGGAATAATTTTATTTATTATTTCTGAAGTTTTTTTCTTTATTTCCTTCTTTTGAGCTTTTTTCCATAGTAGCTTATCCCCAACAATCGAATT

AATTGTCATTTTTCTAGAAGTCATCATTTTGGATTTGAAGCAGCTGCTTGATATTGACATTTTGTTGATGTAGTTTGACTATTCTTATATATTTCAATT

AATTTTAGGAATATATTCTTATAAAATGTTTGATCAAGGATGAAGTGAATATTTTGGAGGTCAGATATTATATAATCAAT

ATTATTCAATAACAGGGGATTTTAATAGAACTACATTGAATATACTAAATGATAAGGGGTGAACTATATCTTTTAGAATT

AATTCCTTTTTCTTCTTGATTACCTGCAGCTATGGCAGCTCCGACTCCTGTTTCTGCTTTAGTACATTCTTCTACATTAG

AATTTATTTTCAAAATGTAAAATCTTATAATGCTGGTATATTAACAGCTTTATCTAATCGAATCGGAGATGTTGCCTTAT

CTTTACATTCAATATCTATTGTAATAACTTTTTTATTTGATTGAATAAGTTTAATATTTATATCTTTTGTTTTATTAATT

AATTTTTATTAGATTTACTTTATTTATTTTGAGATTAAAGTTTTTATTAATAGATTTAGTATATTTTATTGAATGAGAAATT

GAGGGTATCAACCTGAACGTTTACAGGCGGGGATTTATTTATTATTTTATACTTTACTGGCTTCGTTGCCGTTATTAATT

AATTATGAAAGTTACTTTAGTATATTTTTTTTAACTTTTTGTGTTTGTGAAGGGGTTCTAGGGCTATCTATTTTAGTTTC

AATTATTATATTTATTTTTGGAAGTATTGTTTTTATTTCTAGTCGTAAGCATTTACTTTGTACTTTATTGAGATTAGAGT

AATTTTTATAGGAGGAATATTAGTTTTATTCATTTATGTTACTTCTCTTTCATCTAATGAAATATTTTCATTATCTATAAAACTATTTTTTTTATCTTTAAGTATAATT

AATTTTAACAGGATTATTCTTAGCAATACACTATACTGCTGATATTGAAACAGCTTTTAATAGAGTAAATCACATTTATCGTGATGTTAATAATGGTTGATTCCTACGAATT

CTGTTGATAATGCTACTTTAACTCGATTCTTTACATTTCATTTTATTTTACCTTTCATTGTATTAGCTTTAACTATAATT

CTTTTGATTTTGCTGAAGGGGAGTCTGAGTTAGTTTCAGGATTTAATGTAGAATATAGAAGAGGGGGATTTGCTTTAATT

AATTTTTTTATATATCAGAAATACATATGATTTATTGTATTTTGTTTTCCTTTAGGTCTCGTTTGATTTGCATCTTGTTT

AATTTAAGCTATAAACTTTTTGTAATAAACTAGGATTAGATACCCTATTATTAAAAATAAATATTAAAATGCTAAAGTAG

>Gv10-A-08

AATTTTATCTACTTTATTATTAAAAAGAGGGGCAGCTCCTTTTCATTTTTGATTCCCAGGGGTTATAGAAGGATTAAATT

NNNNNNNNNNNNNNNNNNNNNNNNNNNNNNNNNNNNNNNNNNNNNNNNNNNNNNNNNNNNNNNNNNNNNNNNNNNNNNNNNN

CAACAAATCATAAAGATATTGGAACTTTATATTTCATTTTTGGAGTATGATCTGGAATAGTCGGAACTTCTCTAAGAATT

NNNNNNNNNNNNNNNNNTTATTGTTTGAGCTCATCATATATTTACAGTAGGTATAGATGTAGATACTCGAGCTTATTTTACTTCAGCAACTATAATT

CATGATACTTATTACGTAGTTGCCCATTTTCATTACGTTTTATCTATAGGAGCTGTATTTGCTATTATAGCAGGATTTATTCATTGATACCCTTTATTAACAGGAATAGTTATAAACCCTTCATG

CTGGAATACCTCGACGATACTCAGATTTTCCTGATAGTTACTTAACTTGAAATATTATTTCTTCTTTAGGAAGAACAATT

AATTTGTCACACACAACATATATTTGCTCATTTAGTTCCTCAAGGGACTCCTCCTGTTTTAATACCTTTTATAGTATGCATTGAAACTATTAGTAATGTAATCCGACCAGGAACTTTAGCAGTACGATTGACTGCTAATATAATT

GACGAGATATTTCTCGAGAAGGAACTTTTCAAGGACTTCACACTATTCCCGTAACATTAGGATTACGATGAGGAATAATTTTATTTATTATTTCTGAAGTTTTTTTCTTTATTTCCTTCTTTTGAGCTTTTTTCCATAGTAGCTTATCTCCAACAATCGAATT

AATTGTCATTTTTCTAGAAGTCATCATTTTGGATTCGAAGCAGCTGCTTGATATTGACATTTTGTTGATGTAGTTTGACTNNNNNNNNNNNNNNNNNNN

AATTTTAGGAATATATTCTTATAAAATGTTTGATCAAGGATGAAGTGAATATTTTGGAGGTCAGATATTATATAATCAAT

ATTATTCAATAACAGGGGATTTTAATAGAACTACATTGAATATGTTAAATGATAAGGGGTGAACTATATCTTTTAGAATT

AATTCCTTTTTCTTCTTGATTACCTGCAGCTATGGCAGCACCGACTCCTGTTTCTGCTTTAGTACATTCTTCTACATTAG

AATTTATTTTCAAAATGTAAAATCTTATAATGCTGGTATATTAACAGCTTTATTTAATCGAATCGGGGATGTTGCTTTAT

CTTTACATTCAATATCTATTGTAATAACTTTTTTATTTGATTGAATAAGTTTAATATTTATATCTTTTGTTTTATTAANT

AATTTTTATTAGATTTACTTTATTTATTTTAAGATTAAAGTTTTTATTAATAGATTTAGTATATTTTATTGAATGAGAAATT

GAGGATATCAACCTGAACGTTTACAGGCGGGGATTTATTTATTATTTTATACTTTACTGGCTTCGTTGCCGTTATTAATT

ANTTATGAAAGTTACTTTAGTATATTTTTTTTAACTTTTTGTGTTTGTGAAGGGGTTCTAGGGCTATCTATTTTAGTTTC

AATTATTATATTTATTTTTGGAAGTATTGTTTTTATTTCTAGTCGTAAGCATTTACTTTGTACTTTATTGAGATTAGAGT

AATTTTTATAGGAGGAATATTAGTTTTATTCATTTATGTAACTTCTCTTTCATCTAATGAAATATTTTCATTATCTATAAAACTATTTTTTTTATCTTTAAGTATAATT

AATTTTAACAGGATTATTCTTAGCAATACACTATACTGCTGATATTGAAACAGCTTTTAATAGAGTAAATCACATTTATCGTGATGTTAATAATGGTTGATTCCTACGAATT

CTGTTGATAATGCTACTTTAACTCGATTCTTTACCTTTCATTTTATTCTCCCTTTCATTGTATTAGCTTTAACTATAANT

CTTTTGATTTTGCTGAAGGGGAGTCTGAGTTAGTTTCAGGATTTAATGTAGAGTATAGAAGAGGGGGATTTGCTTTAATT

AATTTTTTTATATATCAGAAATATATATGATTTATTGTATTTTGTTTTCCTTTAGGTCTCGTTTGATTTGCATCTTGTTT

AATTTAAGCTATAAACTTTTTGTAATAAACTAGGATTAGATACCCTATTATTAAAAATAAATATTAAAATGCTAAAGTAG

>Gv10-A-09

ANTTTTATCTACTTTATTATTAAAAAGAGGGGCAGCTCCTTTTCATTTTTGATTCCCAGGAGTTATAGAAGGATTAAATT

AATTAATCATATTGGTTGAATATTAATAGCTATAATAAATAACGAACTTTTATGACTAACTTATTTTTTATTATATTCAATT

CAACAAATCATAAAGATATTGGAACTTTATATTTCATTTTTGGAGTATGATCCGGAATAGTCGGAACTTCTCTAAGAATT

ANTTGGATTATTGGGATTTATTGTTTGAGCTCATCATATATTTACAGTAGGTATAGACGTAGATACTCGAGCTTATTTTANNNNNNNNNNNNNNNNN

CATGATACTTATTATGTAGTTGCCCATTTTCATTATGTTTTATCTATAGGAGCTGTATTTGCTATTATAGCAGGATTTATTCATTGATACCCTTTATTAACAGGAATAGTTATAAACCCTTCATG

NNNNNNNNNNNNNNNNNNNNNNNNNNNNNNNNNNNNNNNNNNNNNNNNNNNNNNNNNNNNNNNNNNNNNNNNNNNNNNNN

AATTTGTCACACACAACATATATTTGCTCATTTAGTTCCTCAAGGAACTCCCCCTGTTTTAATACCTTTTATAGTATGCATTGAAACTATCAGTAATGTAATCCGACCAGGAACTTTAGCAGTACGATTAACTGCTAATATAATT

GACGAGATATTTCTCGAGAAGGAACTTTTCAAGGACTTCACACTATTCCCGTAACATTAGGATTACGATGAGGAATAATTTTATTTATTATTTCTGAAGTTTTTTTCTTTATTTCCTTCTTTTGAGCTTTTTTCCATAGTAGCTTATCCCCAACAATCGAATT

AATTGTCATTTTTCTAGAAGTCATCATTTTGGATTTGAAGCAGCTGCTTGATATTGACATTTTGTTGATGTAGTTTGACTATTCTTATATATTTCAATT

ANTTTTAGGAATATATTCTTATAAAATGTTTGATCAAGGATGAAGTGAATATTTTGGAGGTCAGATATTATATAATCAAT

ATTATTCAATAACAGGGGATTTTAATAGAACTACATTGAATATACTAAATGATAAGGGGTGAACTATATCTTTTAGAATT

AATTCCTTTTTCTTCTTGATTACCTGCAGCTATGGCAGCTCCGACTCCTGTTTCTGCTTTAGTACATTCTTCTACATTAG

AATTTATTTTCAAAATGTAAAATCTTATAATGCTGGTATATTAACAGCTTTATCTAATCGAATCGGAGATGTTGCCTTAT

NNNNNNNNNNNNNNNNNNNNNNNNNNNNNNNNNNNNNNNNNNNNNNNNNNNNNNNNNNNNNNNNNNNNNNNNNNNNNNNN

AATTTTTATTAGATTTACTTTATTTATTTTGAGATTAAAGTTTTTATTAATAGATTTAGTATATTTTATTGAATGAGAAATT

GAGGGTATCAACCTGAACGTTTACAGGCGGGGATTTATTTATTATTTTATACTTTACTGGCTTCGTTGCCGTTATTAATT

AATTATGAAAGTTACTTTAGTATATTTTTTTTAACTTTTTGTGTTTGTGAAGGGGTTCTAGGGCTATCTATTTTAGTTTC

ANTTATTATATTTATTTTTGGAAGTATTGTTTTTATTTCTAGTCGTAAGCATTTACTTTGTACTTTATTGAGATTAGAAT

AATTTTTATAGGAGGAATATTAGTTTTATTCATTTATGTTACTTCTCTTTCATCTAATGAAATATTTTCATTATCTATAAAACTATTTTTTTTATCTTTAAGTATAATT

AATTTTAACAGGATTATTCTTAGCAATACACTATACTGCTGATATTGAAACAGCTTTTAATAGAGTAAATCACATTTATCGTGATGTTAATAATGGTTGATTCCTACGAATT

CTGTTGATAATGCTACTTTAACTCGATTCTTTACATTTCATTTTATTTTACCTTTCATTGTATTAGCTTTAACTATAATT

CTTTTGATTTTGCTGAAGGGGAGTCTGAGTTAGTTTCAGGATTTAATGTAGAATATAGAAGAGGGGGATTTGCTTTAATT

AATTTTTTTATATATCAGAAATACATATGATTTATTGTATTTTGTTTTCCTTTAGGTCTCGTTTGATTTGCATCTTGTTT

AATTTAAGCTATAAACTTTTTGTAATAAACTAGGATTAGATACCCTATTATTAAAAATAAATATTAAAATGCTAAAGTAG

>Gv10-A-10

ANTTTTATCTACTTTATTATTAAAAAGAGGGGCAGCTCCTTTTCATTTTTGATTCCCAGGAGTTATAGAAGGATTAAATT

AATTAATCATATTGGTTGAATATTAATAGCTATAATAAATAACGAACTTTTATGACTAACTTATTTTTTATTATATTCAANT

CAACAAATCATAAAGATATTGGAACTTTATATTTCATTTTTGGAGTATGATCCGGAATAGTCGGAACTTCTCTAAGAATT

NNNNNNNNNNNNNNNNNNNNNNNNNNNNNNNNNNNNNNNNNNNNNNNNNNNNNNNNNNNNNNNNNNNNNNNNNNNNNNNNNNNNNNNNNNNNNNNNN

CATGATACTTATTATGTAGTTGCCCATTTTCATTATGTTTTATCTATAGGAGCTGTATTTGCTATTATAGCAGGATTTATTCATTGATACCCTTTATTAACAGGAATAGTTATAAACCCTTCATG

NNNNNNNNNNNNNNNNNNNNNNNNNNNNNNNNNNNNNNNNNNNNNNNNNNNNNNNNNNNNNNNNNNNNNNNNNNNNNNNN

ANTTTGTCACACACAACATATATTTGCTCATTTAGTTCCTCAAGGAACTCCCCCTGTTTTAATACCTTTTATAGTATGCATTGAAACTATCAGTAATGTAATCCGACCAGGAACTTTAGCAGTACGATTAACTGCTAATATAATT

GACGAGATATTTCTCGAGAAGGAACTTTTCAAGGACTTCACACTATTCCCGTAACATTAGGATTACGATGAGGAATAATTTTATTTATTATTTCTGAAGTTTTTTTCTTTATTTCCTTCTTTTGAGCTTTTTTCCATAGTAGCTTATCCCCAACAATCGAATT

AATTGTCATTTTTCTAGAAGTCATCATTTTGGATTTGAAGCAGCTGCTTGATATTGACATTTTGTTGATGTAGTTTGACTNNNNNNNNNNNNNNNNNNN

AATTTTAGGAATATATTCTTATAAAATGTTTGATCAAGGATGAAGTGAATATTTTGGAGGTCAGATATTATATAATCAAT

ATTATTCAATAACAGGGGATTTTAATAGAACTACATTGAATATACTAAATGATAAGGGGTGAACTATATCTTTTAGAATT

AATTCCTTTTTCTTCTTGATTACCTGCAGCTATGGCAGCTCCGACTCCTGTTTCTGCTTTAGTACATTCTTCTACATTAG

ANTTTATTTTCAAAATGTAAAATCTTATAATGCTGGTATATTAACAGCTTTATCTAATCGAATCGGAGATGTTGCCTTAT

CTTTACATTCAATATCTATTGTAATAACTTTTTTATTTGATTGAATAAGTTTAATATTTATATCTTTTGTTTTATTAATT

AATTTTTATTAGATTTACTTTATTTATTTTGAGATTAAAGTTTTTATTAATAGATTTAGTATATTTTATTGAATGAGAAATT

GAGGGTATCAACCTGAACGTTTACAGGCGGGGATTTATTTATTATTTTATACTTTACTGGCTTCGTTGCCGTTATTAATT

AATTATGAAAGTTACTTTAGTATATTTTTTTTAACTTTTTGTGTTTGTGAAGGGGTTCTAGGGCTATCTATTTTAGTTTC

ANTTATTATATTTATTTTTGGAAGTATTGTTTTTATTTCTAGTCGTAAGCATTTACTTTGTACTTTATTGAGATTAGAAT

AATTTTTATAGGAGGAATATTAGTTTTATTCATTTATGTTACTTCTCTTTCATCTAATGAAATATTTTCATTATCTATAAAACTATTTTTTTTATCTTTAAGTATAATT

AATTTTAACAGGATTATTCTTAGCAATACACTATACTGCTGATATTGAAACAGCTTTTAATAGAGTAAATCACATTTATCGTGATGTTAATAATGGTTGATTCCTACGAACT

CTGTTGATAATGCTACTTTAACTCGATTCTTTACATTTCATTTTATTTTACCTTTCATTGTATTAGCTTTAACTATAATT

CTTTTGATTTTGCTGAAGGGGAGTCTGAGTTAGTTTCAGGATTTAATGTAGAATATAGAAGAGGGGGATTTGCTTTAATT

AATTTTTTTATATATCAGAAATACATATGATTTATTGTATTTTGTTTTCCTTTAGGTCTCGTTTGATTTGCATCTTGTTT

AATTTAAGCTATAAACTTTTTGTAATAAACTAGGATTAGATACCCTATTATTAAAAATAAATATTAAAATGCTAAAGTAG

>Gv10-B-02

AATTTTATCTACTTTATTATTAAAAAGAGGGGCAGCTCCTTTTCATTTTTGATTCCCAGGAGTTATAGAAGGATTAAATT

AATTAATCATATTGGTTGAATATTAATAGCTATAATAAATAACGAACTTTTATGACTAACTTATTTTTTATTATATTCAANN

CAACAAATCATAAAGATATTGGAACTTTATATTTCATTTTTGGAGTATGATCCGGAATAGTCGGAACTTCTCTAAGAATT

ANTTGGATTATTGGGATTTATTGTTTGAGCTCATCATATATTTACAGTAGGTATAGACGTAGATACTCGAGCTTATTTTACTTCAGCAACTATAATT

CATGATACTTATTATGTAGTTGCCCATTTTCATTATGTTTTATCTATAGGAGCTGTATTTGCTATTATAGCAGGATTTATTCATTGATACCCTTTATTAACAGGAATAGTTATAAACCCTTCATG

CTGGAATACCTCGACGATACTCAGATTTTCCTGATAGTTACTTAACTTGAAATATTGTTTCTTCTTTAGGAAGAACAATT

AATTTGTCACACACAACATATATTTGCTCATTTAGTTCCTCAAGGAACTCCCCCTGTTTTAATACCTTTTATAGTATGCATTGAAACTATCAGTAATGTAATCCGACCAGGAACTTTAGCAGTACGATTAACTGCTAATATAATT

GACGAGATATTTCTCGAGAAGGAACTTTTCAAGGACTTCACACTATTCCCGTAACATTAGGATTACGATGAGGAATAATTTTATTTATTATTTCTGAAGTTTTTTTCTTTATTTCCTTCTTTTGAGCTTTTTTCCATAGTAGCTTATCCCCAACAATCGAATT

AATTGTCATTTTTCTAGAAGTCATCATTTTGGATTTGAAGCAGCTGCTTGATATTGACATTTTGTTGATGTAGTTTGACTNNNNNNNNNNNNNNNNNNN

ANTTTTAGGAATATATTCTTATAAAATGTTTGATCAAGGATGAAGTGAATATTTTGGAGGTCAGATATTATATAATCAAT

ATTATTCAATAACAGGGGATTTTAATAGAACTACATTGAATATACTAAATGATAAGGGGTGAACTATATCTTTTAGAATT

ANTTCCTTTTTCTTCTTGATTACCTGCAGCTATGGCAGCTCCGACTCCTGTTTCTGCTTTAGTACATTCTTCTACATTAG

AATTTATTTTCAAAATGTAAAATCTTATAATGCTGGTATATTAACAGCTTTATCTAATCGAATCGGAGATGTTGCCTTAT

CTTTACATTCAATATCTATTGTAATAACTTTTTTATTTGATTGAATAAGTTTAATATTTATATCTTTTGTTTTATTAATT

AATTTTTATTAGATTTACTTTATTTATTTTGAGATTAAAGTTTTTATTAATAGATTTAGTATATTTTATTGAATGAGAAATT

GAGGGTATCAACCTGAACGTTTACAGGCGGGGATTTATTTATTATTTTATACTTTACTGGCTTCGTTGCCGTTATTAATT

AATTATGAAAGTTACTTTAGTATATTTTTTTTAACTTTTTGTGTTTGTGAAGGGGTTCTAGGGCTATCTATTTTAGTTTC

AATTATTATATTTATTTTTGGAAGTATTGTTTTTATTTCTAGTCGTAAGCATTTACTTTGTACTTTATTGAGATTAGAAT

AATTTTTATAGGAGGAATATTAGTTTTATTCATTTATGTTACTTCTCTTTCATCTAATGAAATATTTTCATTATCTATAAAACTATTTTTTTTATCTTTAAGTATAATT

AATTTTAACAGGATTATTCTTAGCAATACACTATACTGCTGATATTGAAACAGCTTTTAATAGAGTAAATCACATTTATCGTGATGTTAATAATGGTTGATTCCTACGAATT

CTGTTGATAATGCTACTTTAACTCGATTCTTTACATTTCATTTTATTTTACCTTTCATTGTATTAGCTTTAACTATAATT

CTTTTGATTTTGCTGAAGGGGAGTCTGAGTTAGTTTCAGGATTTAATGTAGAATATAGAAGAGGGGGATTTGCTTTAATT

AATTTTTTTATATATCAGAAATACATATGATTTATTGTATTTTGTTTTCCTTTAGGTCTCGTTTGATTTGCATCTTGTTT

AATTTAAGCTATAAACTTTTTGTAATAAACTAGGATTAGATACCCTATTATTAAAAATAAATATTAAAATGCTAAAGTAG

>Gv10-B-03

AATTTTATCTACTTTATTATTAAAAAGAGGGGCAGCTCCTTTTCATTTTTGATTCCCAGGAGTTATAGAAGGATTAAATT

AATTAATCATATTGGTTGAATATTAATAGCTATAATAAATAACGAACTTTTATGGCTAACTTATTTTTTATTATATTCAANT

CAACAAATCATAAAGATATTGGAACTTTATATTTCATTTTTGGAGTATGATCCGGAATAGTCGGAACTTCTCTAAGAANT

AATTGGATTATTGGGATTTATTGTTTGAGCTCATCATATATTTACAGTAGGTATAGACGTAGATACTCGAGCTTATTTTACTTCAGCAACTATAANT

CATGATACTTATTATGTAGTTGCCCATTTTCATTATGTTTTATCTATAGGAGCTGTATTTGCTATTATAGCAGGATTTATTCATTGATACCCTTTATTAACAGGAATAGTTATAAACCCTTCATG

CTGGAATACCTCGACGATACTCAGATTTTCCTGATAGTTACTTAACTTGAAATATTATTTCTTCTTTAGGAAGAACAATT

AATTTGTCACACACAACATATATTTGCTCATTTAGTTCCTCAAGGAACTCCCCCTGTTTTAATACCTTTTATAGTATGCATTGAAACTATCAGTAATGTAATCCGACCAGGAACTTTAGCAGTACGATTAACTGCTAATATAATT

GACGAGATATTTCTCGAGAAGGAACTTTTCAAGGACTTCACACTATTCCCGTAACATTAGGATTACGATGAGGAATAATTTTATTTATTATTTCTGAAGTTTTTTTCTTTATTTCCTTCTTTTGAGCTTTTTTCCATAGTAGCTTATCCCCAACAATCGAATT

AATTGTCATTTTTCTAGAAGTCATCATTTTGGATTTGAAGCAGCTGCTTGATATTGACATTTTGTTGATGTAGTTTGACTATTCTTATATATTTCAATT

ANTTTTAGGAATATATTCTTATAAAATGTTTGATCAAGGATGAAGTGAATATTTTGGAGGTCAGATATTATATAATCAAT

ATTATTCAATAACAGGGGATTTTAATAGAACTACATTGAATATACTAAATGATAAGGGGTGAACTATATCTTTTAGAATT

AATTCCTTTTTCTTCTTGATTACCTGCAGCTATGGCAGCTCCGACTCCTGTTTCTGCTTTAGTACATTCTTCTACATTAG

AATTTATTTTCAAAATGTAAAATCTTATAATGCTGGTATATTAACAGCTTTATCTAATCGAATCGGAGATGTTGCCTTAT

CTTTACATTCAATATCTATTGTAATAACTTTTTTATTTGATTGAATAAGTTTAATATTTATATCTTTTGTTTTATTAATT

ANTTTTTATTAGATTTACTTTATTTATTTTGAGATTAAAGTTTTTATTAATAGATTTAGTATATTTTATTGAATGAGAAATT

GAGGGTATCAACCTGAACGTTTACAGGCGGGGATTTATTTATTATTTTATACTTTACTGGCTTCGTTGCCGTTATTAATT

AATTATGAAAGTTACTTTAGTATATTTTTTTTAACTTTTTGTGTTTGTGAAGGGGTTCTAGGGCTATCTATTTTAGTTTC

AATTATTATATTTATTTTTGGAAGTATTGTTTTTATTTCTAGTCGTAAGCATTTACTTTGTACTTTATTGAGATTAGAAT

ANTTTTTATAGGAGGAATATTAGTTTTATTCATTTATGTTACTTCTCTTTCATCTAATGAAATATTTTCATTATCTATAAAACTATTTTTTTTATCTTTAAGTATAATT

AATTTTAACAGGATTATTCTTAGCAATACACTATACTGCTGATATTGAAACAGCTTTTAATAGAGTAAATCACATTTATCGTGATGTTAATAATGGTTGATTCCTACGAATT

CTGTTGATAATGCTACTTTAACTCGATTCTTTACATTTCATTTTATTTTACCTTTCATTGTATTAGCTTTAACTATAATT

CTTTTGATTTTGCTGAAGGGGAGTCTGAGTTAGTTTCAGGATTTAATGTAGAATATAGAAGAGGGGGATTTGCTTTAATT

AATTTTTTTATATATCAGAAATACATATGATTTATTGTATTTTGTTTTCCTTTAGGTCTCGTTTGATTTGCATCTTGTTT

AATTTAAGCTATAAACTTTTTGTAATAAACTAGGATTAGATACCCTATTATTAAAAATAAATATTAAAATGCTAAAGTAG

>Gv10-B-04

AATTTTATCTACTTTATTATTAAAAAGAGGGGCAGCTCCTTTTCATTTTTGATTCCCAGGAGTTATAGAAGGATTAAATT

AATTAATCATATTGGTTGAATATTAATAGCTATAATAAATAACGAACTTTTATGACTAACTTATTTTTTATTATATTCAANT

CAACAAATCATAAAGATATTGGAACTTTATATTTCATTTTTGGAGTATGATCCGGAATAGTCGGAACTTCTCTAAGAATT

ANTTGGATTATTGGGATTTATTGTTTGAGCTCATCATATATTTACAGTAGGTATAGACGTAGATACTCGAGCTTATTTTACTTCAGCAACTATAANT

CATGATACTTATTATGTAGTTGCCCATTTTCATTATGTTTTATCTATAGGAGCTGTATTTGCTATTATAGCAGGATTTATTCATTGATACCCTTTATTAACAGGAATAGTTATAAACCCTTCATG

CTGGAATACCTCGACGATACTCAGATTTTCCTGATAGTTACTTAACTTAAAATATTATTTCTTCTTTAGGAAGAACAANT

NNNNNNNNNNNNNNNNNNNNNNNNNNNNNNNNNNNNNNNNNNNNNNNNNNNNNNNNNNNNNNNNNCTTTTATAGTATGCATTGAAACTATCAGTAATGTAATCCGACCAGGAACTTTAGCAGTACGATTAACTGCTAATATAATT

GACGAGATATTTCTCGAGAAGGAACTTTTCAAGGACTTCACACTATTCCCGTAACATTAGGATTACGATGAGGAATANTTTTATTTATTATTTCTGAAGTTTTTTTCTTTATTTCCTTCTTTTGAGCTTTTTTCCATAGTAGCTTATCCCCAACAATCGAATT

AATTGTCATTTTTCTAGAAGTCATCATTTTGGATTTGAAGCAGCTGCTTGATATTGACATTTTGTTGATGTAGTTTGACTATTCTTATATATTTCAATT

AATTTTAGGAATATATTCTTATAAAATGTTTGATCAAGGATGAAGTGAATATTTTGGAGGTCAGATATTATATAATCAAT

ATTATTCAATAACAGGGGATTTTAATAGAACTACATTGAATATACTAAATGATAAGGGGTGAACTATATCTTTTAGAATT

AATTCCTTTTTCTTCTTGATTACCTGCAGCTATGGCAGCTCCGACTCCTGTTTCTGCTTTAGTACATTCTTCTACATTAG

AATTTATTTTCAAAATGTAAAATCTTATAATGCTGGTATATTAACAGCTTTATCTAATCGAATCGGAGATGTTGCCTTAT

NNNNNNNNNNNNNNNNNNNNNNNNNNNNNNNNNNNNNNNNNNNNNNNNNNNNNNNNNNNNNNNNNNNNNNNNNNNNNNNN

AATTTTTATTAGATTTACTTTATTTATTTTGAGATTAAAGTTTTTATTAATAGATTTAGTATATTTTATTGAATGAGAAATT

GAGGGTATCAACCTGAACGTTTACAGGCGGGGATTTATTTATTATTTTATACTTTACTGGCTTCGTTGCCGTTATTAATT

AATTATGAAAGTTACTTTAGTATATTTTTTTTAACTTTTTGTGTTTGTGAAGGGGTTCTAGGGCTATCTATTTTAGTTTC

ANTTATTATATTTATTTTTGGAAGTATTGTTTTTATTTCTAGTCGTAAGCATTTACTTTGTACTTTATTGAGATTAGAAT

NNNNNNNNNNNNNNNNNNNNNNNNNNNNNTCATTTATGTTACTTCTCTTTCATCTAATGAAATATTTTCATTATCTATAAAACTATTTTTTTTATCTTTAAGTATAATT

AATTTTAACAGGATTATTCTTAGCAATACACTATACTGCTGATATTGAAACAGCTTTTAATAGAGTAAATCACATTTATCGTGATGTTAATAATGGTTGATTCCTACGAATT

CTGTTGATAATGCTACTTTAACTCGATTCTTTACATTTCATTTTATTTTACCTTTCATTGTATTAGCTTTAACTATAATT

CTTTTGATTTTGCTGAAGGGGAGTCTGAGTTAGTTTCAGGATTTAATGTAGAATATAGAAGAGGGGGATTTGCTTTAATT

ANTTTTTTTATATATCAGAAATACATATGATTTATTGTATTTTGTTTTCCTTTAGGTCTCGTTTGATTTGCATCTTGTTT

ANTTTAAGCTATAAACTTTTTGTAATAAACTAGGATTAGATACCCTATTATTAAAAATAAATATTAAAATGCTAAAGTAG

>Gv10-B-06

AATTTTATCTACTTTATTATTAAAAAGAGGGGCAGCTCCTTTTCATTTTTGATTCCCAGGAGTTATAGAAGGATTAAATT

AATTAATCATATTGGTTGAATATTAATAGCTATAATAAATAACGAACTTTTATGACTAACTTATTTTTTATTATATTCAANN

CAACAAATCATAAAGATATTGGAACTTTATATTTCATTTTTGGAGTATGATCCGGAATAGTCGGAACTTCTCTAAGAATT

AATTGGATTATTGGGATTTATTGTTTGAGCTCATCATATATTTACAGTAGGTATAGACGTAGATACTCGAGCTTATTTTACTTCAGCAACTATAATT

CATGATACTTATTATGTAGTTGCCCATTTTCATTATGTTTTATCTATAGGAGCTGTATTTGCTATTATAGCAGGATTTATTCATTGATACCCTTTATTAACAGGAATAGTTATAAACCCTTCATG

CTGGAATACCTCGACGATACTCAGATTTTCCCGATAGTTACTTAACTTGAAATATTATTTCTTCTTTAGGAAGAACAANT

AATTTGTCACACACAACATATATTTGCTCATTTAGTTCCTCAAGGAACTCCCCCTGTTTTAATACCTTTTATAGTATGCATTGAAACTATCAGTAATGTAATCCGACCAGGAACTTTAGCAGTACGATTAACTGCTAATATAATT

GACGAGATATTTCTCGAGAAGGAACTTTTCAAGGACTTCACACTATTCCCGTAACATTAGGATTACGATGAGGAATAATTTTATTTATTATTTCTGAAGTTTTTTTCTTTATTTCCTTCTTTTGAGCTTTTTTCCATAGTAGCTTATCCCCAACAATCGAATT

AATTGTCATTTTTCTAGAAGTCATCATTTTGGATTTGAAGCAGCTGCTTGATATTGACATTTTGTTGATGTAGTTTGACTATTCTTATATATTTCAATT

AATTTTAGGAATATATTCTTATAAAATGTTTGATCAAGGATGAAGTGAATATTTTGGAGGTCAGATATTATATAATCAAT

ATTATTCAATAACAGGGGATTTTAATAGAACTACATTGAATATACTAAATGATAAGGGGTGAACTATATCTTTTAGAATT

AATTCCTTTTTCTTCTTGATTACCTGCAGCTATGGCAGCTCCGACTCCTGTTTCTGCTTTAGTACATTCTTCTACATTAG

AATTTATTTTCAAAATGTAAAATCTTATAATGCTGGTATATTAACAGCTTTATCTAATCGAATCGGAGATGTTGCCTTAT

CTTTACATTCAATATCTATTGTAATAACTTTTTTATTTGATTGAATAAGTTTAATATTTATATCTTTTGTTTTATTAATT

AATTTTTATTAGATTTACTTTATTTATTTTGAGATTAAAGTTTTTATTAATAGATTTAGTATATTTTATTGAATGAGAAATT

GAGGGTATCAACCTGAACGTTTACAGGCGGGGATTTATTTATTATTTTATACTTTACTGGCTTCGTTGCCGTTATTAATT

AATTATGAAAGTTACTTTAGTATATTTTTTTTAACTTTTTGTGTTTGTGAAGGGGTTCTAGGGCTATCTATTTTAGTTTC

AATTATTATATTTATTTTTGGAAGTATTGTTTTTATTTCTAGTCGTAAGCATTTACTTTGTACTTTATTGAGATTAGAAT

AATTTTTATAGGAGGAATATTAGTTTTATTCATTTATGTTACTTCTCTTTCATCTAATGAAATATTTTCATTATCTATAAAACTATTTTTTTTATCTTTAAGTATAATT

AATTTTAACAGGATTATTCTTAGCAATACACTATACTGCTGATATTGAAACAGCTTTTAATAGAGTAAATCACATTTATCGTGATGTTAATAATGGTTGATTCCTACGAATT

CTGTTGATAATGCTACTTTAACTCGATTCTTTACATTTCATTTTATTTTACCTTTCATTGTATTAGCTTTAACTATAATT

CTTTTGATTTTGCTGAAGGGGAGTCTGAGTTAGTTTCAGGATTTAATGTAGAATATAGAAGAGGGGGATTTGCTTTAATT

AATTTTTTTATATATCAGAAATACATATGATTTATTGTATTTTGTTTTCCTTTAGGTCTCGTTTGATTTGCATCTTGTTT

AATTTAAGCTATAAACTTTTTGTAATAAACTAGGATTAGATACCCTATTATTAAAAATAAATATTAAAATGCTAAAGTAG

>Gv10-B-07

AATTTTATCTACTTTATTATTAAAAAGAGGGGCAGCTCCTTTTCATTTTTGATTCCCAGGAGTTATAGAAGGATTAAATT

AATTAATCATATTGGTTGAATATTAATAGCTATAATAAATAACGAACTTTTATGACTAACTTATTTTTTATTATATTCAATT

CAACAAATCATAAAGATATTGGAACTTTATATTTCATTTTTGGAGTATGATCCGGAATAGTCGGAACTTCTCTAAGAATT

AATTGGATTATTGGGATTTATTGTTTGAGCTCATCATATATTTACAGTAGGTATAGACGTAGATACTCGAGCTTATTTTACTTCAGCAACTATAATT

CATGATACTTATTATGTAGTTGCCCATTTTCATTATGTTTTATCTATAGGAGCTGTATTTGCTATTATAGCAGGATTTATTCATTGATACCCTTTATTAACAGGAATAGTTATAAACCCTTCATG

CTGGAATACCTCGACGATACTCGGATTTTCCCGATAGTTACTTAACTTGAAATATTATTTCTTCTTTAGGAAGAACAATT

AATTTGTCACACACAACATATATTTGCTCATTTAGTTCCTCAAGGAACTCCCCCTGTTTTAATACCTTTTATAGTATGCATTGAAACTATCAGTAATGTAATCCGACCAGGAACTTTAGCAGTACGATTAACTGCTAATATAATT

GACGAGATATTTCTCGAGAAGGAACTTTTCAAGGACTTCACACTATTCCCGTAACATTAGGATTACGATGAGGAATAATTTTATTTATTATTTCTGAAGTTTTTTTCTTTATTTCCTTCTTTTGAGCTTTTTTCCATAGTAGCTTATCCCCAACAATCGAATT

AATTGTCATTTTTCTAGAAGTCATCATTTTGGATTTGAAGCAGCTGCTTGATATTGACATTTTGTTGATGTAGTTTGACTATTCTTATATATTTCAATT

AATTTTAGGAATATATTCTTATAAAATGTTTGATCAAGGATGAAGTGAATATTTTGGAGGTCAGATATTATATAATCAAT

ATTATTCAATAACAGGGGATTTTAATAGAACTACATTGAATATACTAAATGATAAGGGGTGAACTATATCTTTTAGAATT

AATTCCTTTTTCTTCTTGATTACCTGCAGCTATGGCAGCTCCGACTCCTGTTTCTGCTTTAGTACATTCTTCTACATTAG

AATTTATTTTCAAAATGTAAAATCTTATAATGCTGGTATATTAACAGCTTTATCTAATCGAATCGGAGATGTTGCCTTAT

CTTTACATTCAATATCTATTGTAATAACTTTTTTATTTGATTGAATAAGTTTAATATTTATATCTTTTGTTTTATTAATT

AATTTTTATTAGATTTACTTTATTTATTTTGAGATTAAAGTTTTTATTAATAGATTTAGTATATTTTATTGAATGAGAAATT

GAGGGTATCAACCTGAACGTTTACAGGCGGGGATTTATTTATTATTTTATACTTTACTGGCTTCGTTGCCGTTATTAATT

AATTATGAAAGTTACTTTAGTATATTTTTTTTAACTTTTTGTGTTTGTGAAGGGGTTCTAGGGCTATCTATTTTAGTTTC

AATTATTATATTTATTTTTGGAAGTATTGTTTTTATTTCTAGTCGTAAGCATTTACTTTGTACTTTATTGAGATTAGAAT

AATTTTTATAGGAGGAATATTAGTTTTATTCATTTATGTTACTTCTCTTTCATCTAATGAAATATTTTCATTATCTATAAAACTATTTTTTTTATCTTTAAGTATAATT

AATTTTAACAGGATTATTCTTAGCAATACACTATACTGCTGATATTGAAACAGCTTTTAATAGAGTAAATCACATTTATCGTGATGTTAATAATGGTTGATTCCTACGAATT

CTGTTGATAATGCTACTTTAACTCGATTCTTTACATTTCATTTTATTTTACCTTTCATTGTATTAGCTTTAACTATAATT

CTTTTGATTTTGCTGAAGGGGAGTCTGAGTTAGTTTCAGGATTTAATGTAGAATATAGAAGAGGGGGATTTGCTTTAATT

AATTTTTTTATATATCAGAAATACATATGATTTATTGTATTTTGTTTTCCTTTAGGTCTCGTTTGATTTGCATCTTGTTT

AATTTAAGCTATAAACTTTTTGTAATAAACTAGGATTAGATACCCTATTATTAAAAATAAATATTAAAATGCTAAAGTAG

>Gv10-B-08

AATTTTATCTACTTTATTATTAAAAAGAGGGGCAGCTCCTTTTCATTTTTGATTCCCAGGAGTTATAGAAGGATTAAATT

AATTAATCATATTGGTTGAATATTAATAGCTATAATAAATAACGAACTTTTATGACTAACTTATTTTTTATTATATTCAATT

CAACAAATCATAAAGATATTGGAACTTTATATTTCATTTTTGGAGTATGATCCGGAATAGTCGGAACTTCTCTAAGAATT

AATTGGATTATTGGGATTTATTGTTTGAGCTCATCATATATTTACAGTAGGTATAGACGTAGATACTCGAGCTTATTTTACTTCAGCAACTATAATT

CATGATACTTATTATGTAGTTGCCCATTTTCATTATGTTTTATCTATAGGAGCTGTATTTGCTATTATAGCAGGATTTATTCATTGATACCCTTTATTAACAGGAATAGTTATAAACCCTTCATG

CTGGAATACCTCGACGATACTCAGATTTTCCCGATAGTTACTTAACTTGAAATATTATTTCTTCTTTAGGAAGAACAATT

AATTTGTCACACACAACATATATTTGCTCATTTAGTTCCTCAAGGAACTCCCCCTGTTTTAATACCTTTTATAGTATGCATTGAAACTATCAGTAATGTAATCCGACCAGGAACTTTAGCAGTACGATTAACTGCTAATATAATT

GACGAGATATTTCTCGAGAAGGAACTTTTCAAGGACTTCACACTATTCCCGTAACATTAGGATTACGATGAGGAATAATTTTATTTATTATTTCTGAAGTTTTTTTCTTTATTTCCTTCTTTTGAGCTTTTTTCCATAGTAGCTTATCCCCAACAATCGAATT

AATTGTCATTTTTCTAGAAGTCATCATTTTGGATTTGAAGCAGCTGCTTGATATTGACATTTTGTTGATGTAGTTTGACTATTCTTATATATTTCAATT

AATTTTAGGAATATATTCTTATAAAATGTTTGATCAAGGATGAAGTGAATATTTTGGAGGTCAGATATTATATAATCAAT

ATTATTCAATAACAGGGGATTTTAATAGAACTACATTGAATATACTAAATGATAAGGGGTGAACTATATCTTTTAGAATT

AATTCCTTTTTCTTCTTGATTACCTGCAGCTATGGCAGCTCCGACTCCTGTTTCTGCTTTAGTACATTCTTCTACATTAG

AATTTATTTTCAAAATGTAAAATCTTATAATGCTGGTATATTAACAGCTTTATCTAATCGAATCGGAGATGTTGCCTTAT

CTTTACATTCAATATCTATTGTAATAACTTTTTTATTTGATTGAATAAGTTTAATATTTATATCTTTTGTTTTATTAATT

AATTTTTATTAGATTTACTTTATTTATTTTGAGATTAAAGTTTTTATTAATAGATTTAGTATATTTTATTGAATGAGAAATT

GAGGGTATCAACCTGAACGTTTACAGGCGGGGATTTATTTATTATTTTATACTTTACTGGCTTCGTTGCCGTTATTAATT

AATTATGAAAGTTACTTTAGTATATTTTTTTTAACTTTTTGTGTTTGTGAAGGGGTTCTAGGGCTATCTATTTTAGTTTC

AATTATTATATTTATTTTTGGAAGTATTGTTTTTATTTCTAGTCGTAAGCATTTACTTTGTACTTTATTGAGATTAGAAT

AATTTTTATAGGAGGAATATTAGTTTTATTCATTTATGTTACTTCTCTTTCATCTAATGAAATATTTTCATTATCTATAAAACTATTTTTTTTATCTTTAAGTATAATT

AATTTTAACAGGATTATTCTTAGCAATACACTATACTGCTGATATTGAAACAGCTTTTAATAGAGTAAATCACATTTATCGTGATGTTAATAATGGTTGATTCCTACGAATT

CTGTTGATAATGCTACTTTAACTCGATTCTTTACATTTCATTTTATTTTACCTTTCATTGTATTAGCTTTAACTATAATT

CTTTTGATTTTGCTGAAGGGGAGTCTGAGTTAGTTTCAGGATTTAATGTAGAATATAGAAGAGGGGGATTTGCTTTAATT

AATTTTTTTATATATCAGAAATACATATGATTTATTGTATTTTGTTTTCCTTTAGGTCTCGTTTGATTTGCATCTTGTTT

AATTTAAGCTATAAACTTTTTGTAATAAACTAGGATTAGATACCCTATTATTAAAAATAAATATTAAAATGCTAAAGTAG

>Gv10-B-09

AATTTTATCTACTTTATTATTAAAAAGAGGGGCAGCTCCTTTTCATTTTTGATTCCCAGGAGTTATAGAAGGATTAAATT

AATTAATCATATTGGTTGAATATTAATAGCTATAATAAATAACGAACTTTTATGACTAACTTATTTTTTATTATATTCAATT

CAACAAATCATAAAGATATTGGAACTTTATATTTCATTTTTGGAGTATGATCCGGAATAGTCGGAACTTCTCTAAGAATT

AATTGGATTATTGGGATTTATTGTTTGAGCTCATCATATATTTACAGTAGGTATAGACGTAGATACTCGAGCTTATTTTACTTCAGCAACTATAATT

CATGATACTTATTATGTAGTTGCCCATTTTCATTATGTTTTATCTATAGGAGCTGTATTTGCTATTATAGCAGGATTTATTCATTGATACCCTTTATTAACAGGAATAGTTATAAACCCTTCATG

CTGGAATACCTCGACGATACTCAGATTTTCCCGATAGTTACTTAACTTGAAATATTATTTCTTCTTTAGGAAGAACAATT

AATTTGTCACACACAACATATATTTGCTCATTTAGTTCCTCAAGGAACTCCCCCTGTTTTAATACCTTTTATAGTATGCATTGAAACTATCAGTAATGTAATCCGACCAGGAACTTTAGCAGTACGATTAACTGCTAATATAATT

GACGAGATATTTCTCGAGAAGGAACTTTTCAAGGACTTCACACTATTCCCGTAACATTAGGATTACGATGAGGAATAATTTTATTTATTATTTCTGAAGTTTTTTTCTTTATTTCCTTCTTTTGAGCTTTTTTCCATAGTAGCTTATCCCCAACAATCGAATT

AATTGTCATTTTTCTAGAAGTCATCATTTTGGATTTGAAGCAGCTGCTTGATATTGACATTTTGTTGATGTAGTTTGACTATTCTTATATATTTCAATT

AATTTTAGGAATATATTCTTATAAAATGTTTGATCAAGGATGAAGTGAATATTTTGGAGGTCAGATATTATATAATCAAT

ATTATTCAATAACAGGGGATTTTAATAGAACTACATTGAATATACTAAATGATAAGGGGTGAACTATATCTTTTAGAATT

AATTCCTTTTTCTTCTTGATTACCTGCAGCTATGGCAGCTCCGACTCCTGTTTCTGCTTTAGTACATTCTTCTACATTAG

AATTTATTTTCAAAATGTAAAATCTTATAATGCTGGTATATTAACAGCTTTATCTAATCGAATCGGAGATGTTGCCTTAT

CTTTACATTCAATATCTATTGTAATAACTTTTTTATTTGATTGAATAAGTTTAATATTTATATCTTTTGTTTTATTAATT

AATTTTTATTAGATTTACTTTATTTATTTTGAGATTAAAGTTTTTATTAATAGATTTAGTATATTTTATTGAATGAGAAATT

GAGGGTATCAACCTGAACGTTTACAGGCGGGGATTTATTTATTATTTTATACTTTACTGGCTTCGTTGCCGTTATTAATT

AATTATGAAAGTTACTTTAGTATATTTTTTTTAACTTTTTGTGTTTGTGAAGGGGTTCTAGGGCTATCTATTTTAGTTTC

AATTATTATATTTATTTTTGGAAGTATTGTTTTTATTTCTAGTCGTAAGCATTTACTTTGTACTTTATTGAGATTAGAAT

AATTTTTATAGGAGGAATATTAGTTTTATTCATTTATGTTACTTCTCTTTCATCTAATGAAATATTTTCATTATCTATAAAACTATTTTTTTTATCTTTAAGTATAATT

AATTTTAACAGGATTATTCTTAGCAATACACTATACTGCTGATATTGAAACAGCTTTTAATAGAGTAAATCACATTTATCGTGATGTTAATAATGGTTGATTCCTACGAATT

CTGTTGATAATGCTACTTTAACTCGATTCTTTACATTTCATTTTATTTTACCTTTCATTGTATTAGCTTTAACTATAATT

CTTTTGATTTTGCTGAAGGGGAGTCTGAGTTAGTTTCAGGATTTAATGTAGAATATAGAAGAGGGGGATTTGCTTTAATT

AATTTTTTTATATATCAGAAATACATATGATTTATTGTATTTTGTTTTCCTTTAGGTCTCGTTTGATTTGCATCTTGTTT

AATTTAAGCTATAAACTTTTTGTAATAAACTAGGATTAGATACCCTATTATTAAAAATAAATATTAAAATGCTAAAGTAG

>Gv10-B-10

AATTTTATCTACTTTATTATTAAAAAGAGGAGCAGCTCCTTTTCATTTTTGATTCCCAGGAGTTATAGAAGGATTAAATT

AATTAATCATATTGGTTGAATATTAATAGCTATAATAAATAACGAACTTTTATGACTAACTTATTTTTTATTATATTCAATT

CAACAAATCATAAAGATATTGGAACTTTATATTTCATTTTTGGAGTATGATCCGGAATAGTCGGAACTTCTCTAAGAATT

AATTGGATTATTGGGATTTATTGTTTGAGCTCATCATATATTTACAGTAGGTATAGACGTAGATACTCGAGCTTATTTTACTTCAGCAACTATAATT

CATGATACTTATTATGTAGTTGCCCATTTTCATTATGTTTTATCTATAGGAGCTGTATTTGCTATTATAGCAGGATTTATTCATTGATACCCTTTATTAACAGGAATAGTTATAAACCCTTCATG

CTGGAATACCTCGACGATACTCAGATTTTCCCGATAGTTACTTAACTTGAAATATTATTTCTTCTTTAGGAAGAACAATT

AATTTGTCACACACAACATATATTTGCTCATTTAGTTCCTCAAGGAACTCCCCCTGTTTTAATACCTTTTATAGTATGCATTGAAACTATTAGTAATGTAATCCGACCAGGAACTTTAGCAGTACGATTAACTGCTAATATAATT

GACGAGATATTTCTCGAGAAGGAACTTTTCAAGGACTTCACACTATTCCCGTAACATTAGGATTACGATGAGGAATAATTTTATTTATTATTTCTGAAGTTTTTTTCTTTATTTCCTTCTTTTGAGCTTTTTTCCATAGTAGCTTATCCCCAACAATCGAATT

AATTGTCATTTTTCTAGAAGTCATCATTTTGGATTTGAAGCAGCTGCTTGATATTGACATTTTGTTGATGTAGTTTGACTATTCTTATATATTTCAATT

AATTTTAGGAATATATTCTTATAAAATGTTTGATCAAGGATGAAGTGAATATTTTGGAGGTCAGATATTATATAATCAAT

ATTATTCAATAACAGGGGATTTTAATAGAACTACATTGAATATACTAAATGATAAGGGGTGAACTATATCTTTTAGAATT

AATTCCTTTTTCTTCTTGATTACCTGCAGCTATGGCAGCTCCGACTCCTGTTTCTGCTTTAGTACATTCTTCTACATTAG

AATTTATTTTCAAAATGTAAAATCTTATAATGCTGGTATATTAACAGCTTTATCTAATCGAATCGGAGATGTTGCCTTAT

CTTTACATTCAATATCTATTGTAATAACTTTTTTATTTGATTGAATAAGTTTAATATTTATATCTTTTGTTTTATTAATT

AATTTTTATTAGATTTACTTTATTTATTTTGAGATTAAAGTTTTTATTAATAGATTTAGTATATTTTATTGAATGAGAAATT

GAGGGTATCAACCTGAACGTTTACAGGCGGGGATTTATTTATTATTTTATACTTTACTGGCTTCGTTGCCGTTATTAATT

AATTATGAAAGTTACTTTAGTATATTTTTTTTAACTTTTTGTGTTTGTGAAGGGGTTCTAGGGCTATCTATTTTAGTTTC

AATTATTATATTTATTTTTGGAAGTATTGTTTTTATTTCTAGTCGTAAGCATTTACTTTGTACTTTATTGAGATTAGAGT

AATTTTTATAGGAGGAATATTAGTTTTATTCATTTATGTTACTTCTCTTTCATCTAATGAAATATTTTCATTATCTATAAAACTATTTTTTTTATCTTTAAGTATAATT

AATTTTAACAGGATTATTCTTAGCAATACACTATACTGCTGATATTGAAACAGCTTTTAATAGAGTAAATCACATTTATCGTGATGTTAATAATGGTTGATTCCTACGAATT

CTGTTGATAATGCTACTTTAACTCGATTCTTTACATTTCATTTTATTTTACCTTTCATTGTATTAGCTTTAACTATAATT

CTTTTGATTTTGCTGAAGGGGAGTCTGAGTTAGTTTCAGGATTTAATGTAGAATATAGAAGAGGGGGATTTGCTTTAATT

AATTTTTTTATATATCAGAAATACATATGATTTATTGTATTTTGTTTTCCTTTAGGTCTCGTTTGATTTGCATCTTGTTT

AATTTAAGCTATAAACTTTTTGTAATAAACTAGGATTAGATACCCTATTATTAAAAATAAATATTAAAATGCTAAAGTAG

>Gv10-C-01

NATTTTATCTACTTTATTATTAAAAAGAGGGGCAGCTCCTTTTCATTTTTGATTCCCAGGAGTTATAGAAGGATTAAATT

AATTAATCATATTGGTTGAATATTAATAGCTATAATAAATAACGAACTTTTATGACTAACTTATTTTTTATTATATTCAATT

CAACAAATCATAAAGATATTGGAACTTTATATTTCATTTTTGGAGTATGATCCGGAATAGTCGGAACTTCTCTAAGAATT

AATTGGATTATTGGGATTTATTGTTTGAGCTCATCATATATTTACAGTAGGTATAGACGTAGATACTCGAGCTTATTTTACTTCAGCAACTATAATT

CATGATACTTATTATGTAGTTGCCCATTTTCATTATGTTTTATCTATAGGAGCTGTATTTGCTATTATAGCAGGATTTATTCATTGATACCCTTTATTAACAGGAATAGTTATAAACCCTTCATG

CTGGAATACCTCGACGATACTCAGATTTTCCCGATAGTTACTTAACTTGAAATATTATTTCTTCTTTAGGAAGAACAATT

AATTTGTCACACACAACATATATTTGCTCATTTAGTTCCTCAAGGAACTCCCCCTGTTTTAATACCTTTTATAGTATGCATTGAAACTATCAGTAATGTAATCCGACCAGGAACTTTAGCAGTACGATTAACTGCTAATATAATT

GACGAGATATTTCTCGAGAAGGAACTTTTCAAGGACTTCACACTATTCCCGTAACATTAGGATTACGATGAGGAATAATTTTATTTATTATTTCTGAAGTTTTTTTCTTTATTTCCTTCTTTTGAGCTTTTTTCCATAGTAGCTTATCCCCAACAATCGAATT

AATTGTCATTTTTCTAGAAGTCATCATTTTGGATTTGAAGCAGCTGCTTGATATTGACATTTTGTTGATGTAGTTTGACTATTCTTATATATTTCAATT

AATTTTAGGAATATATTCTTATAAAATGTTTGATCAAGGATGAACTGAATATTTTGGAGGTCAGATATTATATAATCAAT

ATTATTCAATAACAGGGGATTTTAATAGAACTACATTGAATATACTAAATGATAAGGGGTGAACTATATCTTTTAGAATT

AATTCCTTTTTCTTCTTGATTACCTGCAGCTATGGCAGCTCCGACTCCTGTTTCTGCTTTAGTACATTCTTCTACATTAG

AATTTATTTTCAAAATGTAAAATCTTATAATGCTGGTATATTAACAGCTTTATCTAATCGAATCGGAGATGTTGCCTTAT

CTTTACATTCAATATCTATTGTAATAACTTTTTTATTTGATTGAATAAGTTTAATATTTATATCTTTTGTTTTATTAATT

AATTTTTATTAGATTTACTTTATTTATTTTGAGATTAAAGTTTTTATTAATAGATTTAGTATATTTTATTGAATGAGAAATT

GAGGGTATCAACCTGAACGTTTACAGGCGGGGATTTATTTATTATTTTATACTTTACTGGCTTCGTTGCCGTTATTAATT

AATTATGAAAGTTACTTTAGTATATTTTTTTTAACTTTTTGTGTTTGTGAAGGGGTTCTAGGGCTATCTATTTTAGTTTC

AATTATTATATTTATTTTTGGAAGTATTGTTTTTATTTCTAGTCGTAAGCATTTACTTTGTACTTTATTGAGATTAGAAT

AATTTTTATAGGAGGAATATTAGTTTTATTCATTTATGTTACTTCTCTTTCATCTAATGAAATATTTTCATTATCTATAAAACTATTTTTTTTATCTTTAAGTATAATT

AATTTTAACAGGATTATTCTTAGCAATACACTATACTGCTGATATTGAAACAGCTTTTAATAGAGTAAATCACATTTATCGTGATGTTAATAATGGTTGATTCCTACGAATT

CTGTTGATAATGCTACTTTAACTCGATTCTTTACATTTCATTTTATTTTACCTTTCATTGTATTAGCTTTAACTATAATT

CTTTTGATTTTGCTGAAGGGGAGTCTGAGTTAGTTTCAGGATTTAATGTAGAATATAGAAGAGGGGGATTTGCTTTAATT

AATTTTTTTATATATCAGAAATACATATGATTTATTGTATTTTGTTTTCCTTTAGGTCTCGTTTGATTTGCATCTTGTTT

AATTTAAGCTATAAACTTTTTGTAATAAACTAGGATTAGATACCCTATTATTAAAAATAAATATTAAAATGCTAAAGTAG

>Gv10-C-02

AATTTTATCTACTTTATTATTAAAAAGAGGAGCAGCTCCTTTTCATTTTTGATTCCCAGGAGTTATAGAAGGATTAAATT

AATTAATCATATTGGTTGAATATTAATAGCTATAATAAATAACGAACTTTTATGACTAACTTATTTTTTATTATATTCAATN

CAACAAATCATAAAGATATTGGAACTTTATATTTCATTTTTGGAGTATGATCCGGAATAGTCGGAACTTCTCTAAGAATT

AATTGGATTATTGGGATTTATTGTTTGAGCTCATCATATATTTACAGTAGGTATAGACGTAGATACTCGAGCTTATTTTACTTCAGCAACTATAATT

CATGATACTTATTATGTAGTTGCCCATTTTCATTATGTTTTATCTATAGGAGCTGTATTTGCTATTATAGCAGGATTTATTCATTGATACCCTTTATTAACAGGAATAGTTATAAACCCTTCATG

CTGGAATACCTCGACGATACTCAGATTTTCCCGATAGTTACTTAACTTGAAATATTATTTCTTCTTTAGGAAGAACAATN

AATTTGTCACACACAACATATATTTGCTCATTTAGTTCCTCAAGGAACTCCCCCTGTTTTAATACCTTTTATAGTATGCATTGAAACTATTAGTAATGTAATCCGACCAGGAACTTTAGCAGTACGATTAACTGCTAATATAATT

GACGAGATATTTCTCGAGAAGGAACTTTTCAAGGACTTCACACTATTCCCGTAACATTAGGATTACGATGAGGAATNNNNNNNNNNNNNNNNNNNNNNNNNNNNNNNNNNNNNNNNNNNNNNNNNNNNNNNNNNNNNNNNNNNNNNNNNNNNNNNNTCGAATT

AATTGTCATTTTTCTAGAAGTCATCATTTTGGATTTGAAGCAGCTGCTTGATATTGACATTTTGTTGATGTAGTTTGACTATTCTTATATATTTCAATT

AATTTTAGGAATATATTCTTATAAAATGTTTGATCAAGGATGAAGTGAATATTTTGGAGGTCAGATATTATATAATCAAT

ATTATTCAATAACAGGGGATTTTAATAGAACTACATTGAATATACTAAATGATAAGGGGTGAACTATATCTTTTAGAATT

AATTCCTTTTTCTTCTTGATTACCTGCAGCTATGGCAGCTCCGACTCCTGTTTCTGCTTTAGTACATTCTTCTACATTAG

AATTTATTTTCAAAATGTAAAATCTTATAATGCTGGTATATTAACAGCTTTATCTAATCGAATCGGAGATGTTGCCTTAT

CTTTACATTCAATATCTATTGTAATAACTTTTTTATTTGATTGAATAAGTTTAATATTTATATCTTTTGTTTTATTAATT

AATTTTTATTAGATTTACTTTATTTATTTTGAGATTAAAGTTTTTATTAATAGATTTAGTATATTTTATTGAATGAGAAATT

GAGGGTATCAACCTGAACGTTTACAGGCGGGGATTTATTTATTATTTTATACTTTACTGGCTTCGTTGCCGTTATTAATT

NATTATGAAAGTTACTTTAGTATATTTTTTTTAACTTTTTGTGTTTGTGAAGGGGTTCTAGGGCTATCTATTTTAGTTTC

AATTATTATATTTATTTTTGGAAGTATTGTTTTTATTTCTAGTCGTAAGCATTTACTTTGTACTTTATTGAGATTAGAGT

AATTTTTATAGGAGGAATATTAGTTTTATTCATTTATGTTACTTCTCTTTCATCTAATGAAATATTTTCATTATCTATAAAACTATTTTTTTTATCTTTAAGTATAATT

AATTTTAACAGGATTATTCTTAGCAATACACTATACTGCTGATATTGAAACAGCTTTTAATAGAGTAAATCACATTTATCGTGATGTTAATAATGGTTGATTCCTACGAATT

CTGTTGATAATGCTACTTTAACTCGATTCTTTACATTTCATTTTATTTTACCTTTCATTGTATTAGCTTTAACTATAATT

CTTTTGATTTTGCTGAAGGGGAGTCTGAGTTAGTTTCAGGATTTAATGTAGAATATAGAAGAGGGGGATTTGCTTTAATT

AATTTTTTTATATATCAGAAATACATATGATTTATTGTATTTTGTTTTCCTTTAGGTCTCGTTTGATTTGCATCTTGTTT

NATTTAAGCTATAAACTTTTTGTAATAAACTAGGATTAGATACCCTATTATTAAAAATAAATATTAAAATGCTAAAGTAG

>Gv10-C-03

AATTTTATCTACTTTATTATTAAAAAGAGGGGCAGCTCCTTTTCATTTTTGATTCCCAGGAGTTATAGAAGGATTAAATT

AATTAATCATATTGGTTGAATATTAATAGCTATAATAAATAACGAACTTTTATGACTAACTTATTTTTTATTATATTCAATT

CAACAAATCATAAAGATATTGGAACTTTATATTTCATTTTTGGAGTATGATCCGGAATAGTCGGAACTTCTCTAAGAATT

AATTGGATTATTGGGATTTATTGTTTGAGCTCATCATATATTTACAGTAGGTATAGACGTAGATACTCGAGCTTATTTTACTTCAGCAACTATAATT

CATGATACTTATTATGTAGTTGCCCATTTTCATTATGTTTTATCTATAGGAGCTGTATTTGCTATTATAGCAGGATTTATTCATTGATACCCTTTATTAACAGGAATAGTTATAAACCCTTCATG

CTGGAATACCTCGACGATACTCAGATTTTCCCGATAGTTACTTAACTTGAAATATTATTTCTTCTTTAGGAAGAACAATT

AATTTGTCACACACAACATATATTTGCTCATTTAGTTCCTCAAGGAACTCCCCCTGTTTTAATACCTTTTATAGTATGCATTGAAACTATCAGTAATGTAATCCGACCAGGAACTTTAGCAGTACGATTAACTGCTAATATAATT

GACGAGATATTTCTCGAGAAGGAACTTTTCAAGGACTTCACACTATTCCCGTAACATTAGGATTACGATGAGGAATAATTTTATTTATTATTTCTGAAGTTTTTTTCTTTATTTCCTTCTTTTGAGCTTTTTTCCATAGTAGCTTATCCCCAACAATCGAATT

AATTGTCATTTTTCTAGAAGTCATCATTTTGGATTTGAAGCAGCTGCTTGATATTGACATTTTGTTGATGTAGTTTGACTATTCTTATATATTTCAATT

AATTTTAGGAATATATTCTTATAAAATGTTTGATCAAGGATGAAGTGAATATTTTGGAGGTCAGATATTATATAATCAAT

ATTATTCAATAACAGGGGATTTTAATAGAACTACATTGAATATACTAAATGATAAGGGGTGAACTATATCTTTTAGAATT

AATTCCTTTTTCTTCTTGATTACCTGCAGCTATGGCAGCTCCGACTCCTGTTTCTGCTTTAGTACATTCTTCTACATTAG

AATTTATTTTCAAAATGTAAAATCTTATAATGCTGGTATATTAACAGCTTTATCTAATCGAATCGGAGATGTTGCCTTAT

NNNNNNNNNNNNNNNNNNNNNNNNNNNNNNNNNNNNNNNNNNNNNNNNNNNNNNNNNNNNNNNNNNNNNNNNNNNNNNNN

AATTTTTATTAGATTTACTTTATTTATTTTGAGATTAAAGTTTTTATTAATAGATTTAGTATATTTTATTGAATGAGAAATT

GAGGGTATCAACCTGAACGTTTACAGGCGGGGATTTATTTATTATTTTATACTTTACTGGCTTCGTTGCCGTTATTAATT

AATTATGAAAGTTACTTTAGTATATTTTTTTTAACTTTTTGTGTTTGTGAAGGGGTTCTAGGGCTATCTATTTTAGTTTC

AATTATTATATTTATTTTTGGAAGTATTGTTTTTATTTCTAGTCGTAAGCATTTACTTTGTACTTTATTGAGATTAGAAT

AATTTTTATAGGAGGAATATTAGTTTTATTCATTTATGTTACTTCTCTTTCATCTAATGAAATATTTTCATTATCTATAAAACTATTTTTTTTATCTTTAAGTATAATT

AATTTTAACAGGATTATTCTTAGCAATACACTATACTGCTGATATTGAAACAGCTTTTAATAGAGTAAATCACATTTATCGTGATGTTAATAATGGTTGATTCCTACGAATT

CTGTTGATAATGCTACTTTAACTCGATTCTTTACATTTCATTTTATTTTACCTTTCATTGTATTAGCTTTAACTATAATT

CTTTTGATTTTGCTGAAGGGGAGTCTGAGTTAGTTTCAGGATTTAATGTAGAATATAGAAGAGGGGGATTTGCTTTAATT

AATTTTTTTATATATCAGAAATACATATGATTTATTGTATTTTGTTTTCCTTTAGGTCTCGTTTGATTTGCATCTTGTTT

AATTTAAGCTATAAACTTTTTGTAATAAACTAGGATTAGATACCCTATTATTAAAAATAAATATTAAAATGCTAAAGTAG

>Gv10-C-04

AATTTTATCTACTTTATTATTAAAAAGAGGGGCAGCTCCTTTTCATTTTTGATTCCCAGGAGTTATAGAAGGATTAAATT

AATTAATCATATTGGTTGAATATTAATAGCTATAATAAATAACGAACTTTTATGACTAACTTATTTTTTATTATATTCAATT

CAACAAATCATAAAGATATTGGAACTTTATATTTCATTTTTGGAGTATGATCCGGAATAGTCGGAACTTCTCTAAGAATT

AATTGGATTATTGGGATTTATTGTTTGAGCTCATCATATATTTACAGTAGGTATAGACGTAGATACTCGAGCTTATTTTACTTCAGCAACTATAATT

CATGATACTTATTATGTAGTTGCCCATTTTCATTATGTTTTATCTATAGGAGCTGTATTTGCTATTATAGCAGGATTTATTCATTGATACCCTTTATTAACAGGAATAGTTATAAACCCTTCATG

CTGGAATACCTCGACGATACTCAGATTTTCCTGATAGTTACTTAACTTGAAATATTATTTCTTCTTTAGGAAGAACAATT

AATTTGTCACACACAACATATATTTGCTCATTTAGTTCCTCAAGGAACTCCCCCTGTTTTAATACCTTTTATAGTATGCATTGAAACTATCAGTAATGTAATCCGACCAGGAACTTTAGCAGTACGATTAACTGCTAATATAATT

GACGAGATATTTCTCGAGAAGGAACTTTTCAAGGACTTCACACTATTCCCGTAACATTAGGATTACGATGAGGAATAATTTTATTTATTATTTCTGAAGTTTTTTTCTTTATTTCCTTCTTTTGAGCTTTTTTCCATAGTAGCTTATCCCCAACAATCGAATT

AATTGTCATTTTTCTAGAAGTCATCATTTTGGATTTGAAGCAGCTGCTTGATATTGACATTTTGTTGATGTAGTTTGACTATTCTTATATATTTCAATT

AATTTTAGGAATATATTCTTATAAAATGTTTGATCAAGGATGAAGTGAATATTTTGGAGGTCAGATATTATATAATCAAT

ATTATTCAATAACAGGGGATTTTAATAGAACTACATTGAATATACTAAATGATAAGGGGTGAACTATATCTTTTAGAATT

AATTCCTTTTTCTTCTTGATTACCTGCAGCTATGGCAGCTCCGACTCCTGTTTCTGCTTTAGTACATTCTTCTACATTAG

AATTTATTTTCAAAATGTAAAATCTTATAATGCTGGTATATTAACAGCTTTATCTAATCGAATCGGAGATGTTGCCTTAT

CTTTACATTCAATATCTATTGTAATAACTTTTTTATTTGATTGAATAAGTTTAATATTTATATCTTTTGTTTTATTAATT

AATTTTTATTAGATTTACTTTATTTATTTTGAGATTAAAGTTTTTATTAATAGATTTAGTATATTTTATTGAATGAGAAATT

GAGGGTATCAACCTGAACGTTTACAGGCGGGGATTTATTTATTATTTTATACTTTACTGGCTTCGTTGCCGTTATTAATT

AATTATGAAAGTTACTTTAGTATATTTTTTTTAACTTTTTGTGTTTGTGAAGGGGTTCTAGGGCTATCTATTTTAGTTTC

AATTATTATATTTATTTTTGGAAGTATTGTTTTTATTTCTAGTCGTAAGCATTTACTTTGTACTTTATTGAGATTAGAAT

AATTTTTATAGGAGGAATATTAGTTTTATTCATTTATGTTACTTCTCTTTCATCTAATGAAATATTTTCATTATCTATAAAACTATTTTTTTTATCTTTAAGTATAATT

AATTTTAACAGGATTATTCTTAGCAATACACTATACTGCTGATATTGAAACAGCTTTTAATAGAGTAAATCACATTTATCGTGATGTTAATAATGGTTGATTCCTACGAATT

CTGTTGATAATGCTACTTTAACTCGATTCTTTACATTTCATTTTATTTTACCTTTCATTGTATTAGCTTTAACTATAATT

CTTTTGATTTTGCTGAAGGGGAGTCTGAGTTAGTTTCAGGATTTAATGTAGAATATAGAAGAGGGGGATTTGCTTTAATT

AATTTTTTTATATATCAGAAATACATATGATTTATTGTATTTTGTTTTCCTTTAGGTCTCGTTTGATTTGCATCTTGTTT

AATTTAAGCTATAAACTTTTTGTAATAAACTAGGATTAGATACCCTATTATTAAAAATAAATATTAAAATGCTAAAGTAG

>Gv10-C-05

AATTTTATCTACTTTATTATTAAAAAGAGGGGCAGCTCCTTTTCATTTTTGATTCCCAGGAGTTATAGAAGGATTAAATT

AATTAATCATATTGGTTGAATATTAATAGCTATAATAAATAACGAACTTTTATGACTAACTTATTTTTTATTATATTCAATT

CAACAAATCATAAAGATATTGGAACTTTATATTTCATTTTTGGAGTATGATCCGGAATAGTCGGAACTTCTCTAAGAATT

AATTGGATTATTGGGATTTATTGTTTGAGCTCATCATATATTTACAGTAGGTATAGACGTAGATACTCGAGCTTATTTTACTTCAGCAACTATAATT

CATGATACTTATTATGTAGTTGCCCATTTTCATTATGTTTTATCTATAGGAGCTGTATTTGCTATTATAGCAGGATTTATTCATTGATACCCTTTATTAACAGGAATAGTTATAAACCCTTCATG

CTGGAATACCTCGACGATACTCAGATTTTCCCGATAGTTACTTAACTTGAAATATTATTTCTTCTTTAGGAAGAACAATT

AATTTGTCACACACAACATATATTTGCTCATTTAGTTCCTCAAGGAACTCCCCCTGTTTTAATACCTTTTATAGTATGCATTGAAACTATCAGTAATGTAATCCGACCAGGAACTTTAGCAGTACGATTAACTGCTAATATAATT

GACGAGATATTTCTCGAGAAGGAACTTTTCAAGGACTTCACACTATTCCCGTAACATTAGGATTACGATGAGGAATAATTTTATTTATTATTTCTGAAGTTTTTTTCTTTATTTCCTTCTTTTGAGCTTTTTTCCATAGTAGCTTATCCCCAACAATCGAATT

AATTGTCATTTTTCTAGAAGTCATCATTTTGGATTTGAAGCAGCTGCTTGATATTGACATTTTGTTGATGTAGTTTGACTATTCTTATATATTTCAATT

AATTTTAGGAATATATTCTTATAAAATGTTTGATCAAGGATGAAGTGAATATTTTGGAGGTCAGATATTATATAATCAAT

ATTATTCAATAACAGGGGATTTTAATAGAACTACATTGAATATACTAAATGATAAGGGGTGAACTATATCTTTTAGAATT

AATTCCTTTTTCTTCTTGATTACCTGCAGCTATGGCAGCTCCGACTCCTGTTTCTGCTTTAGTACATTCTTCTACATTAG

AATTTATTTTCAAAATGTAAAATCTTATAATGCTGGTATATTAACAGCTTTATCTAATCGAATCGGAGATGTTGCCTTAT

CTTTACATTCAATATCTATTGTAATAACTTTTTTATTTGATTGAATAAGTTTAATATTTATATCTTTTGTTTTATTAATN

AATTTTTATTAGATTTACTTTATTTATTTTGAGATTAAAGTTTTTATTAATAGATTTAGTATATTTTATTGAATGAGAAATT

GAGGGTATCAACCTGAACGTTTACAGGCGGGGATTTATTTATTATTTTATACTTTACTGGCTTCGTTGCCGTTATTAATT

AATTATGAAAGTTACTTTAGTATATTTTTTTTAACTTTTTGTGTTTGTGAAGGGGTTCTAGGGCTATCTATTTTAGTTTC

AATTATTATATTTATTTTTGGAAGTATTGTTTTTATTTCTAGTCGTAAGCATTTACTTTGTACTTTATTGAGATTAGAAT

AATTTTTATAGGAGGAATATTAGTTTTATTCATTTATGTTACTTCTCTTTCATCTAATGAAATATTTTCATTATCTATAAAACTATTTTTTTTATCTTTAAGTATAATT

AATTTTAACAGGATTATTCTTAGCAATACACTATACTGCTGATATTGAAACAGCTTTTAATAGAGTAAATCACATTTATCGTGATGTTAATAATGGTTGATTCCTACGAATT

CTGTTGATAATGCTACTTTAACTCGATTCTTTACATTTCATTTTATTTTACCTTTCATTGTATTAGCTTTAACTATAATT

CTTTTGATTTTGCTGAAGGGGAGTCTGAGTTAGTTTCAGGATTTAATGTAGAATATAGAAGAGGGGGATTTGCTTTAATT

AATTTTTTTATATATCAGAAATACATATGATTTATTGTATTTTGTTTTCCTTTAGGTCTCGTTTGATTTGCATCTTGTTT

AATTTAAGCTATAAACTTTTTGTAATAAACTAGGATTAGATACCCTATTATTAAAAATAAATATTAAAATGCTAAAGTAG

>Gv10-C-06

AATTTTATCTACTTTATTATTAAAAAGAGGAGCAGCTCCTTTTCATTTTTGATTCCCAGGAGTTATAGAAGGATTAAATT

AATTAATCATATTGGTTGAATATTAATAGCTATAATAAATAACGAACTTTTATGACTAACTTATTTTTTATTATATTCAATN

CAACAAATCATAAAGATATTGGAACTTTATATTTCATTTTTGGAGTATGATCCGGAATAGTCGGAACTTCTCTAAGAATT

AATTGGATTATTGGGATTTATTGTTTGAGCTCATCATATATTTACAGTAGGTATAGACGTAGATACTCGAGCTTATTTTACTTCAGCAACTATAATT

CATGATACTTATTATGTAGTTGCCCATTTTCATTATGTTTTATCTATAGGAGCTGTATTTGCTATTATAGCAGGATTTATTCATTGATACCCTTTATTAACAGGAATAGTTATAAACCCTTCATG

CTGGAATACCTCGACGATACTCAGATTTTCCCGATAGTTACTTAACTTGAAATATTATTTCTTCTTTAGGAAGAACAATN

AATTTGTCACACACAACATATATTTGCTCATTTAGTTCCTCAAGGAACTCCCCCTGTTTTAATACCTTTTATAGTATGCATTGAAACTATTAGTAATGTAATCCGACCAGGAACTTTAGCAGTACGATTAACTGCTAATATAATT

GACGAGATATTTCTCGAGAAGGAACTTTTCAAGGACTTCACACTATTCCCGTAACATTAGGATTACGATGAGGAATAATTTTATTTATTATTTCTGAAGTTTTTTTCTTTATTTCCTTCTTTTGAGCTTTTTTCCATAGTAGCTTATCCCCAACAATCGAATT

AATTGTCATTTTTCTAGAAGTCATCATTTTGGATTTGAAGCAGCTGCTTGATATTGACATTTTGTTGATGTAGTTTGACTATTCTTATATATTTCAATT

AATTTTAGGAATATATTCTTATAAAATGTTTGATCAAGGATGAAGTGAATATTTTGGAGGTCAGATATTATATAATCAAT

ATTATTCAATAACAGGGGATTTTAATAGAACTACATTGAATATACTAAATGATAAGGGGTGAACTATATCTTTTAGAATT

AATTCCTTTTTCTTCTTGATTACCTGCAGCTATGGCAGCTCCGACTCCTGTTTCTGCTTTAGTACATTCTTCTACATTAG

AATTTATTTTCAAAATGTAAAATCTTATAATGCTGGTATATTAACAGCTTTATCTAATCGAATCGGAGATGTTGCCTTAT

CTTTACATTCAATATCTATTGTAATAACTTTTTTATTTGATTGAATAAGTTTAATATTTATATCTTTTGTTTTATTAATN

AATTTTTATTAGATTTACTTTATTTATTTTGAGATTAAAGTTTTTATTAATAGATTTAGTATATTTTATTGAATGAGAAATT

GAGGGTATCAACCTGAACGTTTACAGGCGGGGATTTATTTATTATTTTATACTTTACTGGCTTCGTTGCCGTTATTAATT

AATTATGAAAGTTACTTTAGTATATTTTTTTTAACTTTTTGTGTTTGTGAAGGGGTTCTAGGGCTATCTATTTTAGTTTC

AATTATTATATTTATTTTTGGAAGTATTGTTTTTATTTCTAGTCGTAAGCATTTACTTTGTACTTTATTGAGATTAGAGT

AATTTTTATAGGAGGAATATTAGTTTTATTCATTTATGTTACTTCTCTTTCATCTAATGAAATATTTTCATTATCTATAAAACTATTTTTTTTATCTTTAAGTATAATT

AATTTTAACAGGATTATTCTTAGCAATACACTATACTGCTGATATTGAAACAGCTTTTAATAGAGTAAATCACATTTATCGTGATGTTAATAATGGTTGATTCCTACGAATT

CTGTTGATAATGCTACTTTAACTCGATTCTTTACATTTCATTTTATTTTACCTTTCATTGTATTAGCTTTAACTATAATT

CTTTTGATTTTGCTGAAGGGGAGTCTGAGTTAGTTTCAGGATTTAATGTAGAATATAGAAGAGGGGGATTTGCTTTAATT

AATTTTTTTATATATCAGAAATACATATGATTTATTGTATTTTGTTTTCCTTTAGGTCTCGTTTGATTTGCATCTTGTTT

AATTTAAGCTATAAACTTTTTGTAATAAACTAGGATTAGATACCCTATTATTAAAAATAAATATTAAAATGCTAAAGTAG

>Gv10-C-07

AATTTTATCTACTTTATTATTAAAAAGAGGGGCAGCTCCTTTTCATTTTTGATTCCCAGGAGTTATAGAAGGATTAAATT

NATTAATCATATTGGTTGAATATTAATAGCTATAATAAATAACGAACTTTTATGACTAACTTATTTTTTATTATATTCAATT

CAACAAATCATAAAGATATTGGAACTTTATATTTCATTTTTGGAGTATGATCCGGAATAGTCGGAACTTCTCTAAGAATT

AATTGGATTATTGGGATTTATTGTTTGAGCTCATCATATATTTACAGTAGGTATAGACGTAGATACTCGAGCTTATTTTACTTCAGCAACTATAATT

CATGATACTTATTATGTAGTTGCCCATTTTCATTATGTTTTATCTATAGGAGCTGTATTTGCTATTATAGCAGGATTTATTCATTGATACCCTTTATTAACAGGAATAGTTATAAACCCTTCATG

CTGGAATACCTCGACGATACTCAGATTTTCCCGATAGTTACTTAACTTGAAATATTATTTCTTCTTTAGGAAGAACAATT

AATTTGTCACACACAACATATATTTGCTCATTTAGTTCCTCAAGGAACTCCCCCTGTTTTAATACCTTTTATAGTATGCATTGAAACTATCAGTAATGTAATCCGACCAGGAACTTTAGCAGTACGATTAACTGCTAATATAATT

GACGAGATATTTCTCGAGAAGGAACTTTTCAAGGACTTCACACTATTCCCGTAACATTAGGATTACGATGAGGAATAATTTTATTTATTATTTCTGAAGTTTTTTTCTTTATTTCCTTCTTTTGAGCTTTTTTCCATAGTAGCTTATCCCCAACAATCGAATT

AATTGTCATTTTTCTAGAAGTCATCATTTTGGATTTGAAGCAGCTGCTTGATATTGACATTTTGTTGATGTAGTTTGACTATTCTTATATATTTCAATT

AATTTTAGGAATATATTCTTATAAAATGTTTGATCAAGGATGAAGTGAATATTTTGGAGGTCAGATATTATATAATCAAT

ATTATTCAATAACAGGGGATTTTAATAGAACTACATTGAATATACTAAATGATAAGGGGTGAACTATATCTTTTAGAATT

NATTCCTTTTTCTTCTTGATTACCTGCAGCTATGGCAGCTCCGACTCCTGTTTCTGCTTTAGTACATTCTTCTACATTAG

AATTTATTTTCAAAATGTAAAATCTTATAATGCTGGTATATTAACAGCTTTATCTAATCGAATCGGAGATGTTGCCTTAT

CTTTACATTCAATATCTATTGTAATAACTTTTTTATTTGATTGAATAAGTTTAATATTTATATCTTTTGTTTTATTAATN

AATTTTTATTAGATTTACTTTATTTATTTTGAGATTAAAGTTTTTATTAATAGATTTAGTATATTTTATTGAATGAGAAATT

GAGGGTATCAACCTGAACGTTTACAGGCGGGGATTTATTTATTATTTTATACTTTACTGGCTTCGTTGCCGTTATTAATT

AATTATGAAAGTTACTTTAGTATATTTTTTTTAACTTTTTGTGTTTGTGAAGGGGTTCTAGGGCTATCTATTTTAGTTTC

NATTATTATATTTATTTTTGGAAGTATTGTTTTTATTTCTAGTCGTAAGCATTTACTTTGTACTTTATTGAGATTAGAAT

AATTTTTATAGGAGGAATATTAGTTTTATTCATTTATGTTACTTCTCTTTCATCTAATGAAATATTTTCATTATCTATAAAACTATTTTTTTTATCTTTAAGTATAATT

AATTTTAACAGGATTATTCTTAGCAATACACTATACTGCTGATATTGAAACAGCTTTTAATAGAGTAAATCACATTTATCGTGATGTTAATAATGGTTGATTCCTACGAATT

CTGTTGATAATGCTACTTTAACTCGATTCTTTACATTTCATTTTATTTTACCTTTCATTGTATTAGCTTTAACTATAATT

CTTTTGATTTTGCTGAAGGGGAGTCTGAGTTAGTTTCAGGATTTAATGTAGAATATAGAAGAGGGGGATTTGCTTTAATT

AATTTTTTTATATATCAGAAATACATATGATTTATTGTATTTTGTTTTCCTTTAGGTCTCGTTTGATTTGCATCTTGTTT

AATTTAAGCTATAAACTTTTTGTAATAAACTAGGATTAGATACCCTATTATTAAAAATAAATATTAAAATGCTAAAGTAG

>Gv10-C-08

NATTTTATCTACTTTATTATTAAAAAGAGGGGCAGCTCCTTTTCATTTTTGATTCCCAGGAGTTATAGAAGGATTAAATT

AATTAATCATATTGGTTGAATATTAATAGCTATAATAAATAACGAACTTTTATGACTAACTTATTTTTTATTATATTCAATT

CAACAAATCATAAAGATATTGGAACTTTATATTTCATTTTTGGAGTATGATCCGGAATAGTCGGAACTTCTCTAAGAATT

AATTGGATTATTGGGATTTATTGTTTGAGCTCATCATATATTTACAGTAGGTATAGACGTAGATACTCGAGCTTATTTTACTTCAGCAACTATAATT

CATGATACTTATTATGTAGTTGCCCATTTTCATTATGTTTTATCTATAGGAGCTGTATTTGCTATTATAGCAGGATTTATTCATTGATACCCTTTATTAACAGGAATAGTTATAAACCCTTCATG

CTGGAATACCTCGACGATACTCAGATTTTCCCGATAGTTACTTAACTTGAAATATTATTTCTTCTTTAGGAAGAACAATN

AATTTGTCACACACAACATATATTTGCTCATTTAGTTCCTCAAGGAACTCCCCCTGTTTTAATACCTTTTATAGTATGCATTGAAACTATCAGTAATGTAATCCGACCAGGAACTTTAGCAGTACGATTAACTGCTAATATAATT

GACGAGATATTTCTCGAGAAGGAACTTTTCAAGGACTTCACACTATTCCCGTAACATTAGGATTACGATGAGGAATAATTTTATTTATTATTTCTGAAGTTTTTTTCTTTATTTCCTTCTTTTGAGCTTTTTTCCATAGTAGCTTATCCCCAACAATCGAATT

AATTGTCATTTTTCTAGAAGTCATCATTTTGGATTTGAAGCAGCTGCTTGATATTGACATTTTGTTGATGTAGTTTGACTATTCTTATATATTTCAATT

AATTTTAGGAATATATTCTTATAAAATGTTTGATCAAGGATGAAGTGAATATTTTGGAGGTCAGATATTATATAATCAAT

ATTATTCAATAACAGGGGATTTTAATAGAACTACATTGAATATACTAAATGATAAGGGGTGAACTATATCTTTTAGAATT

AATTCCTTTTTCTTCTTGATTACCTGCAGCTATGGCAGCTCCGACTCCTGTTTCTGCTTTAGTACATTCTTCTACATTAG

AATTTATTTTCAAAATGTAAAATCTTATAATGCTGGTATATTAACAGCTTTATCTAATCGAATCGGAGATGTTGCCTTAT

CTTTACATTCAATATCTATTGTAATAACTTTTTTATTTGATTGAATAAGTTTAATATTTATATCTTTTGTTTTATTAATN

AATTTTTATTAGATTTACTTTATTTATTTTGAGATTAAAGTTTTTATTAATAGATTTAGTATATTTTATTGAATGAGAAATT

GAGGGTATCAACCTGAACGTTTACAGGCGGGGATTTATTTATTATTTTATACTTTACTGGCTTCGTTGCCGTTATTAATT

AATTATGAAAGTTACTTTAGTATATTTTTTTTAACTTTTTGTGTTTGTGAAGGGGTTCTAGGGCTATCTATTTTAGTTTC

AATTATTATATTTATTTTTGGAAGTATTGTTTTTATTTCTAGTCGTAAGCATTTACTTTGTACTTTATTGAGATTAGAAT

NATTTTTATAGGAGGAATATTAGTTTTATTCATTTATGTTACTTCTCTTTCATCTAATGAAATATTTTCATTATCTATAAAACTATTTTTTTTATCTTTAAGTATAATT

AATTTTAACAGGATTATTCTTAGCAATACACTATACTGCTGATATTGAAACAGCTTTTAATAGAGTAAATCACATTTATCGTGATGTTAATAATGGTTGATTCCTACGAATT

CTGTTGATAATGCTACTTTAACTCGATTCTTTACATTTCATTTTATTTTACCTTTCATTGTATTAGCTTTAACTATAATT

CTTTTGATTTTGCTGAAGGGGAGTCTGAGTTAGTTTCAGGATTTAATGTAGAATATAGAAGAGGGGGATTTGCTTTAATT

AATTTTTTTATATATCAGAAATACATATGATTTATTGTATTTTGTTTTCCTTTAGGTCTCGTTTGATTTGCATCTTGTTT

AATTTAAGCTATAAACTTTTTGTAATAAACTAGGATTAGATACCCTATTATTAAAAATAAATATTAAAATGCTAAAGTAG

>Gv10-C-09

AATTTTATCTACTTTATTATTAAAAAGAGGGGCAGCTCCTTTTCATTTTTGATTCCCAGGAGTTATAGAAGGATTAAATT

AATTAATCATATTGGTTGAATATTAATAGCTATAATAAATAACGAACTTTTATGACTAACTTATTTTTTATTATATTCAATN

CAACAAATCATAAAGATATTGGAACTTTATATTTCATTTTTGGAGTATGATCCGGAATAGTCGGAACTTCTCTAAGAATT

AATTGGATTATTGGGATTTATTGTTTGAGCTCATCATATATTTACAGTAGGTATAGACGTAGATACTCGAGCTTATTTTACTTCAGCAACTATAATN

CATGATACTTATTATGTAGTTGCCCATTTTCATTATGTTTTATCTATAGGAGCTGTATTTGCTATTATAGCAGGATTTATTCATTGATACCCTTTATTAACAGGAATAGTTATAAACCCTTCATG

CTGGAATACCTCGACGATACTCGGATTTTCCCGATAGTTACTTAACTTGAAATATTATTTCTTCTTTAGGAAGAACAATN

AATTTGTCACACACAACATATATTTGCTCATTTAGTTCCTCAAGGAACTCCCCCTGTTTTAATACCTTTTATAGTATGCATTGAAACTATCAGTAATGTAATCCGACCAGGAACTTTAGCAGTACGATTAACTGCTAATATAATT

GACGAGATATTTCTCGAGAAGGAACTTTTCAAGGACTTCACACTATTCCCGTAACATTAGGATTACGATGAGGAATAATTTTATTTATTATTTCTGAAGTTTTTTTCTTTATTTCCTTCTTTTGAGCTTTTTTCCATAGTAGCTTATCCCCAACAATCGAATT

AATTGTCATTTTTCTAGAAGTCATCATTTTGGATTTGAAGCAGCTGCTTGATATTGACATTTTGTTGATGTAGTTTGACTATTCTTATATATTTCAATT

AATTTTAGGAATATATTCTTATAAAATGTTTGATCAAGGATGAAGTGAATATTTTGGAGGTCAGATATTATATAATCAAT

ATTATTCAATAACAGGGGATTTTAATAGAACTACATTGAATATACTAAATGATAAGGGGTGAACTATATCTTTTAGAATT

AATTCCTTTTTCTTCTTGATTACCTGCAGCTATGGCAGCTCCGACTCCTGTTTCTGCTTTAGTACATTCTTCTACATTAG

AATTTATTTTCAAAATGTAAAATCTTATAATGCTGGTATATTAACAGCTTTATCTAATCGAATCGGAGATGTTGCCTTAT

CTTTACATTCAATATCTATTGTAATAACTTTTTTATTTGATTGAATAAGTTTAATATTTATATCTTTTGTTTTATTAATT

AATTTTTATTAGATTTACTTTATTTATTTTGAGATTAAAGTTTTTATTAATAGATTTAGTATATTTTATTGAATGAGAAATT

GAGGGTATCAACCTGAACGTTTACAGGCGGGGATTTATTTATTATTTTATACTTTACTGGCTTCGTTGCCGTTATTAATT

AATTATGAAAGTTACTTTAGTATATTTTTTTTAACTTTTTGTGTTTGTGAAGGGGTTCTAGGGCTATCTATTTTAGTTTC

AATTATTATATTTATTTTTGGAAGTATTGTTTTTATTTCTAGTCGTAAGCATTTACTTTGTACTTTATTGAGATTAGAAT

AATTTTTATAGGAGGAATATTAGTTTTATTCATTTATGTTACTTCTCTTTCATCTAATGAAATATTTTCATTATCTATAAAACTATTTTTTTTATCTTTAAGTATAATT

AATTTTAACAGGATTATTCTTAGCAATACACTATACTGCTGATATTGAAACAGCTTTTAATAGAGTAAATCACATTTATCGTGATGTTAATAATGGTTGATTCCTACGAATT

CTGTTGATAATGCTACTTTAACTCGATTCTTTACATTTCATTTTATTTTACCTTTCATTGTATTAGCTTTAACTATAATT

CTTTTGATTTTGCTGAAGGGGAGTCTGAGTTAGTTTCAGGATTTAATGTAGAATATAGAAGAGGGGGATTTGCTTTAATT

AATTTTTTTATATATCAGAAATACATATGATTTATTGTATTTTGTTTTCCTTTAGGTCTCGTTTGATTTGCATCTTGTTT

AATTTAAGCTATAAACTTTTTGTAATAAACTAGGATTAGATACCCTATTATTAAAAATAAATATTAAAATGCTAAAGTAG

>Gv10-C-10

AATTTTATCTACTTTATTATTAAAAAGAGGGGCAGCTCCTTTTCATTTTTGATTCCCAGGAGTTATAGAAGGATTAAATT

NATTAATCATATTGGTTGAATATTAATAGCTATAATAAATAACGAACTTTTATGACTAACTTATTTTTTATTATATTCAATN

CAACAAATCATAAAGATATTGGAACTTTATATTTCATTTTTGGAGTATGATCCGGAATAGTCGGAACTTCTCTAAGAATT

AATTGGATTATTGGGATTTATTGTTTGAGCTCATCATATATTTACAGTAGGTATAGACGTAGATACTCGAGCTTATTTTACTTCAGCAACTATAATT

CATGATACTTATTATGTAGTTGCCCATTTTCATTATGTTTTATCTATAGGAGCTGTATTTGCTATTATAGCAGGATTTATTCATTGATACCCTTTATTAACAGGAATAGTTATAAACCCTTCATG

CTGGAATACCTCGACGATACTCAGATTTTCCCGATAGTTACTTAACTTGAAATATTATTTCTTCTTTAGGAAGAACAATN

AATTTGTCACACACAACATATATTTGCTCATTTAGTTCCTCAAGGAACTCCCCCTGTTTTAATACCTTTTATAGTATGCATTGAAACTATCAGTAATGTAATCCGACCAGGAACTTTAGCAGTACGATTAACTGCTAATATAATT

GACGAGATATTTCTCGAGAAGGAACTTTTCAAGGACTTCACACTATTCCCGTAACATTAGGATTACGATGAGGAATAATTTTATTTATTATTTCTGAAGTTTTTTTCTTTATTTCCTTCTTTTGAGCTTTTTTCCATAGTAGCTTATCCCCAACAATCGAATT

AATTGTCATTTTTCTAGAAGTCATCATTTTGGATTTGAAGCAGCTGCTTGATATTGACATTTTGTTGATGTAGTTTGACTATTCTTATATATTTCAATN

AATTTTAGGAATATATTCTTATAAAATGTTTGATCAAGGATGAAGTGAATATTTTGGAGGTCAGATATTATATAATCAAT

ATTATTCAATAACAGGGGATTTTAATAGAACTACATTGAATATACTAAATGATAAGGGGTGAACTATATCTTTTAGAATT

AATTCCTTTTTCTTCTTGATTACCTGCAGCTATGGCAGCTCCGACTCCTGTTTCTGCTTTAGTACATTCTTCTACATTAG

AATTTATTTTCAAAATGTAAAATCTTATAATGCTGGTATATTAACAGCTTTATCTAATCGAATCGGAGATGTTGCCTTAT

CTTTACATTCAATATCTATTGTAATAACTTTTTTATTTGATTGAATAAGTTTAATATTTATATCTTTTGTTTTATTAATT

AATTTTTATTAGATTTACTTTATTTATTTTGAGATTAAAGTTTTTATTAATAGATTTAGTATATTTTATTGAATGAGAAATT

GAGGGTATCAACCTGAACGTTTACAGGCGGGGATTTATTTATTATTTTATACTTTACTGGCTTCGTTGCCGTTATTAATT

AATTATGAAAGTTACTTTAGTATATTTTTTTTAACTTTTTGTGTTTGTGAAGGGGTTCTAGGGCTATCTATTTTAGTTTC

AATTATTATATTTATTTTTGGAAGTATTGTTTTTATTTCTAGTCGTAAGCATTTACTTTGTACTTTATTGAGATTAGAAT

AATTTTTATAGGAGGAATATTAGTTTTATTCATTTATGTTACTTCTCTTTCATCTAATGAAATATTTTCATTATCTATAAAACTATTTTTTTTATCTTTAAGTATAATT

AATTTTAACAGGATTATTCTTAGCAATACACTATACTGCTGATATTGAAACAGCTTTTAATAGAGTAAATCACATTTATCGTGATGTTAATAATGGTTGATTCCTACGAATT

CTGTTGATAATGCTACTTTAACTCGATTCTTTACATTTCATTTTATTTTACCTTTCATTGTATTAGCTTTAACTATAATT

CTTTTGATTTTGCTGAAGGGGAGTCTGAGTTAGTTTCAGGATTTAATGTAGAATATAGAAGAGGGGGATTTGCTTTAATT

AATTTTTTTATATATCAGAAATACATATGATTTATTGTATTTTGTTTTCCTTTAGGACTCGTTTGATTTGCATCTTGTTT

AATTTAAGCTATAAACTTTTTGTAATAAACTAGGATTAGATACCCTATTATTAAAAATAAATATTAAAATGCTAAAGTAG

>Gv10-E-02

AATTTTATCTACTTTATTATTAAAAAGAGGGGCAGCTCCTTTTCATTTTTGATTCCCAGGAGTTATAGAAGGATTAAATT

AATTAATCATATTGGTTGAATATTAATAGCTATAATAAATAACGAACTTTTATGACTAACTTATTTTTTATTATATTCAANN

CAACAAATCATAAAGATATTGGAACTTTATATTTCATTTTTGGAGTATGATCCGGAATAGTCGGAACTTCTCTAAGAATT

AATTGGATTATTGGGATTTATTGTTTGAGCTCATCATATATTTACAGTAGGTATAGACGTAGATACTCGAGCTTATTTTACTTCAGCAACTATAATT

CATGATACTTATTATGTAGTTGCCCATTTTCATTATGTTTTATCTATAGGAGCTGTATTTGCTATTATAGCAGGATTTATTCATTGATACCCTTTATTAACAGGAATAGTTATAAACCCTTCATG

CTGGAATACCTCGACGATACTCAGATTTTCCCGATAGTTACTTAACTTGAAATATTATTTCTTCTTTAGGAAGAACAATT

AATTTGTCACACACAACATATATTTGCTCATTTAGTTCCTCAAGGAACTCCCCCTGTTTTAATACCTTTTATAGTATGCATTGAAACTATCAGTAATGTAATCCGACCAGGAACTTTAGCAGTACGATTAACTGCTAATATAATT

GACGAGATATTTCTCGAGAAGGAACTTTTCAAGGACTTCACACTATTCCCGTAACATTAGGATTACGATGAGGAATAATTTTATTTATTATTTCTGAAGTTTTTTTCTTTATTTCCTTCTTTTGAGCTTTTTTCCATAGTAGCTTATCCCCAACAATCGAATT

AATTGTCATTTTTCTAGAAGTCATCATTTTGGATTTGAAGCAGCTGCTTGATATTGACATTTTGTTGATGTAGTTTGACTATTCTTATATATTTCAATT

AATTTTAGGAATATATTCTTATAAAATGTTTGATCAAGGATGAAGTGAATATTTTGGAGGTCAGATATTATATAATCAAT

ATTATTCAATAACAGGGGATTTTAATAGAACTACATTGAATATACTAAATGATAAGGGGTGAACTATATCTTTTAGAATT

AATTCCTTTTTCTTCTTGATTACCTGCAGCTATGGCAGCTCCGACTCCTGTTTCTGCTTTAGTACATTCTTCTACATTAG

AATTTATTTTCAAAATGTAAAATCTTATAATGCTGGTATATTAACAGCTTTATCTAATCGAATCGGAGATGTTGCCTTAT

CTTTACATTCAATATCTATTGTAATAACTTTTTTATTTGATTGAATAAGTTTAATATTTATATCTTTTGTTTTATTAATT

AATTTTTATTAGATTTACTTTATTTATTTTGAGATTAAAGTTTTTATTAATAGATTTAGTATATTTTATTGAATGAGAAATT

GAGGGTATCAACCTGAACGTTTACAGGCGGGGATTTATTTATTATTTTATACTTTACTGGCTTCGTTGCCGTTATTAATT

AATTATGAAAGTTACTTTAGTATATTTTTTTTAACTTTTTGTGTTTGTGAAGGGGTTCTAGGGCTATCTATTTTAGTTTC

AATTATTATATTTATTTTTGGAAGTATTGTTTTTATTTCTAGTCGTAAGCATTTACTTTGTACTTTATTGAGATTAGAAT

AATTTTTATAGGAGGAATATTAGTTTTATTCATTTATGTTACTTCTCTTTCATCTAATGAAATATTTTCATTATCTATAAAACTATTTTTTTTATCTTTAAGTATAATT

AATTTTAACAGGATTATTCTTAGCAATACACTATACTGCTGATATTGAAACAGCTTTTAATAGAGTAAATCACATTTATCGTGATGTTAATAATGGTTGATTCCTACGAATT

CTGTTGATAATGCTACTTTAACTCGATTCTTTACATTTCATTTTATTTTACCTTTCATTGTATTAGCTTTAACTATAATT

CTTTTGATTTTGCTGAAGGGGAGTCTGAGTTAGTTTCAGGATTTAATGTAGAATATAGAAGAGGGGGATTTGCTTTAATT

AATTTTTTTATATATCAGAAATACATATGATTTATTGTATTTTGTTTTCCTTTAGGTCTCGTTTGATTTGCATCTTGTTT

AATTTAAGCTATAAACTTTTTGTAATAAACTAGGATTAGATACCCTATTATTAAAAATAAATATTAAAATGCTAAAGTAG

>Gv10-E-03

AATTTTATCTACTTTATTATTAAAAAGAGGGGCAGCTCCTTTTCATTTTTGATTCCCAGGAGTTATAGAAGGATTAAATT

AATTAATCATATTGGTTGAATATTAATAGCTATAATAAATAACGAACTTTTATGACTAACTTATTTTTTATTATATTCAATT

CAACAAATCATAAAGATATTGGAACTTTATATTTCATTTTTGGAGTATGATCCGGAATAGTCGGAACTTCTCTAAGAATT

AATTGGATTATTGGGATTTATTGTTTGAGCTCATCATATATTTACAGTAGGTATAGACGTAGATACTCGAGCTTATTTTACTTCAGCAACTATAATT

CATGATACTTATTATGTAGTTGCCCATTTTCATTATGTTTTATCTATAGGAGCTGTATTTGCTATTATAGCAGGATTTATTCATTGATACCCTTTATTAACAGGAATAGTTATAAACCCTTCATG

CTGGAATACCTCGACGATACTCAGATTTTCCCGATAGTTACTTAACTTGAAATATTATTTCTTCTTTAGGAAGAACAATT

AATTTGTCACACACAACATATATTTGCTCATTTAGTTCCTCAAGGAACTCCCCCTGTTTTAATACCTTTTATAGTATGCATTGAAACTATCAGTAATGTAATCCGACCAGGAACTTTAGCAGTACGATTAACTGCTAATATAATT

GACGAGATATTTCTCGAGAAGGAACTTTTCAAGGACTTCACACTATTCCCGTAACATTAGGATTACGATGAGGAATAATTTTATTTATTATTTCTGAAGTTTTTTTCTTTATTTCCTTCTTTTGAGCTTTTTTCCATAGTAGCTTATCCCCAACAATCGAATT

AATTGTCATTTTTCTAGAAGTCATCATTTTGGATTTGAAGCAGCTGCTTGATATTGACATTTTGTTGATGTAGTTTGACTATTCTTATATATTTCAATT

AATTTTAGGAATATATTCTTATAAAATGTTTGATCAAGGATGAAGTGAATATTTTGGAGGTCAGATATTATATAATCAAT

ATTATTCAATAACAGGGGATTTTAATAGAACTACATTGAATATACTAAATGATAAGGGGTGAACTATATCTTTTAGAATT

AATTCCTTTTTCTTCTTGATTACCTGCAGCTATGGCAGCTCCGACTCCTGTTTCTGCTTTAGTACATTCTTCTACATTAG

AATTTATTTTCAAAATGTAAAATCTTATAATGCTGGTATATTAACAGCTTTATCTAATCGAATCGGAGATGTTGCCTTAT

CTTTACATTCAATATCTATTGTAATAACTTTTTTATTTGATTGAATAAGTTTAATATTTATATCTTTTGTTTTATTAATT

AATTTTTATTAGATTTACTTTATTTATTTTGAGATTAAAGTTTTTATTAATAGATTTAGTATATTTTATTGAATGAGAAATT

GAGGGTATCAACCTGAACGTTTACAGGCGGGGATTTATTTATTATTTTATACTTTACTGGCTTCGTTGCCGTTATTAATT

AATTATGAAAGTTACTTTAGTATATTTTTTTTAACTTTTTGTGTTTGTGAAGGGGTTCTAGGGCTATCTATTTTAGTTTC

AATTATTATATTTATTTTTGGAAGTATTGTTTTTATTTCTAGTCGTAAGCATTTACTTTGTACTTTATTGAGATTAGAAT

AATTTTTATAGGAGGAATATTAGTTTTATTCATTTATGTTACTTCTCTTTCATCTAATGAAATATTTTCATTATCTATAAAACTATTTTTTTTATCTTTAAGTATAATT

AATTTTAACAGGATTATTCTTAGCAATACACTATACTGCTGATATTGAAACAGCTTTTAATAGAGTAAATCACATTTATCGTGATGTTAATAATGGTTGATTCCTACGAATT

CTGTTGATAATGCTACTTTAACTCGATTCTTTACATTTCATTTTATTTTACCTTTCATTGTATTAGCTTTAACTATAATT

CTTTTGATTTTGCTGAAGGGGAGTCTGAGTTAGTTTCAGGATTTAATGTAGAATATAGAAGAGGGGGATTTGCTTTAATT

AATTTTTTTATATATCAGAAATACATATGATTTATTGTATTTTGTTTTCCTTTAGGTCTCGTTTGATTTGCATCTTGTTT

AATTTAAGCTATAAACTTTTTGTAATAAACTAGGATTAGATACCCTATTATTAAAAATAAATATTAAAATGCTAAAGTAG

>Gv10-E-04

AATTTTATCTACTTTATTATTAAAAAGAGGAGCAGCTCCTTTTCATTTTTGATTCCCAGGAGTTATAGAAGGATTAAATT

AATTAATCATATTGGTTGAATATTAATAGCTATAATAAATAACGAACTTTTATGACTAACTTATTTTTTATTATATTCAATT

CAACAAATCATAAAGATATTGGAACTTTATATTTCATTTTTGGAGTATGATCCGGAATAGTCGGAACTTCTCTAAGAATT

AATTGGATTATTGGGATTTATTGTTTGAGCTCATCATATATTTACAGTAGGTATAGACGTAGATACTCGAGCTTATTTTACTTCAGCAACTATAATT

CATGATACTTATTATGTAGTTGCCCATTTTCATTATGTTTTATCTATAGGAGCTGTATTTGCTATTATAGCAGGATTTATTCATTGATACCCTTTATTAACAGGAATAGTTATAAACCCTTCATG

CTGGAATACCTCGACGATACTCGGATTTTCCCGATAGTTACTTAACTTGAAATATTATTTCTTCTTTAGGAAGAACAATT

AATTTGTCACACACAACATATATTTGCTCATTTAGTTCCTCAAGGAACTCCCCCTGTTTTAATACCTTTTATAGTATGCATTGAAACTATTAGTAATGTAATCCGACCAGGAACTTTAGCAGTACGATTAACTGCTAATATAATT

GACGAGATATTTCTCGAGAAGGAACTTTTCAAGGACTTCACACTATTCCCGTAACATTAGGATTACGATGAGGAATAATTTTATTTATTATTTCTGAAGTTTTTTTCTTTATTTCCTTCTTTTGAGCTTTTTTCCATAGTAGCTTATCCCCAACAATCGAATT

AATTGTCATTTTTCTAGAAGTCATCATTTTGGATTTGAAGCAGCTGCTTGATATTGACATTTTGTTGATGTAGTTTGACTATTCTTATATATTTCAATT

AATTTTAGGAATATATTCTTATAAAATGTTTGATCAAGGATGAAGTGAATATTTTGGAGGTCAGATATTATATAATCAAT

ATTATTCAATAACAGGGGATTTTAATAGAACTACATTGAATATACTAAATGATAAGGGGTGAACTATATCTTTTAGAATT

AATTCCTTTTTCTTCTTGATTACCTGCAGCTATGGCAGCTCCGACTCCTGTTTCTGCTTTAGTACATTCTTCTACATTAG

AATTTATTTTCAAAATGTAAAATCTTATAATGCTGGTATATTAACAGCTTTATCTAATCGAATCGGAGATGTTGCCTTAT

CTTTACATTCAATATCTATTGTAATAACTTTTTTATTTGATTGAATAAGTTTAATATTTATATCTTTTGTTTTATTAATT

AATTTTTATTAGATTTACTTTATTTATTTTGAGATTAAAGTTTTTATTAATAGATTTAGTATATTTTATTGAATGAGAAATT

GAGGGTATCAACCTGAACGTTTACAGGCGGGGATTTATTTATTATTTTATACTTTACTGGCTTCGTTGCCGTTATTAATT

AATTATGAAAGTTACTTTAGTATATTTTTTTTAACTTTTTGTGTTTGTGAAGGGGTTCTAGGGCTATCTATTTTAGTTTC

AATTATTATATTTATTTTTGGAAGTATTGTTTTTATTTCTAGTCGTAAGCATTTACTTTGTACTTTATTGAGATTAGAGT

AATTTTTATAGGAGGAATATTAGTTTTATTCATTTATGTTACTTCTCTTTCATCTAATGAAATATTTTCATTATCTATAAAACTATTTTTTTTATCTTTAAGTATAATT

AATTTTAACAGGATTATTCTTAGCAATACACTATACTGCTGATATTGAAACAGCTTTTAATAGAGTAAATCACATTTATCGTGATGTTAATAATGGTTGATTCCTACGAATT

CTGTTGATAATGCTACTTTAACTCGATTCTTTACATTTCATTTTATTTTACCTTTCATTGTATTAGCTTTAACTATAATT

CTTTTGATTTTGCTGAAGGGGAGTCTGAGTTAGTTTCAGGATTTAATGTAGAATATAGAAGAGGGGGATTTGCTTTAATT

AATTTTTTTATATATCAGAAATACATATGATTTATTGTATTTTGTTTTCCTTTAGGTCTCGTTTGATTTGCATCTTGTTT

AATTTAAGCTATAAACTTTTTGTAATAAACTAGGATTAGATACCCTATTATTAAAAATAAATATTAAAATGCTAAAGTAG

>Gv10-E-05

AATTTTATCTACTTTATTATTAAAAAGAGGGGCAGCTCCTTTTCATTTTTGATTCCCAGGAGTTATAGAAGGATTAAATT

AATTAATCATATTGGTTGAATATTAATAGCTATAATAAATAACGAACTTTTATGACTAACTTATTTTTTATTATATTCAATT

CAACAAATCATAAAGATATTGGAACTTTATATTTCATTTTTGGAGTATGATCCGGAATAGTCGGAACTTCTCTAAGAATT

AATTGGATTATTGGGATTTATTGTTTGAGCTCATCATATATTTACAGTAGGTATAGACGTAGATACTCGAGCTTATTTTACTTCAGCAACTATAATN

CATGATACTTATTATGTAGTTGCCCATTTTCATTATGTTTTATCTATAGGAGCTGTATTTGCTATTATAGCAGGATTTATTCATTGATACCCTTTATTAACAGGAATAGTTATAAACCCTTCATG

NNNNNNNNNNNNNNNNNNNNNNNNNNNNNNNNNNNNNNNNNNNNNNNNNNNNNNNNNNNNNNNNNNNNNNNNNNNNNNNN

AATTTGTCACACACAACATATATTTGCTCATTTAGTTCCTCAAGGAACTCCCCCTGTTTTAATACCTTTTATAGTATGCATTGAAACTATCAGTAATGTAATCCGACCAGGAACTTTAGCAGTACGATTAACTGCTAATATAATT

GACGAGATATTTCTCGAGAAGGAACTTTTCAAGGACTTCACACTATTCCCGTAACATTAGGATTACGATGAGGAATAATTTTATTTATTATTTCTGAAGTTTTTTTCTTTATTTCCTTCTTTTGAGCTTTTTTCCATAGTAGCTTATCCCCAACAATCGAATT

AATTGTCATTTTTCTAGAAGTCATCATTTTGGATTTGAAGCAGCTGCTTGATATTGACATTTTGTTGATGTAGTTTGACTATTCTTATATATTTCAATT

AATTTTAGGAATATATTCTTATAAAATGTTTGATCAAGGATGAAGTGAATATTTTGGAGGTCAGATATTATATAATCAAT

ATTATTCAATAACAGGGGATTTTAATAGAACTACATTGAATATACTAAATGATAAGGGGTGAACTATATCTTTTAGAATT

AATTCCTTTTTCTTCTTGATTACCTGCAGCTATGGCAGCTCCGACTCCTGTTTCTGCTTTAGTACATTCTTCTACATTAG

AATTTATTTTCAAAATGTAAAATCTTATAATGCTGGTATATTAACAGCTTTATCTAATCGAATCGGAGATGTTGCCTTAT

CTTTACATTCAATATCTATTGTAATAACTTTTTTATTTGATTGAATAAGTTTAATATTTATATCTTTTGTTTTATTAATT

AATTTTTATTAGATTTACTTTATTTATTTTGAGATTAAAGTTTTTATTAATAGATTTAGTATATTTTATTGAATGAGAAATT

GAGGGTATCAACCTGAACGTTTACAGGCGGGGATTTATTTATTATTTTATACTTTACTGGCTTCGTTGCCGTTATTAATT

AATTATGAAAGTTACTTTAGTATATTTTTTTTAACTTTTTGTGTTTGTGAAGGGGTTCTAGGGCTATCTATTTTAGTTTC

AATTATTATATTTATTTTTGGAAGTATTGTTTTTATTTCTAGTCGTAAGCATTTACTTTGTACTTTATTGAGATTAGAAT

AATTTTTATAGGAGGAATATTAGTTTTATTCATTTATGTTACTTCTCTTTCATCTAATGAAATATTTTCATTATCTATAAAACTATTTTTTTTATCTTTAAGTATAATT

AATTTTAACAGGATTATTCTTAGCAATACACTATACTGCTGATATTGAAACAGCTTTTAATAGAGTAAATCACATTTATCGTGATGTTAATAATGGTTGATTCCTACGAATT

CTGTTGATAATGCTACTTTAACTCGATTCTTTACATTTCATTTTATTTTACCTTTCATTGTATTAGCTTTAACTATAATT

CTTTTGATTTTGCTGAAGGGGAGTCTGAGTTAGTTTCAGGATTTAATGTAGAATATAGAAGAGGGGGATTTGCTTTAATT

AATTTTTTTATATATCAGAAATACATATGATTTATTGTATTTTGTTTTCCTTTAGGTCTCGTTTGATTTGCATCTTGTTT

AATTTAAGCTATAAACTTTTTGTAATAAACTAGGATTAGATACCCTATTATTAAAAATAAATATTAAAATGCTAAAGTAG

>Gv10-E-06

AATTTTATCTACTTTATTATTAAAAAGAGGGGCAGCTCCTTTTCATTTTTGATTCCCAGGAGTTATAGAAGGATTAAATT

AATTAATCATATTGGTTGAATATTAATAGCTATAATAAATAACGAACTTTTATGACTAACTTATTTTTTATTATATTCAATT

CAACAAATCATAAAGATATTGGAACTTTATATTTCATTTTTGGAGTATGATCCGGAATAGTCGGAACTTCTCTAAGAATT

AATTGGATTATTGGGATTTATTGTTTGAGCTCATCATATATTTACAGTAGGTATAGACGTAGATACTCGAGCTTATTTTACTTCAGCAACTATAATT

CATGATACTTATTATGTAGTTGCCCATTTTCATTATGTTTTATCTATAGGAGCTGTATTTGCTATTATAGCAGGATTTATTCATTGATACCCTTTATTAACAGGAATAGTTATAAACCCTTCATG

CTGGAATACCTCGACGATACTCAGATTTTCCCGATAGTTACTTAACTTGAAATATTATTTCTTCTTTAGGAAGAACAATT

AATTTGTCACACACAACATATATTTGCTCATTTAGTTCCTCAAGGAACTCCCCCTGTTTTAATACCTTTTATAGTATGCATTGAAACTATCAGTAATGTAATCCGACCAGGAACTTTAGCAGTACGATTAACTGCTAATATAATT

GACGAGATATTTCTCGAGAAGGAACTTTTCAAGGACTTCACACTATTCCCGTAACATTAGGATTACGATGAGGAATAATTTTATTTATTATTTCTGAAGTTTTTTTCTTTATTTCCTTCTTTTGAGCTTTTTTCCATAGTAGCTTATCCCCAACAATCGAATT

AATTGTCATTTTTCTAGAAGTCATCATTTTGGATTTGAAGCAGCTGCTTGATATTGACATTTTGTTGATGTAGTTTGACTATTCTTATATATTTCAATT

AATTTTAGGAATATATTCTTATAAAATGTTTGATCAAGGATGAAGTGAATATTTTGGAGGTCAGATATTATATAATCAAT

ATTATTCAATAACAGGGGATTTTAATAGAACTACATTGAATATACTAAATGATAAGGGGTGAACTATATCTTTTAGAATT

AATTCCTTTTTCTTCTTGATTACCTGCAGCTATGGCAGCTCCGACTCCTGTTTCTGCTTTAGTACATTCTTCTACATTAG

AATTTATTTTCAAAATGTAAAATCTTATAATGCTGGTATATTAACAGCTTTATCTAATCGAATCGGAGATGTTGCCTTAT

CTTTACATTCAATATCTATTGTAATAACTTTTTTATTTGATTGAATAAGTTTAATATTTATATCTTTTGTTTTATTAATT

AATTTTTATTAGATTTACTTTATTTATTTTGAGATTAAAGTTTTTATTAATAGATTTAGTATATTTTATTGAATGAGAAATT

GAGGGTATCAACCTGAACGTTTACAGGCGGGGATTTATTTATTATTTTATACTTTACTGGCTTCGTTGCCGTTATTAATT

AATTATGAAAGTTACTTTAGTATATTTTTTTTAACTTTTTGTGTTTGTGAAGGGGTTCTAGGGCTATCTATTTTAGTTTC

AATTATTATATTTATTTTTGGAAGTATTGTTTTTATTTCTAGTCGTAAGCATTTACTTTGTACTTTATTGAGATTAGAAT

AATTTTTATAGGAGGAATATTAGTTTTATTCATTTATGTTACTTCTCTTTCATCTAATGAAATATTTTCATTATCTATAAAACTATTTTTTTTATCTTTAAGTATAATT

AATTTTAACAGGATTATTCTTAGCAATACACTATACTGCTGATATTGAAACAGCTTTTAATAGAGTAAATCACATTTATCGTGATGTTAATAATGGTTGATTCCTACGAATT

CTGTTGATAATGCTACTTTAACTCGATTCTTTACATTTCATTTTATTTTACCTTTCATTGTATTAGCTTTAACTATAATT

CTTTTGATTTTGCTGAAGGGGAGTCTGAGTTAGTTTCAGGATTTAATGTAGAATATAGAAGAGGGGGATTTGCTTTAATT

AATTTTTTTATATATCAGAAATACATATGATTTATTGTATTTTGTTTTCCTTTAGGTCTCGTTTGATTTGCATCTTGTTT

AATTTAAGCTATAAACTTTTTGTAATAAACTAGGATTAGATACCCTATTATTAAAAATAAATATTAAAATGCTAAAGTAG

>Gv10-E-07

AATTTTATCTACTTTATTATTAAAAAGAGGAGCAGCTCCTTTTCATTTTTGATTCCCAGGAGTTATAGAAGGATTAAATT

AATTAATCATATTGGTTGAATATTAATAGCTATAATAAATAACGAACTTTTATGACTAACTTATTTTTTATTATATTCAATN

CAACAAATCATAAAGATATTGGAACTTTATATTTCATTTTTGGAGTATGATCCGGAATAGTCGGAACTTCTCTAAGAATT

AATTGGATTATTGGGATTTATTGTTTGAGCTCATCATATATTTACAGTAGGTATAGACGTAGATACTCGAGCTTATTTTACTTCAGCAACTATAATT

CATGATACTTATTATGTAGTTGCCCATTTTCATTATGTTTTATCTATAGGAGCTGTATTTGCTATTATAGCAGGATTTATTCATTGATACCCTTTATTAACAGGAATAGTTATAAACCCTTCATG

CTGGAATACCTCGACGATACTCAGATTTTCCCGATAGTTACTTAACTTGAAATATTATTTCTTCTTTAGGAAGAACAATN

AATTTGTCACACACAACATATATTTGCTCATTTAGTTCCTCAAGGAACTCCCCCTGTTTTAATACCTTTTATAGTATGCATTGAAACTATTAGTAATGTAATCCGACCAGGAACTTTAGCAGTACGATTAACTGCTAATATAATT

GACGAGATATTTCTCGAGAAGGAACTTTTCAAGGACTTCACACTATTCCCGTAACATTAGGATTACGATGAGGAATAATTTTATTTATTATTTCTGAAGTTTTTTTCTTTATTTCCTTCTTTTGAGCTTTTTTCCATAGTAGCTTATCCCCAACAATCGAATT

AATTGTCATTTTTCTAGAAGTCATCATTTTGGATTTGAAGCAGCTGCTTGATATTGACATTTTGTTGATGTAGTTTGACTATTCTTATATATTTCAATT

AATTTTAGGAATATATTCTTATAAAATGTTTGATCAAGGATGAAGTGAATATTTTGGAGGTCAGATATTATATAATCAAT

ATTATTCAATAACAGGGGATTTTAATAGAACTACATTGAATATACTAAATGATAAGGGGTGAACTATATCTTTTAGAATT

AATTCCTTTTTCTTCTTGATTACCTGCAGCTATGGCAGCTCCGACTCCTGTTTCTGCTTTAGTACATTCTTCTACATTAG

AATTTATTTTCAAAATGTAAAATCTTATAATGCTGGTATATTAACAGCTTTATCTAATCGAATCGGAGATGTTGCCTTAT

CTTTACATTCAATATCTATTGTAATAACTTTTTTATTTGATTGAATAAGTTTAATATTTATATCTTTTGTTTTATTAATN

AATTTTTATTAGATTTACTTTATTTATTTTGAGATTAAAGTTTTTATTAATAGATTTAGTATATTTTATTGAATGAGAAATT

GAGGGTATCAACCTGAACGTTTACAGGCGGGGATTTATTTATTATTTTATACTTTACTGGCTTCGTTGCCGTTATTAATT

AATTATGAAAGTTACTTTAGTATATTTTTTTTAACTTTTTGTGTTTGTGAAGGGGTTCTAGGGCTATCTATTTTAGTTTC

AATTATTATATTTATTTTTGGAAGTATTGTTTTTATTTCTAGTCGTAAGCATTTACTTTGTACTTTATTGAGATTAGAGT

AATTTTTATAGGAGGAATATTAGTTTTATTCATTTATGTTACTTCTCTTTCATCTAATGAAATATTTTCATTATCTATAAAACTATTTTTTTTATCTTTAAGTATAATT

AATTTTAACAGGATTATTCTTAGCAATACACTATACTGCTGATATTGAAACAGCTTTTAATAGAGTAAATCACATTTATCGTGATGTTAATAATGGTTGATTCCTACGAATT

CTGTTGATAATGCTACTTTAACTCGATTCTTTACATTTCATTTTATTTTACCTTTCATTGTATTAGCTTTAACTATAATT

CTTTTGATTTTGCTGAAGGGGAGTCTGAGTTAGTTTCAGGATTTAATGTAGAATATAGAAGAGGGGGATTTGCTTTAATT

AATTTTTTTATATATCAGAAATACATATGATTTATTGTATTTTGTTTTCCTTTAGGTCTCGTTTGATTTGCATCTTGTTT

AATTTAAGCTATAAACTTTTTGTAATAAACTAGGATTAGATACCCTATTATTAAAAATAAATATTAAAATGCTAAAGTAG

>Gv10-E-08

AATTTTATCTACTTTATTATTAAAAAGAGGGGCAGCTCCTTTTCATTTTTGATTCCCAGGAGTTATAGAAGGATTAAATT

AATTAATCATATTGGTTGAATATTAATAGCTATAATAAATAACGAACTTTTATGACTAACTTATTTTTTATTATATTCAATT

CAACAAATCATAAAGATATTGGAACTTTATATTTCATTTTTGGAGTATGATCCGGAATAGTCGGAACTTCTCTAAGAATT

AATTGGATTATTGGGATTTATTGTTTGAGCTCATCATATATTTACAGTAGGTATAGACGTAGATACTCGAGCTTATTTTACTTCAGCAACTATAATT

CATGATACTTATTATGTAGTTGCCCATTTTCATTATGTTTTATCTATAGGAGCTGTATTTGCTATTATAGCAGGATTTATTCATTGATACCCTTTATTAACAGGAATAGTTATAAACCCTTCATG

CTGGAATACCTCGACGATACTCAGATTTTCCCGATAGTTACTTAACTTGAAATATTATTTCTTCTTTAGGAAGAACAATT

AATTTGTCACACACAACATATATTTGCTCATTTAGTTCCTCAAGGAACTCCCCCTGTTTTAATACCTTTTATAGTATGCATTGAAACTATCAGTAATGTAATCCGACCAGGAACTTTAGCAGTACGATTAACTGCTAATATAATT

GACGAGATATTTCTCGAGAAGGAACTTTTCAAGGACTTCACACTATTCCCGTAACATTAGGATTACGATGAGGAATAATTTTATTTATTATTTCTGAAGTTTTTTTCTTTATTTCCTTCTTTTGAGCTTTTTTCCATAGTAGCTTATCCCCAACAATCGAATT

AATTGTCATTTTTCTAGAAGTCATCATTTTGGATTTGAAGCAGCTGCTTGATATTGACATTTTGTTGATGTAGTTTGACTATTCTTATATATTTCAATT

AATTTTAGGAATATATTCTTATAAAATGTTTGATCAAGGATGAAGTGAATATTTTGGAGGTCAGATATTATATAATCAAT

ATTATTCAATAACAGGGGATTTTAATAGAACTACATTGAATATACTAAATGATAAGGGGTGAACTATATCTTTTAGAATT

AATTCCTTTTTCTTCTTGATTACCTGCAGCTATGGCAGCTCCGACTCCTGTTTCTGCTTTAGTACATTCTTCTACATTAG

AATTTATTTTCAAAATGTAAAATCTTATAATGCTGGTATATTAACAGCTTTATCTAATCGAATCGGAGATGTTGCCTTAT

CTTTACATTCAATATCTATTGTAATAACTTTTTTATTTGATTGAATAAGTTTAATATTTATATCTTTTGTTTTATTAATT

AATTTTTATTAGATTTACTTTATTTATTTTGAGATTAAAGTTTTTATTAATAGATTTAGTATATTTTATTGAATGAGAAATT

GAGGGTATCAACCTGAACGTTTACAGGCGGGGATTTATTTATTATTTTATACTTTACTGGCTTCGTTGCCGTTATTAATT

AATTATGAAAGTTACTTTAGTATATTTTTTTTAACTTTTTGTGTTTGTGAAGGGGTTCTAGGGCTATCTATTTTAGTTTC

AATTATTATATTTATTTTTGGAAGTATTGTTTTTATTTCTAGTCGTAAGCATTTACTTTGTACTTTATTGAGATTAGAAT

AATTTTTATAGGAGGAATATTAGTTTTATTCATTTATGTTACTTCTCTTTCATCTAATGAAATATTTTCATTATCTATAAAACTATTTTTTTTATCTTTAAGTATAATT

AATTTTAACAGGATTATTCTTAGCAATACACTATACTGCTGATATTGAAACAGCTTTTAATAGAGTAAATCACATTTATCGTGATGTTAATAATGGTTGATTCCTACGAATT

CTGTTGATAATGCTACTTTAACTCGATTCTTTACATTTCATTTTATTTTACCTTTCATTGTATTAGCTTTAACTATAATT

CTTTTGATTTTGCTGAAGGGGAGTCTGAGTTAGTTTCAGGATTTAATGTAGAATATAGAAGAGGGGGATTTGCTTTAATT

AATTTTTTTATATATCAGAAATACATATGATTTATTGTATTTTGTTTTCCTTTAGGTCTCGTTTGATTTGCATCTTGTTT

AATTTAAGCTATAAACTTTTTGTAATAAACTAGGATTAGATACCCTATTATTAAAAATAAATATTAAAATGCTAAAGTAG

>Gv10-E-09

AATTTTATCTACTTTATTATTAAAAAGAGGGGCAGCTCCTTTTCATTTTTGATTCCCAGGAGTTATAGAAGGATTAAATT

AATTAATCATATTGGTTGAATATTAATAGCTATAATAAATAACGAACTTTTATGACTAACTTATTTTTTATTATATTCAATT

CAACAAATCATAAAGATATTGGAACTTTATATTTCATTTTTGGAGTATGATCCGGAATAGTCGGAACTTCTCTAAGAATT

AATTGGATTATTGGGATTTATTGTTTGAGCTCATCATATATTTACAGTAGGTATAGACGTAGATACTCGAGCTTATTTTACTTCAGCAACTATAATT

CATGATACTTATTATGTAGTTGCCCATTTTCATTATGTTTTATCTATAGGAGCTGTATTTGCTATTATAGCAGGATTTATTCATTGATACCCTTTATTAACAGGAATAGTTATAAACCCTTCATG

NNNNNNNNNNNNNNNNNNNNNNNNNNNNNNNNNNNNNNNNNNNNNNNNNNNNNNNNNNNNNNNNNNNNNNNNNNNNNNNN

AATTTGTCACACACAACATATATTTGCTCATTTAGTTCCTCAAGGAACTCCCCCTGTTTTAATACCTTTTATAGTATGCATTGAAACTATCAGTAATGTAATCCGACCAGGAACTTTAGCAGTACGATTAACTGCTAATATAATT

GACGAGATATTTCTCGAGAAGGAACTTTTCAAGGACTTCACACTATTCCCGTAACATTAGGATTACGATGAGGAATAATTTTATTTATTATTTCTGAAGTTTTTTTCTTTATTTCCTTCTTTTGAGCTTTTTTCCATAGTAGCTTATCCCCAACAATCGAATT

AATTGTCATTTTTCTAGAAGTCATCATTTTGGATTTGAAGCAGCTGCTTGATATTGACATTTTGTTGATGTAGTTTGACTATTCTTATATATTTCAATT

AATTTTAGGAATATATTCTTATAAAATGTTTGATCAAGGATGAAGTGAATATTTTGGAGGTCAGATATTATATAATCAAT

ATTATTCAATAACAGGGGATTTTAATAGAACTACATTGAATATACTAAATGATAAGGGGTGAACTATATCTTTTAGAATT

AATTCCTTTTTCTTCTTGATTACCTGCAGCTATGGCAGCTCCGACTCCTGTTTCTGCTTTAGTACATTCTTCTACATTAG

AATTTATTTTCAAAATGTAAAATCTTATAATGCTGGTATATTAACAGCTTTATCTAATCGAATCGGAGATGTTGCCTTAT

CTTTACATTCAATATCTATTGTAATAACTTTTTTATTTGATTGAATAAGTTTAATATTTATATCTTTTGTTTTATTAATT

AATTTTTATTAGATTTACTTTATTTATTTTGAGATTAAAGTTTTTATTAATAGATTTAGTATATTTTATTGAATGAGAAATT

GAGGGTATCAACCTGAACGTTTACAGGCGGGGATTTATTTATTATTTTATACTTTACTGGCTTCGTTGCCGTTATTAATT

AATTATGAAAGTTACTTTAGTATATTTTTTTTAACTTTTTGTGTTTGTGAAGGGGTTCTAGGGCTATCTATTTTAGTTTC

AATTATTATATTTATTTTTGGAAGTATTGTTTTTATTTCTAGTCGTAAGCATTTACTTTGTACTTTATTGAGATTAGAAT

AATTTTTATAGGAGGAATATTAGTTTTATTCATTTATGTTACTTCTCTTTCATCTAATGAAATATTTTCATTATCTATAAAACTATTTTTTTTATCTTTAAGTATAATT

AATTTTAACAGGATTATTCTTAGCAATACACTATACTGCTGATATTGAAACAGCTTTTAATAGAGTAAATCACATTTATCGTGATGTTAATAATGGTTGATTCCTACGAATT

CTGTTGATAATGCTACTTTAACTCGATTCTTTACATTTCATTTTATTTTACCTTTCATTGTATTAGCTTTAACTATAATT

CTTTTGATTTTGCTGAAGGGGAGTCTGAGTTAGTTTCAGGATTTAATGTAGAATATAGAAGAGGGGGATTTGCTTTAATT

AATTTTTTTATATATCAGAAATACATATGATTTATTGTATTTTGTTTTCCTTTAGGTCTCGTTTGATTTGCATCTTGTTT

AATTTAAGCTATAAACTTTTTGTAATAAACTAGGATTAGATACCCTATTATTAAAAATAAATATTAAAATGCTAAAGTAG

>Gv10-F-01

AATTTTATCTACTTTATTATTAAAAAGAGGGGCAGCTCCTTTTCATTTTTGATTCCCAGGAGTTATAGAAGGATTAAATT

AATTAATCATATTGGTTGAATATTAATAGCTATAATAAATAACGAACTTTTATGACTAACTTATTTTTTATTATATTCAATT

CAACAAATCATAAAGATATTGGAACTTTATATTTCATTTTTGGAGTATGATCCGGAATAGTCGGAACTTCTCTAAGAATT

AATTGGATTATTGGGATTTATTGTTTGAGCTCATCATATATTTACAGTAGGTATAGACGTAGATACTCGAGCTTATTTTACTTCAGCAACTATAATT

CATGATACTTATTATGTAGTTGCCCATTTTCATTATGTTTTATCTATAGGAGCTGTATTTGCTATTATAGCAGGATTTATTCATTGATACCCTTTATTAACAGGAATAGTTATAAACCCTTCATG

CTGGAATACCTCGACGATACTCAGATTTTCCCGATAGTTACTTAACTTGAAATATTATTTCTTCTTTAGGAAGAACAATT

AATTTGTCACACACAACATATATTTGCTCATTTAGTTCCTCAAGGAACTCCCCCTGTTTTAATACCTTTTATAGTATGCATTGAAACTATCAGTAATGTAATCCGACCAGGAACTTTAGCAGTACGATTAACTGCTAATATAATT

GACGAGATATTTCTCGAGAAGGAACTTTTCAAGGACTTCACACTATTCCCGTAACATTAGGATTACGATGAGGAATAATTTTATTTATTATTTCTGAAGTTTTTTTCTTTATTTCCTTCTTTTGAGCTTTTTTCCATAGTAGCTTATCCCCAACAATCGAATT

AATTGTCATTTTTCTAGAAGTCATCATTTTGGATTTGAAGCAGCTGCTTGATATTGACATTTTGTTGATGTAGTTTGACTATTCTTATATATTTCAATT

AATTTTAGGAATATATTCTTATAAAATGTTTGATCAAGGATGAAGTGAATATTTTGGAGGTCAGATATTATATAATCAAT

ATTATTCAATAACAGGGGATTTTAATAGAACTACATTGAATATACTAAATGATAAGGGGTGAACTATATCTTTTAGAATT

AATTCCTTTTTCTTCTTGATTACCTGCAGCTATGGCAGCTCCGACTCCTGTTTCTGCTTTAGTACATTCTTCTACATTAG

AATTTATTTTCAAAATGTAAAATCTTATAATGCTGGTATATTAACAGCTTTATCTAATCGAATCGGAGATGTTGCCTTAT

CTTTACATTCAATATCTATTGTAATAACTTTTTTATTTGATTGAATAAGTTTAATATTTATATCTTTTGTTTTATTAATT

AATTTTTATTAGATTTACTTTATTTATTTTGAGATTAAAGTTTTTATTAATAGATTTAGTATATTTTATTGAATGAGAAATT

GAGGGTATCAACCTGAACGTTTACAGGCGGGGATTTATTTATTATTTTATACTTTACTGGCTTCGTTGCCGTTATTAATT

AATTATGAAAGTTACTTTAGTATATTTTTTTTAACTTTTTGTGTTTGTGAAGGGGTTCTAGGGCTATCTATTTTAGTTTC

AATTATTATATTTATTTTTGGAAGTATTGTTTTTATTTCTAGTCGTAAGCATTTACTTTGTACTTTATTGAGATTAGAAT

AATTTTTATAGGAGGAATATTAGTTTTATTCATTTATGTTACTTCTCTTTCATCTAATGAAATATTTTCATTATCTATAAAACTATTTTTTTTATCTTTAAGTATAATT

AATTTTAACAGGATTATTCTTAGCAATACACTATACTGCTGATATTGAAACAGCTTTTAATAGAGTAAATCACATTTATCGTGATGTTAATAATGGTTGATTCCTACGAATT

CTGTTGATAATGCTACTTTAACTCGATTCTTTACATTTCATTTTATTTTACCTTTCATTGTATTAGCTTTAACTATAATT

CTTTTGATTTTGCTGAAGGGGAGTCTGAGTTAGTTTCAGGATTTAATGTAGAATATAGAAGAGGGGGATTTGCTTTAATT

AATTTTTTTATATATCAGAAATACATATGATTTATTGTATTTTGTTTTCCTTTAGGTCTCGTTTGATTTGCATCTTGTTT

AATTTAAGCTATAAACTTTTTGTAATAAACTAGGATTAGATACCCTATTATTAAAAATAAATATTAAAATGCTAAAGTAG

>Gv10-F-02

AATTTTATCTACTTTATTATTAAAAAGAGGGGCAGCTCCTTTTCATTTTTGATTCCCAGGAGTTATAGAAGGATTAAATT

AATTAATCATATTGGTTGAATATTAATAGCTATAATAAATAACGAACTTTTATGACTAACTTATTTTTTATTATATTCAANN

CAACAAATCATAAAGATATTGGAACTTTATATTTCATTTTTGGAGTATGATCCGGAATAGTCGGAACTTCTCTAAGAATT

AATTGGATTATTGGGATTTATTGTTTGAGCTCATCATATATTTACAGTAGGTATAGACGTAGATACTCGAGCTTATTTTANNNNNNNNNNNNNNNNN

CATGATACTTATTATGTAGTTGCCCATTTTCATTATGTTTTATCTATAGGAGCTGTATTTGCTATTATAGCAGGATTTATTCATTGATACCCTTTATTAACAGGAATAGTTATAAACCCTTCATG

CTGGAATACCTCGACGATACTCAGATTTTCCCGATAGTTACTTAACTTGAAATATTATTTCTTCTTTAGGAAGAACAATT

AATTTGTCACACACAACATATATTTGCTCATTTAGTTCCTCAAGGAACTCCCCCTGTTTTAATACCTTTTATAGTATGCATTGAAACTATCAGTAATGTAATCCGACCAGGAACTTTAGCAGTACGATTAACTGCTAATATAATT

GACGAGATATTTCTCGAGAAGGAACTTTTCAAGGACTTCACACTATTCCCGTAACATTAGGATTACGATGAGGAATAATTTTATTTATTATTTCTGAAGTTTTTTTCTTTATTTCCTTCTTTTGAGCTTTTTTCCATAGTAGCTTATCCCCAACAATCGAATT

AATTGTCATTTTTCTAGAAGTCATCATTTTGGATTTGAAGCAGCTGCTTGATATTGACATTTTGTTGATGTAGTTTGACTATTCTTATATATTTCAATT

AATTTTAGGAATATATTCTTATAAAATGTTTGATCAAGGATGAAGTGAATATTTTGGAGGTCAGATATTATATAATCAAT

ATTATTCAATAACAGGGGATTTTAATAGAACTACATTGAATATACTAAATGATAAGGGGTGAACTATATCTTTTAGAATT

AATTCCTTTTTCTTCTTGATTACCTGCAGCTATGGCAGCTCCGACTCCTGTTTCTGCTTTAGTACATTCTTCTACATTAG

AATTTATTTTCAAAATGTAAAATCTTATAATGCTGGTATATTAACAGCTTTATCTAATCGAATCGGAGATGTTGCCTTAT

CTTTACATTCAATATCTATTGTAATAACTTTTTTATTTGATTGAATAAGTTTAATATTTATATCTTTTGTTTTATTAATT

AATTTTTATTAGATTTACTTTATTTATTTTGAGATTAAAGTTTTTATTAATAGATTTAGTATATTTTATTGAATGAGAAATT

GAGGGTATCAACCTGAACGTTTACAGGCGGGGATTTATTTATTATTTTATACTTTACTGGCTTCGTTGCCGTTATTAATT

AATTATGAAAGTTACTTTAGTATATTTTTTTTAACTTTTTGTGTTTGTGAAGGGGTTCTAGGGCTATCTATTTTAGTTTC

AATTATTATATTTATTTTTGGAAGTATTGTTTTTATTTCTAGTCGTAAGCATTTACTTTGTACTTTATTGAGATTAGAAT

AATTTTTATAGGAGGAATATTAGTTTTATTCATTTATGTTACTTCTCTTTCATCTAATGAAATATTTTCATTATCTATAAAACTATTTTTTTTATCTTTAAGTATAATT

AATTTTAACAGGATTATTCTTAGCAATACACTATACTGCTGATATTGAAACAGCTTTTAATAGAGTAAATCACATTTATCGTGATGTTAATAATGGTTGATTCCTACGAATT

CTGTTGATAATGCTACTTTAACTCGATTCTTTACATTTCATTTTATTTTACCTTTCATTGTATTAGCTTTAACTATAATT

CTTTTGATTTTGCTGAAGGGGAGTCTGAGTTAGTTTCAGGATTTAATGTAGAATATAGAAGAGGGGGATTTGCTTTAATT

AATTTTTTTATATATCAGAAATACATATGATTTATTGTATTTTGTTTTCCTTTAGGTCTCGTTTGATTTGCATCTTGTTT

AATTTAAGCTATAAACTTTTTGTAATAAACTAGGATTAGATACCCTATTATTAAAAATAAATATTAAAATGCTAAAGTAG

>Gv10-F-08

AATTTTATCTACTTTATTATTAAAAAGAGGGGCAGCTCCTTTTCATTTTTGATTCCCAGGAGTTATAGAAGGATTAAATT

AATTAATCATATTGGTTGAATATTAATAGCTATAATAAATAACGAACTTTTATGACTAACTTATTTTTTATTATATTCAATT

CAACAAATCATAAAGATATTGGAACTTTATATTTCATTTTTGGAGTATGATCCGGAATAGTCGGAACTTCTCTAAGAATT

AATTGGATTATTGGGATTTATTGTTTGAGCTCATCATATATTTACAGTAGGTATAGACGTAGATACTCGAGCTTATTTTACTTCAGCAACTATAATT

CATGATACTTATTATGTAGTTGCCCATTTTCATTATGTTTTATCTATAGGAGCTGTATTTGCTATTATAGCAGGATTTATTCATTGATACCCTTTATTAACAGGAATAGTTATAAACCCTTCATG

CTGGAATACCTCGACGATACTCAGATTTTCCTGATAGTTACTTAACTTGAAATATTATTTCTTCTTTAGGAAGAACAATT

AATTTGTCACACACAACATATATTTGCTCATTTAGTTCCTCAAGGAACTCCCCCTGTTTTAATACCTTTTATAGTATGCATTGAAACTATCAGTAATGTAATCCGACCAGGAACTTTAGCAGTACGATTAACTGCTAATATAATT

GACGAGATATTTCTCGAGAAGGAACTTTTCAAGGACTTCACACTATTCCCGTAACATTAGGATTACGATGAGGAATAATTTTATTTATTATTTCTGAAGTTTTTTTCTTTATTTCCTTCTTTTGAGCTTTTTTCCATAGTAGCTTATCCCCAACAATCGAATT

AATTGTCATTTTTCTAGAAGTCATCATTTTGGATTTGAAGCAGCTGCTTGATATTGACATTTTGTTGATGTAGTTTGACTATTCTTATATATTTCAATT

AATTTTAGGAATATATTCTTATAAAATGTTTGATCAAGGATGAAGTGAATATTTTGGAGGTCAGATATTATATAATCAAT

ATTATTCAATAACAGGGGATTTTAATAGAACTACATTGAATATACTAAATGATAAGGGGTGAACTATATCTTTTAGAATT

AATTCCTTTTTCTTCTTGATTACCTGCAGCTATGGCAGCTCCGACTCCTGTTTCTGCTTTAGTACATTCTTCTACATTAG

AATTTATTTTCAAAATGTAAAATCTTATAATGCTGGTATATTAACAGCTTTATCTAATCGAATCGGAGATGTTGCCTTAT

CTTTACATTCAATATCTATTGTAATAACTTTTTTATTTGATTGAATAAGTTTAATATTTATATCTTTTGTTTTATTAATT

AATTTTTATTAGATTTACTTTATTTATTTTGAGATTAAAGTTTTTATTAATAGATTTAGTATATTTTATTGAATGAGAAATT

GAGGGTATCAACCTGAACGTTTACAGGCGGGGATTTATTTATTATTTTATACTTTACTGGCTTCGTTGCCGTTATTAATT

AATTATGAAAGTTACTTTAGTATATTTTTTTTAACTTTTTGTGTTTGTGAAGGGGTTCTAGGGCTATCTATTTTAGTTTC

AATTATTATATTTATTTTTGGAAGTATTGTTTTTATTTCTAGTCGTAAGCATTTACTTTGTACTTTATTGAGATTAGAAT

AATTTTTATAGGAGGAATATTAGTTTTATTCATTTATGTTACTTCTCTTTCATCTAATGAAATATTTTCATTATCTATAAAACTATTTTTTTTATCTTTAAGTATAATT

AATTTTAACAGGATTATTCTTAGCAATACACTATACTGCTGATATTGAAACAGCTTTTAATAGAGTAAATCACATTTATCGTGATGTTAATAATGGTTGATTCCTACGAATT

CTGTTGATAATGCTACTTTAACTCGATTCTTTACATTTCATTTTATTTTACCTTTCATTGTATTAGCTTTAACTATAATT

CTTTTGATTTTGCTGAAGGGGAGTCTGAGTTAGTTTCAGGATTTAATGTAGAATATAGAAGAGGGGGATTTGCTTTAATT

AATTTTTTTATATATCAGAAATACATATGATTTATTGTATTTTGTTTTCCTTTAGGACTCGTTTGATTTGCATCTTGTTT

AATTTAAGCTATAAACTTTTTGTAATAAACTAGGATTAGATACCCTATTATTAAAAATAAATATTAAAATGCTAAAGTAG

>Gv10-F-10

AATTTTATCTACTTTATTATTAAAAAGAGGGGCAGCTCCTTTTCATTTTTGATTCCCAGGGGTTATAGAAGGATTAAATT

AATTAATCATATTGGTTGAATATTAATAGCTATAATAAATAACGAACTTTTATGATTAACTTATTTTTTATTATATTCAATT

CAACAAATCATAAAGATATTGGAACTTTATATTTCATTTTTGGAGTATGATCTGGAATAGTCGGAACTTCTCTAAGAATT

AATTGGATTATTGGGATTTATTGTTTGAGCTCATCATATATTTACAGTAGGTATAGATGTAGATACTCGAGCTTATTTTACTTCAGCAACTATAATT

CATGATACTTATTACGTAGTTGCCCATTTTCATTACGTTTTATCTATAGGAGCTGTATTTGCTATTATAGCAGGATTTATTCATTGATACCCTTTATTAACAGGAATAGTTATAAACCCTTCATG

CTGGAATACCTCGACGATACTCAGATTTTCCTGATAGTTACTTAACTTGAAATATTATTTCTTCTTTAGGAAGAACAATT

AATTTGTCACACACAACATATATTTGCTCATTTAGTTCCTCAAGGGACTCCTCCTGTTTTAATACCTTTTATAGTATGCATTGAAACTATTAGTAATGTAATCCGACCAGGAACTTTAGCAGTACGATTGACTGCTAATATAATT

GACGAGATATTTCTCGAGAAGGAACTTTTCAAGGACTTCACACTATTCCCGTAACATTAGGATTACGATGAGGAATAATTTTATTTATTATTTCTGAAGTTTTTTTCTTTATTTCCTTCTTTTGAGCTTTTTTCCATAGTAGCTTATCTCCAACAATCGAATT

AATTGTCATTTTTCTAGAAGTCATCATTTTGGATTCGAAGCAGCTGCTTGATATTGACATTTTGTTGATGTAGTTTGACTATTCTTATATATTTCAATT

AATTTTAGGAATATATTCTTATAAAATGTTTGATCAAGGATGAAGTGAATATTTTGGAGGTCAGATATTATATAATCAAT

ATTATTCAATAACAGGGGATTTTAATAGAACTACATTGAATATGTTAAATGATAAGGGGTGAACTATATCTTTTAGAATT

AATTCCTTTTTCTTCTTGATTACCTGCAGCTATGGCAGCACCGACTCCTGTTTCTGCTTTAGTACATTCTTCTACATTAG

NNNNNNNNNNNNNNNNNNNNNNNNNNNNNNNNNNNNNNNNNNNNNNNNNNNNNNNNNNNNNNNNNNNNNNNNNNNNNNNN

CTTTACATTCAATATCTATTGTAATAACTTTTTTATTTGATTGAATAAGTTTAATATTTATATCTTTTGTTTTATTAATT

AATTTTTATTAGATTTACTTTATTTATTTTAAGATTAAAGTTTTTATTAATAGATTTAGTATATTTTATTGAATGAGAAATT

GAGGATATCAACCTGAACGTTTACAGGCGGGGATTTATTTATTATTTTATACTTTACTGGCTTCGTTGCCGTTATTAATT

AATTATGAAAGTTACTTTAGTATATTTTTTTTAACTTTTTGTGTTTGTGAAGGGGTTCTAGGGCTATCTATTTTAGTTTC

AATTATTATATTTATTTTTGGAAGTATTGTTTTTATTTCTAGTCGTAAGCATTTACTTTGTACTTTATTGAGATTAGAGT

AATTTTTATAGGAGGAATATTAGTTTTATTCATTTATGTAACTTCTCTTTCATCTAATGAAATATTTTCATTATCTATAAAACTATTTTTTTTATCTTTAAGTATAATT

AATTTTAACAGGATTATTCTTAGCAATACACTATACTGCTGATATTGAAACAGCTTTTAATAGAGTAAATCACATTTATCGTGATGTTAATAATGGTTGATTCCTACGAATT

CTGTTGATAATGCTACTTTAACTCGATTCTTTACCTTTCATTTTATTCTCCCTTTCATTGTATTAGCTTTAACTATAATT

CTTTTGATTTTGCTGAAGGGGAGTCTGAGTTAGTTTCAGGATTTAATGTAGAGTATAGAAGAGGGGGATTTGCTTTAATT

AATTTTTTTATATATCAGAAATATATATGATTTATTGTATTTTGTTTTCCTTTAGGTCTCGTTTGATTTGCATCTTGTTT

AATTTAAGCTATAAACTTTTTGTAATAAACTAGGATTAGATACCCTATTATTAAAAATAAATATTAAAATGCTAAAGTAG

>Gv10-G-05

AATTTTATCTACTTTATTATTAAAAAGAGGGGCAGCTCCTTTTCATTTTTGATTCCCAGGAGTTATAGAAGGATTAAATT

AATTAATCATATTGGTTGAATATTAATAGCTATAATAAATAACGAACTTTTATGACTAACTTATTTTTTATTATATTCAATT

CAACAAATCATAAAGATATTGGAACTTTATATTTCATTTTTGGAGTATGATCCGGAATAGTCGGAACTTCTCTAAGAATT

AATTGGATTATTGGGATTTATTGTTTGAGCTCATCATATATTTACAGTAGGTATAGACGTAGATACTCGAGCTTATTTTACTTCAGCAACTATAATT

CATGATACTTATTATGTAGTTGCCCATTTTCATTATGTTTTATCTATAGGAGCTGTATTTGCTATTATAGCAGGATTTATTCATTGATACCCTTTATTAACAGGAATAGTTATAAACCCTTCATG

CTGGAATACCTCGACGATACTCAGATTTTCCTGATAGTTACTTAACTTGAAATATTATTTCTTCTTTAGGAAGAACAATT

AATTTGTCACACACAACATATATTTGCTCATTTAGTTCCTCAAGGAACTCCCCCTGTTTTAATACCTTTTATAGTATGCATTGAAACTATCAGTAATGTAATCCGACCAGGAACTTTAGCAGTACGATTAACTGCTAATATAATT

GACGAGATATTTCTCGAGAAGGAACTTTTCAAGGACTTCACACTATTCCCGTAACATTAGGATTACGATGAGGAATAATTTTATTTATTATTTCTGAAGTTTTTTTCTTTATTTCCTTCTTTTGAGCTTTTTTCCATAGTAGCTTATCCCCAACAATCGAATT

AATTGTCATTTTTCTAGAAGTCATCATTTTGGATTTGAAGCAGCTGCTTGATATTGACATTTTGTTGATGTAGTTTGACTATTCTTATATATTTCAATT

AATTTTAGGAATATATTCTTATAAAATGTTTGATCAAGGATGAAGTGAATATTTTGGAGGTCAGATATTATATAATCAAT

ATTATTCAATAACAGGGGATTTTAATAGAACTACATTGAATATACTAAATGATAAGGGGTGAACTATATCTTTTAGAATT

AATTCCTTTTTCTTCTTGATTACCTGCAGCTATGGCAGCTCCGACTCCTGTTTCTGCTTTAGTACATTCTTCTACATTAG

AATTTATTTTCAAAATGTAAAATCTTATAATGCTGGTATATTAACAGCTTTATCTAATCGAATCGGAGATGTTGCCTTAT

CTTTACATTCAATATCTATTGTAATAACTTTTTTATTTGATTGAATAAGTTTAATATTTATATCTTTTGTTTTATTAATT

AATTTTTATTAGATTTACTTTATTTATTTTGAGATTAAAGTTTTTATTAATAGATTTAGTATATTTTATTGAATGAGAAATT

GAGGGTATCAACCTGAACGTTTACAGGCGGGGATTTATTTATTATTTTATACTTTACTGGCTTCGTTGCCGTTATTAATT

AATTATGAAAGTTACTTTAGTATATTTTTTTTAACTTTTTGTGTTTGTGAAGGGGTTCTAGGGCTATCTATTTTAGTTTC

AATTATTATATTTATTTTTGGAAGTATTGTTTTTATTTCTAGTCGTAAGCATTTACTTTGTACTTTATTGAGATTAGAAT

AATTTTTATAGGAGGAATATTAGTTTTATTCATTTATGTTACTTCTCTTTCATCTAATGAAATATTTTCATTATCTATAAAACTATTTTTTTTATCTTTAAGTATAATT

AATTTTAACAGGATTATTCTTAGCAATACACTATACTGCTGATATTGAAACAGCTTTTAATAGAGTAAATCACATTTATCGTGATGTTAATAATGGTTGATTCCTACGAATT

CTGTTGATAATGCTACTTTAACTCGATTCTTTACATTTCATTTTATTTTACCTTTCATTGTATTAGCTTTAACTATAATT

CTTTTGATTTTGCTGAAGGGGAGTCTGAGTTAGTTTCAGGATTTAATGTAGAATATAGAAGAGGGGGATTTGCTTTAATT

AATTTTTTTATATATCAGAAATACATATGATTTATTGTATTTTGTTTTCCTTTAGGTCTCGTTTGATTTGCATCTTGTTT

AATTTAAGCTATAAACTTTTTGTAATAAACTAGGATTAGATACCCTATTATTAAAAATAAATATTAAAATGCTAAAGTAG

>Gv10-G-06

AATTTTATCTACTTTATTATTAAAAAGAGGGGCAGCTCCTTTTCATTTTTGATTCCCAGGAGTTATAGAAGGATTAAATT

AATTAATCATATTGGTTGAATATTAATAGCTATAATAAATAACGAACTTTTATGACTAACTTATTTTTTATTATATTCAATT

CAACAAATCATAAAGATATTGGAACTTTATATTTCATTTTTGGAGTATGATCCGGAATAGTCGGAACTTCTCTAAGAATT

AATTGGATTATTGGGATTTATTGTTTGAGCTCATCATATATTTACAGTAGGTATAGACGTAGATACTCGAGCTTATTTTACTTCAGCAACTATAATT

CATGATACTTATTATGTAGTTGCCCATTTTCATTATGTTTTATCTATAGGAGCTGTATTTGCTATTATAGCAGGATTTATTCATTGATACCCTTTATTAACAGGAATAGTTATAAACCCTTCATG

NNNNNNNNNNNNNNNNNNNNNNNNNNNNNNNNNNNNNNNNNNNNNNNNNNNNNNNNNNNNNNNNNNNNNNNNNNNNNNNN

AATTTGTCACACACAACATATATTTGCTCATTTAGTTCCTCAAGGAACTCCCCCTGTTTTAATACCTTTTATAGTATGCATTGAAACTATCAGTAATGTAATCCGACCAGGAACTTTAGCAGTACGATTAACTGCTAATATAATT

GACGAGATATTTCTCGAGAAGGAACTTTTCAAGGACTTCACACTATTCCCGTAACATTAGGATTACGATGAGGAATAATTTTATTTATTATTTCTGAAGTTTTTTTCTTTATTTCCTTCTTTTGAGCTTTTTTCCATAGTAGCTTATCCCCAACAATCGAATT

AATTGTCATTTTTCTAGAAGTCATCATTTTGGATTTGAAGCAGCTGCTTGATATTGACATTTTGTTGATGTAGTTTGACTATTCTTATATATTTCAATT

AATTTTAGGAATATATTCTTATAAAATGTTTGATCAAGGATGAAGTGAATATTTTGGAGGTCAGATATTATATAATCAAT

ATTATTCAATAACAGGGGATTTTAATAGAACTACATTGAATATACTAAATGATAAGGGGTGAACTATATCTTTTAGAATT

AATTCCTTTTTCTTCTTGATTACCTGCAGCTATGGCAGCTCCGACTCCTGTTTCTGCTTTAGTACATTCTTCTACATTAG

AATTTATTTTCAAAATGTAAAATCTTATAATGCTGGTATATTAACAGCTTTATCTAATCGAATCGGAGATGTTGCCTTAT

CTTTACATTCAATATCTATTGTAATAACTTTTTTATTTGATTGAATAAGTTTAATATTTATATCTTTTGTTTTATTAATT

AATTTTTATTAGATTTACTTTATTTATTTTGAGATTAAAGTTTTTATTAATAGATTTAGTATATTTTATTGAATGAGAAATT

GAGGGTATCAACCTGAACGTTTACAGGCGGGGATTTATTTATTATTTTATACTTTACTGGCTTCGTTGCCGTTATTAATT

AATTATGAAAGTTACTTTAGTATATTTTTTTTAACTTTTTGTGTTTGTGAAGGGGTTCTAGGGCTATCTATTTTAGTTTC

AATTATTATATTTATTTTTGGAAGTATTGTTTTTATTTCTAGTCGTAAGCATTTACTTTGTACTTTATTGAGATTAGAAT

AATTTTTATAGGAGGAATATTAGTTTTATTCATTTATGTTACTTCTCTTTCATCTAATGAAATATTTTCATTATCTATAAAACTATTTTTTTTATCTTTAAGTATAATT

AATTTTAACAGGATTATTCTTAGCAATACACTATACTGCTGATATTGAAACAGCTTTTAATAGAGTAAATCACATTTATCGTGATGTTAATAATGGTTGATTCCTACGAATT

CTGTTGATAATGCTACTTTAACTCGATTCTTTACATTTCATTTTATTTTACCTTTCATTGTATTAGCTTTAACTATAATT

CTTTTGATTTTGCTGAAGGGGAGTCTGAGTTAGTTTCAGGATTTAATGTAGAATATAGAAGAGGGGGATTTGCTTTAATT

AATTTTTTTATATATCAGAAATACATATGATTTATTGTATTTTGTTTTCCTTTAGGTCTCGTTTGATTTGCATCTTGTTT

AATTTAAGCTATAAACTTTTTGTAATAAACTAGGATTAGATACCCTATTATTAAAAATAAATATTAAAATGCTAAAGTAG

>Gv10-G-07

AATTTTATCTACTTTATTATTAAAAAGAGGGGCAGCTCCTTTTCATTTTTGATTCCCAGGAGTTATAGAAGGATTAAATT

AATTAATCATATTGGTTGAATATTAATAGCTATAATAAATAACGAACTTTTATGACTAACTTATTTTTTATTATATTCGATT

CAACAAATCATAAAGATATTGGAACTTTATATTTCATTTTTGGAGTATGATCCGGAATAGTCGGAACTTCTCTAAGAATT

AATTGGATTATTGGGATTTATTGTTTGAGCTCATCATATATTTACAGTAGGTATAGACGTAGATACTCGAGCTTATTTTACTTCAGCAACTATAATT

CATGATACTTATTATGTAGTTGCCCATTTTCATTATGTTTTATCTATAGGAGCTGTATTTGCTATTATAGCAGGATTTATTCATTGATACCCTTTATTAACAGGAATAGTTATAAACCCTTCATG

CTGGAATACCTCGACGATACTCAGATTTTCCCGATAGTTACTTAACTTGAAATATTATTTCTTCTTTAGGAAGAACAATT

AATTTGTCACACACAACATATATTTGCTCATTTAGTTCCTCAAGGAACTCCCCCTGTTTTAATACCTTTTATAGTATGCATTGAAACTATCAGTAATGTAATCCGACCAGGAACTTTAGCAGTACGATTAACTGCTAATATAATT

GACGAGATATTTCTCGAGAAGGAACTTTTCAAGGACTTCACACTATTCCCGTAACATTAGGATTACGATGAGGAATAATTTTATTTATTATTTCTGAAGTTTTTTTCTTTATTTCCTTCTTTTGAGCTTTTTTCCATAGTAGCTTATCCCCAACAATCGAATT

AATTGTCATTTTTCTAGAAGTCATCATTTTGGATTTGAAGCAGCTGCTTGATATTGACATTTTGTTGATGTAGTTTGACTATTCTTATATATTTCAATT

AATTTTAGGAATATATTCTTATAAAATGTTTGATCAAGGATGAAGTGAATATTTTGGAGGTCAGATATTATATAATCAAT

ATTATTCAATAACAGGGGATTTTAATAGAACTACATTGAATATACTAAATGATAAGGGGTGAACTATATCTTTTAGAATT

AATTCCTTTTTCTTCTTGATTACCTGCAGCTATGGCAGCTCCGACTCCTGTTTCTGCTTTAGTACATTCTTCTACATTAG

AATTTATTTTCAAAATGTAAAATCTTATAATGCTGGTATATTAACAGCTTTATCTAATCGAATCGGAGATGTTGCCTTAT

CTTTACATTCAATATCTATTGTAATAACTTTTTTATTTGATTGAATAAGTTTAATATTTATATCTTTTGTTTTATTAATT

AATTTTTATTAGATTTACTTTATTTATTTTGAGATTAAAGTTTTTATTAATAGATTTAGTATATTTTATTGAATGAGAAATT

GAGGGTATCAACCTGAACGTTTACAGGCGGGGATTTATTTATTATTTTATACTTTACTGGCTTCGTTGCCGTTATTAATT

AATTATGAAAGTTACTTTAGTATATTTTTTTTAACTTTTTGTGTTTGTGAAGGGGTTCTAGGGCTATCTATTTTAGTTTC

AATTATTATATTTATTTTTGGAAGTATTGTTTTTATTTCTAGTCGTAAGCATTTACTTTGTACTTTATTGAGATTAGAAT

AATTTTTATAGGAGGAATATTAGTTTTATTCATTTATGTTACTTCTCTTTCATCTAATGAAATATTTTCATTATCTATAAAACTATTTTTTTTATCTTTAAGTATAATT

AATTTTAACAGGATTATTCTTAGCAATACACTATACTGCTGATATTGAAACAGCTTTTAATAGAGTAAATCACATTTATCGTGATGTTAATAATGGTTGATTCCTACGAATT

CTGTTGATAATGCTACTTTAACTCGATTCTTTACATTTCATTTTATTTTACCTTTCATTGTATTAGCTTTAACTATAATT

CTTTTGATTTTGCTGAAGGGGAGTCTGAGTTAGTTTCAGGATTTAATGTAGAATATAGAAGAGGGGGATTTGCTTTAATT

AATTTTTTTATATATCAGAAATACATATGATTTATTGTATTTTGTTTTCCTTTAGGTCTCGTTTGATTTGCATCTTGTTT

AATTTAAGCTATAAACTTTTTGTAATAAACTAGGATTAGATACCCTATTATTAAAAATAAATATTAAAATGCTAAAGTAG

>Gv10-G-08

AATTTTATCTACTTTATTATTAAAAAGAGGAGCAGCTCCTTTTCATTTTTGATTCCCAGGAGTTATAGAAGGATTAAATT

AATTAATCATATTGGTTGAATATTAATAGCTATAATAAATAACGAACTTTTATGACTAACTTATTTTTTATTATATTCAATT

CAACAAATCATAAAGATATTGGAACTTTATATTTCATTTTTGGAGTATGATCCGGAATAGTCGGAACTTCTCTAAGAATT

AATTGGATTATTGGGATTTATTGTTTGAGCTCATCATATATTTACAGTAGGTATAGACGTAGATACTCGAGCTTATTTTACTTCAGCAACTATAATT

CATGATACTTATTATGTAGTTGCCCATTTTCATTATGTTTTATCTATAGGAGCTGTATTTGCTATTATAGCAGGATTTATTCATTGATACCCTTTATTAACAGGAATAGTTATAAACCCTTCATG

CTGGAATACCTCGACGATACTCGGATTTTCCCGATAGTTACTTAACTTGAAATATTATTTCTTCTTTAGGAAGAACAATT

AATTTGTCACACACAACATATATTTGCTCATTTAGTTCCTCAAGGAACTCCCCCTGTTTTAATACCTTTTATAGTATGCATTGAAACTATTAGTAATGTAATCCGACCAGGAACTTTAGCAGTACGATTAACTGCTAATATAATT

GACGAGATATTTCTCGAGAAGGAACTTTTCAAGGACTTCACACTATTCCCGTAACATTAGGATTACGATGAGGAATAATTTTATTTATTATTTCTGAAGTTTTTTTCTTTATTTCCTTCTTTTGAGCTTTTTTCCATAGTAGCTTATCCCCAACAATCGAATT

AATTGTCATTTTTCTAGAAGTCATCATTTTGGATTTGAAGCAGCTGCTTGATATTGACATTTTGTTGATGTAGTTTGACTATTCTTATATATTTCAATT

AATTTTAGGAATATATTCTTATAAAATGTTTGATCAAGGATGAAGTGAATATTTTGGAGGTCAGATATTATATAATCAAT

ATTATTCAATAACAGGGGATTTTAATAGAACTACATTGAATATACTAAATGATAAGGGGTGAACTATATCTTTTAGAATT

AATTCCTTTTTCTTCTTGATTACCTGCAGCTATGGCAGCTCCGACTCCTGTTTCTGCTTTAGTACATTCTTCTACATTAG

AATTTATTTTCAAAATGTAAAATCTTATAATGCTGGTATATTAACAGCTTTATCTAATCGAATCGGAGATGTTGCCTTAT

CTTTACATTCAATATCTATTGTAATAACTTTTTTATTTGATTGAATAAGTTTAATATTTATATCTTTTGTTTTATTAATT

AATTTTTATTAGATTTACTTTATTTATTTTGAGATTAAAGTTTTTATTAATAGATTTAGTATATTTTATTGAATGAGAAATT

GAGGGTATCAACCTGAACGTTTACAGGCGGGGATTTATTTATTATTTTATACTTTACTGGCTTCGTTGCCGTTATTAATT

AATTATGAAAGTTACTTTAGTATATTTTTTTTAACTTTTTGTGTTTGTGAAGGGGTTCTAGGGCTATCTATTTTAGTTTC

AATTATTATATTTATTTTTGGAAGTATTGTTTTTATTTCTAGTCGTAAGCATTTACTTTGTACTTTATTGAGATTAGAGT

AATTTTTATAGGAGGAATATTAGTTTTATTCATTTATGTTACTTCTCTTTCATCTAATGAAATATTTTCATTATCTATAAAACTATTTTTTTTATCTTTAAGTATAATT

AATTTTAACAGGATTATTCTTAGCAATACACTATACTGCTGATATTGAAACAGCTTTTAATAGAGTAAATCACATTTATCGTGATGTTAATAATGGTTGATTCCTACGAATT

CTGTTGATAATGCTACTTTAACTCGATTCTTTACATTTCATTTTATTTTACCTTTCATTGTATTAGCTTTAACTATAATT

CTTTTGATTTTGCTGAAGGGGAGTCTGAGTTAGTTTCAGGATTTAATGTAGAATATAGAAGAGGGGGATTTGCTTTAATT

AATTTTTTTATATATCAGAAATACATATGATTTATTGTATTTTGTTTTCCTTTAGGTCTCGTTTGATTTGCATCTTGTTT

AATTTAAGCTATAAACTTTTTGTAATAAACTAGGATTAGATACCCTATTATTAAAAATAAATATTAAAATGCTAAAGTAG

>Gv10-G-09

AATTTTATCTACTTTATTATTAAAAAGAGGGGCAGCTCCTTTTCATTTTTGATTCCCAGGAGTTATAGAAGGATTAAATT

AATTAATCATATTGGTTGAATATTAATAGCTATAATAAATAACGAACTTTTATGACTAACTTATTTTTTATTATATTCAATT

CAACAAATCATAAAGATATTGGAACTTTATATTTCATTTTTGGAGTATGATCCGGAATAGTCGGAACTTCTCTAAGAATT

AATTGGATTATTGGGATTTATTGTTTGAGCTCATCATATATTTACAGTAGGTATAGACGTAGATACTCGAGCTTATTTTACTTCAGCAACTATAATT

CATGATACTTATTATGTAGTTGCCCATTTTCATTATGTTTTATCTATAGGAGCTGTATTTGCTATTATAGCAGGATTTATTCATTGATACCCTTTATTAACAGGAATAGTTATAAACCCTTCATG

NNNNNNNNNNNNNNNNNNNNNNNNNNNNNNNNNNNNNNNNNNNNNNNNNNNNNNNNNNNNNNNNNNNNNNNNNNNNNNNN

AATTTGTCACACACAACATATATTTGCTCATTTAGTTCCTCAAGGAACTCCCCCTGTTTTAATACCTTTTATAGTATGCATTGAAACTATCAGTAATGTAATCCGACCAGGAACTTTAGCAGTACGATTAACTGCTAATATAATT

GACGAGATATTTCTCGAGAAGGAACTTTTCAAGGACTTCACACTATTCCCGTAACATTAGGATTACGATGAGGAATAATTTTATTTATTATTTCTGAAGTTTTTTTCTTTATTTCCTTCTTTTGAGCTTTTTTCCATAGTAGCTTATCCCCAACAATCGAATT

AATTGTCATTTTTCTAGAAGTCATCATTTTGGATTTGAAGCAGCTGCTTGATATTGACATTTTGTTGATGTAGTTTGACTATTCTTATATATTTCAATT

AATTTTAGGAATATATTCTTATAAAATGTTTGATCAAGGATGAAGTGAATATTTTGGAGGTCAGATATTATATAATCAAT

ATTATTCAATAACAGGGGATTTTAATAGAACTACATTGAATATACTAAATGATAAGGGGTGAACTATATCTTTTAGAATT

AATTCCTTTTTCTTCTTGATTACCTGCAGCTATGGCAGCTCCGACTCCTGTTTCTGCTTTAGTACATTCTTCTACATTAG

AATTTATTTTCAAAATGTAAAATCTTATAATGCTGGTATATTAACAGCTTTATCTAATCGAATCGGAGATGTTGCCTTAT

CTTTACATTCAATATCTATTGTAATAACTTTTTTATTTGATTGAATAAGTTTAATATTTATATCTTTTGTTTTATTAATT

AATTTTTATTAGATTTACTTTATTTATTTTGAGATTAAAGTTTTTATTAATAGATTTAGTATATTTTATTGAATGAGAAATT

GAGGGTATCAACCTGAACGTTTACAGGCGGGGATTTATTTATTATTTTATACTTTACTGGCTTCGTTGCCGTTATTAATT

AATTATGAAAGTTACTTTAGTATATTTTTTTTAACTTTTTGTGTTTGTGAAGGGGTTCTAGGGCTATCTATTTTAGTTTC

AATTATTATATTTATTTTTGGAAGTATTGTTTTTATTTCTAGTCGTAAGCATTTACTTTGTACTTTATTGAGATTAGAAT

AATTTTTATAGGAGGAATATTAGTTTTATTCATTTATGTTACTTCTCTTTCATCTAATGAAATATTTTCATTATCTATAAAACTATTTTTTTTATCTTTAAGTATAATT

AATTTTAACAGGATTATTCTTAGCAATACACTATACTGCTGATATTGAAACAGCTTTTAATAGAGTAAATCACATTTATCGTGATGTTAATAATGGTTGATTCCTACGAATT

CTGTTGATAATGCTACTTTAACTCGATTCTTTACATTTCATTTTATTTTACCTTTCATTGTATTAGCTTTAACTATAATT

CTTTTGATTTTGCTGAAGGGGAGTCTGAGTTAGTTTCAGGATTTAATGTAGAATATAGAAGAGGGGGATTTGCTTTAATT

AATTTTTTTATATATCAGAAATACATATGATTTATTGTATTTTGTTTTCCTTTAGGTCTCGTTTGATTTGCATCTTGTTT

AATTTAAGCTATAAACTTTTTGTAATAAACTAGGATTAGATACCCTATTATTAAAAATAAATATTAAAATGCTAAAGTAG

>Gv10-G-10

AATTTTATCTACTTTATTATTAAAAAGAGGGGCAGCTCCTTTTCATTTTTGATTCCCAGGAGTTATAGAAGGATTAAATT

AATTAATCATATTGGTTGAATATTAATAGCTATAATAAATAACGAACTTTTATGACTAACTTATTTTTTATTATATTCAATT

CAACAAATCATAAAGATATTGGAACTTTATATTTCATTTTTGGAGTATGATCCGGAATAGTCGGAACTTCTCTAAGAATT

AATTGGATTATTGGGATTTATTGTTTGAGCTCATCATATATTTACAGTAGGTATAGACGTAGATACTCGAGCTTATTTTACTTCAGCAACTATAATT

CATGATACTTATTATGTAGTTGCCCATTTTCATTATGTTTTATCTATAGGAGCTGTATTTGCTATTATAGCAGGATTTATTCATTGATACCCTTTATTAACAGGAATAGTTATAAACCCTTCATG

CTGGAATACCTCGACGATACTCAGATTTTCCTGATAGTTACTTAACTTGAAATATTGTTTCTTCTTTAGGAAGAACAATT

AATTTGTCACACACAACATATATTTGCTCATTTAGTTCCTCAAGGAACTCCCCCTGTTTTAATACCTTTTATAGTATGCATTGAAACTATCAGTAATGTAATCCGACCAGGAACTTTAGCAGTACGATTAACTGCTAATATAATT

GACGAGATATTTCTCGAGAAGGAACTTTTCAAGGACTTCACACTATTCCCGTAACATTAGGATTACGATGAGGAATAATTTTATTTATTATTTCTGAAGTTTTTTTCTTTATTTCCTTCTTTTGAGCTTTTTTCCATAGTAGCTTATCCCCAACAATCGAATT

AATTGTCATTTTTCTAGAAGTCATCATTTTGGATTTGAAGCAGCTGCTTGATATTGACATTTTGTTGATGTAGTTTGACTATTCTTATATATTTCAATT

AATTTTAGGAATATATTCTTATAAAATGTTTGATCAAGGATGAAGTGAATATTTTGGAGGTCAGATATTATATAATCAAT

ATTATTCAATAACAGGGGATTTTAATAGAACTACATTGAATATACTAAATGATAAGGGGTGAACTATATCTTTTAGAATT

AATTCCTTTTTCTTCTTGATTACCTGCAGCTATGGCAGCTCCGACTCCTGTTTCTGCTTTAGTACATTCTTCTACATTAG

AATTTATTTTCAAAATGTAAAATCTTATAATGCTGGTATATTAACAGCTTTATCTAATCGAATCGGAGATGTTGCCTTAT

CTTTACATTCAATATCTATTGTAATAACTTTTTTATTTGATTGAATAAGTTTAATATTTATATCTTTTGTTTTATTAATT

AATTTTTATTAGATTTACTTTATTTATTTTGAGATTAAAGTTTTTATTAATAGATTTAGTATATTTTATTGAATGAGAAATT

GAGGGTATCAACCTGAACGTTTACAGGCGGGGATTTATTTATTATTTTATACTTTACTGGCTTCGTTGCCGTTATTAATT

AATTATGAAAGTTACTTTAGTATATTTTTTTTAACTTTTTGTGTTTGTGAAGGGGTTCTAGGGCTATCTATTTTAGTTTC

AATTATTATATTTATTTTTGGAAGTATTGTTTTTATTTCTAGTCGTAAGCATTTACTTTGTACTTTATTGAGATTAGAAT

AATTTTTATAGGAGGAATATTAGTTTTATTCATTTATGTTACTTCTCTTTCATCTAATGAAATATTTTCATTATCTATAAAACTATTTTTTTTATCTTTAAGTATAATT

AATTTTAACAGGATTATTCTTAGCAATACACTATACTGCTGATATTGAAACAGCTTTTAATAGAGTAAATCACATTTATCGTGATGTTAATAATGGTTGATTCCTACGAATT

CTGTTGATAATGCTACTTTAACTCGATTCTTTACATTTCATTTTATTTTACCTTTCATTGTATTAGCTTTAACTATAATT

CTTTTGATTTTGCTGAAGGGGAGTCTGAGTTAGTTTCAGGATTTAATGTAGAATATAGAAGAGGGGGATTTGCTTTAATT

AATTTTTTTATATATCAGAAATACATATGATTTATTGTATTTTGTTTTCCTTTAGGTCTCGTTTGATTTGCATCTTGTTT

AATTTAAGCTATAAACTTTTTGTAATAAACTAGGATTAGATACCCTATTATTAAAAATAAATATTAAAATGCTAAAGTAG

>Gv10-H-01

AATTTTATCTACTTTATTATTAAAAAGAGGGGCAGCTCCTTTTCATTTTTGATTCCCAGGAGTTATAGAAGGATTAAATT

AATTAATCATATTGGTTGAATATTAATAGCTATAATAAATAACGAACTTTTATGACTAACTTATTTTTTATTATATTCAATT

CAACAAATCATAAAGATATTGGAACTTTATATTTCATTTTTGGAGTATGATCCGGAATAGTCGGAACTTCTCTAAGAATT

AATTGGATTATTGGGATTTATTGTTTGAGCTCATCATATATTTACAGTAGGTATAGACGTAGATACTCGAGCTTATTTTACTTCAGCAACTATAATT

CATGATACTTATTATGTAGTTGCCCATTTTCATTATGTTTTATCTATAGGAGCTGTATTTGCTATTATAGCAGGATTTATTCATTGATACCCTTTATTAACAGGAATAGTTATAAACCCTTCATG

CTGGAATACCTCGACGATACTCAGATTTTCCCGATAGTTACTTAACTTGAAATATTATTTCTTCTTTAGGAAGAACAATT

AATTTGTCACACACAACATATATTTGCTCATTTAGTTCCTCAAGGAACTCCCCCTGTTTTAATACCTTTTATAGTATGCATTGAAACTATCAGTAATGTAATCCGACCAGGAACTTTAGCAGTACGATTAACTGCTAATATAATT

GACGAGATATTTCTCGAGAAGGAACTTTTCAAGGACTTCACACTATTCCCGTAACATTAGGATTACGATGAGGAATAATTTTATTTATTATTTCTGAAGTTTTTTTCTTTATTTCCTTCTTTTGAGCTTTTTTCCATAGTAGCTTATCCCCAACAATCGAATT

AATTGTCATTTTTCTAGAAGTCATCATTTTGGATTTGAAGCAGCTGCTTGATATTGACATTTTGTTGATGTAGTTTGACTATTCTTATATATTTCAATT

AATTTTAGGAATATATTCTTATAAAATGTTTGATCAAGGATGAAGTGAATATTTTGGAGGTCAGATATTATATAATCAAT

ATTATTCAATAACAGGGGATTTTAATAGAACTACATTGAATATACTAAATGATAAGGGGTGAACTATATCTTTTAGAATT

AATTCCTTTTTCTTCTTGATTACCTGCAGCTATGGCAGCTCCGACTCCTGTTTCTGCTTTAGTACATTCTTCTACATTAG

AATTTATTTTCAAAATGTAAAATCTTATAATGCTGGTATATTAACAGCTTTATCTAATCGAATCGGAGATGTTGCCTTAT

CTTTACATTCAATATCTATTGTAATAACTTTTTTATTTGATTGAATAAGTTTAATATTTATATCTTTTGTTTTATTAATT

AATTTTTATTAGATTTACTTTATTTATTTTGAGATTAAAGTTTTTATTAATAGATTTAGTATATTTTATTGAATGAGAAATT

GAGGGTATCAACCTGAACGTTTACAGGCGGGGATTTATTTATTATTTTATACTTTACTGGCTTCGTTGCCGTTATTAATT

AATTATGAAAGTTACTTTAGTATATTTTTTTTAACTTTTTGTGTTTGTGAAGGGGTTCTAGGGCTATCTATTTTAGTTTC

AATTATTATATTTATTTTTGGAAGTATTGTTTTTATTTCTAGTCGTAAGCATTTACTTTGTACTTTATTGAGATTAGAAT

AATTTTTATAGGAGGAATATTAGTTTTATTCATTTATGTTACTTCTCTTTCATCTAATGAAATATTTTCATTATCTATAAAACTATTTTTTTTATCTTTAAGTATAATT

AATTTTAACAGGATTATTCTTAGCAATACACTATACTGCTGATATTGAAACAGCTTTTAATAGAGTAAATCACATTTATCGTGATGTTAATAATGGTTGATTCCTACGAATT

CTGTTGATAATGCTACTTTAACTCGATTCTTTACATTTCATTTTATTTTACCTTTCATTGTATTAGCTTTAACTATAATT

CTTTTGATTTTGCTGAAGGGGAGTCTGAGTTAGTTTCAGGATTTAATGTAGAATATAGAAGAGGGGGATTTGCTTTAATT

AATTTTTTTATATATCAGAAATACATATGATTTATTGTATTTTGTTTTCCTTTAGGTCTCGTTTGATTTGCATCTTGTTT

AATTTAAGCTATAAACTTTTTGTAATAAACTAGGATTAGATACCCTATTATTAAAAATAAATATTAAAATGCTAAAGTAG

>Gv10-H-02

AATTTTATCTACTTTATTATTAAAAAGAGGAGCAGCTCCTTTTCATTTTTGATTCCCAGGAGTTATAGAAGGATTAAATT

AATTAATCATATTGGTTGAATATTAATAGCTATAATAAATAACGAACTTTTATGACTAACTTATTTTTTATTATATTCAATT

CAACAAATCATAAAGATATTGGAACTTTATATTTCATTTTTGGAGTATGATCCGGAATAGTCGGAACTTCTCTAAGAATT

AATTGGATTATTGGGATTTATTGTTTGAGCTCATCATATATTTACAGTAGGTATAGACGTAGATACTCGAGCTTATTTTACTTCAGCAACTATAATT

CATGATACTTATTATGTAGTTGCCCATTTTCATTATGTTTTATCTATAGGAGCTGTATTTGCTATTATAGCAGGATTTATTCATTGATACCCTTTATTAACAGGAATAGTTATAAACCCTTCATG

CTGGAATACCTCGACGATACTCAGATTTTCCCGATAGTTACTTAACTTGAAATATTATTTCTTCTTTAGGAAGAACAATT

AATTTGTCACACACAACATATATTTGCTCATTTAGTTCCTCAAGGAACTCCCCCTGTTTTAATACCTTTTATAGTATGCATTGAAACTATTAGTAATGTAATCCGACCAGGAACTTTAGCAGTACGATTAACTGCTAATATAATT

GACGAGATATTTCTCGAGAAGGAACTTTTCAAGGACTTCACACTATTCCCGTAACATTAGGATTACGATGAGGAATAATTTTATTTATTATTTCTGAAGTTTTTTTCTTTATTTCCTTCTTTTGAGCTTTTTTCCATAGTAGCTTATCCCCAACAATCGAATT

AATTGTCATTTTTCTAGAAGTCATCATTTTGGATTTGAAGCAGCTGCTTGATATTGACATTTTGTTGATGTAGTTTGACTATTCTTATATATTTCAATT

AATTTTAGGAATATATTCTTATAAAATGTTTGATCAAGGATGAAGTGAATATTTTGGAGGTCAGATATTATATAATCAAT

ATTATTCAATAACAGGGGATTTTAATAGAACTACATTGAATATACTAAATGATAAGGGGTGAACTATATCTTTTAGAATT

AATTCCTTTTTCTTCTTGATTACCTGCAGCTATGGCAGCTCCGACTCCTGTTTCTGCTTTAGTACATTCTTCTACATTAG

AATTTATTTTCAAAATGTAAAATCTTATAATGCTGGTATATTAACAGCTTTATCTAATCGAATCGGAGATGTTGCCTTAT

CTTTACATTCAATATCTATTGTAATAACTTTTTTATTTGATTGAATAAGTTTAATATTTATATCTTTTGTTTTATTAATT

AATTTTTATTAGATTTACTTTATTTATTTTGAGATTAAAGTTTTTATTAATAGATTTAGTATATTTTATTGAATGAGAAATT

GAGGGTATCAACCTGAACGTTTACAGGCGGGGATTTATTTATTATTTTATACTTTACTGGCTTCGTTGCCGTTATTAATT

AATTATGAAAGTTACTTTAGTATATTTTTTTTAACTTTTTGTGTTTGTGAAGGGGTTCTAGGGCTATCTATTTTAGTTTC

AATTATTATATTTATTTTTGGAAGTATTGTTTTTATTTCTAGTCGTAAGCATTTACTTTGTACTTTATTGAGATTAGAGT

AATTTTTATAGGAGGAATATTAGTTTTATTCATTTATGTTACTTCTCTTTCATCTAATGAAATATTTTCATTATCTATAAAACTATTTTTTTTATCTTTAAGTATAATT

AATTTTAACAGGATTATTCTTAGCAATACACTATACTGCTGATATTGAAACAGCTTTTAATAGAGTAAATCACATTTATCGTGATGTTAATAATGGTTGATTCCTACGAATT

CTGTTGATAATGCTACTTTAACTCGATTCTTTACATTTCATTTTATTTTACCTTTCATTGTATTAGCTTTAACTATAATT

CTTTTGATTTTGCTGAAGGGGAGTCTGAGTTAGTTTCAGGATTTAATGTAGAATATAGAAGAGGGGGATTTGCTTTAATT

AATTTTTTTATATATCAGAAATACATATGATTTATTGTATTTTGTTTTCCTTTAGGTCTCGTTTGATTTGCATCTTGTTT

AATTTAAGCTATAAACTTTTTGTAATAAACTAGGATTAGATACCCTATTATTAAAAATAAATATTAAAATGCTAAAGTAG

>Gv10-H-03

AATTTTATCTACTTTATTATTAAAAAGAGGAGCAGCTCCTTTTCATTTTTGATTCCCAGGAGTTATAGAAGGATTAAATT

AATTAATCATATTGGTTGAATATTAATAGCTATAATAAATAACGAACTTTTATGACTAACTTATTTTTTATTATATTCAATT

CAACAAATCATAAAGATATTGGAACTTTATATTTCATTTTTGGAGTATGATCCGGAATAGTCGGAACTTCTCTAAGAATT

AATTGGATTATTGGGATTTATTGTTTGAGCTCATCATATATTTACAGTAGGTATAGACGTAGATACTCGAGCTTATTTTACTTCAGCAACTATAATT

CATGATACTTATTATGTAGTTGCCCATTTTCATTATGTTTTATCTATAGGAGCTGTATTTGCTATTATAGCAGGATTTATTCATTGATACCCTTTATTAACAGGAATAGTTATAAACCCTTCATG

CTGGAATACCTCGACGATACTCGGATTTTCCCGATAGTTACTTAACTTGAAATATTATTTCTTCTTTAGGAAGAACAATT

AATTTGTCACACACAACATATATTTGCTCATTTAGTTCCTCAAGGAACTCCCCCTGTTTTAATACCTTTTATAGTATGCATTGAAACTATTAGTAATGTAATCCGACCAGGAACTTTAGCAGTACGATTAACTGCTAATATAATT

GACGAGATATTTCTCGAGAAGGAACTTTTCAAGGACTTCACACTATTCCCGTAACATTAGGATTACGATGAGGAATAATTTTATTTATTATTTCTGAAGTTTTTTTCTTTATTTCCTTCTTTTGAGCTTTTTTCCATAGTAGCTTATCCCCAACAATCGAATT

AATTGTCATTTTTCTAGAAGTCATCATTTTGGATTTGAAGCAGCTGCTTGATATTGACATTTTGTTGATGTAGTTTGACTATTCTTATATATTTCAATT

AATTTTAGGAATATATTCTTATAAAATGTTTGATCAAGGATGAAGTGAATATTTTGGAGGTCAGATATTATATAATCAAT

ATTATTCAATAACAGGGGATTTTAATAGAACTACATTGAATATACTAAATGATAAGGGGTGAACTATATCTTTTAGAATT

AATTCCTTTTTCTTCTTGATTACCTGCAGCTATGGCAGCTCCGACTCCTGTTTCTGCTTTAGTACATTCTTCTACATTAG

AATTTATTTTCAAAATGTAAAATCTTATAATGCTGGTATATTAACAGCTTTATCTAATCGAATCGGAGATGTTGCCTTAT

CTTTACATTCAATATCTATTGTAATAACTTTTTTATTTGATTGAATAAGTTTAATATTTATATCTTTTGTTTTATTAATT

AATTTTTATTAGATTTACTTTATTTATTTTGAGATTAAAGTTTTTATTAATAGATTTAGTATATTTTATTGAATGAGAAATT

GAGGGTATCAACCTGAACGTTTACAGGCGGGGATTTATTTATTATTTTATACTTTACTGGCTTCGTTGCCGTTATTAATT

AATTATGAAAGTTACTTTAGTATATTTTTTTTAACTTTTTGTGTTTGTGAAGGGGTTCTAGGGCTATCTATTTTAGTTTC

AATTATTATATTTATTTTTGGAAGTATTGTTTTTATTTCTAGTCGTAAGCATTTACTTTGTACTTTATTGAGATTAGAAT

AATTTTTATAGGAGGAATATTAGTTTTATTCATTTATGTTACTTCTCTTTCATCTAATGAAATATTTTCATTATCTATAAAACTATTTTTTTTATCTTTAAGTATAATT

AATTTTAACAGGATTATTCTTAGCAATACACTATACTGCTGATATTGAAACAGCTTTTAATAGAGTAAATCACATTTATCGTGATGTTAATAATGGTTGATTCCTACGAATT

CTGTTGATAATGCTACTTTAACTCGATTCTTTACATTTCATTTTATTTTACCTTTCATTGTATTAGCTTTAACTATAATT

CTTTTGATTTTGCTGAAGGGGAGTCTGAGTTAGTTTCAGGATTTAATGTAGAATATAGAAGAGGGGGATTTGCTTTAATT

AATTTTTTTATATATCAGAAATACATATGATTTATTGTATTTTGTTTTCCTTTAGGTCTCGTTTGATTTGCATCTTGTTT

AATTTAAGCTATAAACTTTTTGTAATAAACTAGGATTAGATACCCTATTATTAAAAATAAATATTAAAATGCTAAAGTAG

>Gv10-H-04

AATTTTATCTACTTTATTATTAAAAAGAGGGGCAGCTCCTTTTCATTTTTGATTCCCAGGAGTTATAGAAGGATTAAATT

AATTAATCATATTGGTTGAATATTAATAGCTATAATAAATAACGAACTTTTATGACTAACTTATTTTTTATTATATTCAATT

CAACAAATCATAAAGATATTGGAACTTTATATTTCATTTTTGGAGTATGATCCGGAATAGTCGGAACTTCTCTAAGAATT

AATTGGATTATTGGGATTTATTGTTTGAGCTCATCATATATTTACAGTAGGTATAGACGTAGATACTCGAGCTTATTTTACTTCAGCAACTATAATT

CATGATACTTATTATGTAGTTGCCCATTTTCATTATGTTTTATCTATAGGAGCTGTATTTGCTATTATAGCAGGATTTATTCATTGATACCCTTTATTAACAGGAATAGTTATAAACCCTTCATG

CTGGAATACCTCGACGATACTCAGATTTTCCCGATAGTTACTTAACTTGAAATATTATTTCTTCTTTAGGAAGAACAATT

AATTTGTCACACACAACATATATTTGCTCATTTAGTTCCTCAAGGAACTCCCCCTGTTTTAATACCTTTTATAGTATGCATTGAAACTATCAGTAATGTAATCCGACCAGGAACTTTAGCAGTACGATTAACTGCTAATATAATT

GACGAGATATTTCTCGAGAAGGAACTTTTCAAGGACTTCACACTATTCCCGTAACATTAGGATTACGATGAGGAATAATTTTATTTATTATTTCTGAAGTTTTTTTCTTTATTTCCTTCTTTTGAGCTTTTTTCCATAGTAGCTTATCCCCAACAATCGAATT

AATTGTCATTTTTCTAGAAGTCATCATTTTGGATTTGAAGCAGCTGCTTGATATTGACATTTTGTTGATGTAGTTTGACTATTCTTATATATTTCAATT

AATTTTAGGAATATATTCTTATAAAATGTTTGATCAAGGATGAAGTGAATATTTTGGAGGTCAGATATTATATAATCAAT

ATTATTCAATAACAGGGGATTTTAATAGAACTACATTGAATATACTAAATGATAAGGGGTGAACTATATCTTTTAGAATT

AATTCCTTTTTCTTCTTGATTACCTGCAGCTATGGCAGCTCCGACTCCTGTTTCTGCTTTAGTACATTCTTCTACATTAG

AATTTATTTTCAAAATGTAAAATCTTATAATGCTGGTATATTAACAGCTTTATCTAATCGAATCGGAGATGTTGCCTTAT

CTTTACATTCAATATCTATTGTAATAACTTTTTTATTTGATTGAATAAGTTTAATATTTATATCTTTTGTTTTATTAATT

AATTTTTATTAGATTTACTTTATTTATTTTGAGATTAAAGTTTTTATTAATAGATTTAGTATATTTTATTGAATGAGAAATT

GAGGGTATCAACCTGAACGTTTACAGGCGGGGATTTATTTATTATTTTATACTTTACTGGCTTCGTTGCCGTTATTAATT

AATTATGAAAGTTACTTTAGTATATTTTTTTTAACTTTTTGTGTTTGTGAAGGGGTTCTAGGGCTATCTATTTTAGTTTC

AATTATTATATTTATTTTTGGAAGTATTGTTTTTATTTCTAGTCGTAAGCATTTACTTTGTACTTTATTGAGATTAGAAT

AATTTTTATAGGAGGAATATTAGTTTTATTCATTTATGTTACTTCTCTTTCATCTAATGAAATATTTTCATTATCTATAAAACTATTTTTTTTATCTTTAAGTATAATT

AATTTTAACAGGATTATTCTTAGCAATACACTATACTGCTGATATTGAAACAGCTTTTAATAGAGTAAATCACATTTATCGTGATGTTAATAATGGTTGATTCCTACGAATT

CTGTTGATAATGCTACTTTAACTCGATTCTTTACATTTCATTTTATTTTACCTTTCATTGTATTAGCTTTAACTATAATT

CTTTTGATTTTGCTGAAGGGGAGTCTGAGTTAGTTTCAGGATTTAATGTAGAATATAGAAGAGGGGGATTTGCTTTAATT

AATTTTTTTATATATCAGAAATACATATGATTTATTGTATTTTGTTTTCCTTTAGGTCTCGTTTGATTTGCATCTTGTTT

AATTTAAGCTATAAACTTTTTGTAATAAACTAGGATTAGATACCCTATTATTAAAAATAAATATTAAAATGCTAAAGTAG

>Gv10-H-05

ANTTTTATCTACTTTATTATTAAAAAGAGGGGCAGCTCCTTTTCATTTTTGATTCCCAGGAGTTATAGAAGGATTAAATT

AATTAATCATATTGGTTGAATATTAATAGCTATAATAAATAACGAACTTTTATGACTAACTTATTTTTTATTATATTCAATT

CAACAAATCATAAAGATATTGGAACTTTATATTTCATTTTTGGAGTATGATCCGGAATAGTCGGAACTTCTCTAAGAATT

AATTGGATTATTGGGATTTATTGTTTGAGCTCATCATATATTTACAGTAGGTATAGACGTAGATACTCGAGCTTATTTTACTTCAGCAACTATAATT

CATGATACTTATTATGTAGTTGCCCATTTTCATTATGTTTTATCTATAGGAGCTGTATTTGCTATTATAGCAGGATTTATTCATTGATACCCTTTATTAACAGGAATAGTTATAAACCCTTCATG

NNNNNNNNNNNNNNNNNNNNNNNNNNNNNNNNNNNNNNNNNNNNNNNNNNNNNNNNNNNNNNNNNNNNNNNNNNNNNNNN

AATTTGTCACACACAACATATATTTGCTCATTTAGTTCCTCAAGGAACTCCCCCTGTTTTAATACCTTTTATAGTATGCATTGAAACTATCAGTAATGTAATCCGACCAGGAACTTTAGCAGTACGATTAACTGCTAATATAATT

GACGAGATATTTCTCGAGAAGGAACTTTTCAAGGACTTCACACTATTCCCGTAACATTAGGATTACGATGAGGAATAATTTTATTTATTATTTCTGAAGTTTTTTTCTTTATTTCCTTCTTTTGAGCTTTTTTCCATAGTAGCTTATCCCCAACAATCGAATT

AATTGTCATTTTTCTAGAAGTCATCATTTTGGATTTGAAGCAGCTGCTTGATATTGACATTTTGTTGATGTAGTTTGACTATTCTTATATATTTCAATT

AATTTTAGGAATATATTCTTATAAAATGTTTGATCAAGGATGAAGTGAATATTTTGGAGGTCAGATATTATATAATCAAT

ATTATTCAATAACAGGGGATTTTAATAGAACTACATTGAATATACTAAATGATAAGGGGTGAACTATATCTTTTAGAATT

ANTTCCTTTTTCTTCTTGATTACCTGCAGCTATGGCAGCTCCGACTCCTGTTTCTGCTTTAGTACATTCTTCTACATTAG

AATTTATTTTCAAAATGTAAAATCTTATAATGCTGGTATATTAACAGCTTTATCTAATCGAATCGGAGATGTTGCCTTAT

CTTTACATTCAATATCTATTGTAATAACTTTTTTATTTGATTGAATAAGTTTAATATTTATATCTTTTGTTTTATTAATT

AATTTTTATTAGATTTACTTTATTTATTTTGAGATTAAAGTTTTTATTAATAGATTTAGTATATTTTATTGAATGAGAAATT

GAGGGTATCAACCTGAACGTTTACAGGCGGGGATTTATTTATTATTTTATACTTTACTGGCTTCGTTGCCGTTATTAATT

AATTATGAAAGTTACTTTAGTATATTTTTTTTAACTTTTTGTGTTTGTGAAGGGGTTCTAGGGCTATCTATTTTAGTTTC

AATTATTATATTTATTTTTGGAAGTATTGTTTTTATTTCTAGTCGTAAGCATTTACTTTGTACTTTATTGAGATTAGAAT

AATTTTTATAGGAGGAATATTAGTTTTATTCATTTATGTTACTTCTCTTTCATCTAATGAAATATTTTCATTATCTATAAAACTATTTTTTTTATCTTTAAGTATAATT

AATTTTAACAGGATTATTCTTAGCAATACACTATACTGCTGATATTGAAACAGCTTTTAATAGAGTAAATCACATTTATCGTGATGTTAATAATGGTTGATTCCTACGAATT

CTGTTGATAATGCTACTTTAACTCGATTCTTTACATTTCATTTTATTTTACCTTTCATTGTATTAGCTTTAACTATAATT

CTTTTGATTTTGCTGAAGGGGAGTCTGAGTTAGTTTCAGGATTTAATGTAGAATATAGAAGAGGGGGATTTGCTTTAATT

AATTTTTTTATATATCAGAAATACATATGATTTATTGTATTTTGTTTTCCTTTAGGTCTCGTTTGATTTGCATCTTGTTT

AATTTAAGCTATAAACTTTTTGTAATAAACTAGGATTAGATACCCTATTATTAAAAATAAATATTAAAATGCTAAAGTAG

>Gv10-H-06
[truncated: 190,981 more chars]
