# Supplementary material for: Contrasting genetic structure between mitochondrial and nuclear markers in the dengue fever mosquito from Rio de Janeiro: implications for vector control
Source: Evol Appl. 2015 Sep 7;8(9):901–15. doi: 10.1111/eva.12301 (PMC4610386; doi:10.1111/eva.12301)
Supplement: Supplementary file 8 — File S6.jModelTest-2.1.7 results. [file eva0008-0901-sd8.pdf]

```
-------------------------- jModeltest 2.1.7 v20150530 --------------------------
(c) 2011-onwards D. Darriba, G.L. Taboada, R. Doallo and D. Posada,
(1) Department of Biochemistry, Genetics and Immunology
    University of Vigo, 36310 Vigo, Spain.
(2) Department of Electronics and Systems
    University of A Coruna, 15071 A Coruna, Spain.
e-mail: ddarriba@udc.es, dposada@uvigo.es
--------------------------------------------------------------------------------

Thu Jun 25 08:17:28 AEST 2015
Linux 3.2.0-76-virtual, arch: amd64, bits: 64, numcores: 16

jModelTest 2.1.7 v20150530
Copyright (C) 2011 D. Darriba, G.L. Taboada, R. Doallo and D. Posada
This program comes with ABSOLUTELY NO WARRANTY
This is free software, and you are welcome to redistribute it under certain
conditions

Notice: This program may contain errors. Please inspect results carefully.

--------------------------------------------------------------------------------
Citation: Darriba D, Taboada GL, Doallo R and Posada D. 2012.
          "jModelTest 2: more models, new heuristics and parallel computing".
          Nature Methods 9(8), 772.
--------------------------------------------------------------------------------

Reading data file "S1_File_relaxed_phylip_format_mtSNPs.phy"... OK.
  number of sequences: 63
  number of sites: 92


---------------------------------------------------------------
*                                                             *
*        COMPUTATION OF LIKELIHOOD SCORES WITH PHYML          *
*                                                             *
---------------------------------------------------------------

::Settings::

 Phyml version = 3.0
 Phyml binary = PhyML_3.0_linux64
 Phyml path = /usr/local/bin/jmodeltest-2.1.7/exe/phyml/
 Candidate models = 44
   number of substitution schemes = 11
   including models with equal/unequal base frequencies (+F)
   including only models without a proportion of invariable sites
   including models with/without rate variation among sites (+G) (nCat = 4)
 Optimized free parameters (K) = substitution parameters + 123 branch lengths +
topology
 Base tree for likelihood calculations = ML tree
 Tree topology search operation = BEST
```

::Progress::

```
Model             Exec. Time      Total Time      -lnL
--------------------------------------------------------------------------
JC+G             00h:00:54:00    00h:00:55:04    787.9410
K80+G            00h:00:59:06    00h:01:01:01    717.4189
TPM1+G           00h:00:58:05    00h:01:01:01    717.4185
TrNef+G          00h:01:05:06    00h:01:08:01    716.6899
F81+G            00h:01:07:03    00h:01:08:06    783.6567
TPM2+G           00h:01:10:02    00h:01:12:05    717.3468
TPM1uf+G                 00h:01:15:03    00h:01:17:00    709.2155
TPM2uf+G                 00h:01:15:05    00h:01:17:00    709.2632
TrN+G            00h:01:16:09    00h:01:19:02    709.2498
HKY+G            00h:01:18:05    00h:01:20:07    709.2660
GTR+G            00h:01:19:02    00h:01:21:01    708.7285
TVM+G            00h:01:19:08    00h:01:21:04    708.7379
TIM1+G           00h:01:19:04    00h:01:21:06    709.2020
TIM2+G           00h:01:23:00    00h:01:25:00    709.2499
TPM3uf+G                 00h:01:25:02    00h:01:26:06    708.8918
TIM3+G           00h:01:26:07    00h:01:28:07    708.8822
HKY              00h:00:21:05    00h:01:38:06    709.2666
F81              00h:00:21:09    00h:01:38:09    783.7626
TPM1uf           00h:00:21:01    00h:01:41:09    709.2122
TrN              00h:00:23:05    00h:01:42:08    709.2481
TPM3uf           00h:00:22:00    00h:01:43:04    708.8916
TPM2uf           00h:00:22:06    00h:01:43:07    709.2637
TIM1             00h:00:24:01    00h:01:45:07    709.1974
TIM2             00h:00:22:03    00h:01:47:03    709.2502
TIM3             00h:00:22:08    00h:01:49:04    708.8837
TVM              00h:00:21:09    00h:01:50:06    708.7383
TPM3+G           00h:00:58:09    00h:01:54:03    716.6482
JC               00h:00:19:03    00h:01:58:02    788.0534
K80              00h:00:19:06    00h:02:01:05    717.4183
TIM2ef+G                 00h:01:01:01    00h:02:02:02    716.6145
TPM1             00h:00:20:03    00h:02:03:07    717.4256
GTR              00h:00:26:02    00h:02:04:08    708.7288
TIM1ef+G                 00h:01:03:07    00h:02:04:09    716.6895
TrNef            00h:00:22:02    00h:02:05:00    716.6959
TPM2             00h:00:22:05    00h:02:06:02    717.3541
TPM3             00h:00:20:09    00h:02:06:06    716.6111
TVMef+G          00h:00:58:08    00h:02:07:04    716.4471
TIM1ef           00h:00:20:05    00h:02:07:09    716.6955
TIM2ef           00h:00:20:07    00h:02:10:01    716.6205
TIM3ef+G                 00h:01:02:03    00h:02:10:04    715.8251
TIM3ef           00h:00:20:00    00h:02:10:06    715.8311
TVMef            00h:00:18:00    00h:02:12:03    716.4454
SYM+G            00h:00:59:09    00h:02:12:04    715.6230
SYM              00h:00:18:00    00h:02:16:03    715.6226
```

::Results::

Model = JC
partition = 000000
-lnL = 788.0534
K = 124

Model = JC+G
partition = 000000
-lnL = 787.9410
K = 125
gamma shape = 15351.2710

Model = F81
partition = 000000
-lnL = 783.7626
K = 127
freqA = 0.2360
freqC = 0.2407
freqG = 0.1901
freqT = 0.3332

Model = F81+G
partition = 000000
-lnL = 783.6567
K = 128
freqA = 0.2359
freqC = 0.2407
freqG = 0.1901
freqT = 0.3333
gamma shape = 15351.2710

Model = K80
partition = 010010
-lnL = 717.4183
K = 125
kappa = 14.0552 (ti/tv = 7.0276)

Model = K80+G
partition = 010010
-lnL = 717.4189
K = 126
kappa = 14.0550 (ti/tv = 7.0275)
gamma shape = 15351.2710

Model = HKY
partition = 010010
-lnL = 709.2666
K = 128
freqA = 0.2603
freqC = 0.2057
freqG = 0.1735
freqT = 0.3605
kappa = 15.2030 (ti/tv = 7.3851)

```
Model = HKY+G
partition = 010010
-lnL = 709.2660
K = 129
freqA = 0.2603
freqC = 0.2057
freqG = 0.1735
freqT = 0.3606
kappa = 15.2082 (ti/tv = 7.3877)
gamma shape = 15185.0220

Model = TrNef
partition = 010020
-lnL = 716.6959
K = 126
R(a) [AC] =  1.0000
R(b) [AG] =  12.2219
R(c) [AT] =  1.0000
R(d) [CG] =  1.0000
R(e) [CT] =  15.6453
R(f) [GT] =  1.0000

Model = TrNef+G
partition = 010020
-lnL = 716.6899
K = 127
R(a) [AC] =  1.0000
R(b) [AG] =  12.2255
R(c) [AT] =  1.0000
R(d) [CG] =  1.0000
R(e) [CT] =  15.6530
R(f) [GT] =  1.0000
gamma shape = 15351.2710

Model = TrN
partition = 010020
-lnL = 709.2481
K = 129
freqA = 0.2631
freqC = 0.2032
freqG = 0.1762
freqT = 0.3575
R(a) [AC] =  1.0000
R(b) [AG] =  14.8029
R(c) [AT] =  1.0000
R(d) [CG] =  1.0000
R(e) [CT] =  15.5549
R(f) [GT] =  1.0000

Model = TrN+G
partition = 010020
```

-lnL = 709.2498
K = 130
freqA = 0.2631
freqC = 0.2032
freqG = 0.1761
freqT = 0.3576
R(a) [AC] = 1.0000
R(b) [AG] = 14.8029
R(c) [AT] = 1.0000
R(d) [CG] = 1.0000
R(e) [CT] = 15.5358
R(f) [GT] = 1.0000
gamma shape = 15185.0220

Model = TPM1
partition = 012210
-lnL = 717.4256
K = 126
R(a) [AC] = 1.0000
R(b) [AG] = 13.9537
R(c) [AT] = 0.9858
R(d) [CG] = 0.9858
R(e) [CT] = 13.9537
R(f) [GT] = 1.0000

Model = TPM1+G
partition = 012210
-lnL = 717.4185
K = 127
R(a) [AC] = 1.0000
R(b) [AG] = 13.9546
R(c) [AT] = 0.9857
R(d) [CG] = 0.9857
R(e) [CT] = 13.9546
R(f) [GT] = 1.0000
gamma shape = 15351.2710

Model = TPM1uf
partition = 012210
-lnL = 709.2121
K = 129
freqA = 0.2608
freqC = 0.2053
freqG = 0.1730
freqT = 0.3609
R(a) [AC] = 1.0000
R(b) [AG] = 13.9426
R(c) [AT] = 0.8444
R(d) [CG] = 0.8444
R(e) [CT] = 13.9426
R(f) [GT] = 1.0000

```
Model = TPM1uf+G
partition = 012210
-lnL = 709.2155
K = 130
freqA = 0.2608
freqC = 0.2053
freqG = 0.1730
freqT = 0.3609
R(a) [AC] =  1.0000
R(b) [AG] =  13.9412
R(c) [AT] =  0.8443
R(d) [CG] =  0.8443
R(e) [CT] =  13.9412
R(f) [GT] =  1.0000
gamma shape = 15185.0220

Model = TPM2
partition = 010212
-lnL = 717.3541
K = 126
R(a) [AC] =  1.2215
R(b) [AG] =  15.8208
R(c) [AT] =  1.2215
R(d) [CG] =  1.0000
R(e) [CT] =  15.8208
R(f) [GT] =  1.0000

Model = TPM2+G
partition = 010212
-lnL = 717.3468
K = 127
R(a) [AC] =  1.2217
R(b) [AG] =  15.8229
R(c) [AT] =  1.2217
R(d) [CG] =  1.0000
R(e) [CT] =  15.8229
R(f) [GT] =  1.0000
gamma shape = 15351.2710

Model = TPM2uf
partition = 010212
-lnL = 709.2637
K = 129
freqA = 0.2606
freqC = 0.2056
freqG = 0.1732
freqT = 0.3605
R(a) [AC] =  0.9607
R(b) [AG] =  14.8347
R(c) [AT] =  0.9607
R(d) [CG] =  1.0000
R(e) [CT] =  14.8347
```

R(f) [GT] = 1.0000

Model = TPM2uf+G
partition = 010212
-lnL = 709.2632
K = 130
freqA = 0.2606
freqC = 0.2057
freqG = 0.1732
freqT = 0.3606
R(a) [AC] = 0.9597
R(b) [AG] = 14.8280
R(c) [AT] = 0.9597
R(d) [CG] = 1.0000
R(e) [CT] = 14.8280
R(f) [GT] = 1.0000
gamma shape = 15185.0220

Model = TPM3
partition = 012012
-lnL = 716.6111
K = 126
R(a) [AC] = 0.4362
R(b) [AG] = 9.7769
R(c) [AT] = 1.0000
R(d) [CG] = 0.4362
R(e) [CT] = 9.7769
R(f) [GT] = 1.0000

Model = TPM3+G
partition = 012012
-lnL = 716.6482
K = 127
R(a) [AC] = 0.4361
R(b) [AG] = 9.7706
R(c) [AT] = 1.0000
R(d) [CG] = 0.4361
R(e) [CT] = 9.7706
R(f) [GT] = 1.0000
gamma shape = 15185.0220

Model = TPM3uf
partition = 012012
-lnL = 708.8916
K = 129
freqA = 0.2603
freqC = 0.2092
freqG = 0.1736
freqT = 0.3569
R(a) [AC] = 0.5991
R(b) [AG] = 13.0358
R(c) [AT] = 1.0000

```
R(d) [CG] =  0.5991
R(e) [CT] =  13.0358
R(f) [GT] =  1.0000

Model = TPM3uf+G
partition = 012012
-lnL = 708.8918
K = 130
freqA = 0.2603
freqC = 0.2092
freqG = 0.1736
freqT = 0.3569
R(a) [AC] =  0.5990
R(b) [AG] =  13.0346
R(c) [AT] =  1.0000
R(d) [CG] =  0.5990
R(e) [CT] =  13.0346
R(f) [GT] =  1.0000
gamma shape = 55386.6210

Model = TIM1ef
partition = 012230
-lnL = 716.6955
K = 127
R(a) [AC] =  1.0000
R(b) [AG] =  12.1341
R(c) [AT] =  0.9857
R(d) [CG] =  0.9857
R(e) [CT] =  15.5327
R(f) [GT] =  1.0000

Model = TIM1ef+G
partition = 012230
-lnL = 716.6895
K = 128
R(a) [AC] =  1.0000
R(b) [AG] =  12.1376
R(c) [AT] =  0.9858
R(d) [CG] =  0.9858
R(e) [CT] =  15.5389
R(f) [GT] =  1.0000
gamma shape = 15351.2710

Model = TIM1
partition = 012230
-lnL = 709.1974
K = 130
freqA = 0.2636
freqC = 0.2028
freqG = 0.1757
freqT = 0.3579
R(a) [AC] =  1.0000
```

R(b) [AG] = 13.5765
R(c) [AT] = 0.8448
R(d) [CG] = 0.8448
R(e) [CT] = 14.2568
R(f) [GT] = 1.0000

Model = TIM1+G
partition = 012230
-lnL = 709.2020
K = 131
freqA = 0.2635
freqC = 0.2029
freqG = 0.1756
freqT = 0.3580
R(a) [AC] = 1.0000
R(b) [AG] = 13.5737
R(c) [AT] = 0.8448
R(d) [CG] = 0.8448
R(e) [CT] = 14.2399
R(f) [GT] = 1.0000
gamma shape = 15185.0220

Model = TIM2ef
partition = 010232
-lnL = 716.6205
K = 127
R(a) [AC] = 1.2262
R(b) [AG] = 13.7913
R(c) [AT] = 1.2262
R(d) [CG] = 1.0000
R(e) [CT] = 17.6611
R(f) [GT] = 1.0000

Model = TIM2ef+G
partition = 010232
-lnL = 716.6145
K = 128
R(a) [AC] = 1.2264
R(b) [AG] = 13.7931
R(c) [AT] = 1.2264
R(d) [CG] = 1.0000
R(e) [CT] = 17.6671
R(f) [GT] = 1.0000
gamma shape = 15351.2710

Model = TIM2
partition = 010232
-lnL = 709.2502
K = 130
freqA = 0.2634
freqC = 0.2032
freqG = 0.1758

freqT = 0.3576
R(a) [AC] = 0.9639
R(b) [AG] = 14.4752
R(c) [AT] = 0.9639
R(d) [CG] = 1.0000
R(e) [CT] = 15.1897
R(f) [GT] = 1.0000

Model = TIM2+G
partition = 010232
-lnL = 709.2499
K = 131
freqA = 0.2634
freqC = 0.2032
freqG = 0.1758
freqT = 0.3576
R(a) [AC] = 0.9636
R(b) [AG] = 14.4759
R(c) [AT] = 0.9636
R(d) [CG] = 1.0000
R(e) [CT] = 15.1849
R(f) [GT] = 1.0000
gamma shape = 15185.0220

Model = TIM3ef
partition = 012032
-lnL = 715.8311
K = 127
R(a) [AC] = 0.4715
R(b) [AG] = 9.5906
R(c) [AT] = 1.0000
R(d) [CG] = 0.4715
R(e) [CT] = 12.2824
R(f) [GT] = 1.0000

Model = TIM3ef+G
partition = 012032
-lnL = 715.8251
K = 128
R(a) [AC] = 0.4715
R(b) [AG] = 9.5902
R(c) [AT] = 1.0000
R(d) [CG] = 0.4715
R(e) [CT] = 12.2846
R(f) [GT] = 1.0000
gamma shape = 15351.2710

Model = TIM3
partition = 012032
-lnL = 708.8837
K = 130
freqA = 0.2627

```
freqC = 0.2071
freqG = 0.1759
freqT = 0.3544
R(a) [AC] =  0.6008
R(b) [AG] =  12.7497
R(c) [AT] =  1.0000
R(d) [CG] =  0.6008
R(e) [CT] =  13.2857
R(f) [GT] =  1.0000

Model = TIM3+G
partition = 012032
-lnL = 708.8822
K = 131
freqA = 0.2627
freqC = 0.2070
freqG = 0.1759
freqT = 0.3544
R(a) [AC] =  0.6002
R(b) [AG] =  12.7424
R(c) [AT] =  1.0000
R(d) [CG] =  0.6002
R(e) [CT] =  13.2868
R(f) [GT] =  1.0000
gamma shape = 55386.6210

Model = TVMef
partition = 012314
-lnL = 716.4454
K = 128
R(a) [AC] =  0.5888
R(b) [AG] =  10.3346
R(c) [AT] =  1.1017
R(d) [CG] =  0.2906
R(e) [CT] =  10.3346
R(f) [GT] =  1.0000

Model = TVMef+G
partition = 012314
-lnL = 716.4471
K = 129
R(a) [AC] =  0.5889
R(b) [AG] =  10.3339
R(c) [AT] =  1.1010
R(d) [CG] =  0.2907
R(e) [CT] =  10.3339
R(f) [GT] =  1.0000
gamma shape = 15185.0220

Model = TVM
partition = 012314
-lnL = 708.7383
```

```
K = 131
freqA = 0.2607
freqC = 0.2091
freqG = 0.1733
freqT = 0.3570
R(a) [AC] =  0.6352
R(b) [AG] =  11.5718
R(c) [AT] =  0.8154
R(d) [CG] =  0.3733
R(e) [CT] =  11.5718
R(f) [GT] =  1.0000

Model = TVM+G
partition = 012314
-lnL = 708.7379
K = 132
freqA = 0.2607
freqC = 0.2091
freqG = 0.1732
freqT = 0.3570
R(a) [AC] =  0.6350
R(b) [AG] =  11.5683
R(c) [AT] =  0.8149
R(d) [CG] =  0.3736
R(e) [CT] =  11.5683
R(f) [GT] =  1.0000
gamma shape = 15185.0220

Model = SYM
partition = 012345
-lnL = 715.6226
K = 129
R(a) [AC] =  0.6333
R(b) [AG] =  10.0656
R(c) [AT] =  1.0856
R(d) [CG] =  0.3104
R(e) [CT] =  12.8961
R(f) [GT] =  1.0000

Model = SYM+G
partition = 012345
-lnL = 715.6230
K = 130
R(a) [AC] =  0.6333
R(b) [AG] =  10.0639
R(c) [AT] =  1.0856
R(d) [CG] =  0.3103
R(e) [CT] =  12.8986
R(f) [GT] =  1.0000
gamma shape = 15351.2710

Model = GTR
```

```
   partition = 012345
   -lnL = 708.7288
   K = 132
   freqA = 0.2631
   freqC = 0.2069
   freqG = 0.1755
   freqT = 0.3545
   R(a) [AC] =  0.6384
   R(b) [AG] =  11.3356
   R(c) [AT] =  0.8169
   R(d) [CG] =  0.3742
   R(e) [CT] =  11.8064
   R(f) [GT] =  1.0000

   Model = GTR+G
   partition = 012345
   -lnL = 708.7285
   K = 133
   freqA = 0.2630
   freqC = 0.2070
   freqG = 0.1754
   freqT = 0.3546
   R(a) [AC] =  0.6385
   R(b) [AG] =  11.3417
   R(c) [AT] =  0.8174
   R(d) [CG] =  0.3743
   R(e) [CT] =  11.8064
   R(f) [GT] =  1.0000
   gamma shape = 15185.0220


Computation of likelihood scores completed. It took 00h:02:18:01.




---------------------------------------------------------------
*                                                             *
*             AKAIKE INFORMATION CRITERION (AIC)              *
*                                                             *
---------------------------------------------------------------

 Model selected:
   Model = HKY
   partition = 010010
   -lnL = 709.2666
   K = 128
   freqA = 0.2603
   freqC = 0.2057
   freqG = 0.1735
   freqT = 0.3605
   kappa = 15.2030 (ti/tv = 7.3851)
```

Tree for the best AIC model =
(Vi23-4:0.00000001,Vi23-5:0.00000058,(Vi10-5:0.03561308,(Vi12-5:0.01307189,(Br53-1:0.00000001,((BrM21-1:0.00000051,(Br47-1:0.00000024,(BrM54-1:0.00000001,(BrM22-1:0.01037483,((Vi23-2:0.02266554,(Vi12-1:0.00000050,Vi23-1:0.01199993):0.00000125):0.02462101,((In521:0.01111465,(In501:0.02348288,(In526:0.00000001,(In535:0.01113005,In556:0.01173030):0.01123786):0.01059872):0.05953795):0.00000065,((Gv10-H-03:0.02257912,Gv10-C-02:0.00000062):0.01116350,((Gv10-B-06:0.00000001,(Gv10-C-01:0.01122578,(Gv10-A-01:0.00000001,((Gv10-A-10:0.01243267,(Gv10-B-03:0.01115861,Gv10-E-05:0.00000097):0.00000097):0.00000075,(Gv10-F-08:0.01127201,Gv10-B-02:0.01126435):0.00000067):0.01113747):0.00000060):0.00000069):0.02245354,(In501a:0.00000001,(Vi10-2:0.04716243,((In534:0.00000001,In553:0.01121676):0.01121676,(Vi10-3:0.02266601,Vi12-3:0.00000047):0.01117005):0.00000056):0.00000059):0.00000001):0.00000060):0.00000083):0.00967116):0.08682646):0.01467517):0.00000384):0.00000381):0.20885083,(((Gv10-A-08:0.00000151,Gv10-F-10:0.00000001):0.02393215,((Br32-1:0.00000047,((BrK55-1:0.00000093,(BrM48-2:0.01186974,(Br57-1:0.01194923,(Br22-1:0.00000001,(Br32-2:0.01185144,BrM59-1:0.02310690):0.00000062):0.00000062):0.00000063):0.00000058):0.01113786,(Br26-2:0.00000001,Br30-1:0.00000061):0.00000061):0.01119346):0.01145164,(BrM23-1:0.00489765,Br30-2:0.00000064):0.00606830):0.01079505):0.05289481,(Vi10-1:0.01188110,(BrM56-1:0.01216771,((BrM17-1:0.00000001,BrM45-1:0.03700713):0.01160172,(BrK11-1:0.00000001,(BrK47-1:0.01110008,(BrM02-1:0.01111694,(BrM51-1:0.01122866,((BrM31-1:0.01110008,(BrM55-1:0.00000001,(BrM60-3:0.00000001,(BrK02-1:0.00000047,BrM03-1:0.00000001):0.00000071):0.00000046):0.00000054):0.00000054,(Br48-1:0.00000001,(BrM26-1:0.01122867,(BrM11-1:0.00543080,Br22-2:0.01318413):0.00569500):0.00000064):0.00000001):0.00000057):0.00000069):0.00000060):0.01115710):0.00000060):0.00000087):0.02761076):0.04170545):0.07536507):0.09001970):0.04816843):0.01031336):0.02329206);

* AIC MODEL SELECTION : Selection uncertainty

Model	-lnL	K	AIC	delta	weight	cumWeight
HKY	709.26655	128	1674.533100	0.000000	0.222094	0.222094
TPM3uf	708.89163	129	1675.783260	1.250160	0.118869	0.340963
TPM1uf	709.21215	129	1676.424300	1.891200	0.086272	0.427235
TrN	709.24809	129	1676.496180	1.963080	0.083226	0.510461
TPM2uf	709.26369	129	1676.527380	1.994280	0.081938	0.592399
HKY+G	709.26596	129	1676.531920	1.998820	0.081752	0.674151
TIM3	708.88370	130	1677.767400	3.234300	0.044078	0.718229
TPM3uf+G	708.89184	130	1677.783680	3.250580	0.043720	0.761949
TIM1	709.19738	130	1678.394760	3.861660	0.032210	0.794159
TPM1uf+G	709.21548	130	1678.430960	3.897860	0.031632	0.825791
TrN+G	709.24979	130	1678.499580	3.966480	0.030565	0.856356
TIM2	709.25024	130	1678.500480	3.967380	0.030551	0.886908
TPM2uf+G	709.26324	130	1678.526480	3.993380	0.030157	0.917065
TVM	708.73832	131	1679.476640	4.943540	0.018753	0.935817
TIM3+G	708.88216	131	1679.764320	5.231220	0.016240	0.952057
TIM1+G	709.20200	131	1680.404000	5.870900	0.011795	0.963852
TIM2+G	709.24994	131	1680.499880	5.966780	0.011243	0.975095
GTR	708.72882	132	1681.457640	6.924540	0.006965	0.982059
TVM+G	708.73787	132	1681.475740	6.942640	0.006902	0.988961

GTR+G	708.72848	133	1683.456960	8.923860	0.002563	0.991524
K80	717.41834	125	1684.836680	10.303580	0.001286	0.992810
TPM3	716.61110	126	1685.222200	10.689100	0.001060	0.993870
TrNef	716.69590	126	1685.391800	10.858700	0.000974	0.994844
TIM3ef	715.83111	127	1685.662220	11.129120	0.000851	0.995695
TPM2	717.35409	126	1686.708180	12.175080	0.000504	0.996199
K80+G	717.41890	126	1686.837800	12.304700	0.000473	0.996672
TPM1	717.42561	126	1686.851220	12.318120	0.000470	0.997142
TIM2ef	716.62053	127	1687.241060	12.707960	0.000386	0.997528
TPM3+G	716.64816	127	1687.296320	12.763220	0.000376	0.997904
TrNef+G	716.68991	127	1687.379820	12.846720	0.000361	0.998264
TIM1ef	716.69553	127	1687.391060	12.857960	0.000358	0.998623
TIM3ef+G	715.82506	128	1687.650120	13.117020	0.000315	0.998938
TPM2+G	717.34682	127	1688.693640	14.160540	0.000187	0.999125
TPM1+G	717.41854	127	1688.837080	14.303980	0.000174	0.999299
TVMef	716.44544	128	1688.890880	14.357780	0.000169	0.999468
TIM2ef+G	716.61454	128	1689.229080	14.695980	0.000143	0.999611
SYM	715.62256	129	1689.245120	14.712020	0.000142	0.999753
TIM1ef+G	716.68954	128	1689.379080	14.845980	0.000133	0.999886
TVMef+G	716.44711	129	1690.894220	16.361120	6.22e-005	0.999948
SYM+G	715.62298	130	1691.245960	16.712860	5.22e-005	1.000000
F81	783.76257	127	1821.525140	146.992040	2.68e-033	1.000000
F81+G	783.65670	128	1823.313400	148.780300	1.09e-033	1.000000
JC	788.05344	124	1824.106880	149.573780	7.36e-034	1.000000
JC+G	787.94097	125	1825.881940	151.348840	3.03e-034	1.000000

```
-lnL:   negative log likelihod
 K:     number of estimated parameters
 AIC:   Akaike Information Criterion
 delta: AIC difference
 weight:        AIC weight
 cumWeight:     cumulative AIC weight
```

Model selection results also available at the "Model > Show model table" menu

* AIC MODEL SELECTION : Confidence interval

There are 44 models in the 100% confidence interval: [HKY TPM3uf TPM1uf TrN TPM2uf HKY+G TIM3 TPM3uf+G TIM1 TPM1uf+G TrN+G TIM2 TPM2uf+G TVM TIM3+G TIM1+G TIM2+G GTR TVM+G GTR+G K80 TPM3 TrNef TIM3ef TPM2 K80+G TPM1 TIM2ef TPM3+G TrNef+G TIM1ef TIM3ef+G TPM2+G TPM1+G TVMef TIM2ef+G SYM TIM1ef+G TVMef+G SYM+G F81 F81+G JC JC+G]

* AIC MODEL SELECTION : Parameter importance

Parameter	Importance
fA	0.9915
fC	0.9915
fG	0.9915

```
fT          0.9915
kappa       0.3056
titv        0.3056
rAC         0.4162
rAG         0.6944
rAT         0.3538
rCG         0.4242
rCT         0.6944
rGT         0.6944
alpha(G)            0.2688
----------------------
Values have been rounded.
 (I):  considers only +I models.
 (G):  considers only +G models.
 (IG): considers only +I+G models.


* AIC MODEL SELECTION : Model averaged estimates

           Model-averaged
Parameter       estimates
-------------------------
fA             0.2612
fC             0.2059
fG             0.1741
fT             0.3589
kappa         15.1978
titv           7.3838
rAC            0.7373
rAG           13.7755
rAT            0.8945
rCG            0.6741
rCT           14.0541
rGT            1.0000
alpha(G)           24152.3614
-------------------------
Numbers have been rounded.
 (I):  considers only +I models.
 (G):  considers only +G models.
 (IG): considers only +I+G models.


* AIC MODEL SELECTION : Best Model's command line

phyml  -i /tmp/jmodeltest6250482485722261938.phy -d nt -n 1 -b 0 --run_id HKY -m
010010 -f m -c 1 --no_memory_check -o tlr -s BEST



---------------------------------------------------------------
*                                                             *
*             AKAIKE INFORMATION CRITERION (AIC)              *
```

```
*                                                            *
--------------------------------------------------------------

 Model selected: 
   Model = HKY
   partition = 010010
   -lnL = 709.2666
   K = 128
   freqA = 0.2603 
   freqC = 0.2057 
   freqG = 0.1735 
   freqT = 0.3605 
   kappa = 15.2030 (ti/tv = 7.3851)

Tree for the best AIC model = 
(Vi23-4:0.00000001,Vi23-5:0.00000058,(Vi10-5:0.03561308,(Vi12-5:0.01307189,(Br53-
1:0.00000001,((BrM21-1:0.00000051,(Br47-1:0.00000024,(BrM54-1:0.00000001,(BrM22-1
:0.01037483,((Vi23-2:0.02266554,(Vi12-1:0.00000050,Vi23-1:0.01199993):0.00000125)
:0.02462101,((In521:0.01111465,(In501:0.02348288,(In526:0.00000001,(In535:0.01113
005,In556:0.01173030):0.01123786):0.01059872):0.05953795):0.00000065,((Gv10-H-03:
0.02257912,Gv10-C-02:0.00000062):0.01116350,((Gv10-B-06:0.00000001,(Gv10-C-01:0.0
1122578,(Gv10-A-01:0.00000001,((Gv10-A-10:0.01243267,(Gv10-B-03:0.01115861,Gv10-E
-05:0.00000097):0.00000097):0.00000075,(Gv10-F-08:0.01127201,Gv10-B-02:0.01126435
):0.00000067):0.01113747):0.00000060):0.00000069):0.02245354,(In501a:0.00000001,(
Vi10-2:0.04716243,((In534:0.00000001,In553:0.01121676):0.01121676,(Vi10-3:0.02266
601,Vi12-3:0.00000047):0.01117005):0.00000056):0.00000059):0.00000001):0.00000060
):0.00000083):0.00967116):0.08682646):0.01467517):0.00000384):0.00000381):0.20885
083,(((Gv10-A-08:0.00000151,Gv10-F-10:0.00000001):0.02393215,((Br32-1:0.00000047,
((BrK55-1:0.00000093,(BrM48-2:0.01186974,(Br57-1:0.01194923,(Br22-1:0.00000001,(B
r32-2:0.01185144,BrM59-1:0.02310690):0.00000062):0.00000062):0.00000063):0.000000
58):0.01113786,(Br26-2:0.00000001,Br30-1:0.00000061):0.00000061):0.01119346):0.01
145164,(BrM23-1:0.00489765,Br30-2:0.00000064):0.00606830):0.01079505):0.05289481,
(Vi10-1:0.01188110,(BrM56-1:0.01216771,((BrM17-1:0.00000001,BrM45-1:0.03700713):0
.01160172,(BrK11-1:0.00000001,(BrK47-1:0.01110008,(BrM02-1:0.01111694,(BrM51-1:0.
01122866,((BrM31-1:0.01110008,(BrM55-1:0.00000001,(BrM60-3:0.00000001,(BrK02-1:0.
00000047,BrM03-1:0.00000001):0.00000071):0.00000046):0.00000054):0.00000054,(Br48
-1:0.00000001,(BrM26-1:0.01122867,(BrM11-1:0.00543080,Br22-2:0.01318413):0.005695
00):0.00000064):0.00000001):0.00000057):0.00000069):0.00000060):0.01115710):0.000
00060):0.00000087):0.02761076):0.04170545):0.07536507):0.09001970):0.04816843):0.
01031336):0.02329206);


* AIC MODEL SELECTION : Selection uncertainty

Model             -lnL    K       AIC       delta       weight   cumWeight
-------------------------------------------------------------------------
HKY          709.26655   128  1674.533100   0.000000    0.222094   0.222094
TPM3uf       708.89163   129  1675.783260   1.250160    0.118869   0.340963
TPM1uf       709.21215   129  1676.424300   1.891200    0.086272   0.427235
TrN          709.24809   129  1676.496180   1.963080    0.083226   0.510461
TPM2uf       709.26369   129  1676.527380   1.994280    0.081938   0.592399
HKY+G        709.26596   129  1676.531920   1.998820    0.081752   0.674151
```

TIM3	708.88370	130	1677.767400	3.234300	0.044078	0.718229
TPM3uf+G	708.89184	130	1677.783680	3.250580	0.043720	0.761949
TIM1	709.19738	130	1678.394760	3.861660	0.032210	0.794159
TPM1uf+G	709.21548	130	1678.430960	3.897860	0.031632	0.825791
TrN+G	709.24979	130	1678.499580	3.966480	0.030565	0.856356
TIM2	709.25024	130	1678.500480	3.967380	0.030551	0.886908
TPM2uf+G	709.26324	130	1678.526480	3.993380	0.030157	0.917065
TVM	708.73832	131	1679.476640	4.943540	0.018753	0.935817
TIM3+G	708.88216	131	1679.764320	5.231220	0.016240	0.952057
TIM1+G	709.20200	131	1680.404000	5.870900	0.011795	0.963852
TIM2+G	709.24994	131	1680.499880	5.966780	0.011243	0.975095
GTR	708.72882	132	1681.457640	6.924540	0.006965	0.982059
TVM+G	708.73787	132	1681.475740	6.942640	0.006902	0.988961
GTR+G	708.72848	133	1683.456960	8.923860	0.002563	0.991524
K80	717.41834	125	1684.836680	10.303580	0.001286	0.992810
TPM3	716.61110	126	1685.222200	10.689100	0.001060	0.993870
TrNef	716.69590	126	1685.391800	10.858700	0.000974	0.994844
TIM3ef	715.83111	127	1685.662220	11.129120	0.000851	0.995695
TPM2	717.35409	126	1686.708180	12.175080	0.000504	0.996199
K80+G	717.41890	126	1686.837800	12.304700	0.000473	0.996672
TPM1	717.42561	126	1686.851220	12.318120	0.000470	0.997142
TIM2ef	716.62053	127	1687.241060	12.707960	0.000386	0.997528
TPM3+G	716.64816	127	1687.296320	12.763220	0.000376	0.997904
TrNef+G	716.68991	127	1687.379820	12.846720	0.000361	0.998264
TIM1ef	716.69553	127	1687.391060	12.857960	0.000358	0.998623
TIM3ef+G	715.82506	128	1687.650120	13.117020	0.000315	0.998938
TPM2+G	717.34682	127	1688.693640	14.160540	0.000187	0.999125
TPM1+G	717.41854	127	1688.837080	14.303980	0.000174	0.999299
TVMef	716.44544	128	1688.890880	14.357780	0.000169	0.999468
TIM2ef+G	716.61454	128	1689.229080	14.695980	0.000143	0.999611
SYM	715.62256	129	1689.245120	14.712020	0.000142	0.999753
TIM1ef+G	716.68954	128	1689.379080	14.845980	0.000133	0.999886
TVMef+G	716.44711	129	1690.894220	16.361120	6.22e-005	0.999948
SYM+G	715.62298	130	1691.245960	16.712860	5.22e-005	1.000000
F81	783.76257	127	1821.525140	146.992040	2.68e-033	1.000000
F81+G	783.65670	128	1823.313400	148.780300	1.09e-033	1.000000
JC	788.05344	124	1824.106880	149.573780	7.36e-034	1.000000
JC+G	787.94097	125	1825.881940	151.348840	3.03e-034	1.000000

-lnL: negative log likelihod
K: number of estimated parameters
AIC: Akaike Information Criterion
delta: AIC difference
weight: AIC weight
cumWeight: cumulative AIC weight

Model selection results also available at the "Model > Show model table" menu

* AIC MODEL SELECTION : Confidence interval

There are 14 models in the 95% confidence interval: [HKY TPM3uf TPM1uf TrN

TPM2uf HKY+G TIM3 TPM3uf+G TIM1 TPM1uf+G TrN+G TIM2 TPM2uf+G TVM]

```
* AIC MODEL SELECTION : Parameter importance

Parameter   Importance
----------------------
fA          1.0000
fC          1.0000
fG          1.0000
fT          1.0000
kappa       0.3247
titv        0.3247
rAC         0.3933
rAG         0.6753
rAT         0.3329
rCG         0.4013
rCT         0.6753
rGT         0.6753
alpha(G)            0.2328
----------------------
Values have been rounded.
 (I):  considers only +I models.
 (G):  considers only +G models.
 (IG): considers only +I+G models.


* AIC MODEL SELECTION : Model averaged estimates

           Model-averaged
Parameter       estimates
-------------------------
fA             0.2611
fC             0.2059
fG             0.1740
fT             0.3590
kappa         15.2044
titv           7.3858
rAC            0.7414
rAG           13.8755
rAT            0.8961
rCG            0.6861
rCT           14.1166
rGT            1.0000
alpha(G)           23253.9422
-------------------------
Numbers have been rounded.
 (I):  considers only +I models.
 (G):  considers only +G models.
 (IG): considers only +I+G models.
```

* AIC MODEL SELECTION : Best Model's command line

phyml -i /tmp/jmodeltest6250482485722261938.phy -d nt -n 1 -b 0 --run_id HKY -m 010010 -f m -c 1 --no_memory_check -o tlr -s BEST

```
---------------------------------------------------------------
*                                                             *
*                    MODEL AVERAGED PHYLOGENY                 *
*                                                             *
---------------------------------------------------------------
```

Selection criterion: . . . . AIC
Confidence interval: 0.95
Consensus type:. . . . . . . 50% majority rule

Using 15 models in the 0.95 confidence interval = HKY TPM3uf TPM1uf TrN TPM2uf HKY+G TIM3 TPM3uf+G TIM1 TPM1uf+G TrN+G TIM2 TPM2uf+G TVM TIM3+G

Species in order:

1. Vi23-4
2. Vi23-5
3. Vi10-5
4. Vi12-5
5. Br53-1
6. BrM21-1
7. Br47-1
8. BrM54-1
9. BrM22-1
10. Vi23-2
11. Vi12-1
12. Vi23-1
13. In521
14. In501
15. In526
16. In535
17. In556
18. Gv10-H-03
19. Gv10-C-02
20. Gv10-B-06
21. Gv10-C-01
22. Gv10-A-01
23. Gv10-A-10
24. Gv10-B-03
25. Gv10-E-05
26. Gv10-F-08
27. Gv10-B-02
28. In501a
29. Vi10-2

30. In534
31. In553
32. Vi10-3
33. Vi12-3
34. Gv10-A-08
35. Gv10-F-10
36. Br32-1
37. BrK55-1
38. BrM48-2
39. Br57-1
40. Br22-1
41. Br32-2
42. BrM59-1
43. Br26-2
44. Br30-1
45. BrM23-1
46. Br30-2
47. Vi10-1
48. BrM56-1
49. BrM17-1
50. BrM45-1
51. BrK11-1
52. BrK47-1
53. BrM02-1
54. BrM51-1
55. BrM31-1
56. BrM55-1
57. BrM60-3
58. BrK02-1
59. BrM03-1
60. Br48-1
61. BrM26-1
62. BrM11-1
63. Br22-2

Bipartitions included in the consensus tree

```
123456789111111111122222222223333333333444444444455555555556666
         012345678901234567890123456789012345678901234567890123
*************************************************************** ( 1.0 )
--************************************************************* ( 1.0 )
---************************************************************ ( 1.0 )
----*********************************************************** ( 1.0 )
-----********************************************************** ( 1.0 )
-----****************************------------------------------ ( 1.0 )
------***************************------------------------------ ( 0.5682 )
-------**************************------------------------------ ( 0.51682 )
--------*************************------------------------------ ( 1.0 )
---------************************------------------------------ ( 1.0 )
---------***--------------------------------------------------- ( 1.0 )
----------**--------------------------------------------------- ( 0.53695 )
------------*********************------------------------------ ( 1.0 )
```

```
-------------****---------------------------------------------- ( 1.0 )
--------------***---------------------------------------------- ( 1.0 )
---------------**---------------------------------------------- ( 1.0 )
-----------------**-------------------------------------------- ( 1.0 )
-------------------********------------------------------------ ( 1.0 )
--------------------*******------------------------------------ ( 0.69305 )
---------------------******------------------------------------ ( 0.76181 )
----------------------*****------------------------------------ ( 1.0 )
----------------------***-------------------------------------- ( 0.50804 )
-----------------------**-------------------------------------- ( 0.50804 )
-------------------------**------------------------------------ ( 0.50484 )
-----------------------------****------------------------------ ( 0.72688 )
-----------------------------**-------------------------------- ( 1.0 )
-------------------------------**------------------------------ ( 1.0 )
---------------------------------****************************** ( 1.0 )
---------------------------------*************----------------- ( 1.0 )
---------------------------------**---------------------------- ( 1.0 )
-----------------------------------***********----------------- ( 1.0 )
-----------------------------------*********------------------- ( 0.57078 )
------------------------------------********------------------- ( 0.84665 )
--------------------------------------------**----------------- ( 0.57078 )
----------------------------------------------***************** ( 1.0 )
-----------------------------------------------**************** ( 1.0 )
------------------------------------------------*************** ( 1.0 )
------------------------------------------------**------------- ( 1.0 )
--------------------------------------------------************* ( 0.84923 )
---------------------------------------------------************ ( 1.0 )
----------------------------------------------------*********** ( 0.91258 )
-----------------------------------------------------********** ( 0.91258 )
------------------------------------------------------********* ( 1.0 )
------------------------------------------------------*****---- ( 0.53868 )
-------------------------------------------------------****---- ( 1.0 )
--------------------------------------------------------***---- ( 1.0 )
---------------------------------------------------------**---- ( 1.0 )
------------------------------------------------------------*-* ( 0.54874 )
```

```
        +--63 Br30-2
    +-103
    |   +--62 BrM23-1
+-104
|   |       +--61 Br30-1
|   |       |
|   |       +--60 Br26-2
|   |       |
|   |       +--59 BrM59-1
|   |       |
|   |       +--58 Br32-2
|   |   +-101
|   |   |   +--57 Br22-1
|   |   |   |
|   |   |   +--56 Br57-1
```

```
            |   |   |   |
            |   |   |   +--55 BrM48-2
            |   |   |   |
            |   |   |   +--54 BrK55-1
            |   +-102
            |       +--53 Br32-1
        +-105
        |   |   +--52 Gv10-F-10
        |   +-100
        |       +--51 Gv10-A-08
   +--106
   |    |                                                   +--50
BrM03-1
   |    |                                               +--88
   |    |                                               |   +--49
BrK02-1
   |    |                                           +--89
   |    |                                           |   +--48 BrM60-3
   |    |                                       +--90
   |    |                                       |   +--47 BrM55-1
   |    |                                   +--91
   |    |                                   |   +--46 BrM31-1
   |    |                                   |
   |    |                                   |   +--45 Br22-2
   |    |                                   +--87
   |    |                                   |   +--44 BrM26-1
   |    |                               +--92
   |    |                               |   +--43 BrM11-1
   |    |                               |   |
   |    |                               |   +--42 Br48-1
   |    |                           +--93
   |    |                           |   +--41 BrM51-1
   |    |                       +--94
   |    |                       |   +--40 BrM02-1
   |    |                   +--95
   |    |                   |   +--39 BrK47-1
   |    |               +--96
   |    |               |   +--38 BrK11-1
   |    |           +--97
   |    |           |   |   +--37 BrM45-1
   |    |           |   +--86
   |    |           |       +--36 BrM17-1
   |    |       +--98
   |    |       |   +--35 BrM56-1
   |    +--99
   |            +--34 Vi10-1
+---107
|      |                                          +--33 Vi23-1
|      |                                      +--79
|      |                                      |   +--32 Vi12-1
|      |                                  +--80
|      |                                  |   +--31 Vi23-2
```

```
            |     |                         +---81
            |     |                         |    |           +--30 In553
            |     |                         |    |       +--76
            |     |                         |    |       |   +--29 In534
            |     |                         |    |   +--77
            |     |                         |    |   |   |   +--28 Vi12-3
            |     |                         |    |   |   +--75
            |     |                         |    |   |       +--27 Vi10-3
            |     |                         |    |   |
            |     |                         |    |   |                   +--26
Gv10-B-02
            |     |                         |    |   |               +--70
            |     |                         |    |   |               |   +--25
Gv10-F-08
            |     |                         |    |   |           +--71
            |     |                         |    |   |           |   |
+--24 Gv10-E-05
            |     |                         |    |   |           |   |   +--68
            |     |                         |    |   |           |   |   |
+--23 Gv10-B-03
            |     |                         |    |   |           |   +--69
            |     |                         |    |   |           |       +--22
Gv10-A-10
            |     |                         |    |   |       +--72
            |     |                         |    |   |       |   +--21 Gv10-A-01
            |     |                         |    |   |   +--73
            |     |                         |    |   |   |   +--20 Gv10-C-01
            |     |                         |    |   +--74
            |     |                         |    |   |   +--19 Gv10-B-06
            |     |                         |    |   |
            |     |                         |    |   |           +--18 In556
            |     |                         |    |   |       +--65
            |     |                         |    |   |       |   +--17 In535
            |     |                         |    |   |   +--66
            |     |                         |    |   |   |   +--16 In526
            |     |                         |    |   +--67
            |     |                         |    |   |   +--15 In501
            |     |                         |    |   |
            |     |                         |    |   |   +--14 Gv10-C-02
            |     |                         |    |   +--64
            |     |                         |    |   |   +--13 Gv10-H-03
            |     |                         |    +--78
            |     |                         |        +--12 Vi10-2
            |     |                         |        |
            |     |                         |        +--11 In501a
            |     |                         |        |
            |     |                         |        +--10 In521
            |     |                     +--82
            |     |                     |   +---9 BrM22-1
            |     |                 +--83
            |     |                 |   +---8 BrM54-1
            |     |             +--84
```

```
            |     |             |   +---7 Br47-1
            |     +------------85
            |                      +---6 BrM21-1
        +-108
        |   +---5 Br53-1
    +-109
    |   +---4 Vi12-5
+-110
|   +---3 Vi10-5
|
+---2 Vi23-5
|
+---1 Vi23-4
```

(Vi23-4:0.000000,Vi23-5:0.000001,(Vi10-5:0.035613,(Vi12-5:0.013070,(Br53-1:0.0000
00,((BrM21-1:0.000000,(Br47-1:0.000000,(BrM54-1:0.000000,(BrM22-1:0.010375,((In52
1:0.011115,In501a:0.000000,Vi10-2:0.047162,(Gv10-H-03:0.022579,Gv10-C-02:0.000001
)1.00:0.011164,(In501:0.023483,(In526:0.000000,(In535:0.011130,In556:0.011730)1.0
0:0.011238)1.00:0.010599)1.00:0.059538,(Gv10-B-06:0.000000,(Gv10-C-01:0.011226,(G
v10-A-01:0.000000,((Gv10-A-10:0.012433,(Gv10-B-03:0.011159,Gv10-E-05:0.000001)0.5
1:0.000001)0.51:0.000001,(Gv10-F-08:0.011272,Gv10-B-02:0.011264)0.50:0.000001)1.0
0:0.011137)0.76:0.000001)0.69:0.000001)1.00:0.022454,((Vi10-3:0.022666,Vi12-3:0.0
00000)1.00:0.011170,(In534:0.000000,In553:0.011215)1.00:0.011217)0.73:0.000001)1.
00:0.009671,(Vi23-2:0.022666,(Vi12-1:0.000000,Vi23-1:0.012000)0.54:0.000001)1.00:
0.024623)1.00:0.086828)1.00:0.014676)0.52:0.000004)0.57:0.000004)1.00:0.208851,((
Vi10-1:0.011881,(BrM56-1:0.012168,((BrM17-1:0.000000,BrM45-1:0.037007)1.00:0.0116
02,(BrK11-1:0.000000,(BrK47-1:0.011100,(BrM02-1:0.011117,(BrM51-1:0.011229,(Br48-
1:0.000000,BrM11-1:0.011094,(BrM26-1:0.010854,Br22-2:0.013204)0.55:0.000538,(BrM3
1-1:0.011100,(BrM55-1:0.000000,(BrM60-3:0.000000,(BrK02-1:0.000000,BrM03-1:0.0000
00)1.00:0.000001)1.00:0.000000)1.00:0.000001)0.54:0.000001)1.00:0.000001)0.91:0.0
00001)0.91:0.000001)1.00:0.011157)0.85:0.000001)1.00:0.000001)1.00:0.027611)1.00:
0.041705,((Gv10-A-08:0.000002,Gv10-F-10:0.000000)1.00:0.023923,((Br32-1:0.000001,
(BrK55-1:0.000001,BrM48-2:0.011867,Br57-1:0.011939,Br22-1:0.000000,Br32-2:0.01184
9,BrM59-1:0.023106,Br26-2:0.000000,Br30-1:0.000001)0.85:0.011188)0.57:0.011452,(B
rM23-1:0.004903,Br30-2:0.000001)0.57:0.006068)1.00:0.010795)1.00:0.052903)1.00:0.
075406)1.00:0.090020)1.00:0.048168)1.00:0.010313)1.00:0.023292);

Note: this tree is unrooted. Branch lengths are the expected number of substitutions per site. Labels next to parentheses represent phylogenetic uncertainty due to model selection (see documentation)

```
---------------------------------------------------------------
*                                                             *
*                    MODEL AVERAGED PHYLOGENY                 *
*                                                             *
---------------------------------------------------------------
```

Selection criterion: . . . . AIC
Confidence interval: 0.95

Consensus type:. . . . . . . 50% majority rule

Using 15 models in the 0.95 confidence interval = HKY TPM3uf TPM1uf TrN TPM2uf HKY+G TIM3 TPM3uf+G TIM1 TPM1uf+G TrN+G TIM2 TPM2uf+G TVM TIM3+G

Species in order:

1. Vi23-4
2. Vi23-5
3. Vi10-5
4. Vi12-5
5. Br53-1
6. BrM21-1
7. Br47-1
8. BrM54-1
9. BrM22-1
10. Vi23-2
11. Vi12-1
12. Vi23-1
13. In521
14. In501
15. In526
16. In535
17. In556
18. Gv10-H-03
19. Gv10-C-02
20. Gv10-B-06
21. Gv10-C-01
22. Gv10-A-01
23. Gv10-A-10
24. Gv10-B-03
25. Gv10-E-05
26. Gv10-F-08
27. Gv10-B-02
28. In501a
29. Vi10-2
30. In534
31. In553
32. Vi10-3
33. Vi12-3
34. Gv10-A-08
35. Gv10-F-10
36. Br32-1
37. BrK55-1
38. BrM48-2
39. Br57-1
40. Br22-1
41. Br32-2
42. BrM59-1
43. Br26-2
44. Br30-1

```
    45. BrM23-1
    46. Br30-2
    47. Vi10-1
    48. BrM56-1
    49. BrM17-1
    50. BrM45-1
    51. BrK11-1
    52. BrK47-1
    53. BrM02-1
    54. BrM51-1
    55. BrM31-1
    56. BrM55-1
    57. BrM60-3
    58. BrK02-1
    59. BrM03-1
    60. Br48-1
    61. BrM26-1
    62. BrM11-1
    63. Br22-2

Bipartitions included in the consensus tree

    123456789111111111122222222223333333333444444444455555555556666
             012345678901234567890123456789012345678901234567890123
    *************************************************************** ( 1.0 )
    --************************************************************* ( 1.0 )
    ---************************************************************ ( 1.0 )
    ----*********************************************************** ( 1.0 )
    -----********************************************************** ( 1.0 )
    -----****************************------------------------------ ( 1.0 )
    ------***************************------------------------------ ( 0.5682 )
    -------**************************------------------------------ ( 0.51682 )
    --------*************************------------------------------ ( 1.0 )
    ---------************************------------------------------ ( 1.0 )
    ---------***--------------------------------------------------- ( 1.0 )
    ----------**--------------------------------------------------- ( 0.53695 )
    ------------*********************------------------------------ ( 1.0 )
    -------------****---------------------------------------------- ( 1.0 )
    --------------***---------------------------------------------- ( 1.0 )
    ---------------**---------------------------------------------- ( 1.0 )
    -----------------**-------------------------------------------- ( 1.0 )
    -------------------********------------------------------------ ( 1.0 )
    --------------------*******------------------------------------ ( 0.69305 )
    ---------------------******------------------------------------ ( 0.76181 )
    ----------------------*****------------------------------------ ( 1.0 )
    ----------------------***-------------------------------------- ( 0.50804 )
    -----------------------**-------------------------------------- ( 0.50804 )
    -------------------------**------------------------------------ ( 0.50484 )
    -----------------------------****------------------------------ ( 0.72688 )
    -----------------------------**-------------------------------- ( 1.0 )
    -------------------------------**------------------------------ ( 1.0 )
    ---------------------------------****************************** ( 1.0 )
```

```
---------------------------------*************----------------- ( 1.0 )
---------------------------------**---------------------------- ( 1.0 )
-----------------------------------***********----------------- ( 1.0 )
-----------------------------------*********------------------- ( 0.57078 )
------------------------------------********------------------- ( 0.84665 )
--------------------------------------------**----------------- ( 0.57078 )
----------------------------------------------***************** ( 1.0 )
-----------------------------------------------**************** ( 1.0 )
------------------------------------------------*************** ( 1.0 )
------------------------------------------------**------------- ( 1.0 )
--------------------------------------------------************* ( 0.84923 )
---------------------------------------------------************ ( 1.0 )
----------------------------------------------------*********** ( 0.91258 )
-----------------------------------------------------********** ( 0.91258 )
------------------------------------------------------********* ( 1.0 )
------------------------------------------------------*****---- ( 0.53868 )
-------------------------------------------------------****---- ( 1.0 )
--------------------------------------------------------***---- ( 1.0 )
---------------------------------------------------------**---- ( 1.0 )
------------------------------------------------------------*-* ( 0.54874 )


                                   +--63 Br30-2
                               +-103
                               |   +--62 BrM23-1
                           +-104
                           |   |       +--61 Br30-1
                           |   |       |
                           |   |       +--60 Br26-2
                           |   |       |
                           |   |       +--59 BrM59-1
                           |   |       |
                           |   |       +--58 Br32-2
                           |   |   +-101
                           |   |   |   +--57 Br22-1
                           |   |   |   |
                           |   |   |   +--56 Br57-1
                           |   |   |   |
                           |   |   |   +--55 BrM48-2
                           |   |   |   |
                           |   |   |   +--54 BrK55-1
                           |   +-102
                           |       +--53 Br32-1
                       +-105
                       |   |   +--52 Gv10-F-10
                       |   +-100
                       |       +--51 Gv10-A-08
                  +--106
                  |    |                                               +--50
BrM03-1
                  |    |                                           +--88
                  |    |                                           |   +--49
```

```
BrK02-1
                |    |                                                +--89
                |    |                                                |   +--48 BrM60-3
                |    |                                            +--90
                |    |                                            |   +--47 BrM55-1
                |    |                                        +--91
                |    |                                        |   +--46 BrM31-1
                |    |                                        |
                |    |                                        |   +--45 Br22-2
                |    |                                        +--87
                |    |                                        |   +--44 BrM26-1
                |    |                                    +--92
                |    |                                    |   +--43 BrM11-1
                |    |                                    |   |
                |    |                                    |   +--42 Br48-1
                |    |                                +--93
                |    |                                |   +--41 BrM51-1
                |    |                            +--94
                |    |                            |   +--40 BrM02-1
                |    |                        +--95
                |    |                        |   +--39 BrK47-1
                |    |                    +--96
                |    |                    |   +--38 BrK11-1
                |    |                +--97
                |    |                |   |   +--37 BrM45-1
                |    |                |   +--86
                |    |                |       +--36 BrM17-1
                |    |            +--98
                |    |            |   +--35 BrM56-1
                |    +--99
                |        +--34 Vi10-1
          +---107
          |     |                                                 +--33 Vi23-1
          |     |                                             +--79
          |     |                                             |   +--32 Vi12-1
          |     |                                         +--80
          |     |                                         |   +--31 Vi23-2
          |     |                                    +---81
          |     |                                    |    |           +--30 In553
          |     |                                    |    |       +--76
          |     |                                    |    |       |   +--29 In534
          |     |                                    |    |   +--77
          |     |                                    |    |   |   |   +--28 Vi12-3
          |     |                                    |    |   |   +--75
          |     |                                    |    |   |       +--27 Vi10-3
          |     |                                    |    |   |
          |     |                                    |    |   |                       +--26
Gv10-B-02
          |     |                                    |    |   |                   +--70
          |     |                                    |    |   |                   |   +--25
Gv10-F-08
          |     |                                    |    |   |               +--71
```

```
            |     |                         |    |   |           |   |
+--24 Gv10-E-05
            |     |                         |    |   |           |   |   +--68
            |     |                         |    |   |           |   |   |
+--23 Gv10-B-03
            |     |                         |    |   |           |   +--69
            |     |                         |    |   |           |       +--22
Gv10-A-10
            |     |                         |    |   |       +--72
            |     |                         |    |   |       |   +--21 Gv10-A-01
            |     |                         |    |   |   +--73
            |     |                         |    |   |   |   +--20 Gv10-C-01
            |     |                         |    |   +--74
            |     |                         |    |   |   +--19 Gv10-B-06
            |     |                         |    |   |
            |     |                         |    |   |           +--18 In556
            |     |                         |    |   |       +--65
            |     |                         |    |   |       |   +--17 In535
            |     |                         |    |   |   +--66
            |     |                         |    |   |   |   +--16 In526
            |     |                         |    |   +--67
            |     |                         |    |   |   +--15 In501
            |     |                         |    |   |
            |     |                         |    |   |   +--14 Gv10-C-02
            |     |                         |    |   +--64
            |     |                         |    |   |   +--13 Gv10-H-03
            |     |                         |    +--78
            |     |                         |        +--12 Vi10-2
            |     |                         |        |
            |     |                         |        +--11 In501a
            |     |                         |        |
            |     |                         |        +--10 In521
            |     |                     +--82
            |     |                     |   +---9 BrM22-1
            |     |                 +--83
            |     |                 |   +---8 BrM54-1
            |     |             +--84
            |     |             |   +---7 Br47-1
            |     +------------85
            |                   +---6 BrM21-1
        +-108
        |   +---5 Br53-1
    +-109
    |   +---4 Vi12-5
+-110
|   +---3 Vi10-5
|
+---2 Vi23-5
|
+---1 Vi23-4
```

(Vi23-4:0.000000,Vi23-5:0.000001,(Vi10-5:0.035613,(Vi12-5:0.013070,(Br53-1:0.000000,((BrM21-1:0.000000,(Br47-1:0.000000,(BrM54-1:0.000000,(BrM22-1:0.010375,((In521:0.011115,In501a:0.000000,Vi10-2:0.047162,(Gv10-H-03:0.022579,Gv10-C-02:0.000001)1.00:0.011164,(In501:0.023483,(In526:0.000000,(In535:0.011130,In556:0.011730)1.00:0.011238)1.00:0.010599)1.00:0.059538,(Gv10-B-06:0.000000,(Gv10-C-01:0.011226,(Gv10-A-01:0.000000,((Gv10-A-10:0.012433,(Gv10-B-03:0.011159,Gv10-E-05:0.000001)0.51:0.000001)0.51:0.000001,(Gv10-F-08:0.011272,Gv10-B-02:0.011264)0.50:0.000001)1.00:0.011137)0.76:0.000001)0.69:0.000001)1.00:0.022454,((Vi10-3:0.022666,Vi12-3:0.000000)1.00:0.011170,(In534:0.000000,In553:0.011215)1.00:0.011217)0.73:0.000001)1.00:0.009671,(Vi23-2:0.022666,(Vi12-1:0.000000,Vi23-1:0.012000)0.54:0.000001)1.00:0.024623)1.00:0.086828)1.00:0.014676)0.52:0.000004)0.57:0.000004)1.00:0.208851,((Vi10-1:0.011881,(BrM56-1:0.012168,((BrM17-1:0.000000,BrM45-1:0.037007)1.00:0.011602,(BrK11-1:0.000000,(BrK47-1:0.011100,(BrM02-1:0.011117,(BrM51-1:0.011229,(Br48-1:0.000000,BrM11-1:0.011094,(BrM26-1:0.010854,Br22-2:0.013204)0.55:0.000538,(BrM31-1:0.011100,(BrM55-1:0.000000,(BrM60-3:0.000000,(BrK02-1:0.000000,BrM03-1:0.000000)1.00:0.000001)1.00:0.000000)1.00:0.000001)0.54:0.000001)1.00:0.000001)0.91:0.000001)0.91:0.000001)1.00:0.011157)0.85:0.000001)1.00:0.000001)1.00:0.027611)1.00:0.041705,((Gv10-A-08:0.000002,Gv10-F-10:0.000000)1.00:0.023923,((Br32-1:0.000001,(BrK55-1:0.000001,BrM48-2:0.011867,Br57-1:0.011939,Br22-1:0.000000,Br32-2:0.011849,BrM59-1:0.023106,Br26-2:0.000000,Br30-1:0.000001)0.85:0.011188)0.57:0.011452,(BrM23-1:0.004903,Br30-2:0.000001)0.57:0.006068)1.00:0.010795)1.00:0.052903)1.00:0.075406)1.00:0.090020)1.00:0.048168)1.00:0.010313)1.00:0.023292);

Note: this tree is unrooted. Branch lengths are the expected number of substitutions per site. Labels next to parentheses represent phylogenetic uncertainty due to model selection (see documentation)

```
------------------------- jModeltest 2.1.7 v20150530 --------------------------
(c) 2011-onwards D. Darriba, G.L. Taboada, R. Doallo and D. Posada,
(1) Department of Biochemistry, Genetics and Immunology
    University of Vigo, 36310 Vigo, Spain.
(2) Department of Electronics and Systems
    University of A Coruna, 15071 A Coruna, Spain.
e-mail: ddarriba@udc.es, dposada@uvigo.es
--------------------------------------------------------------------------------

Wed Jun 24 12:30:25 AEST 2015
Linux 3.2.0-76-virtual, arch: amd64, bits: 64, numcores: 16

jModelTest 2.1.7 v20150530
Copyright (C) 2011 D. Darriba, G.L. Taboada, R. Doallo and D. Posada
This program comes with ABSOLUTELY NO WARRANTY
This is free software, and you are welcome to redistribute it under certain
conditions

Notice: This program may contain errors. Please inspect results carefully.

--------------------------------------------------------------------------------
Citation: Darriba D, Taboada GL, Doallo R and Posada D. 2012.
          "jModelTest 2: more models, new heuristics and parallel computing".
          Nature Methods 9(8), 772.
--------------------------------------------------------------------------------

Reading data file "S2_File_relaxed_phylip_format_nuclearSNPs.phy"... OK.
  number of sequences: 80
  number of sites: 5815


---------------------------------------------------------------
*                                                             *
*        COMPUTATION OF LIKELIHOOD SCORES WITH PHYML          *
*                                                             *
---------------------------------------------------------------

::Settings::

 Phyml version = 3.0
 Phyml binary = PhyML_3.0_linux64
 Phyml path = /usr/local/bin/jmodeltest-2.1.7/exe/phyml/
 Candidate models = 44
   number of substitution schemes = 11
   including models with equal/unequal base frequencies (+F)
   including only models without a proportion of invariable sites
   including models with/without rate variation among sites (+G) (nCat = 4)
 Optimized free parameters (K) = substitution parameters + 157 branch lengths +
topology
 Base tree for likelihood calculations = ML tree
 Tree topology search operation = BEST
```

::Progress::

Model	Exec. Time	Total Time	-lnL
TPM3uf+G	00h:55:06:05	00h:55:08:04	61658.0498
TVM+G	01h:01:05:07	01h:01:08:01	61558.8827
GTR+G	01h:01:50:02	01h:01:52:00	61558.1060
TPM1uf+G	01h:02:18:02	01h:02:20:04	61734.2289
TrNef+G	01h:04:03:06	01h:04:06:03	61736.9019
TrN+G	01h:04:43:09	01h:04:46:07	61733.8373
JC+G	01h:06:15:00	01h:06:16:04	65022.9432
TIM2+G	01h:06:35:00	01h:06:36:06	61625.6673
F81+G	01h:06:34:09	01h:06:37:08	64978.4529
TPM1+G	01h:08:36:02	01h:08:37:06	61729.7674
TPM2uf+G	01h:11:26:00	01h:11:27:08	61641.0374
TIM3+G	01h:23:42:06	01h:23:44:06	61677.9402
F81	00h:21:57:01	01h:28:13:05	71202.2927
TPM2uf	00h:18:04:07	01h:29:32:05	68310.1535
TPM1uf	00h:22:42:06	01h:31:20:03	68377.7152
TrN	00h:27:58:06	01h:34:36:05	68408.1535
HKY	00h:29:07:02	01h:35:43:08	68368.7775
TPM3uf	00h:17:17:07	01h:41:02:03	68324.2042
HKY+G	01h:47:53:07	01h:47:55:00	61734.2389
TIM1+G	01h:47:57:05	01h:47:58:09	61732.7638
TIM3	00h:22:49:06	01h:54:09:09	68295.5642
TIM2	00h:26:45:09	01h:56:18:05	68270.6044
TIM1	00h:30:08:06	01h:58:22:02	68372.8865
JC	00h:18:04:01	01h:59:06:04	71122.5971
TVM	00h:26:00:07	02h:00:37:02	68184.6193
GTR	00h:26:56:02	02h:02:40:00	68181.7387
K80	00h:19:58:01	02h:07:53:02	68334.1545
TPM2+G	02h:08:02:05	02h:08:03:09	61656.4629
TPM3+G	01h:13:06:08	02h:08:15:02	61674.9791
TrNef	00h:20:18:08	02h:08:17:08	68298.2962
TPM2	00h:17:06:08	02h:13:25:03	68215.1133
TPM1	00h:19:28:07	02h:13:38:06	68301.0598
TVMef+G	01h:09:55:01	02h:14:01:04	61603.8177
SYM+G	01h:13:06:07	02h:17:53:04	61566.0936
TIM3ef	00h:18:21:07	02h:21:01:08	68266.4356
TIM2ef	00h:21:14:07	02h:21:51:09	68182.2332
TPM3	00h:25:06:04	02h:23:28:05	68269.2482
SYM	00h:16:15:05	02h:24:19:05	68116.7293
TIM1ef	00h:27:55:07	02h:27:02:01	68331.5166
TVMef	00h:20:45:02	02h:28:38:04	68107.7087
TIM3ef+G	01h:26:19:09	02h:28:40:04	61655.2850
TIM2ef+G	01h:33:15:04	02h:35:07:05	61634.5858
TIM1ef+G	01h:39:25:09	02h:40:34:01	61739.0038
K80+G	02h:54:17:08	02h:54:19:04	61737.3571

::Results::

Model = JC
partition = 000000
-lnL = 71122.5971
K = 158

Model = JC+G
partition = 000000
-lnL = 65022.9431
K = 159
gamma shape = 0.3100

Model = F81
partition = 000000
-lnL = 71202.2927
K = 161
freqA = 0.2651
freqC = 0.2409
freqG = 0.2401
freqT = 0.2540

Model = F81+G
partition = 000000
-lnL = 64978.4529
K = 162
freqA = 0.2740
freqC = 0.2346
freqG = 0.2350
freqT = 0.2564
gamma shape = 0.3100

Model = K80
partition = 010010
-lnL = 68334.1545
K = 159
kappa = 4.3761 (ti/tv = 2.1881)

Model = K80+G
partition = 010010
-lnL = 61737.3571
K = 160
kappa = 5.2739 (ti/tv = 2.6369)
gamma shape = 0.2930

Model = HKY
partition = 010010
-lnL = 68368.7774
K = 162
freqA = 0.2579
freqC = 0.2519
freqG = 0.2439
freqT = 0.2463
kappa = 4.3939 (ti/tv = 2.1960)

```
Model = HKY+G
partition = 010010
-lnL = 61734.2389
K = 163
freqA = 0.2686
freqC = 0.2412
freqG = 0.2336
freqT = 0.2566
kappa = 5.2131 (ti/tv = 2.5990)
gamma shape = 0.2940

Model = TrNef
partition = 010020
-lnL = 68298.2962
K = 160
R(a) [AC] =  1.0000
R(b) [AG] =  4.5470
R(c) [AT] =  1.0000
R(d) [CG] =  1.0000
R(e) [CT] =  4.2985
R(f) [GT] =  1.0000

Model = TrNef+G
partition = 010020
-lnL = 61736.9019
K = 161
R(a) [AC] =  1.0000
R(b) [AG] =  5.3795
R(c) [AT] =  1.0000
R(d) [CG] =  1.0000
R(e) [CT] =  5.1697
R(f) [GT] =  1.0000
gamma shape = 0.2930

Model = TrN
partition = 010020
-lnL = 68408.1535
K = 163
freqA = 0.2554
freqC = 0.2539
freqG = 0.2431
freqT = 0.2476
R(a) [AC] =  1.0000
R(b) [AG] =  4.4915
R(c) [AT] =  1.0000
R(d) [CG] =  1.0000
R(e) [CT] =  4.2917
R(f) [GT] =  1.0000

Model = TrN+G
partition = 010020
```

```
-lnL = 61733.8373
K = 164
freqA = 0.2668
freqC = 0.2429
freqG = 0.2320
freqT = 0.2584
R(a) [AC] =  1.0000
R(b) [AG] =  5.3138
R(c) [AT] =  1.0000
R(d) [CG] =  1.0000
R(e) [CT] =  5.1132
R(f) [GT] =  1.0000
gamma shape = 0.2940

Model = TPM1
partition = 012210
-lnL = 68301.0598
K = 160
R(a) [AC] =  1.0000
R(b) [AG] =  4.4152
R(c) [AT] =  0.9962
R(d) [CG] =  0.9962
R(e) [CT] =  4.4152
R(f) [GT] =  1.0000

Model = TPM1+G
partition = 012210
-lnL = 61729.7674
K = 161
R(a) [AC] =  1.0000
R(b) [AG] =  5.2854
R(c) [AT] =  1.0008
R(d) [CG] =  1.0008
R(e) [CT] =  5.2854
R(f) [GT] =  1.0000
gamma shape = 0.2930

Model = TPM1uf
partition = 012210
-lnL = 68377.7152
K = 163
freqA = 0.2576
freqC = 0.2507
freqG = 0.2461
freqT = 0.2457
R(a) [AC] =  1.0000
R(b) [AG] =  4.3788
R(c) [AT] =  1.0068
R(d) [CG] =  1.0068
R(e) [CT] =  4.3788
R(f) [GT] =  1.0000
```

Model = TPM1uf+G
partition = 012210
-lnL = 61734.2289
K = 164
freqA = 0.2686
freqC = 0.2412
freqG = 0.2336
freqT = 0.2566
R(a) [AC] = 1.0000
R(b) [AG] = 5.2272
R(c) [AT] = 1.0055
R(d) [CG] = 1.0055
R(e) [CT] = 5.2272
R(f) [GT] = 1.0000
gamma shape = 0.2940

Model = TPM2
partition = 010212
-lnL = 68215.1133
K = 160
R(a) [AC] = 1.7362
R(b) [AG] = 5.9297
R(c) [AT] = 1.7362
R(d) [CG] = 1.0000
R(e) [CT] = 5.9297
R(f) [GT] = 1.0000

Model = TPM2+G
partition = 010212
-lnL = 61656.4629
K = 161
R(a) [AC] = 1.7511
R(b) [AG] = 7.1695
R(c) [AT] = 1.7511
R(d) [CG] = 1.0000
R(e) [CT] = 7.1695
R(f) [GT] = 1.0000
gamma shape = 0.2940

Model = TPM2uf
partition = 010212
-lnL = 68310.1535
K = 163
freqA = 0.2394
freqC = 0.2519
freqG = 0.2620
freqT = 0.2467
R(a) [AC] = 1.7894
R(b) [AG] = 5.9917
R(c) [AT] = 1.7894
R(d) [CG] = 1.0000
R(e) [CT] = 5.9917

R(f) [GT] = 1.0000

Model = TPM2uf+G
partition = 010212
-lnL = 61641.0374
K = 164
freqA = 0.2421
freqC = 0.2413
freqG = 0.2589
freqT = 0.2577
R(a) [AC] = 1.8427
R(b) [AG] = 7.2675
R(c) [AT] = 1.8427
R(d) [CG] = 1.0000
R(e) [CT] = 7.2675
R(f) [GT] = 1.0000
gamma shape = 0.2960

Model = TPM3
partition = 012012
-lnL = 68269.2482
K = 160
R(a) [AC] = 0.6607
R(b) [AG] = 3.6092
R(c) [AT] = 1.0000
R(d) [CG] = 0.6607
R(e) [CT] = 3.6092
R(f) [GT] = 1.0000

Model = TPM3+G
partition = 012012
-lnL = 61674.9791
K = 161
R(a) [AC] = 0.6686
R(b) [AG] = 4.3651
R(c) [AT] = 1.0000
R(d) [CG] = 0.6686
R(e) [CT] = 4.3651
R(f) [GT] = 1.0000
gamma shape = 0.2940

Model = TPM3uf
partition = 012012
-lnL = 68324.2042
K = 163
freqA = 0.2572
freqC = 0.2655
freqG = 0.2460
freqT = 0.2314
R(a) [AC] = 0.6106
R(b) [AG] = 3.4656
R(c) [AT] = 1.0000

R(d) [CG] = 0.6106
R(e) [CT] = 3.4656
R(f) [GT] = 1.0000

Model = TPM3uf+G
partition = 012012
-lnL = 61658.0498
K = 164
freqA = 0.2691
freqC = 0.2624
freqG = 0.2335
freqT = 0.2351
R(a) [AC] = 0.6190
R(b) [AG] = 4.1696
R(c) [AT] = 1.0000
R(d) [CG] = 0.6190
R(e) [CT] = 4.1696
R(f) [GT] = 1.0000
gamma shape = 0.2950

Model = TIM1ef
partition = 012230
-lnL = 68331.5166
K = 161
R(a) [AC] = 1.0000
R(b) [AG] = 4.4943
R(c) [AT] = 0.9998
R(d) [CG] = 0.9998
R(e) [CT] = 4.2550
R(f) [GT] = 1.0000

Model = TIM1ef+G
partition = 012230
-lnL = 61739.0038
K = 162
R(a) [AC] = 1.0000
R(b) [AG] = 5.3652
R(c) [AT] = 0.9957
R(d) [CG] = 0.9957
R(e) [CT] = 5.1514
R(f) [GT] = 1.0000
gamma shape = 0.2930

Model = TIM1
partition = 012230
-lnL = 68372.8865
K = 164
freqA = 0.2542
freqC = 0.2547
freqG = 0.2416
freqT = 0.2496
R(a) [AC] = 1.0000

```
R(b) [AG] =  4.5441
R(c) [AT] =  1.0142
R(d) [CG] =  1.0142
R(e) [CT] =  4.2981
R(f) [GT] =  1.0000

Model = TIM1+G
partition = 012230
-lnL = 61732.7638
K = 165
freqA = 0.2667
freqC = 0.2427
freqG = 0.2321
freqT = 0.2585
R(a) [AC] =  1.0000
R(b) [AG] =  5.3456
R(c) [AT] =  1.0063
R(d) [CG] =  1.0063
R(e) [CT] =  5.1183
R(f) [GT] =  1.0000
gamma shape = 0.2940

Model = TIM2ef
partition = 010232
-lnL = 68182.2332
K = 161
R(a) [AC] =  1.7307
R(b) [AG] =  6.0785
R(c) [AT] =  1.7307
R(d) [CG] =  1.0000
R(e) [CT] =  5.7367
R(f) [GT] =  1.0000

Model = TIM2ef+G
partition = 010232
-lnL = 61634.5858
K = 162
R(a) [AC] =  1.7462
R(b) [AG] =  7.2513
R(c) [AT] =  1.7462
R(d) [CG] =  1.0000
R(e) [CT] =  7.0416
R(f) [GT] =  1.0000
gamma shape = 0.2940

Model = TIM2
partition = 010232
-lnL = 68270.6044
K = 164
freqA = 0.2346
freqC = 0.2563
freqG = 0.2593
```

freqT = 0.2499
R(a) [AC] = 1.8030
R(b) [AG] = 6.3029
R(c) [AT] = 1.8030
R(d) [CG] = 1.0000
R(e) [CT] = 5.8036
R(f) [GT] = 1.0000

Model = TIM2+G
partition = 010232
-lnL = 61625.6673
K = 165
freqA = 0.2411
freqC = 0.2435
freqG = 0.2568
freqT = 0.2587
R(a) [AC] = 1.8378
R(b) [AG] = 7.3979
R(c) [AT] = 1.8378
R(d) [CG] = 1.0000
R(e) [CT] = 7.1540
R(f) [GT] = 1.0000
gamma shape = 0.2960

Model = TIM3ef
partition = 012032
-lnL = 68266.4356
K = 161
R(a) [AC] = 0.6609
R(b) [AG] = 3.7113
R(c) [AT] = 1.0000
R(d) [CG] = 0.6609
R(e) [CT] = 3.5075
R(f) [GT] = 1.0000

Model = TIM3ef+G
partition = 012032
-lnL = 61655.2850
K = 162
R(a) [AC] = 0.6700
R(b) [AG] = 4.4754
R(c) [AT] = 1.0000
R(d) [CG] = 0.6700
R(e) [CT] = 4.2547
R(f) [GT] = 1.0000
gamma shape = 0.2940

Model = TIM3
partition = 012032
-lnL = 68295.5642
K = 164
freqA = 0.2530

```
freqC = 0.2685
freqG = 0.2450
freqT = 0.2335
R(a) [AC] =  0.6112
R(b) [AG] =  3.5482
R(c) [AT] =  1.0000
R(d) [CG] =  0.6112
R(e) [CT] =  3.4163
R(f) [GT] =  1.0000

Model = TIM3+G
partition = 012032
-lnL = 61677.9402
K = 165
freqA = 0.2667
freqC = 0.2637
freqG = 0.2320
freqT = 0.2375
R(a) [AC] =  0.6157
R(b) [AG] =  4.2209
R(c) [AT] =  1.0000
R(d) [CG] =  0.6157
R(e) [CT] =  4.0620
R(f) [GT] =  1.0000
gamma shape = 0.2960

Model = TVMef
partition = 012314
-lnL = 68107.7087
K = 162
R(a) [AC] =  1.1301
R(b) [AG] =  4.7178
R(c) [AT] =  1.6466
R(d) [CG] =  0.6185
R(e) [CT] =  4.7178
R(f) [GT] =  1.0000

Model = TVMef+G
partition = 012314
-lnL = 61603.8177
K = 163
R(a) [AC] =  1.1715
R(b) [AG] =  5.7355
R(c) [AT] =  1.6549
R(d) [CG] =  0.6101
R(e) [CT] =  5.7355
R(f) [GT] =  1.0000
gamma shape = 0.2960

Model = TVM
partition = 012314
-lnL = 68184.6193
```

```
K = 165
freqA = 0.2393
freqC = 0.2667
freqG = 0.2631
freqT = 0.2310
R(a) [AC] =  1.1020
R(b) [AG] =  4.6451
R(c) [AT] =  1.7205
R(d) [CG] =  0.5723
R(e) [CT] =  4.6451
R(f) [GT] =  1.0000

Model = TVM+G
partition = 012314
-lnL = 61558.8827
K = 166
freqA = 0.2418
freqC = 0.2625
freqG = 0.2602
freqT = 0.2356
R(a) [AC] =  1.1354
R(b) [AG] =  5.6417
R(c) [AT] =  1.7373
R(d) [CG] =  0.5643
R(e) [CT] =  5.6417
R(f) [GT] =  1.0000
gamma shape = 0.2960

Model = SYM
partition = 012345
-lnL = 68116.7293
K = 163
R(a) [AC] =  1.1336
R(b) [AG] =  4.7835
R(c) [AT] =  1.6399
R(d) [CG] =  0.6070
R(e) [CT] =  4.5802
R(f) [GT] =  1.0000

Model = SYM+G
partition = 012345
-lnL = 61566.0936
K = 164
R(a) [AC] =  1.1638
R(b) [AG] =  5.8354
R(c) [AT] =  1.6479
R(d) [CG] =  0.6129
R(e) [CT] =  5.5773
R(f) [GT] =  1.0000
gamma shape = 0.2950

Model = GTR
```

```
   partition = 012345
   -lnL = 68181.7387
   K = 166
   freqA = 0.2352
   freqC = 0.2711
   freqG = 0.2586
   freqT = 0.2351
   R(a) [AC] =  1.1060
   R(b) [AG] =  4.8345
   R(c) [AT] =  1.7226
   R(d) [CG] =  0.5732
   R(e) [CT] =  4.4753
   R(f) [GT] =  1.0000

   Model = GTR+G
   partition = 012345
   -lnL = 61558.1060
   K = 167
   freqA = 0.2395
   freqC = 0.2649
   freqG = 0.2577
   freqT = 0.2378
   R(a) [AC] =  1.1361
   R(b) [AG] =  5.7950
   R(c) [AT] =  1.7371
   R(d) [CG] =  0.5645
   R(e) [CT] =  5.4939
   R(f) [GT] =  1.0000
   gamma shape = 0.2960


Computation of likelihood scores completed. It took 02h:54:20:00.




---------------------------------------------------------------
*                                                             *
*              AKAIKE INFORMATION CRITERION (AIC)             *
*                                                             *
---------------------------------------------------------------

 Model selected: 
   Model = TVM+G
   partition = 012314
   -lnL = 61558.8827
   K = 166
   freqA = 0.2418
   freqC = 0.2625
   freqG = 0.2602
   freqT = 0.2356
   R(a) [AC] =  1.1354
```

```
   R(b) [AG] =  5.6417
   R(c) [AT] =  1.7373
   R(d) [CG] =  0.5643
   R(e) [CT] =  5.6417
   R(f) [GT] =  1.0000
   gamma shape = 0.2960
```

Tree for the best AIC model =
(BrM57-1:0.05184835,BrM52-1:0.02481505,((Br22-1:0.02667606,(BrK47-1:0.02701996,Br
53-1:0.04815373):0.02208776):0.01871902,(BrM55-1:0.04000213,(((Br32-2:0.01240354,
Br32-1:0.00642996):0.04154640,(((((BrM54-1:0.02701382,(BrM03-1:0.02438801,BrM48-1
:0.01571118):0.01712576):0.00396741,(BrK01-1:0.03076228,Br48-1:0.02813910):0.0166
3572):0.00652506,(BrM23-1:0.02953622,BrK55-1:0.04354231):0.00851064):0.00873969,(
((BrM17-1:0.04790729,(((BrM21-1:0.02014617,BrM46-1:0.01850240):0.00955021,(BrM11-
1:0.02447278,(BrM02-1:0.03426506,BrM45-1:0.02159593):0.01278838):0.00941077):0.01
133245,(BrK60-1:0.02767988,BrM27-1:0.05315693):0.01126936):0.00430149):0.01542212
,(BrM51-1:0.02858213,(BrM60-3:0.03202376,BrM56-1:0.02381812):0.00978972):0.012414
28):0.00832603,(((Gv10-C-05:0.03742512,(((Gv10-F-02:0.03588325,(Gv10-F-10:0.02018
756,Gv10-C-01:0.02574039):0.00621799):0.00878015,(Gv10-H-05:0.05069153,(Gv10-C-10
:0.03051322,Gv10-B-10:0.02649966):0.00868768):0.01495560):0.00999886,(((Gv10-A-01
:0.02576386,Gv10-F-08:0.02650691):0.01317507,(Gv10-E-05:0.02155003,Gv10-B-02:0.02
565866):0.02025356):0.00726833,(Gv10-H-03:0.03871319,(Gv10-B-06:0.03513156,(Gv10-
H-07:0.02167510,(Gv10-A-10:0.02247929,Gv10-A-08:0.04187486):0.01247824):0.0088060
7):0.00383106):0.01138859):0.00964483):0.01929322):0.01833202,(Vi10-2:0.04823806,
((((In527:0.02881058,In501a:0.03531808):0.00547463,(In526:0.02679181,(In521:0.029
85924,(In553:0.01972704,In556:0.01803529):0.00578453):0.01577886):0.00702218):0.0
0812514,((In525:0.03279834,(In534:0.02107552,In547:0.02513353):0.00995907):0.0079
4735,(In535:0.02528658,In501:0.07093160):0.01316442):0.00653160):0.02934495,(Vi10
-3:0.03290398,(((Vi12-1:0.02855969,Vi23-1:0.03010831):0.01518933,(Vi12-3:0.034653
53,(Vi10-5:0.02521269,(Vi23-4:0.00274956,Vi23-5:0.00346170):0.03588859):0.0111435
3):0.00630121):0.00719443,(Vi12-5:0.03024567,Vi10-1:0.02404323):0.01317145):0.009
58228):0.00963673):0.01494860):0.00928272):0.05962352,((BrM22-1:0.03659096,(Br47-
1:0.00276123,Br47-2:0.00381655):0.04294608):0.00868814,(BrM59-1:0.02168672,(BrK04
-1:0.02484032,BrK02-1:0.03544618):0.01089521):0.00765011):0.00655924):0.00666815)
:0.00811131):0.00454601,(Br22-2:0.03157420,((Br46-1:0.00594556,Br46-2:0.00261128)
:0.04079829,(BrK06-1:0.03215895,(Br30-1:0.00631662,Br30-2:0.00176267):0.04133231)
:0.01201340):0.01266911):0.01223314):0.00432845):0.00571023,(BrM50-1:0.06126366,(
(Br26-2:0.00195747,Br26-1:0.00928642):0.02330916,(Br57-1:0.00118969,Br57-2:0.0060
3894):0.01954434):0.03123921):0.01782641):0.01004522):0.01302960):0.03111852);

* AIC MODEL SELECTION : Selection uncertainty

Model	-lnL	K	AIC	delta	weight	cumWeight
TVM+G	61558.88268	166	123449.765360	0.000000	0.553915	0.553915
GTR+G	61558.10598	167	123450.211960	0.446600	0.443063	0.996977
SYM+G	61566.09357	164	123460.187140	10.421780	0.003023	1.000000
TVMef+G	61603.81770	163	123533.635400	83.870040	3.40e-019	1.000000
TIM2+G	61625.66732	165	123581.334640	131.569280	1.49e-029	1.000000
TIM2ef+G	61634.58579	162	123593.171580	143.406220	4.01e-032	1.000000
TPM2uf+G	61641.03736	164	123610.074720	160.309360	8.56e-036	1.000000

TIM3ef+G	61655.28503	162	123634.570060	184.804700	4.11e-041	1.000000
TPM2+G	61656.46286	161	123634.925720	185.160360	3.44e-041	1.000000
TPM3uf+G	61658.04982	164	123644.099640	194.334280	3.50e-043	1.000000
TPM3+G	61674.97907	161	123671.958140	222.192780	3.13e-049	1.000000
TIM3+G	61677.94019	165	123685.880380	236.115020	2.96e-052	1.000000
TPM1+G	61729.76738	161	123781.534760	331.769400	5.02e-073	1.000000
HKY+G	61734.23893	163	123794.477860	344.712500	7.76e-076	1.000000
K80+G	61737.35709	160	123794.714180	344.948820	6.90e-076	1.000000
TIM1+G	61732.76377	165	123795.527540	345.762180	4.59e-076	1.000000
TrN+G	61733.83731	164	123795.674620	345.909260	4.27e-076	1.000000
TrNef+G	61736.90190	161	123795.803800	346.038440	4.00e-076	1.000000
TPM1uf+G	61734.22890	164	123796.457800	346.692440	2.88e-076	1.000000
TIM1ef+G	61739.00377	162	123802.007540	352.242180	1.80e-077	1.000000
F81+G	64978.45294	162	130280.905880	6831.140520	0.00e+000	1.000000
JC+G	65022.94315	159	130363.886300	6914.120940	0.00e+000	1.000000
TVMef	68107.70867	162	136539.417340	13089.651980	0.00e+000	1.000000
SYM	68116.72925	163	136559.458500	13109.693140	0.00e+000	1.000000
TIM2ef	68182.23319	161	136686.466380	13236.701020	0.00e+000	1.000000
GTR	68181.73869	166	136695.477380	13245.712020	0.00e+000	1.000000
TVM	68184.61931	165	136699.238620	13249.473260	0.00e+000	1.000000
TPM2	68215.11327	160	136750.226540	13300.461180	0.00e+000	1.000000
TIM3ef	68266.43558	161	136854.871160	13405.105800	0.00e+000	1.000000
TPM3	68269.24816	160	136858.496320	13408.730960	0.00e+000	1.000000
TIM2	68270.60438	164	136869.208760	13419.443400	0.00e+000	1.000000
TrNef	68298.29619	160	136916.592380	13466.827020	0.00e+000	1.000000
TIM3	68295.56421	164	136919.128420	13469.363060	0.00e+000	1.000000
TPM1	68301.05979	160	136922.119580	13472.354220	0.00e+000	1.000000
TPM2uf	68310.15349	163	136946.306980	13496.541620	0.00e+000	1.000000
TPM3uf	68324.20415	163	136974.408300	13524.642940	0.00e+000	1.000000
TIM1ef	68331.51662	161	136985.033240	13535.267880	0.00e+000	1.000000
K80	68334.15449	159	136986.308980	13536.543620	0.00e+000	1.000000
HKY	68368.77745	162	137061.554900	13611.789540	0.00e+000	1.000000
TIM1	68372.88645	164	137073.772900	13624.007540	0.00e+000	1.000000
TPM1uf	68377.71524	163	137081.430480	13631.665120	0.00e+000	1.000000

```
TrN         68408.15354   163  137142.307080  13692.541720   0.00e+000   1.000000

JC          71122.59713   158  142561.194260  19111.428900   0.00e+000   1.000000

F81         71202.29269   161  142726.585380  19276.820020   0.00e+000   1.000000
--------------------------------------------------------------------------
-lnL:   negative log likelihod
 K:     number of estimated parameters
 AIC:   Akaike Information Criterion
 delta: AIC difference
 weight:        AIC weight
 cumWeight:     cumulative AIC weight

Model selection results also available at the "Model > Show model table" menu


* AIC MODEL SELECTION : Confidence interval

There are 2 models in the 95% confidence interval: [ TVM+G GTR+G ]


* AIC MODEL SELECTION : Parameter importance

Parameter   Importance
----------------------
fA          1.0000
fC          1.0000
fG          1.0000
fT          1.0000
kappa       0.0000
titv        0.0000
rAC         1.0000
rAG         1.0000
rAT         1.0000
rCG         1.0000
rCT         1.0000
rGT         1.0000
alpha(G)            1.0000
----------------------
Values have been rounded.
 (I):  considers only +I models.
 (G):  considers only +G models.
 (IG): considers only +I+G models.


* AIC MODEL SELECTION : Model averaged estimates

           Model-averaged
Parameter       estimates
-------------------------
fA             0.2408
```

```
fC              0.2636
fG              0.2591
fT              0.2366
kappa           0.0000
titv            0.0000
rAC             1.1357
rAG             5.7098
rAT             1.7372
rCG             0.5644
rCT             5.5760
rGT             1.0000
alpha(G)                0.2960
-------------------------
Numbers have been rounded.
 (I):  considers only +I models.
 (G):  considers only +G models.
 (IG): considers only +I+G models.


* AIC MODEL SELECTION : Best Model's command line

phyml  -i /tmp/jmodeltest954472080326291458.phy -d nt -n 1 -b 0 --run_id TVM+G -m
012314 -f m -c 4 -a e --no_memory_check -o tlr -s BEST



---------------------------------------------------------------
*                                                             *
*                    MODEL AVERAGED PHYLOGENY                 *
*                                                             *
---------------------------------------------------------------

Selection criterion: . . . . AIC
Confidence interval: . . . . 0.95
Consensus type:. . . . . . . 50% majority rule


Using 2 models in the 0.95 confidence interval = TVM+G GTR+G

Species in order:

    1. BrM57-1
    2. BrM52-1
    3. Br22-1
    4. BrK47-1
    5. Br53-1
    6. BrM55-1
    7. Br32-2
    8. Br32-1
    9. BrM54-1
    10. BrM03-1
    11. BrM48-1
```

12. BrK01-1
13. Br48-1
14. BrM23-1
15. BrK55-1
16. BrM17-1
17. BrM21-1
18. BrM46-1
19. BrM11-1
20. BrM02-1
21. BrM45-1
22. BrK60-1
23. BrM27-1
24. BrM51-1
25. BrM60-3
26. BrM56-1
27. Gv10-C-05
28. Gv10-F-02
29. Gv10-F-10
30. Gv10-C-01
31. Gv10-H-05
32. Gv10-C-10
33. Gv10-B-10
34. Gv10-A-01
35. Gv10-F-08
36. Gv10-E-05
37. Gv10-B-02
38. Gv10-H-03
39. Gv10-B-06
40. Gv10-H-07
41. Gv10-A-10
42. Gv10-A-08
43. Vi10-2
44. In527
45. In501a
46. In526
47. In521
48. In553
49. In556
50. In525
51. In534
52. In547
53. In535
54. In501
55. Vi10-3
56. Vi12-1
57. Vi23-1
58. Vi12-3
59. Vi10-5
60. Vi23-4
61. Vi23-5
62. Vi12-5
63. Vi10-1

```
    64. BrM22-1
    65. Br47-1
    66. Br47-2
    67. BrM59-1
    68. BrK04-1
    69. BrK02-1
    70. Br22-2
    71. Br46-1
    72. Br46-2
    73. BrK06-1
    74. Br30-1
    75. Br30-2
    76. BrM50-1
    77. Br26-2
    78. Br26-1
    79. Br57-1
    80. Br57-2

Bipartitions included in the consensus tree


12345678911111111112222222222333333333344444444445555555555666666666677777777778

01234567890123456789012345678901234567890123456789012345678901234567890

********************************************************************************
( 1.0 )

--******************************************************************************
( 1.0 )

--***---------------------------------------------------------------------------
( 1.0 )

---**---------------------------------------------------------------------------
( 1.0 )

-----***************************************************************************
( 1.0 )

------**************************************************************************
( 1.0 )

------*********************************************************************-----
( 1.0 )

------**------------------------------------------------------------------------
( 1.0 )

--------*******************************************************************-----
( 1.0 )
```

```
--------*************************************************************-----------
( 1.0 )

--------*******-----------------------------------------------------------------
( 1.0 )

--------*****-------------------------------------------------------------------
( 1.0 )

--------***---------------------------------------------------------------------
( 1.0 )

---------**---------------------------------------------------------------------
( 1.0 )

-----------**-------------------------------------------------------------------
( 1.0 )

-------------**-----------------------------------------------------------------
( 1.0 )

---------------******************************************************-----------
( 1.0 )

---------------***********------------------------------------------------------
( 1.0 )

---------------********---------------------------------------------------------
( 1.0 )

----------------*******---------------------------------------------------------
( 1.0 )

----------------*****-----------------------------------------------------------
( 1.0 )

----------------**--------------------------------------------------------------
( 1.0 )

------------------***-----------------------------------------------------------
( 1.0 )

-------------------**-----------------------------------------------------------
( 1.0 )

---------------------**---------------------------------------------------------
( 1.0 )

-----------------------***------------------------------------------------------
( 1.0 )

------------------------**------------------------------------------------------
```

```
( 1.0 )

--------------------------*******************************************-----------
( 1.0 )

--------------------------*************************************-----------------
( 1.0 )

--------------------------****************--------------------------------------
( 1.0 )

---------------------------***************--------------------------------------
( 1.0 )

---------------------------******-----------------------------------------------
( 1.0 )

---------------------------***--------------------------------------------------
( 1.0 )

----------------------------**--------------------------------------------------
( 1.0 )

------------------------------***-----------------------------------------------
( 1.0 )

-------------------------------**-----------------------------------------------
( 1.0 )

---------------------------------*********--------------------------------------
( 1.0 )

---------------------------------****-------------------------------------------
( 1.0 )

---------------------------------**---------------------------------------------
( 1.0 )

-----------------------------------**-------------------------------------------
( 1.0 )

-------------------------------------*****--------------------------------------
( 1.0 )

--------------------------------------****--------------------------------------
( 1.0 )

---------------------------------------***--------------------------------------
( 1.0 )

----------------------------------------**--------------------------------------
( 1.0 )
```

```
------------------------------------------*********************-----------------
( 1.0 )

-------------------------------------------********************-----------------
( 1.0 )

-------------------------------------------***********--------------------------
( 1.0 )

-------------------------------------------******-------------------------------
( 1.0 )

-------------------------------------------**-----------------------------------
( 1.0 )

---------------------------------------------****-------------------------------
( 1.0 )

----------------------------------------------***-------------------------------
( 1.0 )

-----------------------------------------------**-------------------------------
( 1.0 )

-------------------------------------------------*****--------------------------
( 1.0 )

-------------------------------------------------***----------------------------
( 1.0 )

--------------------------------------------------**----------------------------
( 1.0 )

----------------------------------------------------**--------------------------
( 1.0 )

------------------------------------------------------*********-----------------
( 1.0 )

-------------------------------------------------------********-----------------
( 1.0 )

-------------------------------------------------------******-------------------
( 1.0 )

-------------------------------------------------------**-----------------------
( 1.0 )

---------------------------------------------------------****-------------------
( 1.0 )
```

```
----------------------------------------------------------***-------------------
( 1.0 )

-----------------------------------------------------------**-------------------
( 1.0 )

-------------------------------------------------------------**-----------------
( 1.0 )

---------------------------------------------------------------******-----------
( 1.0 )

---------------------------------------------------------------***--------------
( 1.0 )

----------------------------------------------------------------**--------------
( 1.0 )

------------------------------------------------------------------***-----------
( 1.0 )

-------------------------------------------------------------------**-----------
( 1.0 )

---------------------------------------------------------------------******-----
( 1.0 )

----------------------------------------------------------------------*****-----
( 1.0 )

----------------------------------------------------------------------**--------
( 1.0 )

------------------------------------------------------------------------***-----
( 1.0 )

-------------------------------------------------------------------------**-----
( 1.0 )

---------------------------------------------------------------------------*****
( 1.0 )

----------------------------------------------------------------------------****
( 1.0 )

----------------------------------------------------------------------------**--
( 1.0 )

------------------------------------------------------------------------------**
( 1.0 )
```

```
            +----80 Br53-1
        +-155
        |   +--79 BrK47-1
    +-156
    |   +--78 Br22-1
+-157
|   |                   +--77 Br26-1
|   |               +-150
|   |               |   +--76 Br26-2
|   |           +-151
|   |           |   |   +--75 Br57-2
|   |           |   +-149
|   |           |       +--74 Br57-1
|   |       +-152
|   |       |   +------73 BrM50-1
|   |   +-153
|   |   |   |                        +--72 Br30-2
|   |   |   |                   +--143
|   |   |   |                   |    +--71 Br30-1
|   |   |   |               +-144
|   |   |   |               |   +--70 BrK06-1
|   |   |   |           +-145
|   |   |   |           |   |    +--69 Br46-2
|   |   |   |           |   +--142
|   |   |   |           |        +--68 Br46-1
|   |   |   |       +-146
|   |   |   |       |   +--67 Br22-2
|   |   |   |   +-147
|   |   |   |   |   |           +----66 BrK55-1
|   |   |   |   |   |       +-139
|   |   |   |   |   |       |   +--65 BrM23-1
|   |   |   |   |   |   +-140
|   |   |   |   |   |   |   |           +--64 BrM48-1
|   |   |   |   |   |   |   |       +-136
|   |   |   |   |   |   |   |       |   +--63 BrM03-1
|   |   |   |   |   |   |   |   +-137
|   |   |   |   |   |   |   |   |   +--62 BrM54-1
|   |   |   |   |   |   |   +-138
|   |   |   |   |   |   |       |   +--61 Br48-1
|   |   |   |   |   |   |       +-135
|   |   |   |   |   |   |           +--60 BrK01-1
|   |   |   |   |   +-141
|   |   |   |   |       |                   +-----59 BrM27-1
|   |   |   |   |       |               +-130
|   |   |   |   |       |               |   +--58 BrK60-1
|   |   |   |   |       |           +-131
|   |   |   |   |       |           |   |       +--57 BrM46-1
|   |   |   |   |       |           |   |   +-128
|   |   |   |   |       |           |   |   |   +--56 BrM21-1
|   |   |   |   |       |           |   +-129
|   |   |   |   |       |           |       |       +--55 BrM45-1
|   |   |   |   |       |           |       |   +-126
```

```
|   |   |   |   |       |           |       |   |   +--54 BrM02-1
|   |   |   |   |       |           |       +-127
|   |   |   |   |       |           |           +--53 BrM11-1
|   |   |   |   |       |       +-132
|   |   |   |   |       |       |   +----52 BrM17-1
|   |   |   |   |       |   +-133
|   |   |   |   |       |   |   |       +--51 BrM56-1
|   |   |   |   |       |   |   |   +-124
|   |   |   |   |       |   |   |   |   +--50 BrM60-3
|   |   |   |   |       |   |   +-125
|   |   |   |   |       |   |       +--49 BrM51-1
|   |   |   |   |       +-134
|   |   |   |   |           |                               +--48 Vi10-1
|   |   |   |   |           |                           +-117
|   |   |   |   |           |                           |   +--47 Vi12-5
|   |   |   |   |           |                       +-118
|   |   |   |   |           |                       |   |               +--46
Vi23-5
|   |   |   |   |           |                       |   |           +-113
|   |   |   |   |           |                       |   |           |   +--45
Vi23-4
|   |   |   |   |           |                       |   |       +-114
|   |   |   |   |           |                       |   |       |   +--44 Vi10-5
|   |   |   |   |           |                       |   |   +-115
|   |   |   |   |           |                       |   |   |   +--43 Vi12-3
|   |   |   |   |           |                       |   +-116
|   |   |   |   |           |                       |       |   +--42 Vi23-1
|   |   |   |   |           |                       |       +-112
|   |   |   |   |           |                       |           +--41 Vi12-1
|   |   |   |   |           |                   +-119
|   |   |   |   |           |                   |   +--40 Vi10-3
|   |   |   |   |           |               +-120
|   |   |   |   |           |               |   |           +--39 In501a
|   |   |   |   |           |               |   |       +-109
|   |   |   |   |           |               |   |       |   +--38 In527
|   |   |   |   |           |               |   |   +-110
|   |   |   |   |           |               |   |   |   |           +--37 In556
|   |   |   |   |           |               |   |   |   |       +-106
|   |   |   |   |           |               |   |   |   |       |   +--36 In553
|   |   |   |   |           |               |   |   |   |   +-107
|   |   |   |   |           |               |   |   |   |   |   +--35 In521
|   |   |   |   |           |               |   |   |   +-108
|   |   |   |   |           |               |   |   |       +--34 In526
|   |   |   |   |           |               |   +-111
|   |   |   |   |           |               |       |       +-------33 In501
|   |   |   |   |           |               |       |   +-104
|   |   |   |   |           |               |       |   |   +--32 In535
|   |   |   |   |           |               |       +-105
|   |   |   |   |           |               |           |       +--31 In547
|   |   |   |   |           |               |           |   +-102
|   |   |   |   |           |               |           |   |   +--30 In534
|   |   |   |   |           |               |           +-103
```

```
|   |   |   |   |           |               |               +--29 In525
|   |   |   |   |           |           +-121
|   |   |   |   |           |           |   +----28 Vi10-2
|   |   |   |   |           |   +-----122
|   |   |   |   |           |   |       |                   +--27 Gv10-C-01
|   |   |   |   |           |   |       |               +--97
|   |   |   |   |           |   |       |               |   +--26 Gv10-F-10
|   |   |   |   |           |   |       |           +--98
|   |   |   |   |           |   |       |           |   +--25 Gv10-F-02
|   |   |   |   |           |   |       |       +--99
|   |   |   |   |           |   |       |       |   |       +--24 Gv10-B-10
|   |   |   |   |           |   |       |       |   |   +--95
|   |   |   |   |           |   |       |       |   |   |   +--23 Gv10-C-10
|   |   |   |   |           |   |       |       |   +--96
|   |   |   |   |           |   |       |       |       +-----22 Gv10-H-05
|   |   |   |   |           |   |       |   +-100
|   |   |   |   |           |   |       |   |   |           +--21 Gv10-F-08
|   |   |   |   |           |   |       |   |   |       +--92
|   |   |   |   |           |   |       |   |   |       |   +--20 Gv10-A-01
|   |   |   |   |           |   |       |   |   |   +--93
|   |   |   |   |           |   |       |   |   |   |   |   +--19 Gv10-B-02
|   |   |   |   |           |   |       |   |   |   |   +--91
|   |   |   |   |           |   |       |   |   |   |       +--18 Gv10-E-05
|   |   |   |   |           |   |       |   |   +--94
|   |   |   |   |           |   |       |   |       |               +---17
Gv10-A-08
|   |   |   |   |           |   |       |   |       |           +--87
|   |   |   |   |           |   |       |   |       |           |   +--16
Gv10-A-10
|   |   |   |   |           |   |       |   |       |       +--88
|   |   |   |   |           |   |       |   |       |       |   +--15 Gv10-H-07
|   |   |   |   |           |   |       |   |       |   +--89
|   |   |   |   |           |   |       |   |       |   |   +--14 Gv10-B-06
|   |   |   |   |           |   |       |   |       +--90
|   |   |   |   |           |   |       |   |           +---13 Gv10-H-03
|   |   |   |   |           |   |       +-101
|   |   |   |   |           |   |           +---12 Gv10-C-05
|   |   |   |   |           +-123
|   |   |   |   |               |           +--11 BrK02-1
|   |   |   |   |               |       +--84
|   |   |   |   |               |       |   +--10 BrK04-1
|   |   |   |   |               |   +--85
|   |   |   |   |               |   |   +---9 BrM59-1
|   |   |   |   |               +--86
|   |   |   |   |                   |        +---8 Br47-2
|   |   |   |   |                   |   +---82
|   |   |   |   |                   |   |    +---7 Br47-1
|   |   |   |   |                   +--83
|   |   |   |   |                       +----6 BrM22-1
|   |   |   +-148
|   |   |       |    +---5 Br32-1
|   |   |       +---81
```

```
|   |   |            +---4 Br32-2
|   +-154
|       +----3 BrM55-1
|
+---2 BrM52-1
|
+------1 BrM57-1
```

```
(BrM57-1:0.051848,BrM52-1:0.024815,((BrM55-1:0.040002,(((Br32-2:0.012404,Br32-1:0
.006430)1.00:0.041546,((((((BrM22-1:0.036591,(Br47-1:0.002761,Br47-2:0.003817)1.0
0:0.042946)1.00:0.008688,(BrM59-1:0.021687,(BrK04-1:0.024840,BrK02-1:0.035446)1.0
0:0.010895)1.00:0.007650)1.00:0.006559,((Gv10-C-05:0.037425,(((Gv10-H-03:0.038713
,(Gv10-B-06:0.035132,(Gv10-H-07:0.021675,(Gv10-A-10:0.022479,Gv10-A-08:0.041875)1
.00:0.012478)1.00:0.008806)1.00:0.003831)1.00:0.011389,((Gv10-E-05:0.021550,Gv10-
B-02:0.025659)1.00:0.020254,(Gv10-A-01:0.025764,Gv10-F-08:0.026507)1.00:0.013175)
1.00:0.007268)1.00:0.009645,((Gv10-H-05:0.050692,(Gv10-C-10:0.030513,Gv10-B-10:0.
026500)1.00:0.008688)1.00:0.014956,(Gv10-F-02:0.035883,(Gv10-F-10:0.020188,Gv10-C
-01:0.025740)1.00:0.006218)1.00:0.008780)1.00:0.009999)1.00:0.019293)1.00:0.01833
2,(Vi10-2:0.048238,((((In525:0.032798,(In534:0.021076,In547:0.025134)1.00:0.00995
9)1.00:0.007947,(In535:0.025287,In501:0.070932)1.00:0.013164)1.00:0.006532,((In52
6:0.026792,(In521:0.029859,(In553:0.019727,In556:0.018035)1.00:0.005785)1.00:0.01
5779)1.00:0.007022,(In527:0.028811,In501a:0.035318)1.00:0.005475)1.00:0.008125)1.
00:0.029345,(Vi10-3:0.032904,(((Vi12-1:0.028560,Vi23-1:0.030108)1.00:0.015189,(Vi
12-3:0.034654,(Vi10-5:0.025213,(Vi23-4:0.002750,Vi23-5:0.003462)1.00:0.035889)1.0
0:0.011144)1.00:0.006301)1.00:0.007194,(Vi12-5:0.030246,Vi10-1:0.024043)1.00:0.01
3171)1.00:0.009582)1.00:0.009637)1.00:0.014949)1.00:0.009283)1.00:0.059624)1.00:0
.006668,((BrM51-1:0.028582,(BrM60-3:0.032024,BrM56-1:0.023818)1.00:0.009790)1.00:
0.012414,(BrM17-1:0.047907,(((BrM11-1:0.024473,(BrM02-1:0.034265,BrM45-1:0.021596
)1.00:0.012788)1.00:0.009411,(BrM21-1:0.020146,BrM46-1:0.018502)1.00:0.009550)1.0
0:0.011332,(BrK60-1:0.027680,BrM27-1:0.053157)1.00:0.011269)1.00:0.004301)1.00:0.
015422)1.00:0.008326)1.00:0.008111,(((BrK01-1:0.030762,Br48-1:0.028139)1.00:0.016
636,(BrM54-1:0.027014,(BrM03-1:0.024388,BrM48-1:0.015711)1.00:0.017126)1.00:0.003
967)1.00:0.006525,(BrM23-1:0.029536,BrK55-1:0.043542)1.00:0.008511)1.00:0.008740)
1.00:0.004546,(Br22-2:0.031574,((Br46-1:0.005946,Br46-2:0.002611)1.00:0.040798,(B
rK06-1:0.032159,(Br30-1:0.006317,Br30-2:0.001763)1.00:0.041332)1.00:0.012013)1.00
:0.012669)1.00:0.012233)1.00:0.004328)1.00:0.005710,(BrM50-1:0.061264,((Br57-1:0.
001190,Br57-2:0.006039)1.00:0.019544,(Br26-2:0.001957,Br26-1:0.009286)1.00:0.0233
09)1.00:0.031239)1.00:0.017826)1.00:0.010045)1.00:0.013030,(Br22-1:0.026676,(BrK4
7-1:0.027020,Br53-1:0.048154)1.00:0.022088)1.00:0.018719)1.00:0.031119);
```

Note: this tree is unrooted. Branch lengths are the expected number of substitutions per site. Labels next to parentheses represent phylogenetic uncertainty due to model selection (see documentation)
